# Integrated multi-omics assessment of lineage plasticity in a prostate cancer patient with brain and dural metastases

**DOI:** 10.1038/s41698-024-00713-8

**Published:** 2024-09-30

**Authors:** Megan L. Ludwig, David Moline, Alec Horrmann, Ella Boytim, Gabrianne Larson, Ali T. Arafa, Masooma Sayeda, John R. Lozada, Hannah E. Bergom, Abderrahman Day, Sandhyarani Dasaraju, Scott M. Dehm, Paari Murugan, Justin Hwang, Justin M. Drake, Emmanuel S. Antonarakis

**Affiliations:** 1 Department of Pharmacology, University of Minnesota, Minneapolis, MN USA; 2 Department of Medicine, Division of Hematology, Oncology, and Transplantation, University of Minnesota, Minneapolis, MN USA; 3 Department of Laboratory Medicine and Pathology, University of Minnesota, Minneapolis, MN USA; 4 Masonic Cancer Center, University of Minnesota, Minneapolis, MN USA; 5 Department of Urology, University of Minnesota, Minneapolis, MN USA

**Keywords:** Molecular medicine, Metastasis, Metastasis

## Abstract

Metastases to the brain are rare in prostate cancer. Here, we describe a patient with two treatment-emergent metastatic lesions, one to the brain with neuroendocrine prostate cancer (NEPC) histology and one to the dural membrane of adenocarcinoma histology. We performed genomic, transcriptomic, and proteomic characterization of these lesions and the primary tumor to investigate molecular features promoting these metastases. The two metastatic lesions had high genomic similarity, including *TP53* mutation and *PTEN* deletion, with the most striking difference being the additional loss of *RB1* in the NEPC lesion. Interestingly, the dural lesion expressed both androgen receptor and neuroendocrine markers, suggesting amphicrine carcinoma (AMPC). When analyzing pioneer transcription factors, the AMPC lesion exhibited elevated FOXA1 activity while the brain NEPC lesion showed elevated HOXC10, NFYB, and OTX2 expression suggesting novel roles in NEPC formation or brain tropism. Our results highlight the utility of performing multi-omic characterization, especially in rare cancer subtypes.

## Introduction

Prostate cancer (PC) is the most common male cancer in the United States and is the second leading cause of cancer-related death^1^. Treatment is curative for many patients with localized PC, with a five-year survival of >95%, but once metastatic prostate cancer develops, survival declines to near 30%^2^. The most common site of metastasis in PC is bone (80%) followed by lymph nodes (20%), and liver (10%)^3^. Metastasis to the brain is rare, occurring in about 1% of patients, and has especially poor prognosis where the median survival is 3–12 months^4–6^. Multiple mechanisms of metastatic seeding have been proposed in prostate cancer patients^7,8^. While many metastatic foci share clonal origins from the primary tumor, metastatic clones themselves have also been found to seed further metastases^9,10^. However, in these studies, rarely are the clones characterized beyond the genomic level which is needed to identify the acquired alterations that infer metastatic lineage.

During progression of prostate cancer, activation of *AR* and/or *FOXA1*, *PTEN* loss, and *TP53* inactivation are common alterations^11–14^. These clonal evolutions convey survival benefits to the tumor, imparting driver roles in either therapeutic resistance or metastatic spread^10^. A lethal transition that can emerge is AR-null small cell/neuroendocrine prostate cancer (NEPC). NEPC has similar histological features to small cell lung cancer and is often molecularly characterized by *RB1* deletion with or without *TP53* mutation, and expression of neuroendocrine genes *SYP* or *CHGA*^15–17^. Another subtype of prostate cancer, known as amphicrine carcinoma (AMPC), expresses neuroendocrine markers in addition to luminal-lineage markers such as prostate-specific antigen (PSA) and AR^18^. AMPC is thought to be distinct from tumors in which adenocarcinoma and neuroendocrine cells are intermixed^16^ and is defined by the co-expression of AR-related and neuroendocrine markers within individual cells^19^. It remains unknown if AMPC represents an intermediate transitional state of prostate cancer progression towards developing NEPC.

Here we present a case of a prostate cancer patient with an AMPC metastasis in the dura that may have possibly seeded a brain metastasis with NEPC histology. Using an integrated multi-omics approach, we interrogated the primary prostatic tumor and the two metastatic lesions at the genomic, transcriptomic, and proteomic level to identify molecular mechanisms that may have promoted metastatic spread and cellular plasticity. Our case findings suggest potential novel roles for transcription factors HOXC10, NFYB, and OTX2 in NEPC development, and also highlight the importance of FOXA1 activity in the AMPC subtype. These transcription factors should be investigated in additional AMPC and NEPC cases, ideally in larger cohorts, to substantiate these findings.

## Results

### Clinical case

A 69-year-old man with no family history of prostate, breast, or ovarian cancers presented with left clavicular pain. Laboratory tests showed an elevated PSA level of 1190 ng/mL and an alkaline phosphatase level of 1627 U/L. A prostatic biopsy showed Gleason 5 + 4 = 9 (Grade Group 5) adenocarcinoma in 11 out of 12 cores, with perineural invasion. A technetium-99 bone scan demonstrated widespread osseous lesions throughout the axial and appendicular skeleton, including the calvarium. A Caris Life Sciences molecular test performed on the prostate biopsy revealed a pathogenic *TP53* mutation (p.C176Y), a tumor mutational burden (TMB) of 1 mutation per megabase, and low (3%) genomic loss of heterozygosity (gLOH). Germline testing using the 84-gene Invitae assay was unremarkable.

**Fig. 1:**
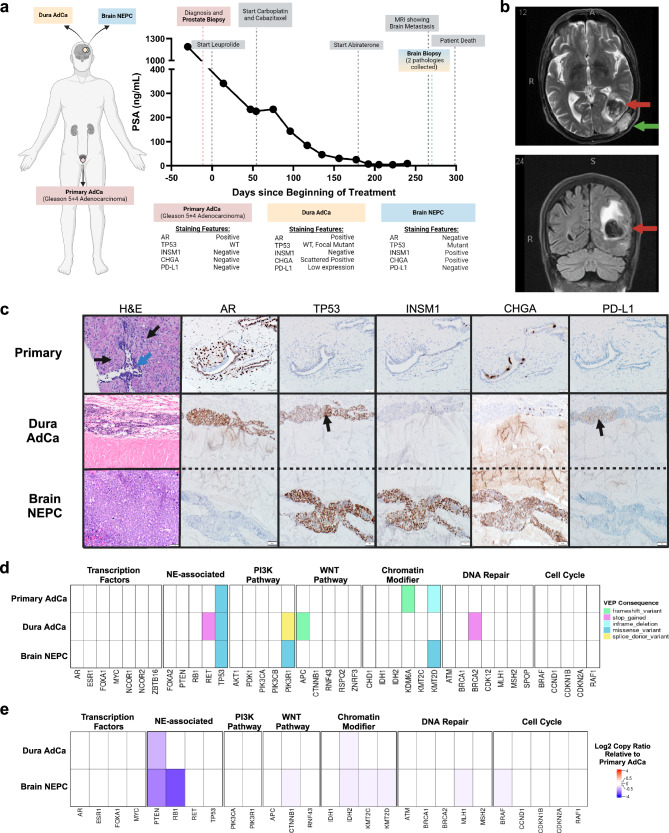
Patient and biopsy characteristics and genomic features. **a** A timeline for the patient from diagnosis to death, with treatment and PSA levels indicated. The staining features are summarized from IHC staining in (**c**). Created with Biorender.com. **b** Brain MRI scan (T1- and T2-weighted images after gadolinium contrast) showed lobulated enhancing, expansile intraosseous lesions in the posterior left parietal bone, conglomerated in appearance and measuring ~5.1 × 1.6 cm. There is cortical destruction of the inner and outer tables of the calvarium. There is mass effect on the underlying dura with associated thickening and enhancement. Anteriorly, one of these enhancing components infiltrates the adjacent left occipital brain parenchyma (red arrow) and results in a 3.3 cm round-shaped intraparenchymal hemorrhage with surrounding vasogenic edema and mass affect. There is a second dural-based 1.2 × 1.4 cm metastatic focus (green arrow) next to left posterior temporal lobe with suspicion of adjacent brain parenchyma invasion. The cerebral ventricles are proportionate to the sulci, without midline shift. **c** Top H&E: Grade Group 5 acinar adenocarcinoma (black arrows) infiltrating prostate stroma and surrounding normal glands (blue arrow). Gleason pattern 4 carcinoma was also present (not shown). Middle H&E: Cribriform acinar adenocarcinoma involving the peridural connective tissue. Bottom H&E: Underlying high-grade neuroendocrine carcinoma (NEPC) with sheet-like growth pattern and high mitotic activity replacing brain parenchyma. Staining for AR, TP53, INSM1, CHGA, and PD-L1 is shown. The black arrow in TP53 for the dura adenocarcinoma is a focal area of staining suggestive of mutated pattern of TP53, as similar to TP53 in the NEPC component. The black arrow in PD-L1indicates low (5%) staining in the dural adenocarcinoma. **d** Genes of interest in prostate cancer with their most significant functional impact as calculated by variant effect predictor (VEP) consequences, indicated by color. **e** Heatmap of log2 copy ratio of dura adenocarcinoma or brain NEPC as compared to primary prostate.

The patient began androgen deprivation therapy using an LHRH analog, resulting in a drop in his PSA level to 226 ng/mL. Two months later, he was enrolled in the CASCARA clinical trial (NCT03934840) and received a combination of cabazitaxel (20 mg/m²), and carboplatin (AUC = 4) for six cycles. This regimen further reduced his PSA level to 4.2 ng/mL after 5 months. Subsequently, abiraterone (1000 mg) and prednisone (5 mg) were added to his treatment regimen as part of the CASCARA clinical trial. However, within 7 months of systemic therapy initiation, his PSA level rose to 9.2 ng/mL, indicating castration-resistant prostate cancer (CRPC). A schematic of the patient’s treatment trajectory is depicted in Fig. 1a.

One month after developing CRPC, the patient presented with aphasia. Magnetic resonance imaging (MRI) of the brain showed a left parieto-occipital calvarial metastasis infiltrating the underlying dura, as well as a left temporal-occipital lobe brain metastasis with associated intraparenchymal hemorrhage and surrounding mass effect (Fig. 1b). A biopsy of the brain lesion demonstrated poorly differentiated markedly cellular lobules of tumor cells with prominent peripheral nuclear palisading and occasional central necrosis, in addition to extensive intravascular growth. These cells demonstrated high nuclear to cytoplasmic ratio, minimal cytoplasm, and finely granular chromatin, consistent with a small cell/neuroendocrine appearance. By immunohistochemistry (IHC), the tumor cells showed strong reactivity for INSM1 and CHGA, and diffuse nuclear TP53 reactivity suggestive of an underlying pathogenic mutation^20^, while PD-L1 and AR immunostains were negative (Fig. 1c). Caris Life Sciences genomic analysis of the brain biopsy showed a *TP53* mutation (p.C176Y), TMB of 1 mut/Mb, and high gLOH (17%). A separate tumor with adenocarcinoma histology was seen within the dura, displaying moderately differentiated, infiltrative nests and lobules of tumor cells with acinar and cribriform growth pattern, with infiltration into the vascular spaces. By IHC, the tumor cells in this dural region were positive for AR (diffuse) and PD-L1 (patchy, tumor proportion score 5%) without significant reactivity to INSM1 and CHGA (Fig. 1c). This increase in PD-L1 expression is broadly consistent with the literature indicating that PD-L1 increases in metastatic CRPC relative to primary and hormone-sensitive prostate cancer^21^, and is likely a result of previous androgen axis blockade causing adaptive upregulation^22^.

Following brain biopsy, the patient was offered palliative brain-directed radiotherapy but he declined it. Unfortunately, his performance status deteriorated rapidly and he was not a candidate for additional systemic therapy. Thus, a decision was made to stop aggressive treatment and pursue home hospice. The interval of time between brain biopsy and death was 30 days. An autopsy was not performed, in accordance with the wishes of the patient and his wife.

### AR and neuroendocrine features distinguish AMPC (dural) and NEPC (brain) lesions

The primary prostate tumor exhibited very few mutations in prostate cancer-relevant genes^23^ except for the pathogenic *TP53* mutation (p.C176Y) with an allele frequency (AF) of 0.42 (Fig. 1d, Supplementary Table 1). The mutations in the chromatin modifier genes *KDM6A* and *KMT2D* displayed low AFs (0.02, 0.01, respectively), suggesting minimal impact on tumor biology especially as they were not carried into the metastatic lesions. Notably, the AF of the *TP53* mutation increased to 0.84 in the dural adenocarcinoma and 0.94 in the brain NEPC metastases, further supporting the importance of this driver alteration. With respect to copy number analysis, we observed loss of *PTEN* in the metastatic dural adenocarcinoma when compared to the primary prostate, while the brain NEPC displayed both *PTEN* and *RB1* losses as is common in NEPC^16,17,24^ (Fig. 1e, Supplementary Table 2). Thus, the brain lesion displayed inactivation of three tumor suppressors (*TP53*/*PTEN*/*RB1*), while the dura and primary tumors displayed inactivation of two and one tumor suppressors, respectively. When directly comparing the variants present within all three samples and their allele frequencies, we observed high correlation (*r* = 0.93, *p*-value < 0.0001) between the metastatic dural adenocarcinoma and the brain NEPC lesion (Supplementary Fig. 1) which was not dependent on sequence coverage (Supplementary Fig. 2). The lack of genetic drift between these clones suggested that the origin of the metastatic brain NEPC may have been seeded from the metastatic dural adenocarcinoma (linear evolution), rather than both arising from the primary prostate (divergent evolution). The mutations in the metastatic dura adenocarcinoma not preserved in the brain NEPC (Fig. 1d) had low allele frequency (AF < 0.1) and so were likely not part of the seeding clone.

**Fig. 2:**
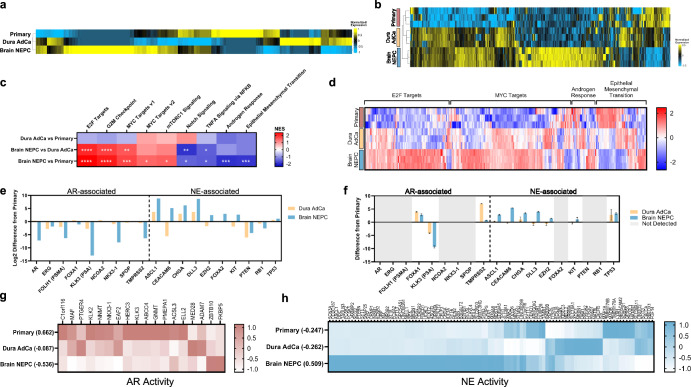
AR and NE features distinguish sample subtypes. Heatmap of transcriptome (**a**) and proteome (**b**) with unsupervised hierarchical clustering. **c** Heatmap of normalized enrichment scores from GSEA analysis, with significance marked (*FDR < 0.05, **FDR < 0.01, ***FDR < 0.001, ****FDR < 0.0001). **d** Heatmap of protein expression of genes in Hallmark pathways identified in (**c**). Panel of AR- and NE-associated genes and the difference of expression in transcriptome (**e**) and proteome (**f**) in the metastatic dura adenocarcinoma or metastatic brain neuroendocrine samples from the primary prostate. Error bars indicate standard error of the mean. Heatmap of AR activity (**g**) or NE activity (**h**) genes in the transcriptome, with the summative z-score calculation for each sample.

For a deeper interrogation of the molecular features of these lesions, we performed transcriptomic and proteomic analyses. The transcriptomes of all three samples were highly correlated (*r* > 0.8, *p*-value < 0.001) (Supplementary Fig. 3a), though the primary prostate and metastatic dural adenocarcinoma were most similar by hierarchical clustering (Fig. 2a); these two tumor lesions were also most similarly clustered by proteome analysis (Fig. 2b) demonstrating the highest correlation (*r* = 0.59, *p*-value < 0.001) (Supplementary Fig. 3b). The similarity between the primary prostate and metastatic dural adenocarcinoma was further reflected in gene set enrichment analysis (GSEA) of the transcriptome, as there were no hallmark pathways significantly enriched in this comparison (Fig. 2c). However, the metastatic brain NEPC lesion had a significant enrichment of E2F and MYC targets, which are transcriptional profiles that reflect altered proliferation (E2F)^25^ or possibly drive neuroendocrine differentiation in prostate cancer (MYCN)^26^. As expected, the NEPC lesion was characterized by loss of AR signaling as indicated by depletion of androgen-responsive gene expression as compared to the primary prostate tumor. Surprisingly, genes in the epithelial-to-mesenchymal transition (EMT) pathway were depleted in the metastatic brain NEPC, as NEPC development generally involves transdifferentiation via EMT processes^27^. Concordant with the transcriptome data, evaluating protein expression in these pathways revealed upregulation of E2F and MYC targets, as well as depletion of EMT signaling in the metastatic brain NEPC (Fig. 2d). The downregulation of the androgen response pathway in the metastatic brain NEPC was less clear from the proteomic data, which may be due to the lack of adequate coverage of proteins in this pathway.

Next, we further interrogated genes associated with AR and NE phenotypes, as we suspected that the metastatic dural adenocarcinoma may be an AMPC that expressed both sets of genes. Several AR-associated genes (*AR, FOLH1, KLK3, NKX3-1, TMPRSS2)* showed minimal change in the metastatic dura adenocarcinoma as compared to the primary prostate tumor, while there was a marked downregulation of these AR-related transcripts in the metastatic brain NEPC lesion (Fig. 2e). Other AR-related genes (*FOXA1, NCOA2, SPOP*) exhibited minimal differences in transcript expression in either metastatic site relative to the primary tumor. However, FOXA1 protein expression increased in both the metastatic dural adenocarcinoma and the brain NEPC lesion, while TMPRSS2 protein expression increased in the dural adenocarcinoma specifically (Fig. 2f). With respect to neuroendocrine-associated genes, we observed an expected increase in ASCL1, CHGA, and DLL3 expression at both the transcript and protein levels in the metastatic brain NEPC lesion. In the dural adenocarcinoma, we observed the RNA transcripts of these NE-associated genes to be upregulated, but only a minor increase in CHGA was reflected at the protein level. To further explore measurements of AR and NE activity, we utilized our transcriptome data to generate gene signature scores^28,29^ and observed that AR activity was highest in the primary tumor, intermediate in the dural adenocarcinoma, and markedly downregulated in the brain NEPC lesion (Fig. 2g). This intermediate AR-dependent state of the metastatic dural adenocarcinoma may explain why the androgen response pathway was not significantly different in the GSEA analysis from either the primary prostate or metastatic brain NEPC lesion. With respect to NE activity, the metastatic brain lesion was highly enriched for neuroendocrine markers, while both the metastatic dural adenocarcinoma and the primary tumor displayed similarly low levels of NE activity (Fig. 2h).

### Transcription factor reprogramming of prostate cancer subtypes

**Fig. 3:**
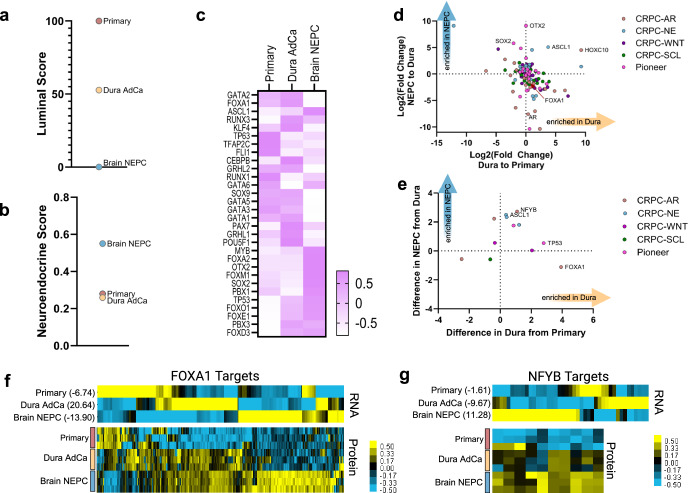
Transcription factor reprogramming of prostate cancer subtypes. **a** Luminal score based on transcript expression using scaled summative z-score. **b** Neuroendocrine score based on transcript expression using Pearson correlation. **c** Heatmap of transcriptome expression of pioneer transcription factors. Log2 fold changes in the transcriptome (**d**) and proteome (**e**) of transcription factors from (**c**) and four CRPC subtype classifications^30^ CRPC-AR, -NE, -WNT (Wnt signaling), or -SCL (stem cell-like) (Supplementary Fig 4). Heatmaps of transcriptome and proteome as indicated of predicted FOXA1 (**f**) or NFYB (**g**) direct targets with summative z-scores indicated.

We next turned to cell-lineage gene signatures^28,29^ to understand the cell plasticity changes that were occurring in the metastatic dural adenocarcinoma and brain NEPC lesion. The primary prostate sample scored highest for a luminal cell identity^28^, followed by the metastatic dural adenocarcinoma (Fig. 3a) which supported this metastasis retaining luminal epithelial features such as observed in the AMPC subtype. With respect to neuroendocrine cell identity score^29^, the metastatic brain tumor scored high while the metastatic dural adenocarcinoma and primary tumor both scored low (Fig. 3b). Thus, while the metastatic dural adenocarcinoma may have modestly expressed some NE-associated genes and features, it retained a predominant luminal-cell epithelial identity.

To better understand lineage plasticity changes that occurred in the metastases, we examined the expression level of four transcription factor regulatory networks that classify CRPC^30^. The metastatic brain lesion highly expressed many of the transcription factors reflective of the CRPC-NE subtype (Supplementary Fig. 4). Consistent with the notion of retaining aspects of AR signaling and luminal identity, the metastatic dural adenocarcinoma had strong expression of transcription factors reflective of the CRPC-AR subtype. We also observed expression of transcription factors regulating the CRPC-WNT subtype, which was interesting as activated WNT signaling has been associated with prostate cancer progression to AR-indifferent and NEPC phenotypes^31^. However, as transcription factors for AMPC are currently undefined, we also sought to extend our evaluation to interrogate broader pioneer transcription factors from literature review^32^ (Fig. 3c). The most striking enrichment of pioneer transcription factors occurred in the transcriptome of the metastatic brain NEPC lesion, displaying marked upregulation of OTX2 and SOX2 (Fig. 3d). SOX2 is a known neuroendocrine-associated factor^33^, but OTX2 is understudied in prostate cancer. OTX2 mediates the development of the central nervous system and has been noted as a potential driver in medulloblastoma^34^ where OTX2 has been observed to bind in a complex with MYC to drive gene expression^35^. In both the dural adenocarcinoma and the brain NEPC lesion, we also observed an upregulation of HOXC10, despite it being a putative factor of the CRPC-AR regulatory network^30^. This was intriguing given HOXC10’s involvement in motor neuron differentiation and association with cancer progression^36^, indicating that this transcription factor may have multiple roles in prostate cancer. We next evaluated these transcription factors using the proteome, and we found that FOXA1 and NFYB were the most upregulated transcription factors in the metastatic dural adenocarcinoma (Fig. 3e), although this was not concordant with the transcriptome data. We used the Harmonizome^37^ and JASPAR databases^38^ (which predict target genes of transcription factors based on binding-site motifs) to evaluate the expression of FOXA1 target genes, finding that the metastatic dural adenocarcinoma had the highest z-scores of transcriptome expression (Fig. 3f). In looking at ONECUT2, a transcription factor associated with NEPC and repressor of FOXA1^39^, we instead found that ONECUT2 had a sixfold increase in transcript expression in the metastatic dural adenocarcinoma over the primary prostate (Supplementary Tables 3 and 4). In the brain NEPC lesion, there was a further 2.6-fold increase in ONECUT2 expression and expected decrease in FOXA1 activity. We also evaluated the predicted target genes of NFYB, as we were surprised to see this protein enriched in the metastatic brain NEPC lesion given its connection to CRPC-AR and its regulation of epithelial cell survival^40^. NFYB target genes were indeed enriched in the metastatic brain NEPC lesion (Fig. 3g), suggesting that NFYB activity may also be relevant in the NEPC subtype.

## Discussion

Amphicrine prostate cancers may be under-diagnosed due to their retained adenocarcinoma histology, especially if there is no additional need to evaluate expression of neuroendocrine markers. While it is not known if the AMPC subtype represents a transitional state in the process of transdifferentiation to NEPC, the results of our case study support this possibility. Our multi-omic profiling also suggested the possibility that the metastatic dural adenocarcinoma may have seeded the brain NEPC lesion. This has not been suggested before with other AMPC tumors, possibly because this is the first time that a known AMPC and NEPC tumor have been identified in the same patient with the ability to interrogate potential phylogenetic relationships. While it is difficult to make conclusions about the possible clonal relationships between AMPC and NEPC from a singular case study, we propose this metastatic seeding hypothesis so that future research can collect the needed information to further explore this potential relationship. The genomic-level similarity between the AMPC and NEPC tumors in our study was surprising, with only the loss of *RB1* in the metastatic brain NEPC (as compared to the primary prostate and dural adenocarcinoma) being the most obvious genomic feature that could have predicted further transition from AMPC to a neuroendocrine carcinoma. In the SU2C-PCF dataset, the AMPC tumors with genomic profiling were similarly not *RB1*-deficient, and these patients had better overall survival than those with NEPC^19^. Further influence from the tumor microenvironment, metastatic site, or therapeutic pressure may have also played key roles in the transition from AMPC to NEPC in our case, and this places further emphasis on *RB1* function on prostate cancer progression^41,42^.

AMPC is defined by the presence of both AR signaling markers (AR, PSA, or NKX3.1) and neuroendocrine markers (CHGA or SYP) within a cell. These tumors often show features of high-grade adenocarcinoma without the typical morphology of small-cell or large-cell neuroendocrine carcinoma, such as nuclear molding or extensive geographic necrosis. Clinically, AMPC can present either de novo or as treatment-emergent, with the latter often showing a more aggressive course and poorer outcomes though these patients still exhibit better overall survival than NEPC cases^19^. Given the unique molecular profile of AMPC, comprising both AR-related and neuroendocrine features, there is a strong rationale for exploring dual-targeted therapies in this subset. 177Lu-dotatate, known for its efficacy in treating somatostatin receptor-positive neuroendocrine tumors, could potentially target both NEPC tumors and the neuroendocrine component of AMPC^19^, as exemplified by the confirmed *SSTR5* expression in our patient. Concurrently, targeting PSMA with 177Lu-PSMA-617, which is likely expressed in AMPC due to retained AR signaling, potentially offers a strategy to address the AR-driven component. The combination or sequential use of these targeted radioligand therapies could theoretically address both NEPC and AMPC tumors, potentially enhancing treatment outcomes by targeting intra-patient tumoral heterogeneity. Furthermore, while AMPC might initially respond to classical AR-targeted approaches due to its partial AR dependency, the presence of neuroendocrine differentiation suggests a potential for early hormonal resistance or a need for alternative therapies targeting neuroendocrine signaling, which might be less effective in NEPC due to its typical loss of AR signaling. Therefore, epigenetic therapies targeting pathways such as EZH2 could be explored, given the loss of the transcriptional repressor REST in both AMPC and NEPC, indicating a potential role for epigenetic dysregulation in these prostate cancers. Such insights could refine therapeutic approaches, potentially leading to more effective management of both AMPC and NEPC lesions within the same patient.

Cell lineage plasticity is a known contributor to cancer progression and therapeutic resistance, and so we interrogated transcription factors of regulatory networks associated with cellular identity. While known NE factors such as ASCL1 and SOX2 were upregulated in the metastatic brain NEPC metastasis, we also identified OTX2 as a potential novel factor implicated in NEPC and/or brain tropism. Given that OTX2 can bind in a complex with MYC for synergistic upregulation of gene targets in medulloblastoma^35^, in conjunction with an enrichment of MYC target genes observed here, further studies are warranted to explore this possible relationship. In small cell lung carcinomas, OTX2 was also associated with MYC activity, though this was regulated by NEUROD1 and not ASCL1^43^. Little is known about OTX2 in the context of prostate cancer, though mRNA levels have been observed to be elevated in a small cell carcinoma prostate PDX model^44^. Further, *OTX2* displays significantly differential methylation status in prostate cancer brain metastases compared to normal prostate tissue^45^ suggesting that OTX2 may have a role to play in brain tropism.

Our analysis of transcription factors also highlights the possible importance of FOXA1 in the AMPC subtype, in which FOXA1 may be preventing further transition to NEPC^46^ or be undergoing reprogramming^47^. Further studies are warranted to understand the regulation of this balance between CRPC-AR and NEPC lineage states in AMPC, as perhaps there are targetable, dual mechanisms also at work as recent evidence has suggested for ONECUT2^48^. Notably, this finding was not observed in the transcript levels of *FOXA1* directly, but rather in the proteome. This may be due to discordance between transcript and protein expression^49^ or additional post-translational regulatory mechanisms that upregulate FOXA1 protein activity but do not require upregulation of the *FOXA1* transcript. This finding would have been missed using traditional RNA-seq experiments and supports the need for proteome characterization, especially for understudied cancer subtypes such as AMPC. Molecular characterization at the proteome level also pointed to a potential novel role for NFYB in NEPC. NFYB is one of three subunits that form the heterotrimer complex, NFY, which drives transcription of cell cycle genes, especially those regulating G2M phase^50^ which we also observed to be enriched in the metastatic brain NEPC lesion. All three subunits (NFYA/B/C) were detected in the metastatic brain NEPC at the protein level, and perhaps through influence of other cofactors or microenvironmental signals, have adapted during metastasis to the brain. Interestingly, NYFB has been suggested as a therapeutic target in glioblastoma due to its involvement in proliferation and oxidative phosphorylation^51^, and further studies are warranted to explore NYFB’s role in promoting NEPC and/or brain tropism.

There are limited studies that molecularly characterize brain metastases in prostate cancer patients^4^. Previous investigation into the genomics of brain metastases identified higher frequency of NOTCH aberrations (12%) as a unique feature of prostate cancer brain metastasis compared to other metastatic sites (5%)^52^. However, here we didn’t observe any mutations in the NOTCH pathway or dysregulation of NOTCH signaling at the RNA or protein level. Further characterization of 51 prostate cancer brain metastases noted at least one genomic alteration in 15 homologous recombination repair genes^53^ from the PROfound clinical trial for PARP inhibitors^54^. We similarly observed a slight loss of copy number in the NEPC brain metastatic lesion as compared to the primary prostate in two of these genes, *CHEK2* and *RAD54L*, suggesting potential benefit from PARP inhibition. Other work highlighted reduced expression of PTEN and ERG proteins in brain metastasis from prostate cancer, with loss of ERG protein being an especially important marker^55^. In our case, we observed a loss of *PTEN* copy number in the brain metastasis, along with *RB1* loss and *TP53* inactivation. We also saw a 3.6-fold reduction in the RNA transcript expression of *ERG* in the brain metastasis as compared to the primary prostate, supporting the potential association of reduced ERG expression in brain metastasis.

In summary, our multi-omics case study provides a unique and valuable resource of deep molecular characterization of a metastatic brain NEPC tumor, a metastatic dural AMPC lesion, and a high-grade primary prostate cancer. Our work highlights the need for more investigation into the AMPC subtype, especially at the protein level, as it may be in an early transitionary state towards NEPC or may seed further NEPC metastases. Our multi-omics analysis suggests that there may be early expression markers at both the transcript and proteome levels indicative of this potential lineage plasticity, such as ASCL1 or CHGA status. Other NE signatures, such as the NE score^29^, may not provide enough discrimination as AMPC still retains several aspects of its prostate luminal-cell identity. Pioneer transcription factors (such as ASCL1 or OTX2) capable of reprogramming cancer cells may identify novel transcriptional and proteomic profiles that could be targeted to inhibit complete transdifferentiation into a pure small cell/neuroendocrine lineage state.

## Methods

### Patient approval

This case study was conducted in accordance with the Declaration of Helsinki and was performed under a protocol approved by the University of Minnesota ethics committee and institutional review board (Study 00013584: Evaluation of Genomic Events in Prostate Cancer). The patient provided written informed consent to participate.

### Whole exome sequencing (WES) data processing

Raw read (FASTQ) and exome targeting (BED) files were obtained from Caris Life Sciences (Irving, Texas) and processed as previously described^56^ using nf-core/sarek (3.4.0) workflows^57^. In short, reads were quality-assessed and trimmed using FastQC (0.12.1) and FastP (0.23.24) respectively before mapping to the GRCh38 reference genome with BWA (0.7.17-r1188). Duplicate reads were removed, base quality scores were recalibrated, and strand bias was accounted for using Genome Analysis Toolkit (GATK) best practices^58^. GATK Mutect2 was used for somatic variant calling, with a required variant read depth ≥50, allele frequency ≥0.01, and pass Mutect2 filter. Variants were annotated by Ensembl Variant Effect Predictor (v110)^59^ (Supplementary Table 1). As we lacked a normal sample, we used the primary prostate as a reference as a more robust measurement of true changes to copy numbers in the metastases. A list of reference and germline polymorphic variants for GRCh38 was acquired from the dbSNP database to calculate their associated read depths for each sample via snp-pileup (0.5.2). FACETS (0.6.2)^60^ calculated log2 copy ratio of the dural adenocarcinoma or brain NEPC samples as compared to the primary prostate, with areas too low (<50) or too excessive (>1000) in coverage removed (Supplementary Table 2). These data processing steps were conducted using the Minnesota Supercomputing Institute. FACETS-suite R package (2.0.8) was used to subset the copy number calls per gene and plot the log2 copy ratio using ComplexHeatmap in R (2.20.0), with a log2 copy ratio ±0.5 to indicate gain or loss from primary.

### Whole transcriptome sequencing (WTS) data processing

Raw FASTQ files were obtained from Caris Life Sciences (Irving, Texas) and preprocessed as previously described^56^. Transcriptome sequence data processing and analysis were performed using pipelines at the Minnesota Supercomputing Institute and University of Minnesota Informatics Institute (UMII) at the University of Minnesota. Raw reads were trimmed, aligned to the GRCh38 human genome, and gene-level read counts were generated using the CHURP pipeline. Genes with zero expression across all three samples were filtered out and DESeq2 (1.42.0) normalized counts were calculated for use in downstream transcriptomic analyses and visualizations (Supplementary Tables 3 and 4). These downstream gene expression analyses and visualizations were conducted using R (4.3.2) and RStudio (2022.12.0 + 353).

### Proteomics sample preparation and mass spectrometry (MS) analysis

Tissues were H&E stained, cut into 10 µm thick slides, and relevant sections of tissue were collected via laser capture microdissection (Leica LMD6500). Sample preparation for mass spectrometry was adapted^61^ with modifications specified. The samples were lysed with 50% Trifluoroethanol (Sigma Aldrich, 96924) and 300 mM Tris (Sigma Aldrich, T1503) at pH 8.5. Samples were then sonicated, incubated for 1 h at 90 °C, incubated overnight at 65 °C, and again sonicated. 10 mM Chloroacetamide (Sigma Aldrich, C0267) and 5 mM tris(2-carboxyethyl)phosphine (Thermo Scientific, 77720) were added at room temperature. The samples were vacuum dried and reconstituted with mass spectrometry grade water (Thermo Scientific, 51140), then digested with lysyl endopeptidase (WAKO, 125-05061) at 1:100 and trypsin (Worthington, LSO2115) at 1:75 ratio of enzyme to protein overnight. Peptides were cleaned using reverse phase MCX chemistry (Waters, 186000254) prior to injection on Thermo Scientific™ Orbitrap Eclipse with high field asymmetric ion mobility spectrometry (FAIMS) interface mass spectrometer. Samples were run in triplicate, and raw MS files were analyzed using MaxQuant (v1.6.10.43)^62^. MS/MS fragmentation spectra were searched using ANDROMEDA as previously described^49,63^. Peptide and protein identifications were collated as previously described for proteomic data^49^. In short, we used our in-house pipeline to average the intensity of peptides that mapped to the same protein, performed variance-stabilized normalization^64,65^, and then imputed missing values based on the lowest 1% quantile. Quantitative data for peptide and protein identifications are in Supplementary Tables 5–7.

### Hierarchical clustering

Hierarchical clustering on centered and normalized data was performed using Cluster 3.0 with Pearson correlation and average linkage^66^. Clustering results were visualized with ComplexHeatmap (2.18.0) or GraphPad Prism (v10).

### Activity and cell lineage scores

AR activity, NE activity, and luminal scores were calculated by summative z-score of defined gene sets^28^. The luminal scores were further scaled such that 0 was the lowest score and 100 was the highest. The NE score as in Fig. 3b was calculated by Pearson correlation with the CRPC-NE dataset as described^29^.

### Gene set enrichment analysis (GSEA)

For GSEA, the difference between RNA normalized counts was calculated and divided by the mean expression value which were then used to generate a ranked profile and conduct a pre-ranked GSEA using Hallmark Signatures^56^ as described^67^. Full outputs of GSEA analysis are in Supplementary Tables 8–10.

## Supplementary information

Supplement

## Data Availability

The whole-exome DNA sequencing and whole-transcriptome sequencing was performed at Caris® Life Sciences, and the data is available at the Sequence Read Archive (SRA) under BioProject Accession number PRJNA1163704. The proteomics data is available at Mass Spectrometry Interactive Virtual Environment (MassIVE) under MSV000095007.

## Code availability

The basic operational source code is available at Zenodo Digital Repository (10.5281/zenodo.13821252).
