# Supplementary material for: Integrated multi-omics assessment of lineage plasticity in a prostate cancer patient with brain and dural metastases
Source: NPJ Precis Oncol. 2024 Sep 30;8:215. doi: 10.1038/s41698-024-00713-8 (PMC11443004; doi:10.1038/s41698-024-00713-8)
Supplement: Supplementary file 1 — Supplement [file 41698_2024_713_MOESM1_ESM.pdf]

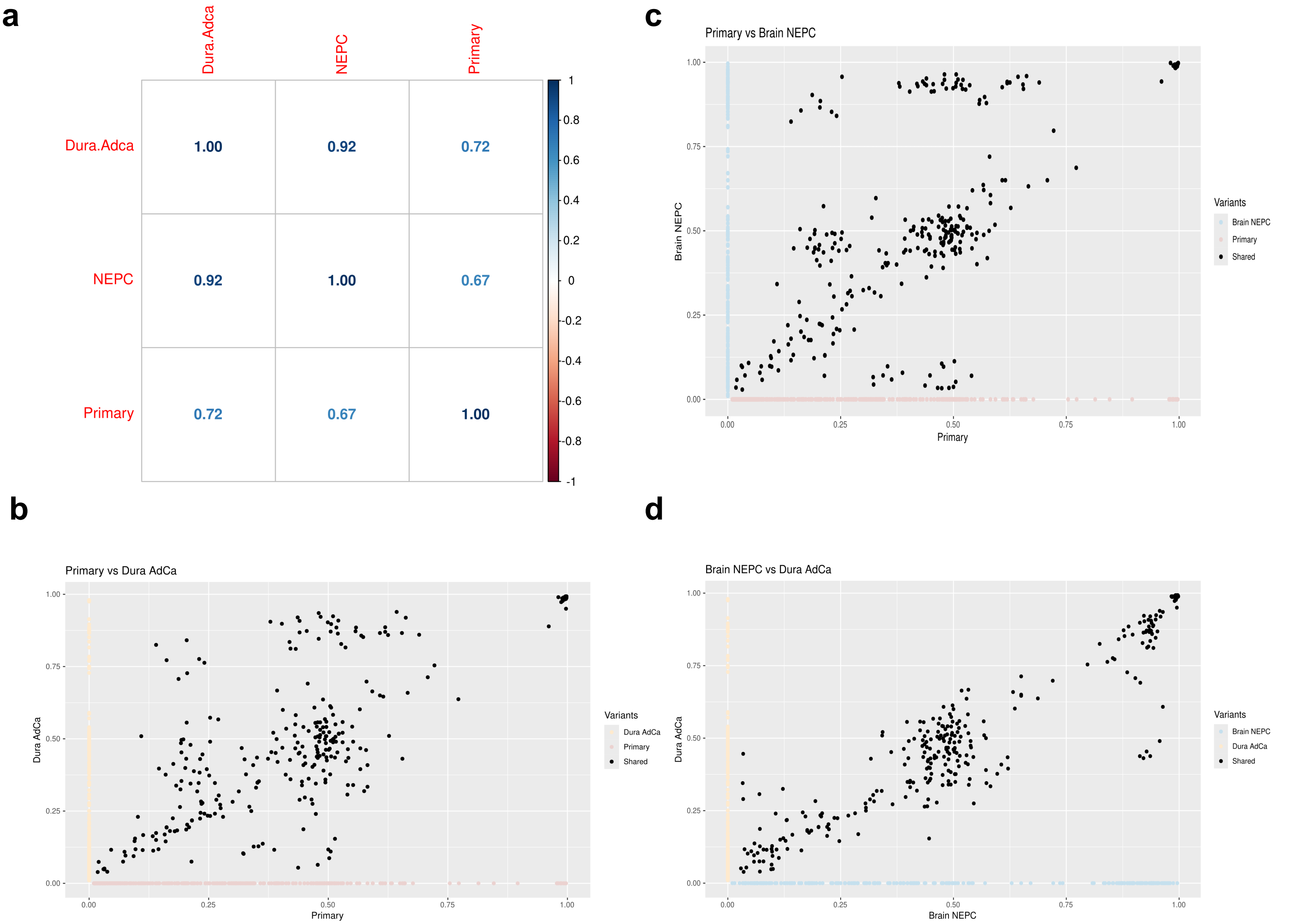

Supplementary Fig 1. a) Pearson correlation of variant allele frequencies for variants shared across all samples. P-values for all comparisons were < 0.0001. Variants plotted based on allele frequencies between **b)** primary prostate and dural adenocarcinoma, **c)** primary prostate and brain NEPC or **d)** brain NEPC and dural adenocarcinoma. Shared variants are noted in black, and those unique to a sample colored for primary prostate (red), dura adenocarcinoma (yellow) and brain NEPC (blue).

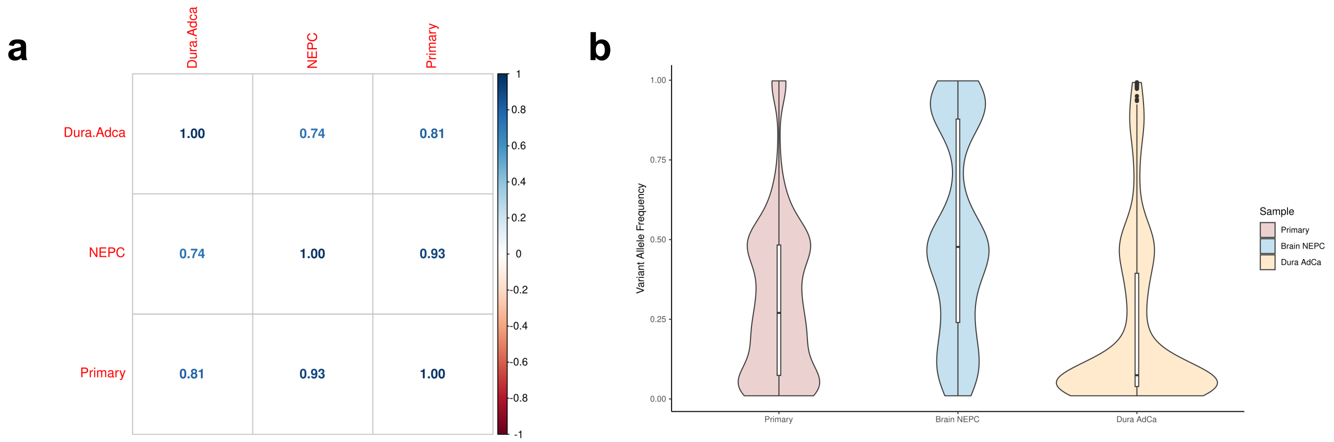

Supplementary Fig 2. a) Pearson correlation of depth of coverage for variants shared for all samples. P-values for all comparisons were < 0.0001. **b)** Violin plot of all variants that passed filters (read depth ≥ 50, allele frequence ≥ 0.01, and Mutect2 PASS) for each sample.

a Spearman correlation of transcriptome

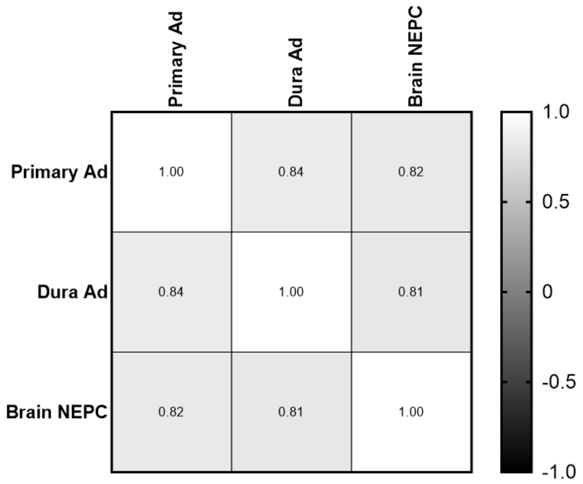

b Spearman correlation of proteome

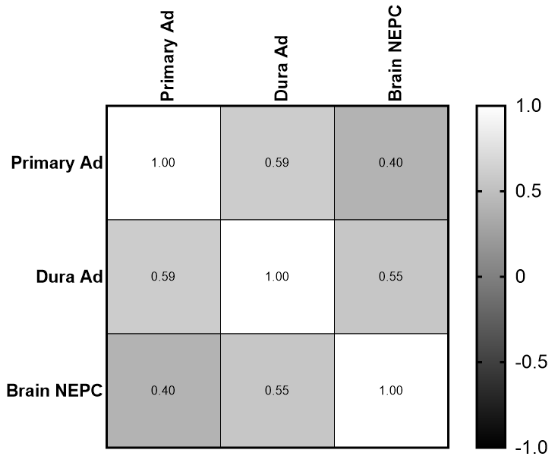

Supplementary Fig 3. Spearman correlation of transcriptome **(a)** or proteome **(b)** for genes shared across all samples. P-values for all comparisons for a and b are <0.0001.

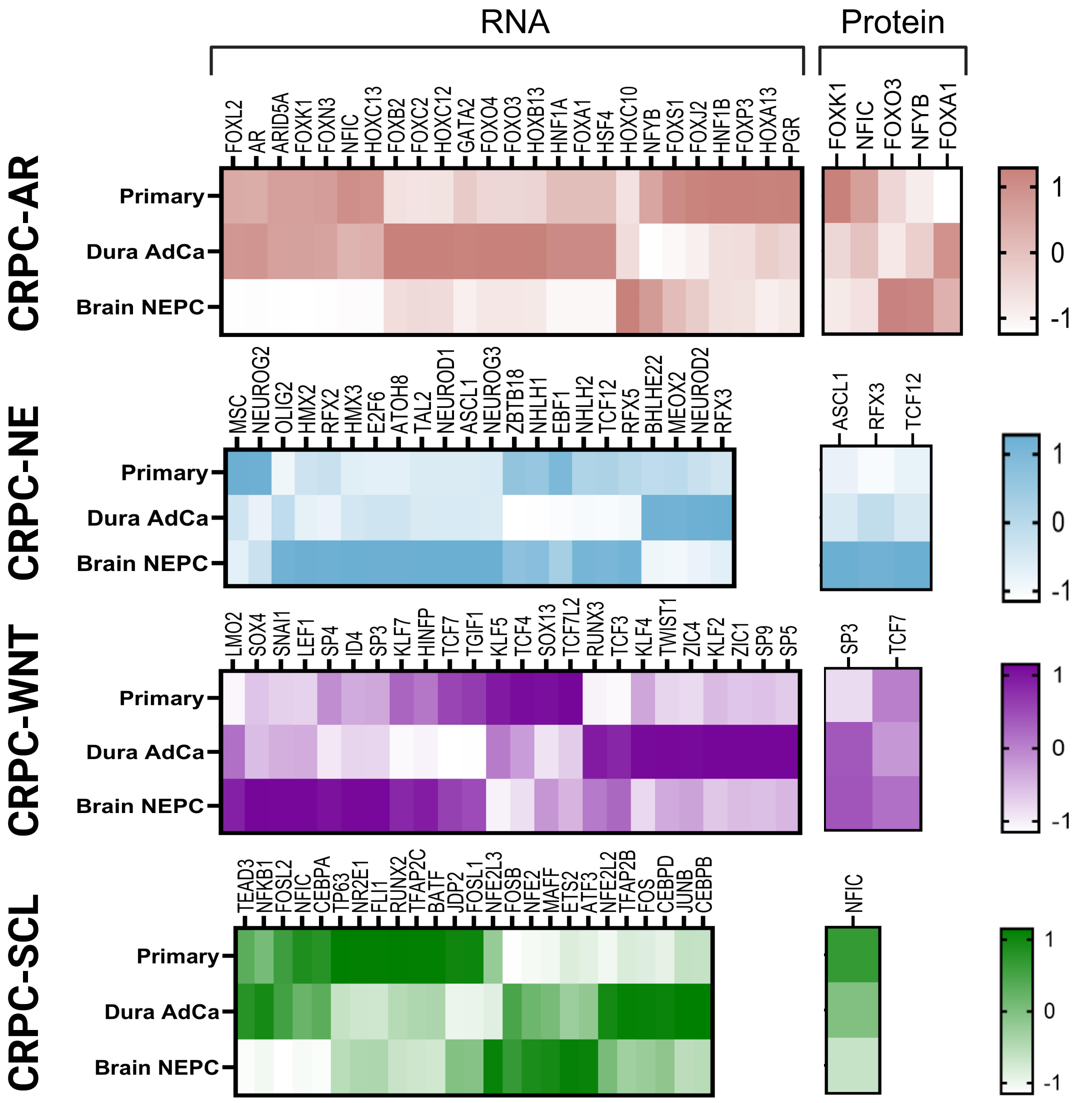

Supplementary Fig 4. Heatmap of transcription factor expression regulating the four CRPC subtypes as indicated for the transcriptome (left) and proteome (right).

Supplementary Table 1. Genomic mutations with read depth ≥50, allele frequency ≥0.01, and passed Mutect2 filter.

Chromosome	Position	Alternate	Reference	Allele Frequency (AF)	Total depth of coverage (DP)	Filter	Gene_Symbol	Consequence	Sample
chr1	17594	T	C	0.217	224	PASS	WASH7P	intron_variant&non_coding_transcript_variant	Primary
chr1	183274	A	G	0.14	88	PASS	DDX11L17	intron_variant&non_coding_transcript_variant	Primary
chr1	184142	T	C	0.038	289	PASS	DDX11L17	non_coding_transcript_exon_variant	Primary
chr1	999495	T	C	0.582	166	PASS	HES4	intron_variant	Primary
chr1	1223422	G	A	0.655	200	PASS	SDF4	intron_variant	Primary
chr1	1293731	ACGCCC(	A	0.985	57	PASS	ACAP3	intron_variant	Primary
chr1	11648799	T	C	0.66	329	PASS	FBXO2	synonymous_variant	Primary
chr1	12883168	TG	CA	0.566	352	PASS	PRAMEF4	missense_variant	Primary
chr1	13224919	A	T	0.235	739	PASS	PRAMEF18	missense_variant	Primary
chr1	13225068	G	A	0.232	1699	PASS	PRAMEF18	missense_variant	Primary
chr1	13225208	G	C	0.241	3417	PASS	PRAMEF18	synonymous_variant	Primary
chr1	13318650	G	A	0.175	564	PASS	PRAMEF15	synonymous_variant	Primary
chr1	16646619	A	AGCCAT(	0.145	1989	PASS	CROCCP2	intron_variant&non_coding_transcript_variant	Primary
chr1	16647559	G	A	0.016	1160	PASS	CROCCP2	intron_variant&non_coding_transcript_variant	Primary
chr1	16761036	A	G	0.169	1925	PASS	CROCC	intron_variant&non_coding_transcript_variant	Primary
chr1	21424888	A	C	0.074	188	PASS	NBPF2P	intron_variant&non_coding_transcript_variant	Primary
chr1	26166079	A	G	0.459	364	PASS	C1orf232	missense_variant	Primary
chr1	26780633	T	C	0.075	4053	PASS	ARID1A	synonymous_variant	Primary
chr1	33569487	G	A	0.201	744	PASS	CSMD2	missense_variant	Primary
chr1	34900204	A	G	0.021	340	PASS	DLGAP3	missense_variant	Primary
chr1	36308186	A	G	0.493	406	PASS	SH3D21	missense_variant	Primary
chr1	37690239	C	T	0.012	485	PASS	C1orf109	5_prime_utr_variant	Primary
chr1	37690242	C	T	0.012	493	PASS	C1orf109	5_prime_utr_variant	Primary
chr1	39413978	T	C	0.054	125	PASS	MACF1	intron_variant	Primary
chr1	39413989	G	A	0.043	131	PASS	MACF1	intron_variant	Primary
chr1	44139048	TCTGGTT	T	0.981	2255	PASS	KLF18	inframe_insertion	Primary
chr1	50419109	A	G	0.089	72	PASS	DMRTA2	synonymous_variant	Primary
chr1	55092049	C	A	0.451	184	PASS	USP24	synonymous_variant	Primary
chr1	65254515	T	G	0.393	203	PASS	DNAJC6	intron_variant&non_coding_transcript_variant	Primary
chr1	65610151	GAGAAG.	G	0.033	203	PASS	LEPR	intron_variant	Primary
chr1	75339642	C	A	0.05	60	PASS	SLC44A5	splice_polypyrimidine_tract_variant&intron_variant	Primary
chr1	99686767	T	C	0.077	53	PASS	PALMD	missense_variant	Primary
chr1	99712018	T	C	0.203	108	PASS	FRRS1	intron_variant	Primary
chr1	111231179	C	T	0.516	393	PASS	CHI3L2	intron_variant	Primary
chr1	120258388	C	T	0.058	309	PASS	.	intron_variant&non_coding_transcript_variant	Primary
chr1	120543014	C	T	0.404	225	PASS	.	splice_donor_region_variant&intron_variant&non_codin	Primary
chr1	120814068	T	C	0.103	235	PASS	NBPF26	intron_variant	Primary
chr1	120890143	G	A	0.032	1584	PASS	PPIAL4A	missense_variant	Primary
chr1	143796246	C	T	0.994	185	PASS	LINC02591	non_coding_transcript_exon_variant	Primary
chr1	145241802	C	T	0.567	661	PASS	PPIAL4D	missense_variant	Primary
chr1	145292353	C	T	0.134	285	PASS	NBPF20	intron_variant	Primary
chr1	145292462	A	T	0.087	179	PASS	NBPF20	missense_variant	Primary
chr1	145588056	G	C	0.018	762	PASS	NBPF25P	non_coding_transcript_exon_variant	Primary
chr1	146622487	C	G	0.426	82	PASS	.	non_coding_transcript_exon_variant	Primary
chr1	147631053	G	C	0.451	167	PASS	ACP6	stop_lost	Primary
chr1	147968240	A	G	0.381	57	PASS	GPR89B	intron_variant	Primary
chr1	148536251	C	G	0.084	58	PASS	NBPF14	missense_variant	Primary
chr1	149176847	C	T	0.483	134	PASS	.	intron_variant&non_coding_transcript_variant	Primary
chr1	149607944	C	G	0.203	201	PASS	.	intron_variant&non_coding_transcript_variant	Primary
chr1	149607947	C	G	0.201	201	PASS	LINC00869	intron_variant&non_coding_transcript_variant	Primary
chr1	151368992	T	C	0.197	540	PASS	SELENBP1	intron_variant	Primary
chr1	152213623	T	C	0.615	567	PASS	HRNR	missense_variant	Primary
chr1	152214116	G	T	0.052	182	PASS	HRNR	missense_variant	Primary
chr1	152214592	T	G	0.097	3472	PASS	HRNR	missense_variant	Primary
chr1	152216443	T	C	0.065	1276	PASS	HRNR	missense_variant	Primary
chr1	152351895	T	G	0.02	1838	PASS	FLG2	missense_variant	Primary
chr1	155178123	T	C	0.017	484	PASS	TRIM46	missense_variant	Primary
chr1	159066010	C	G	0.146	532	PASS	AIM2	missense_variant	Primary
chr1	160031429	C	A	0.476	402	PASS	PIGM	missense_variant	Primary
chr1	160195116	T	C	0.431	262	PASS	CASQ1	synonymous_variant	Primary
chr1	160752273	TG	CA	0.515	153	PASS	SLAMF7	intron_variant	Primary
chr1	161543282	G	A	0.044	182	PASS	FCGR3A	intron_variant	Primary
chr1	171185664	A	G	0.486	116	PASS	.	intron_variant&non_coding_transcript_variant	Primary
chr1	174220968	A	G	0.482	183	PASS	RABGAP1L	splice_region_variant&splice_polypyrimidine_tract_varia	Primary
chr1	201209796	G	A	0.302	762	PASS	IGFN1	missense_variant	Primary
chr1	202919887	G	A	0.014	606	PASS	KLHL12	missense_variant	Primary
chr1	205984165	T	G	0.997	474	PASS	RAB7B	intron_variant	Primary
chr1	209630592	C	G	0.169	522	PASS	LAMB3	intron_variant	Primary
chr1	212386985	A	G	0.343	592	PASS	PACC1	synonymous_variant	Primary
chr1	212780065	C	G	0.111	70	PASS	NSL1	intron_variant	Primary
chr1	214607263	C	T	0.07	59	PASS	CENPF	intron_variant	Primary
chr1	220599753	G	A	0.319	90	PASS	MARK1	intron_variant	Primary
chr1	224191000	G	C	0.133	169	PASS	DEGS1	intron_variant	Primary
chr1	235504390	A	G	0.593	102	PASS	B3GALNT2	5_prime_utr_variant	Primary
chr1	242449317	G	A	0.298	445	PASS	PLD5	intron_variant	Primary
chr1	243165585	T	G	0.133	315	PASS	CEP170	stop_gained	Primary
chr1	243165909	T	G	0.056	203	PASS	CEP170	missense_variant	Primary
chr1	243172878	T	C	0.109	159	PASS	CEP170	intron_variant	Primary
chr1	248453100	A	G	0.162	2688	PASS	OR2T2	synonymous_variant	Primary
chr1	248453107	T	C	0.162	2713	PASS	OR2T2	missense_variant	Primary
chr1	248453582	A	T	0.012	1271	PASS	OR2T2	missense_variant	Primary
chr2	17714908	T	C	0.473	291	PASS	SMC6	synonymous_variant	Primary
chr2	24161505	T	G	0.346	278	PASS	FAM228B	splice_acceptor_variant	Primary
chr2	25130488	C	A	0.522	513	PASS	EFR3B	intron_variant	Primary

chr2	26284269	GTGGTA	G	0.044	150	PASS	HADHB	intron_variant	Primary
chr2	27206532	T	C	0.496	438	PASS	SLC5A6	splice_region_variant&synonymous_variant	Primary
chr2	31178617	G	T	0.452	316	PASS	CAPN14	intron_variant	Primary
chr2	39707026	A	G	0.272	305	PASS	TMEM178A	intron_variant	Primary
chr2	43801586	A	G	0.251	85	PASS	DYNC2LI1	intron_variant	Primary
chr2	55208654	A	C	0.025	264	PASS	CLHC1	missense_variant	Primary
chr2	68655630	A	T	0.333	207	PASS	PROKR1	3_prime_utr_variant	Primary
chr2	69479044	A	C	0.338	230	PASS	AAK1	missense_variant	Primary
chr2	73214257	G	C	0.307	1027	PASS	SMYD5	5_prime_utr_variant	Primary
chr2	73214385	T	C	0.316	637	PASS	SMYD5	intron_variant	Primary
chr2	74088024	T	C	0.067	82	PASS	TET3	synonymous_variant	Primary
chr2	75646803	G	C	0.608	488	PASS	MRPL19	5_prime_utr_variant	Primary
chr2	80302939	A	T	0.074	1174	PASS	LRRTM1	missense_variant	Primary
chr2	87130049	G	T	0.328	366	PASS	.	intron_variant&non_coding_transcript_variant	Primary
chr2	89640376	T	C	0.248	152	PASS	KMT2CP4	intron_variant&non_coding_transcript_variant	Primary
chr2	96840093	A	G	0.016	809	PASS	ANKRD39	splice_region_variant&3_prime_utr_variant&nmd_trans	Primary
chr2	108765714	G	A	0.028	626	PASS	RANBP2	synonymous_variant	Primary
chr2	111123759	A	C	0.514	930	PASS	BCL2L11	missense_variant	Primary
chr2	111150001	A	G	0.483	2342	PASS	BCL2L11	intron_variant	Primary
chr2	111150163	G	T	0.501	2403	PASS	MIR4435-2HG	intron_variant&non_coding_transcript_variant	Primary
chr2	121387253	A	G	0.316	387	PASS	CLASP1	intron_variant	Primary
chr2	124790192	T	C	0.034	297	PASS	CNTNAP5	intron_variant	Primary
chr2	131626767	A	G	0.343	589	PASS	POTEKP	non_coding_transcript_exon_variant	Primary
chr2	134936927	G	T	0.323	554	PASS	CCNT2	synonymous_variant	Primary
chr2	135162954	A	G	0.307	453	PASS	ZRANB3	intron_variant&non_coding_transcript_variant	Primary
chr2	143587732	T	G	0.061	95	PASS	ARHGAP15	intron_variant	Primary
chr2	148483570	C	T	0.643	785	PASS	MBD5	synonymous_variant	Primary
chr2	163611058	T	C	0.478	661	PASS	FIGN	synonymous_variant	Primary
chr2	206444098	A	C	0.391	94	PASS	ADAM23	intron_variant	Primary
chr2	227523753	CTTTTAT	C	0.028	236	PASS	AGFG1	splice_polypyrimidine_tract_variant&intron_variant	Primary
chr2	241230797	A	G	0.045	346	PASS	HDLBP	missense_variant	Primary
chr3	9918131	A	G	0.03	166	PASS	.	intron_variant&non_coding_transcript_variant	Primary
chr3	10213059	CA	TG	0.54	121	PASS	IRAK2	intron_variant	Primary
chr3	11017287	G	A	0.51	1201	PASS	SLC6A1	missense_variant	Primary
chr3	12004864	G	C	0.467	147	PASS	SYN2	missense_variant	Primary
chr3	13503039	A	G	0.263	288	PASS	HDAC11	intron_variant	Primary
chr3	14158878	C	G	0.496	881	PASS	XPC	synonymous_variant	Primary
chr3	14178475	T	C	0.504	1527	PASS	XPC	missense_variant	Primary
chr3	16885062	A	G	0.508	54	PASS	PLCL2	missense_variant	Primary
chr3	27344259	C	G	0.109	200	PASS	NEK10	intron_variant	Primary
chr3	38412994	G	C	0.218	437	PASS	XYLB	missense_variant	Primary
chr3	46673502	A	G	0.521	344	PASS	ALS2CL	intron_variant	Primary
chr3	47857472	C	G	0.162	469	PASS	MAP4	missense_variant	Primary
chr3	49005095	C	A	0.455	884	PASS	P4HTM	intron_variant	Primary
chr3	51385335	G	T	0.404	65	PASS	MANF	5_prime_utr_variant	Primary
chr3	52806206	A	C	0.146	216	PASS	ITIH3	intron_variant	Primary
chr3	54969807	T	C	0.186	525	PASS	CACNA2D3	missense_variant	Primary
chr3	57368206	C	T	0.527	168	PASS	DNAH12	synonymous_variant	Primary
chr3	57377088	G	A	0.505	140	PASS	DNAH12	synonymous_variant	Primary
chr3	57380252	A	G	0.506	157	PASS	DNAH12	intron_variant	Primary
chr3	57556401	G	C	0.039	668	PASS	PDE12	missense_variant	Primary
chr3	64147434	G	C	0.01	841	PASS	PRICKLE2	synonymous_variant	Primary
chr3	85961641	T	G	0.032	232	PASS	CADM2	missense_variant	Primary
chr3	88139852	G	A	0.467	183	PASS	ZNF654	missense_variant	Primary
chr3	101671336	T	C	0.212	271	PASS	ZBTB11	missense_variant	Primary
chr3	101699987	A	C	0.173	68	PASS	PDCL3P4	non_coding_transcript_exon_variant	Primary
chr3	124699935	A	G	0.494	534	PASS	KALRN	missense_variant	Primary
chr3	132691283	C	A	0.193	76	PASS	NPHP3	missense_variant	Primary
chr3	179973339	G	A	0.192	226	PASS	.	intron_variant&non_coding_transcript_variant	Primary
chr3	184038505	C	T	0.179	463	PASS	HTR3D	missense_variant	Primary
chr3	184038509	C	T	0.184	457	PASS	HTR3D	synonymous_variant	Primary
chr3	195618957	GA	AG	0.627	373	PASS	MUC20P1	non_coding_transcript_exon_variant	Primary
chr3	195740966	G	A	0.226	227	PASS	MUC20	downstream_gene_variant	Primary
chr3	195740982	T	C	0.243	174	PASS	MUC20	downstream_gene_variant	Primary
chr3	195780885	G	C	0.282	1364	PASS	MUC4	missense_variant	Primary
chr3	195780891	G	T	0.305	1473	PASS	MUC4	synonymous_variant	Primary
chr3	195781775	C	CGGTGA	0.631	577	PASS	MUC4	inframe_deletion	Primary
chr3	195784172	C	T	0.203	3986	PASS	MUC4	missense_variant	Primary
chr3	195784271	C	T	0.326	2663	PASS	MUC4	missense_variant	Primary
chr3	195786231	G	A	0.565	3144	PASS	MUC4	synonymous_variant	Primary
chr3	195786336	G	A	0.33	2728	PASS	MUC4	synonymous_variant	Primary
chr4	867135	A	G	0.677	272	PASS	GAK	missense_variant	Primary
chr4	1395596	C	CGCCTG	0.013	1062	PASS	UVSSA	3_prime_utr_variant	Primary
chr4	2251199	A	C	0.381	287	PASS	MXD4	synonymous_variant	Primary
chr4	9230471	A	C	0.032	3881	PASS	.	intron_variant&non_coding_transcript_variant	Primary
chr4	9230473	C	T	0.03	3806	PASS	.	intron_variant&non_coding_transcript_variant	Primary
chr4	9248548	A	G	0.065	1288	PASS	.	intron_variant&non_coding_transcript_variant	Primary
chr4	9268697	T	C	0.112	169	PASS	USP17L22	missense_variant	Primary
chr4	16186869	C	G	0.61	54	PASS	TAPT1	missense_variant	Primary
chr4	64299993	T	A	0.051	74	PASS	TECRL	missense_variant	Primary
chr4	70637844	T	G	0.541	254	PASS	ENAM	splice_donor_variant	Primary
chr4	74038340	A	T	0.246	843	PASS	CXCL3	missense_variant	Primary
chr4	76740701	T	A	0.476	754	PASS	SHROOM3	missense_variant	Primary
chr4	86771504	G	A	0.021	203	PASS	PTPN13	missense_variant	Primary
chr4	87810662	A	G	0.035	167	PASS	IBSP	synonymous_variant	Primary
chr4	108869028	A	G	0.069	72	PASS	COL25A1	intron_variant	Primary

chr4	122201050	A	G	0.194	192	PASS	BLTP1	synonymous_variant	Primary
chr4	150928642	A	T	0.219	51	PASS	LRBA	intron_variant	Primary
chr4	155710866	T	C	0.53	642	PASS	GUCY1A1	missense_variant	Primary
chr4	185471082	T	C	0.465	120	PASS	CCDC110	intron_variant	Primary
chr4	186706900	G	C	0.65	3074	PASS	FAT1	synonymous_variant	Primary
chr4	186709443	G	C	0.633	2757	PASS	FAT1	missense_variant	Primary
chr4	189982875	CT	TG	0.179	149	PASS	TUBB7P	non_coding_transcript_exon_variant	Primary
chr5	181614	TG	CC	0.405	530	PASS	PLEKHG4B	missense_variant	Primary
chr5	840458	T	C	0.376	1430	PASS	ZDHHC11	intron_variant	Primary
chr5	878575	T	C	0.253	370	PASS	BRD9	intron_variant	Primary
chr5	1432496	G	A	0.51	347	PASS	SLC6A3	synonymous_variant	Primary
chr5	10239213	T	C	0.014	1306	PASS	ATPSCKMT	missense_variant	Primary
chr5	23526890	G	C	0.047	851	PASS	PRDM9	missense_variant	Primary
chr5	32780689	G	C	0.481	478	PASS	NPR3	intron_variant	Primary
chr5	57214174	A	C	0.573	439	PASS	GPBP1	missense_variant	Primary
chr5	65452748	A	G	0.034	182	PASS	ADAMTS6	missense_variant	Primary
chr5	65552595	T	C	0.054	50	PASS	CENPK	intron_variant	Primary
chr5	71012424	T	C	0.496	820	PASS	NAIP	synonymous_variant	Primary
chr5	77077415	GC	AG	0.474	82	PASS	ZBED3	missense_variant	Primary
chr5	87268448	T	C	0.321	74	PASS	RASA1	5_prime_utr_variant	Primary
chr5	109820217	C	T	0.06	153	PASS	MAN2A1	splice_region_variant&splice_polypyrimidine_tract_varia	Primary
chr5	111092331	T	C	0.387	540	PASS	.	upstream_gene_variant	Primary
chr5	112836522	A	G	0.264	782	PASS	APC	intron_variant	Primary
chr5	132696603	A	AATTTTT	0.221	119	PASS	KIF3A	3_prime_utr_variant	Primary
chr5	140648249	G	A	0.363	256	PASS	IK	intron_variant	Primary
chr5	141194636	G	C	0.773	245	PASS	PCDHB10	missense_variant	Primary
chr5	156660433	T	C	0.059	232	PASS	SGCD	intron_variant	Primary
chr5	159174135	A	G	0.385	132	PASS	RNF145	synonymous_variant	Primary
chr5	169591213	CAATGCT	C	0.063	83	PASS	SPDL1	stop_gained&frameshift_variant	Primary
chr5	169591216	ACCAATA	A	0.063	82	PASS	SPDL1	stop_gained&protein_altering_variant	Primary
chr5	172968611	T	G	0.328	582	PASS	RPL26L1	intron_variant	Primary
chr5	175442865	A	G	0.011	707	PASS	DRD1	missense_variant	Primary
chr5	175926725	C	T	0.06	693	PASS	.	intron_variant&non_coding_transcript_variant	Primary
chr5	176010999	C	T	0.252	495	PASS	THOC3	intron_variant	Primary
chr5	177672139	A	T	0.142	315	PASS	.	non_coding_transcript_exon_variant	Primary
chr5	178147850	A	G	0.582	1029	PASS	RMND5B	missense_variant	Primary
chr5	179076409	T	G	0.542	974	PASS	ZNF354C	intron_variant	Primary
chr5	179125085	G	T	0.013	2044	PASS	ADAMTS2	missense_variant	Primary
chr6	147522	A	G	0.263	261	PASS	SEPTIN14P6	upstream_gene_variant	Primary
chr6	311798	G	T	0.203	348	PASS	DUSP22	intron_variant	Primary
chr6	4125973	T	C	0.436	275	PASS	ECI2	intron_variant	Primary
chr6	4996429	A	C	0.506	105	PASS	RPP40	splice_polypyrimidine_tract_variant&intron_variant	Primary
chr6	25777988	T	C	0.176	279	PASS	SLC17A4	missense_variant	Primary
chr6	30730973	CTGTATA	C	0.056	105	PASS	FLOT1	stop_gained&frameshift_variant	Primary
chr6	31027040	A	G	0.013	683	PASS	MUC22	missense_variant	Primary
chr6	31202450	GC	AG	0.482	560	PASS	HCG27	non_coding_transcript_exon_variant	Primary
chr6	31202488	G	A	0.054	766	PASS	HCG27	non_coding_transcript_exon_variant	Primary
chr6	31541266	T	G	0.501	160	PASS	DDX39B	intron_variant	Primary
chr6	31632919	A	G	0.529	271	PASS	PRRC2A	missense_variant	Primary
chr6	31688737	T	C	0.515	419	PASS	ABHD16A	synonymous_variant	Primary
chr6	32011008	T	C	0.074	530	PASS	TNXA	non_coding_transcript_exon_variant	Primary
chr6	32038540	T	C	0.503	200	PASS	CYP21A2	synonymous_variant	Primary
chr6	32038560	A	C	0.514	198	PASS	CYP21A2	synonymous_variant	Primary
chr6	32203761	A	G	0.512	244	PASS	NOTCH4	intron_variant	Primary
chr6	32223104	A	G	0.441	240	PASS	NOTCH4	intron_variant	Primary
chr6	34417760	A	G	0.237	460	PASS	RPS10	intron_variant	Primary
chr6	43299968	T	C	0.195	814	PASS	SLC22A7	synonymous_variant	Primary
chr6	47879234	A	T	0.496	1231	PASS	PTCHD4	missense_variant	Primary
chr6	54169507	T	A	0.059	50	PASS	MLIP	intron_variant	Primary
chr6	69697648	A	G	0.049	53	PASS	LMBRD1	splice_region_variant&splice_polypyrimidine_tract_varia	Primary
chr6	97064696	C	T	0.298	311	PASS	KLHL32	synonymous_variant	Primary
chr6	106975088	A	G	0.412	111	PASS	CD24	upstream_gene_variant	Primary
chr6	109145410	C	G	0.032	82	PASS	CEP57L1	intron_variant	Primary
chr6	111373273	AGTTTTT	A	0.029	218	PASS	REV3L	stop_gained&frameshift_variant	Primary
chr6	116121523	T	C	0.317	446	PASS	COL10A1	missense_variant	Primary
chr6	117403216	T	C	0.337	1611	PASS	ROS1	missense_variant	Primary
chr6	117519881	T	C	0.569	288	PASS	DCBLD1	missense_variant	Primary
chr6	142133970	A	G	0.477	223	PASS	GJE1	missense_variant	Primary
chr6	169667944	A	G	0.496	739	PASS	WDR27	intron_variant	Primary
chr7	2572660	AA	TC	0.432	60	PASS	IQCE	intron_variant	Primary
chr7	6144151	G	T	0.026	296	PASS	USP42	synonymous_variant	Primary
chr7	6918556	G	A	0.028	281	PASS	.	intergenic_variant	Primary
chr7	6918581	A	G	0.033	269	PASS	.	intergenic_variant	Primary
chr7	10983192	AT	TG	0.023	179	PASS	PHF14	intron_variant	Primary
chr7	10983202	GGTCTGA	G	0.025	160	PASS	PHF14	intron_variant	Primary
chr7	11376710	C	G	0.058	69	PASS	.	intron_variant&non_coding_transcript_variant	Primary
chr7	22798375	C	G	0.284	282	PASS	.	intergenic_variant	Primary
chr7	34969503	C	A	0.047	131	PASS	DPY19L1	missense_variant	Primary
chr7	34969508	A	G	0.056	132	PASS	DPY19L1	synonymous_variant	Primary
chr7	37225044	C	T	0.432	738	PASS	ELMO1	splice_polypyrimidine_tract_variant&intron_variant	Primary
chr7	38217025	T	TTGACAA	0.022	733	PASS	STARD3NL	inframe_deletion&splice_region_variant	Primary
chr7	44007335	A	G	0.04	1238	PASS	.	splice_region_variant&splice_polypyrimidine_tract_varia	Primary
chr7	48389244	C	A	0.358	291	PASS	ABCA13	intron_variant	Primary
chr7	56116313	T	A	0.521	77	PASS	NUPR2	start_lost	Primary
chr7	57132116	T	C	0.23	345	PASS	ZNF479	intron_variant	Primary
chr7	66877042	C	T	0.214	78	PASS	GTF2IP23	upstream_gene_variant	Primary

chr7	67284024	A	G	0.25	129	PASS	SPDYE21	intron_variant	Primary
chr7	73328589	A	G	0.488	184	PASS	FKBP6	synonymous_variant	Primary
chr7	74822869	T	G	0.017	549	PASS	GTF2IRD2	intron_variant	Primary
chr7	75112541	C	T	0.159	71	PASS	GTF2IRD2B	splice_donor_region_variant&intron_variant	Primary
chr7	77953963	G	C	0.517	225	PASS	PHTF2	intron_variant	Primary
chr7	97986932	T	C	0.397	447	PASS	OCM2	intron_variant	Primary
chr7	98193049	A	G	0.53	511	PASS	LMTK2	missense_variant	Primary
chr7	99531295	G	A	0.531	371	PASS	ZKSCAN5	synonymous_variant	Primary
chr7	99760901	G	A	0.476	192	PASS	CYP3A4	missense_variant	Primary
chr7	100682882	T	C	0.48	203	PASS	GIGYF1	intron_variant	Primary
chr7	100867817	C	T	0.497	110	PASS	TRIP6	intron_variant	Primary
chr7	100952493	G	A	0.349	855	PASS	MUC3A	synonymous_variant	Primary
chr7	100952498	T	C	0.352	845	PASS	MUC3A	missense_variant	Primary
chr7	100993860	A	G	0.167	56	PASS	MUC12	synonymous_variant	Primary
chr7	100996246	T	G	0.118	340	PASS	MUC12	missense_variant	Primary
chr7	100997978	T	G	0.105	1170	PASS	MUC12	missense_variant	Primary
chr7	101000917	T	G	0.132	276	PASS	MUC12	missense_variant	Primary
chr7	111521382	T	C	0.306	245	PASS	IMMP2L	synonymous_variant	Primary
chr7	111846967	A	G	0.48	163	PASS	DOCK4	intron_variant	Primary
chr7	128880045	A	C	0.502	397	PASS	KCP	missense_variant	Primary
chr7	130129605	C	T	0.5	392	PASS	KLHDC10	intron_variant	Primary
chr7	130549432	G	C	0.479	190	PASS	COPG2	intron_variant	Primary
chr7	130550642	T	C	0.505	311	PASS	COPG2	synonymous_variant	Primary
chr7	132508576	G	A	0.66	399	PASS	PLXNA4	missense_variant	Primary
chr7	142059474	C	T	0.391	1083	PASS	MGAM	splice_region_variant&synonymous_variant	Primary
chr7	142059540	C	T	0.402	1549	PASS	MGAM	synonymous_variant	Primary
chr7	142078849	T	C	0.284	1247	PASS	MGAM	synonymous_variant	Primary
chr7	142085767	G	C	0.042	1534	PASS	MGAM	intron_variant	Primary
chr7	142328735	G	A	0.014	994	PASS	TRBV6-1	missense_variant	Primary
chr7	142389438	C	T	0.093	1505	PASS	TRBV5-3	missense_variant	Primary
chr7	142463547	G	C	0.191	687	PASS	TRBV5-4	missense_variant	Primary
chr7	142564042	T	C	0.499	1429	PASS	TRBV12-4	synonymous_variant	Primary
chr7	142588240	A	G	0.44	452	PASS	TRBV14	synonymous_variant	Primary
chr7	142598094	T	C	0.44	522	PASS	TRBV16	intron_variant	Primary
chr7	142774511	T	C	0.352	1519	PASS	PRSS2	3_prime_utr_variant	Primary
chr7	142774515	CA	TG	0.355	1502	PASS	PRSS2	3_prime_utr_variant	Primary
chr7	142791707	C	G	0.046	859	PASS	TRBC1	missense_variant	Primary
chr7	142791866	A	G	0.102	1011	PASS	TRBC1	synonymous_variant	Primary
chr7	142801387	C	T	0.278	823	PASS	TRBC2	synonymous_variant	Primary
chr7	149005223	A	G	0.478	259	PASS	PDIA4	synonymous_variant	Primary
chr7	152252528	A	G	0.484	1812	PASS	KMT2C	intron_variant	Primary
chr8	166783	G	A	0.207	214	PASS	OR4F21	missense_variant	Primary
chr8	1680556	AT	GA	0.354	226	PASS	DLGAP2	intron_variant	Primary
chr8	8020009	C	G	0.062	180	PASS	FAM90A24P	missense_variant	Primary
chr8	8020012	G	C	0.122	194	PASS	FAM90A24P	missense_variant	Primary
chr8	12121949	G	A	0.098	74	PASS	FAM66D	intron_variant&non_coding_transcript_variant	Primary
chr8	12435885	T	C	0.076	282	PASS	FAM86B2	intron_variant	Primary
chr8	18872551	G	C	0.343	640	PASS	PSD3	missense_variant	Primary
chr8	22227651	T	C	0.536	132	PASS	PHYHIP	intron_variant	Primary
chr8	28041178	A	G	0.018	424	PASS	NUGGC	missense_variant	Primary
chr8	37848719	G	C	0.205	253	PASS	BRF2	intron_variant	Primary
chr8	42756343	G	A	0.56	751	PASS	CHRNA6	synonymous_variant	Primary
chr8	51408154	A	C	0.327	436	PASS	PXDNL	missense_variant	Primary
chr8	124998624	A	G	0.582	401	PASS	SQLE	5_prime_utr_variant	Primary
chr8	131946634	C	G	0.393	162	PASS	EFR3A	splice_donor_variant	Primary
chr8	143606582	G	C	0.619	264	PASS	PYCR3	missense_variant	Primary
chr8	143858206	A	G	0.335	735	PASS	EPPK1	synonymous_variant	Primary
chr8	143865286	T	C	0.333	73	PASS	EPPK1	synonymous_variant	Primary
chr8	143865354	G	GGGC	0.981	73	PASS	EPPK1	inframe_deletion	Primary
chr8	144264608	A	G	0.575	893	PASS	BOP1	synonymous_variant	Primary
chr8	144318024	T	TG	0.014	931	PASS	DGAT1	splice_region_variant&splice_polypyrimidine_tract_varia	Primary
chr8	144931491	A	G	0.609	726	PASS	ZNF16	synonymous_variant	Primary
chr9	12700	A	G	0.199	99	PASS	DDX11L5	intron_variant&non_coding_transcript_variant	Primary
chr9	7011765	T	C	0.445	563	PASS	KDM4C	synonymous_variant	Primary
chr9	28149027	A	G	0.025	383	PASS	LINGO2	intron_variant	Primary
chr9	29999644	T	C	0.079	59	PASS	.	intergenic_variant	Primary
chr9	32408621	G	A	0.038	196	PASS	ACO1	missense_variant	Primary
chr9	35547564	A	G	0.05	870	PASS	RUSC2	missense_variant	Primary
chr9	37436646	T	C	0.028	305	PASS	GRHPR	splice_polypyrimidine_tract_variant&intron_variant	Primary
chr9	41926666	A	G	0.267	281	PASS	CNTNAP3B	intron_variant	Primary
chr9	78031131	A	G	0.021	863	PASS	GNAQ	synonymous_variant	Primary
chr9	78317564	A	C	0.552	122	PASS	PSAT1	intron_variant	Primary
chr9	78317567	T	A	0.541	126	PASS	PSAT1	intron_variant	Primary
chr9	81992641	C	T	0.519	660	PASS	SPATA31D1	missense_variant	Primary
chr9	82058619	CA	GC	0.19	978	PASS	.	intron_variant&non_coding_transcript_variant	Primary
chr9	104617172	G	A	0.253	201	PASS	OR13C9	downstream_gene_variant	Primary
chr9	109167140	GC	CG	0.98	60	PASS	FRRS1L	5_prime_utr_variant	Primary
chr9	130319410	G	A	0.992	173	PASS	HMCN2	intron_variant	Primary
chr9	130319644	A	G	0.503	334	PASS	HMCN2	missense_variant	Primary
chr9	130321760	G	A	0.494	218	PASS	HMCN2	intron_variant	Primary
chr9	130327557	C	T	0.489	226	PASS	HMCN2	intron_variant	Primary
chr9	130338375	G	A	0.473	325	PASS	HMCN2	intron_variant	Primary
chr9	130338512	G	A	0.432	228	PASS	HMCN2	intron_variant	Primary
chr9	136290477	G	A	0.991	148	PASS	CCDC187	intron_variant	Primary
chr9	136291438	T	C	0.405	362	PASS	CCDC187	missense_variant	Primary
chr9	136459582	T	C	0.592	203	PASS	SEC16A	intron_variant	Primary

chr9	136463802	A	G	0.034	236	PASS	SEC16A	intron_variant	Primary
chr9	136740862	C	T	0.483	478	PASS	LCN10	missense_variant	Primary
chr9	137064633	T	C	0.411	438	PASS	SAPCD2	3_prime_utr_variant	Primary
chr9	137213879	A	G	0.459	539	PASS	NDOR1	splice_donor_variant	Primary
chr9	137877982	A	G	0.319	197	PASS	CACNA1B	missense_variant	Primary
chr9	137877990	A	G	0.313	222	PASS	CACNA1B	synonymous_variant	Primary
chr10	8009327	T	C	0.167	350	PASS	TAF3	synonymous_variant	Primary
chr10	12117710	A	C	0.44	255	PASS	DHTKD1	missense_variant	Primary
chr10	19327707	A	T	0.431	68	PASS	MALRD1	intron_variant	Primary
chr10	25598926	G	A	0.232	617	PASS	GPR158	synonymous_variant	Primary
chr10	27983919	A	C	0.03	354	PASS	ODAD2	missense_variant	Primary
chr10	31908696	G	T	0.478	827	PASS	ARHGAP12	missense_variant	Primary
chr10	43077384	A	G	0.307	598	PASS	RET	intron_variant	Primary
chr10	47367976	T	C	0.625	776	PASS	ZNF488	missense_variant	Primary
chr10	47762988	A	G	0.395	66	PASS	SYT15B	5_prime_utr_variant	Primary
chr10	49155796	T	C	0.292	201	PASS	TMEM273	3_prime_utr_variant	Primary
chr10	52768241	C	T	0.344	921	PASS	MBL2	missense_variant	Primary
chr10	89402218	G	A	0.323	130	PASS	LIPA	intron_variant	Primary
chr10	93325895	G	A	0.619	673	PASS	MYOF	synonymous_variant	Primary
chr10	102644855	A	G	0.305	674	PASS	TRIM8	missense_variant	Primary
chr10	110498227	A	G	0.448	374	PASS	DUSP5	missense_variant	Primary
chr10	125827540	T	A	0.248	215	PASS	BCCIP	intron_variant	Primary
chr10	125852632	A	G	0.489	491	PASS	DHX32	missense_variant	Primary
chr10	133626722	C	G	0.336	543	PASS	FRG2B	missense_variant	Primary
chr11	618647	G	GCTCTG	0.025	717	PASS	CDHR5	inframe_deletion	Primary
chr11	788236	T	C	0.024	356	PASS	CEND1	missense_variant	Primary
chr11	1018116	G	T	0.062	546	PASS	MUC6	missense_variant	Primary
chr11	1181260	T	C	0.996	349	PASS	MUC5AC	splice_polypyrimidine_tract_variant&intron_variant	Primary
chr11	1182098	CG	C	0.992	145	PASS	MUC5AC	intron_variant	Primary
chr11	17736452	C	G	0.017	515	PASS	KCNC1	missense_variant	Primary
chr11	18709691	G	A	0.69	231	PASS	IGSF22	splice_region_variant&splice_polypyrimidine_tract_vari	Primary
chr11	22624986	T	C	0.482	1472	PASS	FANCF	synonymous_variant	Primary
chr11	46375034	G	A	0.469	612	PASS	DGKZ	missense_variant	Primary
chr11	46672223	G	A	0.464	290	PASS	ATG13	intron_variant	Primary
chr11	47242986	T	C	0.513	909	PASS	ACP2	intron_variant	Primary
chr11	47441634	T	C	0.495	521	PASS	RAPSN	missense_variant	Primary
chr11	55368686	A	G	0.021	403	PASS	OR4A15	missense_variant	Primary
chr11	60040402	A	G	0.033	287	PASS	OOSP2	upstream_gene_variant	Primary
chr11	61102576	T	C	0.495	172	PASS	CD5	synonymous_variant	Primary
chr11	62528827	A	G	0.014	2092	PASS	AHNAK	synonymous_variant	Primary
chr11	64287893	T	C	0.552	385	PASS	GPR137	missense_variant	Primary
chr11	67452275	A	C	0.477	294	PASS	GPR152	missense_variant	Primary
chr11	75267758	A	G	0.051	56	PASS	ARRB1	intron_variant	Primary
chr11	89870756	C	T	0.233	647	PASS	TRIM64B	synonymous_variant	Primary
chr11	94512682	G	A	0.404	115	PASS	C11orf97	intron_variant	Primary
chr11	105043516	T	G	0.165	234	PASS	CARD16	3_prime_utr_variant	Primary
chr11	113931878	TG	CA	0.654	205	PASS	HTR3B	intron_variant	Primary
chr11	120905676	AGATGA	A	0.065	96	PASS	GRIK4	intron_variant	Primary
chr12	1593898	T	C	0.013	518	PASS	FBXL14	missense_variant	Primary
chr12	5969435	C	G	0.241	176	PASS	VWF	intron_variant	Primary
chr12	10882675	T	G	0.274	394	PASS	PRH1	missense_variant	Primary
chr12	10930916	T	C	0.2	513	PASS	PRH2	missense_variant	Primary
chr12	27387171	T	C	0.056	94	PASS	BMAL2	intron_variant	Primary
chr12	40515401	AA	GG	0.437	311	PASS	MUC19	intron_variant	Primary
chr12	40560981	A	C	0.157	78	PASS	MUC19	intron_variant	Primary
chr12	49003516	G	C	0.814	57	PASS	PRKAG1	intron_variant	Primary
chr12	49051625	A	AGCCTC	0.011	3115	PASS	KMT2D	inframe_deletion	Primary
chr12	52519021	T	C	0.018	1050	PASS	KRT5	missense_variant	Primary
chr12	52950384	C	T	0.03	364	PASS	KRT18	synonymous_variant	Primary
chr12	52950399	T	C	0.029	373	PASS	KRT18	synonymous_variant	Primary
chr12	54182416	G	A	0.204	563	PASS	SMUG1	missense_variant	Primary
chr12	54508543	T	C	0.51	248	PASS	NCKAP1L	intron_variant	Primary
chr12	56718542	G	GGGGGA	0.031	445	PASS	NACA	inframe_deletion	Primary
chr12	62104733	AA	CC	0.14	452	PASS	TAFA2	intron_variant	Primary
chr12	64222275	T	C	0.503	365	PASS	KICS2	5_prime_utr_variant	Primary
chr12	68295209	G	A	0.043	64	PASS	MDM1	3_prime_utr_variant	Primary
chr12	72272838	C	A	0.658	634	PASS	TRHDE	synonymous_variant	Primary
chr12	101338126	G	C	0.471	283	PASS	UTP20	synonymous_variant	Primary
chr12	101651384	AGAAGG	A	0.233	199	PASS	MYBPC1	stop_gained&frameshift_variant	Primary
chr12	101717207	G	T	0.074	128	PASS	CHPT1	intron_variant	Primary
chr12	103984764	T	C	0.014	413	PASS	TDG	missense_variant	Primary
chr12	108940930	C	T	0.27	227	PASS	SVOP	intron_variant	Primary
chr12	111520955	G	T	0.204	430	PASS	ATXN2	missense_variant	Primary
chr12	122702193	T	TAAGGA	0.039	946	PASS	HCAR2	frameshift_variant&stop_lost	Primary
chr12	124314124	C	T	0.237	232	PASS	RFLNA	intron_variant	Primary
chr12	124912584	T	C	0.023	1902	PASS	UBC	synonymous_variant	Primary
chr12	124912812	C	T	0.019	2172	PASS	UBC	synonymous_variant	Primary
chr12	124913538	C	T	0.011	2116	PASS	UBC	synonymous_variant	Primary
chr12	124913577	A	G	0.028	2099	PASS	UBC	synonymous_variant	Primary
chr12	131982290	AT	CC	0.44	456	PASS	EP400	missense_variant	Primary
chr12	133010734	A	G	0.445	406	PASS	ZNF26	synonymous_variant	Primary
chr12	133100956	C	T	0.58	215	PASS	ZNF140	intron_variant	Primary
chr13	23869336	A	G	0.031	194	PASS	MIPEP	missense_variant	Primary
chr13	23891902	C	A	0.125	1347	PASS	C1QTNF9B	missense_variant	Primary
chr13	23896721	G	T	0.047	417	PASS	PCOTH	intron_variant&non_coding_transcript_variant	Primary
chr13	111265556	C	G	0.547	449	PASS	ARHGEF7	intron_variant	Primary

chr13	114323914	C	A	0.342	416	PASS	CHAMP1	synonymous_variant	Primary
chr14	19097884	T	G	0.158	169	PASS	DUXAP9	intron_variant&non_coding_transcript_variant	Primary
chr14	19097966	A	C	0.123	73	PASS	DUXAP9	intron_variant&non_coding_transcript_variant	Primary
chr14	23417278	T	G	0.478	572	PASS	MYH7	stop_gained	Primary
chr14	23424064	G	A	0.509	779	PASS	MYH7	missense_variant	Primary
chr14	23967451	C	A	0.058	497	PASS	DHRS4	intron_variant	Primary
chr14	32546179	A	G	0.227	401	PASS	AKAP6	missense_variant	Primary
chr14	45032186	C	G	0.49	161	PASS	TOGARAM1	intron_variant	Primary
chr14	45137184	G	A	0.484	1912	PASS	FANCM	missense_variant	Primary
chr14	45164495	A	G	0.493	2541	PASS	FANCM	missense_variant	Primary
chr14	47061434	T	C	0.02	292	PASS	MDGA2	missense_variant	Primary
chr14	50613419	T	G	0.53	91	PASS	ATL1	intron_variant	Primary
chr14	77827834	CT	C	0.846	158	PASS	ADCK1	intron_variant	Primary
chr14	90304150	A	G	0.412	748	PASS	NRDE2	missense_variant	Primary
chr14	91729459	T	A	0.243	56	PASS	CATSPERB	stop_gained	Primary
chr14	100567885	T	C	0.579	236	PASS	BEGAIN	intron_variant	Primary
chr14	104741279	T	C	0.392	256	PASS	ADSS1	intron_variant	Primary
chr14	104932945	C	A	0.394	463	PASS	PLD4	missense_variant	Primary
chr14	105241244	C	G	0.457	95	PASS	BRF1	intron_variant	Primary
chr14	105622991	GGGGGA	G	0.097	175	PASS	IGHG4	intron_variant	Primary
chr14	105625935	C	G	0.234	714	PASS	IGHG4	synonymous_variant	Primary
chr14	105707471	A	G	0.339	1593	PASS	IGHA1	synonymous_variant	Primary
chr14	105767772	G	A	0.093	217	PASS	IGHG3	intron_variant	Primary
chr14	105863968	G	A	0.593	161	PASS	IGHJ2	downstream_gene_variant	Primary
chr14	106012330	C	G	0.497	961	PASS	IGHV4-4	synonymous_variant	Primary
chr14	106062072	G	T	0.215	1041	PASS	IGHV3-7	downstream_gene_variant	Primary
chr14	106062074	G	T	0.215	1041	PASS	IGHV3-7	downstream_gene_variant	Primary
chr14	106062169	A	G	0.504	2167	PASS	IGHV3-7	synonymous_variant	Primary
chr14	106211435	C	G	0.011	889	PASS	IGHV3-20	5_prime_utr_variant	Primary
chr14	106335083	C	T	0.325	6301	PASS	IGHV3-30	missense_variant	Primary
chr14	106349771	A	T	0.537	2157	PASS	IGHV4-31	5_prime_utr_variant	Primary
chr14	106675148	C	A	0.145	1691	PASS	IGHV3-66	missense_variant	Primary
chr14	106750387	C	T	0.986	71	PASS	.	non_coding_transcript_exon_variant	Primary
chr15	21609551	T	G	0.417	102	PASS	OR1Q1BP	non_coding_transcript_exon_variant	Primary
chr15	21652711	G	A	0.986	71	PASS	OR4N4C	missense_variant	Primary
chr15	22431965	T	G	0.173	339	PASS	.	non_coding_transcript_exon_variant	Primary
chr15	28385304	C	G	0.093	81	PASS	GOLGA8F	missense_variant	Primary
chr15	28705459	G	A	0.038	114	PASS	GOLGA8M	intron_variant	Primary
chr15	28705483	T	C	0.037	168	PASS	GOLGA8M	splice_region_variant&synonymous_variant	Primary
chr15	30614305	T	A	0.128	114	PASS	GOLGA8H	splice_donor_region_variant&intron_variant	Primary
chr15	30800018	T	G	0.044	271	PASS	GOLGA8UP	splice_polypyrimidine_tract_variant&intron_variant&no	Primary
chr15	30800585	A	G	0.053	326	PASS	GOLGA8UP	non_coding_transcript_exon_variant	Primary
chr15	31483284	T	G	0.057	67	PASS	OTUD7A	3_prime_utr_variant	Primary
chr15	34154595	T	C	0.619	187	PASS	KATNBL1	intron_variant	Primary
chr15	34237538	ATCTTCT	A	0.027	265	PASS	SLC12A6	stop_gained&inframe_insertion	Primary
chr15	34254487	T	C	0.322	342	PASS	SLC12A6	missense_variant	Primary
chr15	35542455	C	G	0.038	192	PASS	DPH6	missense_variant	Primary
chr15	44828439	G	A	0.184	1691	PASS	SORD2P	intron_variant&non_coding_transcript_variant	Primary
chr15	44828663	C	T	0.198	799	PASS	SORD2P	intron_variant&non_coding_transcript_variant	Primary
chr15	50107012	T	A	0.057	56	PASS	ATP8B4	splice_region_variant&splice_polypyrimidine_tract_vari	Primary
chr15	54013596	A	T	0.614	413	PASS	UNC13C	missense_variant	Primary
chr15	61884130	T	C	0.379	100	PASS	VPS13C	missense_variant&splice_region_variant	Primary
chr15	64957167	C	T	0.987	84	PASS	ANKDD1A	synonymous_variant	Primary
chr15	67255028	C	G	0.517	318	PASS	IQCH	intron_variant	Primary
chr15	69014683	CC	AG	0.52	357	PASS	NOX5	upstream_gene_variant	Primary
chr15	74619971	T	G	0.187	794	PASS	CLK3	intron_variant	Primary
chr15	79025348	A	G	0.205	408	PASS	RASGRF1	missense_variant	Primary
chr15	80989762	A	G	0.197	392	PASS	MESD	synonymous_variant	Primary
chr15	81374132	T	C	0.017	374	PASS	TMC3	5_prime_utr_variant	Primary
chr15	82343155	A	C	0.772	308	PASS	GOLGA6L10	missense_variant	Primary
chr15	82345216	C	A	0.655	1372	PASS	GOLGA6L10	missense_variant	Primary
chr15	82345980	GT	CC	0.226	318	PASS	GOLGA6L10	intron_variant	Primary
chr15	82430096	A	G	0.978	50	PASS	GOLGA6L9	missense_variant	Primary
chr15	82431812	C	T	0.986	172	PASS	GOLGA6L9	intron_variant	Primary
chr15	82434266	AC	GT	0.206	906	PASS	GOLGA6L9	missense_variant	Primary
chr15	82434465	T	C	0.049	112	PASS	GOLGA6L9	stop_gained	Primary
chr15	82524069	C	CGTGAA	0.11	158	PASS	GOLGA6L17P	non_coding_transcript_exon_variant	Primary
chr15	82524937	A	G	0.214	289	PASS	GOLGA6L17P	intron_variant&non_coding_transcript_variant	Primary
chr15	90257134	T	C	0.162	439	PASS	TTLL13	splice_region_variant&splice_polypyrimidine_tract_vari	Primary
chr15	90918293	T	C	0.034	179	PASS	MAN2A2	stop_gained	Primary
chr15	101922112	C	T	0.277	196	PASS	OR4F4	3_prime_utr_variant	Primary
chr15	101966111	G	C	0.113	198	PASS	.	non_coding_transcript_exon_variant	Primary
chr16	281826	C	G	0.438	234	PASS	ARHGDIG	missense_variant	Primary
chr16	2114921	T	G	0.07	65	PASS	PKD1	missense_variant	Primary
chr16	2324392	T	C	0.55	165	PASS	ABCA3	intron_variant	Primary
chr16	3089669	A	G	0.075	539	PASS	ZSCAN10	missense_variant	Primary
chr16	5090241	T	C	0.031	543	PASS	EEF2KMT	synonymous_variant	Primary
chr16	5090243	TG	CA	0.03	546	PASS	EEF2KMT	missense_variant	Primary
chr16	8645986	CGCCTG	C	0.996	449	PASS	METTL22	intron_variant	Primary
chr16	9764661	A	G	0.5	5386	PASS	GRIN2A	synonymous_variant	Primary
chr16	11398466	A	G	0.2	550	PASS	.	missense_variant	Primary
chr16	11999364	T	C	0.446	873	PASS	SNX29	splice_donor_region_variant&intron_variant	Primary
chr16	14918846	C	T	0.139	85	PASS	PKD1P3	non_coding_transcript_exon_variant	Primary
chr16	14951909	T	C	0.271	1454	PASS	NPIPA1	missense_variant	Primary
chr16	14975345	C	T	0.071	1059	PASS	PDXDC1	intron_variant	Primary
chr16	15129957	T	C	0.28	275	PASS	PDXDC1	intron_variant	Primary

chr16	16208729	C	T	0.344	627	PASS	ABCC6	missense_variant&splice_region_variant	Primary
chr16	18347905	G	A	0.14	255	PASS	PKD1P4	non_coding_transcript_exon_variant	Primary
chr16	28757992	C	CA	0.079	71	PASS	NPIPB9	intron_variant	Primary
chr16	28757997	T	G	0.079	71	PASS	NPIPB9	intron_variant	Primary
chr16	29086307	T	A	0.086	65	PASS	.	intron_variant&non_coding_transcript_variant	Primary
chr16	29086308	ATGTGTC	A	0.086	64	PASS	PLA2G10CP	intron_variant&non_coding_transcript_variant	Primary
chr16	29383175	C	T	0.029	562	PASS	NPIPB11	missense_variant	Primary
chr16	31793797	T	A	0.212	220	PASS	KRBOX5	non_coding_transcript_exon_variant	Primary
chr16	50668036	T	C	0.351	321	PASS	.	non_coding_transcript_exon_variant	Primary
chr16	51142131	A	C	0.202	278	PASS	SALL1	missense_variant	Primary
chr16	55812849	T	C	0.202	679	PASS	CES1	intron_variant	Primary
chr16	58284658	C	G	0.498	473	PASS	PRSS54	missense_variant	Primary
chr16	74389175	G	C	0.141	232	PASS	NPIPB15	intron_variant	Primary
chr17	138824	A	C	0.99	311	PASS	SCGB1C2	intron_variant	Primary
chr17	2998283	A	G	0.2	550	PASS	RAP1GAP2	synonymous_variant	Primary
chr17	6758681	A	G	0.023	1002	PASS	XAF1	intron_variant	Primary
chr17	7675085	T	C	0.419	2250	PASS	TP53	missense_variant	Primary
chr17	8509911	T	C	0.025	405	PASS	MYH10	synonymous_variant	Primary
chr17	10728978	T	C	0.022	323	PASS	TMEM220	intron_variant	Primary
chr17	16382069	C	T	0.02	567	PASS	UBB	synonymous_variant	Primary
chr17	18091852	TG	CA	0.989	129	PASS	DRG2	intron_variant	Primary
chr17	21043507	G	A	0.308	520	PASS	USP22	upstream_gene_variant	Primary
chr17	22204310	T	G	0.039	317	PASS	UBBP4	non_coding_transcript_exon_variant	Primary
chr17	28048003	A	G	0.478	88	PASS	NLK	intron_variant	Primary
chr17	28369358	G	C	0.502	645	PASS	VTN	missense_variant	Primary
chr17	29632116	T	TTG	0.233	865	PASS	SSH2	frameshift_variant	Primary
chr17	31295017	A	C	0.023	150	PASS	OMG	missense_variant	Primary
chr17	36171845	A	G	0.387	91	PASS	.	intron_variant&non_coding_transcript_variant	Primary
chr17	36195196	A	G	0.708	344	PASS	CCL3L3	3_prime_utr_variant	Primary
chr17	36195296	G	A	0.302	957	PASS	CCL3L3	missense_variant	Primary
chr17	36195375	G	A	0.294	1165	PASS	CCL3L3	missense_variant&splice_region_variant	Primary
chr17	36195866	C	T	0.297	1257	PASS	CCL3L3	synonymous_variant	Primary
chr17	36196527	G	A	0.997	379	PASS	CCL3L3	intron_variant	Primary
chr17	36211278	T	G	0.998	728	PASS	CCL4L2	intron_variant	Primary
chr17	36211979	C	T	0.996	350	PASS	CCL4L2	intron_variant	Primary
chr17	36212342	GA	CG	0.998	610	PASS	CCL4L2	missense_variant	Primary
chr17	36254409	A	G	0.268	54	PASS	TBC1D3I	missense_variant	Primary
chr17	36307282	A	T	0.16	78	PASS	.	splice_polypyrimidine_tract_variant&intron_variant&non	Primary
chr17	36332312	A	G	0.394	64	PASS	TBC1D3G	missense_variant	Primary
chr17	36452733	A	G	0.39	240	PASS	TBC1D3JP	non_coding_transcript_exon_variant	Primary
chr17	37635608	T	A	0.374	381	PASS	DDX52	intron_variant	Primary
chr17	39745244	G	A	0.036	188	PASS	GRB7	missense_variant&splice_region_variant	Primary
chr17	40818851	AGCTGCC	A	0.514	304	PASS	KRT10	inframe_insertion	Primary
chr17	41034702	GCAGCAC	G	0.345	935	PASS	KRTAP1-3	inframe_insertion	Primary
chr17	41117835	C	G	0.056	626	PASS	KRTAP4-11	missense_variant	Primary
chr17	41140002	T	C	0.301	753	PASS	KRTAP4-6	synonymous_variant	Primary
chr17	41140077	T	TGGCTGC	0.014	768	PASS	KRTAP4-6	inframe_deletion	Primary
chr17	41275814	T	G	0.02	1009	PASS	KRTAP9-7	synonymous_variant	Primary
chr17	41422764	GC	AG	0.164	103	PASS	KRT37	intron_variant	Primary
chr17	41503412	T	C	0.046	459	PASS	KRT13	missense_variant	Primary
chr17	41567235	A	T	0.182	187	PASS	KRT9	splice_region_variant&3_prime_utr_variant	Primary
chr17	46171133	C	G	0.309	1786	PASS	KANSL1	synonymous_variant	Primary
chr17	46171730	C	A	0.366	1128	PASS	KANSL1	synonymous_variant	Primary
chr17	46171833	G	T	0.34	1005	PASS	KANSL1	missense_variant	Primary
chr17	46243907	C	A	0.896	55	PASS	MAPK8IP1P1	non_coding_transcript_exon_variant	Primary
chr17	46259756	T	C	0.244	934	PASS	.	non_coding_transcript_exon_variant	Primary
chr17	46259843	T	C	0.155	1442	PASS	.	non_coding_transcript_exon_variant	Primary
chr17	46528798	A	G	0.074	202	PASS	LRRC37A2	intron_variant	Primary
chr17	46548670	A	G	0.198	770	PASS	LRRC37A2	synonymous_variant	Primary
chr17	46548981	C	T	0.39	1683	PASS	LRRC37A2	missense_variant	Primary
chr17	47033320	C	G	0.147	167	PASS	.	splice_region_variant&non_coding_transcript_exon_vari	Primary
chr17	47923156	T	C	0.185	605	PASS	SP2	synonymous_variant	Primary
chr17	58617559	C	G	0.491	226	PASS	TEX14	missense_variant	Primary
chr17	64822018	C	G	0.115	694	PASS	.	non_coding_transcript_exon_variant	Primary
chr17	67138031	G	A	0.474	428	PASS	HELZ	synonymous_variant	Primary
chr17	69304746	T	C	0.54	69	PASS	ABCA5	missense_variant	Primary
chr17	72736813	G	A	0.032	150	PASS	SLC39A11	intron_variant	Primary
chr17	75937609	G	C	0.49	465	PASS	FBF1	splice_polypyrimidine_tract_variant&intron_variant	Primary
chr17	78974783	A	G	0.023	475	PASS	LGALS3BP	missense_variant	Primary
chr17	79784185	C	G	0.506	587	PASS	CBX2	missense_variant	Primary
chr17	81379949	A	G	0.507	66	PASS	LINC03048	intron_variant&non_coding_transcript_variant	Primary
chr17	81810972	G	A	0.33	388	PASS	GCGR	intron_variant	Primary
chr17	82374399	A	T	0.253	208	PASS	UTS2R	synonymous_variant	Primary
chr18	7013998	A	G	0.492	1009	PASS	LAMA1	synonymous_variant	Primary
chr18	7926563	T	TGTAGCC	0.012	651	PASS	PTPRM	splice_acceptor_variant&coding_sequence_variant&intro	Primary
chr18	10759688	A	G	0.011	860	PASS	PIEZO2	intron_variant	Primary
chr18	23813122	C	G	0.204	224	PASS	LAMA3	intron_variant	Primary
chr18	34882022	A	T	0.754	179	PASS	DTNA	intron_variant	Primary
chr18	46098362	AAC	A	0.183	59	PASS	ATP5F1A	upstream_gene_variant	Primary
chr18	46509716	T	C	0.259	448	PASS	LOXHD1	synonymous_variant	Primary
chr18	47035248	C	G	0.268	378	PASS	ELOA2	missense_variant	Primary
chr18	47035259	T	C	0.261	367	PASS	ELOA2	synonymous_variant	Primary
chr18	58351055	T	C	0.032	186	PASS	NEDD4L	intron_variant	Primary
chr18	63331755	A	AC	0.279	1202	PASS	KDSR	3_prime_utr_variant	Primary
chr18	63331757	A	AG	0.279	1202	PASS	KDSR	3_prime_utr_variant	Primary
chr19	425073	A	G	0.022	351	PASS	SHC2	intron_variant	Primary

chr19	1622424	A	G	0.722	241	PASS	TCF3	splice_polypyrimidine_tract_variant&intron_variant	Primary
chr19	7505306	G	A	0.026	228	PASS	TEX45	intron_variant	Primary
chr19	8891843	C	G	0.274	1074	PASS	MUC16	missense_variant	Primary
chr19	8895694	G	A	0.112	284	PASS	MUC16	synonymous_variant	Primary
chr19	8916981	A	C	0.021	407	PASS	MUC16	intron_variant	Primary
chr19	8949340	A	C	0.186	834	PASS	MUC16	missense_variant	Primary
chr19	14160744	A	AGAG	0.022	175	PASS	ADGRL1	intron_variant	Primary
chr19	17487090	T	C	0.015	406	PASS	SLC27A1	intron_variant	Primary
chr19	18931976	A	C	0.074	54	PASS	HOMER3	splice_region_variant&synonymous_variant	Primary
chr19	18938522	A	G	0.505	702	PASS	HOMER3	intron_variant	Primary
chr19	19505356	G	C	0.43	313	PASS	GATAD2A	missense_variant	Primary
chr19	38950233	T	C	0.475	580	PASS	FBXO17	synonymous_variant	Primary
chr19	39891772	T	C	0.033	1174	PASS	FCGBP	missense_variant	Primary
chr19	40048850	T	C	0.108	343	PASS	ZNF780B	intron_variant	Primary
chr19	40370082	A	C	0.53	770	PASS	PLD3	intron_variant	Primary
chr19	40442025	A	C	0.073	149	PASS	SERTAD3	missense_variant	Primary
chr19	40778492	CA	TG	0.49	279	PASS	RAB4B	intron_variant	Primary
chr19	41016802	G	A	0.02	411	PASS	CYP2B6	missense_variant	Primary
chr19	41579543	T	G	0.229	603	PASS	CEACAM21	missense_variant	Primary
chr19	44496520	C	A	0.522	98	PASS	ZNF180	intron_variant	Primary
chr19	45785972	T	C	0.042	460	PASS	DMWD	synonymous_variant	Primary
chr19	47680164	A	G	0.206	268	PASS	BICRA	missense_variant	Primary
chr19	48510162	A	G	0.471	424	PASS	LMTK3	synonymous_variant	Primary
chr19	49101346	T	C	0.193	423	PASS	SNRNP70	intron_variant	Primary
chr19	49696520	G	T	0.047	55	PASS	CPT1C	intron_variant	Primary
chr19	52799895	A	G	0.05	243	PASS	ZNF28	synonymous_variant	Primary
chr19	53140881	T	A	0.022	494	PASS	ZNF347	synonymous_variant	Primary
chr19	53183041	A	C	0.193	230	PASS	ZNF665	intron_variant	Primary
chr19	54220739	A	C	0.495	867	PASS	LILRB3	synonymous_variant	Primary
chr19	54222756	C	T	0.666	1361	PASS	LILRB3	missense_variant	Primary
chr19	54222987	T	C	0.313	1132	PASS	LILRB3	5_prime_utr_variant	Primary
chr19	54242117	C	T	0.448	1207	PASS	LILRA6	missense_variant	Primary
chr19	54336935	C	T	0.114	1836	PASS	LILRA4	synonymous_variant	Primary
chr19	54463591	A	T	0.988	121	PASS	LENG9	5_prime_utr_variant	Primary
chr19	54595586	T	A	0.065	369	PASS	LILRA1	intron_variant	Primary
chr19	54667677	G	A	0.095	481	PASS	LILRB4	missense_variant	Primary
chr19	54727771	T	C	0.421	941	PASS	KIR3DL3	synonymous_variant	Primary
chr19	54755120	T	G	0.266	894	PASS	KIR2DP1	intron_variant&non_coding_transcript_variant	Primary
chr19	54773534	A	C	0.3	2756	PASS	KIR2DL1	missense_variant	Primary
chr19	54773593	T	G	0.275	2693	PASS	KIR2DL1	missense_variant	Primary
chr19	54773692	C	A	0.209	1264	PASS	KIR2DL1	intron_variant	Primary
chr19	54773699	G	A	0.205	1192	PASS	KIR2DL1	intron_variant	Primary
chr19	54837942	G	A	0.198	628	PASS	KIR2DS4	intron_variant	Primary
chr19	54855560	G	A	0.066	663	PASS	KIR3DL2	intron_variant	Primary
chr19	54855661	A	C	0.017	1346	PASS	KIR3DL2	missense_variant	Primary
chr19	54865908	A	G	0.025	509	PASS	KIR3DL2	splice_region_variant&synonymous_variant	Primary
chr19	57491772	TG	CA	0.076	185	PASS	ZNF419	intron_variant	Primary
chr20	2483536	G	A	0.013	1086	PASS	ZNF343	synonymous_variant	Primary
chr20	8018995	A	G	0.312	653	PASS	TMX4	intron_variant	Primary
chr20	10641597	T	C	0.015	1085	PASS	JAG1	missense_variant	Primary
chr20	24665463	A	G	0.567	595	PASS	SYNDIG1	missense_variant	Primary
chr20	41204565	T	C	0.011	1090	PASS	ZHX3	missense_variant	Primary
chr20	42677938	T	G	0.012	1895	PASS	PTPRT	missense_variant	Primary
chr20	46022943	GGGA	G	0.961	232	PASS	SLC12A5	intron_variant	Primary
chr20	47633652	A	G	0.364	1415	PASS	NCOA3	intron_variant	Primary
chr20	54476047	A	G	0.16	678	PASS	DOK5	intron_variant	Primary
chr20	56637826	A	G	0.42	1000	PASS	TFAP2C	missense_variant	Primary
chr21	5033666	A	G	0.608	248	PASS	.	intergenic_variant	Primary
chr21	28931146	C	T	0.511	118	PASS	LTN1	intron_variant	Primary
chr21	45939993	A	G	0.231	138	PASS	PCBP3	intron_variant	Primary
chr22	15820147	A	G	0.283	172	PASS	DUXAP8	intron_variant&non_coding_transcript_variant	Primary
chr22	17369970	A	G	0.526	132	PASS	CECR2	intron_variant	Primary
chr22	17549225	T	C	0.198	864	PASS	CECR2	missense_variant	Primary
chr22	18787762	A	G	0.166	483	PASS	.	intron_variant&non_coding_transcript_variant	Primary
chr22	20703127	C	T	0.053	427	PASS	TMEM191A	splice_donor_5th_base_variant&intron_variant&non_co	Primary
chr22	20996080	T	C	0.477	1595	PASS	LZTR1	synonymous_variant	Primary
chr22	22376327	T	C	0.185	895	PASS	IGLV5-45	missense_variant	Primary
chr22	22376333	G	A	0.187	893	PASS	IGLV5-45	missense_variant	Primary
chr22	26622751	A	C	0.492	149	PASS	CRYBA4	intron_variant	Primary
chr22	29489222	G	GCTGAG	0.015	404	PASS	NEFH	inframe_deletion	Primary
chr22	31095586	A	G	0.457	526	PASS	SMTN	missense_variant	Primary
chr22	32360836	CT	GC	0.023	470	PASS	RFPL3S	splice_region_variant&non_coding_transcript_exon_vari	Primary
chr22	36935279	T	C	0.492	237	PASS	CSF2RB	intron_variant	Primary
chr22	41987337	C	T	0.492	396	PASS	SEPTIN3	intron_variant	Primary
chr22	42126611	G	C	0.465	978	PASS	CYP2D6	missense_variant	Primary
chr22	42127803	T	C	0.505	1140	PASS	NDUFA6-DT	intron_variant&non_coding_transcript_variant	Primary
chr22	42129130	G	C	0.448	1102	PASS	CYP2D6	synonymous_variant	Primary
chr22	42129950	C	A	0.474	183	PASS	NDUFA6-DT	intron_variant&non_coding_transcript_variant	Primary
chr22	42140200	G	A	0.427	486	PASS	.	downstream_gene_variant	Primary
chr22	42143622	C	A	0.441	139	PASS	CYP2D7	intron_variant	Primary
chr22	48646730	TT	GC	0.027	460	PASS	TAFA5	missense_variant	Primary
chr22	50220737	T	TGGGAG	0.011	3017	PASS	TUBGCP6	inframe_deletion	Primary
chr22	50220783	A	AGACAC	0.016	3271	PASS	TUBGCP6	inframe_deletion	Primary
chrX	315221	C	T	0.662	649	PASS	GTPBP6	splice_region_variant&intron_variant	Primary
chrX	346853	A	G	0.03	559	PASS	PPP2R3B	intron_variant	Primary
chrX	1191074	C	T	0.557	335	PASS	CRLF2	synonymous_variant	Primary

chrX	1191089	A	G	0.558	318	PASS	CRLF2	synonymous_variant	Primary
chrX	2859990	A	G	0.997	400	PASS	GYG2	synonymous_variant	Primary
chrX	19351308	A	G	0.45	241	PASS	PDHA1	missense_variant	Primary
chrX	45053912	G	GAAGC	0.02	950	PASS	KDM6A	frameshift_variant	Primary
chrX	49162895	C	G	0.993	203	PASS	MAGIX	missense_variant	Primary
chrX	53565023	A	G	0.992	151	PASS	HUWE1	intron_variant	Primary
chrX	73453605	G	T	0.436	62	PASS	CDX4	stop_gained	Primary
chrX	74591813	A	G	0.096	180	PASS	RLIM	missense_variant	Primary
chrX	74593081	A	G	0.987	88	PASS	RLIM	intron_variant	Primary
chrX	76428444	A	ACGAGCA	0.01	1118	PASS	MAGEE1	inframe_deletion	Primary
chrX	77448031	T	C	0.978	50	PASS	FGF16	intron_variant	Primary
chrX	101656080	C	T	0.995	225	PASS	ARMCX2	synonymous_variant	Primary
chrX	103500463	T	G	0.997	333	PASS	RAB40A	missense_variant	Primary
chrX	107597779	A	T	0.403	340	PASS	FRMPD3	missense_variant	Primary
chrX	115191561	A	G	0.997	433	PASS	RBMXL3	missense_variant	Primary
chrX	149598196	A	G	0.066	56	PASS	TMEM185A	stop_gained	Primary
chrX	150512284	C	T	0.994	221	PASS	MAMLD1	3_prime_utr_variant	Primary
chrX	152915171	C	CT	0.421	266	PASS	ZNF185	frameshift_variant	Primary
chrX	153421236	A	C	0.018	325	PASS	ZFP92	missense_variant	Primary
chrX	153447803	A	G	0.033	181	PASS	HAUS7	3_prime_utr_variant	Primary
chrX	153958514	A	G	0.995	253	PASS	HCFC1	intron_variant	Primary
chrX	156021706	G	C	0.146	83	PASS	WASH6P	missense_variant	Primary
chrY	8695423	A	G	0.993	177	PASS	LINC00279	intron_variant&non_coding_transcript_variant	Primary
chrY	12859387	C	G	0.057	56	PASS	USP9Y	missense_variant	Primary
chrY	13479565	C	T	0.997	504	PASS	UTY	missense_variant	Primary
chr1	17594	T	C	0.191	145	PASS	WASH7P	intron_variant&non_coding_transcript_variant	Dura AdCa
chr1	183189	C	G	0.116	59	PASS	DDX11L17	non_coding_transcript_exon_variant	Dura AdCa
chr1	183274	A	G	0.15	108	PASS	DDX11L17	intron_variant&non_coding_transcript_variant	Dura AdCa
chr1	184142	T	C	0.04	146	PASS	DDX11L17	non_coding_transcript_exon_variant	Dura AdCa
chr1	999495	T	C	0.451	154	PASS	HES4	intron_variant	Dura AdCa
chr1	1223422	G	A	0.431	157	PASS	SDF4	intron_variant	Dura AdCa
chr1	1264845	C	T	0.098	72	PASS	UBE2J2	intron_variant	Dura AdCa
chr1	1637398	C	G	0.036	156	PASS	CDK11B	intron_variant	Dura AdCa
chr1	1716403	T	C	0.023	328	PASS	CDK11A	missense_variant	Dura AdCa
chr1	6474096	A	G	0.029	254	PASS	PLEKHG5	missense_variant	Dura AdCa
chr1	6544895	T	A	0.036	250	PASS	NOL9	missense_variant	Dura AdCa
chr1	12883168	TG	CA	0.602	167	PASS	PRAMEF4	missense_variant	Dura AdCa
chr1	13224919	A	T	0.275	172	PASS	PRAMEF18	missense_variant	Dura AdCa
chr1	13225068	G	A	0.224	310	PASS	PRAMEF18	missense_variant	Dura AdCa
chr1	13225208	G	C	0.241	593	PASS	PRAMEF18	synonymous_variant	Dura AdCa
chr1	13306781	G	A	0.278	124	PASS	PRAMEF33	missense_variant	Dura AdCa
chr1	13318650	G	A	0.225	280	PASS	PRAMEF15	synonymous_variant	Dura AdCa
chr1	15034902	G	C	0.023	94	PASS	KAZN	intron_variant	Dura AdCa
chr1	16646619	A	AGCCATC	0.118	1111	PASS	CROCCP2	intron_variant&non_coding_transcript_variant	Dura AdCa
chr1	16761036	A	G	0.181	1083	PASS	CROCC	intron_variant&non_coding_transcript_variant	Dura AdCa
chr1	17422634	A	G	0.024	240	PASS	RCC2	intron_variant	Dura AdCa
chr1	21706190	T	A	0.036	115	PASS	USP48	splice_region_variant&splice_polypyrimidine_tract_varia	Dura AdCa
chr1	21706203	T	C	0.039	106	PASS	USP48	splice_polypyrimidine_tract_variant&intron_variant	Dura AdCa
chr1	23802500	A	G	0.028	226	PASS	HMGCL	missense_variant	Dura AdCa
chr1	26166079	A	G	0.527	282	PASS	C1orf232	missense_variant	Dura AdCa
chr1	27760024	A	C	0.033	173	PASS	FAM76A	intron_variant	Dura AdCa
chr1	33569487	G	A	0.399	390	PASS	CSMD2	missense_variant	Dura AdCa
chr1	35736604	A	T	0.033	140	PASS	CLSPN	splice_region_variant&synonymous_variant	Dura AdCa
chr1	36308186	A	G	0.568	325	PASS	SH3D21	missense_variant	Dura AdCa
chr1	37984313	T	A	0.032	149	PASS	SF3A3	intron_variant	Dura AdCa
chr1	40315539	T	C	0.048	77	PASS	COL9A2	intron_variant	Dura AdCa
chr1	42653855	G	C	0.043	64	PASS	CCDC30	missense_variant	Dura AdCa
chr1	44130720	G	T	0.03	194	PASS	KLF17	missense_variant	Dura AdCa
chr1	44139048	TCTGGTT	T	0.99	455	PASS	KLF18	inframe_insertion	Dura AdCa
chr1	44139505	A	G	0.012	815	PASS	KLF18	synonymous_variant	Dura AdCa
chr1	44139841	G	A	0.069	747	PASS	KLF18	synonymous_variant	Dura AdCa
chr1	44851040	T	G	0.17	53	PASS	EIF2B3	intron_variant	Dura AdCa
chr1	46309992	A	G	0.027	395	PASS	UQCRH	intron_variant	Dura AdCa
chr1	51448176	CCATTAA	C	0.038	151	PASS	EPS15	intron_variant	Dura AdCa
chr1	52446432	ACTAGGT	A	0.07	144	PASS	TUT4	stop_gained&frameshift_variant	Dura AdCa
chr1	55089733	TGAAGTC	T	0.059	98	PASS	USP24	inframe_insertion	Dura AdCa
chr1	55092049	C	A	0.438	131	PASS	USP24	synonymous_variant	Dura AdCa
chr1	62084601	T	C	0.033	279	PASS	PATJ	missense_variant	Dura AdCa
chr1	64140183	CTGAGCA	C	0.024	257	PASS	ROR1	frameshift_variant	Dura AdCa
chr1	64140185	CATAGTT	C	0.024	258	PASS	ROR1	stop_gained&frameshift_variant	Dura AdCa
chr1	65254515	T	G	0.44	119	PASS	DNAJC6	intron_variant&non_coding_transcript_variant	Dura AdCa
chr1	70365344	T	C	0.896	150	PASS	HHLA3	intron_variant&non_coding_transcript_variant	Dura AdCa
chr1	84410938	GCTTGGC	G	0.085	223	PASS	DNASE2B	stop_gained&frameshift_variant	Dura AdCa
chr1	92792250	T	A	0.064	61	PASS	EVI5	intron_variant	Dura AdCa
chr1	99712018	T	C	0.431	85	PASS	FRRS1	intron_variant	Dura AdCa
chr1	101804649	A	ACCCCAT	0.035	127	PASS	OLFM3	frameshift_variant	Dura AdCa
chr1	101846856	A	G	0.867	56	PASS	OLFM3	intron_variant	Dura AdCa
chr1	111120691	TATACTC	T	0.07	98	PASS	DRAM2	frameshift_variant&splice_region_variant	Dura AdCa
chr1	111120693	TTACTTA	T	0.07	98	PASS	DRAM2	stop_gained&inframe_insertion&splice_region_variant	Dura AdCa
chr1	111231179	C	T	0.49	131	PASS	CHI3L2	intron_variant	Dura AdCa
chr1	116067082	GGCTTTC	G	0.056	127	PASS	SLC22A15	stop_gained&frameshift_variant	Dura AdCa
chr1	120258388	C	T	0.069	115	PASS	.	intron_variant&non_coding_transcript_variant	Dura AdCa
chr1	120494371	G	GGCCATC	0.021	220	PASS	PDE4DIPP2	non_coding_transcript_exon_variant	Dura AdCa
chr1	120543014	C	T	0.469	109	PASS	.	splice_donor_region_variant&intron_variant&non_codin	Dura AdCa
chr1	120890143	G	A	0.051	312	PASS	PPIAL4A	missense_variant	Dura AdCa
chr1	143796246	C	T	0.982	65	PASS	LINC02591	non_coding_transcript_exon_variant	Dura AdCa

chr1	144905217	A	G	0.04	216	PASS	SRGAP2B	splice_region_variant&synonymous_variant	Dura AdCa
chr1	145241802	C	T	0.395	153	PASS	PPIAL4D	missense_variant	Dura AdCa
chr1	145292353	C	T	0.113	116	PASS	NBPF20	intron_variant	Dura AdCa
chr1	145574836	T	TTTTGAT	0.066	152	PASS	NBPF25P	non_coding_transcript_exon_variant	Dura AdCa
chr1	147631053	G	C	0.297	98	PASS	ACP6	stop_lost	Dura AdCa
chr1	148572622	C	A	0.056	85	PASS	NBPF14	splice_region_variant&splice_polypyrimidine_tract_varia	Dura AdCa
chr1	149176847	C	T	0.513	147	PASS	.	intron_variant&non_coding_transcript_variant	Dura AdCa
chr1	149607944	C	G	0.172	185	PASS	LINC00869	intron_variant&non_coding_transcript_variant	Dura AdCa
chr1	149607947	C	G	0.171	188	PASS	.	intron_variant&non_coding_transcript_variant	Dura AdCa
chr1	151368992	T	C	0.498	379	PASS	SELENBP1	intron_variant	Dura AdCa
chr1	152213623	T	C	0.646	250	PASS	HRNR	missense_variant	Dura AdCa
chr1	152214116	G	T	0.066	62	PASS	HRNR	missense_variant	Dura AdCa
chr1	152214176	C	T	0.022	467	PASS	HRNR	missense_variant	Dura AdCa
chr1	152214592	T	G	0.118	2687	PASS	HRNR	missense_variant	Dura AdCa
chr1	152313565	CGTGGC(	C	0.014	661	PASS	FLG	stop_gained&frameshift_variant	Dura AdCa
chr1	152351448	C	A	0.019	454	PASS	FLG2	missense_variant	Dura AdCa
chr1	152351463	T	G	0.033	460	PASS	FLG2	missense_variant	Dura AdCa
chr1	154012427	CAATT	C	0.049	118	PASS	NUP210L	intron_variant	Dura AdCa
chr1	154012428	CATTTAT	C	0.05	117	PASS	NUP210L	intron_variant	Dura AdCa
chr1	158843989	A	C	0.05	88	PASS	MNDA	missense_variant	Dura AdCa
chr1	158849178	A	C	0.071	68	PASS	MNDA	splice_polypyrimidine_tract_variant&intron_variant	Dura AdCa
chr1	159066010	C	G	0.397	227	PASS	AIM2	missense_variant	Dura AdCa
chr1	160031429	C	A	0.542	192	PASS	PIGM	missense_variant	Dura AdCa
chr1	160152218	C	T	0.043	61	PASS	ATP1A4	intron_variant	Dura AdCa
chr1	160195116	T	C	0.467	133	PASS	CASQ1	synonymous_variant	Dura AdCa
chr1	160752273	TG	CA	0.507	105	PASS	SLAMF7	intron_variant	Dura AdCa
chr1	167787824	G	C	0.047	94	PASS	MPZL1	missense_variant	Dura AdCa
chr1	174220968	A	G	0.441	111	PASS	RABGAP1L	splice_region_variant&splice_polypyrimidine_tract_varia	Dura AdCa
chr1	185734850	T	C	0.029	155	PASS	HMCN1	missense_variant	Dura AdCa
chr1	185734856	T	C	0.038	161	PASS	HMCN1	missense_variant	Dura AdCa
chr1	186178656	A	ACAGCT(	0.043	117	PASS	HMCN1	inframe_deletion	Dura AdCa
chr1	193180474	T	C	0.03	226	PASS	B3GALT2	synonymous_variant	Dura AdCa
chr1	198732592	ATCTCAT	A	0.098	267	PASS	PTPRC	intron_variant	Dura AdCa
chr1	202962649	GAGAGT(	G	0.049	321	PASS	CYB5R1	stop_gained&frameshift_variant	Dura AdCa
chr1	205984165	T	G	0.992	194	PASS	RAB7B	intron_variant	Dura AdCa
chr1	212386985	A	G	0.409	282	PASS	PACC1	synonymous_variant	Dura AdCa
chr1	212780065	C	G	0.118	70	PASS	NSL1	intron_variant	Dura AdCa
chr1	216048619	TAGATAT	T	0.111	132	PASS	USH2A	stop_gained&protein_altering_variant	Dura AdCa
chr1	219918368	CGTGAG(	C	0.033	230	PASS	SLC30A10	stop_gained&frameshift_variant	Dura AdCa
chr1	220599753	G	A	0.468	67	PASS	MARK1	intron_variant	Dura AdCa
chr1	235504390	A	G	0.365	56	PASS	B3GALNT2	5_prime_utr_variant	Dura AdCa
chr1	240092805	C	T	0.852	79	PASS	FMN2	synonymous_variant	Dura AdCa
chr1	241674645	C	T	0.184	59	PASS	WDR64	splice_region_variant&synonymous_variant	Dura AdCa
chr1	241853411	TACACA(	T	0.036	172	PASS	EXO1	stop_gained&frameshift_variant	Dura AdCa
chr1	242449317	G	A	0.135	113	PASS	PLD5	intron_variant	Dura AdCa
chr1	243165585	T	G	0.163	90	PASS	CEP170	stop_gained	Dura AdCa
chr1	244054903	T	C	0.011	686	PASS	ZBTB18	stop_gained	Dura AdCa
chr1	247100759	G	GTACATT	0.018	377	PASS	ZNF669	inframe_deletion	Dura AdCa
chr1	247605799	T	TGCTTCA	0.012	434	PASS	OR2G3	inframe_deletion	Dura AdCa
chr1	248453100	A	G	0.193	524	PASS	OR2T2	synonymous_variant	Dura AdCa
chr1	248453107	T	C	0.194	529	PASS	OR2T2	missense_variant	Dura AdCa
chr2	1666526	A	G	0.028	181	PASS	PXDN	intron_variant	Dura AdCa
chr2	14634426	A	C	0.024	216	PASS	LRATD1	synonymous_variant	Dura AdCa
chr2	17714908	T	C	0.505	138	PASS	SMC6	synonymous_variant	Dura AdCa
chr2	25130488	C	A	0.518	236	PASS	EFR3B	intron_variant	Dura AdCa
chr2	27206532	T	C	0.509	187	PASS	SLC5A6	splice_region_variant&synonymous_variant	Dura AdCa
chr2	27374205	A	AAGGGTA	0.029	137	PASS	SNX17	intron_variant	Dura AdCa
chr2	27650185	A	G	0.039	200	PASS	GPN1	synonymous_variant	Dura AdCa
chr2	27650261	C	G	0.049	121	PASS	GPN1	3_prime_utr_variant	Dura AdCa
chr2	30459712	A	AAAAGC'	0.021	240	PASS	LCLAT1	intron_variant	Dura AdCa
chr2	31178617	G	T	0.415	97	PASS	CAPN14	intron_variant	Dura AdCa
chr2	38861250	AGAGTC'	A	0.039	176	PASS	DHX57	stop_gained&frameshift_variant	Dura AdCa
chr2	38929721	G	A	0.029	103	PASS	ARHGEF33	missense_variant	Dura AdCa
chr2	38935890	TAACAT(	T	0.057	106	PASS	ARHGEF33	intron_variant	Dura AdCa
chr2	42286381	TCCTTTC	T	0.041	332	PASS	EML4	splice_region_variant&intron_variant	Dura AdCa
chr2	43801586	A	G	0.373	93	PASS	DYNC2LI1	intron_variant	Dura AdCa
chr2	58160123	G	A	0.866	207	PASS	FANCL	synonymous_variant	Dura AdCa
chr2	71073543	A	G	0.114	183	PASS	NAGK	synonymous_variant	Dura AdCa
chr2	71078302	GTGT	G	0.151	119	PASS	NAGK	splice_polypyrimidine_tract_variant&intron_variant	Dura AdCa
chr2	71644058	T	C	0.886	104	PASS	DYSF	synonymous_variant	Dura AdCa
chr2	73214257	G	C	0.117	415	PASS	SMYD5	5_prime_utr_variant	Dura AdCa
chr2	73214385	T	C	0.147	236	PASS	SMYD5	intron_variant	Dura AdCa
chr2	73448695	GCCTTTT	G	0.047	127	PASS	ALMS1	frameshift_variant	Dura AdCa
chr2	74459919	T	C	0.783	260	PASS	WBP1	synonymous_variant	Dura AdCa
chr2	75646803	G	C	0.866	217	PASS	MRPL19	5_prime_utr_variant	Dura AdCa
chr2	75687859	T	A	0.88	82	PASS	GCFC2	missense_variant	Dura AdCa
chr2	89221621	A	T	0.452	97	PASS	IGKV2-28	downstream_gene_variant	Dura AdCa
chr2	89640376	T	C	0.234	166	PASS	KMT2CP4	intron_variant&non_coding_transcript_variant	Dura AdCa
chr2	90069816	CAATAC(	C	0.089	59	PASS	IGKV6D-41	intron_variant	Dura AdCa
chr2	95176282	CCACAC/	C	0.027	283	PASS	ZNF2	intron_variant	Dura AdCa
chr2	98121421	A	G	0.506	362	PASS	VWA3B	missense_variant	Dura AdCa
chr2	99394804	GGCATG/	G	0.061	125	PASS	EIF5B	protein_altering_variant	Dura AdCa
chr2	100988173	T	C	0.49	245	PASS	NPAS2	missense_variant	Dura AdCa
chr2	101402553	A	G	0.52	340	PASS	RFX8	synonymous_variant	Dura AdCa
chr2	102225970	C	T	0.485	205	PASS	IL1RL2	missense_variant	Dura AdCa
chr2	102719187	G	A	0.48	342	PASS	MFSD9	missense_variant	Dura AdCa

chr2	108383481	G	C	0.505	166	PASS	SULT1C4	missense_variant	Dura AdCa
chr2	111123759	A	C	0.435	375	PASS	BCL2L11	missense_variant	Dura AdCa
chr2	111150001	A	G	0.557	386	PASS	BCL2L11	intron_variant	Dura AdCa
chr2	111150163	G	T	0.462	423	PASS	BCL2L11	intron_variant	Dura AdCa
chr2	113236565	A	G	0.251	383	PASS	PAX8-AS1	intron_variant&non_coding_transcript_variant	Dura AdCa
chr2	127261254	AGAATCT	A	0.06	236	PASS	ERCC3	stop_gained&frameshift_variant	Dura AdCa
chr2	134936927	G	T	0.102	163	PASS	CCNT2	synonymous_variant	Dura AdCa
chr2	135135776	G	A	0.038	75	PASS	RAB3GAP1	synonymous_variant	Dura AdCa
chr2	135135797	T	A	0.034	84	PASS	RAB3GAP1	missense_variant	Dura AdCa
chr2	141055198	T	A	0.029	173	PASS	LRP1B	stop_gained	Dura AdCa
chr2	148483570	C	T	0.939	184	PASS	MBD5	synonymous_variant	Dura AdCa
chr2	159136289	GTCTTT	G	0.05	143	PASS	TANC1	frameshift_variant	Dura AdCa
chr2	159136291	TGGAGCC	T	0.051	141	PASS	TANC1	stop_gained&frameshift_variant	Dura AdCa
chr2	159332677	TCATGCC	T	0.036	170	PASS	BAZ2B	frameshift_variant	Dura AdCa
chr2	162188328	ATGAAAA	A	0.078	156	PASS	FAP	stop_gained&frameshift_variant	Dura AdCa
chr2	163611058	T	C	0.508	340	PASS	FIGN	synonymous_variant	Dura AdCa
chr2	165154626	TTCTTGG	T	0.079	109	PASS	SCN3A	stop_gained&frameshift_variant	Dura AdCa
chr2	165595000	C	G	0.073	75	PASS	CSRNP3	intron_variant	Dura AdCa
chr2	167247053	GCCCTGT	G	0.059	146	PASS	XIRP2	stop_gained&frameshift_variant	Dura AdCa
chr2	169067149	C	G	0.049	126	PASS	DHRS9	upstream_gene_variant	Dura AdCa
chr2	169906040	T	TA	0.039	155	PASS	UBR3	frameshift_variant	Dura AdCa
chr2	178557542	A	T	0.035	200	PASS	TTN	synonymous_variant	Dura AdCa
chr2	178704131	A	ATAGTGT	0.018	188	PASS	TTN	splice_donor_variant&splice_donor_5th_base_variant&c	Dura AdCa
chr2	178706595	A	T	0.027	180	PASS	TTN	missense_variant	Dura AdCa
chr2	179446806	AAGGCCC	A	0.033	124	PASS	ZNF385B	intron_variant	Dura AdCa
chr2	182838511	T	C	0.836	87	PASS	FRZB	missense_variant	Dura AdCa
chr2	189012698	C	T	0.057	103	PASS	COL3A1	3_prime_utr_variant	Dura AdCa
chr2	197615990	AGTTTGA	A	0.054	108	PASS	RFTN2	splice_polypyrimidine_tract_variant&intron_variant	Dura AdCa
chr2	201492394	A	T	0.073	62	PASS	C2CD6	missense_variant	Dura AdCa
chr2	206444098	A	C	0.389	75	PASS	ADAM23	intron_variant	Dura AdCa
chr2	216113088	GGTGTCT	G	0.045	241	PASS	XRCC5	stop_gained&frameshift_variant	Dura AdCa
chr2	218881099	A	C	0.071	68	PASS	WNT10A	missense_variant	Dura AdCa
chr2	223984920	TGATGGT	T	0.03	248	PASS	SERPINE2	stop_gained&frameshift_variant	Dura AdCa
chr2	231170703	A	T	0.068	61	PASS	PSMD1	synonymous_variant	Dura AdCa
chr2	232381426	T	C	0.407	296	PASS	ALPP	intron_variant	Dura AdCa
chr2	233457356	CAGGAG.	C	0.043	164	PASS	DGKD	intron_variant	Dura AdCa
chr2	239126446	A	G	0.04	146	PASS	HDAC4	intron_variant	Dura AdCa
chr3	10213059	CA	TG	0.481	115	PASS	IRAK2	intron_variant	Dura AdCa
chr3	11017287	G	A	0.583	696	PASS	SLC6A1	missense_variant	Dura AdCa
chr3	12004864	G	C	0.275	157	PASS	SYN2	missense_variant	Dura AdCa
chr3	13503039	A	G	0.347	200	PASS	HDAC11	intron_variant	Dura AdCa
chr3	14158878	C	G	0.559	391	PASS	XPC	synonymous_variant	Dura AdCa
chr3	14178475	T	C	0.613	800	PASS	XPC	missense_variant	Dura AdCa
chr3	27344259	C	G	0.509	165	PASS	NEK10	intron_variant	Dura AdCa
chr3	33218659	T	C	0.394	71	PASS	SUSD5	intron_variant	Dura AdCa
chr3	33400153	G	A	0.392	122	PASS	FBXL2	intron_variant&non_coding_transcript_variant	Dura AdCa
chr3	33842000	A	G	0.38	271	PASS	PDCD6IP	missense_variant	Dura AdCa
chr3	37065953	GTCAATC	G	0.08	84	PASS	LRRFIP2	splice_polypyrimidine_tract_variant&intron_variant	Dura AdCa
chr3	37111099	GGCCTCT	G	0.093	69	PASS	LRRFIP2	intron_variant	Dura AdCa
chr3	44446632	C	CTTTCCA	0.023	344	PASS	ZNF445	inframe_deletion	Dura AdCa
chr3	46673502	A	G	0.909	142	PASS	ALS2CL	intron_variant	Dura AdCa
chr3	47857472	C	G	0.772	210	PASS	MAP4	missense_variant	Dura AdCa
chr3	48183045	T	A	0.05	80	PASS	CDC25A	splice_polypyrimidine_tract_variant&intron_variant	Dura AdCa
chr3	48381707	A	G	0.782	114	PASS	FBXW12	synonymous_variant	Dura AdCa
chr3	49005095	C	A	0.873	297	PASS	P4HTM	intron_variant	Dura AdCa
chr3	50330624	T	C	0.051	373	PASS	RASSF1	missense_variant	Dura AdCa
chr3	57368206	C	T	0.828	100	PASS	DNAH12	synonymous_variant	Dura AdCa
chr3	57377088	G	A	0.871	132	PASS	DNAH12	synonymous_variant	Dura AdCa
chr3	57380252	A	G	0.897	74	PASS	DNAH12	intron_variant	Dura AdCa
chr3	58501333	T	A	0.018	229	PASS	KCTD6	missense_variant	Dura AdCa
chr3	69964963	CCTA	C	0.018	511	PASS	MITF	inframe_insertion	Dura AdCa
chr3	69964966	ACTGAAT	A	0.018	520	PASS	MITF	frameshift_variant	Dura AdCa
chr3	77558088	A	C	0.051	151	PASS	ROBO2	missense_variant	Dura AdCa
chr3	88139852	G	A	0.403	263	PASS	ZNF654	missense_variant	Dura AdCa
chr3	101671336	T	C	0.479	254	PASS	ZBTB11	missense_variant	Dura AdCa
chr3	108583144	A	C	0.461	83	PASS	CIP2A	intron_variant	Dura AdCa
chr3	113659042	T	TACAGAC	0.017	396	PASS	USF3	inframe_deletion	Dura AdCa
chr3	121460076	T	C	0.025	397	PASS	POLQ	missense_variant	Dura AdCa
chr3	122928646	CATCACA	C	0.041	258	PASS	SEMA5B	intron_variant	Dura AdCa
chr3	122928649	CTTTAAC	C	0.042	249	PASS	SEMA5B	intron_variant	Dura AdCa
chr3	123946748	A	T	0.026	183	PASS	CCDC14	intron_variant	Dura AdCa
chr3	124699935	A	G	0.349	344	PASS	KALRN	missense_variant	Dura AdCa
chr3	128119302	A	T	0.023	176	PASS	RUVBL1	intron_variant	Dura AdCa
chr3	132691283	C	A	0.496	168	PASS	NPHP3	missense_variant	Dura AdCa
chr3	140175914	C	T	0.113	79	PASS	CLSTN2	intron_variant	Dura AdCa
chr3	142371322	TTAGCAT	T	0.033	193	PASS	XRN1	stop_gained&inframe_insertion	Dura AdCa
chr3	142686375	AAGAGG.	A	0.041	226	PASS	PLS1	stop_gained&frameshift_variant&splice_region_variant	Dura AdCa
chr3	143652386	GTTTAAA	G	0.078	103	PASS	SLC9A9	intron_variant	Dura AdCa
chr3	143652389	TAAAGTC	T	0.079	100	PASS	SLC9A9	intron_variant	Dura AdCa
chr3	149167981	AAAACAA	A	0.061	127	PASS	HPS3	inframe_insertion&splice_region_variant	Dura AdCa
chr3	149167984	GTTATCT	G	0.061	123	PASS	HPS3	splice_donor_variant	Dura AdCa
chr3	151350163	TTC	T	0.039	181	PASS	MED12L	frameshift_variant	Dura AdCa
chr3	151445829	A	AGTTTCC	0.028	393	PASS	IGSF10	frameshift_variant	Dura AdCa
chr3	155916098	TGACACA	T	0.024	609	PASS	GMPS	frameshift_variant	Dura AdCa
chr3	155916100	AACAAG.	A	0.025	596	PASS	GMPS	stop_gained&frameshift_variant	Dura AdCa
chr3	157442871	AATTTTT	A	0.033	288	PASS	PTX3	stop_gained&frameshift_variant	Dura AdCa

chr3	169822988	TCTACCT	T	0.054	121	PASS	LRRIQ4	intron_variant	Dura AdCa
chr3	170281992	GCAGTTT	G	0.034	466	PASS	PRKCI	intron_variant	Dura AdCa
chr3	179973339	G	A	0.485	145	PASS	.	intron_variant&non_coding_transcript_variant	Dura AdCa
chr3	183341475	C	T	0.096	72	PASS	MCF2L2	intron_variant	Dura AdCa
chr3	184038505	C	T	0.18	395	PASS	HTR3D	missense_variant	Dura AdCa
chr3	184038509	C	T	0.183	397	PASS	HTR3D	synonymous_variant	Dura AdCa
chr3	195226778	CACCATT	C	0.031	390	PASS	XXYLT1	inframe_insertion	Dura AdCa
chr3	195226782	AAACAA	A	0.031	385	PASS	XXYLT1	stop_gained&protein_altering_variant	Dura AdCa
chr3	195618957	GA	AG	0.51	272	PASS	MUC20P1	non_coding_transcript_exon_variant	Dura AdCa
chr3	195780850	G	A	0.036	268	PASS	MUC4	missense_variant	Dura AdCa
chr3	195782362	A	AGCGGG	0.09	794	PASS	MUC4	inframe_deletion	Dura AdCa
chr3	195782376	A	ACCGGT	0.058	1184	PASS	MUC4	inframe_deletion	Dura AdCa
chr3	195782572	G	A	0.13	573	PASS	MUC4	missense_variant	Dura AdCa
chr3	195783262	TTGGTGA	T	0.345	977	PASS	MUC4	inframe_insertion	Dura AdCa
chr3	195784172	C	T	0.186	1644	PASS	MUC4	missense_variant	Dura AdCa
chr3	195785416	A	G	0.306	1278	PASS	MUC4	missense_variant	Dura AdCa
chr3	195786727	A	G	0.212	1507	PASS	MUC4	missense_variant	Dura AdCa
chr3	195787453	A	G	0.784	84	PASS	MUC4	missense_variant	Dura AdCa
chr3	195787461	G	C	0.16	94	PASS	MUC4	missense_variant	Dura AdCa
chr3	196270466	A	G	0.519	350	PASS	PCYT1A	synonymous_variant	Dura AdCa
chr4	373193	G	A	0.053	100	PASS	ZNF141	synonymous_variant	Dura AdCa
chr4	373202	C	T	0.052	100	PASS	ZNF141	synonymous_variant	Dura AdCa
chr4	2251199	A	C	0.454	216	PASS	MXD4	synonymous_variant	Dura AdCa
chr4	2328999	T	C	0.07	56	PASS	ZFYVE28	intron_variant	Dura AdCa
chr4	6026261	C	CAAAAA	0.028	104	PASS	C4orf50	splice_acceptor_variant&5_prime_utr_variant&intron_v	Dura AdCa
chr4	7014459	T	TTTTGCT	0.046	102	PASS	TBC1D14	inframe_deletion	Dura AdCa
chr4	9230471	A	C	0.048	1380	PASS	.	intron_variant&non_coding_transcript_variant	Dura AdCa
chr4	9230473	C	T	0.049	1354	PASS	.	intron_variant&non_coding_transcript_variant	Dura AdCa
chr4	9248548	A	G	0.06	431	PASS	.	intron_variant&non_coding_transcript_variant	Dura AdCa
chr4	9268697	T	C	0.115	61	PASS	USP17L22	missense_variant	Dura AdCa
chr4	9368706	C	T	0.037	208	PASS	.	intron_variant&non_coding_transcript_variant	Dura AdCa
chr4	15723507	C	T	0.06	79	PASS	BST1	intron_variant	Dura AdCa
chr4	16186869	C	G	0.46	86	PASS	TAPT1	missense_variant	Dura AdCa
chr4	25026839	T	TCAGCA	0.033	148	PASS	LGI2	splice_donor_variant&splice_donor_5th_base_variant&	Dura AdCa
chr4	26482222	A	G	0.065	63	PASS	CCKAR	intron_variant	Dura AdCa
chr4	30927343	T	C	0.098	59	PASS	PCDH7	intron_variant	Dura AdCa
chr4	37445904	AAGCGT	A	0.053	252	PASS	NWD2	frameshift_variant	Dura AdCa
chr4	37445906	CGTTGTC	C	0.053	254	PASS	NWD2	stop_gained&inframe_insertion	Dura AdCa
chr4	37847210	ATGCTTC	A	0.023	256	PASS	PGM2	frameshift_variant	Dura AdCa
chr4	38915120	A	G	0.058	99	PASS	FAM114A1	intron_variant	Dura AdCa
chr4	41620613	TAGGAAT	T	0.022	406	PASS	LIMCH1	inframe_insertion	Dura AdCa
chr4	54258827	CACCACC	C	0.032	369	PASS	PDGFRA	intron_variant	Dura AdCa
chr4	54274958	GCTGCCC	G	0.04	537	PASS	PDGFRA	stop_gained&protein_altering_variant	Dura AdCa
chr4	54278315	A	T	0.029	146	PASS	PDGFRA	intron_variant	Dura AdCa
chr4	69489690	C	G	0.108	52	PASS	UGT2B4	intron_variant	Dura AdCa
chr4	69647410	C	CAAAGG	0.021	321	PASS	UGT2A1	frameshift_variant	Dura AdCa
chr4	70637844	T	G	0.464	176	PASS	.	splice_donor_variant&non_coding_transcript_variant	Dura AdCa
chr4	71453479	C	CATTCTC	0.023	226	PASS	SLC4A4	splice_acceptor_variant&coding_sequence_variant&intro	Dura AdCa
chr4	71453674	T	G	0.035	129	PASS	SLC4A4	splice_donor_5th_base_variant&intron_variant	Dura AdCa
chr4	73142187	T	A	0.085	67	PASS	ANKRD17	intron_variant	Dura AdCa
chr4	74038340	A	T	0.395	424	PASS	CXCL3	missense_variant	Dura AdCa
chr4	74241562	A	T	0.046	134	PASS	MTHFD2L	intron_variant	Dura AdCa
chr4	76740701	T	A	0.438	385	PASS	SHROOM3	missense_variant	Dura AdCa
chr4	78363508	T	TCCAGAC	0.015	326	PASS	FRAS1	splice_acceptor_variant&coding_sequence_variant&intro	Dura AdCa
chr4	82456885	G	T	0.092	65	PASS	ENOPH1	intron_variant	Dura AdCa
chr4	83462706	GATTATA	G	0.024	474	PASS	ABRAXAS1	stop_gained&frameshift_variant	Dura AdCa
chr4	84766363	TATGAGC	T	0.059	131	PASS	WDFY3	frameshift_variant	Dura AdCa
chr4	86735605	C	G	0.034	130	PASS	PTPN13	synonymous_variant	Dura AdCa
chr4	88486193	T	G	0.04	239	PASS	HERC5	stop_gained	Dura AdCa
chr4	105940249	ATTTTTA	A	0.075	176	PASS	NPNT	intron_variant	Dura AdCa
chr4	105940251	AAAGTAC	A	0.075	174	PASS	NPNT	intron_variant	Dura AdCa
chr4	106924081	CTCCACC	C	0.028	320	PASS	DKK2	frameshift_variant	Dura AdCa
chr4	106924082	GTTACTT	G	0.029	318	PASS	DKK2	stop_gained&frameshift_variant	Dura AdCa
chr4	109851805	T	A	0.015	394	PASS	LRIT3	stop_gained	Dura AdCa
chr4	113509558	G	C	0.049	122	PASS	CAMK2D	intron_variant	Dura AdCa
chr4	125317555	A	G	0.088	600	PASS	FAT4	missense_variant	Dura AdCa
chr4	125434296	C	T	0.436	122	PASS	FAT4	missense_variant	Dura AdCa
chr4	138219325	AATGAGA	A	0.046	199	PASS	SLC7A11	frameshift_variant	Dura AdCa
chr4	150928642	A	T	0.388	81	PASS	LRBA	intron_variant	Dura AdCa
chr4	155710866	T	C	0.521	212	PASS	GUCY1A1	missense_variant	Dura AdCa
chr4	158682445	G	T	0.06	83	PASS	ETFDH	intron_variant	Dura AdCa
chr4	159347013	TATAAAC	T	0.046	149	PASS	RAPGEF2	intron_variant	Dura AdCa
chr4	159347017	TACCATT	T	0.046	143	PASS	RAPGEF2	intron_variant	Dura AdCa
chr4	162111279	A	G	0.042	142	PASS	FSTL5	stop_gained	Dura AdCa
chr4	165224001	A	G	0.15	243	PASS	KLHL2	intron_variant	Dura AdCa
chr4	165487532	CATTGTA	C	0.048	136	PASS	CPE	stop_gained&frameshift_variant	Dura AdCa
chr4	165487535	CCTGCTC	C	0.049	127	PASS	CPE	frameshift_variant	Dura AdCa
chr4	173374079	A	G	0.087	62	PASS	SAP30	intron_variant	Dura AdCa
chr4	174977785	G	GTGATGC	0.011	1285	PASS	ADAM29	stop_lost&3_prime_utr_variant	Dura AdCa
chr4	177440439	TGCTGTC	T	0.052	127	PASS	AGA	splice_polypyrimidine_tract_variant&intron_variant	Dura AdCa
chr4	182731123	C	T	0.073	56	PASS	TENM3	missense_variant	Dura AdCa
chr4	183694013	C	T	0.139	130	PASS	TRAPPC11	missense_variant	Dura AdCa
chr4	185405762	T	G	0.139	57	PASS	UFSP2	intron_variant	Dura AdCa
chr4	185471082	T	C	0.306	55	PASS	CCDC110	intron_variant	Dura AdCa
chr4	186238251	C	T	0.152	62	PASS	KLKB1	splice_region_variant&splice_polypyrimidine_tract_varia	Dura AdCa
chr4	186598131	TGCAGTC	T	0.056	138	PASS	FAT1	splice_region_variant&splice_polypyrimidine_tract_varia	Dura AdCa

chr4	186618709	T	C	0.031	242	PASS	FAT1	missense_variant	Dura AdCa
chr5	205756	A	AGAGAT/	0.012	416	PASS	CCDC127	inframe_deletion	Dura AdCa
chr5	878575	T	C	0.233	297	PASS	BRD9	intron_variant	Dura AdCa
chr5	1432496	G	A	0.488	218	PASS	SLC6A3	synonymous_variant	Dura AdCa
chr5	32780689	G	C	0.49	225	PASS	NPR3	intron_variant	Dura AdCa
chr5	34811682	T	TTCTCTT	0.029	212	PASS	RAI14	intron_variant	Dura AdCa
chr5	37020455	G	C	0.067	85	PASS	NIPBL	splice_region_variant&splice_polypyrimidine_tract_varia	Dura AdCa
chr5	57214174	A	C	0.852	164	PASS	GPBP1	missense_variant	Dura AdCa
chr5	61332326	GGGC	G	0.474	162	PASS	ZSWIM6	inframe_insertion	Dura AdCa
chr5	62580946	T	C	0.35	167	PASS	LRRC70	missense_variant	Dura AdCa
chr5	68293766	A	AACACT(	0.025	441	PASS	PIK3R1	splice_donor_variant&splice_donor_5th_base_variant&c	Dura AdCa
chr5	75620372	TCCCCCC	T	0.069	134	PASS	ANKDD1B	stop_gained&frameshift_variant	Dura AdCa
chr5	79512870	T	C	0.04	54	PASS	HOMER1	5_prime_utr_variant	Dura AdCa
chr5	79512873	T	C	0.042	52	PASS	HOMER1	5_prime_utr_variant	Dura AdCa
chr5	87333374	AGCTCC/	A	0.09	219	PASS	CCNH	intron_variant&nmd_transcript_variant	Dura AdCa
chr5	87333377	ACAAAT(	A	0.09	217	PASS	RASA1	intron_variant	Dura AdCa
chr5	90619073	A	G	0.068	67	PASS	ADGRV1	splice_polypyrimidine_tract_variant&intron_variant	Dura AdCa
chr5	96793534	ACTTTCT	A	0.098	68	PASS	ERAP1	intron_variant	Dura AdCa
chr5	98779727	A	C	0.018	401	PASS	RGMB	missense_variant	Dura AdCa
chr5	100895796	AAGTTTA	A	0.057	77	PASS	ST8SIA4	splice_polypyrimidine_tract_variant&intron_variant	Dura AdCa
chr5	103129686	C	G	0.058	110	PASS	PPIP5K2	missense_variant	Dura AdCa
chr5	111092331	T	C	0.116	250	PASS	WDR36	upstream_gene_variant	Dura AdCa
chr5	112839132	AATTTGT	A	0.051	273	PASS	APC	frameshift_variant	Dura AdCa
chr5	122151988	A	T	0.052	85	PASS	ZNF474	5_prime_utr_variant	Dura AdCa
chr5	128519394	CTCT	C	0.095	60	PASS	FBN2	intron_variant	Dura AdCa
chr5	128519395	AATAAG/	A	0.098	58	PASS	FBN2	intron_variant	Dura AdCa
chr5	138166713	AGGCTG	A	0.072	69	PASS	BRD8	frameshift_variant	Dura AdCa
chr5	138166714	AGAGAG	A	0.072	69	PASS	BRD8	frameshift_variant	Dura AdCa
chr5	138917803	C	T	0.025	252	PASS	CTNNA1	missense_variant	Dura AdCa
chr5	140648249	G	A	0.137	136	PASS	IK	intron_variant	Dura AdCa
chr5	141331788	G	T	0.043	97	PASS	PCDHGA1	synonymous_variant	Dura AdCa
chr5	143300607	A	T	0.029	157	PASS	NR3C1	missense_variant	Dura AdCa
chr5	150238651	GAGCAC(	G	0.044	170	PASS	CAMK2A	intron_variant	Dura AdCa
chr5	159174135	A	G	0.521	217	PASS	RNF145	synonymous_variant	Dura AdCa
chr5	172968611	T	G	0.377	296	PASS	RPL26L1	intron_variant	Dura AdCa
chr5	175926725	C	T	0.05	185	PASS	.	intron_variant&non_coding_transcript_variant	Dura AdCa
chr5	175959996	T	C	0.152	421	PASS	THOC3	synonymous_variant	Dura AdCa
chr5	177672139	A	T	0.228	229	PASS	.	non_coding_transcript_exon_variant	Dura AdCa
chr5	178147850	A	G	0.409	417	PASS	RMND5B	missense_variant	Dura AdCa
chr5	179076409	T	G	0.434	359	PASS	ZNF354C	intron_variant	Dura AdCa
chr6	147522	A	G	0.241	96	PASS	SEPTIN14P6	upstream_gene_variant	Dura AdCa
chr6	4125973	T	C	0.487	106	PASS	ECI2	intron_variant	Dura AdCa
chr6	4996429	A	C	0.439	75	PASS	RPP40	splice_polypyrimidine_tract_variant&intron_variant	Dura AdCa
chr6	8080065	ACACAT1	A	0.057	95	PASS	EEF1E1	intron_variant	Dura AdCa
chr6	10586221	C	CTGACC(	0.014	361	PASS	GCNT2	intron_variant	Dura AdCa
chr6	10874944	GCCACC(	G	0.033	180	PASS	GCM2	splice_polypyrimidine_tract_variant&intron_variant	Dura AdCa
chr6	24405209	T	A	0.027	242	PASS	MRS2	missense_variant	Dura AdCa
chr6	25777988	T	C	0.413	127	PASS	SLC17A4	missense_variant	Dura AdCa
chr6	30703938	A	T	0.01	733	PASS	MDC1	stop_gained	Dura AdCa
chr6	30704388	A	ACCTAG1	0.034	1985	PASS	MDC1	inframe_deletion	Dura AdCa
chr6	30704511	CCCTAG1	C	0.027	1161	PASS	MDC1	inframe_insertion	Dura AdCa
chr6	30704520	C	CCCGAG/	0.012	2030	PASS	MDC1	inframe_deletion	Dura AdCa
chr6	30705205	C	G	0.012	906	PASS	MDC1	synonymous_variant	Dura AdCa
chr6	30705308	C	CGGAGC1	0.01	1820	PASS	MDC1	inframe_deletion	Dura AdCa
chr6	30705328	C	CACAAC1	0.025	1944	PASS	MDC1	inframe_deletion	Dura AdCa
chr6	30986772	C	G	0.012	686	PASS	MUC21	missense_variant	Dura AdCa
chr6	31010791	ATTCTTA	A	0.085	133	PASS	MUC22	intron_variant	Dura AdCa
chr6	31202450	GC	AG	0.505	308	PASS	HCG27	non_coding_transcript_exon_variant	Dura AdCa
chr6	31622954	A	G	0.064	81	PASS	PRRC2A	intron_variant	Dura AdCa
chr6	31632919	A	G	0.536	197	PASS	PRRC2A	missense_variant	Dura AdCa
chr6	31688737	T	C	0.55	244	PASS	ABHD16A	synonymous_variant	Dura AdCa
chr6	31810526	T	C	0.02	346	PASS	HSPA1L	missense_variant	Dura AdCa
chr6	32011008	T	C	0.056	312	PASS	TNXA	non_coding_transcript_exon_variant	Dura AdCa
chr6	32027420	T	C	0.045	135	PASS	C4B	missense_variant	Dura AdCa
chr6	32029823	G	C	0.475	549	PASS	C4B	intron_variant	Dura AdCa
chr6	32223104	A	G	0.479	218	PASS	NOTCH4	intron_variant	Dura AdCa
chr6	32554821	G	A	0.225	465	PASS	HLA-DRB6	non_coding_transcript_exon_variant	Dura AdCa
chr6	32554826	C	G	0.222	470	PASS	HLA-DRB6	non_coding_transcript_exon_variant	Dura AdCa
chr6	32746281	G	T	0.038	676	PASS	HLA-DQA2	missense_variant	Dura AdCa
chr6	32746314	G	A	0.035	635	PASS	HLA-DQA2	missense_variant	Dura AdCa
chr6	34417760	A	G	0.446	118	PASS	RPS10	intron_variant	Dura AdCa
chr6	35872740	TTAGGA/	T	0.047	145	PASS	SRPK1	splice_polypyrimidine_tract_variant&intron_variant	Dura AdCa
chr6	39714659	A	C	0.038	81	PASS	KIF6	intron_variant	Dura AdCa
chr6	39714663	A	C	0.037	83	PASS	KIF6	intron_variant	Dura AdCa
chr6	41780279	G	GGGAGG	0.014	301	PASS	FRS3	upstream_gene_variant	Dura AdCa
chr6	41780315	G	GGGGGA	0.012	412	PASS	PRICKLE4	upstream_gene_variant	Dura AdCa
chr6	43188137	A	G	0.03	175	PASS	CUL9	intron_variant	Dura AdCa
chr6	46799256	CTAAAC1	C	0.108	52	PASS	MEP1A	intron_variant	Dura AdCa
chr6	47879234	A	T	0.541	170	PASS	PTCHD4	missense_variant	Dura AdCa
chr6	52069577	A	G	0.035	134	PASS	PKHD1	intron_variant	Dura AdCa
chr6	63685295	A	G	0.469	161	PASS	PHF3	missense_variant	Dura AdCa
chr6	70755016	T	C	0.728	71	PASS	SMAP1	missense_variant	Dura AdCa
chr6	75695845	A	G	0.111	113	PASS	SENP6	missense_variant	Dura AdCa
chr6	83607670	C	A	0.04	74	PASS	SNAP91	intron_variant	Dura AdCa
chr6	89661496	C	T	0.884	135	PASS	MDN1	missense_variant	Dura AdCa
chr6	97064696	C	T	0.091	105	PASS	KLHL32	synonymous_variant	Dura AdCa

chr6	106975088	A	G	0.114	65	PASS	CD24	upstream_gene_variant	Dura AdCa
chr6	107707976	A	G	0.139	206	PASS	SCML4	missense_variant	Dura AdCa
chr6	110621142	T	C	0.045	209	PASS	CDK19	missense_variant	Dura AdCa
chr6	116121523	T	C	0.084	136	PASS	COL10A1	missense_variant	Dura AdCa
chr6	116558149	GTGATG(	G	0.128	118	PASS	CALHM4	stop_gained&frameshift_variant	Dura AdCa
chr6	117519881	T	C	0.857	97	PASS	DCBLD1	missense_variant	Dura AdCa
chr6	118911780	A	G	0.073	55	PASS	MCM9	splice_polypyrimidine_tract_variant&intron_variant	Dura AdCa
chr6	125889651	A	G	0.886	72	PASS	NCOA7	missense_variant	Dura AdCa
chr6	129154520	A	G	0.051	117	PASS	LAMA2	missense_variant	Dura AdCa
chr6	142133970	A	G	0.463	155	PASS	GJE1	missense_variant	Dura AdCa
chr6	149718012	A	G	0.465	305	PASS	LATS1	5_prime_utr_variant	Dura AdCa
chr6	149718157	C	T	0.479	291	PASS	.	non_coding_transcript_exon_variant	Dura AdCa
chr6	149718225	G	C	0.488	277	PASS	.	non_coding_transcript_exon_variant	Dura AdCa
chr6	160043250	CCCGGG'	C	0.039	220	PASS	IGF2R	frameshift_variant	Dura AdCa
chr6	160043253	GGTGAA(	G	0.039	220	PASS	IGF2R	stop_gained&frameshift_variant	Dura AdCa
chr6	167172375	A	G	0.032	137	PASS	TCP10L2	intron_variant&non_coding_transcript_variant	Dura AdCa
chr6	167911223	A	ATTTTCA	0.013	466	PASS	AFDN	splice_polypyrimidine_tract_variant&intron_variant	Dura AdCa
chr6	169667944	A	G	0.436	357	PASS	WDR27	intron_variant	Dura AdCa
chr7	726577	GACGAT	G	0.154	90	PASS	PRKAR1B	intron_variant	Dura AdCa
chr7	726583	G	A	0.137	99	PASS	PRKAR1B	intron_variant	Dura AdCa
chr7	2572660	AA	TC	0.816	53	PASS	IQCE	intron_variant	Dura AdCa
chr7	30686470	C	T	0.051	88	PASS	CRHR2	upstream_gene_variant	Dura AdCa
chr7	32559175	AA	CC	0.043	62	PASS	AVL9	missense_variant	Dura AdCa
chr7	32559180	C	G	0.042	62	PASS	AVL9	missense_variant	Dura AdCa
chr7	37225044	C	T	0.811	123	PASS	ELMO1	splice_polypyrimidine_tract_variant&intron_variant	Dura AdCa
chr7	44007301	A	G	0.75	232	PASS	SPDYE1	synonymous_variant	Dura AdCa
chr7	50368279	GGGATA'	G	0.071	82	PASS	IKZF1	intron_variant	Dura AdCa
chr7	55939499	T	G	0.109	78	PASS	ZNF713	missense_variant	Dura AdCa
chr7	57132116	T	C	0.776	112	PASS	ZNF479	intron_variant	Dura AdCa
chr7	64979541	A	AAGAAA'	0.092	72	PASS	ZNF117	splice_region_variant&splice_polypyrimidine_tract_varia	Dura AdCa
chr7	73328589	A	G	0.914	50	PASS	FKBP6	synonymous_variant	Dura AdCa
chr7	73447351	G	C	0.036	87	PASS	BAZ1B	missense_variant	Dura AdCa
chr7	77953963	G	C	0.885	70	PASS	PHTF2	intron_variant	Dura AdCa
chr7	78521465	GACCAG/	G	0.034	237	PASS	MAGI2	stop_gained&frameshift_variant	Dura AdCa
chr7	83135238	GTTGTTT	G	0.039	251	PASS	PCLO	stop_gained&frameshift_variant	Dura AdCa
chr7	92307486	G	C	0.035	175	PASS	ANKIB1	missense_variant	Dura AdCa
chr7	92307491	AATTAG(	A	0.036	174	PASS	ANKIB1	stop_gained&inframe_insertion	Dura AdCa
chr7	100682882	T	C	0.846	88	PASS	GIGYF1	intron_variant	Dura AdCa
chr7	100952147	TG	CA	0.44	520	PASS	MUC3A	missense_variant	Dura AdCa
chr7	100952493	G	A	0.331	203	PASS	MUC3A	synonymous_variant	Dura AdCa
chr7	100952498	T	C	0.349	200	PASS	MUC3A	missense_variant	Dura AdCa
chr7	103319617	G	C	0.031	151	PASS	DNAJC2	missense_variant	Dura AdCa
chr7	108079749	AGTTCTI	A	0.073	137	PASS	LAMB4	stop_gained&protein_altering_variant	Dura AdCa
chr7	111846967	A	G	0.935	66	PASS	DOCK4	intron_variant	Dura AdCa
chr7	127699019	A	T	0.081	54	PASS	SND1	intron_variant	Dura AdCa
chr7	128880045	A	C	0.087	203	PASS	KCP	missense_variant	Dura AdCa
chr7	130129605	C	T	0.117	137	PASS	KLHDC10	intron_variant	Dura AdCa
chr7	130550642	T	C	0.11	101	PASS	COPG2	synonymous_variant	Dura AdCa
chr7	131445908	GAGGAA(	G	0.051	146	PASS	MKLN1	splice_donor_region_variant&intron_variant	Dura AdCa
chr7	134450935	T	C	0.032	191	PASS	AKR1B1	intron_variant	Dura AdCa
chr7	139341370	G	GAGA	0.018	343	PASS	FMC1	5_prime_utr_variant	Dura AdCa
chr7	142022437	A	G	0.055	90	PASS	MGAM	missense_variant&splice_region_variant	Dura AdCa
chr7	142345971	C	T	0.59	385	PASS	TRBV4-2	synonymous_variant	Dura AdCa
chr7	142451194	CA	GG	0.169	237	PASS	TRBV6-5	missense_variant	Dura AdCa
chr7	142463547	G	C	0.325	243	PASS	TRBV5-4	missense_variant	Dura AdCa
chr7	142564042	T	C	0.903	191	PASS	TRBV12-4	synonymous_variant	Dura AdCa
chr7	142588240	A	G	0.908	104	PASS	TRBV14	synonymous_variant	Dura AdCa
chr7	142598094	T	C	0.868	142	PASS	TRBV16	intron_variant	Dura AdCa
chr7	142774511	T	C	0.348	295	PASS	PRSS2	3_prime_utr_variant	Dura AdCa
chr7	142774515	CA	TG	0.353	286	PASS	PRSS2	3_prime_utr_variant	Dura AdCa
chr7	142791704	A	C	0.115	314	PASS	TRBC1	missense_variant	Dura AdCa
chr7	142791707	C	G	0.116	312	PASS	TRBC1	missense_variant	Dura AdCa
chr7	142791866	A	G	0.23	425	PASS	TRBC1	synonymous_variant	Dura AdCa
chr7	149005223	A	G	0.064	171	PASS	PDIA4	synonymous_variant	Dura AdCa
chr7	152252528	A	G	0.922	198	PASS	KMT2C	intron_variant	Dura AdCa
chr7	158759980	T	A	0.048	58	PASS	ESYT2	intron_variant	Dura AdCa
chr7	159031917	T	C	0.881	227	PASS	VIPR2	intron_variant	Dura AdCa
chr8	1680556	AT	GA	0.128	111	PASS	DLGAP2	intron_variant	Dura AdCa
chr8	7359734	T	G	0.54	62	PASS	ZNF705G	intron_variant	Dura AdCa
chr8	7541016	G	C	0.379	168	PASS	PRR23D1	synonymous_variant	Dura AdCa
chr8	11284101	T	G	0.033	131	PASS	.	non_coding_transcript_exon_variant	Dura AdCa
chr8	18872551	G	C	0.086	190	PASS	PSD3	missense_variant	Dura AdCa
chr8	21750758	A	G	0.024	331	PASS	GFRA2	synonymous_variant	Dura AdCa
chr8	22227651	T	C	0.816	86	PASS	PHYHIP	intron_variant	Dura AdCa
chr8	23449922	CCTATTT	C	0.092	135	PASS	ENTPD4	5_prime_utr_variant	Dura AdCa
chr8	23449924	ACTAGT/	A	0.092	135	PASS	ENTPD4	5_prime_utr_variant	Dura AdCa
chr8	24917240	TTGA	T	0.099	54	PASS	NEFM	inframe_insertion	Dura AdCa
chr8	26363765	C	G	0.017	287	PASS	PPP2R2A	missense_variant	Dura AdCa
chr8	30838036	G	C	0.048	97	PASS	TEX15	missense_variant	Dura AdCa
chr8	33511942	A	G	0.875	62	PASS	TTI2	intron_variant	Dura AdCa
chr8	38264413	G	A	0.025	106	PASS	PLPP5	3_prime_utr_variant	Dura AdCa
chr8	38264423	G	A	0.022	123	PASS	PLPP5	3_prime_utr_variant	Dura AdCa
chr8	39071313	G	C	0.042	216	PASS	ADAM9	missense_variant	Dura AdCa
chr8	41978582	C	CTACACT	0.014	486	PASS	KAT6A	splice_donor_5th_base_variant&intron_variant	Dura AdCa
chr8	52940004	T	C	0.465	373	PASS	NPBWR1	missense_variant	Dura AdCa
chr8	54055194	TCCTTTT	T	0.136	91	PASS	LYPLA1	intron_variant	Dura AdCa

chr8	66665153	G	C	0.022	352	PASS	VCPIP1	synonymous_variant	Dura AdCa
chr8	68815981	GCTAGTC	G	0.054	139	PASS	C8orf34	intron_variant	Dura AdCa
chr8	81694301	AGGTATC	A	0.026	276	PASS	SLC10A5	stop_gained&frameshift_variant	Dura AdCa
chr8	85473391	AAGATTC	A	0.051	147	PASS	CA2	intron_variant	Dura AdCa
chr8	88116494	G	C	0.023	163	PASS	MMP16	intron_variant	Dura AdCa
chr8	93797378	ATGTGTT	A	0.027	529	PASS	TMEM67	stop_gained&protein_altering_variant	Dura AdCa
chr8	93797379	CAAAGAC	C	0.027	526	PASS	TMEM67	inframe_insertion	Dura AdCa
chr8	103921828	A	AGTGTTT	0.03	144	PASS	RIMS2	intron_variant	Dura AdCa
chr8	124998624	A	G	0.334	336	PASS	SQLE	5_prime_utr_variant	Dura AdCa
chr8	131946634	C	G	0.667	183	PASS	EFR3A	splice_donor_variant	Dura AdCa
chr8	143606178	T	G	0.769	213	PASS	PYCR3	intron_variant	Dura AdCa
chr8	143858206	A	G	0.265	425	PASS	EPPK1	synonymous_variant	Dura AdCa
chr8	143865354	G	GGGC	0.975	58	PASS	EPPK1	inframe_deletion	Dura AdCa
chr8	143928985	G	C	0.039	62	PASS	PLEC	intron_variant	Dura AdCa
chr8	144057755	A	G	0.029	172	PASS	OPLAH	intron_variant	Dura AdCa
chr8	144264608	A	G	0.319	576	PASS	BOP1	synonymous_variant	Dura AdCa
chr8	144318358	A	G	0.34	512	PASS	DGAT1	synonymous_variant	Dura AdCa
chr8	144391547	C	A	0.308	270	PASS	ADCK5	intron_variant	Dura AdCa
chr8	144426161	T	G	0.346	283	PASS	VPS28	intron_variant	Dura AdCa
chr8	144513765	G	T	0.077	277	PASS	RECQL4	intron_variant	Dura AdCa
chr8	144931491	A	G	0.368	346	PASS	ZNF16	synonymous_variant	Dura AdCa
chr9	5522632	AAACAGC	A	0.046	154	PASS	.	intron_variant&non_coding_transcript_variant	Dura AdCa
chr9	7011765	T	C	0.339	127	PASS	KDM4C	synonymous_variant	Dura AdCa
chr9	8389333	A	G	0.038	261	PASS	PTPRD	stop_gained	Dura AdCa
chr9	8484244	CAACTGC	C	0.05	115	PASS	PTPRD	stop_gained&protein_altering_variant	Dura AdCa
chr9	22451048	C	T	0.446	69	PASS	DMRTA1	splice_polypyrimidine_tract_variant&intron_variant	Dura AdCa
chr9	27190589	T	TCAACAT	0.017	434	PASS	TEK	frameshift_variant	Dura AdCa
chr9	35842535	A	G	0.079	250	PASS	TMEM8B	missense_variant	Dura AdCa
chr9	41926631	A	T	0.035	86	PASS	CNTNAP3B	intron_variant	Dura AdCa
chr9	78031131	A	G	0.017	557	PASS	GNAQ	synonymous_variant	Dura AdCa
chr9	78241618	G	GAAGAGC	0.021	237	PASS	CEP78	splice_acceptor_variant&coding_sequence_variant&intro	Dura AdCa
chr9	78308346	T	G	0.051	92	PASS	PSAT1	intron_variant	Dura AdCa
chr9	78317564	A	C	0.34	64	PASS	PSAT1	intron_variant	Dura AdCa
chr9	78317567	T	A	0.34	64	PASS	PSAT1	intron_variant	Dura AdCa
chr9	81992641	C	T	0.453	219	PASS	SPATA31D1	missense_variant	Dura AdCa
chr9	82058619	CA	GC	0.264	407	PASS	.	intron_variant&non_coding_transcript_variant	Dura AdCa
chr9	86007425	A	C	0.019	310	PASS	NAA35	missense_variant	Dura AdCa
chr9	97366728	GTTCCAT	G	0.038	220	PASS	CCDC180	intron_variant	Dura AdCa
chr9	97366729	GGTCAAA	G	0.038	217	PASS	CCDC180	intron_variant	Dura AdCa
chr9	97460787	TTCTTC	T	0.046	168	PASS	TDRD7	intron_variant	Dura AdCa
chr9	97460789	ACCTGCA	A	0.046	168	PASS	TDRD7	intron_variant	Dura AdCa
chr9	99227934	A	G	0.014	475	PASS	SEC61B	missense_variant	Dura AdCa
chr9	101317635	CTACTAA	C	0.1	56	PASS	PLPPR1	intron_variant	Dura AdCa
chr9	104617172	G	A	0.573	113	PASS	OR13C9	downstream_gene_variant	Dura AdCa
chr9	111722532	TCCATTT	T	0.03	266	PASS	SHOC1	stop_gained&frameshift_variant	Dura AdCa
chr9	123010502	CAAGGTT	C	0.037	167	PASS	RABGAP1	stop_gained&frameshift_variant	Dura AdCa
chr9	125559714	TTTTCC	T	0.029	272	PASS	MAPKAP1	frameshift_variant	Dura AdCa
chr9	125559717	TAAAGGC	T	0.029	264	PASS	MAPKAP1	frameshift_variant	Dura AdCa
chr9	125929787	CAAGAAT	C	0.063	244	PASS	PBX3	stop_gained&frameshift_variant	Dura AdCa
chr9	125929790	AGACGTC	A	0.064	237	PASS	PBX3	frameshift_variant	Dura AdCa
chr9	127738860	A	G	0.525	405	PASS	SH2D3C	synonymous_variant	Dura AdCa
chr9	128970940	TTCCTCT	T	0.044	203	PASS	NUP188	frameshift_variant	Dura AdCa
chr9	128970942	CTCCTTA	C	0.044	195	PASS	NUP188	stop_gained&inframe_insertion	Dura AdCa
chr9	128998146	C	T	0.514	330	PASS	NUP188	splice_region_variant&splice_polypyrimidine_tract_varia	Dura AdCa
chr9	130319410	G	A	0.989	131	PASS	HMCN2	intron_variant	Dura AdCa
chr9	130319644	A	G	0.466	275	PASS	HMCN2	missense_variant	Dura AdCa
chr9	130321760	G	A	0.359	190	PASS	HMCN2	intron_variant	Dura AdCa
chr9	130327557	C	T	0.636	168	PASS	HMCN2	intron_variant	Dura AdCa
chr9	130338375	G	A	0.555	275	PASS	HMCN2	intron_variant	Dura AdCa
chr9	130338512	G	A	0.582	238	PASS	HMCN2	intron_variant	Dura AdCa
chr9	130612602	T	A	0.078	97	PASS	FUBP3	intron_variant	Dura AdCa
chr9	136250842	A	C	0.026	361	PASS	CCDC187	3_prime_utr_variant	Dura AdCa
chr9	136290477	G	A	0.987	117	PASS	CCDC187	intron_variant	Dura AdCa
chr9	136291438	T	C	0.6	252	PASS	CCDC187	missense_variant	Dura AdCa
chr9	136459582	T	C	0.664	256	PASS	SEC16A	intron_variant	Dura AdCa
chr9	136740862	C	T	0.531	326	PASS	LCN10	missense_variant	Dura AdCa
chr9	136863311	T	G	0.039	139	PASS	EDF1	missense_variant	Dura AdCa
chr9	137031182	T	C	0.296	418	PASS	FUT7	missense_variant	Dura AdCa
chr9	137064633	T	C	0.376	356	PASS	SAPCD2	3_prime_utr_variant	Dura AdCa
chr9	137213879	A	G	0.362	349	PASS	NDOR1	splice_donor_variant	Dura AdCa
chr9	137220533	T	C	0.34	455	PASS	RNF208	stop_gained	Dura AdCa
chr9	137877982	A	G	0.191	122	PASS	CACNA1B	missense_variant	Dura AdCa
chr9	137877990	A	G	0.223	146	PASS	CACNA1B	synonymous_variant	Dura AdCa
chr10	5132650	A	C	0.4	152	PASS	AKR1C5P	non_coding_transcript_exon_variant	Dura AdCa
chr10	12117710	A	C	0.452	150	PASS	DHTKD1	missense_variant	Dura AdCa
chr10	15103534	A	AAAGAA.	0.02	279	PASS	RPP38	frameshift_variant	Dura AdCa
chr10	19327707	A	T	0.55	81	PASS	MALRD1	intron_variant	Dura AdCa
chr10	25598926	G	A	0.43	218	PASS	GPR158	synonymous_variant	Dura AdCa
chr10	28534306	T	G	0.038	189	PASS	WAC	intron_variant	Dura AdCa
chr10	31908696	G	T	0.434	252	PASS	ARHGAP12	missense_variant	Dura AdCa
chr10	43077384	A	G	0.093	245	PASS	RET	intron_variant	Dura AdCa
chr10	43086608	C	T	0.082	93	PASS	RET	intron_variant	Dura AdCa
chr10	43112909	CTCCAGT	C	0.021	368	PASS	RET	stop_gained&frameshift_variant	Dura AdCa
chr10	43386718	C	G	0.04	215	PASS	HNRNPF	stop_gained	Dura AdCa
chr10	45303617	TA	T	0.87	166	PASS	OR13A1	frameshift_variant	Dura AdCa
chr10	45463744	T	C	0.131	181	PASS	MARCHF8	synonymous_variant	Dura AdCa

chr10	47367976	T	C	0.859	228	PASS	ZNF488	missense_variant	Dura AdCa
chr10	47762988	A	G	0.17	54	PASS	SYT15B	5_prime_utr_variant	Dura AdCa
chr10	48424208	TGGTCCT	T	0.083	89	PASS	MAPK8	intron_variant	Dura AdCa
chr10	48652213	G	A	0.851	76	PASS	ARHGAP22	intron_variant	Dura AdCa
chr10	49155796	T	C	0.1	58	PASS	TMEM273	3_prime_utr_variant	Dura AdCa
chr10	49515466	T	C	0.041	108	PASS	ERCC6	intron_variant	Dura AdCa
chr10	49744055	G	C	0.114	164	PASS	OGDHL	missense_variant	Dura AdCa
chr10	52768241	C	T	0.127	113	PASS	MBL2	missense_variant	Dura AdCa
chr10	62085759	A	AATCAA	0.014	348	PASS	ARID5B	inframe_deletion	Dura AdCa
chr10	63619825	CTCATCT	C	0.124	78	PASS	REEP3	intron_variant	Dura AdCa
chr10	68416810	TCTTGAA	T	0.023	546	PASS	DNA2	stop_gained&frameshift_variant	Dura AdCa
chr10	73138116	A	C	0.067	63	PASS	ECD	intron_variant	Dura AdCa
chr10	73852191	A	G	0.039	152	PASS	CAMK2G	intron_variant	Dura AdCa
chr10	91992300	ATCCTTT	A	0.095	76	PASS	BTAF1	stop_gained&frameshift_variant	Dura AdCa
chr10	92999360	A	G	0.041	147	PASS	EXOC6	intron_variant	Dura AdCa
chr10	93325895	G	A	0.886	200	PASS	MYOF	synonymous_variant	Dura AdCa
chr10	93431454	T	G	0.021	220	PASS	MYOF	missense_variant	Dura AdCa
chr10	96955218	TCCTCGA	T	0.073	150	PASS	LCOR	intron_variant	Dura AdCa
chr10	96955220	CTTGTGC	C	0.072	153	PASS	LCOR	intron_variant	Dura AdCa
chr10	99360881	A	G	0.026	294	PASS	CNNM1	synonymous_variant	Dura AdCa
chr10	102362622	CACAGG	C	0.061	96	PASS	GBF1	stop_gained&frameshift_variant	Dura AdCa
chr10	102368746	A	G	0.053	117	PASS	GBF1	missense_variant	Dura AdCa
chr10	102644855	A	G	0.134	356	PASS	TRIM8	missense_variant	Dura AdCa
chr10	102834849	TGTGATA	T	0.035	281	PASS	CYP17A1	stop_gained&frameshift_variant	Dura AdCa
chr10	110498227	A	G	0.541	258	PASS	DUSP5	missense_variant	Dura AdCa
chr10	113835241	TCCAATC	T	0.034	233	PASS	DCLRE1A	frameshift_variant	Dura AdCa
chr10	114485441	TGTCAAC	T	0.055	116	PASS	ABLIM1	intron_variant	Dura AdCa
chr10	120880780	T	A	0.383	113	PASS	WDR11	intron_variant	Dura AdCa
chr10	121517319	G	C	0.022	328	PASS	FGFR2	missense_variant&splice_region_variant	Dura AdCa
chr10	122640355	T	TCTGATC	0.012	267	PASS	DMBT1	frameshift_variant	Dura AdCa
chr10	124826680	A	G	0.024	469	PASS	ABRAXAS2	missense_variant	Dura AdCa
chr10	125852632	A	G	0.54	171	PASS	DHX32	missense_variant	Dura AdCa
chr11	614181	AATCTCT	A	0.045	131	PASS	IRF7	stop_gained&frameshift_variant	Dura AdCa
chr11	1181260	T	C	0.988	136	PASS	MUC5AC	splice_polypyrimidine_tract_variant&intron_variant	Dura AdCa
chr11	1182098	CG	C	0.98	67	PASS	MUC5AC	intron_variant	Dura AdCa
chr11	1608023	A	G	0.107	116	PASS	KRTAP5-3	synonymous_variant	Dura AdCa
chr11	2418343	A	C	0.041	135	PASS	TRPM5	missense_variant	Dura AdCa
chr11	5151980	AATATAC	A	0.063	92	PASS	OR52A1	stop_gained&frameshift_variant	Dura AdCa
chr11	5857088	GTTGTCC	G	0.08	104	PASS	OR52E8	inframe_insertion	Dura AdCa
chr11	6558649	T	C	0.047	114	PASS	DNHD1	missense_variant	Dura AdCa
chr11	7796349	G	A	0.05	52	PASS	OR5P2	synonymous_variant	Dura AdCa
chr11	8452743	G	A	0.07	57	PASS	STK33	intron_variant	Dura AdCa
chr11	8452749	T	C	0.065	61	PASS	STK33	intron_variant	Dura AdCa
chr11	18709691	G	A	0.86	60	PASS	IGSF22	splice_region_variant&splice_polypyrimidine_tract_varia	Dura AdCa
chr11	22624986	T	C	0.479	111	PASS	FANCF	synonymous_variant	Dura AdCa
chr11	32613407	T	A	0.071	81	PASS	CCDC73	intron_variant	Dura AdCa
chr11	33287089	G	C	0.034	174	PASS	HIPK3	missense_variant	Dura AdCa
chr11	34651698	GAGATGA	G	0.055	122	PASS	EHF	intron_variant	Dura AdCa
chr11	34651700	GAATTTT	G	0.055	120	PASS	EHF	intron_variant	Dura AdCa
chr11	43493913	TCCCTCC	T	0.073	101	PASS	TTC17	3_prime_utr_variant	Dura AdCa
chr11	43493917	TAAGAAT	T	0.076	94	PASS	TTC17	3_prime_utr_variant	Dura AdCa
chr11	47441634	T	C	0.509	292	PASS	RAPSN	missense_variant	Dura AdCa
chr11	47804683	TGGTGGC	T	0.066	89	PASS	NUP160	intron_variant	Dura AdCa
chr11	56159674	C	A	0.038	110	PASS	OR8K5	stop_gained	Dura AdCa
chr11	56159677	ACAGTAT	A	0.039	108	PASS	OR8K5	stop_gained&frameshift_variant	Dura AdCa
chr11	61102576	T	C	0.485	111	PASS	CD5	synonymous_variant	Dura AdCa
chr11	62527519	G	A	0.015	486	PASS	AHNAK	missense_variant	Dura AdCa
chr11	62527803	GTGAGGA	G	0.025	521	PASS	AHNAK	stop_gained&frameshift_variant	Dura AdCa
chr11	62528270	A	G	0.018	707	PASS	AHNAK	synonymous_variant	Dura AdCa
chr11	62994612	T	C	0.032	237	PASS	SLC22A8	synonymous_variant	Dura AdCa
chr11	64287893	T	C	0.49	323	PASS	GPR137	missense_variant	Dura AdCa
chr11	67452275	A	C	0.44	215	PASS	GPR152	missense_variant	Dura AdCa
chr11	73873461	C	G	0.046	99	PASS	COA4	intron_variant	Dura AdCa
chr11	76792827	G	T	0.021	229	PASS	TSKU	intron_variant	Dura AdCa
chr11	86256406	G	C	0.016	525	PASS	EED	missense_variant	Dura AdCa
chr11	89782118	C	A	0.363	159	PASS	.	non_coding_transcript_exon_variant	Dura AdCa
chr11	89870756	C	T	0.244	187	PASS	TRIM64B	synonymous_variant	Dura AdCa
chr11	94512682	G	A	0.444	71	PASS	C11orf97	intron_variant	Dura AdCa
chr11	94997923	TACAGAT	T	0.049	219	PASS	KDM4D	stop_gained&frameshift_variant	Dura AdCa
chr11	108353857	A	G	0.024	205	PASS	ATM	synonymous_variant	Dura AdCa
chr11	113931878	TG	CA	0.866	78	PASS	HTR3B	intron_variant	Dura AdCa
chr11	117918645	C	CCTGGAC	0.022	699	PASS	TMPRSS13	inframe_deletion	Dura AdCa
chr11	118104886	T	C	0.042	56	PASS	TMPRSS4	intron_variant	Dura AdCa
chr11	118502404	C	CTACCCC	0.016	337	PASS	KMT2A	frameshift_variant	Dura AdCa
chr11	122885982	TGACGAC	T	0.042	166	PASS	JHY	stop_gained&frameshift_variant	Dura AdCa
chr11	132435187	AT	TA	0.065	51	PASS	OPCML	intron_variant	Dura AdCa
chr11	132435190	C	G	0.065	50	PASS	OPCML	intron_variant	Dura AdCa
chr12	66973	T	A	0.744	225	PASS	IQSEC3	missense_variant	Dura AdCa
chr12	2682469	T	C	0.03	139	PASS	CACNA1C-AS1	intron_variant&non_coding_transcript_variant	Dura AdCa
chr12	2797289	A	G	0.067	370	PASS	.	splice_polypyrimidine_tract_variant&intron_variant&non	Dura AdCa
chr12	2874006	G	GCTCCAC	0.02	296	PASS	FOXM1	frameshift_variant	Dura AdCa
chr12	5969435	C	G	0.763	103	PASS	VWF	intron_variant	Dura AdCa
chr12	8850290	G	A	0.519	146	PASS	A2ML1	intron_variant	Dura AdCa
chr12	10126650	TTTATGA	T	0.038	245	PASS	CLEC7A	stop_gained&protein_altering_variant	Dura AdCa
chr12	10882675	T	G	0.194	280	PASS	PRH1	missense_variant	Dura AdCa
chr12	11393075	T	TGTTGCC	0.039	444	PASS	PRB2	inframe_deletion	Dura AdCa

chr12	12940880	T	TCTGAGC	0.039	182	PASS	GPRC5D-AS1	intron_variant&non_coding_transcript_variant	Dura AdCa
chr12	12940904	TTCAACC	T	0.049	134	PASS	GPRC5D-AS1	intron_variant&non_coding_transcript_variant	Dura AdCa
chr12	21852270	A	G	0.025	167	PASS	ABCC9	intron_variant	Dura AdCa
chr12	25144612	CATGGTC	C	0.039	183	PASS	DNAI7	frameshift_variant	Dura AdCa
chr12	26597178	ATTCTTG	A	0.029	144	PASS	ITPR2	intron_variant	Dura AdCa
chr12	26597184	GGCATGC	G	0.03	136	PASS	ITPR2	intron_variant	Dura AdCa
chr12	38320549	G	T	0.393	330	PASS	ALG10B	missense_variant	Dura AdCa
chr12	40414886	CTCAGAC	C	0.049	238	PASS	MUC19	stop_gained&frameshift_variant	Dura AdCa
chr12	40515401	AA	GG	0.054	105	PASS	MUC19	intron_variant	Dura AdCa
chr12	50350690	A	ATGGTGA	0.026	252	PASS	FAM186A	frameshift_variant	Dura AdCa
chr12	52489930	C	T	0.04	408	PASS	KRT6A	intron_variant	Dura AdCa
chr12	52519021	T	C	0.039	426	PASS	KRT5	missense_variant	Dura AdCa
chr12	54182416	G	A	0.841	314	PASS	SMUG1	missense_variant	Dura AdCa
chr12	54508543	T	C	0.924	97	PASS	NCKAP1L	intron_variant	Dura AdCa
chr12	62104733	AA	CC	0.825	154	PASS	TAFA2	intron_variant	Dura AdCa
chr12	64222275	T	C	0.125	192	PASS	KICS2	5_prime_utr_variant	Dura AdCa
chr12	66377232	GGGGTGC	G	0.068	152	PASS	GRIP1	stop_gained&frameshift_variant	Dura AdCa
chr12	68722088	T	G	0.037	161	PASS	NUP107	splice_polypyrimidine_tract_variant&intron_variant	Dura AdCa
chr12	68836732	C	CCTGAAA	0.015	321	PASS	MDM2	splice_donor_variant&splice_donor_5th_base_variant&c	Dura AdCa
chr12	68870437	C	G	0.052	78	PASS	CPM	intron_variant	Dura AdCa
chr12	70559632	A	T	0.054	71	PASS	PTPRB	splice_region_variant&splice_polypyrimidine_tract_varia	Dura AdCa
chr12	80256318	G	T	0.048	88	PASS	OTOGL	intron_variant	Dura AdCa
chr12	90970116	CTCTTTG	C	0.041	140	PASS	EPYC	stop_gained&inframe_insertion	Dura AdCa
chr12	101338126	G	C	0.608	159	PASS	UTP20	synonymous_variant	Dura AdCa
chr12	108940930	C	T	0.567	157	PASS	SVOP	intron_variant	Dura AdCa
chr12	110044441	A	G	0.045	87	PASS	C12orf76	intron_variant	Dura AdCa
chr12	110163053	ACCATGT	A	0.061	76	PASS	IFT81	stop_gained&frameshift_variant	Dura AdCa
chr12	111520955	G	T	0.556	189	PASS	ATXN2	missense_variant	Dura AdCa
chr12	119704490	CACATTC	C	0.038	210	PASS	CIT	intron_variant	Dura AdCa
chr12	120526477	T	G	0.204	112	PASS	COQ5	intron_variant	Dura AdCa
chr12	121417627	T	C	0.027	264	PASS	RNF34	stop_gained	Dura AdCa
chr12	122569631	A	C	0.08	87	PASS	KNTC1	intron_variant	Dura AdCa
chr12	122702193	T	TAAGGA	0.065	485	PASS	HCAR2	frameshift_variant&stop_lost	Dura AdCa
chr12	123313656	AGTTTTC	A	0.04	151	PASS	SBNO1	stop_gained&frameshift_variant	Dura AdCa
chr12	123313659	CCTGTTT	C	0.04	148	PASS	SBNO1	frameshift_variant	Dura AdCa
chr12	124314124	C	T	0.278	150	PASS	RFLNA	intron_variant	Dura AdCa
chr12	124912584	T	C	0.041	615	PASS	UBC	synonymous_variant	Dura AdCa
chr12	124913577	A	G	0.028	695	PASS	UBC	synonymous_variant	Dura AdCa
chr12	131982290	AT	CC	0.289	348	PASS	EP400	missense_variant	Dura AdCa
chr12	133010734	A	G	0.352	232	PASS	ZNF26	synonymous_variant	Dura AdCa
chr12	133058128	G	A	0.027	220	PASS	ZNF84	synonymous_variant	Dura AdCa
chr12	133058134	A	G	0.027	218	PASS	ZNF84	synonymous_variant	Dura AdCa
chr12	133100956	C	T	0.698	208	PASS	ZNF140	intron_variant	Dura AdCa
chr12	133193378	AAGGGCT	A	0.024	166	PASS	ZNF268	intron_variant	Dura AdCa
chr13	20796964	C	T	0.055	82	PASS	XPO4	missense_variant	Dura AdCa
chr13	21387632	AAGAAG	A	0.083	109	PASS	ZDHHC20	frameshift_variant&splice_region_variant	Dura AdCa
chr13	23891902	C	A	0.046	504	PASS	C1QTNF9B	missense_variant	Dura AdCa
chr13	24685340	A	ATTGGAA	0.03	164	PASS	ATP12A	splice_donor_variant&splice_donor_5th_base_variant&c	Dura AdCa
chr13	28467035	T	TGCCATT	0.038	266	PASS	FLT1	frameshift_variant	Dura AdCa
chr13	32336895	GTCTGTT	G	0.028	276	PASS	BRCA2	stop_gained&frameshift_variant	Dura AdCa
chr13	32337144	AATTTTC	A	0.074	320	PASS	BRCA2	stop_gained&frameshift_variant	Dura AdCa
chr13	32337995	GATAACA	G	0.104	304	PASS	BRCA2	frameshift_variant	Dura AdCa
chr13	32340733	CCCCATC	C	0.046	238	PASS	BRCA2	stop_gained&frameshift_variant	Dura AdCa
chr13	32342571	TTCAGG	T	0.059	98	PASS	BRCA2	intron_variant	Dura AdCa
chr13	34541522	A	G	0.322	97	PASS	LINC00457	intron_variant&non_coding_transcript_variant	Dura AdCa
chr13	42301327	ATTTGTC	A	0.067	115	PASS	AKAP11	stop_gained&frameshift_variant	Dura AdCa
chr13	42302030	AGTATAC	A	0.049	115	PASS	AKAP11	protein_altering_variant	Dura AdCa
chr13	42302032	GATAATT	G	0.049	116	PASS	AKAP11	stop_gained&protein_altering_variant	Dura AdCa
chr13	45398102	G	A	0.049	55	PASS	TPT1-AS1	intron_variant&non_coding_transcript_variant	Dura AdCa
chr13	48364778	T	G	0.057	76	PASS	RB1	intron_variant	Dura AdCa
chr13	51245424	T	C	0.571	62	PASS	FAM124A	intron_variant	Dura AdCa
chr13	79539795	A	T	0.056	82	PASS	NDFIP2	intron_variant	Dura AdCa
chr13	102735811	A	C	0.085	62	PASS	CCDC168	missense_variant	Dura AdCa
chr13	114323914	C	A	0.13	125	PASS	CHAMP1	synonymous_variant	Dura AdCa
chr14	19097884	T	G	0.169	138	PASS	DUXAP9	intron_variant&non_coding_transcript_variant	Dura AdCa
chr14	21525168	T	C	0.024	213	PASS	SALL2	missense_variant	Dura AdCa
chr14	23417278	T	G	0.453	296	PASS	MYH7	stop_gained	Dura AdCa
chr14	23424064	G	A	0.497	260	PASS	MYH7	missense_variant	Dura AdCa
chr14	23955080	C	T	0.063	764	PASS	DHRS4	synonymous_variant	Dura AdCa
chr14	23967451	C	A	0.068	248	PASS	DHRS4	intron_variant	Dura AdCa
chr14	24155568	AAAGCTC	A	0.024	343	PASS	RNF31	intron_variant	Dura AdCa
chr14	31129426	ATCTGGC	A	0.028	262	PASS	HECTD1	stop_gained&frameshift_variant	Dura AdCa
chr14	45032186	C	G	0.39	117	PASS	KLHL28	intron_variant	Dura AdCa
chr14	45164495	A	G	0.498	368	PASS	FANCM	missense_variant	Dura AdCa
chr14	45175747	A	C	0.013	487	PASS	FANCM	missense_variant	Dura AdCa
chr14	50613419	T	G	0.558	117	PASS	ATL1	intron_variant	Dura AdCa
chr14	70246727	CA	TG	0.044	203	PASS	ADAM21P1	non_coding_transcript_exon_variant	Dura AdCa
chr14	77416593	C	T	0.104	83	PASS	NOXRED1	intron_variant	Dura AdCa
chr14	77416654	T	TC	0.108	124	PASS	NOXRED1	intron_variant	Dura AdCa
chr14	77416691	C	A	0.063	128	PASS	NOXRED1	intron_variant	Dura AdCa
chr14	90304150	A	G	0.535	252	PASS	NRDE2	missense_variant	Dura AdCa
chr14	90318141	AATCGGC	A	0.077	81	PASS	NRDE2	intron_variant	Dura AdCa
chr14	91339157	G	A	0.029	173	PASS	CCDC88C	intron_variant	Dura AdCa
chr14	95124397	CTGCTCC	C	0.034	638	PASS	DICER1	inframe_insertion	Dura AdCa
chr14	100567885	T	C	0.461	131	PASS	BEGAIN	intron_variant	Dura AdCa
chr14	104741279	T	C	0.499	162	PASS	ADSS1	intron_variant	Dura AdCa

chr14	104932945	C	A	0.42	281	PASS	PLD4	missense_variant	Dura AdCa
chr14	105241244	C	G	0.532	65	PASS	BRF1	intron_variant	Dura AdCa
chr14	105272810	A	G	0.023	414	PASS	BRF1	missense_variant	Dura AdCa
chr14	105625935	C	G	0.283	289	PASS	IGHG4	synonymous_variant	Dura AdCa
chr14	105625998	C	G	0.294	240	PASS	IGHG4	synonymous_variant	Dura AdCa
chr14	105707471	A	G	0.25	557	PASS	IGHA1	synonymous_variant	Dura AdCa
chr14	105767772	G	A	0.094	112	PASS	IGHG3	intron_variant	Dura AdCa
chr14	105767851	G	A	0.062	93	PASS	IGHG3	synonymous_variant	Dura AdCa
chr14	105863968	G	A	0.461	98	PASS	MIR4537	upstream_gene_variant	Dura AdCa
chr14	106012330	C	G	0.425	307	PASS	IGHV4-4	synonymous_variant	Dura AdCa
chr14	106062072	G	T	0.218	307	PASS	IGHV3-7	downstream_gene_variant	Dura AdCa
chr14	106062074	G	T	0.217	307	PASS	IGHV3-7	downstream_gene_variant	Dura AdCa
chr14	106062169	A	G	0.377	510	PASS	IGHV3-7	synonymous_variant	Dura AdCa
chr14	106335083	C	T	0.429	1875	PASS	IGHV3-30	missense_variant	Dura AdCa
chr14	106349771	A	T	0.422	446	PASS	IGHV4-31	5_prime_utr_variant	Dura AdCa
chr14	106627496	GT	CC	0.017	650	PASS	IGHV4-59	missense_variant	Dura AdCa
chr14	106675068	C	A	0.018	318	PASS	IGHV3-66	synonymous_variant	Dura AdCa
chr15	31002354	A	T	0.036	168	PASS	TRPM1	missense_variant	Dura AdCa
chr15	34154595	T	C	0.87	72	PASS	KATNBL1	intron_variant	Dura AdCa
chr15	34254487	T	C	0.104	110	PASS	SLC12A6	missense_variant	Dura AdCa
chr15	41278897	ATTAACC	A	0.042	178	PASS	CHP1	intron_variant	Dura AdCa
chr15	44828439	G	A	0.294	511	PASS	SORD2P	intron_variant&non_coding_transcript_variant	Dura AdCa
chr15	48121861	TCATCAC	T	0.079	69	PASS	SLC24A5	stop_gained&inframe_insertion	Dura AdCa
chr15	48767150	CCCTATT	C	0.076	119	PASS	CEP152	stop_gained&frameshift_variant	Dura AdCa
chr15	61884130	T	C	0.905	58	PASS	VPS13C	missense_variant&splice_region_variant	Dura AdCa
chr15	65395129	A	G	0.771	121	PASS	IGDCC4	missense_variant	Dura AdCa
chr15	67255028	C	G	0.169	123	PASS	IQCH	intron_variant	Dura AdCa
chr15	69014683	CC	AG	0.864	88	PASS	NOX5	upstream_gene_variant	Dura AdCa
chr15	70672087	GGAACA.	G	0.102	54	PASS	UACA	intron_variant	Dura AdCa
chr15	72290086	ATGCAGT	A	0.025	309	PASS	CELF6	intron_variant	Dura AdCa
chr15	72407248	C	G	0.15	61	PASS	TMEM202	intron_variant	Dura AdCa
chr15	74619971	T	G	0.707	198	PASS	CLK3	intron_variant	Dura AdCa
chr15	79025348	A	G	0.727	146	PASS	RASGRF1	missense_variant	Dura AdCa
chr15	82342744	GT	G	0.341	102	PASS	GOLGA6L10	3_prime_utr_variant	Dura AdCa
chr15	82343155	A	C	0.637	211	PASS	GOLGA6L10	missense_variant	Dura AdCa
chr15	82345980	GT	CC	0.318	110	PASS	GOLGA6L10	intron_variant	Dura AdCa
chr15	82347489	C	G	0.03	201	PASS	GOLGA6L10	intron_variant	Dura AdCa
chr15	82434465	T	C	0.133	82	PASS	GOLGA6L9	stop_gained	Dura AdCa
chr15	82524937	A	G	0.075	68	PASS	GOLGA6L17P	intron_variant&non_coding_transcript_variant	Dura AdCa
chr15	89683799	G	C	0.054	125	PASS	PEX11A	missense_variant	Dura AdCa
chr15	90913804	CATAAGT	C	0.05	132	PASS	MAN2A2	intron_variant	Dura AdCa
chr15	90913805	CCTTGCA	C	0.05	131	PASS	MAN2A2	intron_variant	Dura AdCa
chr15	94402576	ACCACAT	A	0.086	71	PASS	MCTP2	intron_variant	Dura AdCa
chr15	101712011	AACACTT	A	0.071	89	PASS	TARS3	splice_polypyrimidine_tract_variant&intron_variant	Dura AdCa
chr15	101966111	G	C	0.167	140	PASS	WASH3P	non_coding_transcript_exon_variant	Dura AdCa
chr16	281826	C	G	0.449	185	PASS	ARHGDIG	missense_variant	Dura AdCa
chr16	2235168	A	G	0.023	239	PASS	E4F1	intron_variant	Dura AdCa
chr16	2324392	T	C	0.542	131	PASS	ABCA3	intron_variant	Dura AdCa
chr16	4783733	T	C	0.043	126	PASS	SEPTIN12	synonymous_variant	Dura AdCa
chr16	10482212	TAAAATC	T	0.082	141	PASS	ATF7IP2	stop_gained&frameshift_variant	Dura AdCa
chr16	11999364	T	C	0.473	210	PASS	SNX29	splice_donor_region_variant&intron_variant	Dura AdCa
chr16	14918846	C	T	0.102	59	PASS	PKD1P3	non_coding_transcript_exon_variant	Dura AdCa
chr16	14951901	A	AGTGTCT	0.022	577	PASS	NPIPA1	frameshift_variant	Dura AdCa
chr16	14951909	T	C	0.304	420	PASS	NPIPA1	missense_variant	Dura AdCa
chr16	14975345	C	T	0.075	676	PASS	PDXDC1	intron_variant	Dura AdCa
chr16	15129957	T	C	0.23	151	PASS	PDXDC1	intron_variant	Dura AdCa
chr16	15363750	G	GGAGCTC	0.021	606	PASS	NPIPA5	inframe_deletion	Dura AdCa
chr16	18347905	G	A	0.174	173	PASS	PKD1P4	non_coding_transcript_exon_variant	Dura AdCa
chr16	21201719	T	C	0.036	154	PASS	ZP2	synonymous_variant	Dura AdCa
chr16	23629560	A	C	0.044	156	PASS	PALB2	intron_variant	Dura AdCa
chr16	25140083	C	G	0.054	71	PASS	LCMT1	intron_variant	Dura AdCa
chr16	27481256	A	G	0.041	142	PASS	GTF3C1	intron_variant	Dura AdCa
chr16	29364735	A	T	0.03	343	PASS	.	non_coding_transcript_exon_variant	Dura AdCa
chr16	29383175	C	T	0.029	246	PASS	NPIPB11	missense_variant	Dura AdCa
chr16	31793797	T	A	0.345	212	PASS	KRBOX5	non_coding_transcript_exon_variant	Dura AdCa
chr16	50668036	T	C	0.435	352	PASS	.	non_coding_transcript_exon_variant	Dura AdCa
chr16	55810697	T	G	0.129	90	PASS	CES1	intron_variant	Dura AdCa
chr16	55812849	T	C	0.038	196	PASS	CES1	intron_variant	Dura AdCa
chr16	56405102	G	A	0.089	57	PASS	AMFR	intron_variant	Dura AdCa
chr16	56509915	G	A	0.157	78	PASS	BBS2	intron_variant	Dura AdCa
chr16	57759844	T	C	0.086	51	PASS	KIFC3	splice_region_variant&splice_polypyrimidine_tract_varia	Dura AdCa
chr16	58582782	T	C	0.042	112	PASS	CNOT1	intron_variant	Dura AdCa
chr16	66388463	GTCTTTG	G	0.077	86	PASS	CDH5	intron_variant	Dura AdCa
chr16	66724706	G	T	0.038	77	PASS	DYNC1LI2	intron_variant	Dura AdCa
chr16	84074744	A	G	0.066	67	PASS	MBTPS1	splice_region_variant&splice_polypyrimidine_tract_varia	Dura AdCa
chr16	85082633	A	G	0.07	66	PASS	KIAA0513	intron_variant	Dura AdCa
chr16	87603463	ACTTAAT	A	0.051	207	PASS	JPH3	stop_gained&frameshift_variant	Dura AdCa
chr16	88678500	T	C	0.041	192	PASS	SNAI3	missense_variant	Dura AdCa
chr16	88727505	C	G	0.027	90	PASS	PIEZO1	intron_variant	Dura AdCa
chr16	88951011	G	GCCTGGA	0.746	452	PASS	CBFA2T3	intron_variant	Dura AdCa
chr17	138824	A	C	0.977	180	PASS	SCGB1C2	intron_variant	Dura AdCa
chr17	2998283	A	G	0.054	305	PASS	RAP1GAP2	synonymous_variant	Dura AdCa
chr17	4282884	CA	C	0.043	182	PASS	UBE2G1	frameshift_variant	Dura AdCa
chr17	4282887	TATGTAA	T	0.043	178	PASS	UBE2G1	stop_gained&frameshift_variant	Dura AdCa
chr17	7675085	T	C	0.835	998	PASS	TP53	missense_variant	Dura AdCa
chr17	10448799	ATTCTGT	A	0.041	151	PASS	MYH4	splice_polypyrimidine_tract_variant&intron_variant	Dura AdCa

chr17	10699268	CCTTCTG	C	0.067	84	PASS	TMEM220	splice_acceptor_variant	Dura AdCa
chr17	13496831	AGC	A	0.024	687	PASS	HS3ST3A1	splice_polypyrimidine_tract_variant&intron_variant	Dura AdCa
chr17	18091852	TG	CA	0.986	95	PASS	DRG2	intron_variant	Dura AdCa
chr17	21043507	G	A	0.068	384	PASS	USP22	upstream_gene_variant	Dura AdCa
chr17	28048003	A	G	0.557	127	PASS	NLK	intron_variant	Dura AdCa
chr17	28369358	G	C	0.523	291	PASS	VTN	missense_variant	Dura AdCa
chr17	29495569	C	G	0.032	137	PASS	TAOK1	missense_variant	Dura AdCa
chr17	29632116	T	TTG	0.307	239	PASS	SSH2	frameshift_variant	Dura AdCa
chr17	32369294	T	G	0.047	110	PASS	ZNF207	splice_acceptor_variant	Dura AdCa
chr17	35363203	CAAATTC	C	0.032	262	PASS	SLFN11	frameshift_variant	Dura AdCa
chr17	35363205	AGAAGCT	A	0.032	267	PASS	SLFN11	stop_gained	Dura AdCa
chr17	35741246	A	G	0.473	314	PASS	RASL10B	missense_variant	Dura AdCa
chr17	36195196	A	G	0.713	268	PASS	CCL3L3	3_prime_utr_variant	Dura AdCa
chr17	36196527	G	A	0.984	170	PASS	CCL3L3	intron_variant	Dura AdCa
chr17	36211278	T	G	0.988	125	PASS	CCL4L2	intron_variant	Dura AdCa
chr17	36211979	C	T	0.993	192	PASS	CCL4L2	intron_variant	Dura AdCa
chr17	36212342	GA	CG	0.993	214	PASS	CCL4L2	missense_variant	Dura AdCa
chr17	36254584	T	G	0.155	165	PASS	TBC1D3I	intron_variant	Dura AdCa
chr17	36307282	A	T	0.145	83	PASS	.	splice_polypyrimidine_tract_variant&intron_variant&nor	Dura AdCa
chr17	36452733	A	G	0.451	108	PASS	TBC1D3JP	non_coding_transcript_exon_variant	Dura AdCa
chr17	36953143	A	G	0.09	183	PASS	AATF	missense_variant	Dura AdCa
chr17	37635608	T	A	0.431	208	PASS	DDX52	intron_variant	Dura AdCa
chr17	38119271	T	C	0.867	53	PASS	NPEPPSP1	intron_variant&non_coding_transcript_variant	Dura AdCa
chr17	40818851	AGCTGCC	A	0.154	244	PASS	KRT10	inframe_insertion	Dura AdCa
chr17	41160573	A	C	0.5	465	PASS	KRTAP4-4	missense_variant	Dura AdCa
chr17	41275687	T	C	0.046	157	PASS	KRTAP9-10P	upstream_gene_variant	Dura AdCa
chr17	41275814	T	G	0.074	353	PASS	KRTAP9-7	synonymous_variant	Dura AdCa
chr17	41422764	GC	AG	0.236	71	PASS	KRT37	intron_variant	Dura AdCa
chr17	41438269	T	G	0.515	372	PASS	KRT38	missense_variant	Dura AdCa
chr17	41477545	CCACTTT	C	0.025	443	PASS	KRT35	stop_gained&frameshift_variant	Dura AdCa
chr17	41567235	A	T	0.338	100	PASS	KRT9	splice_region_variant&3_prime_utr_variant	Dura AdCa
chr17	42669593	C	A	0.041	159	PASS	PLEKHH3	missense_variant	Dura AdCa
chr17	42874175	G	A	0.25	170	PASS	AOC4P	non_coding_transcript_exon_variant	Dura AdCa
chr17	43060548	A	G	0.062	71	PASS	BRCA1	intron_variant	Dura AdCa
chr17	44212035	CCTGGTT	C	0.047	135	PASS	UBTF	intron_variant	Dura AdCa
chr17	45547982	T	C	0.101	202	PASS	.	intron_variant&non_coding_transcript_variant	Dura AdCa
chr17	45547991	G	C	0.111	207	PASS	.	intron_variant&non_coding_transcript_variant	Dura AdCa
chr17	46259843	T	C	0.111	708	PASS	.	non_coding_transcript_exon_variant	Dura AdCa
chr17	46528798	A	G	0.109	171	PASS	ARL17A	intron_variant	Dura AdCa
chr17	46548670	A	G	0.129	392	PASS	LRRC37A2	synonymous_variant	Dura AdCa
chr17	46553527	G	A	0.139	168	PASS	ARL17A	3_prime_utr_variant	Dura AdCa
chr17	47291081	GTGGATC	G	0.048	159	PASS	ITGB3	inframe_insertion	Dura AdCa
chr17	47291084	AATGACT	A	0.049	155	PASS	ITGB3	stop_gained&frameshift_variant	Dura AdCa
chr17	47923156	T	C	0.27	342	PASS	SP2	synonymous_variant	Dura AdCa
chr17	50186515	ATGTCAT	A	0.046	304	PASS	COL1A1	splice_polypyrimidine_tract_variant&intron_variant	Dura AdCa
chr17	50186517	GGGTTCT	G	0.047	302	PASS	COL1A1	splice_polypyrimidine_tract_variant&intron_variant	Dura AdCa
chr17	50863591	C	T	0.05	300	PASS	TOB1	missense_variant	Dura AdCa
chr17	58617559	C	G	0.306	108	PASS	TEX14	missense_variant	Dura AdCa
chr17	59275089	GT	AC	0.036	66	PASS	GDPD1	3_prime_utr_variant	Dura AdCa
chr17	59275100	C	T	0.066	74	PASS	GDPD1	3_prime_utr_variant	Dura AdCa
chr17	64822018	C	G	0.103	321	PASS	PLEKHM1P1	non_coding_transcript_exon_variant	Dura AdCa
chr17	67138031	G	A	0.345	258	PASS	HELZ	synonymous_variant	Dura AdCa
chr17	69304746	T	C	0.307	51	PASS	ABCA5	missense_variant	Dura AdCa
chr17	75937609	G	C	0.446	222	PASS	FBF1	splice_polypyrimidine_tract_variant&intron_variant	Dura AdCa
chr17	79784185	C	G	0.608	295	PASS	CBX2	missense_variant	Dura AdCa
chr17	80643748	G	C	0.012	367	PASS	RPTOR	missense_variant	Dura AdCa
chr17	82374399	A	T	0.49	144	PASS	UTS2R	synonymous_variant	Dura AdCa
chr18	712568	A	G	0.506	250	PASS	ENOSF1	missense_variant	Dura AdCa
chr18	2554916	A	G	0.536	162	PASS	METTL4	synonymous_variant	Dura AdCa
chr18	2934338	G	A	0.47	202	PASS	LPIN2	intron_variant	Dura AdCa
chr18	6995488	T	A	0.5	86	PASS	LAMA1	intron_variant	Dura AdCa
chr18	7013998	A	G	0.429	415	PASS	LAMA1	synonymous_variant	Dura AdCa
chr18	7015977	T	C	0.465	77	PASS	LAMA1	intron_variant	Dura AdCa
chr18	8706188	T	C	0.471	103	PASS	MTCL1	synonymous_variant	Dura AdCa
chr18	9887023	T	G	0.025	386	PASS	TXNDC2	missense_variant	Dura AdCa
chr18	12955468	C	T	0.052	210	PASS	SEH1L	synonymous_variant	Dura AdCa
chr18	24358591	G	T	0.052	70	PASS	OSBPL1A	intron_variant	Dura AdCa
chr18	57573406	C	T	0.086	50	PASS	FECH	intron_variant	Dura AdCa
chr18	57602938	T	G	0.061	79	PASS	NARS1	synonymous_variant	Dura AdCa
chr18	58044732	T	C	0.055	111	PASS	NEDD4L	intron_variant	Dura AdCa
chr18	58045108	A	G	0.115	493	PASS	NEDD4L	intron_variant	Dura AdCa
chr18	58671560	A	G	0.069	152	PASS	MALT1	5_prime_utr_variant	Dura AdCa
chr18	63331755	A	AC	0.089	199	PASS	KDSR	3_prime_utr_variant	Dura AdCa
chr18	63331757	A	AG	0.089	199	PASS	KDSR	3_prime_utr_variant	Dura AdCa
chr18	63801022	A	G	0.062	70	PASS	SERPINB7	intron_variant	Dura AdCa
chr19	1622424	A	G	0.754	188	PASS	TCF3	splice_polypyrimidine_tract_variant&intron_variant	Dura AdCa
chr19	2917465	TACACAC	T	0.035	200	PASS	ZNF57	frameshift_variant	Dura AdCa
chr19	7550967	G	GCC	0.039	116	PASS	PNPLA6	intron_variant	Dura AdCa
chr19	7550972	A	AATCATC	0.034	146	PASS	PNPLA6	splice_acceptor_variant&coding_sequence_variant&intro	Dura AdCa
chr19	8086248	G	C	0.416	283	PASS	FBN3	missense_variant	Dura AdCa
chr19	8321398	TA	T	0.469	251	PASS	RPS28	upstream_gene_variant	Dura AdCa
chr19	8323285	A	G	0.514	89	PASS	RPS28	3_prime_utr_variant	Dura AdCa
chr19	8510850	C	CCCGCGC	0.586	187	PASS	ZNF414	inframe_deletion&splice_region_variant	Dura AdCa
chr19	8605262	T	C	0.513	297	PASS	ADAMTS10	missense_variant	Dura AdCa
chr19	8861010	C	G	0.493	99	PASS	MUC16	missense_variant	Dura AdCa
chr19	8891843	C	G	0.272	384	PASS	MUC16	missense_variant	Dura AdCa

chr19	8902191	T	G	0.023	314	PASS	MUC16	synonymous_variant	Dura AdCa
chr19	8938611	TTCTGCT	T	0.034	178	PASS	MUC16	stop_gained&frameshift_variant	Dura AdCa
chr19	8979177	TGCAGA	T	0.031	338	PASS	MUC16	frameshift_variant	Dura AdCa
chr19	8979181	GGTGTG(	G	0.031	338	PASS	MUC16	stop_gained	Dura AdCa
chr19	12957208	A	G	0.058	66	PASS	GADD45GIP1	missense_variant	Dura AdCa
chr19	13814607	A	G	0.028	312	PASS	ZSWIM4	missense_variant	Dura AdCa
chr19	18938522	A	G	0.493	263	PASS	HOMER3	intron_variant	Dura AdCa
chr19	19245400	T	C	0.018	434	PASS	NCAN	missense_variant	Dura AdCa
chr19	19505356	G	C	0.511	219	PASS	GATAD2A	missense_variant	Dura AdCa
chr19	19650028	T	A	0.049	111	PASS	ATP13A1	intron_variant	Dura AdCa
chr19	23935301	T	G	0.083	158	PASS	.	intron_variant&non_coding_transcript_variant	Dura AdCa
chr19	23935307	TTGAGG/	T	0.084	154	PASS	.	intron_variant&non_coding_transcript_variant	Dura AdCa
chr19	30557233	AAACGT/	A	0.049	150	PASS	ZNF536	3_prime_utr_variant	Dura AdCa
chr19	36627382	TCAGTG1	T	0.053	134	PASS	ZNF382	stop_gained&inframe_insertion	Dura AdCa
chr19	38950233	T	C	0.474	223	PASS	FBXO17	synonymous_variant	Dura AdCa
chr19	40370082	A	C	0.488	361	PASS	PLD3	intron_variant	Dura AdCa
chr19	40396791	G	GTACCTC	0.025	620	PASS	PRX	inframe_deletion	Dura AdCa
chr19	40763048	T	C	0.038	115	PASS	SNRPA	missense_variant	Dura AdCa
chr19	40778492	CA	TG	0.522	153	PASS	RAB4B	intron_variant	Dura AdCa
chr19	40881553	A	G	0.039	271	PASS	CYP2A7	intron_variant	Dura AdCa
chr19	41125726	T	C	0.067	218	PASS	CYP2F1	intron_variant	Dura AdCa
chr19	41125759	G	T	0.083	185	PASS	CYP2F1	intron_variant	Dura AdCa
chr19	41579543	T	G	0.347	208	PASS	CEACAM21	missense_variant	Dura AdCa
chr19	43016033	T	A	0.018	248	PASS	PSG11-AS1	intron_variant&non_coding_transcript_variant	Dura AdCa
chr19	44496520	C	A	0.517	88	PASS	ZNF180	intron_variant	Dura AdCa
chr19	45421364	T	G	0.031	390	PASS	ERCC1	missense_variant	Dura AdCa
chr19	46519851	AAAAAT	A	0.037	116	PASS	PPP5D1P	intron_variant&non_coding_transcript_variant	Dura AdCa
chr19	48510162	A	G	0.41	220	PASS	LMTK3	synonymous_variant	Dura AdCa
chr19	49101346	T	C	0.453	316	PASS	SNRNP70	intron_variant	Dura AdCa
chr19	49860193	T	C	0.02	233	PASS	PTOV1	intron_variant	Dura AdCa
chr19	51587738	C	CACAGG/	0.018	273	PASS	ZNF175	inframe_deletion	Dura AdCa
chr19	52414851	T	G	0.077	54	PASS	ZNF528	intron_variant	Dura AdCa
chr19	53116425	GCAGTG(	G	0.062	177	PASS	ZNF415	frameshift_variant	Dura AdCa
chr19	53116427	AAGTTC/	A	0.063	172	PASS	ZNF415	stop_gained&frameshift_variant	Dura AdCa
chr19	53183041	A	C	0.354	72	PASS	ZNF665	intron_variant	Dura AdCa
chr19	54220007	T	C	0.071	117	PASS	LILRB3	intron_variant	Dura AdCa
chr19	54220739	A	C	0.462	363	PASS	LILRB3	synonymous_variant	Dura AdCa
chr19	54222756	C	T	0.659	606	PASS	LILRB3	missense_variant	Dura AdCa
chr19	54222987	T	C	0.318	526	PASS	LILRB3	5_prime_utr_variant	Dura AdCa
chr19	54242117	C	T	0.409	460	PASS	LILRA6	missense_variant	Dura AdCa
chr19	54280655	G	A	0.039	160	PASS	LILRB2	intron_variant	Dura AdCa
chr19	54280660	T	C	0.041	154	PASS	LILRB2	intron_variant	Dura AdCa
chr19	54463591	A	T	0.979	74	PASS	LENG9	5_prime_utr_variant	Dura AdCa
chr19	54667677	G	A	0.146	157	PASS	LILRB4	missense_variant	Dura AdCa
chr19	54727771	T	C	0.36	269	PASS	KIR3DL3	synonymous_variant	Dura AdCa
chr19	54755120	T	G	0.289	396	PASS	KIR2DP1	intron_variant&non_coding_transcript_variant	Dura AdCa
chr19	54773534	A	C	0.282	470	PASS	KIR2DL1	missense_variant	Dura AdCa
chr19	54773593	T	G	0.26	461	PASS	KIR2DL1	missense_variant	Dura AdCa
chr19	54773692	C	A	0.194	229	PASS	KIR2DL1	intron_variant	Dura AdCa
chr19	54773699	G	A	0.203	215	PASS	KIR2DL1	intron_variant	Dura AdCa
chr19	54788295	A	G	0.066	299	PASS	KIR3DP1	non_coding_transcript_exon_variant	Dura AdCa
chr19	54855560	G	A	0.109	183	PASS	KIR3DL2	intron_variant	Dura AdCa
chr19	54855661	A	C	0.03	347	PASS	KIR3DL2	missense_variant	Dura AdCa
chr19	57377872	G	GTGAAT(	0.012	264	PASS	ZNF547	inframe_deletion	Dura AdCa
chr19	57491772	TG	CA	0.096	73	PASS	ZNF419	intron_variant	Dura AdCa
chr19	57573086	A	C	0.032	190	PASS	ZNF416	missense_variant	Dura AdCa
chr19	57676432	T	G	0.071	142	PASS	ZSCAN4	missense_variant	Dura AdCa
chr19	58261877	A	T	0.024	180	PASS	ZNF544	missense_variant	Dura AdCa
chr20	3584835	TAACAA(	T	0.034	136	PASS	ATRN	stop_gained&frameshift_variant	Dura AdCa
chr20	7906324	C	T	0.058	92	PASS	HAO1	missense_variant	Dura AdCa
chr20	7906327	ATTTTAT	A	0.059	92	PASS	HAO1	frameshift_variant&splice_region_variant	Dura AdCa
chr20	16748437	TTCTCAC	T	0.05	206	PASS	OTOR	stop_gained&frameshift_variant	Dura AdCa
chr20	21162004	A	C	0.031	135	PASS	KIZ	missense_variant	Dura AdCa
chr20	23389900	G	A	0.051	84	PASS	NAPB	intron_variant	Dura AdCa
chr20	24665463	A	G	0.467	288	PASS	SYNDIG1	missense_variant	Dura AdCa
chr20	25339350	A	G	0.048	150	PASS	ABHD12	stop_gained&splice_region_variant	Dura AdCa
chr20	32146902	GCCAAT(	G	0.046	117	PASS	TM9SF4	intron_variant	Dura AdCa
chr20	44966698	A	G	0.024	166	PASS	STK4	intron_variant	Dura AdCa
chr20	46022943	GGGA	G	0.889	79	PASS	SLC12A5	intron_variant	Dura AdCa
chr20	54476047	A	G	0.376	330	PASS	DOK5	intron_variant	Dura AdCa
chr20	56637826	A	G	0.427	422	PASS	TFAP2C	missense_variant	Dura AdCa
chr20	58467261	A	G	0.047	157	PASS	APCDD1L	missense_variant	Dura AdCa
chr20	62137444	CTGGTT(	C	0.037	262	PASS	PSMA7	intron_variant	Dura AdCa
chr21	5033666	A	G	0.65	133	PASS	.	intergenic_variant	Dura AdCa
chr21	5033966	C	G	0.419	121	PASS	.	intergenic_variant	Dura AdCa
chr21	13610379	G	GACTCC1	0.028	212	PASS	POTED	inframe_deletion	Dura AdCa
chr21	26954827	C	G	0.016	292	PASS	ADAMTS5	missense_variant	Dura AdCa
chr21	28931146	C	T	0.5	128	PASS	LTN1	intron_variant	Dura AdCa
chr21	32653413	A	C	0.063	63	PASS	SYNJ1	intron_variant	Dura AdCa
chr21	35898262	C	T	0.081	71	PASS	RUNX1	intron_variant	Dura AdCa
chr21	45939993	A	G	0.383	95	PASS	PCBP3	intron_variant	Dura AdCa
chr21	46416365	A	G	0.019	375	PASS	PCNT	synonymous_variant	Dura AdCa
chr22	17103599	A	AG	0.175	79	PASS	IL17RA	intron_variant	Dura AdCa
chr22	17549225	T	C	0.128	266	PASS	CECR2	missense_variant	Dura AdCa
chr22	18079905	C	G	0.03	100	PASS	PEX26	missense_variant	Dura AdCa
chr22	20148813	T	G	0.059	73	PASS	CCDC188	splice_polypyrimidine_tract_variant&intron_variant	Dura AdCa

chr22	20390768	CTAATTT	C	0.063	89	PASS	ZNF74	upstream_gene_variant	Dura AdCa
chr22	29292638	GTGGGTG	G	0.049	262	PASS	EWSR1	intron_variant	Dura AdCa
chr22	30285838	A	G	0.057	127	PASS	CASTOR1	synonymous_variant	Dura AdCa
chr22	30337901	C	T	0.054	171	PASS	SF3A1	splice_region_variant&splice_polypyrimidine_tract_varia	Dura AdCa
chr22	31095586	A	G	0.691	176	PASS	SMTN	missense_variant	Dura AdCa
chr22	31125822	A	T	0.051	85	PASS	INPP5J	stop_gained	Dura AdCa
chr22	31792896	C	G	0.077	69	PASS	DEPDC5	intron_variant	Dura AdCa
chr22	37011274	A	AAGTCCA	0.024	191	PASS	TST	frameshift_variant	Dura AdCa
chr22	38080830	T	C	0.036	164	PASS	SLC16A8	intron_variant	Dura AdCa
chr22	39661119	A	T	0.054	52	PASS	CACNA1I	missense_variant	Dura AdCa
chr22	40882015	CCATAGA	C	0.036	167	PASS	XPNPEP3	stop_gained&frameshift_variant	Dura AdCa
chr22	41729794	A	C	0.077	57	PASS	MEI1	intron_variant	Dura AdCa
chr22	42126611	G	C	0.29	253	PASS	CYP2D6	missense_variant	Dura AdCa
chr22	42128812	GA	AG	0.054	258	PASS	CYP2D6	missense_variant	Dura AdCa
chr22	42128818	TC	T	0.053	261	PASS	CYP2D6	frameshift_variant	Dura AdCa
chr22	42128980	T	C	0.068	339	PASS	NDUFA6-DT	intron_variant&non_coding_transcript_variant	Dura AdCa
chr22	42129008	G	T	0.038	268	PASS	NDUFA6-DT	intron_variant&non_coding_transcript_variant	Dura AdCa
chr22	42129130	G	C	0.187	296	PASS	CYP2D6	synonymous_variant	Dura AdCa
chr22	42129950	C	A	0.24	52	PASS	CYP2D6	intron_variant	Dura AdCa
chr22	42143622	C	A	0.209	56	PASS	CYP2D7	intron_variant	Dura AdCa
chr22	44132895	G	A	0.035	84	PASS	PARVB	splice_region_variant&synonymous_variant	Dura AdCa
chr22	46335918	A	C	0.081	66	PASS	TRMU	intron_variant	Dura AdCa
chrX	315221	C	T	0.919	252	PASS	GTPBP6	splice_region_variant&intron_variant	Dura AdCa
chrX	1191074	C	T	0.872	181	PASS	CRLF2	synonymous_variant	Dura AdCa
chrX	1191089	A	G	0.885	174	PASS	CRLF2	synonymous_variant	Dura AdCa
chrX	2859990	A	G	0.993	220	PASS	GYG2	synonymous_variant	Dura AdCa
chrX	9711568	G	C	0.029	89	PASS	TBL1X	intron_variant	Dura AdCa
chrX	16693206	GCAGTGG	G	0.051	166	PASS	CTPS2	stop_gained&frameshift_variant	Dura AdCa
chrX	49078148	A	G	0.066	62	PASS	WDR45	intron_variant	Dura AdCa
chrX	49162895	C	G	0.989	138	PASS	MAGIX	missense_variant	Dura AdCa
chrX	50192728	TCTGCAA	T	0.041	148	PASS	AKAP4	frameshift_variant	Dura AdCa
chrX	50368996	GGTTTCA	G	0.065	93	PASS	DGKK	stop_gained&inframe_insertion	Dura AdCa
chrX	53565023	A	G	0.986	91	PASS	HUWE1	intron_variant	Dura AdCa
chrX	65497250	T	C	0.053	118	PASS	ZC3H12B	missense_variant	Dura AdCa
chrX	73453605	G	T	0.92	99	PASS	CDX4	stop_gained	Dura AdCa
chrX	74591813	A	G	0.155	55	PASS	RLIM	missense_variant	Dura AdCa
chrX	74593081	A	G	0.973	51	PASS	RLIM	intron_variant	Dura AdCa
chrX	77998615	AAAACAT	A	0.068	91	PASS	ATP7A	stop_gained&frameshift_variant	Dura AdCa
chrX	101656080	C	T	0.986	99	PASS	ARMCX2	synonymous_variant	Dura AdCa
chrX	103500463	T	G	0.992	137	PASS	RAB40A	missense_variant	Dura AdCa
chrX	103825482	CATGAAG	C	0.06	172	PASS	RAB9B	stop_gained&frameshift_variant	Dura AdCa
chrX	105905385	T	C	0.875	57	PASS	NRK	intron_variant	Dura AdCa
chrX	106205772	AACAAGA	A	0.068	80	PASS	PWWP3B	frameshift_variant	Dura AdCa
chrX	106205775	AGCAGTT	A	0.066	84	PASS	PWWP3B	stop_gained&frameshift_variant	Dura AdCa
chrX	107597779	A	T	0.898	167	PASS	FRMPD3	missense_variant	Dura AdCa
chrX	107650066	C	T	0.059	52	PASS	PRPS1	3_prime_utr_variant	Dura AdCa
chrX	115191561	A	G	0.95	251	PASS	RBMXL3	missense_variant	Dura AdCa
chrX	136347662	G	C	0.061	100	PASS	ADGRG4	missense_variant	Dura AdCa
chrX	143631075	GATTGTC	G	0.085	85	PASS	SLITRK4	stop_gained&frameshift_variant	Dura AdCa
chrX	143631077	GAGCTTA	G	0.086	82	PASS	SLITRK4	stop_gained&frameshift_variant	Dura AdCa
chrX	150512284	C	T	0.987	104	PASS	MAMLD1	3_prime_utr_variant	Dura AdCa
chrX	152915171	C	CT	0.812	159	PASS	ZNF185	frameshift_variant	Dura AdCa
chrX	153420914	T	TTACGCC	0.02	329	PASS	ZFP92	inframe_deletion	Dura AdCa
chrX	153550226	A	G	0.037	170	PASS	ATP2B3	missense_variant	Dura AdCa
chrX	153955119	T	C	0.032	228	PASS	HCFC1	missense_variant	Dura AdCa
chrX	153958514	A	G	0.987	105	PASS	HCFC1	intron_variant	Dura AdCa
chrX	154413569	TAGGTGC	T	0.055	141	PASS	TAFAZZIN	splice_region_variant&intron_variant	Dura AdCa
chr1	183274	A	G	0.116	77	PASS	DDX11L17	intron_variant&non_coding_transcript_variant	Brain NEPC
chr1	184142	T	C	0.071	126	PASS	DDX11L17	non_coding_transcript_exon_variant	Brain NEPC
chr1	972913	T	A	0.938	52	PASS	PLEKHN1	missense_variant	Brain NEPC
chr1	1223422	G	A	0.921	117	PASS	SDF4	intron_variant	Brain NEPC
chr1	2408773	A	G	0.879	120	PASS	PEX10	synonymous_variant	Brain NEPC
chr1	12883168	TG	CA	0.636	213	PASS	PRAMEF4	missense_variant	Brain NEPC
chr1	13224919	A	T	0.305	779	PASS	PRAMEF18	missense_variant	Brain NEPC
chr1	13225068	G	A	0.243	1790	PASS	PRAMEF18	missense_variant	Brain NEPC
chr1	13225208	G	C	0.209	3596	PASS	PRAMEF18	synonymous_variant	Brain NEPC
chr1	13225514	C	T	0.25	109	PASS	PRAMEF18	intron_variant	Brain NEPC
chr1	13306781	G	A	0.162	586	PASS	PRAMEF33	missense_variant	Brain NEPC
chr1	13318650	G	A	0.236	321	PASS	PRAMEF15	synonymous_variant	Brain NEPC
chr1	16646619	A	AGCCATC	0.132	1401	PASS	CROCCP2	intron_variant&non_coding_transcript_variant	Brain NEPC
chr1	16761036	A	G	0.185	1264	PASS	CROCC	intron_variant&non_coding_transcript_variant	Brain NEPC
chr1	26166079	A	G	0.426	240	PASS	C1orf232	missense_variant	Brain NEPC
chr1	33569487	G	A	0.445	581	PASS	CSMD2	missense_variant	Brain NEPC
chr1	36308186	A	G	0.47	204	PASS	SH3D21	missense_variant	Brain NEPC
chr1	42521285	C	T	0.899	64	PASS	CCDC30	intron_variant	Brain NEPC
chr1	43338669	A	G	0.065	1245	PASS	MPL	missense_variant	Brain NEPC
chr1	44139048	TCTGGTT	T	0.998	944	PASS	KLF18	inframe_insertion	Brain NEPC
chr1	44139334	A	G	0.073	1289	PASS	KLF18	synonymous_variant	Brain NEPC
chr1	44139841	G	A	0.038	1318	PASS	KLF18	synonymous_variant	Brain NEPC
chr1	55092049	C	A	0.913	109	PASS	USP24	synonymous_variant	Brain NEPC
chr1	74612659	T	C	0.085	809	PASS	ERICH3	missense_variant	Brain NEPC
chr1	81907118	G	A	0.325	1101	PASS	ADGRL2	missense_variant	Brain NEPC
chr1	99712018	T	C	0.458	204	PASS	FRRS1	intron_variant	Brain NEPC
chr1	111231179	C	T	0.538	358	PASS	CHI3L2	intron_variant	Brain NEPC
chr1	120543014	C	T	0.441	523	PASS	.	splice_donor_region_variant&intron_variant&non_codin	Brain NEPC
chr1	120890143	G	A	0.029	1685	PASS	PPIAL4A	missense_variant	Brain NEPC

chr1	143796246	C	T	0.992	164	PASS	LINC02591	non_coding_transcript_exon_variant	Brain NEPC
chr1	145241802	C	T	0.621	840	PASS	PPIAL4D	missense_variant	Brain NEPC
chr1	145292353	C	T	0.163	327	PASS	NBPF20	intron_variant	Brain NEPC
chr1	145588056	G	C	0.026	632	PASS	NBPF25P	non_coding_transcript_exon_variant	Brain NEPC
chr1	146622487	C	G	0.389	149	PASS	.	non_coding_transcript_exon_variant	Brain NEPC
chr1	147631053	G	C	0.394	132	PASS	ACP6	stop_lost	Brain NEPC
chr1	147968240	A	G	0.458	109	PASS	GPR89B	intron_variant	Brain NEPC
chr1	148572642	C	T	0.083	209	PASS	NBPF14	intron_variant	Brain NEPC
chr1	149176847	C	T	0.486	54	PASS	.	intron_variant&non_coding_transcript_variant	Brain NEPC
chr1	151368992	T	C	0.413	365	PASS	SELENBP1	intron_variant	Brain NEPC
chr1	152213623	T	C	0.65	489	PASS	HRNR	missense_variant	Brain NEPC
chr1	152214592	T	G	0.097	3669	PASS	HRNR	missense_variant	Brain NEPC
chr1	152219783	C	T	0.163	1887	PASS	HRNR	missense_variant	Brain NEPC
chr1	157831950	C	G	0.18	222	PASS	CD5L	3_prime_utr_variant	Brain NEPC
chr1	159066010	C	G	0.448	881	PASS	AIM2	missense_variant	Brain NEPC
chr1	160031429	C	A	0.526	168	PASS	PIGM	missense_variant	Brain NEPC
chr1	160195116	T	C	0.484	323	PASS	CASQ1	synonymous_variant	Brain NEPC
chr1	160752273	TG	CA	0.436	323	PASS	SLAMF7	intron_variant	Brain NEPC
chr1	171185664	A	G	0.57	175	PASS	.	intron_variant&non_coding_transcript_variant	Brain NEPC
chr1	174220968	A	G	0.529	159	PASS	RABGAP1L	splice_region_variant&splice_polypyrimidine_tract_varia	Brain NEPC
chr1	205984165	T	G	0.995	311	PASS	RAB7B	intron_variant	Brain NEPC
chr1	212386985	A	G	0.392	486	PASS	PACC1	synonymous_variant	Brain NEPC
chr1	220599753	G	A	0.539	85	PASS	MARK1	intron_variant	Brain NEPC
chr1	224191000	G	C	0.191	138	PASS	DEGS1	intron_variant	Brain NEPC
chr1	243165585	T	G	0.22	545	PASS	CEP170	stop_gained	Brain NEPC
chr1	243172878	T	C	0.232	112	PASS	CEP170	intron_variant	Brain NEPC
chr1	248453100	A	G	0.201	1776	PASS	OR2T2	synonymous_variant	Brain NEPC
chr1	248453107	T	C	0.201	1789	PASS	OR2T2	missense_variant	Brain NEPC
chr2	17714908	T	C	0.464	453	PASS	SMC6	synonymous_variant	Brain NEPC
chr2	25130488	C	A	0.497	492	PASS	EFR3B	intron_variant	Brain NEPC
chr2	27206532	T	C	0.481	433	PASS	SLC5A6	splice_region_variant&synonymous_variant	Brain NEPC
chr2	31178617	G	T	0.527	329	PASS	CAPN14	intron_variant	Brain NEPC
chr2	43801586	A	G	0.476	219	PASS	DYNC2LI1	intron_variant	Brain NEPC
chr2	63856871	A	G	0.862	107	PASS	UGP2	intron_variant	Brain NEPC
chr2	69479044	A	C	0.105	165	PASS	AAK1	missense_variant	Brain NEPC
chr2	74459919	T	C	0.924	175	PASS	WBP1	synonymous_variant	Brain NEPC
chr2	75646803	G	C	0.926	135	PASS	MRPL19	5_prime_utr_variant	Brain NEPC
chr2	89221621	A	T	0.498	204	PASS	IGKV2-28	downstream_gene_variant	Brain NEPC
chr2	89640376	T	C	0.205	459	PASS	KMT2CP4	intron_variant&non_coding_transcript_variant	Brain NEPC
chr2	97208411	A	G	0.158	81	PASS	ANKRD36	intron_variant	Brain NEPC
chr2	97549630	A	G	0.21	173	PASS	ANKRD36B	splice_polypyrimidine_tract_variant&intron_variant	Brain NEPC
chr2	111123759	A	C	0.544	561	PASS	BCL2L11	missense_variant	Brain NEPC
chr2	111150001	A	G	0.498	3168	PASS	MIR4435-2HG	intron_variant&non_coding_transcript_variant	Brain NEPC
chr2	111150163	G	T	0.502	3119	PASS	MIR4435-2HG	intron_variant&non_coding_transcript_variant	Brain NEPC
chr2	113443854	A	G	0.147	118	PASS	ZNG1B	missense_variant	Brain NEPC
chr2	121732050	C	A	0.965	372	PASS	NIFK	intron_variant	Brain NEPC
chr2	134936927	G	T	0.044	446	PASS	CCNT2	synonymous_variant	Brain NEPC
chr2	138671160	C	T	0.059	605	PASS	NXPH2	missense_variant	Brain NEPC
chr2	148483570	C	T	0.957	634	PASS	MBD5	synonymous_variant	Brain NEPC
chr2	163611058	T	C	0.503	593	PASS	FIGN	synonymous_variant	Brain NEPC
chr2	171028370	T	A	0.368	277	PASS	TLK1	missense_variant	Brain NEPC
chr2	181898985	C	A	0.671	86	PASS	ITPRID2	intron_variant	Brain NEPC
chr2	197470105	A	G	0.314	619	PASS	COQ10B	synonymous_variant	Brain NEPC
chr2	203351532	GT	G	0.865	189	PASS	ABI2	intron_variant	Brain NEPC
chr2	232381426	T	C	0.407	365	PASS	ALPP	intron_variant	Brain NEPC
chr3	11017287	G	A	0.492	818	PASS	SLC6A1	missense_variant	Brain NEPC
chr3	12004864	G	C	0.545	54	PASS	SYN2	missense_variant	Brain NEPC
chr3	13503039	A	G	0.443	236	PASS	HDAC11	intron_variant	Brain NEPC
chr3	14158878	C	G	0.513	1006	PASS	XPC	synonymous_variant	Brain NEPC
chr3	14178475	T	C	0.498	658	PASS	XPC	missense_variant	Brain NEPC
chr3	27287772	A	T	0.65	55	PASS	NEK10	intron_variant	Brain NEPC
chr3	27297207	C	T	0.475	677	PASS	NEK10	missense_variant	Brain NEPC
chr3	27344259	C	G	0.342	370	PASS	NEK10	intron_variant	Brain NEPC
chr3	32159585	T	G	0.436	288	PASS	GPD1L	missense_variant	Brain NEPC
chr3	33400153	G	A	0.474	109	PASS	UBP1	intron_variant	Brain NEPC
chr3	33842000	A	G	0.474	594	PASS	PDCD6IP	missense_variant	Brain NEPC
chr3	46673502	A	G	0.948	114	PASS	ALS2CL	intron_variant	Brain NEPC
chr3	47857472	C	G	0.857	235	PASS	MAP4	missense_variant	Brain NEPC
chr3	48381707	A	G	0.874	392	PASS	FBXW12	synonymous_variant	Brain NEPC
chr3	49005095	C	A	0.935	268	PASS	P4HTM	intron_variant	Brain NEPC
chr3	49375600	C	CA	0.059	1153	PASS	RHOA	splice_polypyrimidine_tract_variant&intron_variant	Brain NEPC
chr3	50358224	A	G	0.952	252	PASS	TMEM115	synonymous_variant	Brain NEPC
chr3	50365459	A	G	0.975	272	PASS	CACNA2D2	missense_variant	Brain NEPC
chr3	51361872	T	A	0.952	345	PASS	DOCK3	missense_variant	Brain NEPC
chr3	57368206	C	T	0.919	94	PASS	DNAH12	synonymous_variant	Brain NEPC
chr3	57377088	G	A	0.928	251	PASS	DNAH12	synonymous_variant	Brain NEPC
chr3	57380252	A	G	0.939	78	PASS	DNAH12	intron_variant	Brain NEPC
chr3	72846298	C	CT	0.533	58	PASS	SHQ1	intron_variant	Brain NEPC
chr3	86968949	A	T	0.51	564	PASS	VGLL3	missense_variant	Brain NEPC
chr3	88139852	G	A	0.512	549	PASS	ZNF654	missense_variant	Brain NEPC
chr3	101671336	T	C	0.436	345	PASS	ZBTB11	missense_variant	Brain NEPC
chr3	108583144	A	C	0.417	70	PASS	CIP2A	intron_variant	Brain NEPC
chr3	124699935	A	G	0.504	522	PASS	KALRN	missense_variant	Brain NEPC
chr3	132691283	C	A	0.45	168	PASS	NPHP3	missense_variant	Brain NEPC
chr3	179973339	G	A	0.491	512	PASS	.	intron_variant&non_coding_transcript_variant	Brain NEPC
chr3	184038505	C	T	0.176	294	PASS	HTR3D	missense_variant	Brain NEPC

chr3	184038509	C	T	0.176	293	PASS	HTR3D	synonymous_variant	Brain NEPC
chr3	195618957	GA	AG	0.568	352	PASS	MUC20P1	non_coding_transcript_exon_variant	Brain NEPC
chr3	195740966	G	A	0.259	362	PASS	MUC20	downstream_gene_variant	Brain NEPC
chr3	195740982	T	C	0.243	279	PASS	MUC20	downstream_gene_variant	Brain NEPC
chr3	195781775	C	CGGTGA(	0.721	340	PASS	MUC4	inframe_deletion	Brain NEPC
chr3	195784172	C	T	0.224	3106	PASS	MUC4	missense_variant	Brain NEPC
chr3	195785472	A	G	0.323	2247	PASS	MUC4	synonymous_variant	Brain NEPC
chr3	195785502	GGAT	G	0.327	2329	PASS	MUC4	inframe_insertion	Brain NEPC
chr3	195786727	A	G	0.26	2603	PASS	MUC4	missense_variant	Brain NEPC
chr3	195788077	A	G	0.24	660	PASS	MUC4	missense_variant	Brain NEPC
chr3	195788085	G	C	0.742	723	PASS	MUC4	missense_variant	Brain NEPC
chr4	1214317	C	G	0.909	91	PASS	CTBP1	intron_variant	Brain NEPC
chr4	2198639	A	G	0.936	247	PASS	POLN	missense_variant	Brain NEPC
chr4	2250604	A	G	0.944	85	PASS	MXD4	synonymous_variant	Brain NEPC
chr4	2251199	A	C	0.928	119	PASS	MXD4	synonymous_variant	Brain NEPC
chr4	2931682	A	G	0.891	101	PASS	MFSD10	intron_variant	Brain NEPC
chr4	8612086	A	G	0.94	368	PASS	CPZ	synonymous_variant	Brain NEPC
chr4	9230471	A	C	0.096	3348	PASS	.	intron_variant&non_coding_transcript_variant	Brain NEPC
chr4	9230473	C	T	0.1	3298	PASS	.	intron_variant&non_coding_transcript_variant	Brain NEPC
chr4	9268697	T	C	0.086	188	PASS	USP17L22	missense_variant	Brain NEPC
chr4	10443362	G	A	0.939	572	PASS	ZNF518B	synonymous_variant	Brain NEPC
chr4	36092076	G	A	0.918	85	PASS	ARAP2	intron_variant	Brain NEPC
chr4	55080004	A	G	0.49	3074	PASS	KDR	synonymous_variant	Brain NEPC
chr4	70637844	T	G	0.448	522	PASS	ENAM	splice_donor_variant	Brain NEPC
chr4	73576589	C	G	0.034	311	PASS	RASSF6	intron_variant	Brain NEPC
chr4	74038340	A	T	0.441	516	PASS	CXCL3	missense_variant	Brain NEPC
chr4	75596341	T	C	0.892	115	PASS	CDKL2	splice_acceptor_variant	Brain NEPC
chr4	76740701	T	A	0.936	274	PASS	SHROOM3	missense_variant	Brain NEPC
chr4	86689645	A	C	0.895	253	PASS	PTPN13	intron_variant	Brain NEPC
chr4	86689656	A	G	0.898	260	PASS	PTPN13	intron_variant	Brain NEPC
chr4	94489017	G	A	0.897	274	PASS	PDLIM5	intron_variant	Brain NEPC
chr4	125434296	C	T	0.422	602	PASS	FAT4	missense_variant	Brain NEPC
chr4	150928642	A	T	0.489	78	PASS	LRBA	intron_variant	Brain NEPC
chr4	155710866	T	C	0.509	1004	PASS	GUCY1A1	missense_variant	Brain NEPC
chr5	181614	TG	CC	0.414	424	PASS	PLEKHG4B	missense_variant	Brain NEPC
chr5	878575	T	C	0.267	241	PASS	BRD9	intron_variant	Brain NEPC
chr5	1432496	G	A	0.513	220	PASS	SLC6A3	synonymous_variant	Brain NEPC
chr5	5239928	T	A	0.456	545	PASS	ADAMTS16	splice_donor_region_variant&intron_variant	Brain NEPC
chr5	32780689	G	C	0.479	555	PASS	NPR3	intron_variant	Brain NEPC
chr5	55220230	T	A	0.894	63	PASS	MCIDAS	3_prime_utr_variant	Brain NEPC
chr5	57214174	A	C	0.879	414	PASS	GPBP1	missense_variant	Brain NEPC
chr5	65609399	G	A	0.061	423	PASS	TRIM23	synonymous_variant	Brain NEPC
chr5	68296196	A	G	0.04	1474	PASS	PIK3R1	missense_variant	Brain NEPC
chr5	75029221	A	G	0.398	576	PASS	GCNT4	missense_variant	Brain NEPC
chr5	98863506	C	T	0.919	61	PASS	CHD1	synonymous_variant	Brain NEPC
chr5	111092331	T	C	0.079	172	PASS	.	upstream_gene_variant	Brain NEPC
chr5	140648249	G	A	0.059	156	PASS	IK	intron_variant	Brain NEPC
chr5	157288643	C	G	0.282	779	PASS	CYFIP2	intron_variant	Brain NEPC
chr5	159174135	A	G	0.343	439	PASS	RNF145	synonymous_variant	Brain NEPC
chr5	172968611	T	G	0.597	701	PASS	RPL26L1	intron_variant	Brain NEPC
chr5	176010999	C	T	0.204	474	PASS	THOC3	intron_variant	Brain NEPC
chr5	178147850	A	G	0.606	1142	PASS	RMND5B	missense_variant	Brain NEPC
chr5	179076409	T	G	0.62	1063	PASS	ZNF354C	intron_variant	Brain NEPC
chr6	147522	A	G	0.282	265	PASS	SEPTIN14P6	upstream_gene_variant	Brain NEPC
chr6	4125973	T	C	0.44	338	PASS	ECI2	intron_variant	Brain NEPC
chr6	4996429	A	C	0.514	165	PASS	RPP40	splice_polypyrimidine_tract_variant&intron_variant	Brain NEPC
chr6	25777988	T	C	0.45	506	PASS	SLC17A4	missense_variant	Brain NEPC
chr6	30987289	A	ACCAGCA	0.015	1092	PASS	MUC21	inframe_deletion	Brain NEPC
chr6	31029723	AAGGCT(	A	0.543	1182	PASS	MUC22	inframe_insertion	Brain NEPC
chr6	31202450	GC	AG	0.516	326	PASS	HCG27	non_coding_transcript_exon_variant	Brain NEPC
chr6	31688737	T	C	0.467	273	PASS	ABHD16A	synonymous_variant	Brain NEPC
chr6	32029823	G	C	0.476	695	PASS	C4B	intron_variant	Brain NEPC
chr6	32554821	G	A	0.278	708	PASS	HLA-DRB6	non_coding_transcript_exon_variant	Brain NEPC
chr6	32554826	C	G	0.268	701	PASS	HLA-DRB6	non_coding_transcript_exon_variant	Brain NEPC
chr6	34417760	A	G	0.491	505	PASS	RPS10	intron_variant	Brain NEPC
chr6	34883049	C	G	0.287	161	PASS	TAF11	missense_variant	Brain NEPC
chr6	43013204	T	G	0.39	276	PASS	MEA1	missense_variant	Brain NEPC
chr6	47879234	A	T	0.487	1366	PASS	PTCHD4	missense_variant	Brain NEPC
chr6	63778108	C	T	0.091	417	PASS	EYS	missense_variant	Brain NEPC
chr6	117403216	T	C	0.064	1525	PASS	ROS1	missense_variant	Brain NEPC
chr6	117519881	T	C	0.897	354	PASS	DCBLD1	missense_variant	Brain NEPC
chr6	122451974	C	T	0.891	196	PASS	SERINC1	missense_variant	Brain NEPC
chr6	125889651	A	G	0.886	151	PASS	NCOA7	missense_variant	Brain NEPC
chr6	142133970	A	G	0.39	530	PASS	GJE1	missense_variant	Brain NEPC
chr6	163060783	T	A	0.132	430	PASS	PACRG	intron_variant	Brain NEPC
chr6	169667944	A	G	0.479	437	PASS	WDR27	intron_variant	Brain NEPC
chr7	2568893	A	G	0.917	83	PASS	IQCE	intron_variant	Brain NEPC
chr7	2712852	T	C	0.938	97	PASS	AMZ1	missense_variant	Brain NEPC
chr7	34969503	C	A	0.137	102	PASS	DPY19L1	missense_variant	Brain NEPC
chr7	34969508	A	G	0.141	110	PASS	DPY19L1	synonymous_variant	Brain NEPC
chr7	37225044	C	T	0.943	430	PASS	ELMO1	splice_polypyrimidine_tract_variant&intron_variant	Brain NEPC
chr7	57132116	T	C	0.853	118	PASS	ZNF479	intron_variant	Brain NEPC
chr7	77953963	G	C	0.933	133	PASS	PHTF2	intron_variant	Brain NEPC
chr7	100682882	T	C	0.948	82	PASS	GIGYF1	intron_variant	Brain NEPC
chr7	100952493	G	A	0.404	476	PASS	MUC3A	synonymous_variant	Brain NEPC
chr7	100952498	T	C	0.398	464	PASS	MUC3A	missense_variant	Brain NEPC

chr7	100954571	C	G	0.332	416	PASS	MUC3A	missense_variant	Brain NEPC
chr7	100954574	C	G	0.326	407	PASS	MUC3A	missense_variant	Brain NEPC
chr7	111846967	A	G	0.964	154	PASS	DOCK4	intron_variant	Brain NEPC
chr7	128880045	A	C	0.113	119	PASS	KCP	missense_variant	Brain NEPC
chr7	130129605	C	T	0.037	150	PASS	KLHDC10	intron_variant	Brain NEPC
chr7	130192705	A	T	0.926	241	PASS	TMEM209	missense_variant	Brain NEPC
chr7	130549432	G	C	0.086	116	PASS	COPG2	intron_variant	Brain NEPC
chr7	130550642	T	C	0.052	219	PASS	COPG2	synonymous_variant	Brain NEPC
chr7	132164067	A	C	0.045	168	PASS	PLXNA4	intron_variant	Brain NEPC
chr7	142078849	T	C	0.854	855	PASS	MGAM	synonymous_variant	Brain NEPC
chr7	142085767	G	C	0.129	470	PASS	MGAM	intron_variant	Brain NEPC
chr7	142451194	CA	GG	0.232	650	PASS	TRBV6-5	missense_variant	Brain NEPC
chr7	142463547	G	C	0.122	257	PASS	TRBV5-4	missense_variant	Brain NEPC
chr7	142564042	T	C	0.923	746	PASS	TRBV12-4	synonymous_variant	Brain NEPC
chr7	142588240	A	G	0.932	252	PASS	TRBV14	synonymous_variant	Brain NEPC
chr7	142598094	T	C	0.952	280	PASS	TRBV16	intron_variant	Brain NEPC
chr7	142774511	T	C	0.403	919	PASS	PRSS2	3_prime_utr_variant	Brain NEPC
chr7	142774515	CA	TG	0.404	912	PASS	PRSS2	3_prime_utr_variant	Brain NEPC
chr7	142791704	A	C	0.097	360	PASS	TRBC1	missense_variant	Brain NEPC
chr7	142791707	C	G	0.108	368	PASS	TRBC1	missense_variant	Brain NEPC
chr7	142791866	A	G	0.172	377	PASS	TRBC1	synonymous_variant	Brain NEPC
chr7	142802530	T	C	0.949	241	PASS	TRBC2	3_prime_utr_variant	Brain NEPC
chr7	143183843	C	T	0.943	381	PASS	TAS2R39	missense_variant	Brain NEPC
chr7	143286052	G	A	0.9	70	PASS	TMEM139	missense_variant	Brain NEPC
chr7	149005223	A	G	0.097	102	PASS	PDIA4	synonymous_variant	Brain NEPC
chr7	149072336	T	C	0.907	206	PASS	ZNF786	missense_variant	Brain NEPC
chr7	149282310	A	C	0.868	85	PASS	ZNF783	synonymous_variant	Brain NEPC
chr7	149783037	T	C	0.955	265	PASS	SSPOP	intron_variant&non_coding_transcript_variant	Brain NEPC
chr7	149804049	A	G	0.939	71	PASS	SSPOP	non_coding_transcript_exon_variant	Brain NEPC
chr7	152181521	G	T	0.06	2029	PASS	KMT2C	synonymous_variant	Brain NEPC
chr7	152252528	A	G	0.922	1090	PASS	KMT2C	intron_variant	Brain NEPC
chr7	154944075	T	C	0.943	68	PASS	PAXIP1	3_prime_utr_variant	Brain NEPC
chr7	155739906	T	C	0.873	71	PASS	RBM33	synonymous_variant	Brain NEPC
chr8	166783	G	A	0.304	194	PASS	OR4F21	missense_variant	Brain NEPC
chr8	1680556	AT	GA	0.098	156	PASS	DLGAP2	intron_variant	Brain NEPC
chr8	3201560	T	A	0.853	100	PASS	CSMD1	intron_variant	Brain NEPC
chr8	20165160	T	C	0.942	86	PASS	SLC18A1	intron_variant	Brain NEPC
chr8	22227651	T	C	0.932	63	PASS	PHYHIP	intron_variant	Brain NEPC
chr8	30138778	A	G	0.889	106	PASS	MBOAT4	intron_variant	Brain NEPC
chr8	31080997	G	A	0.058	1289	PASS	WRN	missense_variant	Brain NEPC
chr8	37848719	G	C	0.421	332	PASS	BRF2	intron_variant	Brain NEPC
chr8	40786704	G	C	0.308	972	PASS	ZMAT4	intron_variant	Brain NEPC
chr8	42756343	G	A	0.629	1043	PASS	CHRNA6	synonymous_variant	Brain NEPC
chr8	124998624	A	G	0.582	200	PASS	SQLE	5_prime_utr_variant	Brain NEPC
chr8	131946634	C	G	0.533	748	PASS	EFR3A	splice_donor_variant	Brain NEPC
chr8	143274796	C	T	0.502	1159	PASS	GLI4	missense_variant	Brain NEPC
chr8	143606582	G	C	0.412	241	PASS	PYCR3	missense_variant	Brain NEPC
chr8	143858206	A	G	0.442	774	PASS	EPPK1	synonymous_variant	Brain NEPC
chr8	144264608	A	G	0.419	799	PASS	BOP1	synonymous_variant	Brain NEPC
chr8	144430589	T	C	0.245	226	PASS	TONSL	intron_variant	Brain NEPC
chr9	7011765	T	C	0.534	608	PASS	KDM4C	synonymous_variant	Brain NEPC
chr9	22451048	C	T	0.398	211	PASS	DMRTA1	splice_polypyrimidine_tract_variant&intron_variant	Brain NEPC
chr9	33540187	TT	CA	0.053	50	PASS	ANKRD18B	missense_variant	Brain NEPC
chr9	41926666	A	G	0.288	444	PASS	CNTNAP3B	intron_variant	Brain NEPC
chr9	78317564	A	C	0.494	125	PASS	PSAT1	intron_variant	Brain NEPC
chr9	78317567	T	A	0.489	129	PASS	PSAT1	intron_variant	Brain NEPC
chr9	81992641	C	T	0.487	641	PASS	SPATA31D1	missense_variant	Brain NEPC
chr9	82058619	CA	GC	0.436	872	PASS	.	intron_variant&non_coding_transcript_variant	Brain NEPC
chr9	89386454	A	G	0.079	351	PASS	SEMA4D	synonymous_variant	Brain NEPC
chr9	104617172	G	A	0.495	330	PASS	OR13C9	downstream_gene_variant	Brain NEPC
chr9	114236902	A	C	0.125	57	PASS	COL27A1	intron_variant	Brain NEPC
chr9	128998146	C	T	0.459	715	PASS	NUP188	splice_region_variant&splice_polypyrimidine_tract_vari	Brain NEPC
chr9	130319410	G	A	0.983	80	PASS	HMCN2	intron_variant	Brain NEPC
chr9	130319644	A	G	0.503	175	PASS	HMCN2	missense_variant	Brain NEPC
chr9	130321760	G	A	0.536	162	PASS	HMCN2	intron_variant	Brain NEPC
chr9	130327557	C	T	0.529	109	PASS	HMCN2	intron_variant	Brain NEPC
chr9	130338375	G	A	0.434	163	PASS	HMCN2	intron_variant	Brain NEPC
chr9	130338512	G	A	0.532	127	PASS	HMCN2	intron_variant	Brain NEPC
chr9	133205969	G	T	0.18	449	PASS	OBP2B	intron_variant	Brain NEPC
chr9	136290477	G	A	0.984	89	PASS	CCDC187	intron_variant	Brain NEPC
chr9	136291438	T	C	0.5	224	PASS	CCDC187	missense_variant	Brain NEPC
chr9	136459582	T	C	0.518	199	PASS	SEC16A	intron_variant	Brain NEPC
chr9	136740862	C	T	0.482	394	PASS	LCN10	missense_variant	Brain NEPC
chr9	137064633	T	C	0.503	230	PASS	SAPCD2	3_prime_utr_variant	Brain NEPC
chr9	137213879	A	G	0.438	260	PASS	NDOR1	splice_donor_variant	Brain NEPC
chr9	137220533	T	C	0.477	378	PASS	RNF208	stop_gained	Brain NEPC
chr10	12117710	A	C	0.362	357	PASS	DHTKD1	missense_variant	Brain NEPC
chr10	19327707	A	T	0.508	404	PASS	MALRD1	intron_variant	Brain NEPC
chr10	25598926	G	A	0.449	704	PASS	GPR158	synonymous_variant	Brain NEPC
chr10	31908696	G	T	0.462	1278	PASS	ARHGAP12	missense_variant	Brain NEPC
chr10	47367976	T	C	0.94	354	PASS	ZNF488	missense_variant	Brain NEPC
chr10	52768241	C	T	0.071	774	PASS	MBL2	missense_variant	Brain NEPC
chr10	78029459	G	A	0.418	123	PASS	POLR3A	5_prime_utr_variant	Brain NEPC
chr10	86499769	C	G	0.945	230	PASS	WAPL	missense_variant	Brain NEPC
chr10	86924605	T	C	0.808	142	PASS	BMPR1A	3_prime_utr_variant	Brain NEPC
chr10	92291418	T	C	0.886	84	PASS	MARCHF5	5_prime_utr_variant	Brain NEPC

chr10	93325895	G	A	0.942	364	PASS	MYOF	synonymous_variant	Brain NEPC
chr10	101596654	G	C	0.871	196	PASS	DPCD	intron_variant	Brain NEPC
chr10	103590376	A	G	0.953	83	PASS	NEURL1	3_prime_utr_variant	Brain NEPC
chr10	110498227	A	G	0.477	191	PASS	DUSP5	missense_variant	Brain NEPC
chr10	120880780	T	A	0.487	196	PASS	WDR11	intron_variant	Brain NEPC
chr10	121482083	C	T	0.01	586	PASS	FGFR2	intron_variant	Brain NEPC
chr10	125852632	A	G	0.495	707	PASS	DHX32	missense_variant	Brain NEPC
chr10	133626722	C	G	0.383	566	PASS	FRG2B	missense_variant	Brain NEPC
chr11	372543	A	G	0.913	97	PASS	B4GALNT4	intron_variant	Brain NEPC
chr11	800835	T	C	0.928	73	PASS	PIDD1	missense_variant	Brain NEPC
chr11	940991	G	A	0.873	91	PASS	AP2A2	intron_variant	Brain NEPC
chr11	1013601	T	C	0.108	193	PASS	MUC6	missense_variant	Brain NEPC
chr11	1181260	T	C	0.988	147	PASS	MUC5AC	splice_polypyrimidine_tract_variant&intron_variant	Brain NEPC
chr11	3683204	C	A	0.063	1207	PASS	NUP98	synonymous_variant	Brain NEPC
chr11	7000644	A	T	0.898	425	PASS	ZNF214	missense_variant	Brain NEPC
chr11	17926764	C	T	0.873	199	PASS	SERGEF	intron_variant	Brain NEPC
chr11	18348064	T	C	0.89	129	PASS	GTF2H1	intron_variant	Brain NEPC
chr11	18709691	G	A	0.94	102	PASS	IGSF22	splice_region_variant&splice_polypyrimidine_tract_varia	Brain NEPC
chr11	27057152	C	G	0.961	171	PASS	BBOX1-AS1	intron_variant&non_coding_transcript_variant	Brain NEPC
chr11	47441634	T	C	0.472	318	PASS	RAPSN	missense_variant	Brain NEPC
chr11	48245442	T	C	0.093	353	PASS	OR4X2	synonymous_variant	Brain NEPC
chr11	61102576	T	C	0.502	71	PASS	CD5	synonymous_variant	Brain NEPC
chr11	64287893	T	C	0.401	281	PASS	GPR137	missense_variant	Brain NEPC
chr11	67452275	A	C	0.458	125	PASS	GPR152	missense_variant	Brain NEPC
chr11	75268982	C	G	0.438	251	PASS	ARRB1	intron_variant	Brain NEPC
chr11	89782118	C	A	0.389	610	PASS	.	non_coding_transcript_exon_variant	Brain NEPC
chr11	89870756	C	T	0.166	597	PASS	TRIM64B	synonymous_variant	Brain NEPC
chr11	113931878	TG	CA	0.934	390	PASS	HTR3B	intron_variant	Brain NEPC
chr11	117280014	A	G	0.736	83	PASS	RNF214	intron_variant	Brain NEPC
chr12	66973	T	A	0.844	167	PASS	IQSEC3	missense_variant	Brain NEPC
chr12	5969435	C	G	0.841	86	PASS	VWF	intron_variant	Brain NEPC
chr12	6599800	A	G	0.033	2556	PASS	CHD4	synonymous_variant	Brain NEPC
chr12	8221906	A	G	0.987	113	PASS	FAM90A1	synonymous_variant	Brain NEPC
chr12	9115692	G	T	0.491	362	PASS	A2M	intron_variant	Brain NEPC
chr12	24818019	A	C	0.535	627	PASS	BCAT1	missense_variant	Brain NEPC
chr12	29439321	C	T	0.522	240	PASS	OVCH1-AS1	intron_variant&non_coding_transcript_variant	Brain NEPC
chr12	40515401	AA	GG	0.041	336	PASS	MUC19	intron_variant	Brain NEPC
chr12	49050016	A	G	0.938	1239	PASS	KMT2D	missense_variant	Brain NEPC
chr12	51251592	G	GA	0.325	81	PASS	SMAGP	intron_variant	Brain NEPC
chr12	52519021	T	C	0.035	477	PASS	KRT5	missense_variant	Brain NEPC
chr12	54182416	G	A	0.866	221	PASS	SMUG1	missense_variant	Brain NEPC
chr12	54508543	T	C	0.949	358	PASS	NCKAP1L	intron_variant	Brain NEPC
chr12	62104733	AA	CC	0.824	439	PASS	TAFA2	intron_variant	Brain NEPC
chr12	101338126	G	C	0.478	645	PASS	UTP20	synonymous_variant	Brain NEPC
chr12	101651384	AGAAGG	A	0.405	311	PASS	MYBPC1	stop_gained&frameshift_variant	Brain NEPC
chr12	108940930	C	T	0.455	141	PASS	SVOP	intron_variant	Brain NEPC
chr12	111520955	G	T	0.397	441	PASS	ATXN2	missense_variant	Brain NEPC
chr12	124314124	C	T	0.463	160	PASS	RFLNA	intron_variant	Brain NEPC
chr12	131982290	AT	CC	0.444	261	PASS	EP400	missense_variant	Brain NEPC
chr12	133010734	A	G	0.489	836	PASS	ZNF26	synonymous_variant	Brain NEPC
chr12	133100956	C	T	0.72	182	PASS	ZNF140	intron_variant	Brain NEPC
chr13	24764197	C	G	0.05	154	PASS	RNF17	5_prime_utr_variant	Brain NEPC
chr13	34541522	A	G	0.355	91	PASS	LINC00457	intron_variant&non_coding_transcript_variant	Brain NEPC
chr13	113433759	A	G	0.878	67	PASS	ADPRHL1	missense_variant	Brain NEPC
chr14	19097884	T	G	0.289	65	PASS	DUXAP9	intron_variant&non_coding_transcript_variant	Brain NEPC
chr14	23402776	A	G	0.038	248	PASS	MYH6	missense_variant	Brain NEPC
chr14	23417278	T	G	0.467	382	PASS	MYH7	stop_gained	Brain NEPC
chr14	23424064	G	A	0.493	643	PASS	MYH7	missense_variant	Brain NEPC
chr14	45032186	C	G	0.572	147	PASS	KLHL28	intron_variant	Brain NEPC
chr14	45164495	A	G	0.469	3217	PASS	FANCM	missense_variant	Brain NEPC
chr14	50613419	T	G	0.567	175	PASS	ATL1	intron_variant	Brain NEPC
chr14	50770406	T	G	0.142	2331	PASS	NIN	synonymous_variant	Brain NEPC
chr14	63317716	C	G	0.037	221	PASS	GPHB5	missense_variant	Brain NEPC
chr14	90304150	A	G	0.512	767	PASS	NRDE2	missense_variant	Brain NEPC
chr14	91729459	T	A	0.118	56	PASS	CATSPERB	stop_gained	Brain NEPC
chr14	93348008	T	C	0.08	916	PASS	UNC79	intron_variant	Brain NEPC
chr14	100567885	T	C	0.5	94	PASS	BEGAIN	intron_variant	Brain NEPC
chr14	104741279	T	C	0.477	127	PASS	ADSS1	intron_variant	Brain NEPC
chr14	104932945	C	A	0.443	214	PASS	PLD4	missense_variant	Brain NEPC
chr14	105625935	C	G	0.194	376	PASS	IGHG4	synonymous_variant	Brain NEPC
chr14	105707471	A	G	0.306	715	PASS	IGHA1	synonymous_variant	Brain NEPC
chr14	105767772	G	A	0.099	166	PASS	IGHG3	intron_variant	Brain NEPC
chr14	106012330	C	G	0.444	577	PASS	IGHV4-4	synonymous_variant	Brain NEPC
chr14	106062072	G	T	0.131	614	PASS	IGHV3-7	downstream_gene_variant	Brain NEPC
chr14	106062074	G	T	0.13	614	PASS	IGHV3-7	downstream_gene_variant	Brain NEPC
chr14	106062169	A	G	0.451	1791	PASS	IGHV3-7	synonymous_variant	Brain NEPC
chr14	106335083	C	T	0.317	5315	PASS	IGHV3-30	missense_variant	Brain NEPC
chr14	106349771	A	T	0.509	1182	PASS	IGHV4-31	5_prime_utr_variant	Brain NEPC
chr14	106592708	A	G	0.306	1637	PASS	IGHV3-53	synonymous_variant	Brain NEPC
chr14	106704050	T	C	0.182	284	PASS	IGHV1-68	non_coding_transcript_exon_variant	Brain NEPC
chr14	106704052	G	A	0.182	284	PASS	IGHV1-68	non_coding_transcript_exon_variant	Brain NEPC
chr15	21609551	T	G	0.845	81	PASS	OR1Q1BP	non_coding_transcript_exon_variant	Brain NEPC
chr15	22431965	T	G	0.187	179	PASS	.	non_coding_transcript_exon_variant	Brain NEPC
chr15	25736076	G	C	0.885	97	PASS	ATP10A	synonymous_variant	Brain NEPC
chr15	29124687	G	C	0.896	140	PASS	ENTREP2	missense_variant&splice_region_variant	Brain NEPC
chr15	32446493	A	G	0.326	126	PASS	GOLGA8O	missense_variant	Brain NEPC

chr15	34154595	T	C	0.938	166	PASS	KATNBL1	intron_variant	Brain NEPC
chr15	34254487	T	C	0.066	370	PASS	SLC12A6	missense_variant	Brain NEPC
chr15	40296272	T	C	0.898	74	PASS	PLCB2	intron_variant	Brain NEPC
chr15	54013596	A	T	0.952	417	PASS	UNC13C	missense_variant	Brain NEPC
chr15	61884130	T	C	0.938	101	PASS	VPS13C	missense_variant&splice_region_variant	Brain NEPC
chr15	64957167	C	T	0.977	50	PASS	ANKDD1A	synonymous_variant	Brain NEPC
chr15	69014683	CC	AG	0.921	160	PASS	NOX5	upstream_gene_variant	Brain NEPC
chr15	74619971	T	G	0.903	307	PASS	CLK3	intron_variant	Brain NEPC
chr15	79025348	A	G	0.885	162	PASS	RASGRF1	missense_variant	Brain NEPC
chr15	82343155	A	C	0.687	119	PASS	GOLGA6L10	missense_variant	Brain NEPC
chr15	82345980	GT	CC	0.341	234	PASS	GOLGA6L10	intron_variant	Brain NEPC
chr15	82431812	C	T	0.98	56	PASS	GOLGA6L9	intron_variant	Brain NEPC
chr15	82434266	AC	GT	0.186	316	PASS	GOLGA6L9	missense_variant	Brain NEPC
chr15	82524937	A	G	0.07	80	PASS	GOLGA6L17P	intron_variant&non_coding_transcript_variant	Brain NEPC
chr15	101287733	A	G	0.873	166	PASS	SNRPA1	intron_variant	Brain NEPC
chr15	101966111	G	C	0.143	75	PASS	WASH3P	non_coding_transcript_exon_variant	Brain NEPC
chr16	281826	C	G	0.507	126	PASS	ARHGDIG	missense_variant	Brain NEPC
chr16	2324392	T	C	0.475	89	PASS	ABCA3	intron_variant	Brain NEPC
chr16	8645986	CGCCTGC	C	0.996	468	PASS	METTL22	intron_variant	Brain NEPC
chr16	9764661	A	G	0.497	5288	PASS	GRIN2A	synonymous_variant	Brain NEPC
chr16	11999364	T	C	0.431	1408	PASS	SNX29	splice_donor_region_variant&intron_variant	Brain NEPC
chr16	14951909	T	C	0.322	1182	PASS	NPIPA1	missense_variant	Brain NEPC
chr16	14975345	C	T	0.079	411	PASS	PDXDC1	intron_variant	Brain NEPC
chr16	15104429	G	GCTGAGC	0.269	532	PASS	PDXDC1	intron_variant	Brain NEPC
chr16	15129957	T	C	0.207	160	PASS	PDXDC1	intron_variant	Brain NEPC
chr16	18347905	G	A	0.18	215	PASS	PKD1P4	non_coding_transcript_exon_variant	Brain NEPC
chr16	19642309	T	A	0.412	204	PASS	VPS35L	intron_variant	Brain NEPC
chr16	22534710	CCGAG	C	0.118	157	PASS	NPIPB5	frameshift_variant	Brain NEPC
chr16	22534712	G	T	0.118	157	PASS	NPIPB5	missense_variant	Brain NEPC
chr16	29485610	A	ACACACT	0.138	213	PASS	NPIPB12	frameshift_variant	Brain NEPC
chr16	29485618	G	GGGAGG	0.143	200	PASS	NPIPB12	frameshift_variant	Brain NEPC
chr16	31793797	T	A	0.573	204	PASS	KRBOX5	non_coding_transcript_exon_variant	Brain NEPC
chr16	50668036	T	C	0.433	336	PASS	.	non_coding_transcript_exon_variant	Brain NEPC
chr16	58284658	C	G	0.897	302	PASS	PRSS54	missense_variant	Brain NEPC
chr16	67660036	T	C	0.972	287	PASS	ACD	missense_variant	Brain NEPC
chr16	72104500	T	C	0.08	354	PASS	DHX38	synonymous_variant	Brain NEPC
chr16	83780017	T	C	0.915	222	PASS	CDH13	synonymous_variant	Brain NEPC
chr16	84000706	T	G	0.868	104	PASS	NECAB2	intron_variant	Brain NEPC
chr16	84489419	C	T	0.898	160	PASS	MEAK7	missense_variant	Brain NEPC
chr16	88951011	G	GCCTGGA	0.872	598	PASS	CBFA2T3	intron_variant	Brain NEPC
chr16	89285522	T	C	0.075	765	PASS	ANKRD11	synonymous_variant	Brain NEPC
chr17	138824	A	C	0.993	240	PASS	SCGB1C2	intron_variant	Brain NEPC
chr17	7421264	A	G	0.968	229	PASS	SPEM1	missense_variant	Brain NEPC
chr17	7667396	C	G	0.964	150	PASS	TP53	downstream_gene_variant	Brain NEPC
chr17	7675085	T	C	0.936	1649	PASS	TP53	missense_variant	Brain NEPC
chr17	9728413	T	C	0.975	455	PASS	USP43	missense_variant	Brain NEPC
chr17	10542822	A	C	0.066	68	PASS	MYH2	intron_variant	Brain NEPC
chr17	18091852	TG	CA	0.992	167	PASS	DRG2	intron_variant	Brain NEPC
chr17	18978657	T	C	0.969	491	PASS	FAM83G	missense_variant	Brain NEPC
chr17	28048003	A	G	0.426	243	PASS	NLK	intron_variant	Brain NEPC
chr17	28369358	G	C	0.443	431	PASS	VTN	missense_variant	Brain NEPC
chr17	36171845	A	G	0.302	88	PASS	.	intron_variant&non_coding_transcript_variant	Brain NEPC
chr17	36195196	A	G	0.65	191	PASS	CCL3L3	3_prime_utr_variant	Brain NEPC
chr17	36195296	G	A	0.288	612	PASS	CCL3L3	missense_variant	Brain NEPC
chr17	36195375	G	A	0.274	770	PASS	CCL3L3	missense_variant&splice_region_variant	Brain NEPC
chr17	36195866	C	T	0.336	1052	PASS	CCL3L3	synonymous_variant	Brain NEPC
chr17	36196527	G	A	0.995	304	PASS	CCL3L3	intron_variant	Brain NEPC
chr17	36211278	T	G	0.998	666	PASS	CCL4L2	intron_variant	Brain NEPC
chr17	36211979	C	T	0.992	187	PASS	CCL4L2	intron_variant	Brain NEPC
chr17	36212342	GA	CG	0.997	526	PASS	CCL4L2	missense_variant	Brain NEPC
chr17	36307282	A	T	0.247	137	PASS	.	splice_polypyrimidine_tract_variant&intron_variant&non	Brain NEPC
chr17	36452733	A	G	0.436	166	PASS	TBC1D3JP	non_coding_transcript_exon_variant	Brain NEPC
chr17	37635608	T	A	0.4	513	PASS	DDX52	intron_variant	Brain NEPC
chr17	40390022	C	A	0.49	3523	PASS	TOP2A	synonymous_variant	Brain NEPC
chr17	40818851	AGCTGCC	A	0.446	151	PASS	KRT10	inframe_insertion	Brain NEPC
chr17	41140002	T	C	0.28	582	PASS	KRTAP4-6	synonymous_variant	Brain NEPC
chr17	41275814	T	G	0.058	683	PASS	KRTAP9-7	synonymous_variant	Brain NEPC
chr17	41567235	A	T	0.476	163	PASS	KRT9	splice_region_variant&3_prime_utr_variant	Brain NEPC
chr17	42329523	T	C	0.505	1522	PASS	STAT3	intron_variant	Brain NEPC
chr17	45547982	T	C	0.158	888	PASS	.	intron_variant&non_coding_transcript_variant	Brain NEPC
chr17	45547991	G	C	0.178	912	PASS	.	intron_variant&non_coding_transcript_variant	Brain NEPC
chr17	46259756	T	C	0.874	256	PASS	.	non_coding_transcript_exon_variant	Brain NEPC
chr17	46528798	A	G	0.098	293	PASS	ARL17A	intron_variant	Brain NEPC
chr17	56960853	C	A	0.943	362	PASS	COIL	missense_variant	Brain NEPC
chr17	63482589	T	C	0.967	455	PASS	ACE	synonymous_variant	Brain NEPC
chr17	64897294	G	A	0.127	178	PASS	LRRC37A3	5_prime_utr_variant	Brain NEPC
chr17	67138031	G	A	0.033	838	PASS	HELZ	synonymous_variant	Brain NEPC
chr17	69304746	T	C	0.07	84	PASS	ABCA5	missense_variant	Brain NEPC
chr17	73206291	A	C	0.054	419	PASS	COG1	intron_variant	Brain NEPC
chr17	75937609	G	C	0.034	552	PASS	FBF1	splice_polypyrimidine_tract_variant&intron_variant	Brain NEPC
chr17	79784185	C	G	0.964	311	PASS	CBX2	missense_variant	Brain NEPC
chr17	82374399	A	T	0.957	97	PASS	UTS2R	synonymous_variant	Brain NEPC
chr18	26865550	G	T	0.867	115	PASS	AQP4-AS1	intron_variant&non_coding_transcript_variant	Brain NEPC
chr18	28147775	G	C	0.934	149	PASS	CDH2	missense_variant	Brain NEPC
chr18	31399595	G	T	0.878	381	PASS	DSG4	intron_variant	Brain NEPC
chr18	34882022	A	T	0.913	130	PASS	DTNA	intron_variant	Brain NEPC

chr18	46509716	T	C	0.833	276	PASS	LOXHD1	synonymous_variant	Brain NEPC
chr18	48252638	C	T	0.094	350	PASS	ZBTB7C	intron_variant	Brain NEPC
chr18	58919052	T	C	0.916	124	PASS	ZNF532	synonymous_variant	Brain NEPC
chr19	1622424	A	G	0.797	149	PASS	TCF3	splice_polypyrimidine_tract_variant&intron_variant	Brain NEPC
chr19	8510850	C	CCCGCG(	0.811	144	PASS	ZNF414	inframe_deletion&splice_region_variant	Brain NEPC
chr19	8891843	C	G	0.365	910	PASS	MUC16	missense_variant	Brain NEPC
chr19	8946501	T	G	0.484	882	PASS	MUC16	missense_variant	Brain NEPC
chr19	8946645	A	C	0.499	634	PASS	MUC16	missense_variant	Brain NEPC
chr19	9350640	G	C	0.173	104	PASS	.	upstream_gene_variant	Brain NEPC
chr19	9350742	A	C	0.196	258	PASS	.	upstream_gene_variant	Brain NEPC
chr19	9350743	G	GT	0.196	258	PASS	ZNF559	downstream_gene_variant	Brain NEPC
chr19	18668216	A	G	0.358	473	PASS	KLHL26	synonymous_variant	Brain NEPC
chr19	18938522	A	G	0.432	545	PASS	HOMER3	intron_variant	Brain NEPC
chr19	19505356	G	C	0.496	188	PASS	GATAD2A	missense_variant	Brain NEPC
chr19	21808896	A	G	0.359	489	PASS	ZNF43	missense_variant	Brain NEPC
chr19	35359288	A	G	0.229	592	PASS	FFAR3	missense_variant	Brain NEPC
chr19	37380192	A	G	0.41	1861	PASS	ZNF527	intron_variant	Brain NEPC
chr19	38950233	T	C	0.538	248	PASS	FBXO17	synonymous_variant	Brain NEPC
chr19	40370082	A	C	0.513	344	PASS	PLD3	intron_variant	Brain NEPC
chr19	40523598	A	C	0.04	147	PASS	SPTBN4	stop_gained	Brain NEPC
chr19	40778492	CA	TG	0.466	132	PASS	RAB4B	intron_variant	Brain NEPC
chr19	41579543	T	G	0.411	462	PASS	CEACAM21	missense_variant	Brain NEPC
chr19	44496520	C	A	0.533	99	PASS	ZNF180	intron_variant	Brain NEPC
chr19	48510162	A	G	0.532	279	PASS	LMTK3	synonymous_variant	Brain NEPC
chr19	49101346	T	C	0.502	309	PASS	SNRNP70	intron_variant	Brain NEPC
chr19	53183041	A	C	0.447	170	PASS	ZNF665	intron_variant	Brain NEPC
chr19	54220739	A	C	0.484	764	PASS	LILRB3	synonymous_variant	Brain NEPC
chr19	54222756	C	T	0.632	796	PASS	LILRB3	missense_variant	Brain NEPC
chr19	54222987	T	C	0.33	587	PASS	LILRB3	5_prime_utr_variant	Brain NEPC
chr19	54239882	A	C	0.186	230	PASS	RPS9	intron_variant&nmd_transcript_variant	Brain NEPC
chr19	54242117	C	T	0.482	740	PASS	LILRA6	missense_variant	Brain NEPC
chr19	54667677	G	A	0.128	405	PASS	LILRB4	missense_variant	Brain NEPC
chr19	54727771	T	C	0.449	825	PASS	KIR3DL3	synonymous_variant	Brain NEPC
chr19	54755120	T	G	0.315	712	PASS	KIR2DP1	intron_variant&non_coding_transcript_variant	Brain NEPC
chr19	54760492	AT	GC	0.491	788	PASS	KIR2DP1	non_coding_transcript_exon_variant	Brain NEPC
chr19	54773534	A	C	0.324	2963	PASS	KIR2DL1	missense_variant	Brain NEPC
chr19	54773593	T	G	0.306	2702	PASS	KIR2DL1	missense_variant	Brain NEPC
chr19	54773692	C	A	0.22	1149	PASS	KIR2DL1	intron_variant	Brain NEPC
chr19	54773699	G	A	0.223	1063	PASS	KIR2DL1	intron_variant	Brain NEPC
chr19	54788295	A	G	0.065	1376	PASS	KIR3DP1	non_coding_transcript_exon_variant	Brain NEPC
chr19	54848199	G	C	0.071	620	PASS	KIR2DS4	3_prime_utr_variant	Brain NEPC
chr19	55644655	T	C	0.039	144	PASS	ZNF581	synonymous_variant	Brain NEPC
chr19	57491772	TG	CA	0.058	168	PASS	ZNF419	intron_variant	Brain NEPC
chr20	1636481	G	A	0.057	1076	PASS	SIRPG	missense_variant	Brain NEPC
chr20	3693091	C	G	0.077	98	PASS	SIGLEC1	missense_variant	Brain NEPC
chr20	24665463	A	G	0.488	320	PASS	SYNDIG1	missense_variant	Brain NEPC
chr20	46022943	GGGA	G	0.943	82	PASS	SLC12A5	intron_variant	Brain NEPC
chr20	54476047	A	G	0.505	363	PASS	DOK5	intron_variant	Brain NEPC
chr20	56637826	A	G	0.489	674	PASS	TFAP2C	missense_variant	Brain NEPC
chr21	5033666	A	G	0.65	285	PASS	.	intergenic_variant	Brain NEPC
chr21	13046287	T	G	0.541	191	PASS	.	intron_variant&non_coding_transcript_variant	Brain NEPC
chr21	28931146	C	T	0.572	143	PASS	LTN1	intron_variant	Brain NEPC
chr21	45939993	A	G	0.494	118	PASS	PCBP3	intron_variant	Brain NEPC
chr22	17581464	T	G	0.94	231	PASS	SLC25A18	intron_variant	Brain NEPC
chr22	19851751	G	A	0.954	267	PASS	RTL10	missense_variant	Brain NEPC
chr22	19915739	C	G	0.948	112	PASS	TXNRD2	intron_variant	Brain NEPC
chr22	20703127	C	T	0.18	86	PASS	TMEM191A	splice_donor_5th_base_variant&intron_variant&non_co(	Brain NEPC
chr22	20996080	T	C	0.065	593	PASS	LZTR1	synonymous_variant	Brain NEPC
chr22	22376327	T	C	0.374	353	PASS	IGLV5-45	missense_variant	Brain NEPC
chr22	22376333	G	A	0.382	353	PASS	IGLV5-45	missense_variant	Brain NEPC
chr22	25203792	A	G	0.94	241	PASS	CRYBB3	missense_variant	Brain NEPC
chr22	26622751	A	C	0.898	68	PASS	CRYBA4	intron_variant	Brain NEPC
chr22	31095586	A	G	0.914	266	PASS	SMTN	missense_variant	Brain NEPC
chr22	36935279	T	C	0.075	85	PASS	CSF2RB	intron_variant	Brain NEPC
chr22	42126611	G	C	0.034	365	PASS	CYP2D6	missense_variant	Brain NEPC
chr22	42129130	G	C	0.071	414	PASS	CYP2D6	synonymous_variant	Brain NEPC
chr22	42129950	C	A	0.106	55	PASS	CYP2D6	intron_variant	Brain NEPC
chrX	315221	C	T	0.959	324	PASS	GTPBP6	splice_region_variant&intron_variant	Brain NEPC
chrX	1191074	C	T	0.877	89	PASS	CRLF2	synonymous_variant	Brain NEPC
chrX	1191089	A	G	0.888	82	PASS	CRLF2	synonymous_variant	Brain NEPC
chrX	2859990	A	G	0.995	308	PASS	GYG2	synonymous_variant	Brain NEPC
chrX	33211248	A	C	0.979	73	PASS	DMD	intron_variant	Brain NEPC
chrX	44148818	T	C	0.836	84	PASS	EFHC2	missense_variant	Brain NEPC
chrX	49162895	C	G	0.986	115	PASS	MAGIX	missense_variant	Brain NEPC
chrX	53565023	A	G	0.992	173	PASS	HUWE1	intron_variant	Brain NEPC
chrX	73453605	G	T	0.936	157	PASS	CDX4	stop_gained	Brain NEPC
chrX	74591813	A	G	0.123	173	PASS	RLIM	missense_variant	Brain NEPC
chrX	74593081	A	G	0.99	140	PASS	RLIM	intron_variant	Brain NEPC
chrX	101656080	C	T	0.995	266	PASS	ARMCX2	synonymous_variant	Brain NEPC
chrX	103500463	T	G	0.994	171	PASS	RAB40A	missense_variant	Brain NEPC
chrX	105905385	T	C	0.899	192	PASS	NRK	intron_variant	Brain NEPC
chrX	107597779	A	T	0.913	237	PASS	FRMPD3	missense_variant	Brain NEPC
chrX	115191561	A	G	0.995	320	PASS	RBMXL3	missense_variant	Brain NEPC
chrX	150512284	C	T	0.986	95	PASS	MAMLD1	3_prime_utr_variant	Brain NEPC
chrX	152915171	C	CT	0.929	151	PASS	ZNF185	frameshift_variant	Brain NEPC
chrX	153958514	A	G	0.992	161	PASS	HCFC1	intron_variant	Brain NEPC

Supplementary Table 2a. Copy Number Variant calls when compared to primary for dura

Gene	Chromoso	Gene_start	Gene_end	Log2Ratio(DuratoPrimary)	Filter
A1BG	19	58345178	58353499	0.063781462	PASS
A1CF	10	50799409	50885675	-0.404852538	PASS
A2M	12	9067664	9116229	0.362961408	suppress_segment_too_large
A2ML1	12	8822472	8887001	0.362961408	suppress_segment_too_large
A3GALT2	1	33306766	33321098	0.033763446	PASS
A4GALT	22	42692112	42721298	-0.575930959	PASS
A4GNT	3	138123718	138132387	0.313454204	suppress_segment_too_large
AAAS	12	53307456	53324864	-0.202191512	PASS
AACS	12	125065379	125143333	0.202141228	PASS
AADAC	3	151814037	151828488	0.318278996	suppress_segment_too_large
AADACL2	3	151733916	151761339	0.318278996	suppress_segment_too_large
AADACL3	1	12716115	12728759	-0.415253068	PASS
AADACL4	1	12644547	12667086	0.003932739	PASS
AADAT	4	170060222	170091699	-0.011382299	PASS
AAGAB	15	67201033	67255195	-0.697600509	PASS
AAK1	2	69457997	69674349	-0.372959126	PASS
AAMDC	11	77821109	77918432	0.062969153	PASS
AAMP	2	218264123	218270257	0.025928102	PASS
AANAT	17	76453351	76470117	0.088516311	PASS
AAR2	20	36236459	36270918	-0.168668583	PASS
AARD	8	116938199	116944487	0.279445683	PASS
AARS	16	70252295	70289543	-0.371357732	PASS
AARS2	6	44299654	44313326	0.048820648	PASS
AARSD1	17	42950526	42964498	0.088516311	PASS
AASDH	4	56338287	56387508	-0.011382299	PASS
AASDHPP	11	106075501	106098710	-0.356188624	PASS
AASS	7	122075647	122144280	-0.388946609	PASS
AATF	17	36948875	37056871	0.060555011	PASS
AATK	17	81117295	81166077	0.088516311	PASS
ABAT	16	8674565	8784575	0.069936844	PASS
ABCA1	9	104781002	104928237	0.365190713	suppress_segment_too_large
ABCA10	17	69147214	69244846	0.088516311	PASS
ABCA12	2	214931542	215138428	0.025928102	PASS
ABCA13	7	48171458	48647496	-2.348494016	PASS
ABCA2	9	137007227	137028922	0.365190713	suppress_segment_too_large
ABCA3	16	2275881	2340746	0.069936844	PASS
ABCA4	1	93992835	94121132	0.033763446	PASS
ABCA5	17	69244311	69327244	0.088516311	PASS
ABCA6	17	69078702	69141888	0.088516311	PASS
ABCA7	19	1040101	1065572	0.079632875	PASS
ABCA8	17	68867292	68955392	0.088516311	PASS
ABCA9	17	68974488	69061064	0.088516311	PASS
ABCB1	7	87503633	87713323	-0.262778904	PASS
ABCB10	1	229516582	229558695	-0.256698369	PASS

ABCB11	2	168915498	169031324	0.039726562	PASS
ABCB4	7	87401697	87480435	-0.262778904	PASS
ABCB5	7	20615207	20777038	-0.69513118	PASS
ABCB6	2	219209766	219219017	0.025928102	PASS
ABCB7	23	75051554	75156732	-0.012567603	PASS
ABCB8	7	151028422	151047782	-0.388946609	PASS
ABCB9	12	122920951	122981649	0.202141228	PASS
ABCC1	16	15949577	16143074	0.069936844	PASS
ABCC10	6	43427366	43450430	0.048820648	PASS
ABCC11	16	48166910	48247568	0.069936844	PASS
ABCC12	16	48081006	48156018	0.069936844	PASS
ABCC2	10	99782640	99852594	-0.367947924	PASS
ABCC3	17	50634777	50692252	0.088516311	PASS
ABCC4	13	95019829	95301475	-0.390786075	PASS
ABCC5	3	183919934	184018015	0.318278996	suppress_segment_too_large
ABCC6	16	16148928	16223522	0.069936844	PASS
ABCC8	11	17392498	17476879	-0.405444717	PASS
ABCC9	12	21797401	21942529	0.362961408	suppress_segment_too_large
ABCD1	23	153724868	153744762	-0.012567603	PASS
ABCD2	12	39550033	39619751	-0.202191512	PASS
ABCD3	1	94418455	94518666	0.033763446	PASS
ABCD4	14	74285423	74303056	0.060212819	PASS
ABCE1	4	145097932	145129179	-0.011382299	PASS
ABCF1	6	30571376	30597179	0.048820648	PASS
ABCF2	7	151212490	151227230	-0.388946609	PASS
ABCF3	3	184186023	184194012	0.318278996	suppress_segment_too_large
ABCG1	21	42199689	42297244	-0.064425456	suppress_segment_too_large
ABCG2	4	88090264	88231322	-0.011382299	PASS
ABCG4	11	119149012	119162653	-0.356188624	PASS
ABCG5	2	43812472	43838865	0.040498426	PASS
ABCG8	2	43831942	43882987	0.040498426	PASS
ABHD1	2	27123789	27130812	0.040498426	PASS
ABHD10	3	111979010	111993363	0.313454204	suppress_segment_too_large
ABHD11	7	73736094	73738867	-0.262778904	PASS
ABHD12	20	25294743	25390983	-0.168668583	PASS
ABHD12B	14	50872160	50904970	0.060212819	PASS
ABHD13	13	108218379	108234255	-0.390786075	PASS
ABHD14A	3	51971426	51981199	-0.234617007	PASS
ABHD14A	3	51974706	51989183	-0.234617007	PASS
ABHD14B	3	51968510	51983409	-0.234617007	PASS
ABHD15	17	29560547	29567137	0.060555011	PASS
ABHD16A	6	31686949	31703444	0.048820648	PASS
ABHD16B	20	63861498	63862988	-0.168668583	PASS
ABHD17A	19	1876810	1885547	0.079632875	PASS
ABHD17B	9	71862452	71910931	0.365190713	suppress_segment_too_large
ABHD17C	15	80679684	80755621	-0.697600509	PASS

ABHD18	4	127965306	128039927	-0.011382299	PASS
ABHD2	15	89087459	89202360	-0.697600509	PASS
ABHD3	18	21650897	21704805	-0.376397843	PASS
ABHD4	14	22598237	22613215	0.060212819	PASS
ABHD5	3	43690108	43734371	-0.234617007	PASS
ABHD6	3	58237506	58295693	-0.234617007	PASS
ABHD8	19	17292131	17310236	0.093487731	suppress_segment_too_large
ABI1	10	26746593	26861087	0.039811807	PASS
ABI2	2	203328219	203447723	0.025928102	PASS
ABI3	17	49210227	49223225	0.088516311	PASS
ABI3BP	3	100749156	100993515	0.313454204	suppress_segment_too_large
ABL1	9	130713946	130887675	0.365190713	suppress_segment_too_large
ABL2	1	179099327	179229684	-0.027073548	PASS
ABLIM1	10	114431113	114685003	0.063874692	PASS
ABLIM2	4	7965310	8158832	0.279089364	PASS
ABLIM3	5	149141483	149260542	-0.293443042	PASS
ABO	9	133250401	133275219	0.365190713	suppress_segment_too_large
ABR	17	1003518	1229738	0.128406487	PASS
ABRA	8	106759483	106770245	0.279445683	PASS
ABRACL	6	139028682	139043302	-0.035448111	PASS
ABRAXAS	4	83459517	83523348	-0.011382299	PASS
ABRAXAS	10	124801785	124836670	0.063874692	PASS
ABT1	6	26596952	26600744	0.048820648	PASS
ABTB1	3	127672935	127680920	0.313454204	suppress_segment_too_large
ABTB2	11	34150988	34358008	-0.405444717	PASS
AC000120.	7	92112159	92179511	-0.262778904	PASS
AC000120.	7	92131774	92245924	-0.262778904	PASS
AC001226.	13	76992078	77129589	-0.390786075	PASS
AC002094.	17	28364288	28368012	0.060555011	PASS
AC002310.	16	30534227	30558279	0.069936844	PASS
AC002310.	16	30525923	30534852	0.069936844	PASS
AC002985.	19	18907013	18934397	0.093487731	suppress_segment_too_large
AC002996.	12	111753890	111791418	0.202141228	PASS
AC003002.	19	57363566	57398584	-0.180829161	PASS
AC003002.	19	57394183	57438457	-0.180829161	PASS
AC003002.	19	57389884	57419994	-0.180829161	PASS
AC003005.	19	57487718	57505381	-0.180829161	PASS
AC003006.	19	57682045	57753575	-0.180829161	PASS
AC003688.	17	7246829	7262089	0.128406487	PASS
AC004076.	19	57437970	57477536	-0.180829161	PASS
AC004080.	7	27163535	27180013	-0.69513118	PASS
AC004233.	16	2988256	3002016	0.069936844	PASS
AC004593.	7	29122340	29514667	-0.69513118	PASS
AC004687.	17	58345206	58417595	0.088516311	PASS
AC004691.	7	30852273	30923812	-0.69513118	PASS
AC004754.	16	249547	269943	0.069936844	PASS

AC004805.	17	65538102	65641033	0.088516311	PASS
AC004832.	22	30409255	30428990	-0.575930959	PASS
AC004922.	7	99325879	99394653	-0.262778904	PASS
AC004997.	22	30285238	30299482	-0.575930959	PASS
AC005020.	7	99558695	99607810	-0.262778904	PASS
AC005041.	2	74393836	74421662	-0.372959126	PASS
AC005154.	7	30497077	30550761	-0.69513118	PASS
AC005258.	19	2269525	2341172	0.079632875	PASS
AC005324.	17	15571491	15651653	-0.139031004	PASS
AC005324.	17	15699692	15744778	-0.139031004	PASS
AC005520.	14	73851971	73932278	0.060212819	PASS
AC005670.	17	46923133	47049932	0.088516311	PASS
AC005697.	17	27798806	27893365	0.060555011	PASS
AC005702.	17	59940908	59973101	0.088516311	PASS
AC005726.	17	28455752	28614197	0.060555011	PASS
AC005779.	19	45179822	45202444	0.14996833	PASS
AC005832.	12	4604944	4662643	-0.086128028	PASS
AC005833.	12	4649101	4774184	-0.086128028	PASS
AC005837.	17	76732978	76738522	0.088516311	PASS
AC005943.	19	1578339	1605445	0.079632875	PASS
AC006030.	2	74211604	74363377	-0.372959126	PASS
AC006059.	3	42705756	42804518	-0.234617007	PASS
AC006064.	12	6556886	6607367	-0.086128028	PASS
AC006254.	3	52813282	52835729	-0.234617007	PASS
AC006486.	19	42234583	42255132	0.093487731	suppress_segment_too_large
AC006518.	12	10881011	10884255	0.362961408	suppress_segment_too_large
AC006538.	19	2717769	2740048	0.079632875	PASS
AC006978.	7	30362400	30412502	-0.69513118	PASS
AC007040.	2	70939318	70995336	-0.372959126	PASS
AC007192.	19	18153158	18178117	0.093487731	suppress_segment_too_large
AC007240.	2	10122739	10199288	0.040498426	PASS
AC007326.	22	18906238	18947732	-0.575930959	PASS
AC007375.	14	77098311	77225462	-0.192197564	PASS
AC007731.	22	20429241	20446620	-0.575930959	PASS
AC007846.	17	5772926	6080792	0.128406487	PASS
AC007998.	18	35443871	35497940	-0.376397843	PASS
AC008012.	12	4273762	4405490	-0.086128028	PASS
AC008073.	2	24124366	24190436	0.040498426	PASS
AC008397.	19	18207961	18255419	0.093487731	suppress_segment_too_large
AC008403.	19	48465837	48472431	0.14996833	PASS
AC008481.	19	11466240	11505698	0.093487731	suppress_segment_too_large
AC008537.	19	40801297	40898282	0.093487731	suppress_segment_too_large
AC008575.	5	112827213	112867582	-0.293443042	PASS
AC008581.	5	77030902	77152155	-0.293443042	PASS
AC008676.	5	157341596	157460100	-0.150979337	PASS
AC008687.	19	49022953	49036895	0.14996833	PASS

AC008687.	19	49017496	49020523	0.14996833	PASS
AC008687.	19	49055793	49065076	0.14996833	PASS
AC008695.	5	131425891	131796983	-0.293443042	PASS
AC008750.	19	51350373	51368099	-0.016761391	PASS
AC008758.	19	12461559	12551474	0.093487731	suppress_segment_too_large
AC008758.	19	12379189	12401274	0.093487731	suppress_segment_too_large
AC008758.	19	12391949	12441082	0.093487731	suppress_segment_too_large
AC008758.	19	12433103	12484816	0.093487731	suppress_segment_too_large
AC008763.	19	7629796	7643048	0.079632875	PASS
AC008763.	19	7678501	7682854	0.079632875	PASS
AC008764.	19	16479067	16628204	0.093487731	suppress_segment_too_large
AC008764.	19	16577642	16660115	0.093487731	suppress_segment_too_large
AC008770.	19	12017888	12052967	0.093487731	suppress_segment_too_large
AC008770.	19	11925075	11979290	0.093487731	suppress_segment_too_large
AC008878.	19	7507052	7519622	0.079632875	PASS
AC008878.	19	7533595	7540059	0.079632875	PASS
AC008878.	19	7382834	7472477	0.079632875	PASS
AC008982.	19	38817471	38840178	0.093487731	suppress_segment_too_large
AC009086.	16	29663279	29695144	0.069936844	PASS
AC009119.	16	83908132	83951445	-0.371357732	PASS
AC009133.	16	29812261	29820092	0.069936844	PASS
AC009163.	16	75528532	75545428	-0.371357732	PASS
AC009163.	16	75412684	75464706	-0.371357732	PASS
AC009336.	2	176151085	176173097	0.039726562	PASS
AC009690.	15	72284727	72375981	-0.697600509	PASS
AC009690.	15	72266746	72319946	-0.697600509	PASS
AC009879.	8	66430185	66445324	-0.201142258	PASS
AC009879.	8	66432486	66518524	-0.201142258	PASS
AC010132.	7	42909273	42932174	-0.69513118	PASS
AC010197.	12	21536203	21657798	0.362961408	suppress_segment_too_large
AC010255.	5	122129622	122183949	-0.293443042	PASS
AC010319.	19	17436611	17460804	0.093487731	suppress_segment_too_large
AC010323.	19	8308283	8321379	0.079632875	PASS
AC010325.	19	50776141	50793142	-0.016761391	PASS
AC010326.	19	57876765	57916591	-0.180829161	PASS
AC010327.	19	55154757	55160671	0.121336758	PASS
AC010422.	19	12643831	12648397	0.093487731	suppress_segment_too_large
AC010422.	19	12664828	12669397	0.093487731	suppress_segment_too_large
AC010422.	19	12525720	12580975	0.093487731	suppress_segment_too_large
AC010422.	19	12668071	12681834	0.093487731	suppress_segment_too_large
AC010463.	19	17267418	17282966	0.093487731	suppress_segment_too_large
AC010487.	19	52880663	52962768	0.206912349	PASS
AC010522.	19	57819719	57858941	-0.180829161	PASS
AC010531.	16	87302876	87334273	-0.371357732	PASS
AC010542.	16	66469812	66517312	-0.371357732	PASS
AC010547.	16	71447600	71489311	-0.371357732	PASS

AC010615.	19	21405159	21491266	0.093487731	suppress_segment_too_large
AC010616.	19	41907705	41928516	0.093487731	suppress_segment_too_large
AC010616.	19	41956879	41994232	0.093487731	suppress_segment_too_large
AC010619.	19	49462752	49486231	0.14996833	PASS
AC010646.	19	17235940	17255448	0.093487731	suppress_segment_too_large
AC011005.	7	129126518	129130793	-0.388946609	PASS
AC011195.	17	58660424	58692018	0.088516311	PASS
AC011330.	15	43614208	43632283	-0.396255771	PASS
AC011448.	19	19516227	19536076	0.093487731	suppress_segment_too_large
AC011452.	19	49909501	49958391	-0.016761391	PASS
AC011455.	19	38915404	38949855	0.093487731	suppress_segment_too_large
AC011462.	19	41350853	41425001	0.093487731	suppress_segment_too_large
AC011473.	19	50996018	51009581	-0.016761391	PASS
AC011479.	19	38289151	38304910	0.093487731	suppress_segment_too_large
AC011498.	19	4528427	4540067	0.079632875	PASS
AC011499.	19	5784005	5785619	0.079632875	PASS
AC011511.	19	10305427	10316009	0.093487731	suppress_segment_too_large
AC011511.	19	10315471	10320678	0.093487731	suppress_segment_too_large
AC011530.	19	45779437	45785973	0.14996833	PASS
AC011604.	12	20815674	21090245	0.362961408	suppress_segment_too_large
AC012184.	16	70299194	70372582	-0.371357732	PASS
AC012213.	8	103398658	103501895	0.279445683	PASS
AC012254.	18	47108188	47176345	-0.376397843	PASS
AC012309.	19	37093019	37210512	0.093487731	suppress_segment_too_large
AC012476.	15	40695129	40732158	-0.396255771	PASS
AC012531.	12	53985845	54034888	-0.202191512	PASS
AC012651.	15	42348103	42412317	-0.396255771	PASS
AC013394.	15	92883413	92949230	-0.697600509	PASS
AC013470.	7	12469621	12542222	-0.69513118	PASS
AC013489.	15	88459501	88546585	-0.697600509	PASS
AC013717.	2	44168851	44320310	0.040498426	PASS
AC015688.	17	27623364	27640777	0.060555011	PASS
AC015802.	17	76563710	76570544	0.088516311	PASS
AC015813.	17	57989038	58007246	0.088516311	PASS
AC017083.	2	68131238	68261230	-0.372959126	PASS
AC018362.	15	42497395	42548786	-0.396255771	PASS
AC018512.	15	43772617	43799133	-0.396255771	PASS
AC018523.	11	14493783	14520344	-0.405444717	PASS
AC018554.	16	60358455	60359480	-0.371357732	PASS
AC018709.	4	186426546	186555328	-0.462744716	PASS
AC018755.	19	51612091	51646889	-0.016761391	PASS
AC019117.	7	17299295	17467234	-0.69513118	PASS
AC020636.	3	150882997	150941743	0.318278996	suppress_segment_too_large
AC020907.	19	35106510	35124297	0.093487731	suppress_segment_too_large
AC020909.	19	50415799	50428409	-0.016761391	PASS
AC020915.	19	58278966	58315197	0.063781462	PASS

AC020915.	19	58228914	58315183	0.063781462	PASS
AC020922.	19	55352301	55359516	0.121336758	PASS
AC021097.	7	151207837	151227166	-0.388946609	PASS
AC021097.	7	151207837	151210378	-0.388946609	PASS
AC021660.	3	98521133	98585498	0.313454204	suppress_segment_too_large
AC022335.	12	21047179	21176895	0.362961408	suppress_segment_too_large
AC022384.	3	10249372	10285796	0.311018244	suppress_segment_too_large
AC022400.	10	73796514	73811651	0.072405807	PASS
AC022414.	5	77086732	77427124	-0.293443042	PASS
AC022506.	12	57517712	57520480	-0.202191512	PASS
AC022826.	8	73688691	73878854	-0.201142258	PASS
AC023055.	12	55757275	55827546	-0.202191512	PASS
AC023509.	12	53506688	53625979	-0.202191512	PASS
AC024592.	19	5866171	5903787	0.079632875	PASS
AC025165.	12	57773028	57787046	-0.202191512	PASS
AC025263.	12	69801669	69855363	-0.202191512	PASS
AC025283.	16	3382113	3397745	0.069936844	PASS
AC025283.	16	3365099	3479550	0.069936844	PASS
AC026316.	3	170418868	170860380	0.318278996	suppress_segment_too_large
AC026398.	5	131807143	131994278	-0.293443042	PASS
AC026464.	16	69335276	69351730	-0.371357732	PASS
AC026464.	16	69335091	69356306	-0.371357732	PASS
AC026464.	16	69299682	69322700	-0.371357732	PASS
AC026464.	16	69328644	69334871	-0.371357732	PASS
AC026786.	12	133130984	133202369	0.270848837	PASS
AC026954.	17	7312661	7319174	0.128406487	PASS
AC027644.	7	66628958	66811189	-0.750061359	PASS
AC027796.	17	3585149	3636249	0.128406487	PASS
AC034102.	12	56101331	56109289	-0.202191512	PASS
AC036214.	8	79918860	80080775	0.279445683	PASS
AC037459.	8	22589274	22602084	-0.345962679	PASS
AC040162.	16	67929614	67936017	-0.371357732	PASS
AC046185.	17	63702845	63752097	0.088516311	PASS
AC048338.	12	122207779	122266423	0.202141228	PASS
AC055811.	17	17202649	17237185	0.024610076	PASS
AC058822.	4	53377839	54295272	-0.011382299	PASS
AC067752.	10	62520448	62672011	0.072405807	PASS
AC067968.	19	44025354	44087318	0.14996833	PASS
AC068234.	17	47253897	47344292	0.088516311	PASS
AC068533.	7	66087761	66152277	-0.750061359	PASS
AC068547.	2	151802651	151973949	-0.475215623	PASS
AC068580.	11	1734821	1763954	-0.333835828	PASS
AC068631.	3	186581995	186597103	0.318278996	suppress_segment_too_large
AC068775.	12	10412312	10436001	0.362961408	suppress_segment_too_large
AC068790.	12	123973944	124088594	0.202141228	PASS
AC068831.	15	90966190	91022566	-0.697600509	PASS

AC068896.	10	124617080	124791727	0.063874692	PASS
AC068946.	2	219075329	219170827	0.025928102	PASS
AC068946.	2	219209772	219222738	0.025928102	PASS
AC069257.	3	196248230	196318222	0.326834516	PASS
AC069368.	15	64841883	64930920	-0.697600509	PASS
AC069444.	3	119703076	119807570	0.313454204	suppress_segment_too_large
AC069503.	12	121888809	121921470	0.202141228	PASS
AC073082.	2	48632291	48755724	0.040498426	PASS
AC073111.	7	150368790	150396915	-0.388946609	PASS
AC073111.	7	150400702	150412470	-0.388946609	PASS
AC073283.	2	47065941	47176511	0.040498426	PASS
AC073508.	17	40627356	40665141	0.060555011	PASS
AC073610.	12	48921963	48939663	-0.202191512	PASS
AC073610.	12	48903503	48957365	-0.202191512	PASS
AC073612.	12	71674204	71698968	-0.202191512	PASS
AC073896.	12	56285916	56316059	-0.202191512	PASS
AC074143.	16	67163385	67165815	-0.371357732	PASS
AC078927.	12	66123917	66169985	-0.202191512	PASS
AC079447.	2	99141485	99322741	0.048238121	PASS
AC079594.	3	160227454	160449829	0.318278996	suppress_segment_too_large
AC083800.	3	119147375	119187807	0.313454204	suppress_segment_too_large
AC084337.	11	6481485	6508978	-0.405444717	PASS
AC087289.	17	75898645	75930129	0.088516311	PASS
AC087632.	15	64181180	64381440	-0.697600509	PASS
AC087721.	15	41284013	41380743	-0.396255771	PASS
AC090004.	3	14135217	14148252	0.311018244	suppress_segment_too_large
AC090227.	18	49795793	49837623	-0.376397843	PASS
AC090360.	18	80034346	80097088	-0.376397843	PASS
AC090517.	15	56652488	56918571	-0.697600509	PASS
AC090527.	15	45587351	45676314	-0.396255771	PASS
AC091057.	15	30624548	30685606	-0.396255771	PASS
AC091167.	15	90249556	90272208	-0.697600509	PASS
AC091167.	15	90266317	90314499	-0.697600509	PASS
AC091167.	15	90234200	90265462	-0.697600509	PASS
AC091551.	18	50968019	51058144	-0.376397843	PASS
AC091959.	5	146203550	146339251	-0.293443042	PASS
AC092017.	1	211082872	211188533	-0.256698369	PASS
AC092042.	3	42809446	43055871	-0.234617007	PASS
AC092073.	19	34396315	34409364	0.093487731	suppress_segment_too_large
AC092111.	12	8138140	8175320	-0.086128028	PASS
AC092143.	16	89919165	89936092	-0.371357732	PASS
AC092329.	19	23222755	23274221	0.093487731	suppress_segment_too_large
AC092442.	4	6064977	6070162	0.279089364	PASS
AC092587.	2	99154998	99195298	0.048238121	PASS
AC092647.	7	55887277	55955239	-0.750061359	PASS
AC092718.	16	81053587	81096296	-0.371357732	PASS

AC092718.	16	81022316	81096284	-0.371357732	PASS
AC092835.	2	95207535	95259774	0.048238121	PASS
AC093155.	1	86993009	87169204	0.033763446	PASS
AC093227.	19	37689865	37719188	0.093487731	suppress_segment_too_large
AC093423.	1	89633140	89933250	0.033763446	PASS
AC093512.	16	30053090	30070420	0.069936844	PASS
AC093525.	16	2513965	2527955	0.069936844	PASS
AC093525.	16	2496032	2520218	0.069936844	PASS
AC093668.	7	102541501	102592444	-0.388946609	PASS
AC093668.	7	102483344	102543764	-0.388946609	PASS
AC093668.	7	102538417	102543849	-0.388946609	PASS
AC093827.	4	86886472	86936178	-0.011382299	PASS
AC093899.	2	169479480	169525922	0.039726562	PASS
AC096887.	3	52969119	53099453	-0.234617007	PASS
AC097104.	12	56449700	56488170	-0.202191512	PASS
AC097625.	23	15561047	15675608	-0.012567603	PASS
AC097634.	3	70959226	71754229	-0.234617007	PASS
AC097636.	3	51798803	51830856	-0.234617007	PASS
AC097637.	3	52221081	52231190	-0.234617007	PASS
AC098484.	1	42767292	42794493	0.033763446	PASS
AC098582.	4	88523810	88649847	-0.011382299	PASS
AC098588.	4	144210701	145098174	-0.011382299	PASS
AC098650.	3	28349178	29767537	0.311018244	suppress_segment_too_large
AC098850.	17	16690261	16804453	0.024610076	PASS
AC099489.	16	11359845	11527245	0.069936844	PASS
AC099811.	17	42119674	42154916	0.060555011	PASS
AC099850.	17	59197566	59215226	0.088516311	PASS
AC100868.	8	132024238	132085655	0.113969953	PASS
AC104109.	5	134167170	134226071	-0.293443042	PASS
AC104151.	16	76553417	76819624	-0.371357732	PASS
AC104304.	3	46581150	46617226	-0.234617007	PASS
AC104389.	11	5254392	5678434	-0.957525224	PASS
AC104389.	11	5248270	5254783	-0.957525224	PASS
AC104452.	3	49416777	49429314	-0.234617007	PASS
AC104472.	3	155763043	155854442	0.318278996	suppress_segment_too_large
AC104532.	19	5904841	5914707	0.079632875	PASS
AC104581.	17	7925931	7930622	0.128406487	PASS
AC104662.	4	25160663	25277306	0.279089364	PASS
AC105052.	7	102582523	102642869	-0.388946609	PASS
AC105052.	7	102637049	102671641	-0.388946609	PASS
AC106774.	5	17491325	17491769	0.055007813	PASS
AC106886.	16	30704067	30745169	0.069936844	PASS
AC107871.	15	68184032	68229718	-0.697600509	PASS
AC107959.	8	23084403	23115536	-0.345962679	PASS
AC108488.	2	3531984	3547957	0.040498426	PASS
AC108941.	4	69810780	69847786	-0.011382299	PASS

AC109583.	3	46742092	46812558	-0.234617007	PASS
AC110275.	8	43018424	43077334	-0.201142258	PASS
AC112128.	3	113361901	113515151	0.313454204	suppress_segment_too_large
AC112229.	2	110402934	110473075	-0.010901494	PASS
AC112504.	3	141738474	141807919	-0.356957581	PASS
AC113348.	5	178694605	178729335	-0.150979337	PASS
AC113554.	17	63484823	63521848	0.088516311	PASS
AC114490.	1	34981533	35031741	0.033763446	PASS
AC114490.	1	34974356	34985313	0.033763446	PASS
AC115220.	7	63998874	64016130	-0.750061359	PASS
AC116366.	5	132410832	132646079	-0.293443042	PASS
AC117378.	12	56714612	56741535	-0.202191512	PASS
AC117457.	3	178419123	178843300	0.318278996	suppress_segment_too_large
AC118470.	1	247711813	247723764	-0.368111568	PASS
AC118549.	1	77562416	77683419	0.033763446	PASS
AC118553.	1	99970011	100083321	0.033763446	PASS
AC119396.	19	7348943	7383385	0.079632875	PASS
AC119674.	1	56173433	56524495	0.033763446	PASS
AC120057.	17	7240427	7244635	0.128406487	PASS
AC120114.	16	29817239	29830627	0.069936844	PASS
AC124312.	15	24955034	25000276	-0.396255771	PASS
AC126283.	4	109713916	109801999	-0.011382299	PASS
AC126755.	16	18358086	18379331	0.069936844	PASS
AC127029.	17	63917203	63931385	0.088516311	PASS
AC129492.	17	8150816	8162975	0.128406487	PASS
AC131160.	3	183815568	183884889	0.318278996	suppress_segment_too_large
AC134669.	17	31305213	31318831	0.060555011	PASS
AC134684.	8	7579644	7582653	-0.345962679	PASS
AC134980.	15	22015233	22095857	-0.750600437	PASS
AC135050.	16	31083439	31094956	0.069936844	PASS
AC135068.	15	21637909	21638992	-0.750600437	PASS
AC135068.	15	21651844	21652968	-0.750600437	PASS
AC135178.	17	8368638	8383187	0.128406487	PASS
AC136428.	16	33844784	33845229	0.069936844	PASS
AC137834.	12	57249609	57296484	-0.202191512	PASS
AC138696.	8	143247110	143276403	0.307788765	PASS
AC138811.	16	18788063	18801519	0.069936844	PASS
AC138894.	16	28456371	28492098	0.069936844	PASS
AC139491.	5	176063748	176126959	-0.150979337	PASS
AC139530.	17	81703371	81720539	0.088516311	PASS
AC140504.	16	15395754	15515348	0.069936844	PASS
AC142391.	5	139442316	139462707	-0.293443042	PASS
AC144573.	8	37934340	37965953	-0.201142258	PASS
AC233992.	8	144353228	144355609	0.307788765	PASS
AC235565.	23	102360395	102471812	-0.012567603	PASS
AC239799.	1	146113652	146229000	-0.157385092	PASS

AC243547.	1	145927258	145977811	-0.157385092	PASS
AC243756.	1	146344131	146344790	-0.157385092	PASS
AC243967.	19	41708612	41761282	0.093487731	suppress_segment_too_large
AC244197.	23	149482543	149533935	-0.012567603	PASS
AC244517.	5	141223372	141245861	-0.293443042	PASS
AC245033.	15	82536788	82573194	-0.697600509	PASS
AC245748.	19	44329695	44401608	0.14996833	PASS
AC253536.	22	23971378	23980469	-0.575930959	PASS
ACAA1	3	38103129	38137242	-0.234617007	PASS
ACAA2	18	49782167	49813960	-0.376397843	PASS
ACACA	17	37084988	37406818	0.060555011	PASS
ACACB	12	109116595	109268226	0.202141228	PASS
ACAD10	12	111686056	111757107	0.202141228	PASS
ACAD11	3	132558138	132660723	0.313454204	suppress_segment_too_large
ACAD8	11	134253495	134265855	-0.419612865	PASS
ACAD9	3	128879596	128916067	0.313454204	suppress_segment_too_large
ACADL	2	210187939	210225491	0.025928102	PASS
ACADM	1	75724347	75787575	0.033763446	PASS
ACADS	12	120725735	120740008	0.202141228	PASS
ACADSB	10	123008979	123058311	0.063874692	PASS
ACADVL	17	7217125	7225273	0.128406487	PASS
ACAN	15	88803442	88875354	-0.697600509	PASS
ACAP1	17	7336529	7351478	0.128406487	PASS
ACAP2	3	195274736	195443078	0.326834516	PASS
ACAP3	1	1292376	1309609	0.373964873	suppress_segment_too_large
ACAT1	11	108121516	108147776	-0.356188624	PASS
ACAT2	6	159760328	159779055	-0.035448111	PASS
ACBD3	1	226144679	226186730	-0.256698369	PASS
ACBD4	17	45132600	45144181	0.088516311	PASS
ACBD5	10	27195214	27242130	0.039811807	PASS
ACBD6	1	180269653	180502954	-0.027073548	PASS
ACBD7	10	15077523	15088776	0.039811807	PASS
ACCS	11	44065925	44084222	0.117376172	PASS
ACCSL	11	44047981	44059977	0.117376172	PASS
ACD	16	67657512	67660815	-0.371357732	PASS
ACE	17	63477061	63498380	0.088516311	PASS
ACE2	23	15561033	15602148	-0.012567603	PASS
ACER1	19	6306142	6333629	0.079632875	PASS
ACER2	9	19409059	19452020	-0.310444659	PASS
ACER3	11	76860867	77026797	0.062969153	PASS
ACHE	7	100889994	100896974	-0.262778904	PASS
ACIN1	14	23058564	23095614	0.060212819	PASS
ACKR1	1	159203307	159206500	-0.467855874	PASS
ACKR2	3	42804752	42887974	-0.234617007	PASS
ACKR3	2	236567787	236582358	0.025928102	PASS
ACKR4	3	132597237	132618967	0.313454204	suppress_segment_too_large

ACLY	17	41866908	41930542	0.060555011	PASS
ACMSD	2	134838547	134902034	-0.475215623	PASS
ACO1	9	32384603	32454769	-0.310444659	PASS
ACO2	22	41469125	41528989	-0.575930959	PASS
ACOD1	13	76948497	76958642	-0.390786075	PASS
ACOT1	14	73537114	73543794	0.060212819	PASS
ACOT11	1	54542257	54639192	0.033763446	PASS
ACOT12	5	81330005	81394179	-0.293443042	PASS
ACOT13	6	24667035	24705065	0.048820648	PASS
ACOT2	14	73567620	73575658	0.060212819	PASS
ACOT4	14	73591706	73596496	0.060212819	PASS
ACOT6	14	73610945	73619888	0.060212819	PASS
ACOT7	1	6264269	6394391	0.373964873	suppress_segment_too_large
ACOT8	20	45841721	45857406	-0.168668583	PASS
ACOT9	23	23702253	23766475	-0.012567603	PASS
ACOX1	17	75941507	75979363	0.088516311	PASS
ACOX2	3	58505136	58537319	-0.234617007	PASS
ACOX3	4	8366282	8440723	0.279089364	PASS
ACOXL	2	110732573	111118222	-0.010901494	PASS
ACP1	2	264140	278283	0.040498426	PASS
ACP2	11	47239302	47248906	0.117376172	PASS
ACP4	19	50790415	50795224	-0.016761391	PASS
ACP5	19	11574660	11579008	0.093487731	suppress_segment_too_large
ACP6	1	147629652	147670496	-0.157385092	PASS
ACP7	19	39083913	39111493	0.093487731	suppress_segment_too_large
ACPP	3	132317367	132368298	0.313454204	suppress_segment_too_large
ACR	22	50738196	50745334	-0.808211634	PASS
ACRBP	12	6638075	6647460	-0.086128028	PASS
ACRV1	11	125671522	125681123	-0.419612865	PASS
ACSBG1	15	78167468	78245688	-0.697600509	PASS
ACSBG2	19	6135247	6193094	0.079632875	PASS
ACSF2	17	50426158	50474845	0.088516311	PASS
ACSF3	16	89088375	89164121	-0.371357732	PASS
ACSL1	4	184755595	184826818	-0.462744716	PASS
ACSL3	2	222860934	222944639	0.025928102	PASS
ACSL4	23	109624244	109733403	-0.012567603	PASS
ACSL5	10	112374018	112428380	0.063874692	PASS
ACSL6	5	131949973	132012243	-0.293443042	PASS
ACSM1	16	20623237	20698890	0.069936844	PASS
ACSM2A	16	20451461	20487667	0.069936844	PASS
ACSM2B	16	20536226	20576427	0.069936844	PASS
ACSM3	16	20610243	20797581	0.069936844	PASS
ACSM4	12	7304284	7328724	-0.086128028	PASS
ACSM5	16	20409534	20441336	0.069936844	PASS
ACSM6	10	95194200	95228928	-0.39678585	PASS
ACSS1	20	25006230	25058980	-0.168668583	PASS

ACSS2	20	34872146	34927962	-0.168668583	PASS
ACSS3	12	80936414	81261205	0.202141228	PASS
ACTA1	1	229431245	229434098	-0.256698369	PASS
ACTA2	10	88935074	88991339	-1.64589449	suppress_large_homdel
ACTB	7	5527147	5563784	-0.69513118	PASS
ACTBL2	5	57480016	57482809	-0.293443042	PASS
ACTC1	15	34788095	34795726	-0.396255771	PASS
ACTG1	17	81509971	81523847	0.088516311	PASS
ACTG2	2	73892314	73919865	-0.372959126	PASS
ACTL10	20	33666498	33668525	-0.168668583	PASS
ACTL6A	3	179562880	179588408	0.318278996	suppress_segment_too_large
ACTL6B	7	100643097	100656461	-0.262778904	PASS
ACTL7A	9	108862266	108863759	0.365190713	suppress_segment_too_large
ACTL7B	9	108854589	108856967	0.365190713	suppress_segment_too_large
ACTL8	1	17755313	17827063	0.033763446	PASS
ACTL9	19	8697397	8698822	0.079632875	PASS
ACTN1	14	68874143	68979440	0.060212819	PASS
ACTN2	1	236686454	236764631	-0.256698369	PASS
ACTN3	11	66546395	66563329	0.182439925	suppress_segment_too_large
ACTN4	19	38647649	38731583	0.093487731	suppress_segment_too_large
ACTR10	14	58200080	58235636	0.060212819	PASS
ACTR1A	10	102461881	102502711	-0.367947924	PASS
ACTR1B	2	97655963	97664107	0.048238121	PASS
ACTR2	2	65227753	65271253	-0.372959126	PASS
ACTR3	2	113889960	113962596	-0.010901494	PASS
ACTR3B	7	152759749	152855378	-0.411415677	PASS
ACTR3C	7	150243916	150323725	-0.388946609	PASS
ACTR5	20	38748442	38772520	-0.168668583	PASS
ACTR6	12	100199122	100241865	0.202141228	PASS
ACTR8	3	53867066	53882202	-0.234617007	PASS
ACTRT1	23	128050962	128052398	-0.012567603	PASS
ACTRT2	1	3021483	3022903	0.373964873	suppress_segment_too_large
ACTRT3	3	169766921	169769895	0.318278996	suppress_segment_too_large
ACVR1	2	157736444	157875862	0.039726562	PASS
ACVR1B	12	51951667	51997078	-0.202191512	PASS
ACVR1C	2	157526767	157629005	0.039726562	PASS
ACVR2A	2	147844517	147930826	-0.475215623	PASS
ACVR2B	3	38453851	38493142	-0.234617007	PASS
ACVRL1	12	51906908	51923361	-0.202191512	PASS
ACY1	3	51983278	51989202	-0.234617007	PASS
ACY3	11	67642555	67650659	0.182439925	suppress_segment_too_large
ACYP1	14	75053221	75069483	-0.192197564	PASS
ACYP2	2	53970838	54305300	0.040498426	PASS
AD000671	19	35745678	35754519	0.093487731	suppress_segment_too_large
AD000671	19	35739252	35745432	0.093487731	suppress_segment_too_large
ADA	20	44619522	44652233	-0.168668583	PASS

ADA2	22	17178790	17258235	-0.575930959	PASS
ADAD1	4	122378966	122429802	-0.011382299	PASS
ADAD2	16	84191138	84197168	-0.371357732	PASS
ADAL	15	43330657	43354555	-0.396255771	PASS
ADAM10	15	58588807	58749978	-0.697600509	PASS
ADAM11	17	44759031	44781846	0.088516311	PASS
ADAM12	10	126012381	126388455	0.063874692	PASS
ADAM15	1	155050566	155062775	0.103015788	PASS
ADAM17	2	9488486	9556732	0.040498426	PASS
ADAM18	8	39584489	39730064	-0.201142258	PASS
ADAM19	5	157395534	157575775	-0.150979337	PASS
ADAM2	8	39743735	39838289	-0.201142258	PASS
ADAM20	14	70522358	70535015	0.060212819	PASS
ADAM21	14	70452157	70459899	0.060212819	PASS
ADAM22	7	87934143	88202889	-0.262778904	PASS
ADAM23	2	206443539	206621130	0.025928102	PASS
ADAM28	8	24294040	24359018	-0.345962679	PASS
ADAM29	4	174829668	174978180	-0.011382299	PASS
ADAM30	1	119893533	119896495	-0.157385092	PASS
ADAM32	8	39106990	39284911	-0.201142258	PASS
ADAM33	20	3667965	3682246	-0.168668583	PASS
ADAM7	8	24440930	24526970	-0.345962679	PASS
ADAM8	10	133262403	133276868	0.063874692	PASS
ADAM9	8	38996869	39105144	-0.201142258	PASS
ADAMDE(	8	24384285	24406013	-0.345962679	PASS
ADAMTS1	21	26835747	26845409	0.12860881	suppress_segment_too_large
ADAMTS1	19	8580242	8610735	0.079632875	PASS
ADAMTS1	5	33523535	33892192	0.055007813	PASS
ADAMTS1	9	133414358	133459402	0.365190713	suppress_segment_too_large
ADAMTS1	10	70672803	70762441	0.072405807	PASS
ADAMTS1	11	130448974	130476641	-0.419612865	PASS
ADAMTS1	5	5140330	5320304	0.055007813	PASS
ADAMTS1	15	99971589	100342005	-0.697600509	PASS
ADAMTS1	16	77247813	77435114	-0.371357732	PASS
ADAMTS1	5	129460265	129738683	-0.293443042	PASS
ADAMTS2	5	179110851	179345430	-0.150979337	PASS
ADAMTS2	12	43353866	43551921	-0.202191512	PASS
ADAMTS3	4	72280969	72569386	-0.011382299	PASS
ADAMTS4	1	161184308	161199056	-0.027073548	PASS
ADAMTS5	21	26917912	26966513	0.12860881	suppress_segment_too_large
ADAMTS6	5	65148736	65481920	-0.293443042	PASS
ADAMTS7	15	78759203	78811431	-0.697600509	PASS
ADAMTS8	11	130404925	130428993	-0.419612865	PASS
ADAMTS9	3	64515654	64688000	-0.234617007	PASS
ADAMTSI	9	17906563	18910950	-0.310444659	PASS
ADAMTSI	9	133532164	133575519	0.365190713	suppress_segment_too_large

ADAMTSL	15	83654086	84039842	-0.697600509	PASS
ADAMTSL	1	150549369	150560937	0.103015788	PASS
ADAMTSL	19	1505018	1513604	0.079632875	PASS
ADAP1	7	897901	955407	-0.69513118	PASS
ADAP2	17	30906344	30959322	0.060555011	PASS
ADAR	1	154582057	154628013	0.103015788	PASS
ADARB1	21	45073853	45226560	-0.064425456	suppress_segment_too_large
ADARB2	10	1177318	1737476	0.039811807	PASS
ADAT1	16	75596981	75623300	-0.371357732	PASS
ADAT2	6	143422832	143450673	-0.035448111	PASS
ADAT3	19	1905378	1913447	0.079632875	PASS
ADCK1	14	77800083	77935012	-0.192197564	PASS
ADCK2	7	140673153	140696261	-0.388946609	PASS
ADCK5	8	144373101	144393242	0.307788765	PASS
ADCY1	7	45574140	45723116	-0.69513118	PASS
ADCY10	1	167809388	167914215	-0.027073548	PASS
ADCY2	5	7396208	7830081	0.055007813	PASS
ADCY3	2	24819169	24919839	0.040498426	PASS
ADCY4	14	24318349	24335093	0.060212819	PASS
ADCY5	3	123282296	123449758	0.313454204	suppress_segment_too_large
ADCY6	12	48766194	48789037	-0.202191512	PASS
ADCY7	16	50246137	50318135	0.069936844	PASS
ADCY8	8	130780301	131042426	0.113969953	PASS
ADCY9	16	3953387	4116185	0.069936844	PASS
ADCYAP1	18	904943	912172	-0.052305056	suppress_segment_too_large
ADCYAP1	7	31052461	31111479	-0.69513118	PASS
ADD1	4	2843857	2930076	0.279089364	PASS
ADD2	2	70607618	70768225	-0.372959126	PASS
ADD3	10	109996368	110135565	0.063874692	PASS
ADGB	6	146598965	146815462	-0.035448111	PASS
ADGRA1	10	133070929	133131675	0.063874692	PASS
ADGRA2	8	37784191	37844896	-0.201142258	PASS
ADGRA3	4	22345071	22516054	0.279089364	PASS
ADGRB1	8	142449430	142545009	0.113969953	PASS
ADGRB2	1	31727117	31764893	0.033763446	PASS
ADGRB3	6	68635367	69389511	-0.368590549	PASS
ADGRD1	12	130953907	131141469	0.270848837	PASS
ADGRD2	9	124451425	124478589	0.365190713	suppress_segment_too_large
ADGRE1	19	6887566	6940459	0.079632875	PASS
ADGRE2	19	14732393	14778541	0.093487731	suppress_segment_too_large
ADGRE3	19	14619117	14690027	0.093487731	suppress_segment_too_large
ADGRE5	19	14380501	14408725	0.093487731	suppress_segment_too_large
ADGRF1	6	46997703	47042363	0.048820648	PASS
ADGRF2	6	47656436	47697797	0.048820648	PASS
ADGRF3	2	26308173	26346817	0.040498426	PASS
ADGRF4	6	47685864	47722021	0.048820648	PASS

ADGRF5	6	46852512	46954943	0.048820648	PASS
ADGRG1	16	57610652	57665580	-0.371357732	PASS
ADGRG2	23	18989309	19122637	-0.012567603	PASS
ADGRG3	16	57668187	57689378	-0.371357732	PASS
ADGRG4	23	136300963	136416888	-0.012567603	PASS
ADGRG5	16	57542421	57591681	-0.371357732	PASS
ADGRG6	6	142301854	142446266	-0.035448111	PASS
ADGRG7	3	100609589	100695479	0.313454204	suppress_segment_too_large
ADGRL1	19	14147743	14206187	0.093487731	suppress_segment_too_large
ADGRL2	1	81306160	81992436	0.033763446	PASS
ADGRL3	4	61201258	62078335	-0.011382299	PASS
ADGRL4	1	78889764	79006718	0.033763446	PASS
ADGRV1	5	90529344	91164437	-0.293443042	PASS
ADH1A	4	99276367	99291028	-0.011382299	PASS
ADH1B	4	99304964	99352760	-0.011382299	PASS
ADH1C	4	99336492	99353027	-0.011382299	PASS
ADH4	4	99123657	99157792	-0.011382299	PASS
ADH5	4	99070978	99088801	-0.011382299	PASS
ADH6	4	99202638	99219537	-0.011382299	PASS
ADH7	4	99412261	99435737	-0.011382299	PASS
ADHFE1	8	66432486	66468907	-0.201142258	PASS
ADI1	2	3497361	3519736	0.040498426	PASS
ADIG	20	38581195	38588463	-0.168668583	PASS
ADIPOQ	3	186842690	186858463	0.318278996	suppress_segment_too_large
ADIPOR1	1	202940823	202958572	-0.256698369	PASS
ADIPOR2	12	1688574	1788678	-0.086128028	PASS
ADIRF	10	86968192	86983934	-0.292453993	PASS
ADK	10	74151185	74709303	0.072405807	PASS
ADM	11	10304680	10307397	-0.405444717	PASS
ADM2	22	50481556	50486440	-0.808211634	PASS
ADM5	19	49688664	49690575	0.14996833	PASS
ADNP	20	50888916	50931421	-0.168668583	PASS
ADNP2	18	80109031	80147523	-0.376397843	PASS
ADO	10	62804857	62808483	0.072405807	PASS
ADORA1	1	203090654	203167405	-0.256698369	PASS
ADORA2A	22	24417879	24442360	-0.575930959	PASS
ADORA2E	17	15944917	15975746	0.024610076	PASS
ADORA3	1	111499429	111503760	0.033763446	PASS
ADPGK	15	72751369	72785846	-0.697600509	PASS
ADPRH	3	119579268	119589945	0.313454204	suppress_segment_too_large
ADPRHL1	13	113399610	113453524	-0.390786075	PASS
ADPRHL2	1	36088875	36093932	0.033763446	PASS
ADPRM	17	10697594	10711233	-0.139031004	PASS
ADRA1A	8	26748150	26867273	-0.345962679	PASS
ADRA1B	5	159865080	159972544	-0.150979337	PASS
ADRA1D	20	4220631	4249074	-0.168668583	PASS

ADRA2A	10	111077163	111080907	0.063874692	PASS
ADRA2B	2	96112875	96116245	0.048238121	PASS
ADRA2C	4	3766348	3768526	0.279089364	PASS
ADRB1	10	114044056	114046908	0.063874692	PASS
ADRB2	5	148825245	148828687	-0.293443042	PASS
ADRB3	8	37962991	37966965	-0.201142258	PASS
ADRM1	20	62302093	62308862	-0.168668583	PASS
ADSL	22	40346500	40390463	-0.575930959	PASS
ADSS	1	244408494	244452134	-0.368111568	PASS
ADSSL1	14	104724186	104747325	-0.192197564	PASS
ADTRP	6	11712054	11807046	0.048820648	PASS
AEBP1	7	44104361	44114562	-0.69513118	PASS
AEBP2	12	19404045	19720801	0.362961408	suppress_segment_too_large
AEN	15	88621296	88632282	-0.697600509	PASS
AES	19	3052910	3063107	0.079632875	PASS
AF241726.	23	37349330	38687674	-0.012567603	PASS
AFAP1	4	7758714	7939926	0.279089364	PASS
AFAP1L1	5	149271871	149341802	-0.293443042	PASS
AFAP1L2	10	114294824	114404756	0.063874692	PASS
AFDN	6	167826922	167972023	-0.035448111	PASS
AFF1	4	86935002	87141054	-0.011382299	PASS
AFF2	23	148500619	149000663	-0.012567603	PASS
AFF3	2	99545419	100142739	0.048238121	PASS
AFF4	5	132875379	132963634	-0.293443042	PASS
AFG1L	6	108294894	108526796	-0.368590549	PASS
AFG3L2	18	12328944	12377314	-0.052305056	suppress_segment_too_large
AFM	4	73481683	73504001	-0.011382299	PASS
AFMID	17	78187317	78207701	0.088516311	PASS
AFP	4	73431138	73456174	-0.011382299	PASS
AFTPH	2	64524305	64593005	-0.372959126	PASS
AGA	4	177430770	177442503	-0.011382299	PASS
AGAP1	2	235494089	236131800	0.025928102	PASS
AGAP2	12	57723761	57742157	-0.202191512	PASS
AGAP3	7	151085831	151144436	-0.388946609	PASS
AGAP4	10	45825594	45853875	-0.404852538	PASS
AGAP5	10	73674285	73698159	0.072405807	PASS
AGAP6	10	49982190	50010499	-0.404852538	PASS
AGAP9	10	47501854	47523638	-0.404852538	PASS
AGBL1	15	86079973	87029052	-0.697600509	PASS
AGBL2	11	47659591	47715389	0.117376172	PASS
AGBL3	7	134986508	135147963	-0.388946609	PASS
AGBL4	1	48532855	50023913	0.033763446	PASS
AGBL5	2	27042364	27070622	0.040498426	PASS
AGER	6	32180968	32184324	0.048820648	PASS
AGFG1	2	227472152	227561214	0.025928102	PASS
AGFG2	7	100539211	100568219	-0.262778904	PASS

AGGF1	5	77029251	77065234	-0.293443042	PASS
AGK	7	141551189	141655244	-0.388946609	PASS
AGL	1	99850084	99924023	0.033763446	PASS
AGMAT	1	15572353	15585110	0.033763446	PASS
AGMO	7	15200318	15562015	-0.69513118	PASS
AGO1	1	35869808	35930528	0.033763446	PASS
AGO2	8	140520156	140635619	0.113969953	PASS
AGO3	1	35930718	36072500	0.033763446	PASS
AGO4	1	35808172	35857890	0.033763446	PASS
AGPAT1	6	32168212	32178096	0.048820648	PASS
AGPAT2	9	136673143	136687423	0.365190713	suppress_segment_too_large
AGPAT3	21	43865186	43986536	-0.064425456	suppress_segment_too_large
AGPAT4	6	161129979	161274061	-0.035448111	PASS
AGPAT5	8	6708357	6759666	-0.345962679	PASS
AGPS	2	177392644	177559299	0.039726562	PASS
AGR2	7	16791811	16833433	-0.69513118	PASS
AGR3	7	16859405	16881987	-0.69513118	PASS
AGRN	1	1020123	1056118	0.373964873	suppress_segment_too_large
AGRP	16	67482571	67483813	-0.371357732	PASS
AGT	1	230702523	230714297	-0.256698369	PASS
AGTPBP1	9	85546539	85742029	0.365190713	suppress_segment_too_large
AGTR1	3	148697784	148743008	0.318278996	suppress_segment_too_large
AGTR2	23	116170722	116174972	-0.012567603	PASS
AGTRAP	1	11736084	11754802	0.373964873	suppress_segment_too_large
AGXT	2	240868479	240880502	0.025928102	PASS
AGXT2	5	34998101	35048135	0.055007813	PASS
AHCTF1	1	246839098	246931967	-0.368111568	PASS
AHCY	20	34280268	34311802	-0.168668583	PASS
AHCYL1	1	109984686	110023741	0.033763446	PASS
AHCYL2	7	129225023	129430211	-0.388946609	PASS
AHDC1	1	27534035	27604431	0.033763446	PASS
AHI1	6	135283532	135497776	-0.035448111	PASS
AHNAK	11	62433542	62556235	0.182439925	suppress_segment_too_large
AHNAK2	14	104937244	104978357	-0.192197564	PASS
AHR	7	16916359	17346152	-0.69513118	PASS
AHRR	5	304176	438291	0.055007813	PASS
AHSA1	14	77457870	77469474	-0.192197564	PASS
AHSG	3	186612923	186621318	0.318278996	suppress_segment_too_large
AHSP	16	31527864	31528803	0.069936844	PASS
AICDA	12	8602166	8612871	0.362961408	suppress_segment_too_large
AIDA	1	222668013	222713210	-0.256698369	PASS
AIF1	6	31615184	31617021	0.048820648	PASS
AIF1L	9	131096476	131123152	0.365190713	suppress_segment_too_large
AIFM1	23	130129362	130165887	-0.012567603	PASS
AIFM2	10	70098223	70132934	0.072405807	PASS
AIFM3	22	20965108	20981360	-0.575930959	PASS

AIG1	6	143060496	143340304	-0.035448111	PASS
AIM2	1	159062484	159147096	-0.467855874	PASS
AIMP1	4	106315544	106349226	-0.011382299	PASS
AIMP2	7	6009245	6023834	-0.69513118	PASS
AIP	11	67483041	67491103	0.182439925	suppress_segment_too_large
AIPL1	17	6393693	6435199	0.128406487	PASS
AIRE	21	44285838	44298648	-0.064425456	suppress_segment_too_large
AJAP1	1	4654732	4792534	0.373964873	suppress_segment_too_large
AJM1	9	136844415	136848801	0.365190713	suppress_segment_too_large
AJUBA	14	22971174	22982642	0.060212819	PASS
AK1	9	127866480	127877743	0.365190713	suppress_segment_too_large
AK2	1	33007940	33080996	0.033763446	PASS
AK3	9	4709559	4742043	-0.310444659	PASS
AK4	1	65147549	65232145	0.033763446	PASS
AK5	1	77282051	77559969	0.033763446	PASS
AK6	5	69350984	69370013	-0.293443042	PASS
AK7	14	96392111	96489427	-0.192197564	PASS
AK8	9	132725578	132878777	0.365190713	suppress_segment_too_large
AK9	6	109492856	109691217	-0.368590549	PASS
AKAIN1	18	5145285	5197503	-0.052305056	suppress_segment_too_large
AKAP1	17	57085092	57121349	0.088516311	PASS
AKAP10	17	19904302	19978343	0.024610076	PASS
AKAP11	13	42272153	42323267	-0.390786075	PASS
AKAP12	6	151239999	151358557	-0.035448111	PASS
AKAP13	15	85380571	85749358	-0.697600509	PASS
AKAP14	23	119895837	119920716	-0.012567603	PASS
AKAP17A	23	1591593	1602514	-0.012567603	PASS
AKAP2	9	110048598	110172512	0.365190713	suppress_segment_too_large
AKAP3	12	4615508	4649047	-0.086128028	PASS
AKAP4	23	50190768	50201013	-0.012567603	PASS
AKAP5	14	64465499	64474503	0.060212819	PASS
AKAP6	14	32329273	32837681	0.060212819	PASS
AKAP7	6	131135666	131283535	-0.458570262	PASS
AKAP8	19	15353385	15379798	0.093487731	suppress_segment_too_large
AKAP8L	19	15380048	15419141	0.093487731	suppress_segment_too_large
AKAP9	7	91940867	92110673	-0.262778904	PASS
AKIP1	11	8911139	8920084	-0.405444717	PASS
AKIRIN1	1	38991223	39006059	0.033763446	PASS
AKIRIN2	6	87675072	87702209	-0.368590549	PASS
AKNA	9	114334156	114394405	0.365190713	suppress_segment_too_large
AKNAD1	1	108815898	108858228	0.033763446	PASS
AKR1A1	1	45550543	45570049	0.033763446	PASS
AKR1B1	7	134442350	134459284	-0.388946609	PASS
AKR1B10	7	134527592	134541408	-0.388946609	PASS
AKR1B15	7	134549136	134579875	-0.388946609	PASS
AKR1C1	10	4963253	4983283	0.039811807	PASS

AKR1C2	10	4987400	5018031	0.039811807	PASS
AKR1C3	10	5035354	5107686	0.039811807	PASS
AKR1C4	10	5195462	5218949	0.039811807	PASS
AKR1C8P	10	5154140	5185187	0.039811807	PASS
AKR1D1	7	138002324	138117986	-0.388946609	PASS
AKR1E2	10	4786629	4848062	0.039811807	PASS
AKR7A2	1	19303965	19312146	0.033763446	PASS
AKR7A3	1	19282558	19289250	0.033763446	PASS
AKT1	14	104769349	104795751	-0.192197564	PASS
AKT1S1	19	49869033	49878459	0.14996833	PASS
AKT2	19	40230317	40285536	0.093487731	suppress_segment_too_large
AKT3	1	243488233	243851079	-0.648598641	PASS
AKTIP	16	53491040	53504411	-0.371357732	PASS
AL020996.	1	25811470	25823744	0.033763446	PASS
AL021546.	12	120438198	120460006	0.202141228	PASS
AL021997.	6	28267121	28281580	0.048820648	PASS
AL022238.	22	40346529	40410054	-0.575930959	PASS
AL022318.	22	39014363	39033276	-0.575930959	PASS
AL024498.	6	10747794	10930423	0.048820648	PASS
AL031281.	1	22025511	22101360	0.033763446	PASS
AL031315.	6	169419969	169702047	-0.035448111	PASS
AL031708.	16	1632259	1686715	0.069936844	PASS
AL031777.	6	26195595	26199293	0.048820648	PASS
AL031847.	1	6159430	6197757	0.373964873	suppress_segment_too_large
AL033529.	1	32465057	32600792	0.033763446	PASS
AL034430.	20	10413520	10431922	-0.168668583	PASS
AL034430.	20	10420546	10420737	-0.168668583	PASS
AL035078.	11	31812391	32104665	-0.405444717	PASS
AL035460.	20	2814968	2818066	-0.168668583	PASS
AL049557.	1	111722064	111755824	0.033763446	PASS
AL049629.	11	33698261	33722467	-0.405444717	PASS
AL049634.	20	1540144	1620009	-0.168668583	PASS
AL049650.	20	2467212	2508907	-0.168668583	PASS
AL049697.	6	87408012	87511634	-0.368590549	PASS
AL049779.	14	67659820	67690367	0.060212819	PASS
AL049839.	14	94592058	94624646	-0.192197564	PASS
AL049844.	6	143807092	143844617	-0.035448111	PASS
AL049844.	6	143857318	143938471	-0.035448111	PASS
AL080251.	1	18849273	18921121	0.033763446	PASS
AL096711.	6	127438406	127519001	-0.458570262	PASS
AL096814.	6	42155426	42163439	0.048820648	PASS
AL096870.	14	24332310	24337382	0.060212819	PASS
AL109811.	1	11012662	11030528	0.373964873	suppress_segment_too_large
AL109827.	20	35632340	35674544	-0.168668583	PASS
AL110118.	14	93184973	93218586	-0.192197564	PASS
AL117339.	10	38094368	38360098	0.039811807	PASS

AL117348.	1	225886696	225924278	-0.256698369	PASS
AL121594.	14	35122549	35317474	0.060212819	PASS
AL121722.	20	22560553	22584261	-0.168668583	PASS
AL121753.	20	35267885	35280043	-0.168668583	PASS
AL121758.	20	646626	675800	-0.168668583	PASS
AL121845.	20	63738270	63740398	-0.168668583	PASS
AL121845.	20	63708864	63739103	-0.168668583	PASS
AL121900.	20	18567453	18744216	-0.168668583	PASS
AL132671.	6	117318211	117573571	-0.368590549	PASS
AL132780.	14	22946270	22982258	0.060212819	PASS
AL133352.	10	100505628	100529881	-0.367947924	PASS
AL133353.	10	99651595	99732112	-0.367947924	PASS
AL133410.	9	35812960	35828732	-0.310444659	PASS
AL133500.	23	72301691	72572843	-0.012567603	PASS
AL135905.	6	63572012	63583587	0.048820648	PASS
AL136295.	14	24189157	24213473	0.060212819	PASS
AL136295.	14	24120956	24131829	0.060212819	PASS
AL136295.	14	24180395	24190416	0.060212819	PASS
AL136295.	14	24151218	24167402	0.060212819	PASS
AL136373.	1	46532166	46543969	0.033763446	PASS
AL136454.	1	192716132	192716653	-0.027073548	PASS
AL136531.	20	1317571	1393096	-0.168668583	PASS
AL138752.	9	37588413	38068687	-0.310444659	PASS
AL139011.	1	160216800	160285130	-0.027073548	PASS
AL139142.	1	173596060	173669837	-0.027073548	PASS
AL139300.	14	103562962	103685924	-0.192197564	PASS
AL139353.	14	31334312	31457441	0.060212819	PASS
AL157392.	10	13610047	13655929	0.039811807	PASS
AL157935.	9	127867850	127906022	0.365190713	suppress_segment_too_large
AL159163.	6	166858094	166956124	-0.035448111	PASS
AL160269.	9	96235331	96352058	0.365190713	suppress_segment_too_large
AL160272.	9	117704157	117877478	0.365190713	suppress_segment_too_large
AL162231.	9	34664163	34666112	-0.310444659	PASS
AL162231.	9	34646645	34657113	-0.310444659	PASS
AL162377.	13	51763951	51845159	-0.390786075	PASS
AL162417.	9	133098121	133163914	0.365190713	suppress_segment_too_large
AL163195.	14	20582892	20609795	0.060212819	PASS
AL163636.	14	20684587	20700576	0.060212819	PASS
AL353588.	6	44273194	44378957	0.048820648	PASS
AL355102.	14	96204844	96263929	-0.192197564	PASS
AL355312.	6	149817937	149896011	-0.035448111	PASS
AL355315.	10	97584374	97673910	-0.367947924	PASS
AL355916.	14	61529128	61657964	0.060212819	PASS
AL355987.	9	136738167	136748453	0.365190713	suppress_segment_too_large
AL355987.	9	136791379	136800595	0.365190713	suppress_segment_too_large
AL357673.	1	54132686	54200073	0.033763446	PASS

AL358075.	1	46043661	46176488	0.033763446	PASS
AL358113.	9	69035750	69255187	-0.310444659	PASS
AL358472.	1	153975850	153986246	0.103015788	PASS
AL358472.	1	153959151	153975887	0.103015788	PASS
AL359736.	13	23979700	24321031	-0.390786075	PASS
AL359922.	9	21802636	22032986	-0.310444659	PASS
AL360181.	10	133390834	133420495	0.063874692	PASS
AL365205.	6	41780349	41790141	0.048820648	PASS
AL365214.	6	112236806	112350094	-0.368590549	PASS
AL365232.	6	73209746	73263196	-0.368590549	PASS
AL365273.	10	95860548	95924844	-0.39678585	PASS
AL391650.	1	26169516	26171831	0.033763446	PASS
AL445238.	13	63832361	63833531	-0.390786075	PASS
AL445685.	1	52020153	52033350	0.033763446	PASS
AL445989.	13	63746741	63751080	-0.390786075	PASS
AL449266.	1	108837660	108963484	0.033763446	PASS
AL451062.	1	43650149	43931014	0.033763446	PASS
AL512428.	6	24797373	24809798	0.048820648	PASS
AL512506.	13	43883800	44028526	-0.390786075	PASS
AL512785.	1	162365407	162383531	-0.027073548	PASS
AL513165.	9	37512547	37592469	-0.310444659	PASS
AL583836.	10	94688154	94853073	-0.39678585	PASS
AL589666.	6	85557978	85615234	-0.368590549	PASS
AL590132.	1	210858125	211134146	-0.256698369	PASS
AL590764.	23	71103987	71111575	-0.012567603	PASS
AL591806.	1	160997957	161038962	-0.027073548	PASS
AL592490.	13	61409858	61427849	-0.390786075	PASS
AL603832.	1	112702614	112711433	0.033763446	PASS
AL645922.	6	31927698	31952048	0.048820648	PASS
AL645941.	6	32937364	32953122	0.048820648	PASS
AL662820.	6	33321386	33329286	0.048820648	PASS
AL662884.	6	32150495	32154373	0.048820648	PASS
AL662899.	6	31686962	31714072	0.048820648	PASS
AL662899.	6	31666102	31673546	0.048820648	PASS
AL669918.	6	32813767	32838822	0.048820648	PASS
AL672043.	1	46175486	46176478	0.033763446	PASS
AL672142.	9	128941478	128957021	0.365190713	suppress_segment_too_large
AL691442.	1	155063748	155086807	0.103015788	PASS
AL713999.	1	155169409	155187272	0.103015788	PASS
AL772284.	23	119073226	119076373	-0.012567603	PASS
AL807752.	9	137031241	137040436	0.365190713	suppress_segment_too_large
AL807752.	9	136977518	136985435	0.365190713	suppress_segment_too_large
AL845331.	2	94588522	94604573	-0.340781246	PASS
AL928654.	14	105487199	105492267	-0.192197564	PASS
AL929554.	9	137168854	137171984	0.365190713	suppress_segment_too_large
ALAD	9	113386317	113401333	0.365190713	suppress_segment_too_large

ALAS1	3	52198086	52214327	-0.234617007	PASS
ALAS2	23	55009055	55031064	-0.012567603	PASS
ALB	4	73397114	73421412	-0.011382299	PASS
ALCAM	3	105366909	105576900	0.313454204	suppress_segment_too_large
ALDH16A	19	49453169	49471048	0.14996833	PASS
ALDH18A	10	95605929	95656706	-0.39678585	PASS
ALDH1A1	9	72900662	73080442	0.365190713	suppress_segment_too_large
ALDH1A2	15	57953424	58497866	-0.697600509	PASS
ALDH1A3	15	100877714	100916626	-0.697600509	PASS
ALDH1B1	9	38392664	38398661	-0.310444659	PASS
ALDH1L1	3	126103562	126197994	0.313454204	suppress_segment_too_large
ALDH1L2	12	105019784	105084577	0.202141228	PASS
ALDH2	12	111766887	111817529	0.202141228	PASS
ALDH3A1	17	19737984	19748943	0.024610076	PASS
ALDH3A2	17	19648136	19677598	0.024610076	PASS
ALDH3B1	11	68008578	68029282	0.182439925	suppress_segment_too_large
ALDH3B2	11	67662162	67681200	0.182439925	suppress_segment_too_large
ALDH4A1	1	18871430	18902781	0.033763446	PASS
ALDH5A1	6	24494852	24537207	0.048820648	PASS
ALDH6A1	14	74056850	74084493	0.060212819	PASS
ALDH7A1	5	126531200	126595418	-0.293443042	PASS
ALDH8A1	6	134917390	134950122	-0.035448111	PASS
ALDH9A1	1	165662216	165698863	-0.027073548	PASS
ALDOA	16	30064164	30070457	0.069936844	PASS
ALDOB	9	101420578	101449664	0.365190713	suppress_segment_too_large
ALDOC	17	28573115	28577264	0.060555011	PASS
ALG1	16	5033923	5087379	0.069936844	PASS
ALG10	12	34022281	34029694	0.362961408	suppress_segment_too_large
ALG10B	12	38316578	38329728	0.362961408	suppress_segment_too_large
ALG11	13	52012398	52032171	-0.390786075	PASS
ALG12	22	49900229	49918458	-0.808211634	PASS
ALG13	23	111665811	111760649	-0.012567603	PASS
ALG14	1	94974407	95072945	0.033763446	PASS
ALG1L	3	125929275	125937039	0.313454204	suppress_segment_too_large
ALG1L2	3	130081831	130113227	0.313454204	suppress_segment_too_large
ALG2	9	99216426	99221956	0.365190713	suppress_segment_too_large
ALG3	3	184242301	184249548	0.318278996	suppress_segment_too_large
ALG5	13	36949775	37000261	-0.390786075	PASS
ALG6	1	63367575	63438562	0.033763446	PASS
ALG8	11	78100936	78139660	0.062969153	PASS
ALG9	11	111782195	111871581	-0.356188624	PASS
ALK	2	29192774	29921566	0.040498426	PASS
ALKAL1	8	52534037	52565507	-0.201142258	PASS
ALKAL2	2	279558	288851	0.040498426	PASS
ALKBH1	14	77672404	77708020	-0.192197564	PASS
ALKBH2	12	109088188	109093631	0.202141228	PASS

ALKBH3	11	43880811	43920266	0.117376172	PASS
ALKBH4	7	102456238	102464876	-0.388946609	PASS
ALKBH5	17	18183078	18209954	0.024610076	PASS
ALKBH6	19	36009120	36014239	0.093487731	suppress_segment_too_large
ALKBH7	19	6372433	6375251	0.079632875	PASS
ALKBH8	11	107502726	107565746	-0.356188624	PASS
ALLC	2	3658195	3702671	0.040498426	PASS
ALMS1	2	73385758	73610793	-0.372959126	PASS
ALOX12	17	6996065	7010736	0.128406487	PASS
ALOX12B	17	8072636	8087716	0.128406487	PASS
ALOX15	17	4630902	4642294	0.128406487	PASS
ALOX15B	17	8039017	8049134	0.128406487	PASS
ALOX5	10	45374176	45446119	-0.404852538	PASS
ALOX5AP	13	30713478	30764426	-0.390786075	PASS
ALOXE3	17	8095900	8119047	0.128406487	PASS
ALPG	2	232406843	232410714	0.025928102	PASS
ALPI	2	232456123	232460032	0.025928102	PASS
ALPK1	4	112285509	112442620	-0.011382299	PASS
ALPK2	18	58481247	58628957	-0.376397843	PASS
ALPK3	15	84816680	84873482	-0.697600509	PASS
ALPL	1	21509372	21578412	0.033763446	PASS
ALPP	2	232378534	232382889	0.025928102	PASS
ALS2	2	201700554	201781189	0.025928102	PASS
ALS2CL	3	46668997	46693704	-0.234617007	PASS
ALS2CR12	2	201288271	201357398	0.025928102	PASS
ALX1	12	85280107	85301784	0.202141228	PASS
ALX3	1	110059994	110070700	0.033763446	PASS
ALX4	11	44260440	44310166	0.117376172	PASS
ALYREF	17	81887844	81891586	0.088516311	PASS
AMACR	5	33986178	34008108	0.055007813	PASS
AMBN	4	70592256	70607288	-0.011382299	PASS
AMBP	9	114060127	114078472	0.365190713	suppress_segment_too_large
AMBRA1	11	46396414	46594125	0.117376172	PASS
AMD1	6	110874770	110895713	-0.368590549	PASS
AMDHD1	12	95943293	95968716	0.202141228	PASS
AMDHD2	16	2520357	2531422	0.069936844	PASS
AMELX	23	11293413	11300761	-0.012567603	PASS
AMER1	23	64185117	64205744	-0.012567603	PASS
AMER2	13	25161684	25172288	-0.390786075	PASS
AMER3	2	130755435	130768134	-0.475215623	PASS
AMFR	16	56361452	56425538	-0.371357732	PASS
AMH	19	2249309	2252073	0.079632875	PASS
AMHR2	12	53423855	53431534	-0.202191512	PASS
AMIGO1	1	109504175	109509738	0.033763446	PASS
AMIGO2	12	47075707	47079951	-0.202191512	PASS
AMIGO3	3	49716834	49719695	-0.234617007	PASS

AMMECR	23	110194186	110440233	-0.012567603	PASS
AMMECR	2	127861630	127885922	-0.475215623	PASS
AMN	14	102922656	102933596	-0.192197564	PASS
AMN1	12	31671137	31729174	0.362961408	suppress_segment_too_large
AMOT	23	112774503	112840815	-0.012567603	PASS
AMOTL1	11	94706431	94876753	0.062969153	PASS
AMOTL2	3	134355874	134375479	0.313454204	suppress_segment_too_large
AMPD1	1	114673090	114695618	0.033763446	PASS
AMPD2	1	109616104	109632051	0.033763446	PASS
AMPD3	11	10308313	10507579	-0.405444717	PASS
AMPH	7	38383704	38631567	-0.69513118	PASS
AMT	3	49416775	49422753	-0.234617007	PASS
AMTN	4	70518540	70532742	-0.011382299	PASS
AMY1A	1	103655290	103664554	0.033763446	PASS
AMY1B	1	103687415	103696680	0.033763446	PASS
AMY1C	1	103750406	103758690	0.033763446	PASS
AMY2A	1	103616811	103625780	0.033763446	PASS
AMY2B	1	103553815	103579534	0.033763446	PASS
AMZ1	7	2679522	2775500	-0.69513118	PASS
AMZ2	17	68247574	68257164	0.088516311	PASS
ANAPC1	2	111611639	111884690	-0.010901494	PASS
ANAPC10	4	144831908	145098541	-0.011382299	PASS
ANAPC11	17	81890790	81900991	0.088516311	PASS
ANAPC13	3	134477706	134486716	0.313454204	suppress_segment_too_large
ANAPC15	11	72106378	72112780	0.062969153	PASS
ANAPC16	10	72216000	72235860	0.072405807	PASS
ANAPC2	9	137174784	137188549	0.365190713	suppress_segment_too_large
ANAPC4	4	25377213	25418498	0.279089364	PASS
ANAPC5	12	121308245	121399896	0.202141228	PASS
ANAPC7	12	110372900	110403730	0.202141228	PASS
ANG	14	20684177	20698971	0.060212819	PASS
ANGEL1	14	76786178	76826246	-0.192197564	PASS
ANGEL2	1	212992182	213015826	-0.256698369	PASS
ANGPT1	8	107249482	107498055	0.279445683	PASS
ANGPT2	8	6499651	6563409	-0.345962679	PASS
ANGPT4	20	869899	916317	-0.168668583	PASS
ANGPTL1	1	178849705	178871052	-0.027073548	PASS
ANGPTL2	9	127087332	127122883	0.365190713	suppress_segment_too_large
ANGPTL3	1	62597487	62606159	0.033763446	PASS
ANGPTL4	19	8363289	8374373	0.079632875	PASS
ANGPTL5	11	101890674	101916522	0.062969153	PASS
ANGPTL6	19	10092338	10102796	0.093487731	suppress_segment_too_large
ANGPTL7	1	11189341	11195981	0.373964873	suppress_segment_too_large
ANGPTL8	19	11237450	11241943	0.093487731	suppress_segment_too_large
ANHX	12	133218312	133236095	0.270848837	PASS
ANK1	8	41653220	41896762	-0.201142258	PASS

ANK2	4	112818109	113383740	-0.011382299	PASS
ANK3	10	60026298	60733490	0.072405807	PASS
ANKAR	2	189674290	189761193	0.025928102	PASS
ANKDD1A	15	64911902	64958700	-0.697600509	PASS
ANKDD1E	5	75611459	75671846	-0.293443042	PASS
ANKEF1	20	9986126	10058303	-0.168668583	PASS
ANKFN1	17	55882301	56511659	0.088516311	PASS
ANKFY1	17	4163907	4263995	0.128406487	PASS
ANKH	5	14704800	14871785	0.055007813	PASS
ANKHD1	5	140401814	140539856	-0.293443042	PASS
ANKHD1-]	5	140401908	140549569	-0.293443042	PASS
ANKIB1	7	92246234	92401384	-0.262778904	PASS
ANKK1	11	113387791	113400418	-0.356188624	PASS
ANKLE1	19	17281645	17287646	0.093487731	suppress_segment_too_large
ANKLE2	12	132725503	132761888	0.270848837	PASS
ANKMY1	2	240479422	240569209	0.025928102	PASS
ANKMY2	7	16599776	16645817	-0.69513118	PASS
ANKRA2	5	73552335	73565686	-0.293443042	PASS
ANKRD1	10	90912096	90921276	-0.39678585	PASS
ANKRD10	13	110878540	110915069	-0.390786075	PASS
ANKRD11	16	89267619	89490561	-0.371357732	PASS
ANKRD12	18	9136228	9285985	-0.052305056	suppress_segment_too_large
ANKRD13	12	109999186	110039763	0.202141228	PASS
ANKRD13	17	29589769	29614761	0.060555011	PASS
ANKRD13	1	70260588	70354734	0.033763446	PASS
ANKRD13	11	67288547	67302485	0.182439925	suppress_segment_too_large
ANKRD16	10	5861617	5889906	0.039811807	PASS
ANKRD17	4	73073376	73258798	-0.011382299	PASS
ANKRD18	9	38571358	38620660	-0.310444659	PASS
ANKRD18	9	33524394	33573009	-0.310444659	PASS
ANKRD2	10	97572499	97583884	-0.367947924	PASS
ANKRD20	9	67859147	67902094	-0.310444659	PASS
ANKRD20	9	40223285	40266392	-0.310444659	PASS
ANKRD20	9	66106815	66153135	-0.310444659	PASS
ANKRD20	9	64369394	64413142	-0.310444659	PASS
ANKRD22	10	88822132	88851818	-1.64589449	suppress_large_homdel
ANKRD23	2	96824526	96857934	0.048238121	PASS
ANKRD24	19	4183354	4224814	0.079632875	PASS
ANKRD26	10	26991914	27100498	0.039811807	PASS
ANKRD27	19	32597007	32676597	0.093487731	suppress_segment_too_large
ANKRD28	3	15667236	15859771	0.311018244	suppress_segment_too_large
ANKRD29	18	23598926	23662885	-0.376397843	PASS
ANKRD30	10	37125788	37384111	0.039811807	PASS
ANKRD30	18	14728272	14852738	-0.052305056	suppress_segment_too_large
ANKRD30	2	132147591	132257969	-0.475215623	PASS
ANKRD31	5	75068275	75236878	-0.293443042	PASS

ANKRD33	12	51887960	51891664	-0.202191512	PASS
ANKRD33	5	10564330	10657816	0.055007813	PASS
ANKRD34	1	145959442	145964582	-0.157385092	PASS
ANKRD34	5	80556755	80570488	-0.293443042	PASS
ANKRD34	15	79293285	79298235	-0.697600509	PASS
ANKRD35	1	145866560	145885866	-0.157385092	PASS
ANKRD36	2	97113496	97250199	0.048238121	PASS
ANKRD36	2	97492663	97589965	0.048238121	PASS
ANKRD36	2	95836919	95991831	0.048238121	PASS
ANKRD37	4	185396021	185400628	-0.462744716	PASS
ANKRD39	2	96836611	96858095	0.048238121	PASS
ANKRD40	17	50693190	50707924	0.088516311	PASS
ANKRD42	11	83193739	83260694	0.062969153	PASS
ANKRD44	2	196967017	197311173	0.025928102	PASS
ANKRD45	1	173609561	173669862	-0.027073548	PASS
ANKRD46	8	100509752	100559784	0.279445683	PASS
ANKRD49	11	94493629	94499583	0.062969153	PASS
ANKRD50	4	124664052	124712732	-0.011382299	PASS
ANKRD52	12	56237807	56258391	-0.202191512	PASS
ANKRD53	2	70978380	70985499	-0.372959126	PASS
ANKRD54	22	37830855	37849327	-0.575930959	PASS
ANKRD55	5	56099678	56233359	-0.293443042	PASS
ANKRD6	6	89433170	89633834	-0.368590549	PASS
ANKRD60	20	58218495	58228653	-0.168668583	PASS
ANKRD61	7	6031376	6036386	-0.69513118	PASS
ANKRD62	18	12093853	12129749	-0.052305056	suppress_segment_too_large
ANKRD63	15	40281444	40282586	-0.396255771	PASS
ANKRD65	1	1418420	1421769	0.373964873	suppress_segment_too_large
ANKRD66	6	46746917	46759506	0.048820648	PASS
ANKRD7	7	118214669	118496171	-0.388946609	PASS
ANKRD9	14	102501760	102509799	-0.192197564	PASS
ANKS1A	6	34889265	35091413	0.048820648	PASS
ANKS1B	12	98726457	99984654	0.202141228	PASS
ANKS3	16	4696510	4734378	0.069936844	PASS
ANKS4B	16	21233665	21253845	0.069936844	PASS
ANKS6	9	98731329	98796965	0.365190713	suppress_segment_too_large
ANKUB1	3	149761100	149968385	0.318278996	suppress_segment_too_large
ANKZF1	2	219229757	219236669	0.025928102	PASS
ANLN	7	36389806	36453791	-0.69513118	PASS
ANO1	11	70078302	70189528	0.182439925	suppress_segment_too_large
ANO10	3	43354859	43691594	-0.234617007	PASS
ANO2	12	5531869	5946232	-0.086128028	PASS
ANO3	11	26309599	26663288	-0.405444717	PASS
ANO4	12	100717526	101128641	0.202141228	PASS
ANO5	11	21799934	22283357	-0.405444717	PASS
ANO6	12	45215987	45440404	-0.202191512	PASS

ANO7	2	241188509	241225377	0.025928102	PASS
ANO8	19	17323223	17334829	0.093487731	suppress_segment_too_large
ANO9	11	417933	442011	-0.333835828	PASS
ANOS1	23	8528874	8732186	-0.012567603	PASS
ANP32A	15	68778535	68820897	-0.697600509	PASS
ANP32B	9	97983361	98015943	0.365190713	suppress_segment_too_large
ANP32D	12	48472665	48473624	-0.202191512	PASS
ANP32E	1	150218417	150236156	0.103015788	PASS
ANPEP	15	89784889	89815401	-0.697600509	PASS
ANTXR1	2	69013178	69249327	-0.372959126	PASS
ANTXR2	4	79901149	80125454	-0.011382299	PASS
ANTXRL	10	46286345	46330207	-0.404852538	PASS
ANXA1	9	73151757	73170393	0.365190713	suppress_segment_too_large
ANXA10	4	168092515	168187690	-0.011382299	PASS
ANXA11	10	80150889	80205572	0.072405807	PASS
ANXA13	8	123680794	123737407	0.279445683	PASS
ANXA2	15	60347134	60402883	-0.697600509	PASS
ANXA2R	5	43039081	43043170	-0.293443042	PASS
ANXA3	4	78551519	78610451	-0.011382299	PASS
ANXA4	2	69644425	69827100	-0.372959126	PASS
ANXA5	4	121667955	121697113	-0.011382299	PASS
ANXA6	5	151100712	151157882	-0.293443042	PASS
ANXA7	10	73375101	73414076	0.072405807	PASS
ANXA8	10	47460162	47484158	-0.404852538	PASS
ANXA8L1	10	46375627	46391784	-0.404852538	PASS
ANXA9	1	150982017	150995634	0.103015788	PASS
AOAH	7	36512949	36724549	-0.69513118	PASS
AOC1	7	150824627	150861504	-0.388946609	PASS
AOC2	17	42844600	42850707	0.088516311	PASS
AOC3	17	42851184	42858130	0.088516311	PASS
AOX1	2	200585868	200677064	0.025928102	PASS
AP000275.	21	32578822	32612281	0.12860881	suppress_segment_too_large
AP000295.	21	33229901	33297160	0.12860881	suppress_segment_too_large
AP000311.	21	33584687	33931607	0.12860881	suppress_segment_too_large
AP000350.	22	23862188	23895223	-0.575930959	PASS
AP000646.	11	121024125	121113108	-0.356188624	PASS
AP000721.	11	63974620	63988346	0.182439925	suppress_segment_too_large
AP000781.	11	57387365	57424059	-0.129266491	PASS
AP000812.	11	72105924	72109329	0.062969153	PASS
AP000812.	11	72080777	72110782	0.062969153	PASS
AP000944.	11	65261928	65326543	0.182439925	suppress_segment_too_large
AP001267.	11	118401602	118482737	-0.356188624	PASS
AP001273.	11	93741664	93812378	0.062969153	PASS
AP001458.	11	62649694	62665178	0.182439925	suppress_segment_too_large
AP001781.	11	111786286	111879425	-0.356188624	PASS
AP001931.	11	57742163	57793243	-0.129266491	PASS

AP002373.	11	114400682	114443932	-0.356188624	PASS
AP002495.	11	71865509	71928654	0.062969153	PASS
AP002512.	11	56459221	56473352	-0.129266491	PASS
AP002748.	11	66509079	66533613	0.182439925	suppress_segment_too_large
AP002884.	11	112086903	112193805	-0.356188624	PASS
AP002990.	11	62559603	62591531	0.182439925	suppress_segment_too_large
AP003108.	11	61429220	61485822	0.182439925	suppress_segment_too_large
AP003419.	11	67351572	67373584	0.182439925	suppress_segment_too_large
AP1AR	4	112231737	112270047	-0.011382299	PASS
AP1B1	22	29327680	29423179	-0.575930959	PASS
AP1G1	16	71729000	71809201	-0.371357732	PASS
AP1G2	14	23559565	23568070	0.060212819	PASS
AP1M1	19	16197578	16245907	0.093487731	suppress_segment_too_large
AP1M2	19	10572671	10587315	0.093487731	suppress_segment_too_large
AP1S1	7	101154400	101161596	-0.388946609	PASS
AP1S2	23	15825806	15854931	-0.012567603	PASS
AP1S3	2	223751686	223838027	0.025928102	PASS
AP2A1	19	49766968	49807113	0.14996833	PASS
AP2A2	11	924894	1012245	-0.333835828	PASS
AP2B1	17	35578046	35726409	0.060555011	PASS
AP2M1	3	184174689	184184091	0.318278996	suppress_segment_too_large
AP2S1	19	46838136	46850992	0.14996833	PASS
AP3B1	5	78000525	78294755	-0.293443042	PASS
AP3B2	15	82659281	82709914	-0.697600509	PASS
AP3D1	19	2100988	2164468	0.079632875	PASS
AP3M1	10	74120255	74151063	0.072405807	PASS
AP3M2	8	42152946	42171673	-0.201142258	PASS
AP3S1	5	115841481	115914081	-0.293443042	PASS
AP3S2	15	89830599	89894638	-0.697600509	PASS
AP4B1	1	113894748	113905201	0.033763446	PASS
AP4E1	15	50908672	51005900	-0.396255771	PASS
AP4M1	7	100101549	100110345	-0.262778904	PASS
AP4S1	14	31025106	31096450	0.060212819	PASS
AP5B1	11	65775893	65780802	0.182439925	suppress_segment_too_large
AP5M1	14	57268909	57298742	0.060212819	PASS
AP5S1	20	3820524	3828837	-0.168668583	PASS
AP5Z1	7	4775615	4794395	-0.69513118	PASS
APAF1	12	98645141	98735433	0.202141228	PASS
APBA1	9	69427530	69672306	0.365190713	suppress_segment_too_large
APBA2	15	28884483	29118315	-0.396255771	PASS
APBA3	19	3750819	3761699	0.079632875	PASS
APBB1	11	6395124	6419414	-0.405444717	PASS
APBB1IP	10	26438203	26567803	0.039811807	PASS
APBB2	4	40810027	41216714	-0.011382299	PASS
APBB3	5	140558268	140564781	-0.293443042	PASS
APC	5	112707498	112846239	-0.293443042	PASS

APC2	19	1446302	1473244	0.079632875	PASS
APCDD1	18	10454628	10489948	-0.052305056	suppress_segment_too_large
APCDD1L	20	58459101	58515131	-0.168668583	PASS
APCS	1	159587825	159588865	-0.467855874	PASS
APEH	3	49674002	49683963	-0.234617007	PASS
APELA	4	164877004	164898965	-0.011382299	PASS
APEX1	14	20455191	20457772	0.060212819	PASS
APEX2	23	55000357	55009057	-0.012567603	PASS
APH1A	1	150265399	150269580	0.103015788	PASS
APH1B	15	63276018	63309126	-0.697600509	PASS
API5	11	43311963	43344529	0.117376172	PASS
APIP	11	34853094	34916499	-0.405444717	PASS
APLF	2	68467561	68655862	-0.372959126	PASS
APLN	23	129645259	129654937	-0.012567603	PASS
APLNR	11	57233577	57237314	-0.129266491	PASS
APLP1	19	35867899	35879791	0.093487731	suppress_segment_too_large
APLP2	11	130068147	130144811	-0.419612865	PASS
APMAP	20	24962925	24992979	-0.168668583	PASS
APOA1	11	116835751	116837950	-0.356188624	PASS
APOA2	1	161222292	161223631	-0.027073548	PASS
APOA4	11	116820700	116823306	-0.356188624	PASS
APOA5	11	116789367	116792420	-0.356188624	PASS
APOB	2	21001429	21044073	0.040498426	PASS
APOBEC1	12	7649400	7665903	-0.086128028	PASS
APOBEC2	6	41053304	41064511	0.048820648	PASS
APOBEC3.	22	38952741	38992778	-0.575930959	PASS
APOBEC3.	22	38982347	38992804	-0.575930959	PASS
APOBEC3(	22	39014083	39020352	-0.575930959	PASS
APOBEC3.	22	39021113	39033276	-0.575930959	PASS
APOBEC3.	22	39040604	39053910	-0.575930959	PASS
APOBEC3(	22	39077005	39087743	-0.575930959	PASS
APOBEC3.	22	39097224	39104067	-0.575930959	PASS
APOBEC4	1	183646404	183653316	-0.027073548	PASS
APOBR	16	28494649	28498970	0.069936844	PASS
APOC1	19	44914247	44919349	0.14996833	PASS
APOC2	19	44945982	44949565	0.14996833	PASS
APOC3	11	116829706	116833072	-0.356188624	PASS
APOC4	19	44942238	44945496	0.14996833	PASS
APOC4-AI	19	44942238	44949565	0.14996833	PASS
APOD	3	195568702	195584205	0.326834516	PASS
APOE	19	44905754	44909393	0.14996833	PASS
APOF	12	56360569	56362823	-0.202191512	PASS
APOH	17	66212033	66256525	0.088516311	PASS
APOL1	22	36253010	36267530	-0.575930959	PASS
APOL2	22	36226203	36239954	-0.575930959	PASS
APOL3	22	36140330	36166177	-0.575930959	PASS

APOL4	22	36189124	36204840	-0.575930959	PASS
APOL5	22	35717872	35729483	-0.575930959	PASS
APOL6	22	35648395	35668409	-0.575930959	PASS
APOLD1	12	12725917	12829975	0.362961408	suppress_segment_too_large
APOM	6	31652416	31658210	0.048820648	PASS
APOO	23	23833353	23907934	-0.012567603	PASS
APOOL	23	85003826	85093316	-0.012567603	PASS
APOPT1	14	103562962	103607523	-0.192197564	PASS
APP	21	25880550	26171128	0.12860881	suppress_segment_too_large
APPBP2	17	60443149	60526219	0.088516311	PASS
APPL1	3	57227726	57278105	-0.234617007	PASS
APPL2	12	105173296	105236238	0.202141228	PASS
APRT	16	88809339	88811944	-0.371357732	PASS
APTX	9	32972606	33025168	-0.310444659	PASS
AQP1	7	30911694	30925516	-0.69513118	PASS
AQP10	1	154321090	154325325	0.103015788	PASS
AQP11	11	77589391	77610355	0.062969153	PASS
AQP12A	2	240691845	240698483	0.025928102	PASS
AQP12B	2	240676418	240682906	0.025928102	PASS
AQP2	12	49950741	49958881	-0.202191512	PASS
AQP3	9	33441154	33447633	-0.310444659	PASS
AQP4	18	26852038	26865818	-0.376397843	PASS
AQP5	12	49961870	49965681	-0.202191512	PASS
AQP6	12	49967194	49977139	-0.202191512	PASS
AQP7	9	33383179	33402682	-0.310444659	PASS
AQP8	16	25215731	25228940	0.069936844	PASS
AQP9	15	58138169	58185911	-0.697600509	PASS
AQR	15	34851782	34969839	-0.396255771	PASS
AR	23	67544032	67730619	-0.012567603	PASS
ARAF	23	47561100	47571920	-0.012567603	PASS
ARAP1	11	72685069	72793599	0.062969153	PASS
ARAP2	4	35948221	36244509	-0.011382299	PASS
ARAP3	5	141653401	141682221	-0.293443042	PASS
ARC	8	142611044	142614472	0.113969953	PASS
ARCN1	11	118572390	118603033	-0.356188624	PASS
AREG	4	74445134	74455009	-0.011382299	PASS
AREL1	14	74653437	74713115	-0.192197564	PASS
ARF1	1	228082660	228099212	-0.256698369	PASS
ARF3	12	48935723	48957551	-0.202191512	PASS
ARF4	3	57571363	57598220	-0.234617007	PASS
ARF5	7	127588345	127591705	-0.388946609	PASS
ARF6	14	49893092	49897054	0.060212819	PASS
ARFGAP1	20	63272785	63289793	-0.168668583	PASS
ARFGAP2	11	47164299	47177125	0.117376172	PASS
ARFGAP3	22	42796502	42858106	-0.575930959	PASS
ARFGEF1	8	67173511	67343677	-0.201142258	PASS

ARFGEF2	20	48921890	49036693	-0.168668583	PASS
ARFGEF3	6	138161921	138344663	-0.035448111	PASS
ARFIP1	4	152779937	152918463	-0.011382299	PASS
ARFIP2	11	6474683	6481479	-0.405444717	PASS
ARFRP1	20	63698642	63708025	-0.168668583	PASS
ARG1	6	131573144	131584332	-0.458570262	PASS
ARG2	14	67619798	67651720	0.060212819	PASS
ARGFX	3	121570704	121586634	0.313454204	suppress_segment_too_large
ARGLU1	13	106541673	106568164	-0.390786075	PASS
ARHGAP1	11	46677080	46700615	0.117376172	PASS
ARHGAP1	4	147732063	148072780	-0.011382299	PASS
ARHGAP1	15	32615144	32639949	-0.396255771	PASS
ARHGAP1	15	30624494	30638805	-0.396255771	PASS
ARHGAP1	10	31805404	31928876	0.039811807	PASS
ARHGAP1	2	143091362	143768352	-0.475215623	PASS
ARHGAP1	16	24919385	25015666	0.069936844	PASS
ARHGAP1	6	129576132	129710225	-0.458570262	PASS
ARHGAP1	10	97222173	97292673	-0.367947924	PASS
ARHGAP1	10	97153045	97292637	-0.367947924	PASS
ARHGAP2	11	110577042	110713189	-0.356188624	PASS
ARHGAP2	10	24583609	24723668	0.039811807	PASS
ARHGAP2	10	48446034	48656265	-0.404852538	PASS
ARHGAP2	17	38419280	38512392	0.060555011	PASS
ARHGAP2	4	85475114	86002670	-0.011382299	PASS
ARHGAP2	2	68679601	68826833	-0.372959126	PASS
ARHGAP2	5	142770377	143229011	-0.293443042	PASS
ARHGAP2	17	45393902	45434421	0.088516311	PASS
ARHGAP2	18	6729718	6915716	-0.052305056	suppress_segment_too_large
ARHGAP2	1	94148988	94275068	0.033763446	PASS
ARHGAP3	1	161046946	161069970	-0.027073548	PASS
ARHGAP3	3	119294373	119420714	0.313454204	suppress_segment_too_large
ARHGAP3	11	128965060	129279324	-0.419612865	PASS
ARHGAP3	19	35774532	35788822	0.093487731	suppress_segment_too_large
ARHGAP3	19	46918676	47005077	0.14996833	PASS
ARHGAP3	23	131058242	131089883	-0.012567603	PASS
ARHGAP3	8	144529179	144605816	0.307788765	PASS
ARHGAP4	23	153907367	153934999	-0.012567603	PASS
ARHGAP4	20	38601934	38651035	-0.168668583	PASS
ARHGAP4	11	100687653	100993941	0.062969153	PASS
ARHGAP4	17	12789539	12991643	-0.139031004	PASS
ARHGAP4	19	1065923	1086628	0.079632875	PASS
ARHGAP5	14	32076114	32159728	0.060212819	PASS
ARHGAP6	23	11137543	11665701	-0.012567603	PASS
ARHGAP8	22	44752558	44862788	-0.808211634	PASS
ARHGAP9	12	57472255	57488814	-0.202191512	PASS
ARHGDIA	17	81867721	81871406	0.088516311	PASS

ARHGDIB	12	14942017	14961728	0.362961408	suppress_segment_too_large
ARHGDIG	16	268727	283010	0.069936844	PASS
ARHGEF1	19	41883161	41930150	0.093487731	suppress_segment_too_large
ARHGEF1	8	1823976	1958641	-0.313874214	PASS
ARHGEF1	1	17539835	17697874	0.033763446	PASS
ARHGEF1	1	156934840	157045370	0.103015788	PASS
ARHGEF1	11	120336914	120489936	-0.356188624	PASS
ARHGEF1	17	8310241	8322516	0.128406487	PASS
ARHGEF1	1	3454426	3481113	0.373964873	suppress_segment_too_large
ARHGEF1	11	73308289	73369388	0.062969153	PASS
ARHGEF1	19	7395113	7472477	0.079632875	PASS
ARHGEF1	1	16197854	16212609	0.033763446	PASS
ARHGEF2	1	155946851	156007070	0.103015788	PASS
ARHGEF2	12	57610180	57619379	-0.202191512	PASS
ARHGEF2	3	154121003	154257827	0.318278996	suppress_segment_too_large
ARHGEF2	5	73626158	73941993	-0.293443042	PASS
ARHGEF3	3	56727418	57079329	-0.234617007	PASS
ARHGEF3	2	38889841	38975449	0.040498426	PASS
ARHGEF3	7	144186083	144195655	-0.388946609	PASS
ARHGEF3	5	149551947	149634968	-0.293443042	PASS
ARHGEF3	4	105552620	105708093	-0.011382299	PASS
ARHGEF3	9	35658875	35675866	-0.310444659	PASS
ARHGEF4	2	130836916	131047263	-0.475215623	PASS
ARHGEF4	14	21070270	21090240	0.060212819	PASS
ARHGEF5	7	144355288	144380632	-0.388946609	PASS
ARHGEF6	23	136665547	136782088	-0.012567603	PASS
ARHGEF7	13	111114559	111305737	-0.390786075	PASS
ARHGEF9	23	63634967	63809274	-0.012567603	PASS
ARID1A	1	26693236	26782110	0.033763446	PASS
ARID1B	6	156776020	157210779	-0.035448111	PASS
ARID2	12	45729665	45908040	-0.202191512	PASS
ARID3A	19	925781	975934	0.079632875	PASS
ARID3B	15	74541177	74598131	-0.697600509	PASS
ARID3C	9	34621379	34628107	-0.310444659	PASS
ARID4A	14	58298385	58373887	0.060212819	PASS
ARID4B	1	235131634	235328219	-0.256698369	PASS
ARID5A	2	96536743	96552638	0.048238121	PASS
ARID5B	10	61901300	62096944	0.072405807	PASS
ARIH1	15	72474326	72602985	-0.697600509	PASS
ARIH2	3	48918821	48986382	-0.234617007	PASS
ARIH2OS	3	48917782	48919385	-0.234617007	PASS
ARL1	12	101393120	101407820	0.202141228	PASS
ARL10	5	176365468	176401865	-0.150979337	PASS
ARL11	13	49628299	49633872	-0.390786075	PASS
ARL13A	23	100969708	100990829	-0.012567603	PASS
ARL13B	3	93980139	94055668	0.313454204	suppress_segment_too_large

ARL14	3	160677159	160678452	0.318278996	suppress_segment_too_large
ARL14EP	11	30323051	30338227	-0.405444717	PASS
ARL14EPL	5	116032324	116060118	-0.293443042	PASS
ARL15	5	53883945	54310582	-0.293443042	PASS
ARL16	17	81681174	81683924	0.088516311	PASS
ARL17A	17	46516702	46579682	0.088516311	PASS
ARL17B	17	46274784	46361797	0.088516311	PASS
ARL2	11	65014113	65022184	0.182439925	suppress_segment_too_large
ARL2-SNX	11	65014182	65040570	0.182439925	suppress_segment_too_large
ARL2BP	16	57245098	57253635	-0.371357732	PASS
ARL3	10	102673731	102714407	-0.367947924	PASS
ARL4A	7	12686856	12690934	-0.69513118	PASS
ARL4C	2	234493041	234497053	0.025928102	PASS
ARL4D	17	43398959	43401137	0.088516311	PASS
ARL5A	2	151788984	151828492	-0.475215623	PASS
ARL5B	10	18659405	18681639	0.039811807	PASS
ARL5C	17	39156894	39167484	0.060555011	PASS
ARL6	3	97764521	97801242	0.313454204	suppress_segment_too_large
ARL6IP1	16	18791667	18801678	0.069936844	PASS
ARL6IP4	12	122980060	122982913	0.202141228	PASS
ARL6IP5	3	69084944	69106066	-0.234617007	PASS
ARL6IP6	2	152717893	152761253	-0.475215623	PASS
ARL8A	1	202133404	202144743	-0.256698369	PASS
ARL8B	3	5122220	5180912	0.311018244	suppress_segment_too_large
ARL9	4	56505209	56525481	-0.011382299	PASS
ARMC1	8	65602456	65634217	-0.201142258	PASS
ARMC10	7	103074881	103099764	-0.388946609	PASS
ARMC12	6	35737032	35749079	0.048820648	PASS
ARMC2	6	108848416	108974472	-0.368590549	PASS
ARMC3	10	22928024	23038523	0.039811807	PASS
ARMC4	10	27812164	27999048	0.039811807	PASS
ARMC5	16	31458080	31467166	0.069936844	PASS
ARMC6	19	19033575	19060311	0.093487731	suppress_segment_too_large
ARMC7	17	75109952	75130265	0.088516311	PASS
ARMC8	3	138187248	138298389	0.313454204	suppress_segment_too_large
ARMC9	2	231198546	231374837	0.025928102	PASS
ARMCX1	23	101550531	101554700	-0.012567603	PASS
ARMCX2	23	101655281	101659891	-0.012567603	PASS
ARMCX3	23	101622797	101627843	-0.012567603	PASS
ARMCX4	23	101418287	101533459	-0.012567603	PASS
ARMCX5	23	102599168	102604159	-0.012567603	PASS
ARMCX6	23	101615118	101618001	-0.012567603	PASS
ARMH1	1	44674692	44725591	0.033763446	PASS
ARMH2	6	24797321	24798889	0.048820648	PASS
ARMH3	10	101845599	102056193	-0.367947924	PASS
ARMH4	14	57999735	58298139	0.060212819	PASS

ARMS2	10	122454653	122457352		0.063874692	PASS
ARMT1	6	151452258	151470101		-0.035448111	PASS
ARNT	1	150809705	150876768		0.103015788	PASS
ARNT2	15	80404350	80597937		-0.697600509	PASS
ARNTL	11	13276652	13387266		-0.405444717	PASS
ARNTL2	12	27332854	27425289		0.362961408	suppress_segment_too_large
ARPC1A	7	99325910	99366262		-0.262778904	PASS
ARPC1B	7	99374249	99394816		-0.262778904	PASS
ARPC2	2	218217094	218254356		0.025928102	PASS
ARPC3	12	110434825	110450422		0.202141228	PASS
ARPC4	3	9792495	9807726		0.311018244	suppress_segment_too_large
ARPC4-TT	3	9793082	9835401		0.311018244	suppress_segment_too_large
ARPC5	1	183620846	183635757		-0.027073548	PASS
ARPC5L	9	124862130	124877733		0.365190713	suppress_segment_too_large
ARPIN	15	89895006	89912956		-0.697600509	PASS
ARPP19	15	52547045	52569883		-0.396255771	PASS
ARPP21	3	35638945	35794496		-0.234617007	PASS
ARR3	23	70268305	70281840		-0.012567603	PASS
ARRB1	11	75264182	75351705		0.062969153	PASS
ARRB2	17	4710489	4721499		0.128406487	PASS
ARRDC1	9	137605654	137615360		0.365190713	suppress_segment_too_large
ARRDC2	19	18001132	18014102		0.093487731	suppress_segment_too_large
ARRDC3	5	91368724	91383359		-0.293443042	PASS
ARRDC4	15	97960698	97973838		-0.697600509	PASS
ARRDC5	19	4890437	4902867		0.079632875	PASS
ARSA	22	50622754	50628173		-0.808211634	PASS
ARSB	5	78777209	78986087		-0.293443042	PASS
ARSD	23	2903970	2929351		-0.012567603	PASS
ARSE	23	2934632	2968310		-0.012567603	PASS
ARSF	23	3041471	3112726		-0.012567603	PASS
ARSG	17	68259182	68422731		0.088516311	PASS
ARSH	23	3006613	3033571		-0.012567603	PASS
ARSI	5	150296343	150339307		-0.293443042	PASS
ARSJ	4	113900284	113979727		-0.011382299	PASS
ARSK	5	95555074	95605064		-0.293443042	PASS
ART1	11	3645128	3664416		-0.333835828	PASS
ART3	4	76011184	76112802		-0.011382299	PASS
ART4	12	14825569	14843533		0.362961408	suppress_segment_too_large
ART5	11	3638503	3642316		-0.333835828	PASS
ARTN	1	43933320	43937241		0.033763446	PASS
ARV1	1	230978981	231000595		-0.256698369	PASS
ARVCF	22	19969896	20016808		-0.575930959	PASS
ARX	23	25003694	25016420		-0.012567603	PASS
AS3MT	10	102869516	102901899		-0.367947924	PASS
ASAH1	8	18055992	18084998		-0.345962679	PASS
ASAH2	10	50182778	50248610		-0.404852538	PASS

ASAH2B	10	50739318	50816495	-0.404852538	PASS
ASAP1	8	130052104	130443660	0.113969953	PASS
ASAP2	2	9206765	9405683	0.040498426	PASS
ASAP3	1	23428563	23484568	0.033763446	PASS
ASB1	2	238426742	238452250	0.025928102	PASS
ASB10	7	151175698	151187832	-0.388946609	PASS
ASB11	23	15281697	15315656	-0.012567603	PASS
ASB12	23	64224196	64230631	-0.012567603	PASS
ASB13	10	5638867	5666595	0.039811807	PASS
ASB14	3	57268347	57292682	-0.234617007	PASS
ASB15	7	123567010	123639481	-0.388946609	PASS
ASB16	17	44170447	44179083	0.088516311	PASS
ASB17	1	75918873	75932431	0.033763446	PASS
ASB18	2	236194872	236264409	0.025928102	PASS
ASB2	14	93934153	93976791	-0.192197564	PASS
ASB3	2	53532672	53787610	0.040498426	PASS
ASB4	7	95478444	95540232	-0.262778904	PASS
ASB5	4	176213673	176277571	-0.011382299	PASS
ASB6	9	129634604	129642169	0.365190713	suppress_segment_too_large
ASB7	15	100602534	100651705	-0.697600509	PASS
ASB8	12	48147788	48181213	-0.202191512	PASS
ASB9	23	15235288	15270467	-0.012567603	PASS
ASCC1	10	72096032	72217134	0.072405807	PASS
ASCC2	22	29788608	29838304	-0.575930959	PASS
ASCC3	6	100508194	100881372	-0.368590549	PASS
ASCL1	12	102957686	102960516	0.202141228	PASS
ASCL2	11	2268495	2270952	-0.333835828	PASS
ASCL3	11	8937579	8938211	-0.405444717	PASS
ASCL4	12	107774385	107776644	0.202141228	PASS
ASCL5	1	201113953	201127184	-0.256698369	PASS
ASF1A	6	118894220	118909167	-0.368590549	PASS
ASF1B	19	14119509	14136956	0.093487731	suppress_segment_too_large
ASGR1	17	7173431	7179564	0.128406487	PASS
ASGR2	17	7101322	7115700	0.128406487	PASS
ASH1L	1	155335268	155562807	0.103015788	PASS
ASH2L	8	38105242	38144076	-0.201142258	PASS
ASIC1	12	50057548	50083611	-0.202191512	PASS
ASIC2	17	33013087	34174964	0.060555011	PASS
ASIC3	7	151048292	151052756	-0.388946609	PASS
ASIC4	2	219514170	219538772	0.025928102	PASS
ASIC5	4	155829729	155866273	-0.011382299	PASS
ASIP	20	34194569	34269344	-0.168668583	PASS
ASL	7	66075798	66093558	-0.750061359	PASS
ASMT	23	1615001	1643081	-0.012567603	PASS
ASMTL	23	1403139	1453762	-0.012567603	PASS
ASNA1	19	12737139	12748323	0.093487731	suppress_segment_too_large

ASNS	7	97852118	97872542	-0.262778904	PASS
ASNSD1	2	189661385	189670831	0.025928102	PASS
ASPA	17	3472374	3503419	0.128406487	PASS
ASPDH	19	50511600	50514690	-0.016761391	PASS
ASPG	14	104085679	104115581	-0.192197564	PASS
ASPH	8	61500556	61714640	-0.201142258	PASS
ASPHD1	16	29900375	29919864	0.069936844	PASS
ASPHD2	22	26429273	26445015	-0.575930959	PASS
ASPM	1	197084128	197146694	-0.027073548	PASS
ASPN	9	92456205	92482506	0.365190713	suppress_segment_too_large
ASPRV1	2	69960089	69962265	-0.372959126	PASS
ASPSCR1	17	81976807	82017406	0.088516311	PASS
ASRGL1	11	62337448	62393412	0.182439925	suppress_segment_too_large
ASS1	9	130444929	130501274	0.365190713	suppress_segment_too_large
ASTE1	3	131013875	131027649	0.313454204	suppress_segment_too_large
ASTL	2	96123850	96138436	0.048238121	PASS
ASTN1	1	176857302	177164973	-0.027073548	PASS
ASTN2	9	116425225	117415070	0.365190713	suppress_segment_too_large
ASXL1	20	32358330	32439319	-0.168668583	PASS
ASXL2	2	25733753	25878516	0.040498426	PASS
ASXL3	18	33578233	33751192	-0.376397843	PASS
ASZ1	7	117363222	117428123	-0.388946609	PASS
ATAD1	10	87751512	87841343	-1.64589449	suppress_large_homdel
ATAD2	8	123319850	123416350	0.279445683	PASS
ATAD2B	2	23748664	23927114	0.040498426	PASS
ATAD3A	1	1512151	1534687	0.373964873	suppress_segment_too_large
ATAD3B	1	1471769	1497848	0.373964873	suppress_segment_too_large
ATAD3C	1	1449689	1470158	0.373964873	suppress_segment_too_large
ATAD5	17	30831970	30895869	0.060555011	PASS
ATAT1	6	30626842	30646823	0.048820648	PASS
ATCAY	19	3879864	3928079	0.079632875	PASS
ATE1	10	121740421	121928801	0.063874692	PASS
ATF1	12	50763710	50821122	-0.202191512	PASS
ATF2	2	175072250	175168382	0.039726562	PASS
ATF3	1	212565334	212620777	-0.256698369	PASS
ATF4	22	39519695	39522685	-0.575930959	PASS
ATF5	19	49928702	49933935	-0.016761391	PASS
ATF6	1	161766294	161964070	-0.027073548	PASS
ATF6B	6	32115264	32128253	0.048820648	PASS
ATF7	12	53507856	53626410	-0.202191512	PASS
ATF7IP	12	14365632	14502935	0.362961408	suppress_segment_too_large
ATF7IP2	16	10326434	10483638	0.069936844	PASS
ATG10	5	81972025	82276857	-0.293443042	PASS
ATG101	12	52069246	52077494	-0.202191512	PASS
ATG12	5	115828200	115841858	-0.293443042	PASS
ATG13	11	46617527	46674818	0.117376172	PASS

ATG14	14	55366392	55411858	0.060212819	PASS
ATG16L1	2	233210051	233295674	0.025928102	PASS
ATG16L2	11	72814308	72843674	0.062969153	PASS
ATG2A	11	64894546	64917248	0.182439925	suppress_segment_too_large
ATG2B	14	96279202	96363870	-0.192197564	PASS
ATG3	3	112532509	112562046	0.313454204	suppress_segment_too_large
ATG4A	23	108091668	108154671	-0.012567603	PASS
ATG4C	1	62784135	62865513	0.033763446	PASS
ATG4D	19	10543895	10553418	0.093487731	suppress_segment_too_large
ATG5	6	106045423	106325791	-0.368590549	PASS
ATG7	3	11272309	11557665	0.311018244	suppress_segment_too_large
ATG9A	2	219219380	219229717	0.025928102	PASS
ATG9B	7	151012209	151024499	-0.388946609	PASS
ATIC	2	215311817	215349773	0.025928102	PASS
ATL1	14	50532509	50633068	0.060212819	PASS
ATL2	2	38294880	38377285	0.040498426	PASS
ATL3	11	63624087	63671921	0.182439925	suppress_segment_too_large
ATM	11	108222484	108369102	-0.356188624	PASS
ATMIN	16	81035847	81047358	-0.371357732	PASS
ATN1	12	6924463	6942321	-0.086128028	PASS
ATOH1	4	93828753	93830964	-0.011382299	PASS
ATOH7	10	68230624	68232103	0.072405807	PASS
ATOH8	2	85751344	85788066	0.066915641	PASS
ATOX1	5	151742316	151772532	-0.293443042	PASS
ATP10A	15	25677273	25865172	-0.396255771	PASS
ATP10B	5	160563120	160852214	-0.150979337	PASS
ATP10D	4	47485288	47593486	-0.011382299	PASS
ATP11A	13	112690329	112887168	-0.390786075	PASS
ATP11B	3	182793500	182921635	0.318278996	suppress_segment_too_large
ATP11C	23	139726346	139945276	-0.012567603	PASS
ATP12A	13	24680411	24712493	-0.390786075	PASS
ATP13A1	19	19645198	19663693	0.093487731	suppress_segment_too_large
ATP13A2	1	16985958	17011928	0.033763446	PASS
ATP13A3	3	194402672	194498364	0.326834516	PASS
ATP13A4	3	193402077	193593111	-0.627320105	PASS
ATP13A5	3	193274790	193378843	-0.627320105	PASS
ATP1A1	1	116372668	116410261	0.033763446	PASS
ATP1A2	1	160115759	160143591	-0.027073548	PASS
ATP1A3	19	41966582	41997497	0.093487731	suppress_segment_too_large
ATP1A4	1	160151570	160186977	-0.027073548	PASS
ATP1B1	1	169105697	169132722	-0.027073548	PASS
ATP1B2	17	7646627	7657768	0.128406487	PASS
ATP1B3	3	141876124	141926514	-0.356957581	PASS
ATP1B4	23	120362085	120383249	-0.012567603	PASS
ATP23	12	57906039	57959148	-0.202191512	PASS
ATP2A1	16	28878405	28904509	0.069936844	PASS

ATP2A2	12	110280756	110351093	0.202141228	PASS
ATP2A3	17	3923870	3964464	0.128406487	PASS
ATP2B1	12	89588049	89709300	0.202141228	PASS
ATP2B2	3	10324023	10708007	0.311018244	suppress_segment_too_large
ATP2B3	23	153517676	153582939	-0.012567603	PASS
ATP2B4	1	203626561	203744081	-0.256698369	PASS
ATP2C1	3	130850595	131016712	0.313454204	suppress_segment_too_large
ATP2C2	16	84368527	84464187	-0.371357732	PASS
ATP4A	19	35550043	35563658	0.093487731	suppress_segment_too_large
ATP4B	13	113648804	113658186	-0.390786075	PASS
ATP5F1A	18	46080248	46104334	-0.376397843	PASS
ATP5F1B	12	56638175	56646068	-0.202191512	PASS
ATP5F1C	10	7788129	7807815	0.039811807	PASS
ATP5F1D	19	1241746	1244826	0.079632875	PASS
ATP5F1E	20	59025467	59032382	-0.168668583	PASS
ATP5IF1	1	28236109	28246906	0.033763446	PASS
ATP5MC1	17	48892765	48895871	0.088516311	PASS
ATP5MC2	12	53632726	53677408	-0.202191512	PASS
ATP5MC3	2	175176258	175184607	0.039726562	PASS
ATP5MD	10	103389041	103396492	-0.367947924	PASS
ATP5ME	4	672436	674338	0.279089364	PASS
ATP5MF	7	99448475	99466331	-0.262778904	PASS
ATP5MF-P	7	99419749	99466197	-0.262778904	PASS
ATP5MG	11	118401154	118431496	-0.356188624	PASS
ATP5MGL	22	42639803	42640601	-0.575930959	PASS
ATP5MPL	14	103912288	103928269	-0.192197564	PASS
ATP5PB	1	111448864	111462773	0.033763446	PASS
ATP5PD	17	75038863	75046985	0.088516311	PASS
ATP5PF	21	25716503	25735673	0.12860881	suppress_segment_too_large
ATP5PO	21	33903453	33915980	0.12860881	suppress_segment_too_large
ATP5S	14	50312326	50335558	0.060212819	PASS
ATP6AP1	23	154428632	154436516	-0.012567603	PASS
ATP6AP1L	5	82279462	82386977	-0.293443042	PASS
ATP6AP2	23	40579372	40606848	-0.012567603	PASS
ATP6V0A1	17	42458844	42522611	0.088516311	PASS
ATP6V0A2	12	123712318	123761755	0.202141228	PASS
ATP6V0A4	7	138706294	138799560	-0.388946609	PASS
ATP6V0B	1	43974487	43978295	0.033763446	PASS
ATP6V0C	16	2513870	2520218	0.069936844	PASS
ATP6V0D1	16	67438014	67481237	-0.371357732	PASS
ATP6V0D2	8	85987323	86154228	0.279445683	PASS
ATP6V0E1	5	172983757	173035445	-0.150979337	PASS
ATP6V0E2	7	149872968	149891204	-0.388946609	PASS
ATP6V1A	3	113747019	113812056	0.313454204	suppress_segment_too_large
ATP6V1B1	2	70935868	70965431	-0.372959126	PASS
ATP6V1B2	8	20197367	20226819	-0.345962679	PASS

ATP6V1C1	8	103021063	103073051	0.279445683	PASS
ATP6V1C2	2	10721100	10785110	0.040498426	PASS
ATP6V1D	14	67294371	67360265	0.060212819	PASS
ATP6V1E1	22	17592136	17628818	-0.575930959	PASS
ATP6V1E2	2	46490750	46542557	0.040498426	PASS
ATP6V1F	7	128862826	128865844	-0.388946609	PASS
ATP6V1FN	7	128866308	128872044	-0.388946609	PASS
ATP6V1G1	9	114587746	114598373	0.365190713	suppress_segment_too_large
ATP6V1G2	6	31544462	31548427	0.048820648	PASS
ATP6V1G2	6	31530219	31546608	0.048820648	PASS
ATP6V1G3	1	198523222	198540945	-0.027073548	PASS
ATP6V1H	8	53715557	53843558	-0.201142258	PASS
ATP7A	23	77910656	78050395	-0.012567603	PASS
ATP7B	13	51930436	52012125	-0.390786075	PASS
ATP8A1	4	42408373	42657105	-0.011382299	PASS
ATP8A2	13	25372071	26025851	-0.390786075	PASS
ATP8B1	18	57646426	57803315	-0.376397843	PASS
ATP8B2	1	154325553	154351307	0.103015788	PASS
ATP8B3	19	1782075	1812276	0.079632875	PASS
ATP8B4	15	49858238	50182817	-0.396255771	PASS
ATP9A	20	51596514	51768634	-0.168668583	PASS
ATP9B	18	79069285	79378287	-0.376397843	PASS
ATPAF1	1	46632737	46673867	0.033763446	PASS
ATPAF2	17	17977409	18039209	0.024610076	PASS
ATR	3	142449235	142578826	0.318278996	suppress_segment_too_large
ATRAID	2	27212027	27217178	0.040498426	PASS
ATRIP	3	48446710	48467645	-0.234617007	PASS
ATRN	20	3471040	3651122	-0.168668583	PASS
ATRNL1	10	115093365	115948992	0.063874692	PASS
ATRX	23	77504878	77786233	-0.012567603	PASS
ATXN1	6	16299112	16761491	0.048820648	PASS
ATXN10	22	45671798	45845307	-0.808211634	PASS
ATXN1L	16	71845991	71885268	-0.371357732	PASS
ATXN2	12	111443485	111599676	0.202141228	PASS
ATXN2L	16	28823035	28837237	0.069936844	PASS
ATXN3	14	92044496	92106625	-0.192197564	PASS
ATXN3L	23	13318236	13320399	-0.012567603	PASS
ATXN7	3	63864557	64003462	-0.234617007	PASS
ATXN7L1	7	105605067	105876604	-0.388946609	PASS
ATXN7L2	1	109483479	109492804	0.033763446	PASS
ATXN7L3	17	44191805	44200113	0.088516311	PASS
ATXN7L3l	12	74537827	74545430	0.202141228	PASS
AUH	9	91213815	91361913	0.365190713	suppress_segment_too_large
AUNIP	1	25831913	25859458	0.033763446	PASS
AUP1	2	74526645	74529939	-0.372959126	PASS
AURKA	20	56369389	56392337	-0.168668583	PASS

AURKAIP	1	1373730	1375495	0.373964873	suppress_segment_too_large
AURKB	17	8204733	8210600	0.128406487	PASS
AURKC	19	57230802	57235548	-0.180829161	PASS
AUTS2	7	69598296	70793495	-0.750061359	PASS
AVEN	15	33866227	34039176	-0.396255771	PASS
AVIL	12	57797376	57818704	-0.202191512	PASS
AVL9	7	32495426	32588721	-0.69513118	PASS
AVP	20	3082556	3084724	-0.168668583	PASS
AVPI1	10	97677424	97687323	-0.367947924	PASS
AVPR1A	12	63142759	63150942	-0.202191512	PASS
AVPR1B	1	206109692	206117699	-0.256698369	PASS
AVPR2	23	153902531	153907166	-0.012567603	PASS
AWAT1	23	70234655	70240627	-0.012567603	PASS
AWAT2	23	70040542	70049938	-0.012567603	PASS
AXDND1	1	179365720	179554735	-0.027073548	PASS
AXIN1	16	287440	352673	0.069936844	PASS
AXIN2	17	65528563	65561647	0.088516311	PASS
AXL	19	41219203	41261766	0.093487731	suppress_segment_too_large
AZGP1	7	99966720	99976157	-0.262778904	PASS
AZI2	3	28315003	28349127	0.311018244	suppress_segment_too_large
AZIN1	8	102826357	102893864	0.279445683	PASS
AZIN2	1	33081104	33120530	0.033763446	PASS
AZU1	19	825097	832018	0.079632875	PASS
B2M	15	44711487	44718877	-0.396255771	PASS
B3GALNT	3	161083883	161105384	0.318278996	suppress_segment_too_large
B3GALNT	1	235449923	235504481	-0.256698369	PASS
B3GALT1	2	167868948	167874041	0.039726562	PASS
B3GALT2	1	193179045	193186654	-0.027073548	PASS
B3GALT4	6	33277132	33284832	0.048820648	PASS
B3GALT5	21	39556442	39673137	-0.064425456	suppress_segment_too_large
B3GALT6	1	1232226	1235041	0.373964873	suppress_segment_too_large
B3GAT1	11	134378504	134411918	-0.419612865	PASS
B3GAT2	6	70856679	70957038	-0.368590549	PASS
B3GAT3	11	62615296	62622175	0.182439925	suppress_segment_too_large
B3GLCT	13	31199936	31332276	-0.390786075	PASS
B3GNT2	2	62196113	62224731	-0.372959126	PASS
B3GNT3	19	17794828	17813082	0.093487731	suppress_segment_too_large
B3GNT4	12	122203543	122208952	0.202141228	PASS
B3GNT5	3	183253244	183298504	0.318278996	suppress_segment_too_large
B3GNT6	11	77034398	77041973	0.062969153	PASS
B3GNT7	2	231395543	231401164	0.025928102	PASS
B3GNT8	19	41425359	41428730	0.093487731	suppress_segment_too_large
B3GNT9	16	67148105	67151214	-0.371357732	PASS
B3GNTL1	17	82942155	83051810	0.088516311	PASS
B4GALNT	12	57623410	57633355	-0.202191512	PASS
B4GALNT	17	49132460	49176840	0.088516311	PASS

B4GALNT	12	460364	563509	-0.086128028	PASS
B4GALNT	11	369796	382116	-0.333835828	PASS
B4GALT1	9	33104082	33167356	-0.310444659	PASS
B4GALT2	1	43978943	43991170	0.033763446	PASS
B4GALT3	1	161171310	161177968	-0.027073548	PASS
B4GALT4	3	119211732	119241103	0.313454204	suppress_segment_too_large
B4GALT5	20	49632945	49713878	-0.168668583	PASS
B4GALT6	18	31622247	31685836	-0.376397843	PASS
B4GALT7	5	177600100	177610347	-0.150979337	PASS
B4GAT1	11	66345372	66347692	0.182439925	suppress_segment_too_large
B9D1	17	19337554	19378193	0.024610076	PASS
B9D2	19	41354421	41364173	0.093487731	suppress_segment_too_large
BAALC	8	103140710	103230305	0.279445683	PASS
BAAT	9	101360417	101383519	0.365190713	suppress_segment_too_large
BABAM1	19	17267350	17281249	0.093487731	suppress_segment_too_large
BABAM2	2	27889941	28338901	0.040498426	PASS
BACE1	11	117285207	117316259	-0.356188624	PASS
BACE2	21	41167801	41282518	-0.064425456	suppress_segment_too_large
BACH1	21	29194071	29630751	0.12860881	suppress_segment_too_large
BACH2	6	89926529	90296908	-0.368590549	PASS
BAD	11	64269830	64284704	0.182439925	suppress_segment_too_large
BAG1	9	33247820	33264720	-0.310444659	PASS
BAG2	6	57172326	57189833	0.048820648	PASS
BAG3	10	119651370	119677819	0.063874692	PASS
BAG4	8	38176533	38213301	-0.201142258	PASS
BAG5	14	103556544	103562831	-0.192197564	PASS
BAG6	6	31639028	31652705	0.048820648	PASS
BAHCC1	17	81395475	81466332	0.088516311	PASS
BAHD1	15	40439721	40468242	-0.396255771	PASS
BAIAP2	17	81035122	81117432	0.088516311	PASS
BAIAP2L1	7	98291651	98401068	-0.262778904	PASS
BAIAP2L2	22	38084889	38110670	-0.575930959	PASS
BAIAP3	16	1333601	1349441	0.069936844	PASS
BAK1	6	33572547	33580293	0.048820648	PASS
BAMBI	10	28677342	28682939	0.039811807	PASS
BANF1	11	66002079	66004149	0.182439925	suppress_segment_too_large
BANF2	20	17693672	17735871	-0.168668583	PASS
BANK1	4	101411286	102074812	-0.011382299	PASS
BANP	16	87949244	88077318	-0.371357732	PASS
BAP1	3	52401013	52410350	-0.234617007	PASS
BARD1	2	214725646	214809711	0.025928102	PASS
BARHL1	9	132582185	132590266	0.365190713	suppress_segment_too_large
BARHL2	1	90711539	90717237	0.033763446	PASS
BARX1	9	93951622	93955372	0.365190713	suppress_segment_too_large
BARX2	11	129375940	129452279	-0.419612865	PASS
BASP1	5	17065598	17276843	0.055007813	PASS

BATF	14	75522425	75547015	-0.192197564	PASS
BATF2	11	64987943	64997045	0.182439925	suppress_segment_too_large
BATF3	1	212686418	212699985	-0.256698369	PASS
BAX	19	48954815	48961798	0.14996833	PASS
BAZ1A	14	34752731	34875647	0.060212819	PASS
BAZ1B	7	73440398	73522278	-0.262778904	PASS
BAZ2A	12	56595596	56636816	-0.202191512	PASS
BAZ2B	2	159318979	159616692	0.039726562	PASS
BBC3	19	47220822	47232766	0.14996833	PASS
BBIP1	10	110898730	110919274	0.063874692	PASS
BBOF1	14	74019353	74082863	0.060212819	PASS
BBOX1	11	27040725	27127809	-0.405444717	PASS
BBS1	11	66510606	66533627	0.182439925	suppress_segment_too_large
BBS10	12	76344474	76348442	0.202141228	PASS
BBS12	4	122732702	122744943	-0.011382299	PASS
BBS2	16	56466836	56520283	-0.371357732	PASS
BBS4	15	72686179	72738476	-0.697600509	PASS
BBS5	2	169479178	169506655	0.039726562	PASS
BBS7	4	121824440	121870497	-0.011382299	PASS
BBS9	7	33129244	33606068	-0.69513118	PASS
BBX	3	107522936	107811324	0.313454204	suppress_segment_too_large
BCAM	19	44809059	44821421	0.14996833	PASS
BCAN	1	156641390	156659532	0.103015788	PASS
BCAP29	7	107579977	107629170	-0.388946609	PASS
BCAP31	23	153700492	153724746	-0.012567603	PASS
BCAR1	16	75228187	75268053	-0.371357732	PASS
BCAR3	1	93561786	93847150	0.033763446	PASS
BCAS1	20	53936777	54070594	-0.168668583	PASS
BCAS2	1	114567557	114581639	0.033763446	PASS
BCAS3	17	60677453	61392838	0.088516311	PASS
BCAS4	20	50794894	50882676	-0.168668583	PASS
BCAT1	12	24810022	24949459	0.362961408	suppress_segment_too_large
BCAT2	19	48795062	48811029	0.14996833	PASS
BCCIP	10	125823546	125853695	0.063874692	PASS
BCDIN3D	12	49836039	49843129	-0.202191512	PASS
BCHE	3	165772904	165837472	0.318278996	suppress_segment_too_large
BCKDHA	19	41397460	41425005	0.093487731	suppress_segment_too_large
BCKDHB	6	80106647	80346270	-0.368590549	PASS
BCKDK	16	31106107	31112791	0.069936844	PASS
BCL10	1	85265776	85276904	0.033763446	PASS
BCL11A	2	60450520	60555154	-0.372959126	PASS
BCL11B	14	99169287	99271524	-0.192197564	PASS
BCL2	18	63123346	63320128	-0.376397843	PASS
BCL2A1	15	79960889	79971446	-0.697600509	PASS
BCL2L1	20	31664452	31723989	-0.168668583	PASS
BCL2L10	15	52109263	52112775	-0.396255771	PASS

BCL2L11	2	111119378	111168447	-0.010901494	PASS
BCL2L12	19	49665142	49673916	0.14996833	PASS
BCL2L13	22	17628855	17730855	-0.575930959	PASS
BCL2L14	12	12049844	12211084	0.362961408	suppress_segment_too_large
BCL2L15	1	113878168	113887547	0.033763446	PASS
BCL2L2	14	23298790	23311759	0.060212819	PASS
BCL2L2-P.	14	23306835	23325369	0.060212819	PASS
BCL3	19	44747705	44760044	0.14996833	PASS
BCL6	3	187721377	187745727	0.318278996	suppress_segment_too_large
BCL6B	17	7023020	7030290	0.128406487	PASS
BCL7A	12	122019422	122062044	0.202141228	PASS
BCL7B	7	73536356	73558002	-0.262778904	PASS
BCL7C	16	30833626	30894960	0.069936844	PASS
BCL9	1	147541412	147626216	-0.157385092	PASS
BCL9L	11	118893875	118925608	-0.356188624	PASS
BCLAF1	6	136256627	136289851	-0.035448111	PASS
BCLAF3	23	19912860	19970298	-0.012567603	PASS
BCO1	16	81238448	81291142	-0.371357732	PASS
BCO2	11	112175467	112224699	-0.356188624	PASS
BCOR	23	40049815	40177329	-0.012567603	PASS
BCORL1	23	129981107	130058083	-0.012567603	PASS
BCR	22	23179704	23318037	-0.575930959	PASS
BCS1L	2	218658764	218663443	0.025928102	PASS
BDH1	3	197509783	197573323	0.326834516	PASS
BDH2	4	103079435	103099883	-0.011382299	PASS
BDKRB1	14	96255824	96268967	-0.192197564	PASS
BDKRB2	14	96204679	96244166	-0.192197564	PASS
BDNF	11	27654893	27722058	-0.405444717	PASS
BDP1	5	71455615	71567820	-0.293443042	PASS
BEAN1	16	66427297	66493529	-0.371357732	PASS
BECN1	17	42810134	42833350	0.088516311	PASS
BECN2	1	241957767	241959062	-0.648598641	PASS
BEGAIN	14	100537147	100587413	-0.192197564	PASS
BEND2	23	18162931	18220883	-0.012567603	PASS
BEND3	6	107065182	107115269	-0.368590549	PASS
BEND4	4	42110938	42152878	-0.011382299	PASS
BEND5	1	48727523	48776969	0.033763446	PASS
BEND6	6	56955126	57027342	0.048820648	PASS
BEND7	10	13438484	13528974	0.039811807	PASS
BEST1	11	61949821	61965515	0.182439925	suppress_segment_too_large
BEST2	19	12751702	12758458	0.093487731	suppress_segment_too_large
BEST3	12	69643360	69699476	-0.202191512	PASS
BEST4	1	44783585	44787705	0.033763446	PASS
BET1	7	93962762	94004382	-0.262778904	PASS
BET1L	11	167784	207428	-0.333835828	PASS
BEX1	23	103062651	103064240	-0.012567603	PASS

BEX2	23	103309346	103311046	-0.012567603	PASS
BEX3	23	103376340	103378077	-0.012567603	PASS
BEX4	23	103215092	103217246	-0.012567603	PASS
BEX5	23	102153708	102156057	-0.012567603	PASS
BFAR	16	14632815	14669236	0.069936844	PASS
BFSP1	20	17493905	17569220	-0.168668583	PASS
BFSP2	3	133399995	133475222	0.313454204	suppress_segment_too_large
BGLAP	1	156241962	156243321	0.103015788	PASS
BGN	23	153494939	153509554	-0.012567603	PASS
BHLHA15	7	98211427	98212979	-0.262778904	PASS
BHLHA9	17	1270559	1271460	0.128406487	PASS
BHLHB9	23	102720688	102753540	-0.012567603	PASS
BHLHE22	8	64580367	64583628	-0.201142258	PASS
BHLHE23	20	63005927	63007035	-0.168668583	PASS
BHLHE40	3	4979116	4985323	0.311018244	suppress_segment_too_large
BHLHE41	12	26120026	26125127	0.362961408	suppress_segment_too_large
BHMG1	19	45733251	45764534	0.14996833	PASS
BHMT	5	79111779	79132290	-0.293443042	PASS
BHMT2	5	79069717	79089466	-0.293443042	PASS
BICC1	10	58513140	58831437	0.072405807	PASS
BICD1	12	32106835	32383633	0.362961408	suppress_segment_too_large
BICD2	9	92711363	92764812	0.365190713	suppress_segment_too_large
BICDL1	12	119989869	120094494	0.202141228	PASS
BICDL2	16	3027682	3036926	0.069936844	PASS
BICRA	19	47608196	47703277	0.14996833	PASS
BICRAL	6	42746958	42868560	0.048820648	PASS
BID	22	17734138	17774770	-0.575930959	PASS
BIK	22	43110748	43129712	-0.575930959	PASS
BIN1	2	127048027	127107355	-0.475215623	PASS
BIN2	12	51281038	51324668	-0.202191512	PASS
BIN3	8	22620418	22669148	-0.345962679	PASS
BIRC2	11	102347211	102378670	0.062969153	PASS
BIRC3	11	102317450	102339403	0.062969153	PASS
BIRC5	17	78214186	78225636	0.088516311	PASS
BIRC6	2	32357028	32618899	0.040498426	PASS
BIRC7	20	63235883	63240507	-0.168668583	PASS
BIRC8	19	53289601	53291626	0.206912349	PASS
BIVM	13	102799049	102841535	-0.390786075	PASS
BIVM-ERC	13	102799110	102875994	-0.390786075	PASS
BLCAP	20	37492472	37527931	-0.168668583	PASS
BLID	11	122115354	122116215	-0.356188624	PASS
BLK	8	11486894	11564604	-0.345962679	PASS
BLM	15	90717327	90816165	-0.697600509	PASS
BLMH	17	30248195	30292056	0.060555011	PASS
BLNK	10	96191702	96271587	-0.367947924	PASS
BLOC1S1	12	55716037	55720087	-0.202191512	PASS

BLOC1S1-	12	55716036	55724703	-0.202191512	PASS
BLOC1S2	10	100273280	100286712	-0.367947924	PASS
BLOC1S3	19	45178745	45181801	0.14996833	PASS
BLOC1S4	4	6716055	6717671	0.279089364	PASS
BLOC1S5	6	8013567	8064414	0.048820648	PASS
BLOC1S5-	6	7881522	8064364	0.048820648	PASS
BLOC1S6	15	45587123	45615999	-0.396255771	PASS
BLVRA	7	43758680	43807342	-0.69513118	PASS
BLVRB	19	40447765	40465840	0.093487731	suppress_segment_too_large
BLZF1	1	169367970	169396540	-0.027073548	PASS
BMF	15	40087890	40108892	-0.396255771	PASS
BMI1	10	22321211	22331484	0.039811807	PASS
BMP1	8	22164736	22212326	-0.345962679	PASS
BMP10	2	68865481	68871517	-0.372959126	PASS
BMP15	23	50910784	50916607	-0.012567603	PASS
BMP2	20	6767664	6780280	-0.168668583	PASS
BMP2K	4	78776342	78916372	-0.011382299	PASS
BMP3	4	81030965	81057531	-0.011382299	PASS
BMP4	14	53949736	53958761	0.060212819	PASS
BMP5	6	55753645	55875564	0.048820648	PASS
BMP6	6	7726797	7881422	0.048820648	PASS
BMP7	20	57168748	57266629	-0.168668583	PASS
BMP8A	1	39491646	39525935	0.033763446	PASS
BMP8B	1	39757182	39788861	0.033763446	PASS
BMPER	7	33904308	34156356	-0.69513118	PASS
BMPR1A	10	86756601	86932838	-0.292453993	PASS
BMPR1B	4	94757968	95158448	-0.011382299	PASS
BMPR2	2	202376936	202567751	0.025928102	PASS
BMS1	10	42782801	42834937	-0.404852538	PASS
BMT2	7	112819147	112939916	-0.388946609	PASS
BMX	23	15464246	15556529	-0.012567603	PASS
BNC1	15	83255903	83284714	-0.697600509	PASS
BNC2	9	16409503	16870843	-0.310444659	PASS
BNIP1	5	173144442	173164387	-0.150979337	PASS
BNIP2	15	59659146	59689534	-0.697600509	PASS
BNIP3	10	131966455	131982013	0.063874692	PASS
BNIP3L	8	26382898	26505636	-0.345962679	PASS
BNIPL	1	151036321	151047720	0.103015788	PASS
BOC	3	113211003	113287459	0.313454204	suppress_segment_too_large
BOD1	5	173607514	173616660	-0.150979337	PASS
BOD1L1	4	13568738	13627723	0.279089364	PASS
BOD1L2	18	57147062	57150300	-0.376397843	PASS
BOLA1	1	149887890	149900798	0.103015788	PASS
BOLA2	16	29443056	29454964	0.069936844	PASS
BOLA2-SM	16	29443230	29454651	0.069936844	PASS
BOLA3	2	74135398	74147994	-0.372959126	PASS

BOLL	2	197726879	197786762	0.025928102	PASS
BOP1	8	144262102	144291370	0.307788765	PASS
BORA	13	72727749	72756198	-0.390786075	PASS
BORCS5	12	12357079	12469694	0.362961408	suppress_segment_too_large
BORCS6	17	8188333	8190907	0.128406487	PASS
BORCS7	10	102854223	102864961	-0.367947924	PASS
BORCS7-A	10	102854272	102901899	-0.367947924	PASS
BORCS8	19	19176903	19192591	0.093487731	suppress_segment_too_large
BORCS8-M	19	19145567	19192158	0.093487731	suppress_segment_too_large
BPGM	7	134646808	134679813	-0.388946609	PASS
BPHL	6	3118374	3153578	0.048820648	PASS
BPI	20	38304123	38337505	-0.168668583	PASS
BPIFA1	20	33235995	33243311	-0.168668583	PASS
BPIFA2	20	33161768	33181412	-0.168668583	PASS
BPIFA3	20	33217310	33227758	-0.168668583	PASS
BPIFB1	20	33273480	33309878	-0.168668583	PASS
BPIFB2	20	33007600	33023709	-0.168668583	PASS
BPIFB3	20	33055424	33073628	-0.168668583	PASS
BPIFB4	20	33079644	33111751	-0.168668583	PASS
BPIFB6	20	33031648	33044047	-0.168668583	PASS
BPIFC	22	32413847	32464484	-0.575930959	PASS
BPNT1	1	220057482	220090462	-0.256698369	PASS
BPTF	17	67825524	67984378	0.088516311	PASS
BRAF	7	140719327	140924928	-0.388946609	PASS
BRAP	12	111642146	111685986	0.202141228	PASS
BRAT1	7	2537877	2555727	-0.69513118	PASS
BRCA1	17	43044295	43170245	0.088516311	PASS
BRCA2	13	32315474	32400266	-0.390786075	PASS
BRCC3	23	155071420	155123074	-0.012567603	PASS
BRD1	22	49773283	49827512	-0.808211634	PASS
BRD2	6	32968660	32981505	0.048820648	PASS
BRD3	9	134030305	134068535	0.365190713	suppress_segment_too_large
BRD3OS	9	134025439	134034666	0.365190713	suppress_segment_too_large
BRD4	19	15235519	15332545	0.093487731	suppress_segment_too_large
BRD7	16	50313487	50368934	0.069936844	PASS
BRD8	5	138139766	138178986	-0.293443042	PASS
BRD9	5	850291	892824	0.055007813	PASS
BRDT	1	91949371	92014426	0.033763446	PASS
BRF1	14	105209286	105315589	-0.192197564	PASS
BRF2	8	37843268	37849904	-0.201142258	PASS
BRI3	7	98252379	98310441	-0.262778904	PASS
BRI3BP	12	124993700	125031231	0.202141228	PASS
BRICD5	16	2209253	2211950	0.069936844	PASS
BRINP1	9	119153458	119369467	0.365190713	suppress_segment_too_large
BRINP2	1	177171497	177282422	-0.027073548	PASS
BRINP3	1	190097662	190478404	-0.027073548	PASS

BRIP1	17	61681266	61863521	0.088516311	PASS
BRIX1	5	34915376	34925996	0.055007813	PASS
BRK1	3	10115592	10127190	0.311018244	suppress_segment_too_large
BRMS1	11	66337333	66345125	0.182439925	suppress_segment_too_large
BRMS1L	14	35826318	35932325	0.060212819	PASS
BROX	1	222712553	222735196	-0.256698369	PASS
BRPF1	3	9731729	9748018	0.311018244	suppress_segment_too_large
BRPF3	6	36196744	36232790	0.048820648	PASS
BRS3	23	136487887	136493780	-0.012567603	PASS
BRSK1	19	55282072	55312533	0.121336758	PASS
BRSK2	11	1389899	1462689	-0.333835828	PASS
BRWD1	21	39184176	39321559	-0.064425456	suppress_segment_too_large
BRWD3	23	80670854	80809688	-0.012567603	PASS
BSCL2	11	62690275	62709845	0.182439925	suppress_segment_too_large
BSDC1	1	32365103	32394731	0.033763446	PASS
BSG	19	571277	583493	0.079632875	PASS
BSN	3	49554489	49671545	-0.234617007	PASS
BSND	1	54998193	55011784	0.033763446	PASS
BSPH1	19	47968046	47992170	0.14996833	PASS
BSPRY	9	113349541	113371233	0.365190713	suppress_segment_too_large
BST1	4	15702950	15738313	0.279089364	PASS
BST2	19	17402939	17405648	0.093487731	suppress_segment_too_large
BSX	11	122977570	122981720	-0.356188624	PASS
BTAF1	10	91923769	92030325	-0.39678585	PASS
BTBD1	15	83016422	83067354	-0.697600509	PASS
BTBD10	11	13388001	13463297	-0.405444717	PASS
BTBD11	12	107318413	107659642	0.202141228	PASS
BTBD16	10	122271306	122338162	0.063874692	PASS
BTBD17	17	74356416	74361946	0.088516311	PASS
BTBD18	11	57743514	57751781	-0.129266491	PASS
BTBD19	1	44808482	44815585	0.033763446	PASS
BTBD2	19	1985438	2034881	0.079632875	PASS
BTBD3	20	11890723	11926609	-0.168668583	PASS
BTBD6	14	105248490	105251093	-0.192197564	PASS
BTBD7	14	93237550	93333092	-0.192197564	PASS
BTBD8	1	92080305	92184725	0.033763446	PASS
BTBD9	6	38168451	38640148	0.048820648	PASS
BTC	4	74744759	74794686	-0.011382299	PASS
BTD	3	15601341	15647640	0.311018244	suppress_segment_too_large
BTF3	5	73498408	73505635	-0.293443042	PASS
BTF3L4	1	52056125	52090716	0.033763446	PASS
BTG1	12	92140278	92145897	0.202141228	PASS
BTG2	1	203305491	203309602	-0.256698369	PASS
BTG3	21	17593653	17612947	0.12860881	suppress_segment_too_large
BTG4	11	111467526	111512354	-0.356188624	PASS
BTK	23	101349447	101390796	-0.012567603	PASS

BTLA	3	112463968	112499561	0.313454204	suppress_segment_too_large
BTN1A1	6	26501221	26510422	0.048820648	PASS
BTN2A1	6	26457904	26476621	0.048820648	PASS
BTN2A2	6	26383096	26394874	0.048820648	PASS
BTN3A1	6	26402237	26415216	0.048820648	PASS
BTN3A2	6	26365159	26378320	0.048820648	PASS
BTN3A3	6	26440472	26453415	0.048820648	PASS
BTNL2	6	32393963	32407128	0.048820648	PASS
BTNL3	5	180988845	181006727	-0.150979337	PASS
BTNL8	5	180899077	180950906	-0.150979337	PASS
BTNL9	5	181040225	181061523	-0.150979337	PASS
BTRC	10	101354033	101557321	-0.367947924	PASS
BUB1	2	110637698	110678114	-0.010901494	PASS
BUB1B	15	40161023	40221136	-0.396255771	PASS
BUB1B-PA	15	40217428	40276396	-0.396255771	PASS
BUB3	10	123154277	123170467	0.063874692	PASS
BUD13	11	116748170	116772988	-0.356188624	PASS
BUD23	7	73683025	73705161	-0.262778904	PASS
BUD31	7	99408641	99419616	-0.262778904	PASS
BVES	6	105096822	105137174	-0.368590549	PASS
BX276092.	23	71667542	71671524	-0.012567603	PASS
BYSL	6	41921188	41933046	0.048820648	PASS
BZW1	2	200810594	200827338	0.025928102	PASS
BZW2	7	16646131	16706523	-0.69513118	PASS
C10orf105	10	71711702	71737824	0.072405807	PASS
C10orf113	10	21125763	21146559	0.039811807	PASS
C10orf120	10	122697709	122699822	0.063874692	PASS
C10orf142	10	44292569	44294649	-0.404852538	PASS
C10orf143	10	130020025	130110817	0.063874692	PASS
C10orf53	10	49679651	49710261	-0.404852538	PASS
C10orf55	10	73909969	73922777	0.072405807	PASS
C10orf62	10	97589727	97590934	-0.367947924	PASS
C10orf67	10	23201916	23344845	0.039811807	PASS
C10orf71	10	49299193	49327487	-0.404852538	PASS
C10orf82	10	116663696	116670264	0.063874692	PASS
C10orf88	10	122930903	122954403	0.063874692	PASS
C10orf90	10	126424997	126670446	0.063874692	PASS
C10orf95	10	102449837	102451543	-0.367947924	PASS
C10orf99	10	84173738	84185294	0.072405807	PASS
C11orf1	11	111878935	111885975	-0.356188624	PASS
C11orf16	11	8920076	8933006	-0.405444717	PASS
C11orf21	11	2295645	2303049	-0.333835828	PASS
C11orf24	11	68261335	68272001	0.182439925	suppress_segment_too_large
C11orf40	11	4571423	4577820	-0.333835828	PASS
C11orf42	11	6205566	6211132	-0.957525224	PASS
C11orf45	11	128899565	128906035	-0.419612865	PASS

C11orf49	11	46936689	47164385	0.117376172	PASS
C11orf52	11	111918032	111926872	-0.356188624	PASS
C11orf53	11	111245805	111286401	-0.356188624	PASS
C11orf54	11	93741591	93764749	0.062969153	PASS
C11orf58	11	16613132	16756881	-0.405444717	PASS
C11orf65	11	108308519	108467531	-0.356188624	PASS
C11orf68	11	65916808	65919117	0.182439925	suppress_segment_too_large
C11orf71	11	114391443	114400511	-0.356188624	PASS
C11orf74	11	36594493	36659290	-0.405444717	PASS
C11orf80	11	66744451	66843328	0.182439925	suppress_segment_too_large
C11orf86	11	66975277	66977004	0.182439925	suppress_segment_too_large
C11orf87	11	109422120	109429114	-0.356188624	PASS
C11orf88	11	111514785	111537031	-0.356188624	PASS
C11orf91	11	33698261	33700801	-0.405444717	PASS
C11orf94	11	45906513	45907282	0.117376172	PASS
C11orf95	11	63759892	63768775	0.182439925	suppress_segment_too_large
C11orf96	11	43942505	43944338	0.117376172	PASS
C11orf97	11	94512432	94532123	0.062969153	PASS
C11orf98	11	62662816	62665217	0.182439925	suppress_segment_too_large
C12orf10	12	53299686	53307177	-0.202191512	PASS
C12orf29	12	88033846	88050160	0.202141228	PASS
C12orf4	12	4487728	4538508	-0.086128028	PASS
C12orf40	12	39626167	39908300	-0.202191512	PASS
C12orf42	12	103237591	103496010	0.202141228	PASS
C12orf43	12	121000486	121016502	0.202141228	PASS
C12orf45	12	104986310	105074197	0.202141228	PASS
C12orf49	12	116710185	116738070	0.202141228	PASS
C12orf50	12	87980035	88034037	0.202141228	PASS
C12orf54	12	48482498	48496386	-0.202191512	PASS
C12orf56	12	64264762	64391192	-0.202191512	PASS
C12orf57	12	6942978	6946003	-0.086128028	PASS
C12orf60	12	14803572	14906586	0.362961408	suppress_segment_too_large
C12orf65	12	123232916	123257959	0.202141228	PASS
C12orf66	12	64186316	64222296	-0.202191512	PASS
C12orf71	12	27081058	27082514	0.362961408	suppress_segment_too_large
C12orf73	12	103950202	103965708	0.202141228	PASS
C12orf75	12	105235290	105396097	0.202141228	PASS
C12orf76	12	110027028	110073686	0.202141228	PASS
C13orf42	13	51082119	51200252	-0.390786075	PASS
C14orf119	14	23094765	23100462	0.060212819	PASS
C14orf132	14	96039324	96093889	-0.192197564	PASS
C14orf177	14	98711613	98717766	-0.192197564	PASS
C14orf178	14	77760830	77769742	-0.192197564	PASS
C14orf180	14	104579684	104590515	-0.192197564	PASS
C14orf28	14	44897295	44907257	0.060212819	PASS
C14orf39	14	60396469	60515543	0.060212819	PASS

C14orf93	14	22985908	23010166	0.060212819	PASS
C15orf38-/	15	89834308	89912882	-0.697600509	PASS
C15orf39	15	75195643	75212169	-0.697600509	PASS
C15orf40	15	82988441	83011641	-0.697600509	PASS
C15orf41	15	36579611	36810248	-0.396255771	PASS
C15orf48	15	45430529	45448761	-0.396255771	PASS
C15orf53	15	38628005	39180499	-0.396255771	PASS
C15orf61	15	67521068	67530143	-0.697600509	PASS
C15orf62	15	40770080	40772449	-0.396255771	PASS
C15orf65	15	55408548	55418764	-0.697600509	PASS
C16orf45	16	15434295	15625028	0.069936844	PASS
C16orf46	16	81053497	81077267	-0.371357732	PASS
C16orf54	16	29742463	29746006	0.069936844	PASS
C16orf58	16	31489471	31509309	0.069936844	PASS
C16orf70	16	67109958	67148539	-0.371357732	PASS
C16orf71	16	4734272	4749396	0.069936844	PASS
C16orf72	16	9091648	9121640	0.069936844	PASS
C16orf74	16	85690084	85751129	-0.371357732	PASS
C16orf78	16	49373823	49399431	0.069936844	PASS
C16orf86	16	67666816	67668758	-0.371357732	PASS
C16orf87	16	46796607	46831411	0.069936844	PASS
C16orf89	16	5044122	5066110	0.069936844	PASS
C16orf90	16	3493484	3495480	0.069936844	PASS
C16orf91	16	1419744	1420800	0.069936844	PASS
C16orf92	16	30023334	30027736	0.069936844	PASS
C16orf95	16	87083562	87317420	-0.371357732	PASS
C16orf96	16	4556490	4600714	0.069936844	PASS
C17orf100	17	6651718	6693202	0.128406487	PASS
C17orf107	17	4899418	4902932	0.128406487	PASS
C17orf113	17	42038232	42050614	0.060555011	PASS
C17orf47	17	58541587	58544368	0.088516311	PASS
C17orf49	17	7014495	7017525	0.128406487	PASS
C17orf50	17	35760897	35765079	0.060555011	PASS
C17orf51	17	21428381	21574517	0.060555011	PASS
C17orf53	17	44141906	44162476	0.088516311	PASS
C17orf58	17	67991101	67996431	0.088516311	PASS
C17orf64	17	60392429	60431421	0.088516311	PASS
C17orf67	17	56791913	56838773	0.088516311	PASS
C17orf75	17	32324565	32350023	0.060555011	PASS
C17orf78	17	37375986	37392708	0.060555011	PASS
C17orf80	17	73232233	73248947	0.088516311	PASS
C17orf97	17	410327	431062	0.128406487	PASS
C17orf98	17	38835088	38841455	0.060555011	PASS
C17orf99	17	78146353	78166177	0.088516311	PASS
C18orf21	18	35972083	35979286	-0.376397843	PASS
C18orf25	18	46173553	46266991	-0.376397843	PASS

C18orf32	18	49477250	49487252	-0.376397843	PASS
C18orf54	18	54357917	54385218	-0.376397843	PASS
C18orf63	18	74315813	74359187	-0.376397843	PASS
C19orf12	19	29698886	29715789	0.093487731	suppress_segment_too_large
C19orf18	19	57958437	57974534	-0.180829161	PASS
C19orf24	19	1275438	1279249	0.079632875	PASS
C19orf25	19	1461143	1479556	0.079632875	PASS
C19orf33	19	38304161	38305009	0.093487731	suppress_segment_too_large
C19orf38	19	10836575	10869790	0.093487731	suppress_segment_too_large
C19orf44	19	16496311	16521352	0.093487731	suppress_segment_too_large
C19orf47	19	40319536	40348527	0.093487731	suppress_segment_too_large
C19orf48	19	50797704	50804929	-0.016761391	PASS
C19orf53	19	13774168	13778462	0.093487731	suppress_segment_too_large
C19orf54	19	40740856	40751553	0.093487731	suppress_segment_too_large
C19orf57	19	13882348	13906452	0.093487731	suppress_segment_too_large
C19orf66	19	10086122	10093252	0.093487731	suppress_segment_too_large
C19orf67	19	14081619	14085875	0.093487731	suppress_segment_too_large
C19orf70	19	5678421	5680896	0.079632875	PASS
C19orf71	19	3539154	3544030	0.079632875	PASS
C19orf73	19	49118397	49119140	0.14996833	PASS
C19orf81	19	50649445	50659310	-0.016761391	PASS
C19orf84	19	51388289	51390574	-0.016761391	PASS
C1D	2	68041130	68110948	-0.372959126	PASS
C1GALT1	7	7156934	7248651	-0.69513118	PASS
C1GALT1(	23	120625793	120630150	-0.012567603	PASS
C1GALT1(	2	43675151	43676322	0.040498426	PASS
C1QA	1	22636506	22639608	0.033763446	PASS
C1QB	1	22652762	22661538	0.033763446	PASS
C1QBP	17	5432777	5448830	0.128406487	PASS
C1QC	1	22643630	22648110	0.033763446	PASS
C1QL1	17	44959693	44968071	0.088516311	PASS
C1QL2	2	119156243	119158889	-0.475215623	PASS
C1QL3	10	16513743	16522005	0.039811807	PASS
C1QL4	12	49332411	49337188	-0.202191512	PASS
C1QTNF1	17	79022814	79049788	0.088516311	PASS
C1QTNF12	1	1242446	1246722	0.373964873	suppress_segment_too_large
C1QTNF2	5	160347751	160370641	-0.150979337	PASS
C1QTNF3	5	34019448	34043832	0.055007813	PASS
C1QTNF3-	5	33987174	34124528	0.055007813	PASS
C1QTNF4	11	47589664	47594659	0.117376172	PASS
C1QTNF5	11	119338939	119340940	-0.356188624	PASS
C1QTNF6	22	37180167	37199385	-0.575930959	PASS
C1QTNF7	4	15339818	15446166	0.279089364	PASS
C1QTNF8	16	1090005	1096244	0.069936844	PASS
C1QTNF9	13	24307166	24322535	-0.390786075	PASS
C1QTNF9I	13	23890525	23902655	-0.390786075	PASS

C1R	12	7080199	7092607	-0.086128028	PASS
C1RL	12	7089587	7109273	-0.086128028	PASS
C1S	12	6988259	7071032	-0.086128028	PASS
C1orf100	1	244352635	244389663	-0.368111568	PASS
C1orf105	1	172420688	172468831	-0.027073548	PASS
C1orf109	1	37681570	37692249	0.033763446	PASS
C1orf112	1	169662007	169854080	-0.027073548	PASS
C1orf115	1	220689845	220699157	-0.256698369	PASS
C1orf116	1	207018521	207032756	-0.256698369	PASS
C1orf122	1	37806979	37809454	0.033763446	PASS
C1orf123	1	53214099	53220617	0.033763446	PASS
C1orf127	1	10946471	10982037	0.373964873	suppress_segment_too_large
C1orf131	1	231223763	231241187	-0.256698369	PASS
C1orf141	1	67092165	67231853	0.033763446	PASS
C1orf146	1	92217940	92245813	0.033763446	PASS
C1orf158	1	12746215	12763699	-0.415253068	PASS
C1orf159	1	1081818	1116361	0.373964873	suppress_segment_too_large
C1orf162	1	111473792	111478512	0.033763446	PASS
C1orf167	1	11761787	11789585	0.373964873	suppress_segment_too_large
C1orf174	1	3889125	3900293	0.373964873	suppress_segment_too_large
C1orf185	1	51102221	51148086	0.033763446	PASS
C1orf189	1	154199372	154206333	0.103015788	PASS
C1orf194	1	109105951	109113857	0.033763446	PASS
C1orf198	1	230837119	230869589	-0.256698369	PASS
C1orf21	1	184387058	184629020	-0.027073548	PASS
C1orf210	1	43281883	43285617	0.033763446	PASS
C1orf216	1	35713875	35719472	0.033763446	PASS
C1orf226	1	162366908	162386818	-0.027073548	PASS
C1orf232	1	26164161	26168581	0.033763446	PASS
C1orf35	1	228100726	228105411	-0.256698369	PASS
C1orf43	1	154206696	154220637	0.103015788	PASS
C1orf50	1	42767269	42779488	0.033763446	PASS
C1orf52	1	85249953	85259672	0.033763446	PASS
C1orf53	1	197902647	197907367	-0.027073548	PASS
C1orf54	1	150268200	150280916	0.103015788	PASS
C1orf56	1	151047740	151051986	0.103015788	PASS
C1orf61	1	156404250	156430701	0.103015788	PASS
C1orf68	1	152719522	152720470	0.103015788	PASS
C1orf74	1	209779208	209784559	-0.256698369	PASS
C1orf87	1	59987269	60073770	0.033763446	PASS
C1orf94	1	34166883	34219131	0.033763446	PASS
C2	6	31897785	31945672	0.048820648	PASS
C20orf141	20	2814987	2815833	-0.168668583	PASS
C20orf144	20	33662284	33665619	-0.168668583	PASS
C20orf173	20	35523186	35529652	-0.168668583	PASS
C20orf194	20	3249305	3407625	-0.168668583	PASS

C20orf202	20	1203454	1208274	-0.168668583	PASS
C20orf203	20	32631652	32673941	-0.168668583	PASS
C20orf204	20	64034344	64039962	-0.168668583	PASS
C20orf27	20	3753508	3768387	-0.168668583	PASS
C20orf85	20	58150904	58161150	-0.168668583	PASS
C20orf96	20	270863	290778	-0.168668583	PASS
C21orf58	21	46300181	46323875	-0.064425456	suppress_segment_too_large
C21orf62	21	32793564	32813743	0.12860881	suppress_segment_too_large
C21orf91	21	17788967	17819386	0.12860881	suppress_segment_too_large
C22orf15	22	23763021	23765861	-0.575930959	PASS
C22orf23	22	37943050	37953669	-0.575930959	PASS
C22orf31	22	29058672	29061844	-0.575930959	PASS
C22orf39	22	19351368	19448232	-0.575930959	PASS
C22orf42	22	32149006	32159322	-0.575930959	PASS
C22orf46	22	41688939	41698136	-0.575930959	PASS
C2CD2	21	41885112	41953890	-0.064425456	suppress_segment_too_large
C2CD2L	11	119102198	119118544	-0.356188624	PASS
C2CD3	11	74012714	74171210	0.062969153	PASS
C2CD4A	15	62066977	62070917	-0.697600509	PASS
C2CD4B	15	62163535	62165283	-0.697600509	PASS
C2CD4C	19	405438	409170	0.079632875	PASS
C2CD4D	1	151837819	151840557	0.103015788	PASS
C2CD5	12	22448583	22544546	0.362961408	suppress_segment_too_large
C2CD6	2	201487425	201619178	0.025928102	PASS
C2orf15	2	99141485	99151487	0.048238121	PASS
C2orf16	2	27537386	27582721	0.040498426	PASS
C2orf40	2	106063246	106078159	-0.532032081	PASS
C2orf42	2	70149880	70248615	-0.372959126	PASS
C2orf49	2	105337359	105349211	-0.532032081	PASS
C2orf50	2	11133053	11146790	0.040498426	PASS
C2orf66	2	196805002	196810276	0.025928102	PASS
C2orf68	2	85606654	85612066	0.066915641	PASS
C2orf69	2	199911256	199955935	0.025928102	PASS
C2orf70	2	26562582	26579532	0.040498426	PASS
C2orf72	2	231037490	231049719	0.025928102	PASS
C2orf73	2	54330034	54383742	0.040498426	PASS
C2orf74	2	61145068	61164829	-0.372959126	PASS
C2orf76	2	119302225	119366828	-0.475215623	PASS
C2orf78	2	73784189	73817147	-0.372959126	PASS
C2orf80	2	208165343	208190073	0.025928102	PASS
C2orf81	2	74414176	74421591	-0.372959126	PASS
C2orf83	2	227610090	227648606	0.025928102	PASS
C2orf88	2	189879609	190203484	0.025928102	PASS
C2orf91	2	41935368	41956806	0.040498426	PASS
C3	19	6677704	6730562	0.079632875	PASS
C3AR1	12	8058302	8066471	-0.086128028	PASS

C3orf14	3	62318973	62336213	-0.234617007	PASS
C3orf18	3	50558025	50571027	-0.234617007	PASS
C3orf20	3	14675099	14773034	0.311018244	suppress_segment_too_large
C3orf22	3	126526999	126558965	0.313454204	suppress_segment_too_large
C3orf30	3	119146150	119160042	0.313454204	suppress_segment_too_large
C3orf33	3	155762617	155806351	0.318278996	suppress_segment_too_large
C3orf38	3	88149743	88168729	0.313454204	suppress_segment_too_large
C3orf49	3	63819362	63848636	-0.234617007	PASS
C3orf52	3	112086335	112131004	0.313454204	suppress_segment_too_large
C3orf56	3	127193131	127198185	0.313454204	suppress_segment_too_large
C3orf58	3	143971798	144048719	0.318278996	suppress_segment_too_large
C3orf62	3	49268602	49277909	-0.234617007	PASS
C3orf67	3	58717365	59050084	-0.234617007	PASS
C3orf70	3	185078050	185153014	0.318278996	suppress_segment_too_large
C3orf80	3	160225496	160228213	0.318278996	suppress_segment_too_large
C3orf84	3	49177632	49191858	-0.234617007	PASS
C3orf85	3	109118252	109150514	0.313454204	suppress_segment_too_large
C4A	6	31982024	32002681	0.048820648	PASS
C4B	6	32014795	32035418	0.048820648	PASS
C4BPA	1	207104262	207144972	-0.256698369	PASS
C4BPB	1	207088842	207099993	-0.256698369	PASS
C4orf17	4	99511004	99542303	-0.011382299	PASS
C4orf19	4	37453941	37623495	-0.011382299	PASS
C4orf3	4	119296419	119304445	-0.011382299	PASS
C4orf33	4	129093317	129116640	-0.011382299	PASS
C4orf36	4	86876205	86892422	-0.011382299	PASS
C4orf45	4	158893134	159038760	-0.011382299	PASS
C4orf46	4	158666679	158672255	-0.011382299	PASS
C4orf47	4	185426249	185449826	-0.462744716	PASS
C4orf48	4	2041993	2043970	0.279089364	PASS
C4orf50	4	5897373	6200555	0.279089364	PASS
C4orf51	4	145680115	145771032	-0.011382299	PASS
C4orf54	4	99636529	99654648	-0.011382299	PASS
C5	9	120952335	121050276	0.365190713	suppress_segment_too_large
C5AR1	19	47290023	47322066	0.14996833	PASS
C5AR2	19	47332147	47347327	0.14996833	PASS
C5orf15	5	133955502	133968787	-0.293443042	PASS
C5orf22	5	31532266	31555058	0.055007813	PASS
C5orf24	5	134845680	134859737	-0.293443042	PASS
C5orf30	5	103258702	103278660	-0.293443042	PASS
C5orf34	5	43486701	43515145	-0.293443042	PASS
C5orf38	5	2752131	2755397	0.055007813	PASS
C5orf46	5	147880726	147906538	-0.293443042	PASS
C5orf47	5	173973779	174006140	-0.150979337	PASS
C5orf49	5	7830378	7851490	0.055007813	PASS
C5orf51	5	41904188	41921636	-0.293443042	PASS

C5orf52	5	157671553	157680143		-0.150979337	PASS
C5orf58	5	170232447	170252575		-0.150979337	PASS
C5orf60	5	179641544	179645046		-0.150979337	PASS
C5orf63	5	127042558	127073492		-0.293443042	PASS
C5orf67	5	56511567	56606232		-0.293443042	PASS
C6	5	41142234	41261438		-0.293443042	PASS
C6orf10	6	32288526	32371912		0.048820648	PASS
C6orf106	6	34587288	34696859		0.048820648	PASS
C6orf118	6	165279664	165309607		-0.035448111	PASS
C6orf120	6	169702190	169704856		-0.035448111	PASS
C6orf132	6	42101118	42142619		0.048820648	PASS
C6orf136	6	30647039	30653210		0.048820648	PASS
C6orf141	6	49550646	49561907		0.048820648	PASS
C6orf15	6	31111223	31112559		0.048820648	PASS
C6orf163	6	87344849	87365463		-0.368590549	PASS
C6orf201	6	4079206	4130951		0.048820648	PASS
C6orf203	6	107028213	107051342		-0.368590549	PASS
C6orf222	6	36315757	36336885		0.048820648	PASS
C6orf223	6	44000580	44005958		0.048820648	PASS
C6orf226	6	42890265	42890816		0.048820648	PASS
C6orf47	6	31658298	31660772		0.048820648	PASS
C6orf48	6	31834608	31839766		0.048820648	PASS
C6orf52	6	10671418	10694797		0.048820648	PASS
C6orf58	6	127519455	127591817		-0.458570262	PASS
C6orf62	6	24704861	24720836		0.048820648	PASS
C6orf89	6	36871870	36928964		0.048820648	PASS
C7	5	40909252	40982939		-0.293443042	PASS
C7orf25	7	42908726	42912305		-0.69513118	PASS
C7orf26	7	6590017	6608726		-0.69513118	PASS
C7orf31	7	25134697	25180356		-0.69513118	PASS
C7orf33	7	148590565	148615860		-0.388946609	PASS
C7orf43	7	100154420	100158715		-0.262778904	PASS
C7orf50	7	996986	1138260		-0.69513118	PASS
C7orf55-LU	7	139341360	139422599		-0.388946609	PASS
C7orf57	7	48035511	48061304		-0.69513118	PASS
C7orf61	7	100456615	100464271		-0.262778904	PASS
C7orf77	7	124777292	124790810		-0.388946609	PASS
C8A	1	56854806	56918221		0.033763446	PASS
C8B	1	56929210	56966140		0.033763446	PASS
C8G	9	136945185	136946975		0.365190713	suppress_segment_too_large
C8orf33	8	145052378	145066685		0.307788765	PASS
C8orf34	8	68330722	68819022		-0.201142258	PASS
C8orf37	8	95244919	95269201		0.279445683	PASS
C8orf44	8	66667615	66685564		-0.201142258	PASS
C8orf48	8	13566843	13568287		-0.345962679	PASS
C8orf58	8	22599601	22604150		-0.345962679	PASS

C8orf59	8	85214076	85220421	0.279445683	PASS
C8orf74	8	10672637	10700593	-0.345962679	PASS
C8orf76	8	123219957	123241398	0.279445683	PASS
C8orf82	8	144525733	144529132	0.307788765	PASS
C8orf86	8	38510834	38560939	-0.201142258	PASS
C8orf88	8	90958637	90985257	0.279445683	PASS
C8orf89	8	73241329	73259502	-0.201142258	PASS
C9	5	39284262	39424868	0.055007813	PASS
C9orf116	9	135495181	135501734	0.365190713	suppress_segment_too_large
C9orf129	9	93318199	93346414	0.365190713	suppress_segment_too_large
C9orf131	9	35041095	35045991	-0.310444659	PASS
C9orf135	9	69820793	69906232	0.365190713	suppress_segment_too_large
C9orf152	9	110190048	110208189	0.365190713	suppress_segment_too_large
C9orf153	9	86220265	86259657	0.365190713	suppress_segment_too_large
C9orf16	9	128160260	128163928	0.365190713	suppress_segment_too_large
C9orf24	9	34379019	34397832	-0.310444659	PASS
C9orf3	9	94726701	95087218	0.365190713	suppress_segment_too_large
C9orf40	9	74946581	74952886	0.365190713	suppress_segment_too_large
C9orf43	9	113410054	113429684	0.365190713	suppress_segment_too_large
C9orf50	9	129612225	129620776	0.365190713	suppress_segment_too_large
C9orf57	9	72051376	72072721	0.365190713	suppress_segment_too_large
C9orf64	9	83938311	83956986	0.365190713	suppress_segment_too_large
C9orf66	9	212824	215893	-0.310444659	PASS
C9orf72	9	27546545	27573866	-0.310444659	PASS
C9orf78	9	129827290	129835863	0.365190713	suppress_segment_too_large
C9orf84	9	111686173	111795008	0.365190713	suppress_segment_too_large
C9orf85	9	71911510	71986054	0.365190713	suppress_segment_too_large
C9orf92	9	16203935	16410990	-0.310444659	PASS
CA1	8	85327608	85379014	0.279445683	PASS
CA10	17	51630313	52160017	0.088516311	PASS
CA11	19	48637942	48646312	0.14996833	PASS
CA12	15	63321378	63382161	-0.697600509	PASS
CA13	8	85220587	85284073	0.279445683	PASS
CA14	1	150257159	150265078	0.103015788	PASS
CA2	8	85463852	85481493	0.279445683	PASS
CA3	8	85373436	85449040	0.279445683	PASS
CA4	17	60149936	60170899	0.088516311	PASS
CA5A	16	87881546	87936580	-0.371357732	PASS
CA5B	23	15688830	15788409	-0.012567603	PASS
CA6	1	8945867	8975092	0.373964873	suppress_segment_too_large
CA7	16	66844379	66854153	-0.371357732	PASS
CA8	8	60187347	60281412	-0.201142258	PASS
CA9	9	35673856	35681159	-0.310444659	PASS
CAAP1	9	26840685	26892804	-0.310444659	PASS
CAB39	2	230712845	230821075	0.025928102	PASS
CAB39L	13	49308650	49444126	-0.390786075	PASS

CABCOCC	10	61662961	61766766	0.072405807	PASS
CABIN1	22	24011192	24178628	-0.575930959	PASS
CABLES1	18	23134564	23260467	-0.376397843	PASS
CABLES2	20	62388632	62407285	-0.168668583	PASS
CABP1	12	120640552	120667324	0.202141228	PASS
CABP2	11	67518912	67524517	0.182439925	suppress_segment_too_large
CABP4	11	67452406	67460313	0.182439925	suppress_segment_too_large
CABP5	19	48029953	48044053	0.14996833	PASS
CABP7	22	29720084	29731839	-0.575930959	PASS
CABS1	4	70334966	70337116	-0.011382299	PASS
CABYR	18	24138956	24161603	-0.376397843	PASS
CACFD1	9	133459965	133470848	0.365190713	suppress_segment_too_large
CACHD1	1	64470792	64693058	0.033763446	PASS
CACNA1A	19	13206442	13633025	0.093487731	suppress_segment_too_large
CACNA1B	9	137877789	138124624	0.365190713	suppress_segment_too_large
CACNA1C	12	1970786	2697950	-0.086128028	PASS
CACNA1D	3	53328963	53813733	-0.234617007	PASS
CACNA1E	1	181317690	181808084	-0.027073548	PASS
CACNA1F	23	49205063	49233371	-0.012567603	PASS
CACNA1G	17	50561068	50627474	0.088516311	PASS
CACNA1H	16	1153121	1221772	0.069936844	PASS
CACNA1I	22	39570753	39689737	-0.575930959	PASS
CACNA1S	1	201039512	201112566	-0.256698369	PASS
CACNA2D	7	81946444	82443798	-0.262778904	PASS
CACNA2D	3	50362799	50504244	-0.234617007	PASS
CACNA2D	3	54122547	55074557	-0.234617007	PASS
CACNA2D	12	1791957	1918836	-0.086128028	PASS
CACNB1	17	39173456	39197703	0.060555011	PASS
CACNB2	10	18140677	18543557	0.039811807	PASS
CACNB3	12	48813794	48828941	-0.202191512	PASS
CACNB4	2	151832768	152099475	-0.475215623	PASS
CACNG1	17	67044590	67056797	0.088516311	PASS
CACNG2	22	36563921	36703558	-0.575930959	PASS
CACNG3	16	24255553	24362801	0.069936844	PASS
CACNG4	17	66964910	67033398	0.088516311	PASS
CACNG5	17	66835117	66885486	0.088516311	PASS
CACNG6	19	53992288	54012669	0.206912349	PASS
CACNG7	19	53909335	53943941	0.206912349	PASS
CACNG8	19	53963040	53990215	0.206912349	PASS
CACTIN	19	3610641	3626815	0.079632875	PASS
CACUL1	10	118674167	118755249	0.063874692	PASS
CACYBP	1	174999163	175011715	-0.027073548	PASS
CAD	2	27217390	27243943	0.040498426	PASS
CADM1	11	115169218	115504957	-0.356188624	PASS
CADM2	3	84958981	86074429	0.313454204	suppress_segment_too_large
CADM3	1	159171609	159203313	-0.467855874	PASS

CADM4	19	43622368	43639839	0.14996833	PASS
CADPS	3	62398346	62875389	-0.234617007	PASS
CADPS2	7	122318425	122886759	-0.388946609	PASS
CAGE1	6	7326656	7389743	0.048820648	PASS
CALB1	8	90058608	90095475	0.279445683	PASS
CALB2	16	71358713	71390438	-0.371357732	PASS
CALCA	11	14966668	14972354	-0.405444717	PASS
CALCB	11	14904997	15082342	-0.405444717	PASS
CALCOCC	12	53708517	53727745	-0.202191512	PASS
CALCOCC	17	48830988	48866522	0.088516311	PASS
CALCR	7	93424487	93574730	-0.262778904	PASS
CALCRL	2	187343129	187448460	0.025928102	PASS
CALD1	7	134744252	134970728	-0.388946609	PASS
CALHM1	10	103453387	103458888	-0.367947924	PASS
CALHM2	10	103446786	103452402	-0.367947924	PASS
CALHM3	10	103472804	103479240	-0.367947924	PASS
CALHM4	6	116529013	116558868	-0.368590549	PASS
CALHM5	6	116511646	116524792	-0.368590549	PASS
CALHM6	6	116461370	116463779	-0.368590549	PASS
CALM1	14	90396502	90408261	-0.192197564	PASS
CALM2	2	47160082	47176601	0.040498426	PASS
CALM3	19	46601074	46610793	0.14996833	PASS
CALML3	10	5524009	5526771	0.039811807	PASS
CALML4	15	68190705	68206110	-0.697600509	PASS
CALML5	10	5498697	5499555	0.039811807	PASS
CALML6	1	1914827	1917296	0.373964873	suppress_segment_too_large
CALN1	7	71779491	72447151	-0.750061359	PASS
CALR	19	12938578	12944489	0.093487731	suppress_segment_too_large
CALR3	19	16479057	16496192	0.093487731	suppress_segment_too_large
CALU	7	128739292	128771807	-0.388946609	PASS
CALY	10	133324072	133336935	0.063874692	PASS
CAMK1	3	9757342	9769992	0.311018244	suppress_segment_too_large
CAMK1D	10	12349482	12835545	0.039811807	PASS
CAMK1G	1	209583717	209613938	-0.256698369	PASS
CAMK2A	5	150219491	150290291	-0.293443042	PASS
CAMK2B	7	44217150	44334577	-0.69513118	PASS
CAMK2D	4	113451032	113761927	-0.011382299	PASS
CAMK2G	10	73812501	73874591	0.072405807	PASS
CAMK2N1	1	20482391	20486220	0.033763446	PASS
CAMK2N2	3	184259213	184261463	0.318278996	suppress_segment_too_large
CAMK4	5	111223653	111494886	-0.293443042	PASS
CAMKK1	17	3860315	3894891	0.128406487	PASS
CAMKK2	12	121237691	121298308	0.202141228	PASS
CAMKMT	2	44361950	44772592	0.040498426	PASS
CAMKV	3	49857988	49870222	-0.234617007	PASS
CAMLG	5	134738501	134752160	-0.293443042	PASS

CAMP	3	48223347	48225491	-0.234617007	PASS
CAMSAP1	9	135808487	135907228	0.365190713	suppress_segment_too_large
CAMSAP2	1	200739558	200860704	-0.256698369	PASS
CAMSAP3	19	7595902	7618304	0.079632875	PASS
CAMTA1	1	6785324	7769706	0.373964873	suppress_segment_too_large
CAMTA2	17	4967992	4987652	0.128406487	PASS
CAND1	12	67269281	67319951	-0.202191512	PASS
CAND2	3	12796472	12871916	0.311018244	suppress_segment_too_large
CANT1	17	78991717	79009867	0.088516311	PASS
CANX	5	179678628	179730925	-0.150979337	PASS
CAP1	1	40040233	40072649	0.033763446	PASS
CAP2	6	17393216	17557792	0.048820648	PASS
CAPG	2	85394748	85418432	0.066915641	PASS
CAPN1	11	65180566	65212006	0.182439925	suppress_segment_too_large
CAPN10	2	240586716	240617705	0.025928102	PASS
CAPN11	6	44158811	44184402	0.048820648	PASS
CAPN12	19	38730187	38769904	0.093487731	suppress_segment_too_large
CAPN13	2	30722771	30820542	0.040498426	PASS
CAPN14	2	31173056	31233858	0.040498426	PASS
CAPN15	16	527717	554636	0.069936844	PASS
CAPN2	1	223701593	223776018	-0.256698369	PASS
CAPN3	15	42359500	42412318	-0.396255771	PASS
CAPN5	11	77066941	77126155	0.062969153	PASS
CAPN6	23	111245103	111270523	-0.012567603	PASS
CAPN7	3	15206152	15252918	0.311018244	suppress_segment_too_large
CAPN8	1	223538007	223665734	-0.256698369	PASS
CAPN9	1	230747384	230802003	-0.256698369	PASS
CAPNS1	19	36139575	36150353	0.093487731	suppress_segment_too_large
CAPNS2	16	55566672	55567687	-0.371357732	PASS
CAPRIN1	11	34051683	34101156	-0.405444717	PASS
CAPRIN2	12	30709552	30754951	0.362961408	suppress_segment_too_large
CAPS	19	5911707	5915877	0.079632875	PASS
CAPS2	12	75275979	75390928	0.202141228	PASS
CAPSL	5	35904295	35938779	0.055007813	PASS
CAPZA1	1	112619173	112671619	0.033763446	PASS
CAPZA2	7	116811070	116922049	-0.388946609	PASS
CAPZA3	12	18738101	18739187	0.362961408	suppress_segment_too_large
CAPZB	1	19338776	19485539	0.033763446	PASS
CARD10	22	37490362	37519542	-0.575930959	PASS
CARD11	7	2906141	3043945	-0.69513118	PASS
CARD14	17	80169992	80209331	0.088516311	PASS
CARD16	11	105041326	105101431	0.062969153	PASS
CARD17	11	105092469	105101431	0.062969153	PASS
CARD18	11	105137721	105531697	0.062969153	PASS
CARD19	9	93096218	93113283	0.365190713	suppress_segment_too_large
CARD6	5	40841184	40860175	-0.293443042	PASS

CARD8	19	48180770	48255946	0.14996833	PASS
CARD9	9	136361903	136373681	0.365190713	suppress_segment_too_large
CARF	2	202912214	202987063	0.025928102	PASS
CARHSP1	16	8852942	8869012	0.069936844	PASS
CARM1	19	10871513	10923070	0.093487731	suppress_segment_too_large
CARMIL1	6	25279078	25620530	0.048820648	PASS
CARMIL2	16	67644919	67657569	-0.371357732	PASS
CARMIL3	14	24052000	24069728	0.060212819	PASS
CARNMT1	9	74981020	75028423	0.365190713	suppress_segment_too_large
CARNS1	11	67414968	67425607	0.182439925	suppress_segment_too_large
CARS	11	3000922	3057613	-0.333835828	PASS
CARS2	13	110641412	110713603	-0.390786075	PASS
CARTPT	5	71719163	71721048	-0.293443042	PASS
CASC1	12	25108420	25195162	0.362961408	suppress_segment_too_large
CASC10	10	21492658	21497262	0.039811807	PASS
CASC3	17	40140318	40172183	0.060555011	PASS
CASC4	15	44288729	44415758	-0.396255771	PASS
CASD1	7	94509219	94557019	-0.262778904	PASS
CASK	23	41514934	41923645	-0.012567603	PASS
CASKIN1	16	2177180	2196525	0.069936844	PASS
CASKIN2	17	75500261	75515583	0.088516311	PASS
CASP1	11	105025443	105035250	0.062969153	PASS
CASP10	2	201182881	201229406	0.025928102	PASS
CASP14	19	15049384	15058293	0.093487731	suppress_segment_too_large
CASP2	7	143288215	143307696	-0.388946609	PASS
CASP3	4	184627696	184649509	-0.462744716	PASS
CASP4	11	104942866	104969436	0.062969153	PASS
CASP5	11	104994235	105023168	0.062969153	PASS
CASP6	4	109688622	109703583	-0.011382299	PASS
CASP7	10	113679162	113730907	0.063874692	PASS
CASP8	2	201233443	201287711	0.025928102	PASS
CASP8AP2	6	89829894	89874436	-0.368590549	PASS
CASP9	1	15490832	15526534	0.033763446	PASS
CASQ1	1	160190556	160201886	-0.027073548	PASS
CASQ2	1	115700007	115768781	0.033763446	PASS
CASR	3	122183683	122291629	0.313454204	suppress_segment_too_large
CASS4	20	56412112	56460387	-0.168668583	PASS
CAST	5	96525267	96779595	-0.293443042	PASS
CASTOR1	22	30285117	30289627	-0.575930959	PASS
CASTOR2	7	74964818	75024798	-0.262778904	PASS
CASTOR3	7	100200653	100272232	-0.262778904	PASS
CASZ1	1	10636604	10796650	0.373964873	suppress_segment_too_large
CAT	11	34438925	34472062	-0.405444717	PASS
CATIP	2	218356856	218368099	0.025928102	PASS
CATSPER1	11	66016752	66026517	0.182439925	suppress_segment_too_large
CATSPER2	15	43628503	43668118	-0.396255771	PASS

CATSPER3	5	134967906	135011707	-0.293443042	PASS
CATSPER4	1	26190561	26202968	0.033763446	PASS
CATSPERB	14	91580696	91780707	-0.192197564	PASS
CATSPERD	19	5720677	5778734	0.079632875	PASS
CATSPERE	1	244454377	244641177	-0.368111568	PASS
CATSPERG	19	38335775	38370943	0.093487731	suppress_segment_too_large
CATSPERZ	11	64300391	64304770	0.182439925	suppress_segment_too_large
CAV1	7	116524785	116561184	-0.388946609	PASS
CAV2	7	116287380	116508541	-0.388946609	PASS
CAV3	3	8733800	8841808	0.311018244	suppress_segment_too_large
CAVIN1	17	42402452	42423517	0.088516311	PASS
CAVIN2	2	191834302	191847255	0.025928102	PASS
CAVIN3	11	6318946	6320647	-0.405444717	PASS
CAVIN4	9	100578079	100587906	0.365190713	suppress_segment_too_large
CBARP	19	1228287	1238027	0.079632875	PASS
CBFA2T2	20	33490075	33650036	-0.168668583	PASS
CBFA2T3	16	88874858	88977204	-0.371357732	PASS
CBFB	16	67029116	67101058	-0.371357732	PASS
CBL	11	119206276	119313926	-0.356188624	PASS
CBLB	3	105655461	105869552	0.313454204	suppress_segment_too_large
CBLC	19	44777869	44800652	0.14996833	PASS
CBLL1	7	107743697	107761667	-0.388946609	PASS
CBLL2	23	22272943	22274461	-0.012567603	PASS
CBLN1	16	49277917	49281831	0.069936844	PASS
CBLN2	18	72536680	72638521	-0.376397843	PASS
CBLN3	14	24426532	24430954	0.060212819	PASS
CBLN4	20	55997440	56005472	-0.168668583	PASS
CBR1	21	36069941	36073166	-0.064425456	suppress_segment_too_large
CBR3	21	36134912	36146566	-0.064425456	suppress_segment_too_large
CBR4	4	168863770	169010275	-0.011382299	PASS
CBS	21	43053191	43076943	-0.064425456	suppress_segment_too_large
CBSL	21	6444869	6468040	-0.304975024	PASS
CBWD1	9	121038	179147	-0.310444659	PASS
CBWD2	2	113437691	113496189	-0.010901494	PASS
CBWD3	9	68232003	68300015	-0.310444659	PASS
CBWD5	9	65668805	65734041	-0.310444659	PASS
CBWD6	9	41131306	41199261	-0.310444659	PASS
CBX1	17	48070052	48101521	0.088516311	PASS
CBX2	17	79778132	79787983	0.088516311	PASS
CBX3	7	26201162	26213356	-0.69513118	PASS
CBX4	17	79833156	79839429	0.088516311	PASS
CBX5	12	54230940	54280133	-0.202191512	PASS
CBX6	22	38861450	38872314	-0.575930959	PASS
CBX7	22	39120167	39152674	-0.575930959	PASS
CBX8	17	79792132	79801683	0.088516311	PASS
CBY1	22	38656636	38673854	-0.575930959	PASS

CBY3	5	179678583	179680974	-0.150979337	PASS
CC2D1A	19	13906201	13930879	0.093487731	suppress_segment_too_large
CC2D1B	1	52345723	52366193	0.033763446	PASS
CC2D2A	4	15469865	15601557	0.279089364	PASS
CC2D2B	10	95907603	96032684	-0.39678585	PASS
CCAR1	10	68721012	68792377	0.072405807	PASS
CCAR2	8	22604632	22621514	-0.345962679	PASS
CCBE1	18	59430939	59697662	-0.376397843	PASS
CCDC102A	16	57512178	57536599	-0.371357732	PASS
CCDC102B	18	68715209	69055189	-0.376397843	PASS
CCDC103	17	44899142	44905390	0.088516311	PASS
CCDC105	19	15010744	15023269	0.093487731	suppress_segment_too_large
CCDC106	19	55641062	55653161	0.121336758	PASS
CCDC107	9	35658290	35661511	-0.310444659	PASS
CCDC110	4	185445182	185471759	-0.462744716	PASS
CCDC112	5	115267188	115296831	-0.293443042	PASS
CCDC113	16	58231157	58283836	-0.371357732	PASS
CCDC114	19	48296457	48321894	0.14996833	PASS
CCDC115	2	130338241	130342349	-0.475215623	PASS
CCDC116	22	21632716	21637327	-0.575930959	PASS
CCDC117	22	28772674	28789301	-0.575930959	PASS
CCDC12	3	46921726	46982010	-0.234617007	PASS
CCDC120	23	49053572	49069857	-0.012567603	PASS
CCDC121	2	27625639	27629012	0.040498426	PASS
CCDC122	13	43823909	43879727	-0.390786075	PASS
CCDC124	19	17933016	17943991	0.093487731	suppress_segment_too_large
CCDC125	5	69280175	69332809	-0.293443042	PASS
CCDC126	7	23597379	23644708	-0.69513118	PASS
CCDC127	5	196871	218215	0.055007813	PASS
CCDC13	3	42708269	42773253	-0.234617007	PASS
CCDC130	19	13731760	13763296	0.093487731	suppress_segment_too_large
CCDC134	22	41800679	41826299	-0.575930959	PASS
CCDC136	7	128790757	128822132	-0.388946609	PASS
CCDC137	17	81666364	81673904	0.088516311	PASS
CCDC138	2	108786757	108885477	-0.010901494	PASS
CCDC14	3	123897305	123961408	0.313454204	suppress_segment_too_large
CCDC140	2	222298147	222305217	0.025928102	PASS
CCDC141	2	178829757	179050086	0.039726562	PASS
CCDC142	2	74471986	74483408	-0.372959126	PASS
CCDC144A	17	16689537	16777881	0.024610076	PASS
CCDC144N	17	20836447	20896140	0.024610076	PASS
CCDC146	7	77122434	77329533	-0.262778904	PASS
CCDC148	2	158171081	158456753	0.039726562	PASS
CCDC149	4	24806117	24980204	0.279089364	PASS
CCDC15	11	124954121	125041489	-0.419612865	PASS
CCDC150	2	196639554	196763490	0.025928102	PASS

CCDC151	19	11420604	11435782	0.093487731	suppress_segment_too_large
CCDC152	5	42756801	42802360	-0.293443042	PASS
CCDC153	11	119189638	119196769	-0.356188624	PASS
CCDC154	16	1434383	1444556	0.069936844	PASS
CCDC155	19	49388218	49417994	0.14996833	PASS
CCDC157	22	30356635	30378658	-0.575930959	PASS
CCDC158	4	76312997	76421868	-0.011382299	PASS
CCDC159	19	11344684	11354944	0.093487731	suppress_segment_too_large
CCDC160	23	134237047	134246207	-0.012567603	PASS
CCDC163	1	45493866	45500079	0.033763446	PASS
CCDC166	8	143706694	143708109	0.307788765	PASS
CCDC167	6	37482920	37499922	0.048820648	PASS
CCDC168	13	102729369	102759070	-0.390786075	PASS
CCDC169	13	36222008	36297840	-0.390786075	PASS
CCDC169-	13	36168794	36297842	-0.390786075	PASS
CCDC17	1	45620044	45624057	0.033763446	PASS
CCDC170	6	151494030	151621193	-0.035448111	PASS
CCDC171	9	15553086	16061663	-0.310444659	PASS
CCDC172	10	116324428	116380029	0.063874692	PASS
CCDC173	2	169645425	169694433	0.039726562	PASS
CCDC174	3	14651746	14672659	0.311018244	suppress_segment_too_large
CCDC175	14	59504539	59576831	0.060212819	PASS
CCDC177	14	69569816	69574837	0.060212819	PASS
CCDC178	18	32937402	33441101	-0.376397843	PASS
CCDC179	11	22846931	22860426	-0.405444717	PASS
CCDC18	1	93179919	93278730	0.033763446	PASS
CCDC180	9	97307304	97378524	0.365190713	suppress_segment_too_large
CCDC181	1	169394870	169460669	-0.027073548	PASS
CCDC182	17	57744481	57745312	0.088516311	PASS
CCDC183	9	136796350	136807741	0.365190713	suppress_segment_too_large
CCDC184	12	48183584	48185926	-0.202191512	PASS
CCDC185	1	223393417	223395470	-0.256698369	PASS
CCDC186	10	114120862	114174232	0.063874692	PASS
CCDC187	9	136249971	136306901	0.365190713	suppress_segment_too_large
CCDC188	22	20148427	20151065	-0.575930959	PASS
CCDC189	16	30757423	30762710	0.069936844	PASS
CCDC190	1	162824458	162868815	-0.027073548	PASS
CCDC191	3	113964137	114056613	0.313454204	suppress_segment_too_large
CCDC192	5	127703389	127941634	-0.293443042	PASS
CCDC196	14	66411704	66498677	0.060212819	PASS
CCDC198	14	57469301	57493867	0.060212819	PASS
CCDC200	17	43216941	43305397	0.088516311	PASS
CCDC22	23	49235467	49250526	-0.012567603	PASS
CCDC24	1	43991359	43996528	0.033763446	PASS
CCDC25	8	27733311	27772653	-0.345962679	PASS
CCDC27	1	3746460	3771645	0.373964873	suppress_segment_too_large

CCDC28A	6	138773509	138793319	-0.035448111	PASS
CCDC28B	1	32200386	32205387	0.033763446	PASS
CCDC3	10	12896625	13099652	0.039811807	PASS
CCDC30	1	42463330	42654664	0.033763446	PASS
CCDC32	15	40528683	40565057	-0.396255771	PASS
CCDC33	15	74202705	74336472	-0.697600509	PASS
CCDC34	11	27330827	27363868	-0.405444717	PASS
CCDC36	3	49198428	49258104	-0.234617007	PASS
CCDC38	12	95867048	95942974	0.202141228	PASS
CCDC39	3	180602858	180870933	0.318278996	suppress_segment_too_large
CCDC40	17	80036632	80100613	0.088516311	PASS
CCDC42	17	8729934	8745219	0.128406487	PASS
CCDC43	17	44673069	44689779	0.088516311	PASS
CCDC47	17	63745250	63776351	0.088516311	PASS
CCDC50	3	191329077	191398670	-0.627320105	PASS
CCDC51	3	48432164	48440456	-0.234617007	PASS
CCDC54	3	107377194	107378637	0.313454204	suppress_segment_too_large
CCDC57	17	82101460	82212830	0.088516311	PASS
CCDC58	3	122359591	122383231	0.313454204	suppress_segment_too_large
CCDC59	12	82223681	82358805	0.202141228	PASS
CCDC6	10	59788763	59906656	0.072405807	PASS
CCDC60	12	119334712	119541047	0.202141228	PASS
CCDC61	19	45995461	46021318	0.14996833	PASS
CCDC62	12	122774327	122827528	0.202141228	PASS
CCDC63	12	110846769	110907535	0.202141228	PASS
CCDC65	12	48904110	48931840	-0.202191512	PASS
CCDC66	3	56557161	56621818	-0.234617007	PASS
CCDC68	18	54901509	54959508	-0.376397843	PASS
CCDC69	5	151181052	151224145	-0.293443042	PASS
CCDC7	10	32446140	32882874	0.039811807	PASS
CCDC70	13	51861981	51866232	-0.390786075	PASS
CCDC71	3	49162535	49166321	-0.234617007	PASS
CCDC71L	7	106656765	106660996	-0.388946609	PASS
CCDC73	11	32602246	32794658	-0.405444717	PASS
CCDC74A	2	131527675	131533666	-0.475215623	PASS
CCDC74B	2	130139287	130145134	-0.475215623	PASS
CCDC77	12	389273	442645	-0.086128028	PASS
CCDC78	16	722582	726954	0.069936844	PASS
CCDC8	19	46410329	46413662	0.14996833	PASS
CCDC80	3	112596794	112649530	0.313454204	suppress_segment_too_large
CCDC81	11	86374736	86423109	0.062969153	PASS
CCDC82	11	96352769	96389956	0.062969153	PASS
CCDC83	11	85855101	85920021	0.062969153	PASS
CCDC84	11	118998142	119015791	-0.356188624	PASS
CCDC85A	2	56184123	56386173	-0.372959126	PASS
CCDC85B	11	65890112	65891635	0.182439925	suppress_segment_too_large

CCDC85C	14	99500180	99604026	-0.192197564	PASS
CCDC86	11	60842071	60851081	0.182439925	suppress_segment_too_large
CCDC87	11	66590176	66593083	0.182439925	suppress_segment_too_large
CCDC88A	2	55287842	55419895	0.040498426	PASS
CCDC88B	11	64340223	64357534	0.182439925	suppress_segment_too_large
CCDC88C	14	91271323	91417844	-0.192197564	PASS
CCDC89	11	85684866	85686277	0.062969153	PASS
CCDC9	19	47255980	47273701	0.14996833	PASS
CCDC90B	11	83259097	83286407	0.062969153	PASS
CCDC91	12	28133249	28581511	0.362961408	suppress_segment_too_large
CCDC92	12	123918660	123972831	0.202141228	PASS
CCDC93	2	117915478	118014133	-0.475215623	PASS
CCDC96	4	7040849	7042939	0.279089364	PASS
CCDC97	19	41310189	41324883	0.093487731	suppress_segment_too_large
CCDC9B	15	40331452	40340967	-0.396255771	PASS
CCER1	12	90905622	90955176	0.202141228	PASS
CCER2	19	38908980	38912158	0.093487731	suppress_segment_too_large
CCHCR1	6	31142439	31158238	0.048820648	PASS
CCIN	9	36169394	36171334	-0.310444659	PASS
CCK	3	42257825	42266207	-0.234617007	PASS
CCKAR	4	26481400	26490462	-0.458181871	PASS
CCKBR	11	6259736	6272127	-0.405444717	PASS
CCL1	17	34360328	34363231	0.060555011	PASS
CCL11	17	34285668	34288334	0.060555011	PASS
CCL13	17	34356452	34358610	0.060555011	PASS
CCL14	17	35983291	35987004	0.060555011	PASS
CCL15	17	35996440	36002038	0.060555011	PASS
CCL15-CC	17	35983656	36001621	0.060555011	PASS
CCL16	17	35976493	35981496	0.060555011	PASS
CCL17	16	57404767	57416062	-0.371357732	PASS
CCL18	17	36064280	36072032	0.060555011	PASS
CCL19	9	34689567	34691277	-0.310444659	PASS
CCL2	17	34255218	34257203	0.060555011	PASS
CCL20	2	227805739	227817564	0.025928102	PASS
CCL21	9	34709005	34710124	-0.310444659	PASS
CCL22	16	57358772	57366190	-0.371357732	PASS
CCL23	17	36013056	36017968	0.060555011	PASS
CCL24	7	75811665	75823356	-0.262778904	PASS
CCL25	19	8052767	8062650	0.079632875	PASS
CCL26	7	75769533	75789896	-0.262778904	PASS
CCL27	9	34661880	34664048	-0.310444659	PASS
CCL28	5	43376645	43412391	-0.293443042	PASS
CCL3	17	36088256	36090169	0.060555011	PASS
CCL3L1	17	36194869	36196758	0.060555011	PASS
CCL4	17	36103590	36105621	0.060555011	PASS
CCL4L2	17	36210924	36212878	0.060555011	PASS

CCL5	17	35871491	35880793	0.060555011	PASS
CCL7	17	34270221	34272242	0.060555011	PASS
CCL8	17	34319036	34321402	0.060555011	PASS
CCM2	7	44999475	45076469	-0.69513118	PASS
CCM2L	20	32010450	32032180	-0.168668583	PASS
CCNA1	13	36431520	36442882	-0.390786075	PASS
CCNA2	4	121816444	121823933	-0.011382299	PASS
CCNB1	5	69167010	69178245	-0.293443042	PASS
CCNB1IP1	14	20311368	20333312	0.060212819	PASS
CCNB2	15	59105078	59125045	-0.697600509	PASS
CCNB3	23	50202713	50351910	-0.012567603	PASS
CCNC	6	99542380	99568973	-0.368590549	PASS
CCND1	11	69641087	69654474	0.182439925	suppress_segment_too_large
CCND2	12	4273772	4305350	-0.086128028	PASS
CCND3	6	41934933	42050357	0.048820648	PASS
CCNDBP1	15	43185118	43197176	-0.396255771	PASS
CCNE1	19	29811898	29824308	0.093487731	suppress_segment_too_large
CCNE2	8	94879770	94896678	0.279445683	PASS
CCNF	16	2429394	2458854	0.069936844	PASS
CCNG1	5	163437569	163446151	-0.150979337	PASS
CCNG2	4	77157151	77433388	-0.011382299	PASS
CCNH	5	87318416	87413019	-0.293443042	PASS
CCNI	4	77047158	77076005	-0.011382299	PASS
CCNI2	5	132747445	132754403	-0.293443042	PASS
CCNJ	10	96043394	96060870	-0.39678585	PASS
CCNJL	5	160249106	160345396	-0.150979337	PASS
CCNK	14	99481169	99535044	-0.192197564	PASS
CCNL1	3	157146508	157160760	0.318278996	suppress_segment_too_large
CCNL2	1	1385711	1399328	0.373964873	suppress_segment_too_large
CCNO	5	55231152	55233680	-0.293443042	PASS
CCNQ	23	153587919	153600045	-0.012567603	PASS
CCNT1	12	48688458	48716998	-0.202191512	PASS
CCNT2	2	134918235	134959342	-0.475215623	PASS
CCNY	10	35247025	35572669	0.039811807	PASS
CCNYL1	2	207711540	207761839	0.025928102	PASS
CCP110	16	19523811	19553408	0.069936844	PASS
CCPG1	15	55340032	55408510	-0.697600509	PASS
CCR1	3	46201709	46208396	-0.234617007	PASS
CCR10	17	42678889	42683917	0.088516311	PASS
CCR2	3	46353734	46360928	-0.234617007	PASS
CCR3	3	46163604	46266706	-0.234617007	PASS
CCR4	3	32951574	32956349	0.311018244	suppress_segment_too_large
CCR5	3	46370854	46376206	-0.234617007	PASS
CCR6	6	167111807	167139696	-0.035448111	PASS
CCR7	17	40553769	40565472	0.060555011	PASS
CCR8	3	39329706	39333511	-0.234617007	PASS

CCR9	3	45886504	45903177		-0.234617007	PASS
CCRL2	3	46407163	46412997		-0.234617007	PASS
CCS	11	66592821	66606019		0.182439925	suppress_segment_too_large
CCSAP	1	229321005	229343294		-0.256698369	PASS
CCSER1	4	90127535	91601913		-0.011382299	PASS
CCSER2	10	84328558	84518521		0.072405807	PASS
CCT2	12	69585334	69601570		-0.202191512	PASS
CCT3	1	156308968	156367873		0.103015788	PASS
CCT4	2	61868089	61888804		-0.372959126	PASS
CCT5	5	10249921	10266412		0.055007813	PASS
CCT6A	7	56051630	56063989		-0.750061359	PASS
CCT6B	17	34927859	34981078		0.060555011	PASS
CCT7	2	73233420	73253021		-0.372959126	PASS
CCT8	21	29055805	29073797		0.12860881	suppress_segment_too_large
CCT8L2	22	16590751	16592810		-0.575930959	PASS
CCZ1	7	5898725	5926550		-0.69513118	PASS
CCZ1B	7	6794134	6826770		-0.69513118	PASS
CD101	1	117001750	117036476		0.033763446	PASS
CD109	6	73695785	73828316		-0.368590549	PASS
CD14	5	140631728	140633701		-0.293443042	PASS
CD151	11	832843	839831		-0.333835828	PASS
CD160	1	145719471	145739288		-0.157385092	PASS
CD163	12	7470813	7503893		-0.086128028	PASS
CD163L1	12	7346685	7479897		-0.086128028	PASS
CD164	6	109366514	109382812		-0.368590549	PASS
CD164L2	1	27379176	27383380		0.033763446	PASS
CD177	19	43353659	43363172		-0.459876623	PASS
CD180	5	67179613	67196799		-0.293443042	PASS
CD19	16	28931939	28939346		0.069936844	PASS
CD1A	1	158254137	158258269		-0.467855874	PASS
CD1B	1	158327951	158331531		-0.467855874	PASS
CD1C	1	158289786	158293630		-0.467855874	PASS
CD1D	1	158179947	158184896		-0.467855874	PASS
CD1E	1	158353696	158357553		-0.467855874	PASS
CD2	1	116754385	116769228		0.033763446	PASS
CD200	3	112332347	112362812		0.313454204	suppress_segment_too_large
CD200R1	3	112921209	112975122		0.313454204	suppress_segment_too_large
CD200R1L	3	112815709	112846856		0.313454204	suppress_segment_too_large
CD207	2	70830214	70835822		-0.372959126	PASS
CD209	19	7739994	7747564		0.079632875	PASS
CD22	19	35319261	35347355		0.093487731	suppress_segment_too_large
CD226	18	69831158	69961803		-0.376397843	PASS
CD24	6	106969831	106975627		-0.368590549	PASS
CD244	1	160830160	160862855		-0.027073548	PASS
CD247	1	167430640	167518610		-0.027073548	PASS
CD248	11	66314487	66317044		0.182439925	suppress_segment_too_large

CD27	12	6444867	6451718	-0.086128028	PASS
CD274	9	5450503	5470566	-0.310444659	PASS
CD276	15	73683966	73714518	-0.697600509	PASS
CD28	2	203706475	203738912	0.025928102	PASS
CD2AP	6	47477789	47627263	0.048820648	PASS
CD2BP2	16	30350766	30355361	0.069936844	PASS
CD300A	17	74466399	74484796	0.088516311	PASS
CD300C	17	74541108	74546143	0.088516311	PASS
CD300E	17	74609887	74623738	0.088516311	PASS
CD300LB	17	74521174	74531474	0.088516311	PASS
CD300LD	17	74579365	74592283	0.088516311	PASS
CD300LF	17	74694311	74712978	0.088516311	PASS
CD300LG	17	43847148	43863629	0.088516311	PASS
CD302	2	159768630	159798255	0.039726562	PASS
CD320	19	8302127	8308356	0.079632875	PASS
CD33	19	51225064	51243860	-0.016761391	PASS
CD34	1	207880972	207911402	-0.256698369	PASS
CD36	7	80369575	80679277	-0.262778904	PASS
CD37	19	49335171	49343335	0.14996833	PASS
CD38	4	15778275	15853230	0.279089364	PASS
CD3D	11	118338954	118342744	-0.356188624	PASS
CD3E	11	118304545	118316175	-0.356188624	PASS
CD3EAP	19	45406209	45410766	0.14996833	PASS
CD3G	11	118344344	118355161	-0.356188624	PASS
CD4	12	6786858	6820808	-0.086128028	PASS
CD40	20	46118272	46129863	-0.168668583	PASS
CD40LG	23	136648193	136660390	-0.012567603	PASS
CD44	11	35138870	35232402	-0.405444717	PASS
CD46	1	207752057	207795513	-0.256698369	PASS
CD47	3	108043094	108091862	0.313454204	suppress_segment_too_large
CD48	1	160678746	160711851	-0.027073548	PASS
CD5	11	61102395	61127852	0.182439925	suppress_segment_too_large
CD52	1	26317957	26320523	0.033763446	PASS
CD53	1	110871188	110899928	0.033763446	PASS
CD55	1	207321376	207386804	-0.256698369	PASS
CD58	1	116514535	116571039	0.033763446	PASS
CD59	11	33703010	33736491	-0.405444717	PASS
CD5L	1	157830914	157898256	-0.467855874	PASS
CD6	11	60971680	61020377	0.182439925	suppress_segment_too_large
CD63	12	55725323	55729707	-0.202191512	PASS
CD68	17	7579467	7582113	0.128406487	PASS
CD69	12	9752486	9760901	0.362961408	suppress_segment_too_large
CD7	17	82314868	82317602	0.088516311	PASS
CD70	19	6583183	6604103	0.079632875	PASS
CD72	9	35609533	35646810	-0.310444659	PASS
CD74	5	150401637	150412929	-0.293443042	PASS

CD79A	19	41877120	41881372	0.093487731	suppress_segment_too_large
CD79B	17	63928740	63932354	0.088516311	PASS
CD80	3	119524293	119559602	0.313454204	suppress_segment_too_large
CD81	11	2376177	2397419	-0.333835828	PASS
CD82	11	44564427	44620363	0.117376172	PASS
CD83	6	14117256	14136918	0.048820648	PASS
CD84	1	160541095	160579516	-0.027073548	PASS
CD86	3	122055366	122121139	0.313454204	suppress_segment_too_large
CD8A	2	86784610	86808396	0.066915641	PASS
CD8B	2	86815339	86861924	0.066915641	PASS
CD8B2	2	106487364	106544297	-0.532032081	PASS
CD9	12	6199715	6238271	-0.086128028	PASS
CD93	20	23079349	23086340	-0.168668583	PASS
CD96	3	111292719	111665750	0.313454204	suppress_segment_too_large
CD99	23	2691133	2741309	-0.012567603	PASS
CD99L2	23	150766337	150898816	-0.012567603	PASS
CDA	1	20588948	20618908	0.033763446	PASS
CDADC1	13	49247925	49293485	-0.390786075	PASS
CDAN1	15	42723544	42737128	-0.396255771	PASS
CDC123	10	12195965	12250589	0.039811807	PASS
CDC14A	1	100345001	100520278	0.033763446	PASS
CDC14B	9	96490241	96619830	0.365190713	suppress_segment_too_large
CDC14C	7	48919765	48927454	-2.348494016	PASS
CDC16	13	114234887	114272723	-0.390786075	PASS
CDC20	1	43358955	43363203	0.033763446	PASS
CDC20B	5	55112995	55173175	-0.293443042	PASS
CDC23	5	138187648	138213343	-0.293443042	PASS
CDC25A	3	48157146	48188402	-0.234617007	PASS
CDC25B	20	3786772	3806121	-0.168668583	PASS
CDC25C	5	138285265	138338355	-0.293443042	PASS
CDC26	9	113255835	113275589	0.365190713	suppress_segment_too_large
CDC27	17	47117703	47189422	0.088516311	PASS
CDC34	19	531712	542092	0.079632875	PASS
CDC37	19	10391134	10420121	0.093487731	suppress_segment_too_large
CDC37L1	9	4679559	4708398	-0.310444659	PASS
CDC40	6	110180141	110254275	-0.368590549	PASS
CDC42	1	22052627	22092946	0.033763446	PASS
CDC42BP/	1	226989865	227318474	-0.256698369	PASS
CDC42BPI	14	102932379	103057462	-0.192197564	PASS
CDC42BP(	11	64823387	64844569	0.182439925	suppress_segment_too_large
CDC42EP1	22	37560447	37569405	-0.575930959	PASS
CDC42EP2	11	65314818	65322429	0.182439925	suppress_segment_too_large
CDC42EP3	2	37641882	37738468	0.040498426	PASS
CDC42EP4	17	73283624	73312175	0.088516311	PASS
CDC42EP5	19	54465026	54473264	-0.146906334	PASS
CDC42SE1	1	151050971	151070325	0.103015788	PASS

CDC42SE2	5	131245493	131398447	-0.293443042	PASS
CDC45	22	19479459	19520612	-0.575930959	PASS
CDC5L	6	44387525	44450426	0.048820648	PASS
CDC6	17	40287633	40304657	0.060555011	PASS
CDC7	1	91500893	91525764	0.033763446	PASS
CDC73	1	193121800	193254815	-0.027073548	PASS
CDCA2	8	25458997	25507920	-0.345962679	PASS
CDCA3	12	6844793	6852066	-0.086128028	PASS
CDCA4	14	105009573	105021148	-0.192197564	PASS
CDCA5	11	65066300	65084164	0.182439925	suppress_segment_too_large
CDCA7	2	173354820	173368997	0.039726562	PASS
CDCA7L	7	21900900	21946084	-0.69513118	PASS
CDCA8	1	37692418	37709719	0.033763446	PASS
CDCP1	3	45082278	45146422	-0.234617007	PASS
CDCP2	1	54132968	54153770	0.033763446	PASS
CDH1	16	68737225	68835548	-0.371357732	PASS
CDH10	5	24487100	24644978	0.055007813	PASS
CDH11	16	64943753	65126112	-0.371357732	PASS
CDH12	5	21750673	22853622	0.055007813	PASS
CDH13	16	82626803	83800640	-0.371357732	PASS
CDH15	16	89171767	89195492	-0.371357732	PASS
CDH16	16	66908122	66918984	-0.371357732	PASS
CDH17	8	94127171	94217303	0.279445683	PASS
CDH18	5	19472951	20575873	0.055007813	PASS
CDH19	18	66501083	66604138	-0.376397843	PASS
CDH2	18	27950966	28177446	-0.376397843	PASS
CDH20	18	61333582	61555773	-0.376397843	PASS
CDH22	20	46173733	46308498	-0.168668583	PASS
CDH23	10	71396934	71815947	0.072405807	PASS
CDH24	14	23047062	23057538	0.060212819	PASS
CDH26	20	59958427	60034011	-0.168668583	PASS
CDH3	16	68636189	68722616	-0.371357732	PASS
CDH4	20	61252426	61940617	-0.168668583	PASS
CDH5	16	66366622	66404786	-0.371357732	PASS
CDH6	5	31193750	31329146	0.055007813	PASS
CDH7	18	65750252	65890341	-0.376397843	PASS
CDH8	16	61647242	62037035	-0.371357732	PASS
CDH9	5	26880600	27121150	0.055007813	PASS
CDHR1	10	84194635	84219621	0.072405807	PASS
CDHR2	5	176542511	176595974	-0.150979337	PASS
CDHR3	7	105876796	106033773	-0.388946609	PASS
CDHR4	3	49790732	49799835	-0.234617007	PASS
CDHR5	11	616565	626078	-0.333835828	PASS
CDIP1	16	4510675	4538828	0.069936844	PASS
CDIPT	16	29858357	29863736	0.069936844	PASS
CDK1	10	60778331	60794852	0.072405807	PASS

CDK10	16	89680737	89696364	-0.371357732	PASS
CDK11A	1	1702730	1724324	0.373964873	suppress_segment_too_large
CDK11B	1	1635227	1659012	0.373964873	suppress_segment_too_large
CDK12	17	39461511	39564907	0.060555011	PASS
CDK13	7	39949646	40099580	-0.69513118	PASS
CDK14	7	90466424	91210590	-0.262778904	PASS
CDK15	2	201790461	201895550	0.025928102	PASS
CDK16	23	47217860	47229997	-0.012567603	PASS
CDK17	12	96278261	96400560	0.202141228	PASS
CDK18	1	205504595	205532793	-0.256698369	PASS
CDK19	6	110609978	110815958	-0.368590549	PASS
CDK2	12	55966769	55972784	-0.202191512	PASS
CDK20	9	87966441	87974753	0.365190713	suppress_segment_too_large
CDK2AP1	12	123260971	123272334	0.202141228	PASS
CDK2AP2	11	67506497	67508649	0.182439925	suppress_segment_too_large
CDK3	17	76000906	76005999	0.088516311	PASS
CDK4	12	57747727	57756013	-0.202191512	PASS
CDK5	7	151053812	151058530	-0.388946609	PASS
CDK5R1	17	32486619	32491256	0.060555011	PASS
CDK5R2	2	218959655	218962162	0.025928102	PASS
CDK5RAP	20	33358839	33401561	-0.168668583	PASS
CDK5RAP	9	120388869	120580170	0.365190713	suppress_segment_too_large
CDK5RAP	17	47967810	47981774	0.088516311	PASS
CDK6	7	92604921	92836594	-0.262778904	PASS
CDK7	5	69234795	69277430	-0.293443042	PASS
CDK8	13	26254104	26405238	-0.390786075	PASS
CDK9	9	127785679	127790787	0.365190713	suppress_segment_too_large
CDKAL1	6	20534457	21232404	0.048820648	PASS
CDKL1	14	50329404	50416461	0.060212819	PASS
CDKL2	4	75578005	75630716	-0.011382299	PASS
CDKL3	5	134286350	134371047	-0.293443042	PASS
CDKL4	2	39175646	39229588	0.040498426	PASS
CDKL5	23	18425583	18653629	-0.012567603	PASS
CDKN1A	6	36676460	36687339	0.048820648	PASS
CDKN1B	12	12715058	12722371	0.362961408	suppress_segment_too_large
CDKN1C	11	2883213	2885773	-0.333835828	PASS
CDKN2A	9	21967753	21995301	-0.310444659	PASS
CDKN2AIl	4	183444591	183448198	-0.462744716	PASS
CDKN2AIl	5	134402087	134411898	-0.293443042	PASS
CDKN2B	9	22002903	22009363	-0.310444659	PASS
CDKN2C	1	50960745	50974633	0.033763446	PASS
CDKN2D	19	10566462	10569059	0.093487731	suppress_segment_too_large
CDKN3	14	54396849	54420218	0.060212819	PASS
CDNF	10	14819250	14838575	0.039811807	PASS
CDO1	5	115804733	115816954	-0.293443042	PASS
CDON	11	125955796	126063335	-0.419612865	PASS

CDPF1	22	46244011	46250679	-0.808211634	PASS
CDR1	23	140783578	140784366	-0.012567603	PASS
CDR2	16	22345936	22437165	0.069936844	PASS
CDR2L	17	74987632	75005800	0.088516311	PASS
CDRT1	17	15565483	15619512	-0.139031004	PASS
CDRT15	17	14235673	14236862	-0.139031004	PASS
CDRT15L2	17	20579724	20580911	0.024610076	PASS
CDRT4	17	15436015	15503608	-0.139031004	PASS
CDS1	4	84582979	84651338	-0.011382299	PASS
CDS2	20	5126786	5197887	-0.168668583	PASS
CDSN	6	31115090	31120446	0.048820648	PASS
CDT1	16	88803213	88809258	-0.371357732	PASS
CDV3	3	133573730	133590261	0.313454204	suppress_segment_too_large
CDX1	5	150166795	150184558	-0.293443042	PASS
CDX2	13	27962137	27971139	-0.390786075	PASS
CDX4	23	73447254	73455245	-0.012567603	PASS
CDYL	6	4706159	4955551	0.048820648	PASS
CDYL2	16	80597906	80804329	-0.371357732	PASS
CEACAM1	19	42507304	42561234	-0.459876623	PASS
CEACAM1	19	44699151	44710714	0.14996833	PASS
CEACAM1	19	51478622	51490952	-0.016761391	PASS
CEACAM1	19	44662278	44684359	0.14996833	PASS
CEACAM2	19	44501677	44529788	0.14996833	PASS
CEACAM2	19	41549518	41586844	0.093487731	suppress_segment_too_large
CEACAM3	19	41796437	41811553	0.093487731	suppress_segment_too_large
CEACAM4	19	41618971	41627074	0.093487731	suppress_segment_too_large
CEACAM5	19	41708585	41729798	0.093487731	suppress_segment_too_large
CEACAM6	19	41750977	41772208	0.093487731	suppress_segment_too_large
CEACAM7	19	41673307	41706976	0.093487731	suppress_segment_too_large
CEACAM8	19	42580241	42595055	-0.459876623	PASS
CEBPA	19	33299934	33302564	0.093487731	suppress_segment_too_large
CEBPB	20	50190734	50192689	-0.168668583	PASS
CEBPD	8	47736909	47739086	-0.201142258	PASS
CEBPE	14	23117304	23119616	0.060212819	PASS
CEBPG	19	33373330	33382686	0.093487731	suppress_segment_too_large
CEBPZ	2	37201612	37231713	0.040498426	PASS
CEBPZOS	2	37196488	37216193	0.040498426	PASS
CECR2	22	17359949	17558149	-0.575930959	PASS
CEL	9	133061978	133071861	0.365190713	suppress_segment_too_large
CELA1	12	51328443	51346679	-0.202191512	PASS
CELA2A	1	15456728	15472091	0.033763446	PASS
CELA2B	1	15465909	15491400	0.033763446	PASS
CELA3A	1	22001656	22012539	0.033763446	PASS
CELA3B	1	21977021	21998642	0.033763446	PASS
CELF1	11	47465933	47565569	0.117376172	PASS
CELF2	10	10798397	11336675	0.039811807	PASS

CELF3	1	151702404	151716814	0.103015788	PASS
CELF4	18	37243047	37566037	-0.376397843	PASS
CELF5	19	3224703	3297076	0.079632875	PASS
CELF6	15	72284727	72320129	-0.697600509	PASS
CELSR1	22	46360834	46537170	-0.808211634	PASS
CELSR2	1	109250019	109275750	0.033763446	PASS
CELSR3	3	48636469	48662915	-0.234617007	PASS
CEMIP	15	80779343	80951776	-0.697600509	PASS
CEMIP2	9	71683366	71816690	0.365190713	suppress_segment_too_large
CEMP1	16	2530035	2531417	0.069936844	PASS
CEND1	11	787104	790123	-0.333835828	PASS
CENPA	2	26764289	26801067	0.040498426	PASS
CENPB	20	3783851	3786690	-0.168668583	PASS
CENPBD1	16	89969773	89972832	-0.371357732	PASS
CENPC	4	67468748	67545606	-0.011382299	PASS
CENPE	4	103105806	103198409	-0.011382299	PASS
CENPF	1	214603195	214664588	-0.256698369	PASS
CENPH	5	69189548	69210357	-0.293443042	PASS
CENPI	23	101098218	101163681	-0.012567603	PASS
CENPJ	13	24882284	24922889	-0.390786075	PASS
CENPK	5	65517766	65563171	-0.293443042	PASS
CENPL	1	173799550	173824720	-0.027073548	PASS
CENPM	22	41938721	41947164	-0.575930959	PASS
CENPN	16	81006498	81033114	-0.371357732	PASS
CENPO	2	24793136	24822376	0.040498426	PASS
CENPP	9	92325484	92620533	0.365190713	suppress_segment_too_large
CENPQ	6	49463378	49493107	0.048820648	PASS
CENPS	1	10430443	10442809	0.373964873	suppress_segment_too_large
CENPS-CC	1	10430102	10452153	0.373964873	suppress_segment_too_large
CENPT	16	67828157	67847811	-0.371357732	PASS
CENPU	4	184694618	184734133	-0.462744716	PASS
CENPV	17	16342534	16353656	0.024610076	PASS
CENPVL3	23	51617024	51618968	-0.012567603	PASS
CENPW	6	126340174	126348875	-0.458570262	PASS
CENPX	17	82018702	82024107	0.088516311	PASS
CEP104	1	3812081	3857214	0.373964873	suppress_segment_too_large
CEP112	17	65635538	66192084	0.088516311	PASS
CEP120	5	123344885	123423592	-0.293443042	PASS
CEP126	11	101915015	102001058	0.062969153	PASS
CEP128	14	80476983	80959517	-0.192197564	PASS
CEP131	17	81189593	81222999	0.088516311	PASS
CEP135	4	55948871	56033363	-0.011382299	PASS
CEP152	15	48712928	48811146	-0.396255771	PASS
CEP162	6	84124241	84227635	-0.368590549	PASS
CEP164	11	117314557	117413268	-0.356188624	PASS
CEP170	1	243124428	243255348	-0.648598641	PASS

CEP170B	14	104865280	104896770	-0.192197564	PASS
CEP19	3	196706277	196712293	0.326834516	PASS
CEP192	18	12991362	13125052	-0.052305056	suppress_segment_too_large
CEP250	20	35455164	35519280	-0.168668583	PASS
CEP290	12	88049014	88142216	0.202141228	PASS
CEP295	11	93661639	93730358	0.062969153	PASS
CEP295NL	17	78870910	78903217	0.088516311	PASS
CEP350	1	179954738	180114880	-0.027073548	PASS
CEP41	7	130393771	130442433	-0.388946609	PASS
CEP44	4	174283677	174333380	-0.011382299	PASS
CEP55	10	93496632	93529092	-0.39678585	PASS
CEP57	11	95789965	95832693	0.062969153	PASS
CEP57L1	6	109095110	109163932	-0.368590549	PASS
CEP63	3	134485721	134575017	0.313454204	suppress_segment_too_large
CEP68	2	65056366	65087004	-0.372959126	PASS
CEP70	3	138494344	138594538	0.313454204	suppress_segment_too_large
CEP72	5	612272	667168	0.055007813	PASS
CEP76	18	12661833	12702777	-0.052305056	suppress_segment_too_large
CEP78	9	78236062	78279690	0.365190713	suppress_segment_too_large
CEP83	12	94306449	94459988	0.202141228	PASS
CEP85	1	26234200	26278808	0.033763446	PASS
CEP85L	6	118460772	118710075	-0.368590549	PASS
CEP89	19	32875925	32971991	0.093487731	suppress_segment_too_large
CEP95	17	64506588	64542461	0.088516311	PASS
CEP97	3	101723925	101770562	0.313454204	suppress_segment_too_large
CEPT1	1	111139627	111185102	0.033763446	PASS
CER1	9	14719724	14722717	-0.310444659	PASS
CERCAM	9	128411751	128437351	0.365190713	suppress_segment_too_large
CERK	22	46684411	46738261	-0.808211634	PASS
CERKL	2	181536676	181680665	0.039726562	PASS
CERS1	19	18868545	18896727	0.093487731	suppress_segment_too_large
CERS2	1	150960583	150975004	0.103015788	PASS
CERS3	15	100400395	100544995	-0.697600509	PASS
CERS4	19	8206736	8262421	0.079632875	PASS
CERS5	12	50129306	50167533	-0.202191512	PASS
CERS6	2	168455862	168775137	0.039726562	PASS
CES1	16	55802851	55833337	-0.371357732	PASS
CES2	16	66934444	66945096	-0.371357732	PASS
CES3	16	66961237	66975148	-0.371357732	PASS
CES4A	16	66988589	67009758	-0.371357732	PASS
CES5A	16	55846154	55956031	-0.371357732	PASS
CETN1	18	580357	582114	-0.052305056	suppress_segment_too_large
CETN2	23	152826973	152830777	-0.012567603	PASS
CETN3	5	90392261	90409786	-0.293443042	PASS
CETP	16	56961850	56983845	-0.371357732	PASS
CFAP100	3	126394939	126436556	0.313454204	suppress_segment_too_large

CFAP126	1	161364731	161367874	-0.027073548	PASS
CFAP157	9	127706989	127716002	0.365190713	suppress_segment_too_large
CFAP161	15	81007033	81149175	-0.697600509	PASS
CFAP20	16	58113588	58129450	-0.371357732	PASS
CFAP206	6	87407983	87464465	-0.368590549	PASS
CFAP221	2	119544432	119662251	-0.475215623	PASS
CFAP298	21	32592079	32612866	0.12860881	suppress_segment_too_large
CFAP299	4	80335720	80963756	-0.011382299	PASS
CFAP300	11	102047443	102084560	0.062969153	PASS
CFAP36	2	55519604	55545879	0.040498426	PASS
CFAP410	21	44328944	44339402	-0.064425456	suppress_segment_too_large
CFAP43	10	104129888	104232362	-0.367947924	PASS
CFAP44	3	113286947	113441610	0.313454204	suppress_segment_too_large
CFAP45	1	159872364	159900163	-0.027073548	PASS
CFAP46	10	132808392	132942823	0.063874692	PASS
CFAP47	23	35919734	36385319	-0.012567603	PASS
CFAP52	17	9576627	9643459	-0.139031004	PASS
CFAP53	18	50227193	50266522	-0.376397843	PASS
CFAP54	12	96489571	96875555	0.202141228	PASS
CFAP57	1	43172149	43254358	0.033763446	PASS
CFAP58	10	104353764	104455090	-0.367947924	PASS
CFAP61	20	20052514	20360702	-0.168668583	PASS
CFAP65	2	219002846	219041527	0.025928102	PASS
CFAP69	7	90245174	90311063	-0.262778904	PASS
CFAP70	10	73253759	73358859	0.072405807	PASS
CFAP73	12	113149858	113159276	0.202141228	PASS
CFAP74	1	1921951	2003837	0.373964873	suppress_segment_too_large
CFAP77	9	132410043	132573317	0.365190713	suppress_segment_too_large
CFAP97	4	185159665	185209504	-0.462744716	PASS
CFAP97D1	17	43780435	43787612	0.088516311	PASS
CFAP99	4	2418974	2462942	0.279089364	PASS
CFB	6	31945650	31952084	0.048820648	PASS
CFC1	2	130592168	130599575	-0.475215623	PASS
CFD	19	859643	863630	0.079632875	PASS
CFDP1	16	75293698	75433485	-0.371357732	PASS
CFH	1	196651878	196747504	-0.027073548	PASS
CFHR1	1	196819757	196832189	-0.027073548	PASS
CFHR2	1	196943756	196959226	-0.027073548	PASS
CFHR3	1	196774795	196795406	-0.027073548	PASS
CFHR4	1	196888014	196918713	-0.027073548	PASS
CFHR5	1	196977556	197009674	-0.027073548	PASS
CFI	4	109740694	109802179	-0.011382299	PASS
CFL1	11	65823022	65862026	0.182439925	suppress_segment_too_large
CFL2	14	34706769	34714823	0.060212819	PASS
CFLAR	2	201116104	201176687	0.025928102	PASS
CFP	23	47623172	47630305	-0.012567603	PASS

CFTR	7	117465784	117715971	-0.388946609	PASS
CGA	6	87085498	87095406	-0.368590549	PASS
CGAS	6	73413515	73452276	-0.368590549	PASS
CGB1	19	49035610	49036895	0.14996833	PASS
CGB2	19	49031912	49033238	0.14996833	PASS
CGB3	19	49022869	49024333	0.14996833	PASS
CGB5	19	49043884	49045311	0.14996833	PASS
CGB7	19	49054275	49058860	0.14996833	PASS
CGB8	19	49047638	49049106	0.14996833	PASS
CGGBP1	3	88051944	88149885	0.313454204	suppress_segment_too_large
CGN	1	151510510	151538692	0.103015788	PASS
CGNL1	15	57375967	57550727	-0.697600509	PASS
CGREF1	2	27098889	27119115	0.040498426	PASS
CGRRF1	14	54509812	54539309	0.060212819	PASS
CH25H	10	89205629	89207314	-1.64589449	suppress_large_homdel
CHAC1	15	40952962	40956519	-0.396255771	PASS
CHAC2	2	53767792	53775196	0.040498426	PASS
CHAD	17	50464496	50468966	0.088516311	PASS
CHADL	22	41229510	41240934	-0.575930959	PASS
CHAF1A	19	4402662	4445018	0.079632875	PASS
CHAF1B	21	36385378	36419015	-0.064425456	suppress_segment_too_large
CHAMP1	13	114314335	114337626	-0.390786075	PASS
CHAT	10	49609095	49665104	-0.404852538	PASS
CHCHD1	10	73782047	73783652	0.072405807	PASS
CHCHD10	22	23765834	23768443	-0.575930959	PASS
CHCHD2	7	56101569	56106576	-0.750061359	PASS
CHCHD3	7	132784868	133082088	-0.388946609	PASS
CHCHD4	3	14112077	14124870	0.311018244	suppress_segment_too_large
CHCHD5	2	112584240	112589275	-0.010901494	PASS
CHCHD6	3	126704220	126960406	0.313454204	suppress_segment_too_large
CHCHD7	8	56211686	56218798	-0.201142258	PASS
CHD1	5	98853985	98928957	-0.293443042	PASS
CHD1L	1	147242641	147295766	-0.157385092	PASS
CHD2	15	92900189	93028005	-0.697600509	PASS
CHD3	17	7884806	7912760	0.128406487	PASS
CHD4	12	6570082	6614524	-0.086128028	PASS
CHD5	1	6101793	6180123	0.373964873	suppress_segment_too_large
CHD6	20	41402101	41618494	-0.168668583	PASS
CHD7	8	60678778	60868028	-0.201142258	PASS
CHD8	14	21385194	21456126	0.060212819	PASS
CHD9	16	53055033	53329150	-0.371357732	PASS
CHDH	3	53812335	53846390	-0.234617007	PASS
CHEK1	11	125625136	125676255	-0.419612865	PASS
CHEK2	22	28687743	28742422	-0.575930959	PASS
CHERP	19	16517889	16542530	0.093487731	suppress_segment_too_large
CHFR	12	132822187	132956304	0.270848837	PASS

CHGA	14	92923080	92935293	-0.192197564	PASS
CHGB	20	5911430	5925361	-0.168668583	PASS
CHI3L1	1	203178931	203186749	-0.256698369	PASS
CHI3L2	1	111200771	111243440	0.033763446	PASS
CHIA	1	111290862	111320566	0.033763446	PASS
CHIC1	23	73563200	73687102	-0.012567603	PASS
CHIC2	4	54009789	54064690	-0.011382299	PASS
CHID1	11	867860	915058	-0.333835828	PASS
CHIT1	1	203212827	203273641	-0.256698369	PASS
CHKA	11	68052859	68121444	0.182439925	suppress_segment_too_large
CHKB	22	50578949	50601455	-0.808211634	PASS
CHKB-CP7	22	50568869	50582965	-0.808211634	PASS
CHL1	3	196596	409417	0.311018244	suppress_segment_too_large
CHM	23	85861180	86047562	-0.012567603	PASS
CHML	1	241628853	241640254	-0.648598641	PASS
CHMP1A	16	89644431	89657845	-0.371357732	PASS
CHMP1B	18	11851396	11854449	-0.052305056	suppress_segment_too_large
CHMP2A	19	58551566	58555124	0.063781462	PASS
CHMP2B	3	87227271	87255548	0.313454204	suppress_segment_too_large
CHMP3	2	86503431	86563479	0.066915641	PASS
CHMP4A	14	24209583	24213869	0.060212819	PASS
CHMP4B	20	33811304	33854366	-0.168668583	PASS
CHMP4C	8	81732434	81759515	0.279445683	PASS
CHMP5	9	33264879	33282069	-0.310444659	PASS
CHMP6	17	80991598	81009517	0.088516311	PASS
CHMP7	8	23243637	23262000	-0.345962679	PASS
CHN1	2	174799363	175005369	0.039726562	PASS
CHN2	7	29146569	29514328	-0.69513118	PASS
CHODL	21	17901263	18267373	0.12860881	suppress_segment_too_large
CHORDC1	11	90201160	90223364	0.062969153	PASS
CHP1	15	41230839	41281890	-0.396255771	PASS
CHP2	16	23754627	23758951	0.069936844	PASS
CHPF	2	219538947	219543787	0.025928102	PASS
CHPF2	7	151232489	151238827	-0.388946609	PASS
CHPT1	12	101696947	101744140	0.202141228	PASS
CHRAC1	8	140511298	140517137	0.113969953	PASS
CHRD	3	184380073	184390736	0.318278996	suppress_segment_too_large
CHRDL1	23	110673856	110795819	-0.012567603	PASS
CHRDL2	11	74696429	74731385	0.062969153	PASS
CHRFAM7	15	30357766	30393849	-0.396255771	PASS
CHRM1	11	62908679	62921807	0.182439925	suppress_segment_too_large
CHRM2	7	136868669	137020255	-0.388946609	PASS
CHRM3	1	239386565	239915452	-0.648598641	PASS
CHRM4	11	46385098	46386608	0.117376172	PASS
CHRM5	15	33968720	34067457	-0.396255771	PASS
CHRNA1	2	174747592	174787935	0.039726562	PASS

CHRNA10	11	3665587	3671384	-0.333835828	PASS
CHRNA2	8	27459761	27479883	-0.345962679	PASS
CHRNA3	15	78593052	78621295	-0.697600509	PASS
CHRNA4	20	63343223	63378401	-0.168668583	PASS
CHRNA5	15	78565520	78595269	-0.697600509	PASS
CHRNA6	8	42752620	42796392	-0.201142258	PASS
CHRNA7	15	31923438	32173018	-0.396255771	PASS
CHRNA9	4	40335329	40355217	-0.011382299	PASS
CHRNB1	17	7445061	7457707	0.128406487	PASS
CHRNB2	1	154567781	154580026	0.103015788	PASS
CHRNB3	8	42697376	42737407	-0.201142258	PASS
CHRNB4	15	78624119	78727754	-0.697600509	PASS
CHRND	2	232525993	232536667	0.025928102	PASS
CHRNE	17	4897774	4934438	0.128406487	PASS
CHRNG	2	232539727	232546403	0.025928102	PASS
CHST1	11	45648877	45665622	0.117376172	PASS
CHST10	2	100391860	100417656	0.048238121	PASS
CHST11	12	104455295	104762014	0.202141228	PASS
CHST12	7	2403560	2448483	-0.69513118	PASS
CHST13	3	126524283	126543291	0.313454204	suppress_segment_too_large
CHST14	15	40470998	40474571	-0.396255771	PASS
CHST15	10	124007666	124093607	0.063874692	PASS
CHST2	3	143119331	143124014	0.318278996	suppress_segment_too_large
CHST3	10	71964365	72013564	0.072405807	PASS
CHST4	16	71525233	71538746	-0.371357732	PASS
CHST5	16	75528535	75535247	-0.371357732	PASS
CHST6	16	75472052	75495445	-0.371357732	PASS
CHST7	23	46573784	46598408	-0.012567603	PASS
CHST8	19	33621955	33773509	0.093487731	suppress_segment_too_large
CHST9	18	26906481	27185317	-0.376397843	PASS
CHSY1	15	101175723	101251932	-0.697600509	PASS
CHSY3	5	129904472	130186634	-0.293443042	PASS
CHTF18	16	788046	800737	0.069936844	PASS
CHTF8	16	69118010	69132584	-0.371357732	PASS
CHTOP	1	153633982	153646306	0.103015788	PASS
CHUK	10	100188298	100229619	-0.367947924	PASS
CHURC1	14	64914361	64944591	0.060212819	PASS
CHURC1-I	14	64914485	65061803	0.060212819	PASS
CIAO1	2	96266132	96273349	0.048238121	PASS
CIAO2A	15	64072559	64094018	-0.697600509	PASS
CIAO2B	16	66932055	66934423	-0.371357732	PASS
CIAO3	16	729753	741329	0.069936844	PASS
CIAPIN1	16	57428169	57447528	-0.371357732	PASS
CIART	1	150282543	150287093	0.103015788	PASS
CIB1	15	90229975	90234047	-0.697600509	PASS
CIB2	15	78104606	78131544	-0.697600509	PASS

CIB3	19	16161368	16173525	0.093487731	suppress_segment_too_large
CIB4	2	26581202	26641368	0.040498426	PASS
CIC	19	42268537	42295797	0.093487731	suppress_segment_too_large
CIDEA	18	12254319	12277595	-0.052305056	suppress_segment_too_large
CIDEB	14	24305096	24311430	0.060212819	PASS
CIDEC	3	9866711	9880254	0.311018244	suppress_segment_too_large
CIITA	16	10866222	10943021	0.069936844	PASS
CILP	15	65194758	65211488	-0.697600509	PASS
CILP2	19	19538248	19546659	0.093487731	suppress_segment_too_large
CINP	14	102341102	102362916	-0.192197564	PASS
CIP2A	3	108549869	108589644	0.313454204	suppress_segment_too_large
CIPC	14	77098097	77117287	-0.192197564	PASS
CIR1	2	174348022	174395715	0.039726562	PASS
CIRBP	19	1259384	1274880	0.079632875	PASS
CISD1	10	58269058	58289586	0.072405807	PASS
CISD2	4	102868974	102892807	-0.011382299	PASS
CISD3	17	38730235	38735044	0.060555011	PASS
CISH	3	50606490	50611831	-0.234617007	PASS
CIT	12	119685790	119877291	0.202141228	PASS
CITED1	23	72301638	72307187	-0.012567603	PASS
CITED2	6	139371807	139374620	-0.035448111	PASS
CITED4	1	40861051	40862366	0.033763446	PASS
CIZ1	9	128166064	128204383	0.365190713	suppress_segment_too_large
CKAP2	13	52455429	52476628	-0.390786075	PASS
CKAP2L	2	112736607	112764677	-0.010901494	PASS
CKAP4	12	106237877	106304279	0.202141228	PASS
CKAP5	11	46743048	46846308	0.117376172	PASS
CKB	14	103519659	103523111	-0.192197564	PASS
CKLF	16	66552563	66566251	-0.371357732	PASS
CKLF-CM'	16	66552587	66579135	-0.371357732	PASS
CKM	19	45306414	45322977	0.14996833	PASS
CKMT1A	15	43692886	43699222	-0.396255771	PASS
CKMT1B	15	43593054	43604901	-0.396255771	PASS
CKMT2	5	81233285	81266397	-0.293443042	PASS
CKS1B	1	154974653	154979249	0.103015788	PASS
CKS2	9	89311198	89316703	0.365190713	suppress_segment_too_large
CLASP1	2	121337776	121649587	-0.475215623	PASS
CLASP2	3	33496245	33718356	0.311018244	suppress_segment_too_large
CLASRP	19	45039040	45070956	0.14996833	PASS
CLBA1	14	104985775	105010482	-0.192197564	PASS
CLC	19	39731250	39738028	0.093487731	suppress_segment_too_large
CLCA1	1	86468368	86500289	0.033763446	PASS
CLCA2	1	86424086	86456558	0.033763446	PASS
CLCA4	1	86547078	86580754	0.033763446	PASS
CLCC1	1	108929508	108963457	0.033763446	PASS
CLCF1	11	67364168	67374177	0.182439925	suppress_segment_too_large

CLCN1	7	143316126	143352083	-0.388946609	PASS
CLCN2	3	184346185	184361651	0.318278996	suppress_segment_too_large
CLCN3	4	169612633	169723673	-0.011382299	PASS
CLCN4	23	10156945	10237660	-0.012567603	PASS
CLCN5	23	49922615	50099235	-0.012567603	PASS
CLCN6	1	11806096	11848079	0.373964873	suppress_segment_too_large
CLCN7	16	1444934	1475580	0.069936844	PASS
CLCNKA	1	16018875	16034050	0.033763446	PASS
CLCNKB	1	16043736	16057308	0.033763446	PASS
CLDN1	3	190305701	190322475	0.318278996	suppress_segment_too_large
CLDN10	13	95433604	95579759	-0.390786075	PASS
CLDN11	3	170418865	170454733	0.318278996	suppress_segment_too_large
CLDN12	7	90383721	90513402	-0.262778904	PASS
CLDN14	21	36460621	36576569	-0.064425456	suppress_segment_too_large
CLDN15	7	101232092	101238820	-0.388946609	PASS
CLDN16	3	190322541	190412143	0.318278996	suppress_segment_too_large
CLDN17	21	30165564	30166756	0.12860881	suppress_segment_too_large
CLDN18	3	137998735	138033655	0.313454204	suppress_segment_too_large
CLDN19	1	42733093	42740254	0.033763446	PASS
CLDN2	23	106900164	106930861	-0.012567603	PASS
CLDN20	6	155264013	155276548	-0.035448111	PASS
CLDN22	4	183318194	183320774	-0.462744716	PASS
CLDN23	8	8701938	8704106	-0.345962679	PASS
CLDN24	4	183321764	183322426	-0.462744716	PASS
CLDN25	11	113779747	113780500	-0.356188624	PASS
CLDN3	7	73768997	73770270	-0.262778904	PASS
CLDN34	23	9967358	9968352	-0.012567603	PASS
CLDN4	7	73799542	73832693	-0.262778904	PASS
CLDN5	22	19523024	19527545	-0.575930959	PASS
CLDN6	16	3014712	3020071	0.069936844	PASS
CLDN7	17	7259903	7263983	0.128406487	PASS
CLDN8	21	30214006	30216073	0.12860881	suppress_segment_too_large
CLDN9	16	3012456	3014505	0.069936844	PASS
CLDND1	3	98497912	98523066	0.313454204	suppress_segment_too_large
CLDND2	19	51367098	51369003	-0.016761391	PASS
CLEC10A	17	7074537	7080307	0.128406487	PASS
CLEC11A	19	50723329	50725718	-0.016761391	PASS
CLEC12A	12	9951316	9995694	0.362961408	suppress_segment_too_large
CLEC12B	12	10010627	10018619	0.362961408	suppress_segment_too_large
CLEC14A	14	38254103	38256369	0.060212819	PASS
CLEC16A	16	10944488	11182189	0.069936844	PASS
CLEC17A	19	14583084	14611157	0.093487731	suppress_segment_too_large
CLEC18A	16	69950705	69964452	-0.371357732	PASS
CLEC18B	16	74408270	74421953	-0.371357732	PASS
CLEC18C	16	70173322	70187361	-0.371357732	PASS
CLEC19A	16	19285739	19322145	0.069936844	PASS

CLEC1A	12	10069554	10111627	0.362961408	suppress_segment_too_large
CLEC1B	12	9985642	10013424	0.362961408	suppress_segment_too_large
CLEC2A	12	9898673	9932381	0.362961408	suppress_segment_too_large
CLEC2B	12	9852984	9870136	0.362961408	suppress_segment_too_large
CLEC2D	12	9664969	9699555	0.362961408	suppress_segment_too_large
CLEC2L	7	139523856	139544984	-0.388946609	PASS
CLEC3A	16	78022515	78066761	-0.371357732	PASS
CLEC3B	3	45001548	45036071	-0.234617007	PASS
CLEC4A	12	8123632	8138607	-0.086128028	PASS
CLEC4C	12	7729415	7751605	-0.086128028	PASS
CLEC4D	12	8509475	8522366	0.362961408	suppress_segment_too_large
CLEC4E	12	8533305	8540963	0.362961408	suppress_segment_too_large
CLEC4F	2	70808643	70820600	-0.372959126	PASS
CLEC4G	19	7728957	7733906	0.079632875	PASS
CLEC4M	19	7763149	7769605	0.079632875	PASS
CLEC5A	7	141927357	141947007	-0.388946609	PASS
CLEC6A	12	8455926	8478330	-0.086128028	PASS
CLEC7A	12	10116777	10130258	0.362961408	suppress_segment_too_large
CLEC9A	12	10030677	10066027	0.362961408	suppress_segment_too_large
CLECL1	12	9715860	9733299	0.362961408	suppress_segment_too_large
CLGN	4	140388455	140427968	-0.011382299	PASS
CLHC1	2	55174791	55232563	0.040498426	PASS
CLIC1	6	31730581	31739763	0.048820648	PASS
CLIC2	23	155276211	155334657	-0.012567603	PASS
CLIC3	9	136994635	136996803	0.365190713	suppress_segment_too_large
CLIC4	1	24745357	24844324	0.033763446	PASS
CLIC5	6	45880827	46080348	0.048820648	PASS
CLIC6	21	34669389	34718227	0.12860881	suppress_segment_too_large
CLINT1	5	157785743	157859175	-0.150979337	PASS
CLIP1	12	122271432	122422632	0.202141228	PASS
CLIP2	7	74289475	74405943	-0.262778904	PASS
CLIP3	19	36014660	36033343	0.093487731	suppress_segment_too_large
CLIP4	2	29097705	29189643	0.040498426	PASS
CLK1	2	200853009	200864744	0.025928102	PASS
CLK2	1	155262868	155278491	0.103015788	PASS
CLK3	15	74598500	74645414	-0.697600509	PASS
CLK4	5	178602664	178630615	-0.150979337	PASS
CLLU1OS	12	92420094	92428148	0.202141228	PASS
CLMN	14	95181940	95319906	-0.192197564	PASS
CLMP	11	123069865	123195281	-0.356188624	PASS
CLN3	16	28474111	28495575	0.069936844	PASS
CLN5	13	76990660	77019143	-0.390786075	PASS
CLN6	15	68206992	68257211	-0.697600509	PASS
CLN8	8	1755778	1801711	-0.313874214	PASS
CLNK	4	10486395	10684865	0.279089364	PASS
CLNS1A	11	77514936	77637805	0.062969153	PASS

CLOCK	4	55427903	55547138	-0.011382299	PASS
CLP1	11	57648992	57661868	-0.129266491	PASS
CLPB	11	72285495	72434680	0.062969153	PASS
CLPP	19	6361452	6368910	0.079632875	PASS
CLPS	6	35794982	35797344	0.048820648	PASS
CLPSL1	6	35781017	35793675	0.048820648	PASS
CLPSL2	6	35776594	35779552	0.048820648	PASS
CLPTM1	19	44954585	44993341	0.14996833	PASS
CLPTM1L	5	1317744	1345099	0.055007813	PASS
CLPX	15	65148219	65185342	-0.697600509	PASS
CLRN1	3	150926163	150972999	0.318278996	suppress_segment_too_large
CLRN2	4	17515165	17527104	0.279089364	PASS
CLRN3	10	127877841	127892947	0.063874692	PASS
CLSPN	1	35720218	35769967	0.033763446	PASS
CLSTN1	1	9729026	9824526	0.373964873	suppress_segment_too_large
CLSTN2	3	139935185	140577397	-0.356957581	PASS
CLSTN3	12	7129698	7158945	-0.086128028	PASS
CLTA	9	36190856	36304781	-0.310444659	PASS
CLTB	5	176392455	176416569	-0.150979337	PASS
CLTC	17	59619689	59696956	0.088516311	PASS
CLTCL1	22	19179473	19291716	-0.575930959	PASS
CLTRN	23	15627318	15675012	-0.012567603	PASS
CLU	8	27596917	27615031	-0.345962679	PASS
CLUAP1	16	3500924	3539048	0.069936844	PASS
CLUH	17	2689386	2712663	0.128406487	PASS
CLUL1	18	596988	650334	-0.052305056	suppress_segment_too_large
CLVS1	8	61057158	61501645	-0.201142258	PASS
CLVS2	6	122995971	123072927	-1.97829255	PASS
CLYBL	13	99606669	99897134	-0.390786075	PASS
CMA1	14	24505353	24508265	0.060212819	PASS
CMAS	12	22046174	22065674	0.362961408	suppress_segment_too_large
CMBL	5	10275875	10308026	0.055007813	PASS
CMC1	3	28241584	28325142	0.311018244	suppress_segment_too_large
CMC2	16	80966448	81020270	-0.371357732	PASS
CMC4	23	155061622	155071362	-0.012567603	PASS
CMIP	16	81445170	81711762	-0.371357732	PASS
CMKLR1	12	108288044	108339341	0.202141228	PASS
CMPK1	1	47333797	47378839	0.033763446	PASS
CMPK2	2	6840570	6866635	0.040498426	PASS
CMSS1	3	99817834	100181732	0.313454204	suppress_segment_too_large
CMTM1	16	66566393	66579137	-0.371357732	PASS
CMTM2	16	66579448	66588275	-0.371357732	PASS
CMTM3	16	66603874	66613892	-0.371357732	PASS
CMTM4	16	66614750	66696707	-0.371357732	PASS
CMTM5	14	23376773	23379772	0.060212819	PASS
CMTM6	3	32481312	32503408	0.311018244	suppress_segment_too_large

CMTM7	3	32391671	32483067	0.311018244	suppress_segment_too_large
CMTM8	3	32238679	32370325	0.311018244	suppress_segment_too_large
CMTR1	6	37433219	37482827	0.048820648	PASS
CMTR2	16	71281389	71289715	-0.371357732	PASS
CMYA5	5	79689877	79800240	-0.293443042	PASS
CNBD1	8	86866442	87615219	0.279445683	PASS
CNBD2	20	35954564	36030700	-0.168668583	PASS
CNBP	3	129169484	129183922	0.313454204	suppress_segment_too_large
CNDP1	18	74534440	74587212	-0.376397843	PASS
CNDP2	18	74495816	74523454	-0.376397843	PASS
CNEP1R1	16	50024410	50037088	0.069936844	PASS
CNFN	19	42387019	42390287	0.093487731	suppress_segment_too_large
CNGA1	4	47935977	48016672	-0.011382299	PASS
CNGA2	23	151738451	151745304	-0.012567603	PASS
CNGA3	2	98346155	98398601	0.048238121	PASS
CNGA4	11	6234765	6244429	-0.405444717	PASS
CNGB1	16	57882340	57971116	-0.371357732	PASS
CNGB3	8	86553977	86743675	0.279445683	PASS
CNIH1	14	54423560	54441431	0.060212819	PASS
CNIH2	11	66278190	66285301	0.182439925	suppress_segment_too_large
CNIH3	1	224434660	224740549	-0.256698369	PASS
CNIH4	1	224356850	224379459	-0.256698369	PASS
CNKSR1	1	26177403	26189886	0.033763446	PASS
CNKSR2	23	21372801	21654695	-0.012567603	PASS
CNKSR3	6	154387504	154510659	-0.035448111	PASS
CNMD	13	52703264	52739812	-0.390786075	PASS
CNN1	19	11538717	11550323	0.093487731	suppress_segment_too_large
CNN2	19	1026581	1039068	0.079632875	PASS
CNN3	1	94896949	94927278	0.033763446	PASS
CNNM1	10	99329099	99394330	-0.367947924	PASS
CNNM2	10	102918293	103090221	-0.367947924	PASS
CNNM3	2	96816245	96833911	0.048238121	PASS
CNNM4	2	96760902	96811891	0.048238121	PASS
CNOT1	16	58519951	58629886	-0.371357732	PASS
CNOT10	3	32685145	32773875	0.311018244	suppress_segment_too_large
CNOT11	2	101252802	101270316	0.048238121	PASS
CNOT2	12	70242994	70354993	-0.202191512	PASS
CNOT3	19	54137717	54155708	0.206912349	PASS
CNOT4	7	135361795	135510127	-0.388946609	PASS
CNOT6	5	180494412	180578405	-0.150979337	PASS
CNOT6L	4	77713387	77819615	-0.011382299	PASS
CNOT7	8	17224964	17246878	-0.345962679	PASS
CNOT8	5	154857553	154876793	-0.293443042	PASS
CNOT9	2	218568580	218597080	0.025928102	PASS
CNP	17	41966741	41977731	0.060555011	PASS
CNPPD1	2	219171897	219178106	0.025928102	PASS

CNPY1	7	155474206	155555450	-0.411415677	PASS
CNPY2	12	56309842	56316336	-0.202191512	PASS
CNPY3	6	42929192	42939287	0.048820648	PASS
CNPY4	7	100119613	100125511	-0.262778904	PASS
CNR1	6	88139864	88166359	-0.368590549	PASS
CNR2	1	23870526	23913362	0.033763446	PASS
CNRIP1	2	68284171	68320051	-0.372959126	PASS
CNST	1	246566444	246668584	-0.368111568	PASS
CNTD1	17	42798792	42811587	0.088516311	PASS
CNTD2	19	40222208	40226697	0.093487731	suppress_segment_too_large
CNTF	11	58622673	58625733	-0.129266491	PASS
CNTFR	9	34551432	34590140	-0.310444659	PASS
CNTLN	9	17134982	17503923	-0.310444659	PASS
CNTN1	12	40692442	41072418	-0.202191512	PASS
CNTN2	1	205042937	205078284	-0.256698369	PASS
CNTN3	3	74262568	74521140	-0.234617007	PASS
CNTN4	3	2098813	3057956	0.311018244	suppress_segment_too_large
CNTN5	11	99020953	100358885	0.062969153	PASS
CNTN6	3	1092576	1404217	0.311018244	suppress_segment_too_large
CNTNAP1	17	42682613	42699814	0.088516311	PASS
CNTNAP2	7	146116002	148420998	-0.388946609	PASS
CNTNAP3	9	39072767	39288315	-0.310444659	PASS
CNTNAP3	9	41890314	42129510	-0.310444659	PASS
CNTNAP3	9	61330717	61453030	-0.310444659	PASS
CNTNAP4	16	76277278	76559238	-0.371357732	PASS
CNTNAP5	2	124025287	124915287	-0.475215623	PASS
CNTRL	9	121074863	121177610	0.365190713	suppress_segment_too_large
CNTROB	17	7932101	7949918	0.128406487	PASS
COA1	7	43608456	43729717	-0.69513118	PASS
COA3	17	42795147	42798704	0.088516311	PASS
COA4	11	73872667	73876988	0.062969153	PASS
COA5	2	98599310	98608515	0.048238121	PASS
COA6	1	234373456	234384049	-0.256698369	PASS
COA7	1	52684451	52698366	0.033763446	PASS
COASY	17	42561467	42566277	0.088516311	PASS
COBL	7	51016212	51316818	-0.750061359	PASS
COBLL1	2	164653624	164843679	0.039726562	PASS
COCH	14	30874514	30895065	0.060212819	PASS
COG1	17	73192632	73208507	0.088516311	PASS
COG2	1	230642489	230693982	-0.256698369	PASS
COG3	13	45464898	45536630	-0.390786075	PASS
COG4	16	70480568	70523565	-0.371357732	PASS
COG5	7	107201555	107564514	-0.388946609	PASS
COG6	13	39655627	39791665	-0.390786075	PASS
COG7	16	23388493	23453180	0.069936844	PASS
COG8	16	69320140	69339667	-0.371357732	PASS

COIL	17	56938187	56961054	0.088516311	PASS
COL10A1	6	116118923	116158747	-0.368590549	PASS
COL11A1	1	102876467	103108872	0.033763446	PASS
COL11A2	6	33162681	33192499	0.048820648	PASS
COL12A1	6	75084326	75206051	-0.368590549	PASS
COL13A1	10	69801867	69964275	0.072405807	PASS
COL14A1	8	120059780	120372036	0.279445683	PASS
COL15A1	9	98943179	99070792	0.365190713	suppress_segment_too_large
COL16A1	1	31652247	31704319	0.033763446	PASS
COL17A1	10	104031286	104085957	-0.367947924	PASS
COL18A1	21	45405137	45513720	-0.064425456	suppress_segment_too_large
COL19A1	6	69866571	70209976	-0.368590549	PASS
COL1A1	17	50183289	50201632	0.088516311	PASS
COL1A2	7	94394561	94431232	-0.262778904	PASS
COL20A1	20	63293186	63334851	-0.168668583	PASS
COL21A1	6	56056590	56394094	0.048820648	PASS
COL22A1	8	138588235	138914006	0.113969953	PASS
COL23A1	5	178237618	178590555	-0.150979337	PASS
COL24A1	1	85729233	86156943	0.033763446	PASS
COL25A1	4	108808725	109302752	-0.011382299	PASS
COL26A1	7	101362820	101559024	-0.388946609	PASS
COL27A1	9	114155560	114312511	0.365190713	suppress_segment_too_large
COL28A1	7	7356203	7535853	-0.69513118	PASS
COL2A1	12	47972965	48004554	-0.202191512	PASS
COL3A1	2	188974320	189012746	0.025928102	PASS
COL4A1	13	110148963	110307233	-0.390786075	PASS
COL4A2	13	110305812	110513209	-0.390786075	PASS
COL4A3	2	227164565	227314792	0.025928102	PASS
COL4A3BI	5	75356345	75512138	-0.293443042	PASS
COL4A4	2	227002711	227164453	0.025928102	PASS
COL4A5	23	108439844	108697545	-0.012567603	PASS
COL4A6	23	108155607	108439497	-0.012567603	PASS
COL5A1	9	134641774	134844843	0.365190713	suppress_segment_too_large
COL5A2	2	189031896	189225312	0.025928102	PASS
COL5A3	19	9959561	10010471	0.093487731	suppress_segment_too_large
COL6A1	21	45981737	46005050	-0.064425456	suppress_segment_too_large
COL6A2	21	46098097	46132849	-0.064425456	suppress_segment_too_large
COL6A3	2	237324003	237414375	0.025928102	PASS
COL6A5	3	130345516	130484844	0.313454204	suppress_segment_too_large
COL6A6	3	130560334	130678155	0.313454204	suppress_segment_too_large
COL7A1	3	48564073	48595267	-0.234617007	PASS
COL8A1	3	99638475	99799226	0.313454204	suppress_segment_too_large
COL8A2	1	36095236	36125220	0.033763446	PASS
COL9A1	6	70215061	70303083	-0.368590549	PASS
COL9A2	1	40300487	40317813	0.033763446	PASS
COL9A3	20	62816244	62841159	-0.168668583	PASS

COLCA2	11	111298555	111308735	-0.356188624	PASS
COLEC10	8	118995452	119106582	0.279445683	PASS
COLEC11	2	3594832	3644644	0.040498426	PASS
COLEC12	18	316740	500722	-0.052305056	suppress_segment_too_large
COLGALT	19	17555594	17583162	0.093487731	suppress_segment_too_large
COLGALT	1	183929854	184037729	-0.027073548	PASS
COLQ	3	15450133	15521751	0.311018244	suppress_segment_too_large
COMMD1	2	61888724	62147247	-0.372959126	PASS
COMMD1(	5	116084991	116412762	-0.293443042	PASS
COMMD2	3	149738470	149752499	0.318278996	suppress_segment_too_large
COMMD3	10	22315974	22320308	0.039811807	PASS
COMMD3-	10	22316388	22329542	0.039811807	PASS
COMMD4	15	75335891	75343224	-0.697600509	PASS
COMMD5	8	144841042	144853736	0.307788765	PASS
COMMD6	13	75525219	75549439	-0.390786075	PASS
COMMD7	20	32702691	32743997	-0.168668583	PASS
COMMD8	4	47450796	47463719	-0.011382299	PASS
COMMD9	11	36269284	36289449	-0.405444717	PASS
COMP	19	18782773	18791314	0.093487731	suppress_segment_too_large
COMT	22	19941607	19969975	-0.575930959	PASS
COMTD1	10	75233969	75236030	0.072405807	PASS
COP1	1	175944831	176207493	-0.027073548	PASS
COPA	1	160288686	160343400	-0.027073548	PASS
COPB1	11	14443440	14500027	-0.405444717	PASS
COPB2	3	139355600	139389732	0.313454204	suppress_segment_too_large
COPE	19	18899514	18919397	0.093487731	suppress_segment_too_large
COPG1	3	129249606	129277773	0.313454204	suppress_segment_too_large
COPG2	7	130506238	130668748	-0.388946609	PASS
COPRS	17	31851864	31859337	0.060555011	PASS
COPS2	15	49106068	49155661	-0.396255771	PASS
COPS3	17	17246820	17281293	0.024610076	PASS
COPS4	4	83034447	83075818	-0.011382299	PASS
COPS5	8	67043079	67083783	-0.201142258	PASS
COPS6	7	100088954	100092200	-0.262778904	PASS
COPS7A	12	6723741	6731875	-0.086128028	PASS
COPS7B	2	231781671	231809254	0.025928102	PASS
COPS8	2	237085312	237100466	0.025928102	PASS
COPS9	2	240126563	240136807	0.025928102	PASS
COPZ1	12	54301202	54351849	-0.202191512	PASS
COPZ2	17	48026167	48038030	0.088516311	PASS
COQ10A	12	56266858	56270966	-0.202191512	PASS
COQ10B	2	197453423	197475308	0.025928102	PASS
COQ2	4	83261536	83284914	-0.011382299	PASS
COQ3	6	99369400	99394204	-0.368590549	PASS
COQ4	9	128322536	128334072	0.365190713	suppress_segment_too_large
COQ5	12	120503274	120534434	0.202141228	PASS

COQ6	14	73949926	73963670	0.060212819	PASS
COQ7	16	19067599	19080095	0.069936844	PASS
COQ8A	1	226897536	226987545	-0.256698369	PASS
COQ8B	19	40691529	40718207	0.093487731	suppress_segment_too_large
COQ9	16	57447425	57461275	-0.371357732	PASS
CORIN	4	47593998	47838106	-0.011382299	PASS
CORO1A	16	30182827	30189076	0.069936844	PASS
CORO1B	11	67435510	67443821	0.182439925	suppress_segment_too_large
CORO1C	12	108645109	108731596	0.202141228	PASS
CORO2A	9	98120975	98192640	0.365190713	suppress_segment_too_large
CORO2B	15	68578969	68727806	-0.697600509	PASS
CORO6	17	29614756	29622907	0.060555011	PASS
CORO7	16	4354542	4425705	0.069936844	PASS
CORO7-PA	16	4340251	4420494	0.069936844	PASS
CORT	1	10449719	10451902	0.373964873	suppress_segment_too_large
COTL1	16	84565594	84618077	-0.371357732	PASS
COX10	17	14069496	14208677	-0.139031004	PASS
COX11	17	54951902	54968785	0.088516311	PASS
COX14	12	50111979	50120457	-0.202191512	PASS
COX15	10	99711844	99732100	-0.367947924	PASS
COX16	14	70325081	70359731	0.060212819	PASS
COX17	3	119654513	119677454	0.313454204	suppress_segment_too_large
COX18	4	73052362	73069755	-0.011382299	PASS
COX19	7	898778	975599	-0.69513118	PASS
COX20	1	244835322	244845057	-0.368111568	PASS
COX4I1	16	85798633	85807044	-0.371357732	PASS
COX4I2	20	31637888	31645006	-0.168668583	PASS
COX5A	15	74919791	74938168	-0.697600509	PASS
COX5B	2	97646040	97648383	0.048238121	PASS
COX6A1	12	120438090	120440742	0.202141228	PASS
COX6A2	16	31427731	31428428	0.069936844	PASS
COX6B1	19	35648223	35658861	0.093487731	suppress_segment_too_large
COX6B2	19	55349306	55354814	0.121336758	PASS
COX6C	8	99873200	99894062	0.279445683	PASS
COX7A1	19	36150922	36152869	0.093487731	suppress_segment_too_large
COX7A2	6	75237675	75250323	-0.368590549	PASS
COX7A2L	2	42333546	42425088	0.040498426	PASS
COX7B	23	77899438	77907373	-0.012567603	PASS
COX7B2	4	46734827	46909235	-0.011382299	PASS
COX7C	5	86617904	86620962	-0.293443042	PASS
COX8A	11	63974607	63976543	0.182439925	suppress_segment_too_large
COX8C	14	93347191	93348356	-0.192197564	PASS
CP	3	149162410	149222055	0.318278996	suppress_segment_too_large
CPA1	7	130380339	130388114	-0.388946609	PASS
CPA2	7	130266827	130289798	-0.388946609	PASS
CPA3	3	148865256	148897196	0.318278996	suppress_segment_too_large

CPA4	7	130293134	130324180	-0.388946609	PASS
CPA5	7	130344816	130368730	-0.388946609	PASS
CPA6	8	67422038	67746385	-0.201142258	PASS
CPAMD8	19	16892947	17026815	0.093487731	suppress_segment_too_large
CPB1	3	148791102	148860187	0.318278996	suppress_segment_too_large
CPB2	13	46053195	46105033	-0.390786075	PASS
CPD	17	30378905	30469989	0.060555011	PASS
CPE	4	165361194	165498320	-0.011382299	PASS
CPEB1	15	82543201	82648861	-0.697600509	PASS
CPEB2	4	15002674	15070153	0.279089364	PASS
CPEB3	10	92046692	92291087	-0.39678585	PASS
CPEB4	5	173888280	173961976	-0.150979337	PASS
CPED1	7	120988677	121297444	-0.388946609	PASS
CPLANE1	5	37106228	37249428	0.055007813	PASS
CPLANE2	1	16231700	16237162	0.033763446	PASS
CPLX1	4	784957	826198	0.279089364	PASS
CPLX2	5	175796310	175884020	-0.150979337	PASS
CPLX3	15	74826547	74831802	-0.697600509	PASS
CPLX4	18	59275156	59318649	-0.376397843	PASS
CPM	12	68842197	68971570	-0.202191512	PASS
CPN1	10	100042193	100081877	-0.367947924	PASS
CPN2	3	194339765	194351328	0.326834516	PASS
CPNE1	20	35626031	35664956	-0.168668583	PASS
CPNE2	16	57092537	57148367	-0.371357732	PASS
CPNE3	8	86514427	86561498	0.279445683	PASS
CPNE4	3	131533555	132285410	0.313454204	suppress_segment_too_large
CPNE5	6	36740775	36840002	0.048820648	PASS
CPNE6	14	24070837	24078100	0.060212819	PASS
CPNE7	16	89575768	89597246	-0.371357732	PASS
CPNE8	12	38646822	38907430	-0.202191512	PASS
CPNE9	3	9703807	9729908	0.311018244	suppress_segment_too_large
CPO	2	206939554	206969474	0.025928102	PASS
CPOX	3	98579446	98593723	0.313454204	suppress_segment_too_large
CPPED1	16	12659799	12804017	0.069936844	PASS
CPQ	8	96645227	97149654	0.279445683	PASS
CPS1	2	210477682	210679107	0.025928102	PASS
CPSF1	8	144393229	144409349	0.307788765	PASS
CPSF2	14	92121937	92172145	-0.192197564	PASS
CPSF3	2	9423568	9473101	0.040498426	PASS
CPSF4	7	99438922	99457371	-0.262778904	PASS
CPSF4L	17	73248449	73262352	0.088516311	PASS
CPSF6	12	69239537	69274358	-0.202191512	PASS
CPSF7	11	61402641	61430031	0.182439925	suppress_segment_too_large
CPT1A	11	68754620	68844410	0.182439925	suppress_segment_too_large
CPT1B	22	50568861	50578465	-0.808211634	PASS
CPT1C	19	49690898	49713731	0.14996833	PASS

CPT2	1	53196429	53214197	0.033763446	PASS
CPTP	1	1324756	1328897	0.373964873	suppress_segment_too_large
CPVL	7	28995231	29195451	-0.69513118	PASS
CPXCR1	23	88747225	88754785	-0.012567603	PASS
CPXM1	20	2794069	2800637	-0.168668583	PASS
CPXM2	10	123706207	123940267	0.063874692	PASS
CPZ	4	8592660	8619759	0.279089364	PASS
CR1	1	207496147	207641765	-0.256698369	PASS
CR1L	1	207645113	207738416	-0.256698369	PASS
CR2	1	207454230	207489895	-0.256698369	PASS
CRABP1	15	78340324	78348230	-0.697600509	PASS
CRABP2	1	156699606	156705816	0.103015788	PASS
CRACR2A	12	3606633	3764819	-0.086128028	PASS
CRACR2B	11	826144	831991	-0.333835828	PASS
CRADD	12	93677375	93894840	0.202141228	PASS
CRAMP1	16	1612325	1677908	0.069936844	PASS
CRAT	9	129094810	129111189	0.365190713	suppress_segment_too_large
CRB1	1	197268204	197478455	-0.027073548	PASS
CRB2	9	123356170	123380324	0.365190713	suppress_segment_too_large
CRB3	19	6463777	6467221	0.079632875	PASS
CRBN	3	3144628	3179727	0.311018244	suppress_segment_too_large
CRCP	7	66114604	66154568	-0.750061359	PASS
CRCT1	1	152514502	152516010	0.103015788	PASS
CREB1	2	207529737	207603431	0.025928102	PASS
CREB3	9	35732335	35737004	-0.310444659	PASS
CREB3L1	11	46277661	46321422	0.117376172	PASS
CREB3L2	7	137874979	138002067	-0.388946609	PASS
CREB3L3	19	4153601	4173054	0.079632875	PASS
CREB3L4	1	153967534	153974363	0.103015788	PASS
CREB5	7	28299321	28825894	-0.69513118	PASS
CREBBP	16	3725054	3880726	0.069936844	PASS
CREBL2	12	12611827	12645108	0.362961408	suppress_segment_too_large
CREBRF	5	173056352	173139284	-0.150979337	PASS
CREBZF	11	85659708	85682908	0.062969153	PASS
CREG1	1	167529117	167553805	-0.027073548	PASS
CREG2	2	101345551	101387595	0.048238121	PASS
CRELD1	3	9933822	9945413	0.311018244	suppress_segment_too_large
CRELD2	22	49918167	49927540	-0.808211634	PASS
CREM	10	35126791	35212958	0.039811807	PASS
CRH	8	66176382	66178725	-0.201142258	PASS
CRHBP	5	76952713	76981158	-0.293443042	PASS
CRHR1	17	45784280	45835828	0.088516311	PASS
CRHR2	7	30651943	30700129	-0.69513118	PASS
CRIM1	2	36355926	36551135	0.040498426	PASS
CRIP1	14	105486317	105488947	-0.192197564	PASS
CRIP2	14	105472962	105480170	-0.192197564	PASS

CRIP3	6	43299710	43308797	0.048820648	PASS
CRIPT	2	46616416	46625742	0.040498426	PASS
CRISP1	6	49834257	49877096	0.048820648	PASS
CRISP2	6	49692358	49713590	0.048820648	PASS
CRISP3	6	49727384	49744437	0.048820648	PASS
CRISPLD1	8	74984515	75034558	-0.201142258	PASS
CRISPLD2	16	84819984	84920768	-0.371357732	PASS
CRK	17	1420689	1463162	0.128406487	PASS
CRKL	22	20917426	20953749	-0.575930959	PASS
CRLF1	19	18572220	18607741	0.093487731	suppress_segment_too_large
CRLF2	23	1187549	1212750	-0.012567603	PASS
CRLF3	17	30769388	30824776	0.060555011	PASS
CRLS1	20	6006090	6040053	-0.168668583	PASS
CRMP1	4	5748084	5893058	0.279089364	PASS
CRNKL1	20	20034368	20056046	-0.168668583	PASS
CRNN	1	152409243	152414263	0.103015788	PASS
CROCC	1	16740273	16972979	0.033763446	PASS
CROCC2	2	240906330	240993311	0.025928102	PASS
CROT	7	87345681	87399795	-0.262778904	PASS
CRP	1	159712289	159714589	-0.467855874	PASS
CRTAC1	10	97865000	98030828	-0.367947924	PASS
CRTAM	11	122838500	122872639	-0.356188624	PASS
CRTAP	3	33113979	33147773	0.311018244	suppress_segment_too_large
CRTC1	19	18683677	18782333	0.093487731	suppress_segment_too_large
CRTC2	1	153947669	153958625	0.103015788	PASS
CRTC3	15	90529925	90645345	-0.697600509	PASS
CRX	19	47819779	47843330	0.14996833	PASS
CRY1	12	106991364	107093829	0.202141228	PASS
CRY2	11	45847118	45883248	0.117376172	PASS
CRYAA	21	43169008	43172805	-0.064425456	suppress_segment_too_large
CRYAA2	21	6560714	6564489	-0.304975024	PASS
CRYAB	11	111908565	111923722	-0.356188624	PASS
CRYBA1	17	29246863	29254494	0.060555011	PASS
CRYBA2	2	218990189	218993421	0.025928102	PASS
CRYBA4	22	26621964	26630672	-0.575930959	PASS
CRYBB1	22	26599278	26618088	-0.575930959	PASS
CRYBB2	22	25219522	25231869	-0.575930959	PASS
CRYBB3	22	25199850	25207363	-0.575930959	PASS
CRYBG1	6	106360808	106571978	-0.368590549	PASS
CRYBG2	1	26321698	26360080	0.033763446	PASS
CRYBG3	3	97822040	97944963	0.313454204	suppress_segment_too_large
CRYGA	2	208160740	208163576	0.025928102	PASS
CRYGB	2	208142573	208146168	0.025928102	PASS
CRYGC	2	208128137	208129830	0.025928102	PASS
CRYGD	2	208121607	208124501	0.025928102	PASS
CRYGN	7	151428835	151440813	-0.388946609	PASS

CRYGS	3	186538441	186546702	0.318278996	suppress_segment_too_large
CRYL1	13	20403666	20525873	-0.390786075	PASS
CRYM	16	21238874	21303083	0.069936844	PASS
CRYZ	1	74705482	74733408	0.033763446	PASS
CRYZL1	21	33589341	33643926	0.12860881	suppress_segment_too_large
CS	12	56271699	56300392	-0.202191512	PASS
CSAD	12	53157663	53180909	-0.202191512	PASS
CSAG1	23	152727484	152733735	-0.012567603	PASS
CSAG3	23	152753921	152760222	-0.012567603	PASS
CSDC2	22	41560763	41577741	-0.575930959	PASS
CSDE1	1	114716913	114758676	0.033763446	PASS
CSE1L	20	49046246	49096960	-0.168668583	PASS
CSF1	1	109910242	109930992	0.033763446	PASS
CSF1R	5	150053291	150113372	-0.293443042	PASS
CSF2	5	132073790	132076170	-0.293443042	PASS
CSF2RA	23	1268800	1310381	-0.012567603	PASS
CSF2RB	22	36913628	36940449	-0.575930959	PASS
CSF3	17	40015361	40017813	0.060555011	PASS
CSF3R	1	36466043	36483278	0.033763446	PASS
CSGALNA	8	19404161	19758029	-0.345962679	PASS
CSGALNA	10	43138486	43185308	-0.404852538	PASS
CSH1	17	63894909	63896661	0.088516311	PASS
CSH2	17	63872012	63873766	0.088516311	PASS
CSHL1	17	63909597	63918838	0.088516311	PASS
CSK	15	74782057	74803198	-0.697600509	PASS
CSKMT	11	62665309	62668496	0.182439925	suppress_segment_too_large
CSMD1	8	2935353	4994972	-1.076352548	PASS
CSMD2	1	33513999	34165842	0.033763446	PASS
CSMD3	8	112222928	113437099	0.279445683	PASS
CSN1S1	4	69931081	69946574	-0.011382299	PASS
CSN2	4	69955256	69961007	-0.011382299	PASS
CSN3	4	70242588	70251428	-0.011382299	PASS
CSNK1A1	5	149492197	149551552	-0.293443042	PASS
CSNK1A1I	13	37103259	37105664	-0.390786075	PASS
CSNK1D	17	82239023	82273731	0.088516311	PASS
CSNK1E	22	38290691	38318084	-0.575930959	PASS
CSNK1G1	15	64165517	64356259	-0.697600509	PASS
CSNK1G2	19	1941149	1981338	0.079632875	PASS
CSNK1G3	5	123512099	123617045	-0.293443042	PASS
CSNK2A1	20	472498	543835	-0.168668583	PASS
CSNK2A2	16	58157907	58197920	-0.371357732	PASS
CSNK2A3	11	11351942	11353357	-0.405444717	PASS
CSNK2B	6	31665236	31670343	0.048820648	PASS
CSPG4	15	75674322	75712848	-0.697600509	PASS
CSPG5	3	47562239	47580792	-0.234617007	PASS
CSPP1	8	67062426	67196263	-0.201142258	PASS

CSRNP1	3	39141855	39154562	-0.234617007	PASS
CSRNP2	12	51061205	51083664	-0.202191512	PASS
CSRNP3	2	165469647	165689407	0.039726562	PASS
CSRP1	1	201483530	201509456	-0.256698369	PASS
CSRP2	12	76858715	76879060	0.202141228	PASS
CSRP3	11	19182037	19210571	-0.405444717	PASS
CST1	20	23747553	23751268	-0.168668583	PASS
CST11	20	23450403	23452857	-0.168668583	PASS
CST2	20	23823769	23826731	-0.168668583	PASS
CST3	20	23626706	23638473	-0.168668583	PASS
CST4	20	23685640	23689040	-0.168668583	PASS
CST5	20	23875935	23879750	-0.168668583	PASS
CST6	11	66011841	66013505	0.182439925	suppress_segment_too_large
CST7	20	24949230	24959928	-0.168668583	PASS
CST8	20	23491101	23496018	-0.168668583	PASS
CST9	20	23602410	23605876	-0.168668583	PASS
CST9L	20	23564732	23568749	-0.168668583	PASS
CSTA	3	122325244	122341972	0.313454204	suppress_segment_too_large
CSTB	21	43772511	43776445	-0.064425456	suppress_segment_too_large
CSTF1	20	56392371	56406369	-0.168668583	PASS
CSTF2	23	100820359	100840932	-0.012567603	PASS
CSTF2T	10	51695487	51699591	-0.404852538	PASS
CSTF3	11	33077188	33162371	-0.405444717	PASS
CSTL1	20	23439685	23444930	-0.168668583	PASS
CT45A1	23	135713453	135723539	-0.012567603	PASS
CT45A10	23	135881063	135889086	-0.012567603	PASS
CT45A3	23	135759846	135768191	-0.012567603	PASS
CT47B1	23	120872603	120875929	-0.012567603	PASS
CT55	23	135156536	135171398	-0.012567603	PASS
CT62	15	71110244	71115500	-0.697600509	PASS
CT83	23	116461682	116463003	-0.012567603	PASS
CTAG2	23	154651972	154653579	-0.012567603	PASS
CTAGE1	18	22413601	22417915	-0.376397843	PASS
CTAGE15	7	143571801	143574387	-0.388946609	PASS
CTAGE4	7	144183466	144186053	-0.388946609	PASS
CTAGE6	7	143755089	143757696	-0.388946609	PASS
CTAGE8	7	144266701	144269288	-0.388946609	PASS
CTAGE9	6	131708684	131711017	-0.458570262	PASS
CTBP1	4	1211448	1249953	0.279089364	PASS
CTBP2	10	124984317	125161170	0.063874692	PASS
CTBS	1	84549606	84574480	0.033763446	PASS
CTC1	17	8224821	8248095	0.128406487	PASS
CTCF	16	67562407	67639185	-0.371357732	PASS
CTCFL	20	57495966	57525652	-0.168668583	PASS
CTDNEP1	17	7243591	7252491	0.128406487	PASS
CTDP1	18	79679801	79756623	-0.376397843	PASS

CTDSP1	2	218398256	218405941	0.025928102	PASS
CTDSP2	12	57819927	57846739	-0.202191512	PASS
CTDSPL	3	37861960	37984469	-0.234617007	PASS
CTDSPL2	15	44427234	44529038	-0.396255771	PASS
CTF1	16	30896607	30903560	0.069936844	PASS
CTGF	6	131948176	131951373	-0.458570262	PASS
CTH	1	70411218	70439851	0.033763446	PASS
CTHRC1	8	103371515	103382997	0.279445683	PASS
CTIF	18	48539046	48863217	-0.376397843	PASS
CTLA4	2	203867771	203873965	0.025928102	PASS
CTNNA1	5	138610967	138935034	-0.293443042	PASS
CTNNA2	2	79185231	80648861	-0.372959126	PASS
CTNNA3	10	65912518	67696169	0.072405807	PASS
CTNNAL1	9	108942569	109013529	0.365190713	suppress_segment_too_large
CTNNB1	3	41194741	41260096	-0.234617007	PASS
CTNNBIP1	1	9848276	9910336	0.373964873	suppress_segment_too_large
CTNNBL1	20	37693955	37872129	-0.168668583	PASS
CTNND1	11	57753243	57819546	-0.129266491	PASS
CTNND2	5	10971840	11904043	0.055007813	PASS
CTNS	17	3636468	3661542	0.128406487	PASS
CTPS1	1	40979335	41012565	0.033763446	PASS
CTPS2	23	16588003	16712936	-0.012567603	PASS
CTR9	11	10750987	10801625	-0.405444717	PASS
CTRB1	16	75219000	75226338	-0.371357732	PASS
CTRB2	16	75204096	75207185	-0.371357732	PASS
CTRC	1	15438439	15449242	0.033763446	PASS
CTRL	16	67927640	67932414	-0.371357732	PASS
CTSA	20	45890144	45898820	-0.168668583	PASS
CTSB	8	11842524	11869448	-0.345962679	PASS
CTSC	11	88293592	88337787	0.062969153	PASS
CTSD	11	1752752	1764573	-0.333835828	PASS
CTSE	1	206009264	206023909	-0.256698369	PASS
CTSF	11	66563463	66568841	0.182439925	suppress_segment_too_large
CTSG	14	24573522	24576260	0.060212819	PASS
CTSH	15	78921058	78949574	-0.697600509	PASS
CTSK	1	150796208	150808323	0.103015788	PASS
CTSL	9	87725519	87731393	0.365190713	suppress_segment_too_large
CTSO	4	155924118	155953917	-0.011382299	PASS
CTSS	1	150730196	150765957	0.103015788	PASS
CTSV	9	97029679	97039643	0.365190713	suppress_segment_too_large
CTSW	11	65879809	65883741	0.182439925	suppress_segment_too_large
CTSZ	20	58995185	59007247	-0.168668583	PASS
CTTN	11	70398404	70436584	0.182439925	suppress_segment_too_large
CTTNBP2	7	117710651	117874139	-0.388946609	PASS
CTTNBP2N	1	112396181	112463456	0.033763446	PASS
CTU1	19	51097606	51108370	-0.016761391	PASS

CTU2	16	88706463	88715386	-0.371357732	PASS
CTXN1	19	7924485	7926166	0.079632875	PASS
CTXN2	15	48178122	48203756	-0.396255771	PASS
CTXN3	5	127649044	127658630	-0.293443042	PASS
CTXND1	15	80195484	80252213	-0.697600509	PASS
CTXND2	1	150887136	150913292	0.103015788	PASS
CUBN	10	16823966	17129817	0.039811807	PASS
CUEDC1	17	57861243	57955323	0.088516311	PASS
CUEDC2	10	102423245	102432661	-0.367947924	PASS
CUL1	7	148697914	148801036	-0.388946609	PASS
CUL2	10	35008551	35090642	0.039811807	PASS
CUL3	2	224470150	224585397	0.025928102	PASS
CUL4A	13	113208193	113267108	-0.390786075	PASS
CUL4B	23	120524609	120575794	-0.012567603	PASS
CUL5	11	108008733	108107776	-0.356188624	PASS
CUL7	6	43037617	43053945	0.048820648	PASS
CUL9	6	43182175	43224587	0.048820648	PASS
CUTA	6	33416442	33418317	0.048820648	PASS
CUTC	10	99702558	99756134	-0.367947924	PASS
CUX1	7	101815904	102283958	-0.388946609	PASS
CUX2	12	111034024	111350554	0.202141228	PASS
CUZD1	10	122832149	122850793	0.063874692	PASS
CWC15	11	94962622	94973612	0.062969153	PASS
CWC22	2	179944876	180007113	0.039726562	PASS
CWC25	17	38800434	38825481	0.060555011	PASS
CWC27	5	64768930	65018763	-0.293443042	PASS
CWF19L1	10	100232298	100267680	-0.367947924	PASS
CWF19L2	11	107326345	107457844	-0.356188624	PASS
CWH43	4	48986247	49062081	-0.011382299	PASS
CX3CL1	16	57372458	57385048	-0.371357732	PASS
CX3CR1	3	39263494	39281735	-0.234617007	PASS
CXADR	21	17512382	17593579	0.12860881	suppress_segment_too_large
CXCL1	4	73869393	73871242	-0.011382299	PASS
CXCL10	4	76021117	76023497	-0.011382299	PASS
CXCL11	4	76033682	76041415	-0.011382299	PASS
CXCL12	10	44370165	44386493	-0.404852538	PASS
CXCL13	4	77511753	77611834	-0.011382299	PASS
CXCL14	5	135570679	135579279	-0.293443042	PASS
CXCL16	17	4733526	4739922	0.128406487	PASS
CXCL17	19	42428288	42443048	0.093487731	suppress_segment_too_large
CXCL2	4	74097035	74099293	-0.011382299	PASS
CXCL3	4	74036589	74038807	-0.011382299	PASS
CXCL5	4	73995642	73998779	-0.011382299	PASS
CXCL6	4	73836497	73849064	-0.011382299	PASS
CXCL8	4	73740506	73743716	-0.011382299	PASS
CXCL9	4	76001275	76007488	-0.011382299	PASS

CXCR1	2	218162845	218166995	0.025928102	PASS
CXCR2	2	218125289	218137253	0.025928102	PASS
CXCR3	23	71615916	71618517	-0.012567603	PASS
CXCR4	2	136114349	136118165	-0.475215623	PASS
CXCR5	11	118883766	118897799	-0.356188624	PASS
CXCR6	3	45940933	45948353	-0.234617007	PASS
CXXC1	18	50282343	50288304	-0.376397843	PASS
CXXC4	4	104468312	104494901	-0.011382299	PASS
CXXC5	5	139647299	139683882	-0.293443042	PASS
CXorf21	23	30558824	30577844	-0.012567603	PASS
CXorf36	23	45148374	45200901	-0.012567603	PASS
CXorf38	23	40626921	40647554	-0.012567603	PASS
CXorf40A	23	149540355	149550510	-0.012567603	PASS
CXorf40B	23	149929527	149938811	-0.012567603	PASS
CXorf56	23	119538149	119565408	-0.012567603	PASS
CXorf57	23	106611930	106679442	-0.012567603	PASS
CXorf58	23	23907801	23939509	-0.012567603	PASS
CXorf65	23	71103889	71106788	-0.012567603	PASS
CXorf66	23	139955725	139965520	-0.012567603	PASS
CXorf67	23	51406915	51408843	-0.012567603	PASS
CYB561	17	63432304	63446378	0.088516311	PASS
CYB561A3	11	61348745	61362299	0.182439925	suppress_segment_too_large
CYB561D1	1	109494052	109502932	0.033763446	PASS
CYB561D2	3	50350695	50358460	-0.234617007	PASS
CYB5A	18	74250847	74292016	-0.376397843	PASS
CYB5B	16	69424525	69466266	-0.371357732	PASS
CYB5D1	17	7857746	7862282	0.128406487	PASS
CYB5D2	17	4143168	4187310	0.128406487	PASS
CYB5R1	1	202961869	202967280	-0.256698369	PASS
CYB5R2	11	7665100	7677222	-0.405444717	PASS
CYB5R3	22	42617840	42649568	-0.575930959	PASS
CYB5R4	6	83859643	83967424	-0.368590549	PASS
CYB5RL	1	54169651	54200036	0.033763446	PASS
CYBA	16	88643283	88651152	-0.371357732	PASS
CYBB	23	37780011	37813461	-0.012567603	PASS
CYBC1	17	82442589	82450829	0.088516311	PASS
CYBRD1	2	171522247	171558133	0.039726562	PASS
CYC1	8	144095027	144097525	0.307788765	PASS
CYCS	7	25120091	25125361	-0.69513118	PASS
CYFIP1	15	22867052	22981063	-0.396255771	PASS
CYFIP2	5	157266079	157395595	-0.150979337	PASS
CYGB	17	76527356	76551175	0.088516311	PASS
CYHR1	8	144449582	144465677	0.307788765	PASS
CYLC1	23	83861126	83886699	-0.012567603	PASS
CYLC2	9	102995311	103018488	0.365190713	suppress_segment_too_large
CYLD	16	50742050	50801935	0.069936844	PASS

CYP11A1	15	74337759	74367740	-0.697600509	PASS
CYP11B1	8	142872356	142879846	0.113969953	PASS
CYP11B2	8	142910559	142917843	0.113969953	PASS
CYP17A1	10	102830531	102837533	-0.367947924	PASS
CYP19A1	15	51208057	51338610	-0.396255771	PASS
CYP1A1	15	74719542	74725610	-0.697600509	PASS
CYP1A2	15	74748844	74756202	-0.697600509	PASS
CYP1B1	2	38066973	38109902	0.040498426	PASS
CYP20A1	2	203238940	203305611	0.025928102	PASS
CYP21A2	6	32038265	32041670	0.048820648	PASS
CYP24A1	20	54153449	54173973	-0.168668583	PASS
CYP26A1	10	93073475	93077890	-0.39678585	PASS
CYP26B1	2	72129238	72148038	-0.372959126	PASS
CYP26C1	10	93060808	93069536	-0.39678585	PASS
CYP27A1	2	218781749	218815293	0.025928102	PASS
CYP27B1	12	57762334	57768986	-0.202191512	PASS
CYP27C1	2	127184120	127220078	-0.475215623	PASS
CYP2A13	19	41088472	41096195	0.093487731	suppress_segment_too_large
CYP2A6	19	40843538	40850447	0.093487731	suppress_segment_too_large
CYP2A7	19	40875439	40882752	0.093487731	suppress_segment_too_large
CYP2B6	19	40991250	41018398	0.093487731	suppress_segment_too_large
CYP2C18	10	94683621	94736190	-0.39678585	PASS
CYP2C19	10	94762681	94853205	-0.39678585	PASS
CYP2C8	10	95036772	95069497	-0.39678585	PASS
CYP2C9	10	94938658	94989391	-0.39678585	PASS
CYP2D6	22	42126499	42130906	-0.575930959	PASS
CYP2E1	10	133520406	133561220	0.063874692	PASS
CYP2F1	19	41114432	41128366	0.093487731	suppress_segment_too_large
CYP2J2	1	59893308	59926790	0.033763446	PASS
CYP2R1	11	14877440	14892252	-0.405444717	PASS
CYP2S1	19	41193049	41207539	0.093487731	suppress_segment_too_large
CYP2U1	4	107931369	107953457	-0.011382299	PASS
CYP2W1	7	983199	989640	-0.69513118	PASS
CYP39A1	6	46549580	46652830	0.048820648	PASS
CYP3A4	7	99756960	99784265	-0.262778904	PASS
CYP3A43	7	99828013	99866102	-0.262778904	PASS
CYP3A5	7	99648194	99680026	-0.262778904	PASS
CYP3A7	7	99684957	99735196	-0.262778904	PASS
CYP3A7-C	7	99685226	99735093	-0.262778904	PASS
CYP46A1	14	99684304	99727301	-0.192197564	PASS
CYP4A11	1	46929177	46941484	0.033763446	PASS
CYP4A22	1	47137435	47149741	0.033763446	PASS
CYP4B1	1	46757838	46819413	0.033763446	PASS
CYP4F11	19	15912367	15934867	0.093487731	suppress_segment_too_large
CYP4F12	19	15672757	15697174	0.093487731	suppress_segment_too_large
CYP4F2	19	15878023	15898120	0.093487731	suppress_segment_too_large

CYP4F22	19	15508493	15552317	0.093487731	suppress_segment_too_large
CYP4F3	19	15640897	15662825	0.093487731	suppress_segment_too_large
CYP4F8	19	15615218	15630638	0.093487731	suppress_segment_too_large
CYP4V2	4	186191520	186213456	-0.462744716	PASS
CYP4X1	1	47023568	47050751	0.033763446	PASS
CYP4Z1	1	47067488	47118319	0.033763446	PASS
CYP51A1	7	92112151	92134803	-0.262778904	PASS
CYP7A1	8	58490178	58500236	-0.201142258	PASS
CYP7B1	8	64587763	64798761	-0.201142258	PASS
CYP8B1	3	42856005	42876165	-0.234617007	PASS
CYR61	1	85580761	85583962	0.033763446	PASS
CYREN	7	135092363	135170795	-0.388946609	PASS
CYS1	2	10056780	10080944	0.040498426	PASS
CYSLTR1	23	78271464	78327691	-0.012567603	PASS
CYSLTR2	13	48653711	48711226	-0.390786075	PASS
CYSRT1	9	137224635	137226311	0.365190713	suppress_segment_too_large
CYSTM1	5	140174642	140282052	-0.293443042	PASS
CYTH1	17	78674048	78782297	0.088516311	PASS
CYTH2	19	48469032	48482314	0.14996833	PASS
CYTH3	7	6161776	6272644	-0.69513118	PASS
CYTH4	22	37282027	37315345	-0.575930959	PASS
CYTIP	2	157414619	157488961	-0.475215623	PASS
CYTL1	4	5014586	5019472	0.279089364	PASS
CYYR1	21	26466209	26573284	0.12860881	suppress_segment_too_large
DAAM1	14	59188646	59371405	0.060212819	PASS
DAAM2	6	39792298	39904877	0.048820648	PASS
DAB1	1	56994778	58546734	0.033763446	PASS
DAB2	5	39371675	39462300	0.055007813	PASS
DAB2IP	9	121567057	121785530	0.365190713	suppress_segment_too_large
DACH1	13	71437966	71867192	-0.390786075	PASS
DACH2	23	86148458	86832604	-0.012567603	PASS
DACT1	14	58633967	58648321	0.060212819	PASS
DACT2	6	168292830	168319754	-0.035448111	PASS
DACT3	19	46647612	46661138	0.14996833	PASS
DAD1	14	22564905	22589269	0.060212819	PASS
DAG1	3	49468703	49535618	-0.234617007	PASS
DAGLA	11	61680433	61747001	0.182439925	suppress_segment_too_large
DAGLB	7	6409126	6484190	-0.69513118	PASS
DALRD3	3	49015488	49022293	-0.234617007	PASS
DAND5	19	12965159	12974762	0.093487731	suppress_segment_too_large
DAO	12	108858932	108901043	0.202141228	PASS
DAOA	13	105465867	105491034	-0.390786075	PASS
DAP	5	10679230	10761272	0.055007813	PASS
DAP3	1	155687960	155739010	0.103015788	PASS
DAPK1	9	87497228	87708633	0.365190713	suppress_segment_too_large
DAPK2	15	63907036	64072033	-0.697600509	PASS

DAPK3	19	3958453	3971123	0.079632875	PASS
DAPL1	2	158795317	158862781	0.039726562	PASS
DAPP1	4	99816833	99870154	-0.011382299	PASS
DARS	2	135906677	135986100	-0.475215623	PASS
DARS2	1	173824568	173858808	-0.027073548	PASS
DAW1	2	227871054	227924344	0.025928102	PASS
DAXX	6	33318558	33323016	0.048820648	PASS
DAZAP1	19	1407569	1435687	0.079632875	PASS
DAZAP2	12	51238292	51271362	-0.202191512	PASS
DAZL	3	16586792	16670306	0.311018244	suppress_segment_too_large
DBF4	7	87876216	87909541	-0.262778904	PASS
DBF4B	17	44708608	44752264	0.088516311	PASS
DBH	9	133635348	133659344	0.365190713	suppress_segment_too_large
DBI	2	119366921	119372560	-0.475215623	PASS
DBN1	5	177456608	177474401	-0.150979337	PASS
DBNDD1	16	90004865	90020128	-0.371357732	PASS
DBNDD2	20	45406057	45410610	-0.168668583	PASS
DBNL	7	44044640	44069456	-0.69513118	PASS
DBP	19	48630030	48637438	0.14996833	PASS
DBR1	3	138161012	138174949	0.313454204	suppress_segment_too_large
DBT	1	100186919	100249834	0.033763446	PASS
DBX1	11	20156155	20160613	-0.405444717	PASS
DBX2	12	45014672	45051099	-0.202191512	PASS
DCAF1	3	51395867	51500002	-0.234617007	PASS
DCAF10	9	37800502	37867666	-0.310444659	PASS
DCAF11	14	24114195	24125242	0.060212819	PASS
DCAF12	9	34086387	34127399	-0.310444659	PASS
DCAF12L1	23	126549383	126552851	-0.012567603	PASS
DCAF12L2	23	126163499	126166097	-0.012567603	PASS
DCAF13	8	103414714	103443453	0.279445683	PASS
DCAF15	19	13952492	13961449	0.093487731	suppress_segment_too_large
DCAF16	4	17800655	17810758	0.279089364	PASS
DCAF17	2	171434217	171485052	0.039726562	PASS
DCAF4	14	72926332	72959703	0.060212819	PASS
DCAF4L1	4	41981696	41986467	-0.011382299	PASS
DCAF4L2	8	87870743	87874068	0.279445683	PASS
DCAF5	14	69050881	69153150	0.060212819	PASS
DCAF6	1	167935783	168075843	-0.027073548	PASS
DCAF7	17	63550461	63594266	0.088516311	PASS
DCAF8	1	160215715	160262531	-0.027073548	PASS
DCAF8L1	23	27977993	27981449	-0.012567603	PASS
DCAF8L2	23	27590382	27748821	-0.012567603	PASS
DCAKD	17	45023340	45061109	0.088516311	PASS
DCANP1	5	135444214	135447348	-0.293443042	PASS
DCBLD1	6	117453817	117569858	-0.368590549	PASS
DCBLD2	3	98795941	98901689	0.313454204	suppress_segment_too_large

DCC	18	52340172	53535903	-0.376397843	PASS
DCD	12	54644591	54648493	-0.202191512	PASS
DCDC1	11	30830369	31369810	-0.405444717	PASS
DCDC2	6	24171756	24358052	0.048820648	PASS
DCDC2B	1	32209094	32216196	0.033763446	PASS
DCDC2C	2	3703592	3847411	0.040498426	PASS
DCHS1	11	6621323	6655854	-0.405444717	PASS
DCHS2	4	154231742	154495570	-0.011382299	PASS
DCK	4	70992538	71030914	-0.011382299	PASS
DCLK1	13	35768652	36131306	-0.390786075	PASS
DCLK2	4	150078274	150257457	-0.011382299	PASS
DCLK3	3	36712422	36764349	-0.234617007	PASS
DCLRE1A	10	113834725	113854383	0.063874692	PASS
DCLRE1B	1	113905141	113914086	0.033763446	PASS
DCLRE1C	10	14897359	14954432	0.039811807	PASS
DCN	12	91140484	91183123	0.202141228	PASS
DCP1A	3	53283428	53347610	-0.234617007	PASS
DCP1B	12	1946054	2004535	-0.086128028	PASS
DCP2	5	112976702	113020970	-0.293443042	PASS
DCPS	11	126303752	126345754	-0.419612865	PASS
DCST1	1	155033824	155050930	0.103015788	PASS
DCST2	1	155018520	155033781	0.103015788	PASS
DCSTAMP	8	104339087	104356689	0.279445683	PASS
DCT	13	94436808	94479682	-0.390786075	PASS
DCTD	4	182890060	182917936	-0.462744716	PASS
DCTN1	2	74361154	74392087	-0.372959126	PASS
DCTN2	12	57530102	57547331	-0.202191512	PASS
DCTN3	9	34613545	34620523	-0.310444659	PASS
DCTN4	5	150708440	150759109	-0.293443042	PASS
DCTN5	16	23641392	23677455	0.069936844	PASS
DCTN6	8	30156297	30183640	-0.345962679	PASS
DCTPP1	16	30423619	30430075	0.069936844	PASS
DCUN1D1	3	182938074	182985953	0.318278996	suppress_segment_too_large
DCUN1D2	13	113455819	113490952	-0.390786075	PASS
DCUN1D3	16	20854925	20900384	0.069936844	PASS
DCUN1D4	4	51843000	51916837	-0.011382299	PASS
DCUN1D5	11	103062076	103092215	0.062969153	PASS
DCX	23	111293779	111412429	-0.012567603	PASS
DCXR	17	82035136	82037732	0.088516311	PASS
DDA1	19	17309518	17323301	0.093487731	suppress_segment_too_large
DDAH1	1	85318481	85578363	0.033763446	PASS
DDAH2	6	31727038	31730617	0.048820648	PASS
DDB1	11	61299451	61342596	0.182439925	suppress_segment_too_large
DDB2	11	47214465	47239240	0.117376172	PASS
DDC	7	50458436	50565457	-0.750061359	PASS
DDHD1	14	53036745	53153282	0.060212819	PASS

DDHD2	8	38225218	38275558	-0.201142258	PASS
DDI1	11	104036580	104039188	0.062969153	PASS
DDI2	1	15617500	15669044	0.033763446	PASS
DDIAS	11	82899975	82958277	0.062969153	PASS
DDIT3	12	57516588	57521737	-0.202191512	PASS
DDIT4	10	72273920	72276036	0.072405807	PASS
DDIT4L	4	100185870	100190782	-0.011382299	PASS
DDN	12	48995149	48999309	-0.202191512	PASS
DDO	6	110391771	110415562	-0.368590549	PASS
DDOST	1	20651767	20661544	0.033763446	PASS
DDR1	6	30876421	30900156	0.048820648	PASS
DDR2	1	162631373	162787400	-0.027073548	PASS
DDRGK1	20	3190350	3204685	-0.168668583	PASS
DDT	22	23971365	23979828	-0.575930959	PASS
DDTL	22	23966901	23972532	-0.575930959	PASS
DDX1	2	15591178	15631111	0.040498426	PASS
DDX10	11	108665025	108940930	-0.356188624	PASS
DDX11	12	31073845	31104791	0.362961408	suppress_segment_too_large
DDX17	22	38483440	38507660	-0.575930959	PASS
DDX18	2	117814650	117832379	-0.475215623	PASS
DDX19A	16	70346829	70373383	-0.371357732	PASS
DDX19B	16	70289663	70335283	-0.371357732	PASS
DDX20	1	111755245	111768016	0.033763446	PASS
DDX21	10	68956128	68985073	0.072405807	PASS
DDX23	12	48829764	48852842	-0.202191512	PASS
DDX24	14	94048291	94081245	-0.192197564	PASS
DDX25	11	125903348	125943702	-0.419612865	PASS
DDX27	20	49219295	49244077	-0.168668583	PASS
DDX28	16	68021274	68023442	-0.371357732	PASS
DDX31	9	132592997	132670401	0.365190713	suppress_segment_too_large
DDX39A	19	14408819	14419383	0.093487731	suppress_segment_too_large
DDX39B	6	31530219	31542448	0.048820648	PASS
DDX3X	23	41333284	41364472	-0.012567603	PASS
DDX4	5	55738017	55817157	-0.293443042	PASS
DDX41	5	177511577	177517469	-0.150979337	PASS
DDX42	17	63773603	63819317	0.088516311	PASS
DDX43	6	73394748	73417569	-0.368590549	PASS
DDX46	5	134758771	134855133	-0.293443042	PASS
DDX47	12	12813316	12829981	0.362961408	suppress_segment_too_large
DDX49	19	18919675	18928633	0.093487731	suppress_segment_too_large
DDX5	17	64499616	64508199	0.088516311	PASS
DDX50	10	68901278	68946847	0.072405807	PASS
DDX51	12	132136594	132144335	0.270848837	PASS
DDX52	17	37609739	37643464	0.060555011	PASS
DDX53	23	22999961	23003589	-0.012567603	PASS
DDX54	12	113157174	113185479	0.202141228	PASS

DDX55	12	123602077	123620941	0.202141228	PASS
DDX56	7	44565417	44575051	-0.69513118	PASS
DDX58	9	32455705	32526324	-0.310444659	PASS
DDX59	1	200623896	200669969	-0.256698369	PASS
DDX6	11	118747766	118791149	-0.356188624	PASS
DDX60	4	168216293	168318807	-0.011382299	PASS
DDX60L	4	168356735	168537786	-0.011382299	PASS
DEAF1	11	644233	706715	-0.333835828	PASS
1-Dec	9	114850968	115402644	0.365190713	suppress_segment_too_large
DECR1	8	90001405	90052092	0.279445683	PASS
DECR2	16	401826	412487	0.069936844	PASS
DEDD	1	161120974	161132688	-0.027073548	PASS
DEDD2	19	42198598	42220140	0.093487731	suppress_segment_too_large
DEF6	6	35297852	35321771	0.048820648	PASS
DEF8	16	89947925	89968060	-0.371357732	PASS
DEFA3	8	7015869	7018301	-0.345962679	PASS
DEFA4	8	6935822	6938338	-0.345962679	PASS
DEFA5	8	7055304	7056734	-0.345962679	PASS
DEFA6	8	6924693	6926076	-0.345962679	PASS
DEFB1	8	6870575	6878022	-0.345962679	PASS
DEFB104A	8	7836471	7841242	-0.345962679	PASS
DEFB105A	8	7821966	7823889	-0.345962679	PASS
DEFB106A	8	7825172	7829053	-0.345962679	PASS
DEFB106B	8	7482504	7486400	-0.345962679	PASS
DEFB107A	8	7811720	7815716	-0.345962679	PASS
DEFB108B	11	71833200	71837710	0.062969153	PASS
DEFB110	6	50009138	50021981	0.048820648	PASS
DEFB112	6	50043575	50048651	0.048820648	PASS
DEFB113	6	49968677	49969625	0.048820648	PASS
DEFB114	6	49960249	49964105	0.048820648	PASS
DEFB115	20	31257664	31259632	-0.168668583	PASS
DEFB116	20	31303212	31308585	-0.168668583	PASS
DEFB118	20	31368618	31373923	-0.168668583	PASS
DEFB119	20	31377164	31390603	-0.168668583	PASS
DEFB121	20	31404845	31412838	-0.168668583	PASS
DEFB123	20	31440519	31450257	-0.168668583	PASS
DEFB124	20	31465506	31476757	-0.168668583	PASS
DEFB125	20	87250	97094	-0.168668583	PASS
DEFB126	20	142369	145751	-0.168668583	PASS
DEFB127	20	157470	159163	-0.168668583	PASS
DEFB128	20	187853	189681	-0.168668583	PASS
DEFB129	20	227258	229886	-0.168668583	PASS
DEFB131A	4	9444534	9450514	0.279089364	PASS
DEFB131B	11	71878453	71884561	0.062969153	PASS
DEFB132	20	257736	261096	-0.168668583	PASS
DEFB133	6	49946021	49950265	0.048820648	PASS

DEFB134	8	11993174	11996312	-0.345962679	PASS
DEFB135	8	11982321	11984590	-0.345962679	PASS
DEFB136	8	11973937	11974599	-0.345962679	PASS
DEFB4A	8	7894629	7896711	-0.345962679	PASS
DEFB4B	8	7414860	7416863	-0.345962679	PASS
DEGS1	1	224175756	224193441	-0.256698369	PASS
DEGS2	14	100143957	100160163	-0.192197564	PASS
DEK	6	18223868	18264823	0.048820648	PASS
DELE1	5	141923808	141942047	-0.293443042	PASS
DENND1A	9	123379654	123930152	0.365190713	suppress_segment_too_large
DENND1B	1	197504748	197775696	-0.027073548	PASS
DENND1C	19	6467207	6482557	0.079632875	PASS
DENND2A	7	140518420	140673993	-0.388946609	PASS
DENND2C	1	114582848	114670422	0.033763446	PASS
DENND2D	1	111187174	111204535	0.033763446	PASS
DENND3	8	141117278	141195808	0.113969953	PASS
DENND4A	15	65658046	65792293	-0.697600509	PASS
DENND4B	1	153929501	153946696	0.103015788	PASS
DENND4C	9	19230435	19373545	-0.310444659	PASS
DENND5A	11	9138825	9265390	-0.405444717	PASS
DENND5B	12	31382223	31591097	0.362961408	suppress_segment_too_large
DENND6A	3	57625457	57693089	-0.234617007	PASS
DENND6B	22	50309030	50327060	-0.808211634	PASS
DENR	12	122752774	122771064	0.202141228	PASS
DEPDC1	1	68474152	68497221	0.033763446	PASS
DEPDC1B	5	60596912	60700190	-0.293443042	PASS
DEPDC4	12	100203669	100267079	0.202141228	PASS
DEPDC5	22	31753867	31908033	-0.575930959	PASS
DEPDC7	11	33015864	33033582	-0.405444717	PASS
DEPP1	10	44970981	44978810	-0.404852538	PASS
DEPTOR	8	119873717	120050913	0.279445683	PASS
DERA	12	15911172	16037282	0.362961408	suppress_segment_too_large
DERL1	8	123013164	123042423	0.279445683	PASS
DERL2	17	5471251	5486811	0.128406487	PASS
DERL3	22	23834503	23839128	-0.575930959	PASS
DES	2	219418377	219426739	0.025928102	PASS
DESI1	22	41598028	41621096	-0.575930959	PASS
DESI2	1	244652935	244709033	-0.368111568	PASS
DET1	15	88494440	88546675	-0.697600509	PASS
DEUP1	11	93329971	93438487	0.062969153	PASS
DEXI	16	10928891	10942460	0.069936844	PASS
DFFA	1	10456522	10472526	0.373964873	suppress_segment_too_large
DFFB	1	3857267	3885429	0.373964873	suppress_segment_too_large
DGAT1	8	144314584	144326910	0.307788765	PASS
DGAT2	11	75759512	75801535	0.062969153	PASS
DGAT2L6	23	70177483	70205545	-0.012567603	PASS

DGCR2	22	19036282	19122454	-0.575930959	PASS
DGCR6	22	18906028	18914238	-0.575930959	PASS
DGCR6L	22	20314276	20320080	-0.575930959	PASS
DGCR8	22	20080232	20111877	-0.575930959	PASS
DGKA	12	55927319	55954027	-0.202191512	PASS
DGKB	7	14145049	14974777	-0.69513118	PASS
DGKD	2	233354507	233472104	0.025928102	PASS
DGKE	17	56834099	56869567	0.088516311	PASS
DGKG	3	186105668	186362237	0.318278996	suppress_segment_too_large
DGKH	13	42040036	42256578	-0.390786075	PASS
DGKI	7	137381037	137847092	-0.388946609	PASS
DGKK	23	50365409	50470738	-0.012567603	PASS
DGKQ	4	958887	986895	0.279089364	PASS
DGKZ	11	46332905	46380554	0.117376172	PASS
DGLUCY	14	91060333	91225632	-0.192197564	PASS
DGUOK	2	73926826	73958961	-0.372959126	PASS
DHCR24	1	54849627	54887271	0.033763446	PASS
DHCR7	11	71428193	71452868	0.182439925	suppress_segment_too_large
DHDDS	1	26432282	26471294	0.033763446	PASS
DHDH	19	48933682	48944969	0.14996833	PASS
DHFR	5	80626228	80654983	-0.293443042	PASS
DHFR2	3	94047836	94063389	0.313454204	suppress_segment_too_large
DHH	12	49086656	49094819	-0.202191512	PASS
DHODH	16	72008588	72027664	-0.371357732	PASS
DHPS	19	12675717	12681902	0.093487731	suppress_segment_too_large
DHRS1	14	24290598	24299833	0.060212819	PASS
DHRS11	17	36591798	36600806	0.060555011	PASS
DHRS12	13	51767993	51804157	-0.390786075	PASS
DHRS13	17	28897781	28903071	0.060555011	PASS
DHRS2	14	23630115	23645639	0.060212819	PASS
DHRS3	1	12567910	12617731	0.003932739	PASS
DHRS4	14	23953586	23969279	0.060212819	PASS
DHRS4L2	14	23969874	24006408	0.060212819	PASS
DHRS7	14	60144120	60169856	0.060212819	PASS
DHRS7B	17	21123364	21193265	0.024610076	PASS
DHRS7C	17	9771434	9791297	-0.139031004	PASS
DHRS9	2	169064789	169096167	0.039726562	PASS
DHRSX	23	2219516	2502805	-0.012567603	PASS
DHTKD1	10	12068972	12123225	0.039811807	PASS
DHX15	4	24517441	24584550	0.279089364	PASS
DHX16	6	30653119	30673037	0.048820648	PASS
DHX29	5	55256245	55307722	-0.293443042	PASS
DHX30	3	47802909	47850195	-0.234617007	PASS
DHX32	10	125836337	125896436	0.063874692	PASS
DHX33	17	5440912	5469060	0.128406487	PASS
DHX34	19	47349281	47382704	0.14996833	PASS

DHX35	20	38962299	39039723	-0.168668583	PASS
DHX36	3	154272546	154324497	0.318278996	suppress_segment_too_large
DHX37	12	124946825	124989122	0.202141228	PASS
DHX38	16	72093562	72112912	-0.371357732	PASS
DHX40	17	59565525	59608345	0.088516311	PASS
DHX57	2	38797729	38875934	0.040498426	PASS
DHX58	17	42101404	42112733	0.060555011	PASS
DHX8	17	43483865	43610338	0.088516311	PASS
DHX9	1	182839369	182887751	-0.027073548	PASS
DIABLO	12	122207662	122227534	0.202141228	PASS
DIAPH1	5	141515016	141619055	-0.293443042	PASS
DIAPH2	23	96684663	97604997	-0.012567603	PASS
DIAPH3	13	59665583	60163987	-0.390786075	PASS
DICER1	14	95086228	95158010	-0.192197564	PASS
DIDO1	20	62877738	62937952	-0.168668583	PASS
DIMT1	5	62387254	62403939	-0.293443042	PASS
DIO1	1	53891239	53911086	0.033763446	PASS
DIO2	14	80197527	80387757	-0.192197564	PASS
DIO3	14	101561351	101563452	-0.192197564	PASS
DIP2A	21	46458899	46569852	-0.064425456	suppress_segment_too_large
DIP2B	12	50504985	50748667	-0.202191512	PASS
DIP2C	10	274190	689668	0.039811807	PASS
DIRAS1	19	2714567	2721418	0.079632875	PASS
DIRAS2	9	90609832	90643105	0.365190713	suppress_segment_too_large
DIRAS3	1	68045896	68051631	0.033763446	PASS
DIRC1	2	188733738	188790104	0.025928102	PASS
DIRC2	3	122794795	122881139	0.313454204	suppress_segment_too_large
DIS3	13	72752169	72782096	-0.390786075	PASS
DIS3L	15	66293217	66333898	-0.697600509	PASS
DIS3L2	2	231961245	232344350	0.025928102	PASS
DISC1	1	231626815	232041272	-0.256698369	PASS
DISP1	1	222872271	223005995	-0.256698369	PASS
DISP2	15	40358235	40378639	-0.396255771	PASS
DISP3	1	11479166	11537584	0.373964873	suppress_segment_too_large
DIXDC1	11	111927144	112022584	-0.356188624	PASS
DKC1	23	154762742	154777689	-0.012567603	PASS
DKK1	10	52314296	52318042	-0.404852538	PASS
DKK2	4	106921802	107283806	-0.011382299	PASS
DKK3	11	11963106	12009769	-0.405444717	PASS
DKK4	8	42374068	42377232	-0.201142258	PASS
DKKL1	19	49361783	49375116	0.14996833	PASS
DLAT	11	112024814	112064390	-0.356188624	PASS
DLC1	8	13083361	13604610	-0.345962679	PASS
DLD	7	107890970	107931730	-0.388946609	PASS
DLEC1	3	38039205	38124025	-0.234617007	PASS
DLEU7	13	50711008	50843939	-0.390786075	PASS

DLG1	3	197042560	197299300	0.326834516	PASS
DLG2	11	83455012	85627922	0.062969153	PASS
DLG3	23	70444861	70505490	-0.012567603	PASS
DLG4	17	7187169	7219841	0.128406487	PASS
DLG5	10	77790791	77926526	0.072405807	PASS
DLGAP1	18	3496032	4455335	-0.052305056	suppress_segment_too_large
DLGAP2	8	737596	1708474	-0.313874214	PASS
DLGAP3	1	34865436	34929585	0.033763446	PASS
DLGAP4	20	36306336	36528637	-0.168668583	PASS
DLGAP5	14	55148112	55191678	0.060212819	PASS
DLK1	14	100725705	100738224	-0.192197564	PASS
DLK2	6	43450352	43456632	0.048820648	PASS
DLL1	6	170282206	170306565	-0.035448111	PASS
DLL3	19	39498895	39508481	0.093487731	suppress_segment_too_large
DLL4	15	40929333	40939073	-0.396255771	PASS
DLST	14	74881891	74903745	-0.192197564	PASS
DLX1	2	172084740	172089677	0.039726562	PASS
DLX2	2	172099439	172102900	0.039726562	PASS
DLX3	17	49990005	49995224	0.088516311	PASS
DLX4	17	49968970	49974959	0.088516311	PASS
DLX5	7	97020392	97024950	-0.262778904	PASS
DLX6	7	97005548	97011039	-0.262778904	PASS
DMAC1	9	7796490	7888380	-0.310444659	PASS
DMAC2	19	41431318	41440717	0.093487731	suppress_segment_too_large
DMAP1	1	44213455	44220681	0.033763446	PASS
DMBT1	10	122560665	122643736	0.063874692	PASS
DMBX1	1	46506996	46514226	0.033763446	PASS
DMC1	22	38518949	38570286	-0.575930959	PASS
DMD	23	31097677	33339441	-0.012567603	PASS
DMGDH	5	78997606	79236038	-0.293443042	PASS
DMKN	19	35497220	35513658	0.093487731	suppress_segment_too_large
DMP1	4	87650307	87664361	-0.011382299	PASS
DMPK	19	45769717	45782552	0.14996833	PASS
DMRT1	9	841690	969090	-0.310444659	PASS
DMRT2	9	1049858	1057552	-0.310444659	PASS
DMRT3	9	976964	991731	-0.310444659	PASS
DMRTA1	9	22446841	22455740	-0.310444659	PASS
DMRTA2	1	50417550	50423500	0.033763446	PASS
DMRTB1	1	53459399	53467488	0.033763446	PASS
DMRTC1	23	72872025	72943814	-0.012567603	PASS
DMRTC1E	23	72777009	72848802	-0.012567603	PASS
DMRTC2	19	41844743	41852333	0.093487731	suppress_segment_too_large
DMTF1	7	87152361	87196337	-0.262778904	PASS
DMTN	8	22048995	22082527	-0.345962679	PASS
DMWD	19	45782947	45792802	0.14996833	PASS
DMXL1	5	119037772	119249138	-0.293443042	PASS

DMXL2	15	51447711	51622833	-0.396255771	PASS
DNA2	10	68414064	68472121	0.072405807	PASS
DNAAF1	16	84145287	84178767	-0.371357732	PASS
DNAAF2	14	49625174	49635230	0.060212819	PASS
DNAAF3	19	55158661	55166722	0.121336758	PASS
DNAAF4	15	55410525	55508234	-0.697600509	PASS
DNAAF5	7	726701	786475	-0.69513118	PASS
DNAH1	3	52316319	52400491	-0.234617007	PASS
DNAH10	12	123762188	123936206	0.202141228	PASS
DNAH11	7	21543215	21901839	-0.69513118	PASS
DNAH12	3	57293699	57544344	-0.234617007	PASS
DNAH14	1	224896262	225399292	-0.256698369	PASS
DNAH17	17	78423697	78577394	0.088516311	PASS
DNAH2	17	7717354	7833744	0.128406487	PASS
DNAH3	16	20933111	21159441	0.069936844	PASS
DNAH5	5	13690331	13944543	0.055007813	PASS
DNAH6	2	84516455	84819589	0.066915641	PASS
DNAH7	2	195737703	196068812	0.025928102	PASS
DNAH8	6	38715341	39030529	0.048820648	PASS
DNAH9	17	11598431	11969748	-0.139031004	PASS
DNAI1	9	34457414	34520989	-0.310444659	PASS
DNAI2	17	74274247	74314884	0.088516311	PASS
DNAJA1	9	33025211	33039907	-0.310444659	PASS
DNAJA2	16	46955362	46973788	0.069936844	PASS
DNAJA3	16	4425805	4456775	0.069936844	PASS
DNAJA4	15	78264086	78282196	-0.697600509	PASS
DNAJB1	19	14514770	14529770	0.093487731	suppress_segment_too_large
DNAJB11	3	186567403	186585800	0.318278996	suppress_segment_too_large
DNAJB12	10	72332830	72355230	0.072405807	PASS
DNAJB13	11	73950319	73970366	0.062969153	PASS
DNAJB14	4	99896248	99946726	-0.011382299	PASS
DNAJB2	2	219279267	219286900	0.025928102	PASS
DNAJB4	1	77979175	78017964	0.033763446	PASS
DNAJB5	9	34989641	34998900	-0.310444659	PASS
DNAJB6	7	157335381	157417439	-0.411415677	PASS
DNAJB7	22	40859549	40862126	-0.575930959	PASS
DNAJB8	3	128462439	128467248	0.313454204	suppress_segment_too_large
DNAJB9	7	108569568	108574850	-0.388946609	PASS
DNAJC1	10	21756537	22003769	0.039811807	PASS
DNAJC10	2	182716041	182794464	-0.169541924	PASS
DNAJC11	1	6634168	6701924	0.373964873	suppress_segment_too_large
DNAJC12	10	67796665	67838166	0.072405807	PASS
DNAJC13	3	132417526	132539032	0.313454204	suppress_segment_too_large
DNAJC14	12	55820960	55830824	-0.202191512	PASS
DNAJC15	13	43023203	43114224	-0.390786075	PASS
DNAJC16	1	15526813	15592379	0.033763446	PASS

DNAJC17	15	40765155	40807478	-0.396255771	PASS
DNAJC18	5	139408588	139444491	-0.293443042	PASS
DNAJC19	3	180983709	180989774	0.318278996	suppress_segment_too_large
DNAJC2	7	103312474	103344873	-0.388946609	PASS
DNAJC21	5	34929559	34958964	0.055007813	PASS
DNAJC22	12	49346917	49357546	-0.202191512	PASS
DNAJC24	11	31369840	31431849	-0.405444717	PASS
DNAJC25	9	111631352	111654351	0.365190713	suppress_segment_too_large
DNAJC25-	9	111631386	111670229	0.365190713	suppress_segment_too_large
DNAJC27	2	24943636	24972094	0.040498426	PASS
DNAJC28	21	33485530	33491720	0.12860881	suppress_segment_too_large
DNAJC3	13	95677139	95794989	-0.390786075	PASS
DNAJC30	7	73680969	73683453	-0.262778904	PASS
DNAJC4	11	64230278	64234286	0.182439925	suppress_segment_too_large
DNAJC5	20	63895182	63936031	-0.168668583	PASS
DNAJC5B	8	66021560	66100526	-0.201142258	PASS
DNAJC5G	2	27275421	27281499	0.040498426	PASS
DNAJC6	1	65248219	65415869	0.033763446	PASS
DNAJC7	17	41976433	42021376	0.060555011	PASS
DNAJC8	1	28199456	28233025	0.033763446	PASS
DNAJC9	10	73183362	73248862	0.072405807	PASS
DNAL1	14	73644875	73703732	0.060212819	PASS
DNAL4	22	38778508	38794198	-0.575930959	PASS
DNALI1	1	37556919	37566857	0.033763446	PASS
DNASE1	16	3611728	3680143	0.069936844	PASS
DNASE1L	23	154401238	154412112	-0.012567603	PASS
DNASE1L[illegible]	16	2235816	2238711	0.069936844	PASS
DNASE1L[illegible]	3	58192257	58214697	-0.234617007	PASS
DNASE2	19	12875211	12881468	0.093487731	suppress_segment_too_large
DNASE2B	1	84398532	84415018	0.033763446	PASS
DND1	5	140670794	140673586	-0.293443042	PASS
DNER	2	229357629	229714558	0.025928102	PASS
DNHD1	11	6497260	6593758	-0.405444717	PASS
DNLZ	9	136359480	136363789	0.365190713	suppress_segment_too_large
DNM1	9	128191655	128255248	0.365190713	suppress_segment_too_large
DNM1L	12	32679200	32745650	0.362961408	suppress_segment_too_large
DNM2	19	10718079	10833488	0.093487731	suppress_segment_too_large
DNM3	1	171841498	172418466	-0.027073548	PASS
DNMBP	10	99875577	100009919	-0.367947924	PASS
DNMT1	19	10133345	10231286	0.093487731	suppress_segment_too_large
DNMT3A	2	25227855	25342590	0.040498426	PASS
DNMT3B	20	32762385	32809356	-0.168668583	PASS
DNMT3L	21	44246339	44262216	-0.064425456	suppress_segment_too_large
DNPEP	2	219373527	219400022	0.025928102	PASS
DNPH1	6	43225629	43229484	0.048820648	PASS
DNTT	10	96304396	96338564	-0.367947924	PASS

DNTTIP1	20	45791937	45811427	-0.168668583	PASS
DNTTIP2	1	93866283	93879918	0.033763446	PASS
DOC2A	16	30005509	30023270	0.069936844	PASS
DOC2B	17	142789	181636	0.128406487	PASS
DOCK1	10	126905409	127452517	0.063874692	PASS
DOCK10	2	224765090	225042445	0.025928102	PASS
DOCK11	23	118495898	118686163	-0.012567603	PASS
DOCK2	5	169637247	170083382	-0.150979337	PASS
DOCK3	3	50675241	51384198	-0.234617007	PASS
DOCK4	7	111726110	112206411	-0.388946609	PASS
DOCK5	8	25184723	25418082	-0.345962679	PASS
DOCK6	19	11199295	11262481	0.093487731	suppress_segment_too_large
DOCK7	1	62454298	62688368	0.033763446	PASS
DOCK8	9	214854	465259	-0.310444659	PASS
DOCK9	13	98793429	99086625	-0.390786075	PASS
DOHH	19	3490822	3500940	0.079632875	PASS
DOK1	2	74549026	74557554	-0.372959126	PASS
DOK2	8	21908873	21913860	-0.345962679	PASS
DOK3	5	177501907	177511274	-0.150979337	PASS
DOK4	16	57471922	57487327	-0.371357732	PASS
DOK5	20	54475597	54651171	-0.168668583	PASS
DOK6	18	69401055	69849087	-0.376397843	PASS
DOK7	4	3463311	3501473	0.279089364	PASS
DOLK	9	128945530	128947619	0.365190713	suppress_segment_too_large
DOLPP1	9	129081100	129090438	0.365190713	suppress_segment_too_large
DONSON	21	33559542	33588708	0.12860881	suppress_segment_too_large
DOP1A	6	83067666	83171350	-0.368590549	PASS
DOP1B	21	36156782	36294274	-0.064425456	suppress_segment_too_large
DOT1L	19	2164149	2232578	0.079632875	PASS
DPAGT1	11	119096503	119108331	-0.356188624	PASS
DPCD	10	101570560	101609668	-0.367947924	PASS
DPEP1	16	89613308	89638456	-0.371357732	PASS
DPEP2	16	67987394	68000586	-0.371357732	PASS
DPEP2NB	16	68013436	68015911	-0.371357732	PASS
DPEP3	16	67975663	67980829	-0.371357732	PASS
DPF1	19	38211006	38229714	0.093487731	suppress_segment_too_large
DPF2	11	65333754	65353249	0.182439925	suppress_segment_too_large
DPF3	14	72619296	72894116	0.060212819	PASS
DPH1	17	2030110	2043430	0.128406487	PASS
DPH2	1	43970000	43973369	0.033763446	PASS
DPH3	3	16257978	16264972	0.311018244	suppress_segment_too_large
DPH5	1	100989623	101026088	0.033763446	PASS
DPH6	15	35217345	35546193	-0.396255771	PASS
DPH7	9	137554904	137578935	0.365190713	suppress_segment_too_large
DPM1	20	50934867	50958555	-0.168668583	PASS
DPM2	9	127935099	127938484	0.365190713	suppress_segment_too_large

DPM3	1	155139891	155140595	0.103015788	PASS
DPP10	2	114442299	115845752	-0.010901494	PASS
DPP3	11	66480013	66509657	0.182439925	suppress_segment_too_large
DPP4	2	161992241	162074542	0.039726562	PASS
DPP6	7	153887097	154894285	-0.411415677	PASS
DPP7	9	137110542	137115177	0.365190713	suppress_segment_too_large
DPP8	15	65442463	65517704	-0.697600509	PASS
DPP9	19	4675224	4724673	0.079632875	PASS
DPPA2	3	109293788	109316517	0.313454204	suppress_segment_too_large
DPPA3	12	7711454	7717559	-0.086128028	PASS
DPPA4	3	109326141	109337572	0.313454204	suppress_segment_too_large
DPPA5	6	73353062	73354295	-0.368590549	PASS
DPRX	19	53632056	53637009	0.206912349	PASS
DPT	1	168695459	168729264	-0.027073548	PASS
DPY19L1	7	34928876	35038271	-0.69513118	PASS
DPY19L2	12	63558913	63668939	-0.202191512	PASS
DPY19L3	19	32405543	32485895	0.093487731	suppress_segment_too_large
DPY19L4	8	94719703	94793836	0.279445683	PASS
DPY30	2	31867809	32039812	0.040498426	PASS
DPYD	1	97077743	97995000	0.033763446	PASS
DPYS	8	104330324	104467053	0.279445683	PASS
DPYSL2	8	26514022	26658178	-0.345962679	PASS
DPYSL3	5	147390811	147510056	-0.293443042	PASS
DPYSL4	10	132186900	132205776	0.063874692	PASS
DPYSL5	2	26847747	26950351	0.040498426	PASS
DQX1	2	74518131	74526336	-0.372959126	PASS
DR1	1	93345888	93369498	0.033763446	PASS
DRAM1	12	101877351	102012130	0.202141228	PASS
DRAM2	1	111117333	111140216	0.033763446	PASS
DRAP1	11	65919257	65921561	0.182439925	suppress_segment_too_large
DRAXIN	1	11691729	11725857	0.373964873	suppress_segment_too_large
DRC1	2	26401916	26456711	0.040498426	PASS
DRC3	17	17972813	18016889	0.024610076	PASS
DRC7	16	57694793	57731805	-0.371357732	PASS
DRD1	5	175440039	175444208	-0.150979337	PASS
DRD2	11	113409615	113475691	-0.356188624	PASS
DRD3	3	114128652	114199407	0.313454204	suppress_segment_too_large
DRD4	11	637293	640706	-0.333835828	PASS
DRD5	4	9781680	9784009	0.279089364	PASS
DRG1	22	31399523	31530634	-0.575930959	PASS
DRG2	17	18087886	18107971	0.024610076	PASS
DRGX	10	49364181	49396016	-0.404852538	PASS
DRICH1	22	23608452	23632321	-0.575930959	PASS
DROSHA	5	31400497	31532196	0.055007813	PASS
DRP2	23	101219769	101264497	-0.012567603	PASS
DSC1	18	31129236	31162856	-0.376397843	PASS

DSC2	18	31058840	31102522		-0.376397843	PASS
DSC3	18	30990008	31042815		-0.376397843	PASS
DSCAM	21	40010999	40847139		-0.064425456	suppress_segment_too_large
DSCAML1	11	117427773	117817525		-0.356188624	PASS
DSCC1	8	119833976	119856010		0.279445683	PASS
DSE	6	116254173	116444860		-0.368590549	PASS
DSEL	18	67506582	67516980		-0.376397843	PASS
DSG1	18	31318089	31357029		-0.376397843	PASS
DSG2	18	31498043	31549008		-0.376397843	PASS
DSG3	18	31447795	31478702		-0.376397843	PASS
DSG4	18	31376777	31414912		-0.376397843	PASS
DSN1	20	36751791	36773818		-0.168668583	PASS
DSP	6	7541575	7586717		0.048820648	PASS
DSPP	4	87608553	87616910		-0.011382299	PASS
DST	6	56457987	56954628		0.048820648	PASS
DSTN	20	17569863	17609919		-0.168668583	PASS
DSTYK	1	205142505	205211566		-0.256698369	PASS
DTD1	20	18587893	18766644		-0.168668583	PASS
DTD2	14	31446036	31457510		0.060212819	PASS
DTHD1	4	36281622	36345756		-0.011382299	PASS
DTL	1	212035553	212107400		-0.256698369	PASS
DTNA	18	34493290	34891844		-0.376397843	PASS
DTNB	2	25377198	25673647		0.040498426	PASS
DTNBP1	6	15522801	15663058		0.048820648	PASS
DTWD1	15	49620980	49656232		-0.396255771	PASS
DTWD2	5	118837322	118988545		-0.293443042	PASS
DTX1	12	113056709	113098028		0.202141228	PASS
DTX2	7	76461676	76505995		-0.262778904	PASS
DTX3	12	57604622	57609804		-0.202191512	PASS
DTX3L	3	122564238	122575203		0.313454204	suppress_segment_too_large
DTX4	11	59171430	59208587		-0.129266491	PASS
DUOX1	15	45129933	45165576		-0.396255771	PASS
DUOX2	15	45092650	45114344		-0.396255771	PASS
DUOXA1	15	45117367	45129938		-0.396255771	PASS
DUOXA2	15	45114321	45118421		-0.396255771	PASS
DUPD1	10	75037836	75058514		0.072405807	PASS
DUS1L	17	82057506	82065887		0.088516311	PASS
DUS2	16	67987746	68079320		-0.371357732	PASS
DUS3L	19	5784832	5791238		0.079632875	PASS
DUS4L	7	107563484	107578464		-0.388946609	PASS
DUSP1	5	172768090	172771195		-0.150979337	PASS
DUSP10	1	221701424	221742176		-0.256698369	PASS
DUSP11	2	73762184	73780157		-0.372959126	PASS
DUSP12	1	161749758	161757238		-0.027073548	PASS
DUSP13	10	75094432	75109221		0.072405807	PASS
DUSP14	17	37489831	37513501		0.060555011	PASS

DUSP15	20	31847637	31870747	-0.168668583	PASS
DUSP16	12	12474210	12562383	0.362961408	suppress_segment_too_large
DUSP18	22	30652051	30667890	-0.575930959	PASS
DUSP19	2	183078559	183100005	-0.169541924	PASS
DUSP2	2	96143166	96145440	0.048238121	PASS
DUSP21	23	44844004	44844888	-0.012567603	PASS
DUSP22	6	291630	351355	0.048820648	PASS
DUSP23	1	159780932	159782543	-0.467855874	PASS
DUSP26	8	33591332	33600106	-0.345962679	PASS
DUSP27	1	167094045	167129165	-0.027073548	PASS
DUSP28	2	240560054	240564014	0.025928102	PASS
DUSP3	17	43766121	43778988	0.088516311	PASS
DUSP4	8	29333064	29350668	-0.345962679	PASS
DUSP5	10	110497838	110511544	0.063874692	PASS
DUSP6	12	89347232	89353271	0.202141228	PASS
DUSP7	3	52048919	52056550	-0.234617007	PASS
DUSP8	11	1554044	1571920	-0.333835828	PASS
DUSP9	23	153642492	153651326	-0.012567603	PASS
DUT	15	48331011	48343373	-0.396255771	PASS
DUXA	19	57154021	57167443	-0.180829161	PASS
DVL1	1	1335276	1349350	0.373964873	suppress_segment_too_large
DVL2	17	7225341	7234545	0.128406487	PASS
DVL3	3	184155311	184173614	0.318278996	suppress_segment_too_large
DXO	6	31969810	31972292	0.048820648	PASS
DYDC1	10	80336105	80356755	0.072405807	PASS
DYDC2	10	80344745	80368073	0.072405807	PASS
DYM	18	49041474	49461347	-0.376397843	PASS
DYNAP	18	54587757	54599493	-0.376397843	PASS
DYNC1H1	14	101964510	102056443	-0.192197564	PASS
DYNC1I1	7	95772506	96110322	-0.262778904	PASS
DYNC1I2	2	171687409	171748420	0.039726562	PASS
DYNC1LI1	3	32525971	32570874	0.311018244	suppress_segment_too_large
DYNC1LI2	16	66720893	66751798	-0.371357732	PASS
DYNC2H1	11	103109410	103479863	0.062969153	PASS
DYNC2LI1	2	43774039	43810010	0.040498426	PASS
DYNLL1	12	120469850	120498493	0.202141228	PASS
DYNLL2	17	58083415	58095536	0.088516311	PASS
DYNLRB1	20	34516409	34540958	-0.168668583	PASS
DYNLRB2	16	80540734	80550760	-0.371357732	PASS
DYNLT1	6	158636474	158644739	-0.035448111	PASS
DYNLT3	23	37836757	37847637	-0.012567603	PASS
DYRK1A	21	37365573	37526358	-0.064425456	suppress_segment_too_large
DYRK1B	19	39825350	39834201	0.093487731	suppress_segment_too_large
DYRK2	12	67648338	67665406	-0.202191512	PASS
DYRK3	1	206635536	206684419	-0.256698369	PASS
DYRK4	12	4562204	4615302	-0.086128028	PASS

DYSF	2	71453722	71686768	-0.372959126	PASS
DYTN	2	206651621	206718396	0.025928102	PASS
DZANK1	20	18383367	18467281	-0.168668583	PASS
DZIP1	13	95578202	95644703	-0.390786075	PASS
DZIP1L	3	138061990	138115818	0.313454204	suppress_segment_too_large
DZIP3	3	108589682	108694846	0.313454204	suppress_segment_too_large
E2F1	20	33675683	33686404	-0.168668583	PASS
E2F2	1	23506430	23531220	0.033763446	PASS
E2F3	6	20401906	20493715	0.048820648	PASS
E2F4	16	67192169	67198918	-0.371357732	PASS
E2F5	8	85177225	85217158	0.279445683	PASS
E2F6	2	11444375	11466177	0.040498426	PASS
E2F7	12	77021247	77065580	0.202141228	PASS
E2F8	11	19224063	19241620	-0.405444717	PASS
E4F1	16	2223566	2235742	0.069936844	PASS
EAF1	3	15427355	15450635	0.311018244	suppress_segment_too_large
EAF2	3	121835183	121886526	0.313454204	suppress_segment_too_large
EAPP	14	34515929	34539711	0.060212819	PASS
EARS2	16	23522014	23557731	0.069936844	PASS
EBAG9	8	109539711	109565996	0.279445683	PASS
EBF1	5	158695916	159099761	-0.150979337	PASS
EBF2	8	25841730	26045397	-0.345962679	PASS
EBF3	10	129835283	129963841	0.063874692	PASS
EBF4	20	2692878	2760108	-0.168668583	PASS
EBI3	19	4229498	4237531	0.079632875	PASS
EBLN1	10	22208814	22210021	0.039811807	PASS
EBLN2	3	73061659	73063337	-0.234617007	PASS
EBNA1BP2	1	43164175	43270936	0.033763446	PASS
EBP	23	48521158	48528716	-0.012567603	PASS
EBPL	13	49660723	49691475	-0.390786075	PASS
ECD	10	73130155	73169055	0.072405807	PASS
ECE1	1	21217247	21345572	0.033763446	PASS
ECE2	3	184276011	184293031	0.318278996	suppress_segment_too_large
ECEL1	2	232479827	232487828	0.025928102	PASS
ECH1	19	38815422	38832005	0.093487731	suppress_segment_too_large
ECHDC1	6	127288710	127343609	-0.458570262	PASS
ECHDC2	1	52895910	52927212	0.033763446	PASS
ECHDC3	10	11742366	11764070	0.039811807	PASS
ECHS1	10	133362480	133373689	0.063874692	PASS
ECI1	16	2239395	2252300	0.069936844	PASS
ECI2	6	4115689	4135597	0.048820648	PASS
ECM1	1	150508062	150513789	0.103015788	PASS
ECM2	9	92493554	92536655	0.365190713	suppress_segment_too_large
ECPAS	9	111360692	111484745	0.365190713	suppress_segment_too_large
ECSCR	5	139448556	139462743	-0.293443042	PASS
ECSIT	19	11505916	11529174	0.093487731	suppress_segment_too_large

ECT2	3	172750682	172821474	0.318278996	suppress_segment_too_large
ECT2L	6	138795926	138904070	-0.035448111	PASS
EDA	23	69616067	70039469	-0.012567603	PASS
EDA2R	23	66595637	66639298	-0.012567603	PASS
EDAR	2	108894471	108989372	-0.010901494	PASS
EDARADI	1	236348257	236502915	-0.256698369	PASS
EDC3	15	74630558	74696292	-0.697600509	PASS
EDC4	16	67873023	67884503	-0.371357732	PASS
EDDM3A	14	20745892	20748380	0.060212819	PASS
EDDM3B	14	20768427	20770948	0.060212819	PASS
EDEM1	3	5187646	5219957	0.311018244	suppress_segment_too_large
EDEM2	20	35115357	35147364	-0.168668583	PASS
EDEM3	1	184690231	184754913	-0.027073548	PASS
EDF1	9	136862119	136866286	0.365190713	suppress_segment_too_large
EDIL3	5	83940554	84384793	-0.293443042	PASS
EDN1	6	12290363	12297194	0.048820648	PASS
EDN2	1	41478775	41484673	0.033763446	PASS
EDN3	20	59300427	59325992	-0.168668583	PASS
EDNRA	4	147480917	147544954	-0.011382299	PASS
EDNRB	13	77895481	77975529	-0.390786075	PASS
EDRF1	10	125719515	125764143	0.063874692	PASS
EEA1	12	92770637	92929331	0.202141228	PASS
EED	11	86244544	86278813	0.062969153	PASS
EEF1A1	6	73515750	73523797	-0.368590549	PASS
EEF1A2	20	63488012	63499322	-0.168668583	PASS
EEF1AKM	13	20728731	20773958	-0.390786075	PASS
EEF1AKM	10	124748149	124791870	0.063874692	PASS
EEF1AKM	12	57771492	57782541	-0.202191512	PASS
EEF1AKM	3	184249650	184259585	0.318278996	suppress_segment_too_large
EEF1AKM	3	184249695	184293031	0.318278996	suppress_segment_too_large
EEF1B2	2	206159585	206162928	0.025928102	PASS
EEF1D	8	143579697	143599541	0.307788765	PASS
EEF1E1	6	8073360	8102578	0.048820648	PASS
EEF1E1-BI	6	8015726	8102530	0.048820648	PASS
EEF1G	11	62559601	62574086	0.182439925	suppress_segment_too_large
EEF2	19	3976056	3985469	0.079632875	PASS
EEF2K	16	22206282	22288732	0.069936844	PASS
EEF2KMT	16	5084304	5097808	0.069936844	PASS
EEFSEC	3	128153454	128408646	0.313454204	suppress_segment_too_large
EEPD1	7	36153149	36301543	-0.69513118	PASS
EFCAB1	8	48710789	48735311	-0.201142258	PASS
EFCAB10	7	105565120	105600875	-0.388946609	PASS
EFCAB11	14	89794669	89954777	-0.192197564	PASS
EFCAB12	3	129401321	129428651	0.313454204	suppress_segment_too_large
EFCAB13	17	47323290	47441312	0.088516311	PASS
EFCAB14	1	46675159	46719064	0.033763446	PASS

EFCAB2	1	244969705	245127164	-0.368111568	PASS
EFCAB3	17	62343941	62416479	0.088516311	PASS
EFCAB5	17	29929200	30108452	0.060555011	PASS
EFCAB6	22	43528744	43812337	-0.575930959	PASS
EFCAB7	1	63523372	63572693	0.033763446	PASS
EFCAB8	20	32858923	32961609	-0.168668583	PASS
EFCAB9	5	172194172	172203452	-0.150979337	PASS
EFCC1	3	129001629	129040742	0.313454204	suppress_segment_too_large
EFEMP1	2	55865967	55924139	0.040498426	PASS
EFEMP2	11	65866441	65873592	0.182439925	suppress_segment_too_large
EFHB	3	19879472	19947025	0.311018244	suppress_segment_too_large
EFHC1	6	52362123	52529886	0.048820648	PASS
EFHC2	23	44147882	44343672	-0.012567603	PASS
EFHD1	2	232606057	232682781	0.025928102	PASS
EFHD2	1	15409895	15430343	0.033763446	PASS
EFL1	15	82130230	82262763	-0.697600509	PASS
EFNA1	1	155127460	155134857	0.103015788	PASS
EFNA2	19	1286154	1300237	0.079632875	PASS
EFNA3	1	155078872	155087538	0.103015788	PASS
EFNA4	1	155063731	155069553	0.103015788	PASS
EFNA5	5	107376889	107670895	-0.293443042	PASS
EFNB1	23	68828997	68842147	-0.012567603	PASS
EFNB2	13	106489731	106535662	-0.390786075	PASS
EFNB3	17	7705202	7711378	0.128406487	PASS
EFR3A	8	131904088	132013642	0.113969953	PASS
EFR3B	2	25042130	25159137	0.040498426	PASS
EFS	14	23356402	23365752	0.060212819	PASS
EFTUD2	17	44849943	44899662	0.088516311	PASS
EGF	4	109912884	110012266	-0.011382299	PASS
EGFL6	23	13569605	13633575	-0.012567603	PASS
EGFL7	9	136658856	136672678	0.365190713	suppress_segment_too_large
EGFL8	6	32164583	32168281	0.048820648	PASS
EGFLAM	5	38258409	38465021	0.055007813	PASS
EGFR	7	55019021	55211628	-0.750061359	PASS
EGLN1	1	231363751	231425044	-0.256698369	PASS
EGLN2	19	40798996	40808433	0.093487731	suppress_segment_too_large
EGLN3	14	33924231	34462774	0.060212819	PASS
EGR1	5	138465490	138469315	-0.293443042	PASS
EGR2	10	62811996	62919900	0.072405807	PASS
EGR3	8	22687659	22693302	-0.345962679	PASS
EGR4	2	73290929	73293705	-0.372959126	PASS
EHBP1	2	62673851	63046487	-0.372959126	PASS
EHBP1L1	11	65576038	65592650	0.182439925	suppress_segment_too_large
EHD1	11	64851642	64888296	0.182439925	suppress_segment_too_large
EHD2	19	47713343	47743134	0.14996833	PASS
EHD3	2	31234337	31269447	0.040498426	PASS

EHD4	15	41895939	41972578	-0.396255771	PASS
EHF	11	34621093	34661057	-0.405444717	PASS
EHHADH	3	185190624	185281990	0.318278996	suppress_segment_too_large
EHMT1	9	137618963	137870016	0.365190713	suppress_segment_too_large
EHMT2	6	31879759	31897687	0.048820648	PASS
EI24	11	125569216	125584687	-0.419612865	PASS
EID1	15	48877886	48880183	-0.396255771	PASS
EID2	19	39538705	39540333	0.093487731	suppress_segment_too_large
EID2B	19	39530990	39532854	0.093487731	suppress_segment_too_large
EID3	12	104303739	104305205	0.202141228	PASS
EIF1	17	41688885	41692668	0.060555011	PASS
EIF1AD	11	65996545	66002176	0.182439925	suppress_segment_too_large
EIF1AX	23	20124518	20141844	-0.012567603	PASS
EIF1B	3	40309684	40312424	-0.234617007	PASS
EIF2A	3	150546678	150584242	0.318278996	suppress_segment_too_large
EIF2AK1	7	6022244	6059230	-0.69513118	PASS
EIF2AK2	2	37099210	37157065	0.040498426	PASS
EIF2AK3	2	88556741	88627576	-0.340781246	PASS
EIF2AK4	15	39934146	40035591	-0.396255771	PASS
EIF2B1	12	123620406	123633766	0.202141228	PASS
EIF2B2	14	75002911	75012366	-0.192197564	PASS
EIF2B3	1	44850522	44986722	0.033763446	PASS
EIF2B4	2	27364352	27370486	0.040498426	PASS
EIF2B5	3	184135038	184146112	0.318278996	suppress_segment_too_large
EIF2D	1	206571292	206612463	-0.256698369	PASS
EIF2S1	14	67359997	67386516	0.060212819	PASS
EIF2S2	20	34088298	34112332	-0.168668583	PASS
EIF2S3	23	24054716	24077971	-0.012567603	PASS
EIF2S3B	12	10505602	10523135	0.362961408	suppress_segment_too_large
EIF3A	10	119033670	119080823	0.063874692	PASS
EIF3B	7	2354086	2380745	-0.69513118	PASS
EIF3C	16	28688558	28735730	0.069936844	PASS
EIF3D	22	36510850	36529436	-0.575930959	PASS
EIF3E	8	108201216	108435333	0.279445683	PASS
EIF3F	11	7970251	8001862	-0.405444717	PASS
EIF3G	19	10115017	10119918	0.093487731	suppress_segment_too_large
EIF3H	8	116642130	116766925	0.279445683	PASS
EIF3I	1	32221928	32231604	0.033763446	PASS
EIF3J	15	44537057	44563029	-0.396255771	PASS
EIF3K	19	38619082	38636955	0.093487731	suppress_segment_too_large
EIF3L	22	37848868	37889407	-0.575930959	PASS
EIF3M	11	32583798	32606262	-0.405444717	PASS
EIF4A1	17	7572706	7579005	0.128406487	PASS
EIF4A2	3	186783205	186789900	0.318278996	suppress_segment_too_large
EIF4A3	17	80135214	80147183	0.088516311	PASS
EIF4B	12	53006158	53042209	-0.202191512	PASS

EIF4E	4	98871684	98930637	-0.011382299	PASS
EIF4E1B	5	176630618	176646641	-0.150979337	PASS
EIF4E2	2	232550052	232583644	0.025928102	PASS
EIF4E3	3	71675416	71754773	-0.234617007	PASS
EIF4EBP1	8	38030341	38060365	-0.201142258	PASS
EIF4EBP2	10	70404379	70428618	0.072405807	PASS
EIF4EBP3	5	140547666	140549578	-0.293443042	PASS
EIF4ENIF1	22	31436977	31496108	-0.575930959	PASS
EIF4G1	3	184314495	184335358	0.318278996	suppress_segment_too_large
EIF4G2	11	10797050	10809110	-0.405444717	PASS
EIF4G3	1	20806292	21176888	0.033763446	PASS
EIF4H	7	74174245	74197101	-0.262778904	PASS
EIF5	14	103333544	103345025	-0.192197564	PASS
EIF5A	17	7306999	7312463	0.128406487	PASS
EIF5A2	3	170888415	170908693	0.318278996	suppress_segment_too_large
EIF5AL1	10	79512601	79516440	0.072405807	PASS
EIF5B	2	99337353	99401326	0.048238121	PASS
EIF6	20	35278911	35284985	-0.168668583	PASS
EIPR1	2	3188925	3377882	0.040498426	PASS
ELAC1	18	50967991	50988121	-0.376397843	PASS
ELAC2	17	12992391	13018187	-0.139031004	PASS
ELANE	19	851014	856247	0.079632875	PASS
ELAVL1	19	7958579	8005659	0.079632875	PASS
ELAVL2	9	23690104	23826337	-0.310444659	PASS
ELAVL3	19	11451326	11481046	0.093487731	suppress_segment_too_large
ELAVL4	1	50048014	50203786	0.033763446	PASS
ELF1	13	40932028	41061440	-0.390786075	PASS
ELF2	4	139028112	139177218	-0.011382299	PASS
ELF3	1	202007945	202017188	-0.256698369	PASS
ELF4	23	130064874	130110716	-0.012567603	PASS
ELF5	11	34478793	34513805	-0.405444717	PASS
ELFN1	7	1688119	1747954	-0.69513118	PASS
ELFN2	22	37367960	37427470	-0.575930959	PASS
ELK1	23	47635521	47650604	-0.012567603	PASS
ELK3	12	96194382	96269835	0.202141228	PASS
ELK4	1	205597556	205631962	-0.256698369	PASS
ELL	19	18442663	18522127	0.093487731	suppress_segment_too_large
ELL2	5	95885098	95962071	-0.293443042	PASS
ELL3	15	43772600	43777543	-0.396255771	PASS
ELMO1	7	36854361	37449249	-0.69513118	PASS
ELMO2	20	46366049	46432985	-0.168668583	PASS
ELMO3	16	67199111	67204029	-0.371357732	PASS
ELMOD1	11	107591091	107666779	-0.356188624	PASS
ELMOD2	4	140524158	140553770	-0.011382299	PASS
ELMOD3	2	85354394	85391752	0.066915641	PASS
ELMSAN1	14	73715122	73790285	0.060212819	PASS

ELN	7	74027789	74069907	-0.262778904	PASS
ELOA	1	23743155	23762059	0.033763446	PASS
ELOA2	18	47032572	47035621	-0.376397843	PASS
ELOA3C	18	46968695	46969912	-0.376397843	PASS
ELOA3D	18	46962768	46964408	-0.376397843	PASS
ELOB	16	2771414	2777297	0.069936844	PASS
ELOC	8	73939169	73972287	-0.201142258	PASS
ELOF1	19	11551147	11559236	0.093487731	suppress_segment_too_large
ELOVL1	1	43363397	43368074	0.033763446	PASS
ELOVL2	6	10980759	11044314	0.048820648	PASS
ELOVL3	10	102226328	102229589	-0.367947924	PASS
ELOVL4	6	79914812	79947580	-0.368590549	PASS
ELOVL5	6	53267398	53349179	0.048820648	PASS
ELOVL6	4	110045846	110199199	-0.011382299	PASS
ELOVL7	5	60751791	60844389	-0.293443042	PASS
ELP1	9	108867517	108934116	0.365190713	suppress_segment_too_large
ELP2	18	36129444	36180556	-0.376397843	PASS
ELP3	8	28089673	28191156	-0.345962679	PASS
ELP4	11	31509700	31790328	-0.405444717	PASS
ELP5	17	7251416	7259940	0.128406487	PASS
ELP6	3	47495640	47513761	-0.234617007	PASS
ELSPBP1	19	47994651	48025153	0.14996833	PASS
EMB	5	50396192	50443248	-0.293443042	PASS
EMC1	1	19215664	19251552	0.033763446	PASS
EMC10	19	50476400	50490870	-0.016761391	PASS
EMC2	8	108443601	108486918	0.279445683	PASS
EMC3	3	9962537	10011116	0.311018244	suppress_segment_too_large
EMC4	15	34224999	34230156	-0.396255771	PASS
EMC6	17	3668815	3669668	0.128406487	PASS
EMC7	15	34084017	34101948	-0.396255771	PASS
EMC8	16	85771758	85799608	-0.371357732	PASS
EMC9	14	24138959	24141588	0.060212819	PASS
EMCN	4	100395341	100880126	-0.011382299	PASS
EMD	23	154379197	154381523	-0.012567603	PASS
EME1	17	50373220	50381483	0.088516311	PASS
EME2	16	1773207	1781708	0.069936844	PASS
EMG1	12	6970893	6997428	-0.086128028	PASS
EMID1	22	29205851	29259597	-0.575930959	PASS
EMILIN1	2	27078567	27086408	0.040498426	PASS
EMILIN2	18	2847030	2915993	-0.052305056	suppress_segment_too_large
EMILIN3	20	41359966	41366827	-0.168668583	PASS
EML1	14	99737693	99942060	-0.192197564	PASS
EML2	19	45606994	45645629	0.14996833	PASS
EML3	11	62602218	62612765	0.182439925	suppress_segment_too_large
EML4	2	42169350	42332548	0.040498426	PASS
EML5	14	88612431	88792752	-0.192197564	PASS

EML6	2	54723499	54972025	0.040498426	PASS
EMP1	12	13196716	13219939	0.362961408	suppress_segment_too_large
EMP2	16	10528422	10580698	0.069936844	PASS
EMP3	19	48321509	48330553	0.14996833	PASS
EMSY	11	76444923	76553025	0.062969153	PASS
EMX1	2	72916260	72936071	-0.372959126	PASS
EMX2	10	117542444	117549546	0.063874692	PASS
EN1	2	118842171	118847678	-0.475215623	PASS
EN2	7	155458129	155464831	-0.411415677	PASS
ENAH	1	225486835	225653142	-0.256698369	PASS
ENAM	4	70628744	70686816	-0.011382299	PASS
ENC1	5	74627406	74641424	-0.293443042	PASS
ENDOD1	11	95089810	95132645	0.062969153	PASS
ENDOG	9	128818474	128822677	0.365190713	suppress_segment_too_large
ENDOU	12	47709734	47725567	-0.202191512	PASS
ENDOV	17	80415165	80438086	0.088516311	PASS
ENG	9	127815012	127854756	0.365190713	suppress_segment_too_large
ENGASE	17	79074939	79088599	0.088516311	PASS
ENHO	9	34521040	34523041	-0.310444659	PASS
ENKD1	16	67662945	67667265	-0.371357732	PASS
ENKUR	10	24981979	25062279	0.039811807	PASS
ENO1	1	8861000	8879250	0.373964873	suppress_segment_too_large
ENO2	12	6913745	6923698	-0.086128028	PASS
ENO3	17	4948092	4957131	0.128406487	PASS
ENO4	10	116849512	116911788	0.063874692	PASS
ENOPH1	4	82430562	82461091	-0.011382299	PASS
ENOSF1	18	670324	712676	-0.052305056	suppress_segment_too_large
ENOX1	13	43213518	43786908	-0.390786075	PASS
ENOX2	23	130623369	130903317	-0.012567603	PASS
ENPEP	4	110365733	110565285	-0.011382299	PASS
ENPP1	6	131808016	131895155	-0.458570262	PASS
ENPP2	8	119557086	119673453	0.279445683	PASS
ENPP3	6	131628442	131747418	-0.458570262	PASS
ENPP4	6	46129993	46146699	0.048820648	PASS
ENPP5	6	46159187	46170971	0.048820648	PASS
ENPP6	4	184088706	184221230	-0.462744716	PASS
ENPP7	17	79730919	79742222	0.088516311	PASS
ENSA	1	150600851	150629612	0.103015788	PASS
ENTHD1	22	39743044	39893864	-0.575930959	PASS
ENTPD1	10	95711779	95869695	-0.39678585	PASS
ENTPD2	9	137048098	137054045	0.365190713	suppress_segment_too_large
ENTPD3	3	40387156	40428744	-0.234617007	PASS
ENTPD4	8	23385783	23457695	-0.345962679	PASS
ENTPD5	14	73958010	74019399	0.060212819	PASS
ENTPD6	20	25195693	25226729	-0.168668583	PASS
ENTPD7	10	99659506	99706240	-0.367947924	PASS

ENTPD8	9	137434364	137441816	0.365190713	suppress_segment_too_large
ENTR1	9	136401922	136410609	0.365190713	suppress_segment_too_large
ENY2	8	109334324	109345953	0.279445683	PASS
EOGT	3	68975214	69013961	-0.234617007	PASS
EOMES	3	27715949	27722711	0.311018244	suppress_segment_too_large
EP300	22	41091786	41180079	-0.575930959	PASS
EP400	12	131949920	132081102	0.270848837	PASS
EPAS1	2	46293667	46386703	0.040498426	PASS
EPB41	1	28887091	29120046	0.033763446	PASS
EPB41L1	20	36091504	36232799	-0.168668583	PASS
EPB41L2	6	130839347	131063322	-0.458570262	PASS
EPB41L3	18	5392381	5630700	-0.052305056	suppress_segment_too_large
EPB41L4A	5	112142441	112419316	-0.293443042	PASS
EPB41L4B	9	109171975	109320964	0.365190713	suppress_segment_too_large
EPB41L5	2	120013005	120179119	-0.475215623	PASS
EPB42	15	43197227	43221125	-0.396255771	PASS
EPC1	10	32267751	32378798	0.039811807	PASS
EPC2	2	148644440	148787568	-0.475215623	PASS
EPCAM	2	47345158	47387601	0.040498426	PASS
EPDR1	7	37683843	37951941	-0.69513118	PASS
EPG5	18	45847609	45967339	-0.376397843	PASS
EPGN	4	74308473	74315307	-0.011382299	PASS
EPHA1	7	143390289	143408892	-0.388946609	PASS
EPHA10	1	37713880	37765133	0.033763446	PASS
EPHA2	1	16124337	16156087	0.033763446	PASS
EPHA3	3	89107524	89482134	0.313454204	suppress_segment_too_large
EPHA4	2	221418027	221574202	0.025928102	PASS
EPHA5	4	65319563	65670495	-0.011382299	PASS
EPHA6	3	96814581	97752460	0.313454204	suppress_segment_too_large
EPHA7	6	93240020	93419547	-0.368590549	PASS
EPHA8	1	22563564	22603594	0.033763446	PASS
EPHB1	3	134597801	135260467	0.313454204	suppress_segment_too_large
EPHB2	1	22710839	22921500	0.033763446	PASS
EPHB3	3	184561784	184582409	0.318278996	suppress_segment_too_large
EPHB4	7	100802565	100827521	-0.262778904	PASS
EPHB6	7	142855061	142871094	-0.388946609	PASS
EPHX1	1	225810092	225845563	-0.256698369	PASS
EPHX2	8	27490779	27545564	-0.345962679	PASS
EPHX3	19	15226919	15233435	0.093487731	suppress_segment_too_large
EPHX4	1	92029982	92063536	0.033763446	PASS
EPM2A	6	145382535	145736023	-0.035448111	PASS
EPM2AIP1	3	36985043	36993168	-0.234617007	PASS
EPN1	19	55675226	55709858	0.121336758	PASS
EPN2	17	19215615	19336715	0.024610076	PASS
EPN3	17	50532543	50543750	0.088516311	PASS
EPO	7	100720800	100723700	-0.262778904	PASS

EPOP	17	38671703	38675421	0.060555011	PASS
EPOR	19	11377205	11384342	0.093487731	suppress_segment_too_large
EPPIN	20	45540626	45547752	-0.168668583	PASS
EPPIN-WF	20	45536986	45547426	-0.168668583	PASS
EPPK1	8	143857324	143878464	0.307788765	PASS
EPRS	1	219968601	220046658	-0.256698369	PASS
EPS15	1	51354263	51519328	0.033763446	PASS
EPS15L1	19	16355239	16472085	0.093487731	suppress_segment_too_large
EPS8	12	15620158	15882329	0.362961408	suppress_segment_too_large
EPS8L1	19	55072020	55087923	0.121336758	PASS
EPS8L2	11	694438	727727	-0.333835828	PASS
EPS8L3	1	109750080	109764027	0.033763446	PASS
EPSTI1	13	42886388	42992271	-0.390786075	PASS
EPX	17	58192737	58205174	0.088516311	PASS
EPYC	12	90963679	91005026	0.202141228	PASS
EQTN	9	27284659	27297139	-0.310444659	PASS
ERAL1	17	28854938	28861067	0.060555011	PASS
ERAP1	5	96760810	96808100	-0.293443042	PASS
ERAP2	5	96875939	96919716	-0.293443042	PASS
ERAS	23	48826513	48830138	-0.012567603	PASS
ERBB2	17	39687914	39730426	0.060555011	PASS
ERBB3	12	56076799	56103505	-0.202191512	PASS
ERBB4	2	211375717	212538841	0.025928102	PASS
ERBIN	5	65926475	66082549	-0.293443042	PASS
ERC1	12	990509	1495933	-0.086128028	PASS
ERC2	3	55508308	56468363	-0.234617007	PASS
ERCC1	19	45407333	45478828	0.14996833	PASS
ERCC2	19	45349837	45370918	0.14996833	PASS
ERCC3	2	127257290	127294176	-0.475215623	PASS
ERCC4	16	13920157	13952345	0.069936844	PASS
ERCC5	13	102807146	102876001	-0.390786075	PASS
ERCC6	10	49455368	49539538	-0.404852538	PASS
ERCC6L	23	72204657	72239047	-0.012567603	PASS
ERCC6L2	9	95875701	96014571	0.365190713	suppress_segment_too_large
ERCC8	5	60873831	60945073	-0.293443042	PASS
EREG	4	74365143	74388751	-0.011382299	PASS
ERF	19	42247572	42255157	0.093487731	suppress_segment_too_large
ERFE	2	238158982	238168900	0.025928102	PASS
ERG	21	38380027	38661780	-0.064425456	suppress_segment_too_large
ERG28	14	75649791	75661189	-0.192197564	PASS
ERGIC1	5	172834275	172952685	-0.150979337	PASS
ERGIC2	12	29337352	29381189	0.362961408	suppress_segment_too_large
ERGIC3	20	35542021	35557634	-0.168668583	PASS
ERH	14	69380123	69398627	0.060212819	PASS
ERI1	8	9002147	9116746	-0.345962679	PASS
ERI2	16	20780193	20900349	0.069936844	PASS

ERI3	1	44221070	44355260	0.033763446	PASS
ERICH1	8	614746	738106	-0.313874214	PASS
ERICH2	2	170783786	170798971	0.039726562	PASS
ERICH3	1	74568111	74673738	0.033763446	PASS
ERICH4	19	41443158	41444765	0.093487731	suppress_segment_too_large
ERICH5	8	98064311	98093610	0.279445683	PASS
ERICH6	3	150659885	150703971	0.318278996	suppress_segment_too_large
ERICH6B	13	45534522	45615739	-0.390786075	PASS
ERLEC1	2	53787044	53818819	0.040498426	PASS
ERLIN1	10	100150094	100188334	-0.367947924	PASS
ERLIN2	8	37736601	37759101	-0.201142258	PASS
ERMAP	1	42817088	42844993	0.033763446	PASS
ERMARD	6	169751622	169781584	-0.035448111	PASS
ERMN	2	157318625	157327713	-0.475215623	PASS
ERMP1	9	5765076	5833117	-0.310444659	PASS
ERN1	17	64039142	64130819	0.088516311	PASS
ERN2	16	23690326	23713500	0.069936844	PASS
ERO1A	14	52639916	52695900	0.060212819	PASS
ERO1B	1	236215555	236281985	-0.256698369	PASS
ERP27	12	14914035	14939082	0.362961408	suppress_segment_too_large
ERP29	12	112013316	112023451	0.202141228	PASS
ERP44	9	99979179	100099040	0.365190713	suppress_segment_too_large
ERRFI1	1	8004404	8026308	0.373964873	suppress_segment_too_large
ERV3-1	7	64990809	65006743	-0.750061359	PASS
ERVFRD-1	6	11102489	11111732	0.048820648	PASS
ERVK3-1	19	58305319	58315663	0.063781462	PASS
ERVMER3	4	52722618	52751640	-0.011382299	PASS
ERVV-1	19	53013921	53016122	0.206912349	PASS
ERVV-2	19	53044738	53051076	0.206912349	PASS
ERVW-1	7	92468380	92477986	-0.262778904	PASS
ESAM	11	124752583	124762290	-0.419612865	PASS
ESCO1	18	21529281	21600884	-0.376397843	PASS
ESCO2	8	27771949	27812640	-0.345962679	PASS
ESD	13	46771256	46797232	-0.390786075	PASS
ESF1	20	13714322	13784886	-0.168668583	PASS
ESM1	5	54977864	55022671	-0.293443042	PASS
ESPL1	12	53268299	53293643	-0.202191512	PASS
ESPN	1	6424776	6461367	0.373964873	suppress_segment_too_large
ESPNL	2	238100157	238133287	0.025928102	PASS
ESR1	6	151656691	152129619	-0.035448111	PASS
ESR2	14	64084232	64338112	0.060212819	PASS
ESRP1	8	94641074	94707466	0.279445683	PASS
ESRP2	16	68229111	68238102	-0.371357732	PASS
ESRRA	11	64305572	64316743	0.182439925	suppress_segment_too_large
ESRRB	14	76310712	76501837	-0.192197564	PASS
ESRRG	1	216503246	217137755	-0.256698369	PASS

ESS2	22	19130279	19144684	-0.575930959	PASS
ESX1	23	104250038	104254933	-0.012567603	PASS
ESYT1	12	56118250	56144671	-0.202191512	PASS
ESYT2	7	158730995	158830253	-0.411415677	PASS
ESYT3	3	138434586	138481686	0.313454204	suppress_segment_too_large
ETAA1	2	67397319	67412089	-0.372959126	PASS
ETF1	5	138506095	138543300	-0.293443042	PASS
ETFA	15	76215355	76311472	-0.697600509	PASS
ETFB	19	51345169	51366418	-0.016761391	PASS
ETFBKMT	12	31647160	31673114	0.362961408	suppress_segment_too_large
ETFDH	4	158672125	158709623	-0.011382299	PASS
ETFRF1	12	25195216	25209645	0.362961408	suppress_segment_too_large
ETHE1	19	43506719	43527244	0.14996833	PASS
ETNK1	12	22625075	22690665	0.362961408	suppress_segment_too_large
ETNK2	1	204131062	204152003	-0.256698369	PASS
ETNPPL	4	108742040	108763054	-0.011382299	PASS
ETS1	11	128458761	128587558	-0.419612865	PASS
ETS2	21	38805307	38824955	-0.064425456	suppress_segment_too_large
ETV1	7	13891228	13991425	-0.69513118	PASS
ETV2	19	35641745	35644871	0.093487731	suppress_segment_too_large
ETV3	1	157121191	157138474	0.103015788	PASS
ETV3L	1	157092044	157099808	0.103015788	PASS
ETV4	17	43527844	43579620	0.088516311	PASS
ETV5	3	186046308	186110318	0.318278996	suppress_segment_too_large
ETV6	12	11649854	11895402	0.362961408	suppress_segment_too_large
ETV7	6	36354091	36387800	0.048820648	PASS
EVA1A	2	75469302	75569722	-0.372959126	PASS
EVA1B	1	36322031	36324154	0.033763446	PASS
EVA1C	21	32412006	32515397	0.12860881	suppress_segment_too_large
EVC	4	5711197	5814305	0.279089364	PASS
EVC2	4	5542772	5709548	0.279089364	PASS
EVI2A	17	31317560	31321884	0.060555011	PASS
EVI2B	17	31303766	31314112	0.060555011	PASS
EVI5	1	92508696	92792404	0.033763446	PASS
EVI5L	19	7830233	7864976	0.079632875	PASS
EVL	14	99971449	100144236	-0.192197564	PASS
EVPL	17	76004502	76027452	0.088516311	PASS
EVPLL	17	18377662	18389647	0.024610076	PASS
EVX1	7	27242700	27250493	-0.69513118	PASS
EVX2	2	176077472	176083913	0.039726562	PASS
EWSR1	22	29268009	29300525	-0.575930959	PASS
EXD1	15	41182725	41230743	-0.396255771	PASS
EXD2	14	69191511	69244020	0.060212819	PASS
EXD3	9	137306896	137423262	0.365190713	suppress_segment_too_large
EXO1	1	241847967	241895148	-0.648598641	PASS
EXO5	1	40508741	40516556	0.033763446	PASS

EXOC1	4	55853616	55905034	-0.011382299	PASS
EXOC2	6	485133	693111	0.048820648	PASS
EXOC3	5	443158	471937	0.055007813	PASS
EXOC3L1	16	67184366	67190204	-0.371357732	PASS
EXOC3L2	19	45212621	45245431	0.14996833	PASS
EXOC3L4	14	103100144	103110559	-0.192197564	PASS
EXOC4	7	133253073	134066589	-0.388946609	PASS
EXOC5	14	57200507	57269008	0.060212819	PASS
EXOC6	10	92834713	93059493	-0.39678585	PASS
EXOC6B	2	72175984	72826041	-0.372959126	PASS
EXOC7	17	76081017	76121576	0.088516311	PASS
EXOC8	1	231332753	231337852	-0.256698369	PASS
EXOG	3	38496127	38542161	-0.234617007	PASS
EXOSC1	10	97436142	97446017	-0.367947924	PASS
EXOSC10	1	11066618	11099881	0.373964873	suppress_segment_too_large
EXOSC2	9	130693721	130704894	0.365190713	suppress_segment_too_large
EXOSC3	9	37766978	37801437	-0.310444659	PASS
EXOSC4	8	144078626	144080647	0.307788765	PASS
EXOSC5	19	41386374	41397479	0.093487731	suppress_segment_too_large
EXOSC6	16	70246778	70251930	-0.371357732	PASS
EXOSC7	3	44975241	45036066	-0.234617007	PASS
EXOSC8	13	36998816	37009613	-0.390786075	PASS
EXOSC9	4	121801317	121817021	-0.011382299	PASS
EXPH5	11	108505431	108593738	-0.356188624	PASS
EXT1	8	117794490	118111853	0.279445683	PASS
EXT2	11	44095549	44245429	0.117376172	PASS
EXTL1	1	26019884	26036464	0.033763446	PASS
EXTL2	1	100872372	100895998	0.033763446	PASS
EXTL3	8	28600469	28755599	-0.345962679	PASS
EYA1	8	71197433	71592025	-0.201142258	PASS
EYA2	20	46894624	47188844	-0.168668583	PASS
EYA3	1	27970344	28088696	0.033763446	PASS
EYA4	6	133240598	133532120	-0.458570262	PASS
EYS	6	63719980	65707225	0.048820648	PASS
EZH1	17	42700275	42745049	0.088516311	PASS
EZH2	7	148807383	148884321	-0.388946609	PASS
EZR	6	158765741	158819412	-0.035448111	PASS
F10	13	113122814	113149529	-0.390786075	PASS
F11	4	186265945	186288806	-0.462744716	PASS
F11R	1	160995211	161021348	-0.027073548	PASS
F12	5	177402140	177409576	-0.150979337	PASS
F13A1	6	6144085	6321013	0.048820648	PASS
F13B	1	197038741	197067267	-0.027073548	PASS
F2	11	46719180	46739506	0.117376172	PASS
F2R	5	76716043	76735781	-0.293443042	PASS
F2RL1	5	76818933	76835315	-0.293443042	PASS

F2RL2	5	76615482	76623434	-0.293443042	PASS
F2RL3	19	16888860	16892606	0.093487731	suppress_segment_too_large
F3	1	94529225	94541800	0.033763446	PASS
F5	1	169514166	169586588	-0.027073548	PASS
F7	13	113105788	113120681	-0.390786075	PASS
F8	23	154835788	155026940	-0.012567603	PASS
F8A1	23	154886349	154888061	-0.012567603	PASS
F9	23	139530758	139563458	-0.012567603	PASS
FA2H	16	74712955	74774831	-0.371357732	PASS
FAAH	1	46394265	46413848	0.033763446	PASS
FAAH2	23	57286706	57489196	-0.012567603	PASS
FAAP100	17	81539885	81553961	0.088516311	PASS
FAAP20	1	2184461	2212720	0.373964873	suppress_segment_too_large
FAAP24	19	32972209	32978222	0.093487731	suppress_segment_too_large
FABP1	2	88122982	88128116	-0.340781246	PASS
FABP12	8	81524981	81531378	0.279445683	PASS
FABP2	4	119317250	119322390	-0.011382299	PASS
FABP3	1	31365625	31376850	0.033763446	PASS
FABP4	8	81478419	81483263	0.279445683	PASS
FABP5	8	81280363	81284777	0.279445683	PASS
FABP6	5	160187367	160238735	-0.150979337	PASS
FABP7	6	122779475	122784074	-1.97829255	PASS
FABP9	8	81458341	81461579	0.279445683	PASS
FADD	11	70203163	70207390	0.182439925	suppress_segment_too_large
FADS1	11	61799625	61829318	0.182439925	suppress_segment_too_large
FADS2	11	61792980	61867354	0.182439925	suppress_segment_too_large
FADS3	11	61873519	61892051	0.182439925	suppress_segment_too_large
FADS6	17	74877299	74893781	0.088516311	PASS
FAF1	1	50437028	50960263	0.033763446	PASS
FAF2	5	176447628	176510074	-0.150979337	PASS
FAH	15	80152490	80186946	-0.697600509	PASS
FAHD1	16	1826941	1840207	0.069936844	PASS
FAHD2A	2	95402721	95416616	0.048238121	PASS
FAHD2B	2	97083583	97094882	0.048238121	PASS
FAIM	3	138608606	138633376	0.313454204	suppress_segment_too_large
FAIM2	12	49866896	49904217	-0.202191512	PASS
FAM102A	9	127940579	127980513	0.365190713	suppress_segment_too_large
FAM102B	1	108560089	108644900	0.033763446	PASS
FAM104A	17	73207353	73236753	0.088516311	PASS
FAM104B	23	55143102	55161310	-0.012567603	PASS
FAM107A	3	58564117	58627610	-0.234617007	PASS
FAM107B	10	14518557	14774897	0.039811807	PASS
FAM110A	20	833715	857463	-0.168668583	PASS
FAM110B	8	57994509	58204279	-0.201142258	PASS
FAM110C	2	38814	46870	0.040498426	PASS
FAM110D	1	26159020	26163962	0.033763446	PASS

FAM111A	11	59142748	59155039	-0.129266491	PASS
FAM111B	11	59107185	59127410	-0.129266491	PASS
FAM114A	4	38867677	38945739	-0.011382299	PASS
FAM114A	5	153990128	154038936	-0.293443042	PASS
FAM117A	17	49710332	49789180	0.088516311	PASS
FAM117B	2	202635188	202769757	0.025928102	PASS
FAM118A	22	45308968	45341955	-0.808211634	PASS
FAM118B	11	126211414	126262986	-0.419612865	PASS
FAM120A	9	93451722	93566107	0.365190713	suppress_segment_too_large
FAM120A	9	93431441	93453592	0.365190713	suppress_segment_too_large
FAM120B	6	170290703	170407065	-0.035448111	PASS
FAM120C	23	54068324	54183281	-0.012567603	PASS
FAM122A	9	68780034	68784608	-0.310444659	PASS
FAM122B	23	134769566	134797232	-0.012567603	PASS
FAM122C	23	134796395	134854835	-0.012567603	PASS
FAM124A	13	51222334	51284241	-0.390786075	PASS
FAM124B	2	224378698	224402085	0.025928102	PASS
FAM126A	7	22889371	23014130	-0.69513118	PASS
FAM126B	2	200973718	201071671	0.025928102	PASS
FAM129A	1	184790724	184974550	-0.027073548	PASS
FAM129B	9	127505339	127578989	0.365190713	suppress_segment_too_large
FAM129C	19	17523301	17553839	0.093487731	suppress_segment_too_large
FAM131A	3	184335926	184348421	0.318278996	suppress_segment_too_large
FAM131B	7	143353400	143362770	-0.388946609	PASS
FAM131C	1	16057769	16073632	0.033763446	PASS
FAM133A	23	93674013	93712274	-0.012567603	PASS
FAM133B	7	92560793	92590394	-0.262778904	PASS
FAM135A	6	70412941	70561174	-0.368590549	PASS
FAM135B	8	138130023	138496822	0.113969953	PASS
FAM136A	2	70295975	70302090	-0.372959126	PASS
FAM13A	4	88725955	89111398	-0.011382299	PASS
FAM13B	5	137937960	138051961	-0.293443042	PASS
FAM13C	10	59246129	59363181	0.072405807	PASS
FAM149A	4	186104419	186172667	-0.462744716	PASS
FAM149B	10	73168166	73244504	0.072405807	PASS
FAM151A	1	54609182	54623556	0.033763446	PASS
FAM151B	5	80487969	80542563	-0.293443042	PASS
FAM153A	5	177707981	177783398	-0.150979337	PASS
FAM153B	5	176060689	176116015	-0.150979337	PASS
FAM153C	5	178006405	178055559	-0.150979337	PASS
FAM155A	13	107163510	107866735	-0.390786075	PASS
FAM155B	23	69505241	69532508	-0.012567603	PASS
FAM156A	23	52926402	52995472	-0.012567603	PASS
FAM160A	4	151409216	151663632	-0.011382299	PASS
FAM160A	11	6211335	6234711	-0.405444717	PASS
FAM160B	10	114821744	114899832	0.063874692	PASS

FAM160B2	8	22089159	22104898	-0.345962679	PASS
FAM161A	2	61824854	61854143	-0.372959126	PASS
FAM161B	14	73931501	73950414	0.060212819	PASS
FAM162A	3	122384176	122412334	0.313454204	suppress_segment_too_large
FAM162B	6	116752197	116765723	-0.368590549	PASS
FAM163A	1	179743163	179816198	-0.027073548	PASS
FAM163B	9	133578415	133586197	0.365190713	suppress_segment_too_large
FAM166A	9	137243584	137247770	0.365190713	suppress_segment_too_large
FAM166B	9	35561831	35563899	-0.310444659	PASS
FAM167A	8	11421463	11475908	-0.345962679	PASS
FAM167B	1	32247233	32248856	0.033763446	PASS
FAM168A	11	73400487	73598189	0.062969153	PASS
FAM168B	2	131047876	131093460	-0.475215623	PASS
FAM169A	5	74777574	74866951	-0.293443042	PASS
FAM169B	15	98437162	98547728	-0.697600509	PASS
FAM170A	5	119629559	119635822	-0.293443042	PASS
FAM170B	10	49131154	49134008	-0.404852538	PASS
FAM171A	10	15211643	15371062	0.039811807	PASS
FAM171A2	17	44353215	44363875	0.088516311	PASS
FAM171B	2	186693971	186765965	-0.169541924	PASS
FAM172A	5	93618069	94111699	-0.293443042	PASS
FAM173A	16	720581	722601	0.069936844	PASS
FAM173B	5	10226330	10249897	0.055007813	PASS
FAM174A	5	100535305	100586741	-0.293443042	PASS
FAM174B	15	92617443	92809884	-0.697600509	PASS
FAM177A	14	35044907	35113130	0.060212819	PASS
FAM177B	1	222737207	222750805	-0.256698369	PASS
FAM178B	2	96875882	96986592	0.048238121	PASS
FAM180A	7	135728348	135748846	-0.388946609	PASS
FAM180B	11	47586693	47589194	0.117376172	PASS
FAM181A	14	93918894	93929608	-0.192197564	PASS
FAM181B	11	82729941	82733864	0.062969153	PASS
FAM183A	1	43145153	43156396	0.033763446	PASS
FAM184A	6	118959763	119149387	-0.368590549	PASS
FAM184B	4	17629306	17781512	0.279089364	PASS
FAM185A	7	102748971	102809225	-0.388946609	PASS
FAM186A	12	50326230	50396622	-0.202191512	PASS
FAM186B	12	49582885	49605639	-0.202191512	PASS
FAM187A	17	44899712	44905390	0.088516311	PASS
FAM187B	19	35224800	35228729	0.093487731	suppress_segment_too_large
FAM189A	15	29120254	29570723	-0.396255771	PASS
FAM189A2	9	69324567	69392558	0.365190713	suppress_segment_too_large
FAM189B	1	155247205	155255483	0.103015788	PASS
FAM192A	16	57152466	57186116	-0.371357732	PASS
FAM193A	4	2536631	2732565	0.279089364	PASS
FAM193B	5	177519788	177554541	-0.150979337	PASS

FAM198A	3	42979267	43060211	-0.234617007	PASS
FAM198B	4	158124474	158173318	-0.011382299	PASS
FAM199X	23	104166620	104195902	-0.012567603	PASS
FAM19A1	3	68004216	68545625	-0.234617007	PASS
FAM19A2	12	61708259	62279150	-0.202191512	PASS
FAM19A3	1	112720419	112727235	0.033763446	PASS
FAM19A4	3	68731766	68953297	-0.234617007	PASS
FAM19A5	22	48489460	48850912	-0.808211634	PASS
FAM200A	7	99546308	99558536	-0.262778904	PASS
FAM200B	4	15681662	15705565	0.279089364	PASS
FAM204A	10	118297930	118342328	0.063874692	PASS
FAM205A	9	34723055	34729467	-0.310444659	PASS
FAM205C	9	34889064	34895778	-0.310444659	PASS
FAM206A	9	108934181	108950744	0.365190713	suppress_segment_too_large
FAM207A	21	44940010	44976989	-0.064425456	suppress_segment_too_large
FAM208A	3	56620133	56683237	-0.234617007	PASS
FAM208B	10	5684838	5763740	0.039811807	PASS
FAM209A	20	56517187	56526142	-0.168668583	PASS
FAM209B	20	56533246	56536520	-0.168668583	PASS
FAM20A	17	68535113	68601389	0.088516311	PASS
FAM20B	1	179025804	179076562	-0.027073548	PASS
FAM20C	7	192969	260745	-0.69513118	PASS
FAM210A	18	13663347	13726663	-0.052305056	suppress_segment_too_large
FAM210B	20	56358915	56368663	-0.168668583	PASS
FAM214A	15	52581317	52709817	-0.396255771	PASS
FAM214B	9	35104112	35116341	-0.310444659	PASS
FAM216A	12	110468364	110490385	0.202141228	PASS
FAM216B	13	42781550	42791549	-0.390786075	PASS
FAM217A	6	4049434	4087344	0.048820648	PASS
FAM217B	20	59933764	59948680	-0.168668583	PASS
FAM218A	4	164956948	164959122	-0.011382299	PASS
FAM219A	9	34398184	34458570	-0.310444659	PASS
FAM219B	15	74899987	74907121	-0.697600509	PASS
FAM220A	7	6329409	6348981	-0.69513118	PASS
FAM221A	7	23680130	23703249	-0.69513118	PASS
FAM221B	9	35816391	35828747	-0.310444659	PASS
FAM222A	12	109714228	109770507	0.202141228	PASS
FAM222B	17	28755978	28855232	0.060555011	PASS
FAM227A	22	38578120	38656629	-0.575930959	PASS
FAM227B	15	49326962	49620931	-0.396255771	PASS
FAM228A	2	24175069	24200849	0.040498426	PASS
FAM228B	2	24076526	24169640	0.040498426	PASS
FAM229A	1	32361270	32364278	0.033763446	PASS
FAM229B	6	112087599	112102790	-0.368590549	PASS
FAM234A	16	234546	268971	0.069936844	PASS
FAM234B	12	13044284	13142521	0.362961408	suppress_segment_too_large

FAM236C	23	72912615	72913401	-0.012567603	PASS
FAM236D	23	72807425	72808210	-0.012567603	PASS
FAM237A	2	206642412	206649011	0.025928102	PASS
FAM240A	3	46612435	46626543	-0.234617007	PASS
FAM241A	4	112145397	112195256	-0.011382299	PASS
FAM241B	10	69630251	69633599	0.072405807	PASS
FAM24A	10	122910701	122913111	0.063874692	PASS
FAM24B	10	122849078	122879641	0.063874692	PASS
FAM25A	10	87020306	87024730	-0.292453993	PASS
FAM25C	10	47995355	47999791	-0.404852538	PASS
FAM32A	19	16185380	16192046	0.093487731	suppress_segment_too_large
FAM3A	23	154506159	154516242	-0.012567603	PASS
FAM3B	21	41304212	41357431	-0.064425456	suppress_segment_too_large
FAM3C	7	121348851	121396364	-0.388946609	PASS
FAM3D	3	58633946	58666848	-0.234617007	PASS
FAM43A	3	194686544	194689037	0.326834516	PASS
FAM43B	1	20552439	20555010	0.033763446	PASS
FAM45A	10	119104086	119137984	0.063874692	PASS
FAM47A	23	34129756	34132314	-0.012567603	PASS
FAM47B	23	34942796	34944915	-0.012567603	PASS
FAM47C	23	37008397	37011666	-0.012567603	PASS
FAM47E	4	76214040	76283780	-0.011382299	PASS
FAM47E-S	4	76251721	76311129	-0.011382299	PASS
FAM49A	2	16549459	16666331	0.040498426	PASS
FAM49B	8	129839593	130017129	0.113969953	PASS
FAM50A	23	154444126	154450654	-0.012567603	PASS
FAM50B	6	3849373	3851320	0.048820648	PASS
FAM53A	4	1617915	1684302	0.279089364	PASS
FAM53B	10	124619292	124744269	0.063874692	PASS
FAM53C	5	138331935	138349729	-0.293443042	PASS
FAM57A	17	732412	742972	0.128406487	PASS
FAM57B	16	30024427	30052978	0.069936844	PASS
FAM69A	1	92832737	92961522	0.033763446	PASS
FAM69B	9	136712570	136724050	0.365190713	suppress_segment_too_large
FAM69C	18	74434099	74457944	-0.376397843	PASS
FAM71A	1	212624474	212626778	-0.256698369	PASS
FAM71B	5	157161846	157166264	-0.293443042	PASS
FAM71C	12	99647753	99650046	0.202141228	PASS
FAM71D	14	67189393	67228550	0.060212819	PASS
FAM71E1	19	50466785	50476753	-0.016761391	PASS
FAM71E2	19	55354908	55363260	0.121336758	PASS
FAM71F1	7	128709061	128731743	-0.388946609	PASS
FAM71F2	7	128672288	128687872	-0.388946609	PASS
FAM72A	1	206186179	206204414	-0.256698369	PASS
FAM72B	1	121167646	121185539	-0.157385092	PASS
FAM72C	1	143955364	143971965	-0.157385092	PASS

FAM72D	1	145096000	145112696	-0.157385092	PASS
FAM76A	1	27725979	27763122	0.033763446	PASS
FAM76B	11	95768942	95790409	0.062969153	PASS
FAM78A	9	131258076	131276547	0.365190713	suppress_segment_too_large
FAM78B	1	166057426	166166969	-0.027073548	PASS
FAM81A	15	59372693	59523549	-0.697600509	PASS
FAM81B	5	95391344	95450454	-0.293443042	PASS
FAM83A	8	123178960	123210079	0.279445683	PASS
FAM83B	6	54846771	54942022	0.048820648	PASS
FAM83C	20	35285731	35292401	-0.168668583	PASS
FAM83D	20	38926312	38953106	-0.168668583	PASS
FAM83E	19	48600810	48614854	0.14996833	PASS
FAM83F	22	39994949	40043529	-0.575930959	PASS
FAM83G	17	18968789	19004804	0.024610076	PASS
FAM83H	8	143723933	143733801	0.307788765	PASS
FAM84A	2	14632686	14650814	0.040498426	PASS
FAM84B	8	126552442	126558393	0.113969953	PASS
FAM86B1	8	12182096	12194133	-0.345962679	PASS
FAM86B2	8	12425614	12436343	-0.345962679	PASS
FAM86C1	11	71787510	71801236	0.062969153	PASS
FAM89A	1	231018958	231040246	-0.256698369	PASS
FAM89B	11	65572349	65574198	0.182439925	suppress_segment_too_large
FAM8A1	6	17600355	17611719	0.048820648	PASS
FAM90A1	12	8221260	8227618	-0.086128028	PASS
FAM91A1	8	123768456	123815452	0.279445683	PASS
FAM92A	8	93698561	93731527	0.279445683	PASS
FAM92B	16	85098358	85112508	-0.371357732	PASS
FAM95C	9	38540569	38577207	-0.310444659	PASS
FAM98A	2	33583658	33599382	0.040498426	PASS
FAM98B	15	38454127	38487710	-0.396255771	PASS
FAM98C	19	38403135	38409088	0.093487731	suppress_segment_too_large
FAM9A	23	8790795	8801383	-0.012567603	PASS
FAM9B	23	9024232	9164639	-0.012567603	PASS
FAM9C	23	13035618	13044682	-0.012567603	PASS
FAN1	15	30903852	30943108	-0.396255771	PASS
FANCA	16	89737549	89816657	-0.371357732	PASS
FANCB	23	14835961	14873069	-0.012567603	PASS
FANCC	9	95099054	95426796	0.365190713	suppress_segment_too_large
FANCD2	3	10026414	10101930	0.311018244	suppress_segment_too_large
FANCD2O	3	10081317	10108231	0.311018244	suppress_segment_too_large
FANCE	6	35452356	35467103	0.048820648	PASS
FANCF	11	22622890	22625841	-0.405444717	PASS
FANCG	9	35073835	35080016	-0.310444659	PASS
FANCI	15	89243949	89317261	-0.697600509	PASS
FANCL	2	58159243	58241372	-0.372959126	PASS
FANCM	14	45135940	45200890	0.060212819	PASS

FANK1	10	125896539	126009592	0.063874692	PASS
FAP	2	162170684	162245151	0.039726562	PASS
FAR1	11	13668670	13732346	-0.405444717	PASS
FAR2	12	29149103	29340980	0.362961408	suppress_segment_too_large
FARP1	13	98142562	98455176	-0.390786075	PASS
FARS2	6	5261044	5829192	0.048820648	PASS
FARSA	19	12922479	12934037	0.093487731	suppress_segment_too_large
FARSB	2	222570536	222656337	0.025928102	PASS
FAS	10	88990582	89015785	-1.64589449	RESCUE
FASLG	1	172659018	172666874	-0.027073548	PASS
FASN	17	82078333	82098332	0.088516311	PASS
FASTK	7	151076593	151080866	-0.388946609	PASS
FASTKD1	2	169529749	169573875	0.039726562	PASS
FASTKD2	2	206765357	206792509	0.025928102	PASS
FASTKD3	5	7859159	7869037	0.055007813	PASS
FASTKD5	20	3146519	3159897	-0.168668583	PASS
FAT1	4	186587783	186726722	-0.462744716	PASS
FAT2	5	151504093	151568944	-0.293443042	PASS
FAT3	11	92352096	92896470	0.062969153	PASS
FAT4	4	125316399	125492932	-0.011382299	PASS
FATE1	23	151716035	151723194	-0.012567603	PASS
FAU	11	65120628	65122473	0.182439925	suppress_segment_too_large
FAXC	6	99271169	99350062	-0.368590549	PASS
FAXDC2	5	154818491	154859252	-0.293443042	PASS
FBF1	17	75909574	75941140	0.088516311	PASS
FBH1	10	5890203	5937594	0.039811807	PASS
FBL	19	39834458	39846414	0.093487731	suppress_segment_too_large
FBLIM1	1	15756607	15786594	0.033763446	PASS
FBLL1	5	168529116	168530634	-0.150979337	PASS
FBLN1	22	45502238	45601135	-0.808211634	PASS
FBLN2	3	13549131	13638422	0.311018244	suppress_segment_too_large
FBLN5	14	91869412	91947987	-0.192197564	PASS
FBLN7	2	112138385	112188216	-0.010901494	PASS
FBN1	15	48408306	48645849	-0.396255771	PASS
FBN2	5	128257909	128659185	-0.293443042	PASS
FBN3	19	8065402	8149846	0.079632875	PASS
FBP1	9	94603133	94640249	0.365190713	suppress_segment_too_large
FBP2	9	94558720	94593793	0.365190713	suppress_segment_too_large
FBRS	16	30658431	30670814	0.069936844	PASS
FBRSL1	12	132489551	132585188	0.270848837	PASS
FBXL12	19	9810267	9827816	0.093487731	suppress_segment_too_large
FBXL13	7	102813230	103074843	-0.388946609	PASS
FBXL14	12	1565993	1594165	-0.086128028	PASS
FBXL15	10	102419189	102423136	-0.367947924	PASS
FBXL16	16	692498	705829	0.069936844	PASS
FBXL17	5	107859035	108382098	-0.293443042	PASS

FBXL18	7	5431335	5513798	-0.69513118	PASS
FBXL19	16	30923055	30948783	0.069936844	PASS
FBXL2	3	33277025	33403662	0.311018244	suppress_segment_too_large
FBXL20	17	39252644	39402523	0.060555011	PASS
FBXL22	15	63597353	63602428	-0.697600509	PASS
FBXL3	13	76992598	77027195	-0.390786075	PASS
FBXL4	6	98868538	98948006	-0.368590549	PASS
FBXL5	4	15604539	15681679	0.279089364	PASS
FBXL6	8	144355431	144359376	0.307788765	PASS
FBXL7	5	15500196	15939795	0.055007813	PASS
FBXL8	16	67159931	67164570	-0.371357732	PASS
FBXO10	9	37510892	37576349	-0.310444659	PASS
FBXO11	2	47789316	47905793	0.040498426	PASS
FBXO15	18	74073353	74147865	-0.376397843	PASS
FBXO16	8	28348287	28490318	-0.345962679	PASS
FBXO17	19	38941401	38975910	0.093487731	suppress_segment_too_large
FBXO2	1	11637018	11655785	0.373964873	suppress_segment_too_large
FBXO21	12	117141988	117190531	0.202141228	PASS
FBXO22	15	75903859	75942510	-0.697600509	PASS
FBXO24	7	100583982	100601117	-0.262778904	PASS
FBXO25	8	406428	477967	-0.313874214	PASS
FBXO27	19	38990714	39032785	0.093487731	suppress_segment_too_large
FBXO28	1	224114087	224162047	-0.256698369	PASS
FBXO3	11	33740939	33774543	-0.405444717	PASS
FBXO30	6	145793502	145814753	-0.035448111	PASS
FBXO31	16	87326987	87392142	-0.371357732	PASS
FBXO32	8	123497889	123541206	0.279445683	PASS
FBXO33	14	39397669	39432500	0.060212819	PASS
FBXO34	14	55271303	55361918	0.060212819	PASS
FBXO36	2	229922302	230013109	0.025928102	PASS
FBXO38	5	148383935	148442836	-0.293443042	PASS
FBXO39	17	6776223	6797101	0.128406487	PASS
FBXO4	5	41925254	41941743	-0.293443042	PASS
FBXO40	3	121593119	121630295	0.313454204	suppress_segment_too_large
FBXO41	2	73254682	73284431	-0.372959126	PASS
FBXO42	1	16246839	16352454	0.033763446	PASS
FBXO43	8	100133360	100145800	0.279445683	PASS
FBXO44	1	11654375	11663327	0.373964873	suppress_segment_too_large
FBXO45	3	196568611	196589059	0.326834516	PASS
FBXO46	19	45710629	45730904	0.14996833	PASS
FBXO47	17	38936432	38967402	0.060555011	PASS
FBXO48	2	68459419	68467258	-0.372959126	PASS
FBXO5	6	152970519	152983579	-0.035448111	PASS
FBXO6	1	11664124	11674354	0.373964873	suppress_segment_too_large
FBXO7	22	32474676	32498829	-0.575930959	PASS
FBXO8	4	174236658	174284264	-0.011382299	PASS

FBXO9	6	53051991	53100873	0.048820648	PASS
FBXW10	17	18744024	18779349	0.024610076	PASS
FBXW11	5	171861549	172006873	-0.150979337	PASS
FBXW12	3	48372219	48401259	-0.234617007	PASS
FBXW2	9	120751978	120793412	0.365190713	suppress_segment_too_large
FBXW4	10	101610664	101695295	-0.367947924	PASS
FBXW5	9	136940435	136944696	0.365190713	suppress_segment_too_large
FBXW7	4	152320544	152536063	-0.011382299	PASS
FBXW8	12	116910956	117031148	0.202141228	PASS
FBXW9	19	12688053	12696643	0.093487731	suppress_segment_too_large
FCAMR	1	206957965	206970625	-0.256698369	PASS
FCAR	19	54874248	54890472	-0.146906334	PASS
FCER1A	1	159289714	159308224	-0.467855874	PASS
FCER1G	1	161215234	161220699	-0.027073548	PASS
FCER2	19	7688758	7702146	0.079632875	PASS
FCF1	14	74713144	74738620	-0.192197564	PASS
FCGBP	19	39863323	39934626	0.093487731	suppress_segment_too_large
FCGR1A	1	149782671	149792518	-0.157385092	PASS
FCGR1B	1	121087345	121096310	-0.157385092	PASS
FCGR2A	1	161505430	161524013	-0.027073548	PASS
FCGR2B	1	161663147	161678654	-0.027073548	PASS
FCGR3A	1	161541759	161550737	-0.027073548	PASS
FCGR3B	1	161623196	161631963	-0.027073548	PASS
FCGRT	19	49506816	49526333	0.14996833	PASS
FCHO1	19	17747718	17788568	0.093487731	suppress_segment_too_large
FCHO2	5	72955981	73090522	-0.293443042	PASS
FCHSD1	5	141639302	141651419	-0.293443042	PASS
FCHSD2	11	72836745	73142261	0.062969153	PASS
FCMR	1	206904386	206923247	-0.256698369	PASS
FCN1	9	134905890	134917963	0.365190713	suppress_segment_too_large
FCN2	9	134880812	134887520	0.365190713	suppress_segment_too_large
FCN3	1	27369112	27374824	0.033763446	PASS
FCRL1	1	157794403	157820105	-0.467855874	PASS
FCRL2	1	157745733	157777132	-0.467855874	PASS
FCRL3	1	157674321	157700857	-0.467855874	PASS
FCRL4	1	157573749	157598080	-0.467855874	PASS
FCRL5	1	157513377	157552520	0.103015788	PASS
FCRL6	1	159800511	159816251	-0.467855874	PASS
FCRLA	1	161706972	161714352	-0.027073548	PASS
FCRLB	1	161721563	161728143	-0.027073548	PASS
FDCSP	4	70226071	70235252	-0.011382299	PASS
FDFT1	8	11795573	11839309	-0.345962679	PASS
FDPS	1	155308748	155320666	0.103015788	PASS
FDX1	11	110429883	110464881	-0.356188624	PASS
FDX2	19	10310045	10316015	0.093487731	suppress_segment_too_large
FDXACB1	11	111874056	111881243	-0.356188624	PASS

FDXR	17	74862497	74873031	0.088516311	PASS
FECH	18	57548283	57586772	-0.376397843	PASS
FEM1A	19	4791681	4801273	0.079632875	PASS
FEM1B	15	68277803	68295865	-0.697600509	PASS
FEM1C	5	115520908	115544894	-0.293443042	PASS
FEN1	11	61792637	61797244	0.182439925	suppress_segment_too_large
FER	5	108747822	109196841	-0.293443042	PASS
FER1L5	2	96642737	96704887	0.048238121	PASS
FER1L6	8	123851987	124120061	0.279445683	PASS
FERD3L	7	19144782	19145421	-0.69513118	PASS
FERMT1	20	6074845	6123544	-0.168668583	PASS
FERMT2	14	52857268	52952435	0.060212819	PASS
FERMT3	11	64206678	64223886	0.182439925	suppress_segment_too_large
FES	15	90883695	90895776	-0.697600509	PASS
FETUB	3	186635969	186653141	0.318278996	suppress_segment_too_large
FEV	2	218981087	218985657	0.025928102	PASS
FEZ1	11	125442881	125592568	-0.419612865	PASS
FEZ2	2	36531805	36646087	0.040498426	PASS
FEZF1	7	122301394	122310691	-0.388946609	PASS
FEZF2	3	62369681	62374324	-0.234617007	PASS
FFAR1	19	35351552	35353862	0.093487731	suppress_segment_too_large
FFAR2	19	35443907	35451767	0.093487731	suppress_segment_too_large
FFAR3	19	35358460	35360485	0.093487731	suppress_segment_too_large
FFAR4	10	93566665	93604480	-0.39678585	PASS
FGA	4	154583126	154590766	-0.011382299	PASS
FGB	4	154562956	154571086	-0.011382299	PASS
FGD1	23	54445454	54496166	-0.012567603	PASS
FGD2	6	37005646	37029070	0.048820648	PASS
FGD3	9	92947451	93036236	0.365190713	suppress_segment_too_large
FGD4	12	32399529	32646050	0.362961408	suppress_segment_too_large
FGD5	3	14810853	14934565	0.311018244	suppress_segment_too_large
FGD6	12	95076749	95217482	0.202141228	PASS
FGF1	5	142592178	142698070	-0.293443042	PASS
FGF10	5	44303544	44389706	-0.293443042	PASS
FGF11	17	7438273	7444937	0.128406487	PASS
FGF12	3	192139395	192767764	-0.627320105	PASS
FGF13	23	138614731	139222777	-0.012567603	PASS
FGF14	13	101710804	102402457	-0.390786075	PASS
FGF16	23	77447405	77457278	-0.012567603	PASS
FGF17	8	22042398	22048809	-0.345962679	PASS
FGF18	5	171419656	171457623	-0.150979337	PASS
FGF19	11	69698232	69704642	0.182439925	suppress_segment_too_large
FGF2	4	122826708	122898236	-0.011382299	PASS
FGF20	8	16992169	17002181	-0.345962679	PASS
FGF21	19	48755559	48758333	0.14996833	PASS
FGF22	19	639879	644371	0.079632875	PASS

FGF23	12	4368227	4379728	-0.086128028	PASS
FGF3	11	69809969	69819024	0.182439925	suppress_segment_too_large
FGF4	11	69771016	69775403	0.182439925	suppress_segment_too_large
FGF5	4	80266599	80336680	-0.011382299	PASS
FGF6	12	4428155	4445614	-0.086128028	PASS
FGF7	15	49423096	49488775	-0.396255771	PASS
FGF8	10	101770130	101780369	-0.367947924	PASS
FGF9	13	21671383	21704498	-0.390786075	PASS
FGFBP1	4	15935569	15938740	0.279089364	PASS
FGFBP2	4	15960243	15969309	0.279089364	PASS
FGFBP3	10	91906589	91909483	-0.39678585	PASS
FGFR1	8	38400215	38468834	-0.201142258	PASS
FGFR1OP	6	166999182	167094789	-0.035448111	PASS
FGFR1OP2	12	26938383	26966650	0.362961408	suppress_segment_too_large
FGFR2	10	121478334	121598458	0.063874692	PASS
FGFR3	4	1793293	1808872	0.279089364	PASS
FGFR4	5	177086886	177098144	-0.150979337	PASS
FGFRL1	4	1009936	1026897	0.279089364	PASS
FGG	4	154604134	154612967	-0.011382299	PASS
FGGY	1	59296638	59810647	0.033763446	PASS
FGL1	8	17864380	17910365	-0.345962679	PASS
FGL2	7	77193371	77199848	-0.262778904	PASS
FGR	1	27612064	27635277	0.033763446	PASS
FH	1	241497603	241519761	-0.648598641	PASS
FHAD1	1	15247272	15400283	0.033763446	PASS
FHDC1	4	152936352	152979696	-0.011382299	PASS
FHIT	3	59749310	61251459	-0.234617007	PASS
FHL1	23	136146702	136211359	-0.012567603	PASS
FHL2	2	105357712	105438513	-0.532032081	PASS
FHL3	1	37996770	38005606	0.033763446	PASS
FHL5	6	96562548	96616636	-0.368590549	PASS
FHOD1	16	67229387	67247658	-0.371357732	PASS
FHOD3	18	36297714	36780055	-0.376397843	PASS
FIBCD1	9	130902438	130939286	0.365190713	suppress_segment_too_large
FIBIN	11	26994184	26996121	-0.405444717	PASS
FIBP	11	65883741	65888539	0.182439925	suppress_segment_too_large
FICD	12	108515185	108525837	0.202141228	PASS
FIG4	6	109691312	109825428	-0.368590549	PASS
FIGLA	2	70777310	70790643	-0.372959126	PASS
FIGN	2	163593396	163736012	0.039726562	PASS
FIGNL1	7	50444128	50542535	-0.750061359	PASS
FIGNL2	12	51817840	51848766	-0.202191512	PASS
FILIP1	6	75291859	75493738	-0.368590549	PASS
FILIP1L	3	99830141	100114513	0.313454204	suppress_segment_too_large
FIP1L1	4	53377643	53460861	-0.011382299	PASS
FIS1	7	101239458	101252316	-0.388946609	PASS

FITM1	14	24131275	24132849	0.060212819	PASS
FITM2	20	44302838	44311169	-0.168668583	PASS
FIZ1	19	55591371	55601970	0.121336758	PASS
FJX1	11	35618419	35620868	-0.405444717	PASS
FKBP10	17	41812680	41823217	0.060555011	PASS
FKBP11	12	48921518	48926474	-0.202191512	PASS
FKBP14	7	30010587	30026684	-0.69513118	PASS
FKBP15	9	113161006	113221361	0.365190713	suppress_segment_too_large
FKBP1A	20	1368978	1393172	-0.168668583	PASS
FKBP1B	2	24049701	24063681	0.040498426	PASS
FKBP1C	6	63211446	63213024	0.048820648	PASS
FKBP2	11	64241003	64244132	0.182439925	suppress_segment_too_large
FKBP3	14	45115600	45135319	0.060212819	PASS
FKBP4	12	2794953	2805423	-0.086128028	PASS
FKBP5	6	35573585	35728583	0.048820648	PASS
FKBP6	7	73328164	73358637	-0.262778904	PASS
FKBP7	2	178463664	178478600	0.039726562	PASS
FKBP8	19	18531751	18544077	0.093487731	suppress_segment_too_large
FKBP9	7	32957404	33006931	-0.69513118	PASS
FKBPL	6	32128707	32130291	0.048820648	PASS
FKRP	19	46746046	46776988	0.14996833	PASS
FKTN	9	105558130	105641118	0.365190713	suppress_segment_too_large
FLAD1	1	154983338	154993111	0.103015788	PASS
FLCN	17	17212212	17237188	0.024610076	PASS
FLG	1	152302175	152325203	0.103015788	PASS
FLG2	1	152348735	152360006	0.103015788	PASS
FLI1	11	128686535	128813267	-0.419612865	PASS
FLII	17	18244836	18258916	0.024610076	PASS
FLNA	23	154348524	154374638	-0.012567603	PASS
FLNB	3	58008400	58172251	-0.234617007	PASS
FLNC	7	128830377	128859274	-0.388946609	PASS
FLOT1	6	30727709	30742733	0.048820648	PASS
FLOT2	17	28879335	28897679	0.060555011	PASS
FLRT1	11	64103188	64119173	0.182439925	suppress_segment_too_large
FLRT2	14	85530144	85654426	-0.192197564	PASS
FLRT3	20	14322988	14337616	-0.168668583	PASS
FLT1	13	28300344	28495145	-0.390786075	PASS
FLT3	13	28003274	28100592	-0.390786075	PASS
FLT3LG	19	49474207	49486231	0.14996833	PASS
FLT4	5	180601506	180649624	-0.150979337	PASS
FLVCR1	1	212858255	212899363	-0.256698369	PASS
FLVCR2	14	75578617	75663214	-0.192197564	PASS
FLYWCH1	16	2911937	2951208	0.069936844	PASS
FLYWCH2	16	2883186	2899382	0.069936844	PASS
FMC1	7	139339457	139346319	-0.388946609	PASS
FMN1	15	32765545	33194733	-0.396255771	PASS

FMN2	1	240014348	240475189	-0.648598641	PASS
FMNL1	17	45221444	45247320	0.088516311	PASS
FMNL2	2	152335237	152649834	-0.475215623	PASS
FMNL3	12	49636499	49708165	-0.202191512	PASS
FMO1	1	171248471	171285978	-0.027073548	PASS
FMO2	1	171185208	171211230	-0.027073548	PASS
FMO3	1	171090877	171117819	-0.027073548	PASS
FMO4	1	171314208	171342084	-0.027073548	PASS
FMO5	1	147175351	147243050	-0.157385092	PASS
FMOD	1	203340628	203351758	-0.256698369	PASS
FMR1	23	147911951	147951125	-0.012567603	PASS
FMR1NB	23	147981329	148026667	-0.012567603	PASS
FN1	2	215360440	215436172	0.025928102	PASS
FN3K	17	82735575	82751197	0.088516311	PASS
FN3KRP	17	82716683	82730328	0.088516311	PASS
FNBP1	9	129887187	130043194	0.365190713	suppress_segment_too_large
FNBP1L	1	93448131	93554661	0.033763446	PASS
FNBP4	11	47716517	47767443	0.117376172	PASS
FNDC1	6	159169397	159272109	-0.035448111	PASS
FNDC10	1	1598012	1600096	0.373964873	suppress_segment_too_large
FNDC11	20	63547891	63556708	-0.168668583	PASS
FNDC3A	13	48975912	49209779	-0.390786075	PASS
FNDC3B	3	172039628	172401665	0.318278996	suppress_segment_too_large
FNDC4	2	27491883	27495245	0.040498426	PASS
FNDC5	1	32862268	32872482	0.033763446	PASS
FNDC7	1	108712657	108742740	0.033763446	PASS
FNDC8	17	35121579	35130732	0.060555011	PASS
FNDC9	5	157341600	157345721	-0.150979337	PASS
FNIP1	5	131641714	131797063	-0.293443042	PASS
FNIP2	4	158769138	158908049	-0.011382299	PASS
FNTA	8	43034194	43085788	-0.201142258	PASS
FNTB	14	64986720	65062652	0.060212819	PASS
FO393400.	20	32632183	32743567	-0.168668583	PASS
FO681492.	10	47750864	47763592	-0.404852538	PASS
FOCAD	9	20658309	20995955	-0.310444659	PASS
FOLH1	11	49146635	49208670	-0.621795272	PASS
FOLR1	11	72189558	72196323	0.062969153	PASS
FOLR2	11	72216601	72221950	0.062969153	PASS
FOLR3	11	72114869	72139892	0.062969153	PASS
FOPNL	16	15865720	15888625	0.069936844	PASS
FOS	14	75278774	75282230	-0.192197564	PASS
FOSB	19	45467995	45475179	0.14996833	PASS
FOSL1	11	65892049	65900573	0.182439925	suppress_segment_too_large
FOSL2	2	28392448	28417312	0.040498426	PASS
FOXA1	14	37589984	37596059	0.060212819	PASS
FOXA2	20	22581005	22585455	-0.168668583	PASS

FOXA3	19	45863989	45873797	0.14996833	PASS
FOXB1	15	60004222	60061730	-0.697600509	PASS
FOXB2	9	77019655	77020953	0.365190713	suppress_segment_too_large
FOXC1	6	1609972	1613897	0.048820648	PASS
FOXC2	16	86566829	86569728	-0.371357732	PASS
FOXD1	5	73444827	73448527	-0.293443042	PASS
FOXD2	1	47436017	47440691	0.033763446	PASS
FOXD3	1	63323041	63325126	0.033763446	PASS
FOXD4	9	116231	118204	-0.310444659	PASS
FOXD4L1	2	113498665	113501155	-0.010901494	PASS
FOXD4L3	9	68302867	68304905	-0.310444659	PASS
FOXD4L4	9	65736555	65738784	-0.310444659	PASS
FOXD4L5	9	65282101	65285209	-0.310444659	PASS
FOXD4L6	9	41126430	41128463	-0.310444659	PASS
FOXE1	9	97853254	97856715	0.365190713	suppress_segment_too_large
FOXE3	1	47416072	47418052	0.033763446	PASS
FOXF1	16	86510527	86515418	-0.371357732	PASS
FOXF2	6	1389576	1395603	0.048820648	PASS
FOXG1	14	28760330	28770277	0.060212819	PASS
FOXH1	8	144473412	144476335	0.307788765	PASS
FOXI1	5	170105897	170109725	-0.150979337	PASS
FOXI2	10	127737235	127741186	0.063874692	PASS
FOXI3	2	88446787	88452656	-0.340781246	PASS
FOXJ1	17	76136333	76141299	0.088516311	PASS
FOXJ2	12	8032703	8055503	-0.086128028	PASS
FOXJ3	1	42176539	42335877	0.033763446	PASS
FOXK1	7	4682309	4771443	-0.69513118	PASS
FOXK2	17	82519713	82644662	0.088516311	PASS
FOXL1	16	86576368	86582160	-0.371357732	PASS
FOXL2	3	138944224	138947140	0.313454204	suppress_segment_too_large
FOXL2NB	3	138947234	138953451	0.313454204	suppress_segment_too_large
FOXM1	12	2857681	2877155	-0.086128028	PASS
FOXN1	17	28506243	28538896	0.060555011	PASS
FOXN2	2	48314637	48379294	0.040498426	PASS
FOXN3	14	89124871	89619149	-0.192197564	PASS
FOXN4	12	109277979	109309220	0.202141228	PASS
FOXO1	13	40469953	40666597	-0.390786075	PASS
FOXO3	6	108559835	108684774	-0.368590549	PASS
FOXO4	23	71096197	71103535	-0.012567603	PASS
FOXO6	1	41361922	41383590	0.033763446	PASS
FOXP1	3	70952817	71583993	-0.234617007	PASS
FOXP2	7	114086327	114693772	-0.388946609	PASS
FOXP3	23	49250436	49264826	-0.012567603	PASS
FOXP4	6	41546426	41602384	0.048820648	PASS
FOXQ1	6	1312440	1314748	0.048820648	PASS
FOXR1	11	118971707	118981291	-0.356188624	PASS

FOXR2	23	55623400	55625325	-0.012567603	PASS
FOXRED1	11	126269055	126278131	-0.419612865	PASS
FOXRED2	22	36487190	36507101	-0.575930959	PASS
FOXS1	20	31844301	31845619	-0.168668583	PASS
FP565260.1	21	5130871	5154734	-0.304975024	PASS
FP565260.2	21	5116343	5133805	-0.304975024	PASS
FP565260.3	21	5022493	5040666	-0.304975024	PASS
FPGS	9	127794597	127814327	0.365190713	suppress_segment_too_large
FPGT	1	74198212	74234086	0.033763446	PASS
FPGT-TNN	1	74198235	74544393	0.033763446	PASS
FPR1	19	51745172	51804110	-0.016761391	PASS
FPR2	19	51752026	51770526	-0.016761391	PASS
FPR3	19	51795163	51826189	-0.016761391	PASS
FRA10AC1	10	93667883	93702572	-0.39678585	PASS
FRAS1	4	78057570	78544269	-0.011382299	PASS
FRAT1	10	97319267	97321915	-0.367947924	PASS
FRAT2	10	97332497	97334709	-0.367947924	PASS
FREM1	9	14734666	14910995	-0.310444659	PASS
FREM2	13	38687129	38887131	-0.390786075	PASS
FREM3	4	143577302	143700675	-0.011382299	PASS
FRG1	4	189940788	189963204	-0.462744716	PASS
FRG2	4	190024351	190027257	-0.462744716	PASS
FRG2B	10	133623895	133626795	0.063874692	PASS
FRG2C	3	75664330	75667220	-0.234617007	PASS
FRK	6	115931149	116060758	-0.368590549	PASS
FRMD1	6	168053166	168101511	-0.035448111	PASS
FRMD3	9	83242990	83538546	0.365190713	suppress_segment_too_large
FRMD4A	10	13643706	14462142	0.039811807	PASS
FRMD4B	3	69169990	69542583	-0.234617007	PASS
FRMD5	15	43870761	44195252	-0.396255771	PASS
FRMD6	14	51489100	51730727	0.060212819	PASS
FRMD7	23	132076993	132128020	-0.012567603	PASS
FRMD8	11	65386599	65413525	0.182439925	suppress_segment_too_large
FRMPD1	9	37651000	37746904	-0.310444659	PASS
FRMPD2	10	48153088	48274870	-0.404852538	PASS
FRMPD3	23	107522450	107605251	-0.012567603	PASS
FRMPD4	23	12138466	12724523	-0.012567603	PASS
FRRS1	1	99708632	99766631	0.033763446	PASS
FRRS1L	9	109130293	109167295	0.365190713	suppress_segment_too_large
FRS2	12	69470349	69579789	-0.202191512	PASS
FRS3	6	41770176	41786542	0.048820648	PASS
FRY	13	31846713	32299122	-0.390786075	PASS
FRYL	4	48497361	48780322	-0.011382299	PASS
FRZB	2	182833275	182867162	-0.169541924	PASS
FSBP	8	94372170	94436952	0.279445683	PASS
FSCB	14	44504342	44507279	0.060212819	PASS

FSCN1	7	5592823	5606655	-0.69513118	PASS
FSCN2	17	81528396	81537130	0.088516311	PASS
FSCN3	7	127591409	127602144	-0.388946609	PASS
FSD1	19	4304600	4323843	0.079632875	PASS
FSD1L	9	105447796	105552433	0.365190713	suppress_segment_too_large
FSD2	15	82755362	82806070	-0.697600509	PASS
FSHB	11	30231016	30235261	-0.405444717	PASS
FSHR	2	48962157	49154537	0.040498426	PASS
FSIP1	15	39588357	39782830	-0.396255771	PASS
FSIP2	2	185738895	185833290	-0.169541924	PASS
FST	5	53480409	53487134	-0.293443042	PASS
FSTL1	3	120392293	120451253	0.313454204	suppress_segment_too_large
FSTL3	19	676365	683399	0.079632875	PASS
FSTL4	5	133196455	133612564	-0.293443042	PASS
FSTL5	4	161383897	162164035	-0.011382299	PASS
FTCD	21	46136262	46155567	-0.064425456	suppress_segment_too_large
FTCDNL1	2	199760544	199851173	0.025928102	PASS
FTH1	11	61959718	61967660	0.182439925	suppress_segment_too_large
FTHL17	23	31071241	31072053	-0.012567603	PASS
FTL	19	48965301	48966878	0.14996833	PASS
FTMT	5	121851955	121852833	-0.293443042	PASS
FTO	16	53701692	54158512	-0.371357732	PASS
FTSJ1	23	48476021	48486364	-0.012567603	PASS
FTSJ3	17	63819433	63830012	0.088516311	PASS
FUBP1	1	77944055	77979110	0.033763446	PASS
FUBP3	9	130578965	130638352	0.365190713	suppress_segment_too_large
FUCA1	1	23845077	23868294	0.033763446	PASS
FUCA2	6	143494811	143511690	-0.035448111	PASS
FUK	16	70454421	70480274	-0.371357732	PASS
FUNDC1	23	44523639	44543001	-0.012567603	PASS
FUNDC2	23	155025980	155060303	-0.012567603	PASS
FUOM	10	133355154	133358035	0.063874692	PASS
FURIN	15	90868592	90883458	-0.697600509	PASS
FUS	16	31180110	31194871	0.069936844	PASS
FUT1	19	48748011	48755390	0.14996833	PASS
FUT10	8	33370824	33473422	-0.345962679	PASS
FUT11	10	73772291	73780251	0.072405807	PASS
FUT2	19	48695971	48705950	0.14996833	PASS
FUT3	19	5842888	5851474	0.079632875	PASS
FUT4	11	94543840	94549898	0.062969153	PASS
FUT5	19	5865826	5870540	0.079632875	PASS
FUT6	19	5830610	5839731	0.079632875	PASS
FUT7	9	137030174	137033010	0.365190713	suppress_segment_too_large
FUT8	14	65410592	65744121	0.060212819	PASS
FUT9	6	96015984	96215612	-0.368590549	PASS
FUZ	19	49806869	49817376	0.14996833	PASS

FXN	9	69035259	69120427	-0.310444659	PASS
FXR1	3	180868141	180982753	0.318278996	suppress_segment_too_large
FXR2	17	7591230	7614871	0.128406487	PASS
FXYD1	19	35138808	35143109	0.093487731	suppress_segment_too_large
FXYD2	11	117800844	117828698	-0.356188624	PASS
FXYD3	19	35115879	35124324	0.093487731	suppress_segment_too_large
FXYD4	10	43371642	43376335	-0.404852538	PASS
FXYD5	19	35154730	35169883	0.093487731	suppress_segment_too_large
FXYD6	11	117836976	117877486	-0.356188624	PASS
FXYD6-FX	11	117820163	117876667	-0.356188624	PASS
FXYD7	19	35143250	35154301	0.093487731	suppress_segment_too_large
FYB1	5	39105255	39274528	0.055007813	PASS
FYB2	1	56718804	56819696	0.033763446	PASS
FYCO1	3	45917899	45995824	-0.234617007	PASS
FYN	6	111660332	111873452	-0.368590549	PASS
FYTTD1	3	197737179	197787596	0.326834516	PASS
FZD1	7	91264364	91271326	-0.262778904	PASS
FZD10	12	130162459	130165740	-0.240609783	PASS
FZD2	17	44557459	44559570	0.088516311	PASS
FZD3	8	28494205	28574268	-0.345962679	PASS
FZD4	11	86945679	86955391	0.062969153	PASS
FZD5	2	207762586	207769563	0.025928102	PASS
FZD6	8	103298433	103332866	0.279445683	PASS
FZD7	2	202034587	202038445	0.025928102	PASS
FZD8	10	35638249	35642278	0.039811807	PASS
FZD9	7	73433783	73436120	-0.262778904	PASS
FZR1	19	3506273	3538330	0.079632875	PASS
G0S2	1	209675420	209676388	-0.256698369	PASS
G2E3	14	30559123	30620063	0.060212819	PASS
G3BP1	5	151771045	151812785	-0.293443042	PASS
G3BP2	4	75642782	75724525	-0.011382299	PASS
G6PC	17	42900797	42913369	0.088516311	PASS
G6PC2	2	168901240	168910000	0.039726562	PASS
G6PC3	17	44070735	44076344	0.088516311	PASS
G6PD	23	154531390	154547572	-0.012567603	PASS
GAA	17	80101556	80119879	0.088516311	PASS
GAB1	4	143336762	143474568	-0.011382299	PASS
GAB2	11	78215297	78418348	0.062969153	PASS
GAB3	23	154675249	154751583	-0.012567603	PASS
GAB4	22	16961936	17008222	-0.575930959	PASS
GABARAI	17	7240014	7242770	0.128406487	PASS
GABARAI	12	10212458	10223130	0.362961408	suppress_segment_too_large
GABARAI	16	75566351	75577881	-0.371357732	PASS
GABBR1	6	29555629	29633976	0.048820648	PASS
GABBR2	9	98288082	98709197	0.365190713	suppress_segment_too_large
GABPA	21	25734570	25772460	0.12860881	suppress_segment_too_large

GABPB1	15	50275392	50355408	-0.396255771	PASS
GABPB2	1	151070578	151125542	0.103015788	PASS
GABRA1	5	161847063	161899981	-0.150979337	PASS
GABRA2	4	46248427	46475230	-0.011382299	PASS
GABRA3	23	152166234	152451358	-0.012567603	PASS
GABRA4	4	46918900	46994407	-0.011382299	PASS
GABRA5	15	26866363	26949207	-0.396255771	PASS
GABRA6	5	161547063	161702593	-0.150979337	PASS
GABRB1	4	46993723	47426444	-0.011382299	PASS
GABRB2	5	161288429	161549044	-0.150979337	PASS
GABRB3	15	26543546	26939539	-0.396255771	PASS
GABRD	1	2019298	2030758	0.373964873	suppress_segment_too_large
GABRE	23	151953124	151974680	-0.012567603	PASS
GABRG1	4	46035769	46124081	-0.011382299	PASS
GABRG2	5	162000057	162162977	-0.150979337	PASS
GABRG3	15	26971282	27541991	-0.396255771	PASS
GABRP	5	170763350	170814047	-0.150979337	PASS
GABRQ	23	152638183	152657534	-0.012567603	PASS
GABRR1	6	89177501	89231278	-0.368590549	PASS
GABRR2	6	89257208	89315299	-0.368590549	PASS
GABRR3	3	97986673	98035304	0.313454204	suppress_segment_too_large
GAD1	2	170813213	170861151	0.039726562	PASS
GAD2	10	26216307	26304558	0.039811807	PASS
GADD45A	1	67685061	67688338	0.033763446	PASS
GADD45B	19	2476122	2478259	0.079632875	PASS
GADD45G	9	89605013	89606555	0.365190713	suppress_segment_too_large
GADD45G	19	12953119	12957236	0.093487731	suppress_segment_too_large
GADL1	3	30726200	30894765	0.311018244	suppress_segment_too_large
GAGE1	23	49589496	49608536	-0.012567603	PASS
GAGE10	23	49303669	49319844	-0.012567603	PASS
GAGE12J	23	49322057	49329384	-0.012567603	PASS
GAGE13	23	49331616	49338952	-0.012567603	PASS
GAGE2A	23	49589529	49596827	-0.012567603	PASS
GAGE2E	23	49341192	49345922	-0.012567603	PASS
GAK	4	849276	932373	0.279089364	PASS
GAL	11	68683779	68691175	0.182439925	suppress_segment_too_large
GAL3ST1	22	30554635	30574587	-0.575930959	PASS
GAL3ST3	11	66041952	66049180	0.182439925	suppress_segment_too_large
GAL3ST4	7	100159244	100168750	-0.262778904	PASS
GALC	14	87837820	87993665	-0.192197564	PASS
GALE	1	23795599	23800804	0.033763446	PASS
GALK1	17	75751594	75765711	0.088516311	PASS
GALK2	15	49155656	49367869	-0.396255771	PASS
GALM	2	38665910	38741237	0.040498426	PASS
GALNS	16	88813734	88856970	-0.371357732	PASS
GALNT1	18	35581117	35711834	-0.376397843	PASS

GALNT10	5	154190730	154420984	-0.293443042	PASS
GALNT11	7	152025674	152122347	-0.388946609	PASS
GALNT12	9	98807699	98850081	0.365190713	suppress_segment_too_large
GALNT13	2	153871913	154453849	-0.475215623	PASS
GALNT14	2	30910467	31155202	0.040498426	PASS
GALNT15	3	16174649	16231992	0.311018244	suppress_segment_too_large
GALNT16	14	69259277	69357033	0.060212819	PASS
GALNT17	7	71132169	71713600	-0.750061359	PASS
GALNT18	11	11270876	11622005	-0.405444717	PASS
GALNT2	1	230057990	230282124	-0.256698369	PASS
GALNT3	2	165747591	165794682	0.039726562	PASS
GALNT4	12	89519408	89524806	0.202141228	PASS
GALNT5	2	157257598	157314211	-0.475215623	PASS
GALNT6	12	51351247	51392867	-0.202191512	PASS
GALNT7	4	173168753	173323967	-0.011382299	PASS
GALNT8	12	4720341	4851927	-0.086128028	PASS
GALNT9	12	132196372	132329349	0.270848837	PASS
GALNTL5	7	151956379	152019934	-0.388946609	PASS
GALNTL6	4	171812254	173041559	-0.011382299	PASS
GALP	19	56176020	56185775	-0.180829161	PASS
GALR1	18	77250549	77277896	-0.376397843	PASS
GALR2	17	76074794	76077541	0.088516311	PASS
GALR3	22	37823382	37825495	-0.575930959	PASS
GALT	9	34638133	34651035	-0.310444659	PASS
GAMT	19	1397026	1401570	0.079632875	PASS
GAN	16	81314947	81390884	-0.371357732	PASS
GANAB	11	62624826	62646726	0.182439925	suppress_segment_too_large
GANC	15	42273233	42356935	-0.396255771	PASS
GAP43	3	115623324	115721490	0.313454204	suppress_segment_too_large
GAPDH	12	6533927	6538374	-0.086128028	PASS
GAPDHS	19	35533412	35545316	0.093487731	suppress_segment_too_large
GAPT	5	58491435	58497090	-0.293443042	PASS
GAPVD1	9	125261794	125367207	0.365190713	suppress_segment_too_large
GAR1	4	109815510	109824740	-0.011382299	PASS
GAREM1	18	32124877	32470484	-0.376397843	PASS
GAREM2	2	26173091	26189663	0.040498426	PASS
GARNL3	9	127224265	127393660	0.365190713	suppress_segment_too_large
GARS	7	30594681	30634033	-0.69513118	PASS
GART	21	33503931	33543491	0.12860881	suppress_segment_too_large
GAS1	9	86944363	86947189	0.365190713	suppress_segment_too_large
GAS2	11	22625509	22813055	-0.405444717	PASS
GAS2L1	22	29306582	29312785	-0.575930959	PASS
GAS2L2	17	35744511	35752878	0.060555011	PASS
GAS2L3	12	100573683	100628286	0.202141228	PASS
GAS6	13	113820549	113864067	-0.390786075	PASS
GAS7	17	9910609	10198551	-0.139031004	PASS

GAS8	16	90019629	90044975	-0.371357732	PASS
GAST	17	41712326	41715969	0.060555011	PASS
GATA1	23	48786554	48794311	-0.012567603	PASS
GATA2	3	128479427	128493185	0.313454204	suppress_segment_too_large
GATA3	10	8045378	8075203	0.039811807	PASS
GATA4	8	11676959	11760002	-0.345962679	PASS
GATA5	20	62463497	62475970	-0.168668583	PASS
GATA6	18	22169443	22202528	-0.376397843	PASS
GATAD1	7	92447448	92459432	-0.262778904	PASS
GATAD2A	19	19385826	19508931	0.093487731	suppress_segment_too_large
GATAD2B	1	153789030	153923360	0.103015788	PASS
GATB	4	151670504	151761023	-0.011382299	PASS
GATC	12	120446438	120463749	0.202141228	PASS
GATD1	11	767220	777488	-0.333835828	PASS
GATD3B	21	5079294	5128425	-0.304975024	PASS
GATM	15	45361124	45402327	-0.396255771	PASS
GBA	1	155234452	155244699	0.103015788	PASS
GBA2	9	35736866	35749228	-0.310444659	PASS
GBE1	3	81489699	81762161	0.313454204	suppress_segment_too_large
GBF1	10	102245532	102382899	-0.367947924	PASS
GBGT1	9	133152948	133163945	0.365190713	suppress_segment_too_large
GBP1	1	89052319	89065360	0.033763446	PASS
GBP2	1	89106132	89150456	0.033763446	PASS
GBP3	1	89006666	89022894	0.033763446	PASS
GBP4	1	89181148	89198932	0.033763446	PASS
GBP5	1	89258950	89272804	0.033763446	PASS
GBP6	1	89364058	89386461	0.033763446	PASS
GBP7	1	89131751	89176040	0.033763446	PASS
GBX1	7	151148589	151174745	-0.388946609	PASS
GBX2	2	236165236	236168369	0.025928102	PASS
GC	4	71741693	71804041	-0.011382299	PASS
GCA	2	162318840	162371595	0.039726562	PASS
GCAT	22	37807905	37817176	-0.575930959	PASS
GCC1	7	127580618	127593611	-0.388946609	PASS
GCC2	2	108448561	108509415	-0.010901494	PASS
GCDH	19	12891026	12914207	0.093487731	suppress_segment_too_large
GCFC2	2	75652000	75710989	-0.372959126	PASS
GCG	2	162142873	162152404	0.039726562	PASS
GCGR	17	81804132	81814013	0.088516311	PASS
GCH1	14	54842008	54902852	0.060212819	PASS
GCHFR	15	40764020	40767710	-0.396255771	PASS
GCK	7	44144271	44198170	-0.69513118	PASS
GCKR	2	27496842	27523684	0.040498426	PASS
GCLC	6	53497341	53616970	0.048820648	PASS
GCLM	1	93885205	93909456	0.033763446	PASS
GCM1	6	53126964	53148829	0.048820648	PASS

GCM2	6	10873223	10881941	0.048820648	PASS
GCN1	12	120127203	120194710	0.202141228	PASS
GCNA	23	71578411	71613583	-0.012567603	PASS
GCNT1	9	76419850	76651203	0.365190713	suppress_segment_too_large
GCNT2	6	10492223	10629368	0.048820648	PASS
GCNT3	15	59594875	59640239	-0.697600509	PASS
GCNT4	5	75025346	75030899	-0.293443042	PASS
GCNT7	20	56491492	56525925	-0.168668583	PASS
GCOM1	15	57591908	57714745	-0.697600509	PASS
GCSAM	3	112120841	112133305	0.313454204	suppress_segment_too_large
GCSAML	1	247507058	247577690	-0.368111568	PASS
GCSAML-.	1	247524679	247532613	-0.368111568	PASS
GCSH	16	81081938	81096425	-0.371357732	PASS
GDA	9	72114595	72257193	0.365190713	suppress_segment_too_large
GDAP1	8	74321130	74488872	-0.201142258	PASS
GDAP1L1	20	44247099	44280917	-0.168668583	PASS
GDAP2	1	117863485	117929630	0.033763446	PASS
GDE1	16	19501689	19522145	0.069936844	PASS
GDF1	19	18868545	18896096	0.093487731	suppress_segment_too_large
GDF10	10	47300386	47313547	-0.404852538	PASS
GDF11	12	55743280	55757278	-0.202191512	PASS
GDF15	19	18374731	18389176	0.093487731	suppress_segment_too_large
GDF2	10	47322490	47326270	-0.404852538	PASS
GDF3	12	7689782	7695776	-0.086128028	PASS
GDF5	20	35433347	35454746	-0.168668583	PASS
GDF6	8	96142330	96160792	0.279445683	PASS
GDF7	2	20666664	20679245	0.040498426	PASS
GDF9	5	132861181	132866884	-0.293443042	PASS
GDI1	23	154436913	154443467	-0.012567603	PASS
GDI2	10	5765223	5842132	0.039811807	PASS
GDNF	5	37812677	37839686	0.055007813	PASS
GDPD1	17	59220467	59275967	0.088516311	PASS
GDPD2	23	70423031	70433390	-0.012567603	PASS
GDPD3	16	30104810	30113856	0.069936844	PASS
GDPD4	11	77216558	77301687	0.062969153	PASS
GDPD5	11	75434640	75525903	0.062969153	PASS
GDPGP1	15	90233808	90245811	-0.697600509	PASS
GEM	8	94249253	94262350	0.279445683	PASS
GEMIN2	14	39114223	39136973	0.060212819	PASS
GEMIN4	17	744414	753999	0.128406487	PASS
GEMIN5	5	154887416	154938209	-0.293443042	PASS
GEMIN6	2	38751534	38785000	0.040498426	PASS
GEMIN7	19	45079195	45091524	0.14996833	PASS
GEMIN8	23	14008279	14029893	-0.012567603	PASS
GEN1	2	17753858	17788941	0.040498426	PASS
GET4	7	876552	896436	-0.69513118	PASS

GFAP	17	44903161	44916937	0.088516311	PASS
GFER	16	1984207	1987749	0.069936844	PASS
GFI1	1	92474762	92486876	0.033763446	PASS
GFI1B	9	132944000	132991687	0.365190713	suppress_segment_too_large
GFM1	3	158644278	158692575	0.318278996	suppress_segment_too_large
GFM2	5	74721204	74767371	-0.293443042	PASS
GFOD1	6	13357830	13487662	0.048820648	PASS
GFOD2	16	67674531	67719421	-0.371357732	PASS
GFPT1	2	69319769	69387254	-0.372959126	PASS
GFPT2	5	180300690	180353387	-0.150979337	PASS
GFRA1	10	116056925	116273467	0.063874692	PASS
GFRA2	8	21690403	21812357	-0.345962679	PASS
GFRA3	5	138252379	138274671	-0.293443042	PASS
GFRA4	20	3659292	3663399	-0.168668583	PASS
GFRAL	6	55327469	55402493	0.048820648	PASS
GFY	19	49423749	49428818	0.14996833	PASS
GGA1	22	37608475	37633564	-0.575930959	PASS
GGA2	16	23463542	23521995	0.069936844	PASS
GGA3	17	75236599	75262363	0.088516311	PASS
GGACT	13	100530164	100589528	-0.390786075	PASS
GGCT	7	30496621	30504844	-0.69513118	PASS
GGCX	2	85544723	85561547	0.066915641	PASS
GGH	8	63015079	63039171	-0.201142258	PASS
GGN	19	38384265	38388082	0.093487731	suppress_segment_too_large
GGNBP2	17	36544888	36589848	0.060555011	PASS
GGPS1	1	235327350	235344532	-0.256698369	PASS
GGT1	22	24583750	24629005	-0.575930959	PASS
GGT2	22	21207973	21227637	-0.575930959	PASS
GGT5	22	24219654	24245142	-0.575930959	PASS
GGT6	17	4556927	4560818	0.128406487	PASS
GGT7	20	34844720	34872860	-0.168668583	PASS
GGTLC1	20	23985050	23988779	-0.168668583	PASS
GGTLC2	22	22644475	22647903	-0.575930959	PASS
GH1	17	63917200	63918838	0.088516311	PASS
GH2	17	63880215	63881935	0.088516311	PASS
GHDC	17	42188799	42194532	0.060555011	PASS
GHITM	10	84139440	84153245	0.072405807	PASS
GHR	5	42423777	42721878	-0.293443042	PASS
GHRH	20	37251082	37261835	-0.168668583	PASS
GHRHR	7	30938669	30993254	-0.69513118	PASS
GHRL	3	10285675	10292947	0.311018244	suppress_segment_too_large
GHSR	3	172445133	172448456	0.318278996	suppress_segment_too_large
GID4	17	18039292	18068404	0.024610076	PASS
GID8	20	62938119	62948475	-0.168668583	PASS
GIF	11	59829268	59845501	-0.129266491	PASS
GIGYF1	7	100679507	100694037	-0.262778904	PASS

GIGYF2	2	232697299	232860575	0.025928102	PASS
GIMAP1	7	150716557	150724284	-0.388946609	PASS
GIMAP1-(	7	150716668	150743646	-0.388946609	PASS
GIMAP2	7	150685697	150693641	-0.388946609	PASS
GIMAP4	7	150567277	150573955	-0.388946609	PASS
GIMAP5	7	150722253	150750033	-0.388946609	PASS
GIMAP6	7	150625375	150632648	-0.388946609	PASS
GIMAP7	7	150514830	150521073	-0.388946609	PASS
GIMAP8	7	150450630	150479392	-0.388946609	PASS
GIMD1	4	106357485	106368825	-0.011382299	PASS
GIN1	5	103086000	103120151	-0.293443042	PASS
GINM1	6	149566294	149591748	-0.035448111	PASS
GINS1	20	25407727	25452628	-0.168668583	PASS
GINS2	16	85676198	85690073	-0.371357732	PASS
GINS3	16	58295080	58406144	-0.371357732	PASS
GINS4	8	41529206	41545046	-0.201142258	PASS
GIP	17	48958554	48968596	0.088516311	PASS
GIPC1	19	14477760	14496149	0.093487731	suppress_segment_too_large
GIPC2	1	77979542	78138449	0.033763446	PASS
GIPC3	19	3585553	3593541	0.079632875	PASS
GIPR	19	45668244	45683724	0.14996833	PASS
GIT1	17	29573469	29594054	0.060555011	PASS
GIT2	12	109929792	109996389	0.202141228	PASS
GJA1	6	121435577	121449727	-0.368590549	PASS
GJA10	6	89894469	89921760	-0.368590549	PASS
GJA3	13	20138255	20161049	-0.390786075	PASS
GJA4	1	34792998	34795747	0.033763446	PASS
GJA5	1	147756199	147773362	-0.157385092	PASS
GJA8	1	147907956	147909257	-0.157385092	PASS
GJA9	1	38874451	38881587	0.033763446	PASS
GJA9-MY(	1	38864501	38881617	0.033763446	PASS
GJB1	23	71215194	71225516	-0.012567603	PASS
GJB2	13	20187463	20193010	-0.390786075	PASS
GJB3	1	34781189	34786369	0.033763446	PASS
GJB4	1	34759741	34763724	0.033763446	PASS
GJB5	1	34755047	34758512	0.033763446	PASS
GJB6	13	20221962	20232395	-0.390786075	PASS
GJB7	6	87282978	87329278	-0.368590549	PASS
GJC1	17	44798448	44830816	0.088516311	PASS
GJC2	1	228149852	228159826	-0.256698369	PASS
GJC3	7	99923269	99929620	-0.262778904	PASS
GJD2	15	34751032	34754965	-0.396255771	PASS
GJD3	17	40362931	40363815	0.060555011	PASS
GJD4	10	35605410	35608935	0.039811807	PASS
GJE1	6	142133090	142135151	-0.035448111	PASS
GK	23	30653359	30731456	-0.012567603	PASS

GK2	4	79406352	79408293	-0.011382299	PASS
GK3P	4	165277812	165279679	-0.011382299	PASS
GK5	3	142157527	142225607	-0.356957581	PASS
GKAP1	9	83739421	83829516	0.365190713	suppress_segment_too_large
GKN1	2	68974573	68980974	-0.372959126	PASS
GKN2	2	68945232	68952970	-0.372959126	PASS
GLA	23	101397803	101408012	-0.012567603	PASS
GLB1	3	32996608	33097230	0.311018244	suppress_segment_too_large
GLB1L	2	219236606	219245478	0.025928102	PASS
GLB1L2	11	134331874	134378341	-0.419612865	PASS
GLB1L3	11	134274245	134319564	-0.419612865	PASS
GLCCI1	7	7968794	8094272	-0.69513118	PASS
GLCE	15	69160584	69272217	-0.697600509	PASS
GLDC	9	6532464	6645783	-0.310444659	PASS
GLDN	15	51341629	51408013	-0.396255771	PASS
GLE1	9	128504700	128542288	0.365190713	suppress_segment_too_large
GLG1	16	74447427	74607144	-0.371357732	PASS
GLI1	12	57460135	57472262	-0.202191512	PASS
GLI2	2	120735623	120992653	-0.475215623	PASS
GLI3	7	41960950	42237870	-0.69513118	PASS
GLI4	8	143267433	143276931	0.307788765	PASS
GLIPR1	12	75480680	75503853	0.202141228	PASS
GLIPR1L1	12	75334639	75370560	0.202141228	PASS
GLIPR1L2	12	75391070	75432688	0.202141228	PASS
GLIPR2	9	36136536	36163913	-0.310444659	PASS
GLIS1	1	53506237	53738106	0.033763446	PASS
GLIS2	16	4314761	4339597	0.069936844	PASS
GLIS3	9	3824127	4348392	-0.310444659	PASS
GLMN	1	92246402	92298987	0.033763446	PASS
GLMP	1	156290089	156295689	0.103015788	PASS
GLO1	6	38675925	38703141	0.048820648	PASS
GLOD4	17	757097	783390	0.128406487	PASS
GLOD5	23	48761750	48773648	-0.012567603	PASS
GLP1R	6	39048798	39087743	0.048820648	PASS
GLP2R	17	9822206	9892102	-0.139031004	PASS
GLRA1	5	151822513	151924842	-0.293443042	PASS
GLRA2	23	14529298	14731812	-0.012567603	PASS
GLRA3	4	174636914	174829314	-0.011382299	PASS
GLRA4	23	103707224	103728655	-0.012567603	PASS
GLRB	4	157076057	157172090	-0.011382299	PASS
GLRX	5	95751319	95823005	-0.293443042	PASS
GLRX2	1	193090866	193106114	-0.027073548	PASS
GLRX3	10	130136399	130184521	0.063874692	PASS
GLRX5	14	95533503	95544724	-0.192197564	PASS
GLS	2	190880827	190965552	0.025928102	PASS
GLS2	12	56470944	56488414	-0.202191512	PASS

GLT1D1	12	128853427	128984968	-0.240609783	PASS
GLT6D1	9	135623656	135639540	0.365190713	suppress_segment_too_large
GLT8D1	3	52694488	52706032	-0.234617007	PASS
GLT8D2	12	103988984	104064183	0.202141228	PASS
GLTP	12	109850943	109880488	0.202141228	PASS
GLTPD2	17	4788959	4790390	0.128406487	PASS
GLUD1	10	87050486	87094866	-0.292453993	PASS
GLUD2	23	121047588	121050080	-0.012567603	PASS
GLUL	1	182381704	182392206	-0.027073548	PASS
GLYAT	11	58640426	58731974	-0.129266491	PASS
GLYATL1	11	58905398	59043527	-0.129266491	PASS
GLYATL1	11	59086307	59094786	-0.129266491	PASS
GLYATL2	11	58834065	58904215	-0.129266491	PASS
GLYATL3	6	49499958	49527047	0.048820648	PASS
GLYCTK	3	52287089	52293476	-0.234617007	PASS
GLYR1	16	4803203	4847342	0.069936844	PASS
GM2A	5	151212150	151270440	-0.293443042	PASS
GMCL1	2	69829642	69881396	-0.372959126	PASS
GMCL2	5	178184503	178187432	-0.150979337	PASS
GMDS	6	1623806	2245692	0.048820648	PASS
GMEB1	1	28668732	28719353	0.033763446	PASS
GMEB2	20	63587602	63627041	-0.168668583	PASS
GMFB	14	54474484	54489196	0.060212819	PASS
GMFG	19	39328353	39342372	0.093487731	suppress_segment_too_large
GMIP	19	19629476	19643667	0.093487731	suppress_segment_too_large
GML	8	142834247	142916506	0.113969953	PASS
GMNC	3	190852877	190892429	0.318278996	suppress_segment_too_large
GMNN	6	24774931	24786099	0.048820648	PASS
GMPPA	2	219498867	219506989	0.025928102	PASS
GMPPB	3	49716844	49723951	-0.234617007	PASS
GMPR	6	16238580	16295549	0.048820648	PASS
GMPR2	14	24232422	24239242	0.060212819	PASS
GMPS	3	155870536	155944026	0.318278996	suppress_segment_too_large
GNA11	19	3094410	3124004	0.079632875	PASS
GNA12	7	2728112	2844324	-0.69513118	PASS
GNA13	17	65010715	65056839	0.088516311	PASS
GNA14	9	77423079	77648307	0.365190713	suppress_segment_too_large
GNA15	19	3136193	3163769	0.079632875	PASS
GNAI1	7	79768028	80226181	-0.262778904	PASS
GNAI2	3	50226292	50259355	-0.234617007	PASS
GNAI3	1	109548611	109618321	0.033763446	PASS
GNAL	18	11688956	11885685	-0.052305056	suppress_segment_too_large
GNAO1	16	56191347	56357457	-0.371357732	PASS
GNAQ	9	77716087	78031458	0.365190713	suppress_segment_too_large
GNAS	20	58839718	58911192	-0.168668583	PASS
GNAT1	3	50191612	50196516	-0.234617007	PASS

GNAT2	1	109603267	109619929	0.033763446	PASS
GNAT3	7	80458671	80512020	-0.262778904	PASS
GNAZ	22	23070361	23125037	-0.575930959	PASS
GNB1	1	1785285	1891117	0.373964873	suppress_segment_too_large
GNB1L	22	19783224	19854939	-0.575930959	PASS
GNB2	7	100673531	100679174	-0.262778904	PASS
GNB3	12	6839954	6847393	-0.086128028	PASS
GNB4	3	179396089	179451590	0.318278996	suppress_segment_too_large
GNB5	15	52115105	52191369	-0.396255771	PASS
GNE	9	36214441	36277056	-0.310444659	PASS
GNG10	9	111661335	111670246	0.365190713	suppress_segment_too_large
GNG11	7	93921699	93928610	-0.262778904	PASS
GNG12	1	67701466	67833467	0.033763446	PASS
GNG13	16	798041	800733	0.069936844	PASS
GNG14	19	12687998	12688422	0.093487731	suppress_segment_too_large
GNG2	14	51826195	51979342	0.060212819	PASS
GNG3	11	62707658	62709201	0.182439925	suppress_segment_too_large
GNG4	1	235547687	235650754	-0.256698369	PASS
GNG5	1	84498325	84506565	0.033763446	PASS
GNG7	19	2511219	2702709	0.079632875	PASS
GNG8	19	46634076	46634685	0.14996833	PASS
GNGT1	7	93591573	93911265	-0.262778904	PASS
GNGT2	17	49202791	49210574	0.088516311	PASS
GNL1	6	30541377	30557174	0.048820648	PASS
GNL2	1	37566816	37595935	0.033763446	PASS
GNL3	3	52681156	52694492	-0.234617007	PASS
GNL3L	23	54530211	54561071	-0.012567603	PASS
GNLY	2	85685175	85698854	0.066915641	PASS
GNMT	6	42960758	42963880	0.048820648	PASS
GNPAT	1	231241207	231277973	-0.256698369	PASS
GNPDA1	5	141991749	142013041	-0.293443042	PASS
GNPDA2	4	44682200	44726595	-0.011382299	PASS
GNPNAT1	14	52775194	52791668	0.060212819	PASS
GNPTAB	12	101745497	101830938	0.202141228	PASS
GNPTG	16	1351923	1364113	0.069936844	PASS
GNRH1	8	25419260	25424654	-0.345962679	PASS
GNRH2	20	3043622	3045747	-0.168668583	PASS
GNRHR	4	67739328	67754360	-0.011382299	PASS
GNS	12	64713445	64759447	-0.202191512	PASS
GOLGA1	9	124878357	124948492	0.365190713	suppress_segment_too_large
GOLGA2	9	128255829	128275995	0.365190713	suppress_segment_too_large
GOLGA3	12	132768909	132828858	0.270848837	PASS
GOLGA4	3	37243177	37366751	-0.234617007	PASS
GOLGA5	14	92794231	92839963	-0.192197564	PASS
GOLGA6A	15	74069857	74082550	-0.697600509	PASS
GOLGA6B	15	72654738	72666394	-0.697600509	PASS

GOLGA6C	15	75258599	75270199	-0.697600509	PASS
GOLGA6D	15	75282835	75295807	-0.697600509	PASS
GOLGA6L	15	23128268	23136822	-0.396255771	PASS
GOLGA6L	15	82339998	82349475	-0.697600509	PASS
GOLGA6L	15	23439498	23447234	-0.396255771	PASS
GOLGA6L	15	22458903	22469230	-0.750600437	PASS
GOLGA6L	15	84235773	84245368	-0.697600509	PASS
GOLGA6L	15	28841833	28848675	-0.396255771	PASS
GOLGA6L	15	82430018	82439153	-0.697600509	PASS
GOLGA7	8	41490396	41510980	-0.201142258	PASS
GOLGA7B	10	97850239	97871580	-0.367947924	PASS
GOLGA8A	15	34379068	34437466	-0.396255771	PASS
GOLGA8B	15	34525207	34588503	-0.396255771	PASS
GOLGA8F	15	28378621	28392018	-0.396255771	PASS
GOLGA8G	15	28519611	28533014	-0.396255771	PASS
GOLGA8H	15	30604126	30614561	-0.396255771	PASS
GOLGA8J	15	30083053	30093499	-0.396255771	PASS
GOLGA8K	15	32392782	32403292	-0.396255771	PASS
GOLGA8M	15	28701954	28738384	-0.396255771	PASS
GOLGA8N	15	32593456	32607310	-0.396255771	PASS
GOLGA8C	15	32445106	32455634	-0.396255771	PASS
GOLGA8Q	15	30552078	30562501	-0.396255771	PASS
GOLGA8R	15	30403740	30414162	-0.396255771	PASS
GOLGA8S	15	23354748	23367231	-0.396255771	PASS
GOLGA8T	15	30135149	30145567	-0.396255771	PASS
GOLGB1	3	121663199	121749767	0.313454204	suppress_segment_too_large
GOLIM4	3	168008677	168095975	0.318278996	suppress_segment_too_large
GOLM1	9	86026146	86100173	0.365190713	suppress_segment_too_large
GOLPH3	5	32124704	32174350	0.055007813	PASS
GOLPH3L	1	150646225	150697144	0.103015788	PASS
GOLT1A	1	204198160	204214092	-0.256698369	PASS
GOLT1B	12	21501781	21518408	0.362961408	suppress_segment_too_large
GON4L	1	155749662	155859400	0.103015788	PASS
GON7	14	93202894	93207094	-0.192197564	PASS
GOPC	6	117560269	117602542	-0.368590549	PASS
GORAB	1	170532129	170553446	-0.027073548	PASS
GORASP1	3	39096659	39108363	-0.234617007	PASS
GORASP2	2	170928464	170967129	0.039726562	PASS
GOSR1	17	30477362	30527592	0.060555011	PASS
GOSR2	17	46923075	46975524	0.088516311	PASS
GOT1	10	99396870	99431569	-0.367947924	PASS
GOT1L1	8	37934281	37940129	-0.201142258	PASS
GOT2	16	58707131	58734357	-0.371357732	PASS
GP1BA	17	4932297	4935030	0.128406487	PASS
GP1BB	22	19722945	19724771	-0.575930959	PASS
GP2	16	20309572	20327808	0.069936844	PASS

GP5	3	194394821	194398354	0.326834516	PASS
GP6	19	55013705	55038264	0.121336758	PASS
GP9	3	129060767	129062406	0.313454204	suppress_segment_too_large
GPA33	1	167052836	167166479	-0.027073548	PASS
GPAA1	8	144082590	144086216	0.307788765	PASS
GPALPP1	13	44989529	45037669	-0.390786075	PASS
GPAM	10	112149864	112215377	0.063874692	PASS
GPANK1	6	31661229	31666283	0.048820648	PASS
GPAT2	2	96021946	96039451	0.048238121	PASS
GPAT3	4	83535914	83605875	-0.011382299	PASS
GPAT4	8	41577187	41625001	-0.201142258	PASS
GPATCH1	19	33080880	33130542	0.093487731	suppress_segment_too_large
GPATCH1	2	37084451	37099244	0.040498426	PASS
GPATCH2	1	217426992	217631082	-0.256698369	PASS
GPATCH2	14	76151916	76254342	-0.192197564	PASS
GPATCH3	1	26890488	26900466	0.033763446	PASS
GPATCH4	1	156594487	156601496	0.103015788	PASS
GPATCH8	17	44395284	44503430	0.088516311	PASS
GPBAR1	2	218259496	218263859	0.025928102	PASS
GPBP1	5	57173948	57264679	-0.293443042	PASS
GPBP1L1	1	45627304	45688113	0.033763446	PASS
GPC1	2	240435671	240468078	0.025928102	PASS
GPC2	7	100169606	100177372	-0.262778904	PASS
GPC3	23	133535745	133985895	-0.012567603	PASS
GPC4	23	133300103	133415490	-0.012567603	PASS
GPC5	13	91398607	92873682	-0.390786075	PASS
GPC6	13	93226842	94407401	-0.390786075	PASS
GPCPD1	20	5544404	5611026	-0.168668583	PASS
GPD1	12	50103819	50111319	-0.202191512	PASS
GPD1L	3	32105689	32168713	0.311018244	suppress_segment_too_large
GPD2	2	156435290	156613735	-0.475215623	PASS
GPER1	7	1082208	1093815	-0.69513118	PASS
GPHA2	11	64934471	64935888	0.182439925	suppress_segment_too_large
GPHB5	14	63312835	63318879	0.060212819	PASS
GPHN	14	66507407	67181803	0.060212819	PASS
GPI	19	34359480	34402413	0.093487731	suppress_segment_too_large
GPIHBP1	8	143213193	143217170	0.307788765	PASS
GPKOW	23	49113389	49123801	-0.012567603	PASS
GPLD1	6	24424565	24495205	0.048820648	PASS
GPM6A	4	175632934	176002664	-0.011382299	PASS
GPM6B	23	13771031	13938638	-0.012567603	PASS
GPN1	2	27628247	27651508	0.040498426	PASS
GPN2	1	26876133	26890297	0.033763446	PASS
GPN3	12	110452484	110469268	0.202141228	PASS
GPNMB	7	23235967	23275108	-0.69513118	PASS
GPR1	2	206175316	206218047	0.025928102	PASS

GPR101	23	137030148	137031674	-0.012567603	PASS
GPR107	9	130053426	130140169	0.365190713	suppress_segment_too_large
GPR108	19	6729914	6737603	0.079632875	PASS
GPR119	23	130384440	130385447	-0.012567603	PASS
GPR12	13	26755200	26760785	-0.390786075	PASS
GPR132	14	105049389	105065445	-0.192197564	PASS
GPR135	14	59429022	59465342	0.060212819	PASS
GPR137	11	64270062	64289500	0.182439925	suppress_segment_too_large
GPR137B	1	236142505	236221865	-0.256698369	PASS
GPR137C	14	52553148	52637713	0.060212819	PASS
GPR139	16	20031485	20073917	0.069936844	PASS
GPR141	7	37683796	37833788	-0.69513118	PASS
GPR142	17	74367407	74372622	0.088516311	PASS
GPR143	23	9725346	9786297	-0.012567603	PASS
GPR146	7	1044576	1059261	-0.69513118	PASS
GPR148	2	130729070	130730336	-0.475215623	PASS
GPR149	3	154337672	154429715	0.318278996	suppress_segment_too_large
GPR15	3	98531899	98533150	0.313454204	suppress_segment_too_large
GPR150	5	95620078	95622142	-0.293443042	PASS
GPR151	5	146513103	146516190	-0.293443042	PASS
GPR152	11	67451301	67452729	0.182439925	suppress_segment_too_large
GPR153	1	6247346	6260975	0.373964873	suppress_segment_too_large
GPR155	2	174431571	174487094	0.039726562	PASS
GPR156	3	120164645	120285094	0.313454204	suppress_segment_too_large
GPR157	1	9100305	9129170	0.373964873	suppress_segment_too_large
GPR158	10	25174802	25602226	0.039811807	PASS
GPR160	3	170037929	170085403	0.318278996	suppress_segment_too_large
GPR161	1	168079543	168137667	-0.027073548	PASS
GPR162	12	6821545	6829972	-0.086128028	PASS
GPR17	2	127645864	127652639	-0.475215623	PASS
GPR171	3	151197832	151203201	0.318278996	suppress_segment_too_large
GPR173	23	53049091	53080615	-0.012567603	PASS
GPR174	23	79144663	79175315	-0.012567603	PASS
GPR176	15	39799032	39920892	-0.396255771	PASS
GPR179	17	38325530	38343847	0.060555011	PASS
GPR18	13	99254714	99261744	-0.390786075	PASS
GPR180	13	94601903	94634645	-0.390786075	PASS
GPR182	12	56994446	56998441	-0.202191512	PASS
GPR183	13	99294530	99307405	-0.390786075	PASS
GPR19	12	12660891	12696207	0.362961408	suppress_segment_too_large
GPR20	8	141356500	141367267	0.113969953	PASS
GPR21	9	123034527	123035696	0.365190713	suppress_segment_too_large
GPR22	7	107470018	107475659	-0.388946609	PASS
GPR25	1	200872955	200874178	-0.256698369	PASS
GPR26	10	123666355	123694607	0.063874692	PASS
GPR27	3	71754050	71756496	-0.234617007	PASS

GPR3	1	27392644	27395814	0.033763446	PASS
GPR31	6	167156271	167158329	-0.035448111	PASS
GPR32	19	50770464	50771732	-0.016761391	PASS
GPR33	14	31482875	31488142	0.060212819	PASS
GPR34	23	41688973	41697277	-0.012567603	PASS
GPR35	2	240605431	240631259	0.025928102	PASS
GPR37	7	124745997	124765627	-0.388946609	PASS
GPR37L1	1	202122858	202133592	-0.256698369	PASS
GPR39	2	132416574	132646559	-0.475215623	PASS
GPR4	19	45589764	45602208	0.14996833	PASS
GPR42	19	35370929	35372962	0.093487731	suppress_segment_too_large
GPR45	2	105241743	105243049	-0.532032081	PASS
GPR50	23	151176653	151181465	-0.012567603	PASS
GPR52	1	174448111	174449198	-0.027073548	PASS
GPR55	2	230907318	230961066	0.025928102	PASS
GPR6	6	109978256	109980718	-0.368590549	PASS
GPR61	1	109539872	109548406	0.033763446	PASS
GPR62	3	51955314	51957504	-0.234617007	PASS
GPR63	6	96794126	96837463	-0.368590549	PASS
GPR65	14	88005124	88014811	-0.192197564	PASS
GPR68	14	91232532	91253925	-0.192197564	PASS
GPR75	2	53852913	53859989	0.040498426	PASS
GPR75-AS	2	53670293	53860160	0.040498426	PASS
GPR78	4	8558725	8619761	0.279089364	PASS
GPR82	23	41724155	41730135	-0.012567603	PASS
GPR83	11	94377311	94401419	0.062969153	PASS
GPR84	12	54362445	54364487	-0.202191512	PASS
GPR85	7	113078331	113087778	-0.388946609	PASS
GPR87	3	151294086	151316952	0.318278996	suppress_segment_too_large
GPR88	1	100538137	100542018	0.033763446	PASS
GPR89A	1	145607990	145670648	-0.157385092	PASS
GPR89B	1	147928393	147993521	-0.157385092	PASS
GPRASP1	23	102651366	102659083	-0.012567603	PASS
GPRASP2	23	102712176	102717733	-0.012567603	PASS
GPRC5A	12	12890782	12917937	0.362961408	suppress_segment_too_large
GPRC5B	16	19856691	19886167	0.069936844	PASS
GPRC5C	17	74424851	74451653	0.088516311	PASS
GPRC5D	12	12940775	12952147	0.362961408	suppress_segment_too_large
GPRC6A	6	116792085	116829037	-0.368590549	PASS
GPRIN1	5	176595802	176610133	-0.150979337	PASS
GPRIN2	10	46549044	46555530	-0.404852538	PASS
GPRIN3	4	89236386	89308010	-0.011382299	PASS
GPS1	17	82050691	82057470	0.088516311	PASS
GPS2	17	7311324	7315564	0.128406487	PASS
GPSM1	9	136327476	136359605	0.365190713	suppress_segment_too_large
GPSM2	1	108875350	108934545	0.033763446	PASS

GPSM3	6	32190766	32195523	0.048820648	PASS
GPT	8	144502973	144507174	0.307788765	PASS
GPT2	16	46884378	46931297	0.069936844	PASS
GPX2	14	64939152	64942905	0.060212819	PASS
GPX3	5	151020438	151028993	-0.293443042	PASS
GPX4	19	1103926	1106791	0.079632875	PASS
GPX5	6	28525925	28534952	0.048820648	PASS
GPX6	6	28503296	28528215	0.048820648	PASS
GPX7	1	52602372	52609051	0.033763446	PASS
GPX8	5	55160118	55167071	-0.293443042	PASS
GRAMD1A	19	34994784	35026471	0.093487731	suppress_segment_too_large
GRAMD1B	11	123358428	123627774	-0.949752196	PASS
GRAMD1C	3	113828182	113947174	0.313454204	suppress_segment_too_large
GRAMD2A	15	72159807	72197785	-0.697600509	PASS
GRAMD2B	5	126360132	126496494	-0.293443042	PASS
GRAMD4	22	46576012	46679790	-0.808211634	PASS
GRAP	17	19020673	19047637	0.024610076	PASS
GRAP2	22	39901082	39973721	-0.575930959	PASS
GRAPL	17	19127469	19159176	0.024610076	PASS
GRASP	12	52006940	52015889	-0.202191512	PASS
GRB10	7	50590063	50793462	-0.750061359	PASS
GRB14	2	164492812	164621848	0.039726562	PASS
GRB2	17	75318076	75405709	0.088516311	PASS
GRB7	17	39737927	39747291	0.060555011	PASS
GREB1	2	11482341	11642788	0.040498426	PASS
GREB1L	18	21242242	21525417	-0.376397843	PASS
GREM1	15	32717974	32745107	-0.396255771	PASS
GREM2	1	240489573	240612149	-0.648598641	PASS
GRHL1	2	9951693	10002277	0.040498426	PASS
GRHL2	8	101492432	101669726	0.279445683	PASS
GRHL3	1	24319322	24364482	0.033763446	PASS
GRHPR	9	37422666	37436990	-0.310444659	PASS
GRIA1	5	153489615	153813869	-0.293443042	PASS
GRIA2	4	157204182	157366075	-0.011382299	PASS
GRIA3	23	123184153	123490915	-0.012567603	PASS
GRIA4	11	105609994	105982092	-0.356188624	PASS
GRID1	10	85599555	86366493	0.072405807	PASS
GRID2	4	92303622	93810157	-0.011382299	PASS
GRID2IP	7	6497462	6551436	-0.69513118	PASS
GRIFIN	7	2474844	2476894	-0.69513118	PASS
GRIK1	21	29536933	29940033	0.12860881	suppress_segment_too_large
GRIK2	6	101181257	102070083	-0.368590549	PASS
GRIK3	1	36795527	37034129	0.033763446	PASS
GRIK4	11	120511700	120988904	-0.356188624	PASS
GRIK5	19	41998321	42069498	0.093487731	suppress_segment_too_large
GRIN1	9	137138390	137168762	0.365190713	suppress_segment_too_large

GRIN2A	16	9753404	10182754	0.069936844	PASS
GRIN2B	12	13437942	13981957	0.362961408	suppress_segment_too_large
GRIN2C	17	74842023	74861504	0.088516311	PASS
GRIN2D	19	48394875	48444931	0.14996833	PASS
GRIN3A	9	101569353	101738580	0.365190713	suppress_segment_too_large
GRIN3B	19	1000419	1009732	0.079632875	PASS
GRINA	8	143990058	143993415	0.307788765	PASS
GRIP1	12	66347431	67069162	-0.202191512	PASS
GRIP2	3	14489111	14556075	0.311018244	suppress_segment_too_large
GRIPAP1	23	48973720	49002264	-0.012567603	PASS
GRK1	13	113667155	113737735	-0.390786075	PASS
GRK2	11	67266410	67286556	0.182439925	suppress_segment_too_large
GRK3	22	25564849	25729294	-0.575930959	PASS
GRK4	4	2963608	3040747	0.279089364	PASS
GRK5	10	119207589	119459742	0.063874692	PASS
GRK6	5	177403204	177442901	-0.150979337	PASS
GRK7	3	141778148	141818490	-0.356957581	PASS
GRM1	6	146027646	146437598	-0.035448111	PASS
GRM2	3	51707070	51718613	-0.234617007	PASS
GRM3	7	86643914	86864884	-0.262778904	PASS
GRM4	6	34018645	34155622	0.048820648	PASS
GRM5	11	88504576	89065945	0.062969153	PASS
GRM6	5	178977587	178996206	-0.150979337	PASS
GRM7	3	6770001	7741533	0.311018244	suppress_segment_too_large
GRM8	7	126438598	127253294	-0.388946609	PASS
GRN	17	44345086	44353102	0.088516311	PASS
GRP	18	59220168	59230774	-0.376397843	PASS
GRPEL1	4	7058906	7068197	0.279089364	PASS
GRPEL2	5	149345430	149354583	-0.293443042	PASS
GRPR	23	16123556	16153021	-0.012567603	PASS
GRSF1	4	70815782	70839945	-0.011382299	PASS
GRTP1	13	113324164	113364148	-0.390786075	PASS
GRWD1	19	48445773	48457022	0.14996833	PASS
GRXCR1	4	42893267	43030658	-0.011382299	PASS
GRXCR2	5	145858521	145937126	-0.293443042	PASS
GSAP	7	77310751	77416400	-0.262778904	PASS
GSC	14	94768216	94770230	-0.192197564	PASS
GSC2	22	19148576	19150283	-0.575930959	PASS
GSDMA	17	39953263	39977766	0.060555011	PASS
GSDMB	17	39904595	39919854	0.060555011	PASS
GSDMC	8	129748196	129786888	0.113969953	PASS
GSDMD	8	143553207	143563062	0.307788765	PASS
GSDME	7	24698351	24758113	-0.69513118	PASS
GSE1	16	85169525	85676204	-0.371357732	PASS
GSG1	12	13083560	13103683	0.362961408	suppress_segment_too_large
GSG1L	16	27787535	28063509	0.069936844	PASS

GSG1L2	17	9802386	9822071	-0.139031004	PASS
GSK3A	19	42230186	42242625	0.093487731	suppress_segment_too_large
GSK3B	3	119821323	120094417	0.313454204	suppress_segment_too_large
GSKIP	14	96363452	96387288	-0.192197564	PASS
GSN	9	121207794	121332843	0.365190713	suppress_segment_too_large
GSPT1	16	11868128	11916082	0.069936844	PASS
GSPT2	23	51743431	51746232	-0.012567603	PASS
GSR	8	30678061	30727999	-0.345962679	PASS
GSS	20	34928433	34956027	-0.168668583	PASS
GSTA1	6	52791664	52803910	0.048820648	PASS
GSTA2	6	52750089	52763569	0.048820648	PASS
GSTA3	6	52896639	52909685	0.048820648	PASS
GSTA4	6	52977948	52995378	0.048820648	PASS
GSTA5	6	52831655	52846095	0.048820648	PASS
GSTCD	4	105708778	105847728	-0.011382299	PASS
GSTK1	7	143244093	143270854	-0.388946609	PASS
GSTM1	1	109687814	109709039	0.033763446	PASS
GSTM2	1	109668022	109709551	0.033763446	PASS
GSTM3	1	109733932	109741038	0.033763446	PASS
GSTM4	1	109656081	109674836	0.033763446	PASS
GSTM5	1	109711780	109775428	0.033763446	PASS
GSTO1	10	104235356	104267459	-0.367947924	PASS
GSTO2	10	104268873	104304945	-0.367947924	PASS
GSTP1	11	67583595	67586656	0.182439925	suppress_segment_too_large
GSTT2B	22	23957414	23961186	-0.575930959	PASS
GSTZ1	14	77320884	77331597	-0.192197564	PASS
GSX1	13	27792643	27794768	-0.390786075	PASS
GSX2	4	54099523	54102505	-0.011382299	PASS
GTDC1	2	143938068	144332568	-0.475215623	PASS
GTF2A1	14	81175452	81221377	-0.192197564	PASS
GTF2A1L	2	48617798	48733148	0.040498426	PASS
GTF2A2	15	59638062	59657541	-0.697600509	PASS
GTF2B	1	88852932	88891944	0.033763446	PASS
GTF2E1	3	120742637	120783069	0.313454204	suppress_segment_too_large
GTF2E2	8	30578318	30658251	-0.345962679	PASS
GTF2F1	19	6379569	6393981	0.079632875	PASS
GTF2F2	13	45120515	45284909	-0.390786075	PASS
GTF2H1	11	18322295	18367044	-0.405444717	PASS
GTF2H2	5	71032670	71067689	-0.293443042	PASS
GTF2H2C	5	69560208	69594723	-0.293443042	PASS
GTF2H3	12	123633739	123662606	0.202141228	PASS
GTF2H4	6	30908184	30914106	0.048820648	PASS
GTF2H5	6	158168350	158199344	-0.035448111	PASS
GTF2I	7	74657667	74760692	-0.262778904	PASS
GTF2IRD1	7	74453790	74602604	-0.262778904	PASS
GTF2IRD2	7	74796144	74851551	-0.262778904	PASS

GTF2IRD2	7	75092573	75149817	-0.262778904	PASS
GTF3A	13	27424544	27435823	-0.390786075	PASS
GTF3C1	16	27459555	27549913	0.069936844	PASS
GTF3C2	2	27325849	27357034	0.040498426	PASS
GTF3C3	2	196763032	196799725	0.025928102	PASS
GTF3C4	9	132670035	132694955	0.365190713	suppress_segment_too_large
GTF3C5	9	133030675	133058503	0.365190713	suppress_segment_too_large
GTF3C6	6	110958560	110967890	-0.368590549	PASS
GTPBP1	22	38705723	38738299	-0.575930959	PASS
GTPBP10	7	90335223	90391455	-0.262778904	PASS
GTPBP2	6	43605316	43629162	0.048820648	PASS
GTPBP3	19	17334920	17342735	0.093487731	suppress_segment_too_large
GTPBP4	10	988019	1019936	0.039811807	PASS
GTPBP6	23	304529	318819	-0.012567603	PASS
GTPBP8	3	112990918	113015060	0.313454204	suppress_segment_too_large
GTSE1	22	46296741	46330810	-0.808211634	PASS
GTSF1	12	54455950	54473602	-0.202191512	PASS
GTSF1L	20	43726164	43726998	-0.168668583	PASS
GUCA1A	6	42155406	42180056	0.048820648	PASS
GUCA1B	6	42184401	42194916	0.048820648	PASS
GUCA1C	3	108907792	108953895	0.313454204	suppress_segment_too_large
GUCA2A	1	42162691	42164718	0.033763446	PASS
GUCA2B	1	42153421	42155824	0.033763446	PASS
GUCD1	22	24540423	24555935	-0.575930959	PASS
GUCY1A1	4	155666711	155732349	-0.011382299	PASS
GUCY1A2	11	106674012	107018524	-0.356188624	PASS
GUCY1B1	4	155758992	155807591	-0.011382299	PASS
GUCY2C	12	14612632	14696585	0.362961408	suppress_segment_too_large
GUCY2D	17	8002594	8020339	0.128406487	PASS
GUCY2F	23	109372906	109482072	-0.012567603	PASS
GUF1	4	44678427	44700926	-0.011382299	PASS
GUK1	1	228139962	228148984	-0.256698369	PASS
GULP1	2	188291669	188595931	0.025928102	PASS
GUSB	7	65960684	65982314	-0.750061359	PASS
GVQW2	6	138725211	138773652	-0.035448111	PASS
GVQW3	11	76381313	76414619	0.062969153	PASS
GXYLT1	12	42081845	42144879	-0.202191512	PASS
GXYLT2	3	72888073	72998138	-0.234617007	PASS
GYG1	3	148991341	149027668	0.318278996	suppress_segment_too_large
GYG2	23	2828788	2882820	-0.012567603	PASS
GYPA	4	144109303	144140786	-0.011382299	PASS
GYPB	4	143996104	144019345	-0.011382299	PASS
GYPC	2	126655933	126696670	-0.475215623	PASS
GYPE	4	143870867	143905563	-0.011382299	PASS
GYS1	19	48968125	48993310	0.14996833	PASS
GYS2	12	21536189	21604847	0.362961408	suppress_segment_too_large

GZF1	20	23362182	23373063	-0.168668583	PASS
GZMA	5	55102648	55110252	-0.293443042	PASS
GZMB	14	24630954	24634267	0.060212819	PASS
GZMH	14	24606480	24609699	0.060212819	PASS
GZMK	5	55024253	55034570	-0.293443042	PASS
GZMM	19	544034	549924	0.079632875	PASS
H1F0	22	37805093	37807436	-0.575930959	PASS
H1FNT	12	48328980	48330279	-0.202191512	PASS
H1FOO	3	129543214	129551467	0.313454204	suppress_segment_too_large
H1FX	3	129314771	129316277	0.313454204	suppress_segment_too_large
H2AFB1	23	154884972	154885558	-0.012567603	PASS
H2AFJ	12	14774383	14778002	0.362961408	suppress_segment_too_large
H2AFV	7	44826791	44848083	-0.69513118	PASS
H2AFX	11	119093854	119095467	-0.356188624	PASS
H2AFY	5	135333900	135399914	-0.293443042	PASS
H2AFY2	10	70052796	70112280	0.072405807	PASS
H2AFZ	4	99948086	99950388	-0.011382299	PASS
H2BFM	23	104039949	104042454	-0.012567603	PASS
H2BFS	21	5972924	43565648	-0.304975024	PASS
H2BFWT	23	104011147	104013687	-0.012567603	PASS
H3F3A	1	226061851	226072001	-0.256698369	PASS
H3F3B	17	75776434	75785893	0.088516311	PASS
H3F3C	12	31791185	31792241	0.362961408	suppress_segment_too_large
H6PD	1	9234775	9271337	0.373964873	suppress_segment_too_large
HAAO	2	42767089	42792593	0.040498426	PASS
HABP2	10	113550837	113589602	0.063874692	PASS
HABP4	9	96450201	96491336	0.365190713	suppress_segment_too_large
HACD1	10	17589032	17617377	0.039811807	PASS
HACD2	3	123490820	123585185	0.313454204	suppress_segment_too_large
HACD3	15	65530418	65578352	-0.697600509	PASS
HACD4	9	20999515	21031636	-0.310444659	PASS
HACE1	6	104728093	104859919	-0.368590549	PASS
HACL1	3	15560704	15601852	0.311018244	suppress_segment_too_large
HADH	4	107989714	108035175	-0.011382299	PASS
HADHA	2	26190635	26244726	0.040498426	PASS
HADHB	2	26243170	26290468	0.040498426	PASS
HAGH	16	1795620	1827194	0.069936844	PASS
HAGHL	16	726936	735525	0.069936844	PASS
HAL	12	95972662	95996365	0.202141228	PASS
HAMP	19	35280716	35285143	0.093487731	suppress_segment_too_large
HAND1	5	154474972	154478264	-0.293443042	PASS
HAND2	4	173524969	173530229	-0.011382299	PASS
HAO1	20	7882981	7940474	-0.168668583	PASS
HAO2	1	119368779	119394130	-0.157385092	PASS
HAP1	17	41717742	41734644	0.060555011	PASS
HAPLN1	5	83637805	83721613	-0.293443042	PASS

HAPLN2	1	156619294	156625725	0.103015788	PASS
HAPLN3	15	88877288	88895626	-0.697600509	PASS
HAPLN4	19	19255641	19262796	0.093487731	suppress_segment_too_large
HARBI1	11	46602861	46617909	0.117376172	PASS
HARS	5	140673904	140691862	-0.293443042	PASS
HARS2	5	140691426	140699318	-0.293443042	PASS
HAS1	19	51713112	51723994	-0.016761391	PASS
HAS2	8	121612116	121641390	0.279445683	PASS
HAS3	16	69105564	69118719	-0.371357732	PASS
HASPIN	17	3723903	3726699	0.128406487	PASS
HAT1	2	171922448	171983682	0.039726562	PASS
HAUS1	18	46104332	46128333	-0.376397843	PASS
HAUS2	15	42548810	42569994	-0.396255771	PASS
HAUS3	4	2227464	2242164	0.279089364	PASS
HAUS4	14	22946228	22957161	0.060212819	PASS
HAUS5	19	35612744	35625349	0.093487731	suppress_segment_too_large
HAUS6	9	19053143	19103119	-0.310444659	PASS
HAUS7	23	153447666	153495516	-0.012567603	PASS
HAUS8	19	17049729	17075625	0.093487731	suppress_segment_too_large
HAVCR1	5	157029413	157059119	-0.293443042	PASS
HAVCR2	5	157085832	157142869	-0.293443042	PASS
HAX1	1	154272511	154275875	0.103015788	PASS
HBA1	16	176680	177522	0.069936844	PASS
HBA2	16	172876	173710	0.069936844	PASS
HBB	11	5225464	5229395	-0.957525224	PASS
HBD	11	5232678	5243657	-0.957525224	PASS
HBE1	11	5268345	5505652	-0.957525224	PASS
HBEGF	5	140332843	140346631	-0.293443042	PASS
HBG1	11	5248079	5249859	-0.957525224	PASS
HBG2	11	5253190	5505605	-0.957525224	PASS
HBM	16	153892	166768	0.069936844	PASS
HBP1	7	107168961	107202529	-0.388946609	PASS
HBQ1	16	180453	181181	0.069936844	PASS
HBS1L	6	134960378	135103056	-0.035448111	PASS
HBZ	16	152687	154503	0.069936844	PASS
HCAR1	12	122726076	122730843	0.202141228	PASS
HCAR2	12	122701293	122703343	0.202141228	PASS
HCAR3	12	122714756	122716892	0.202141228	PASS
HCCS	23	11111301	11123078	-0.012567603	PASS
HCFC1	23	153947553	153971807	-0.012567603	PASS
HCFC1R1	16	3022620	3024286	0.069936844	PASS
HCFC2	12	104064457	104106524	0.202141228	PASS
HCK	20	32052188	32101856	-0.168668583	PASS
HCLS1	3	121631399	121660927	0.313454204	suppress_segment_too_large
HCN1	5	45254950	45696498	-0.293443042	PASS
HCN2	19	589893	617159	0.079632875	PASS

HCN3	1	155277583	155289848	0.103015788	PASS
HCN4	15	73319859	73369264	-0.697600509	PASS
HCRT	17	42184060	42185452	0.060555011	PASS
HCRTR1	1	31617686	31632518	0.033763446	PASS
HCRTR2	6	55106460	55282620	0.048820648	PASS
HCST	19	35902480	35904377	0.093487731	suppress_segment_too_large
HDAC1	1	32292086	32333635	0.033763446	PASS
HDAC10	22	50245183	50251405	-0.808211634	PASS
HDAC11	3	13479724	13506424	0.311018244	suppress_segment_too_large
HDAC2	6	113933028	114011308	-0.368590549	PASS
HDAC3	5	141620876	141636870	-0.293443042	PASS
HDAC4	2	239048168	239401654	0.025928102	PASS
HDAC5	17	44076746	44123702	0.088516311	PASS
HDAC6	23	48801377	48824982	-0.012567603	PASS
HDAC7	12	47782722	47833132	-0.202191512	PASS
HDAC8	23	72329516	72573199	-0.012567603	PASS
HDAC9	7	18086949	19002416	-0.69513118	PASS
HDC	15	50241947	50266026	-0.396255771	PASS
HDDC2	6	125219962	125302078	-0.458570262	PASS
HDDC3	15	90929964	90935196	-0.697600509	PASS
HDGF	1	156742107	156766925	0.103015788	PASS
HDGFL1	6	22569493	22571666	0.048820648	PASS
HDGFL2	19	4472287	4502211	0.079632875	PASS
HDGFL3	15	83112738	83208018	-0.697600509	PASS
HDHD2	18	47107403	47150520	-0.376397843	PASS
HDHD3	9	113373419	113376999	0.365190713	suppress_segment_too_large
HDHD5	22	17137511	17165287	-0.575930959	PASS
HDLBP	2	241227264	241317061	0.025928102	PASS
HDX	23	84317874	84502479	-0.012567603	PASS
HEATR1	1	236549005	236604504	-0.256698369	PASS
HEATR3	16	50065941	50106387	0.069936844	PASS
HEATR4	14	73478484	73558947	0.060212819	PASS
HEATR5A	14	31291791	31420582	0.060212819	PASS
HEATR5B	2	36968383	37084342	0.040498426	PASS
HEATR6	17	60043194	60078931	0.088516311	PASS
HEATR9	17	35854946	35868891	0.060555011	PASS
HEBP1	12	12974864	13000273	0.362961408	suppress_segment_too_large
HEBP2	6	138403531	138422197	-0.035448111	PASS
HECA	6	139135112	139180802	-0.035448111	PASS
HECTD1	14	31100112	31207804	0.060212819	PASS
HECTD2	10	91409280	91514829	-0.39678585	PASS
HECTD3	1	45002540	45011329	0.033763446	PASS
HECTD4	12	112160188	112382439	0.202141228	PASS
HECW1	7	43112599	43566001	-0.69513118	PASS
HECW2	2	196189099	196593692	0.025928102	PASS
HEG1	3	124965710	125055958	0.313454204	suppress_segment_too_large

HELB	12	66302545	66347645	-0.202191512	PASS
HELLS	10	94501434	94613905	-0.39678585	PASS
HELQ	4	83407343	83455856	-0.011382299	PASS
HELT	4	185018841	185020804	-0.462744716	PASS
HELZ	17	67070438	67245989	0.088516311	PASS
HELZ2	20	63558086	63574239	-0.168668583	PASS
HEMGN	9	97926791	97944856	0.365190713	suppress_segment_too_large
HEMK1	3	50569152	50596168	-0.234617007	PASS
HENMT1	1	108648290	108661526	0.033763446	PASS
HEPACAM	11	124919193	124936412	-0.419612865	PASS
HEPACAM	7	93188586	93226524	-0.262778904	PASS
HEPH	23	66162549	66268867	-0.012567603	PASS
HEPHL1	11	94021361	94113751	0.062969153	PASS
HEPN1	11	124919244	124920677	-0.419612865	PASS
HERC1	15	63608618	63833942	-0.697600509	PASS
HERC2	15	28111040	28322152	-0.396255771	PASS
HERC3	4	88592423	88708542	-0.011382299	PASS
HERC4	10	67921899	68075348	0.072405807	PASS
HERC5	4	88457117	88506163	-0.011382299	PASS
HERC6	4	88378739	88443111	-0.011382299	PASS
HERPUD1	16	56932048	56944863	-0.371357732	PASS
HERPUD2	7	35632659	35695571	-0.69513118	PASS
HES1	3	194136145	194138732	0.326834516	PASS
HES2	1	6412418	6424670	0.373964873	suppress_segment_too_large
HES3	1	6244192	6245578	0.373964873	suppress_segment_too_large
HES4	1	998962	1000172	0.373964873	suppress_segment_too_large
HES5	1	2528745	2530245	0.373964873	suppress_segment_too_large
HES6	2	238238267	238240662	0.025928102	PASS
HES7	17	8120590	8124092	0.128406487	PASS
HESX1	3	57197838	57227606	-0.234617007	PASS
HEXA	15	72340919	72376476	-0.697600509	PASS
HEXB	5	74640023	74722647	-0.293443042	PASS
HEXDC	17	82418318	82442645	0.088516311	PASS
HEXIM1	17	45148502	45152101	0.088516311	PASS
HEXIM2	17	45160700	45170040	0.088516311	PASS
HEY1	8	79764010	79767863	0.279445683	PASS
HEY2	6	125747664	125761269	-0.458570262	PASS
HEYL	1	39624153	39639945	0.033763446	PASS
HFE	6	26087281	26098343	0.048820648	PASS
HFM1	1	91260766	91404869	0.033763446	PASS
HGD	3	120628173	120682571	0.313454204	suppress_segment_too_large
HGF	7	81699008	81770438	-0.262778904	PASS
HGFAC	4	3441887	3449495	0.279089364	PASS
HGH1	8	144137769	144140843	0.307788765	PASS
HGS	17	81683326	81703138	0.088516311	PASS
HGSNAT	8	43140455	43202855	-0.201142258	PASS

HHAT	1	210328252	210676296	-0.256698369	PASS
HHATL	3	42692663	42702827	-0.234617007	PASS
HHEX	10	92689951	92695646	-0.39678585	PASS
HHIP	4	144646021	144745271	-0.011382299	PASS
HHIPL1	14	99645110	99680569	-0.192197564	PASS
HHIPL2	1	222522258	222548103	-0.256698369	PASS
HHLA1	8	132061486	132111159	0.113969953	PASS
HHLA2	3	108296490	108378285	0.313454204	suppress_segment_too_large
HHLA3	1	70354805	70385339	0.033763446	PASS
HIBADH	7	27525442	27662995	-0.69513118	PASS
HIBCH	2	190189735	190344193	0.025928102	PASS
HIC1	17	2054154	2063241	0.128406487	PASS
HIC2	22	21417404	21451463	-0.575930959	PASS
HID1	17	74950743	74973166	0.088516311	PASS
HIF1A	14	61695513	61748259	0.060212819	PASS
HIF1AN	10	100529072	100559998	-0.367947924	PASS
HIF3A	19	46297046	46343433	0.14996833	PASS
HIGD1A	3	42784298	42804531	-0.234617007	PASS
HIGD1B	17	44846353	44850480	0.088516311	PASS
HIGD1C	12	50953922	50970506	-0.202191512	PASS
HIGD2A	5	176388747	176389771	-0.150979337	PASS
HIGD2B	15	72675782	72686149	-0.697600509	PASS
HIKESHI	11	86302211	86345931	0.062969153	PASS
HILPDA	7	128455849	128458418	-0.388946609	PASS
HINFP	11	119121587	119136044	-0.356188624	PASS
HINT1	5	131159027	131171735	-0.293443042	PASS
HINT2	9	35812960	35815354	-0.310444659	PASS
HINT3	6	125956781	125980244	-0.458570262	PASS
HIP1	7	75533300	75738962	-0.262778904	PASS
HIP1R	12	122834453	122862960	0.202141228	PASS
HIPK1	1	113929192	113977869	0.033763446	PASS
HIPK2	7	139561570	139777778	-0.388946609	PASS
HIPK3	11	33256672	33357023	-0.405444717	PASS
HIPK4	19	40379271	40390187	0.093487731	suppress_segment_too_large
HIRA	22	19330698	19447450	-0.575930959	PASS
HIRIP3	16	29992321	29996436	0.069936844	PASS
HIST1H1A	6	26017085	26017732	0.048820648	PASS
HIST1H1B	6	27866849	27867529	0.048820648	PASS
HIST1H1C	6	26055787	26056428	0.048820648	PASS
HIST1H1D	6	26234268	26234933	0.048820648	PASS
HIST1H1E	6	26156354	26157107	0.048820648	PASS
HIST1H1T	6	26107419	26108136	0.048820648	PASS
HIST1H2A	6	25726132	25726527	0.048820648	PASS
HIST1H2A	6	26033176	26033568	0.048820648	PASS
HIST1H2A	6	26124145	26139116	0.048820648	PASS
HIST1H2A	6	26198851	26199243	0.048820648	PASS

HIST1H2A	6	26216975	26217483	0.048820648	PASS
HIST1H2A	6	27133042	27135291	0.048820648	PASS
HIST1H2A	6	27147129	27147515	0.048820648	PASS
HIST1H2A	6	27808199	27808701	0.048820648	PASS
HIST1H2A	6	27814354	27814740	0.048820648	PASS
HIST1H2A	6	27837947	27838339	0.048820648	PASS
HIST1H2A	6	27865355	27865747	0.048820648	PASS
HIST1H2A	6	27892757	27893149	0.048820648	PASS
HIST1H2B	6	25726777	25727292	0.048820648	PASS
HIST1H2B	6	26043277	26043657	0.048820648	PASS
HIST1H2B	6	26114873	26123926	0.048820648	PASS
HIST1H2B	6	26158146	26171349	0.048820648	PASS
HIST1H2B	6	26172059	26184655	0.048820648	PASS
HIST1H2B	6	26199520	26200715	0.048820648	PASS
HIST1H2B	6	26215159	26216692	0.048820648	PASS
HIST1H2B	6	26251651	26253710	0.048820648	PASS
HIST1H2B	6	26272976	26273356	0.048820648	PASS
HIST1H2B	6	27125897	27132750	0.048820648	PASS
HIST1H2B	6	27146418	27146798	0.048820648	PASS
HIST1H2B	6	27807444	27807931	0.048820648	PASS
HIST1H2B	6	27815044	27815424	0.048820648	PASS
HIST1H2B	6	27837760	27865798	0.048820648	PASS
HIST1H2B	6	27893463	27893843	0.048820648	PASS
HIST1H3A	6	26020490	26020900	0.048820648	PASS
HIST1H3B	6	26031650	26032060	0.048820648	PASS
HIST1H3C	6	26045411	26045821	0.048820648	PASS
HIST1H3D	6	26196840	26197250	0.048820648	PASS
HIST1H3E	6	26224199	26227473	0.048820648	PASS
HIST1H3F	6	26250195	26250605	0.048820648	PASS
HIST1H3G	6	26269405	26271815	0.048820648	PASS
HIST1H3H	6	27810064	27811300	0.048820648	PASS
HIST1H3I	6	27871905	27872315	0.048820648	PASS
HIST1H3J	6	27890382	27893106	0.048820648	PASS
HIST1H4A	6	26021679	26021990	0.048820648	PASS
HIST1H4B	6	26026815	26027252	0.048820648	PASS
HIST1H4C	6	26103876	26104310	0.048820648	PASS
HIST1H4D	6	26188765	26189076	0.048820648	PASS
HIST1H4E	6	26204552	26206038	0.048820648	PASS
HIST1H4F	6	26240426	26240737	0.048820648	PASS
HIST1H4G	6	26246681	26246977	0.048820648	PASS
HIST1H4H	6	26277609	26285638	0.048820648	PASS
HIST1H4I	6	27138588	27139881	0.048820648	PASS
HIST1H4J	6	27824108	27824480	0.048820648	PASS
HIST1H4K	6	27831216	27831527	0.048820648	PASS
HIST1H4L	6	27873199	27873510	0.048820648	PASS
HIST2H2A	1	149887524	149887916	0.103015788	PASS

HIST2H2A	1	149886975	149887364	0.103015788	PASS
HIST2H2B	1	149884459	149886652	0.103015788	PASS
HIST2H2B	1	149782689	149812373	-0.157385092	PASS
HIST2H3D	1	149813271	149813681	0.103015788	PASS
HIST2H3P	1	143905556	143905966	-0.157385092	PASS
HIST3H2A	1	228456979	228457873	-0.256698369	PASS
HIST3H2B	1	228458107	228460470	-0.256698369	PASS
HIST3H3	1	228424845	228425325	-0.256698369	PASS
HIST4H4	12	14767999	14771131	0.362961408	suppress_segment_too_large
HIVEP1	6	12008762	12164999	0.048820648	PASS
HIVEP2	6	142751467	142956698	-0.035448111	PASS
HIVEP3	1	41506365	42035925	0.033763446	PASS
HJURP	2	233833416	233854566	0.025928102	PASS
HJV	1	146017468	146036746	-0.157385092	PASS
HK1	10	69269984	69401884	0.072405807	PASS
HK2	2	74833981	74893359	-0.372959126	PASS
HK3	5	176880869	176899332	-0.150979337	PASS
HKDC1	10	69220303	69267559	0.072405807	PASS
HKR1	19	37312837	37369365	0.093487731	suppress_segment_too_large
HLA-A	6	29941260	29945884	0.048820648	PASS
HLA-B	6	31269491	31357188	0.048820648	PASS
HLA-C	6	31268749	31272130	0.048820648	PASS
HLA-DMA	6	32948613	32969094	0.048820648	PASS
HLA-DMB	6	32934629	32941070	0.048820648	PASS
HLA-DOA	6	33004178	33009612	0.048820648	PASS
HLA-DOB	6	32812763	32820466	0.048820648	PASS
HLA-DPA1	6	33064569	33080775	0.048820648	PASS
HLA-DPB1	6	33075926	33087201	0.048820648	PASS
HLA-DQA	6	32628179	32647062	0.048820648	PASS
HLA-DQA	6	32741342	32747215	0.048820648	PASS
HLA-DQB	6	32659467	32668383	0.048820648	PASS
HLA-DQB2	6	32756098	32763534	0.048820648	PASS
HLA-DRA	6	32439842	32445046	0.048820648	PASS
HLA-DRB	6	32578769	32589848	0.048820648	PASS
HLA-DRB3	6	32517343	32530287	0.048820648	PASS
HLA-E	6	30489467	30494205	0.048820648	PASS
HLA-F	6	29722775	29738528	0.048820648	PASS
HLA-G	6	29826967	29831125	0.048820648	PASS
HLCS	21	36750888	36990236	-0.064425456	suppress_segment_too_large
HLF	17	55265012	55325065	0.088516311	PASS
HLTF	3	149030127	149086554	0.318278996	suppress_segment_too_large
HLX	1	220879400	220885059	-0.256698369	PASS
HM13	20	31514410	31577923	-0.168668583	PASS
HMBOX1	8	28890394	29064764	-0.345962679	PASS
HMBS	11	119084866	119093549	-0.356188624	PASS
HMCES	3	129278828	129306186	0.313454204	suppress_segment_too_large

HMCN1	1	185734551	186190949	-0.027073548	PASS
HMCN2	9	130265882	130434123	0.365190713	suppress_segment_too_large
HMG20A	15	77420412	77485607	-0.697600509	PASS
HMG20B	19	3572777	3579088	0.079632875	PASS
HMGA1	6	34236873	34246231	0.048820648	PASS
HMGA2	12	65824131	65966295	-0.202191512	PASS
HMGB1	13	30456704	30617597	-0.390786075	PASS
HMGB2	4	173331695	173335125	-0.011382299	PASS
HMGB3	23	150980509	150990775	-0.012567603	PASS
HMGB4	1	33860475	33864791	0.033763446	PASS
HMGCL	1	23801885	23838620	0.033763446	PASS
HMGCLL1	6	55434369	55579214	0.048820648	PASS
HMGCR	5	75336329	75362104	-0.293443042	PASS
HMGCS1	5	43289395	43313512	-0.293443042	PASS
HMGCS2	1	119747996	119768905	-0.157385092	PASS
HMGN1	21	39342315	39349647	-0.064425456	suppress_segment_too_large
HMGN2	1	26472450	26475972	0.033763446	PASS
HMGN3	6	79201245	79234738	-0.368590549	PASS
HMGN4	6	26538405	26546254	0.048820648	PASS
HMGN5	23	81113701	81201942	-0.012567603	PASS
HMGXB3	5	150000046	150053142	-0.293443042	PASS
HMGXB4	22	35257452	35295807	-0.575930959	PASS
HMHB1	5	143812161	143820719	-0.293443042	PASS
HMMR	5	163460203	163491945	-0.150979337	PASS
HMOX1	22	35380361	35394214	-0.575930959	PASS
HMOX2	16	4474690	4510347	0.069936844	PASS
HMSD	18	63949301	63981774	-0.376397843	PASS
HMX1	4	8846076	8871817	0.279089364	PASS
HMX2	10	123148122	123150672	0.063874692	PASS
HMX3	10	123135962	123137741	0.063874692	PASS
HNF1A	12	120978543	121002512	0.202141228	PASS
HNF1B	17	37686432	37745247	0.060555011	PASS
HNF4A	20	44355700	44434596	-0.168668583	PASS
HNF4G	8	75407914	75566843	-0.201142258	PASS
HNMT	2	137964020	138016364	-0.475215623	PASS
HNRNPA0	5	137745651	137754376	-0.293443042	PASS
HNRNPA1	12	54280193	54287088	-0.202191512	PASS
HNRNPA1	13	52642425	52643796	-0.390786075	PASS
HNRNPA1	16	51553436	51647132	-0.371357732	PASS
HNRNPA2	7	26173057	26201529	-0.69513118	PASS
HNRNPA3	2	177212563	177223958	0.039726562	PASS
HNRNPAE	5	178204507	178211163	-0.150979337	PASS
HNRNPC	14	21209136	21269494	0.060212819	PASS
HNRNPCL	1	12847408	12848725	-0.415253068	PASS
HNRNPCL	1	13115496	13116854	-0.415253068	PASS
HNRNPCL	1	13060869	13062229	-0.415253068	PASS

HNRNPD	4	82352498	82374503	-0.011382299	PASS
HNRNPDL	4	82422564	82430408	-0.011382299	PASS
HNRNPF	10	43385617	43409166	-0.404852538	PASS
HNRNPH1	5	179614178	179634784	-0.150979337	PASS
HNRNPH2	23	101408295	101414133	-0.012567603	PASS
HNRNPH3	10	68331174	68343191	0.072405807	PASS
HNRNPK	9	83968083	83980616	0.365190713	suppress_segment_too_large
HNRNPL	19	38836388	38852347	0.093487731	suppress_segment_too_large
HNRNPLL	2	38561978	38603586	0.040498426	PASS
HNRNPM	19	8444767	8489114	0.079632875	PASS
HNRNPR	1	23303771	23344336	0.033763446	PASS
HNRNPU	1	244840638	244864560	-0.368111568	PASS
HNRNPUL	19	41262496	41307598	0.093487731	suppress_segment_too_large
HNRNPUL	11	62712630	62727472	0.182439925	suppress_segment_too_large
HNRNPUL	11	62690275	62727384	0.182439925	suppress_segment_too_large
HOGA1	10	97584323	97612802	-0.367947924	PASS
HOMER1	5	79372636	79514217	-0.293443042	PASS
HOMER2	15	82836946	82986153	-0.697600509	PASS
HOMER3	19	18929201	18941261	0.093487731	suppress_segment_too_large
HOMEZ	14	23272422	23299447	0.060212819	PASS
HOOK1	1	59814786	59876378	0.033763446	PASS
HOOK2	19	12763003	12872740	0.093487731	suppress_segment_too_large
HOOK3	8	42896932	43030539	-0.201142258	PASS
HOPX	4	56647988	56681899	-0.011382299	PASS
HORMAD	1	150698060	150720888	0.103015788	PASS
HORMAD:	22	30080174	30177075	-0.575930959	PASS
HOXA1	7	27092993	27095996	-0.69513118	PASS
HOXA10	7	27170591	27180261	-0.69513118	PASS
HOXA11	7	27181510	27185223	-0.69513118	PASS
HOXA13	7	27193503	27200106	-0.69513118	PASS
HOXA2	7	27100354	27102811	-0.69513118	PASS
HOXA3	7	27106184	27152581	-0.69513118	PASS
HOXA4	7	27128507	27130799	-0.69513118	PASS
HOXA5	7	27141052	27143668	-0.69513118	PASS
HOXA6	7	27145396	27150603	-0.69513118	PASS
HOXA7	7	27153716	27157936	-0.69513118	PASS
HOXA9	7	27162435	27175180	-0.69513118	PASS
HOXB1	17	48528526	48531001	0.088516311	PASS
HOXB13	17	48724763	48729178	0.088516311	PASS
HOXB2	17	48540894	48544989	0.088516311	PASS
HOXB3	17	48548870	48604912	0.088516311	PASS
HOXB4	17	48575513	48580111	0.088516311	PASS
HOXB5	17	48591257	48593961	0.088516311	PASS
HOXB6	17	48595751	48604992	0.088516311	PASS
HOXB7	17	48607227	48633572	0.088516311	PASS
HOXB8	17	48611377	48614939	0.088516311	PASS

HOXB9	17	48621159	48626356	0.088516311	PASS
HOXC10	12	53985065	53990279	-0.202191512	PASS
HOXC11	12	53973126	53977643	-0.202191512	PASS
HOXC12	12	53954834	53958956	-0.202191512	PASS
HOXC13	12	53938765	53946544	-0.202191512	PASS
HOXC4	12	54016931	54056030	-0.202191512	PASS
HOXC5	12	54032853	54035358	-0.202191512	PASS
HOXC6	12	53990624	54030823	-0.202191512	PASS
HOXC8	12	54009106	54012362	-0.202191512	PASS
HOXC9	12	53994895	54003337	-0.202191512	PASS
HOXD1	2	176188579	176190907	0.039726562	PASS
HOXD10	2	176108790	176119942	0.039726562	PASS
HOXD11	2	176104216	176109754	0.039726562	PASS
HOXD12	2	176099730	176101193	0.039726562	PASS
HOXD13	2	176092891	176095938	0.039726562	PASS
HOXD3	2	176136612	176173102	0.039726562	PASS
HOXD4	2	176151222	176153226	0.039726562	PASS
HOXD8	2	176129694	176132695	0.039726562	PASS
HOXD9	2	176122720	176124937	0.039726562	PASS
HP	16	72054592	72061055	-0.371357732	PASS
HP1BP3	1	20742661	20787323	0.033763446	PASS
HPCA	1	32885994	32898441	0.033763446	PASS
HPCAL1	2	10302889	10427617	0.040498426	PASS
HPCAL4	1	39678648	39691689	0.033763446	PASS
HPD	12	121839527	121863596	0.202141228	PASS
HPDL	1	45326905	45328533	0.033763446	PASS
HPF1	4	169729465	169757953	-0.011382299	PASS
HPGD	4	174490177	174523154	-0.011382299	PASS
HPGDS	4	94298535	94342876	-0.011382299	PASS
HPN	19	35040506	35066571	0.093487731	suppress_segment_too_large
HPR	16	72063148	72077246	-0.371357732	PASS
HPRT1	23	134460153	134520513	-0.012567603	PASS
HPS1	10	98416198	98446947	-0.367947924	PASS
HPS3	3	149129584	149173732	0.318278996	suppress_segment_too_large
HPS4	22	26443423	26483837	-0.575930959	PASS
HPS5	11	18278668	18322198	-0.405444717	PASS
HPS6	10	102065390	102068038	-0.367947924	PASS
HPSE	4	83292461	83335153	-0.011382299	PASS
HPSE2	10	98457077	99235862	-0.367947924	PASS
HPX	11	6431049	6442617	-0.405444717	PASS
HR	8	22114415	22133384	-0.345962679	PASS
HRAS	11	532242	537287	-0.333835828	PASS
HRASLS	3	193241125	193277738	-0.627320105	PASS
HRASLS2	11	63552770	63563383	0.182439925	suppress_segment_too_large
HRASLS5	11	63461404	63491194	0.182439925	suppress_segment_too_large
HRC	19	49151198	49155424	0.14996833	PASS

HRCT1	9	35906192	35907141	-0.310444659	PASS
HRG	3	186660216	186678240	0.318278996	suppress_segment_too_large
HRH1	3	11137093	11263557	0.311018244	suppress_segment_too_large
HRH2	5	175658030	175710756	-0.150979337	PASS
HRH3	20	62214970	62220267	-0.168668583	PASS
HRH4	18	24460629	24479957	-0.376397843	PASS
HRK	12	116856144	116881441	0.202141228	PASS
HRNR	1	152212082	152224193	0.103015788	PASS
HS1BP3	2	20560448	20651089	0.040498426	PASS
HS2ST1	1	86914648	87109998	0.033763446	PASS
HS3ST1	4	11393150	11429765	0.279089364	PASS
HS3ST2	16	22814177	22916338	0.069936844	PASS
HS3ST3A1	17	13495689	13601927	-0.139031004	PASS
HS3ST3B1	17	14301083	14349404	-0.139031004	PASS
HS3ST4	16	25692026	26137688	0.069936844	PASS
HS3ST5	6	114055586	114343045	-0.368590549	PASS
HS3ST6	16	1911463	1918440	0.069936844	PASS
HS6ST1	2	128236716	128318577	-0.475215623	PASS
HS6ST2	23	132626016	132961395	-0.012567603	PASS
HS6ST3	13	96090839	96839562	-0.390786075	PASS
HSBP1	16	83807843	83819737	-0.371357732	PASS
HSBP1L1	18	79964561	79970822	-0.376397843	PASS
HSCB	22	28742031	28757515	-0.575930959	PASS
HSD11B1	1	209686178	209734950	-0.256698369	PASS
HSD11B1I	19	5680604	5688523	0.079632875	PASS
HSD11B2	16	67430652	67437553	-0.371357732	PASS
HSD17B1	17	42549214	42555213	0.088516311	PASS
HSD17B1(	23	53431258	53434373	-0.012567603	PASS
HSD17B11	4	87336610	87391386	-0.011382299	PASS
HSD17B12	11	43680558	43856617	0.117376172	PASS
HSD17B13	4	87303789	87322906	-0.011382299	PASS
HSD17B14	19	48813017	48836678	0.14996833	PASS
HSD17B2	16	82035004	82098534	-0.371357732	PASS
HSD17B3	9	96235306	96302176	0.365190713	suppress_segment_too_large
HSD17B4	5	119452443	119637199	-0.293443042	PASS
HSD17B6	12	56752161	56787790	-0.202191512	PASS
HSD17B7	1	162790702	162812817	-0.027073548	PASS
HSD17B8	6	33204642	33206831	0.048820648	PASS
HSD3B1	1	119507198	119515054	-0.157385092	PASS
HSD3B2	1	119414931	119423035	-0.157385092	PASS
HSD3B7	16	30985207	30989152	0.069936844	PASS
HSDL1	16	84122146	84145192	-0.371357732	PASS
HSDL2	9	112379937	112472410	0.365190713	suppress_segment_too_large
HSF1	8	144291591	144314722	0.307788765	PASS
HSF2	6	122399546	122433119	-0.368590549	PASS
HSF2BP	21	43529192	43659493	-0.064425456	suppress_segment_too_large

HSF4	16	67164681	67169945	-0.371357732	PASS
HSF5	17	58420167	58488384	0.088516311	PASS
HSFX1	23	149774068	149776867	-0.012567603	PASS
HSFX3	23	149548210	149549932	-0.012567603	PASS
HSFX4	23	149929645	149931287	-0.012567603	PASS
HSH2D	19	16134028	16158575	0.093487731	suppress_segment_too_large
HSP90AA1	14	102080738	102139699	-0.192197564	PASS
HSP90AB1	6	44246166	44253888	0.048820648	PASS
HSP90B1	12	103930107	103953645	0.202141228	PASS
HSPA12A	10	116671192	116850236	0.063874692	PASS
HSPA12B	20	3732667	3753111	-0.168668583	PASS
HSPA13	21	14371115	14383484	-0.304975024	PASS
HSPA14	10	14838160	14871741	0.039811807	PASS
HSPA1A	6	31815464	31817946	0.048820648	PASS
HSPA1B	6	31827735	31830255	0.048820648	PASS
HSPA1L	6	31809619	31815065	0.048820648	PASS
HSPA2	14	64535905	64546173	0.060212819	PASS
HSPA4	5	133051962	133106449	-0.293443042	PASS
HSPA4L	4	127781821	127840733	-0.011382299	PASS
HSPA5	9	125234853	125241330	0.365190713	suppress_segment_too_large
HSPA6	1	161524540	161526910	-0.027073548	PASS
HSPA8	11	123057489	123063230	-0.356188624	PASS
HSPA9	5	138553756	138575724	-0.293443042	PASS
HSPB1	7	76302544	76304295	-0.262778904	PASS
HSPB11	1	53916574	53945929	0.033763446	PASS
HSPB2	11	111912242	111914093	-0.356188624	PASS
HSPB2-C1	11	111912736	111926871	-0.356188624	PASS
HSPB3	5	54455601	54456384	-0.293443042	PASS
HSPB6	19	35754569	35758079	0.093487731	suppress_segment_too_large
HSPB7	1	16014028	16019594	0.033763446	PASS
HSPB8	12	119178642	119221131	0.202141228	PASS
HSPB9	17	42121431	42123352	0.060555011	PASS
HSPBAP1	3	122740003	122793824	0.313454204	suppress_segment_too_large
HSPBP1	19	55262231	55280381	0.121336758	PASS
HSPD1	2	197486581	197516737	0.025928102	PASS
HSPE1	2	197499994	197503457	0.025928102	PASS
HSPE1-MC	2	197500413	197550726	0.025928102	PASS
HSPG2	1	21822245	21937297	0.033763446	PASS
HSPH1	13	31134974	31162388	-0.390786075	PASS
HTATIP2	11	20363685	20383783	-0.405444717	PASS
HTATSF1	23	136497079	136512346	-0.012567603	PASS
HTD2	3	58306262	58318574	-0.234617007	PASS
HTN1	4	70050402	70058845	-0.011382299	PASS
HTN3	4	70028413	70036538	-0.011382299	PASS
HTR1A	5	63960356	63962507	-0.293443042	PASS
HTR1B	6	77461753	77463773	-0.368590549	PASS

HTR1D	1	23191895	23194729	0.033763446	PASS
HTR1E	6	86937306	87016683	-0.368590549	PASS
HTR1F	3	87990696	87993835	0.313454204	suppress_segment_too_large
HTR2A	13	46831550	46897076	-0.390786075	PASS
HTR2B	2	231108230	231125118	0.025928102	PASS
HTR2C	23	114584078	114910061	-0.012567603	PASS
HTR3A	11	113974881	113990313	-0.356188624	PASS
HTR3B	11	113904677	113946565	-0.356188624	PASS
HTR3C	3	184053047	184060671	0.318278996	suppress_segment_too_large
HTR3D	3	184031544	184039369	0.318278996	suppress_segment_too_large
HTR3E	3	184097064	184106995	0.318278996	suppress_segment_too_large
HTR4	5	148451032	148677235	-0.293443042	PASS
HTR5A	7	155070324	155087392	-0.411415677	PASS
HTR6	1	19665287	19679562	0.033763446	PASS
HTR7	10	90740823	90857698	-0.39678585	PASS
HTRA1	10	122458551	122514908	0.063874692	PASS
HTRA2	2	74529377	74533348	-0.372959126	PASS
HTRA3	4	8269765	8307111	0.279089364	PASS
HTRA4	8	38974164	38988662	-0.201142258	PASS
HTT	4	3041422	3243960	0.279089364	PASS
HUNK	21	31873315	32044633	0.12860881	suppress_segment_too_large
HUS1	7	47963288	47979553	-0.69513118	PASS
HUS1B	6	655939	656963	0.048820648	PASS
HUWE1	23	53532096	53686729	-0.012567603	PASS
HVCN1	12	110627841	110704950	0.202141228	PASS
HYAL1	3	50299889	50312381	-0.234617007	PASS
HYAL2	3	50317790	50322906	-0.234617007	PASS
HYAL3	3	50292831	50299468	-0.234617007	PASS
HYAL4	7	123828983	123877478	-0.388946609	PASS
HYDIN	16	70807378	71230722	-0.371357732	PASS
HYI	1	43451003	43453989	0.033763446	PASS
HYKK	15	78507564	78537372	-0.697600509	PASS
HYLS1	11	125883614	125900648	-0.419612865	PASS
HYOU1	11	119044189	119057202	-0.356188624	PASS
HYPK	15	43796142	43803043	-0.396255771	PASS
HYPM	23	37990817	37991317	-0.012567603	PASS
IAH1	2	9473658	9496543	0.040498426	PASS
IAPP	12	21354959	21379978	0.362961408	suppress_segment_too_large
IARS	9	92210207	92293756	0.365190713	suppress_segment_too_large
IARS2	1	220094102	220148041	-0.256698369	PASS
IBA57	1	228165815	228182257	-0.256698369	PASS
IBSP	4	87799581	87812435	-0.011382299	PASS
IBTK	6	82169983	82247754	-0.368590549	PASS
ICA1	7	8113184	8262687	-0.69513118	PASS
ICA1L	2	202773150	202871985	0.025928102	PASS
ICAM1	19	10270835	10286615	0.093487731	suppress_segment_too_large

ICAM2	17	64002594	64020634	0.088516311	PASS
ICAM3	19	10333776	10339823	0.093487731	suppress_segment_too_large
ICAM4	19	10286967	10288522	0.093487731	suppress_segment_too_large
ICAM5	19	10289981	10296778	0.093487731	suppress_segment_too_large
ICE1	5	5420664	5490234	0.055007813	PASS
ICE2	15	60419609	60479160	-0.697600509	PASS
ICK	6	53001279	53061802	0.048820648	PASS
ICMT	1	6221193	6235972	0.373964873	suppress_segment_too_large
ICOS	2	203936748	203961577	0.025928102	PASS
ID1	20	31605283	31606515	-0.168668583	PASS
ID2	2	8678845	8684453	0.040498426	PASS
ID3	1	23557918	23559794	0.033763446	PASS
ID4	6	19837386	19840684	0.048820648	PASS
IDE	10	92451684	92574076	-0.39678585	PASS
IDH1	2	208236227	208266074	0.025928102	PASS
IDH2	15	90083045	90102504	-0.697600509	PASS
IDH3A	15	78131498	78171949	-0.697600509	PASS
IDH3B	20	2658395	2664219	-0.168668583	PASS
IDH3G	23	153785766	153794523	-0.012567603	PASS
IDI1	10	1039152	1049170	0.039811807	PASS
IDI2	10	1018907	1025859	0.039811807	PASS
IDNK	9	83623049	83644130	0.365190713	suppress_segment_too_large
IDO1	8	39902275	39928444	-0.201142258	PASS
IDO2	8	39934614	40016391	-0.201142258	PASS
IDS	23	149476990	149521096	-0.012567603	PASS
IDUA	4	986997	1004506	0.279089364	PASS
IER2	19	13150415	13154908	0.093487731	suppress_segment_too_large
IER3	6	30743199	30744554	0.048820648	PASS
IER3IP1	18	47152834	47176374	-0.376397843	PASS
IER5	1	181088712	181092899	-0.027073548	PASS
IER5L	9	129175552	129178262	0.365190713	suppress_segment_too_large
IFFO1	12	6538375	6556083	-0.086128028	PASS
IFFO2	1	18904281	18956686	0.033763446	PASS
IFI16	1	158999968	159055155	-0.467855874	PASS
IFI27	14	94104836	94116698	-0.192197564	PASS
IFI27L1	14	94081282	94103846	-0.192197564	PASS
IFI27L2	14	94127779	94130253	-0.192197564	PASS
IFI30	19	18173162	18178117	0.093487731	suppress_segment_too_large
IFI35	17	43006725	43014456	0.088516311	PASS
IFI44	1	78649796	78664078	0.033763446	PASS
IFI44L	1	78619922	78646145	0.033763446	PASS
IFI6	1	27666061	27672218	0.033763446	PASS
IFIH1	2	162267079	162318703	0.039726562	PASS
IFIT1	10	89392546	89406486	-0.39678585	PASS
IFIT1B	10	89378056	89385205	-0.39678585	PASS
IFIT2	10	89283694	89309276	-0.39678585	PASS

IFIT3	10	89327894	89340971	-0.39678585	PASS
IFIT5	10	89414586	89421001	-0.39678585	PASS
IFITM1	11	313506	315272	-0.333835828	PASS
IFITM10	11	1732410	1750591	-0.333835828	PASS
IFITM2	11	307631	315272	-0.333835828	PASS
IFITM3	11	319669	327537	-0.333835828	PASS
IFITM5	11	298200	299526	-0.333835828	PASS
IFNA1	9	21440454	21441316	-0.310444659	PASS
IFNA10	9	21206181	21207143	-0.310444659	PASS
IFNA13	9	21367424	21368962	-0.310444659	PASS
IFNA14	9	21239002	21239991	-0.310444659	PASS
IFNA16	9	21216373	21217311	-0.310444659	PASS
IFNA17	9	21227243	21228222	-0.310444659	PASS
IFNA2	9	21384254	21385388	-0.310444659	PASS
IFNA21	9	21165637	21166660	-0.310444659	PASS
IFNA4	9	21186694	21187671	-0.310444659	PASS
IFNA5	9	21304326	21305312	-0.310444659	PASS
IFNA6	9	21349835	21351378	-0.310444659	PASS
IFNA7	9	21201469	21202205	-0.310444659	PASS
IFNA8	9	21409147	21410185	-0.310444659	PASS
IFNAR1	21	33324477	33359862	0.12860881	suppress_segment_too_large
IFNAR2	21	33229901	33265675	0.12860881	suppress_segment_too_large
IFNB1	9	21077105	21077963	-0.310444659	PASS
IFNE	9	21480839	21482313	-0.310444659	PASS
IFNG	12	68154768	68159747	-0.202191512	PASS
IFNGR1	6	137197484	137219449	-0.035448111	PASS
IFNGR2	21	33402896	33479348	0.12860881	suppress_segment_too_large
IFNK	9	27524314	27526498	-0.310444659	PASS
IFNL1	19	39296325	39298673	0.093487731	suppress_segment_too_large
IFNL2	19	39268514	39270092	0.093487731	suppress_segment_too_large
IFNL3	19	39243553	39245129	0.093487731	suppress_segment_too_large
IFNLR1	1	24154157	24187959	0.033763446	PASS
IFNW1	9	21140214	21142145	-0.310444659	PASS
IFRD1	7	112422968	112481017	-0.388946609	PASS
IFRD2	3	50287732	50292918	-0.234617007	PASS
IFT122	3	129440036	129520507	0.313454204	suppress_segment_too_large
IFT140	16	1510427	1612110	0.069936844	PASS
IFT172	2	27444371	27489789	0.040498426	PASS
IFT20	17	28328325	28335489	0.060555011	PASS
IFT22	7	101313367	101321823	-0.388946609	PASS
IFT27	22	36758202	36776256	-0.575930959	PASS
IFT43	14	75902136	76084585	-0.192197564	PASS
IFT46	11	118544528	118572970	-0.356188624	PASS
IFT52	20	43590931	43647296	-0.168668583	PASS
IFT57	3	108160812	108222570	0.313454204	suppress_segment_too_large
IFT74	9	26947039	27062930	-0.310444659	PASS

IFT80	3	160256986	160399880	0.318278996	suppress_segment_too_large
IFT81	12	110124335	110218797	0.202141228	PASS
IFT88	13	20567069	20691437	-0.390786075	PASS
IGBP1	23	70133449	70166324	-0.012567603	PASS
IGDCC3	15	65327127	65378040	-0.697600509	PASS
IGDCC4	15	65381464	65423072	-0.697600509	PASS
IGF1	12	102395874	102481744	0.202141228	PASS
IGF1R	15	98648539	98964530	-0.697600509	PASS
IGF2	11	2129112	2158658	-0.333835828	PASS
IGF2BP1	17	48997412	49055650	0.088516311	PASS
IGF2BP2	3	185643739	185825056	0.318278996	suppress_segment_too_large
IGF2BP3	7	23310209	23470467	-0.69513118	PASS
IGF2R	6	159969099	160113507	-0.035448111	PASS
IGFALS	16	1790413	1794971	0.069936844	PASS
IGFBP1	7	45888357	45893668	-0.69513118	PASS
IGFBP2	2	216632828	216664436	0.025928102	PASS
IGFBP3	7	45912245	45921874	-0.69513118	PASS
IGFBP4	17	40443461	40457731	0.060555011	PASS
IGFBP5	2	216672105	216695525	0.025928102	PASS
IGFBP6	12	53097436	53102345	-0.202191512	PASS
IGFBP7	4	57030773	57110385	-0.011382299	PASS
IGFBPL1	9	38408994	38424447	-0.310444659	PASS
IGFL1	19	46229752	46231243	0.14996833	PASS
IGFL2	19	46143106	46161299	0.14996833	PASS
IGFL3	19	46120071	46124674	0.14996833	PASS
IGFL4	19	46039748	46077118	0.14996833	PASS
IGFLR1	19	35738801	35742453	0.093487731	suppress_segment_too_large
IGFN1	1	201190825	201228952	-0.256698369	PASS
IGHMBP2	11	68903842	68940602	0.182439925	suppress_segment_too_large
IGIP	5	140125935	140129392	-0.293443042	PASS
IGLL1	22	23573125	23580308	-0.575930959	PASS
IGLL5	22	22887780	22896107	-0.575930959	PASS
IGLON5	19	51311848	51330354	-0.016761391	PASS
IGSF1	23	131273506	131578899	-0.012567603	PASS
IGSF10	3	151425384	151458709	0.318278996	suppress_segment_too_large
IGSF11	3	118900557	119146068	0.313454204	suppress_segment_too_large
IGSF21	1	18107746	18378483	0.033763446	PASS
IGSF22	11	18704305	18726230	-0.405444717	PASS
IGSF23	19	44613630	44636781	0.14996833	PASS
IGSF3	1	116574399	116667755	0.033763446	PASS
IGSF5	21	39745407	39802096	-0.064425456	suppress_segment_too_large
IGSF6	16	21639537	21652660	0.069936844	PASS
IGSF8	1	160091340	160098943	-0.027073548	PASS
IGSF9	1	159927039	159945604	-0.027073548	PASS
IGSF9B	11	133908564	133956985	-0.419612865	PASS
IHH	2	219054420	219060467	0.025928102	PASS

IK	5	140647058	140662479	-0.293443042	PASS
IKBIP	12	98613405	98645113	0.202141228	PASS
IKBKB	8	42271302	42332653	-0.201142258	PASS
IKBKE	1	206470476	206496889	-0.256698369	PASS
IKBKG	23	154541199	154565046	-0.012567603	PASS
IKZF1	7	50304068	50405101	-0.750061359	PASS
IKZF2	2	212999691	213152427	0.025928102	PASS
IKZF3	17	39757715	39864188	0.060555011	PASS
IKZF4	12	56007659	56038435	-0.202191512	PASS
IKZF5	10	122990806	123008817	0.063874692	PASS
IL10	1	206767602	206772494	-0.256698369	PASS
IL10RA	11	117986348	118003037	-0.356188624	PASS
IL10RB	21	33266358	33310187	0.12860881	suppress_segment_too_large
IL11	19	55364389	55370463	0.121336758	PASS
IL11RA	9	34650702	34661892	-0.310444659	PASS
IL12A	3	159988750	159996019	0.318278996	suppress_segment_too_large
IL12B	5	159314783	159330887	-0.150979337	PASS
IL12RB1	19	18058995	18098944	0.093487731	suppress_segment_too_large
IL12RB2	1	67307364	67397090	0.033763446	PASS
IL13	5	132656263	132661110	-0.293443042	PASS
IL13RA1	23	118727572	118794539	-0.012567603	PASS
IL13RA2	23	115003975	115019977	-0.012567603	PASS
IL15	4	141636599	141733987	-0.011382299	PASS
IL15RA	10	5943639	5978187	0.039811807	PASS
IL16	15	81159575	81314058	-0.697600509	PASS
IL17A	6	52186375	52190638	0.048820648	PASS
IL17B	5	149371324	149404202	-0.293443042	PASS
IL17C	16	88638591	88640473	-0.371357732	PASS
IL17D	13	20702127	20723098	-0.390786075	PASS
IL17F	6	52236681	52244537	0.048820648	PASS
IL17RA	22	17084954	17115694	-0.575930959	PASS
IL17RB	3	53846580	53865800	-0.234617007	PASS
IL17RC	3	9917074	9933630	0.311018244	suppress_segment_too_large
IL17RD	3	57089982	57170306	-0.234617007	PASS
IL17RE	3	9902612	9916402	0.311018244	suppress_segment_too_large
IL17REL	22	49994513	50012659	-0.808211634	PASS
IL18	11	112143251	112164117	-0.356188624	PASS
IL18BP	11	71998541	72005715	0.062969153	PASS
IL18R1	2	102311529	102398775	0.048238121	PASS
IL18RAP	2	102418689	102452565	0.048238121	PASS
IL19	1	206798870	206842981	-0.256698369	PASS
IL1A	2	112773915	112784590	-0.010901494	PASS
IL1B	2	112829751	112836903	-0.010901494	PASS
IL1F10	2	113067970	113075850	-0.010901494	PASS
IL1R1	2	102064544	102179874	0.048238121	PASS
IL1R2	2	101991844	102028544	0.048238121	PASS

IL1RAP	3	190514051	190659750	0.318278996	suppress_segment_too_large
IL1RAPL1	23	28587399	29956723	-0.012567603	PASS
IL1RAPL2	23	104566315	105767829	-0.012567603	PASS
IL1RL1	2	102311502	102352037	0.048238121	PASS
IL1RL2	2	102186973	102240002	0.048238121	PASS
IL1RN	2	113107214	113134016	-0.010901494	PASS
IL2	4	122451470	122456725	-0.011382299	PASS
IL20	1	206865354	206869223	-0.256698369	PASS
IL20RA	6	136999971	137045180	-0.035448111	PASS
IL20RB	3	136946230	137011085	0.313454204	suppress_segment_too_large
IL21	4	122610108	122621069	-0.011382299	PASS
IL21R	16	27402162	27452042	0.069936844	PASS
IL22	12	68248242	68253604	-0.202191512	PASS
IL22RA1	1	24119771	24143121	0.033763446	PASS
IL22RA2	6	137143820	137173648	-0.035448111	PASS
IL23A	12	56334174	56340410	-0.202191512	PASS
IL23R	1	67138907	67259979	0.033763446	PASS
IL24	1	206897443	206904139	-0.256698369	PASS
IL25	14	23372809	23376403	0.060212819	PASS
IL26	12	68201351	68225821	-0.202191512	PASS
IL27	16	28499362	28512051	0.069936844	PASS
IL27RA	19	14031748	14053216	0.093487731	suppress_segment_too_large
IL2RA	10	6010689	6062370	0.039811807	PASS
IL2RB	22	37125838	37175054	-0.575930959	PASS
IL2RG	23	71107404	71112108	-0.012567603	PASS
IL3	5	132060529	132063204	-0.293443042	PASS
IL31	12	122172030	122174199	0.202141228	PASS
IL31RA	5	55851379	55922853	-0.293443042	PASS
IL32	16	3065297	3082192	0.069936844	PASS
IL33	9	6215786	6257983	-0.310444659	PASS
IL34	16	70579895	70660682	-0.371357732	PASS
IL36A	2	113005461	113008044	-0.010901494	PASS
IL36B	2	113022091	113052867	-0.010901494	PASS
IL36G	2	112973203	112985665	-0.010901494	PASS
IL36RN	2	113058638	113065382	-0.010901494	PASS
IL37	2	112912971	112918882	-0.010901494	PASS
IL3RA	23	1336616	1382689	-0.012567603	PASS
IL4	5	132673986	132682676	-0.293443042	PASS
IL4I1	19	49889654	49929539	-0.016761391	PASS
IL4R	16	27313668	27364778	0.069936844	PASS
IL5	5	132541444	132556838	-0.293443042	PASS
IL5RA	3	3066326	3126613	0.311018244	suppress_segment_too_large
IL6	7	22725884	22732002	-0.69513118	PASS
IL6R	1	154405193	154469450	0.103015788	PASS
IL6ST	5	55935095	55994993	-0.293443042	PASS
IL7	8	78675743	78805523	0.279445683	PASS

IL7R	5	35852695	35879603	0.055007813	PASS
IL9	5	135892246	135895827	-0.293443042	PASS
IL9R	23	155997581	156010817	-0.012567603	PASS
ILDR1	3	121987323	122022258	0.313454204	suppress_segment_too_large
ILDR2	1	166895711	166975482	-0.027073548	PASS
ILF2	1	153661788	153671048	0.103015788	PASS
ILF3	19	10654261	10692417	0.093487731	suppress_segment_too_large
ILK	11	6603708	6610874	-0.405444717	PASS
ILKAP	2	238170401	238203729	0.025928102	PASS
ILVBL	19	15114984	15125785	0.093487731	suppress_segment_too_large
IMMP1L	11	31432401	31509645	-0.405444717	PASS
IMMP2L	7	110663051	111562517	-0.388946609	PASS
IMMT	2	86143932	86195770	0.066915641	PASS
IMP3	15	75639085	75648706	-0.697600509	PASS
IMP4	2	130342225	130347810	-0.475215623	PASS
IMPA1	8	81657961	81686693	0.279445683	PASS
IMPA2	18	11981025	12030883	-0.052305056	suppress_segment_too_large
IMPACT	18	24426616	24453535	-0.376397843	PASS
IMPAD1	8	56957933	56993844	-0.201142258	PASS
IMPDH1	7	128392277	128410252	-0.388946609	PASS
IMPDH2	3	49024325	49029408	-0.234617007	PASS
IMPG1	6	75921115	76072678	-0.368590549	PASS
IMPG2	3	101222546	101320560	0.313454204	suppress_segment_too_large
INA	10	103277163	103290351	-0.367947924	PASS
INAFM1	19	47274453	47275707	0.14996833	PASS
INAFM2	15	40323664	40326715	-0.396255771	PASS
INAVA	1	200891048	200915735	-0.256698369	PASS
INCA1	17	4988130	4997610	0.128406487	PASS
INCENP	11	62123973	62153163	0.182439925	suppress_segment_too_large
INF2	14	104689606	104722535	-0.192197564	PASS
ING1	13	110712736	110723339	-0.390786075	PASS
ING2	4	183504994	183511096	-0.462744716	PASS
ING3	7	120950749	120977216	-0.388946609	PASS
ING4	12	6650280	6663148	-0.086128028	PASS
INHA	2	219569162	219575713	0.025928102	PASS
INHBA	7	41667168	41705834	-0.69513118	PASS
INHBB	2	120346143	120351808	-0.475215623	PASS
INHBC	12	57434760	57450828	-0.202191512	PASS
INHBE	12	57452323	57459280	-0.202191512	PASS
INIP	9	112683926	112718236	0.365190713	suppress_segment_too_large
INKA1	3	49803254	49805030	-0.234617007	PASS
INKA2	1	111680630	111739424	0.033763446	PASS
INMT	7	30697985	30757602	-0.69513118	PASS
INMT-MIN	7	30752137	30892081	-0.69513118	PASS
INO80	15	40978880	41116354	-0.396255771	PASS
INO80B	2	74455023	74457960	-0.372959126	PASS

INO80B-W	2	74455088	74460884	-0.372959126	PASS
INO80C	18	35452230	35497991	-0.376397843	PASS
INO80D	2	205993721	206086303	0.025928102	PASS
INO80E	16	29995294	30005793	0.069936844	PASS
INPP1	2	190343470	190371665	0.025928102	PASS
INPP4A	2	98444854	98594390	0.048238121	PASS
INPP4B	4	142023160	142847432	-0.011382299	PASS
INPP5A	10	132537820	132783480	0.063874692	PASS
INPP5B	1	37860697	37947057	0.033763446	PASS
INPP5D	2	233059967	233207903	0.025928102	PASS
INPP5E	9	136428619	136439823	0.365190713	suppress_segment_too_large
INPP5F	10	119726042	119829278	0.063874692	PASS
INPP5J	22	31122731	31134696	-0.575930959	PASS
INPP5K	17	1494571	1516888	0.128406487	PASS
INPPL1	11	72223701	72239105	0.062969153	PASS
INS	11	2159779	2161341	-0.333835828	PASS
INS-IGF2	11	2132538	2161209	-0.333835828	PASS
INSC	11	15112424	15247208	-0.405444717	PASS
INSIG1	7	155297776	155310235	-0.411415677	PASS
INSIG2	2	118088452	118110997	-0.475215623	PASS
INSL3	19	17816512	17821574	0.093487731	suppress_segment_too_large
INSL4	9	5231419	5235304	-0.310444659	PASS
INSL5	1	66797741	66801256	0.033763446	PASS
INSL6	9	5123880	5185668	-0.310444659	PASS
INSM1	20	20368121	20370949	-0.168668583	PASS
INSM2	14	35534042	35537054	0.060212819	PASS
INSR	19	7112255	7294034	0.079632875	PASS
INSRR	1	156840063	156859018	0.103015788	PASS
INSYN1	15	73735431	73752747	-0.697600509	PASS
INSYN2	10	127135426	127196158	0.063874692	PASS
INSYN2B	5	169864264	169980740	-0.150979337	PASS
INTS1	7	1470277	1504367	-0.69513118	PASS
INTS10	8	19817140	19852083	-0.345962679	PASS
INTS11	1	1311585	1324691	0.373964873	suppress_segment_too_large
INTS12	4	105682627	105895986	-0.011382299	PASS
INTS13	12	26905181	26938326	0.362961408	suppress_segment_too_large
INTS14	15	65578753	65611289	-0.697600509	PASS
INTS2	17	61865367	61928016	0.088516311	PASS
INTS3	1	153728067	153774808	0.103015788	PASS
INTS4	11	77878720	77994678	0.062969153	PASS
INTS5	11	62646848	62653302	0.182439925	suppress_segment_too_large
INTS6	13	51354077	51454264	-0.390786075	PASS
INTS6L	23	135520643	135582510	-0.012567603	PASS
INTS7	1	211940399	212035542	-0.256698369	PASS
INTS8	8	94813311	94881746	0.279445683	PASS
INTS9	8	28767661	28890242	-0.345962679	PASS

INTU	4	127623271	127726737	-0.011382299	PASS
INVS	9	100099256	100301000	0.365190713	suppress_segment_too_large
IP6K1	3	49724294	49786542	-0.234617007	PASS
IP6K2	3	48688003	48740353	-0.234617007	PASS
IP6K3	6	33721670	33746905	0.048820648	PASS
IPCEF1	6	154154496	154356792	-0.035448111	PASS
IPMK	10	58191517	58267934	0.072405807	PASS
IPO11	5	62403972	62628582	-0.293443042	PASS
IPO13	1	43946939	43968022	0.033763446	PASS
IPO4	14	24180219	24188964	0.060212819	PASS
IPO5	13	97953658	98024297	-0.390786075	PASS
IPO7	11	9384622	9448126	-0.405444717	PASS
IPO8	12	30628988	30695986	0.362961408	suppress_segment_too_large
IPO9	1	201829141	201884294	-0.256698369	PASS
IPP	1	45694324	45750650	0.033763446	PASS
IPPK	9	92613184	92670265	0.365190713	suppress_segment_too_large
IQANK1	8	143734140	143790644	0.307788765	PASS
IQCA1	2	236324147	236507542	0.025928102	PASS
IQCA1L	7	151190873	151205496	-0.388946609	PASS
IQCB1	3	121769763	121835079	0.313454204	suppress_segment_too_large
IQCC	1	32205661	32208687	0.033763446	PASS
IQCD	12	113195441	113221094	0.202141228	PASS
IQCE	7	2558972	2614734	-0.69513118	PASS
IQCF1	3	51894876	51903335	-0.234617007	PASS
IQCF2	3	51861629	51863424	-0.234617007	PASS
IQCF3	3	51826883	51830856	-0.234617007	PASS
IQCF5	3	51873721	51875584	-0.234617007	PASS
IQCF6	3	51778561	51779241	-0.234617007	PASS
IQCG	3	197889075	197960142	0.326834516	PASS
IQCH	15	67254800	67502260	-0.697600509	PASS
IQCJ	3	158962928	159266307	0.318278996	suppress_segment_too_large
IQCJ-SCHI	3	158962235	159897366	0.318278996	suppress_segment_too_large
IQCK	16	19716456	19858467	0.069936844	PASS
IQCM	4	149351903	149896233	-0.011382299	PASS
IQCN	19	18257097	18274509	0.093487731	suppress_segment_too_large
IQGAP1	15	90388218	90502243	-0.697600509	PASS
IQGAP2	5	76403249	76708132	-0.293443042	PASS
IQGAP3	1	156525405	156572604	0.103015788	PASS
IQSEC1	3	12897220	13283281	0.311018244	suppress_segment_too_large
IQSEC2	23	53225828	53321350	-0.012567603	PASS
IQSEC3	12	66765	178460	-0.086128028	PASS
IQUB	7	123452400	123535077	-0.388946609	PASS
IRAK1	23	154010500	154019980	-0.012567603	PASS
IRAK1BP1	6	78867472	78946440	-0.368590549	PASS
IRAK2	3	10164865	10243743	0.311018244	suppress_segment_too_large
IRAK3	12	66188879	66254622	-0.202191512	PASS

IRAK4	12	43758944	43789543	-0.202191512	PASS
IREB2	15	78437431	78501456	-0.697600509	PASS
IRF1	5	132481609	132490798	-0.293443042	PASS
IRF2	4	184387713	184474580	-0.462744716	PASS
IRF2BP1	19	45883607	45886170	0.14996833	PASS
IRF2BP2	1	234604269	234609525	-0.256698369	PASS
IRF2BPL	14	77024543	77028699	-0.192197564	PASS
IRF3	19	49659569	49665875	0.14996833	PASS
IRF4	6	391739	411447	0.048820648	PASS
IRF5	7	128937612	128950035	-0.388946609	PASS
IRF6	1	209785623	209806175	-0.256698369	PASS
IRF7	11	612553	615999	-0.333835828	PASS
IRF8	16	85898803	85922609	-0.371357732	PASS
IRF9	14	24161053	24166565	0.060212819	PASS
IRGC	19	43716010	43720021	0.14996833	PASS
IRGM	5	150846523	150900736	-0.293443042	PASS
IRGQ	19	43584369	43596135	0.14996833	PASS
IRS1	2	226731317	226799759	0.025928102	PASS
IRS2	13	109752698	109786568	-0.390786075	PASS
IRS4	23	108732482	108736409	-0.012567603	PASS
IRX1	5	3596054	3601403	0.055007813	PASS
IRX2	5	2745845	2751662	0.055007813	PASS
IRX3	16	54283304	54286763	-0.371357732	PASS
IRX4	5	1877413	1887236	0.055007813	PASS
IRX5	16	54930862	54934485	-0.371357732	PASS
IRX6	16	55323760	55330760	-0.371357732	PASS
ISCA1	9	86264546	86283102	0.365190713	suppress_segment_too_large
ISCA2	14	74493720	74497106	-0.192197564	PASS
ISCU	12	108562582	108569384	0.202141228	PASS
ISG15	1	1001138	1014540	0.373964873	suppress_segment_too_large
ISG20	15	88636153	88656483	-0.697600509	PASS
ISG20L2	1	156721891	156728799	0.103015788	PASS
ISL1	5	51383391	51394738	-0.293443042	PASS
ISL2	15	76336724	76342476	-0.697600509	PASS
ISLR	15	74173671	74176872	-0.697600509	PASS
ISLR2	15	74100311	74138540	-0.697600509	PASS
ISM1	20	13221771	13300651	-0.168668583	PASS
ISM2	14	77474394	77498850	-0.192197564	PASS
ISOC1	5	129094751	129114028	-0.293443042	PASS
ISOC2	19	55452985	55462343	0.121336758	PASS
ISPD	7	16087527	16421322	-0.69513118	PASS
IST1	16	71845996	71931199	-0.371357732	PASS
ISX	22	35066136	35087387	-0.575930959	PASS
ISY1	3	129127415	129161293	0.313454204	suppress_segment_too_large
ISY1-RAB	3	129087575	129161036	0.313454204	suppress_segment_too_large
ISYNA1	19	18434388	18438301	0.093487731	suppress_segment_too_large

ITCH	20	34363235	34511393	-0.168668583	PASS
ITFG1	16	47154387	47464149	0.069936844	PASS
ITFG2	12	2812622	2859791	-0.086128028	PASS
ITGA1	5	52787896	52959210	-0.293443042	PASS
ITGA10	1	145891208	145910189	-0.157385092	PASS
ITGA11	15	68296533	68432162	-0.697600509	PASS
ITGA2	5	52989326	53094779	-0.293443042	PASS
ITGA2B	17	44372180	44389505	0.088516311	PASS
ITGA3	17	50055968	50090481	0.088516311	PASS
ITGA4	2	181457202	181536187	0.039726562	PASS
ITGA5	12	54395261	54419460	-0.202191512	PASS
ITGA6	2	172427354	172506282	0.039726562	PASS
ITGA7	12	55684568	55716043	-0.202191512	PASS
ITGA8	10	15513949	15720125	0.039811807	PASS
ITGA9	3	37452115	37823514	-0.234617007	PASS
ITGAD	16	31393312	31426505	0.069936844	PASS
ITGAE	17	3714628	3801243	0.128406487	PASS
ITGAL	16	30472658	30523185	0.069936844	PASS
ITGAM	16	31259967	31332892	0.069936844	PASS
ITGAV	2	186590065	186680901	-0.169541924	PASS
ITGAX	16	31355134	31382997	0.069936844	PASS
ITGB1	10	32900319	33005792	0.039811807	PASS
ITGB1BP1	2	9403475	9423547	0.040498426	PASS
ITGB1BP2	23	71301734	71305371	-0.012567603	PASS
ITGB2	21	44885953	44931989	-0.064425456	suppress_segment_too_large
ITGB3	17	47253846	47311816	0.088516311	PASS
ITGB3BP	1	63440770	63593721	0.033763446	PASS
ITGB4	17	75721328	75757818	0.088516311	PASS
ITGB5	3	124761948	124901418	0.313454204	suppress_segment_too_large
ITGB6	2	160099666	160200313	0.039726562	PASS
ITGB7	12	53191318	53207307	-0.202191512	PASS
ITGB8	7	20330702	20415754	-0.69513118	PASS
ITGBL1	13	101452593	101720856	-0.390786075	PASS
ITIH1	3	52777592	52792068	-0.234617007	PASS
ITIH2	10	7703269	7749520	0.039811807	PASS
ITIH3	3	52794768	52809009	-0.234617007	PASS
ITIH4	3	52812975	52831479	-0.234617007	PASS
ITIH5	10	7559270	7666998	0.039811807	PASS
ITIH6	23	54748899	54798240	-0.012567603	PASS
ITK	5	157142933	157255191	-0.293443042	PASS
ITLN1	1	160876539	160885170	-0.027073548	PASS
ITLN2	1	160945025	160954799	-0.027073548	PASS
ITM2A	23	79360384	79367667	-0.012567603	PASS
ITM2B	13	48232612	48270357	-0.390786075	PASS
ITM2C	2	230864639	230879248	0.025928102	PASS
ITPA	20	3208868	3223870	-0.168668583	PASS

ITPK1	14	92936914	93116320	-0.192197564	PASS
ITPKA	15	41493393	41503551	-0.396255771	PASS
ITPKB	1	226631690	226739323	-0.256698369	PASS
ITPKC	19	40717103	40740860	0.093487731	suppress_segment_too_large
ITPR1	3	4493345	4847840	0.311018244	suppress_segment_too_large
ITPR2	12	26336515	26833198	0.362961408	suppress_segment_too_large
ITPR3	6	33620365	33696574	0.048820648	PASS
ITPRID1	7	31514071	31658720	-0.69513118	PASS
ITPRID2	2	181891833	181930738	0.039726562	PASS
ITPRIP	10	104309698	104338448	-0.367947924	PASS
ITPRIPL1	2	96325331	96330517	0.048238121	PASS
ITPRIPL2	16	19113932	19121629	0.069936844	PASS
ITSN1	21	33642400	33899861	0.12860881	suppress_segment_too_large
ITSN2	2	24202864	24360714	0.040498426	PASS
IVD	15	40405795	40435947	-0.396255771	PASS
IVL	1	152908545	152911886	0.103015788	PASS
IVNS1ABI	1	185296388	185317329	-0.027073548	PASS
IWS1	2	127436207	127526886	-0.475215623	PASS
IYD	6	150368892	150405969	-0.035448111	PASS
IZUMO1	19	48740852	48746909	0.14996833	PASS
IZUMO1R	11	94305592	94307721	0.062969153	PASS
IZUMO2	19	50152548	50163195	-0.016761391	PASS
IZUMO3	9	24542952	24545946	-0.310444659	PASS
IZUMO4	19	2096429	2099593	0.079632875	PASS
JADE1	4	128809623	128875224	-0.011382299	PASS
JADE2	5	134524312	134583230	-0.293443042	PASS
JADE3	23	46912276	47061242	-0.012567603	PASS
JAG1	20	10637684	10673999	-0.168668583	PASS
JAG2	14	105140981	105168824	-0.192197564	PASS
JAGN1	3	9890572	9894349	0.311018244	suppress_segment_too_large
JAK1	1	64833229	64966504	0.033763446	PASS
JAK2	9	4984390	5128183	-0.310444659	PASS
JAK3	19	17824780	17848071	0.093487731	suppress_segment_too_large
JAKMIP1	4	6026203	6200591	0.279089364	PASS
JAKMIP2	5	147585439	147782848	-0.293443042	PASS
JAKMIP3	10	132104671	132184809	0.063874692	PASS
JAM2	21	25639272	25717562	0.12860881	suppress_segment_too_large
JAM3	11	134068925	134152001	-0.419612865	PASS
JAML	11	118193740	118225094	-0.356188624	PASS
JARID2	6	15246296	15522040	0.048820648	PASS
JAZF1	7	27830573	28180743	-0.69513118	PASS
JCAD	10	30012800	30115494	0.039811807	PASS
JCHAIN	4	70655541	70681817	-0.011382299	PASS
JDP2	14	75427716	75474111	-0.192197564	PASS
JHY	11	122882528	122959798	-0.356188624	PASS
JKAMP	14	59484443	59505410	0.060212819	PASS

JMJD1C	10	63167221	63521850	0.072405807	PASS
JMJD4	1	227730425	227735411	-0.256698369	PASS
JMJD6	17	76712832	76726799	0.088516311	PASS
JMJD7	15	41828085	41837581	-0.396255771	PASS
JMJD7-PL/	15	41828095	41848155	-0.396255771	PASS
JMJD8	16	681671	684528	0.069936844	PASS
JMY	5	79236189	79327215	-0.293443042	PASS
JOSD1	22	38685543	38701556	-0.575930959	PASS
JOSD2	19	50505998	50511353	-0.016761391	PASS
JPH1	8	74234700	74321328	-0.201142258	PASS
JPH2	20	44111695	44187578	-0.168668583	PASS
JPH3	16	87601835	87698156	-0.371357732	PASS
JPH4	14	23568035	23578800	0.060212819	PASS
JPT1	17	75135248	75168281	0.088516311	PASS
JPT2	16	1678256	1702280	0.069936844	PASS
JRK	8	142657460	142681968	0.113969953	PASS
JRKL	11	96389989	96507574	0.062969153	PASS
JSRP1	19	2252252	2269759	0.079632875	PASS
JTB	1	153974269	153977688	0.103015788	PASS
JUN	1	58780788	58784327	0.033763446	PASS
JUNB	19	12791496	12793315	0.093487731	suppress_segment_too_large
JUND	19	18279760	18281622	0.093487731	suppress_segment_too_large
JUP	17	41754604	41786931	0.060555011	PASS
KAAG1	6	24356903	24358284	0.048820648	PASS
KALRN	3	124080023	124726325	0.313454204	suppress_segment_too_large
KANK1	9	470291	746106	-0.310444659	PASS
KANK2	19	11164267	11197791	0.093487731	suppress_segment_too_large
KANK3	19	8322584	8343262	0.079632875	PASS
KANK4	1	62236979	62319414	0.033763446	PASS
KANSL1	17	46029916	46225389	0.088516311	PASS
KANSL1L	2	210021423	210171383	0.025928102	PASS
KANSL2	12	48653401	48682238	-0.202191512	PASS
KANSL3	2	96593170	96642787	0.048238121	PASS
KANTR	23	53094145	53170914	-0.012567603	PASS
KARS	16	75627474	75648643	-0.371357732	PASS
KAT14	20	18138118	18188387	-0.168668583	PASS
KAT2A	17	42113108	42121358	0.060555011	PASS
KAT2B	3	20040023	20154404	0.311018244	suppress_segment_too_large
KAT5	11	65711996	65719604	0.182439925	suppress_segment_too_large
KAT6A	8	41929479	42052026	-0.201142258	PASS
KAT6B	10	74824927	75032624	0.072405807	PASS
KAT7	17	49788555	49835030	0.088516311	PASS
KAT8	16	31115754	31131393	0.069936844	PASS
KATNA1	6	149594873	149648972	-0.035448111	PASS
KATNAL1	13	30202630	30307484	-0.390786075	PASS
KATNAL2	18	46917492	47102243	-0.376397843	PASS

KATNB1	16	57735730	57757250	-0.371357732	PASS
KATNBL1	15	34140674	34210096	-0.396255771	PASS
KAZALD1	10	101061841	101068131	-0.367947924	PASS
KAZN	1	13892792	15118043	0.033763446	PASS
KBTBD11	8	1973878	2006936	-0.313874214	PASS
KBTBD11·	8	1763888	1958627	-0.313874214	PASS
KBTBD12	3	127915232	127987671	0.313454204	suppress_segment_too_large
KBTBD13	15	65076816	65079939	-0.697600509	PASS
KBTBD2	7	32868172	32894131	-0.69513118	PASS
KBTBD3	11	106051098	106077765	-0.356188624	PASS
KBTBD4	11	47572197	47579015	0.117376172	PASS
KBTBD6	13	41127569	41132746	-0.390786075	PASS
KBTBD7	13	41189833	41194566	-0.390786075	PASS
KBTBD8	3	66998307	67011210	-0.234617007	PASS
KCMF1	2	84971093	85059472	0.066915641	PASS
KCNA1	12	4909905	4918256	-0.086128028	PASS
KCNA10	1	110517217	110519175	0.033763446	PASS
KCNA2	1	110519837	110631474	0.033763446	PASS
KCNA3	1	110672465	110675033	0.033763446	PASS
KCNA4	11	30009741	30017023	-0.405444717	PASS
KCNA5	12	5043989	5046788	-0.086128028	PASS
KCNA6	12	4809176	4813412	-0.086128028	PASS
KCNA7	19	49067418	49072941	0.14996833	PASS
KCNAB1	3	156037701	156539138	0.318278996	suppress_segment_too_large
KCNAB2	1	5991466	6101193	0.373964873	suppress_segment_too_large
KCNAB3	17	7921859	7929803	0.128406487	PASS
KCNB1	20	49293394	49484297	-0.168668583	PASS
KCNB2	8	72537391	72938349	-0.201142258	PASS
KCNC1	11	17734774	17856804	-0.405444717	PASS
KCNC2	12	75040077	75209868	0.202141228	PASS
KCNC3	19	50311937	50333515	-0.016761391	PASS
KCNC4	1	110211343	110283100	0.033763446	PASS
KCND1	23	48961378	48971569	-0.012567603	PASS
KCND2	7	120273668	120750331	-0.388946609	PASS
KCND3	1	111770662	111989155	0.033763446	PASS
KCNE1	21	34446688	34512275	0.12860881	suppress_segment_too_large
KCNE1B	21	7816675	7829926	-0.304975024	PASS
KCNE2	21	34364024	34371389	0.12860881	suppress_segment_too_large
KCNE3	11	74454841	74467729	0.062969153	PASS
KCNE4	2	223051814	223198399	0.025928102	PASS
KCNE5	23	109623700	109625172	-0.012567603	PASS
KCNF1	2	10911937	10914225	0.040498426	PASS
KCNG1	20	51003656	51023129	-0.168668583	PASS
KCNG2	18	79863668	79900184	-0.376397843	PASS
KCNG3	2	42442017	42494097	0.040498426	PASS
KCNG4	16	84218667	84239750	-0.371357732	PASS

KCNH1	1	210676823	211134180	-0.256698369	PASS
KCNH2	7	150944961	150978315	-0.388946609	PASS
KCNH3	12	49539157	49558308	-0.202191512	PASS
KCNH4	17	42156891	42181278	0.060555011	PASS
KCNH5	14	62699454	63102037	0.060212819	PASS
KCNH6	17	63523334	63548977	0.088516311	PASS
KCNH7	2	162371407	162838730	0.039726562	PASS
KCNH8	3	19148454	19535646	0.311018244	suppress_segment_too_large
KCNIP1	5	170353487	170736632	-0.150979337	PASS
KCNIP2	10	101825974	101843920	-0.367947924	PASS
KCNIP3	2	95297304	95386083	0.048238121	PASS
KCNIP4	4	20728616	21948799	0.279089364	PASS
KCNJ1	11	128836315	128867373	-0.419612865	PASS
KCNJ10	1	159998651	160070483	-0.027073548	PASS
KCNJ11	11	17385859	17389331	-0.405444717	PASS
KCNJ12	17	21376197	21419872	0.060555011	PASS
KCNJ13	2	232766464	232776568	0.025928102	PASS
KCNJ14	19	48455509	48466980	0.14996833	PASS
KCNJ15	21	38155549	38307357	-0.064425456	suppress_segment_too_large
KCNJ16	17	70053429	70135608	0.088516311	PASS
KCNJ18	17	21692523	21704612	0.060555011	PASS
KCNJ2	17	70168673	70180048	0.088516311	PASS
KCNJ3	2	154698299	154858352	-0.475215623	PASS
KCNJ4	22	38426327	38455199	-0.575930959	PASS
KCNJ5	11	128891356	128921035	-0.419612865	PASS
KCNJ6	21	37607376	38121345	-0.064425456	suppress_segment_too_large
KCNJ8	12	21764955	21775581	0.362961408	suppress_segment_too_large
KCNJ9	1	160081570	160090563	-0.027073548	PASS
KCNK1	1	233614004	233672512	-0.256698369	PASS
KCNK10	14	88180103	88326907	-0.192197564	PASS
KCNK12	2	47516581	47570939	0.040498426	PASS
KCNK13	14	90061765	90185857	-0.192197564	PASS
KCNK15	20	44745780	44752313	-0.168668583	PASS
KCNK16	6	39314698	39322968	0.048820648	PASS
KCNK17	6	39299001	39314553	0.048820648	PASS
KCNK18	10	117197489	117210299	0.063874692	PASS
KCNK2	1	215005775	215237093	-0.256698369	PASS
KCNK3	2	26692690	26733420	0.040498426	PASS
KCNK4	11	64291302	64300031	0.182439925	suppress_segment_too_large
KCNK5	6	39188973	39229450	0.048820648	PASS
KCNK6	19	38319844	38332076	0.093487731	suppress_segment_too_large
KCNK7	11	65592855	65595996	0.182439925	suppress_segment_too_large
KCNK9	8	139600838	139704109	0.113969953	PASS
KCNMA1	10	76869601	77638369	0.072405807	PASS
KCNMB1	5	170374671	170389677	-0.150979337	PASS
KCNMB2	3	178272932	178844429	0.318278996	suppress_segment_too_large

KCNMB3	3	179236691	179267002	0.318278996	suppress_segment_too_large
KCNMB4	12	70366276	70434292	-0.202191512	PASS
KCNN1	19	17951293	18000080	0.093487731	suppress_segment_too_large
KCNN2	5	114055545	114496500	-0.293443042	PASS
KCNN3	1	154697455	154870280	0.103015788	PASS
KCNN4	19	43766533	43781261	0.14996833	PASS
KCNQ1	11	2444684	2849110	-0.333835828	PASS
KCNQ2	20	63400210	63472677	-0.168668583	PASS
KCNQ3	8	132120858	132481019	0.113969953	PASS
KCNQ4	1	40783814	40840452	0.033763446	PASS
KCNQ5	6	72621792	73198851	-0.368590549	PASS
KCNRG	13	50015254	50020922	-0.390786075	PASS
KCNS1	20	45092310	45101112	-0.168668583	PASS
KCNS2	8	98427022	98432848	0.279445683	PASS
KCNS3	2	17877847	18361616	0.040498426	PASS
KCNT1	9	135702185	135795508	0.365190713	suppress_segment_too_large
KCNT2	1	196225779	196609225	-0.027073548	PASS
KCNU1	8	36784324	36936128	-0.345962679	PASS
KCNV1	8	109963645	109975847	0.279445683	PASS
KCNV2	9	2717502	2730037	-0.310444659	PASS
KCP	7	128862451	128910719	-0.388946609	PASS
KCTD1	18	26454910	26657401	-0.376397843	PASS
KCTD10	12	109448656	109477544	0.202141228	PASS
KCTD11	17	7351889	7354944	0.128406487	PASS
KCTD12	13	76880166	76886390	-0.390786075	PASS
KCTD13	16	29905012	29927035	0.069936844	PASS
KCTD14	11	78015715	78046191	0.062969153	PASS
KCTD15	19	33795933	33815763	0.093487731	suppress_segment_too_large
KCTD16	5	144170832	144485686	-0.293443042	PASS
KCTD17	22	37051736	37063390	-0.575930959	PASS
KCTD18	2	200488952	200519784	0.025928102	PASS
KCTD19	16	67289428	67326763	-0.371357732	PASS
KCTD2	17	75032575	75065889	0.088516311	PASS
KCTD20	6	36442767	36491143	0.048820648	PASS
KCTD21	11	78171249	78188822	0.062969153	PASS
KCTD3	1	215567392	215621807	-0.256698369	PASS
KCTD4	13	45192853	45194717	-0.390786075	PASS
KCTD5	16	2682475	2709030	0.069936844	PASS
KCTD6	3	58492114	58502360	-0.234617007	PASS
KCTD7	7	66628767	66649067	-0.750061359	PASS
KCTD8	4	44173909	44448807	-0.011382299	PASS
KCTD9	8	25427847	25458476	-0.345962679	PASS
KDELC1	13	102784281	102799007	-0.390786075	PASS
KDELC2	11	108472105	108498432	-0.356188624	PASS
KDELR1	19	48382570	48391553	0.14996833	PASS
KDELR2	7	6445953	6484242	-0.69513118	PASS

KDELR3	22	38468062	38483447	-0.575930959	PASS
KDF1	1	26949562	26960406	0.033763446	PASS
KDM1A	1	23019443	23083689	0.033763446	PASS
KDM1B	6	18155329	18223853	0.048820648	PASS
KDM2A	11	67119269	67258087	0.182439925	suppress_segment_too_large
KDM2B	12	121429097	121581015	0.202141228	PASS
KDM3A	2	86440647	86492716	0.066915641	PASS
KDM3B	5	138352596	138437028	-0.293443042	PASS
KDM4A	1	43650158	43705515	0.033763446	PASS
KDM4B	19	4969113	5153595	0.079632875	PASS
KDM4C	9	6720863	7175648	-0.310444659	PASS
KDM4D	11	94973681	94999518	0.062969153	PASS
KDM4E	11	95025258	95027596	0.062969153	PASS
KDM4F	11	95049422	95051338	0.062969153	PASS
KDM5A	12	280129	389454	-0.086128028	PASS
KDM5B	1	202724495	202809470	-0.256698369	PASS
KDM5C	23	53191321	53225422	-0.012567603	PASS
KDM6A	23	44873177	45112602	-0.012567603	PASS
KDM6B	17	7839904	7854796	0.128406487	PASS
KDM7A	7	140084746	140177035	-0.388946609	PASS
KDM8	16	27203495	27221768	0.069936844	PASS
KDR	4	55078259	55125595	-0.011382299	PASS
KDSR	18	63327726	63367510	-0.376397843	PASS
KEAP1	19	10486120	10503741	0.093487731	suppress_segment_too_large
KEL	7	142941114	142962681	-0.388946609	PASS
KERA	12	91050491	91057983	0.202141228	PASS
KHDC1	6	73241314	73310365	-0.368590549	PASS
KHDC1L	6	73223544	73225770	-0.368590549	PASS
KHDC3L	6	73362677	73364171	-0.368590549	PASS
KHDC4	1	155913043	155934400	0.103015788	PASS
KHDRBS1	1	32013829	32060850	0.033763446	PASS
KHDRBS2	6	61679960	62286227	0.048820648	PASS
KHDRBS3	8	135457457	135656722	0.113969953	PASS
KHK	2	27086747	27100772	0.040498426	PASS
KHNYN	14	24429286	24441834	0.060212819	PASS
KHSRP	19	6413348	6424794	0.079632875	PASS
KIAA0040	1	175156987	175192999	-0.027073548	PASS
KIAA0100	17	28614440	28645454	0.060555011	PASS
KIAA0232	4	6781375	6884170	0.279089364	PASS
KIAA0319	6	24544104	24646155	0.048820648	PASS
KIAA0319	1	35433490	35557950	0.033763446	PASS
KIAA0355	19	34254537	34355586	0.093487731	suppress_segment_too_large
KIAA0391	14	35121846	35277614	0.060212819	PASS
KIAA0408	6	127440343	127459391	-0.458570262	PASS
KIAA0513	16	85027751	85094230	-0.371357732	PASS
KIAA0556	16	27550133	27780369	0.069936844	PASS

KIAA0586	14	58427385	58551289	0.060212819	PASS
KIAA0753	17	6578148	6640927	0.128406487	PASS
KIAA0825	5	94152966	94618597	-0.293443042	PASS
KIAA0895	7	36324221	36390125	-0.69513118	PASS
KIAA0895	16	67175602	67184040	-0.371357732	PASS
KIAA0930	22	45190338	45240769	-0.808211634	PASS
KIAA1024	15	79432516	79472290	-0.697600509	PASS
KIAA1024	5	129748079	129766732	-0.293443042	PASS
KIAA1109	4	122152333	122362758	-0.011382299	PASS
KIAA1143	3	44737661	44761662	-0.234617007	PASS
KIAA1147	7	141656728	141702153	-0.388946609	PASS
KIAA1191	5	176346061	176361968	-0.150979337	PASS
KIAA1210	23	119078635	119150579	-0.012567603	PASS
KIAA1211	4	56049073	56328625	-0.011382299	PASS
KIAA1211	2	98793846	98936259	0.048238121	PASS
KIAA1217	10	23694746	24547848	0.039811807	PASS
KIAA1257	3	128909866	129002690	0.313454204	suppress_segment_too_large
KIAA1324	1	109113679	109206781	0.033763446	PASS
KIAA1324	7	86876906	87059699	-0.262778904	PASS
KIAA1328	18	36829106	37232172	-0.376397843	PASS
KIAA1522	1	32741885	32774970	0.033763446	PASS
KIAA1549	7	138831381	138981318	-0.388946609	PASS
KIAA1549	11	33376466	33674102	-0.405444717	PASS
KIAA1551	12	31959370	31993107	0.362961408	suppress_segment_too_large
KIAA1586	6	57046532	57055239	0.048820648	PASS
KIAA1614	1	180913154	180951614	-0.027073548	PASS
KIAA1671	22	24952730	25197448	-0.575930959	PASS
KIAA1755	20	38210488	38260772	-0.168668583	PASS
KIAA1841	2	61065871	61138034	-0.372959126	PASS
KIAA1958	9	112486847	112669397	0.365190713	suppress_segment_too_large
KIAA2012	2	202073289	202205203	0.025928102	PASS
KIAA2013	1	11919591	11926428	0.373964873	suppress_segment_too_large
KIAA2026	9	5881596	6007901	-0.310444659	PASS
KIDINS220	2	8721081	8837630	0.040498426	PASS
KIF11	10	92593286	92655395	-0.39678585	PASS
KIF12	9	114086126	114099291	0.365190713	suppress_segment_too_large
KIF13A	6	17759183	17987623	0.048820648	PASS
KIF13B	8	29067279	29263124	-0.345962679	PASS
KIF14	1	200551497	200620734	-0.027073548	PASS
KIF15	3	44761717	44873376	-0.234617007	PASS
KIF16B	20	16272104	16573434	-0.168668583	PASS
KIF17	1	20664014	20718017	0.033763446	PASS
KIF18A	11	28020620	28108308	-0.405444717	PASS
KIF18B	17	44924709	44947711	0.088516311	PASS
KIF19	17	74326210	74355820	0.088516311	PASS
KIF1A	2	240713761	240821036	0.025928102	PASS

KIF1B	1	10210805	10381603	0.373964873	suppress_segment_too_large
KIF1BP	10	68988721	69043544	0.072405807	PASS
KIF1C	17	4997948	5028401	0.128406487	PASS
KIF20A	5	138178719	138187715	-0.293443042	PASS
KIF20B	10	89701610	89774939	-0.39678585	PASS
KIF21A	12	39293228	39443390	-0.202191512	PASS
KIF21B	1	200969390	201023700	-0.256698369	PASS
KIF22	16	29790719	29805385	0.069936844	PASS
KIF23	15	69414246	69448427	-0.697600509	PASS
KIF24	9	34252381	34311371	-0.310444659	PASS
KIF25	6	167996241	168045089	-0.035448111	PASS
KIF26A	14	104138723	104180894	-0.192197564	PASS
KIF26B	1	245154985	245709431	-0.368111568	PASS
KIF27	9	83836698	83921465	0.365190713	suppress_segment_too_large
KIF2A	5	62306162	62537249	-0.293443042	PASS
KIF2B	17	53822901	53825213	0.088516311	PASS
KIF2C	1	44739818	44767767	0.033763446	PASS
KIF3A	5	132692628	132737638	-0.293443042	PASS
KIF3B	20	32277664	32335011	-0.168668583	PASS
KIF3C	2	25926596	25982749	0.040498426	PASS
KIF4A	23	70290090	70420832	-0.012567603	PASS
KIF4B	5	155013755	155018132	-0.293443042	PASS
KIF5A	12	57549998	57586632	-0.202191512	PASS
KIF5B	10	32009010	32056431	0.039811807	PASS
KIF5C	2	148875250	149026759	-0.475215623	PASS
KIF6	6	39329990	39725405	0.048820648	PASS
KIF7	15	89608789	89655451	-0.697600509	PASS
KIF9	3	47228026	47283451	-0.234617007	PASS
KIFAP3	1	169921326	170085208	-0.027073548	PASS
KIFC1	6	33391536	33409924	0.048820648	PASS
KIFC2	8	144466043	144474202	0.307788765	PASS
KIFC3	16	57758217	57863053	-0.371357732	PASS
KIN	10	7750962	7787981	0.039811807	PASS
KIR2DL1	19	54769811	54784322	-0.146906334	PASS
KIR2DL3	19	54738515	54753052	-0.146906334	PASS
KIR2DL4	19	54803535	54814517	-0.146906334	PASS
KIR3DL1	19	54816468	54830778	-0.146906334	PASS
KIR3DL2	19	54850443	54867207	-0.146906334	PASS
KIR3DL3	19	54724479	54736536	-0.146906334	PASS
KIRREL1	1	157993273	158100262	-0.467855874	PASS
KIRREL2	19	35855861	35867109	0.093487731	suppress_segment_too_large
KIRREL3	11	126423359	127003460	-0.419612865	PASS
KISS1	1	204190341	204196486	-0.256698369	PASS
KISS1R	19	917287	921015	0.079632875	PASS
KIT	4	54657918	54740715	-0.011382299	PASS
KITLG	12	88492793	88580851	0.202141228	PASS

KIZ	20	21125983	21246622	-0.168668583	PASS
KL	13	33016433	33066145	-0.390786075	PASS
KLB	4	39406853	39451536	-0.011382299	PASS
KLC1	14	103561896	103714249	-0.192197564	PASS
KLC2	11	66257294	66267860	0.182439925	suppress_segment_too_large
KLC3	19	45333434	45351520	0.14996833	PASS
KLC4	6	43040777	43075099	0.048820648	PASS
KLF1	19	12884422	12887199	0.093487731	suppress_segment_too_large
KLF10	8	102648779	102655902	0.279445683	PASS
KLF11	2	10042849	10054836	0.040498426	PASS
KLF12	13	73686089	73995056	-0.390786075	PASS
KLF13	15	31326855	31435665	-0.396255771	PASS
KLF14	7	130731235	130734176	-0.388946609	PASS
KLF15	3	126342635	126357442	0.313454204	suppress_segment_too_large
KLF16	19	1852399	1863568	0.079632875	PASS
KLF17	1	44118850	44135140	0.033763446	PASS
KLF18	1	44137821	44141631	0.033763446	PASS
KLF2	19	16324817	16327874	0.093487731	suppress_segment_too_large
KLF3	4	38664196	38701042	-0.011382299	PASS
KLF4	9	107484852	107490482	0.365190713	suppress_segment_too_large
KLF5	13	73054976	73077542	-0.390786075	PASS
KLF6	10	3775996	3785281	0.039811807	PASS
KLF7	2	207074137	207167267	0.025928102	PASS
KLF8	23	56232421	56287889	-0.012567603	PASS
KLF9	9	70384597	70414624	0.365190713	suppress_segment_too_large
KLHDC1	14	49693105	49753152	0.060212819	PASS
KLHDC10	7	130070510	130135720	-0.388946609	PASS
KLHDC2	14	49767608	49786385	0.060212819	PASS
KLHDC3	6	43014103	43021298	0.048820648	PASS
KLHDC4	16	87696485	87765992	-0.371357732	PASS
KLHDC7A	1	18480982	18486126	0.033763446	PASS
KLHDC7B	22	50545891	50551023	-0.808211634	PASS
KLHDC8A	1	205336065	205357090	-0.256698369	PASS
KLHDC8B	3	49171611	49176486	-0.234617007	PASS
KLHDC9	1	161098361	161100346	-0.027073548	PASS
KLHL1	13	69700594	70108493	-0.390786075	PASS
KLHL10	17	41835685	41848384	0.060555011	PASS
KLHL11	17	41853545	41865431	0.060555011	PASS
KLHL12	1	202891100	202928636	-0.256698369	PASS
KLHL13	23	117897813	118117340	-0.012567603	PASS
KLHL14	18	32672671	32773062	-0.376397843	PASS
KLHL15	23	23983720	24027186	-0.012567603	PASS
KLHL17	1	960587	965715	0.373964873	suppress_segment_too_large
KLHL18	3	47282917	47346816	-0.234617007	PASS
KLHL2	4	165207618	165323156	-0.011382299	PASS
KLHL20	1	173714941	173786702	-0.027073548	PASS

KLHL21	1	6590724	6614607	0.373964873	suppress_segment_too_large
KLHL22	22	20441519	20495883	-0.575930959	PASS
KLHL23	2	169694488	169776989	0.039726562	PASS
KLHL24	3	183635568	183684477	0.318278996	suppress_segment_too_large
KLHL25	15	85759323	85795030	-0.697600509	PASS
KLHL26	19	18636965	18671714	0.093487731	suppress_segment_too_large
KLHL28	14	44924319	45042322	0.060212819	PASS
KLHL29	2	23385217	23708611	0.040498426	PASS
KLHL3	5	137617500	137736090	-0.293443042	PASS
KLHL30	2	238138722	238152947	0.025928102	PASS
KLHL31	6	53647901	53665708	0.048820648	PASS
KLHL32	6	96924620	97140754	-0.368590549	PASS
KLHL33	14	20425852	20436158	0.060212819	PASS
KLHL34	23	21654690	21658330	-0.012567603	PASS
KLHL35	11	75422394	75430629	0.062969153	PASS
KLHL36	16	84648525	84667686	-0.371357732	PASS
KLHL38	8	123645527	123652950	0.279445683	PASS
KLHL4	23	87517749	87670050	-0.012567603	PASS
KLHL40	3	42685519	42692544	-0.234617007	PASS
KLHL41	2	169509702	169526262	0.039726562	PASS
KLHL42	12	27780020	27803040	0.362961408	suppress_segment_too_large
KLHL5	4	39045039	39126857	-0.011382299	PASS
KLHL6	3	183487531	183555689	0.318278996	suppress_segment_too_large
KLHL7	7	23105758	23177914	-0.69513118	PASS
KLHL8	4	87160103	87240314	-0.011382299	PASS
KLHL9	9	21329671	21335380	-0.310444659	PASS
KLK1	19	50819148	50823787	-0.016761391	PASS
KLK10	19	51012739	51020175	-0.016761391	PASS
KLK11	19	51022216	51028039	-0.016761391	PASS
KLK12	19	51029092	51035230	-0.016761391	PASS
KLK13	19	51056206	51065114	-0.016761391	PASS
KLK14	19	51077495	51084245	-0.016761391	PASS
KLK15	19	50825289	50837213	-0.016761391	PASS
KLK2	19	50861568	50880567	-0.016761391	PASS
KLK3	19	50854915	50860764	-0.016761391	PASS
KLK4	19	50906352	50910738	-0.016761391	PASS
KLK5	19	50943303	50953093	-0.016761391	PASS
KLK6	19	50958631	50969673	-0.016761391	PASS
KLK7	19	50976482	50984099	-0.016761391	PASS
KLK8	19	50996007	51002711	-0.016761391	PASS
KLK9	19	51003111	51009634	-0.016761391	PASS
KLKB1	4	186208979	186258471	-0.462744716	PASS
KLLN	10	87859161	87863437	-1.64589449	suppress_large_homdel
KLRB1	12	9594551	9607886	0.362961408	suppress_segment_too_large
KLRC1	12	10442264	10454685	0.362961408	suppress_segment_too_large
KLRC2	12	10426854	10442300	0.362961408	suppress_segment_too_large

KLRC3	12	10412312	10420595	0.362961408	suppress_segment_too_large
KLRC4	12	10407382	10409757	0.362961408	suppress_segment_too_large
KLRC4-KI	12	10372353	10410146	0.362961408	suppress_segment_too_large
KLRD1	12	10226058	10329600	0.362961408	suppress_segment_too_large
KLRF1	12	9827481	9845007	0.362961408	suppress_segment_too_large
KLRF2	12	9881489	9895833	0.362961408	suppress_segment_too_large
KLRG1	12	8950044	9010760	0.362961408	suppress_segment_too_large
KLRG2	7	139452690	139483712	-0.388946609	PASS
KLRK1	12	10372353	10391874	0.362961408	suppress_segment_too_large
KMO	1	241532134	241595642	-0.648598641	PASS
KMT2A	11	118436464	118526832	-0.356188624	PASS
KMT2B	19	35718019	35738878	0.093487731	suppress_segment_too_large
KMT2C	7	152134922	152436005	-0.388946609	PASS
KMT2D	12	49018975	49059774	-0.202191512	PASS
KMT2E	7	105014179	105114361	-0.388946609	PASS
KMT5A	12	123383773	123409358	0.202141228	PASS
KMT5B	11	68154863	68213828	0.182439925	suppress_segment_too_large
KMT5C	19	55339853	55348120	0.121336758	PASS
KNCN	1	46545644	46551527	0.033763446	PASS
KNDC1	10	133160447	133226412	0.063874692	PASS
KNG1	3	186717276	186744410	0.318278996	suppress_segment_too_large
KNL1	15	40594020	40664342	-0.396255771	PASS
KNOP1	16	19701934	19718235	0.069936844	PASS
KNSTRN	15	40382721	40394246	-0.396255771	PASS
KNTC1	12	122527246	122626396	0.202141228	PASS
KPNA1	3	122421949	122514945	0.313454204	suppress_segment_too_large
KPNA2	17	68035519	68046842	0.088516311	PASS
KPNA3	13	49699307	49792921	-0.390786075	PASS
KPNA4	3	160494995	160565588	0.318278996	suppress_segment_too_large
KPNA5	6	116681187	116741866	-0.368590549	PASS
KPNA6	1	32108038	32176568	0.033763446	PASS
KPNA7	7	99173574	99207506	-0.262778904	PASS
KPNB1	17	47649476	47685505	0.088516311	PASS
KPRP	1	152759561	152762052	0.103015788	PASS
KPTN	19	47475144	47484268	0.14996833	PASS
KRAS	12	25204789	25250936	0.362961408	suppress_segment_too_large
KRBA1	7	149714781	149734575	-0.388946609	PASS
KRBA2	17	8356902	8376711	0.128406487	PASS
KRBOX1	3	42809483	42942792	-0.234617007	PASS
KRBOX4	23	46446857	46497422	-0.012567603	PASS
KRCC1	2	88027205	88055729	-0.340781246	PASS
KREMEN1	22	29073078	29168333	-0.575930959	PASS
KREMEN2	16	2963944	2968383	0.069936844	PASS
KRI1	19	10553078	10566037	0.093487731	suppress_segment_too_large
KRIT1	7	92198969	92246166	-0.262778904	PASS
KRR1	12	75490861	75511636	0.202141228	PASS

KRT1	12	52674736	52680407		-0.202191512	PASS
KRT10	17	40818117	40822595		0.060555011	PASS
KRT12	17	40860943	40867224		0.060555011	PASS
KRT13	17	41500981	41505705		0.060555011	PASS
KRT14	17	41582279	41586921		0.060555011	PASS
KRT15	17	41513743	41522529		0.060555011	PASS
KRT16	17	41609778	41615899		0.060555011	PASS
KRT17	17	41619441	41624842		0.060555011	PASS
KRT18	12	52948871	52952901		-0.202191512	PASS
KRT19	17	41523617	41528308		0.060555011	PASS
KRT2	12	52644558	52652164		-0.202191512	PASS
KRT20	17	40875941	40885227		0.060555011	PASS
KRT222	17	40654665	40665181		0.060555011	PASS
KRT23	17	40922696	40937634		0.060555011	PASS
KRT24	17	40697991	40703750		0.060555011	PASS
KRT25	17	40748021	40755332		0.060555011	PASS
KRT26	17	40766238	40772162		0.060555011	PASS
KRT27	17	40776808	40782534		0.060555011	PASS
KRT28	17	40792203	40799959		0.060555011	PASS
KRT3	12	52789685	52796117		-0.202191512	PASS
KRT31	17	41393724	41397592		0.060555011	PASS
KRT32	17	41459811	41467429		0.060555011	PASS
KRT33A	17	41346092	41350812		0.060555011	PASS
KRT33B	17	41363494	41369800		0.060555011	PASS
KRT34	17	41377650	41382403		0.060555011	PASS
KRT35	17	41476689	41481140		0.060555011	PASS
KRT36	17	41486136	41492546		0.060555011	PASS
KRT37	17	41420753	41424523		0.060555011	PASS
KRT38	17	41436446	41440921		0.060555011	PASS
KRT39	17	40958417	40966892		0.060555011	PASS
KRT4	12	52806549	52814551		-0.202191512	PASS
KRT40	17	40977716	40987135		0.060555011	PASS
KRT5	12	52514575	52520687		-0.202191512	PASS
KRT6A	12	52487174	52493257		-0.202191512	PASS
KRT6B	12	52446651	52452126		-0.202191512	PASS
KRT6C	12	52468516	52473785		-0.202191512	PASS
KRT7	12	52232520	52252186		-0.202191512	PASS
KRT71	12	52543909	52553147		-0.202191512	PASS
KRT72	12	52585589	52601538		-0.202191512	PASS
KRT73	12	52607570	52618559		-0.202191512	PASS
KRT74	12	52565782	52573825		-0.202191512	PASS
KRT75	12	52424070	52434525		-0.202191512	PASS
KRT76	12	52768155	52777345		-0.202191512	PASS
KRT77	12	52689626	52703463		-0.202191512	PASS
KRT78	12	52837804	52849092		-0.202191512	PASS
KRT79	12	52821410	52834295		-0.202191512	PASS

KRT8	12	52897187	52949954	-0.202191512	PASS
KRT80	12	52168996	52192000	-0.202191512	PASS
KRT81	12	52285913	52291534	-0.202191512	PASS
KRT82	12	52393931	52406355	-0.202191512	PASS
KRT83	12	52314301	52321398	-0.202191512	PASS
KRT84	12	52377812	52385652	-0.202191512	PASS
KRT85	12	52360006	52367481	-0.202191512	PASS
KRT86	12	52249300	52309163	-0.202191512	PASS
KRT9	17	41565841	41572058	0.060555011	PASS
KRTAP1-1	17	41040559	41041461	0.060555011	PASS
KRTAP1-3	17	41033884	41034855	0.060555011	PASS
KRTAP1-4	17	41029697	41030104	0.060555011	PASS
KRTAP1-5	17	41026026	41027202	0.060555011	PASS
KRTAP10-	21	44538981	44540195	-0.064425456	suppress_segment_too_large
KRTAP10-	21	44637356	44638455	-0.064425456	suppress_segment_too_large
KRTAP10-	21	44646414	44647650	-0.064425456	suppress_segment_too_large
KRTAP10-	21	44697172	44698044	-0.064425456	suppress_segment_too_large
KRTAP10-	21	44550357	44551505	-0.064425456	suppress_segment_too_large
KRTAP10-	21	44557790	44558760	-0.064425456	suppress_segment_too_large
KRTAP10-	21	44573724	44638284	-0.064425456	suppress_segment_too_large
KRTAP10-	21	44579455	44580604	-0.064425456	suppress_segment_too_large
KRTAP10-	21	44591268	44592505	-0.064425456	suppress_segment_too_large
KRTAP10-	21	44600597	44602174	-0.064425456	suppress_segment_too_large
KRTAP10-	21	44612079	44612954	-0.064425456	suppress_segment_too_large
KRTAP10-	21	44627123	44628293	-0.064425456	suppress_segment_too_large
KRTAP11-	21	30880644	30881555	0.12860881	suppress_segment_too_large
KRTAP12-	21	44681576	44682163	-0.064425456	suppress_segment_too_large
KRTAP12-	21	44666189	44666927	-0.064425456	suppress_segment_too_large
KRTAP12-	21	44657932	44658341	-0.064425456	suppress_segment_too_large
KRTAP12-	21	44654213	44654659	-0.064425456	suppress_segment_too_large
KRTAP13-	21	30396074	30396822	0.12860881	suppress_segment_too_large
KRTAP13-	21	30371391	30372257	0.12860881	suppress_segment_too_large
KRTAP13-	21	30425265	30425968	0.12860881	suppress_segment_too_large
KRTAP13-	21	30430230	30431026	0.12860881	suppress_segment_too_large
KRTAP15-	21	30440275	30440945	0.12860881	suppress_segment_too_large
KRTAP16-	17	41307700	41309253	0.060555011	PASS
KRTAP17-	17	41314917	41315695	0.060555011	PASS
KRTAP19-	21	30479699	30480344	0.12860881	suppress_segment_too_large
KRTAP19-	21	30487057	30487436	0.12860881	suppress_segment_too_large
KRTAP19-	21	30491464	30491985	0.12860881	suppress_segment_too_large
KRTAP19-	21	30496824	30497133	0.12860881	suppress_segment_too_large
KRTAP19-	21	30501657	30502117	0.12860881	suppress_segment_too_large
KRTAP19-	21	30541535	30541864	0.12860881	suppress_segment_too_large
KRTAP19-	21	30560875	30561314	0.12860881	suppress_segment_too_large
KRTAP19-	21	31038159	31038476	0.12860881	suppress_segment_too_large
KRTAP2-1	17	41046541	41047316	0.060555011	PASS

KRTAP2-2	17	41054498	41055230	0.060555011	PASS
KRTAP2-3	17	41059240	41060114	0.060555011	PASS
KRTAP2-4	17	41065116	41065879	0.060555011	PASS
KRTAP20-	21	30616425	30616699	0.12860881	suppress_segment_too_large
KRTAP20-	21	30635236	30635619	0.12860881	suppress_segment_too_large
KRTAP20-	21	30642864	30643136	0.12860881	suppress_segment_too_large
KRTAP20-	21	30620627	30620850	0.12860881	suppress_segment_too_large
KRTAP21-	21	30754830	30755428	0.12860881	suppress_segment_too_large
KRTAP21-	21	30746794	30747233	0.12860881	suppress_segment_too_large
KRTAP21-	21	30718525	30718777	0.12860881	suppress_segment_too_large
KRTAP22-	21	30601087	30601382	0.12860881	suppress_segment_too_large
KRTAP24-	21	30281309	30282958	0.12860881	suppress_segment_too_large
KRTAP25-	21	30289145	30289514	0.12860881	suppress_segment_too_large
KRTAP26-	21	30319124	30320316	0.12860881	suppress_segment_too_large
KRTAP27-	21	30337013	30337694	0.12860881	suppress_segment_too_large
KRTAP29-	17	41301826	41302851	0.060555011	PASS
KRTAP3-1	17	41008521	41019324	0.060555011	PASS
KRTAP3-2	17	40999193	40999894	0.060555011	PASS
KRTAP3-3	17	40993430	40994133	0.060555011	PASS
KRTAP4-1	17	41184102	41185342	0.060555011	PASS
KRTAP4-1	17	41117181	41118360	0.060555011	PASS
KRTAP4-1	17	41123091	41124167	0.060555011	PASS
KRTAP4-1	17	41101502	41102209	0.060555011	PASS
KRTAP4-2	17	41177446	41178208	0.060555011	PASS
KRTAP4-3	17	41167231	41168194	0.060555011	PASS
KRTAP4-4	17	41159651	41160731	0.060555011	PASS
KRTAP4-5	17	41148924	41149802	0.060555011	PASS
KRTAP4-6	17	41139433	41140487	0.060555011	PASS
KRTAP4-7	17	41084150	41085141	0.060555011	PASS
KRTAP4-8	17	41096981	41098141	0.060555011	PASS
KRTAP4-9	17	41105332	41106488	0.060555011	PASS
KRTAP5-1	11	1584342	1585283	-0.333835828	PASS
KRTAP5-1	11	71565563	71566738	0.062969153	PASS
KRTAP5-1	11	71579714	71603353	0.062969153	PASS
KRTAP5-2	11	1597177	1598294	-0.333835828	PASS
KRTAP5-3	11	1607565	1608463	-0.333835828	PASS
KRTAP5-4	11	1620958	1622138	-0.333835828	PASS
KRTAP5-5	11	1629775	1630707	-0.333835828	PASS
KRTAP5-6	11	1697195	1697755	-0.333835828	PASS
KRTAP5-7	11	71527267	71528674	0.062969153	PASS
KRTAP5-8	11	71538025	71539207	0.062969153	PASS
KRTAP5-9	11	71548418	71549553	0.062969153	PASS
KRTAP6-1	21	30613431	30613930	0.12860881	suppress_segment_too_large
KRTAP6-2	21	30598590	30598902	0.12860881	suppress_segment_too_large
KRTAP6-3	21	30592440	30593075	0.12860881	suppress_segment_too_large
KRTAP7-1	21	30829039	30829759	0.12860881	suppress_segment_too_large

KRTAP8-1	21	30812697	30813252	0.12860881	suppress_segment_too_large
KRTAP9-1	17	41189887	41190639	0.060555011	PASS
KRTAP9-2	17	41226648	41227652	0.060555011	PASS
KRTAP9-3	17	41232463	41233454	0.060555011	PASS
KRTAP9-4	17	41249687	41250653	0.060555011	PASS
KRTAP9-6	17	41265339	41266641	0.060555011	PASS
KRTAP9-7	17	41275659	41276697	0.060555011	PASS
KRTAP9-8	17	41238045	41239004	0.060555011	PASS
KRTAP9-9	17	41255384	41256364	0.060555011	PASS
KRTCAP2	1	155169408	155173475	0.103015788	PASS
KRTCAP3	2	27442366	27446481	0.040498426	PASS
KRTDAP	19	35487324	35495558	0.093487731	suppress_segment_too_large
KSR1	17	27456470	27626438	0.060555011	PASS
KSR2	12	117453012	117968983	0.202141228	PASS
KTI12	1	52032103	52033816	0.033763446	PASS
KTN1	14	55559072	55701526	0.060212819	PASS
KXD1	19	18557762	18569387	0.093487731	suppress_segment_too_large
KY	3	134603138	134651636	0.313454204	suppress_segment_too_large
KYAT1	9	128832942	128882494	0.365190713	suppress_segment_too_large
KYAT3	1	88935773	88992953	0.033763446	PASS
KYNU	2	142877498	143055832	-0.475215623	PASS
L1CAM	23	153861514	153886174	-0.012567603	PASS
L1TD1	1	62194831	62212328	0.033763446	PASS
L2HGDH	14	50237563	50312548	0.060212819	PASS
L34079.1	19	43554692	43593036	0.14996833	PASS
L3HYPDH	14	59460363	59484430	0.060212819	PASS
L3MBTL1	20	43489442	43550950	-0.168668583	PASS
L3MBTL2	22	41205205	41231271	-0.575930959	PASS
L3MBTL3	6	130013699	130141451	-0.458570262	PASS
L3MBTL4	18	5954706	6415237	-0.052305056	suppress_segment_too_large
LACC1	13	43879284	43893932	-0.390786075	PASS
LACRT	12	54630811	54634895	-0.202191512	PASS
LACTB	15	63121800	63142061	-0.697600509	PASS
LACTB2	8	70635318	70669174	-0.201142258	PASS
LACTBL1	1	22953043	22965338	0.033763446	PASS
LAD1	1	201373244	201399915	-0.256698369	PASS
LAG3	12	6772512	6778455	-0.086128028	PASS
LAGE3	23	154477769	154479257	-0.012567603	PASS
LAIR1	19	54351384	54370558	-0.146906334	PASS
LAIR2	19	54497879	54510687	-0.146906334	PASS
LALBA	12	48567684	48570066	-0.202191512	PASS
LAMA1	18	6941744	7117814	-0.052305056	suppress_segment_too_large
LAMA2	6	128883141	129516569	-0.458570262	PASS
LAMA3	18	23689443	23956222	-0.376397843	PASS
LAMA4	6	112108760	112254939	-0.368590549	PASS
LAMA5	20	62307955	62367312	-0.168668583	PASS

LAMB1	7	107923799	108003255	-0.388946609	PASS
LAMB2	3	49121114	49133118	-0.234617007	PASS
LAMB3	1	209614870	209652466	-0.256698369	PASS
LAMB4	7	108023548	108130356	-0.388946609	PASS
LAMC1	1	183023460	183145592	-0.027073548	PASS
LAMC2	1	183186238	183244900	-0.027073548	PASS
LAMC3	9	131009082	131094473	0.365190713	suppress_segment_too_large
LAMP1	13	113297241	113323672	-0.390786075	PASS
LAMP2	23	120427827	120469365	-0.012567603	PASS
LAMP3	3	183122213	183163839	0.318278996	suppress_segment_too_large
LAMP5	20	9514358	9530524	-0.168668583	PASS
LAMTOR1	11	72085895	72103387	0.062969153	PASS
LAMTOR2	1	156054752	156058510	0.103015788	PASS
LAMTOR3	4	99878336	99894490	-0.011382299	PASS
LAMTOR4	7	100148907	100155944	-0.262778904	PASS
LAMTOR5	1	110401249	110407942	0.033763446	PASS
LANCL1	2	210431249	210477652	0.025928102	PASS
LANCL2	7	55365448	55433742	-0.750061359	PASS
LANCL3	23	37571569	37684463	-0.012567603	PASS
LAP3	4	17577192	17607972	0.279089364	PASS
LAPTM4A	2	20032650	20052028	0.040498426	PASS
LAPTM4B	8	97775057	97853013	0.279445683	PASS
LAPTM5	1	30732469	30757820	0.033763446	PASS
LARGE1	22	33162226	33922841	-0.575930959	PASS
LARGE2	11	45921621	45929096	0.117376172	PASS
LARP1	5	154712902	154817607	-0.293443042	PASS
LARP1B	4	128061268	128222931	-0.011382299	PASS
LARP4	12	50392383	50480004	-0.202191512	PASS
LARP4B	10	806914	931705	0.039811807	PASS
LARP6	15	70829130	70854159	-0.697600509	PASS
LARP7	4	112636964	112657592	-0.011382299	PASS
LARS	5	146113038	146182660	-0.293443042	PASS
LARS2	3	45388506	45549421	-0.234617007	PASS
LAS1L	23	65512582	65534775	-0.012567603	PASS
LASP1	17	38869859	38921770	0.060555011	PASS
LAT	16	28984826	28990783	0.069936844	PASS
LAT2	7	74199652	74229834	-0.262778904	PASS
LATS1	6	149658153	149718256	-0.035448111	PASS
LATS2	13	20973032	21061547	-0.390786075	PASS
LAX1	1	203765176	203776233	-0.256698369	PASS
LAYN	11	111540280	111561745	-0.356188624	PASS
LBH	2	30231369	30323730	0.040498426	PASS
LBHD1	11	62662817	62672255	0.182439925	suppress_segment_too_large
LBHD2	14	103084210	103090027	-0.192197564	PASS
LBP	20	38346356	38377023	-0.168668583	PASS
LBR	1	225401502	225428925	-0.256698369	PASS

LBX1	10	101226195	101229794	-0.367947924	PASS
LBX2	2	74497517	74503316	-0.372959126	PASS
LCA5	6	79484991	79537458	-0.368590549	PASS
LCA5L	21	39405844	39445805	-0.064425456	suppress_segment_too_large
LCAT	16	67939750	67944131	-0.371357732	PASS
LCE1A	1	152827473	152827894	0.103015788	PASS
LCE1B	1	152811971	152813109	0.103015788	PASS
LCE1C	1	152804835	152806628	0.103015788	PASS
LCE1D	1	152796751	152798181	0.103015788	PASS
LCE1E	1	152786214	152788426	0.103015788	PASS
LCE1F	1	152776372	152776728	0.103015788	PASS
LCE2A	1	152698364	152699442	0.103015788	PASS
LCE2B	1	152686123	152687401	0.103015788	PASS
LCE2C	1	152675295	152676574	0.103015788	PASS
LCE2D	1	152663396	152664659	0.103015788	PASS
LCE3A	1	152622834	152623103	0.103015788	PASS
LCE3B	1	152613811	152614098	0.103015788	PASS
LCE3C	1	152600662	152601086	0.103015788	PASS
LCE3D	1	152579381	152580504	0.103015788	PASS
LCE3E	1	152565654	152566772	0.103015788	PASS
LCE4A	1	152708160	152709491	0.103015788	PASS
LCE5A	1	152510844	152512177	0.103015788	PASS
LCE6A	1	152842868	152843983	0.103015788	PASS
LCK	1	32251239	32286165	0.033763446	PASS
LCLAT1	2	30447226	30644225	0.040498426	PASS
LCMT1	16	25111729	25178231	0.069936844	PASS
LCMT2	15	43323649	43330605	-0.396255771	PASS
LCN1	9	135521438	135526532	0.365190713	suppress_segment_too_large
LCN10	9	136738167	136743356	0.365190713	suppress_segment_too_large
LCN12	9	136949551	136955497	0.365190713	suppress_segment_too_large
LCN15	9	136759634	136766255	0.365190713	suppress_segment_too_large
LCN2	9	128149071	128153455	0.365190713	suppress_segment_too_large
LCN6	9	136744011	136748528	0.365190713	suppress_segment_too_large
LCN8	9	136754386	136758543	0.365190713	suppress_segment_too_large
LCN9	9	135663322	135666422	0.365190713	suppress_segment_too_large
LCNL1	9	136981904	136986410	0.365190713	suppress_segment_too_large
LCOR	10	96832282	96995959	-0.367947924	PASS
LCORL	4	17841199	18021876	0.279089364	PASS
LCP1	13	46125920	46211871	-0.390786075	PASS
LCP2	5	170246237	170298227	-0.150979337	PASS
LCT	2	135787840	135837180	-0.475215623	PASS
LCTL	15	66547179	66565979	-0.697600509	PASS
LDAH	2	20684014	20823130	0.040498426	PASS
LDB1	10	102107560	102120453	-0.367947924	PASS
LDB2	4	16501541	16898809	0.279089364	PASS
LDB3	10	86668449	86736068	-0.292453993	PASS

LDHA	11	18394388	18408425	-0.405444717	PASS
LDHAL6A	11	18455824	18479600	-0.405444717	PASS
LDHAL6B	15	59206823	59208515	-0.697600509	PASS
LDHB	12	21635342	21757857	0.362961408	suppress_segment_too_large
LDHC	11	18412307	18452058	-0.405444717	PASS
LDHD	16	75111860	75116771	-0.371357732	PASS
LDLR	19	11089362	11133816	0.093487731	suppress_segment_too_large
LDLRAD1	1	54007299	54018186	0.033763446	PASS
LDLRAD2	1	21812265	21825221	0.033763446	PASS
LDLRAD3	11	35943981	36232136	-0.405444717	PASS
LDLRAD4	18	13217498	13652755	-0.052305056	suppress_segment_too_large
LDLRAP1	1	25543580	25568886	0.033763446	PASS
LDOC1	23	141175745	141177125	-0.012567603	PASS
LEAP2	5	132872322	132875046	-0.293443042	PASS
LECT2	5	135922279	135955034	-0.293443042	PASS
LEF1	4	108047545	108168956	-0.011382299	PASS
LEFTY1	1	225886282	225911382	-0.256698369	PASS
LEFTY2	1	225936598	225941489	-0.256698369	PASS
LEKR1	3	156825481	157046129	0.318278996	suppress_segment_too_large
LELP1	1	153203443	153205120	0.103015788	PASS
LEMD1	1	205381378	205457091	-0.256698369	PASS
LEMD2	6	33771202	33789136	0.048820648	PASS
LEMD3	12	65169571	65248327	-0.202191512	PASS
LENEP	1	154993586	154994315	0.103015788	PASS
LENG1	19	54155161	54159882	0.206912349	PASS
LENG8	19	54448887	54462037	-0.146906334	PASS
LENG9	19	54461796	54463711	-0.146906334	PASS
LEO1	15	51938025	51971806	-0.396255771	PASS
LEP	7	128241284	128257628	-0.388946609	PASS
LEPR	1	65420652	65641559	0.033763446	PASS
LEPROT	1	65420587	65436007	0.033763446	PASS
LEPROTL	8	30095398	30177208	-0.345962679	PASS
LETM1	4	1811479	1856247	0.279089364	PASS
LETM2	8	38386207	38409527	-0.201142258	PASS
LETMD1	12	51047962	51060424	-0.202191512	PASS
LEUTX	19	39776595	39786167	0.093487731	suppress_segment_too_large
LEXM	1	54806063	54842252	0.033763446	PASS
LFNG	7	2512529	2529177	-0.69513118	PASS
LGALS1	22	37675608	37679806	-0.575930959	PASS
LGALS12	11	63506084	63516774	0.182439925	suppress_segment_too_large
LGALS13	19	39602501	39607476	0.093487731	suppress_segment_too_large
LGALS14	19	39704306	39709444	0.093487731	suppress_segment_too_large
LGALS16	19	39655894	39660647	0.093487731	suppress_segment_too_large
LGALS2	22	37570246	37582616	-0.575930959	PASS
LGALS3	14	55124110	55145413	0.060212819	PASS
LGALS3Bl	17	78971238	78980109	0.088516311	PASS

LGALS4	19	38801671	38813364	0.093487731	suppress_segment_too_large
LGALS7	19	38770971	38773492	0.093487731	suppress_segment_too_large
LGALS7B	19	38789211	38791749	0.093487731	suppress_segment_too_large
LGALS8	1	236518000	236552981	-0.256698369	PASS
LGALS9	17	27629798	27649560	0.060555011	PASS
LGALS9B	17	20449395	20467539	0.024610076	PASS
LGALS9C	17	18476737	18494945	0.024610076	PASS
LGALSL	2	64453969	64461381	-0.372959126	PASS
LGI1	10	93757809	93806272	-0.39678585	PASS
LGI2	4	24998847	25030879	0.279089364	PASS
LGI3	8	22146825	22157084	-0.345962679	PASS
LGI4	19	35124513	35142451	0.093487731	suppress_segment_too_large
LGMN	14	92703807	92748702	-0.192197564	PASS
LGR4	11	27365961	27472775	-0.405444717	PASS
LGR5	12	71439770	71586310	-0.202191512	PASS
LGR6	1	202193901	202319781	-0.256698369	PASS
LGSN	6	63275951	63319977	0.048820648	PASS
LHB	19	49015980	49017091	0.14996833	PASS
LHCGR	2	48686775	48755730	0.040498426	PASS
LHFPL1	23	112630648	112680054	-0.012567603	PASS
LHFPL2	5	78485215	78770021	-0.293443042	PASS
LHFPL3	7	104328656	104907232	-0.388946609	PASS
LHFPL4	3	9498361	9553802	0.311018244	suppress_segment_too_large
LHFPL5	6	35805293	35833874	0.048820648	PASS
LHFPL6	13	39209116	39603528	-0.390786075	PASS
LHPP	10	124461834	124617888	0.063874692	PASS
LHX1	17	36936785	36944612	0.060555011	PASS
LHX2	9	124001670	124033301	0.365190713	suppress_segment_too_large
LHX3	9	136196250	136205128	0.365190713	suppress_segment_too_large
LHX4	1	180230286	180278982	-0.027073548	PASS
LHX5	12	113462034	113472280	0.202141228	PASS
LHX6	9	122202577	122229626	0.365190713	suppress_segment_too_large
LHX8	1	75128434	75161533	0.033763446	PASS
LHX9	1	197911902	197935478	-0.027073548	PASS
LIAS	4	39458587	39485109	-0.011382299	PASS
LIF	22	30240447	30246851	-0.575930959	PASS
LIFR	5	38474963	38608354	0.055007813	PASS
LIG1	19	48115445	48170603	0.14996833	PASS
LIG3	17	34980494	35009743	0.060555011	PASS
LIG4	13	108207439	108218368	-0.390786075	PASS
LILRA1	19	54593582	54602090	-0.146906334	PASS
LILRA2	19	54572920	54590287	-0.146906334	PASS
LILRA4	19	54333185	54339150	-0.146906334	PASS
LILRA5	19	54307070	54313139	-0.146906334	PASS
LILRA6	19	54236592	54242791	-0.146906334	PASS
LILRB1	19	54617158	54637528	-0.146906334	PASS

LILRB2	19	54273821	54281184	-0.146906334	PASS
LILRB3	19	54216278	54223506	0.206912349	PASS
LILRB4	19	54643889	54670359	-0.146906334	PASS
LILRB5	19	54249431	54257301	-0.146906334	PASS
LIM2	19	51379909	51387960	-0.016761391	PASS
LIMA1	12	50175788	50283546	-0.202191512	PASS
LIMCH1	4	41359607	41700044	-0.011382299	PASS
LIMD1	3	45555394	45686338	-0.234617007	PASS
LIMD2	17	63695902	63701172	0.088516311	PASS
LIME1	20	63736283	63739103	-0.168668583	PASS
LIMK1	7	74082933	74122525	-0.262778904	PASS
LIMK2	22	31212239	31280080	-0.575930959	PASS
LIMS1	2	108534355	108687246	-0.010901494	PASS
LIMS2	2	127638381	127681786	-0.475215623	PASS
LIMS3	2	109898428	109924868	-0.010901494	PASS
LIMS3-LO	2	109898432	109968557	-0.010901494	PASS
LIN28A	1	26410778	26429722	0.033763446	PASS
LIN28B	6	104936616	105083332	-0.368590549	PASS
LIN37	19	35748361	35754519	0.093487731	suppress_segment_too_large
LIN52	14	74084796	74201235	0.060212819	PASS
LIN54	4	82909973	83012926	-0.011382299	PASS
LIN7A	12	80792520	80937925	0.202141228	PASS
LIN7B	19	49114324	49118460	0.14996833	PASS
LIN7C	11	27494576	27506773	-0.405444717	PASS
LIN9	1	226231149	226309869	-0.256698369	PASS
LINC0220[illegible]	15	21552795	21653276	-0.750600437	PASS
LINC0221[illegible]	17	45620344	45835826	0.088516311	PASS
LINC0221[illegible]	5	17444010	17486829	0.055007813	PASS
LINGO1	15	77613027	77820900	-0.697600509	PASS
LINGO2	9	27948078	28670286	-0.310444659	PASS
LINGO3	19	2289784	2292024	0.079632875	PASS
LINGO4	1	151800264	151806154	0.103015788	PASS
LINS1	15	100559369	100603230	-0.697600509	PASS
LIPA	10	89213569	89414557	-0.39678585	PASS
LIPC	15	58410569	58569843	-0.697600509	PASS
LIPE	19	42401507	42427426	0.093487731	suppress_segment_too_large
LIPF	10	88664441	88678814	-1.64589449	suppress_large_homdel
LIPG	18	49560699	49599182	-0.376397843	PASS
LIPH	3	185506262	185552613	0.318278996	suppress_segment_too_large
LIPI	21	14108813	14210891	-0.304975024	PASS
LIPJ	10	88586753	88606976	-1.64589449	suppress_large_homdel
LIPK	10	88724544	88752786	-1.64589449	suppress_large_homdel
LIPM	10	88802730	88820546	-1.64589449	suppress_large_homdel
LIPN	10	88761406	88778242	-1.64589449	suppress_large_homdel
LIPT1	2	99154955	99163157	0.048238121	PASS
LIPT2	11	74491712	74493733	0.062969153	PASS

LITAF	16	11547722	11636381	0.069936844	PASS
LIX1	5	97091867	97142872	-0.293443042	PASS
LIX1L	1	145933423	145958001	-0.157385092	PASS
LKAAEAR	20	64083380	64084359	-0.168668583	PASS
LLCFC1	7	142939343	142940868	-0.388946609	PASS
LLGL1	17	18225587	18244875	0.024610076	PASS
LLGL2	17	75525080	75575208	0.088516311	PASS
LLPH	12	66116555	66130768	-0.202191512	PASS
LMAN1	18	59327823	59359962	-0.376397843	PASS
LMAN1L	15	74812716	74825758	-0.697600509	PASS
LMAN2	5	177331562	177351852	-0.150979337	PASS
LMAN2L	2	96705929	96740064	0.048238121	PASS
LMBR1	7	156668946	156893230	-0.411415677	PASS
LMBR1L	12	49096551	49110900	-0.202191512	PASS
LMBRD1	6	69675793	69867236	-0.368590549	PASS
LMBRD2	5	36098412	36151961	0.055007813	PASS
LMCD1	3	8501707	8574673	0.311018244	suppress_segment_too_large
LMF1	16	853634	981318	0.069936844	PASS
LMF2	22	50502949	50507691	-0.808211634	PASS
LMLN	3	197960200	198043720	0.326834516	PASS
LMNA	1	156082573	156140089	0.103015788	PASS
LMNB1	5	126776623	126837020	-0.293443042	PASS
LMNB2	19	2427638	2456996	0.079632875	PASS
LMNTD1	12	25409307	25648579	0.362961408	suppress_segment_too_large
LMNTD2	11	554855	560779	-0.333835828	PASS
LMO1	11	8224304	8268716	-0.405444717	PASS
LMO2	11	33858576	33892289	-0.405444717	PASS
LMO3	12	16548373	16610594	0.362961408	suppress_segment_too_large
LMO4	1	87328468	87348923	0.033763446	PASS
LMO7	13	75620434	75859870	-0.390786075	PASS
LMOD1	1	201896452	201946588	-0.256698369	PASS
LMOD2	7	123655807	123664290	-0.388946609	PASS
LMOD3	3	69106872	69123032	-0.234617007	PASS
LMTK2	7	98106885	98209633	-0.262778904	PASS
LMTK3	19	48485271	48513935	0.14996833	PASS
LMX1A	1	165201867	165356715	-0.027073548	PASS
LMX1B	9	126614443	126701032	0.365190713	suppress_segment_too_large
LNP1	3	100401193	100456319	0.313454204	suppress_segment_too_large
LNPEP	5	96935394	97037515	-0.293443042	PASS
LNPK	2	175923892	176002839	0.039726562	PASS
LNX1	4	53459301	53701405	-0.011382299	PASS
LNX2	13	27545911	27620404	-0.390786075	PASS
LONP1	19	5691834	5720572	0.079632875	PASS
LONP2	16	48244296	48363122	0.069936844	PASS
LONRF1	8	12721894	12756073	-0.345962679	PASS
LONRF2	2	100273291	100322733	0.048238121	PASS

LONRF3	23	118974614	119018355	-0.012567603	PASS
LOR	1	153259700	153262122	0.103015788	PASS
LOX	5	122063195	122078360	-0.293443042	PASS
LOXHD1	18	46476972	46657220	-0.376397843	PASS
LOXL1	15	73925989	73952137	-0.697600509	PASS
LOXL2	8	23297189	23425328	-0.345962679	PASS
LOXL3	2	74532414	74555690	-0.372959126	PASS
LOXL4	10	98247690	98268250	-0.367947924	PASS
LPA	6	160531483	160664259	-0.035448111	PASS
LPAR1	9	110873263	111038458	0.365190713	suppress_segment_too_large
LPAR2	19	19623668	19628930	0.093487731	suppress_segment_too_large
LPAR3	1	84811602	84893213	0.033763446	PASS
LPAR4	23	78747709	78757094	-0.012567603	PASS
LPAR5	12	6618835	6636447	-0.086128028	PASS
LPAR6	13	48389567	48444704	-0.390786075	PASS
LPCAT1	5	1456480	1523977	0.055007813	PASS
LPCAT2	16	55508998	55586670	-0.371357732	PASS
LPCAT3	12	6976186	7018510	-0.086128028	PASS
LPCAT4	15	34358618	34367278	-0.396255771	PASS
LPGAT1	1	211743457	211830772	-0.256698369	PASS
LPIN1	2	11677595	11827409	0.040498426	PASS
LPIN2	18	2916994	3013315	-0.052305056	suppress_segment_too_large
LPIN3	20	41340920	41360582	-0.168668583	PASS
LPL	8	19901717	19967259	-0.345962679	PASS
LPO	17	58218548	58268518	0.088516311	PASS
LPP	3	188153284	188890671	0.318278996	suppress_segment_too_large
LPXN	11	58526871	58578220	-0.129266491	PASS
LRAT	4	154626945	154753118	-0.011382299	PASS
LRBA	4	150264531	151015727	-0.011382299	PASS
LRCH1	13	46553168	46753040	-0.390786075	PASS
LRCH2	23	115110616	115234072	-0.012567603	PASS
LRCH3	3	197791226	197888436	0.326834516	PASS
LRCH4	7	100574011	100586153	-0.262778904	PASS
LRCOL1	12	132603150	132610543	0.270848837	PASS
LRFN1	19	39306568	39315336	0.093487731	suppress_segment_too_large
LRFN2	6	40391586	40587465	0.048820648	PASS
LRFN3	19	35935358	35945767	0.093487731	suppress_segment_too_large
LRFN4	11	66856647	66860475	0.182439925	suppress_segment_too_large
LRFN5	14	41607570	41904549	0.060212819	PASS
LRG1	19	4536409	4540474	0.079632875	PASS
LRGUK	7	134127299	134264591	-0.388946609	PASS
LRIF1	1	110947185	110964079	0.033763446	PASS
LRIG1	3	66378797	66501263	-0.234617007	PASS
LRIG2	1	113073209	113132260	0.033763446	PASS
LRIG3	12	58872149	58920522	-0.202191512	PASS
LRIT1	10	84231593	84241461	0.072405807	PASS

LRIT2	10	84220495	84225589	0.072405807	PASS
LRIT3	4	109848202	109872315	-0.011382299	PASS
LRMDA	10	75431453	76560167	0.072405807	PASS
LRMP	12	25004342	25108334	0.362961408	suppress_segment_too_large
LRP1	12	57128493	57213351	-0.202191512	PASS
LRP10	14	22871613	22881580	0.060212819	PASS
LRP11	6	149818798	149864026	-0.035448111	PASS
LRP12	8	104489231	104589024	0.279445683	PASS
LRP1B	2	140231423	142131701	-0.475215623	PASS
LRP2	2	169127109	169362685	0.039726562	PASS
LRP2BP	4	185363879	185395899	-0.462744716	PASS
LRP3	19	33177603	33208867	0.093487731	suppress_segment_too_large
LRP4	11	46856868	46918642	0.117376172	PASS
LRP5	11	68312609	68449275	0.182439925	suppress_segment_too_large
LRP5L	22	25351418	25405377	-0.575930959	PASS
LRP6	12	12116025	12267012	0.362961408	suppress_segment_too_large
LRP8	1	53242364	53328070	0.033763446	PASS
LRPAP1	4	3503597	3532559	0.279089364	PASS
LRPPRC	2	43886508	43996005	0.040498426	PASS
LRR1	14	49598697	49614672	0.060212819	PASS
LRRC1	6	53794497	53924121	0.048820648	PASS
LRRC10	12	69608564	69611162	-0.202191512	PASS
LRRC10B	11	61508800	61511018	0.182439925	suppress_segment_too_large
LRRC14	8	144517992	144525178	0.307788765	PASS
LRRC14B	5	191511	195353	0.055007813	PASS
LRRC15	3	194355247	194369743	0.326834516	PASS
LRRC17	7	102912991	102944949	-0.388946609	PASS
LRRC18	10	48909483	48914232	-0.404852538	PASS
LRRC19	9	26993136	27005693	-0.310444659	PASS
LRRC2	3	46515423	46580099	-0.234617007	PASS
LRRC20	10	70298970	70382650	0.072405807	PASS
LRRC23	12	6873569	6914243	-0.086128028	PASS
LRRC24	8	144522377	144527032	0.307788765	PASS
LRRC25	19	18391144	18397617	0.093487731	suppress_segment_too_large
LRRC26	9	137168758	137170051	0.365190713	suppress_segment_too_large
LRRC27	10	132332154	132379918	0.063874692	PASS
LRRC28	15	99251362	99390729	-0.697600509	PASS
LRRC29	16	67207139	67227048	-0.371357732	PASS
LRRC3	21	44455486	44462196	-0.064425456	suppress_segment_too_large
LRRC30	18	7231125	7232047	-0.052305056	suppress_segment_too_large
LRRC31	3	169839179	169869930	0.318278996	suppress_segment_too_large
LRRC32	11	76657524	76670747	0.062969153	PASS
LRRC34	3	169793428	169812986	0.318278996	suppress_segment_too_large
LRRC36	16	67326798	67385203	-0.371357732	PASS
LRRC37A	17	46292733	46337794	0.088516311	PASS
LRRC37A2	17	46511511	46555650	0.088516311	PASS

LRRC37A3	17	64854312	64919480	0.088516311	PASS
LRRC37B	17	32007872	32053504	0.060555011	PASS
LRRC38	1	13474977	13514048	-0.415253068	PASS
LRRC39	1	100148449	100178273	0.033763446	PASS
LRRC3B	3	26622806	26717537	0.311018244	suppress_segment_too_large
LRRC3C	17	39941474	39944747	0.060555011	PASS
LRRC4	7	128027071	128032107	-0.388946609	PASS
LRRC40	1	70144805	70205620	0.033763446	PASS
LRRC41	1	46261196	46303608	0.033763446	PASS
LRRC42	1	53946077	53968168	0.033763446	PASS
LRRC43	12	122167738	122203471	0.202141228	PASS
LRRC45	17	82023302	82031151	0.088516311	PASS
LRRC46	17	47831627	47837713	0.088516311	PASS
LRRC47	1	3778558	3796504	0.373964873	suppress_segment_too_large
LRRC49	15	70853239	71053657	-0.697600509	PASS
LRRC4B	19	50516892	50568045	-0.016761391	PASS
LRRC4C	11	40114203	41459773	-0.405444717	PASS
LRRC52	1	165544007	165563961	-0.027073548	PASS
LRRC53	1	74469878	74512614	0.033763446	PASS
LRRC55	11	57181747	57191717	-0.129266491	PASS
LRRC56	11	537527	554916	-0.333835828	PASS
LRRC57	15	42537820	42548802	-0.396255771	PASS
LRRC58	3	120324509	120349339	0.313454204	suppress_segment_too_large
LRRC59	17	50375059	50397553	0.088516311	PASS
LRRC6	8	132571953	132675617	0.113969953	PASS
LRRC61	7	150322639	150338150	-0.388946609	PASS
LRRC63	13	46211943	46277366	-0.390786075	PASS
LRRC66	4	51993702	52017620	-0.011382299	PASS
LRRC69	8	91101832	91219236	0.279445683	PASS
LRRC7	1	69568398	70151945	0.033763446	PASS
LRRC70	5	62578735	62581448	-0.293443042	PASS
LRRC71	1	156920650	156933094	0.103015788	PASS
LRRC72	7	16526880	16581568	-0.69513118	PASS
LRRC73	6	43506969	43510686	0.048820648	PASS
LRRC74A	14	76826372	76870302	-0.192197564	PASS
LRRC74B	22	21045960	21064168	-0.575930959	PASS
LRRC75A	17	16441577	16492153	0.024610076	PASS
LRRC75B	22	24585620	24593208	-0.575930959	PASS
LRRC8A	9	128882112	128918039	0.365190713	suppress_segment_too_large
LRRC8B	1	89524836	89597864	0.033763446	PASS
LRRC8C	1	89633072	89769903	0.033763446	PASS
LRRC8D	1	89821014	89936611	0.033763446	PASS
LRRC8E	19	7888505	7902021	0.079632875	PASS
LRRC9	14	59919713	60063559	0.060212819	PASS
LRRCC1	8	85107147	85146076	0.279445683	PASS
LRRD1	7	92141643	92179531	-0.262778904	PASS

LRRFIP1	2	237627576	237813682	0.025928102	PASS
LRRFIP2	3	37052656	37183689	-0.234617007	PASS
LRRIQ1	12	85036314	85264457	0.202141228	PASS
LRRIQ3	1	74026015	74198187	0.033763446	PASS
LRRIQ4	3	169821922	169837775	0.318278996	suppress_segment_too_large
LRRK1	15	100919215	101078254	-0.697600509	PASS
LRRK2	12	40196744	40369285	-0.202191512	PASS
LRRN1	3	3799437	3847703	0.311018244	suppress_segment_too_large
LRRN2	1	204617170	204685733	-0.256698369	PASS
LRRN3	7	111091006	111125454	-0.388946609	PASS
LRRN4	20	6040778	6054049	-0.168668583	PASS
LRRN4CL	11	62686402	62689899	0.182439925	suppress_segment_too_large
LRRTM1	2	80288351	80304749	0.066915641	PASS
LRRTM2	5	138868923	138875368	-0.293443042	PASS
LRRTM3	10	66926006	67099830	0.072405807	PASS
LRRTM4	2	76747719	77593319	-0.372959126	PASS
LRSAM1	9	127451486	127503501	0.365190713	suppress_segment_too_large
LRTM1	3	54918237	54967088	-0.234617007	PASS
LRTM2	12	1820267	1836752	-0.086128028	PASS
LRTOMT	11	72080331	72110782	0.062969153	PASS
LRWD1	7	102464929	102473168	-0.388946609	PASS
LSAMP	3	115802363	117139389	0.313454204	suppress_segment_too_large
LSG1	3	194640788	194672477	0.326834516	PASS
LSM1	8	38163321	38176730	-0.201142258	PASS
LSM10	1	36391238	36397892	0.033763446	PASS
LSM11	5	157743695	157760709	-0.150979337	PASS
LSM12	17	44034635	44067619	0.088516311	PASS
LSM14A	19	34172504	34229515	0.093487731	suppress_segment_too_large
LSM14B	20	62122461	62135378	-0.168668583	PASS
LSM2	6	31797396	31806984	0.048820648	PASS
LSM3	3	14178358	14201119	0.311018244	suppress_segment_too_large
LSM4	19	18306230	18323274	0.093487731	suppress_segment_too_large
LSM5	7	32485332	32495283	-0.69513118	PASS
LSM6	4	146175685	146200000	-0.011382299	PASS
LSM7	19	2321517	2328620	0.079632875	PASS
LSM8	7	118184032	118192824	-0.388946609	PASS
LSMEM1	7	112480853	112491062	-0.388946609	PASS
LSMEM2	3	50279027	50288114	-0.234617007	PASS
LSP1	11	1852970	1892267	-0.333835828	PASS
LSR	19	35248330	35267964	0.093487731	suppress_segment_too_large
LSS	21	46188141	46228824	-0.064425456	suppress_segment_too_large
LST1	6	31586124	31588909	0.048820648	PASS
LTA	6	31572054	31574324	0.048820648	PASS
LTA4H	12	96000828	96043520	0.202141228	PASS
LTB	6	31580525	31582522	0.048820648	PASS
LTB4R	14	24311450	24318036	0.060212819	PASS

LTB4R2	14	24305734	24312053	0.060212819	PASS
LTBP1	2	32946972	33399509	0.040498426	PASS
LTBP2	14	74498170	74612378	-0.192197564	PASS
LTBP3	11	65538805	65558930	0.182439925	suppress_segment_too_large
LTBP4	19	40592883	40629818	0.093487731	suppress_segment_too_large
LTBR	12	6375045	6391571	-0.086128028	PASS
LTC4S	5	179793980	179796647	-0.150979337	PASS
LTF	3	46435645	46485234	-0.234617007	PASS
LTK	15	41503638	41513887	-0.396255771	PASS
LTN1	21	28928144	28992956	0.12860881	suppress_segment_too_large
LTO1	11	69653076	69675416	0.182439925	suppress_segment_too_large
LTV1	6	143843344	143863812	-0.035448111	PASS
LUC7L	16	188969	229463	0.069936844	PASS
LUC7L2	7	139340359	139423457	-0.388946609	PASS
LUC7L3	17	50719544	50756213	0.088516311	PASS
LUM	12	91102629	91111831	0.202141228	PASS
LURAP1	1	46203334	46221261	0.033763446	PASS
LURAP1L	9	12775021	12822131	-0.310444659	PASS
LUZP1	1	23084023	23177808	0.033763446	PASS
LUZP2	11	24496970	25082631	-0.405444717	PASS
LUZP4	23	115289727	115307556	-0.012567603	PASS
LUZP6	7	135927274	135927450	-0.388946609	PASS
LVRN	5	115962454	116027619	-0.293443042	PASS
LXN	3	158645822	158672693	0.318278996	suppress_segment_too_large
LY6D	8	142784880	142786592	0.113969953	PASS
LY6E	8	143017982	143023832	0.113969953	PASS
LY6G5B	6	31670167	31673776	0.048820648	PASS
LY6G5C	6	31676684	31684040	0.048820648	PASS
LY6G6C	6	31718648	31721845	0.048820648	PASS
LY6G6D	6	31715356	31717804	0.048820648	PASS
LY6G6E	6	31711771	31714065	0.048820648	PASS
LY6G6F	6	31706885	31710595	0.048820648	PASS
LY6G6F-L	6	31706904	31717918	0.048820648	PASS
LY6H	8	143157914	143160711	0.307788765	PASS
LY6K	8	142700111	142705127	0.113969953	PASS
LY6L	8	143080457	143083001	0.307788765	PASS
LY75	2	159803355	159904749	0.039726562	PASS
LY75-CD3	2	159771851	159904710	0.039726562	PASS
LY86	6	6588108	6654983	0.048820648	PASS
LY9	1	160796074	160828261	-0.027073548	PASS
LY96	8	73991352	74029087	-0.201142258	PASS
LYAR	4	4267701	4290169	0.279089364	PASS
LYG1	2	99284238	99304742	0.048238121	PASS
LYG2	2	99242246	99255282	0.048238121	PASS
LYL1	19	13099033	13103161	0.093487731	suppress_segment_too_large
LYN	8	55879813	56014168	-0.201142258	PASS

LYNX1	8	142771197	142777810	0.113969953	PASS
LYNX1-SL	8	142764334	142778224	0.113969953	PASS
LYPD1	2	132644853	132671579	-0.475215623	PASS
LYPD2	8	142750150	142752534	0.113969953	PASS
LYPD3	19	43460787	43465660	0.14996833	PASS
LYPD4	19	41837074	41844697	0.093487731	suppress_segment_too_large
LYPD5	19	43795929	43827206	0.14996833	PASS
LYPD6	2	149329985	149474148	-0.475215623	PASS
LYPD6B	2	149038107	149215262	-0.475215623	PASS
LYPD8	1	248718649	248755739	-0.368111568	PASS
LYPLA1	8	54046367	54102017	-0.201142258	PASS
LYPLA2	1	23790970	23795539	0.033763446	PASS
LYPLAL1	1	219173844	219212865	-0.256698369	PASS
LYRM1	16	20899868	20925006	0.069936844	PASS
LYRM2	6	89568144	89638753	-0.368590549	PASS
LYRM4	6	5102593	5260939	0.048820648	PASS
LYRM7	5	131170810	131205426	-0.293443042	PASS
LYRM9	17	27878314	27894752	0.060555011	PASS
LYSMD1	1	151159748	151165948	0.103015788	PASS
LYSMD2	15	51723011	51751585	-0.396255771	PASS
LYSMD3	5	90515611	90529584	-0.293443042	PASS
LYSMD4	15	99715697	99733561	-0.697600509	PASS
LYST	1	235661041	235883640	-0.256698369	PASS
LYVE1	11	10556966	10611689	-0.405444717	PASS
LYZ	12	69348341	69354234	-0.202191512	PASS
LYZL1	10	29289061	29318328	0.039811807	PASS
LYZL2	10	30611779	30629762	0.039811807	PASS
LYZL4	3	42397078	42410600	-0.234617007	PASS
LYZL6	17	35934518	35943699	0.060555011	PASS
LZIC	1	9922113	9943407	0.373964873	suppress_segment_too_large
LZTFL1	3	45823316	45916042	-0.234617007	PASS
LZTR1	22	20982013	20999038	-0.575930959	PASS
LZTS1	8	20246165	20303963	-0.345962679	PASS
LZTS2	10	100996618	101007836	-0.367947924	PASS
LZTS3	20	3162617	3173592	-0.168668583	PASS
M1AP	2	74557883	74648338	-0.372959126	PASS
M6PR	12	8940363	8949955	0.362961408	suppress_segment_too_large
MAATS1	3	119703022	119767102	0.313454204	suppress_segment_too_large
MAB21L1	13	35474182	35477209	-0.390786075	PASS
MAB21L2	4	150581922	150584693	-0.011382299	PASS
MAB21L3	1	116111755	116135240	0.033763446	PASS
MAB21L4	2	240886048	240896889	0.025928102	PASS
MACC1	7	20134655	20217404	-0.69513118	PASS
MACF1	1	39081316	39487177	0.033763446	PASS
MACO1	1	25430858	25500209	0.033763446	PASS
MACROD	11	63998558	64166106	0.182439925	suppress_segment_too_large

MACROD2	20	13995369	16053197	-0.168668583	PASS
MAD1L1	7	1815793	2233243	-0.69513118	PASS
MAD2L1	4	120055608	120067074	-0.011382299	PASS
MAD2L1B	6	43629540	43640952	0.048820648	PASS
MAD2L2	1	11674480	11691650	0.373964873	suppress_segment_too_large
MADCAM	19	489176	505342	0.079632875	PASS
MADD	11	47269161	47330031	0.117376172	PASS
MAEA	4	1289851	1340147	0.279089364	PASS
MAEL	1	166975582	167022214	-0.027073548	PASS
MAF	16	79585843	79600714	-0.371357732	PASS
MAF1	8	144104499	144107611	0.307788765	PASS
MAFA	8	143419182	143430406	0.307788765	PASS
MAFB	20	40685848	40689240	-0.168668583	PASS
MAFF	22	38200767	38216511	-0.575930959	PASS
MAFG	17	81918270	81927714	0.088516311	PASS
MAFK	7	1530714	1543043	-0.69513118	PASS
MAG	19	35292125	35313804	0.093487731	suppress_segment_too_large
MAGEA1	23	153179285	153183880	-0.012567603	PASS
MAGEA10	23	152133310	152138578	-0.012567603	PASS
MAGEA11	23	149688228	149717266	-0.012567603	PASS
MAGEA12	23	152733779	152737669	-0.012567603	PASS
MAGEA3	23	152698752	152702347	-0.012567603	PASS
MAGEA4	23	151912509	151925170	-0.012567603	PASS
MAGEA6	23	152766136	152769747	-0.012567603	PASS
MAGEA8	23	149881141	149885835	-0.012567603	PASS
MAGEA9	23	149781930	149787737	-0.012567603	PASS
MAGEB1	23	30243730	30252038	-0.012567603	PASS
MAGEB10	23	27807990	27823014	-0.012567603	PASS
MAGEB16	23	35798342	35803735	-0.012567603	PASS
MAGEB17	23	16167481	16171464	-0.012567603	PASS
MAGEB18	23	26138343	26140735	-0.012567603	PASS
MAGEB2	23	30215560	30220089	-0.012567603	PASS
MAGEB3	23	30230436	30237492	-0.012567603	PASS
MAGEB4	23	30242052	30244193	-0.012567603	PASS
MAGEB5	23	26216169	26218270	-0.012567603	PASS
MAGEB6	23	26192440	26195646	-0.012567603	PASS
MAGEB6E	23	26160601	26161824	-0.012567603	PASS
MAGEC1	23	141903894	141909388	-0.012567603	PASS
MAGEC2	23	142202345	142205290	-0.012567603	PASS
MAGEC3	23	141838316	141897832	-0.012567603	PASS
MAGED1	23	51803007	51902357	-0.012567603	PASS
MAGED2	23	54807599	54816012	-0.012567603	PASS
MAGEE1	23	76427724	76431353	-0.012567603	PASS
MAGEE2	23	75782988	75785236	-0.012567603	PASS
MAGEF1	3	184710367	184712002	0.318278996	suppress_segment_too_large
MAGEH1	23	55452105	55453566	-0.012567603	PASS

MAGEL2	15	23643549	23647867	-0.396255771	PASS
MAGI1	3	65353525	66038834	-0.234617007	PASS
MAGI2	7	78017057	79453574	-0.262778904	PASS
MAGI3	1	113390749	113685923	0.033763446	PASS
MAGIX	23	49162564	49168483	-0.012567603	PASS
MAGOH	1	53226892	53238610	0.033763446	PASS
MAGOHB	12	10604190	10613623	0.362961408	suppress_segment_too_large
MAGT1	23	77826364	77895593	-0.012567603	PASS
MAIP1	2	199955317	200008540	0.025928102	PASS
MAJIN	11	64937517	64972085	0.182439925	suppress_segment_too_large
MAK	6	10762723	10838555	0.048820648	PASS
MAK16	8	33484750	33501260	-0.345962679	PASS
MAL	2	95025677	95053996	-0.340781246	PASS
MAL2	8	119165034	119245673	0.279445683	PASS
MALL	2	110083870	110116566	-0.010901494	PASS
MALRD1	10	19048771	19790401	0.039811807	PASS
MALSU1	7	23298739	23311729	-0.69513118	PASS
MALT1	18	58671386	58754477	-0.376397843	PASS
MAMDC2	9	70043581	70226970	0.365190713	suppress_segment_too_large
MAMDC4	9	136850943	136860799	0.365190713	suppress_segment_too_large
MAML1	5	179732850	179777286	-0.150979337	PASS
MAML2	11	95976598	96343180	0.062969153	PASS
MAML3	4	139716753	140154184	-0.011382299	PASS
MAMLD1	23	150361422	150514178	-0.012567603	PASS
MAMSTR	19	48712742	48719721	0.14996833	PASS
MAN1A1	6	119177209	119349761	-0.368590549	PASS
MAN1A2	1	117367449	117528872	0.033763446	PASS
MAN1B1	9	137086927	137109187	0.365190713	suppress_segment_too_large
MAN1C1	1	25617468	25786207	0.033763446	PASS
MAN2A1	5	109689366	109869625	-0.293443042	PASS
MAN2A2	15	90902218	90922584	-0.697600509	PASS
MAN2B1	19	12646511	12666742	0.093487731	suppress_segment_too_large
MAN2B2	4	6575175	6623362	0.279089364	PASS
MAN2C1	15	75355207	75368630	-0.697600509	PASS
MANBA	4	102630770	102760994	-0.011382299	PASS
MANBAL	20	37289638	37317260	-0.168668583	PASS
MANEA	6	95577543	95609457	-0.368590549	PASS
MANEAL	1	37793802	37801137	0.033763446	PASS
MANF	3	51385047	51389397	-0.234617007	PASS
MANSC1	12	12326056	12350541	0.362961408	suppress_segment_too_large
MANSC4	12	27762738	27771276	0.362961408	suppress_segment_too_large
MAOA	23	43654907	43746824	-0.012567603	PASS
MAOB	23	43766611	43882447	-0.012567603	PASS
MAP10	1	232804892	232808407	-0.256698369	PASS
MAP1A	15	43510958	43531620	-0.396255771	PASS
MAP1B	5	72107234	72209570	-0.293443042	PASS

MAP1LC3.	20	34546854	34560345	-0.168668583	PASS
MAP1LC3.	16	87383995	87404779	-0.371357732	PASS
MAP1LC3.	12	116548105	116576448	0.202141228	PASS
MAP1LC3(	1	241995490	241999073	-0.648598641	PASS
MAP1S	19	17719242	17734516	0.093487731	suppress_segment_too_large
MAP2	2	209424058	209734118	0.025928102	PASS
MAP2K1	15	66386817	66492312	-0.697600509	PASS
MAP2K2	19	4090321	4124129	0.079632875	PASS
MAP2K3	17	21284672	21315240	0.060555011	PASS
MAP2K4	17	12020824	12143830	-0.139031004	PASS
MAP2K5	15	67542709	67807123	-0.697600509	PASS
MAP2K6	17	69414698	69553861	0.088516311	PASS
MAP2K7	19	7903843	7914478	0.079632875	PASS
MAP3K1	5	56815574	56896152	-0.293443042	PASS
MAP3K10	19	40191744	40215575	0.093487731	suppress_segment_too_large
MAP3K11	11	65597755	65615382	0.182439925	suppress_segment_too_large
MAP3K12	12	53479669	53500063	-0.202191512	PASS
MAP3K13	3	185282941	185489097	0.318278996	suppress_segment_too_large
MAP3K14	17	45263119	45317145	0.088516311	PASS
MAP3K15	23	19360056	19515261	-0.012567603	PASS
MAP3K19	2	134964491	135047468	-0.475215623	PASS
MAP3K2	2	127298730	127388465	-0.475215623	PASS
MAP3K20	2	173075435	173268010	0.039726562	PASS
MAP3K21	1	233327768	233385148	-0.256698369	PASS
MAP3K3	17	63622415	63696303	0.088516311	PASS
MAP3K4	6	160991727	161117385	-0.035448111	PASS
MAP3K5	6	136557047	136792518	-0.035448111	PASS
MAP3K6	1	27355184	27366892	0.033763446	PASS
MAP3K7	6	90513573	90587045	-0.368590549	PASS
MAP3K7C	21	29077471	29175889	0.12860881	suppress_segment_too_large
MAP3K8	10	30433937	30461833	0.039811807	PASS
MAP3K9	14	70722526	70809534	0.060212819	PASS
MAP4	3	47850690	48089272	-0.234617007	PASS
MAP4K1	19	38587641	38618882	0.093487731	suppress_segment_too_large
MAP4K2	11	64784914	64803241	0.182439925	suppress_segment_too_large
MAP4K3	2	39249266	39437312	0.040498426	PASS
MAP4K4	2	101696850	101894689	0.048238121	PASS
MAP4K5	14	50418501	50561126	0.060212819	PASS
MAP6	11	75586918	75669120	0.062969153	PASS
MAP6D1	3	183815876	183825594	0.318278996	suppress_segment_too_large
MAP7	6	136342281	136550819	-0.035448111	PASS
MAP7D1	1	36155579	36180849	0.033763446	PASS
MAP7D2	23	20006713	20116917	-0.012567603	PASS
MAP7D3	23	136213220	136256482	-0.012567603	PASS
MAP9	4	155342658	155376970	-0.011382299	PASS
MAPK1	22	21754500	21867680	-0.575930959	PASS

MAPK10	4	85990007	86594625	-0.011382299	PASS
MAPK11	22	50263713	50270767	-0.808211634	PASS
MAPK12	22	50245450	50261825	-0.808211634	PASS
MAPK13	6	36127809	36144524	0.048820648	PASS
MAPK14	6	36027677	36111236	0.048820648	PASS
MAPK15	8	143716259	143722458	0.307788765	PASS
MAPK1IP1	14	55051631	55070192	0.060212819	PASS
MAPK3	16	30114105	30123506	0.069936844	PASS
MAPK4	18	50560078	50731824	-0.376397843	PASS
MAPK6	15	51952106	52067372	-0.396255771	PASS
MAPK7	17	19377721	19383544	0.024610076	PASS
MAPK8	10	48306639	48439360	-0.404852538	PASS
MAPK8IP1	11	45885651	45906465	0.117376172	PASS
MAPK8IP2	22	50600685	50613981	-0.808211634	PASS
MAPK8IP3	16	1706183	1770317	0.069936844	PASS
MAPK9	5	180233143	180292099	-0.150979337	PASS
MAPKAP1	9	125437393	125707234	0.365190713	suppress_segment_too_large
MAPKAPK	1	206684944	206734283	-0.256698369	PASS
MAPKAPK	3	50611520	50649297	-0.234617007	PASS
MAPKAPK	12	111841978	111902238	0.202141228	PASS
MAPKBP1	15	41774434	41827855	-0.396255771	PASS
MAPRE1	20	32819893	32850405	-0.168668583	PASS
MAPRE2	18	34976928	35143470	-0.376397843	PASS
MAPRE3	2	26970612	27027196	0.040498426	PASS
MAPT	17	45894382	46028334	0.088516311	PASS
1-Mar	1	220786759	220819657	-0.256698369	PASS
2-Mar	1	220748225	220784815	-0.256698369	PASS
1-Mar	4	163524298	164384050	-0.011382299	PASS
10-Mar	17	62701314	62808344	0.088516311	PASS
11-Mar	5	16067139	16180762	0.055007813	PASS
2-Mar	19	8413270	8439017	0.079632875	PASS
3-Mar	5	126867714	127030808	-0.293443042	PASS
4-Mar	2	216257865	216372027	0.025928102	PASS
5-Mar	10	92291163	92353964	-0.39678585	PASS
6-Mar	5	10353703	10440388	0.055007813	PASS
7-Mar	2	159712457	159771027	0.039726562	PASS
8-Mar	10	45454585	45594906	-0.404852538	PASS
9-Mar	12	57755098	57760407	-0.202191512	PASS
MARCKS	6	113857362	113863471	-0.368590549	PASS
MARCKSL	1	32333832	32336379	0.033763446	PASS
MARCO	2	118942166	118994660	-0.475215623	PASS
MARF1	16	15594386	15643166	0.069936844	PASS
MARK1	1	220528183	220664461	-0.256698369	PASS
MARK2	11	63838928	63911019	0.182439925	suppress_segment_too_large
MARK3	14	103385392	103503831	-0.192197564	PASS
MARK4	19	45079288	45305283	0.14996833	PASS

MARS	12	57475445	57517569		-0.202191512	PASS
MARS2	2	197705369	197708387		0.025928102	PASS
MARVELI	10	97713173	97718152		-0.367947924	PASS
MARVELI	5	69415065	69444330		-0.293443042	PASS
MARVELI	16	71626161	71642114		-0.371357732	PASS
MAS1	6	159906690	159916530		-0.035448111	PASS
MAS1L	6	29486697	29487956		0.048820648	PASS
MASP1	3	187217285	187292022		0.318278996	suppress_segment_too_large
MASP2	1	11026523	11047233		0.373964873	suppress_segment_too_large
MAST1	19	12833951	12874951		0.093487731	suppress_segment_too_large
MAST2	1	45786987	46036124		0.033763446	PASS
MAST3	19	18097793	18151692		0.093487731	suppress_segment_too_large
MAST4	5	66596361	67169595		-0.293443042	PASS
MASTL	10	27154824	27186924		0.039811807	PASS
MAT1A	10	80271820	80289684		0.072405807	PASS
MAT2A	2	85539165	85545280		0.066915641	PASS
MAT2B	5	163503114	163519336		-0.150979337	PASS
MATK	19	3777970	3802129		0.079632875	PASS
MATN1	1	30711277	30723587		0.033763446	PASS
MATN2	8	97868840	98036716		0.279445683	PASS
MATN3	2	19992111	20012694		0.040498426	PASS
MATN4	20	45293445	45308529		-0.168668583	PASS
MATR3	5	139273752	139331677		-0.293443042	PASS
MAU2	19	19320681	19358755		0.093487731	suppress_segment_too_large
MAVS	20	3846799	3876123		-0.168668583	PASS
MAX	14	65006174	65102695		0.060212819	PASS
MAZ	16	29806106	29811164		0.069936844	PASS
MB	22	35606764	35637951		-0.575930959	PASS
MB21D2	3	192796815	192918161		-0.627320105	PASS
MBD1	18	50266882	50281774		-0.376397843	PASS
MBD2	18	54151601	54224788		-0.376397843	PASS
MBD3	19	1573596	1592801		0.079632875	PASS
MBD3L1	19	8832398	8843340		0.079632875	PASS
MBD3L2	19	7049321	7051735		0.079632875	PASS
MBD3L2B	19	7018969	7021450		0.079632875	PASS
MBD3L3	19	7056209	7058640		0.079632875	PASS
MBD3L4	19	7037748	7040179		0.079632875	PASS
MBD3L5	19	7030578	7033011		0.079632875	PASS
MBD4	3	129430944	129440179		0.313454204	suppress_segment_too_large
MBD5	2	148021011	148516971		-0.475215623	PASS
MBD6	12	57520710	57530148		-0.202191512	PASS
MBIP	14	36298558	36320676		0.060212819	PASS
MBL2	10	52765380	52771700		-0.404852538	PASS
MBLAC1	7	100126694	100128498		-0.262778904	PASS
MBLAC2	5	90458203	90474768		-0.293443042	PASS
MBNL1	3	152243828	152465780		0.318278996	suppress_segment_too_large

MBNL2	13	97221434	97394120		-0.390786075	PASS
MBNL3	23	132369317	132489968		-0.012567603	PASS
MBOAT1	6	20102145	20212399		0.048820648	PASS
MBOAT2	2	8852690	9003813		0.040498426	PASS
MBOAT4	8	30131824	30144686		-0.345962679	PASS
MBOAT7	19	54173412	54189882		0.206912349	PASS
MBP	18	76978827	77133683		-0.376397843	PASS
MBTD1	17	51177425	51260163		0.088516311	PASS
MBTPS1	16	84053761	84116906		-0.371357732	PASS
MBTPS2	23	21839636	21885424		-0.012567603	PASS
MC1R	16	89912119	89920977		-0.371357732	PASS
MC2R	18	13882044	13915707		-0.052305056	suppress_segment_too_large
MC3R	20	56248732	56249815		-0.168668583	PASS
MC4R	18	60371062	60372775		-0.376397843	PASS
MC5R	18	13824149	13827323		-0.052305056	suppress_segment_too_large
MCAM	11	119308529	119321521		-0.356188624	PASS
MCAT	22	43132206	43143394		-0.575930959	PASS
MCC	5	113022099	113488830		-0.293443042	PASS
MCCC1	3	183015218	183116075		0.318278996	suppress_segment_too_large
MCCC2	5	71587288	71658704		-0.293443042	PASS
MCCD1	6	31528717	31530232		0.048820648	PASS
MCEE	2	71109684	71130239		-0.372959126	PASS
MCEMP1	19	7676628	7679826		0.079632875	PASS
MCF2	23	139581770	139708227		-0.012567603	PASS
MCF2L	13	112894378	113099739		-0.390786075	PASS
MCF2L2	3	183178043	183428778		0.318278996	suppress_segment_too_large
MCFD2	2	46901870	46941855		0.040498426	PASS
MCHR1	22	40678750	40682814		-0.575930959	PASS
MCHR2	6	99919910	99994247		-0.368590549	PASS
MCIDAS	5	55219614	55227315		-0.293443042	PASS
MCL1	1	150574551	150579738		0.103015788	PASS
MCM10	10	13161554	13211104		0.039811807	PASS
MCM2	3	127598223	127622436		0.313454204	suppress_segment_too_large
MCM3	6	52264009	52284881		0.048820648	PASS
MCM3AP	21	46235126	46286297		-0.064425456	suppress_segment_too_large
MCM4	8	47960185	47978160		-0.201142258	PASS
MCM5	22	35400063	35425430		-0.575930959	PASS
MCM6	2	135839626	135876426		-0.475215623	PASS
MCM7	7	100092728	100101940		-0.262778904	PASS
MCM8	20	5950652	5998977		-0.168668583	PASS
MCM9	6	118813442	118935162		-0.368590549	PASS
MCMBP	10	119829404	119892556		0.063874692	PASS
MCMDC2	8	66870749	66922048		-0.201142258	PASS
MCOLN1	19	7522626	7534009		0.079632875	PASS
MCOLN2	1	84925583	84997113		0.033763446	PASS
MCOLN3	1	85018082	85048499		0.033763446	PASS

MCPH1	8	6406592	6648504	-1.076352548	PASS
MCRIP1	17	81822361	81833302	0.088516311	PASS
MCRIP2	16	636817	648474	0.069936844	PASS
MCRS1	12	49556544	49568153	-0.202191512	PASS
MCTP1	5	94703741	95284575	-0.293443042	PASS
MCTP2	15	94231538	94483952	-0.697600509	PASS
MCTS1	23	120594010	120621074	-0.012567603	PASS
MCTS2P	20	31547412	31548081	-0.168668583	PASS
MCU	10	72692131	72887694	0.072405807	PASS
MCUB	4	109560205	109688726	-0.011382299	PASS
MCUR1	6	13786557	13814568	0.048820648	PASS
MDC1	6	30699807	30717889	0.048820648	PASS
MDFI	6	41636882	41654246	0.048820648	PASS
MDFIC	7	114922154	115019202	-0.388946609	PASS
MDFIC2	3	70196894	70312726	-0.234617007	PASS
MDGA1	6	37630679	37699306	0.048820648	PASS
MDGA2	14	46840092	47674954	0.060212819	PASS
MDH1	2	63588609	63607197	-0.372959126	PASS
MDH1B	2	206737763	206765547	0.025928102	PASS
MDH2	7	76048051	76067508	-0.262778904	PASS
MDK	11	46380756	46383837	0.117376172	PASS
MDM1	12	68272443	68332381	-0.202191512	PASS
MDM2	12	68808176	68850686	-0.202191512	PASS
MDM4	1	204516379	204558120	-0.256698369	PASS
MDN1	6	89642499	89819723	-0.368590549	PASS
MDP1	14	24213937	24216070	0.060212819	PASS
ME1	6	83210389	83431071	-0.368590549	PASS
ME2	18	50878734	50954257	-0.376397843	PASS
ME3	11	86441108	86672636	0.062969153	PASS
MEA1	6	43011143	43016868	0.048820648	PASS
MEAF6	1	37492575	37514774	0.033763446	PASS
MECOM	3	169083499	169663618	0.318278996	suppress_segment_too_large
MECP2	23	154021573	154137103	-0.012567603	PASS
MECR	1	29192873	29230942	0.033763446	PASS
MED1	17	39404285	39451286	0.060555011	PASS
MED10	5	6371881	6378594	0.055007813	PASS
MED11	17	4731428	4733610	0.128406487	PASS
MED12	23	71118556	71142454	-0.012567603	PASS
MED12L	3	151085697	151437072	0.318278996	suppress_segment_too_large
MED13	17	61942605	62065282	0.088516311	PASS
MED13L	12	115953872	116277719	0.202141228	PASS
MED14	23	40648306	40735858	-0.012567603	PASS
MED15	22	20495913	20587632	-0.575930959	PASS
MED16	19	867630	893218	0.079632875	PASS
MED17	11	93784227	93814963	0.062969153	PASS
MED18	1	28329002	28335967	0.033763446	PASS

MED19	11	57703714	57712323	-0.129266491	PASS
MED20	6	41905354	41921139	0.048820648	PASS
MED21	12	27022546	27066343	0.362961408	suppress_segment_too_large
MED22	9	133338323	133348131	0.365190713	suppress_segment_too_large
MED23	6	131573966	131628229	-0.458570262	PASS
MED24	17	40019097	40061215	0.060555011	PASS
MED25	19	49818279	49840383	0.14996833	PASS
MED26	19	16574907	16629062	0.093487731	suppress_segment_too_large
MED27	9	131860107	132079908	0.365190713	suppress_segment_too_large
MED28	4	17614631	17634105	0.279089364	PASS
MED29	19	39391303	39400637	0.093487731	suppress_segment_too_large
MED30	8	117520713	117540262	0.279445683	PASS
MED31	17	6643315	6651634	0.128406487	PASS
MED4	13	48053323	48095131	-0.390786075	PASS
MED6	14	70581257	70600690	0.060212819	PASS
MED7	5	157137412	157159019	-0.293443042	PASS
MED8	1	43383917	43389808	0.033763446	PASS
MED9	17	17476986	17493226	0.024610076	PASS
MEDAG	13	30906191	30925572	-0.390786075	PASS
MEF2A	15	99565417	99716466	-0.697600509	PASS
MEF2B	19	19145568	19170289	0.093487731	suppress_segment_too_large
MEF2C	5	88717117	88904257	-0.293443042	PASS
MEF2D	1	156463727	156500828	0.103015788	PASS
MEFV	16	3242028	3256627	0.069936844	PASS
MEGF10	5	127290831	127465737	-0.293443042	PASS
MEGF11	15	65895079	66253747	-0.697600509	PASS
MEGF6	1	3489920	3611495	0.373964873	suppress_segment_too_large
MEGF8	19	42325609	42378769	0.093487731	suppress_segment_too_large
MEGF9	9	120600813	120714470	0.365190713	suppress_segment_too_large
MEI1	22	41699499	41799456	-0.575930959	PASS
MEI4	6	77650274	77927028	-0.368590549	PASS
MEIG1	10	14959439	14988050	0.039811807	PASS
MEIKIN	5	131806993	131945698	-0.293443042	PASS
MEIOB	16	1833987	1884294	0.069936844	PASS
MEIOC	17	44656404	44690308	0.088516311	PASS
MEIS1	2	66433452	66573869	-0.372959126	PASS
MEIS2	15	36889204	37101299	-0.396255771	PASS
MEIS3	19	47403124	47419523	0.14996833	PASS
MELK	9	36572862	36677683	-0.310444659	PASS
MELTF	3	196988621	197029816	0.326834516	PASS
MEMO1	2	31865060	32011230	0.040498426	PASS
MEN1	11	64803510	64811294	0.182439925	suppress_segment_too_large
MEOX1	17	43640388	43661954	0.088516311	PASS
MEOX2	7	15611212	15686812	-0.69513118	PASS
MEP1A	6	46793390	46839782	0.048820648	PASS
MEP1B	18	32185069	32220404	-0.376397843	PASS

MEPCE	7	100428790	100434126	-0.262778904	PASS
MEPE	4	87821411	87846817	-0.011382299	PASS
MERTK	2	111898479	112029561	-0.010901494	PASS
MESD	15	80946289	80989878	-0.697600509	PASS
MESP1	15	89748661	89751310	-0.697600509	PASS
MESP2	15	89760591	89778754	-0.697600509	PASS
MEST	7	130486171	130506465	-0.388946609	PASS
MET	7	116672390	116798386	-0.388946609	PASS
METAP1	4	98995620	99062813	-0.011382299	PASS
METAP1D	2	171999583	172082430	0.039726562	PASS
METAP2	12	95473520	95515839	0.202141228	PASS
METRN	16	715115	719655	0.069936844	PASS
METRNL	17	83079691	83095119	0.088516311	PASS
METTL1	12	57768471	57772793	-0.202191512	PASS
METTL11I	1	170146001	170167790	-0.027073548	PASS
METTL13	1	171781664	171814023	-0.027073548	PASS
METTL14	4	118685368	118715433	-0.011382299	PASS
METTL15	11	28108248	28527041	-0.405444717	PASS
METTL16	17	2405562	2511891	0.128406487	PASS
METTL17	14	20989770	20997035	0.060212819	PASS
METTL18	1	169792529	169794966	-0.027073548	PASS
METTL21/	2	207580631	207625928	0.025928102	PASS
METTL21(	13	102685744	102694504	-0.390786075	PASS
METTL22	16	8621683	8649654	0.069936844	PASS
METTL23	17	76726830	76733936	0.088516311	PASS
METTL24	6	110245928	110358272	-0.368590549	PASS
METTL25	12	82358497	82479236	0.202141228	PASS
METTL26	16	634427	636366	0.069936844	PASS
METTL27	7	73834590	73842535	-0.262778904	PASS
METTL2A	17	62423867	62450822	0.088516311	PASS
METTL2B	7	128476729	128506602	-0.388946609	PASS
METTL3	14	21498133	21511375	0.060212819	PASS
METTL4	18	2537525	2571509	-0.052305056	suppress_segment_too_large
METTL5	2	169810081	169824931	0.039726562	PASS
METTL6	3	15381275	15440566	0.311018244	suppress_segment_too_large
METTL7A	12	50923472	50932517	-0.202191512	PASS
METTL7B	12	55681546	55684611	-0.202191512	PASS
METTL8	2	171317405	171434802	0.039726562	PASS
METTL9	16	21597218	21657473	0.069936844	PASS
MEX3A	1	156072013	156081998	0.103015788	PASS
MEX3B	15	82041778	82046141	-0.697600509	PASS
MEX3C	18	51174550	51218304	-0.376397843	PASS
MEX3D	19	1554669	1568058	0.079632875	PASS
MFAP1	15	43804492	43824802	-0.396255771	PASS
MFAP2	1	16974502	16980835	0.033763446	PASS
MFAP3	5	154038906	154220478	-0.293443042	PASS

MFAP3L	4	169986597	170033031	-0.011382299	PASS
MFAP4	17	19383442	19387240	0.024610076	PASS
MFAP5	12	8637346	8662888	0.362961408	suppress_segment_too_large
MFF	2	227325151	227357836	0.025928102	PASS
MFGE8	15	88898683	88913411	-0.697600509	PASS
MFHAS1	8	8783354	8893645	-0.345962679	PASS
MFN1	3	179347692	179394931	0.318278996	suppress_segment_too_large
MFN2	1	11980181	12013514	0.373964873	suppress_segment_too_large
MFNG	22	37469063	37486401	-0.575930959	PASS
MFRP	11	119338942	119346673	-0.356188624	PASS
MFSD1	3	158732198	158829719	0.318278996	suppress_segment_too_large
MFSD10	4	2930561	2934859	0.279089364	PASS
MFSD11	17	76735865	76781449	0.088516311	PASS
MFSD12	19	3538261	3574290	0.079632875	PASS
MFSD13A	10	102461395	102477045	-0.367947924	PASS
MFSD14A	1	100038097	100083377	0.033763446	PASS
MFSD14B	9	94374551	94461042	0.365190713	suppress_segment_too_large
MFSD14C	9	96887373	97013708	0.365190713	suppress_segment_too_large
MFSD2A	1	39955112	39969968	0.033763446	PASS
MFSD2B	2	24010081	24063321	0.040498426	PASS
MFSD3	8	144509074	144511213	0.307788765	PASS
MFSD4A	1	205568885	205602918	-0.256698369	PASS
MFSD4B	6	111259348	111271167	-0.368590549	PASS
MFSD5	12	53251251	53254405	-0.202191512	PASS
MFSD6	2	190408355	190509205	0.025928102	PASS
MFSD6L	17	8797162	8799349	0.128406487	PASS
MFSD8	4	127917732	127966034	-0.011382299	PASS
MFSD9	2	102715840	102736888	0.048238121	PASS
MGA	15	41621224	41773081	-0.396255771	PASS
MGAM	7	141907813	142106747	-0.388946609	PASS
MGAM2	7	142111749	142222324	-0.388946609	PASS
MGARP	4	139266163	139280338	-0.011382299	PASS
MGAT1	5	180784782	180815652	-0.150979337	PASS
MGAT2	14	49620795	49623481	0.060212819	PASS
MGAT3	22	39457344	39492194	-0.575930959	PASS
MGAT4A	2	98619106	98731126	0.048238121	PASS
MGAT4B	5	179797597	179806952	-0.150979337	PASS
MGAT4C	12	85955666	86838904	0.202141228	PASS
MGAT4D	4	140442262	140498377	-0.011382299	PASS
MGAT5	2	134119983	134454621	-0.475215623	PASS
MGAT5B	17	76868456	76950393	0.088516311	PASS
MGLL	3	127689062	128052190	0.313454204	suppress_segment_too_large
MGME1	20	17968913	17991122	-0.168668583	PASS
MGMT	10	129467184	129768007	0.063874692	PASS
MGP	12	14881181	14885926	0.362961408	suppress_segment_too_large
MGRN1	16	4616493	4690974	0.069936844	PASS

MGST1	12	16347142	16609259	0.362961408	suppress_segment_too_large
MGST2	4	139665768	139740745	-0.011382299	PASS
MGST3	1	165630861	165661796	-0.027073548	PASS
MIA	19	40771648	40777490	0.093487731	suppress_segment_too_large
MIA-RAB4	19	40771648	40796943	0.093487731	suppress_segment_too_large
MIA2	14	39230231	39388513	0.060212819	PASS
MIA3	1	222618086	222668012	-0.256698369	PASS
MIB1	18	21704957	21870957	-0.376397843	PASS
MIB2	1	1615415	1630610	0.373964873	suppress_segment_too_large
MICA	6	31399784	31415315	0.048820648	PASS
MICAL1	6	109444062	109465968	-0.368590549	PASS
MICAL2	11	12094008	12359144	-0.405444717	PASS
MICAL3	22	17787649	18024559	-0.575930959	PASS
MICALL1	22	37905657	37942822	-0.575930959	PASS
MICALL2	7	1428465	1459502	-0.69513118	PASS
MICB	6	31494881	31511124	0.048820648	PASS
MICU1	10	72367327	72626191	0.072405807	PASS
MICU2	13	21492691	21604214	-0.390786075	PASS
MICU3	8	17027238	17122644	-0.345962679	PASS
MID1	23	10445310	10833654	-0.012567603	PASS
MID1IP1	23	38801432	38806537	-0.012567603	PASS
MID2	23	107825755	107927193	-0.012567603	PASS
MIDN	19	1248553	1259140	0.079632875	PASS
MIEF1	22	39499432	39518132	-0.575930959	PASS
MIEF2	17	18260534	18266552	0.024610076	PASS
MIEN1	17	39728496	39730787	0.060555011	PASS
MIER1	1	66924895	66988619	0.033763446	PASS
MIER2	19	301444	344815	0.079632875	PASS
MIER3	5	56919602	56971675	-0.293443042	PASS
MIF	22	23894004	23895227	-0.575930959	PASS
MIF4GD	17	75266228	75271227	0.088516311	PASS
MIGA1	1	77779624	77879539	0.033763446	PASS
MIGA2	9	129036621	129072082	0.365190713	suppress_segment_too_large
MIIP	1	12019466	12032045	0.373964873	suppress_segment_too_large
MILR1	17	64449037	64468643	0.088516311	PASS
MINDY1	1	150996086	151008375	0.103015788	PASS
MINDY2	15	58771192	58861900	-0.697600509	PASS
MINDY3	10	15778170	15860520	0.039811807	PASS
MINDY4	7	30771417	30892387	-0.69513118	PASS
MINDY4B	3	150871045	150905439	0.318278996	suppress_segment_too_large
MINK1	17	4833388	4898061	0.128406487	PASS
MINOS1	1	19484403	19629821	0.033763446	PASS
MINOS1-N	1	19597067	19656927	0.033763446	PASS
MINPP1	10	87504875	87553460	-1.64589449	suppress_large_homdel
MIOS	7	7566872	7608929	-0.69513118	PASS
MIOX	22	50486784	50490648	-0.808211634	PASS

MIP	12	56449502	56469166	-0.202191512	PASS
MIPEP	13	23730189	23889419	-0.390786075	PASS
MIPOL1	14	37197913	37552361	0.060212819	PASS
MIS12	17	5486285	5490814	0.128406487	PASS
MIS18A	21	32268219	32279069	0.12860881	suppress_segment_too_large
MIS18BP1	14	45203190	45253540	0.060212819	PASS
MISP	19	751126	764318	0.079632875	PASS
MISP3	19	14072536	14075062	0.093487731	suppress_segment_too_large
MITD1	2	99161427	99181058	0.048238121	PASS
MITF	3	69739435	69968337	-0.234617007	PASS
MIXL1	1	226223618	226227054	-0.256698369	PASS
MKI67	10	128096659	128126385	0.063874692	PASS
MKKS	20	10401009	10434222	-0.168668583	PASS
MKLN1	7	131110096	131496636	-0.388946609	PASS
MKNK1	1	46557408	46616843	0.033763446	PASS
MKNK2	19	2037465	2051244	0.079632875	PASS
MKRN1	7	140453040	140479569	-0.388946609	PASS
MKRN2	3	12557014	12583713	0.311018244	suppress_segment_too_large
MKRN2OS	3	12514934	12561059	0.311018244	suppress_segment_too_large
MKRN3	15	23565674	23630075	-0.396255771	PASS
MKS1	17	58205437	58219605	0.088516311	PASS
MKX	10	27672875	27746060	0.039811807	PASS
MLANA	9	5890802	5910606	-0.310444659	PASS
MLC1	22	50059391	50085902	-0.808211634	PASS
MLEC	12	120686869	120701864	0.202141228	PASS
MLF1	3	158571163	158607252	0.318278996	suppress_segment_too_large
MLF2	12	6747996	6767475	-0.086128028	PASS
MLH1	3	36993332	37050918	-0.234617007	PASS
MLH3	14	75013764	75051532	-0.192197564	PASS
MLIP	6	53929982	54266280	0.048820648	PASS
MLKL	16	74671855	74700960	-0.371357732	PASS
MLLT1	19	6210379	6279948	0.079632875	PASS
MLLT10	10	21524675	21743630	0.039811807	PASS
MLLT11	1	151057758	151068497	0.103015788	PASS
MLLT3	9	20341665	20622543	-0.310444659	PASS
MLLT6	17	38705542	38729803	0.060555011	PASS
MLN	6	33794673	33804011	0.048820648	PASS
MLNR	13	49220338	49222377	-0.390786075	PASS
MLPH	2	237485428	237555318	0.025928102	PASS
MLST8	16	2204248	2209416	0.069936844	PASS
MLX	17	42567068	42573239	0.088516311	PASS
MLXIP	12	122078722	122147347	0.202141228	PASS
MLXIPL	7	73593194	73624543	-0.262778904	PASS
MLYCD	16	83899126	83927026	-0.371357732	PASS
MMAA	4	145599042	145660035	-0.011382299	PASS
MMAB	12	109553737	109573874	0.202141228	PASS

MMACHC	1	45500053	45513382	0.033763446	PASS
MMADHC	2	149569634	149587816	-0.475215623	PASS
MMD	17	55392613	55421992	0.088516311	PASS
MMD2	7	4905989	4959213	-0.69513118	PASS
MME	3	155024124	155183729	0.318278996	suppress_segment_too_large
MMEL1	1	2590639	2632990	0.373964873	suppress_segment_too_large
MMGT1	23	135962070	135974063	-0.012567603	PASS
MMP1	11	102789920	102798160	0.062969153	PASS
MMP10	11	102770503	102780628	0.062969153	PASS
MMP11	22	23768226	23784316	-0.575930959	PASS
MMP12	11	102862736	102875034	0.062969153	PASS
MMP13	11	102942995	102955734	0.062969153	PASS
MMP14	14	22836557	22849027	0.060212819	PASS
MMP15	16	58025566	58046901	-0.371357732	PASS
MMP16	8	88032009	88328025	0.279445683	PASS
MMP17	12	131828393	131851783	0.270848837	PASS
MMP19	12	55835433	55842966	-0.202191512	PASS
MMP2	16	55389700	55506691	-0.371357732	PASS
MMP20	11	102576835	102625332	0.062969153	PASS
MMP21	10	125766453	125775821	0.063874692	PASS
MMP23B	1	1632095	1635263	0.373964873	suppress_segment_too_large
MMP24	20	35226654	35277000	-0.168668583	PASS
MMP24OS	20	35201745	35278131	-0.168668583	PASS
MMP25	16	3046681	3060726	0.069936844	PASS
MMP26	11	4704927	4992429	-0.957525224	PASS
MMP27	11	102691487	102705806	0.062969153	PASS
MMP28	17	35756249	35795707	0.060555011	PASS
MMP3	11	102835801	102843803	0.062969153	PASS
MMP7	11	102520508	102530753	0.062969153	PASS
MMP8	11	102711795	102727050	0.062969153	PASS
MMP9	20	46008908	46016561	-0.168668583	PASS
MMRN1	4	89879532	89954629	-0.011382299	PASS
MMRN2	10	86935540	86969481	-0.292453993	PASS
MMS19	10	97458324	97498794	-0.367947924	PASS
MMS22L	6	97142161	97283217	-0.368590549	PASS
MN1	22	27748277	27801498	-0.575930959	PASS
MNAT1	14	60734742	60969953	0.060212819	PASS
MND1	4	153344649	153415118	-0.011382299	PASS
MNDA	1	158831317	158849506	-0.467855874	PASS
MNS1	15	56421544	56465137	-0.697600509	PASS
MNT	17	2384060	2401118	0.128406487	PASS
MNX1	7	156994051	157010651	-0.411415677	PASS
MOAP1	14	93182196	93184928	-0.192197564	PASS
MOB1A	2	74152528	74178898	-0.372959126	PASS
MOB1B	4	70902326	71022449	-0.011382299	PASS
MOB2	11	1469457	1501247	-0.333835828	PASS

MOB3A	19	2071038	2096673	0.079632875	PASS
MOB3B	9	27325209	27529781	-0.310444659	PASS
MOB3C	1	46607715	46616891	0.033763446	PASS
MOB4	2	197515571	197553699	0.025928102	PASS
MOBP	3	39467198	39529479	-0.234617007	PASS
MOCOS	18	36187519	36272157	-0.376397843	PASS
MOCS1	6	39899578	39934551	0.048820648	PASS
MOCS2	5	53095679	53110063	-0.293443042	PASS
MOCS3	20	50958826	50963931	-0.168668583	PASS
MOG	6	29656981	29672372	0.048820648	PASS
MOGAT1	2	222671786	222709930	0.025928102	PASS
MOGAT2	11	75717819	75732958	0.062969153	PASS
MOGAT3	7	101195007	101201038	-0.388946609	PASS
MOGS	2	74461057	74465410	-0.372959126	PASS
MOK	14	102224500	102305200	-0.192197564	PASS
MON1A	3	49907160	49930173	-0.234617007	PASS
MON1B	16	77190835	77202405	-0.371357732	PASS
MON2	12	62466817	62600479	-0.202191512	PASS
MORC1	3	108958239	109118142	0.313454204	suppress_segment_too_large
MORC2	22	30925130	30968298	-0.575930959	PASS
MORC3	21	36320189	36386148	-0.064425456	suppress_segment_too_large
MORC4	23	106813871	107000244	-0.012567603	PASS
MORF4L1	15	78810487	78898133	-0.697600509	PASS
MORF4L2	23	103675496	103688158	-0.012567603	PASS
MORN1	1	2321253	2391707	0.373964873	suppress_segment_too_large
MORN2	2	38875962	38929072	0.040498426	PASS
MORN3	12	121648742	121672631	0.202141228	PASS
MORN4	10	97614553	97633500	-0.367947924	PASS
MORN5	9	122159908	122200088	0.365190713	suppress_segment_too_large
MOS	8	56112942	56113982	-0.201142258	PASS
MOSMO	16	22007638	22087534	0.069936844	PASS
MOSPD1	23	134887626	134915267	-0.012567603	PASS
MOSPD2	23	14873441	14922327	-0.012567603	PASS
MOSPD3	7	100612102	100615384	-0.262778904	PASS
MOV10	1	112673141	112700746	0.033763446	PASS
MOV10L1	22	50089879	50161690	-0.808211634	PASS
MOXD1	6	132296055	132401545	-0.458570262	PASS
MPC1	6	166364919	166383013	-0.035448111	PASS
MPC1L	23	40623566	40624136	-0.012567603	PASS
MPC2	1	167916729	167937040	-0.027073548	PASS
MPDU1	17	7583529	7592789	0.128406487	PASS
MPDZ	9	13105704	13279590	-0.310444659	PASS
MPEG1	11	59208510	59212951	-0.129266491	PASS
MPG	16	77007	85853	0.069936844	PASS
MPHOSPH	2	71130314	71150101	-0.372959126	PASS
MPHOSPH	16	82147798	82170226	-0.371357732	PASS

MPHOSPH	13	19633681	19673459	-0.390786075	PASS
MPHOSPH	12	123152320	123244014	0.202141228	PASS
MPI	15	74890005	74902219	-0.697600509	PASS
MPIG6B	6	31718594	31726714	0.048820648	PASS
MPL	1	43337849	43352772	0.033763446	PASS
MPLKIP	7	40126023	40134652	-0.69513118	PASS
MPND	19	4343527	4360086	0.079632875	PASS
MPO	17	58269856	58280935	0.088516311	PASS
MPP1	23	154778684	154821007	-0.012567603	PASS
MPP2	17	43875357	43909711	0.088516311	PASS
MPP3	17	43800799	43833170	0.088516311	PASS
MPP4	2	201644870	201698694	0.025928102	PASS
MPP5	14	67241109	67335819	0.060212819	PASS
MPP6	7	24573268	24694193	-0.69513118	PASS
MPP7	10	28050993	28334486	0.039811807	PASS
MPPE1	18	11882622	11909223	-0.052305056	suppress_segment_too_large
MPPED1	22	43411196	43507848	-0.575930959	PASS
MPPED2	11	30384493	30586872	-0.405444717	PASS
MPRIP	17	17042545	17217679	0.024610076	PASS
MPST	22	37019635	37029822	-0.575930959	PASS
MPV17	2	27309492	27325680	0.040498426	PASS
MPV17L	16	15395754	15413268	0.069936844	PASS
MPV17L2	19	18193182	18196948	0.093487731	suppress_segment_too_large
MPZ	1	161304735	161309972	-0.027073548	PASS
MPZL1	1	167721192	167791919	-0.027073548	PASS
MPZL2	11	118253403	118264536	-0.356188624	PASS
MPZL3	11	118226690	118252350	-0.356188624	PASS
MR1	1	181033425	181061938	-0.027073548	PASS
MRAP	21	32291813	32314784	0.12860881	suppress_segment_too_large
MRAP2	6	84033756	84090881	-0.368590549	PASS
MRAS	3	138347648	138405534	0.313454204	suppress_segment_too_large
MRC1	10	17809344	17911170	0.039811807	PASS
MRC2	17	62627401	62693597	0.088516311	PASS
MRE11	11	94415578	94493908	0.062969153	PASS
MREG	2	215942805	216034096	0.025928102	PASS
MRFAP1	4	6640091	6642745	0.279089364	PASS
MRFAP1L	4	6707701	6709880	0.279089364	PASS
MRGBP	20	62796453	62801738	-0.168668583	PASS
MRGPRD	11	68980021	68980986	0.182439925	suppress_segment_too_large
MRGPRE	11	3227698	3232386	-0.333835828	PASS
MRGPRF	11	69004395	69013409	0.182439925	suppress_segment_too_large
MRGPRG	11	3217944	3218813	-0.333835828	PASS
MRGPRX1	11	18933813	18939507	-0.405444717	PASS
MRGPRX2	11	19054455	19060681	-0.405444717	PASS
MRGPRX3	11	18120955	18138480	-0.405444717	PASS
MRGPRX4	11	18172837	18174280	-0.405444717	PASS

MRI1	19	13764532	13774282	0.093487731	suppress_segment_too_large
MRLN	10	59736692	59756041	0.072405807	PASS
MRM1	17	36601572	36608971	0.060555011	PASS
MRM2	7	2234231	2242198	-0.69513118	PASS
MRM3	17	782273	792509	0.128406487	PASS
MRNIP	5	179835133	179862173	-0.150979337	PASS
MRO	18	50795120	50825402	-0.376397843	PASS
MROH1	8	144148016	144261940	0.307788765	PASS
MROH2A	2	233775679	233833423	0.025928102	PASS
MROH2B	5	40998017	41071342	-0.293443042	PASS
MROH6	8	143566187	143572971	0.307788765	PASS
MROH7	1	54641754	54710266	0.033763446	PASS
MROH7-T'	1	54641786	54742308	0.033763446	PASS
MROH8	20	37101226	37179588	-0.168668583	PASS
MROH9	1	170935471	171064765	-0.027073548	PASS
MRPL1	4	77862520	77952790	-0.011382299	PASS
MRPL10	17	47823272	47831534	0.088516311	PASS
MRPL11	11	66435075	66466738	0.182439925	suppress_segment_too_large
MRPL12	17	81703357	81707526	0.088516311	PASS
MRPL13	8	120380761	120445402	0.279445683	PASS
MRPL14	6	44113454	44127457	0.048820648	PASS
MRPL15	8	54135210	54147901	-0.201142258	PASS
MRPL16	11	59806135	59810872	-0.129266491	PASS
MRPL17	11	6680782	6683401	-0.405444717	PASS
MRPL18	6	159789812	159798436	-0.035448111	PASS
MRPL19	2	75646783	75690851	-0.372959126	PASS
MRPL2	6	43054029	43059806	0.048820648	PASS
MRPL20	1	1401908	1407313	0.373964873	suppress_segment_too_large
MRPL21	11	68891276	68903835	0.182439925	suppress_segment_too_large
MRPL22	5	154941070	154969411	-0.293443042	PASS
MRPL23	11	1947278	1984522	-0.333835828	PASS
MRPL24	1	156737303	156741590	0.103015788	PASS
MRPL27	17	50367857	50373214	0.088516311	PASS
MRPL28	16	366969	371289	0.069936844	PASS
MRPL3	3	131462212	131502983	0.313454204	suppress_segment_too_large
MRPL30	2	99181079	99197626	0.048238121	PASS
MRPL32	7	42932200	42948958	-0.69513118	PASS
MRPL33	2	27771717	27988087	0.040498426	PASS
MRPL34	19	17292609	17306843	0.093487731	suppress_segment_too_large
MRPL35	2	86199355	86213794	0.066915641	PASS
MRPL36	5	1798386	1801366	0.055007813	PASS
MRPL37	1	54184041	54225464	0.033763446	PASS
MRPL38	17	75898643	75905413	0.088516311	PASS
MRPL39	21	25585656	25607517	0.12860881	suppress_segment_too_large
MRPL4	19	10251901	10260045	0.093487731	suppress_segment_too_large
MRPL40	22	19431902	19436075	-0.575930959	PASS

MRPL41	9	137551199	137552555	0.365190713	suppress_segment_too_large
MRPL42	12	93467488	93516213	0.202141228	PASS
MRPL43	10	100969458	100987515	-0.367947924	PASS
MRPL44	2	223957404	223967714	0.025928102	PASS
MRPL45	17	38297023	38323218	0.060555011	PASS
MRPL46	15	88459476	88467419	-0.697600509	PASS
MRPL47	3	179588285	179604654	0.318278996	suppress_segment_too_large
MRPL48	11	73787316	73865133	0.062969153	PASS
MRPL49	11	65122183	65127371	0.182439925	suppress_segment_too_large
MRPL50	9	101387633	101398614	0.365190713	suppress_segment_too_large
MRPL51	12	6491886	6493841	-0.086128028	PASS
MRPL52	14	22829879	22835037	0.060212819	PASS
MRPL53	2	74471958	74473322	-0.372959126	PASS
MRPL54	19	3762664	3768575	0.079632875	PASS
MRPL55	1	228106679	228109312	-0.256698369	PASS
MRPL57	13	21176645	21179084	-0.390786075	PASS
MRPL58	17	75012670	75021261	0.088516311	PASS
MRPL9	1	151759643	151763564	0.103015788	PASS
MRPS10	6	42206801	42217865	0.048820648	PASS
MRPS11	15	88467453	88480773	-0.697600509	PASS
MRPS12	19	38930548	38933162	0.093487731	suppress_segment_too_large
MRPS14	1	175010789	175023425	-0.027073548	PASS
MRPS15	1	36455718	36464437	0.033763446	PASS
MRPS16	10	73248843	73252693	0.072405807	PASS
MRPS17	7	55951819	55956501	-0.750061359	PASS
MRPS18A	6	43671303	43687791	0.048820648	PASS
MRPS18B	6	30617709	30626395	0.048820648	PASS
MRPS18C	4	83455932	83469735	-0.011382299	PASS
MRPS2	9	135499984	135504673	0.365190713	suppress_segment_too_large
MRPS21	1	150293720	150308979	0.103015788	PASS
MRPS22	3	139005806	139357223	0.313454204	suppress_segment_too_large
MRPS23	17	57834781	57850056	0.088516311	PASS
MRPS24	7	43866558	43869893	-0.69513118	PASS
MRPS25	3	15042460	15065335	0.311018244	suppress_segment_too_large
MRPS26	20	3045945	3048254	-0.168668583	PASS
MRPS27	5	72219409	72320646	-0.293443042	PASS
MRPS28	8	79918717	80030289	0.279445683	PASS
MRPS30	5	44808925	44820428	-0.293443042	PASS
MRPS31	13	40729135	40771173	-0.390786075	PASS
MRPS33	7	141002610	141015228	-0.388946609	PASS
MRPS34	16	1771890	1773155	0.069936844	PASS
MRPS35	12	27710773	27756295	0.362961408	suppress_segment_too_large
MRPS36	5	69217760	69230129	-0.293443042	PASS
MRPS5	2	95087207	95149434	0.048238121	PASS
MRPS6	21	34073224	34143034	0.12860881	suppress_segment_too_large
MRPS7	17	75261674	75266373	0.088516311	PASS

MRPS9	2	105037983	105099960	-0.532032081	PASS
MRRF	9	122264603	122331343	0.365190713	suppress_segment_too_large
MRS2	6	24402908	24426194	0.048820648	PASS
MRTFA	22	40410281	40636702	-0.575930959	PASS
MRTFB	16	14071321	14266773	0.069936844	PASS
MRTO4	1	19251539	19260128	0.033763446	PASS
MRVI1	11	10573091	10693988	-0.405444717	PASS
MS4A1	11	60455752	60470760	-0.129266491	PASS
MS4A10	11	60785348	60801305	-0.129266491	PASS
MS4A12	11	60492778	60507430	-0.129266491	PASS
MS4A13	11	60515413	60542721	-0.129266491	PASS
MS4A14	11	60378530	60417756	-0.129266491	PASS
MS4A15	11	60756953	60776732	-0.129266491	PASS
MS4A18	11	60729304	60744212	-0.129266491	PASS
MS4A2	11	60088261	60098466	-0.129266491	PASS
MS4A3	11	60056587	60071128	-0.129266491	PASS
MS4A4A	11	60185702	60317944	-0.129266491	PASS
MS4A4E	11	60201253	60243088	-0.129266491	PASS
MS4A5	11	60429589	60455214	-0.129266491	PASS
MS4A6A	11	60172014	60184666	-0.129266491	PASS
MS4A6E	11	60334831	60396596	-0.129266491	PASS
MS4A7	11	60378482	60395951	-0.129266491	PASS
MS4A8	11	60699574	60715811	-0.129266491	PASS
MSANTD1	4	3244369	3271738	0.279089364	PASS
MSANTD2	11	124766498	124800673	-0.419612865	PASS
MSANTD3	9	100427156	100451711	0.365190713	suppress_segment_too_large
MSANTD3	9	100442271	100577636	0.365190713	suppress_segment_too_large
MSANTD4	11	105995623	106022403	-0.356188624	PASS
MSC	8	71841549	71844468	-0.201142258	PASS
MSGN1	2	17816496	17817101	0.040498426	PASS
MSH2	2	47402969	47663146	0.040498426	PASS
MSH3	5	80654648	80876460	-0.293443042	PASS
MSH4	1	75796882	75913238	0.033763446	PASS
MSH5	6	31739948	31762834	0.048820648	PASS
MSH5-SAI	6	31740020	31764851	0.048820648	PASS
MSH6	2	47695530	47810101	0.040498426	PASS
MSI1	12	120341330	120369180	0.202141228	PASS
MSI2	17	57255851	57684685	0.088516311	PASS
MSL1	17	40122298	40136916	0.060555011	PASS
MSL2	3	136148922	136197241	0.313454204	suppress_segment_too_large
MSL3	23	11758159	11775772	-0.012567603	PASS
MSLN	16	760762	768865	0.069936844	PASS
MSLNL	16	769428	783370	0.069936844	PASS
MSMB	10	46033307	46046269	-0.404852538	PASS
MSMO1	4	165327623	165343160	-0.011382299	PASS
MSMP	9	35752990	35756613	-0.310444659	PASS

MSN	23	65588377	65741931	-0.012567603	PASS
MSR1	8	16107878	16567490	-0.345962679	PASS
MSRA	8	10054268	10428891	-0.345962679	PASS
MSRB1	16	1938210	1943326	0.069936844	PASS
MSRB2	10	23095506	23122013	0.039811807	PASS
MSRB3	12	65278643	65491430	-0.202191512	PASS
MSS51	10	73423579	73433561	0.072405807	PASS
MST1	3	49683947	49689501	-0.234617007	PASS
MST1R	3	49887002	49903873	-0.234617007	PASS
MSTN	2	190055697	190062729	0.025928102	PASS
MSTO1	1	155610205	155614967	0.103015788	PASS
MSX1	4	4859666	4863936	0.279089364	PASS
MSX2	5	174724533	174730893	-0.150979337	PASS
MT1A	16	56638666	56640087	-0.371357732	PASS
MT1B	16	56651899	56653204	-0.371357732	PASS
MT1E	16	56625475	56627112	-0.371357732	PASS
MT1F	16	56657694	56660698	-0.371357732	PASS
MT1G	16	56666731	56668065	-0.371357732	PASS
MT1H	16	56669814	56671129	-0.371357732	PASS
MT1HL1	1	237004103	237004418	-0.648598641	PASS
MT1M	16	56632233	56633986	-0.371357732	PASS
MT1X	16	56682424	56684196	-0.371357732	PASS
MT2A	16	56608199	56609497	-0.371357732	PASS
MT3	16	56589074	56591088	-0.371357732	PASS
MT4	16	56565049	56568957	-0.371357732	PASS
MTA1	14	105419820	105470729	-0.192197564	PASS
MTA2	11	62593214	62601840	0.182439925	suppress_segment_too_large
MTA3	2	42494569	42756947	0.040498426	PASS
MTAP	9	21802543	21937651	-0.310444659	PASS
MTBP	8	120445400	120542133	0.279445683	PASS
MTCH1	6	36968141	36986298	0.048820648	PASS
MTCH2	11	47617315	47642623	0.117376172	PASS
MTCL1	18	8705661	8832778	-0.052305056	suppress_segment_too_large
MTCP1	23	155061622	155147937	-0.012567603	PASS
MTDH	8	97644179	97728770	0.279445683	PASS
MTERF1	7	91692008	91880720	-0.262778904	PASS
MTERF2	12	106977291	106987166	0.202141228	PASS
MTERF3	8	96239398	96261610	0.279445683	PASS
MTERF4	2	241072169	241102332	0.025928102	PASS
MTF1	1	37809567	37859620	0.033763446	PASS
MTF2	1	93079235	93139081	0.033763446	PASS
MTFMT	15	65001512	65029639	-0.697600509	PASS
MTFP1	22	30425530	30429053	-0.575930959	PASS
MTFR1	8	65644734	65771261	-0.201142258	PASS
MTFR1L	1	25818640	25832942	0.033763446	PASS
MTFR2	6	136231024	136250335	-0.035448111	PASS

MTG1	10	133394094	133421307	0.063874692	PASS
MTG2	20	62183029	62203568	-0.168668583	PASS
MTHFD1	14	64388031	64463457	0.060212819	PASS
MTHFD1L	6	150865549	151101887	-0.035448111	PASS
MTHFD2	2	74198562	74217565	-0.372959126	PASS
MTHFD2L	4	74114174	74303099	-0.011382299	PASS
MTHFR	1	11785723	11806920	0.373964873	suppress_segment_too_large
MTHFS	15	79833585	79897379	-0.697600509	PASS
MTHFSD	16	86530176	86555235	-0.371357732	PASS
MTIF2	2	55236595	55269347	0.040498426	PASS
MTIF3	13	27435643	27450591	-0.390786075	PASS
MTLN	2	110211529	110245420	-0.010901494	PASS
MTM1	23	150568619	150673322	-0.012567603	PASS
MTMR1	23	150692971	150765103	-0.012567603	PASS
MTMR10	15	30938941	30991607	-0.396255771	PASS
MTMR11	1	149928651	149936869	0.103015788	PASS
MTMR12	5	32226994	32313009	0.055007813	PASS
MTMR14	3	9649433	9702393	0.311018244	suppress_segment_too_large
MTMR2	11	95832882	95925315	0.062969153	PASS
MTMR3	22	29883155	30030866	-0.575930959	PASS
MTMR4	17	58489529	58517905	0.088516311	PASS
MTMR6	13	25246201	25288009	-0.390786075	PASS
MTMR7	8	17298030	17413528	-0.345962679	PASS
MTMR8	23	64268081	64395431	-0.012567603	PASS
MTMR9	8	11284416	11328146	-0.345962679	PASS
MTNR1A	4	186533655	186555567	-0.462744716	PASS
MTNR1B	11	92969720	92985066	0.062969153	PASS
MTO1	6	73461578	73509236	-0.368590549	PASS
MTOR	1	11106535	11262507	0.373964873	suppress_segment_too_large
MTPAP	10	30309801	30374448	0.039811807	PASS
MTPN	7	135926761	135977353	-0.388946609	PASS
MTR	1	236795281	236903981	-0.256698369	PASS
MTREX	5	55307760	55425581	-0.293443042	PASS
MTRF1	13	41216369	41263577	-0.390786075	PASS
MTRF1L	6	152987362	153002685	-0.035448111	PASS
MTRR	5	7851186	7906025	0.055007813	PASS
MTSS1	8	124550790	124728429	0.279445683	PASS
MTSS1L	16	70661204	70686066	-0.371357732	PASS
MTTP	4	99563761	99623999	-0.011382299	PASS
MTURN	7	30134810	30162762	-0.69513118	PASS
MTUS1	8	17643795	17800917	-0.345962679	PASS
MTUS2	13	28820348	29505947	-0.390786075	PASS
MTX1	1	155208699	155213824	0.103015788	PASS
MTX2	2	176269395	176338025	0.039726562	PASS
MTX3	5	79976731	79991265	-0.293443042	PASS
MUC1	1	155185824	155192916	0.103015788	PASS

MUC12	7	100969623	101018949	-0.262778904	PASS
MUC13	3	124905442	124953819	0.313454204	suppress_segment_too_large
MUC15	11	26559033	26572233	-0.405444717	PASS
MUC16	19	8848844	8981342	0.079632875	PASS
MUC17	7	101020072	101058745	-0.388946609	PASS
MUC19	12	40393395	40570832	-0.202191512	PASS
MUC2	11	1074875	1110511	-0.333835828	PASS
MUC20	3	195720882	195741123	0.326834516	PASS
MUC21	6	30983718	30989903	0.048820648	PASS
MUC22	6	31010474	31035402	0.048820648	PASS
MUC3A	7	100949555	100968346	-0.262778904	PASS
MUC4	3	195746765	195812277	0.326834516	PASS
MUC5AC	11	1157953	1201138	-0.333835828	PASS
MUC5B	11	1223066	1262172	-0.333835828	PASS
MUC6	11	1012821	1036706	-0.333835828	PASS
MUC7	4	70430492	70482997	-0.011382299	PASS
MUCL1	12	54830519	54858393	-0.202191512	PASS
MUCL3	6	30934523	30954221	0.048820648	PASS
MUL1	1	20499448	20508161	0.033763446	PASS
MUM1	19	1354711	1378431	0.079632875	PASS
MUM1L1	23	106168305	106208956	-0.012567603	PASS
MUS81	11	65857126	65867653	0.182439925	suppress_segment_too_large
MUSK	9	110668771	110801620	0.365190713	suppress_segment_too_large
MUSTN1	3	52833114	52835219	-0.234617007	PASS
MUT	6	49430360	49463191	0.048820648	PASS
MUTYH	1	45329163	45340470	0.033763446	PASS
MVB12A	19	17405722	17433724	0.093487731	suppress_segment_too_large
MVB12B	9	126326849	126507041	0.365190713	suppress_segment_too_large
MVD	16	88651935	88663161	-0.371357732	PASS
MVK	12	109573255	109598117	0.202141228	PASS
MVP	16	29820394	29848039	0.069936844	PASS
MX1	21	41420304	41459214	-0.064425456	suppress_segment_too_large
MX2	21	41361943	41409390	-0.064425456	suppress_segment_too_large
MXD1	2	69897688	69942945	-0.372959126	PASS
MXD3	5	177301461	177312757	-0.150979337	PASS
MXD4	4	2247432	2262294	0.279089364	PASS
MXI1	10	110207605	110287365	0.063874692	PASS
MXRA5	23	3308565	3346641	-0.012567603	PASS
MXRA7	17	76672551	76711016	0.088516311	PASS
MXRA8	1	1352689	1361777	0.373964873	suppress_segment_too_large
MYADM	19	53866223	53876437	0.206912349	PASS
MYADML	17	81939645	81947233	0.088516311	PASS
MYB	6	135181315	135219173	-0.035448111	PASS
MYBBP1A	17	4538897	4555631	0.128406487	PASS
MYBL1	8	66562175	66614247	-0.201142258	PASS
MYBL2	20	43667019	43716496	-0.168668583	PASS

MYBPC1	12	101568353	101686018	0.202141228	PASS
MYBPC2	19	50432903	50466321	-0.016761391	PASS
MYBPC3	11	47331397	47352702	0.117376172	PASS
MYBPH	1	203167811	203175813	-0.256698369	PASS
MYBPHL	1	109292365	109307041	0.033763446	PASS
MYC	8	127735434	127741434	0.113969953	PASS
MYCBP	1	38862964	38874105	0.033763446	PASS
MYCBP2	13	77044655	77327050	-0.390786075	PASS
MYCBPAI	17	50508384	50531501	0.088516311	PASS
MYCL	1	39895426	39902256	0.033763446	PASS
MYCN	2	15940564	15947007	0.040498426	PASS
MYCT1	6	152697895	152724567	-0.035448111	PASS
MYD88	3	38138478	38143022	-0.234617007	PASS
MYDGF	19	4641374	4670370	0.079632875	PASS
MYEF2	15	48134631	48178517	-0.396255771	PASS
MYEOV	11	69294138	69367726	0.182439925	suppress_segment_too_large
MYF5	12	80716912	80719673	0.202141228	PASS
MYF6	12	80707498	80709474	0.202141228	PASS
MYH1	17	10492307	10518543	-0.139031004	PASS
MYH10	17	8474205	8630761	0.128406487	PASS
MYH11	16	15703172	15857033	0.069936844	PASS
MYH13	17	10300865	10373130	-0.139031004	PASS
MYH14	19	50188186	50310544	-0.016761391	PASS
MYH15	3	108380369	108529322	0.313454204	suppress_segment_too_large
MYH2	17	10521148	10549957	-0.139031004	PASS
MYH3	17	10628526	10657309	-0.139031004	PASS
MYH4	17	10443290	10469559	-0.139031004	PASS
MYH6	14	23381990	23408277	0.060212819	PASS
MYH7	14	23412738	23435718	0.060212819	PASS
MYH7B	20	34975403	35002437	-0.168668583	PASS
MYH8	17	10390322	10421950	-0.139031004	PASS
MYH9	22	36281277	36388067	-0.575930959	PASS
MYL1	2	210290150	210315190	0.025928102	PASS
MYL10	7	101613325	101629296	-0.388946609	PASS
MYL12A	18	3247481	3256236	-0.052305056	suppress_segment_too_large
MYL12B	18	3261479	3278431	-0.052305056	suppress_segment_too_large
MYL2	12	110910819	110920722	0.202141228	PASS
MYL3	3	46857872	46882169	-0.234617007	PASS
MYL4	17	47200446	47223679	0.088516311	PASS
MYL5	4	673580	682033	0.279089364	PASS
MYL6	12	56158161	56163496	-0.202191512	PASS
MYL6B	12	56152256	56159647	-0.202191512	PASS
MYL7	7	44138864	44141332	-0.69513118	PASS
MYL9	20	36541484	36551447	-0.168668583	PASS
MYLIP	6	16129125	16148248	0.048820648	PASS
MYLK	3	123610049	123884331	0.313454204	suppress_segment_too_large

MYLK2	20	31819308	31834689	-0.168668583	PASS
MYLK3	16	46703369	46790407	0.069936844	PASS
MYLK4	6	2663629	2750966	0.048820648	PASS
MYLPF	16	30370934	30377991	0.069936844	PASS
MYMK	9	133514586	133528612	0.365190713	suppress_segment_too_large
MYMX	6	44216939	44218236	0.048820648	PASS
MYNN	3	169772831	169789716	0.318278996	suppress_segment_too_large
MYO10	5	16661914	16936276	0.055007813	PASS
MYO15A	17	18108706	18179806	0.024610076	PASS
MYO15B	17	75588058	75626501	0.088516311	PASS
MYO16	13	108596152	109208007	-0.390786075	PASS
MYO18A	17	29071124	29180412	0.060555011	PASS
MYO18B	22	25742144	26031041	-0.575930959	PASS
MYO19	17	36495633	36543435	0.060555011	PASS
MYO1A	12	57028517	57051198	-0.202191512	PASS
MYO1B	2	191245185	191425389	0.025928102	PASS
MYO1C	17	1464186	1492812	0.128406487	PASS
MYO1D	17	32492522	32877177	0.060555011	PASS
MYO1E	15	59132434	59372900	-0.697600509	PASS
MYO1F	19	8520790	8577577	0.079632875	PASS
MYO1G	7	44962662	44979098	-0.69513118	PASS
MYO1H	12	109347903	109455523	0.202141228	PASS
MYO3A	10	25934071	26212536	0.039811807	PASS
MYO3B	2	170178145	170655171	0.039726562	PASS
MYO5A	15	52307283	52529050	-0.396255771	PASS
MYO5B	18	49822813	50195093	-0.376397843	PASS
MYO5C	15	52192322	52295798	-0.396255771	PASS
MYO6	6	75749192	75919537	-0.368590549	PASS
MYO7A	11	77128264	77215241	0.062969153	PASS
MYO7B	2	127535802	127637729	-0.475215623	PASS
MYO9A	15	71822289	72118577	-0.697600509	PASS
MYO9B	19	17075781	17214537	0.093487731	suppress_segment_too_large
MYOC	1	171635417	171652683	-0.027073548	PASS
MYOCD	17	12665890	12768949	-0.139031004	PASS
MYOCOS	1	171600621	171638799	-0.027073548	PASS
MYOD1	11	17719568	17722131	-0.405444717	PASS
MYOF	10	93306429	93482317	-0.39678585	PASS
MYOG	1	203083132	203086036	-0.256698369	PASS
MYOM1	18	3066807	3220108	-0.052305056	suppress_segment_too_large
MYOM2	8	2045040	2165552	-0.313874214	PASS
MYOM3	1	24056035	24112175	0.033763446	PASS
MYORG	9	34366670	34376853	-0.310444659	PASS
MYOT	5	137867791	137887851	-0.293443042	PASS
MYOZ1	10	73631654	73641757	0.072405807	PASS
MYOZ2	4	119135784	119187789	-0.011382299	PASS
MYOZ3	5	150660874	150679365	-0.293443042	PASS

MYPN	10	68106117	68212017	0.072405807	PASS
MYPOP	19	45890020	45902604	0.14996833	PASS
MYRF	11	61752642	61788518	0.182439925	suppress_segment_too_large
MYRFL	12	69825304	69959097	-0.202191512	PASS
MYRIP	3	39808914	40260321	-0.234617007	PASS
MYSM1	1	58654739	58700092	0.033763446	PASS
MYT1	20	64102394	64242253	-0.168668583	PASS
MYT1L	2	1789113	2331664	0.040498426	PASS
MYZAP	15	57591941	57685364	-0.697600509	PASS
MZB1	5	139387480	139390081	-0.293443042	PASS
MZF1	19	58561931	58573575	0.063781462	PASS
MZT1	13	72708357	72727687	-0.390786075	PASS
MZT2A	2	131464900	131492743	-0.475215623	PASS
MZT2B	2	130181737	130190729	-0.475215623	PASS
N4BP1	16	48538726	48620148	0.069936844	PASS
N4BP2	4	40056826	40158252	-0.011382299	PASS
N4BP2L1	13	32400723	32428311	-0.390786075	PASS
N4BP2L2	13	32432417	32538885	-0.390786075	PASS
N4BP3	5	178113443	178126087	-0.150979337	PASS
N6AMT1	21	28872191	28885371	0.12860881	suppress_segment_too_large
NAA10	23	153929242	153935223	-0.012567603	PASS
NAA11	4	79225694	79326050	-0.011382299	PASS
NAA15	4	139301455	139420033	-0.011382299	PASS
NAA16	13	41311205	41377030	-0.390786075	PASS
NAA20	20	20017116	20033655	-0.168668583	PASS
NAA25	12	112026689	112109022	0.202141228	PASS
NAA30	14	57390544	57415917	0.060212819	PASS
NAA35	9	85941146	86022298	0.365190713	suppress_segment_too_large
NAA38	17	7856685	7885238	0.128406487	PASS
NAA40	11	63938959	63957328	0.182439925	suppress_segment_too_large
NAA50	3	113716460	113746300	0.313454204	suppress_segment_too_large
NAA60	16	3443611	3486963	0.069936844	PASS
NAA80	3	50296402	50299421	-0.234617007	PASS
NAAA	4	75913657	75941051	-0.011382299	PASS
NAALAD2	11	90131515	90192894	0.062969153	PASS
NAALADI	11	65044818	65058549	0.182439925	suppress_segment_too_large
NAALADI	3	174438573	175810552	0.318278996	suppress_segment_too_large
NAB1	2	190646746	190692766	0.025928102	PASS
NAB2	12	57088894	57095476	-0.202191512	PASS
NABP1	2	191678068	191696659	0.025928102	PASS
NABP2	12	56222015	56229854	-0.202191512	PASS
NACA	12	56712428	56731628	-0.202191512	PASS
NACA2	17	61590426	61591202	0.088516311	PASS
NACAD	7	45080438	45088914	-0.69513118	PASS
NACC1	19	13118103	13141141	0.093487731	suppress_segment_too_large
NACC2	9	136006537	136095285	0.365190713	suppress_segment_too_large

NADK	1	1751232	1780457	0.373964873	suppress_segment_too_large
NADK2	5	36192592	36242279	0.055007813	PASS
NADSYN1	11	71453109	71524107	0.182439925	suppress_segment_too_large
NAE1	16	66802875	66873256	-0.371357732	PASS
NAF1	4	163110073	163166921	-0.011382299	PASS
NAGA	22	42058354	42070842	-0.575930959	PASS
NAGK	2	71064344	71079805	-0.372959126	PASS
NAGLU	17	42536172	42544449	0.088516311	PASS
NAGPA	16	5024844	5034141	0.069936844	PASS
NAGS	17	44004546	44009063	0.088516311	PASS
NAIF1	9	128061233	128068206	0.365190713	suppress_segment_too_large
NAIP	5	70968483	71025114	-0.293443042	PASS
NALCN	13	101053776	101416492	-0.390786075	PASS
NAMPT	7	106248285	106286326	-0.388946609	PASS
NANOG	12	7787794	7799141	-0.086128028	PASS
NANOGNI	12	7765216	7774121	-0.086128028	PASS
NANOGP8	15	35084193	35085110	-0.396255771	PASS
NANOS1	10	119029716	119033732	0.063874692	PASS
NANOS2	19	45913214	45914870	0.14996833	PASS
NANOS3	19	13862063	13880757	0.093487731	suppress_segment_too_large
NANP	20	25612935	25624175	-0.168668583	PASS
NANS	9	98056739	98083075	0.365190713	suppress_segment_too_large
NAP1L1	12	76036587	76085033	0.202141228	PASS
NAP1L2	23	73212299	73214848	-0.012567603	PASS
NAP1L3	23	93670930	93673568	-0.012567603	PASS
NAP1L4	11	2944431	2992377	-0.333835828	PASS
NAP1L5	4	88695915	88697829	-0.011382299	PASS
NAPA	19	47487637	47515240	0.14996833	PASS
NAPB	20	23374519	23421519	-0.168668583	PASS
NAPEPLD	7	103099776	103149560	-0.388946609	PASS
NAPG	18	10525905	10552761	-0.052305056	suppress_segment_too_large
NAPRT	8	143574785	143578649	0.307788765	PASS
NAPSA	19	50358477	50365830	-0.016761391	PASS
NARF	17	82458180	82490537	0.088516311	PASS
NARS	18	57600656	57622213	-0.376397843	PASS
NARS2	11	78435961	78574874	0.062969153	PASS
NASP	1	45583846	45618904	0.033763446	PASS
NAT1	8	18170477	18223689	-0.345962679	PASS
NAT10	11	34105602	34147670	-0.405444717	PASS
NAT14	19	55485004	55487568	0.121336758	PASS
NAT16	7	101170493	101180276	-0.388946609	PASS
NAT2	8	18391245	18401218	-0.345962679	PASS
NAT8	2	73640832	73642393	-0.372959126	PASS
NAT8L	4	2059512	2069089	0.279089364	PASS
NAT9	17	74770547	74776367	0.088516311	PASS
NATD1	17	21238870	21253410	0.024610076	PASS

NAV1	1	201622885	201826969	-0.256698369	PASS
NAV2	11	19350724	20121598	-0.405444717	PASS
NAV3	12	77324641	78213008	0.202141228	PASS
NAXD	13	110615460	110639993	-0.390786075	PASS
NAXE	1	156591762	156594299	0.103015788	PASS
NBAS	2	15166909	15561330	0.040498426	PASS
NBDY	23	56729241	56818380	-0.012567603	PASS
NBEA	13	34942287	35673022	-0.390786075	PASS
NBEAL1	2	203014879	203226378	0.025928102	PASS
NBEAL2	3	46979683	47009703	-0.234617007	PASS
NBL1	1	19596979	19658456	0.033763446	PASS
NBN	8	89933336	90003228	0.279445683	PASS
NBPF1	1	16562319	16613562	0.033763446	PASS
NBPF10	1	146064699	146144804	-0.157385092	PASS
NBPF11	1	148102046	148152322	-0.157385092	PASS
NBPF12	1	146938744	146996202	-0.157385092	PASS
NBPF14	1	148531385	148679751	-0.157385092	PASS
NBPF15	1	144421386	144461674	-0.157385092	PASS
NBPF19	1	149390623	149556361	-0.157385092	PASS
NBPF20	1	145289900	145405778	-0.157385092	PASS
NBPF26	1	120723923	120841481	-0.157385092	PASS
NBPF3	1	21440128	21485005	0.033763446	PASS
NBPF4	1	108223341	108244081	0.033763446	PASS
NBPF6	1	108450282	108471002	0.033763446	PASS
NBPF9	1	149054027	149103561	-0.157385092	PASS
NBR1	17	43170481	43211689	0.088516311	PASS
NCALD	8	101686543	102124907	0.279445683	PASS
NCAM1	11	112961247	113278436	-0.356188624	PASS
NCAM2	21	20998315	21543329	0.12860881	suppress_segment_too_large
NCAN	19	19211973	19252233	0.093487731	suppress_segment_too_large
NCAPD2	12	6493356	6531955	-0.086128028	PASS
NCAPD3	11	134150119	134225454	-0.419612865	PASS
NCAPG	4	17810902	17844862	0.279089364	PASS
NCAPG2	7	158631311	158704829	-0.411415677	PASS
NCAPH	2	96335787	96373845	0.048238121	PASS
NCAPH2	22	50508216	50523472	-0.808211634	PASS
NCBP1	9	97633626	97673748	0.365190713	suppress_segment_too_large
NCBP2	3	196935402	196942597	0.326834516	PASS
NCBP2-AS	3	196942623	196943540	0.326834516	PASS
NCBP2L	23	107774899	107795829	-0.012567603	PASS
NCBP3	17	3802165	3846251	0.128406487	PASS
NCCRP1	19	39196961	39201884	0.093487731	suppress_segment_too_large
NCDN	1	35557473	35567274	0.033763446	PASS
NCEH1	3	172630249	172711218	0.318278996	suppress_segment_too_large
NCF1	7	74773962	74789315	-0.262778904	PASS
NCF2	1	183555563	183590876	-0.027073548	PASS

NCF4	22	36860988	36878015	-0.575930959	PASS
NCK1	3	136862208	136949823	0.313454204	suppress_segment_too_large
NCK2	2	105744897	105894274	-0.532032081	PASS
NCKAP1	2	182909115	183038858	-0.169541924	PASS
NCKAP1L	12	54497711	54548238	-0.202191512	PASS
NCKAP5	2	132671799	133568463	-0.475215623	PASS
NCKAP5L	12	49791146	49828750	-0.202191512	PASS
NCKIPSD	3	48673844	48686364	-0.234617007	PASS
NCL	2	231453531	231483641	0.025928102	PASS
NCLN	19	3185563	3209575	0.079632875	PASS
NCMAP	1	24556111	24609328	0.033763446	PASS
NCOA1	2	24491914	24770702	0.040498426	PASS
NCOA2	8	70109762	70403805	-0.201142258	PASS
NCOA3	20	47501902	47656877	-0.168668583	PASS
NCOA4	10	46005088	46030714	-0.404852538	PASS
NCOA5	20	46060985	46089952	-0.168668583	PASS
NCOA6	20	34689097	34825649	-0.168668583	PASS
NCOA7	6	125781161	125932030	-0.458570262	PASS
NCOR1	17	16029157	16218185	0.024610076	PASS
NCOR2	12	124324415	124567589	0.202141228	PASS
NCR1	19	54906150	54916140	-0.146906334	PASS
NCR2	6	41335655	41350887	0.048820648	PASS
NCR3	6	31588895	31592985	0.048820648	PASS
NCR3LG1	11	17351726	17377341	-0.405444717	PASS
NCS1	9	130172578	130237304	0.365190713	suppress_segment_too_large
NCSTN	1	160343272	160358952	-0.027073548	PASS
NDC1	1	53765460	53838860	0.033763446	PASS
NDC80	18	2571511	2616635	-0.052305056	suppress_segment_too_large
NDE1	16	15643267	15726353	0.069936844	PASS
NDEL1	17	8413131	8490411	0.128406487	PASS
NDFIP1	5	142108505	142154443	-0.293443042	PASS
NDFIP2	13	79481124	79556075	-0.390786075	PASS
NDN	15	23685400	23687330	-0.396255771	PASS
NDNF	4	121035613	121073021	-0.011382299	PASS
NDOR1	9	137205685	137217009	0.365190713	suppress_segment_too_large
NDP	23	43948776	43973504	-0.012567603	PASS
NDRG1	8	133237171	133302022	0.113969953	PASS
NDRG2	14	21016763	21070872	0.060212819	PASS
NDRG3	20	36651766	36746078	-0.168668583	PASS
NDRG4	16	58462846	58513628	-0.371357732	PASS
NDST1	5	150485818	150558211	-0.293443042	PASS
NDST2	10	73801911	73811798	0.072405807	PASS
NDST3	4	118033618	118258648	-0.011382299	PASS
NDST4	4	114827763	115113876	-0.011382299	PASS
NDUFA1	23	119871487	119876662	-0.012567603	PASS
NDUFA10	2	239892450	240025402	0.025928102	PASS

NDUFA11	19	5891276	5904006	0.079632875	PASS
NDUFA12	12	94897055	95003770	0.202141228	PASS
NDUFA13	19	19515736	19529054	0.093487731	suppress_segment_too_large
NDUFA2	5	140638740	140647785	-0.293443042	PASS
NDUFA3	19	54102728	54109257	0.206912349	PASS
NDUFA4	7	10931951	10940256	-0.69513118	PASS
NDUFA4L	12	57234903	57240715	-0.202191512	PASS
NDUFA5	7	123536997	123558255	-0.388946609	PASS
NDUFA6	22	42085525	42090955	-0.575930959	PASS
NDUFA7	19	8308768	8321396	0.079632875	PASS
NDUFA8	9	122144058	122159819	0.365190713	suppress_segment_too_large
NDUFA9	12	4649095	4694317	-0.086128028	PASS
NDUFAB1	16	23581002	23596356	0.069936844	PASS
NDUFAF1	15	41387349	41402519	-0.396255771	PASS
NDUFAF2	5	60945129	61153037	-0.293443042	PASS
NDUFAF3	3	49020459	49023495	-0.234617007	PASS
NDUFAF4	6	96889313	96897881	-0.368590549	PASS
NDUFAF5	20	13784950	13821582	-0.168668583	PASS
NDUFAF6	8	94895767	95116455	0.279445683	PASS
NDUFAF7	2	37231631	37253403	0.040498426	PASS
NDUFAF8	17	81239239	81241281	0.088516311	PASS
NDUFB1	14	92116122	92121917	-0.192197564	PASS
NDUFB10	16	1959508	1961975	0.069936844	PASS
NDUFB11	23	47142216	47145504	-0.012567603	PASS
NDUFB2	7	140690777	140722790	-0.388946609	PASS
NDUFB3	2	201071433	201085750	0.025928102	PASS
NDUFB4	3	120596309	120602500	0.313454204	suppress_segment_too_large
NDUFB5	3	179604690	179627647	0.318278996	suppress_segment_too_large
NDUFB6	9	32552999	32573184	-0.310444659	PASS
NDUFB7	19	14566078	14572062	0.093487731	suppress_segment_too_large
NDUFB8	10	100523740	100530000	-0.367947924	PASS
NDUFB9	8	124539101	124580648	0.279445683	PASS
NDUFC1	4	139266880	139302551	-0.011382299	PASS
NDUFC2	11	78068304	78080219	0.062969153	PASS
NDUFC2-I	11	78016971	78079865	0.062969153	PASS
NDUFS1	2	206114817	206159603	0.025928102	PASS
NDUFS2	1	161197104	161214395	-0.027073548	PASS
NDUFS3	11	47565336	47584562	0.117376172	PASS
NDUFS4	5	53560633	53683340	-0.293443042	PASS
NDUFS5	1	39026318	39034636	0.033763446	PASS
NDUFS6	5	1801400	1816605	0.055007813	PASS
NDUFS7	19	1383527	1395589	0.079632875	PASS
NDUFS8	11	68030617	68036644	0.182439925	suppress_segment_too_large
NDUFV1	11	67605653	67612554	0.182439925	suppress_segment_too_large
NDUFV2	18	9102630	9134345	-0.052305056	suppress_segment_too_large
NDUFV3	21	42879644	42913304	-0.064425456	suppress_segment_too_large

NEB	2	151485336	151734487	-0.475215623	PASS
NEBL	10	20779973	21174187	0.039811807	PASS
NECAB1	8	90791550	90959408	0.279445683	PASS
NECAB2	16	83968632	84002776	-0.371357732	PASS
NECAB3	20	33657087	33674463	-0.168668583	PASS
NECAP1	12	8076939	8097859	-0.086128028	PASS
NECAP2	1	16440672	16460078	0.033763446	PASS
NECTIN1	11	119623408	119729084	-0.356188624	PASS
NECTIN2	19	44846175	44889228	0.14996833	PASS
NECTIN3	3	111070071	111275563	0.313454204	suppress_segment_too_large
NECTIN4	1	161070995	161089599	-0.027073548	PASS
NEDD1	12	96907223	96953777	0.202141228	PASS
NEDD4	15	55826922	55993746	-0.697600509	PASS
NEDD4L	18	58044367	58401540	-0.376397843	PASS
NEDD8	14	24216852	24232454	0.060212819	PASS
NEDD8-M	14	24213955	24232352	0.060212819	PASS
NEDD9	6	11183298	11382348	0.048820648	PASS
NEFH	22	29480230	29491390	-0.575930959	PASS
NEFL	8	24950955	24957110	-0.345962679	PASS
NEFM	8	24913012	24919098	-0.345962679	PASS
NEGR1	1	71395940	72282734	0.033763446	PASS
NEIL1	15	75346955	75357114	-0.697600509	PASS
NEIL2	8	11769639	11787346	-0.345962679	PASS
NEIL3	4	177309836	177362943	-0.011382299	PASS
NEK1	4	169392857	169612629	-0.011382299	PASS
NEK10	3	27110085	27369460	0.311018244	suppress_segment_too_large
NEK11	3	131026850	131350465	0.313454204	suppress_segment_too_large
NEK2	1	211658657	211675630	-0.256698369	PASS
NEK3	13	52132639	52159861	-0.390786075	PASS
NEK4	3	52708449	52770949	-0.234617007	PASS
NEK5	13	52033611	52129078	-0.390786075	PASS
NEK6	9	124257606	124353307	0.365190713	suppress_segment_too_large
NEK7	1	198156963	198322420	-0.027073548	PASS
NEK8	17	28725897	28743455	0.060555011	PASS
NEK9	14	75079353	75127344	-0.192197564	PASS
NELFA	4	1982714	2041903	0.279089364	PASS
NELFB	9	137255173	137273546	0.365190713	suppress_segment_too_large
NELFCD	20	58981208	58995133	-0.168668583	PASS
NELFE	6	31952087	31959110	0.048820648	PASS
NELL1	11	20669551	21575681	-0.405444717	PASS
NELL2	12	44508275	44921848	-0.202191512	PASS
NEMF	14	49782083	49853203	0.060212819	PASS
NEMP1	12	57055643	57088063	-0.202191512	PASS
NEMP2	2	190504342	190534722	0.025928102	PASS
NENF	1	212432887	212446379	-0.256698369	PASS
NEO1	15	73051710	73305206	-0.697600509	PASS

NEPRO	3	113002440	113019861	0.313454204	suppress_segment_too_large
NES	1	156668763	156677397	0.103015788	PASS
NET1	10	5412551	5458463	0.039811807	PASS
NETO1	18	72742314	72868146	-0.376397843	PASS
NETO2	16	47077703	47143997	0.069936844	PASS
NEU1	6	31857659	31862906	0.048820648	PASS
NEU2	2	233032672	233035057	0.025928102	PASS
NEU3	11	74988134	75018893	0.062969153	PASS
NEURL1	10	103493979	103592552	-0.367947924	PASS
NEURL1B	5	172641266	172691540	-0.150979337	PASS
NEURL2	20	45888625	45891287	-0.168668583	PASS
NEURL3	2	96497643	96508109	0.048238121	PASS
NEURL4	17	7315628	7329393	0.128406487	PASS
NEUROD1	2	181673088	181680876	0.039726562	PASS
NEUROD2	17	39603536	39609777	0.060555011	PASS
NEUROD4	12	55019945	55030014	-0.202191512	PASS
NEUROD6	7	31337461	31340894	-0.69513118	PASS
NEUROG1	5	135534282	135535949	-0.293443042	PASS
NEUROG2	4	112513516	112516172	-0.011382299	PASS
NEUROG3	10	69571698	69573238	0.072405807	PASS
NEXMIF	23	74732856	74925485	-0.012567603	PASS
NEXN	1	77888513	77943895	0.033763446	PASS
NF1	17	31094927	31382116	0.060555011	PASS
NF2	22	29603556	29698598	-0.575930959	PASS
NFAM1	22	42380410	42432395	-0.575930959	PASS
NFASC	1	204828651	205022822	-0.256698369	PASS
NFAT5	16	69565094	69704666	-0.371357732	PASS
NFATC1	18	79395856	79529325	-0.376397843	PASS
NFATC2	20	51386957	51562831	-0.168668583	PASS
NFATC2IP	16	28950807	28967097	0.069936844	PASS
NFATC3	16	68084751	68229259	-0.371357732	PASS
NFATC4	14	24365673	24379604	0.060212819	PASS
NFE2	12	54292111	54301121	-0.202191512	PASS
NFE2L1	17	48048329	48061487	0.088516311	PASS
NFE2L2	2	177227595	177392697	0.039726562	PASS
NFE2L3	7	26152240	26187125	-0.69513118	PASS
NFE4	7	102973437	102988856	-0.388946609	PASS
NFIA	1	60865259	61462793	0.033763446	PASS
NFIB	9	14081843	14398983	-0.310444659	PASS
NFIC	19	3314403	3469217	0.079632875	PASS
NFIL3	9	91409045	91423862	0.365190713	suppress_segment_too_large
NFIX	19	12995608	13098796	0.093487731	suppress_segment_too_large
NFKB1	4	102501329	102617302	-0.011382299	PASS
NFKB2	10	102394110	102402529	-0.367947924	PASS
NFKBIA	14	35401511	35404749	0.060212819	PASS
NFKBIB	19	38899700	38908893	0.093487731	suppress_segment_too_large

NFKBID	19	35887653	35902303	0.093487731	suppress_segment_too_large
NFKBIE	6	44258166	44265788	0.048820648	PASS
NFKBIL1	6	31546870	31558829	0.048820648	PASS
NFKBIZ	3	101827991	101861022	0.313454204	suppress_segment_too_large
NFRKB	11	129863636	129895590	-0.419612865	PASS
NFS1	20	35668055	35699359	-0.168668583	PASS
NFU1	2	69395750	69437628	-0.372959126	PASS
NFX1	9	33290511	33371157	-0.310444659	PASS
NFXL1	4	47847233	47914667	-0.011382299	PASS
NFYA	6	41072945	41099976	0.048820648	PASS
NFYB	12	104117077	104138289	0.202141228	PASS
NFYC	1	40691648	40771603	0.033763446	PASS
NGB	14	77265483	77271312	-0.192197564	PASS
NGDN	14	23469688	23509862	0.060212819	PASS
NGEF	2	232878686	233013272	0.025928102	PASS
NGF	1	115285918	115338236	0.033763446	PASS
NGFR	17	49495293	49515017	0.088516311	PASS
NGLY1	3	25718944	25790039	0.311018244	suppress_segment_too_large
NGRN	15	90265659	90278141	-0.697600509	PASS
NHEJ1	2	219075317	219160865	0.025928102	PASS
NHLH1	1	160367067	160372848	-0.027073548	PASS
NHLH2	1	115836377	115843917	0.033763446	PASS
NHLRC1	6	18120440	18122687	0.048820648	PASS
NHLRC2	10	113854661	113917194	0.063874692	PASS
NHLRC3	13	39038306	39050109	-0.390786075	PASS
NHLRC4	16	566996	569495	0.069936844	PASS
NHP2	5	178149460	178153967	-0.150979337	PASS
NHS	23	17375420	17735994	-0.012567603	PASS
NHSL1	6	138422043	138692571	-0.035448111	PASS
NHSL2	23	71910818	72161750	-0.012567603	PASS
NICN1	3	49422946	49429326	-0.234617007	PASS
NID1	1	235975830	236065162	-0.256698369	PASS
NID2	14	52004803	52069228	0.060212819	PASS
NIF3L1	2	200889327	200903930	0.025928102	PASS
NIFK	2	121726945	121736923	-0.475215623	PASS
NIM1K	5	43192071	43280850	-0.293443042	PASS
NIN	14	50719763	50831121	0.060212819	PASS
NINJ1	9	93121489	93134288	0.365190713	suppress_segment_too_large
NINJ2	12	564296	663779	-0.086128028	PASS
NINL	20	25452705	25585517	-0.168668583	PASS
NIP7	16	69337996	69343111	-0.371357732	PASS
NIPA1	15	22773063	22829791	-0.396255771	PASS
NIPA2	15	22838641	22868384	-0.396255771	PASS
NIPAL1	4	47914142	48040173	-0.011382299	PASS
NIPAL2	8	98189833	98294393	0.279445683	PASS
NIPAL3	1	24415794	24472976	0.033763446	PASS

NIPAL4	5	157460019	157474717	-0.150979337	PASS
NIPBL	5	36876759	37066413	0.055007813	PASS
NIPSNAP1	22	29554808	29581337	-0.575930959	PASS
NIPSNAP2	7	55951793	56000181	-0.750061359	PASS
NIPSNAP3	9	104747688	104760122	0.365190713	suppress_segment_too_large
NIPSNAP3	9	104764157	104777457	0.365190713	suppress_segment_too_large
NISCH	3	52455118	52493071	-0.234617007	PASS
NIT1	1	161118086	161125445	-0.027073548	PASS
NIT2	3	100334701	100361635	0.313454204	suppress_segment_too_large
NKAIN1	1	31179745	31239554	0.033763446	PASS
NKAIN2	6	123804141	124825657	-1.97829255	PASS
NKAIN3	8	62248591	62999652	-0.201142258	PASS
NKAIN4	20	63240784	63272694	-0.168668583	PASS
NKAP	23	119920672	119943772	-0.012567603	PASS
NKAPD1	11	112074086	112085150	-0.356188624	PASS
NKAPL	6	28259320	28260958	0.048820648	PASS
NKD1	16	50548330	50649249	0.069936844	PASS
NKD2	5	1008829	1038943	0.055007813	PASS
NKG7	19	51371606	51372715	-0.016761391	PASS
NKIRAS1	3	23891660	23946591	0.311018244	suppress_segment_too_large
NKIRAS2	17	42011382	42025644	0.060555011	PASS
NKPD1	19	45149750	45158737	0.14996833	PASS
NKRF	23	119588337	119606443	-0.012567603	PASS
NKTR	3	42600614	42648741	-0.234617007	PASS
NKX1-1	4	1402932	1406331	0.279089364	PASS
NKX1-2	10	124445239	124450184	0.063874692	PASS
NKX2-1	14	36516392	36521149	0.060212819	PASS
NKX2-2	20	21511010	21514026	-0.168668583	PASS
NKX2-3	10	99532933	99536524	-0.367947924	PASS
NKX2-4	20	21395367	21398028	-0.168668583	PASS
NKX2-5	5	173232109	173235357	-0.150979337	PASS
NKX2-6	8	23702451	23706598	-0.345962679	PASS
NKX2-8	14	36580579	36582607	0.060212819	PASS
NKX3-1	8	23678693	23682927	-0.345962679	PASS
NKX3-2	4	13540830	13545050	0.279089364	PASS
NKX6-1	4	84491987	84498450	-0.011382299	PASS
NKX6-2	10	132783179	132786052	0.063874692	PASS
NKX6-3	8	41645178	41650669	-0.201142258	PASS
NLE1	17	35128753	35142315	0.060555011	PASS
NLGN1	3	173396284	174286644	0.318278996	suppress_segment_too_large
NLGN2	17	7404874	7419860	0.128406487	PASS
NLGN3	23	71144831	71171201	-0.012567603	PASS
NLGN4X	23	5840637	6228863	-0.012567603	PASS
NLK	17	28041737	28196381	0.060555011	PASS
NLN	5	65722196	65871725	-0.293443042	PASS
NLRC3	16	3539033	3577400	0.069936844	PASS

NLRC4	2	32224453	32265854	0.040498426	PASS
NLRC5	16	56989485	57083531	-0.371357732	PASS
NLRP1	17	5499427	5619424	0.128406487	PASS
NLRP10	11	7959424	7965426	-0.405444717	PASS
NLRP11	19	55785397	55836800	-0.180829161	PASS
NLRP12	19	53793603	53824394	0.206912349	PASS
NLRP13	19	55891699	55932336	-0.180829161	PASS
NLRP14	11	7020446	7071308	-0.405444717	PASS
NLRP2	19	54953130	55001142	-0.146906334	PASS
NLRP2B	23	57677067	57680260	-0.012567603	PASS
NLRP3	1	247416156	247449108	-0.368111568	PASS
NLRP4	19	55836578	55881854	-0.180829161	PASS
NLRP5	19	55999726	56061813	-0.180829161	PASS
NLRP6	11	278365	285359	-0.333835828	PASS
NLRP7	19	54923509	54966312	-0.146906334	PASS
NLRP8	19	55947832	55988629	-0.180829161	PASS
NLRP9	19	55708432	55738402	0.121336758	PASS
NLRX1	11	119166568	119184016	-0.356188624	PASS
NMB	15	84655129	84658563	-0.697600509	PASS
NMBR	6	142058330	142088799	-0.035448111	PASS
NMD3	3	161104696	161253532	0.318278996	suppress_segment_too_large
NME1	17	51153536	51162428	0.088516311	PASS
NME1-NM	17	51153590	51171744	0.088516311	PASS
NME2	17	51165435	51171747	0.088516311	PASS
NME3	16	1770286	1771730	0.069936844	PASS
NME4	16	396725	410367	0.069936844	PASS
NME5	5	138115172	138139443	-0.293443042	PASS
NME6	3	48290722	48301685	-0.234617007	PASS
NME7	1	169132531	169367967	-0.027073548	PASS
NME8	7	37848597	37900401	-0.69513118	PASS
NME9	3	138261437	138329886	0.313454204	suppress_segment_too_large
NMI	2	151270465	151290057	-0.475215623	PASS
NMNAT1	1	9943428	9985501	0.373964873	suppress_segment_too_large
NMNAT2	1	183248237	183418602	-0.027073548	PASS
NMNAT3	3	139560180	139678017	-0.356957581	PASS
NMRAL1	16	4461680	4495763	0.069936844	PASS
NMRK1	9	75060573	75088217	0.365190713	suppress_segment_too_large
NMRK2	19	3933103	3942416	0.079632875	PASS
NMS	2	100470482	100483280	0.048238121	PASS
NMT1	17	45051610	45109016	0.088516311	PASS
NMT2	10	15102584	15168693	0.039811807	PASS
NMU	4	55595229	55636698	-0.011382299	PASS
NMUR1	2	231523160	231530495	0.025928102	PASS
NMUR2	5	152391532	152433368	-0.293443042	PASS
NNAT	20	37521206	37523693	-0.168668583	PASS
NNMT	11	114257787	114313285	-0.356188624	PASS

NNT	5	43602692	43707405	-0.293443042	PASS
NOA1	4	56963370	56978823	-0.011382299	PASS
NOB1	16	69741867	69754940	-0.371357732	PASS
NOBOX	7	144397240	144410227	-0.388946609	PASS
NOC2L	1	944204	959309	0.373964873	suppress_segment_too_large
NOC3L	10	94333226	94362959	-0.39678585	PASS
NOC4L	12	132144448	132152473	0.270848837	PASS
NOCT	4	139015789	139045939	-0.011382299	PASS
NOD1	7	30424527	30478784	-0.69513118	PASS
NOD2	16	50693588	50734041	0.069936844	PASS
NODAL	10	70432315	70447951	0.072405807	PASS
NOG	17	56593699	56595590	0.088516311	PASS
NOL10	2	10570766	10689987	0.040498426	PASS
NOL11	17	67717833	67744531	0.088516311	PASS
NOL12	22	37681673	37693478	-0.575930959	PASS
NOL3	16	67170154	67175735	-0.371357732	PASS
NOL4	18	33851100	34224952	-0.376397843	PASS
NOL4L	20	32443059	32585074	-0.168668583	PASS
NOL6	9	33461441	33473930	-0.310444659	PASS
NOL7	6	13615327	13632739	0.048820648	PASS
NOL8	9	92297358	92325636	0.365190713	suppress_segment_too_large
NOL9	1	6521347	6554535	0.373964873	suppress_segment_too_large
NOLC1	10	102152176	102163871	-0.367947924	PASS
NOM1	7	156949723	156973182	-0.411415677	PASS
NOMO1	16	14833681	14896160	0.069936844	PASS
NOMO2	16	18417325	18562211	0.069936844	PASS
NOMO3	16	16232495	16294814	0.069936844	PASS
NONO	23	71283192	71301168	-0.012567603	PASS
NOP10	15	34341713	34343177	-0.396255771	PASS
NOP14	4	2937933	2963385	0.279089364	PASS
NOP16	5	176383938	176388975	-0.150979337	PASS
NOP2	12	6556863	6568691	-0.086128028	PASS
NOP53	19	47745522	47757058	0.14996833	PASS
NOP56	20	2652145	2658393	-0.168668583	PASS
NOP58	2	202265716	202303666	0.025928102	PASS
NOP9	14	24299862	24309124	0.060212819	PASS
NOS1	12	117208142	117452170	0.202141228	PASS
NOS1AP	1	162069774	162370475	-0.027073548	PASS
NOS2	17	27756766	27800499	0.060555011	PASS
NOS3	7	150990995	151014588	-0.388946609	PASS
NOSIP	19	49555711	49590262	0.14996833	PASS
NOSTRIN	2	168786539	168865514	0.039726562	PASS
NOTCH1	9	136494433	136545786	0.365190713	suppress_segment_too_large
NOTCH2	1	119911553	120069626	-0.157385092	PASS
NOTCH2N	1	146146203	146229026	-0.157385092	PASS
NOTCH3	19	15159038	15200981	0.093487731	suppress_segment_too_large

NOTCH4	6	32194843	32224067	0.048820648	PASS
NOTO	2	73202258	73212513	-0.372959126	PASS
NOTUM	17	81952507	81961840	0.088516311	PASS
NOV	8	119416306	119424353	0.279445683	PASS
NOVA1	14	26443093	26597754	0.060212819	PASS
NOVA2	19	45933734	45973546	0.14996833	PASS
NOX1	23	100843324	100874345	-0.012567603	PASS
NOX3	6	155395370	155455903	-0.035448111	PASS
NOX4	11	89324356	89498187	0.062969153	PASS
NOX5	15	68930525	69062743	-0.697600509	PASS
NOXA1	9	137423350	137434406	0.365190713	suppress_segment_too_large
NOXO1	16	1978917	1984192	0.069936844	PASS
NOXRED1	14	77394021	77423517	-0.192197564	PASS
NPAP1	15	24675868	24683393	-0.396255771	PASS
NPAS1	19	47019820	47045775	0.14996833	PASS
NPAS2	2	100820152	100996829	0.048238121	PASS
NPAS3	14	32934933	33804176	0.060212819	PASS
NPAS4	11	66421004	66426707	0.182439925	suppress_segment_too_large
NPAT	11	108157215	108222642	-0.356188624	PASS
NPB	17	81900745	81902905	0.088516311	PASS
NPBWR1	8	52938431	52941117	-0.201142258	PASS
NPBWR2	20	64105820	64107171	-0.168668583	PASS
NPC1	18	23506184	23586898	-0.376397843	PASS
NPC1L1	7	44512535	44541315	-0.69513118	PASS
NPC2	14	74476192	74494177	-0.192197564	PASS
NPDC1	9	137039470	137046203	0.365190713	suppress_segment_too_large
NPEPL1	20	58689131	58719238	-0.168668583	PASS
NPEPPS	17	47522942	47623276	0.088516311	PASS
NPFF	12	53506690	53507638	-0.202191512	PASS
NPFFR1	10	70247329	70283676	0.072405807	PASS
NPFFR2	4	72031804	72148067	-0.011382299	PASS
NPHP1	2	110122311	110205066	-0.010901494	PASS
NPHP3	3	132680609	132722442	0.313454204	suppress_segment_too_large
NPHP3-AC	3	132558142	132722459	0.313454204	suppress_segment_too_large
NPHP4	1	5862811	5992473	0.373964873	suppress_segment_too_large
NPHS1	19	35825964	35869287	0.093487731	suppress_segment_too_large
NPHS2	1	179550539	179575952	-0.027073548	PASS
NPIPA1	16	14922802	14952060	0.069936844	PASS
NPIPA2	16	14748066	14765413	0.069936844	PASS
NPIPA3	16	14708944	14726338	0.069936844	PASS
NPIPA5	16	15363624	15381047	0.069936844	PASS
NPIPA7	16	16379055	16393954	0.069936844	PASS
NPIPA8	16	18317942	18336736	0.069936844	PASS
NPIPB11	16	29381354	29404029	0.069936844	PASS
NPIPB12	16	29483642	29505999	0.069936844	PASS
NPIPB15	16	74377878	74392080	-0.371357732	PASS

NPIPB2	16	11927373	11976643	0.069936844	PASS
NPIPB3	16	21402237	21448567	0.069936844	PASS
NPIPB4	16	21834569	21880827	0.069936844	PASS
NPIPB5	16	22479121	22536521	0.069936844	PASS
NPIPB6	16	28342555	28363508	0.069936844	PASS
NPIPB7	16	28456372	28471175	0.069936844	PASS
NPIPB8	16	28637654	28658682	0.069936844	PASS
NPIPB9	16	28751787	28772807	0.069936844	PASS
NPL	1	182789293	182830384	-0.027073548	PASS
NPLOC4	17	81556887	81648465	0.088516311	PASS
NPM1	5	171387116	171411137	-0.150979337	PASS
NPM2	8	22024125	22036897	-0.345962679	PASS
NPM3	10	101781325	101783413	-0.367947924	PASS
NPNT	4	105894775	106004027	-0.011382299	PASS
NPPA	1	11845709	11848345	0.373964873	suppress_segment_too_large
NPPB	1	11857464	11858931	0.373964873	suppress_segment_too_large
NPPC	2	231921820	231926403	0.025928102	PASS
NPR1	1	153678637	153693992	0.103015788	PASS
NPR2	9	35792154	35809732	-0.310444659	PASS
NPR3	5	32689070	32791724	0.055007813	PASS
NPRL2	3	50347330	50351091	-0.234617007	PASS
NPRL3	16	84271	138860	0.069936844	PASS
NPS	10	127549369	127552639	0.063874692	PASS
NPSR1	7	34658239	34878332	-0.69513118	PASS
NPTN	15	73560014	73634134	-0.697600509	PASS
NPTX1	17	80467148	80477843	0.088516311	PASS
NPTX2	7	98617297	98629868	-0.262778904	PASS
NPTXR	22	38818452	38843982	-0.575930959	PASS
NPVF	7	25224570	25228486	-0.69513118	PASS
NPW	16	2009926	2020755	0.069936844	PASS
NPY	7	24284163	24291865	-0.69513118	PASS
NPY1R	4	163323961	163344832	-0.011382299	PASS
NPY2R	4	155208629	155217078	-0.011382299	PASS
NPY4R	10	46461099	46465881	-0.404852538	PASS
NPY4R2	10	47918739	47923524	-0.404852538	PASS
NPY5R	4	163343939	163351934	-0.011382299	PASS
NQO1	16	69706996	69726951	-0.371357732	PASS
NQO2	6	2987987	3028869	0.048820648	PASS
NR0B1	23	30304206	30309598	-0.012567603	PASS
NR0B2	1	26911489	26913966	0.033763446	PASS
NR1D1	17	40092787	40100725	0.060555011	PASS
NR1D2	3	23945260	23980618	0.311018244	suppress_segment_too_large
NR1H2	19	50329653	50382982	-0.016761391	PASS
NR1H3	11	47248300	47269032	0.117376172	PASS
NR1H4	12	100473708	100564413	0.202141228	PASS
NR1I2	3	119780484	119818485	0.313454204	suppress_segment_too_large

NR1I3	1	161229666	161238302	-0.027073548	PASS
NR2C1	12	95020229	95073703	0.202141228	PASS
NR2C2	3	14947584	15053600	0.311018244	suppress_segment_too_large
NR2C2AP	19	19201416	19203424	0.093487731	suppress_segment_too_large
NR2E1	6	108166058	108188809	-0.368590549	PASS
NR2E3	15	71792638	71818259	-0.697600509	PASS
NR2F1	5	93583224	93594615	-0.293443042	PASS
NR2F2	15	96325938	96340263	-0.697600509	PASS
NR2F6	19	17231883	17245940	0.093487731	suppress_segment_too_large
NR3C1	5	143277931	143435512	-0.293443042	PASS
NR3C2	4	148078762	148444698	-0.011382299	PASS
NR4A1	12	52022832	52059507	-0.202191512	PASS
NR4A2	2	156324432	156342348	-0.475215623	PASS
NR4A3	9	99821855	99866891	0.365190713	suppress_segment_too_large
NR5A1	9	124481236	124507430	0.365190713	suppress_segment_too_large
NR5A2	1	200027602	200177424	-0.027073548	PASS
NR6A1	9	124517275	124771310	0.365190713	suppress_segment_too_large
NRAP	10	113588716	113664127	0.063874692	PASS
NRARP	9	137300482	137302251	0.365190713	suppress_segment_too_large
NRAS	1	114704469	114716894	0.033763446	PASS
NRBF2	10	63133247	63155031	0.072405807	PASS
NRBP1	2	27427790	27442259	0.040498426	PASS
NRBP2	8	143833594	143840974	0.307788765	PASS
NRCAM	7	108147623	108456717	-0.388946609	PASS
NRDC	1	51789191	51878937	0.033763446	PASS
NRDE2	14	90267856	90332137	-0.192197564	PASS
NREP	5	111662621	111997464	-0.293443042	PASS
NRF1	7	129611714	129757082	-0.388946609	PASS
NRG1	8	31639386	32767959	-0.345962679	PASS
NRG2	5	139846779	140043299	-0.293443042	PASS
NRG3	10	81875314	82987179	0.072405807	PASS
NRG4	15	75935969	76059795	-0.697600509	PASS
NRGN	11	124739846	124747210	-0.419612865	PASS
NRIP1	21	14961235	15065936	-0.304975024	PASS
NRIP2	12	2825348	2835544	-0.086128028	PASS
NRIP3	11	8980576	9004049	-0.405444717	PASS
NRK	23	105822543	105958610	-0.012567603	PASS
NRL	14	24080107	24115014	0.060212819	PASS
NRM	6	30688047	30691420	0.048820648	PASS
NRN1	6	5997999	6007605	0.048820648	PASS
NRN1L	16	67884805	67888855	-0.371357732	PASS
NRP1	10	33177492	33336262	0.039811807	PASS
NRP2	2	205681990	205798133	0.025928102	PASS
NRROS	3	196639686	196662004	0.326834516	PASS
NRSN1	6	24126122	24154900	0.048820648	PASS
NRSN2	20	346782	359660	-0.168668583	PASS

NRTN	19	5823802	5828324	0.079632875	PASS
NRXN1	2	49918505	51225575	0.040498426	PASS
NRXN2	11	64606174	64723188	0.182439925	suppress_segment_too_large
NRXN3	14	78170373	79868290	-0.192197564	PASS
NSA2	5	74766991	74780113	-0.293443042	PASS
NSD1	5	177133025	177300215	-0.150979337	PASS
NSD2	4	1871424	1982207	0.279089364	PASS
NSD3	8	38269697	38382272	-0.201142258	PASS
NSDHL	23	152830967	152869729	-0.012567603	PASS
NSF	17	46590669	46757464	0.088516311	PASS
NSFL1C	20	1442162	1473842	-0.168668583	PASS
NSG1	4	4348140	4419058	0.279089364	PASS
NSG2	5	174045604	174243501	-0.150979337	PASS
NSL1	1	212726153	212791782	-0.256698369	PASS
NSMAF	8	58583504	58659844	-0.201142258	PASS
NSMCE1	16	27224991	27268794	0.069936844	PASS
NSMCE2	8	125091679	125367120	0.279445683	PASS
NSMCE3	15	29264992	29269829	-0.396255771	PASS
NSMCE4A	10	121957088	121975217	0.063874692	PASS
NSMF	9	137447573	137459334	0.365190713	suppress_segment_too_large
NSRP1	17	30115521	30186475	0.060555011	PASS
NSUN2	5	6599239	6633291	0.055007813	PASS
NSUN3	3	94062916	94128545	0.313454204	suppress_segment_too_large
NSUN4	1	46340177	46365152	0.033763446	PASS
NSUN5	7	73302517	73308867	-0.262778904	PASS
NSUN6	10	18545561	18659285	0.039811807	PASS
NSUN7	4	40749897	40809985	-0.011382299	PASS
NT5C	17	75130225	75131795	0.088516311	PASS
NT5C1A	1	39659121	39672038	0.033763446	PASS
NT5C1B	2	18562872	18589572	0.040498426	PASS
NT5C1B-R	2	18555545	18589564	0.040498426	PASS
NT5C2	10	103088017	103193306	-0.367947924	PASS
NT5C3A	7	33014113	33062797	-0.69513118	PASS
NT5C3B	17	41825181	41836263	0.060555011	PASS
NT5DC1	6	116100849	116249497	-0.368590549	PASS
NT5DC2	3	52524385	52535054	-0.234617007	PASS
NT5DC3	12	103770453	103841197	0.202141228	PASS
NT5DC4	2	112721486	112742879	-0.010901494	PASS
NT5E	6	85449584	85495791	-0.368590549	PASS
NT5M	17	17303335	17347663	0.024610076	PASS
NTAN1	16	15037853	15056079	0.069936844	PASS
NTF3	12	5432112	5521536	-0.086128028	PASS
NTF4	19	49061066	49065054	0.14996833	PASS
NTHL1	16	2039815	2047866	0.069936844	PASS
NTM	11	131370478	132336822	-0.419612865	PASS
NTMT1	9	129608884	129636131	0.365190713	suppress_segment_too_large

NTN1	17	9021542	9244000	-0.139031004	PASS
NTN3	16	2471499	2474145	0.069936844	PASS
NTN4	12	95657807	95791152	0.202141228	PASS
NTN5	19	48661407	48673081	0.14996833	PASS
NTNG1	1	107140007	107483458	0.033763446	PASS
NTNG2	9	132161676	132244534	0.365190713	suppress_segment_too_large
NTPCR	1	232950605	232983882	-0.256698369	PASS
NTRK1	1	156815640	156881850	0.103015788	PASS
NTRK2	9	84668551	85027070	0.365190713	suppress_segment_too_large
NTRK3	15	87859751	88256768	-0.697600509	PASS
NTS	12	85874295	85882992	0.202141228	PASS
NTSR1	20	62708837	62762771	-0.168668583	PASS
NTSR2	2	11658178	11670164	0.040498426	PASS
NUAK1	12	106063340	106140033	0.202141228	PASS
NUAK2	1	205302059	205321791	-0.256698369	PASS
NUB1	7	151341699	151378449	-0.388946609	PASS
NUBP1	16	10743786	10769351	0.069936844	PASS
NUBP2	16	1782901	1789191	0.069936844	PASS
NUBPL	14	31489956	31861224	0.060212819	PASS
NUCB1	19	48900050	48923372	0.14996833	PASS
NUCB2	11	17208153	17349980	-0.405444717	PASS
NUCKS1	1	205712819	205750276	-0.256698369	PASS
NUDC	1	26900238	26946862	0.033763446	PASS
NUDCD1	8	109240919	109334385	0.279445683	PASS
NUDCD2	5	163446526	163460140	-0.150979337	PASS
NUDCD3	7	44379121	44490880	-0.69513118	PASS
NUDT1	7	2242222	2251146	-0.69513118	PASS
NUDT10	23	51332231	51337525	-0.012567603	PASS
NUDT11	23	51490011	51496596	-0.012567603	PASS
NUDT12	5	103548855	103562793	-0.293443042	PASS
NUDT13	10	73110375	73131828	0.072405807	PASS
NUDT14	14	105172938	105181323	-0.192197564	PASS
NUDT15	13	48037567	48047222	-0.390786075	PASS
NUDT16	3	131381671	131388830	0.313454204	suppress_segment_too_large
NUDT16L	16	4693694	4695859	0.069936844	PASS
NUDT17	1	145845629	145848953	-0.157385092	PASS
NUDT18	8	22106872	22109419	-0.345962679	PASS
NUDT19	19	32691961	32713796	0.093487731	suppress_segment_too_large
NUDT2	9	34329506	34343713	-0.310444659	PASS
NUDT21	16	56429133	56452199	-0.371357732	PASS
NUDT22	11	64225941	64230686	0.182439925	suppress_segment_too_large
NUDT3	6	34279679	34392674	0.048820648	PASS
NUDT4	12	93377883	93408146	0.202141228	PASS
NUDT5	10	12165325	12196144	0.039811807	PASS
NUDT6	4	122888697	122922968	-0.011382299	PASS
NUDT7	16	77722492	77742260	-0.371357732	PASS

NUDT8	11	67627938	67629930	0.182439925	suppress_segment_too_large
NUDT9	4	87422582	87459454	-0.011382299	PASS
NUF2	1	163266576	163355764	-0.027073548	PASS
NUFIP1	13	44939249	44989483	-0.390786075	PASS
NUFIP2	17	29255836	29294118	0.060555011	PASS
NUGGC	8	28021964	28083871	-0.345962679	PASS
NUMA1	11	72002864	72080693	0.062969153	PASS
NUMB	14	73275107	73458617	0.060212819	PASS
NUMBL	19	40665905	40690972	0.093487731	suppress_segment_too_large
NUP107	12	68686734	68745809	-0.202191512	PASS
NUP133	1	229440260	229508341	-0.256698369	PASS
NUP153	6	17615035	17706834	0.048820648	PASS
NUP155	5	37288137	37371181	0.055007813	PASS
NUP160	11	47778087	47848555	0.117376172	PASS
NUP188	9	128947699	129007096	0.365190713	suppress_segment_too_large
NUP205	7	135557919	135648757	-0.388946609	PASS
NUP210	3	13316235	13420309	0.311018244	suppress_segment_too_large
NUP210L	1	153992685	154155116	0.103015788	PASS
NUP214	9	131125561	131234670	0.365190713	suppress_segment_too_large
NUP35	2	183117513	183161680	-0.169541924	PASS
NUP37	12	102073103	102120124	0.202141228	PASS
NUP43	6	149724315	149749665	-0.035448111	PASS
NUP50	22	45163841	45188015	-0.808211634	PASS
NUP54	4	76114659	76148515	-0.011382299	PASS
NUP58	13	25301524	25349800	-0.390786075	PASS
NUP62	19	49906825	49929763	-0.016761391	PASS
NUP62CL	23	107123427	107206433	-0.012567603	PASS
NUP85	17	75205659	75235758	0.088516311	PASS
NUP88	17	5360963	5420160	0.128406487	PASS
NUP93	16	56730105	56850286	-0.371357732	PASS
NUP98	11	3671083	3797792	-0.333835828	PASS
NUPL2	7	23181827	23201011	-0.69513118	PASS
NUPR1	16	28532708	28539174	0.069936844	PASS
NUPR2	7	56114681	56116400	-0.750061359	PASS
NUS1	6	117675502	117710640	-0.368590549	PASS
NUSAP1	15	41332694	41381050	-0.396255771	PASS
NUTF2	16	67846732	67872567	-0.371357732	PASS
NUTM1	15	34343315	34357737	-0.396255771	PASS
NUTM2A	10	87225448	87236908	-0.292453993	PASS
NUTM2B	10	79703227	79714681	0.072405807	PASS
NUTM2D	10	87357668	87370695	-0.292453993	PASS
NUTM2F	9	94318196	94328644	0.365190713	suppress_segment_too_large
NUTM2G	9	96928310	96940253	0.365190713	suppress_segment_too_large
NVL	1	224227334	224330387	-0.256698369	PASS
NWD1	19	16719976	16817963	0.093487731	suppress_segment_too_large
NWD2	4	37244220	37449465	-0.011382299	PASS

NXF1	11	62792123	62806302	0.182439925	suppress_segment_too_large
NXF2	23	102247161	102326719	-0.012567603	PASS
NXF3	23	103075810	103093125	-0.012567603	PASS
NXF5	23	101832112	101857577	-0.012567603	PASS
NXN	17	799313	979770	0.128406487	PASS
NXNL1	19	17455425	17460954	0.093487731	suppress_segment_too_large
NXNL2	9	88534033	88584274	0.365190713	suppress_segment_too_large
NXPE1	11	114521645	114559895	-0.356188624	PASS
NXPE2	11	114678386	114706933	-0.356188624	PASS
NXPE3	3	101779202	101827392	0.313454204	suppress_segment_too_large
NXPE4	11	114570591	114595762	-0.356188624	PASS
NXPH1	7	8433955	8752963	-0.69513118	PASS
NXPH2	2	138670772	138780348	-0.475215623	PASS
NXPH3	17	49575858	49583827	0.088516311	PASS
NXPH4	12	57216795	57226449	-0.202191512	PASS
NXT1	20	23350736	23354777	-0.168668583	PASS
NXT2	23	109535781	109544690	-0.012567603	PASS
NYAP1	7	100483927	100494799	-0.262778904	PASS
NYAP2	2	225399710	225654018	0.025928102	PASS
NYNRIN	14	24398786	24419288	0.060212819	PASS
NYX	23	41447434	41475710	-0.012567603	PASS
OAF	11	120210767	120230332	-0.356188624	PASS
OARD1	6	41033627	41097787	0.048820648	PASS
OAS1	12	112906777	112933222	0.202141228	PASS
OAS2	12	112978395	113011723	0.202141228	PASS
OAS3	12	112938352	112973249	0.202141228	PASS
OASL	12	121019111	121039242	0.202141228	PASS
OAT	10	124397303	124418976	0.063874692	PASS
OAZ1	19	2269509	2273490	0.079632875	PASS
OAZ2	15	64687573	64703281	-0.697600509	PASS
OAZ3	1	151762899	151771334	0.103015788	PASS
OBP2A	9	135546139	135549969	0.365190713	suppress_segment_too_large
OBP2B	9	133205277	133209250	0.365190713	suppress_segment_too_large
OBSCN	1	228208130	228378874	-0.256698369	PASS
OBSL1	2	219550729	219571859	0.025928102	PASS
OC90	8	132024220	132059380	0.113969953	PASS
OCA2	15	27754875	28099358	-0.396255771	PASS
OCEL1	19	17226204	17229219	0.093487731	suppress_segment_too_large
OCIAD1	4	48805212	48861817	-0.011382299	PASS
OCIAD2	4	48885019	48906937	-0.011382299	PASS
OCLN	5	69492292	69558104	-0.293443042	PASS
OCM	7	5879827	5886362	-0.69513118	PASS
OCM2	7	97984684	97991169	-0.262778904	PASS
OCRL	23	129539849	129592561	-0.012567603	PASS
OCSTAMP	20	46540946	46550574	-0.168668583	PASS
ODAM	4	70196496	70204576	-0.011382299	PASS

ODAPH	4	75556048	75565885		-0.011382299	PASS
ODC1	2	10439968	10448504		0.040498426	PASS
ODF1	8	102551572	102561017		0.279445683	PASS
ODF2	9	128455186	128501292		0.365190713	suppress_segment_too_large
ODF2L	1	86346824	86396342		0.033763446	PASS
ODF3	11	196738	200261		-0.333835828	PASS
ODF3B	22	50529710	50532580		-0.808211634	PASS
ODF3L1	15	75723977	75727688		-0.697600509	PASS
ODF3L2	19	463346	474983		0.079632875	PASS
ODF4	17	8339864	8346048		0.128406487	PASS
ODR4	1	186375838	186421378		-0.027073548	PASS
OFD1	23	13734745	13769353		-0.012567603	PASS
OGA	10	101784443	101818465		-0.367947924	PASS
OGDH	7	44606572	44709066		-0.69513118	PASS
OGDHL	10	49734643	49762379		-0.404852538	PASS
OGFOD1	16	56451490	56479100		-0.371357732	PASS
OGFOD2	12	122974580	122980043		0.202141228	PASS
OGFOD3	17	82389223	82418637		0.088516311	PASS
OGFR	20	62804835	62814000		-0.168668583	PASS
OGFRL1	6	71288803	71308950		-0.368590549	PASS
OGG1	3	9749944	9788219		0.311018244	suppress_segment_too_large
OGN	9	92383967	92404696		0.365190713	suppress_segment_too_large
OGT	23	71533083	71575897		-0.012567603	PASS
OIP5	15	41309268	41332621		-0.396255771	PASS
OIT3	10	72893581	72933033		0.072405807	PASS
OLA1	2	174072447	174248698		0.039726562	PASS
OLAH	10	15032227	15073852		0.039811807	PASS
OLFM1	9	135075422	135121179		0.365190713	suppress_segment_too_large
OLFM2	19	9853718	9936552		0.093487731	suppress_segment_too_large
OLFM3	1	101802574	101997030		0.033763446	PASS
OLFM4	13	53028759	53052057		-0.390786075	PASS
OLFML1	11	7485388	7511377		-0.405444717	PASS
OLFML2A	9	124777158	124814885		0.365190713	suppress_segment_too_large
OLFML2B	1	161983192	162023854		-0.027073548	PASS
OLFML3	1	113979391	114035572		0.033763446	PASS
OLIG1	21	33070144	33072420		0.12860881	suppress_segment_too_large
OLIG2	21	33025845	33029196		0.12860881	suppress_segment_too_large
OLIG3	6	137492202	137494250		-0.035448111	PASS
OLR1	12	10158301	10172138		0.362961408	suppress_segment_too_large
OMA1	1	58415384	58546802		0.033763446	PASS
OMD	9	92414245	92424461		0.365190713	suppress_segment_too_large
OMG	17	31272013	31297539		0.060555011	PASS
OMP	11	77102840	77103331		0.062969153	PASS
ONECUT1	15	52756989	52791078		-0.396255771	PASS
ONECUT2	18	57435685	57491297		-0.376397843	PASS
ONECUT3	19	1752373	1780988		0.079632875	PASS

OOEP	6	73368555	73395133	-0.368590549	PASS
OOSP2	11	60040275	60048044	-0.129266491	PASS
OOSP4B	11	59978020	60031077	-0.129266491	PASS
OPA1	3	193593144	193697823	0.326834516	PASS
OPA3	19	45527427	45602212	0.14996833	PASS
OPALIN	10	96343216	96359365	-0.367947924	PASS
OPCML	11	132414977	133532519	-0.419612865	PASS
OPHN1	23	68042344	68433913	-0.012567603	PASS
OPLAH	8	144051266	144063965	0.307788765	PASS
OPN1LW	23	154144224	154159032	-0.012567603	PASS
OPN1MW	23	154182596	154196135	-0.012567603	PASS
OPN1SW	7	128772491	128775790	-0.388946609	PASS
OPN3	1	241590102	241677376	-0.648598641	PASS
OPN4	10	86654557	86666848	-0.292453993	PASS
OPN5	6	47781982	47832780	0.048820648	PASS
OPRD1	1	28812142	28871267	0.033763446	PASS
OPRK1	8	53225716	53251697	-0.201142258	PASS
OPRL1	20	64080173	64100643	-0.168668583	PASS
OPRM1	6	154010496	154246867	-0.035448111	PASS
OPRPN	4	70397882	70410194	-0.011382299	PASS
OPTC	1	203494143	203508864	-0.256698369	PASS
OPTN	10	13099449	13138308	0.039811807	PASS
OR10A2	11	6863057	6874717	-0.405444717	PASS
OR10A3	11	7937171	7941708	-0.405444717	PASS
OR10A4	11	6876625	6877619	-0.405444717	PASS
OR10A5	11	6845652	6846705	-0.405444717	PASS
OR10A6	11	7924592	7931268	-0.405444717	PASS
OR10A7	12	55221025	55221975	-0.202191512	PASS
OR10AD1	12	48202083	48203387	-0.202191512	PASS
OR10AG1	11	55965755	55969945	-0.129266491	PASS
OR10C1	6	29439306	29440977	0.048820648	PASS
OR10D3	11	124183345	124188780	-0.949752196	PASS
OR10G2	14	21633836	21634940	0.060212819	PASS
OR10G3	14	21568520	21580076	0.060212819	PASS
OR10G4	11	124012997	124018732	-0.949752196	PASS
OR10G6	11	123994163	123995161	-0.949752196	PASS
OR10G7	11	124036013	124041325	-0.949752196	PASS
OR10G8	11	124026798	124030634	-0.949752196	PASS
OR10G9	11	124023013	124023948	-0.949752196	PASS
OR10H1	19	15804549	15815664	0.093487731	suppress_segment_too_large
OR10H2	19	15728020	15729060	0.093487731	suppress_segment_too_large
OR10H3	19	15737985	15742343	0.093487731	suppress_segment_too_large
OR10H4	19	15944299	15950350	0.093487731	suppress_segment_too_large
OR10H5	19	15787661	15801025	0.093487731	suppress_segment_too_large
OR10J1	1	159437845	159443078	-0.467855874	PASS
OR10J5	1	159535078	159536007	-0.467855874	PASS

OR10K1	1	158461574	158470857	-0.467855874	PASS
OR10K2	1	158418210	158426237	-0.467855874	PASS
OR10P1	12	55636860	55637854	-0.202191512	PASS
OR10Q1	11	58227882	58228918	-0.129266491	PASS
OR10R2	1	158472220	158480936	-0.467855874	PASS
OR10S1	11	123976661	123977781	-0.949752196	PASS
OR10T2	1	158398522	158399466	-0.467855874	PASS
OR10V1	11	59712916	59713845	-0.129266491	PASS
OR10W1	11	58266792	58268260	-0.129266491	PASS
OR10X1	1	158578919	158579899	-0.467855874	PASS
OR10Z1	1	158605268	158612514	-0.467855874	PASS
OR11A1	6	29425504	29457071	0.048820648	PASS
OR11G2	14	20190894	20201075	-0.238381381	PASS
OR11H1	22	15528159	15529139	-0.575930959	PASS
OR11H12	14	18601045	18602129	-0.238381381	PASS
OR11H2	14	19712904	19714332	-0.238381381	PASS
OR11H4	14	20239286	20244349	0.060212819	PASS
OR11H6	14	20223710	20224702	0.060212819	PASS
OR11L1	1	247840928	247841896	-0.368111568	PASS
OR12D2	6	29395631	29398008	0.048820648	PASS
OR12D3	6	29373423	29375291	0.048820648	PASS
OR13A1	10	45302298	45315608	-0.404852538	PASS
OR13C2	9	104604671	104605627	0.365190713	suppress_segment_too_large
OR13C3	9	104535749	104536856	0.365190713	suppress_segment_too_large
OR13C4	9	104526253	104527209	0.365190713	suppress_segment_too_large
OR13C5	9	104598457	104599413	0.365190713	suppress_segment_too_large
OR13C8	9	104569168	104570130	0.365190713	suppress_segment_too_large
OR13C9	9	104617248	104618204	0.365190713	suppress_segment_too_large
OR13D1	9	104694379	104695485	0.365190713	suppress_segment_too_large
OR13F1	9	104504263	104505222	0.365190713	suppress_segment_too_large
OR13G1	1	247670812	247679739	-0.368111568	PASS
OR13H1	23	131543976	131545056	-0.012567603	PASS
OR13J1	9	35869263	35870601	-0.310444659	PASS
OR14A16	1	247814660	247824119	-0.368111568	PASS
OR14A2	1	247722957	247724043	-0.368111568	PASS
OR14C36	1	248348775	248349713	-0.368111568	PASS
OR14I1	1	248681369	248682304	-0.368111568	PASS
OR14J1	6	29301701	29313017	0.048820648	PASS
OR14K1	1	247738615	247739559	-0.368111568	PASS
OR1A1	17	3207539	3218896	0.128406487	PASS
OR1A2	17	3197519	3198448	0.128406487	PASS
OR1B1	9	122628579	122629573	0.365190713	suppress_segment_too_large
OR1C1	1	247754846	247760556	-0.368111568	PASS
OR1D2	17	3088484	3104422	0.128406487	PASS
OR1D5	17	3062669	3063607	0.128406487	PASS
OR1E1	17	3397104	3398410	0.128406487	PASS

OR1E2	17	3432870	3433841	0.128406487	PASS
OR1F1	16	3204247	3205188	0.069936844	PASS
OR1G1	17	3126584	3127581	0.128406487	PASS
OR1I1	19	15082211	15092970	0.093487731	suppress_segment_too_large
OR1J1	9	122476958	122477926	0.365190713	suppress_segment_too_large
OR1J2	9	122510802	122511743	0.365190713	suppress_segment_too_large
OR1J4	9	122519141	122520082	0.365190713	suppress_segment_too_large
OR1K1	9	122800123	122801073	0.365190713	suppress_segment_too_large
OR1L1	9	122661566	122662648	0.365190713	suppress_segment_too_large
OR1L3	9	122675036	122676153	0.365190713	suppress_segment_too_large
OR1L4	9	122723990	122724925	0.365190713	suppress_segment_too_large
OR1L6	9	122742303	122750783	0.365190713	suppress_segment_too_large
OR1L8	9	122567117	122583384	0.365190713	suppress_segment_too_large
OR1M1	19	9087061	9095669	0.093487731	suppress_segment_too_large
OR1N1	9	122526358	122527296	0.365190713	suppress_segment_too_large
OR1N2	9	122553112	122554214	0.365190713	suppress_segment_too_large
OR1Q1	9	122614738	122615682	0.365190713	suppress_segment_too_large
OR1S1	11	58212720	58216084	-0.129266491	PASS
OR1S2	11	58203202	58204181	-0.129266491	PASS
OR2A1	7	144312464	144322668	-0.388946609	PASS
OR2A12	7	144086278	144098953	-0.388946609	PASS
OR2A14	7	144123176	144131188	-0.388946609	PASS
OR2A2	7	144109514	144110564	-0.388946609	PASS
OR2A25	7	144069811	144075870	-0.388946609	PASS
OR2A4	6	131699644	131701401	-0.458570262	PASS
OR2A42	7	144228244	144239605	-0.388946609	PASS
OR2A5	7	144048948	144058845	-0.388946609	PASS
OR2A7	7	144257663	144264792	-0.388946609	PASS
OR2AE1	7	99875987	99877057	-0.262778904	PASS
OR2AG1	11	6783020	6791558	-0.405444717	PASS
OR2AG2	11	6765626	6771976	-0.405444717	PASS
OR2AJ1	1	247924889	247935339	-0.368111568	PASS
OR2AK2	1	247965233	247966386	-0.368111568	PASS
OR2AP1	12	55572468	55575612	-0.202191512	PASS
OR2AT4	11	75081753	75096876	0.062969153	PASS
OR2B11	1	247449118	247458105	-0.368111568	PASS
OR2B2	6	27911185	27912396	0.048820648	PASS
OR2B3	6	29086208	29087313	0.048820648	PASS
OR2B6	6	27957241	27958182	0.048820648	PASS
OR2C1	16	3355889	3357294	0.069936844	PASS
OR2C3	1	247524677	247536440	-0.368111568	PASS
OR2D2	11	6891490	6892599	-0.405444717	PASS
OR2D3	11	6920974	6922043	-0.405444717	PASS
OR2F1	7	143954844	143997312	-0.388946609	PASS
OR2F2	7	143935166	143936279	-0.388946609	PASS
OR2G2	1	247588360	247589313	-0.368111568	PASS

OR2G3	1	247605586	247606515	-0.368111568	PASS
OR2G6	1	248508073	248527337	-0.368111568	PASS
OR2H1	6	29457181	29464328	0.048820648	PASS
OR2H2	6	29585121	29590500	0.048820648	PASS
OR2I1P	6	29550407	29557721	0.048820648	PASS
OR2J1	6	29099657	29102701	0.048820648	PASS
OR2J2	6	29170907	29175811	0.048820648	PASS
OR2J3	6	29108059	29114770	0.048820648	PASS
OR2K2	9	111327483	111330183	0.365190713	suppress_segment_too_large
OR2L13	1	248095184	248101103	-0.368111568	PASS
OR2L2	1	248030070	248042305	-0.368111568	PASS
OR2L3	1	248046836	248063407	-0.368111568	PASS
OR2L5	1	248013660	248024276	-0.368111568	PASS
OR2L8	1	247948858	247949796	-0.368111568	PASS
OR2M2	1	248174821	248181067	-0.368111568	PASS
OR2M3	1	248197265	248212925	-0.368111568	PASS
OR2M4	1	248231417	248244679	-0.368111568	PASS
OR2M5	1	248145148	248146086	-0.368111568	PASS
OR2M7	1	248323630	248324568	-0.368111568	PASS
OR2S2	9	35957108	35958154	-0.310444659	PASS
OR2T1	1	248403048	248408020	-0.368111568	PASS
OR2T10	1	248590487	248597700	-0.368111568	PASS
OR2T11	1	248623557	248635091	-0.368111568	PASS
OR2T12	1	248290139	248303424	-0.368111568	PASS
OR2T2	1	248445512	248455725	-0.368111568	PASS
OR2T27	1	248649838	248655528	-0.368111568	PASS
OR2T29	1	248556912	248562772	-0.368111568	PASS
OR2T3	1	248473351	248474307	-0.368111568	PASS
OR2T33	1	248269917	248277976	-0.368111568	PASS
OR2T34	1	248573801	248574757	-0.368111568	PASS
OR2T35	1	248636356	248645278	-0.368111568	PASS
OR2T4	1	248361581	248362627	-0.368111568	PASS
OR2T5	1	248488589	248491113	-0.368111568	PASS
OR2T6	1	248375746	248391811	-0.368111568	PASS
OR2T8	1	247920252	247921956	-0.368111568	PASS
OR2V1	5	181123122	181131169	-0.150979337	PASS
OR2V2	5	181147586	181159285	-0.150979337	PASS
OR2W1	6	29044213	29045240	0.048820648	PASS
OR2W3	1	247895587	247896531	-0.368111568	PASS
OR2Y1	5	180739042	180740099	-0.150979337	PASS
OR2Z1	19	8721634	8732160	0.079632875	PASS
OR3A1	17	3291017	3298360	0.128406487	PASS
OR3A2	17	3276942	3386317	0.128406487	PASS
OR3A3	17	3411370	3424070	0.128406487	PASS
OR4A15	11	55367884	55368918	-0.129266491	PASS
OR4A16	11	55343151	55344231	-0.129266491	PASS

OR4A47	11	48488717	48489780	-0.621795272	PASS
OR4A5	11	54706832	54707902	-0.621795272	PASS
OR4B1	11	48216810	48217739	-0.621795272	PASS
OR4C11	11	55602360	55607645	-0.129266491	PASS
OR4C12	11	49981473	49982535	-0.621795272	PASS
OR4C13	11	49952391	49953419	-0.621795272	PASS
OR4C15	11	55554307	55555419	-0.129266491	PASS
OR4C16	11	55572128	55573060	-0.129266491	PASS
OR4C3	11	48324920	48328471	-0.621795272	PASS
OR4C46	11	54603069	54603998	-0.621795272	PASS
OR4C5	11	48365485	48366465	-0.621795272	PASS
OR4C6	11	55662201	55666195	-0.129266491	PASS
OR4D1	17	58148449	58159555	0.088516311	PASS
OR4D10	11	59473315	59479361	-0.129266491	PASS
OR4D11	11	59503576	59504511	-0.129266491	PASS
OR4D2	17	58166982	58171411	0.088516311	PASS
OR4D5	11	123939543	123940637	-0.949752196	PASS
OR4D6	11	59456938	59457991	-0.129266491	PASS
OR4D9	11	59511368	59520703	-0.129266491	PASS
OR4E1	14	21667940	21673818	0.060212819	PASS
OR4E2	14	21653835	21667642	0.060212819	PASS
OR4F15	15	101812202	101820197	-0.697600509	PASS
OR4F17	19	107104	117102	0.079632875	PASS
OR4F21	8	166049	167043	-0.106598973	PASS
OR4F4	15	101922042	101923113	-0.697600509	PASS
OR4F5	1	65419	71585	-0.078582216	PASS
OR4F6	15	101803509	101806887	-0.697600509	PASS
OR4K1	14	19930917	19936757	-0.238381381	PASS
OR4K13	14	20029399	20036038	-0.238381381	PASS
OR4K14	14	20014143	20019307	-0.238381381	PASS
OR4K15	14	19975444	19976659	-0.238381381	PASS
OR4K17	14	20110739	20122199	-0.238381381	PASS
OR4K2	14	19875142	19883932	-0.238381381	PASS
OR4K5	14	19920582	19921659	-0.238381381	PASS
OR4L1	14	20060045	20060983	-0.238381381	PASS
OR4M1	14	19773504	19783696	-0.238381381	PASS
OR4N2	14	19719015	19830253	-0.238381381	PASS
OR4N4	15	22094522	22095472	-0.750600437	PASS
OR4N5	14	20138820	20145471	-0.238381381	PASS
OR4P4	11	55635113	55640309	-0.129266491	PASS
OR4Q3	14	19743571	19749469	-0.238381381	PASS
OR4S1	11	48306223	48307152	-0.621795272	PASS
OR4S2	11	55648327	55652854	-0.129266491	PASS
OR4X1	11	48263861	48264778	-0.621795272	PASS
OR4X2	11	48245056	48246080	-0.621795272	PASS
OR51A2	11	4954772	4955713	-0.957525224	PASS

OR51A4	11	4942831	4947605	-0.957525224	PASS
OR51A7	11	4903783	4909462	-0.957525224	PASS
OR51B2	11	5323359	5324297	-0.957525224	PASS
OR51B4	11	5301014	5301946	-0.957525224	PASS
OR51B5	11	5303444	5505652	-0.957525224	PASS
OR51B6	11	5351508	5352446	-0.957525224	PASS
OR51D1	11	4637477	4643060	-0.957525224	PASS
OR51E1	11	4643420	4655488	-0.957525224	PASS
OR51E2	11	4680171	4697854	-0.957525224	PASS
OR51F1	11	4768979	4769938	-0.957525224	PASS
OR51F2	11	4821321	4822456	-0.957525224	PASS
OR51G1	11	4923374	4924339	-0.957525224	PASS
OR51G2	11	4912588	4919350	-0.957525224	PASS
OR51I1	11	5440570	5441514	-0.957525224	PASS
OR51I2	11	5449323	5456518	-0.957525224	PASS
OR51L1	11	4994851	5005536	-0.957525224	PASS
OR51M1	11	5383812	5393263	-0.957525224	PASS
OR51Q1	11	5422111	5423206	-0.957525224	PASS
OR51S1	11	4847489	4849238	-0.957525224	PASS
OR51T1	11	4881819	4882883	-0.957525224	PASS
OR51V1	11	5199735	5200700	-0.957525224	PASS
OR52A1	11	5143219	5154757	-0.957525224	PASS
OR52A5	11	5128776	5138356	-0.957525224	PASS
OR52B2	11	6169330	6170408	-0.957525224	PASS
OR52B4	11	4367263	4368386	-0.333835828	PASS
OR52B6	11	5580877	5581884	-0.957525224	PASS
OR52D1	11	5488685	5489749	-0.957525224	PASS
OR52E2	11	5058650	5059627	-0.957525224	PASS
OR52E4	11	5880629	5887079	-0.957525224	PASS
OR52E5	11	5893208	5902730	-0.957525224	PASS
OR52E6	11	5840928	5841952	-0.957525224	PASS
OR52E8	11	5856674	5857734	-0.957525224	PASS
OR52H1	11	5544489	5548533	-0.957525224	PASS
OR52I1	11	4593614	4596574	-0.333835828	PASS
OR52I2	11	4581743	4593340	-0.333835828	PASS
OR52J3	11	5046526	5047461	-0.957525224	PASS
OR52K1	11	4482646	4493497	-0.333835828	PASS
OR52K2	11	4449295	4450361	-0.333835828	PASS
OR52L1	11	5985892	5986985	-0.957525224	PASS
OR52M1	11	4545191	4546144	-0.333835828	PASS
OR52N1	11	5786471	5791265	-0.957525224	PASS
OR52N2	11	5820314	5821348	-0.957525224	PASS
OR52N4	11	5754243	5755905	-0.957525224	PASS
OR52N5	11	5776165	5783355	-0.957525224	PASS
OR52R1	11	4803433	4804380	-0.957525224	PASS
OR52W1	11	6199146	6200259	-0.957525224	PASS

OR56A1	11	6019336	6034338	-0.957525224	PASS
OR56A3	11	5938751	5951347	-0.957525224	PASS
OR56A4	11	5999445	6006946	-0.957525224	PASS
OR56A5	11	5967177	5968494	-0.957525224	PASS
OR56B1	11	5736448	5738522	-0.957525224	PASS
OR56B4	11	6107684	6108835	-0.957525224	PASS
OR5A1	11	59436469	59451380	-0.129266491	PASS
OR5A2	11	59416969	59426412	-0.129266491	PASS
OR5AC2	3	98087173	98088102	0.313454204	suppress_segment_too_large
OR5AK2	11	56988872	56989867	-0.129266491	PASS
OR5AN1	11	59358895	59371714	-0.129266491	PASS
OR5AP2	11	56641466	56642471	-0.129266491	PASS
OR5AR1	11	56663662	56664687	-0.129266491	PASS
OR5AS1	11	56027654	56038191	-0.129266491	PASS
OR5AU1	14	21148370	21159060	0.060212819	PASS
OR5B12	11	58439077	58442758	-0.129266491	PASS
OR5B17	11	58358124	58359069	-0.129266491	PASS
OR5B2	11	58421264	58428121	-0.129266491	PASS
OR5B21	11	58507175	58508105	-0.129266491	PASS
OR5B3	11	58402464	58406874	-0.129266491	PASS
OR5C1	9	122788933	122789895	0.365190713	suppress_segment_too_large
OR5D13	11	55773438	55774382	-0.129266491	PASS
OR5D14	11	55795556	55796500	-0.129266491	PASS
OR5D16	11	55838752	55839738	-0.129266491	PASS
OR5D18	11	55819607	55820597	-0.129266491	PASS
OR5F1	11	55993681	55994625	-0.129266491	PASS
OR5H1	3	98130721	98138548	0.313454204	suppress_segment_too_large
OR5H14	3	98147479	98156614	0.313454204	suppress_segment_too_large
OR5H15	3	98166696	98169774	0.313454204	suppress_segment_too_large
OR5H2	3	98282888	98283832	0.313454204	suppress_segment_too_large
OR5H6	3	98263252	98265356	0.313454204	suppress_segment_too_large
OR5I1	11	55935456	55936400	-0.129266491	PASS
OR5J2	11	56176618	56177556	-0.129266491	PASS
OR5K1	3	98463201	98472924	0.313454204	suppress_segment_too_large
OR5K2	3	98497604	98498663	0.313454204	suppress_segment_too_large
OR5K3	3	98390666	98391631	0.313454204	suppress_segment_too_large
OR5K4	3	98353854	98354819	0.313454204	suppress_segment_too_large
OR5L1	11	55811367	55812476	-0.129266491	PASS
OR5L2	11	55827219	55828154	-0.129266491	PASS
OR5M1	11	56609236	56614874	-0.129266491	PASS
OR5M10	11	56576736	56577787	-0.129266491	PASS
OR5M11	11	56542262	56543281	-0.129266491	PASS
OR5M3	11	56469274	56473352	-0.129266491	PASS
OR5M8	11	56490349	56491395	-0.129266491	PASS
OR5M9	11	56462469	56463401	-0.129266491	PASS
OR5P2	11	7795905	7796973	-0.405444717	PASS

OR5P3	11	7824818	7830840	-0.405444717	PASS
OR5R1	11	56417258	56418232	-0.129266491	PASS
OR5T1	11	56274154	56276819	-0.129266491	PASS
OR5T2	11	56231282	56234255	-0.129266491	PASS
OR5T3	11	56252200	56253222	-0.129266491	PASS
OR5V1	6	29353749	29431967	0.048820648	PASS
OR5W2	11	55913650	55914582	-0.129266491	PASS
OR6A2	11	6791736	6799689	-0.405444717	PASS
OR6B1	7	144000320	144008793	-0.388946609	PASS
OR6B2	2	240028965	240030456	0.025928102	PASS
OR6B3	2	240045077	240047027	0.025928102	PASS
OR6C1	12	55314343	55322364	-0.202191512	PASS
OR6C2	12	55444069	55453347	-0.202191512	PASS
OR6C3	12	55330043	55332687	-0.202191512	PASS
OR6C4	12	55549602	55555832	-0.202191512	PASS
OR6C6	12	55293988	55296569	-0.202191512	PASS
OR6C65	12	55400430	55401505	-0.202191512	PASS
OR6C68	12	55492378	55493316	-0.202191512	PASS
OR6C70	12	55469200	55470138	-0.202191512	PASS
OR6C74	12	55247198	55248302	-0.202191512	PASS
OR6C75	12	55362975	55369279	-0.202191512	PASS
OR6C76	12	55426254	55427192	-0.202191512	PASS
OR6F1	1	247711436	247716646	-0.368111568	PASS
OR6J1	14	22630929	22644352	0.060212819	PASS
OR6K2	1	158699678	158700652	-0.467855874	PASS
OR6K3	1	158716327	158720720	-0.467855874	PASS
OR6K6	1	158754720	158755891	-0.467855874	PASS
OR6M1	11	123805335	123806387	-0.949752196	PASS
OR6N1	1	158747814	158772195	-0.467855874	PASS
OR6N2	1	158774222	158781204	-0.467855874	PASS
OR6P1	1	158560606	158570580	-0.467855874	PASS
OR6Q1	11	58030930	58031931	-0.129266491	PASS
OR6S1	14	20640696	20641691	0.060212819	PASS
OR6T1	11	123942785	123943873	-0.949752196	PASS
OR6V1	7	143052320	143053347	-0.388946609	PASS
OR6X1	11	123753488	123754545	-0.949752196	PASS
OR6Y1	1	158544550	158554405	-0.467855874	PASS
OR7A10	19	14840466	14848922	0.093487731	suppress_segment_too_large
OR7A17	19	14878203	14886132	0.093487731	suppress_segment_too_large
OR7A5	19	14824251	14835285	0.093487731	suppress_segment_too_large
OR7C1	19	14789260	14835376	0.093487731	suppress_segment_too_large
OR7C2	19	14941489	14942448	0.093487731	suppress_segment_too_large
OR7D2	19	9178979	9188817	0.093487731	suppress_segment_too_large
OR7D4	19	9210276	9219589	0.093487731	suppress_segment_too_large
OR7E24	19	9247344	9252625	0.093487731	suppress_segment_too_large
OR7E47P	12	52079696	52108239	-0.202191512	PASS

OR7G1	19	9114828	9115763	0.093487731	suppress_segment_too_large
OR7G2	19	9100407	9107475	0.093487731	suppress_segment_too_large
OR7G3	19	9126012	9126950	0.093487731	suppress_segment_too_large
OR8A1	11	124566660	124582942	-0.949752196	PASS
OR8B12	11	124539635	124545333	-0.949752196	PASS
OR8B2	11	124382394	124384462	-0.949752196	PASS
OR8B3	11	124395534	124399024	-0.949752196	PASS
OR8B4	11	124423904	124424893	-0.949752196	PASS
OR8B8	11	124437445	124445696	-0.949752196	PASS
OR8D1	11	124302831	124315099	-0.949752196	PASS
OR8D2	11	124319238	124320288	-0.949752196	PASS
OR8D4	11	123902167	123909229	-0.949752196	PASS
OR8G1	11	124241095	124254364	-0.949752196	PASS
OR8G5	11	124256376	124266218	-0.949752196	PASS
OR8H1	11	56288462	56292254	-0.129266491	PASS
OR8H2	11	56103687	56107658	-0.129266491	PASS
OR8H3	11	56122373	56123311	-0.129266491	PASS
OR8I2	11	56093277	56094286	-0.129266491	PASS
OR8J1	11	56354291	56361511	-0.129266491	PASS
OR8J3	11	56134721	56140201	-0.129266491	PASS
OR8K1	11	56345945	56347031	-0.129266491	PASS
OR8K3	11	56315144	56320639	-0.129266491	PASS
OR8K5	11	56159394	56160317	-0.129266491	PASS
OR8S1	12	48525632	48528103	-0.202191512	PASS
OR8U1	11	56375624	56376553	-0.129266491	PASS
OR9A2	7	143026158	143027195	-0.388946609	PASS
OR9A4	7	141916399	141920625	-0.388946609	PASS
OR9G1	11	56699095	56703884	-0.129266491	PASS
OR9G4	11	56741223	56748697	-0.129266491	PASS
OR9I1	11	58116742	58125530	-0.129266491	PASS
OR9K2	12	55126406	55132750	-0.202191512	PASS
OR9Q1	11	58023881	58181616	-0.129266491	PASS
OR9Q2	11	58189070	58194053	-0.129266491	PASS
ORAI1	12	121626550	121642677	0.202141228	PASS
ORAI2	7	102433106	102456821	-0.388946609	PASS
ORAI3	16	30949066	30956461	0.069936844	PASS
ORC1	1	52372829	52404459	0.033763446	PASS
ORC2	2	200908973	200963680	0.025928102	PASS
ORC3	6	87590067	87667453	-0.368590549	PASS
ORC4	2	147930397	148021604	-0.475215623	PASS
ORC5	7	104126341	104208047	-0.388946609	PASS
ORC6	16	46689643	46698394	0.069936844	PASS
ORM1	9	114323056	114326475	0.365190713	suppress_segment_too_large
ORM2	9	114329869	114333252	0.365190713	suppress_segment_too_large
ORMDL1	2	189770323	189784371	0.025928102	PASS
ORMDL2	12	55817919	55821879	-0.202191512	PASS

ORMDL3	17	39921041	39927601	0.060555011	PASS
OS9	12	57693955	57721557	-0.202191512	PASS
OSBP	11	59574398	59616144	-0.129266491	PASS
OSBP2	22	30693782	30907824	-0.575930959	PASS
OSBPL10	3	31657890	32077580	0.311018244	suppress_segment_too_large
OSBPL11	3	125528858	125595090	0.313454204	suppress_segment_too_large
OSBPL1A	18	24162044	24397880	-0.376397843	PASS
OSBPL2	20	62231922	62296213	-0.168668583	PASS
OSBPL3	7	24796539	24981634	-0.69513118	PASS
OSBPL5	11	3087116	3166739	-0.333835828	PASS
OSBPL6	2	178194481	178402891	0.039726562	PASS
OSBPL7	17	47807372	47821834	0.088516311	PASS
OSBPL8	12	76351797	76559809	0.202141228	PASS
OSBPL9	1	51577179	51798427	0.033763446	PASS
OSCAR	19	54094668	54102692	0.206912349	PASS
OSCP1	1	36415827	36450451	0.033763446	PASS
OSER1	20	44196496	44210791	-0.168668583	PASS
OSGEP	14	20446411	20455105	0.060212819	PASS
OSGEPL1	2	189746660	189763227	0.025928102	PASS
OSGIN1	16	83948282	83966332	-0.371357732	PASS
OSGIN2	8	89901849	89927888	0.279445683	PASS
OSM	22	30262829	30266840	-0.575930959	PASS
OSMR	5	38845858	38945596	0.055007813	PASS
OSR1	2	19351485	19358653	0.040498426	PASS
OSR2	8	98944403	98952104	0.279445683	PASS
OST4	2	27070472	27071773	0.040498426	PASS
OSTC	4	108650584	108667820	-0.011382299	PASS
OSTF1	9	75088543	75147265	0.365190713	suppress_segment_too_large
OSTM1	6	108041409	108165854	-0.368590549	PASS
OSTN	3	191199241	191265615	-0.627320105	PASS
OTC	23	38352545	38421450	-0.012567603	PASS
OTOA	16	21663971	21761935	0.069936844	PASS
OTOF	2	26457203	26558698	0.040498426	PASS
OTOG	11	17547373	17647150	-0.405444717	PASS
OTOGL	12	80099537	80380879	0.202141228	PASS
OTOL1	3	161496808	161503942	0.318278996	suppress_segment_too_large
OTOP1	4	4188803	4226889	0.279089364	PASS
OTOP2	17	74924275	74933912	0.088516311	PASS
OTOP3	17	74935719	74949992	0.088516311	PASS
OTOR	20	16748358	16770062	-0.168668583	PASS
OTOS	2	240139026	240144562	0.025928102	PASS
OTP	5	77628712	77639688	-0.293443042	PASS
OTUB1	11	63985853	64001811	0.182439925	suppress_segment_too_large
OTUB2	14	94026329	94048930	-0.192197564	PASS
OTUD1	10	23439458	23442390	0.039811807	PASS
OTUD3	1	19882513	19912945	0.033763446	PASS

OTUD4	4	145110838	145180161	-0.011382299	PASS
OTUD5	23	48922028	48958386	-0.012567603	PASS
OTUD6A	23	70062491	70064179	-0.012567603	PASS
OTUD6B	8	91070196	91087095	0.279445683	PASS
OTUD7A	15	31475398	31870789	-0.396255771	PASS
OTUD7B	1	149937812	150010676	0.103015788	PASS
OTULIN	5	14664664	14699711	0.055007813	PASS
OTULINL	5	14581775	14615007	0.055007813	PASS
OTX1	2	63050057	63057836	-0.372959126	PASS
OTX2	14	56799905	56810479	0.060212819	PASS
OVCA2	17	2041936	2043430	0.128406487	PASS
OVCH1	12	29412474	29497686	0.362961408	suppress_segment_too_large
OVCH2	11	7689438	7706421	-0.405444717	PASS
OVGP1	1	111414314	111427777	0.033763446	PASS
OVOL1	11	65787022	65797219	0.182439925	suppress_segment_too_large
OVOL2	20	17956979	18059188	-0.168668583	PASS
OVOL3	19	36111151	36113711	0.093487731	suppress_segment_too_large
OXA1L	14	22766522	22773041	0.060212819	PASS
OXCT1	5	41730065	41870519	-0.293443042	PASS
OXCT2	1	39769523	39771348	0.033763446	PASS
OXER1	2	42762481	42764261	0.040498426	PASS
OXGR1	13	96985719	96994730	-0.390786075	PASS
OXLD1	17	81665036	81666635	0.088516311	PASS
OXNAD1	3	16265160	16350299	0.311018244	suppress_segment_too_large
OXR1	8	106359476	106752694	0.279445683	PASS
OXSM	3	25782917	25794534	0.311018244	suppress_segment_too_large
OXSR1	3	38165089	38255488	-0.234617007	PASS
OXT	20	3071620	3072517	-0.168668583	PASS
OXTR	3	8750408	8769628	0.311018244	suppress_segment_too_large
P2RX1	17	3896592	3916500	0.128406487	PASS
P2RX2	12	132618776	132622386	0.270848837	PASS
P2RX3	11	57338374	57370600	-0.129266491	PASS
P2RX4	12	121209857	121234106	0.202141228	PASS
P2RX5	17	3672199	3696404	0.128406487	PASS
P2RX5-TA	17	3663063	3696194	0.128406487	PASS
P2RX6	22	21009808	21028830	-0.575930959	PASS
P2RX7	12	121132819	121188032	0.202141228	PASS
P2RY1	3	152834693	152841439	0.318278996	suppress_segment_too_large
P2RY10	23	78945332	78961954	-0.012567603	PASS
P2RY11	19	10111538	10115372	0.093487731	suppress_segment_too_large
P2RY12	3	151337380	151384812	0.318278996	suppress_segment_too_large
P2RY13	3	151326312	151329548	0.318278996	suppress_segment_too_large
P2RY14	3	151212117	151278467	0.318278996	suppress_segment_too_large
P2RY2	11	73218298	73236352	0.062969153	PASS
P2RY4	23	70258170	70259764	-0.012567603	PASS
P2RY6	11	73264505	73298617	0.062969153	PASS

P2RY8	23	1462572	1537107	-0.012567603	PASS
P3H1	1	42746335	42767084	0.033763446	PASS
P3H2	3	189956728	190122437	0.318278996	suppress_segment_too_large
P3H3	12	6828410	6839851	-0.086128028	PASS
P3H4	17	41801947	41812604	0.060555011	PASS
P4HA1	10	73007217	73096974	0.072405807	PASS
P4HA2	5	132191838	132295315	-0.293443042	PASS
P4HA3	11	74235801	74311657	0.062969153	PASS
P4HB	17	81843159	81860694	0.088516311	PASS
P4HTM	3	48989886	49007154	-0.234617007	PASS
PA2G4	12	56104319	56113907	-0.202191512	PASS
PAAF1	11	73876699	73931124	0.062969153	PASS
PABPC1	8	100685816	100722809	0.279445683	PASS
PABPC1L	20	44910062	44959035	-0.168668583	PASS
PABPC1L2	23	73077276	73079512	-0.012567603	PASS
PABPC3	13	25095868	25099254	-0.390786075	PASS
PABPC4	1	39560816	39576790	0.033763446	PASS
PABPC4L	4	134196333	134201748	-0.011382299	PASS
PABPC5	23	91434595	91438584	-0.012567603	PASS
PABPN1	14	23321289	23326185	0.060212819	PASS
PABPN1L	16	88863333	88866660	-0.371357732	PASS
PACRG	6	162727132	163315492	-0.035448111	PASS
PACRGL	4	20696282	20752907	0.279089364	PASS
PACS1	11	66070363	66244747	0.182439925	suppress_segment_too_large
PACS2	14	105300563	105398147	-0.192197564	PASS
PACSIN1	6	34466061	34535231	0.048820648	PASS
PACSIN2	22	42835412	43015145	-0.575930959	PASS
PACSIN3	11	47177525	47186443	0.117376172	PASS
PADI1	1	17205126	17246005	0.033763446	PASS
PADI2	1	17066761	17119435	0.033763446	PASS
PADI3	1	17249098	17284233	0.033763446	PASS
PADI4	1	17308195	17364004	0.033763446	PASS
PADI6	1	17372196	17401699	0.033763446	PASS
PAEP	9	135561756	135566955	0.365190713	suppress_segment_too_large
PAF1	19	39385852	39391195	0.093487731	suppress_segment_too_large
PAFAH1B	17	2593210	2685615	0.128406487	PASS
PAFAH1B[illegible]	11	117144267	117176894	-0.356188624	PASS
PAFAH1B[illegible]	19	42297033	42303546	0.093487731	suppress_segment_too_large
PAFAH2	1	25959767	25998157	0.033763446	PASS
PAG1	8	80967810	81112068	0.279445683	PASS
PAGE1	23	49687450	49695993	-0.012567603	PASS
PAGE2	23	55089008	55092842	-0.012567603	PASS
PAGE2B	23	55075063	55078909	-0.012567603	PASS
PAGE3	23	55258412	55264846	-0.012567603	PASS
PAGE4	23	49829260	49833973	-0.012567603	PASS
PAGE5	23	55220355	55224108	-0.012567603	PASS

PAGR1	16	29815952	29820117	0.069936844	PASS
PAH	12	102836885	102958410	0.202141228	PASS
PAICS	4	56435741	56464579	-0.011382299	PASS
PAIP1	5	43526267	43557758	-0.293443042	PASS
PAIP2	5	139341587	139369720	-0.293443042	PASS
PAIP2B	2	71182739	71227083	-0.372959126	PASS
PAK1	11	77321707	77474635	0.062969153	PASS
PAK1IP1	6	10694695	10709782	0.048820648	PASS
PAK2	3	196739857	196832647	0.326834516	PASS
PAK3	23	110944285	111227361	-0.012567603	PASS
PAK4	19	39125770	39182816	0.093487731	suppress_segment_too_large
PAK5	20	9537389	9839041	-0.168668583	PASS
PAK6	15	40238759	40277487	-0.396255771	PASS
PALB2	16	23603160	23641310	0.069936844	PASS
PALD1	10	70478821	70568449	0.072405807	PASS
PALLD	4	168497066	168928457	-0.011382299	PASS
PALM	19	708939	748329	0.079632875	PASS
PALM2	9	109640788	109946703	0.365190713	suppress_segment_too_large
PALM2-AI	9	109780309	110172512	0.365190713	suppress_segment_too_large
PALM3	19	14053365	14059159	0.093487731	suppress_segment_too_large
PALMD	1	99645943	99694541	0.033763446	PASS
PAM	5	102753981	103031105	-0.293443042	PASS
PAM16	16	4331549	4355607	0.069936844	PASS
PAMR1	11	35431823	35530300	-0.405444717	PASS
PAN2	12	56316223	56334053	-0.202191512	PASS
PAN3	13	28138506	28295335	-0.390786075	PASS
PANK1	10	89579497	89645572	-0.39678585	PASS
PANK2	20	3888839	3929882	-0.168668583	PASS
PANK3	5	168548495	168579600	-0.150979337	PASS
PANK4	1	2508533	2526628	0.373964873	suppress_segment_too_large
PANO1	11	797511	799190	-0.333835828	PASS
PANX1	11	94128928	94181972	0.062969153	PASS
PANX2	22	50170731	50180294	-0.808211634	PASS
PANX3	11	124611490	124620356	-0.949752196	PASS
PAOX	10	133379234	133391694	0.063874692	PASS
PAPLN	14	73237497	73274640	0.060212819	PASS
PAPOLA	14	96501433	96567111	-0.192197564	PASS
PAPOLB	7	4857733	4861994	-0.69513118	PASS
PAPOLG	2	60756230	60802085	-0.372959126	PASS
PAPPA	9	116153804	116402322	0.365190713	suppress_segment_too_large
PAPPA2	1	176463171	176845605	-0.027073548	PASS
PAPSS1	4	107590276	107720452	-0.011382299	PASS
PAPSS2	10	87659613	87747705	-1.64589449	suppress_large_homdel
PAQR3	4	78887127	78939438	-0.011382299	PASS
PAQR4	16	2969245	2973489	0.069936844	PASS
PAQR5	15	69298947	69407780	-0.697600509	PASS

PAQR6	1	156243321	156248117	0.103015788	PASS
PAQR7	1	25861210	25871253	0.033763446	PASS
PAQR8	6	52361421	52407777	0.048820648	PASS
PAQR9	3	142949164	142963682	0.318278996	suppress_segment_too_large
PARD3	10	34109560	34815325	0.039811807	PASS
PARD3B	2	204545793	205620162	0.025928102	PASS
PARD6A	16	67660946	67662778	-0.371357732	PASS
PARD6B	20	50731544	50756795	-0.168668583	PASS
PARD6G	18	80157232	80247546	-0.376397843	PASS
PARG	10	49818279	49970203	-0.404852538	PASS
PARK7	1	7954291	7985505	0.373964873	suppress_segment_too_large
PARL	3	183829271	183884933	0.318278996	suppress_segment_too_large
PARM1	4	74933095	75050115	-0.011382299	PASS
PARN	16	14435701	14632728	0.069936844	PASS
PARP1	1	226360691	226408079	-0.256698369	PASS
PARP10	8	143977153	144012772	0.307788765	PASS
PARP11	12	3791047	3873448	-0.086128028	PASS
PARP12	7	140023744	140063721	-0.388946609	PASS
PARP14	3	122680618	122730840	0.313454204	suppress_segment_too_large
PARP15	3	122577602	122639047	0.313454204	suppress_segment_too_large
PARP16	15	65234460	65300618	-0.697600509	PASS
PARP2	14	20343582	20357905	0.060212819	PASS
PARP3	3	51942345	51948867	-0.234617007	PASS
PARP4	13	24420926	24512810	-0.390786075	PASS
PARP6	15	72241181	72272999	-0.697600509	PASS
PARP8	5	50665899	50846522	-0.293443042	PASS
PARP9	3	122527924	122564577	0.313454204	suppress_segment_too_large
PARPBP	12	102120185	102197520	0.202141228	PASS
PARS2	1	54756898	54764514	0.033763446	PASS
PARVA	11	12377185	12535356	-0.405444717	PASS
PARVB	22	43999211	44172949	-0.808211634	PASS
PARVG	22	44172956	44219533	-0.808211634	PASS
PASD1	23	151563622	151676739	-0.012567603	PASS
PASK	2	241106099	241150264	0.025928102	PASS
PATE1	11	125746293	125749867	-0.419612865	PASS
PATE2	11	125776113	125778819	-0.419612865	PASS
PATE3	11	125788111	125791600	-0.419612865	PASS
PATE4	11	125833316	125840069	-0.419612865	PASS
PATJ	1	61742406	62178675	0.033763446	PASS
PATL1	11	59636716	59668980	-0.129266491	PASS
PATL2	15	44665732	44711316	-0.396255771	PASS
PATZ1	22	31325804	31346232	-0.575930959	PASS
PAWR	12	79574979	79691097	0.202141228	PASS
PAX1	20	21705659	21718486	-0.168668583	PASS
PAX2	10	100735603	100829941	-0.367947924	PASS
PAX3	2	222199888	222298996	0.025928102	PASS

PAX4	7	127610292	127618114	-0.388946609	PASS
PAX5	9	36833275	37034185	-0.310444659	PASS
PAX6	11	31784779	31818062	-0.405444717	PASS
PAX7	1	18631006	18748866	0.033763446	PASS
PAX8	2	113215997	113278950	-0.010901494	PASS
PAX9	14	36657568	36679715	0.060212819	PASS
PAXBP1	21	32733899	32771858	0.12860881	suppress_segment_too_large
PAXIP1	7	154943687	155003084	-0.411415677	PASS
PAXX	9	136992418	136993984	0.365190713	suppress_segment_too_large
PBDC1	23	76172936	76178204	-0.012567603	PASS
PBK	8	27809620	27838095	-0.345962679	PASS
PBLD	10	68282660	68333049	0.072405807	PASS
PBOV1	6	138215986	138218491	-0.035448111	PASS
PBRM1	3	52545352	52685917	-0.234617007	PASS
PBX1	1	164555584	164899296	-0.027073548	PASS
PBX2	6	32184741	32190186	0.048820648	PASS
PBX3	9	125747345	125967377	0.365190713	suppress_segment_too_large
PBX4	19	19561707	19618916	0.093487731	suppress_segment_too_large
PBXIP1	1	154944076	154956123	0.103015788	PASS
PC	11	66848233	66958376	0.182439925	suppress_segment_too_large
PCARE	2	29060976	29074261	0.040498426	PASS
PCBD1	10	70882280	70888784	0.072405807	PASS
PCBD2	5	134904906	135007959	-0.293443042	PASS
PCBP1	2	70087454	70089203	-0.372959126	PASS
PCBP2	12	53452102	53481162	-0.202191512	PASS
PCBP3	21	45643694	45942454	-0.064425456	suppress_segment_too_large
PCBP4	3	51957454	51974016	-0.234617007	PASS
PCCA	13	100089015	100530437	-0.390786075	PASS
PCCB	3	136250306	136337896	0.313454204	suppress_segment_too_large
PCDH1	5	141853111	141879246	-0.293443042	PASS
PCDH10	4	133149315	133208606	-0.011382299	PASS
PCDH11X	23	91779261	92623230	-0.012567603	PASS
PCDH12	5	141943585	141969741	-0.293443042	PASS
PCDH15	10	53802771	55627942	-0.404852538	PASS
PCDH17	13	57631810	57729311	-0.390786075	PASS
PCDH18	4	137518918	137532494	-0.011382299	PASS
PCDH19	23	100291644	100410273	-0.012567603	PASS
PCDH20	13	61409685	61415522	-0.390786075	PASS
PCDH7	4	30720415	31146805	-0.011382299	PASS
PCDH8	13	52842889	52848641	-0.390786075	PASS
PCDH9	13	66302834	67230445	-0.390786075	PASS
PCDHA1	5	140786136	141012347	-0.293443042	PASS
PCDHA10	5	140855883	141012344	-0.293443042	PASS
PCDHA11	5	140868183	141012344	-0.293443042	PASS
PCDHA12	5	140875302	141012344	-0.293443042	PASS
PCDHA13	5	140882208	141012344	-0.293443042	PASS

PCDHA2	5	140794852	141012344	-0.293443042	PASS
PCDHA3	5	140801028	141012344	-0.293443042	PASS
PCDHA4	5	140806929	141012344	-0.293443042	PASS
PCDHA5	5	140821604	141012344	-0.293443042	PASS
PCDHA6	5	140827958	141012344	-0.293443042	PASS
PCDHA7	5	140834248	141012344	-0.293443042	PASS
PCDHA8	5	140841187	141012344	-0.293443042	PASS
PCDHA9	5	140847463	141012344	-0.293443042	PASS
PCDHAC1	5	140926369	141012344	-0.293443042	PASS
PCDHAC2	5	140966235	141012344	-0.293443042	PASS
PCDHB1	5	141051135	141059344	-0.293443042	PASS
PCDHB10	5	141192353	141195642	-0.293443042	PASS
PCDHB11	5	141199582	141203779	-0.293443042	PASS
PCDHB12	5	141208697	141212571	-0.293443042	PASS
PCDHB13	5	141213919	141218979	-0.293443042	PASS
PCDHB14	5	141222932	141227759	-0.293443042	PASS
PCDHB15	5	141245349	141249365	-0.293443042	PASS
PCDHB16	5	141181399	141186399	-0.293443042	PASS
PCDHB2	5	141094578	141098703	-0.293443042	PASS
PCDHB3	5	141100473	141103827	-0.293443042	PASS
PCDHB4	5	141121799	141125623	-0.293443042	PASS
PCDHB5	5	141135218	141138625	-0.293443042	PASS
PCDHB6	5	141150022	141153287	-0.293443042	PASS
PCDHB7	5	141172619	141176383	-0.293443042	PASS
PCDHB8	5	141177790	141180529	-0.293443042	PASS
PCDHB9	5	141187127	141191541	-0.293443042	PASS
PCDHGA1	5	141330571	141512981	-0.293443042	PASS
PCDHGA1	5	141412987	141512979	-0.293443042	PASS
PCDHGA1	5	141421047	141512979	-0.293443042	PASS
PCDHGA1	5	141430589	141512979	-0.293443042	PASS
PCDHGA2	5	141338760	141512979	-0.293443042	PASS
PCDHGA3	5	141343829	141512979	-0.293443042	PASS
PCDHGA4	5	141355025	141512979	-0.293443042	PASS
PCDHGA5	5	141364232	141512979	-0.293443042	PASS
PCDHGA6	5	141373914	141512979	-0.293443042	PASS
PCDHGA7	5	141382739	141512979	-0.293443042	PASS
PCDHGA8	5	141390157	141512979	-0.293443042	PASS
PCDHGA9	5	141402932	141512979	-0.293443042	PASS
PCDHGB1	5	141350102	141512979	-0.293443042	PASS
PCDHGB2	5	141360042	141512979	-0.293443042	PASS
PCDHGB3	5	141370264	141512979	-0.293443042	PASS
PCDHGB4	5	141387698	141512979	-0.293443042	PASS
PCDHGB5	5	141397987	141512979	-0.293443042	PASS
PCDHGB6	5	141408021	141512979	-0.293443042	PASS
PCDHGB7	5	141417645	141512979	-0.293443042	PASS
PCDHGC3	5	141475947	141512979	-0.293443042	PASS

PCDHGC4	5	141484997	141512979	-0.293443042	PASS
PCDHGC5	5	141489121	141512979	-0.293443042	PASS
PCED1A	20	2835314	2841190	-0.168668583	PASS
PCED1B	12	47079603	47236662	-0.202191512	PASS
PCF11	11	83156988	83187451	0.062969153	PASS
PCGF1	2	74505043	74508580	-0.372959126	PASS
PCGF2	17	38733897	38749817	0.060555011	PASS
PCGF3	4	705748	770640	0.279089364	PASS
PCGF5	10	91163012	91284331	-0.39678585	PASS
PCGF6	10	103302796	103351144	-0.367947924	PASS
PCID2	13	113177539	113208715	-0.390786075	PASS
PCIF1	20	45934628	45948023	-0.168668583	PASS
PCK1	20	57561080	57568112	-0.168668583	PASS
PCK2	14	24094053	24110598	0.060212819	PASS
PCLAF	15	64364311	64387687	-0.697600509	PASS
PCLO	7	82754013	83162930	-0.262778904	PASS
PCM1	8	17922840	18029944	-0.345962679	PASS
PCMT1	6	149749443	149811420	-0.035448111	PASS
PCMTD1	8	51817575	51899186	-0.201142258	PASS
PCMTD2	20	64255695	64287821	-0.168668583	PASS
PCNA	20	5114953	5126626	-0.168668583	PASS
PCNP	3	101574095	101594437	0.313454204	suppress_segment_too_large
PCNT	21	46324122	46445769	-0.064425456	suppress_segment_too_large
PCNX1	14	70907405	71115382	0.060212819	PASS
PCNX2	1	232983435	233295713	-0.256698369	PASS
PCNX3	11	65615773	65637439	0.182439925	suppress_segment_too_large
PCNX4	14	60091911	60169133	0.060212819	PASS
PCOLCE	7	100602177	100608175	-0.262778904	PASS
PCOLCE2	3	142815922	142889206	0.318278996	suppress_segment_too_large
PCP2	19	7631611	7633748	0.079632875	PASS
PCP4	21	39867317	39929397	-0.064425456	suppress_segment_too_large
PCP4L1	1	161258727	161285450	-0.027073548	PASS
PCSK1	5	96390415	96434143	-0.293443042	PASS
PCSK1N	23	48831094	48835633	-0.012567603	PASS
PCSK2	20	17226107	17484578	-0.168668583	PASS
PCSK4	19	1481428	1490752	0.079632875	PASS
PCSK5	9	75890644	76362339	0.365190713	suppress_segment_too_large
PCSK6	15	101297142	101525202	-0.697600509	PASS
PCSK7	11	117204337	117232525	-0.356188624	PASS
PCSK9	1	55039548	55064852	0.033763446	PASS
PCTP	17	55750979	55842830	0.088516311	PASS
PCYOX1	2	70257386	70281191	-0.372959126	PASS
PCYOX1L	5	149358007	149369653	-0.293443042	PASS
PCYT1A	3	196214222	196287957	0.326834516	PASS
PCYT1B	23	24558087	24672677	-0.012567603	PASS
PCYT2	17	81900965	81911464	0.088516311	PASS

PDAP1	7	99392048	99408829	-0.262778904	PASS
PDC	1	186443566	186461122	-0.027073548	PASS
PDCD10	3	167683298	167734939	0.318278996	suppress_segment_too_large
PDCD11	10	103396626	103446292	-0.367947924	PASS
PDCD1LG	9	5510570	5571254	-0.310444659	PASS
PDCD2	6	170575295	170584692	-0.035448111	PASS
PDCD2L	19	34404384	34426168	0.093487731	suppress_segment_too_large
PDCD4	10	110871795	110900006	0.063874692	PASS
PDCD5	19	32581068	32587452	0.093487731	suppress_segment_too_large
PDCD6	5	271621	353856	0.055007813	PASS
PDCD6IP	3	33798352	33869707	0.311018244	suppress_segment_too_large
PDCD7	15	65117379	65133836	-0.697600509	PASS
PDCL	9	122798389	122828631	0.365190713	suppress_segment_too_large
PDCL2	4	55556525	55592212	-0.011382299	PASS
PDCL3	2	100562690	100576735	0.048238121	PASS
PDE10A	6	165327287	165988078	-0.035448111	PASS
PDE11A	2	177623252	178108339	0.039726562	PASS
PDE12	3	57556276	57566844	-0.234617007	PASS
PDE1A	2	182140036	182523192	0.039726562	PASS
PDE1B	12	54549350	54579239	-0.202191512	PASS
PDE1C	7	31751179	32299329	-0.69513118	PASS
PDE2A	11	72576141	72674591	0.062969153	PASS
PDE3A	12	20369245	20684381	0.362961408	suppress_segment_too_large
PDE3B	11	14643723	14872044	-0.405444717	PASS
PDE4A	19	10416773	10469631	0.093487731	suppress_segment_too_large
PDE4B	1	65792514	66374579	0.033763446	PASS
PDE4C	19	18208652	18248200	0.093487731	suppress_segment_too_large
PDE4D	5	58969038	60522120	-0.293443042	PASS
PDE4DIP	1	148808181	149048286	-0.157385092	PASS
PDE5A	4	119494395	119628991	-0.011382299	PASS
PDE6A	5	149857955	149944793	-0.293443042	PASS
PDE6B	4	625584	670782	0.279089364	PASS
PDE6C	10	93612588	93666010	-0.39678585	PASS
PDE6D	2	231732425	231786272	0.025928102	PASS
PDE6G	17	81650459	81663112	0.088516311	PASS
PDE6H	12	14973022	14981865	0.362961408	suppress_segment_too_large
PDE7A	8	65717510	65842322	-0.201142258	PASS
PDE7B	6	135851696	136195574	-0.035448111	PASS
PDE8A	15	84980440	85139145	-0.697600509	PASS
PDE8B	5	77210449	77429807	-0.293443042	PASS
PDE9A	21	42653636	42775509	-0.064425456	suppress_segment_too_large
PDF	16	69328621	69330595	-0.371357732	PASS
PDGFA	7	497258	520296	-0.69513118	PASS
PDGFB	22	39223359	39244751	-0.575930959	PASS
PDGFC	4	156760454	156971394	-0.011382299	PASS
PDGFD	11	103907186	104164379	0.062969153	PASS

PDGFRA	4	54229097	54298247	-0.011382299	PASS
PDGFRB	5	150113837	150155872	-0.293443042	PASS
PDGFRL	8	17576433	17644071	-0.345962679	PASS
PDHA1	23	19343893	19361705	-0.012567603	PASS
PDHA2	4	95840019	95841474	-0.011382299	PASS
PDHB	3	58427630	58433857	-0.234617007	PASS
PDHX	11	34915829	35020591	-0.405444717	PASS
PDIA2	16	283152	287215	0.069936844	PASS
PDIA3	15	43746392	43773279	-0.396255771	PASS
PDIA4	7	149003062	149028641	-0.388946609	PASS
PDIA5	3	123067062	123225227	0.313454204	suppress_segment_too_large
PDIA6	2	10783391	10837977	0.040498426	PASS
PDIK1L	1	26111165	26125543	0.033763446	PASS
PDILT	16	20359170	20404737	0.069936844	PASS
PDK1	2	172555373	172608669	0.039726562	PASS
PDK2	17	50094737	50112152	0.088516311	PASS
PDK3	23	24465221	24541862	-0.012567603	PASS
PDK4	7	95583499	95596491	-0.262778904	PASS
PDLIM1	10	95237572	95291024	-0.39678585	PASS
PDLIM2	8	22578279	22598025	-0.345962679	PASS
PDLIM3	4	185500660	185535612	-0.462744716	PASS
PDLIM4	5	132257671	132273454	-0.293443042	PASS
PDLIM5	4	94451857	94668227	-0.011382299	PASS
PDLIM7	5	177483394	177497606	-0.150979337	PASS
PDP1	8	93857807	93926066	0.279445683	PASS
PDP2	16	66878589	66895754	-0.371357732	PASS
PDPK1	16	2537964	2603188	0.069936844	PASS
PDPN	1	13583465	13617957	0.033763446	PASS
PDPR	16	70113626	70162537	-0.371357732	PASS
PDRG1	20	31944342	31952092	-0.168668583	PASS
PDS5A	4	39822863	39977956	-0.011382299	PASS
PDS5B	13	32586427	32778019	-0.390786075	PASS
PDSS1	10	26697659	26746798	0.039811807	PASS
PDSS2	6	107152557	107459564	-0.368590549	PASS
PDX1	13	27920020	27926231	-0.390786075	PASS
PDXDC1	16	14974591	15139339	0.069936844	PASS
PDXK	21	43719094	43762307	-0.064425456	suppress_segment_too_large
PDXP	22	37658727	37666934	-0.575930959	PASS
PDYN	20	1978757	1994285	-0.168668583	PASS
PDZD11	23	70286595	70290514	-0.012567603	PASS
PDZD2	5	31639410	32110931	0.055007813	PASS
PDZD3	11	119185457	119190223	-0.356188624	PASS
PDZD4	23	153802166	153830565	-0.012567603	PASS
PDZD7	10	101007683	101032295	-0.367947924	PASS
PDZD8	10	117277274	117375467	0.063874692	PASS
PDZD9	16	21983865	22001110	0.069936844	PASS

PDZK1	1	145670852	145708148		-0.157385092	PASS
PDZK1IP1	1	47183593	47191044		0.033763446	PASS
PDZRN3	3	73382433	73624940		-0.234617007	PASS
PDZRN4	12	41188448	41574724		-0.202191512	PASS
PEA15	1	160205337	160215376		-0.027073548	PASS
PEAK1	15	77100656	77420144		-0.697600509	PASS
PEAK3	19	2274622	2282176		0.079632875	PASS
PEAR1	1	156893698	156916434		0.103015788	PASS
PEBP1	12	118135858	118145584		0.202141228	PASS
PEBP4	8	22713251	23000000		-0.345962679	PASS
PECAM1	17	64319415	64413776		0.088516311	PASS
PECR	2	215996329	216082955		0.025928102	PASS
PEF1	1	31629862	31644896		0.033763446	PASS
PEG10	7	94656325	94669695		-0.262778904	PASS
PEG3	19	56810077	56840728		-0.180829161	PASS
PELI1	2	64092652	64144454		-0.372959126	PASS
PELI2	14	56117814	56301526		0.060212819	PASS
PELI3	11	66466327	66477337		0.182439925	suppress_segment_too_large
PELO	5	52787940	52804046		-0.293443042	PASS
PELP1	17	4669774	4704337		0.128406487	PASS
PEMT	17	17505563	17591708		0.024610076	PASS
PENK	8	56436674	56446734		-0.201142258	PASS
PEPD	19	33386950	33521794		0.093487731	suppress_segment_too_large
PER1	17	8140472	8156506		0.128406487	PASS
PER2	2	238244038	238290102		0.025928102	PASS
PER3	1	7784320	7845177		0.373964873	suppress_segment_too_large
PERM1	1	975204	982093		0.373964873	suppress_segment_too_large
PERP	6	138088505	138107511		-0.035448111	PASS
PES1	22	30576625	30607083		-0.575930959	PASS
PET100	19	7629737	7631956		0.079632875	PASS
PET117	20	18137873	18143169		-0.168668583	PASS
PEX1	7	92487020	92528531		-0.262778904	PASS
PEX10	1	2403964	2413797		0.373964873	suppress_segment_too_large
PEX11A	15	89677764	89690783		-0.697600509	PASS
PEX11B	1	145911350	145918837		-0.157385092	PASS
PEX11G	19	7476875	7497449		0.079632875	PASS
PEX12	17	35574795	35578863		0.060555011	PASS
PEX13	2	61017225	61051990		-0.372959126	PASS
PEX14	1	10472288	10630758		0.373964873	suppress_segment_too_large
PEX16	11	45909669	45918812		0.117376172	PASS
PEX19	1	160276812	160286348		-0.027073548	PASS
PEX2	8	76980258	77001044		-0.201142258	PASS
PEX26	22	18077920	18131138		-0.575930959	PASS
PEX3	6	143450807	143490010		-0.035448111	PASS
PEX5	12	7188685	7218574		-0.086128028	PASS
PEX5L	3	179794958	180037053		0.318278996	suppress_segment_too_large

PEX6	6	42963870	42979220	0.048820648	PASS
PEX7	6	136822564	136913937	-0.035448111	PASS
PF4	4	73981077	73982124	-0.011382299	PASS
PF4V1	4	73853189	73854155	-0.011382299	PASS
PFAS	17	8247618	8270491	0.128406487	PASS
PFDN1	5	140245039	140303121	-0.293443042	PASS
PFDN2	1	161100556	161118111	-0.027073548	PASS
PFDN4	20	54207847	54228052	-0.168668583	PASS
PFDN5	12	53295291	53299450	-0.202191512	PASS
PFDN6	6	33289302	33298401	0.048820648	PASS
PFKFB1	23	54932961	54998534	-0.012567603	PASS
PFKFB2	1	207034366	207081024	-0.256698369	PASS
PFKFB3	10	6144934	6254644	0.039811807	PASS
PFKFB4	3	48517684	48562015	-0.234617007	PASS
PFKL	21	44300051	44327376	-0.064425456	suppress_segment_too_large
PFKM	12	48105139	48146404	-0.202191512	PASS
PFKP	10	3066333	3137712	0.039811807	PASS
PFN1	17	4945652	4949061	0.128406487	PASS
PFN2	3	149964904	150050788	0.318278996	suppress_segment_too_large
PFN3	5	177400107	177400636	-0.150979337	PASS
PFN4	2	24115371	24123477	0.040498426	PASS
PGA3	11	61203307	61213098	0.182439925	suppress_segment_too_large
PGA5	11	61241042	61251448	0.182439925	suppress_segment_too_large
PGAM1	10	97426160	97433441	-0.367947924	PASS
PGAM2	7	44062727	44065587	-0.69513118	PASS
PGAM4	23	77968874	77969638	-0.012567603	PASS
PGAM5	12	132710819	132722734	0.270848837	PASS
PGAP1	2	196833004	196927796	0.025928102	PASS
PGAP2	11	3797724	3826371	-0.333835828	PASS
PGAP3	17	39671122	39696797	0.060555011	PASS
PGBD1	6	28281572	28302549	0.048820648	PASS
PGBD2	1	248906196	248919946	0.023554025	PASS
PGBD4	15	34102073	34108684	-0.396255771	PASS
PGBD5	1	230314482	230426371	-0.256698369	PASS
PGC	6	41736711	41754109	0.048820648	PASS
PGD	1	10398592	10420144	0.373964873	suppress_segment_too_large
PGF	14	74941834	74955784	-0.192197564	PASS
PGGHG	11	289135	296107	-0.333835828	PASS
PGGT1B	5	115204012	115262872	-0.293443042	PASS
PGK1	23	77910739	78129296	-0.012567603	PASS
PGK2	6	49785651	49787307	0.048820648	PASS
PGLS	19	17511629	17521288	0.093487731	suppress_segment_too_large
PGLYRP1	19	46019153	46023065	0.14996833	PASS
PGLYRP2	19	15468645	15498956	0.093487731	suppress_segment_too_large
PGLYRP3	1	153297862	153310718	0.103015788	PASS
PGLYRP4	1	153330120	153348840	0.103015788	PASS

PGM1	1	63593276	63660245	0.033763446	PASS
PGM2	4	37826633	37862937	-0.011382299	PASS
PGM2L1	11	74330318	74398473	0.062969153	PASS
PGM3	6	83161150	83193936	-0.368590549	PASS
PGM5	9	68328308	68531061	-0.310444659	PASS
PGP	16	2211997	2214807	0.069936844	PASS
PGPEP1	19	18340587	18369950	0.093487731	suppress_segment_too_large
PGPEP1L	15	98968230	99007795	-0.697600509	PASS
PGR	11	101029624	101130524	0.062969153	PASS
PGRMC1	23	119236245	119244466	-0.012567603	PASS
PGRMC2	4	128269237	128288829	-0.011382299	PASS
PGS1	17	78378640	78425114	0.088516311	PASS
PHACTR1	6	12716805	13290484	0.048820648	PASS
PHACTR2	6	143536845	143831185	-0.035448111	PASS
PHACTR3	20	59577509	59847711	-0.168668583	PASS
PHACTR4	1	28369582	28500369	0.033763446	PASS
PHAX	5	126600268	126627252	-0.293443042	PASS
PHB	17	49404049	49414905	0.088516311	PASS
PHB2	12	6965327	6970825	-0.086128028	PASS
PHC1	12	8913896	8941467	0.362961408	suppress_segment_too_large
PHC2	1	33323623	33431052	0.033763446	PASS
PHC3	3	170086732	170181749	0.318278996	suppress_segment_too_large
PHETA1	12	111360651	111369121	0.202141228	PASS
PHETA2	22	42074251	42079441	-0.575930959	PASS
PHEX	23	22032441	22251310	-0.012567603	PASS
PHF1	6	33410399	33416453	0.048820648	PASS
PHF10	6	169703905	169725566	-0.035448111	PASS
PHF11	13	49495610	49528987	-0.390786075	PASS
PHF12	17	28905250	28951771	0.060555011	PASS
PHF13	1	6613685	6624033	0.373964873	suppress_segment_too_large
PHF14	7	10973336	11169630	-0.69513118	PASS
PHF19	9	120855652	120894896	0.365190713	suppress_segment_too_large
PHF2	9	93576407	93679587	0.365190713	suppress_segment_too_large
PHF20	20	35771974	35950381	-0.168668583	PASS
PHF20L1	8	132775358	132848807	0.113969953	PASS
PHF21A	11	45929323	46121178	0.117376172	PASS
PHF21B	22	44881162	45009999	-0.808211634	PASS
PHF23	17	7235028	7239722	0.128406487	PASS
PHF24	9	34957608	34982544	-0.310444659	PASS
PHF3	6	63635820	63779336	0.048820648	PASS
PHF5A	22	41459717	41468725	-0.575930959	PASS
PHF6	23	134373253	134428791	-0.012567603	PASS
PHF7	3	52410657	52423641	-0.234617007	PASS
PHF8	23	53936676	54048958	-0.012567603	PASS
PHGDH	1	119648411	119744226	-0.157385092	PASS
PHGR1	15	40351033	40356434	-0.396255771	PASS

PHIP	6	78935867	79078236	-0.368590549	PASS
PHKA1	23	72578814	72714319	-0.012567603	PASS
PHKA2	23	18892300	18984598	-0.012567603	PASS
PHKB	16	47461123	47701523	0.069936844	PASS
PHKG1	7	56080283	56092996	-0.750061359	PASS
PHKG2	16	30748270	30761176	0.069936844	PASS
PHLDA1	12	76025447	76033932	0.202141228	PASS
PHLDA2	11	2928273	2929455	-0.333835828	PASS
PHLDA3	1	201464383	201469237	-0.256698369	PASS
PHLDB1	11	118606440	118658038	-0.356188624	PASS
PHLDB2	3	111732497	111976517	0.313454204	suppress_segment_too_large
PHLDB3	19	43474954	43504935	0.14996833	PASS
PHLPP1	18	62715450	62980433	-0.376397843	PASS
PHLPP2	16	71637835	71724701	-0.371357732	PASS
PHOSPHO	17	49223362	49230766	0.088516311	PASS
PHOSPHO	2	169694454	169701708	0.039726562	PASS
PHOX2A	11	72239077	72245664	0.062969153	PASS
PHOX2B	4	41744082	41748970	-0.011382299	PASS
PHPT1	9	136848724	136851027	0.365190713	suppress_segment_too_large
PHRF1	11	576486	612222	-0.333835828	PASS
PHTF1	1	113696831	113759489	0.033763446	PASS
PHTF2	7	77798792	77957503	-0.262778904	PASS
PHYH	10	13277796	13302412	0.039811807	PASS
PHYHD1	9	128920895	128942041	0.365190713	suppress_segment_too_large
PHYHIP	8	22219704	22232341	-0.345962679	PASS
PHYHIPL	10	59176590	59247774	0.072405807	PASS
PHYKPL	5	178208497	178232791	-0.150979337	PASS
PI15	8	74824534	74855029	-0.201142258	PASS
PI16	6	36948263	36964837	0.048820648	PASS
PI3	20	45174876	45176544	-0.168668583	PASS
PI4K2A	10	97640686	97676434	-0.367947924	PASS
PI4K2B	4	25233975	25279092	0.279089364	PASS
PI4KA	22	20707691	20859417	-0.575930959	PASS
PI4KB	1	151291797	151327715	0.103015788	PASS
PIANP	12	6693792	6700800	-0.086128028	PASS
PIAS1	15	68054179	68198603	-0.697600509	PASS
PIAS2	18	46803224	46920160	-0.376397843	PASS
PIAS3	1	145848522	145859836	-0.157385092	PASS
PIAS4	19	4007646	4039386	0.079632875	PASS
PIBF1	13	72782059	73016461	-0.390786075	PASS
PICALM	11	85957684	86069882	0.062969153	PASS
PICK1	22	38056311	38075701	-0.575930959	PASS
PID1	2	228850526	229271285	0.025928102	PASS
PIDD1	11	799179	809753	-0.333835828	PASS
PIEZO1	16	88715338	88785220	-0.371357732	PASS
PIEZO2	18	10666483	11148762	-0.052305056	suppress_segment_too_large

PIF1	15	64815632	64825668	-0.697600509	PASS
PIFO	1	111346288	111353013	0.033763446	PASS
PIGA	23	15319451	15335580	-0.012567603	PASS
PIGB	15	55318960	55355648	-0.697600509	PASS
PIGBOS1	15	55317184	55319161	-0.697600509	PASS
PIGC	1	172370189	172444086	-0.027073548	PASS
PIGF	2	46580937	46617119	0.040498426	PASS
PIGG	4	499210	540196	0.279089364	PASS
PIGH	14	67581955	67600287	0.060212819	PASS
PIGK	1	77088990	77219430	0.033763446	PASS
PIGL	17	16217191	16351797	0.024610076	PASS
PIGM	1	160024953	160031991	-0.027073548	PASS
PIGN	18	61905255	62187118	-0.376397843	PASS
PIGO	9	35088688	35096601	-0.310444659	PASS
PIGP	21	37059170	37073170	-0.064425456	suppress_segment_too_large
PIGQ	16	566995	584136	0.069936844	PASS
PIGR	1	206928518	206946466	-0.256698369	PASS
PIGS	17	28553383	28571872	0.060555011	PASS
PIGT	20	45416067	45456934	-0.168668583	PASS
PIGU	20	34560542	34698790	-0.168668583	PASS
PIGV	1	26787472	26798398	0.033763446	PASS
PIGW	17	36535020	36539310	0.060555011	PASS
PIGX	3	196639775	196736007	0.326834516	PASS
PIGY	4	88521573	88521789	-0.011382299	PASS
PIGZ	3	196946343	196969060	0.326834516	PASS
PIH1D1	19	49446298	49453497	0.14996833	PASS
PIH1D2	11	112064010	112074274	-0.356188624	PASS
PIH1D3	23	107206632	107244243	-0.012567603	PASS
PIK3AP1	10	96593312	96720514	-0.367947924	PASS
PIK3C2A	11	17077730	17207983	-0.405444717	PASS
PIK3C2B	1	204422628	204494724	-0.256698369	PASS
PIK3C2G	12	18247614	18648416	0.362961408	suppress_segment_too_large
PIK3C3	18	41955206	42087830	-0.376397843	PASS
PIK3CA	3	179148114	179240093	0.318278996	suppress_segment_too_large
PIK3CB	3	138652699	138834938	0.313454204	suppress_segment_too_large
PIK3CD	1	9651732	9729114	0.373964873	suppress_segment_too_large
PIK3CG	7	106865278	106907145	-0.388946609	PASS
PIK3IP1	22	31281593	31292534	-0.575930959	PASS
PIK3R1	5	68215720	68301821	-0.293443042	PASS
PIK3R2	19	18153118	18170540	0.093487731	suppress_segment_too_large
PIK3R3	1	46040140	46133036	0.033763446	PASS
PIK3R4	3	130678935	130746829	0.313454204	suppress_segment_too_large
PIK3R5	17	8878911	8965712	0.128406487	PASS
PIK3R6	17	8802723	8867677	0.128406487	PASS
PIKFYVE	2	208266267	208358751	0.025928102	PASS
PILRA	7	100367530	100400099	-0.262778904	PASS

PILRB	7	100352176	100367733	-0.262778904	PASS
PIM1	6	37170203	37175426	0.048820648	PASS
PIM2	23	48913182	48919024	-0.012567603	PASS
PIM3	22	49960513	49964080	-0.808211634	PASS
PIMREG	17	6444415	6451469	0.128406487	PASS
PIN1	19	9835257	9849682	0.093487731	suppress_segment_too_large
PIN4	23	72181353	72302926	-0.012567603	PASS
PINK1	1	20633455	20651511	0.033763446	PASS
PINLYP	19	43576800	43583964	0.14996833	PASS
PINX1	8	10725399	10839884	-0.345962679	PASS
PIP	7	143132077	143139746	-0.388946609	PASS
PIP4K2A	10	22534849	22714555	0.039811807	PASS
PIP4K2B	17	38765689	38800126	0.060555011	PASS
PIP4K2C	12	57591174	57603418	-0.202191512	PASS
PIP4P1	14	20457719	20461612	0.060212819	PASS
PIP4P2	8	90993796	91041064	0.279445683	PASS
PIP5K1A	1	151197949	151249536	0.103015788	PASS
PIP5K1B	9	68705414	69009176	-0.310444659	PASS
PIP5K1C	19	3630183	3700479	0.079632875	PASS
PIP5KL1	9	127920879	127930797	0.365190713	suppress_segment_too_large
PIPOX	17	28950513	29057220	0.060555011	PASS
PIR	23	15384799	15493564	-0.012567603	PASS
PIRT	17	10822475	10838445	-0.139031004	PASS
PISD	22	31618491	31662432	-0.575930959	PASS
PITHD1	1	23778405	23788232	0.033763446	PASS
PITPNA	17	1517718	1562816	0.128406487	PASS
PITPNB	22	27851669	27920134	-0.575930959	PASS
PITPNC1	17	67377281	67697261	0.088516311	PASS
PITPNM1	11	67491768	67506263	0.182439925	suppress_segment_too_large
PITPNM2	12	122983480	123150015	0.202141228	PASS
PITPNM3	17	6451264	6556494	0.128406487	PASS
PITRM1	10	3137728	3172841	0.039811807	PASS
PITX1	5	135027735	135034813	-0.293443042	PASS
PITX2	4	110617423	110642123	-0.011382299	PASS
PITX3	10	102230186	102241474	-0.367947924	PASS
PIWIL1	12	130337887	130372637	-0.240609783	PASS
PIWIL2	8	22275297	22357563	-0.345962679	PASS
PIWIL3	22	24719034	24774720	-0.575930959	PASS
PIWIL4	11	94543840	94621421	0.062969153	PASS
PJA1	23	69160851	69165793	-0.012567603	PASS
PJA2	5	109334709	109409994	-0.293443042	PASS
PJVK	2	178451346	178461409	0.039726562	PASS
PKD1	16	2088710	2135898	0.069936844	PASS
PKD1L1	7	47740202	47948491	-0.69513118	PASS
PKD1L3	16	71929538	71999978	-0.371357732	PASS
PKD1P1	16	16317444	16350590	0.069936844	PASS

PKD2	4	88007668	88077777	-0.011382299	PASS
PKD2L1	10	100288154	100330486	-0.367947924	PASS
PKD2L2	5	137887968	137942747	-0.293443042	PASS
PKDCC	2	42048020	42058528	0.040498426	PASS
PKDREJ	22	46255663	46263355	-0.808211634	PASS
PKHD1	6	51615300	52087625	0.048820648	PASS
PKHD1L1	8	109362477	109530330	0.279445683	PASS
PKIA	8	78516139	78605267	0.279445683	PASS
PKIB	6	122471917	122726373	-0.368590549	PASS
PKIG	20	44531785	44624247	-0.168668583	PASS
PKLR	1	155289293	155301438	0.103015788	PASS
PKM	15	72199029	72231822	-0.697600509	PASS
PKMYT1	16	2968024	2980539	0.069936844	PASS
PKN1	19	14433053	14471867	0.093487731	suppress_segment_too_large
PKN2	1	88684222	88836255	0.033763446	PASS
PKN3	9	128702523	128720918	0.365190713	suppress_segment_too_large
PKNOX1	21	42974510	43033931	-0.064425456	suppress_segment_too_large
PKNOX2	11	125164687	125433389	-0.419612865	PASS
PKP1	1	201283452	201332993	-0.256698369	PASS
PKP2	12	32790745	32896840	0.362961408	suppress_segment_too_large
PKP3	11	392614	404908	-0.333835828	PASS
PKP4	2	158456964	158682879	0.039726562	PASS
PLA1A	3	119597842	119629811	0.313454204	suppress_segment_too_large
PLA2G10	16	14672545	14694669	0.069936844	PASS
PLA2G12A	4	109709989	109730077	-0.011382299	PASS
PLA2G12E	10	72935170	72954778	0.072405807	PASS
PLA2G15	16	68245304	68261062	-0.371357732	PASS
PLA2G16	11	63573195	63616883	0.182439925	suppress_segment_too_large
PLA2G1B	12	120322111	120327789	0.202141228	PASS
PLA2G2A	1	19975431	19980416	0.033763446	PASS
PLA2G2C	1	20161253	20177424	0.033763446	PASS
PLA2G2D	1	20111939	20119566	0.033763446	PASS
PLA2G2E	1	19920009	19923617	0.033763446	PASS
PLA2G2F	1	20139326	20150386	0.033763446	PASS
PLA2G3	22	31134809	31140607	-0.575930959	PASS
PLA2G4A	1	186828953	186988981	-0.027073548	PASS
PLA2G4B	15	41837775	41848147	-0.396255771	PASS
PLA2G4C	19	48047843	48110817	0.14996833	PASS
PLA2G4D	15	42067009	42094554	-0.396255771	PASS
PLA2G4E	15	41981582	42051190	-0.396255771	PASS
PLA2G4F	15	42139034	42156636	-0.396255771	PASS
PLA2G5	1	20028179	20091190	0.033763446	PASS
PLA2G6	22	38111495	38205690	-0.575930959	PASS
PLA2G7	6	46704201	46735693	0.048820648	PASS
PLA2R1	2	159932006	160062610	0.039726562	PASS
PLAA	9	26904083	26947463	-0.310444659	PASS

PLAC1	23	134565838	134764322	-0.012567603	PASS
PLAC8	4	83090048	83137075	-0.011382299	PASS
PLAC8L1	5	146084386	146104369	-0.293443042	PASS
PLAC9	10	80131682	80145359	0.072405807	PASS
PLAG1	8	56160904	56211324	-0.201142258	PASS
PLAGL1	6	143940300	144064599	-0.035448111	PASS
PLAGL2	20	32192503	32207791	-0.168668583	PASS
PLAT	8	42175233	42207724	-0.201142258	PASS
PLAU	10	73909177	73917497	0.072405807	PASS
PLAUR	19	43646095	43670547	0.14996833	PASS
PLB1	2	28457145	28643788	0.040498426	PASS
PLBD1	12	14503661	14568349	0.362961408	suppress_segment_too_large
PLBD2	12	113358566	113391625	0.202141228	PASS
PLCB1	20	8077251	8968360	-0.168668583	PASS
PLCB2	15	40278176	40307935	-0.396255771	PASS
PLCB3	11	64251523	64269150	0.182439925	suppress_segment_too_large
PLCB4	20	9068763	9481242	-0.168668583	PASS
PLCD1	3	38007496	38029762	-0.234617007	PASS
PLCD3	17	45108967	45133354	0.088516311	PASS
PLCD4	2	218607765	218637184	0.025928102	PASS
PLCE1	10	94030812	94332823	-0.39678585	PASS
PLCG1	20	41136960	41196801	-0.168668583	PASS
PLCG2	16	81739097	81962693	-0.371357732	PASS
PLCH1	3	155375580	155745067	0.318278996	suppress_segment_too_large
PLCH2	1	2425980	2505530	0.373964873	suppress_segment_too_large
PLCL1	2	197804702	198572581	0.025928102	PASS
PLCL2	3	16802651	17090594	0.311018244	suppress_segment_too_large
PLCXD1	23	276322	303356	-0.012567603	PASS
PLCXD2	3	111674676	111846447	0.313454204	suppress_segment_too_large
PLCXD3	5	41306954	41510628	-0.293443042	PASS
PLCZ1	12	18683169	18738100	0.362961408	suppress_segment_too_large
PLD1	3	171600405	171810950	0.318278996	suppress_segment_too_large
PLD2	17	4807096	4823434	0.128406487	PASS
PLD3	19	40348456	40380439	0.093487731	suppress_segment_too_large
PLD4	14	104924713	104937790	-0.192197564	PASS
PLD5	1	242082986	242524696	-0.648598641	PASS
PLD6	17	17200995	17206315	0.024610076	PASS
PLEC	8	143915147	143976734	0.307788765	PASS
PLEK	2	68365173	68397453	-0.372959126	PASS
PLEK2	14	67386983	67412200	0.060212819	PASS
PLEKHA1	10	122374696	122442602	0.063874692	PASS
PLEKHA2	8	38901235	38973909	-0.201142258	PASS
PLEKHA3	2	178480468	178516462	0.039726562	PASS
PLEKHA4	19	48837097	48868632	0.14996833	PASS
PLEKHA5	12	19129752	19376400	0.362961408	suppress_segment_too_large
PLEKHA6	1	204218851	204377665	-0.256698369	PASS

PLEKHA7	11	16777317	17014443	-0.405444717	PASS
PLEKHA8	7	30027404	30130483	-0.69513118	PASS
PLEKHB1	11	73646178	73662819	0.062969153	PASS
PLEKHB2	2	131104847	131353709	-0.475215623	PASS
PLEKHD1	14	69484692	69531551	0.060212819	PASS
PLEKHF1	19	29665056	29675457	0.093487731	suppress_segment_too_large
PLEKHF2	8	95133804	95156684	0.279445683	PASS
PLEKHG1	6	150599883	150843665	-0.035448111	PASS
PLEKHG2	19	39412585	39428415	0.093487731	suppress_segment_too_large
PLEKHG3	14	64704102	64750247	0.060212819	PASS
PLEKHG4	16	67277510	67289499	-0.371357732	PASS
PLEKHG4]	5	92151	189972	0.055007813	PASS
PLEKHG5	1	6466092	6520061	0.373964873	suppress_segment_too_large
PLEKHG6	12	6310436	6328506	-0.086128028	PASS
PLEKHG7	12	92702843	92772455	0.202141228	PASS
PLEKHH1	14	67533301	67589612	0.060212819	PASS
PLEKHH2	2	43637273	43767987	0.040498426	PASS
PLEKHH3	17	42667914	42676994	0.088516311	PASS
PLEKHJ1	19	2230084	2237704	0.079632875	PASS
PLEKHM1	17	45435900	45490749	0.088516311	PASS
PLEKHM2	1	15684332	15734769	0.033763446	PASS
PLEKHM3	2	207821288	208025560	0.025928102	PASS
PLEKHN1	1	966497	975865	0.373964873	suppress_segment_too_large
PLEKHO1	1	150149183	150164720	0.103015788	PASS
PLEKHO2	15	64841883	64868007	-0.697600509	PASS
PLEKHS1	10	113751454	113783429	0.063874692	PASS
PLET1	11	112248479	112260860	-0.356188624	PASS
PLG	6	160702238	160753315	-0.035448111	PASS
PLGLB1	2	87002559	87021852	-0.340781246	PASS
PLGLB2	2	87748087	87758793	-0.340781246	PASS
PLGRKT	9	5357973	5437878	-0.310444659	PASS
PLIN1	15	89664365	89679427	-0.697600509	PASS
PLIN2	9	19108375	19149290	-0.310444659	PASS
PLIN3	19	4838341	4867768	0.079632875	PASS
PLIN4	19	4502180	4518465	0.079632875	PASS
PLIN5	19	4522531	4535224	0.079632875	PASS
PLK1	16	23677656	23690367	0.069936844	PASS
PLK2	5	58453982	58460260	-0.293443042	PASS
PLK3	1	44800225	44805990	0.033763446	PASS
PLK4	4	127880861	127899195	-0.011382299	PASS
PLK5	19	1524074	1536046	0.079632875	PASS
PLLP	16	57248547	57284687	-0.371357732	PASS
PLN	6	118548298	118560730	-0.368590549	PASS
PLOD1	1	11934205	11975538	0.373964873	suppress_segment_too_large
PLOD2	3	146069440	146163653	0.318278996	suppress_segment_too_large
PLOD3	7	101205977	101218420	-0.388946609	PASS

PLP1	23	103773718	103792619	-0.012567603	PASS
PLP2	23	49171926	49175239	-0.012567603	PASS
PLPBP	8	37762593	37779767	-0.201142258	PASS
PLPP1	5	55424854	55535050	-0.293443042	PASS
PLPP2	19	281040	291504	0.079632875	PASS
PLPP3	1	56494747	56645301	0.033763446	PASS
PLPP4	10	120456954	120589855	0.063874692	PASS
PLPP5	8	38263130	38269243	-0.201142258	PASS
PLPP6	9	4662315	4665258	-0.310444659	PASS
PLPP7	9	131289459	131359022	0.365190713	suppress_segment_too_large
PLPPR1	9	101028709	101325135	0.365190713	suppress_segment_too_large
PLPPR2	19	11355386	11365698	0.093487731	suppress_segment_too_large
PLPPR3	19	812488	821977	0.079632875	PASS
PLPPR4	1	99263953	99309590	0.033763446	PASS
PLPPR5	1	98890245	99005032	0.033763446	PASS
PLRG1	4	154535006	154550435	-0.011382299	PASS
PLS1	3	142596387	142713664	0.318278996	suppress_segment_too_large
PLS3	23	115561174	115650861	-0.012567603	PASS
PLSCR1	3	146515180	146544864	0.318278996	suppress_segment_too_large
PLSCR2	3	146391421	146495991	0.318278996	suppress_segment_too_large
PLSCR3	17	7389727	7394843	0.128406487	PASS
PLSCR4	3	146192339	146251179	0.318278996	suppress_segment_too_large
PLSCR5	3	146576555	146606216	0.318278996	suppress_segment_too_large
PLTP	20	45898621	45912155	-0.168668583	PASS
PLVAP	19	17351448	17377350	0.093487731	suppress_segment_too_large
PLXDC1	17	39063303	39154394	0.060555011	PASS
PLXDC2	10	19816239	20289856	0.039811807	PASS
PLXNA1	3	126988594	127037392	0.313454204	suppress_segment_too_large
PLXNA2	1	208022242	208244320	-0.256698369	PASS
PLXNA3	23	154458281	154477779	-0.012567603	PASS
PLXNA4	7	132123332	132648688	-0.388946609	PASS
PLXNB1	3	48403854	48430051	-0.234617007	PASS
PLXNB2	22	50274979	50307627	-0.808211634	PASS
PLXNB3	23	153764196	153779346	-0.012567603	PASS
PLXNC1	12	94148723	94307675	0.202141228	PASS
PLXND1	3	129555175	129606818	0.313454204	suppress_segment_too_large
PM20D1	1	205828022	205850132	-0.256698369	PASS
PM20D2	6	89146050	89165565	-0.368590549	PASS
PMAIP1	18	59899948	59904306	-0.376397843	PASS
PMCH	12	102196459	102197845	0.202141228	PASS
PMEL	12	55954105	55973317	-0.202191512	PASS
PMEPA1	20	57648392	57711536	-0.168668583	PASS
PMF1	1	156212993	156240042	0.103015788	PASS
PMF1-BGI	1	156212982	156243332	0.103015788	PASS
PMFBP1	16	72112157	72176878	-0.371357732	PASS
PML	15	73994673	74047812	-0.697600509	PASS

PMM1	22	41576894	41589890		-0.575930959	PASS
PMM2	16	8788823	8849331		0.069936844	PASS
PMP2	8	81440326	81447523		0.279445683	PASS
PMP22	17	15229777	15265357		-0.139031004	PASS
PMPCA	9	136410570	136423761		0.365190713	suppress_segment_too_large
PMPCB	7	103297422	103329511		-0.388946609	PASS
PMS1	2	189784085	189877629		0.025928102	PASS
PMS2	7	5973239	6009125		-0.69513118	PASS
PMVK	1	154924734	154936991		0.103015788	PASS
PNCK	23	153669730	153689010		-0.012567603	PASS
PNISR	6	99398051	99425331		-0.368590549	PASS
PNKD	2	218270392	218346793		0.025928102	PASS
PNKP	19	49859882	49878351		0.14996833	PASS
PNLDC1	6	159800249	159820704		-0.035448111	PASS
PNLIP	10	116545931	116567855		0.063874692	PASS
PNLIPRP1	10	116590385	116609175		0.063874692	PASS
PNLIPRP3	10	116427867	116477957		0.063874692	PASS
PNMA1	14	73711783	73714372		0.060212819	PASS
PNMA2	8	26504686	26514092		-0.345962679	PASS
PNMA3	23	153056409	153060467		-0.012567603	PASS
PNMA5	23	152988824	152994127		-0.012567603	PASS
PNMA6A	23	153072482	153075018		-0.012567603	PASS
PNMA6E	23	153395640	153401420		-0.012567603	PASS
PNMA6F	23	153317681	153321822		-0.012567603	PASS
PNMA8A	19	46466491	46471563		0.14996833	PASS
PNMA8B	19	46486906	46496498		0.14996833	PASS
PNMT	17	39667981	39670475		0.060555011	PASS
PNN	14	39175183	39183218		0.060212819	PASS
PNO1	2	68157844	68176238		-0.372959126	PASS
PNOC	8	28316986	28343355		-0.345962679	PASS
PNP	14	20468954	20477094		0.060212819	PASS
PNPLA1	6	36243203	36312229		0.048820648	PASS
PNPLA2	11	818902	825573		-0.333835828	PASS
PNPLA3	22	43923739	43964488		-0.808211634	PASS
PNPLA4	23	7898247	7927739		-0.012567603	PASS
PNPLA5	22	43879678	43892013		-0.808211634	PASS
PNPLA6	19	7534004	7561764		0.079632875	PASS
PNPLA7	9	137459953	137550534		0.365190713	suppress_segment_too_large
PNPLA8	7	108470422	108569666		-0.388946609	PASS
PNPO	17	47941506	47949308		0.088516311	PASS
PNPT1	2	55634265	55693910		0.040498426	PASS
PNRC1	6	89080751	89085160		-0.368590549	PASS
PNRC2	1	23956839	23963462		0.033763446	PASS
POC1A	3	52075253	52154690		-0.234617007	PASS
POC1B	12	89419718	89526024		0.202141228	PASS
POC1B-GA	12	89519408	89526262		0.202141228	PASS

POC5	5	75674124	75717481	-0.293443042	PASS
PODN	1	53062052	53085502	0.033763446	PASS
PODNL1	19	13931187	13953392	0.093487731	suppress_segment_too_large
PODXL	7	131500262	131558217	-0.388946609	PASS
PODXL2	3	127629181	127672809	0.313454204	suppress_segment_too_large
POF1B	23	85277396	85379743	-0.012567603	PASS
POFUT1	20	32207880	32238667	-0.168668583	PASS
POFUT2	21	45263928	45287898	-0.064425456	suppress_segment_too_large
POGK	1	166839447	166856344	-0.027073548	PASS
POGLUT1	3	119468938	119494708	0.313454204	suppress_segment_too_large
POGZ	1	151402724	151459465	0.103015788	PASS
POLA1	23	24693919	24996986	-0.012567603	PASS
POLA2	11	65261762	65305589	0.182439925	suppress_segment_too_large
POLB	8	42338454	42371808	-0.201142258	PASS
POLD1	19	50384204	50418016	-0.016761391	PASS
POLD2	7	44114681	44124358	-0.69513118	PASS
POLD3	11	74493851	74669117	0.062969153	PASS
POLD4	11	67350777	67356972	0.182439925	suppress_segment_too_large
POLDIP2	17	28347177	28357522	0.060555011	PASS
POLDIP3	22	42583721	42614962	-0.575930959	PASS
POLE	12	132623753	132687365	0.270848837	PASS
POLE2	14	49643555	49688422	0.060212819	PASS
POLE3	9	113407235	113410672	0.365190713	suppress_segment_too_large
POLE4	2	74958492	74970128	-0.372959126	PASS
POLG	15	89305198	89334861	-0.697600509	PASS
POLG2	17	64477785	64497036	0.088516311	PASS
POLH	6	43576150	43615660	0.048820648	PASS
POLI	18	54269404	54321266	-0.376397843	PASS
POLK	5	75511756	75601144	-0.293443042	PASS
POLL	10	101578882	101588270	-0.367947924	PASS
POLM	7	44072247	44082540	-0.69513118	PASS
POLN	4	2071918	2242121	0.279089364	PASS
POLQ	3	121431427	121546641	0.313454204	suppress_segment_too_large
POLR1A	2	86020216	86106155	0.066915641	PASS
POLR1B	2	112541915	112577150	-0.010901494	PASS
POLR1C	6	43509702	43562419	0.048820648	PASS
POLR1D	13	27620742	27744237	-0.390786075	PASS
POLR1E	9	37485935	37503697	-0.310444659	PASS
POLR2A	17	7484366	7514616	0.128406487	PASS
POLR2B	4	56977722	57031168	-0.011382299	PASS
POLR2C	16	57462387	57472010	-0.371357732	PASS
POLR2D	2	127843551	127858157	-0.475215623	PASS
POLR2E	19	1086579	1095380	0.079632875	PASS
POLR2F	22	37952607	38041915	-0.575930959	PASS
POLR2G	11	62761544	62766710	0.182439925	suppress_segment_too_large
POLR2H	3	184361718	184368596	0.318278996	suppress_segment_too_large

POLR2I	19	36113710	36115346	0.093487731	suppress_segment_too_large
POLR2J	7	102473118	102478907	-0.388946609	PASS
POLR2J2	7	102665368	102671629	-0.388946609	PASS
POLR2J3	7	102537918	102572653	-0.388946609	PASS
POLR2K	8	100150584	100154002	0.279445683	PASS
POLR2L	11	837356	842545	-0.333835828	PASS
POLR2M	15	57706629	57782762	-0.697600509	PASS
POLR3A	10	77969251	78029545	0.072405807	PASS
POLR3B	12	106357658	106510198	0.202141228	PASS
POLR3C	1	145824088	145842505	-0.157385092	PASS
POLR3D	8	22245104	22254600	-0.345962679	PASS
POLR3E	16	22297375	22335103	0.069936844	PASS
POLR3F	20	18467127	18484643	-0.168668583	PASS
POLR3G	5	90471748	90514553	-0.293443042	PASS
POLR3GL	1	145964702	145978848	-0.157385092	PASS
POLR3H	22	41525804	41544606	-0.575930959	PASS
POLR3K	16	46407	53628	0.069936844	PASS
POLRMT	19	617224	633604	0.079632875	PASS
POM121	7	72879365	72951440	-0.262778904	PASS
POM121C	7	75416787	75486271	-0.262778904	PASS
POM121L1	7	53035642	53036924	-0.750061359	PASS
POM121L2	6	27285903	27312170	0.048820648	PASS
POMC	2	25160853	25168903	0.040498426	PASS
POMGNT1	1	46188682	46220305	0.033763446	PASS
POMGNT2	3	43079232	43106076	-0.234617007	PASS
POMK	8	43093506	43123434	-0.201142258	PASS
POMP	13	28659104	28678925	-0.390786075	PASS
POMT1	9	131502902	131523806	0.365190713	suppress_segment_too_large
POMT2	14	77274956	77320884	-0.192197564	PASS
POMZP3	7	76609986	76627261	-0.262778904	PASS
PON1	7	95297676	95324707	-0.262778904	PASS
PON2	7	95404863	95435329	-0.262778904	PASS
PON3	7	95359944	95396368	-0.262778904	PASS
POP1	8	98117297	98159834	0.279445683	PASS
POP4	19	29604017	29617237	0.093487731	suppress_segment_too_large
POP5	12	120578764	120581398	0.202141228	PASS
POP7	7	100706053	100707495	-0.262778904	PASS
POPDC2	3	119636457	119665324	0.313454204	suppress_segment_too_large
POPDC3	6	105158280	105179995	-0.368590549	PASS
POR	7	75899200	75986855	-0.262778904	PASS
PORCN	23	48508962	48520814	-0.012567603	PASS
POSTN	13	37562583	37598844	-0.390786075	PASS
POT1	7	124822386	124929983	-0.388946609	PASS
POTEB3	15	21408243	21440451	-0.750600437	PASS
POTEC	18	14507339	14543585	-0.052305056	suppress_segment_too_large
POTED	21	13609858	13641585	-0.304975024	PASS

POTEE	2	131218067	131265278	-0.475215623	PASS
POTEF	2	130074030	130129222	-0.475215623	PASS
POTEG	14	19402486	19434341	-0.238381381	PASS
POTEH	22	15690026	15721631	-0.575930959	PASS
POTEI	2	130459455	130509666	-0.475215623	PASS
POTEJ	2	130611481	130658091	-0.475215623	PASS
POTEM	14	18967434	18999012	-0.238381381	PASS
POU1F1	3	87259404	87276587	0.313454204	suppress_segment_too_large
POU2AF1	11	111352252	111455630	-0.356188624	PASS
POU2F1	1	167220829	167427345	-0.027073548	PASS
POU2F2	19	42086110	42196585	0.093487731	suppress_segment_too_large
POU2F3	11	120236640	120319944	-0.356188624	PASS
POU3F1	1	38044611	38046794	0.033763446	PASS
POU3F2	6	98834592	98839470	-0.368590549	PASS
POU3F3	2	104855511	104858574	-0.532032081	PASS
POU3F4	23	83508261	83512127	-0.012567603	PASS
POU4F1	13	78598362	78603560	-0.390786075	PASS
POU4F2	4	146638893	146642474	-0.011382299	PASS
POU4F3	5	146338839	146341722	-0.293443042	PASS
POU5F1	6	31164337	31180731	0.048820648	PASS
POU5F1B	8	127322183	127420066	0.113969953	PASS
POU5F2	5	93733220	93741637	-0.293443042	PASS
POU6F1	12	51186936	51217708	-0.202191512	PASS
POU6F2	7	38977998	39493095	-0.69513118	PASS
PP2D1	3	19979961	20012330	0.311018244	suppress_segment_too_large
PPA1	10	70202830	70233911	0.072405807	PASS
PPA2	4	105369077	105474081	-0.011382299	PASS
PPAN	19	10106289	10111634	0.093487731	suppress_segment_too_large
PPAN-P2R	19	10106223	10114780	0.093487731	suppress_segment_too_large
PPARA	22	46150521	46243756	-0.808211634	PASS
PPARD	6	35342558	35428191	0.048820648	PASS
PPARG	3	12287368	12434356	0.311018244	suppress_segment_too_large
PPARGC1	4	23755041	23904089	0.279089364	PASS
PPARGC1	5	149730298	149855022	-0.293443042	PASS
PPAT	4	56393362	56435615	-0.011382299	PASS
PPBP	4	73987038	73988197	-0.011382299	PASS
PPCDC	15	75023555	75117462	-0.697600509	PASS
PPCS	1	42456117	42473385	0.033763446	PASS
PPDPF	20	63520724	63522206	-0.168668583	PASS
PPDPFL	8	49054311	49076090	-0.201142258	PASS
PPEF1	23	18675909	18827921	-0.012567603	PASS
PPEF2	4	75859864	75902571	-0.011382299	PASS
PPFIA1	11	70270700	70385312	0.182439925	suppress_segment_too_large
PPFIA2	12	81257975	81759553	0.202141228	PASS
PPFIA3	19	49119389	49151026	0.14996833	PASS
PPFIA4	1	203026498	203078740	-0.256698369	PASS

PPFIBP1	12	27523431	27695564	0.362961408	suppress_segment_too_large
PPFIBP2	11	7513298	7657127	-0.405444717	PASS
PPHLN1	12	42238447	42459715	-0.202191512	PASS
PPIA	7	44796680	44824564	-0.69513118	PASS
PPIAL4A	1	120889746	120890405	-0.157385092	PASS
PPIAL4C	1	149583865	149584464	-0.157385092	PASS
PPIAL4D	1	145241630	145242124	-0.157385092	PASS
PPIAL4G	1	148479824	148483679	-0.157385092	PASS
PPIB	15	64155812	64163205	-0.697600509	PASS
PPIC	5	123023250	123036741	-0.293443042	PASS
PPID	4	158709134	158723396	-0.011382299	PASS
PPIE	1	39692182	39763914	0.033763446	PASS
PPIF	10	79347469	79355337	0.072405807	PASS
PPIG	2	169584340	169641406	0.039726562	PASS
PPIH	1	42658425	42676758	0.033763446	PASS
PPIL1	6	36854827	36875024	0.048820648	PASS
PPIL2	22	21666009	21700015	-0.575930959	PASS
PPIL3	2	200870907	200889303	0.025928102	PASS
PPIL4	6	149504733	149546038	-0.035448111	PASS
PPIL6	6	109390215	109441171	-0.368590549	PASS
PPIP5K1	15	43533462	43590253	-0.396255771	PASS
PPIP5K2	5	103120149	103212799	-0.293443042	PASS
PPL	16	4882507	4960741	0.069936844	PASS
PPM1A	14	60245752	60299087	0.060212819	PASS
PPM1B	2	44167969	44244384	0.040498426	PASS
PPM1D	17	60600183	60666280	0.088516311	PASS
PPM1E	17	58755869	58985176	0.088516311	PASS
PPM1F	22	21919420	21952837	-0.575930959	PASS
PPM1G	2	27381194	27409687	0.040498426	PASS
PPM1H	12	62643982	62935037	-0.202191512	PASS
PPM1J	1	112709994	112715477	0.033763446	PASS
PPM1K	4	88257620	88284769	-0.011382299	PASS
PPM1L	3	160755602	161078907	0.318278996	suppress_segment_too_large
PPM1M	3	52245793	52250597	-0.234617007	PASS
PPM1N	19	45488777	45502510	0.14996833	PASS
PPME1	11	74171099	74254703	0.062969153	PASS
PPOX	1	161166410	161178013	-0.027073548	PASS
PPP1CA	11	67398183	67421183	0.182439925	suppress_segment_too_large
PPP1CB	2	28751640	28802940	0.040498426	PASS
PPP1CC	12	110719680	110742939	0.202141228	PASS
PPP1R10	6	30600400	30618612	0.048820648	PASS
PPP1R11	6	30066709	30070333	0.048820648	PASS
PPP1R12A	12	79773563	79935460	0.202141228	PASS
PPP1R12B	1	202348699	202592706	-0.256698369	PASS
PPP1R12C	19	55090913	55117559	0.121336758	PASS
PPP1R13B	14	103733195	103847590	-0.192197564	PASS

PPP1R13L	19	45379634	45406349	0.14996833	PASS
PPP1R14A	19	38251237	38256591	0.093487731	suppress_segment_too_large
PPP1R14B	11	64244480	64246941	0.182439925	suppress_segment_too_large
PPP1R14C	6	150143076	150250357	-0.035448111	PASS
PPP1R14D	15	40815445	40828709	-0.396255771	PASS
PPP1R15A	19	48872392	48876057	0.14996833	PASS
PPP1R15B	1	204403387	204411791	-0.256698369	PASS
PPP1R16A	8	144477969	144502121	0.307788765	PASS
PPP1R16B	20	38805705	38923024	-0.168668583	PASS
PPP1R17	7	31686715	31708455	-0.69513118	PASS
PPP1R18	6	30676389	30687895	0.048820648	PASS
PPP1R1A	12	54575387	54588659	-0.202191512	PASS
PPP1R1B	17	39626740	39636626	0.060555011	PASS
PPP1R1C	2	181954241	182131398	0.039726562	PASS
PPP1R2	3	195514425	195543386	0.326834516	PASS
PPP1R21	2	48440598	48515391	0.040498426	PASS
PPP1R26	9	135479079	135488893	0.365190713	suppress_segment_too_large
PPP1R27	17	81833492	81835050	0.088516311	PASS
PPP1R2B	5	156850538	156852528	-0.293443042	PASS
PPP1R32	11	61481120	61490931	0.182439925	suppress_segment_too_large
PPP1R35	7	100435282	100436565	-0.262778904	PASS
PPP1R36	14	64549902	64589380	0.060212819	PASS
PPP1R37	19	45091396	45148077	0.14996833	PASS
PPP1R3A	7	113876777	114075920	-0.388946609	PASS
PPP1R3B	8	9136255	9151574	-0.345962679	PASS
PPP1R3C	10	91628442	91633054	-0.39678585	PASS
PPP1R3D	20	59936668	59940297	-0.168668583	PASS
PPP1R3E	14	23295643	23302848	0.060212819	PASS
PPP1R3F	23	49269843	49301461	-0.012567603	PASS
PPP1R3G	6	5084581	5089487	0.048820648	PASS
PPP1R42	8	66964099	67056604	-0.201142258	PASS
PPP1R7	2	241149576	241183652	0.025928102	PASS
PPP1R8	1	27830778	27851676	0.033763446	PASS
PPP1R9A	7	94907202	95296415	-0.262778904	PASS
PPP1R9B	17	50133735	50150630	0.088516311	PASS
PPP2CA	5	134194334	134226142	-0.293443042	PASS
PPP2CB	8	30774457	30814314	-0.345962679	PASS
PPP2R1A	19	52190039	52229533	0.206912349	PASS
PPP2R1B	11	111726908	111766427	-0.356188624	PASS
PPP2R2A	8	26291491	26372680	-0.345962679	PASS
PPP2R2B	5	146581146	147084784	-0.293443042	PASS
PPP2R2C	4	6320578	6563600	0.279089364	PASS
PPP2R2D	10	131900644	131959834	0.063874692	PASS
PPP2R3A	3	135965673	136147891	0.313454204	suppress_segment_too_large
PPP2R3B	23	333963	386955	-0.012567603	PASS
PPP2R3C	14	35085467	35122517	0.060212819	PASS

PPP2R5A	1	212285537	212361863	-0.256698369	PASS
PPP2R5B	11	64917553	64934473	0.182439925	suppress_segment_too_large
PPP2R5C	14	101761798	101927989	-0.192197564	PASS
PPP2R5D	6	42984499	43012342	0.048820648	PASS
PPP2R5E	14	63371357	63543374	0.060212819	PASS
PPP3CA	4	101023409	101348278	-0.011382299	PASS
PPP3CB	10	73436428	73496024	0.072405807	PASS
PPP3CC	8	22440819	22541142	-0.345962679	PASS
PPP3R1	2	68178857	68256237	-0.372959126	PASS
PPP3R2	9	101591615	101595021	0.365190713	suppress_segment_too_large
PPP4C	16	30075978	30085377	0.069936844	PASS
PPP4R1	18	9546791	9615240	-0.052305056	suppress_segment_too_large
PPP4R2	3	72996785	73069199	-0.234617007	PASS
PPP4R3A	14	91457611	91510554	-0.192197564	PASS
PPP4R3B	2	55547292	55618880	0.040498426	PASS
PPP4R4	14	94146128	94279735	-0.192197564	PASS
PPP5C	19	46346994	46392981	0.14996833	PASS
PPP5D1	19	46480796	46601200	0.14996833	PASS
PPP6C	9	125146573	125189939	0.365190713	suppress_segment_too_large
PPP6R1	19	55229780	55258995	0.121336758	PASS
PPP6R2	22	50343304	50445090	-0.808211634	PASS
PPP6R3	11	68460731	68615334	0.182439925	suppress_segment_too_large
PPRC1	10	102132994	102150331	-0.367947924	PASS
PPT1	1	40071461	40097727	0.033763446	PASS
PPT2	6	32153441	32163680	0.048820648	PASS
PPT2-EGFl	6	32153845	32171978	0.048820648	PASS
PPTC7	12	110533245	110583320	0.202141228	PASS
PPWD1	5	65563236	65587549	-0.293443042	PASS
PPY	17	43940804	43942468	0.088516311	PASS
PQBP1	23	48897912	48903143	-0.012567603	PASS
PQLC1	18	79902420	79951664	-0.376397843	PASS
PQLC2	1	19312326	19329300	0.033763446	PASS
PQLC2L	3	157543246	157677749	0.318278996	suppress_segment_too_large
PQLC3	2	11155198	11178874	0.040498426	PASS
PRAC1	17	48721719	48722522	0.088516311	PASS
PRAC2	17	48723168	48724758	0.088516311	PASS
PRADC1	2	73228006	73233238	-0.372959126	PASS
PRAF2	23	49071156	49074071	-0.012567603	PASS
PRAG1	8	8317736	8386498	-0.345962679	PASS
PRAM1	19	8490056	8503112	0.079632875	PASS
PRAME	22	22547701	22559361	-0.575930959	PASS
PRAMEF1	1	12791397	12796628	-0.415253068	PASS
PRAMEF1	1	12892896	12898270	-0.415253068	PASS
PRAMEF1	1	12824605	12831410	-0.415253068	PASS
PRAMEF1	1	12774841	12777906	-0.415253068	PASS
PRAMEF1	1	13196330	13201409	-0.415253068	PASS

PRAMEF1	1	13342034	13347134	-0.415253068	PASS
PRAMEF1	1	13315581	13322598	-0.415253068	PASS
PRAMEF1	1	13389632	13392629	-0.415253068	PASS
PRAMEF1	1	13222705	13226106	-0.415253068	PASS
PRAMEF1	1	13369067	13371900	-0.415253068	PASS
PRAMEF2	1	12857086	12861909	-0.415253068	PASS
PRAMEF2	1	13410450	13421328	-0.415253068	PASS
PRAMEF2	1	13148905	13155961	-0.415253068	PASS
PRAMEF2	1	13049476	13056491	-0.415253068	PASS
PRAMEF3	1	13303539	13308907	-0.415253068	PASS
PRAMEF4	1	12879224	12886201	-0.415253068	PASS
PRAMEF5	1	13254212	13263314	-0.415253068	PASS
PRAMEF6	1	12938472	12947580	-0.415253068	PASS
PRAMEF7	1	12916610	12920482	-0.415253068	PASS
PRAMEF8	1	13281035	13285174	-0.415253068	PASS
PRAMEF9	1	13172455	13179464	-0.415253068	PASS
PRAP1	10	133347146	133352683	0.063874692	PASS
PRB1	12	11351823	11395566	0.362961408	suppress_segment_too_large
PRB2	12	11391540	11501041	0.362961408	suppress_segment_too_large
PRB3	12	11265924	11269805	0.362961408	suppress_segment_too_large
PRB4	12	11307083	11310435	0.362961408	suppress_segment_too_large
PRC1	15	90966038	90995629	-0.697600509	PASS
PRCC	1	156750610	156800817	0.103015788	PASS
PRCD	17	76527586	76553578	0.088516311	PASS
PRCP	11	82823502	82970584	0.062969153	PASS
PRDM1	6	106086320	106109939	-0.368590549	PASS
PRDM10	11	129899706	130002835	-0.419612865	PASS
PRDM11	11	45095806	45235110	0.117376172	PASS
PRDM12	9	130664594	130682981	0.365190713	suppress_segment_too_large
PRDM13	6	99606730	99615578	-0.368590549	PASS
PRDM14	8	70051651	70071693	-0.201142258	PASS
PRDM15	21	41798225	41879482	-0.064425456	suppress_segment_too_large
PRDM16	1	3069168	3438621	0.373964873	suppress_segment_too_large
PRDM2	1	13700198	13825079	0.033763446	PASS
PRDM4	12	107732866	107761272	0.202141228	PASS
PRDM5	4	120684919	120922870	-0.011382299	PASS
PRDM6	5	123089121	123194266	-0.293443042	PASS
PRDM7	16	90056566	90092072	-0.371357732	PASS
PRDM8	4	80183879	80204329	-0.011382299	PASS
PRDM9	5	23443586	23528597	0.055007813	PASS
PRDX1	1	45511036	45523047	0.033763446	PASS
PRDX2	19	12796820	12801859	0.093487731	suppress_segment_too_large
PRDX3	10	119167703	119178833	0.063874692	PASS
PRDX4	23	23664262	23686399	-0.012567603	PASS
PRDX5	11	64318088	64321811	0.182439925	suppress_segment_too_large
PRDX6	1	173477266	173488807	-0.027073548	PASS

PREB	2	27130756	27134675	0.040498426	PASS
PRELID1	5	177303774	177306959	-0.150979337	PASS
PRELID2	5	145471799	145835369	-0.293443042	PASS
PRELID3A	18	12407896	12432238	-0.052305056	suppress_segment_too_large
PRELID3B	20	59033145	59042909	-0.168668583	PASS
PRELP	1	203475828	203491352	-0.256698369	PASS
PREP	6	105277565	105403084	-0.368590549	PASS
PREPL	2	44316281	44361862	0.040498426	PASS
PREX1	20	48624252	48827883	-0.168668583	PASS
PREX2	8	67952118	68237030	-0.201142258	PASS
PRF1	10	70597348	70602775	0.072405807	PASS
PRG2	11	57386794	57390657	-0.129266491	PASS
PRG3	11	57376769	57381150	-0.129266491	PASS
PRG4	1	186296279	186314562	-0.027073548	PASS
PRH1	12	10824960	11171608	0.362961408	suppress_segment_too_large
PRH1-PRR	12	10845849	11171600	0.362961408	suppress_segment_too_large
PRH2	12	10929236	10932341	0.362961408	suppress_segment_too_large
PRICKLE1	12	42456757	42590355	-0.202191512	PASS
PRICKLE2	3	64092242	64445476	-0.234617007	PASS
PRICKLE3	23	49175264	49186528	-0.012567603	PASS
PRICKLE4	6	41780762	41787372	0.048820648	PASS
PRIM1	12	56731596	56752373	-0.202191512	PASS
PRIM2	6	57314805	57646849	0.048820648	PASS
PRIMA1	14	93718298	93788481	-0.192197564	PASS
PRIMPOL	4	184649613	184694963	-0.462744716	PASS
PRKAA1	5	40759379	40798374	-0.293443042	PASS
PRKAA2	1	56645322	56715335	0.033763446	PASS
PRKAB1	12	119667753	119681630	0.202141228	PASS
PRKAB2	1	147155106	147172550	-0.157385092	PASS
PRKACA	19	14091688	14118084	0.093487731	suppress_segment_too_large
PRKACB	1	84078062	84238498	0.033763446	PASS
PRKACG	9	69012529	69014113	-0.310444659	PASS
PRKAG1	12	49002274	49018825	-0.202191512	PASS
PRKAG2	7	151556111	151877125	-0.388946609	PASS
PRKAG3	2	218822383	218832086	0.025928102	PASS
PRKAR1A	17	68511780	68551319	0.088516311	PASS
PRKAR1B	7	549197	727650	-0.69513118	PASS
PRKAR2A	3	48744597	48847846	-0.234617007	PASS
PRKAR2B	7	107044649	107161811	-0.388946609	PASS
PRKCA	17	66302636	66810743	0.088516311	PASS
PRKCB	16	23835946	24220611	0.069936844	PASS
PRKCD	3	53156009	53192717	-0.234617007	PASS
PRKCE	2	45651345	46187990	0.040498426	PASS
PRKCG	19	53879190	53907652	0.206912349	PASS
PRKCH	14	61187559	61550976	0.060212819	PASS
PRKCI	3	170222365	170305981	0.318278996	suppress_segment_too_large

PRKCQ	10	6427143	6580301	0.039811807	PASS
PRKCSH	19	11435288	11450968	0.093487731	suppress_segment_too_large
PRKCZ	1	2050470	2185395	0.373964873	suppress_segment_too_large
PRKD1	14	29576479	30191898	0.060212819	PASS
PRKD2	19	46674275	46717127	0.14996833	PASS
PRKD3	2	37250502	37324808	0.040498426	PASS
PRKDC	8	47773108	47960183	-0.201142258	PASS
PRKG1	10	50990891	52298350	-0.404852538	PASS
PRKG2	4	81087370	81215117	-0.011382299	PASS
PRKN	6	161347420	162727771	-0.035448111	PASS
PRKRA	2	178431414	178451512	0.039726562	PASS
PRKRIP1	7	102363872	102426676	-0.388946609	PASS
PRKX	23	3604370	3713608	-0.012567603	PASS
PRL	6	22287244	22297501	0.048820648	PASS
PRLH	2	237566574	237567175	0.025928102	PASS
PRLHR	10	118589989	118595699	0.063874692	PASS
PRLR	5	35048756	35230589	0.055007813	PASS
PRM1	16	11280836	11281350	0.069936844	PASS
PRM2	16	11275639	11276480	0.069936844	PASS
PRM3	16	11273218	11273641	0.069936844	PASS
PRMT1	19	49675786	49689029	0.14996833	PASS
PRMT2	21	46635167	46665124	-0.064425456	suppress_segment_too_large
PRMT3	11	20387530	20509294	-0.405444717	PASS
PRMT5	14	22920511	22929585	0.060212819	PASS
PRMT6	1	107056679	107067636	0.033763446	PASS
PRMT7	16	68310974	68358563	-0.371357732	PASS
PRMT8	12	3381349	3593973	-0.086128028	PASS
PRMT9	4	147637785	147684230	-0.011382299	PASS
PRND	20	4721910	4728460	-0.168668583	PASS
PRNP	20	4686236	4701590	-0.168668583	PASS
PROB1	5	139390592	139395713	-0.293443042	PASS
PROC	2	127418427	127429246	-0.475215623	PASS
PROCA1	17	28703197	28711854	0.060555011	PASS
PROCR	20	35172073	35216240	-0.168668583	PASS
PRODH2	19	35799988	35813299	0.093487731	suppress_segment_too_large
PROK1	1	110451200	110457354	0.033763446	PASS
PROK2	3	71771656	71785206	-0.234617007	PASS
PROKR1	2	68643589	68658247	-0.372959126	PASS
PROKR2	20	5302040	5314369	-0.168668583	PASS
PROM1	4	15963076	16084378	0.279089364	PASS
PROM2	2	95274453	95291308	0.048238121	PASS
PROP1	5	177992235	177996242	-0.150979337	PASS
PROS1	3	93873033	93980003	0.313454204	suppress_segment_too_large
PROSER1	13	39009866	39038076	-0.390786075	PASS
PROSER2	10	11823339	11872277	0.039811807	PASS
PROSER3	19	35758143	35771028	0.093487731	suppress_segment_too_large

PROX1	1	213983181	214041502	-0.256698369	PASS
PROX2	14	74852871	74871940	-0.192197564	PASS
PROZ	13	113158654	113172383	-0.390786075	PASS
PRPF18	10	13586927	13630868	0.039811807	PASS
PRPF19	11	60890730	60906588	0.182439925	suppress_segment_too_large
PRPF3	1	150321476	150353195	0.103015788	PASS
PRPF31	19	54115410	54131719	0.206912349	PASS
PRPF38A	1	52404564	52420839	0.033763446	PASS
PRPF38B	1	108692323	108701803	0.033763446	PASS
PRPF39	14	45084099	45116282	0.060212819	PASS
PRPF4	9	113275343	113292905	0.365190713	suppress_segment_too_large
PRPF40A	2	152651593	152717997	-0.475215623	PASS
PRPF40B	12	49568218	49644666	-0.202191512	PASS
PRPF4B	6	4021267	4064983	0.048820648	PASS
PRPF6	20	63981135	64033100	-0.168668583	PASS
PRPF8	17	1650629	1684882	0.128406487	PASS
PRPH	12	49293252	49298686	-0.202191512	PASS
PRPH2	6	42696600	42722574	0.048820648	PASS
PRPS1	23	107628424	107651026	-0.012567603	PASS
PRPS1L1	7	18026774	18027863	-0.69513118	PASS
PRPS2	23	12791355	12824222	-0.012567603	PASS
PRPSAP1	17	76309486	76384521	0.088516311	PASS
PRPSAP2	17	18840085	18931287	0.024610076	PASS
PRR11	17	59155499	59204705	0.088516311	PASS
PRR12	19	49591643	49626439	0.14996833	PASS
PRR13	12	53441605	53446645	-0.202191512	PASS
PRR14	16	30650717	30656440	0.069936844	PASS
PRR14L	22	31676256	31750140	-0.575930959	PASS
PRR15	7	29563811	29567295	-0.69513118	PASS
PRR15L	17	47951967	47957878	0.088516311	PASS
PRR16	5	120464278	120687332	-0.293443042	PASS
PRR18	6	166305300	166308448	-0.035448111	PASS
PRR19	19	42302098	42310821	0.093487731	suppress_segment_too_large
PRR22	19	5782960	5784765	0.079632875	PASS
PRR23A	3	139005468	139006268	0.313454204	suppress_segment_too_large
PRR23B	3	139019031	139020926	0.313454204	suppress_segment_too_large
PRR23C	3	139042102	139044892	0.313454204	suppress_segment_too_large
PRR23D1	8	7539628	7542450	-0.345962679	PASS
PRR26	10	649948	669581	0.039811807	PASS
PRR27	4	70133616	70176799	-0.011382299	PASS
PRR29	17	63998351	64004304	0.088516311	PASS
PRR3	6	30556886	30563723	0.048820648	PASS
PRR30	2	27136848	27139410	0.040498426	PASS
PRR32	23	126819764	126821786	-0.012567603	PASS
PRR33	11	1888577	1891895	-0.333835828	PASS
PRR34	22	46049478	46054144	-0.808211634	PASS

PRR35	16	560422	565528	0.069936844	PASS
PRR36	19	7868719	7874379	0.079632875	PASS
PRR4	12	10845849	10849475	0.362961408	suppress_segment_too_large
PRR5	22	44668547	44737681	-0.808211634	PASS
PRR5-ARH	22	44702233	44862706	-0.808211634	PASS
PRR5L	11	36296288	36465204	-0.405444717	PASS
PRR7	5	177446445	177456286	-0.150979337	PASS
PRR9	1	153217584	153219317	0.103015788	PASS
PRRC1	5	127517609	127555089	-0.293443042	PASS
PRRC2A	6	31620720	31637771	0.048820648	PASS
PRRC2B	9	131373636	131500197	0.365190713	suppress_segment_too_large
PRRC2C	1	171485551	171593511	-0.027073548	PASS
PRRG1	23	37349275	37457295	-0.012567603	PASS
PRRG2	19	49580646	49591015	0.14996833	PASS
PRRG3	23	151695124	151705924	-0.012567603	PASS
PRRG4	11	32829943	32858123	-0.405444717	PASS
PRRT1	6	32148359	32153083	0.048820648	PASS
PRRT2	16	29811382	29815892	0.069936844	PASS
PRRT3	3	9945542	9952394	0.311018244	suppress_segment_too_large
PRRT4	7	128350325	128361685	-0.388946609	PASS
PRRX1	1	170662728	170739419	-0.027073548	PASS
PRRX2	9	129665641	129722674	0.365190713	suppress_segment_too_large
PRSS1	7	142749468	142753076	-0.388946609	PASS
PRSS12	4	118280038	118353003	-0.011382299	PASS
PRSS16	6	27247701	27256624	0.048820648	PASS
PRSS2	7	142760398	142774564	-0.388946609	PASS
PRSS21	16	2817180	2826304	0.069936844	PASS
PRSS22	16	2852727	2858170	0.069936844	PASS
PRSS23	11	86791059	86952910	0.062969153	PASS
PRSS27	16	2712418	2720551	0.069936844	PASS
PRSS3	9	33750466	33799231	-0.310444659	PASS
PRSS33	16	2783953	2787948	0.069936844	PASS
PRSS35	6	83512538	83525704	-0.368590549	PASS
PRSS36	16	31138925	31150094	0.069936844	PASS
PRSS37	7	141836286	141841487	-0.388946609	PASS
PRSS38	1	227815693	227846470	-0.256698369	PASS
PRSS41	16	2798485	2805302	0.069936844	PASS
PRSS42	3	46829542	46834095	-0.234617007	PASS
PRSS45	3	46742092	46744755	-0.234617007	PASS
PRSS48	4	151277171	151291453	-0.011382299	PASS
PRSS50	3	46712115	46812574	-0.234617007	PASS
PRSS51	8	10482878	10547585	-0.345962679	PASS
PRSS53	16	31083425	31089628	0.069936844	PASS
PRSS54	16	58279997	58295047	-0.371357732	PASS
PRSS55	8	10525546	10554166	-0.345962679	PASS
PRSS56	2	232520463	232525716	0.025928102	PASS

PRSS57	19	685546	695498	0.079632875	PASS
PRSS58	7	142252143	142258058	-0.388946609	PASS
PRSS8	16	31131433	31135762	0.069936844	PASS
PRTFDC1	10	24848607	24952604	0.039811807	PASS
PRTG	15	55611546	55743090	-0.697600509	PASS
PRTN3	19	840960	848175	0.079632875	PASS
PRUNE1	1	151008420	151035713	0.103015788	PASS
PRUNE2	9	76611376	76906087	0.365190713	suppress_segment_too_large
PRX	19	40393768	40413366	0.093487731	suppress_segment_too_large
PRXL2A	10	80407829	80437115	0.072405807	PASS
PRXL2B	1	2586491	2591469	0.373964873	suppress_segment_too_large
PRXL2C	9	96639577	96655303	0.365190713	suppress_segment_too_large
PSAP	10	71816298	71851375	0.072405807	PASS
PSAPL1	4	7430294	7434973	0.279089364	PASS
PSAT1	9	78297143	78330093	0.365190713	suppress_segment_too_large
PSCA	8	142670308	142682724	0.113969953	PASS
PSD	10	102402617	102421539	-0.367947924	PASS
PSD2	5	139795821	139844466	-0.293443042	PASS
PSD3	8	18527301	19084730	-0.345962679	PASS
PSD4	2	113157325	113209396	-0.010901494	PASS
PSEN1	14	73136418	73223691	0.060212819	PASS
PSEN2	1	226870184	226896105	-0.256698369	PASS
PSENEN	19	35745114	35747519	0.093487731	suppress_segment_too_large
PSG1	19	42866464	42879822	-0.459876623	PASS
PSG11	19	43007656	43026512	-0.459876623	PASS
PSG2	19	43064211	43083045	-0.459876623	PASS
PSG3	19	42721638	42740569	-0.459876623	PASS
PSG4	19	43192702	43207299	-0.459876623	PASS
PSG5	19	43166256	43186536	-0.459876623	PASS
PSG6	19	42902079	42919563	-0.459876623	PASS
PSG7	19	42924132	42937178	-0.459876623	PASS
PSG8	19	42752686	42855691	-0.459876623	PASS
PSG9	19	43211791	43269530	-0.459876623	PASS
PSIP1	9	15464066	15511019	-0.310444659	PASS
PSKH1	16	67893272	67929678	-0.371357732	PASS
PSKH2	8	86048373	86088621	0.279445683	PASS
PSMA1	11	14504874	14643635	-0.405444717	PASS
PSMA2	7	42916857	42932223	-0.69513118	PASS
PSMA3	14	58244831	58272012	0.060212819	PASS
PSMA4	15	78540405	78552419	-0.697600509	PASS
PSMA5	1	109399031	109426427	0.033763446	PASS
PSMA6	14	35278633	35317493	0.060212819	PASS
PSMA7	20	62136735	62143440	-0.168668583	PASS
PSMA8	18	26133852	26193355	-0.376397843	PASS
PSMB1	6	170535117	170553341	-0.035448111	PASS
PSMB10	16	67934502	67937087	-0.371357732	PASS

PSMB11	14	23042167	23044276	0.060212819	PASS
PSMB2	1	35599544	35641844	0.033763446	PASS
PSMB3	17	38752736	38764231	0.060555011	PASS
PSMB4	1	151399534	151401944	0.103015788	PASS
PSMB5	14	23016543	23035230	0.060212819	PASS
PSMB6	17	4796144	4798503	0.128406487	PASS
PSMB7	9	124353466	124415444	0.365190713	suppress_segment_too_large
PSMB8	6	32840717	32844703	0.048820648	PASS
PSMB9	6	32844136	32859585	0.048820648	PASS
PSMC1	14	90256495	90275429	-0.192197564	PASS
PSMC2	7	103344254	103369395	-0.388946609	PASS
PSMC3	11	47418769	47426473	0.117376172	PASS
PSMC3IP	17	42572315	42577831	0.088516311	PASS
PSMC4	19	39971005	39981441	0.093487731	suppress_segment_too_large
PSMC5	17	63827152	63832026	0.088516311	PASS
PSMC6	14	52707172	52728587	0.060212819	PASS
PSMD1	2	231056864	231172827	0.025928102	PASS
PSMD10	23	108084207	108091618	-0.012567603	PASS
PSMD11	17	32444261	32483318	0.060555011	PASS
PSMD12	17	67337916	67366627	0.088516311	PASS
PSMD13	11	236546	252984	-0.333835828	PASS
PSMD14	2	161308038	161411717	0.039726562	PASS
PSMD2	3	184298709	184309054	0.318278996	suppress_segment_too_large
PSMD3	17	39980797	39997960	0.060555011	PASS
PSMD4	1	151254703	151267479	0.103015788	PASS
PSMD5	9	120815496	120842984	0.365190713	suppress_segment_too_large
PSMD6	3	64010549	64024010	-0.234617007	PASS
PSMD7	16	74296775	74306288	-0.371357732	PASS
PSMD8	19	38374536	38383824	0.093487731	suppress_segment_too_large
PSMD9	12	121888731	121918297	0.202141228	PASS
PSME1	14	24136158	24138967	0.060212819	PASS
PSME2	14	24143362	24147570	0.060212819	PASS
PSME3	17	42824385	42843758	0.088516311	PASS
PSME4	2	53864067	53970840	0.040498426	PASS
PSMF1	20	1113263	1189415	-0.168668583	PASS
PSMG1	21	39174769	39183851	-0.064425456	suppress_segment_too_large
PSMG2	18	12658043	12725740	-0.052305056	suppress_segment_too_large
PSMG3	7	1567330	1571005	-0.69513118	PASS
PSMG4	6	3231403	3303373	0.048820648	PASS
PSORS1C1	6	31114750	31140092	0.048820648	PASS
PSORS1C2	6	31137536	31139350	0.048820648	PASS
PSPC1	13	19674752	19783019	-0.390786075	PASS
PSPH	7	56011051	56051604	-0.750061359	PASS
PSPN	19	6375148	6379058	0.079632875	PASS
PSRC1	1	109279556	109283186	0.033763446	PASS
PSTK	10	122954381	122997513	0.063874692	PASS

PSTPIP1	15	76993359	77037332	-0.697600509	PASS
PSTPIP2	18	45983536	46072272	-0.376397843	PASS
PTAFR	1	28147166	28193936	0.033763446	PASS
PTAR1	9	69709522	69759959	0.365190713	suppress_segment_too_large
PTBP1	19	797075	812327	0.079632875	PASS
PTBP2	1	96721665	96823738	0.033763446	PASS
PTBP3	9	112217716	112333667	0.365190713	suppress_segment_too_large
PTCD1	7	99416739	99466163	-0.262778904	PASS
PTCD2	5	72320367	72368395	-0.293443042	PASS
PTCD3	2	86106182	86142157	0.066915641	PASS
PTCH1	9	95442980	95517057	0.365190713	suppress_segment_too_large
PTCH2	1	44819844	44843063	0.033763446	PASS
PTCHD1	23	23334016	23404372	-0.012567603	PASS
PTCHD4	6	47878028	48068689	0.048820648	PASS
PTCRA	6	42915989	42925835	0.048820648	PASS
PTDSS1	8	96261715	96336995	0.279445683	PASS
PTDSS2	11	448268	491399	-0.333835828	PASS
PTEN	10	87863113	87971930	-1.64589449	RESCUE
PTER	10	16436943	16513745	0.039811807	PASS
PTF1A	10	23192327	23194252	0.039811807	PASS
PTGDR	14	52267713	52276724	0.060212819	PASS
PTGDR2	11	60850940	60855971	0.182439925	suppress_segment_too_large
PTGDS	9	136975092	136981742	0.365190713	suppress_segment_too_large
PTGER1	19	14472466	14475362	0.093487731	suppress_segment_too_large
PTGER2	14	52314305	52328606	0.060212819	PASS
PTGER3	1	70852353	71047808	0.033763446	PASS
PTGER4	5	40679498	40693735	-0.293443042	PASS
PTGES	9	129738331	129753047	0.365190713	suppress_segment_too_large
PTGES2	9	128120693	128128462	0.365190713	suppress_segment_too_large
PTGES3	12	56663341	56688408	-0.202191512	PASS
PTGES3L	17	42968088	42980433	0.088516311	PASS
PTGES3L-.	17	42950526	42980528	0.088516311	PASS
PTGFR	1	78303884	78539749	0.033763446	PASS
PTGFRN	1	116910057	116990358	0.033763446	PASS
PTGIR	19	46620468	46625118	0.14996833	PASS
PTGIS	20	49503874	49568146	-0.168668583	PASS
PTGR1	9	111549722	111599893	0.365190713	suppress_segment_too_large
PTGR2	14	73851844	73886827	0.060212819	PASS
PTGS1	9	122370530	122395703	0.365190713	suppress_segment_too_large
PTGS2	1	186671791	186680427	-0.027073548	PASS
PTH	11	13492055	13496181	-0.405444717	PASS
PTH1R	3	46877746	46903799	-0.234617007	PASS
PTH2	19	49422414	49423441	0.14996833	PASS
PTH2R	2	208359714	208854503	0.025928102	PASS
PTHLH	12	27958084	27972705	0.362961408	suppress_segment_too_large
PTK2	8	140657900	141002216	0.113969953	PASS

PTK2B	8	27311482	27459391		-0.345962679	PASS
PTK6	20	63528001	63537370		-0.168668583	PASS
PTK7	6	43076268	43161719		0.048820648	PASS
PTMA	2	231706895	231713541		0.025928102	PASS
PTMS	12	6765516	6770952		-0.086128028	PASS
PTN	7	137227341	137343865		-0.388946609	PASS
PTOV1	19	49850735	49860744		0.14996833	PASS
PTP4A1	6	63521746	63583377		0.048820648	PASS
PTP4A2	1	31906421	31944856		0.033763446	PASS
PTP4A3	8	141391993	141432454		0.113969953	PASS
PTPA	9	129110950	129148946		0.365190713	suppress_segment_too_large
PTPDC1	9	94030794	94109856		0.365190713	suppress_segment_too_large
PTPMT1	11	47565430	47573461		0.117376172	PASS
PTPN1	20	50510321	50585241		-0.168668583	PASS
PTPN11	12	112418351	112509913		0.202141228	PASS
PTPN12	7	77537275	77640071		-0.262778904	PASS
PTPN13	4	86594315	86815171		-0.011382299	PASS
PTPN14	1	214348696	214552449		-0.256698369	PASS
PTPN18	2	130356007	130375409		-0.475215623	PASS
PTPN2	18	12785478	12929643		-0.052305056	suppress_segment_too_large
PTPN20	10	46911396	47002488		-0.404852538	PASS
PTPN21	14	88465778	88554733		-0.192197564	PASS
PTPN22	1	113813811	113871759		0.033763446	PASS
PTPN23	3	47381011	47413441		-0.234617007	PASS
PTPN3	9	109375466	109498313		0.365190713	suppress_segment_too_large
PTPN4	2	119759631	119983818		-0.475215623	PASS
PTPN5	11	18727928	18792721		-0.405444717	PASS
PTPN6	12	6946468	6961316		-0.086128028	PASS
PTPN7	1	202147013	202161588		-0.256698369	PASS
PTPN9	15	75463251	75579289		-0.697600509	PASS
PTPRA	20	2864184	3039076		-0.168668583	PASS
PTPRB	12	70515866	70637440		-0.202191512	PASS
PTPRC	1	198638457	198757476		-0.027073548	PASS
PTPRCAP	11	67435510	67438067		0.182439925	suppress_segment_too_large
PTPRD	9	8314246	10612723		-0.310444659	PASS
PTPRE	10	127907061	128085855		0.063874692	PASS
PTPRF	1	43525187	43623666		0.033763446	PASS
PTPRG	3	61561569	62297613		-0.234617007	PASS
PTPRH	19	55181248	55209506		0.121336758	PASS
PTPRJ	11	47980558	48170841		-0.621795272	PASS
PTPRK	6	127968779	128520674		-0.458570262	PASS
PTPRM	18	7566782	8406861		-0.052305056	suppress_segment_too_large
PTPRN	2	219289623	219309648		0.025928102	PASS
PTPRN2	7	157539056	158587788		-0.411415677	PASS
PTPRO	12	15322397	15597399		0.362961408	suppress_segment_too_large
PTPRQ	12	80402178	80680271		0.202141228	PASS

PTPRR	12	70638073	70920843	-0.202191512	PASS
PTPRS	19	5158495	5340803	0.079632875	PASS
PTPRT	20	42072752	43189970	-0.168668583	PASS
PTPRU	1	29236516	29326813	0.033763446	PASS
PTPRZ1	7	121873089	122062036	-0.388946609	PASS
PTRH1	9	127690348	127724873	0.365190713	suppress_segment_too_large
PTRH2	17	59674636	59707626	0.088516311	PASS
PTRHD1	2	24789734	24793382	0.040498426	PASS
PTS	11	112226365	112269955	-0.356188624	PASS
PTTG1	5	160421822	160428744	-0.150979337	PASS
PTTG1IP	21	44849585	44873903	-0.064425456	suppress_segment_too_large
PTTG2	4	37960435	37961125	-0.011382299	PASS
PTX3	3	157436789	157443628	0.318278996	suppress_segment_too_large
PTX4	16	1485886	1488981	0.069936844	PASS
PUDP	23	7048920	7148190	-0.012567603	PASS
PUF60	8	143816344	143829859	0.307788765	PASS
PUM1	1	30931506	31065991	0.033763446	PASS
PUM2	2	20248691	20352234	0.040498426	PASS
PUM3	9	2720469	2844241	-0.310444659	PASS
PURA	5	140107777	140125619	-0.293443042	PASS
PURB	7	44876293	44885361	-0.69513118	PASS
PURG	8	30995802	31033715	-0.345962679	PASS
PUS1	12	131929200	131945896	0.270848837	PASS
PUS10	2	60940222	61018259	-0.372959126	PASS
PUS3	11	125893485	125903221	-0.419612865	PASS
PUS7	7	105439661	105522267	-0.388946609	PASS
PUS7L	12	43718993	43758817	-0.202191512	PASS
PUSL1	1	1308567	1311677	0.373964873	suppress_segment_too_large
PVALB	22	36800684	36819479	-0.575930959	PASS
PVALEF	17	81165507	81183164	0.088516311	PASS
PVR	19	44643798	44663583	0.14996833	PASS
PVRIG	7	100218241	100221490	-0.262778904	PASS
PWP1	12	107685732	107713167	0.202141228	PASS
PWP2	21	44107290	44131181	-0.064425456	suppress_segment_too_large
PWWP2A	5	160061801	160119423	-0.150979337	PASS
PWWP2B	10	132397168	132417863	0.063874692	PASS
PXDC1	6	3722614	3752026	0.048820648	PASS
PXDN	2	1631887	1744852	0.040498426	PASS
PXDNL	8	51319578	51809445	-0.201142258	PASS
PXK	3	58332880	58426126	-0.234617007	PASS
PXMP2	12	132687606	132704991	0.270848837	PASS
PXMP4	20	33702754	33720319	-0.168668583	PASS
PXN	12	120210439	120265771	0.202141228	PASS
PXT1	6	36390551	36442889	0.048820648	PASS
PXYLP1	3	141228726	141367753	-0.356957581	PASS
PYCARD	16	31201485	31203450	0.069936844	PASS

PYCR1	17	81932384	81942412	0.088516311	PASS
PYCR2	1	225919877	225924340	-0.256698369	PASS
PYCR3	8	143603913	143609773	0.307788765	PASS
PYDC1	16	31215962	31217359	0.069936844	PASS
PYDC2	3	191461163	191461456	-0.627320105	PASS
PYGB	20	25248069	25298014	-0.168668583	PASS
PYGL	14	50857891	50944736	0.060212819	PASS
PYGM	11	64746389	64760297	0.182439925	suppress_segment_too_large
PYGO1	15	55538890	55588947	-0.697600509	PASS
PYGO2	1	154957026	154963853	0.103015788	PASS
PYHIN1	1	158930796	158977054	-0.467855874	PASS
PYM1	12	55901413	55932618	-0.202191512	PASS
PYROXD1	12	21437615	21471252	0.362961408	suppress_segment_too_large
PYROXD2	10	98383565	98415184	-0.367947924	PASS
PYURF	4	88520985	88523813	-0.011382299	PASS
PYY	17	43952738	44004469	0.088516311	PASS
PZP	12	9148840	9208370	0.362961408	suppress_segment_too_large
QARS	3	49095932	49105135	-0.234617007	PASS
QDPR	4	17460261	17512234	0.279089364	PASS
QKI	6	163414000	163578596	-0.035448111	PASS
QPCT	2	37342827	37373322	0.040498426	PASS
QPCTL	19	45692483	45703989	0.14996833	PASS
QPRT	16	29679008	29698699	0.069936844	PASS
QRFP	9	130892702	130896812	0.365190713	suppress_segment_too_large
QRFPR	4	121329312	121381059	-0.011382299	PASS
QRICH1	3	49029707	49094363	-0.234617007	PASS
QRICH2	17	76274049	76307998	0.088516311	PASS
QRSL1	6	106629578	106668417	-0.368590549	PASS
QSER1	11	32892820	32993316	-0.405444717	PASS
QSOX1	1	180154834	180204030	-0.027073548	PASS
QSOX2	9	136206333	136245841	0.365190713	suppress_segment_too_large
QTRT1	19	10701430	10713437	0.093487731	suppress_segment_too_large
QTRT2	3	114005833	114088422	0.313454204	suppress_segment_too_large
R3HCC1	8	23270120	23296279	-0.345962679	PASS
R3HCC1L	10	98134624	98244897	-0.367947924	PASS
R3HDM1	2	135531455	135725270	-0.475215623	PASS
R3HDM2	12	57253762	57431005	-0.202191512	PASS
R3HDM4	19	896503	913245	0.079632875	PASS
R3HDML	20	44336986	44351235	-0.168668583	PASS
RAB10	2	26034107	26137454	0.040498426	PASS
RAB11A	15	65726054	65891991	-0.697600509	PASS
RAB11B	19	8389981	8404434	0.079632875	PASS
RAB11FIP	8	37858618	37899467	-0.201142258	PASS
RAB11FIP	10	118004916	118046603	0.063874692	PASS
RAB11FIP	16	425619	523011	0.069936844	PASS
RAB11FIP	17	31391624	31538217	0.060555011	PASS

RAB11FIP	2	73073382	73156721	-0.372959126	PASS
RAB12	18	8609437	8639382	-0.052305056	suppress_segment_too_large
RAB13	1	153981617	153986358	0.103015788	PASS
RAB14	9	121178137	121223014	0.365190713	suppress_segment_too_large
RAB15	14	64945814	64973226	0.060212819	PASS
RAB17	2	237574322	237601614	0.025928102	PASS
RAB18	10	27504174	27542237	0.039811807	PASS
RAB19	7	140404043	140426250	-0.388946609	PASS
RAB1A	2	65070696	65130331	-0.372959126	PASS
RAB1B	11	66268533	66277492	0.182439925	suppress_segment_too_large
RAB20	13	110523066	110561733	-0.390786075	PASS
RAB21	12	71754874	71800285	-0.202191512	PASS
RAB22A	20	58309696	58367507	-0.168668583	PASS
RAB23	6	57186992	57222314	0.048820648	PASS
RAB24	5	177301198	177303744	-0.150979337	PASS
RAB25	1	156061160	156070514	0.103015788	PASS
RAB26	16	2140803	2154165	0.069936844	PASS
RAB27A	15	55202966	55319113	-0.697600509	PASS
RAB27B	18	54717860	54895516	-0.376397843	PASS
RAB28	4	13361354	13484365	0.279089364	PASS
RAB29	1	205767986	205775460	-0.256698369	PASS
RAB2A	8	60516857	60623627	-0.201142258	PASS
RAB2B	14	21459020	21476973	0.060212819	PASS
RAB30	11	82973133	83071923	0.062969153	PASS
RAB31	18	9708165	9862551	-0.052305056	suppress_segment_too_large
RAB32	6	146543693	146554965	-0.035448111	PASS
RAB33A	23	130171649	130184870	-0.012567603	PASS
RAB33B	4	139453232	139476609	-0.011382299	PASS
RAB34	17	28714281	28718429	0.060555011	PASS
RAB35	12	120095095	120117502	0.202141228	PASS
RAB36	22	23145326	23164350	-0.575930959	PASS
RAB37	17	74670578	74747335	0.088516311	PASS
RAB38	11	88113242	88175467	0.062969153	PASS
RAB39A	11	107928503	107963482	-0.356188624	PASS
RAB39B	23	155258241	155264589	-0.012567603	PASS
RAB3A	19	18196784	18204074	0.093487731	suppress_segment_too_large
RAB3B	1	51907956	51990764	0.033763446	PASS
RAB3C	5	58582221	58859394	-0.293443042	PASS
RAB3D	19	11322046	11346270	0.093487731	suppress_segment_too_large
RAB3GAP	2	135052265	135176394	-0.475215623	PASS
RAB3GAP	1	220148293	220272454	-0.256698369	PASS
RAB3IL1	11	61897301	61920269	0.182439925	suppress_segment_too_large
RAB3IP	12	69738681	69823204	-0.202191512	PASS
RAB40A	23	103499130	103519489	-0.012567603	PASS
RAB40AL	23	102937272	102938300	-0.012567603	PASS
RAB40B	17	82654973	82698728	0.088516311	PASS

RAB40C	16	589357	629272	0.069936844	PASS
RAB41	23	70282093	70285002	-0.012567603	PASS
RAB42	1	28592200	28595443	0.033763446	PASS
RAB43	3	129087569	129122801	0.313454204	suppress_segment_too_large
RAB44	6	36697810	36733183	0.048820648	PASS
RAB4A	1	229271062	229305894	-0.256698369	PASS
RAB4B	19	40778216	40796938	0.093487731	suppress_segment_too_large
RAB4B-EC	19	40778242	40808418	0.093487731	suppress_segment_too_large
RAB5A	3	19947079	19985175	0.311018244	suppress_segment_too_large
RAB5B	12	55973913	55996683	-0.202191512	PASS
RAB5C	17	42124976	42155044	0.060555011	PASS
RAB5IF	20	36605734	36612557	-0.168668583	PASS
RAB6A	11	73675638	73761137	0.062969153	PASS
RAB6B	3	133824239	133895836	0.313454204	suppress_segment_too_large
RAB6C	2	129979664	129982738	-0.475215623	PASS
RAB6D	2	131361111	131364142	-0.475215623	PASS
RAB7A	3	128726122	128814796	0.313454204	suppress_segment_too_large
RAB7B	1	205976740	206003461	-0.256698369	PASS
RAB8A	19	16111629	16134234	0.093487731	suppress_segment_too_large
RAB8B	15	63189469	63267782	-0.697600509	PASS
RAB9A	23	13689125	13710506	-0.012567603	PASS
RAB9B	23	103822322	103832228	-0.012567603	PASS
RABAC1	19	41956681	41959390	0.093487731	suppress_segment_too_large
RABEP1	17	5282265	5385812	0.128406487	PASS
RABEP2	16	28904421	28936526	0.069936844	PASS
RABEPK	9	125200542	125234158	0.365190713	suppress_segment_too_large
RABGAP1	9	122940833	123104866	0.365190713	suppress_segment_too_large
RABGAP1	1	174159410	174995308	-0.027073548	PASS
RABGEF1	7	66682164	66811464	-0.750061359	PASS
RABGGTA	14	24265538	24271739	0.060212819	PASS
RABGGTE	1	75786197	75795079	0.033763446	PASS
RABIF	1	202878957	202889135	-0.256698369	PASS
RABL2A	2	113627229	113643396	-0.010901494	PASS
RABL2B	22	50767501	50783663	-0.808211634	PASS
RABL3	3	120686681	120742993	0.313454204	suppress_segment_too_large
RABL6	9	136807943	136841187	0.365190713	suppress_segment_too_large
RAC1	7	6374523	6403977	-0.69513118	PASS
RAC2	22	37225261	37244448	-0.575930959	PASS
RAC3	17	82031624	82034204	0.088516311	PASS
RACGAP1	12	49976923	50033136	-0.202191512	PASS
RACK1	5	181236909	181248096	-0.150979337	PASS
RAD1	5	34905264	34918989	0.055007813	PASS
RAD17	5	69369293	69414801	-0.293443042	PASS
RAD18	3	8775402	8963773	0.311018244	suppress_segment_too_large
RAD21	8	116845935	116874866	0.279445683	PASS
RAD21L1	20	1226056	1296421	-0.168668583	PASS

RAD23A	19	12945855	12953642	0.093487731	suppress_segment_too_large
RAD23B	9	107283137	107332194	0.365190713	suppress_segment_too_large
RAD50	5	132556019	132646344	-0.293443042	PASS
RAD51	15	40694774	40732339	-0.396255771	PASS
RAD51AP1	12	4538798	4560048	-0.086128028	PASS
RAD51AP2	2	17510584	17518439	0.040498426	PASS
RAD51B	14	67819779	68730218	0.060212819	PASS
RAD51C	17	58692573	58735611	0.088516311	PASS
RAD51D	17	35092208	35121522	0.060555011	PASS
RAD51L3-	17	35011349	35121493	0.060555011	PASS
RAD52	12	912077	990053	-0.086128028	PASS
RAD54B	8	94371960	94475109	0.279445683	PASS
RAD54L	1	46247688	46278473	0.033763446	PASS
RAD54L2	3	51541144	51668667	-0.234617007	PASS
RAD9A	11	67317871	67398410	0.182439925	suppress_segment_too_large
RAD9B	12	110501655	110532086	0.202141228	PASS
RADIL	7	4797055	4883719	-0.69513118	PASS
RAE1	20	57351010	57379211	-0.168668583	PASS
RAET1E	6	149883375	149898102	-0.035448111	PASS
RAET1G	6	149916878	149923121	-0.035448111	PASS
RAET1L	6	150018334	150025532	-0.035448111	PASS
RAF1	3	12583601	12664226	0.311018244	suppress_segment_too_large
RAG1	11	36510709	36593156	-0.405444717	PASS
RAG2	11	36575574	36598279	-0.405444717	PASS
RAI1	17	17681473	17811453	0.024610076	PASS
RAI14	5	34656237	34832627	0.055007813	PASS
RAI2	23	17800049	17861337	-0.012567603	PASS
RALA	7	39623483	39708124	-0.69513118	PASS
RALB	2	120240064	120294713	-0.475215623	PASS
RALBP1	18	9475009	9538116	-0.052305056	suppress_segment_too_large
RALGAPA	14	35538352	35809304	0.060212819	PASS
RALGAPA	20	20389552	20712488	-0.168668583	PASS
RALGAPB	20	38472816	38578861	-0.168668583	PASS
RALGDS	9	133097720	133149334	0.365190713	suppress_segment_too_large
RALGPS1	9	126914774	127223166	0.365190713	suppress_segment_too_large
RALGPS2	1	178725147	178921841	-0.027073548	PASS
RALY	20	33993646	34108308	-0.168668583	PASS
RALYL	8	84182787	84921844	0.279445683	PASS
RAMAC	15	82986207	82991057	-0.697600509	PASS
RAMACL	6	166585604	166587594	-0.035448111	PASS
RAMP1	2	237858893	237912114	0.025928102	PASS
RAMP2	17	42758447	42763041	0.088516311	PASS
RAMP3	7	45157791	45186302	-0.69513118	PASS
RAN	12	130871879	130877678	0.270848837	PASS
RANBP1	22	20115938	20127357	-0.575930959	PASS
RANBP10	16	67723066	67806652	-0.371357732	PASS

RANBP17	5	170861870	171300015	-0.150979337	PASS
RANBP2	2	108719481	108785811	-0.010901494	PASS
RANBP3	19	5916139	5978142	0.079632875	PASS
RANBP3L	5	36248434	36302114	0.055007813	PASS
RANBP6	9	6011043	6015625	-0.310444659	PASS
RANBP9	6	13621498	13711564	0.048820648	PASS
RANGAP1	22	41245611	41286251	-0.575930959	PASS
RANGRF	17	8288497	8290092	0.128406487	PASS
RAP1A	1	111542218	111716691	0.033763446	PASS
RAP1B	12	68610839	68671901	-0.202191512	PASS
RAP1GAP	1	21596215	21669363	0.033763446	PASS
RAP1GAP2	17	2755705	3037739	0.128406487	PASS
RAP1GDS	4	98261384	98443861	-0.011382299	PASS
RAP2A	13	97434222	97469128	-0.390786075	PASS
RAP2B	3	153162270	153170620	0.318278996	suppress_segment_too_large
RAP2C	23	132203024	132219480	-0.012567603	PASS
RAPGEF1	9	131576770	131740074	0.365190713	suppress_segment_too_large
RAPGEF2	4	159103013	159360174	-0.011382299	PASS
RAPGEF3	12	47734367	47771040	-0.202191512	PASS
RAPGEF4	2	172735274	173052893	0.039726562	PASS
RAPGEF5	7	22118238	22357144	-0.69513118	PASS
RAPGEF6	5	131423921	131635236	-0.293443042	PASS
RAPGEFL	17	40177010	40195656	0.060555011	PASS
RAPH1	2	203394345	203535410	0.025928102	PASS
RAPSN	11	47437757	47449178	0.117376172	PASS
RARA	17	40309192	40357643	0.060555011	PASS
RARB	3	25174332	25597932	0.311018244	suppress_segment_too_large
RARG	12	53210567	53232980	-0.202191512	PASS
RARRES1	3	158696892	158732696	0.318278996	suppress_segment_too_large
RARRES2	7	150338317	150341674	-0.388946609	PASS
RARRES3	11	63536809	63546462	0.182439925	suppress_segment_too_large
RARS	5	168486445	168519299	-0.150979337	PASS
RARS2	6	87514378	87590003	-0.368590549	PASS
RASA1	5	87267888	87391931	-0.293443042	PASS
RASA2	3	141487047	141615342	-0.356957581	PASS
RASA3	13	113977783	114132611	-0.390786075	PASS
RASA4	7	102573807	102616757	-0.388946609	PASS
RASA4B	7	102482445	102517781	-0.388946609	PASS
RASAL1	12	113098819	113136239	0.202141228	PASS
RASAL2	1	178094141	178484147	-0.027073548	PASS
RASAL3	19	15451624	15464571	0.093487731	suppress_segment_too_large
RASD1	17	17494437	17496395	0.024610076	PASS
RASD2	22	35540868	35554001	-0.575930959	PASS
RASEF	9	82979585	83063177	0.365190713	suppress_segment_too_large
RASGEF1A	10	43194535	43266919	-0.404852538	PASS
RASGEF1B	4	81426393	82044244	-0.011382299	PASS

RASGEF1(	5	180100791	180209153	-0.150979337	PASS
RASGRF1	15	78959947	79090773	-0.697600509	PASS
RASGRF2	5	80960672	81230156	-0.293443042	PASS
RASGRP1	15	38488103	38565575	-0.396255771	PASS
RASGRP2	11	64726911	64745456	0.182439925	suppress_segment_too_large
RASGRP3	2	33436324	33564750	0.040498426	PASS
RASGRP4	19	38409051	38426305	0.093487731	suppress_segment_too_large
RASIP1	19	48720587	48740721	0.14996833	PASS
RASL10A	22	29312933	29319679	-0.575930959	PASS
RASL10B	17	35731649	35743521	0.060555011	PASS
RASL11A	13	27270327	27273690	-0.390786075	PASS
RASL11B	4	52862290	52866835	-0.011382299	PASS
RASL12	15	65053337	65076690	-0.697600509	PASS
RASSF1	3	50329782	50340980	-0.234617007	PASS
RASSF10	11	13009577	13012106	-0.405444717	PASS
RASSF2	20	4780023	4823645	-0.168668583	PASS
RASSF3	12	64507001	64697567	-0.202191512	PASS
RASSF4	10	44959407	44995891	-0.404852538	PASS
RASSF5	1	206507530	206589448	-0.256698369	PASS
RASSF6	4	73571550	73620631	-0.011382299	PASS
RASSF7	11	560404	564021	-0.333835828	PASS
RASSF8	12	25959029	26079892	0.362961408	suppress_segment_too_large
RASSF9	12	85800697	85836570	0.202141228	PASS
RAVER1	19	10316212	10333640	0.093487731	suppress_segment_too_large
RAVER2	1	64745095	64833232	0.033763446	PASS
RAX	18	59267035	59274086	-0.376397843	PASS
RAX2	19	3769089	3772221	0.079632875	PASS
RB1	13	48303726	48599436	-0.390786075	PASS
RB1CC1	8	52622456	52745843	-0.201142258	PASS
RBAK	7	5045821	5069488	-0.69513118	PASS
RBAK-RB.	7	4983718	5073221	-0.69513118	PASS
RBBP4	1	32651142	32686211	0.033763446	PASS
RBBP5	1	205086142	205122015	-0.256698369	PASS
RBBP6	16	24537693	24572863	0.069936844	PASS
RBBP7	23	16839283	16870414	-0.012567603	PASS
RBBP8	18	22798261	23026488	-0.376397843	PASS
RBBP8NL	20	62410237	62427533	-0.168668583	PASS
RBBP9	20	18486540	18497243	-0.168668583	PASS
RBCK1	20	407498	430966	-0.168668583	PASS
RBFA	18	80034358	80046397	-0.376397843	PASS
RBFOX1	16	5239802	7713338	0.069936844	PASS
RBFOX2	22	35738736	36028425	-0.575930959	PASS
RBFOX3	17	79089345	79516148	0.088516311	PASS
RBKS	2	27781364	27891098	0.040498426	PASS
RBL1	20	36996349	37095995	-0.168668583	PASS
RBL2	16	53433977	53491649	-0.371357732	PASS

RBM10	23	47144869	47186813	-0.012567603	PASS
RBM11	21	14216130	14228372	-0.304975024	PASS
RBM12	20	35648925	35664956	-0.168668583	PASS
RBM12B	8	93729356	93741017	0.279445683	PASS
RBM14	11	66616582	66627347	0.182439925	suppress_segment_too_large
RBM14-RI	11	66616626	66646469	0.182439925	suppress_segment_too_large
RBM15	1	110338506	110346681	0.033763446	PASS
RBM15B	3	51391268	51397908	-0.234617007	PASS
RBM17	10	6088987	6117457	0.039811807	PASS
RBM18	9	122237624	122264839	0.365190713	suppress_segment_too_large
RBM19	12	113816738	113966371	0.202141228	PASS
RBM20	10	110644397	110839469	0.063874692	PASS
RBM22	5	150690794	150701107	-0.293443042	PASS
RBM23	14	22893206	22919184	0.060212819	PASS
RBM24	6	17281346	17293875	0.048820648	PASS
RBM25	14	73058436	73123898	0.060212819	PASS
RBM26	13	79311824	79406477	-0.390786075	PASS
RBM27	5	146203600	146289132	-0.293443042	PASS
RBM28	7	128297685	128343908	-0.388946609	PASS
RBM3	23	48574449	48579066	-0.012567603	PASS
RBM33	7	155644451	155781485	-0.411415677	PASS
RBM34	1	235131183	235161457	-0.256698369	PASS
RBM38	20	57391407	57409333	-0.168668583	PASS
RBM39	20	35701347	35742312	-0.168668583	PASS
RBM4	11	66638617	66668374	0.182439925	suppress_segment_too_large
RBM41	23	107064420	107118823	-0.012567603	PASS
RBM42	19	35629030	35637686	0.093487731	suppress_segment_too_large
RBM43	2	151247940	151261879	-0.475215623	PASS
RBM44	2	237798389	237842808	0.025928102	PASS
RBM45	2	178112424	178139011	0.039726562	PASS
RBM46	4	154781213	154828813	-0.011382299	PASS
RBM47	4	40423267	40630875	-0.011382299	PASS
RBM48	7	92528773	92538005	-0.262778904	PASS
RBM4B	11	66664998	66677921	0.182439925	suppress_segment_too_large
RBM5	3	50088908	50119021	-0.234617007	PASS
RBM6	3	49940007	50100045	-0.234617007	PASS
RBM7	11	114400030	114414203	-0.356188624	PASS
RBM8A	1	145917714	145927678	-0.157385092	PASS
RBMS1	2	160272151	160493794	0.039726562	PASS
RBMS2	12	56521929	56596196	-0.202191512	PASS
RBMS3	3	28574791	30010391	0.311018244	suppress_segment_too_large
RBMX	23	136848004	136880764	-0.012567603	PASS
RBMX2	23	130401969	130413343	-0.012567603	PASS
RBMXL1	1	88979456	88992960	0.033763446	PASS
RBMXL2	11	7089027	7090900	-0.405444717	PASS
RBMXL3	23	115189427	115192868	-0.012567603	PASS

RBP1	3	139517434	139539829	-0.356957581	PASS
RBP2	3	139452884	139480747	0.313454204	suppress_segment_too_large
RBP3	10	47348371	47357875	-0.404852538	PASS
RBP4	10	93591687	93601744	-0.39678585	PASS
RBP5	12	7123684	7128942	-0.086128028	PASS
RBP7	1	9997206	10016020	0.373964873	suppress_segment_too_large
RBPJ	4	26163455	26435131	-0.458181871	PASS
RBPJL	20	45306851	45317824	-0.168668583	PASS
RBPMS	8	30384479	30572261	-0.345962679	PASS
RBPMS2	15	64739892	64775587	-0.697600509	PASS
RBSN	3	15070073	15099163	0.311018244	suppress_segment_too_large
RBX1	22	40951347	40973309	-0.575930959	PASS
RC3H1	1	173931214	174022297	-0.027073548	PASS
RC3H2	9	122844556	122905341	0.365190713	suppress_segment_too_large
RCAN1	21	34513142	34615142	0.12860881	suppress_segment_too_large
RCAN2	6	46220736	46491972	0.048820648	PASS
RCAN3	1	24502351	24541040	0.033763446	PASS
RCBTB1	13	49531946	49585583	-0.390786075	PASS
RCBTB2	13	48488959	48533256	-0.390786075	PASS
RCC1	1	28505943	28539300	0.033763446	PASS
RCC1L	7	75027122	75074228	-0.262778904	PASS
RCC2	1	17406760	17439724	0.033763446	PASS
RCCD1	15	90954870	90963125	-0.697600509	PASS
RCE1	11	66842835	66846546	0.182439925	suppress_segment_too_large
RCHY1	4	75479037	75514764	-0.011382299	PASS
RCL1	9	4792869	4885917	-0.310444659	PASS
RCN1	11	32090904	32105755	-0.405444717	PASS
RCN2	15	76931619	76954392	-0.697600509	PASS
RCN3	19	49527618	49546962	0.14996833	PASS
RCOR1	14	102592661	102730576	-0.192197564	PASS
RCOR2	11	63911221	63916844	0.182439925	suppress_segment_too_large
RCOR3	1	211258377	211316385	-0.256698369	PASS
RCSD1	1	167630093	167706249	-0.027073548	PASS
RCVRN	17	9896320	9905621	-0.139031004	PASS
RD3	1	211476522	211492917	-0.256698369	PASS
RD3L	14	103940426	103942561	-0.192197564	PASS
RDH10	8	73294612	73325281	-0.201142258	PASS
RDH11	14	67676801	67695814	0.060212819	PASS
RDH12	14	67701886	67734452	0.060212819	PASS
RDH13	19	55039108	55071291	0.121336758	PASS
RDH14	2	18554723	18560680	0.040498426	PASS
RDH16	12	56951431	56959374	-0.202191512	PASS
RDH5	12	55720367	55724705	-0.202191512	PASS
RDH8	19	10013249	10022279	0.093487731	suppress_segment_too_large
RDM1	17	35918066	35930773	0.060555011	PASS
RDX	11	109864295	110296722	-0.356188624	PASS

REC114	15	73443158	73560014	-0.697600509	PASS
REC8	14	24171853	24180257	0.060212819	PASS
RECK	9	36036433	36124451	-0.310444659	PASS
RECQL	12	21468911	21501669	0.362961408	suppress_segment_too_large
RECQL4	8	144511288	144517845	0.307788765	PASS
RECQL5	17	75626845	75667189	0.088516311	PASS
REEP1	2	86213993	86338083	0.066915641	PASS
REEP2	5	138439017	138446969	-0.293443042	PASS
REEP3	10	63521363	63625123	0.072405807	PASS
REEP4	8	22138020	22141951	-0.345962679	PASS
REEP5	5	112876379	112922539	-0.293443042	PASS
REEP6	19	1490747	1497927	0.079632875	PASS
REG1A	2	79120362	79123419	-0.372959126	PASS
REG1B	2	79085030	79088019	-0.372959126	PASS
REG3A	2	79157003	79159753	-0.372959126	PASS
REG3G	2	79025686	79028505	-0.372959126	PASS
REG4	1	119794018	119811660	-0.157385092	PASS
REL	2	60881521	60931610	-0.372959126	PASS
RELA	11	65653596	65663094	0.182439925	suppress_segment_too_large
RELB	19	45001430	45038198	0.14996833	PASS
RELCH	18	62187255	62310217	-0.376397843	PASS
RELL1	4	37590800	37686376	-0.011382299	PASS
RELL2	5	141636950	141641077	-0.293443042	PASS
RELN	7	103471784	103989516	-0.388946609	PASS
RELT	11	73376264	73397474	0.062969153	PASS
REM1	20	31475293	31484905	-0.168668583	PASS
REM2	14	22883165	22887686	0.060212819	PASS
REN	1	204154819	204190324	-0.256698369	PASS
RENBP	23	153935263	153944691	-0.012567603	PASS
REP15	12	27696519	27697596	0.362961408	suppress_segment_too_large
REPIN1	7	150368189	150374044	-0.388946609	PASS
REPS1	6	138903493	138988261	-0.035448111	PASS
REPS2	23	16946691	17153280	-0.012567603	PASS
RER1	1	2391775	2405444	0.373964873	suppress_segment_too_large
RERE	1	8352397	8817643	0.373964873	suppress_segment_too_large
RERG	12	15107783	15348675	0.362961408	suppress_segment_too_large
RERGL	12	18080869	18320107	0.362961408	suppress_segment_too_large
RESP18	2	219327409	219333177	0.025928102	PASS
REST	4	56907876	56966678	-0.011382299	PASS
RET	10	43077027	43130351	-0.404852538	PASS
RETN	19	7669044	7670454	0.079632875	PASS
RETNLB	3	108743424	108757384	0.313454204	suppress_segment_too_large
RETREG1	5	16473038	16617058	0.055007813	PASS
RETREG2	2	219176225	219185479	0.025928102	PASS
RETREG3	17	42579513	42610623	0.088516311	PASS
RETSAT	2	85342088	85354620	0.066915641	PASS

REV1	2	99400475	99490035	0.048238121	PASS
REV3L	6	111299028	111483715	-0.368590549	PASS
REX1BD	19	18588685	18592336	0.093487731	suppress_segment_too_large
REXO1	19	1815246	1848463	0.079632875	PASS
REXO2	11	114439386	114450279	-0.356188624	PASS
REXO4	9	133406059	133418096	0.365190713	suppress_segment_too_large
REXO5	16	20806429	20849668	0.069936844	PASS
RFC1	4	39287456	39366375	-0.011382299	PASS
RFC2	7	74231499	74254458	-0.262778904	PASS
RFC3	13	33818049	33966558	-0.390786075	PASS
RFC4	3	186789880	186807058	0.318278996	suppress_segment_too_large
RFC5	12	118013588	118033130	0.202141228	PASS
RFESD	5	95646754	95684773	-0.293443042	PASS
RFFL	17	35005990	35089319	0.060555011	PASS
RFK	9	76385517	76394517	0.365190713	suppress_segment_too_large
RFLNA	12	123973241	124316024	0.202141228	PASS
RFLNB	17	439978	445939	0.128406487	PASS
RFNG	17	82047902	82051831	0.088516311	PASS
RFPL1	22	29438583	29442455	-0.575930959	PASS
RFPL2	22	32190435	32203477	-0.575930959	PASS
RFPL3	22	32354885	32361161	-0.575930959	PASS
RFPL4A	19	55759014	55763175	-0.180829161	PASS
RFPL4AL1	19	55769141	55773179	-0.180829161	PASS
RFPL4B	6	112347330	112351296	-0.368590549	PASS
RFT1	3	53088483	53130462	-0.234617007	PASS
RFTN1	3	16313574	16514026	0.311018244	suppress_segment_too_large
RFTN2	2	197568224	197676045	0.025928102	PASS
RFWD3	16	74621394	74666881	-0.371357732	PASS
RFX1	19	13961538	14007039	0.093487731	suppress_segment_too_large
RFX2	19	5993164	6199572	0.079632875	PASS
RFX3	9	3218297	3526004	-0.310444659	PASS
RFX4	12	106582907	106762803	0.202141228	PASS
RFX5	1	151340640	151347357	0.103015788	PASS
RFX6	6	116877212	116932163	-0.368590549	PASS
RFX7	15	56087280	56243266	-0.697600509	PASS
RFX8	2	101397361	101474703	0.048238121	PASS
RFXANK	19	19192229	19201869	0.093487731	suppress_segment_too_large
RFXAP	13	36819224	36829104	-0.390786075	PASS
RGCC	13	41457559	41470882	-0.390786075	PASS
RGL1	1	183636085	183928531	-0.027073548	PASS
RGL2	6	33291654	33299324	0.048820648	PASS
RGL3	19	11384341	11419342	0.093487731	suppress_segment_too_large
RGL4	22	23688136	23699176	-0.575930959	PASS
RGMA	15	93035273	93089204	-0.697600509	PASS
RGMB	5	98768650	98798643	-0.293443042	PASS
RGN	23	47078355	47093314	-0.012567603	PASS

RGP1	9	35749287	35758575	-0.310444659	PASS
RGPD1	2	86907953	87013976	-0.340781246	PASS
RGPD2	2	87755955	87825952	-0.340781246	PASS
RGPD3	2	106391290	106468376	-0.532032081	PASS
RGPD4	2	107826937	107890841	-0.532032081	PASS
RGPD6	2	110513812	110577185	-0.010901494	PASS
RGPD8	2	112370092	112434488	-0.010901494	PASS
RGR	10	84245053	84259960	0.072405807	PASS
RGS1	1	192575727	192580031	-0.027073548	PASS
RGS10	10	119499828	119542708	0.063874692	PASS
RGS11	16	268301	275980	0.069936844	PASS
RGS12	4	3293028	3439913	0.279089364	PASS
RGS13	1	192636138	192660306	-0.027073548	PASS
RGS14	5	177357837	177372601	-0.150979337	PASS
RGS16	1	182598623	182604408	-0.027073548	PASS
RGS17	6	153004459	153131249	-0.035448111	PASS
RGS18	1	192158457	192185815	-0.027073548	PASS
RGS19	20	64073181	64079988	-0.168668583	PASS
RGS2	1	192809039	192812283	-0.027073548	PASS
RGS20	8	53851808	53959303	-0.201142258	PASS
RGS21	1	192316992	192367285	-0.027073548	PASS
RGS22	8	99960936	100131268	0.279445683	PASS
RGS3	9	113444731	113597743	0.365190713	suppress_segment_too_large
RGS4	1	163068775	163076802	-0.027073548	PASS
RGS5	1	163111121	163321791	-0.027073548	PASS
RGS6	14	71932439	72566529	0.060212819	PASS
RGS7	1	240775515	241357230	-0.648598641	PASS
RGS7BP	5	64506257	64612312	-0.293443042	PASS
RGS8	1	182641816	182684576	-0.027073548	PASS
RGS9	17	65100812	65227703	0.088516311	PASS
RGS9BP	19	32675407	32678300	0.093487731	suppress_segment_too_large
RGSL1	1	182409192	182560599	-0.027073548	PASS
RHAG	6	49605158	49636884	0.048820648	PASS
RHBDD1	2	226835581	226999215	0.025928102	PASS
RHBDD2	7	75842602	75888926	-0.262778904	PASS
RHBDD3	22	29259852	29268209	-0.575930959	PASS
RHBDF1	16	58059	76355	0.069936844	PASS
RHBDF2	17	76470891	76501790	0.088516311	PASS
RHBDL1	16	675666	678268	0.069936844	PASS
RHBDL2	1	38885807	38941799	0.033763446	PASS
RHBDL3	17	32266176	32324661	0.060555011	PASS
RHBG	1	156369212	156385219	0.103015788	PASS
RHCE	1	25362249	25430192	0.033763446	PASS
RHCG	15	89471398	89496613	-0.697600509	PASS
RHD	1	25272393	25330445	0.033763446	PASS
RHEB	7	151466012	151520120	-0.388946609	PASS

RHEBL1	12	49064685	49070025	-0.202191512	PASS
RHEX	1	206052723	206102459	-0.256698369	PASS
RHNO1	12	2876258	2889523	-0.086128028	PASS
RHO	3	129528640	129535169	0.313454204	suppress_segment_too_large
RHOA	3	49359145	49412998	-0.234617007	PASS
RHOB	2	20447074	20449445	0.040498426	PASS
RHOBTB1	10	60869438	61001440	0.072405807	PASS
RHOBTB2	8	22987417	23020199	-0.345962679	PASS
RHOBTB3	5	95713522	95824383	-0.293443042	PASS
RHOC	1	112701106	112707434	0.033763446	PASS
RHOD	11	67056818	67072013	0.182439925	suppress_segment_too_large
RHOF	12	121777754	121803403	0.202141228	PASS
RHOG	11	3826978	3840983	-0.333835828	PASS
RHOH	4	40191053	40246967	-0.011382299	PASS
RHOJ	14	63204114	63293219	0.060212819	PASS
RHOQ	2	46541806	46583121	0.040498426	PASS
RHOT1	17	32142454	32253374	0.060555011	PASS
RHOT2	16	668086	674174	0.069936844	PASS
RHOU	1	228735077	228746669	-0.256698369	PASS
RHOV	15	40872214	40874289	-0.396255771	PASS
RHOXF1	23	120109053	120115937	-0.012567603	PASS
RHOXF2	23	120158561	120165630	-0.012567603	PASS
RHOXF2B	23	120070672	120077705	-0.012567603	PASS
RHPN1	8	143368887	143384220	0.307788765	PASS
RHPN2	19	32978593	33064888	0.093487731	suppress_segment_too_large
RIBC1	23	53422690	53431120	-0.012567603	PASS
RIBC2	22	45413691	45432496	-0.808211634	PASS
RIC1	9	5629025	5776557	-0.310444659	PASS
RIC3	11	8106093	8169055	-0.405444717	PASS
RIC8A	11	207511	215113	-0.333835828	PASS
RIC8B	12	106774595	106889316	0.202141228	PASS
RICTOR	5	38937919	39074408	0.055007813	PASS
RIDA	8	98102344	98117241	0.279445683	PASS
RIF1	2	151409883	151508013	-0.475215623	PASS
RIIAD1	1	151710433	151729805	0.103015788	PASS
RILP	17	1646145	1650077	0.128406487	PASS
RILPL1	12	123470054	123533718	0.202141228	PASS
RILPL2	12	123410683	123436717	0.202141228	PASS
RIMBP2	12	130396137	130716281	-0.240609783	PASS
RIMBP3	22	18605815	18611919	-0.575930959	PASS
RIMBP3B	22	21383374	21389478	-0.575930959	PASS
RIMKLA	1	42380795	42422578	0.033763446	PASS
RIMKLB	12	8681600	8783095	0.362961408	suppress_segment_too_large
RIMS1	6	71886703	72403143	-0.368590549	PASS
RIMS2	8	103500748	104256094	0.279445683	PASS
RIMS3	1	40620679	40665657	0.033763446	PASS

RIMS4	20	44751808	44810338	-0.168668583	PASS
RIN1	11	66330242	66336840	0.182439925	suppress_segment_too_large
RIN2	20	19757606	20002459	-0.168668583	PASS
RIN3	14	92513774	92688994	-0.192197564	PASS
RING1	6	33208495	33212722	0.048820648	PASS
RINL	19	38867834	38878279	0.093487731	suppress_segment_too_large
RINT1	7	105532085	105567677	-0.388946609	PASS
RIOK1	6	7389496	7418037	0.048820648	PASS
RIOK2	5	97160867	97183260	-0.293443042	PASS
RIOK3	18	23452823	23486603	-0.376397843	PASS
RIOX1	14	73490964	73493392	0.060212819	PASS
RIOX2	3	97941818	97972457	0.313454204	suppress_segment_too_large
RIPK1	6	3063991	3115187	0.048820648	PASS
RIPK2	8	89757747	89791063	0.279445683	PASS
RIPK3	14	24336021	24340045	0.060212819	PASS
RIPK4	21	41739369	41767106	-0.064425456	suppress_segment_too_large
RIPOR1	16	67518418	67546788	-0.371357732	PASS
RIPOR2	6	24804281	25042170	0.048820648	PASS
RIPOR3	20	50586108	50691528	-0.168668583	PASS
RIPPLY1	23	106900063	106903335	-0.012567603	PASS
RIPPLY2	6	83853266	83857515	-0.368590549	PASS
RIPPLY3	21	37006150	37019659	-0.064425456	suppress_segment_too_large
RIT1	1	155897808	155911404	0.103015788	PASS
RIT2	18	42743227	43115691	-0.376397843	PASS
RITA1	12	113185526	113192368	0.202141228	PASS
RLBP1	15	89209869	89221751	-0.697600509	PASS
RLF	1	40161373	40240921	0.033763446	PASS
RLIM	23	74585217	74614617	-0.012567603	PASS
RLN1	9	5334969	5339873	-0.310444659	PASS
RLN2	9	5299868	5304969	-0.310444659	PASS
RLN3	19	14028148	14031042	0.093487731	suppress_segment_too_large
RMC1	18	23503470	23531807	-0.376397843	PASS
RMDN1	8	86468257	86514357	0.279445683	PASS
RMDN2	2	37923187	38067142	0.040498426	PASS
RMDN3	15	40735884	40755851	-0.396255771	PASS
RMI1	9	83980711	84004074	0.365190713	suppress_segment_too_large
RMI2	16	11249619	11381662	0.069936844	PASS
RMND1	6	151404762	151452181	-0.035448111	PASS
RMND5A	2	86720173	86778041	0.066915641	PASS
RMND5B	5	178130996	178150565	-0.150979337	PASS
RNASE1	14	20801228	20803278	0.060212819	PASS
RNASE10	14	20505537	20511169	0.060212819	PASS
RNASE11	14	20583559	20609884	0.060212819	PASS
RNASE12	14	20590193	20590823	0.060212819	PASS
RNASE13	14	21032820	21034785	0.060212819	PASS
RNASE2	14	20955452	20956436	0.060212819	PASS

RNASE3	14	20891399	20892348	0.060212819	PASS
RNASE4	14	20684100	20701215	0.060212819	PASS
RNASE6	14	20781051	20782467	0.060212819	PASS
RNASE7	14	21042226	21044234	0.060212819	PASS
RNASE8	14	21057822	21058455	0.060212819	PASS
RNASE9	14	20556093	20560931	0.060212819	PASS
RNASEH1	2	3544793	3558616	0.040498426	PASS
RNASEH2	19	12806556	12815201	0.093487731	suppress_segment_too_large
RNASEH2	13	50909678	51024120	-0.390786075	PASS
RNASEH2	11	65714005	65720947	0.182439925	suppress_segment_too_large
RNASEK	17	7012417	7014532	0.128406487	PASS
RNASEK-(	17	7012635	7017520	0.128406487	PASS
RNASEL	1	182573634	182589256	-0.027073548	PASS
RNASET2	6	166929504	166957191	-0.035448111	PASS
RND1	12	48857145	48865898	-0.202191512	PASS
RND2	17	43025241	43032036	0.088516311	PASS
RND3	2	150468195	150539011	-0.475215623	PASS
RNF10	12	120533480	120577594	0.202141228	PASS
RNF103	2	86603393	86623866	0.066915641	PASS
RNF103-C	2	86505668	86721122	0.066915641	PASS
RNF11	1	51236271	51273455	0.033763446	PASS
RNF111	15	58865175	59097419	-0.697600509	PASS
RNF112	17	19411125	19417276	0.024610076	PASS
RNF113A	23	119870475	119871827	-0.012567603	PASS
RNF113B	13	98175785	98177265	-0.390786075	PASS
RNF114	20	49936336	49953892	-0.168668583	PASS
RNF115	1	145738868	145824077	-0.157385092	PASS
RNF121	11	71928701	71997597	0.062969153	PASS
RNF122	8	33547755	33567125	-0.345962679	PASS
RNF123	3	49689499	49721529	-0.234617007	PASS
RNF125	18	32018372	32073213	-0.376397843	PASS
RNF126	19	647526	663277	0.079632875	PASS
RNF128	23	106693794	106796993	-0.012567603	PASS
RNF13	3	149812708	149962139	0.318278996	suppress_segment_too_large
RNF130	5	179911651	180072118	-0.150979337	PASS
RNF133	7	122697712	122699156	-0.388946609	PASS
RNF135	17	30968785	30999911	0.060555011	PASS
RNF138	18	32091855	32131561	-0.376397843	PASS
RNF139	8	124474738	124487914	0.279445683	PASS
RNF14	5	141958328	141990291	-0.293443042	PASS
RNF141	11	10511678	10541230	-0.405444717	PASS
RNF144A	2	6917392	7068286	0.040498426	PASS
RNF144B	6	18387350	18468874	0.048820648	PASS
RNF145	5	159157409	159210053	-0.150979337	PASS
RNF146	6	127266610	127288567	-0.458570262	PASS
RNF148	7	122701664	122702967	-0.388946609	PASS

RNF149	2	101271219	101308701	0.048238121	PASS
RNF150	4	140859807	141212877	-0.011382299	PASS
RNF151	16	1966823	1968975	0.069936844	PASS
RNF152	18	61808063	61894247	-0.376397843	PASS
RNF157	17	76142453	76240373	0.088516311	PASS
RNF165	18	46326809	46463140	-0.376397843	PASS
RNF166	16	88696495	88706421	-0.371357732	PASS
RNF167	17	4940008	4945222	0.128406487	PASS
RNF168	3	196468783	196503768	0.326834516	PASS
RNF169	11	74748868	74842413	0.062969153	PASS
RNF17	13	24764152	24879921	-0.390786075	PASS
RNF170	8	42849637	42897290	-0.201142258	PASS
RNF175	4	153710125	153760235	-0.011382299	PASS
RNF180	5	64165844	64372869	-0.293443042	PASS
RNF181	2	85595725	85597613	0.066915641	PASS
RNF182	6	13924446	13980302	0.048820648	PASS
RNF183	9	113297093	113303376	0.365190713	suppress_segment_too_large
RNF185	22	31160183	31207019	-0.575930959	PASS
RNF186	1	19814029	19815278	0.033763446	PASS
RNF187	1	228487061	228495766	-0.256698369	PASS
RNF19A	8	100257060	100336218	0.279445683	PASS
RNF19B	1	32936445	32964685	0.033763446	PASS
RNF2	1	185045364	185102608	-0.027073548	PASS
RNF20	9	101533851	101563344	0.365190713	suppress_segment_too_large
RNF207	1	6205475	6221299	0.373964873	suppress_segment_too_large
RNF208	9	137220247	137221581	0.365190713	suppress_segment_too_large
RNF212	4	1056250	1113562	0.279089364	PASS
RNF212B	14	23185316	23273477	0.060212819	PASS
RNF213	17	80260866	80398786	0.088516311	PASS
RNF214	11	117232625	117286445	-0.356188624	PASS
RNF215	22	30368811	30421771	-0.575930959	PASS
RNF216	7	5620047	5781739	-0.69513118	PASS
RNF217	6	124962545	125092633	-0.458570262	PASS
RNF219	13	78614291	78659179	-0.390786075	PASS
RNF220	1	44405194	44651724	0.033763446	PASS
RNF222	17	8390704	8397826	0.128406487	PASS
RNF223	1	1070966	1074307	0.373964873	suppress_segment_too_large
RNF224	9	137227271	137229638	0.365190713	suppress_segment_too_large
RNF225	19	58396090	58397079	0.063781462	PASS
RNF24	20	3927309	4015582	-0.168668583	PASS
RNF25	2	218663864	218672411	0.025928102	PASS
RNF26	11	119334527	119337313	-0.356188624	PASS
RNF31	14	24146683	24160661	0.060212819	PASS
RNF32	7	156640281	156677130	-0.411415677	PASS
RNF34	12	121400041	121430623	0.202141228	PASS
RNF38	9	36336396	36487548	-0.310444659	PASS

RNF39	6	30070266	30075887	0.048820648	PASS
RNF4	4	2462220	2625320	0.279089364	PASS
RNF40	16	30761745	30776307	0.069936844	PASS
RNF41	12	56202175	56221933	-0.202191512	PASS
RNF43	17	58352500	58417595	0.088516311	PASS
RNF44	5	176526697	176538025	-0.150979337	PASS
RNF5	6	32178354	32180793	0.048820648	PASS
RNF6	13	26132115	26222493	-0.390786075	PASS
RNF7	3	141738204	141747560	-0.356957581	PASS
RNF8	6	37353972	37394738	0.048820648	PASS
RNFT1	17	59952240	59964761	0.088516311	PASS
RNFT2	12	116738178	116853631	0.202141228	PASS
RNGTT	6	88610272	88963721	-0.368590549	PASS
RNH1	11	494512	507300	-0.333835828	PASS
RNLS	10	88273864	88584530	-1.64589449	suppress_large_homdel
RNMT	18	13726660	13764558	-0.052305056	suppress_segment_too_large
RNPC3	1	103525691	103555239	0.033763446	PASS
RNPEP	1	201982372	202006147	-0.256698369	PASS
RNPEPL1	2	240565804	240581372	0.025928102	PASS
RNPS1	16	2253116	2268412	0.069936844	PASS
ROBO1	3	78597240	79767815	0.313454204	suppress_segment_too_large
ROBO2	3	75906695	77649964	-0.234617007	PASS
ROBO3	11	124865386	124881470	-0.419612865	PASS
ROBO4	11	124883691	124898500	-0.419612865	PASS
ROCK1	18	20946906	21111851	-0.376397843	PASS
ROCK2	2	11179761	11348330	0.040498426	PASS
ROGDI	16	4796968	4802950	0.069936844	PASS
ROM1	11	62611722	62615120	0.182439925	suppress_segment_too_large
ROMO1	20	35699272	35700984	-0.168668583	PASS
ROPN1	3	123968521	123992178	0.313454204	suppress_segment_too_large
ROPN1B	3	125969144	125983454	0.313454204	suppress_segment_too_large
ROPN1L	5	10441524	10472029	0.055007813	PASS
ROR1	1	63774022	64181498	0.033763446	PASS
ROR2	9	91563091	91950162	0.365190713	suppress_segment_too_large
RORA	15	60488284	61229319	-0.697600509	PASS
RORB	9	74497365	74693177	0.365190713	suppress_segment_too_large
RORC	1	151806071	151831872	0.103015788	PASS
ROS1	6	117288300	117425855	-0.368590549	PASS
RP1	8	54509422	54871720	-0.201142258	PASS
RP1L1	8	10606349	10712187	-0.345962679	PASS
RP2	23	46836940	46882358	-0.012567603	PASS
RP9	7	33094797	33109401	-0.69513118	PASS
RPA1	17	1829702	1900082	0.128406487	PASS
RPA2	1	27891524	27914746	0.033763446	PASS
RPA3	7	7636518	7718607	-0.69513118	PASS
RPA4	23	96883908	96885467	-0.012567603	PASS

RPAIN	17	5419641	5432876	0.128406487	PASS
RPAP1	15	41517176	41544269	-0.396255771	PASS
RPAP2	1	92298965	92402056	0.033763446	PASS
RPAP3	12	47661249	47706061	-0.202191512	PASS
RPE	2	210002565	210022259	0.025928102	PASS
RPE65	1	68428822	68449959	0.033763446	PASS
RPEL1	10	103245887	103248016	-0.367947924	PASS
RPF1	1	84479259	84497790	0.033763446	PASS
RPF2	6	110982015	111028263	-0.368590549	PASS
RPGR	23	38269163	38327564	-0.012567603	PASS
RPGRIP1	14	21287939	21351301	0.060212819	PASS
RPGRIP1L	16	53598153	53703938	-0.371357732	PASS
RPH3A	12	112570380	112898881	0.202141228	PASS
RPH3AL	17	212389	386254	0.128406487	PASS
RPIA	2	88691644	88750935	-0.340781246	PASS
RPL10	23	154389955	154409168	-0.012567603	PASS
RPL10A	6	35468408	35470785	0.048820648	PASS
RPL10L	14	46651010	46651821	0.060212819	PASS
RPL11	1	23691742	23696835	0.033763446	PASS
RPL12	9	127447674	127451405	0.365190713	suppress_segment_too_large
RPL13	16	89560657	89566828	-0.371357732	PASS
RPL13A	19	49487554	49492308	0.14996833	PASS
RPL14	3	40457292	40468587	-0.234617007	PASS
RPL15	3	23916545	23924374	0.311018244	suppress_segment_too_large
RPL17	18	49488453	49492523	-0.376397843	PASS
RPL17-C18	18	49481681	49492479	-0.376397843	PASS
RPL18	19	48615328	48619536	0.14996833	PASS
RPL18A	19	17859876	17864153	0.093487731	suppress_segment_too_large
RPL19	17	39200283	39204732	0.060555011	PASS
RPL21	13	27251309	27256691	-0.390786075	PASS
RPL22	1	6185020	6209389	0.373964873	suppress_segment_too_large
RPL22L1	3	170864875	170870483	0.318278996	suppress_segment_too_large
RPL23	17	38847865	38853843	0.060555011	PASS
RPL23A	17	28719393	28724359	0.060555011	PASS
RPL24	3	101681091	101686782	0.313454204	suppress_segment_too_large
RPL26	17	8377516	8383250	0.128406487	PASS
RPL26L1	5	172958729	172969771	-0.150979337	PASS
RPL27	17	42998273	43002959	0.088516311	PASS
RPL27A	11	8682411	8714759	-0.405444717	PASS
RPL28	19	55385345	55403250	0.121336758	PASS
RPL29	3	51993522	51995942	-0.234617007	PASS
RPL3	22	39312882	39320389	-0.575930959	PASS
RPL30	8	98024851	98046469	0.279445683	PASS
RPL31	2	101001715	101024032	0.048238121	PASS
RPL32	3	12834485	12841588	0.311018244	suppress_segment_too_large
RPL34	4	108620566	108630412	-0.011382299	PASS

RPL35	9	124857880	124861981	0.365190713	suppress_segment_too_large
RPL35A	3	197949980	197956610	0.326834516	PASS
RPL36	19	5674947	5691876	0.079632875	PASS
RPL36A	23	101390824	101396154	-0.012567603	PASS
RPL36A-H	23	101391011	101412297	-0.012567603	PASS
RPL36AL	14	49618519	49620685	0.060212819	PASS
RPL37	5	40825262	40835335	-0.293443042	PASS
RPL37A	2	216498189	216579180	0.025928102	PASS
RPL38	17	74203582	74210655	0.088516311	PASS
RPL39	23	119786504	119791643	-0.012567603	PASS
RPL39L	3	187120948	187180908	0.318278996	suppress_segment_too_large
RPL3L	16	1943974	1957606	0.069936844	PASS
RPL4	15	66498015	66524532	-0.697600509	PASS
RPL41	12	56116586	56117943	-0.202191512	PASS
RPL5	1	92831983	92841924	0.033763446	PASS
RPL6	12	112405190	112418838	0.202141228	PASS
RPL7	8	73290267	73295789	-0.201142258	PASS
RPL7A	9	133348214	133351426	0.365190713	suppress_segment_too_large
RPL7L1	6	42879618	42889925	0.048820648	PASS
RPL8	8	144789765	144792587	0.307788765	PASS
RPL9	4	39452521	39458949	-0.011382299	PASS
RPLP0	12	120196686	120201235	0.202141228	PASS
RPLP1	15	69452784	69456194	-0.697600509	PASS
RPLP2	11	809647	812880	-0.333835828	PASS
RPN1	3	128619970	128681075	0.313454204	suppress_segment_too_large
RPN2	20	37178410	37241623	-0.168668583	PASS
RPP14	3	58306247	58324695	-0.234617007	PASS
RPP21	6	30345131	30346884	0.048820648	PASS
RPP25	15	74954416	74957464	-0.697600509	PASS
RPP25L	9	34610486	34612104	-0.310444659	PASS
RPP30	10	90871716	90908553	-0.39678585	PASS
RPP38	10	15097180	15139818	0.039811807	PASS
RPP40	6	4994732	5004063	0.048820648	PASS
RPRD1A	18	35984387	36067576	-0.376397843	PASS
RPRD1B	20	38033546	38127780	-0.168668583	PASS
RPRD2	1	150363091	150476566	0.103015788	PASS
RPRM	2	153477338	153478808	-0.475215623	PASS
RPRML	17	46978156	46979248	0.088516311	PASS
RPS10	6	34417454	34426125	0.048820648	PASS
RPS10-NU	6	34284887	34426071	0.048820648	PASS
RPS11	19	49496365	49499689	0.14996833	PASS
RPS12	6	132814441	132817564	-0.458570262	PASS
RPS13	11	17074389	17077787	-0.405444717	PASS
RPS14	5	150442635	150449756	-0.293443042	PASS
RPS15	19	1438358	1440494	0.079632875	PASS
RPS15A	16	18781295	18790383	0.069936844	PASS

RPS16	19	39433207	39435948	0.093487731	suppress_segment_too_large
RPS17	15	82536750	82540564	-0.697600509	PASS
RPS18	6	33272010	33276510	0.048820648	PASS
RPS19	19	41859918	41872926	0.093487731	suppress_segment_too_large
RPS19BP1	22	39529093	39532855	-0.575930959	PASS
RPS2	16	1962052	1964860	0.069936844	PASS
RPS20	8	56067295	56074581	-0.201142258	PASS
RPS21	20	62387116	62388520	-0.168668583	PASS
RPS23	5	82273358	82278577	-0.293443042	PASS
RPS24	10	78033732	78056813	0.072405807	PASS
RPS25	11	119015712	119018691	-0.356188624	PASS
RPS26	12	56041351	56044697	-0.202191512	PASS
RPS27	1	153990755	153992155	0.103015788	PASS
RPS27A	2	55231903	55235853	0.040498426	PASS
RPS27L	15	63125872	63158021	-0.697600509	PASS
RPS28	19	8321158	8323340	0.079632875	PASS
RPS29	14	49570984	49599164	0.060212819	PASS
RPS3	11	75399486	75422280	0.062969153	PASS
RPS3A	4	151099573	151104652	-0.011382299	PASS
RPS4X	23	72255679	72277300	-0.012567603	PASS
RPS5	19	58386400	58394806	0.063781462	PASS
RPS6	9	19375715	19380254	-0.310444659	PASS
RPS6KA1	1	26529761	26575030	0.033763446	PASS
RPS6KA2	6	166409364	166906451	-0.035448111	PASS
RPS6KA3	23	20149911	20267519	-0.012567603	PASS
RPS6KA4	11	64359148	64372215	0.182439925	suppress_segment_too_large
RPS6KA5	14	90847862	91060636	-0.192197564	PASS
RPS6KA6	23	84058346	84187907	-0.012567603	PASS
RPS6KB1	17	59893046	59950564	0.088516311	PASS
RPS6KB2	11	67428460	67435408	0.182439925	suppress_segment_too_large
RPS6KC1	1	213051233	213274773	-0.256698369	PASS
RPS6KL1	14	74903954	74923396	-0.192197564	PASS
RPS7	2	3575205	3580920	0.040498426	PASS
RPS8	1	44775251	44778779	0.033763446	PASS
RPS9	19	54200742	54249003	0.206912349	PASS
RPSA	3	39406689	39412542	-0.234617007	PASS
RPTN	1	152153595	152159228	0.103015788	PASS
RPTOR	17	80544819	80966371	0.088516311	PASS
RPUSD1	16	784974	788397	0.069936844	PASS
RPUSD2	15	40569300	40574943	-0.396255771	PASS
RPUSD3	3	9837849	9844602	0.311018244	suppress_segment_too_large
RPUSD4	11	126202094	126211692	-0.419612865	PASS
RRAD	16	66921679	66925644	-0.371357732	PASS
RRAGA	9	19049395	19050983	-0.310444659	PASS
RRAGB	23	55717739	55758774	-0.012567603	PASS
RRAGC	1	38838198	38859823	0.033763446	PASS

RRAGD	6	89364636	89412270	-0.368590549	PASS
RRAS	19	49635292	49640201	0.14996833	PASS
RRAS2	11	14277926	14364506	-0.405444717	PASS
RRBP1	20	17613678	17682295	-0.168668583	PASS
RREB1	6	7107597	7251980	0.048820648	PASS
RRH	4	109827994	109844604	-0.011382299	PASS
RRM1	11	4094707	4138876	-0.333835828	PASS
RRM2	2	10120698	10131419	0.040498426	PASS
RRM2B	8	102204502	102239118	0.279445683	PASS
RRN3	16	15060022	15094317	0.069936844	PASS
RRNAD1	1	156728442	156736960	0.103015788	PASS
RRP1	21	43789513	43805293	-0.064425456	suppress_segment_too_large
RRP12	10	97356358	97426076	-0.367947924	PASS
RRP15	1	218285287	218337983	-0.256698369	PASS
RRP1B	21	43659548	43696079	-0.064425456	suppress_segment_too_large
RRP36	6	43021645	43034156	0.048820648	PASS
RRP7A	22	42509968	42519802	-0.575930959	PASS
RRP8	11	6595075	6603620	-0.405444717	PASS
RRP9	3	51933430	51941941	-0.234617007	PASS
RRS1	8	66429028	66430733	-0.201142258	PASS
RS1	23	18639910	18672109	-0.012567603	PASS
RSAD1	17	50478800	50485975	0.088516311	PASS
RSAD2	2	6865806	6898239	0.040498426	PASS
RSBN1	1	113761832	113812476	0.033763446	PASS
RSBN1L	7	77696443	77783022	-0.262778904	PASS
RSC1A1	1	15659869	15661722	0.033763446	PASS
RSF1	11	77659996	77821017	0.062969153	PASS
RSL1D1	16	11833850	11851585	0.069936844	PASS
RSL24D1	15	55180806	55197067	-0.697600509	PASS
RSPH1	21	42472486	42496354	-0.064425456	suppress_segment_too_large
RSPH10B	7	5925550	5970683	-0.69513118	PASS
RSPH10B2	7	6754109	6799365	-0.69513118	PASS
RSPH14	22	23059415	23145021	-0.575930959	PASS
RSPH3	6	158972871	159000187	-0.035448111	PASS
RSPH4A	6	116616479	116632985	-0.368590549	PASS
RSPH6A	19	45795710	45815319	0.14996833	PASS
RSPH9	6	43645046	43672599	0.048820648	PASS
RSPO1	1	37611350	37634923	0.033763446	PASS
RSPO2	8	107899316	108083648	0.279445683	PASS
RSPO3	6	127118604	127197765	-0.458570262	PASS
RSPO4	20	958452	1002264	-0.168668583	PASS
RSPRY1	16	57186137	57240475	-0.371357732	PASS
RSRC1	3	158105855	158545730	0.318278996	suppress_segment_too_large
RSRC2	12	122503458	122527000	0.202141228	PASS
RSRP1	1	25242237	25338213	0.033763446	PASS
RSU1	10	16590611	16817528	0.039811807	PASS

RTBDN	19	12825478	12835428		0.093487731	suppress_segment_too_large
RTCA	1	100266207	100292769		0.033763446	PASS
RTCB	22	32387582	32412255		-0.575930959	PASS
RTEL1	20	63657810	63696253		-0.168668583	PASS
RTEL1-TN	20	63659300	63698684		-0.168668583	PASS
RTF1	15	41408408	41483563		-0.396255771	PASS
RTF2	20	56468585	56519449		-0.168668583	PASS
RTKN	2	74425836	74442422		-0.372959126	PASS
RTKN2	10	62183035	62268707		0.072405807	PASS
RTL1	14	100879753	100903722		-0.192197564	PASS
RTL10	22	19846138	19854896		-0.575930959	PASS
RTL3	23	78656069	78659328		-0.012567603	PASS
RTL4	23	112454500	112457112		-0.012567603	PASS
RTL5	23	72127110	72131901		-0.012567603	PASS
RTL6	22	44492572	44498298		-0.808211634	PASS
RTL8A	23	135050932	135052196		-0.012567603	PASS
RTL8B	23	135020504	135022529		-0.012567603	PASS
RTL8C	23	135032366	135033546		-0.012567603	PASS
RTL9	23	110358816	110456334		-0.012567603	PASS
RTN1	14	59595976	59870966		0.060212819	PASS
RTN2	19	45485289	45497061		0.14996833	PASS
RTN3	11	63681446	63759891		0.182439925	suppress_segment_too_large
RTN4	2	54972187	55112621		0.040498426	PASS
RTN4IP1	6	106571971	106629487		-0.368590549	PASS
RTN4R	22	20241415	20283246		-0.575930959	PASS
RTN4RL1	17	1934677	2025345		0.128406487	PASS
RTN4RL2	11	57460549	57477534		-0.129266491	PASS
RTP1	3	187197486	187201465		0.318278996	suppress_segment_too_large
RTP2	3	187698259	187702557		0.318278996	suppress_segment_too_large
RTP3	3	46497491	46500949		-0.234617007	PASS
RTP4	3	187368332	187372076		0.318278996	suppress_segment_too_large
RTRAF	14	51989475	52010691		0.060212819	PASS
RTTN	18	70003031	70205945		-0.376397843	PASS
RUBCN	3	197671393	197749727		0.326834516	PASS
RUBCNL	13	46342000	46438190		-0.390786075	PASS
RUFY1	5	179550558	179610026		-0.150979337	PASS
RUFY2	10	68341107	68407294		0.072405807	PASS
RUFY3	4	70704204	70807315		-0.011382299	PASS
RUFY4	2	218034960	218090581		0.025928102	PASS
RUNDC1	17	42980565	42993690		0.088516311	PASS
RUNDC3A	17	44308413	44318671		0.088516311	PASS
RUNDC3B	7	87627548	87832296		-0.262778904	PASS
RUNX1	21	34787801	36004667		-0.064425456	suppress_segment_too_large
RUNX1T1	8	91954967	92103286		0.279445683	PASS
RUNX2	6	45328157	45664349		0.048820648	PASS
RUNX3	1	24899511	24965121		0.033763446	PASS

RUSC1	1	155320896	155331114	0.103015788	PASS
RUSC2	9	35490127	35561898	-0.310444659	PASS
RUVBL1	3	128064778	128153914	0.313454204	suppress_segment_too_large
RUVBL2	19	48993448	49015995	0.14996833	PASS
RWDD1	6	116571367	116597675	-0.368590549	PASS
RWDD2A	6	83193379	83198932	-0.368590549	PASS
RWDD2B	21	29004384	29019378	0.12860881	suppress_segment_too_large
RWDD3	1	95234155	95247225	0.033763446	PASS
RWDD4	4	183639635	183659225	-0.462744716	PASS
RXFP1	4	158315311	158653372	-0.011382299	PASS
RXFP2	13	31739542	31803388	-0.390786075	PASS
RXFP3	5	33936386	33938237	0.055007813	PASS
RXFP4	1	155941710	155942949	0.103015788	PASS
RXRA	9	134317098	134440585	0.365190713	suppress_segment_too_large
RXRB	6	33193588	33200688	0.048820648	PASS
RXRG	1	165400917	165445355	-0.027073548	PASS
RXYLT1	12	63779803	63809558	-0.202191512	PASS
RYBP	3	72371825	72446621	-0.234617007	PASS
RYK	3	134065303	134250744	0.313454204	suppress_segment_too_large
RYR1	19	38433699	38587564	0.093487731	suppress_segment_too_large
RYR2	1	237042205	237833988	-0.648598641	PASS
RYR3	15	33310945	33866121	-0.396255771	PASS
S100A1	1	153627926	153632039	0.103015788	PASS
S100A10	1	151982915	151994390	0.103015788	PASS
S100A11	1	152032506	152047907	0.103015788	PASS
S100A12	1	153373706	153375649	0.103015788	PASS
S100A13	1	153618787	153631360	0.103015788	PASS
S100A14	1	153614255	153616986	0.103015788	PASS
S100A16	1	153606886	153613145	0.103015788	PASS
S100A2	1	153561108	153567890	0.103015788	PASS
S100A3	1	153547329	153549372	0.103015788	PASS
S100A4	1	153543613	153550136	0.103015788	PASS
S100A5	1	153537147	153541765	0.103015788	PASS
S100A6	1	153534599	153536244	0.103015788	PASS
S100A7	1	153457744	153460701	0.103015788	PASS
S100A7A	1	153416524	153423225	0.103015788	PASS
S100A7L2	1	153437058	153439949	0.103015788	PASS
S100A8	1	153390032	153391188	0.103015788	PASS
S100A9	1	153357854	153361027	0.103015788	PASS
S100B	21	46598962	46605208	-0.064425456	suppress_segment_too_large
S100G	23	16650158	16654670	-0.012567603	PASS
S100P	4	6693069	6697170	0.279089364	PASS
S100PBP	1	32816767	32858879	0.033763446	PASS
S100Z	5	76850001	76921650	-0.293443042	PASS
S1PR1	1	101236865	101243713	0.033763446	PASS
S1PR2	19	10221433	10231331	0.093487731	suppress_segment_too_large

S1PR3	9	88990863	89005010	0.365190713	suppress_segment_too_large
S1PR4	19	3172346	3180332	0.079632875	PASS
S1PR5	19	10512742	10517931	0.093487731	suppress_segment_too_large
SAA1	11	18266174	18269977	-0.405444717	PASS
SAA2	11	18239223	18248643	-0.405444717	PASS
SAA2-SAA	11	18231423	18248635	-0.405444717	PASS
SAA4	11	18231349	18236893	-0.405444717	PASS
SAAL1	11	18069935	18106091	-0.405444717	PASS
SAC3D1	11	65040901	65044828	0.182439925	suppress_segment_too_large
SACM1L	3	45689056	45745424	-0.234617007	PASS
SACS	13	23328826	23433740	-0.390786075	PASS
SAE1	19	47113274	47210636	0.14996833	PASS
SAFB	19	5623035	5668478	0.079632875	PASS
SAFB2	19	5586999	5624046	0.079632875	PASS
SAG	2	233307816	233347055	0.025928102	PASS
SAGE1	23	135889205	135913061	-0.012567603	PASS
SALL1	16	51135975	51151367	-0.371357732	PASS
SALL2	14	21521081	21537216	0.060212819	PASS
SALL3	18	78980275	79002677	-0.376397843	PASS
SALL4	20	51782331	51802520	-0.168668583	PASS
SAMD1	19	14087840	14091036	0.093487731	suppress_segment_too_large
SAMD10	20	63974113	63980008	-0.168668583	PASS
SAMD11	1	923928	944581	0.373964873	suppress_segment_too_large
SAMD12	8	118189455	118622112	0.279445683	PASS
SAMD13	1	84298366	84389957	0.033763446	PASS
SAMD14	17	50110040	50129882	0.088516311	PASS
SAMD15	14	77376689	77391497	-0.192197564	PASS
SAMD3	6	130144315	130365425	-0.458570262	PASS
SAMD4A	14	54567097	54793315	0.060212819	PASS
SAMD4B	19	39342396	39385710	0.093487731	suppress_segment_too_large
SAMD5	6	147508927	147737547	-0.035448111	PASS
SAMD7	3	169911572	169939175	0.318278996	suppress_segment_too_large
SAMD8	10	75099586	75182123	0.072405807	PASS
SAMD9	7	93099513	93118023	-0.262778904	PASS
SAMD9L	7	93130055	93148369	-0.262778904	PASS
SAMHD1	20	36889773	36951901	-0.168668583	PASS
SAMM50	22	43955421	44010531	-0.808211634	PASS
SAMSN1	21	14485228	14658821	-0.304975024	PASS
SAP130	2	127941217	128028120	-0.475215623	PASS
SAP18	13	21140514	21149084	-0.390786075	PASS
SAP25	7	100572228	100573820	-0.262778904	PASS
SAP30	4	173369969	173377532	-0.011382299	PASS
SAP30BP	17	75667116	75708062	0.088516311	PASS
SAP30L	5	154445957	154461054	-0.293443042	PASS
SAPCD1	6	31762799	31764851	0.048820648	PASS
SAPCD2	9	137062124	137070588	0.365190713	suppress_segment_too_large

SAR1A	10	70147289	70170523	0.072405807	PASS
SAR1B	5	134601144	134649271	-0.293443042	PASS
SARAF	8	30063012	30083208	-0.345962679	PASS
SARDH	9	133663560	133739955	0.365190713	suppress_segment_too_large
SARM1	17	28364356	28404049	0.060555011	PASS
SARNP	12	55752463	55817756	-0.202191512	PASS
SARS	1	109213918	109238169	0.033763446	PASS
SARS2	19	38915266	38930896	0.093487731	suppress_segment_too_large
SART1	11	65961689	65979828	0.182439925	suppress_segment_too_large
SART3	12	108522580	108561400	0.202141228	PASS
SASH1	6	148272304	148552050	-0.035448111	PASS
SASH3	23	129779979	129795201	-0.012567603	PASS
SASS6	1	100083563	100132955	0.033763446	PASS
SAT1	23	23783173	23786226	-0.012567603	PASS
SAT2	17	7626234	7627876	0.128406487	PASS
SATB1	3	18345387	18445588	0.311018244	suppress_segment_too_large
SATB2	2	199269500	199471266	0.025928102	PASS
SATL1	23	85092287	85243961	-0.012567603	PASS
SAV1	14	50632058	50668331	0.060212819	PASS
SAXO1	9	18858906	19049358	-0.310444659	PASS
SAXO2	15	82262810	82284930	-0.697600509	PASS
SAYSD1	6	39104064	39115189	0.048820648	PASS
SBDS	7	66987677	66995601	-0.750061359	PASS
SBF1	22	50445000	50475024	-0.808211634	PASS
SBF2	11	9778667	10294207	-0.405444717	PASS
SBK1	16	28292519	28323849	0.069936844	PASS
SBK2	19	55529733	55537089	0.121336758	PASS
SBK3	19	55540656	55545543	0.121336758	PASS
SBNO1	12	123289109	123364843	0.202141228	PASS
SBNO2	19	1107636	1174283	0.079632875	PASS
SBSN	19	35523367	35528351	0.093487731	suppress_segment_too_large
SBSPON	8	73064540	73124088	-0.201142258	PASS
SC5D	11	121292453	121308694	-0.356188624	PASS
SCAF1	19	49642125	49658642	0.14996833	PASS
SCAF11	12	45919131	45992120	-0.202191512	PASS
SCAF4	21	31671033	31732075	0.12860881	suppress_segment_too_large
SCAF8	6	154733325	154834244	-0.035448111	PASS
SCAI	9	124942608	125143506	0.365190713	suppress_segment_too_large
SCAMP1	5	78360583	78479071	-0.293443042	PASS
SCAMP2	15	74843730	74873365	-0.697600509	PASS
SCAMP3	1	155255979	155262430	0.103015788	PASS
SCAMP4	19	1905214	1926013	0.079632875	PASS
SCAMP5	15	74957219	75021496	-0.697600509	PASS
SCAND1	20	35953617	35959472	-0.168668583	PASS
SCAP	3	47413694	47477126	-0.234617007	PASS
SCAPER	15	76347904	76905444	-0.697600509	PASS

SCARA3	8	27633868	27676776	-0.345962679	PASS
SCARA5	8	27869882	27992727	-0.345962679	PASS
SCARB1	12	124776856	124882668	0.202141228	PASS
SCARB2	4	76158733	76234536	-0.011382299	PASS
SCARF1	17	1633858	1645747	0.128406487	PASS
SCARF2	22	20424815	20437826	-0.575930959	PASS
SCART1	10	133453928	133523558	0.063874692	PASS
SCCPDH	1	246724047	246768137	-0.368111568	PASS
SCD	10	100347124	100364834	-0.367947924	PASS
SCD5	4	82629539	82798857	-0.011382299	PASS
SCEL	13	77535674	77645263	-0.390786075	PASS
SCFD1	14	30622112	30735812	0.060212819	PASS
SCFD2	4	52872982	53366075	-0.011382299	PASS
SCG2	2	223596940	223602503	0.025928102	PASS
SCG3	15	51681353	51721031	-0.396255771	PASS
SCG5	15	32641676	32697098	-0.396255771	PASS
SCGB1A1	11	62405103	62423195	0.182439925	suppress_segment_too_large
SCGB1C1	11	193080	194573	-0.333835828	PASS
SCGB1C2	17	137526	139067	0.128406487	PASS
SCGB1D1	11	62190216	62193539	0.182439925	suppress_segment_too_large
SCGB1D2	11	62242210	62244808	0.182439925	suppress_segment_too_large
SCGB1D4	11	62296277	62299064	0.182439925	suppress_segment_too_large
SCGB2A1	11	62208668	62213939	0.182439925	suppress_segment_too_large
SCGB2A2	11	62270155	62273156	0.182439925	suppress_segment_too_large
SCGB2B2	19	34593329	34675699	0.093487731	suppress_segment_too_large
SCGB3A1	5	180590103	180591540	-0.150979337	PASS
SCGB3A2	5	147870682	147882191	-0.293443042	PASS
SCGN	6	25652201	25701783	0.048820648	PASS
SCHIP1	3	159839861	159897360	0.318278996	suppress_segment_too_large
SCIMP	17	5208961	5234860	0.128406487	PASS
SCIN	7	12570577	12660179	-0.69513118	PASS
SCLT1	4	128864921	129093607	-0.011382299	PASS
SCLY	2	238060889	238099413	0.025928102	PASS
SCMH1	1	41027200	41242154	0.033763446	PASS
SCML1	23	17737449	17754988	-0.012567603	PASS
SCML2	23	18239314	18354727	-0.012567603	PASS
SCML4	6	107704104	107824317	-0.368590549	PASS
SCN10A	3	38696807	38816217	-0.234617007	PASS
SCN11A	3	38845769	38950561	-0.234617007	PASS
SCN1A	2	165984641	166149214	0.039726562	PASS
SCN1B	19	35030466	35040449	0.093487731	suppress_segment_too_large
SCN2A	2	165194993	165392310	0.039726562	PASS
SCN2B	11	118161951	118176673	-0.356188624	PASS
SCN3A	2	165087522	165204067	0.039726562	PASS
SCN3B	11	123629187	123655244	-0.949752196	PASS
SCN4A	17	63938554	63972918	0.088516311	PASS

SCN4B	11	118133377	118152888		-0.356188624	PASS
SCN5A	3	38548057	38649673		-0.234617007	PASS
SCN7A	2	166403573	166494326		0.039726562	PASS
SCN8A	12	51590266	51812864		-0.202191512	PASS
SCN9A	2	166195185	166376001		0.039726562	PASS
SCNM1	1	151156664	151170297		0.103015788	PASS
SCNN1A	12	6346843	6377730		-0.086128028	PASS
SCNN1B	16	23278231	23381299		0.069936844	PASS
SCNN1D	1	1280436	1292029		0.373964873	suppress_segment_too_large
SCNN1G	16	23182715	23216883		0.069936844	PASS
SCO1	17	10672474	10698375		-0.139031004	PASS
SCO2	22	50523568	50526439		-0.808211634	PASS
SCOC	4	140257286	140385726		-0.011382299	PASS
SCP2	1	52927229	53051703		0.033763446	PASS
SCP2D1	20	18813726	18814392		-0.168668583	PASS
SCPEP1	17	56978105	57006768		0.088516311	PASS
SCRG1	4	173384701	173406380		-0.011382299	PASS
SCRIB	8	143790920	143815379		0.307788765	PASS
SCRN1	7	29920103	29990289		-0.69513118	PASS
SCRN2	17	47837692	47841333		0.088516311	PASS
SCRN3	2	174395730	174429575		0.039726562	PASS
SCRT1	8	144330565	144336281		0.307788765	PASS
SCRT2	20	661596	676179		-0.168668583	PASS
SCT	11	626431	627143		-0.333835828	PASS
SCTR	2	119439843	119525301		-0.475215623	PASS
SCUBE1	22	43197283	43343388		-0.575930959	PASS
SCUBE2	11	9019498	9138114		-0.405444717	PASS
SCUBE3	6	35214419	35253079		0.048820648	PASS
SCX	8	144266560	144268481		0.307788765	PASS
SCYL1	11	65525077	65538704		0.182439925	suppress_segment_too_large
SCYL2	12	100267140	100341724		0.202141228	PASS
SCYL3	1	169849631	169894267		-0.027073548	PASS
SDAD1	4	75940950	75990962		-0.011382299	PASS
SDC1	2	20200797	20225433		0.040498426	PASS
SDC2	8	96493351	96611780		0.279445683	PASS
SDC3	1	30869467	30908761		0.033763446	PASS
SDC4	20	45325288	45348424		-0.168668583	PASS
SDCBP	8	58552924	58582860		-0.201142258	PASS
SDCBP2	20	1309909	1329239		-0.168668583	PASS
SDCCAG8	1	243256034	243500092		-0.648598641	PASS
SDE2	1	225982702	225999331		-0.256698369	PASS
SDF2	17	28648356	28662189		0.060555011	PASS
SDF2L1	22	21642261	21644298		-0.575930959	PASS
SDF4	1	1216908	1232031		0.373964873	suppress_segment_too_large
SDHA	5	218241	256700		0.055007813	PASS
SDHAF1	19	35995199	35996315		0.093487731	suppress_segment_too_large

SDHAF2	11	61430042	61447529	0.182439925	suppress_segment_too_large
SDHAF3	7	97116590	97181763	-0.262778904	PASS
SDHAF4	6	70566917	70589569	-0.368590549	PASS
SDHB	1	17018722	17054170	0.033763446	PASS
SDHC	1	161314257	161375340	-0.027073548	PASS
SDHD	11	112086773	112120013	-0.356188624	PASS
SDK1	7	3301448	4269000	-0.69513118	PASS
SDK2	17	73334384	73644089	0.088516311	PASS
SDR16C5	8	56300010	56320776	-0.201142258	PASS
SDR39U1	14	24439766	24442905	0.060212819	PASS
SDR42E1	16	81988855	82011488	-0.371357732	PASS
SDR42E2	16	22165583	22191754	0.069936844	PASS
SDR9C7	12	56923154	56934405	-0.202191512	PASS
SDS	12	113392445	113426301	0.202141228	PASS
SDSL	12	113422237	113438276	0.202141228	PASS
SEBOX	17	28364268	28365244	0.060555011	PASS
SEC11A	15	84669538	84716716	-0.697600509	PASS
SEC11C	18	59139477	59158836	-0.376397843	PASS
SEC13	3	10293131	10321178	0.311018244	suppress_segment_too_large
SEC14L1	17	77088749	77217101	0.088516311	PASS
SEC14L2	22	30396857	30425317	-0.575930959	PASS
SEC14L3	22	30447959	30472049	-0.575930959	PASS
SEC14L4	22	30488913	30505711	-0.575930959	PASS
SEC14L5	16	4958317	5019158	0.069936844	PASS
SEC14L6	22	30522799	30546682	-0.575930959	PASS
SEC16A	9	136440096	136483759	0.365190713	suppress_segment_too_large
SEC16B	1	177923956	177984303	-0.027073548	PASS
SEC22A	3	123201927	123274130	0.313454204	suppress_segment_too_large
SEC22B	1	120150898	120176515	-0.157385092	PASS
SEC22C	3	42547969	42601080	-0.234617007	PASS
SEC23A	14	39031919	39109646	0.060212819	PASS
SEC23B	20	18507482	18561415	-0.168668583	PASS
SEC23IP	10	119892711	119944658	0.063874692	PASS
SEC24A	5	134648789	134727823	-0.293443042	PASS
SEC24B	4	109433772	109540896	-0.011382299	PASS
SEC24C	10	73744384	73772161	0.072405807	PASS
SEC24D	4	118722823	118838683	-0.011382299	PASS
SEC31A	4	82818661	82901166	-0.011382299	PASS
SEC31B	10	100486642	100519864	-0.367947924	PASS
SEC61A1	3	128051641	128071683	0.313454204	suppress_segment_too_large
SEC61A2	10	12129637	12169961	0.039811807	PASS
SEC61B	9	99222064	99230615	0.365190713	suppress_segment_too_large
SEC61G	7	54752250	54759974	-0.750061359	PASS
SEC62	3	169966635	169998373	0.318278996	suppress_segment_too_large
SEC63	6	107867756	107958189	-0.368590549	PASS
SECISBP2	9	89318506	89359662	0.365190713	suppress_segment_too_large

SECISBP2l	15	48988476	49046563	-0.396255771	PASS
SECTM1	17	82321024	82334074	0.088516311	PASS
SEH1L	18	12947133	12987536	-0.052305056	suppress_segment_too_large
SEL1L	14	81471549	81533861	-0.192197564	PASS
SEL1L2	20	13849247	13996443	-0.168668583	PASS
SEL1L3	4	25747427	25863760	-0.458181871	PASS
SELE	1	169722641	169764705	-0.027073548	PASS
SELENBP1	1	151364302	151372733	0.103015788	PASS
SELENOF	1	86862445	86914424	0.033763446	PASS
SELENOH	11	57741250	57743554	-0.129266491	PASS
SELENOI	2	26308547	26395891	0.040498426	PASS
SELENOK	3	53884410	53891988	-0.234617007	PASS
SELENOM	22	31104772	31120069	-0.575930959	PASS
SELENON	1	25800176	25818224	0.033763446	PASS
SELENOO	22	50200979	50217616	-0.808211634	PASS
SELENOP	5	42799880	42887392	-0.293443042	PASS
SELENOS	15	101270817	101277500	-0.697600509	PASS
SELENOT	3	150602875	150630445	0.318278996	suppress_segment_too_large
SELENOV	19	39515113	39520686	0.093487731	suppress_segment_too_large
SELENOW	19	47778572	47784686	0.14996833	PASS
SELL	1	169690667	169711698	-0.027073548	PASS
SELP	1	169588849	169630193	-0.027073548	PASS
SELPLG	12	108622277	108633959	0.202141228	PASS
SEM1	7	96481626	96709891	-0.262778904	PASS
SEMA3A	7	83955777	84492724	-0.262778904	PASS
SEMA3B	3	50267558	50277546	-0.234617007	PASS
SEMA3C	7	80742538	80922359	-0.262778904	PASS
SEMA3D	7	84995553	85186855	-0.262778904	PASS
SEMA3E	7	83363238	83649113	-0.262778904	PASS
SEMA3F	3	50155045	50189075	-0.234617007	PASS
SEMA3G	3	52433053	52445085	-0.234617007	PASS
SEMA4A	1	156147366	156177752	0.103015788	PASS
SEMA4B	15	90160604	90229679	-0.697600509	PASS
SEMA4C	2	96859716	96870757	0.048238121	PASS
SEMA4D	9	89360787	89498130	0.365190713	suppress_segment_too_large
SEMA4F	2	74654228	74683853	-0.372959126	PASS
SEMA4G	10	100969518	100985871	-0.367947924	PASS
SEMA5A	5	9035026	9546075	0.055007813	PASS
SEMA5B	3	122909082	123028605	0.313454204	suppress_segment_too_large
SEMA6A	5	116443616	116574934	-0.293443042	PASS
SEMA6B	19	4542593	4559808	0.079632875	PASS
SEMA6C	1	151131685	151146664	0.103015788	PASS
SEMA6D	15	47184101	47774223	-0.396255771	PASS
SEMA7A	15	74409289	74433958	-0.697600509	PASS
SEMG1	20	45206997	45209772	-0.168668583	PASS
SEMG2	20	45221300	45224458	-0.168668583	PASS

SENP1	12	48042898	48106308	-0.202191512	PASS
SENP2	3	185582496	185633551	0.318278996	suppress_segment_too_large
SENP3	17	7561875	7571969	0.128406487	PASS
SENP3-EIF	17	7563287	7578715	0.128406487	PASS
SENP5	3	196867856	196934714	0.326834516	PASS
SENP6	6	75601509	75718278	-0.368590549	PASS
SENP7	3	101324205	101513241	0.313454204	suppress_segment_too_large
SENP8	15	72114258	72143688	-0.697600509	PASS
SEPHS1	10	13317424	13348298	0.039811807	PASS
SEPHS2	16	30443631	30446181	0.069936844	PASS
SEPSECS	4	25120014	25160442	0.279089364	PASS
1-Sep	16	30378133	30395991	0.069936844	PASS
10-Sep	2	109542982	109614206	-0.010901494	PASS
11-Sep	4	76949703	77040384	-0.011382299	PASS
12-Sep	16	4777669	4788521	0.069936844	PASS
14-Sep	7	55793544	55862789	-0.750061359	PASS
3-Sep	22	41969475	41998221	-0.575930959	PASS
4-Sep	17	58520250	58540818	0.088516311	PASS
5-Sep	22	19714464	19724224	-0.575930959	PASS
SEPT5-GP	22	19717220	19724772	-0.575930959	PASS
6-Sep	23	119615724	119693370	-0.012567603	PASS
7-Sep	7	35800932	35907105	-0.69513118	PASS
8-Sep	5	132750817	132807241	-0.293443042	PASS
9-Sep	17	77280569	77500596	0.088516311	PASS
SERAC1	6	158109515	158168280	-0.035448111	PASS
SERBP1	1	67407810	67430415	0.033763446	PASS
SERF1B	5	70025247	70043113	-0.293443042	PASS
SERF2	15	43777087	43802589	-0.396255771	PASS
SERGEF	11	17788048	18013162	-0.405444717	PASS
SERHL2	22	42553617	42574382	-0.575930959	PASS
SERINC1	6	122443354	122471822	-0.368590549	PASS
SERINC2	1	31409565	31434680	0.033763446	PASS
SERINC3	20	44496221	44522109	-0.168668583	PASS
SERINC4	15	43794162	43800221	-0.396255771	PASS
SERINC5	5	80111651	80256079	-0.293443042	PASS
SERP1	3	150541994	150603228	0.318278996	suppress_segment_too_large
SERP2	13	44373665	44397714	-0.390786075	PASS
SERPINA1	14	94376747	94390693	-0.192197564	PASS
SERPINA1	14	94280455	94293271	-0.192197564	PASS
SERPINA1	14	94442464	94452790	-0.192197564	PASS
SERPINA1	14	94487274	94517844	-0.192197564	PASS
SERPINA3	14	94612377	94624055	-0.192197564	PASS
SERPINA4	14	94561091	94569913	-0.192197564	PASS
SERPINA5	14	94561442	94593120	-0.192197564	PASS
SERPINA6	14	94304248	94323394	-0.192197564	PASS
SERPINA7	23	106032442	106038738	-0.012567603	PASS

SERPINA9	14	94462717	94479689	-0.192197564	PASS
SERPINB1	6	2832332	2842006	0.048820648	PASS
SERPINB1	18	63897174	63936111	-0.376397843	PASS
SERPINB1	18	63647579	63726432	-0.376397843	PASS
SERPINB1	18	63556160	63567011	-0.376397843	PASS
SERPINB1	18	63586989	63604639	-0.376397843	PASS
SERPINB2	18	63871692	63903890	-0.376397843	PASS
SERPINB3	18	63655197	63661963	-0.376397843	PASS
SERPINB4	18	63637259	63644298	-0.376397843	PASS
SERPINB5	18	63476761	63505085	-0.376397843	PASS
SERPINB6	6	2948159	2972165	0.048820648	PASS
SERPINB7	18	63752935	63805376	-0.376397843	PASS
SERPINB8	18	63969925	64019779	-0.376397843	PASS
SERPINB9	6	2887266	2903280	0.048820648	PASS
SERPINC1	1	173903804	173917378	-0.027073548	PASS
SERPIND1	22	20773879	20787720	-0.575930959	PASS
SERPINE1	7	101127089	101139266	-0.388946609	PASS
SERPINE2	2	223975112	224039319	0.025928102	PASS
SERPINE3	13	51335773	51364735	-0.390786075	PASS
SERPINF1	17	1761959	1777574	0.128406487	PASS
SERPINF2	17	1742836	1755268	0.128406487	PASS
SERPING1	11	57597387	57614853	-0.129266491	PASS
SERPINH1	11	75562056	75572783	0.062969153	PASS
SERPINI1	3	167735243	167825568	0.318278996	suppress_segment_too_large
SERPINI2	3	167441789	167479004	0.318278996	suppress_segment_too_large
SERTAD1	19	40421592	40426025	0.093487731	suppress_segment_too_large
SERTAD2	2	64631621	64751005	-0.372959126	PASS
SERTAD3	19	40440844	40444705	0.093487731	suppress_segment_too_large
SERTAD4	1	210232799	210246631	-0.256698369	PASS
SERTM1	13	36673912	36697839	-0.390786075	PASS
SESN1	6	108986437	109094819	-0.368590549	PASS
SESN2	1	28259527	28282491	0.033763446	PASS
SESN3	11	95165513	95232541	0.062969153	PASS
SESTD1	2	179101692	179264790	0.039726562	PASS
SET	9	128683424	128696400	0.365190713	suppress_segment_too_large
SETBP1	18	44680173	45068510	-0.376397843	PASS
SETD1A	16	30957294	30984664	0.069936844	PASS
SETD1B	12	121804180	121832584	0.202141228	PASS
SETD2	3	47016429	47163967	-0.234617007	PASS
SETD3	14	99397746	99480889	-0.192197564	PASS
SETD4	21	36034541	36079389	-0.064425456	suppress_segment_too_large
SETD5	3	9397615	9479240	0.311018244	suppress_segment_too_large
SETD6	16	58515479	58521181	-0.371357732	PASS
SETD7	4	139495941	139606699	-0.011382299	PASS
SETD9	5	56909260	56925532	-0.293443042	PASS
SETDB1	1	150926263	150964744	0.103015788	PASS

SETDB2	13	49444374	49495003		-0.390786075	PASS
SETMAR	3	4303304	4317567		0.311018244	suppress_segment_too_large
SETSIP	1	92074533	92075441		0.033763446	PASS
SETX	9	132261356	132354985		0.365190713	suppress_segment_too_large
SEZ6	17	28954901	29006440		0.060555011	PASS
SEZ6L	22	26169474	26383597		-0.575930959	PASS
SEZ6L2	16	29871159	29899547		0.069936844	PASS
SF1	11	64764606	64778786		0.182439925	suppress_segment_too_large
SF3A1	22	30331988	30356947		-0.575930959	PASS
SF3A2	19	2236504	2248679		0.079632875	PASS
SF3A3	1	37956975	37990921		0.033763446	PASS
SF3B1	2	197389784	197435091		0.025928102	PASS
SF3B2	11	66050729	66069308		0.182439925	suppress_segment_too_large
SF3B3	16	70523788	70577670		-0.371357732	PASS
SF3B4	1	149923317	149928344		0.103015788	PASS
SF3B5	6	144094881	144095573		-0.035448111	PASS
SF3B6	2	24067584	24076443		0.040498426	PASS
SFI1	22	31488688	31618586		-0.575930959	PASS
SFMBT1	3	52903572	53046750		-0.234617007	PASS
SFMBT2	10	7158624	7411486		0.039811807	PASS
SFN	1	26863138	26864457		0.033763446	PASS
SFPQ	1	35176378	35193148		0.033763446	PASS
SFR1	10	104122058	104126385		-0.367947924	PASS
SFRP1	8	41261958	41309497		-0.201142258	PASS
SFRP2	4	153780592	153789120		-0.011382299	PASS
SFRP4	7	37905932	38025695		-0.69513118	PASS
SFRP5	10	97766751	97771952		-0.367947924	PASS
SFSWAP	12	131711081	131799737		0.270848837	PASS
SFT2D1	6	166319728	166342591		-0.035448111	PASS
SFT2D2	1	168225938	168253025		-0.027073548	PASS
SFT2D3	2	127701508	127705242		-0.475215623	PASS
SFTA2	6	30931353	30955636		0.048820648	PASS
SFTA3	14	36473288	36513829		0.060212819	PASS
SFTPA1	10	79610939	79615455		0.072405807	PASS
SFTPA2	10	79555852	79560402		0.072405807	PASS
SFTPB	2	85657314	85668741		0.066915641	PASS
SFTPC	8	22156913	22164479		-0.345962679	PASS
SFTPD	10	79937740	79982614		0.072405807	PASS
SFXN1	5	175477062	175529742		-0.150979337	PASS
SFXN2	10	102714538	102743492		-0.367947924	PASS
SFXN3	10	101031234	101041244		-0.367947924	PASS
SFXN4	10	119140767	119165667		0.063874692	PASS
SFXN5	2	72942036	73075619		-0.372959126	PASS
SGCA	17	50164214	50175931		0.088516311	PASS
SGCB	4	52020706	52038482		-0.011382299	PASS
SGCD	5	155870344	156767788		-0.293443042	PASS

SGCE	7	94524204	94656572	-0.262778904	PASS
SGCG	13	23180952	23325165	-0.390786075	PASS
SGCZ	8	14089864	15238339	-0.345962679	PASS
SGF29	16	28553915	28591790	0.069936844	PASS
SGIP1	1	66533383	66748299	0.033763446	PASS
SGK1	6	134169246	134318112	-0.035448111	PASS
SGK2	20	43558968	43588237	-0.168668583	PASS
SGK3	8	66667596	66862022	-0.201142258	PASS
SGK494	17	28607964	28614200	0.060555011	PASS
SGMS1	10	50305586	50625163	-0.404852538	PASS
SGMS2	4	107824563	107915047	-0.011382299	PASS
SGO1	3	20160593	20186292	0.311018244	suppress_segment_too_large
SGO2	2	200510008	200583782	0.025928102	PASS
SGPL1	10	70815961	70881173	0.072405807	PASS
SGPP1	14	63684214	63728039	0.060212819	PASS
SGPP2	2	222424517	222560948	0.025928102	PASS
SGSH	17	80206716	80220923	0.088516311	PASS
SGSM1	22	24806169	24927578	-0.575930959	PASS
SGSM2	17	2337498	2381058	0.128406487	PASS
SGSM3	22	40370591	40410289	-0.575930959	PASS
SGTA	19	2754714	2783371	0.079632875	PASS
SGTB	5	65665928	65723035	-0.293443042	PASS
SH2B1	16	28846600	28874212	0.069936844	PASS
SH2B2	7	102285091	102321711	-0.388946609	PASS
SH2B3	12	111405948	111451623	0.202141228	PASS
SH2D1A	23	124227868	124373197	-0.012567603	PASS
SH2D1B	1	162395266	162412138	-0.027073548	PASS
SH2D2A	1	156806243	156816862	0.103015788	PASS
SH2D3A	19	6752160	6767588	0.079632875	PASS
SH2D3C	9	127738317	127778741	0.365190713	suppress_segment_too_large
SH2D4A	8	19313617	19396218	-0.345962679	PASS
SH2D4B	10	80537902	80646560	0.072405807	PASS
SH2D5	1	20719732	20732837	0.033763446	PASS
SH2D6	2	85418721	85437029	0.066915641	PASS
SH2D7	15	78077808	78104909	-0.697600509	PASS
SH3BGR	21	39445855	39515506	-0.064425456	suppress_segment_too_large
SH3BGRL	23	81201943	81298547	-0.012567603	PASS
SH3BGRL	6	79631283	79703659	-0.368590549	PASS
SH3BGRL	1	26279176	26281522	0.033763446	PASS
SH3BP1	22	37634654	37656119	-0.575930959	PASS
SH3BP2	4	2793023	2841098	0.279089364	PASS
SH3BP4	2	234951973	235055714	0.025928102	PASS
SH3BP5	3	15254853	15341368	0.311018244	suppress_segment_too_large
SH3BP5L	1	248810446	248826633	-0.368111568	PASS
SH3D19	4	151102751	151325632	-0.011382299	PASS
SH3D21	1	36306387	36324886	0.033763446	PASS

SH3GL1	19	4360370	4400547	0.079632875	PASS
SH3GL2	9	17579082	17797129	-0.310444659	PASS
SH3GL3	15	83447228	83618743	-0.697600509	PASS
SH3GLB1	1	86704570	86748184	0.033763446	PASS
SH3GLB2	9	129007036	129028303	0.365190713	suppress_segment_too_large
SH3KBP1	23	19533975	19887601	-0.012567603	PASS
SH3PXD2/	10	103594027	103855543	-0.367947924	PASS
SH3PXD2I	5	172325000	172454523	-0.150979337	PASS
SH3RF1	4	169094256	169271105	-0.011382299	PASS
SH3RF2	5	145936579	146081791	-0.293443042	PASS
SH3RF3	2	109129348	109504632	-0.010901494	PASS
SH3TC1	4	8182072	8241803	0.279089364	PASS
SH3TC2	5	148923639	149063163	-0.293443042	PASS
SH3YL1	2	217730	266398	0.040498426	PASS
SHANK1	19	50661827	50719450	-0.016761391	PASS
SHANK2	11	70467856	71252577	0.182439925	suppress_segment_too_large
SHANK3	22	50674415	50733298	-0.808211634	PASS
SHARPIN	8	144098633	144108124	0.307788765	PASS
SHB	9	37919134	38069211	-0.310444659	PASS
SHBG	17	7613946	7633383	0.128406487	PASS
SHC1	1	154962298	154974395	0.103015788	PASS
SHC2	19	416583	460996	0.079632875	PASS
SHC3	9	89005771	89178767	0.365190713	suppress_segment_too_large
SHC4	15	48823735	48963444	-0.396255771	PASS
SHCBP1	16	46580554	46621626	0.069936844	PASS
SHCBP1L	1	182899865	182953525	-0.027073548	PASS
SHD	19	4278601	4290724	0.079632875	PASS
SHE	1	154469772	154502113	0.103015788	PASS
SHF	15	45167214	45201175	-0.396255771	PASS
SHH	7	155799986	155812273	-0.411415677	PASS
SHISA2	13	26044597	26051031	-0.390786075	PASS
SHISA3	4	42397839	42402487	-0.011382299	PASS
SHISA4	1	201888680	201892306	-0.256698369	PASS
SHISA5	3	48467798	48504826	-0.234617007	PASS
SHISA6	17	11241263	11564063	-0.139031004	PASS
SHISA7	19	55428740	55442863	0.121336758	PASS
SHISA8	22	41909554	41914667	-0.575930959	PASS
SHISA9	16	12901620	13240413	0.069936844	PASS
SHISAL1	22	44243667	44312851	-0.808211634	PASS
SHISAL2A	1	52633344	52669683	0.033763446	PASS
SHISAL2B	5	64690308	64718090	-0.293443042	PASS
SHKBP1	19	40576851	40591399	0.093487731	suppress_segment_too_large
SHLD1	20	5750393	5863912	-0.168668583	PASS
SHLD2	10	87094161	87191468	-0.292453993	PASS
SHLD3	5	65624765	65630891	-0.293443042	PASS
SHMT1	17	18327860	18363563	0.024610076	PASS

SHMT2	12	57229327	57234935	-0.202191512	PASS
SHOC2	10	110919547	111013667	0.063874692	PASS
SHOX	23	624344	659411	-0.012567603	PASS
SHOX2	3	158095954	158106503	0.318278996	suppress_segment_too_large
SHPK	17	3608262	3636322	0.128406487	PASS
SHPRH	6	145864245	145964423	-0.035448111	PASS
SHQ1	3	72749277	72861914	-0.234617007	PASS
SHROOM1	5	132822141	132830898	-0.293443042	PASS
SHROOM2	23	9786456	9949443	-0.012567603	PASS
SHROOM3	4	76435100	76783253	-0.011382299	PASS
SHROOM4	23	50591647	50814302	-0.012567603	PASS
SHTN1	10	116881482	117126586	0.063874692	PASS
SI	3	164978898	165078495	0.318278996	suppress_segment_too_large
SIAE	11	124633113	124695707	-0.419612865	PASS
SIAH1	16	48356364	48448402	0.069936844	PASS
SIAH2	3	150741127	150763477	0.318278996	suppress_segment_too_large
SIAH3	13	45777243	45851736	-0.390786075	PASS
SIDT1	3	113532296	113629578	0.313454204	suppress_segment_too_large
SIDT2	11	117178733	117197445	-0.356188624	PASS
SIGIRR	11	405716	417455	-0.333835828	PASS
SIGLEC1	20	3686970	3707128	-0.168668583	PASS
SIGLEC10	19	51410021	51417803	-0.016761391	PASS
SIGLEC11	19	49948985	49961172	-0.016761391	PASS
SIGLEC12	19	51491357	51501789	-0.016761391	PASS
SIGLEC14	19	51642553	51646801	-0.016761391	PASS
SIGLEC15	18	45825512	45844080	-0.376397843	PASS
SIGLEC5	19	51611927	51645545	-0.016761391	PASS
SIGLEC6	19	51519525	51531856	-0.016761391	PASS
SIGLEC7	19	51142299	51153526	-0.016761391	PASS
SIGLEC8	19	51450847	51458456	-0.016761391	PASS
SIGLEC9	19	51124908	51136651	-0.016761391	PASS
SIGLECL1	19	51246348	51269330	-0.016761391	PASS
SIGMAR1	9	34634722	34637809	-0.310444659	PASS
SIK1B	21	6111134	6123739	-0.304975024	PASS
SIK2	11	111602391	111730853	-0.356188624	PASS
SIK3	11	116843402	117098437	-0.356188624	PASS
SIKE1	1	114769479	114780685	0.033763446	PASS
SIL1	5	138946720	139293557	-0.293443042	PASS
SIM1	6	100385015	100464929	-0.368590549	PASS
SIM2	21	36699133	36749917	-0.064425456	suppress_segment_too_large
SIMC1	5	176238367	176345991	-0.150979337	PASS
SIN3A	15	75369379	75455842	-0.697600509	PASS
SIN3B	19	16829400	16880353	0.093487731	suppress_segment_too_large
SINHCAF	12	31280584	31327058	0.362961408	suppress_segment_too_large
SIPA1	11	65638097	65650930	0.182439925	suppress_segment_too_large
SIPA1L1	14	71320449	71741229	0.060212819	PASS

SIPA1L2	1	232397965	232561558	-0.256698369	PASS
SIPA1L3	19	37907228	38208372	0.093487731	suppress_segment_too_large
SIRPA	20	1894167	1940592	-0.168668583	PASS
SIRPB1	20	1563521	1620061	-0.168668583	PASS
SIRPB2	20	1470741	1491587	-0.168668583	PASS
SIRPD	20	1534251	1558843	-0.168668583	PASS
SIRPG	20	1629152	1657779	-0.168668583	PASS
SIRT1	10	67884669	67918390	0.072405807	PASS
SIRT2	19	38878555	38899862	0.093487731	suppress_segment_too_large
SIRT3	11	215458	236931	-0.333835828	PASS
SIRT4	12	120302316	120313249	0.202141228	PASS
SIRT5	6	13574529	13615158	0.048820648	PASS
SIRT6	19	4174109	4182604	0.079632875	PASS
SIRT7	17	81911939	81921323	0.088516311	PASS
SIT1	9	35649295	35650950	-0.310444659	PASS
SIVA1	14	104753100	104768494	-0.192197564	PASS
SIX1	14	60643415	60658259	0.060212819	PASS
SIX2	2	45005161	45009430	0.040498426	PASS
SIX3	2	44941898	44946077	0.040498426	PASS
SIX4	14	60709528	60724348	0.060212819	PASS
SIX5	19	45764785	45769226	0.14996833	PASS
SIX6	14	60508951	60512850	0.060212819	PASS
SKA1	18	50374995	50394173	-0.376397843	PASS
SKA2	17	59109951	59155269	0.088516311	PASS
SKA3	13	21153595	21176602	-0.390786075	PASS
SKAP1	17	48133440	48430275	0.088516311	PASS
SKAP2	7	26667062	26995239	-0.69513118	PASS
SKI	1	2228695	2310119	0.373964873	suppress_segment_too_large
SKIDA1	10	21513478	21526368	0.039811807	PASS
SKIL	3	170357678	170396835	0.318278996	suppress_segment_too_large
SKIV2L	6	31959080	31969755	0.048820648	PASS
SKOR1	15	67819704	67834561	-0.697600509	PASS
SKOR2	18	47206322	47251603	-0.376397843	PASS
SKP1	5	134148935	134177038	-0.293443042	PASS
SKP2	5	36151989	36184319	0.055007813	PASS
SLA	8	133036724	133103054	0.113969953	PASS
SLA2	20	36612318	36646216	-0.168668583	PASS
SLAIN1	13	77697854	77764242	-0.390786075	PASS
SLAIN2	4	48341322	48426212	-0.011382299	PASS
SLAMF1	1	160608100	160647295	-0.027073548	PASS
SLAMF6	1	160485030	160523262	-0.027073548	PASS
SLAMF7	1	160739057	160754821	-0.027073548	PASS
SLAMF8	1	159826750	159837249	-0.467855874	PASS
SLAMF9	1	159951492	159954254	-0.027073548	PASS
SLBP	4	1692800	1712555	0.279089364	PASS
SLC10A1	14	69775417	69797289	0.060212819	PASS

SLC10A2	13	103043998	103066846	-0.390786075	PASS
SLC10A3	23	154487306	154490690	-0.012567603	PASS
SLC10A4	4	48483343	48489196	-0.011382299	PASS
SLC10A5	8	81693607	81696174	0.279445683	PASS
SLC10A6	4	86823468	86849263	-0.011382299	PASS
SLC10A7	4	146253975	146521964	-0.011382299	PASS
SLC11A1	2	218382029	218396894	0.025928102	PASS
SLC11A2	12	50979401	51028566	-0.202191512	PASS
SLC12A1	15	48178438	48304078	-0.396255771	PASS
SLC12A2	5	128083766	128189688	-0.293443042	PASS
SLC12A3	16	56865207	56915850	-0.371357732	PASS
SLC12A4	16	67943474	67969601	-0.371357732	PASS
SLC12A5	20	46021690	46060152	-0.168668583	PASS
SLC12A6	15	34229996	34338060	-0.396255771	PASS
SLC12A7	5	1050376	1112035	0.055007813	PASS
SLC12A8	3	125082636	125212864	0.313454204	suppress_segment_too_large
SLC12A9	7	100826820	100867009	-0.262778904	PASS
SLC13A1	7	123113531	123199986	-0.388946609	PASS
SLC13A2	17	28473293	28497781	0.060555011	PASS
SLC13A3	20	46557823	46684467	-0.168668583	PASS
SLC13A4	7	135681237	135729258	-0.388946609	PASS
SLC13A5	17	6684713	6713567	0.128406487	PASS
SLC14A1	18	45687025	45752520	-0.376397843	PASS
SLC14A2	18	45212995	45683686	-0.376397843	PASS
SLC15A1	13	98683801	98752654	-0.390786075	PASS
SLC15A2	3	121894089	121944102	0.313454204	suppress_segment_too_large
SLC15A3	11	60937084	60952530	0.182439925	suppress_segment_too_large
SLC15A4	12	128793191	128823983	-0.240609783	PASS
SLC15A5	12	16188485	16277685	0.362961408	suppress_segment_too_large
SLC16A1	1	112911847	112957013	0.033763446	PASS
SLC16A10	6	111087503	111231194	-0.368590549	PASS
SLC16A11	17	7041630	7043923	0.128406487	PASS
SLC16A12	10	89430299	89556641	-0.39678585	PASS
SLC16A13	17	7036075	7040121	0.128406487	PASS
SLC16A14	2	230034974	230068999	0.025928102	PASS
SLC16A2	23	74421461	74533917	-0.012567603	PASS
SLC16A3	17	82228397	82261129	0.088516311	PASS
SLC16A4	1	110362848	110391082	0.033763446	PASS
SLC16A5	17	75087727	75106162	0.088516311	PASS
SLC16A6	17	68267026	68291267	0.088516311	PASS
SLC16A7	12	59596067	59789855	-0.202191512	PASS
SLC16A8	22	38078134	38084093	-0.575930959	PASS
SLC16A9	10	59650761	59736002	0.072405807	PASS
SLC17A1	6	25782897	25832059	0.048820648	PASS
SLC17A2	6	25912754	25930726	0.048820648	PASS
SLC17A3	6	25833066	25882286	0.048820648	PASS

SLC17A4	6	25754699	25781191	0.048820648	PASS
SLC17A5	6	73593379	73654155	-0.368590549	PASS
SLC17A6	11	22338097	22379503	-0.405444717	PASS
SLC17A7	19	49429401	49442360	0.14996833	PASS
SLC17A8	12	100357079	100422059	0.202141228	PASS
SLC17A9	20	62952647	62969585	-0.168668583	PASS
SLC18A1	8	20144855	20183206	-0.345962679	PASS
SLC18A2	10	117241073	117279430	0.063874692	PASS
SLC18A3	10	49610301	49612720	-0.404852538	PASS
SLC18B1	6	132769370	132813339	-0.458570262	PASS
SLC19A1	21	45493572	45544411	-0.064425456	suppress_segment_too_large
SLC19A2	1	169463909	169486003	-0.027073548	PASS
SLC19A3	2	227683763	227718031	0.025928102	PASS
SLC1A1	9	4490444	4587469	-0.310444659	PASS
SLC1A2	11	35251205	35420063	-0.405444717	PASS
SLC1A3	5	36606355	36688334	0.055007813	PASS
SLC1A4	2	64988477	65023865	-0.372959126	PASS
SLC1A5	19	46774883	46788594	0.14996833	PASS
SLC1A6	19	14950034	15022990	0.093487731	suppress_segment_too_large
SLC1A7	1	53087179	53142632	0.033763446	PASS
SLC20A1	2	112645857	112663827	-0.010901494	PASS
SLC20A2	8	42416475	42541926	-0.201142258	PASS
SLC22A1	6	160121789	160158718	-0.035448111	PASS
SLC22A10	11	63137867	63369718	0.182439925	suppress_segment_too_large
SLC22A11	11	64555626	64572875	0.182439925	suppress_segment_too_large
SLC22A12	11	64590641	64602353	0.182439925	suppress_segment_too_large
SLC22A13	3	38265812	38278757	-0.234617007	PASS
SLC22A14	3	38282294	38318575	-0.234617007	PASS
SLC22A15	1	115976498	116070054	0.033763446	PASS
SLC22A16	6	110424687	110476641	-0.368590549	PASS
SLC22A17	14	23346306	23352912	0.060212819	PASS
SLC22A18	11	2899721	2925246	-0.333835828	PASS
SLC22A18	11	2887780	2903740	-0.333835828	PASS
SLC22A2	6	160171061	160277638	-0.035448111	PASS
SLC22A23	6	3268962	3457022	0.048820648	PASS
SLC22A24	11	63079940	63144221	0.182439925	suppress_segment_too_large
SLC22A25	11	63163776	63229652	0.182439925	suppress_segment_too_large
SLC22A3	6	160348268	160452581	-0.035448111	PASS
SLC22A31	16	89195761	89201664	-0.371357732	PASS
SLC22A4	5	132294443	132344206	-0.293443042	PASS
SLC22A5	5	132369752	132395614	-0.293443042	PASS
SLC22A6	11	62936385	62984983	0.182439925	suppress_segment_too_large
SLC22A7	6	43295694	43305538	0.048820648	PASS
SLC22A8	11	62989154	63015839	0.182439925	suppress_segment_too_large
SLC22A9	11	63369789	63410294	0.182439925	suppress_segment_too_large
SLC23A1	5	139367196	139384553	-0.293443042	PASS

SLC23A2	20	4852356	5010293	-0.168668583	PASS
SLC23A3	2	219161465	219170095	0.025928102	PASS
SLC24A1	15	65611366	65660995	-0.697600509	PASS
SLC24A2	9	19507452	19786928	-0.310444659	PASS
SLC24A3	20	19212646	19722937	-0.168668583	PASS
SLC24A4	14	92322581	92501483	-0.192197564	PASS
SLC24A5	15	48120972	48142672	-0.396255771	PASS
SLC25A1	22	19175575	19178830	-0.575930959	PASS
SLC25A10	17	81712236	81721016	0.088516311	PASS
SLC25A11	17	4937130	4940251	0.128406487	PASS
SLC25A12	2	171784370	171999859	0.039726562	PASS
SLC25A13	7	96120220	96322147	-0.262778904	PASS
SLC25A14	23	130339888	130373361	-0.012567603	PASS
SLC25A15	13	40789412	40810111	-0.390786075	PASS
SLC25A16	10	68477999	68527474	0.072405807	PASS
SLC25A17	22	40769630	40819399	-0.575930959	PASS
SLC25A18	22	17563439	17590994	-0.575930959	PASS
SLC25A19	17	75272981	75289510	0.088516311	PASS
SLC25A2	5	141302629	141304045	-0.293443042	PASS
SLC25A20	3	48856931	48898993	-0.234617007	PASS
SLC25A21	14	36678431	37172866	0.060212819	PASS
SLC25A22	11	790475	798333	-0.333835828	PASS
SLC25A23	19	6436079	6465203	0.079632875	PASS
SLC25A24	1	108134036	108200849	0.033763446	PASS
SLC25A25	9	128068201	128109245	0.365190713	suppress_segment_too_large
SLC25A26	3	66133610	66388116	-0.234617007	PASS
SLC25A27	6	46652915	46678193	0.048820648	PASS
SLC25A28	10	99610522	99620609	-0.367947924	PASS
SLC25A29	14	100291111	100306547	-0.192197564	PASS
SLC25A3	12	98593591	98606379	0.202141228	PASS
SLC25A30	13	45393316	45418455	-0.390786075	PASS
SLC25A31	4	127730378	127774299	-0.011382299	PASS
SLC25A32	8	103398635	103415189	0.279445683	PASS
SLC25A33	1	9539482	9585179	0.373964873	suppress_segment_too_large
SLC25A34	1	15736405	15741396	0.033763446	PASS
SLC25A35	17	8287763	8295343	0.128406487	PASS
SLC25A36	3	140941830	140980961	-0.356957581	PASS
SLC25A37	8	23528805	23575463	-0.345962679	PASS
SLC25A38	3	39383324	39397351	-0.234617007	PASS
SLC25A39	17	44319625	44324870	0.088516311	PASS
SLC25A4	4	185143241	185150382	-0.462744716	PASS
SLC25A40	7	87833568	87876357	-0.262778904	PASS
SLC25A41	19	6426037	6433779	0.079632875	PASS
SLC25A42	19	19063999	19112888	0.093487731	suppress_segment_too_large
SLC25A43	23	119399060	119454478	-0.012567603	PASS
SLC25A44	1	156193932	156212796	0.103015788	PASS

SLC25A45	11	65375192	65383701	0.182439925	suppress_segment_too_large
SLC25A46	5	110738136	110765161	-0.293443042	PASS
SLC25A47	14	100323337	100330378	-0.192197564	PASS
SLC25A48	5	135579202	135889770	-0.293443042	PASS
SLC25A5	23	119468400	119471319	-0.012567603	PASS
SLC25A51	9	37879400	37904353	-0.310444659	PASS
SLC25A52	18	31759562	31760880	-0.376397843	PASS
SLC25A53	23	104099214	104157027	-0.012567603	PASS
SLC25A6	23	1386152	1392724	-0.012567603	PASS
SLC26A1	4	979073	993440	0.279089364	PASS
SLC26A10	12	57619527	57626151	-0.202191512	PASS
SLC26A11	17	80219699	80253500	0.088516311	PASS
SLC26A2	5	149960737	149993455	-0.293443042	PASS
SLC26A3	7	107765467	107803225	-0.388946609	PASS
SLC26A4	7	107660635	107717809	-0.388946609	PASS
SLC26A5	7	103352730	103446177	-0.388946609	PASS
SLC26A6	3	48625723	48635493	-0.234617007	PASS
SLC26A7	8	91209494	91398152	0.279445683	PASS
SLC26A8	6	35943514	36024868	0.048820648	PASS
SLC26A9	1	205913048	205943460	-0.256698369	PASS
SLC27A1	19	17468769	17506168	0.093487731	suppress_segment_too_large
SLC27A2	15	50182196	50236395	-0.396255771	PASS
SLC27A3	1	153774354	153780157	0.103015788	PASS
SLC27A4	9	128340646	128361470	0.365190713	suppress_segment_too_large
SLC27A5	19	58479512	58512413	0.063781462	PASS
SLC27A6	5	128538013	129033642	-0.293443042	PASS
SLC28A1	15	84884654	84945796	-0.697600509	PASS
SLC28A2	15	45252230	45277845	-0.396255771	PASS
SLC28A3	9	84275457	84340683	0.365190713	suppress_segment_too_large
SLC29A1	6	44219505	44234151	0.048820648	PASS
SLC29A2	11	66362521	66372214	0.182439925	suppress_segment_too_large
SLC29A3	10	71319258	71381423	0.072405807	PASS
SLC29A4	7	5274369	5306870	-0.69513118	PASS
SLC2A1	1	42925375	42959176	0.033763446	PASS
SLC2A10	20	46709487	46736347	-0.168668583	PASS
SLC2A11	22	23856703	23886309	-0.575930959	PASS
SLC2A12	6	133988697	134052636	-0.035448111	PASS
SLC2A13	12	39755021	40106089	-0.202191512	PASS
SLC2A14	12	7812512	7891148	-0.086128028	PASS
SLC2A2	3	170996348	171026750	0.318278996	suppress_segment_too_large
SLC2A3	12	7919230	7936275	-0.086128028	PASS
SLC2A4	17	7281667	7288257	0.128406487	PASS
SLC2A4R(	20	63739861	63743505	-0.168668583	PASS
SLC2A5	1	9035107	9088478	0.373964873	suppress_segment_too_large
SLC2A6	9	133471095	133479137	0.365190713	suppress_segment_too_large
SLC2A7	1	9003300	9026345	0.373964873	suppress_segment_too_large

SLC2A8	9	127397138	127408424	0.365190713	suppress_segment_too_large
SLC2A9	4	9771153	10054936	0.279089364	PASS
SLC30A1	1	211571568	211578742	-0.256698369	PASS
SLC30A10	1	219685427	219958647	-0.256698369	PASS
SLC30A2	1	26037252	26046133	0.033763446	PASS
SLC30A3	2	27253684	27275817	0.040498426	PASS
SLC30A4	15	45479611	45522807	-0.396255771	PASS
SLC30A5	5	69093646	69131069	-0.293443042	PASS
SLC30A6	2	32165841	32224379	0.040498426	PASS
SLC30A7	1	100896076	100981753	0.033763446	PASS
SLC30A8	8	116950273	117176714	0.279445683	PASS
SLC30A9	4	41990472	42090457	-0.011382299	PASS
SLC31A1	9	113221562	113264492	0.365190713	suppress_segment_too_large
SLC31A2	9	113150942	113164137	0.365190713	suppress_segment_too_large
SLC32A1	20	38724462	38729372	-0.168668583	PASS
SLC33A1	3	155821024	155854456	0.318278996	suppress_segment_too_large
SLC34A1	5	177379235	177398848	-0.150979337	PASS
SLC34A2	4	25648011	25678748	-0.458181871	PASS
SLC34A3	9	137230757	137236554	0.365190713	suppress_segment_too_large
SLC35A1	6	87470623	87512336	-0.368590549	PASS
SLC35A2	23	48903180	48911958	-0.012567603	PASS
SLC35A3	1	99969351	100035637	0.033763446	PASS
SLC35A4	5	140564456	140569103	-0.293443042	PASS
SLC35A5	3	112561709	112585577	0.313454204	suppress_segment_too_large
SLC35B1	17	49700934	49709014	0.088516311	PASS
SLC35B2	6	44254096	44257890	0.048820648	PASS
SLC35B3	6	8411463	8435611	0.048820648	PASS
SLC35B4	7	134289332	134317051	-0.388946609	PASS
SLC35C1	11	45804072	45813015	0.117376172	PASS
SLC35C2	20	46345980	46364458	-0.168668583	PASS
SLC35D1	1	66999332	67054099	0.033763446	PASS
SLC35D2	9	96313444	96383710	0.365190713	suppress_segment_too_large
SLC35D3	6	136922264	136925639	-0.035448111	PASS
SLC35E1	19	16549831	16572382	0.093487731	suppress_segment_too_large
SLC35E2A	1	1724838	1745999	0.373964873	suppress_segment_too_large
SLC35E2B	1	1659529	1692728	0.373964873	suppress_segment_too_large
SLC35E3	12	68746106	68793964	-0.202191512	PASS
SLC35E4	22	30635652	30669016	-0.575930959	PASS
SLC35F1	6	117907526	118317676	-0.368590549	PASS
SLC35F2	11	107790991	107928293	-0.356188624	PASS
SLC35F3	1	233904933	234324516	-0.256698369	PASS
SLC35F4	14	57563922	57982194	0.060212819	PASS
SLC35F5	2	113705011	113756823	-0.010901494	PASS
SLC35F6	2	26764284	26781231	0.040498426	PASS
SLC35G1	10	93893973	93956062	-0.39678585	PASS
SLC35G2	3	136818647	136855892	0.313454204	suppress_segment_too_large

SLC35G3	17	35192520	35194523		0.060555011	PASS
SLC35G4	18	11609596	11610612		-0.052305056	suppress_segment_too_large
SLC35G5	8	11330888	11332208		-0.345962679	PASS
SLC35G6	17	7481332	7483496		0.128406487	PASS
SLC36A1	5	151437046	151492381		-0.293443042	PASS
SLC36A2	5	151314978	151347590		-0.293443042	PASS
SLC36A3	5	151276762	151303766		-0.293443042	PASS
SLC36A4	11	93144171	93197964		0.062969153	PASS
SLC37A1	21	42496008	42581440		-0.064425456	suppress_segment_too_large
SLC37A2	11	125063067	125090312		-0.419612865	PASS
SLC37A3	7	140293693	140404433		-0.388946609	PASS
SLC37A4	11	119024114	119030906		-0.356188624	PASS
SLC38A1	12	46183063	46270017		-0.202191512	PASS
SLC38A10	17	81245000	81295547		0.088516311	PASS
SLC38A11	2	164896186	164955525		0.039726562	PASS
SLC38A2	12	46358189	46372867		-0.202191512	PASS
SLC38A3	3	50205246	50221486		-0.234617007	PASS
SLC38A4	12	46764761	46832408		-0.202191512	PASS
SLC38A5	23	48458537	48470256		-0.012567603	PASS
SLC38A6	14	60981114	61083733		0.060212819	PASS
SLC38A7	16	58665109	58685104		-0.371357732	PASS
SLC38A8	16	84009667	84042636		-0.371357732	PASS
SLC38A9	5	55625845	55773194		-0.293443042	PASS
SLC39A1	1	153959099	153968184		0.103015788	PASS
SLC39A10	2	195575977	195737702		0.025928102	PASS
SLC39A11	17	72645949	73092712		0.088516311	PASS
SLC39A12	10	17951839	18043292		0.039811807	PASS
SLC39A13	11	47407132	47416501		0.117376172	PASS
SLC39A14	8	22367249	22434129		-0.345962679	PASS
SLC39A2	14	20999255	21001871		0.060212819	PASS
SLC39A3	19	2732204	2740152		0.079632875	PASS
SLC39A4	8	144409742	144416895		0.307788765	PASS
SLC39A5	12	56230049	56237846		-0.202191512	PASS
SLC39A6	18	36108532	36129385		-0.376397843	PASS
SLC39A7	6	33200445	33204439		0.048820648	PASS
SLC39A8	4	102251041	102431258		-0.011382299	PASS
SLC39A9	14	69398015	69462388		0.060212819	PASS
SLC3A1	2	44275458	44321494		0.040498426	PASS
SLC3A2	11	62856102	62888875		0.182439925	suppress_segment_too_large
SLC40A1	2	189560579	189583758		0.025928102	PASS
SLC41A1	1	205789093	205813748		-0.256698369	PASS
SLC41A2	12	104802553	104958744		0.202141228	PASS
SLC41A3	3	126006355	126101561		0.313454204	suppress_segment_too_large
SLC43A1	11	57484534	57515786		-0.129266491	PASS
SLC43A2	17	1569267	1628886		0.128406487	PASS
SLC43A3	11	57406954	57427580		-0.129266491	PASS

SLC44A1	9	105244622	105439171	0.365190713	suppress_segment_too_large
SLC44A2	19	10602457	10644559	0.093487731	suppress_segment_too_large
SLC44A3	1	94820342	94895246	0.033763446	PASS
SLC44A4	6	31863192	31879046	0.048820648	PASS
SLC44A5	1	75202131	75611116	0.033763446	PASS
SLC45A1	1	8317826	8344167	0.373964873	suppress_segment_too_large
SLC45A2	5	33944616	33984730	0.055007813	PASS
SLC45A3	1	205657851	205680459	-0.256698369	PASS
SLC45A4	8	141207166	141308305	0.113969953	PASS
SLC46A1	17	28394756	28407197	0.060555011	PASS
SLC46A2	9	112878920	112890913	0.365190713	suppress_segment_too_large
SLC46A3	13	28700064	28718970	-0.390786075	PASS
SLC47A1	17	19495385	19579034	0.024610076	PASS
SLC47A2	17	19678288	19718979	0.024610076	PASS
SLC48A1	12	47753916	47782753	-0.202191512	PASS
SLC49A3	4	681829	689441	0.279089364	PASS
SLC4A1	17	44248385	44268141	0.088516311	PASS
SLC4A10	2	161424332	161985282	0.039726562	PASS
SLC4A11	20	3227417	3239559	-0.168668583	PASS
SLC4A1AI	2	27663471	27694976	0.040498426	PASS
SLC4A2	7	151057210	151076527	-0.388946609	PASS
SLC4A3	2	219627327	219641980	0.025928102	PASS
SLC4A4	4	71062667	71572087	-0.011382299	PASS
SLC4A5	2	74216242	74343414	-0.372959126	PASS
SLC4A7	3	27372721	27484420	0.311018244	suppress_segment_too_large
SLC4A8	12	51391317	51515763	-0.202191512	PASS
SLC4A9	5	140360202	140375143	-0.293443042	PASS
SLC50A1	1	155135344	155138857	0.103015788	PASS
SLC51A	3	196211487	196243178	0.326834516	PASS
SLC51B	15	65045370	65053396	-0.697600509	PASS
SLC52A1	17	5032600	5052009	0.128406487	PASS
SLC52A2	8	144354135	144361286	0.307788765	PASS
SLC52A3	20	760080	776015	-0.168668583	PASS
SLC5A1	22	32043032	32113029	-0.575930959	PASS
SLC5A10	17	18950345	19022595	0.024610076	PASS
SLC5A11	16	24845841	24911628	0.069936844	PASS
SLC5A12	11	26667019	26723427	-0.405444717	PASS
SLC5A2	16	31483002	31490860	0.069936844	PASS
SLC5A3	21	34073570	34106262	0.12860881	suppress_segment_too_large
SLC5A4	22	32218476	32255341	-0.575930959	PASS
SLC5A5	19	17871973	17895174	0.093487731	suppress_segment_too_large
SLC5A6	2	27199587	27212958	0.040498426	PASS
SLC5A7	2	107986523	108013994	-0.532032081	PASS
SLC5A8	12	101155493	101210407	0.202141228	PASS
SLC5A9	1	48222685	48248644	0.033763446	PASS
SLC6A1	3	10992186	11039249	0.311018244	suppress_segment_too_large

SLC6A11	3	10816200	10940733	0.311018244	suppress_segment_too_large
SLC6A12	12	190077	214570	-0.086128028	PASS
SLC6A13	12	220621	262873	-0.086128028	PASS
SLC6A14	23	116436622	116461458	-0.012567603	PASS
SLC6A15	12	84859488	84913615	0.202141228	PASS
SLC6A16	19	49289638	49325225	0.14996833	PASS
SLC6A17	1	110150486	110202202	0.033763446	PASS
SLC6A18	5	1225355	1246189	0.055007813	PASS
SLC6A19	5	1201595	1225117	0.055007813	PASS
SLC6A2	16	55655604	55706192	-0.371357732	PASS
SLC6A20	3	45755450	45796535	-0.234617007	PASS
SLC6A3	5	1392790	1445430	0.055007813	PASS
SLC6A4	17	30194319	30236002	0.060555011	PASS
SLC6A5	11	20599400	20659285	-0.405444717	PASS
SLC6A6	3	14402576	14489349	0.311018244	suppress_segment_too_large
SLC6A7	5	150189957	150222788	-0.293443042	PASS
SLC6A8	23	153688099	153696593	-0.012567603	PASS
SLC6A9	1	43991500	44031467	0.033763446	PASS
SLC7A1	13	29509410	29595688	-0.390786075	PASS
SLC7A10	19	33208664	33225850	0.093487731	suppress_segment_too_large
SLC7A11	4	138164097	138242349	-0.011382299	PASS
SLC7A13	8	86214052	86321146	0.279445683	PASS
SLC7A14	3	170459584	170586074	0.318278996	suppress_segment_too_large
SLC7A2	8	17497088	17570573	-0.345962679	PASS
SLC7A3	23	70925582	70931125	-0.012567603	PASS
SLC7A4	22	21028718	21032840	-0.575930959	PASS
SLC7A5	16	87830023	87869488	-0.371357732	PASS
SLC7A6	16	68264516	68301823	-0.371357732	PASS
SLC7A6OS	16	68284503	68310946	-0.371357732	PASS
SLC7A7	14	22773222	22829820	0.060212819	PASS
SLC7A8	14	23125295	23183674	0.060212819	PASS
SLC7A9	19	32830509	32869766	0.093487731	suppress_segment_too_large
SLC8A1	2	40097270	40611053	0.040498426	PASS
SLC8A2	19	47428017	47472168	0.14996833	PASS
SLC8A3	14	70044217	70189070	0.060212819	PASS
SLC8B1	12	113298759	113359493	0.202141228	PASS
SLC9A1	1	27098815	27166981	0.033763446	PASS
SLC9A2	2	102619707	102711318	0.048238121	PASS
SLC9A3	5	470458	524332	0.055007813	PASS
SLC9A3R1	17	74748652	74769353	0.088516311	PASS
SLC9A3R2	16	2025356	2039026	0.069936844	PASS
SLC9A4	2	102473303	102533972	0.048238121	PASS
SLC9A5	16	67237683	67272190	-0.371357732	PASS
SLC9A6	23	135973841	136047269	-0.012567603	PASS
SLC9A7	23	46599252	46759172	-0.012567603	PASS
SLC9A8	20	49812713	49892242	-0.168668583	PASS

SLC9A9	3	143265222	143848531	0.318278996	suppress_segment_too_large
SLC9B1	4	102885048	103019739	-0.011382299	PASS
SLC9B2	4	103019868	103085829	-0.011382299	PASS
SLC9C1	3	112140887	112294258	0.313454204	suppress_segment_too_large
SLC9C2	1	173500464	173603094	-0.027073548	PASS
SLCO1A2	12	21264600	21419594	0.362961408	suppress_segment_too_large
SLCO1B1	12	21131202	21239246	0.362961408	suppress_segment_too_large
SLCO1B3	12	20810702	20916911	0.362961408	suppress_segment_too_large
SLCO1B7	12	20962768	21092745	0.362961408	suppress_segment_too_large
SLCO1C1	12	20695355	20753386	0.362961408	suppress_segment_too_large
SLCO2A1	3	133928145	134052184	0.313454204	suppress_segment_too_large
SLCO2B1	11	75100563	75206549	0.062969153	PASS
SLCO3A1	15	91853695	92172435	-0.697600509	PASS
SLCO4A1	20	62642445	62685785	-0.168668583	PASS
SLCO4C1	5	102233986	102296549	-0.293443042	PASS
SLCO5A1	8	69667047	69835064	-0.201142258	PASS
SLCO6A1	5	102371782	102499016	-0.293443042	PASS
SLF1	5	94618347	94739436	-0.293443042	PASS
SLF2	10	100912569	100965136	-0.367947924	PASS
SLFN11	17	35350305	35373701	0.060555011	PASS
SLFN12	17	35411060	35433283	0.060555011	PASS
SLFN12L	17	35464249	35537861	0.060555011	PASS
SLFN13	17	35435096	35448837	0.060555011	PASS
SLFN14	17	35548125	35558098	0.060555011	PASS
SLFN5	17	35243036	35273655	0.060555011	PASS
SLFNL1	1	41015597	41023237	0.033763446	PASS
SLIRP	14	77708071	77761104	-0.192197564	PASS
SLIT1	10	96998038	97185920	-0.367947924	PASS
SLIT2	4	20253260	20620561	0.279089364	PASS
SLIT3	5	168661733	169301129	-0.150979337	PASS
SLITRK1	13	83877205	83882393	-0.390786075	PASS
SLITRK2	23	145817832	145829856	-0.012567603	PASS
SLITRK3	3	165186720	165197109	0.318278996	suppress_segment_too_large
SLITRK4	23	143622790	143635777	-0.012567603	PASS
SLITRK5	13	87672615	87696272	-0.390786075	PASS
SLITRK6	13	85792787	85806683	-0.390786075	PASS
SLK	10	103967201	104029233	-0.367947924	PASS
SLMAP	3	57755450	57929168	-0.234617007	PASS
SLN	11	107707378	107719693	-0.356188624	PASS
SLPI	20	45252239	45254564	-0.168668583	PASS
SLTM	15	58879045	58933653	-0.697600509	PASS
SLU7	5	160401641	160421711	-0.150979337	PASS
SLURP1	8	142740944	142742411	0.113969953	PASS
SLURP2	8	142764338	142769844	0.113969953	PASS
SLX1A	16	30193887	30197561	0.069936844	PASS
SLX1B	16	29454501	29458219	0.069936844	PASS

SLX4	16	3581181	3611598	0.069936844	PASS
SLX4IP	20	10435303	10636829	-0.168668583	PASS
SMAD1	4	145481194	145558079	-0.011382299	PASS
SMAD2	18	47808957	47931146	-0.376397843	PASS
SMAD3	15	67063763	67195195	-0.697600509	PASS
SMAD4	18	51028394	51085045	-0.376397843	PASS
SMAD5	5	136132845	136188747	-0.293443042	PASS
SMAD6	15	66702228	66782848	-0.697600509	PASS
SMAD7	18	48919853	48952052	-0.376397843	PASS
SMAD9	13	36844831	36920765	-0.390786075	PASS
SMAGP	12	51244558	51270890	-0.202191512	PASS
SMAP1	6	70667776	70862015	-0.368590549	PASS
SMAP2	1	40344850	40423326	0.033763446	PASS
SMARCA1	23	129446501	129523500	-0.012567603	PASS
SMARCA2	9	1980290	2193624	-0.310444659	PASS
SMARCA4	19	10960825	11079426	0.093487731	suppress_segment_too_large
SMARCA5	4	143513463	143557486	-0.011382299	PASS
SMARCAI	4	94207611	94291292	-0.011382299	PASS
SMARCAI	2	216412414	216483053	0.025928102	PASS
SMARCB1	22	23786931	23838008	-0.575930959	PASS
SMARCC1	3	47585272	47782106	-0.234617007	PASS
SMARCC2	12	56162983	56189567	-0.202191512	PASS
SMARCD1	12	50084972	50100712	-0.202191512	PASS
SMARCD2	17	63832081	63843065	0.088516311	PASS
SMARCD3	7	151238764	151277896	-0.388946609	PASS
SMARCE1	17	40624962	40648864	0.060555011	PASS
SMC1A	23	53374149	53422728	-0.012567603	PASS
SMC1B	22	45344063	45413619	-0.808211634	PASS
SMC2	9	104094260	104141417	0.365190713	suppress_segment_too_large
SMC3	10	110567691	110604636	0.063874692	PASS
SMC4	3	160399274	160434962	0.318278996	suppress_segment_too_large
SMC5	9	70258962	70354888	0.365190713	suppress_segment_too_large
SMC6	2	17663812	17800242	0.040498426	PASS
SMCHD1	18	2655738	2805017	-0.052305056	suppress_segment_too_large
SMCO1	3	196506877	196515366	0.326834516	PASS
SMCO2	12	27466810	27502185	0.362961408	suppress_segment_too_large
SMCO3	12	14804650	14814182	0.362961408	suppress_segment_too_large
SMCO4	11	93478472	93543508	0.062969153	PASS
SMCP	1	152878317	152885047	0.103015788	PASS
SMCR8	17	18315310	18328055	0.024610076	PASS
SMDT1	22	42079691	42084284	-0.575930959	PASS
SMG1	16	18804853	18926454	0.069936844	PASS
SMG5	1	156249224	156282825	0.103015788	PASS
SMG6	17	2059839	2303771	0.128406487	PASS
SMG7	1	183472216	183598246	-0.027073548	PASS
SMG8	17	59209400	59215247	0.088516311	PASS

SMG9	19	43727992	43754990	0.14996833	PASS
SMIM1	1	3772761	3775982	0.373964873	suppress_segment_too_large
SMIM10	23	134990938	134992473	-0.012567603	PASS
SMIM10L1	12	11171222	11176016	0.362961408	suppress_segment_too_large
SMIM10L2	23	135421943	135428074	-0.012567603	PASS
SMIM10L2	23	135094987	135098634	-0.012567603	PASS
SMIM12	1	34712737	34859816	0.033763446	PASS
SMIM13	6	11094033	11138733	0.048820648	PASS
SMIM14	4	39546330	39639090	-0.011382299	PASS
SMIM15	5	61157709	61162474	-0.293443042	PASS
SMIM17	19	56643145	56655766	-0.180829161	PASS
SMIM18	8	30638600	30646064	-0.345962679	PASS
SMIM19	8	42541155	42555193	-0.201142258	PASS
SMIM2	13	44143543	44161257	-0.390786075	PASS
SMIM20	4	25861830	25929874	-0.458181871	PASS
SMIM21	18	75409476	75427703	-0.376397843	PASS
SMIM22	16	4788397	4796491	0.069936844	PASS
SMIM23	5	171782432	171796126	-0.150979337	PASS
SMIM24	19	3473986	3480542	0.079632875	PASS
SMIM26	20	18567347	18569563	-0.168668583	PASS
SMIM27	9	32551144	32568621	-0.310444659	PASS
SMIM29	6	34246380	34249470	0.048820648	PASS
SMIM3	5	150777946	150796734	-0.293443042	PASS
SMIM31	4	164754064	164803795	-0.011382299	PASS
SMIM35	11	118003634	118088299	-0.356188624	PASS
SMIM36	17	55448433	55511444	0.088516311	PASS
SMIM4	3	52534013	52579237	-0.234617007	PASS
SMIM5	17	75633434	75641404	0.088516311	PASS
SMIM6	17	75646243	75647977	0.088516311	PASS
SMIM7	19	16630751	16660442	0.093487731	suppress_segment_too_large
SMIM8	6	87322583	87399749	-0.368590549	PASS
SMIM9	23	154823348	154834662	-0.012567603	PASS
SMKR1	7	129502479	129512932	-0.388946609	PASS
SMLR1	6	130827406	130837135	-0.458570262	PASS
SMN1	5	70925030	70953942	-0.293443042	PASS
SMN2	5	70049612	70078522	-0.293443042	PASS
SMNDC1	10	110290730	110304951	0.063874692	PASS
SMO	7	129188872	129213545	-0.388946609	PASS
SMOC1	14	69854131	70032366	0.060212819	PASS
SMOC2	6	168441151	168673445	-0.035448111	PASS
SMOX	20	4120980	4187747	-0.168668583	PASS
SMPD1	11	6390431	6394998	-0.405444717	PASS
SMPD2	6	109440763	109443919	-0.368590549	PASS
SMPD3	16	68358325	68448688	-0.371357732	PASS
SMPD4	2	130151392	130182750	-0.475215623	PASS
SMPDL3A	6	122789049	122809720	-1.97829255	PASS

SMPDL3B	1	27934993	27959157	0.033763446	PASS
SMPX	23	21705972	21758163	-0.012567603	PASS
SMR3A	4	70360761	70367158	-0.011382299	PASS
SMR3B	4	70370093	70390244	-0.011382299	PASS
SMS	23	21940573	21994835	-0.012567603	PASS
SMTN	22	31064105	31104757	-0.575930959	PASS
SMTNL1	11	57542641	57550274	-0.129266491	PASS
SMTNL2	17	4583999	4608319	0.128406487	PASS
SMU1	9	33041764	33076659	-0.310444659	PASS
SMUG1	12	54121277	54189008	-0.202191512	PASS
SMURF1	7	99027438	99144100	-0.262778904	PASS
SMURF2	17	64542295	64662068	0.088516311	PASS
SMYD1	2	88067780	88113387	-0.340781246	PASS
SMYD2	1	214281102	214337131	-0.256698369	PASS
SMYD3	1	245749342	246507312	-0.368111568	PASS
SMYD4	17	1779485	1830634	0.128406487	PASS
SMYD5	2	73214222	73227237	-0.372959126	PASS
SNAI1	20	49982999	49988886	-0.168668583	PASS
SNAI2	8	48917604	48921740	-0.201142258	PASS
SNAI3	16	88677682	88686493	-0.371357732	PASS
SNAP23	15	42491233	42545356	-0.396255771	PASS
SNAP25	20	10218830	10307418	-0.168668583	PASS
SNAP29	22	20858983	20891218	-0.575930959	PASS
SNAP47	1	227728539	227781231	-0.256698369	PASS
SNAP91	6	83552880	83709691	-0.368590549	PASS
SNAPC1	14	61762357	61796428	0.060212819	PASS
SNAPC2	19	7920316	7923250	0.079632875	PASS
SNAPC3	9	15422704	15465953	-0.310444659	PASS
SNAPC4	9	136375577	136400168	0.365190713	suppress_segment_too_large
SNAPC5	15	66490135	66497813	-0.697600509	PASS
SNAPIN	1	153658654	153661830	0.103015788	PASS
SNCA	4	89724099	89838315	-0.011382299	PASS
SNCAIP	5	122311354	122464219	-0.293443042	PASS
SNCB	5	176620084	176630556	-0.150979337	PASS
SNCG	10	86958618	86963260	-0.292453993	PASS
SND1	7	127652180	128092609	-0.388946609	PASS
SNED1	2	240998838	241095568	0.025928102	PASS
SNF8	17	48929316	48945117	0.088516311	PASS
SNHG28	1	159834474	159873053	-0.467855874	PASS
SNIP1	1	37534449	37554344	0.033763446	PASS
SNN	16	11668414	11679159	0.069936844	PASS
SNORC	2	232857270	232878708	0.025928102	PASS
SNPH	20	1266280	1309328	-0.168668583	PASS
SNRK	3	43286512	43424764	-0.234617007	PASS
SNRNP200	2	96274336	96305515	0.048238121	PASS
SNRNP25	16	53010	57669	0.069936844	PASS

SNRNP27	2	69893560	69905575	-0.372959126	PASS
SNRNP35	12	123457641	123473154	0.202141228	PASS
SNRNP40	1	31259568	31296782	0.033763446	PASS
SNRNP48	6	7590199	7611967	0.048820648	PASS
SNRNP70	19	49085419	49108605	0.14996833	PASS
SNRPA	19	40750637	40765389	0.093487731	suppress_segment_too_large
SNRPA1	15	101281510	101295282	-0.697600509	PASS
SNRPB	20	2461634	2470853	-0.168668583	PASS
SNRPB2	20	16729961	16742563	-0.168668583	PASS
SNRPC	6	34757406	34773794	0.048820648	PASS
SNRPD1	18	21612267	21633524	-0.376397843	PASS
SNRPD2	19	45687454	45692569	0.14996833	PASS
SNRPD3	22	24555503	24609980	-0.575930959	PASS
SNRPE	1	203861603	203870550	-0.256698369	PASS
SNRPF	12	95858928	95903828	0.202141228	PASS
SNRPG	2	70281362	70293771	-0.372959126	PASS
SNRPN	15	24823637	24978723	-0.396255771	PASS
SNTA1	20	33407955	33443892	-0.168668583	PASS
SNTB1	8	120535745	120813273	0.279445683	PASS
SNTB2	16	69187129	69309052	-0.371357732	PASS
SNTG1	8	49909789	50796691	-0.201142258	PASS
SNTG2	2	950868	1367613	0.040498426	PASS
SNTN	3	63652668	63679020	-0.234617007	PASS
SNU13	22	41673930	41690504	-0.575930959	PASS
SNUPN	15	75598083	75626469	-0.697600509	PASS
SNURF	15	24954986	24977850	-0.396255771	PASS
SNW1	14	77717599	77761207	-0.192197564	PASS
SNX1	15	64094123	64146090	-0.697600509	PASS
SNX10	7	26291895	26374329	-0.69513118	PASS
SNX11	17	48103357	48123074	0.088516311	PASS
SNX12	23	71056332	71073426	-0.012567603	PASS
SNX13	7	17790761	17940501	-0.69513118	PASS
SNX14	6	85505496	85594156	-0.368590549	PASS
SNX15	11	65027408	65040572	0.182439925	suppress_segment_too_large
SNX16	8	81799581	81842866	0.279445683	PASS
SNX17	2	27370496	27377533	0.040498426	PASS
SNX18	5	54517759	54546585	-0.293443042	PASS
SNX19	11	130875436	130916509	-0.419612865	PASS
SNX2	5	122774996	122830108	-0.293443042	PASS
SNX20	16	50666300	50681353	0.069936844	PASS
SNX21	20	45833810	45843275	-0.168668583	PASS
SNX22	15	64151715	64157481	-0.697600509	PASS
SNX24	5	122843439	123029354	-0.293443042	PASS
SNX25	4	185204237	185370185	-0.462744716	PASS
SNX27	1	151612006	151699091	0.103015788	PASS
SNX29	16	11976737	12574289	0.069936844	PASS

SNX3	6	108211222	108261260	-0.368590549	PASS
SNX30	9	112750838	112881671	0.365190713	suppress_segment_too_large
SNX31	8	100572882	100663415	0.279445683	PASS
SNX32	11	65833641	65856896	0.182439925	suppress_segment_too_large
SNX33	15	75647906	75662301	-0.697600509	PASS
SNX4	3	125446644	125520197	0.313454204	suppress_segment_too_large
SNX5	20	17941597	17968980	-0.168668583	PASS
SNX6	14	34561094	34630183	0.060212819	PASS
SNX7	1	98661701	98760500	0.033763446	PASS
SNX8	7	2251770	2354318	-0.69513118	PASS
SNX9	6	157700387	157945077	-0.035448111	PASS
SOAT1	1	179293714	179358680	-0.027073548	PASS
SOAT2	12	53103518	53124538	-0.202191512	PASS
SOBP	6	107489958	107660167	-0.368590549	PASS
SOCS1	16	11254405	11256200	0.069936844	PASS
SOCS2	12	93569814	93583487	0.202141228	PASS
SOCS3	17	78356778	78360077	0.088516311	PASS
SOCS4	14	55027230	55049488	0.060212819	PASS
SOCS5	2	46698952	46780245	0.040498426	PASS
SOCS6	18	70288901	70330200	-0.376397843	PASS
SOCS7	17	38352228	38405593	0.060555011	PASS
SOD1	21	31659622	31668931	0.12860881	suppress_segment_too_large
SOD2	6	159669057	159762529	-0.035448111	PASS
SOD3	4	24789912	24800842	0.279089364	PASS
SOGA1	20	36777442	36863686	-0.168668583	PASS
SOGA3	6	127472794	127519191	-0.458570262	PASS
SOHLH1	9	135693407	135699528	0.365190713	suppress_segment_too_large
SOHLH2	13	36168208	36214615	-0.390786075	PASS
SON	21	33542618	33577481	0.12860881	suppress_segment_too_large
SORBS1	10	95311771	95561414	-0.39678585	PASS
SORBS2	4	185585444	185956652	-0.462744716	PASS
SORBS3	8	22544986	22575788	-0.345962679	PASS
SORCS1	10	106573663	107164534	-0.367947924	PASS
SORCS2	4	7192538	7742836	0.279089364	PASS
SORCS3	10	104641101	105265235	-0.367947924	PASS
SORD	15	45023104	45077185	-0.396255771	PASS
SORL1	11	121452203	121633693	-0.356188624	PASS
SORT1	1	109309568	109397951	0.033763446	PASS
SOS1	2	38981396	39124345	0.040498426	PASS
SOS2	14	50117120	50231558	0.060212819	PASS
SOST	17	43753731	43758788	0.088516311	PASS
SOSTDC1	7	16461481	16530580	-0.69513118	PASS
SOWAHA	5	132813587	132816797	-0.293443042	PASS
SOWAHB	4	76894928	76898147	-0.011382299	PASS
SOWAHC	2	109614334	109618990	-0.010901494	PASS
SOWAHD	23	119758613	119760164	-0.012567603	PASS

SOX1	13	112067647	112070488	-0.390786075	PASS
SOX10	22	37970686	37987422	-0.575930959	PASS
SOX11	2	5692667	5701385	0.040498426	PASS
SOX12	20	325595	330224	-0.168668583	PASS
SOX13	1	204073115	204127743	-0.256698369	PASS
SOX14	3	137764284	137766338	0.313454204	suppress_segment_too_large
SOX15	17	7588178	7590170	0.128406487	PASS
SOX17	8	54457935	54460888	-0.201142258	PASS
SOX18	20	64047582	64049641	-0.168668583	PASS
SOX2	3	181711924	181714436	0.318278996	suppress_segment_too_large
SOX21	13	94709622	94712399	-0.390786075	PASS
SOX3	23	140502985	140505116	-0.012567603	PASS
SOX30	5	157625679	157671480	-0.150979337	PASS
SOX4	6	21592768	21598619	0.048820648	PASS
SOX5	12	23529500	24562544	0.362961408	suppress_segment_too_large
SOX6	11	15966449	16739591	-0.405444717	PASS
SOX7	8	10723768	10730512	-0.345962679	PASS
SOX8	16	981808	986979	0.069936844	PASS
SOX9	17	72121020	72126420	0.088516311	PASS
SP1	12	53380176	53416446	-0.202191512	PASS
SP100	2	230415942	230544090	0.025928102	PASS
SP110	2	230167293	230225729	0.025928102	PASS
SP140	2	230203110	230313215	0.025928102	PASS
SP140L	2	230327184	230403732	0.025928102	PASS
SP2	17	47896150	47928957	0.088516311	PASS
SP3	2	173906459	173965702	0.039726562	PASS
SP4	7	21428034	21514822	-0.69513118	PASS
SP5	2	170715351	170718078	0.039726562	PASS
SP6	17	47844908	47855874	0.088516311	PASS
SP7	12	53326575	53345315	-0.202191512	PASS
SP8	7	20782283	20786886	-0.69513118	PASS
SP9	2	174334946	174338492	0.039726562	PASS
SPA17	11	124673798	124697518	-0.419612865	PASS
SPAAR	9	35909483	35937153	-0.310444659	PASS
SPACA1	6	88047789	88066832	-0.368590549	PASS
SPACA3	17	32970376	32997877	0.060555011	PASS
SPACA4	19	48606743	48607714	0.14996833	PASS
SPACA5B	23	48130657	48132613	-0.012567603	PASS
SPACA6	19	51689128	51712387	-0.016761391	PASS
SPACA7	13	112376319	112434689	-0.390786075	PASS
SPACA9	9	132878027	132890201	0.365190713	suppress_segment_too_large
SPAG1	8	100157906	100259278	0.279445683	PASS
SPAG11A	8	7847876	7868867	-0.345962679	PASS
SPAG11B	8	7442684	7463674	-0.345962679	PASS
SPAG16	2	213284379	214410501	0.025928102	PASS
SPAG17	1	117953861	118185223	0.033763446	PASS

SPAG4	20	35615892	35621049	-0.168668583	PASS
SPAG5	17	28577565	28599279	0.060555011	PASS
SPAG6	10	22345445	22454224	0.039811807	PASS
SPAG7	17	4959226	4967872	0.128406487	PASS
SPAG8	9	35808045	35812272	-0.310444659	PASS
SPAG9	17	50962174	51120865	0.088516311	PASS
SPAM1	7	123925237	123971414	-0.388946609	PASS
SPANXB1	23	141002591	141003706	-0.012567603	PASS
SPANXC	23	141241463	141242496	-0.012567603	PASS
SPANXD	23	141697411	141698739	-0.012567603	PASS
SPANXN1	23	145246828	145256112	-0.012567603	PASS
SPANXN2	23	143711955	143721423	-0.012567603	PASS
SPANXN3	23	143508735	143517475	-0.012567603	PASS
SPANXN4	23	143025918	143034702	-0.012567603	PASS
SPANXN5	23	52796144	52798101	-0.012567603	PASS
SPARC	5	151661096	151687165	-0.293443042	PASS
SPARCL1	4	87473335	87531061	-0.011382299	PASS
SPART	13	36301638	36370180	-0.390786075	PASS
SPAST	2	32063547	32157637	0.040498426	PASS
SPATA1	1	84506300	84566194	0.033763446	PASS
SPATA12	3	57060441	57075432	-0.234617007	PASS
SPATA13	13	23979805	24307074	-0.390786075	PASS
SPATA16	3	172889358	173141268	0.318278996	suppress_segment_too_large
SPATA17	1	217631324	217871696	-0.256698369	PASS
SPATA18	4	52051331	52097292	-0.011382299	PASS
SPATA19	11	133840631	133845538	-0.419612865	PASS
SPATA2	20	49903391	49915508	-0.168668583	PASS
SPATA20	17	50543058	50555852	0.088516311	PASS
SPATA21	1	16387117	16437424	0.033763446	PASS
SPATA22	17	3440019	3513852	0.128406487	PASS
SPATA24	5	139396563	139404088	-0.293443042	PASS
SPATA25	20	45886489	45887635	-0.168668583	PASS
SPATA2L	16	89696343	89701705	-0.371357732	PASS
SPATA3	2	230990324	231025055	0.025928102	PASS
SPATA31A	9	39355669	39361959	-0.310444659	PASS
SPATA31A	9	66986304	66992583	-0.310444659	PASS
SPATA31A	9	60914374	60920650	-0.310444659	PASS
SPATA31A	9	42183659	42189882	-0.310444659	PASS
SPATA31A	9	61190003	61196280	-0.310444659	PASS
SPATA31D	9	81988772	81995256	0.365190713	suppress_segment_too_large
SPATA31D	9	81943586	81950038	0.365190713	suppress_segment_too_large
SPATA31D	9	81928514	81934948	0.365190713	suppress_segment_too_large
SPATA31E	9	87882826	87888899	0.365190713	suppress_segment_too_large
SPATA32	17	45254393	45262112	0.088516311	PASS
SPATA33	16	89657802	89671272	-0.371357732	PASS
SPATA4	4	176184638	176195671	-0.011382299	PASS

SPATA45	1	212830141	212847649	-0.256698369	PASS
SPATA46	1	162373203	162376870	-0.027073548	PASS
SPATA48	7	50096036	50159830	-0.750061359	PASS
SPATA5	4	122923074	123319450	-0.011382299	PASS
SPATA5L1	15	45402331	45421419	-0.396255771	PASS
SPATA6	1	48295372	48472208	0.033763446	PASS
SPATA6L	9	4553386	4666674	-0.310444659	PASS
SPATA7	14	88384924	88470350	-0.192197564	PASS
SPATA8	15	96783389	96785615	-0.697600509	PASS
SPATA9	5	95652181	95698711	-0.293443042	PASS
SPATC1	8	144012414	144047085	0.307788765	PASS
SPATC1L	21	46161148	46184476	-0.064425456	suppress_segment_too_large
SPATS1	6	44342660	44377167	0.048820648	PASS
SPATS2	12	49366584	49527424	-0.202191512	PASS
SPATS2L	2	200305881	200482263	0.025928102	PASS
SPC24	19	11131520	11155808	0.093487731	suppress_segment_too_large
SPC25	2	168834132	168913371	0.039726562	PASS
SPCS1	3	52704955	52711146	-0.234617007	PASS
SPCS2	11	74949247	74979031	0.062969153	PASS
SPCS3	4	176319964	176332245	-0.011382299	PASS
SPDEF	6	34537802	34556333	0.048820648	PASS
SPDL1	5	169583634	169604778	-0.150979337	PASS
SPDYA	2	28782517	28850611	0.040498426	PASS
SPDYC	11	65170154	65173244	0.182439925	suppress_segment_too_large
SPDYE1	7	44000889	44010122	-0.69513118	PASS
SPDYE16	7	76531319	76541459	-0.262778904	PASS
SPDYE2	7	102551232	102562308	-0.388946609	PASS
SPDYE2B	7	102650325	102661398	-0.388946609	PASS
SPDYE3	7	100307702	100322196	-0.262778904	PASS
SPDYE4	17	8753106	8758559	0.128406487	PASS
SPDYE5	7	75493625	75504304	-0.262778904	PASS
SPDYE6	7	102347206	102356444	-0.388946609	PASS
SPECC1	17	20009344	20319026	0.024610076	PASS
SPECC1L	22	24270817	24417740	-0.575930959	PASS
SPECC1L-	22	24270898	24442356	-0.575930959	PASS
SPEF1	20	3777504	3781448	-0.168668583	PASS
SPEF2	5	35617844	35814611	0.055007813	PASS
SPEG	2	219434846	219498287	0.025928102	PASS
SPEM1	17	7420360	7421632	0.128406487	PASS
SPEM2	17	7425615	7427568	0.128406487	PASS
SPEN	1	15847864	15940460	0.033763446	PASS
SPERT	13	45702311	45714559	-0.390786075	PASS
SPESP1	15	68818221	68946811	-0.697600509	PASS
SPG11	15	44562696	44663678	-0.396255771	PASS
SPG21	15	64963021	64990310	-0.697600509	PASS
SPG7	16	89490719	89557768	-0.371357732	PASS

SPHK1	17	76376584	76387860	0.088516311	PASS
SPHK2	19	48619291	48630717	0.14996833	PASS
SPHKAP	2	227979950	228181645	0.025928102	PASS
SPI1	11	47354860	47378576	0.117376172	PASS
SPIB	19	50418938	50431313	-0.016761391	PASS
SPIC	12	101475421	101486997	0.202141228	PASS
SPICE1	3	113442718	113515187	0.313454204	suppress_segment_too_large
SPIDR	8	47260575	47736306	-0.201142258	PASS
SPIN1	9	88388419	88478694	0.365190713	suppress_segment_too_large
SPIN2A	23	57134530	57137625	-0.012567603	PASS
SPIN2B	23	57118551	57121546	-0.012567603	PASS
SPIN3	23	56818298	56995827	-0.012567603	PASS
SPIN4	23	63347228	63351344	-0.012567603	PASS
SPINDOC	11	63813388	63827718	0.182439925	suppress_segment_too_large
SPINK1	5	147824568	147831786	-0.293443042	PASS
SPINK13	5	148268180	148286254	-0.293443042	PASS
SPINK14	5	148169733	148175398	-0.293443042	PASS
SPINK2	4	56809860	56821742	-0.011382299	PASS
SPINK4	9	33218365	33248567	-0.310444659	PASS
SPINK5	5	148025683	148137289	-0.293443042	PASS
SPINK6	5	148202794	148215137	-0.293443042	PASS
SPINK7	5	148312419	148315922	-0.293443042	PASS
SPINK8	3	48306842	48328341	-0.234617007	PASS
SPINK9	5	148321203	148339849	-0.293443042	PASS
SPINT1	15	40844018	40858207	-0.396255771	PASS
SPINT2	19	38244035	38292614	0.093487731	suppress_segment_too_large
SPINT3	20	45512461	45515624	-0.168668583	PASS
SPINT4	20	45722351	45725830	-0.168668583	PASS
SPIRE1	18	12446512	12658134	-0.052305056	suppress_segment_too_large
SPIRE2	16	89818179	89871319	-0.371357732	PASS
SPN	16	29662979	29670876	0.069936844	PASS
SPNS1	16	28974221	28984548	0.069936844	PASS
SPNS2	17	4498838	4539035	0.128406487	PASS
SPNS3	17	4433688	4488208	0.128406487	PASS
SPO11	20	57329759	57343994	-0.168668583	PASS
SPOCD1	1	31790422	31816051	0.033763446	PASS
SPOCK1	5	136975298	137598379	-0.293443042	PASS
SPOCK2	10	72059035	72089032	0.072405807	PASS
SPOCK3	4	166733384	167234796	-0.011382299	PASS
SPON1	11	13962689	14267884	-0.405444717	PASS
SPON2	4	1166932	1208962	0.279089364	PASS
SPOP	17	49598884	49678234	0.088516311	PASS
SPOPL	2	138501801	138573547	-0.475215623	PASS
SPOUT1	9	128819651	128829821	0.365190713	suppress_segment_too_large
SPP1	4	87975650	87983426	-0.011382299	PASS
SPP2	2	234050679	234077134	0.025928102	PASS

SPPL2A	15	50702266	50765808	-0.396255771	PASS
SPPL2B	19	2328615	2354806	0.079632875	PASS
SPPL2C	17	45844835	45847072	0.088516311	PASS
SPPL3	12	120762510	120904371	0.202141228	PASS
SPR	2	72887360	72892158	-0.372959126	PASS
SPRED1	15	38252326	38357249	-0.396255771	PASS
SPRED2	2	65310851	65432637	-0.372959126	PASS
SPRED3	19	38388421	38399587	0.093487731	suppress_segment_too_large
SPRN	10	133420666	133424572	0.063874692	PASS
SPRR1A	1	152985231	152985500	0.103015788	PASS
SPRR1B	1	153031202	153032900	0.103015788	PASS
SPRR2A	1	153056113	153057537	0.103015788	PASS
SPRR2B	1	153070224	153070840	0.103015788	PASS
SPRR2D	1	153039725	153041931	0.103015788	PASS
SPRR2E	1	153093135	153106184	0.103015788	PASS
SPRR2F	1	153112114	153113515	0.103015788	PASS
SPRR2G	1	153149582	153150869	0.103015788	PASS
SPRR3	1	153001747	153003856	0.103015788	PASS
SPRR4	1	152970666	152972574	0.103015788	PASS
SPRTN	1	231337104	231355023	-0.256698369	PASS
SPRY1	4	123396795	123403760	-0.011382299	PASS
SPRY2	13	80335976	80340951	-0.390786075	PASS
SPRY3	23	155767812	155782459	-0.012567603	PASS
SPRY4	5	142310427	142326455	-0.293443042	PASS
SPRYD3	12	53064316	53079420	-0.202191512	PASS
SPRYD4	12	56468567	56479707	-0.202191512	PASS
SPRYD7	13	49912706	49936490	-0.390786075	PASS
SPSB1	1	9292880	9369532	0.373964873	suppress_segment_too_large
SPSB2	12	6870935	6889358	-0.086128028	PASS
SPSB3	16	1776712	1793700	0.069936844	PASS
SPSB4	3	141051402	141148611	-0.356957581	PASS
SPTA1	1	158610706	158686715	-0.467855874	PASS
SPTAN1	9	128552558	128633662	0.365190713	suppress_segment_too_large
SPTB	14	64746283	64879907	0.060212819	PASS
SPTBN1	2	54456285	54671445	0.040498426	PASS
SPTBN2	11	66685248	66729226	0.182439925	suppress_segment_too_large
SPTBN4	19	40466241	40576464	0.093487731	suppress_segment_too_large
SPTBN5	15	41848144	41894077	-0.396255771	PASS
SPTLC1	9	92000087	92115384	0.365190713	suppress_segment_too_large
SPTLC2	14	77505997	77616773	-0.192197564	PASS
SPTLC3	20	13008979	13169103	-0.168668583	PASS
SPTSSA	14	34432789	34462356	0.060212819	PASS
SPTSSB	3	161344792	161372880	0.318278996	suppress_segment_too_large
SPTY2D1	11	18606401	18634791	-0.405444717	PASS
SPTY2D1(	11	18588781	18610255	-0.405444717	PASS
SPX	12	21526307	21541249	0.362961408	suppress_segment_too_large

SPZ1	5	80319625	80321842	-0.293443042	PASS
SQLE	8	124998497	125022283	0.279445683	PASS
SQOR	15	45631148	45691294	-0.396255771	PASS
SQSTM1	5	179806398	179838078	-0.150979337	PASS
SRA1	5	140537340	140558252	-0.293443042	PASS
SRARP	1	16004236	16008807	0.033763446	PASS
SRBD1	2	45388680	45612165	0.040498426	PASS
SRC	20	37344685	37406050	-0.168668583	PASS
SRCAP	16	30698209	30741409	0.069936844	PASS
SRCIN1	17	38530016	38605930	0.060555011	PASS
SRD5A1	5	6633343	6674386	0.055007813	PASS
SRD5A2	2	31522480	31581067	0.040498426	PASS
SRD5A3	4	55346109	55373096	-0.011382299	PASS
SREBF1	17	17810399	17837002	0.024610076	PASS
SREBF2	22	41833079	41907308	-0.575930959	PASS
SREK1	5	66139971	66183615	-0.293443042	PASS
SREK1IP1	5	64718144	64768685	-0.293443042	PASS
SRF	6	43171299	43181507	0.048820648	PASS
SRFBP1	5	121961961	122075570	-0.293443042	PASS
SRGAP1	12	63844293	64162221	-0.202191512	PASS
SRGAP2	1	206203345	206464443	-0.256698369	PASS
SRGAP2B	1	144887265	145095528	-0.157385092	PASS
SRGAP2C	1	121184810	121392822	-0.157385092	PASS
SRGAP3	3	8980591	9363053	0.311018244	suppress_segment_too_large
SRGN	10	69088106	69104811	0.072405807	PASS
SRI	7	88205118	88226993	-0.262778904	PASS
SRL	16	4189374	4242080	0.069936844	PASS
SRM	1	11054584	11060024	0.373964873	suppress_segment_too_large
SRMS	20	63540810	63547504	-0.168668583	PASS
SRP14	15	40035739	40039188	-0.396255771	PASS
SRP19	5	112861188	112898371	-0.293443042	PASS
SRP54	14	34981957	35029567	0.060212819	PASS
SRP68	17	76038775	76072653	0.088516311	PASS
SRP72	4	56467336	56503681	-0.011382299	PASS
SRP9	1	225777813	225790466	-0.256698369	PASS
SRPK1	6	35832966	35921342	0.048820648	PASS
SRPK2	7	105110704	105399308	-0.388946609	PASS
SRPK3	23	153776412	153785732	-0.012567603	PASS
SRPRA	11	126262919	126269144	-0.419612865	PASS
SRPRB	3	133784033	133825772	0.313454204	suppress_segment_too_large
SRPX	23	38149336	38220924	-0.012567603	PASS
SRPX2	23	100644166	100675788	-0.012567603	PASS
SRR	17	2303383	2325260	0.128406487	PASS
SRRD	22	26483877	26494658	-0.575930959	PASS
SRRM1	1	24631716	24673267	0.033763446	PASS
SRRM2	16	2752329	2772538	0.069936844	PASS

SRRM3	7	76201900	76287288	-0.262778904	PASS
SRRM4	12	118981495	119163051	0.202141228	PASS
SRRM5	19	43596617	43614497	0.14996833	PASS
SRRT	7	100875111	100888664	-0.262778904	PASS
SRSF1	17	58003360	58007346	0.088516311	PASS
SRSF10	1	23964804	23980927	0.033763446	PASS
SRSF11	1	70205682	70253052	0.033763446	PASS
SRSF12	6	89095959	89118081	-0.368590549	PASS
SRSF2	17	76734115	76737374	0.088516311	PASS
SRSF3	6	36594353	36605600	0.048820648	PASS
SRSF4	1	29147743	29181987	0.033763446	PASS
SRSF5	14	69726900	69772005	0.060212819	PASS
SRSF6	20	43457928	43464247	-0.168668583	PASS
SRSF7	2	38743599	38751494	0.040498426	PASS
SRSF8	11	95067197	95071224	0.062969153	PASS
SRSF9	12	120461668	120469793	0.202141228	PASS
SRXN1	20	646615	653370	-0.168668583	PASS
SS18	18	26016253	26091217	-0.376397843	PASS
SS18L1	20	62143795	62182484	-0.168668583	PASS
SS18L2	3	42581840	42595114	-0.234617007	PASS
SSB	2	169791933	169812064	0.039726562	PASS
SSBP1	7	141738321	141787922	-0.388946609	PASS
SSBP2	5	81413021	81751797	-0.293443042	PASS
SSBP3	1	54225432	54413479	0.033763446	PASS
SSBP4	19	18418864	18434562	0.093487731	suppress_segment_too_large
SSC4D	7	76389334	76409695	-0.262778904	PASS
SSC5D	19	55488404	55519098	0.121336758	PASS
SSH1	12	108778192	108857590	0.202141228	PASS
SSH2	17	29625938	29930276	0.060555011	PASS
SSH3	11	67303448	67312607	0.182439925	suppress_segment_too_large
SSMEM1	7	130206344	130216844	-0.388946609	PASS
SSNA1	9	137188647	137190370	0.365190713	suppress_segment_too_large
SSPN	12	26121991	26299290	0.362961408	suppress_segment_too_large
SSPO	7	149776042	149833979	-0.388946609	PASS
SSR1	6	7268306	7347446	0.048820648	PASS
SSR2	1	156009048	156020959	0.103015788	PASS
SSR3	3	156540140	156555184	0.318278996	suppress_segment_too_large
SSR4	23	153793516	153798505	-0.012567603	PASS
SSRP1	11	57325985	57335877	-0.129266491	PASS
SSSCA1	11	65570430	65573942	0.182439925	suppress_segment_too_large
SST	3	187668906	187670399	0.318278996	suppress_segment_too_large
SSTR1	14	38207999	38213067	0.060212819	PASS
SSTR2	17	73165012	73176633	0.088516311	PASS
SSTR3	22	37204238	37212322	-0.575930959	PASS
SSTR4	20	23035386	23036812	-0.168668583	PASS
SSTR5	16	1078781	1080142	0.069936844	PASS

SSU72	1	1541673	1574869	0.373964873	suppress_segment_too_large
SSUH2	3	8619400	8745040	0.311018244	suppress_segment_too_large
SSX1	23	48255317	48267444	-0.012567603	PASS
SSX2	23	52696896	52707189	-0.012567603	PASS
SSX2B	23	52751132	52790305	-0.012567603	PASS
SSX2IP	1	84643707	84690803	0.033763446	PASS
SSX3	23	48346428	48356707	-0.012567603	PASS
SSX4	23	48383516	48393347	-0.012567603	PASS
SSX4B	23	48402078	48411910	-0.012567603	PASS
SSX5	23	48186220	48196763	-0.012567603	PASS
SSX7	23	52644090	52654900	-0.012567603	PASS
ST13	22	40824535	40857022	-0.575930959	PASS
ST14	11	130159562	130210376	-0.419612865	PASS
ST18	8	52110839	52460959	-0.201142258	PASS
ST20	15	79898840	79923702	-0.697600509	PASS
ST20-MTH	15	79845150	79923754	-0.697600509	PASS
ST3GAL1	8	133454848	133571940	0.113969953	PASS
ST3GAL2	16	70375978	70439237	-0.371357732	PASS
ST3GAL3	1	43705824	43931165	0.033763446	PASS
ST3GAL4	11	126355640	126440344	-0.419612865	PASS
ST3GAL5	2	85837120	85905199	0.066915641	PASS
ST3GAL6	3	98732236	98821201	0.313454204	suppress_segment_too_large
ST5	11	8693351	8910951	-0.405444717	PASS
ST6GAL1	3	186930485	187078553	0.318278996	suppress_segment_too_large
ST6GAL2	2	106801600	106887108	-0.532032081	PASS
ST6GALN.	17	76624761	76643838	0.088516311	PASS
ST6GALN.	17	76565379	76586956	0.088516311	PASS
ST6GALN.	1	76074719	76634601	0.033763446	PASS
ST6GALN.	9	127907886	127917038	0.365190713	suppress_segment_too_large
ST6GALN.	1	76867441	77065711	0.033763446	PASS
ST6GALN.	9	127885321	127905408	0.365190713	suppress_segment_too_large
ST7	7	116953238	117230103	-0.388946609	PASS
ST7L	1	112523518	112620825	0.033763446	PASS
ST8SIA1	12	22063773	22437041	0.362961408	suppress_segment_too_large
ST8SIA2	15	92393828	92468728	-0.697600509	PASS
ST8SIA3	18	57350813	57371731	-0.376397843	PASS
ST8SIA4	5	100806935	100903266	-0.293443042	PASS
ST8SIA5	18	46667821	46759257	-0.376397843	PASS
ST8SIA6	10	17318383	17454330	0.039811807	PASS
STAB1	3	52495338	52524495	-0.234617007	PASS
STAB2	12	103587273	103766727	0.202141228	PASS
STAC	3	36380344	36548007	-0.234617007	PASS
STAC2	17	39210536	39225872	0.060555011	PASS
STAC3	12	57243453	57251193	-0.202191512	PASS
STAG1	3	136336233	136752403	0.313454204	suppress_segment_too_large
STAG2	23	123960212	124422664	-0.012567603	PASS

STAG3	7	100177563	100221488	-0.262778904	PASS
STAM	10	17644125	17715914	0.039811807	PASS
STAM2	2	152116801	152175992	-0.475215623	PASS
STAMBP	2	73828916	73873659	-0.372959126	PASS
STAMBPL	10	88879734	88975153	-1.64589449	suppress_large_homdel
STAP1	4	67558728	67607337	-0.011382299	PASS
STAP2	19	4324043	4342786	0.079632875	PASS
STAR	8	38143649	38151265	-0.201142258	PASS
STARD10	11	72754729	72793681	0.062969153	PASS
STARD13	13	33103135	33350630	-0.390786075	PASS
STARD3	17	39637065	39663484	0.060555011	PASS
STARD3N	7	38178222	38230671	-0.69513118	PASS
STARD4	5	111496033	111512590	-0.293443042	PASS
STARD5	15	81309053	81324183	-0.697600509	PASS
STARD6	18	54324358	54357964	-0.376397843	PASS
STARD7	2	96184859	96208825	0.048238121	PASS
STARD8	23	68647666	68725842	-0.012567603	PASS
STARD9	15	42575659	42720981	-0.396255771	PASS
STAT1	2	190964358	191020960	0.025928102	PASS
STAT2	12	56341597	56360155	-0.202191512	PASS
STAT3	17	42313324	42388568	0.060555011	PASS
STAT4	2	191029576	191151596	0.025928102	PASS
STAT5A	17	42287547	42311943	0.060555011	PASS
STAT5B	17	42199168	42276707	0.060555011	PASS
STAT6	12	57095408	57132139	-0.202191512	PASS
STATH	4	69995930	70002570	-0.011382299	PASS
STAU1	20	49113339	49188367	-0.168668583	PASS
STAU2	8	73420369	73747708	-0.201142258	PASS
STBD1	4	76306026	76311599	-0.011382299	PASS
STC1	8	23841915	23854807	-0.345962679	PASS
STC2	5	173314713	173329503	-0.150979337	PASS
STEAP1	7	90154375	90164829	-0.262778904	PASS
STEAP1B	7	22419444	22727613	-0.69513118	PASS
STEAP2	7	90167590	90238137	-0.262778904	PASS
STEAP3	2	119223831	119265652	-0.475215623	PASS
STEAP4	7	88270892	88306891	-0.262778904	PASS
STH	17	45999250	45999694	0.088516311	PASS
STIL	1	47250139	47314147	0.033763446	PASS
STIM1	11	3854527	4093210	-0.333835828	PASS
STIM2	4	26857678	27025381	-0.458181871	PASS
STIMATE	3	52836219	52897596	-0.234617007	PASS
STIP1	11	64185272	64204543	0.182439925	suppress_segment_too_large
STK10	5	172042073	172188386	-0.150979337	PASS
STK11	19	1177558	1228435	0.079632875	PASS
STK11IP	2	219597860	219616451	0.025928102	PASS
STK16	2	219245455	219250337	0.025928102	PASS

STK17A	7	43582758	43650713	-0.69513118	PASS
STK17B	2	196133566	196176503	0.025928102	PASS
STK19	6	31971091	31982821	0.048820648	PASS
STK24	13	98445185	98577940	-0.390786075	PASS
STK26	23	132023265	132075943	-0.012567603	PASS
STK3	8	98371228	98942827	0.279445683	PASS
STK31	7	23710167	23832513	-0.69513118	PASS
STK32A	5	147234963	147387852	-0.293443042	PASS
STK32B	4	5051442	5500998	0.279089364	PASS
STK32C	10	132207492	132331847	0.063874692	PASS
STK33	11	8391868	8594289	-0.405444717	PASS
STK35	20	2101611	2177038	-0.168668583	PASS
STK36	2	218672026	218702716	0.025928102	PASS
STK38	6	36493892	36547470	0.048820648	PASS
STK38L	12	27243968	27325959	0.362961408	suppress_segment_too_large
STK39	2	167954020	168248141	0.039726562	PASS
STK4	20	44966474	45079959	-0.168668583	PASS
STK40	1	36339624	36385896	0.033763446	PASS
STKLD1	9	133376367	133406096	0.365190713	suppress_segment_too_large
STMN1	1	25884181	25906991	0.033763446	PASS
STMN2	8	79610814	79666175	0.279445683	PASS
STMN3	20	63639705	63657682	-0.168668583	PASS
STMN4	8	27235323	27258420	-0.345962679	PASS
STMND1	6	17102258	17131372	0.048820648	PASS
STMP1	7	135662496	135693418	-0.388946609	PASS
STN1	10	103882542	103918205	-0.367947924	PASS
STOM	9	121338988	121370304	0.365190713	suppress_segment_too_large
STOML1	15	73978923	73994622	-0.697600509	PASS
STOML2	9	35099776	35103195	-0.310444659	PASS
STOML3	13	38965925	38991066	-0.390786075	PASS
STON1	2	48529383	48598513	0.040498426	PASS
STON1-GT	2	48529925	48776517	0.040498426	PASS
STON2	14	81260656	81436465	-0.192197564	PASS
STOX1	10	68827541	68895432	0.072405807	PASS
STOX2	4	183797692	184023526	-0.462744716	PASS
STPG1	1	24356999	24416934	0.033763446	PASS
STPG2	4	97184093	98143240	-0.011382299	PASS
STPG3	9	137251261	137253483	0.365190713	suppress_segment_too_large
STPG4	2	47045538	47155378	0.040498426	PASS
STRA6	15	74179466	74212267	-0.697600509	PASS
STRA8	7	135231979	135258492	-0.388946609	PASS
STRADA	17	63682336	63741986	0.088516311	PASS
STRADB	2	201387858	201480846	0.025928102	PASS
STRAP	12	15882391	15903478	0.362961408	suppress_segment_too_large
STRBP	9	123109500	123268576	0.365190713	suppress_segment_too_large
STRC	15	43599563	43618800	-0.396255771	PASS

STRIP1	1	110031577	110074641	0.033763446	PASS
STRIP2	7	129434433	129488399	-0.388946609	PASS
STRN	2	36843640	36966472	0.040498426	PASS
STRN3	14	30893799	31026401	0.060212819	PASS
STRN4	19	46719507	46746994	0.14996833	PASS
STS	23	7219456	7354810	-0.012567603	PASS
STT3A	11	125591712	125625215	-0.419612865	PASS
STT3B	3	31532638	31637622	0.311018244	suppress_segment_too_large
STUB1	16	680224	682870	0.069936844	PASS
STUM	1	226548800	226609214	-0.256698369	PASS
STX10	19	13144058	13150383	0.093487731	suppress_segment_too_large
STX11	6	144150526	144188370	-0.035448111	PASS
STX12	1	27773183	27824452	0.033763446	PASS
STX16	20	58651253	58679526	-0.168668583	PASS
STX16-NP	20	58651434	58715410	-0.168668583	PASS
STX17	9	99906633	99970341	0.365190713	suppress_segment_too_large
STX18	4	4415742	4542346	0.279089364	PASS
STX19	3	94014369	94028610	0.313454204	suppress_segment_too_large
STX1A	7	73699206	73719672	-0.262778904	PASS
STX1B	16	30989256	31010661	0.069936844	PASS
STX2	12	130789600	130839266	0.270848837	PASS
STX3	11	59713456	59805882	-0.129266491	PASS
STX4	16	31032889	31042975	0.069936844	PASS
STX5	11	62806897	62832088	0.182439925	suppress_segment_too_large
STX6	1	180972712	181023121	-0.027073548	PASS
STX7	6	132445867	132513198	-0.458570262	PASS
STX8	17	9250471	9576591	-0.139031004	PASS
STXBP1	9	127579370	127696027	0.365190713	suppress_segment_too_large
STXBP2	19	7636881	7647873	0.079632875	PASS
STXBP3	1	108746674	108809526	0.033763446	PASS
STXBP4	17	54968727	55173632	0.088516311	PASS
STXBP5	6	147204425	147390476	-0.035448111	PASS
STXBP5L	3	120908072	121424761	0.313454204	suppress_segment_too_large
STXBP6	14	24809656	25050297	0.060212819	PASS
STYK1	12	10618939	10674318	0.362961408	suppress_segment_too_large
STYX	14	52730180	52774998	0.060212819	PASS
STYXL1	7	75996338	76048004	-0.262778904	PASS
SUB1	5	32531633	32604079	0.055007813	PASS
SUCLA2	13	47745736	48037968	-0.390786075	PASS
SUCLG1	2	84423523	84460045	0.066915641	PASS
SUCLG2	3	67360460	67654614	-0.234617007	PASS
SUCNR1	3	151873643	151884619	0.318278996	suppress_segment_too_large
SUCO	1	172532349	172611833	-0.027073548	PASS
SUDS3	12	118376380	118418031	0.202141228	PASS
SUFU	10	102503987	102633535	-0.367947924	PASS
SUGCT	7	40134977	40860763	-0.69513118	PASS

SUGP1	19	19276018	19320844	0.093487731	suppress_segment_too_large
SUGP2	19	18990888	19034023	0.093487731	suppress_segment_too_large
SUGT1	13	52652709	52700909	-0.390786075	PASS
SULF1	8	69466624	69660915	-0.201142258	PASS
SULF2	20	47656348	47786616	-0.168668583	PASS
SULT1A1	16	28605196	28623625	0.069936844	PASS
SULT1A2	16	28591943	28597109	0.069936844	PASS
SULT1A4	16	29459889	29464976	0.069936844	PASS
SULT1B1	4	69721162	69787961	-0.011382299	PASS
SULT1C2	2	108288639	108309915	-0.010901494	PASS
SULT1C3	2	108247195	108265351	-0.010901494	PASS
SULT1C4	2	108377911	108388057	-0.010901494	PASS
SULT1E1	4	69841212	69860152	-0.011382299	PASS
SULT2A1	19	47870466	47886397	0.14996833	PASS
SULT2B1	19	48552075	48599425	0.14996833	PASS
SULT4A1	22	43824509	43862503	-0.808211634	PASS
SULT6B1	2	37167820	37196598	0.040498426	PASS
SUMF1	3	3700814	4467281	0.311018244	suppress_segment_too_large
SUMF2	7	56064002	56080670	-0.750061359	PASS
SUMO1	2	202206180	202238608	0.025928102	PASS
SUMO2	17	75165586	75182983	0.088516311	PASS
SUMO3	21	44805617	44818779	-0.064425456	suppress_segment_too_large
SUMO4	6	149400227	149401292	-0.035448111	PASS
SUN1	7	816615	896435	-0.69513118	PASS
SUN2	22	38734725	38794143	-0.575930959	PASS
SUN3	7	47987148	48029119	-0.69513118	PASS
SUN5	20	32983773	33004433	-0.168668583	PASS
SUOX	12	55997180	56006641	-0.202191512	PASS
SUPT16H	14	21351472	21384266	0.060212819	PASS
SUPT20H	13	37009312	37059713	-0.390786075	PASS
SUPT3H	6	44809317	45377953	0.048820648	PASS
SUPT4H1	17	58345175	58353093	0.088516311	PASS
SUPT5H	19	39436156	39476670	0.093487731	suppress_segment_too_large
SUPT6H	17	28662091	28702684	0.060555011	PASS
SUPT7L	2	27650812	27663840	0.040498426	PASS
SUPV3L1	10	69180232	69209099	0.072405807	PASS
SURF1	9	133351755	133356676	0.365190713	suppress_segment_too_large
SURF2	9	133356552	133361169	0.365190713	suppress_segment_too_large
SURF4	9	133361449	133376166	0.365190713	suppress_segment_too_large
SURF6	9	133328774	133336398	0.365190713	suppress_segment_too_large
SUSD1	9	112040785	112175408	0.365190713	suppress_segment_too_large
SUSD2	22	24181259	24189110	-0.575930959	PASS
SUSD3	9	93058688	93085138	0.365190713	suppress_segment_too_large
SUSD4	1	223220819	223364202	-0.256698369	PASS
SUSD5	3	33150042	33219215	0.311018244	suppress_segment_too_large
SUSD6	14	69611596	69715142	0.060212819	PASS

SUV39H1	23	48695554	48709012	-0.012567603	PASS
SUV39H2	10	14878820	14904315	0.039811807	PASS
SUZ12	17	31937018	32001045	0.060555011	PASS
SV2A	1	149903318	149917882	0.103015788	PASS
SV2B	15	91099950	91301309	-0.697600509	PASS
SV2C	5	76083172	76353939	-0.293443042	PASS
SVBP	1	42807052	42817252	0.033763446	PASS
SVEP1	9	110365251	110579880	0.365190713	suppress_segment_too_large
SVIL	10	29457338	29736781	0.039811807	PASS
SVIP	11	22813799	22830299	-0.405444717	PASS
SVOP	12	108907741	109021240	0.202141228	PASS
SVOPL	7	138594285	138701352	-0.388946609	PASS
SWAP70	11	9664077	9752991	-0.405444717	PASS
SWI5	9	128275379	128288990	0.365190713	suppress_segment_too_large
SWSAP1	19	11374685	11376951	0.093487731	suppress_segment_too_large
SWT1	1	185157080	185291765	-0.027073548	PASS
SYAP1	23	16719632	16765336	-0.012567603	PASS
SYBU	8	109573978	109691791	0.279445683	PASS
SYCE1	10	133553901	133569835	0.063874692	PASS
SYCE1L	16	77199397	77213215	-0.371357732	PASS
SYCE2	19	12898786	12919276	0.093487731	suppress_segment_too_large
SYCE3	22	50551112	50562905	-0.808211634	PASS
SYCN	19	39202831	39204266	0.093487731	suppress_segment_too_large
SYCP1	1	114854803	114995370	0.033763446	PASS
SYCP2	20	59863563	59933655	-0.168668583	PASS
SYCP2L	6	10886831	10979320	0.048820648	PASS
SYCP3	12	101728648	101739472	0.202141228	PASS
SYDE1	19	15107403	15114988	0.093487731	suppress_segment_too_large
SYDE2	1	85156873	85201046	0.033763446	PASS
SYF2	1	25222679	25232502	0.033763446	PASS
SYK	9	90801787	90898549	0.365190713	suppress_segment_too_large
SYMPK	19	45815410	45863290	0.14996833	PASS
SYN1	23	47571898	47619943	-0.012567603	PASS
SYN2	3	12004402	12191400	0.311018244	suppress_segment_too_large
SYN3	22	32512552	33058372	-0.575930959	PASS
SYNC	1	32680360	32703596	0.033763446	PASS
SYNCRIP	6	85607785	85643792	-0.368590549	PASS
SYNDIG1	20	24469199	24666616	-0.168668583	PASS
SYNDIG1I	14	74405893	74426102	-0.192197564	PASS
SYNE1	6	152121684	152637801	-0.035448111	PASS
SYNE2	14	63852983	64226433	0.060212819	PASS
SYNE3	14	95407266	95475836	-0.192197564	PASS
SYNE4	19	36003299	36008793	0.093487731	suppress_segment_too_large
SYNGAP1	6	33419661	33457541	0.048820648	PASS
SYNGR1	22	39349925	39385588	-0.575930959	PASS
SYNGR2	17	78168558	78173527	0.088516311	PASS

SYNGR3	16	1989660	1994275	0.069936844	PASS
SYNGR4	19	48364395	48376376	0.14996833	PASS
SYNJ1	21	32628759	32728048	0.12860881	suppress_segment_too_large
SYNJ2	6	157981887	158099176	-0.035448111	PASS
SYNJ2BP	14	70366496	70417061	0.060212819	PASS
SYNJ2BP-(	14	70326064	70417074	0.060212819	PASS
SYNM	15	99098217	99135593	-0.697600509	PASS
SYNPO	5	150601080	150659220	-0.293443042	PASS
SYNPO2	4	118850688	119061247	-0.011382299	PASS
SYNPO2L	10	73644881	73663803	0.072405807	PASS
SYNPR	3	63228315	63616921	-0.234617007	PASS
SYNRG	17	37514797	37609496	0.060555011	PASS
SYP	23	49187804	49200259	-0.012567603	PASS
SYPL1	7	106090503	106112576	-0.388946609	PASS
SYPL2	1	109466628	109482137	0.033763446	PASS
SYS1	20	45361937	45376798	-0.168668583	PASS
SYS1-DBN	20	45363200	45410610	-0.168668583	PASS
SYT1	12	78863993	79452008	0.202141228	PASS
SYT10	12	33374238	33439819	0.362961408	suppress_segment_too_large
SYT11	1	155859509	155885199	0.103015788	PASS
SYT12	11	67006778	67050863	0.182439925	suppress_segment_too_large
SYT13	11	45240301	45286319	0.117376172	PASS
SYT14	1	209938174	210171389	-0.256698369	PASS
SYT15	10	46578217	46594173	-0.404852538	PASS
SYT16	14	61811974	62112550	0.060212819	PASS
SYT17	16	19167971	19268334	0.069936844	PASS
SYT2	1	202590596	202710417	-0.256698369	PASS
SYT3	19	50621307	50639827	-0.016761391	PASS
SYT4	18	43267878	43277650	-0.376397843	PASS
SYT5	19	55171199	55180441	0.121336758	PASS
SYT6	1	114089291	114153919	0.033763446	PASS
SYT7	11	61515313	61581148	0.182439925	suppress_segment_too_large
SYT8	11	1828307	1837521	-0.333835828	PASS
SYT9	11	7238778	7469042	-0.405444717	PASS
SYTL1	1	27342020	27353937	0.033763446	PASS
SYTL2	11	85694224	85811159	0.062969153	PASS
SYTL3	6	158650014	158764876	-0.035448111	PASS
SYTL4	23	100674491	100732123	-0.012567603	PASS
SYTL5	23	38006582	38128819	-0.012567603	PASS
SYVN1	11	65121780	65134533	0.182439925	suppress_segment_too_large
SZRD1	1	16352575	16398145	0.033763446	PASS
SZT2	1	43389882	43454247	0.033763446	PASS
TAAR1	6	132644984	132646003	-0.458570262	PASS
TAAR2	6	132617022	132624275	-0.458570262	PASS
TAAR5	6	132588673	132589686	-0.458570262	PASS
TAAR6	6	132570322	132571359	-0.458570262	PASS

TAAR8	6	132552693	132553721	-0.458570262	PASS
TAAR9	6	132538290	132539336	-0.458570262	PASS
TAB1	22	39399741	39437060	-0.575930959	PASS
TAB2	6	149218641	149411613	-0.035448111	PASS
TAB3	23	30827442	30975084	-0.012567603	PASS
TAC1	7	97731908	97740472	-0.262778904	PASS
TAC3	12	57010000	57028883	-0.202191512	PASS
TAC4	17	49838309	49848017	0.088516311	PASS
TACC1	8	38728186	38853028	-0.201142258	PASS
TACC2	10	121989174	122254545	0.063874692	PASS
TACC3	4	1721490	1745176	0.279089364	PASS
TACO1	17	63600872	63608365	0.088516311	PASS
TACR1	2	75046463	75199700	-0.372959126	PASS
TACR2	10	69403903	69416867	0.072405807	PASS
TACR3	4	103586031	103719816	-0.011382299	PASS
TACSTD2	1	58575423	58577773	0.033763446	PASS
TADA1	1	166856510	166876327	-0.027073548	PASS
TADA2A	17	37406874	37479730	0.060555011	PASS
TADA2B	4	7041899	7057952	0.279089364	PASS
TADA3	3	9779860	9793011	0.311018244	suppress_segment_too_large
TAF1	23	71366239	71532374	-0.012567603	PASS
TAF10	11	6606296	6612667	-0.405444717	PASS
TAF11	6	34877462	34888089	0.048820648	PASS
TAF12	1	28589323	28643085	0.033763446	PASS
TAF13	1	109062486	109076002	0.033763446	PASS
TAF15	17	35713791	35864615	0.060555011	PASS
TAF1A	1	222557902	222589933	-0.256698369	PASS
TAF1B	2	9843354	9934416	0.040498426	PASS
TAF1C	16	84177847	84187070	-0.371357732	PASS
TAF1D	11	93729948	93784391	0.062969153	PASS
TAF1L	9	32629454	32635669	-0.310444659	PASS
TAF2	8	119730775	119832863	0.279445683	PASS
TAF3	10	7818504	8016627	0.039811807	PASS
TAF4	20	61953469	62065810	-0.168668583	PASS
TAF4B	18	26225936	26391685	-0.376397843	PASS
TAF5	10	103367967	103389065	-0.367947924	PASS
TAF5L	1	229593111	229626047	-0.256698369	PASS
TAF6	7	100107070	100119841	-0.262778904	PASS
TAF6L	11	62771303	62787342	0.182439925	suppress_segment_too_large
TAF7	5	141260225	141320821	-0.293443042	PASS
TAF7L	23	101268253	101293057	-0.012567603	PASS
TAF8	6	42050513	42087461	0.048820648	PASS
TAF9	5	69364743	69370013	-0.293443042	PASS
TAF9B	23	78129748	78139706	-0.012567603	PASS
TAGAP	6	159034468	159045152	-0.035448111	PASS
TAGLN	11	117199321	117204782	-0.356188624	PASS

TAGLN2	1	159918107	159925732	-0.027073548	PASS
TAGLN3	3	111998664	112013887	0.313454204	suppress_segment_too_large
TAL1	1	47216290	47232220	0.033763446	PASS
TAL2	9	105662457	105663112	0.365190713	suppress_segment_too_large
TALDO1	11	747329	765024	-0.333835828	PASS
TAMM41	3	11790442	11846919	0.311018244	suppress_segment_too_large
TANC1	2	158968671	159232659	0.039726562	PASS
TANC2	17	63009556	63427699	0.088516311	PASS
TANGO2	22	20017014	20065926	-0.575930959	PASS
TANGO6	16	68843604	69085180	-0.371357732	PASS
TANK	2	161136908	161236221	0.039726562	PASS
TAOK1	17	29390464	29551904	0.060555011	PASS
TAOK2	16	29973641	29992261	0.069936844	PASS
TAOK3	12	118149801	118372945	0.202141228	PASS
TAP1	6	32845209	32853978	0.048820648	PASS
TAP2	6	32821833	32838780	0.048820648	PASS
TAPBP	6	33299694	33314387	0.048820648	PASS
TAPBPL	12	6451690	6466517	-0.086128028	PASS
TAPT1	4	16160505	16227410	0.279089364	PASS
TARBP1	1	234391313	234479103	-0.256698369	PASS
TARBP2	12	53500921	53506431	-0.202191512	PASS
TARDBP	1	11012344	11026420	0.373964873	suppress_segment_too_large
TARM1	19	54069895	54081365	0.206912349	PASS
TARS	5	33440696	33469539	0.055007813	PASS
TARS2	1	150487364	150507609	0.103015788	PASS
TARSL2	15	101653598	101724604	-0.697600509	PASS
TAS1R1	1	6555181	6579757	0.373964873	suppress_segment_too_large
TAS1R2	1	18839599	18859682	0.033763446	PASS
TAS1R3	1	1331314	1335306	0.373964873	suppress_segment_too_large
TAS2R1	5	9628997	9712378	0.055007813	PASS
TAS2R10	12	10825317	10826358	0.362961408	suppress_segment_too_large
TAS2R13	12	10907926	10909562	0.362961408	suppress_segment_too_large
TAS2R14	12	10937406	11171573	0.362961408	suppress_segment_too_large
TAS2R16	7	122994704	122995700	-0.388946609	PASS
TAS2R19	12	11021619	11022620	0.362961408	suppress_segment_too_large
TAS2R20	12	10996495	10997875	0.362961408	suppress_segment_too_large
TAS2R3	7	141764097	141765197	-0.388946609	PASS
TAS2R30	12	11132958	11134644	0.362961408	suppress_segment_too_large
TAS2R31	12	11030387	11031407	0.362961408	suppress_segment_too_large
TAS2R38	7	141972631	141973773	-0.388946609	PASS
TAS2R39	7	143183419	143184435	-0.388946609	PASS
TAS2R4	7	141778442	141780819	-0.388946609	PASS
TAS2R40	7	143222037	143223079	-0.388946609	PASS
TAS2R41	7	143477873	143478796	-0.388946609	PASS
TAS2R42	12	11185993	11186937	0.362961408	suppress_segment_too_large
TAS2R46	12	11061365	11062294	0.362961408	suppress_segment_too_large

TAS2R5	7	141790217	141791367	-0.388946609	PASS
TAS2R50	12	10985913	10986912	0.362961408	suppress_segment_too_large
TAS2R60	7	143443453	143444409	-0.388946609	PASS
TAS2R7	12	10801532	10802627	0.362961408	suppress_segment_too_large
TAS2R8	12	10806051	10807293	0.362961408	suppress_segment_too_large
TAS2R9	12	10809094	10810168	0.362961408	suppress_segment_too_large
TASP1	20	13389392	13638940	-0.168668583	PASS
TAT	16	71565660	71577130	-0.371357732	PASS
TATDN1	8	124488485	124539458	0.279445683	PASS
TATDN2	3	10248023	10281218	0.311018244	suppress_segment_too_large
TATDN3	1	212791828	212816626	-0.256698369	PASS
TAX1BP1	7	27739331	27844564	-0.69513118	PASS
TAX1BP3	17	3662896	3668682	0.128406487	PASS
TAZ	23	154411518	154421726	-0.012567603	PASS
TBATA	10	70771239	70785401	0.072405807	PASS
TBC1D1	4	37891087	38139175	-0.011382299	PASS
TBC1D10[illegible]	22	30291990	30327046	-0.575930959	PASS
TBC1D10[illegible]	16	30357102	30370264	0.069936844	PASS
TBC1D10[illegible]	11	67403915	67410089	0.182439925	suppress_segment_too_large
TBC1D12	10	94402504	94535930	-0.39678585	PASS
TBC1D13	9	128787204	128810432	0.365190713	suppress_segment_too_large
TBC1D14	4	6909242	7033118	0.279089364	PASS
TBC1D15	12	71839707	71927248	-0.202191512	PASS
TBC1D16	17	79932343	80035848	0.088516311	PASS
TBC1D17	19	49877425	49888749	0.14996833	PASS
TBC1D19	4	26576437	26755351	-0.458181871	PASS
TBC1D2	9	98198999	98255721	0.365190713	suppress_segment_too_large
TBC1D20	20	435480	462553	-0.168668583	PASS
TBC1D21	15	73873608	73889214	-0.697600509	PASS
TBC1D22[illegible]	22	46762617	47175699	-0.808211634	PASS
TBC1D22[illegible]	6	37257772	37332970	0.048820648	PASS
TBC1D23	3	100261000	100325251	0.313454204	suppress_segment_too_large
TBC1D24	16	2475051	2509560	0.069936844	PASS
TBC1D25	23	48539457	48562609	-0.012567603	PASS
TBC1D26	17	15732247	15749192	-0.139031004	PASS
TBC1D28	17	18635006	18661950	0.024610076	PASS
TBC1D29	17	30553502	30563493	0.060555011	PASS
TBC1D2B	15	77984036	78077724	-0.697600509	PASS
TBC1D30	12	64780516	64881032	-0.202191512	PASS
TBC1D31	8	123041968	123152153	0.279445683	PASS
TBC1D32	6	121079494	121334745	-0.368590549	PASS
TBC1D3B	17	36165681	36176636	0.060555011	PASS
TBC1D3E	17	38127951	38138862	0.060555011	PASS
TBC1D3F	17	36428618	36439566	0.060555011	PASS
TBC1D3G	17	36323884	36334759	0.060555011	PASS
TBC1D3I	17	36253456	36264553	0.060555011	PASS

TBC1D3K	17	37924415	37935365	0.060555011	PASS
TBC1D3L	17	37977972	37989048	0.060555011	PASS
TBC1D4	13	75283503	75482114	-0.390786075	PASS
TBC1D5	3	17157162	18444817	0.311018244	suppress_segment_too_large
TBC1D7	6	13266542	13328583	0.048820648	PASS
TBC1D8	2	101007617	101252866	0.048238121	PASS
TBC1D8B	23	106802680	106876145	-0.012567603	PASS
TBC1D9	4	140620765	140756120	-0.011382299	PASS
TBC1D9B	5	179862066	179907859	-0.150979337	PASS
TBCA	5	77691166	77868780	-0.293443042	PASS
TBCB	19	36114289	36125947	0.093487731	suppress_segment_too_large
TBCC	6	42744481	42746096	0.048820648	PASS
TBCCD1	3	186546073	186570543	0.318278996	suppress_segment_too_large
TBCD	17	82752064	82945922	0.088516311	PASS
TBCE	1	235328570	235452443	-0.256698369	PASS
TBCEL	11	121024072	121090775	-0.356188624	PASS
TBCK	4	106041599	106321495	-0.011382299	PASS
TBK1	12	64451880	64502108	-0.202191512	PASS
TBKBP1	17	47694081	47712050	0.088516311	PASS
TBL1X	23	9463295	9741037	-0.012567603	PASS
TBL1XR1	3	177019340	177228000	0.318278996	suppress_segment_too_large
TBL2	7	73567533	73578791	-0.262778904	PASS
TBL3	16	1972037	1982933	0.069936844	PASS
TBP	6	170554302	170572870	-0.035448111	PASS
TBPL1	6	133952170	133990432	-0.035448111	PASS
TBPL2	14	55413541	55456726	0.060212819	PASS
TBR1	2	161416094	161425870	0.039726562	PASS
TBRG1	11	124622836	124635398	-0.419612865	PASS
TBRG4	7	45100100	45112047	-0.69513118	PASS
TBX1	22	19756703	19783593	-0.575930959	PASS
TBX10	11	67631303	67639560	0.182439925	suppress_segment_too_large
TBX15	1	118883046	118989556	-0.157385092	PASS
TBX18	6	84687351	84764519	-0.368590549	PASS
TBX19	1	168281040	168314426	-0.027073548	PASS
TBX2	17	61399896	61409466	0.088516311	PASS
TBX20	7	35202430	35254147	-0.69513118	PASS
TBX21	17	47733244	47746119	0.088516311	PASS
TBX22	23	80014756	80031769	-0.012567603	PASS
TBX3	12	114670254	114684164	0.202141228	PASS
TBX4	17	61452404	61485110	0.088516311	PASS
TBX5	12	114353931	114408442	0.202141228	PASS
TBX6	16	30085793	30091887	0.069936844	PASS
TBXA2R	19	3594506	3606840	0.079632875	PASS
TBXAS1	7	139777051	140020325	-0.388946609	PASS
TBXT	6	166157656	166168700	-0.035448111	PASS
TC2N	14	91779751	91867536	-0.192197564	PASS

TCAF1	7	143851375	143902198	-0.388946609	PASS
TCAF2	7	143620952	143730409	-0.388946609	PASS
TCAIM	3	44338119	44409451	-0.234617007	PASS
TCAP	17	39664187	39666555	0.060555011	PASS
TCEA1	8	53966552	54022529	-0.201142258	PASS
TCEA2	20	64049836	64072347	-0.168668583	PASS
TCEA3	1	23381061	23424740	0.033763446	PASS
TCEAL1	23	103628704	103630953	-0.012567603	PASS
TCEAL2	23	102125688	102127711	-0.012567603	PASS
TCEAL3	23	103607451	103629690	-0.012567603	PASS
TCEAL4	23	103576231	103587736	-0.012567603	PASS
TCEAL5	23	103273691	103276872	-0.012567603	PASS
TCEAL6	23	102140476	102142970	-0.012567603	PASS
TCEAL7	23	103330196	103332326	-0.012567603	PASS
TCEAL8	23	103252995	103255203	-0.012567603	PASS
TCEAL9	23	103356489	103358462	-0.012567603	PASS
TCEANC	23	13653189	13681964	-0.012567603	PASS
TCEANC2	1	54053584	54112519	0.033763446	PASS
TCERG1	5	146447311	146511961	-0.293443042	PASS
TCERG1L	10	131092391	131311721	0.063874692	PASS
TCF12	15	56918623	57299281	-0.697600509	PASS
TCF15	20	603797	610398	-0.168668583	PASS
TCF19	6	31158542	31167159	0.048820648	PASS
TCF20	22	42160013	42343616	-0.575930959	PASS
TCF21	6	133889138	133895553	-0.035448111	PASS
TCF23	2	27149004	27156974	0.040498426	PASS
TCF24	8	66946501	66962590	-0.201142258	PASS
TCF25	16	89873570	89913627	-0.371357732	PASS
TCF3	19	1609290	1652605	0.079632875	PASS
TCF4	18	55222331	55664787	-0.376397843	PASS
TCF7	5	134114711	134151865	-0.293443042	PASS
TCF7L1	2	85133410	85310388	0.066915641	PASS
TCF7L2	10	112950250	113167678	0.063874692	PASS
TCFL5	20	62841115	62861763	-0.168668583	PASS
TCHH	1	152106317	152115454	0.103015788	PASS
TCHHL1	1	152084144	152089064	0.103015788	PASS
TCHP	12	109900264	109983841	0.202141228	PASS
TCIM	8	40153455	40155308	-0.201142258	PASS
TCIRG1	11	68039016	68050895	0.182439925	suppress_segment_too_large
TCL1A	14	95709967	95714196	-0.192197564	PASS
TCL1B	14	95686417	95692643	-0.192197564	PASS
TCN1	11	59852800	59866575	-0.129266491	PASS
TCN2	22	30606838	30627278	-0.575930959	PASS
TCOF1	5	150357629	150400308	-0.293443042	PASS
TCP1	6	159778498	159789749	-0.035448111	PASS
TCP10	6	167357031	167384510	-0.035448111	PASS

TCP10L	21	32574841	32587373	0.12860881	suppress_segment_too_large
TCP10L2	6	167146414	167196913	-0.035448111	PASS
TCP11	6	35118071	35148610	0.048820648	PASS
TCP11L1	11	33039417	33105943	-0.405444717	PASS
TCP11L2	12	106301929	106347015	0.202141228	PASS
TCP11X2	23	102456862	102471842	-0.012567603	PASS
TCTA	3	49412206	49416475	-0.234617007	PASS
TCTE1	6	44278743	44297688	0.048820648	PASS
TCTE3	6	169740114	169751587	-0.035448111	PASS
TCTEX1D	1	66752459	66778787	0.033763446	PASS
TCTEX1D[illegible]	3	196291219	196318299	0.326834516	PASS
TCTEX1D[illegible]	1	44805913	44806675	0.033763446	PASS
TCTN1	12	110614027	110649430	0.202141228	PASS
TCTN2	12	123671113	123708403	0.202141228	PASS
TCTN3	10	95663396	95694143	-0.39678585	PASS
TDG	12	103965804	103988874	0.202141228	PASS
TDGF1	3	46574534	46582463	-0.234617007	PASS
TDO2	4	155854738	155920406	-0.011382299	PASS
TDP1	14	89954939	90044768	-0.192197564	PASS
TDP2	6	24649977	24667033	0.048820648	PASS
TDRD1	10	114179270	114232304	0.063874692	PASS
TDRD10	1	154502219	154548147	0.103015788	PASS
TDRD12	19	32719753	32829580	0.093487731	suppress_segment_too_large
TDRD15	2	21123917	21143272	0.040498426	PASS
TDRD3	13	60396457	60573878	-0.390786075	PASS
TDRD5	1	179591613	179691272	-0.027073548	PASS
TDRD6	6	46687875	46704319	0.048820648	PASS
TDRD7	9	97411950	97496125	0.365190713	suppress_segment_too_large
TDRD9	14	103928462	104052667	-0.192197564	PASS
TDRKH	1	151770107	151791416	0.103015788	PASS
TDRP	8	489792	545781	-0.313874214	PASS
TEAD1	11	12674591	12944483	-0.405444717	PASS
TEAD2	19	49340595	49362457	0.14996833	PASS
TEAD3	6	35473597	35497076	0.048820648	PASS
TEAD4	12	2959330	3040673	-0.086128028	PASS
TEC	4	48135783	48269864	-0.011382299	PASS
TECPR1	7	98214624	98252251	-0.262778904	PASS
TECPR2	14	102362963	102502481	-0.192197564	PASS
TECR	19	14517085	14565980	0.093487731	suppress_segment_too_large
TECRL	4	64275257	64409468	-0.011382299	PASS
TECTA	11	121101173	121191493	-0.356188624	PASS
TECTB	10	112283400	112305035	0.063874692	PASS
TEDC1	14	105489855	105499575	-0.192197564	PASS
TEDC2	16	2460080	2464963	0.069936844	PASS
TEDDM1	1	182398117	182400616	-0.027073548	PASS
TEF	22	41367333	41399326	-0.575930959	PASS

TEFM	17	30897336	30906820	0.060555011	PASS
TEK	9	27109141	27230175	-0.310444659	PASS
TEKT1	17	6789133	6831761	0.128406487	PASS
TEKT2	1	36084075	36088275	0.033763446	PASS
TEKT3	17	15303811	15341641	-0.139031004	PASS
TEKT4	2	94871433	94876829	-0.340781246	PASS
TEKT5	16	10627501	10694945	0.069936844	PASS
TELO2	16	1493344	1510457	0.069936844	PASS
TEN1	17	75979220	76000586	0.088516311	PASS
TEN1-CDK	17	75979231	76005999	0.088516311	PASS
TENM1	23	124375903	124963817	-0.012567603	PASS
TENM2	5	167284799	168264157	-0.150979337	PASS
TENM3	4	182143987	182803024	-0.462744716	PASS
TENM4	11	78652831	79440948	0.062969153	PASS
TENT2	5	79612120	79686648	-0.293443042	PASS
TENT4A	5	6713007	6757048	0.055007813	PASS
TENT4B	16	50152918	50235310	0.069936844	PASS
TENT5A	6	81491439	81752774	-0.368590549	PASS
TENT5B	1	27005020	27012836	0.033763446	PASS
TENT5C	1	117605934	117628372	0.033763446	PASS
TENT5D	23	80335504	80445311	-0.012567603	PASS
TEP1	14	20365667	20413429	0.060212819	PASS
TEPP	16	57976435	57988116	-0.371357732	PASS
TEPSIN	17	81228277	81239091	0.088516311	PASS
TERB1	16	66754976	66801620	-0.371357732	PASS
TERB2	15	44956702	44979229	-0.396255771	PASS
TERF1	8	73008864	73048122	-0.201142258	PASS
TERF2	16	69355561	69408571	-0.371357732	PASS
TERF2IP	16	75647786	75761872	-0.371357732	PASS
TERT	5	1253147	1295069	0.055007813	PASS
TES	7	116210493	116258783	-0.388946609	PASS
TESC	12	117038923	117099479	0.202141228	PASS
TESK1	9	35605305	35610041	-0.310444659	PASS
TESK2	1	45343883	45491166	0.033763446	PASS
TESMIN	11	68707440	68751564	0.182439925	suppress_segment_too_large
TESPA1	12	54948018	54984746	-0.202191512	PASS
TET1	10	68560656	68694482	0.072405807	PASS
TET2	4	105145875	105279816	-0.011382299	PASS
TET3	2	73986404	74108176	-0.372959126	PASS
TEX10	9	100302077	100352939	0.365190713	suppress_segment_too_large
TEX101	19	43401496	43418597	-0.459876623	PASS
TEX11	23	70528940	70908731	-0.012567603	PASS
TEX12	11	112167372	112172559	-0.356188624	PASS
TEX13A	23	105218929	105220674	-0.012567603	PASS
TEX13B	23	107980864	107982370	-0.012567603	PASS
TEX13C	23	125320120	125325214	-0.012567603	PASS

TEX13D	23	124246249	124336862	-0.012567603	PASS
TEX14	17	58556678	58692055	0.088516311	PASS
TEX15	8	30831544	30913003	-0.345962679	PASS
TEX19	17	82359247	82363776	0.088516311	PASS
TEX2	17	64147227	64263301	0.088516311	PASS
TEX22	14	105398579	105450106	-0.192197564	PASS
TEX26	13	30932703	30975502	-0.390786075	PASS
TEX261	2	70985938	70994945	-0.372959126	PASS
TEX264	3	51662693	51704323	-0.234617007	PASS
TEX29	13	111316184	111344249	-0.390786075	PASS
TEX30	13	102765990	102773811	-0.390786075	PASS
TEX33	22	36991120	37007841	-0.575930959	PASS
TEX35	1	178513077	178548602	-0.027073548	PASS
TEX36	10	125576522	125683144	0.063874692	PASS
TEX37	2	88524651	88529584	-0.340781246	PASS
TEX38	1	46668855	46673594	0.033763446	PASS
TEX43	5	126631722	126636287	-0.293443042	PASS
TEX44	2	231592901	231594283	0.025928102	PASS
TEX45	19	7492976	7508450	0.079632875	PASS
TEX46	1	23010834	23015850	0.033763446	PASS
TEX47	7	88794106	88795721	-0.262778904	PASS
TEX48	9	114666434	114682066	0.365190713	suppress_segment_too_large
TEX49	12	48727435	48765786	-0.202191512	PASS
TEX50	1	173635333	173637134	-0.027073548	PASS
TEX51	2	126898864	126902097	-0.475215623	PASS
TEX9	15	56244009	56445997	-0.697600509	PASS
TF	3	133745956	133796640	0.313454204	suppress_segment_too_large
TFAM	10	58385022	58399221	0.072405807	PASS
TFAP2A	6	10393186	10419659	0.048820648	PASS
TFAP2B	6	50818723	50847613	0.048820648	PASS
TFAP2C	20	56629302	56639283	-0.168668583	PASS
TFAP2D	6	50713828	50772988	0.048820648	PASS
TFAP2E	1	35573370	35595328	0.033763446	PASS
TFAP4	16	4257186	4273075	0.069936844	PASS
TFB1M	6	155257509	155314493	-0.035448111	PASS
TFB2M	1	246540560	246566324	-0.368111568	PASS
TFCP2	12	51093663	51173134	-0.202191512	PASS
TFCP2L1	2	121216587	121285207	-0.475215623	PASS
TFDP1	13	113584721	113641470	-0.390786075	PASS
TFDP2	3	141944428	142149544	-0.356957581	PASS
TFDP3	23	133216669	133218348	-0.012567603	PASS
TFE3	23	49028726	49043486	-0.012567603	PASS
TFEB	6	41683978	41736259	0.048820648	PASS
TFEC	7	115935148	116159896	-0.388946609	PASS
TFF1	21	42362282	42366594	-0.064425456	suppress_segment_too_large
TFF2	21	42346357	42351128	-0.064425456	suppress_segment_too_large

TFF3	21	42311667	42315651	-0.064425456	suppress_segment_too_large
TFG	3	100709331	100748966	0.313454204	suppress_segment_too_large
TFIP11	22	26491225	26512505	-0.575930959	PASS
TFPI	2	187464230	187565760	0.025928102	PASS
TFPI2	7	93885396	93890991	-0.262778904	PASS
TFPT	19	54107013	54115675	0.206912349	PASS
TFR2	7	100620416	100642779	-0.262778904	PASS
TFRC	3	196027183	196082189	0.326834516	PASS
TG	8	132866958	133134903	0.113969953	PASS
TGDS	13	94574051	94596257	-0.390786075	PASS
TGFA	2	70447280	70554193	-0.372959126	PASS
TGFB1	19	41301587	41353911	0.093487731	suppress_segment_too_large
TGFB1I1	16	31471585	31477960	0.069936844	PASS
TGFB2	1	218346235	218444619	-0.256698369	PASS
TGFB3	14	75958099	75982991	-0.192197564	PASS
TGFBI	5	136028895	136063818	-0.293443042	PASS
TGFBR1	9	99104038	99154192	0.365190713	suppress_segment_too_large
TGFBR2	3	30606502	30694142	0.311018244	suppress_segment_too_large
TGFBR3	1	91680343	91906335	0.033763446	PASS
TGFBR3L	19	7916145	7919097	0.079632875	PASS
TGFBRAP	2	105264391	105330034	-0.532032081	PASS
TGIF1	18	3411608	3459978	-0.052305056	suppress_segment_too_large
TGIF2	20	36573488	36593950	-0.168668583	PASS
TGIF2-RAI	20	36574553	36612384	-0.168668583	PASS
TGIF2LX	23	89921882	89922883	-0.012567603	PASS
TGM1	14	24249114	24264432	0.060212819	PASS
TGM2	20	38127387	38166578	-0.168668583	PASS
TGM3	20	2296001	2341078	-0.168668583	PASS
TGM4	3	44874608	44914990	-0.234617007	PASS
TGM5	15	43232595	43266857	-0.396255771	PASS
TGM6	20	2380908	2432753	-0.168668583	PASS
TGM7	15	43276280	43302255	-0.396255771	PASS
TGOLN2	2	85318020	85328425	0.066915641	PASS
TGS1	8	55773142	55825448	-0.201142258	PASS
TH	11	2163929	2171877	-0.333835828	PASS
THADA	2	43230836	43596046	0.040498426	PASS
THAP1	8	42836674	42843325	-0.201142258	PASS
THAP10	15	70881342	70892785	-0.697600509	PASS
THAP11	16	67842082	67844195	-0.371357732	PASS
THAP12	11	76349956	76380971	0.062969153	PASS
THAP2	12	71663009	71680639	-0.202191512	PASS
THAP3	1	6624866	6635586	0.373964873	suppress_segment_too_large
THAP5	7	108554543	108569750	-0.388946609	PASS
THAP6	4	75513946	75550473	-0.011382299	PASS
THAP7	22	20999104	21002196	-0.575930959	PASS
THAP8	19	36034985	36054762	0.093487731	suppress_segment_too_large

THAP9	4	82900684	82919969	-0.011382299	PASS
THBD	20	23045633	23049741	-0.168668583	PASS
THBS1	15	39581079	39599466	-0.396255771	PASS
THBS2	6	169215780	169254044	-0.035448111	PASS
THBS3	1	155195588	155209051	0.103015788	PASS
THBS4	5	79991311	80083287	-0.293443042	PASS
THEG	19	361747	376670	0.079632875	PASS
THEGL	4	56530609	56603507	-0.011382299	PASS
THEM4	1	151873584	151909808	0.103015788	PASS
THEM5	1	151847263	151853697	0.103015788	PASS
THEM6	8	142727203	142736927	0.113969953	PASS
THEMIS	6	127708072	127918631	-0.458570262	PASS
THEMIS2	1	27872543	27886685	0.033763446	PASS
THG1L	5	157731197	157741448	-0.150979337	PASS
THNSL1	10	25016658	25026664	0.039811807	PASS
THNSL2	2	88170295	88186636	-0.340781246	PASS
THOC1	18	214520	268050	-0.052305056	suppress_segment_too_large
THOC2	23	123600561	123733056	-0.012567603	PASS
THOC3	5	175917873	176034680	-0.150979337	PASS
THOC5	22	29505879	29555216	-0.575930959	PASS
THOC6	16	3024027	3027755	0.069936844	PASS
THOC7	3	63833870	63863903	-0.234617007	PASS
THOP1	19	2785460	2815807	0.079632875	PASS
THPO	3	184371935	184381968	0.318278996	suppress_segment_too_large
THRA	17	40058290	40093867	0.060555011	PASS
THRAP3	1	36224416	36305357	0.033763446	PASS
THRB	3	24117153	24495756	0.311018244	suppress_segment_too_large
THRSP	11	78063861	78068351	0.062969153	PASS
THSD1	13	52377167	52416373	-0.390786075	PASS
THSD4	15	71096952	71783383	-0.697600509	PASS
THSD7A	7	11370357	11832198	-0.69513118	PASS
THSD7B	2	136765545	137677717	-0.475215623	PASS
THSD8	19	12802031	12813581	0.093487731	suppress_segment_too_large
THTPA	14	23555988	23560271	0.060212819	PASS
THUMPD1	16	20702816	20742084	0.069936844	PASS
THUMPD2	2	39736060	39779267	0.040498426	PASS
THUMPD3	3	9362842	9386791	0.311018244	suppress_segment_too_large
THY1	11	119417378	119424985	-0.356188624	PASS
THYN1	11	134248279	134253370	-0.419612865	PASS
TIA1	2	70209444	70248660	-0.372959126	PASS
TIAF1	17	29073521	29078857	0.060555011	PASS
TIAL1	10	119571802	119597029	0.063874692	PASS
TIAM1	21	31118416	31559977	0.12860881	suppress_segment_too_large
TIAM2	6	154832697	155257723	-0.035448111	PASS
TICAM1	19	4815932	4831704	0.079632875	PASS
TICAM2	5	115578650	115602479	-0.293443042	PASS

TICRR	15	89575482	89631056	-0.697600509	PASS
TIE1	1	43300993	43323108	0.033763446	PASS
TIFA	4	112274542	112285903	-0.011382299	PASS
TIFAB	5	135444218	135452399	-0.293443042	PASS
TIGAR	12	4307763	4354593	-0.086128028	PASS
TIGD1	2	232547970	232550592	0.025928102	PASS
TIGD2	4	89111500	89114899	-0.011382299	PASS
TIGD3	11	65354767	65357613	0.182439925	suppress_segment_too_large
TIGD4	4	152769354	152779764	-0.011382299	PASS
TIGD5	8	143597835	143603224	0.307788765	PASS
TIGD6	5	149993118	150001167	-0.293443042	PASS
TIGD7	16	3298832	3305729	0.069936844	PASS
TIGIT	3	114276913	114310288	0.313454204	suppress_segment_too_large
TIMD4	5	156919282	156963255	-0.293443042	PASS
TIMELESS	12	56416373	56449403	-0.202191512	PASS
TIMM10	11	57528463	57530803	-0.129266491	PASS
TIMM10B	11	6481447	6484679	-0.405444717	PASS
TIMM13	19	2425624	2427894	0.079632875	PASS
TIMM17A	1	201955491	201970661	-0.256698369	PASS
TIMM17B	23	48893447	48898143	-0.012567603	PASS
TIMM21	18	74148511	74160530	-0.376397843	PASS
TIMM22	17	997117	1003671	0.128406487	PASS
TIMM23	10	45972449	46003734	-0.404852538	PASS
TIMM23B	10	49942033	49974850	-0.404852538	PASS
TIMM29	19	10928733	10933535	0.093487731	suppress_segment_too_large
TIMM44	19	7926718	7943920	0.079632875	PASS
TIMM50	19	39480412	39493785	0.093487731	suppress_segment_too_large
TIMM8A	23	101345659	101349196	-0.012567603	PASS
TIMM8B	11	112084800	112086798	-0.356188624	PASS
TIMM9	14	58408494	58427614	0.060212819	PASS
TIMMDC1	3	119498532	119525090	0.313454204	suppress_segment_too_large
TIMP1	23	47582313	47586789	-0.012567603	PASS
TIMP2	17	78852977	78925387	0.088516311	PASS
TIMP3	22	32801701	32863043	-0.575930959	PASS
TIMP4	3	12153051	12159351	0.311018244	suppress_segment_too_large
TINAG	6	54307859	54390152	0.048820648	PASS
TINAGL1	1	31576485	31587686	0.033763446	PASS
TINF2	14	24239643	24242674	0.060212819	PASS
TIPARP	3	156673235	156706770	0.318278996	suppress_segment_too_large
TIPIN	15	66336206	66386746	-0.697600509	PASS
TIPRL	1	168178933	168202114	-0.027073548	PASS
TIRAP	11	126283065	126298845	-0.419612865	PASS
TJAP1	6	43477523	43506556	0.048820648	PASS
TJP1	15	29699367	29968865	-0.396255771	PASS
TJP2	9	69121264	69274615	0.365190713	suppress_segment_too_large
TJP3	19	3708109	3750813	0.079632875	PASS

TK1	17	78174075	78187233	0.088516311	PASS
TK2	16	66508003	66552544	-0.371357732	PASS
TKFC	11	61333210	61353295	0.182439925	suppress_segment_too_large
TKT	3	53224707	53256052	-0.234617007	PASS
TKTL1	23	154295671	154330350	-0.012567603	PASS
TKTL2	4	163471093	163473746	-0.011382299	PASS
TLCD1	17	28724348	28727935	0.060555011	PASS
TLCD2	17	1702790	1710438	0.128406487	PASS
TLDC1	16	84476421	84554033	-0.371357732	PASS
TLDC2	20	36876121	36894235	-0.168668583	PASS
TLE1	9	81583683	81689305	0.365190713	suppress_segment_too_large
TLE2	19	2997638	3047635	0.079632875	PASS
TLE3	15	70047790	70098176	-0.697600509	PASS
TLE4	9	79571773	79726882	0.365190713	suppress_segment_too_large
TLE6	19	2977446	2995184	0.079632875	PASS
TLK1	2	170990823	171231314	0.039726562	PASS
TLK2	17	62458658	62615481	0.088516311	PASS
TLL1	4	165873258	166103895	-0.011382299	PASS
TLL2	10	96364606	96513918	-0.367947924	PASS
TLN1	9	35696948	35732395	-0.310444659	PASS
TLN2	15	62390526	62844631	-0.697600509	PASS
TLNRD1	15	81000944	81005788	-0.697600509	PASS
TLR1	4	38790677	38856817	-0.011382299	PASS
TLR10	4	38772239	38782990	-0.011382299	PASS
TLR2	4	153684070	153705702	-0.011382299	PASS
TLR3	4	186069152	186088069	-0.462744716	PASS
TLR4	9	117704175	117724730	0.365190713	suppress_segment_too_large
TLR5	1	223109406	223143282	-0.256698369	PASS
TLR6	4	38823715	38856817	-0.011382299	PASS
TLR7	23	12867083	12890380	-0.012567603	PASS
TLR8	23	12906620	12923169	-0.012567603	PASS
TLR9	3	52221080	52226163	-0.234617007	PASS
TLX1	10	101130505	101137789	-0.367947924	PASS
TLX2	2	74513463	74517147	-0.372959126	PASS
TLX3	5	171309284	171312134	-0.150979337	PASS
TM2D1	1	61681046	61725423	0.033763446	PASS
TM2D2	8	38988808	38996824	-0.201142258	PASS
TM2D3	15	101621444	101652391	-0.697600509	PASS
TM4SF1	3	149369022	149377865	0.318278996	suppress_segment_too_large
TM4SF18	3	149318498	149334414	0.318278996	suppress_segment_too_large
TM4SF19	3	196319342	196338503	0.326834516	PASS
TM4SF19-'	3	196316082	196338373	0.326834516	PASS
TM4SF20	2	227362156	227381995	0.025928102	PASS
TM4SF4	3	149473974	149503281	0.318278996	suppress_segment_too_large
TM4SF5	17	4771884	4783213	0.128406487	PASS
TM6SF1	15	83107407	83144854	-0.697600509	PASS

TM6SF2	19	19264364	19273391	0.093487731	suppress_segment_too_large
TM7SF2	11	65111845	65116384	0.182439925	suppress_segment_too_large
TM7SF3	12	26973195	27014434	0.362961408	suppress_segment_too_large
TM9SF1	14	24189143	24195687	0.060212819	PASS
TM9SF2	13	99446311	99564006	-0.390786075	PASS
TM9SF3	10	96518109	96587452	-0.367947924	PASS
TM9SF4	20	32109506	32167258	-0.168668583	PASS
TMA16	4	163494442	163520539	-0.011382299	PASS
TMA7	3	48440257	48444208	-0.234617007	PASS
TMBIM1	2	218274192	218292586	0.025928102	PASS
TMBIM4	12	66135846	66170072	-0.202191512	PASS
TMBIM6	12	49707725	49764934	-0.202191512	PASS
TMC1	9	72521608	72838230	0.365190713	suppress_segment_too_large
TMC2	20	2536607	2641784	-0.168668583	PASS
TMC3	15	81331217	81374213	-0.697600509	PASS
TMC4	19	54160108	54173250	0.206912349	PASS
TMC5	16	19410496	19499113	0.069936844	PASS
TMC6	17	78110458	78132407	0.088516311	PASS
TMC7	16	18983934	19063942	0.069936844	PASS
TMC8	17	78130770	78142968	0.088516311	PASS
TMCC1	3	129647792	129893606	0.313454204	suppress_segment_too_large
TMCC2	1	205228176	205285632	-0.256698369	PASS
TMCC3	12	94567124	94650562	0.202141228	PASS
TMCO1	1	165724293	165827755	-0.027073548	PASS
TMCO2	1	40245947	40251691	0.033763446	PASS
TMCO3	13	113490995	113554590	-0.390786075	PASS
TMCO4	1	19682213	19799945	0.033763446	PASS
TMCO5A	15	37921939	37967724	-0.396255771	PASS
TMCO6	5	140639427	140645408	-0.293443042	PASS
TMED1	19	10832438	10836318	0.093487731	suppress_segment_too_large
TMED10	14	75131470	75176631	-0.192197564	PASS
TMED2	12	123584531	123598577	0.202141228	PASS
TMED3	15	79311062	79427432	-0.697600509	PASS
TMED4	7	44577894	44582287	-0.69513118	PASS
TMED5	1	93149742	93180728	0.033763446	PASS
TMED6	16	69343248	69351809	-0.371357732	PASS
TMED7	5	115613508	115632992	-0.293443042	PASS
TMED7-TI	5	115578642	115626161	-0.293443042	PASS
TMED8	14	77335021	77377109	-0.192197564	PASS
TMED9	5	177592158	177596124	-0.150979337	PASS
TMEFF1	9	100473113	100577636	0.365190713	suppress_segment_too_large
TMEFF2	2	191949043	192195709	0.025928102	PASS
TMEM100	17	55719627	55732121	0.088516311	PASS
TMEM101	17	44011188	44023946	0.088516311	PASS
TMEM102	17	7435443	7437679	0.128406487	PASS
TMEM104	17	74776483	74839779	0.088516311	PASS

TMEM105	17	81311270	81330674	0.088516311	PASS
TMEM106	17	43211835	43220041	0.088516311	PASS
TMEM106	7	12211241	12243367	-0.69513118	PASS
TMEM106	12	47963569	47968878	-0.202191512	PASS
TMEM107	17	8173237	8176399	0.128406487	PASS
TMEM108	3	133038391	133397792	0.313454204	suppress_segment_too_large
TMEM109	11	60913874	60923443	0.182439925	suppress_segment_too_large
TMEM11	17	21197280	21214624	0.024610076	PASS
TMEM110	3	52833121	52897562	-0.234617007	PASS
TMEM114	16	8537605	8590193	0.069936844	PASS
TMEM115	3	50354749	50359610	-0.234617007	PASS
TMEM116	12	111931282	112013185	0.202141228	PASS
TMEM117	12	43835967	44389762	-0.202191512	PASS
TMEM119	12	108589846	108598320	0.202141228	PASS
TMEM120	7	75986837	75994659	-0.262778904	PASS
TMEM120	12	121712752	121783001	0.202141228	PASS
TMEM121	14	105526603	105530202	-0.192197564	PASS
TMEM121	22	17116299	17121367	-0.575930959	PASS
TMEM123	11	102396332	102470384	0.062969153	PASS
TMEM125	1	43269994	43274002	0.033763446	PASS
TMEM126	11	85647967	85656547	0.062969153	PASS
TMEM126	11	85628573	85636539	0.062969153	PASS
TMEM127	2	96248516	96265994	0.048238121	PASS
TMEM128	4	4235542	4248212	0.279089364	PASS
TMEM129	4	1715952	1721358	0.279089364	PASS
TMEM130	7	98846488	98870771	-0.262778904	PASS
TMEM131	2	97756333	97995891	0.048238121	PASS
TMEM131	4	153466346	153636711	-0.011382299	PASS
TMEM132	11	60924463	60937159	0.182439925	suppress_segment_too_large
TMEM132	12	125186836	125662377	-0.240609783	PASS
TMEM132	12	128267403	128707915	-0.240609783	PASS
TMEM132	12	129071725	129903666	-0.240609783	PASS
TMEM132	17	34579487	34639318	0.060555011	PASS
TMEM134	11	67461710	67469272	0.182439925	suppress_segment_too_large
TMEM135	11	87037844	87323758	0.062969153	PASS
TMEM136	11	120325129	120333682	-0.356188624	PASS
TMEM138	11	61362001	61369509	0.182439925	suppress_segment_too_large
TMEM139	7	143279957	143288048	-0.388946609	PASS
TMEM140	7	135148072	135166215	-0.388946609	PASS
TMEM141	9	136791355	136793257	0.365190713	suppress_segment_too_large
TMEM143	19	48332356	48364237	0.14996833	PASS
TMEM144	4	158201604	158255411	-0.011382299	PASS
TMEM145	19	42313325	42325062	0.093487731	suppress_segment_too_large
TMEM147	19	35545595	35547526	0.093487731	suppress_segment_too_large
TMEM14A	6	52671109	52686588	0.048820648	PASS
TMEM14B	6	10747759	10852753	0.048820648	PASS

TMEM14C	6	10722915	10731129	0.048820648	PASS
TMEM150	2	85598548	85603196	0.066915641	PASS
TMEM150	19	55312801	55334048	0.121336758	PASS
TMEM150	4	82483170	82562357	-0.011382299	PASS
TMEM151	11	66291870	66296664	0.182439925	suppress_segment_too_large
TMEM151	6	44270466	44307506	0.048820648	PASS
TMEM154	4	152618632	152680165	-0.011382299	PASS
TMEM155	4	121758930	121765427	-0.011382299	PASS
TMEM156	4	38966744	39032922	-0.011382299	PASS
TMEM158	3	45224466	45226278	-0.234617007	PASS
TMEM159	16	21158377	21180616	0.069936844	PASS
TMEM160	19	47045907	47048630	0.14996833	PASS
TMEM161	19	19119169	19138513	0.093487731	suppress_segment_too_large
TMEM161	5	88189633	88269476	-0.293443042	PASS
TMEM163	2	134455759	134719000	-0.475215623	PASS
TMEM164	23	110002631	110182734	-0.012567603	PASS
TMEM165	4	55395957	55453397	-0.011382299	PASS
TMEM167	5	83052846	83077863	-0.293443042	PASS
TMEM167	1	109089803	109096934	0.033763446	PASS
TMEM168	7	112762382	112790592	-0.388946609	PASS
TMEM169	2	216081866	216102783	0.025928102	PASS
TMEM17	2	62500221	62511894	-0.372959126	PASS
TMEM170	16	75443054	75465497	-0.371357732	PASS
TMEM170	6	11538278	11583524	0.048820648	PASS
TMEM171	5	73120292	73131817	-0.293443042	PASS
TMEM173	5	139475534	139482935	-0.293443042	PASS
TMEM174	5	73173195	73175143	-0.293443042	PASS
TMEM175	4	932387	958656	0.279089364	PASS
TMEM176	7	150800403	150805120	-0.388946609	PASS
TMEM176	7	150791285	150801360	-0.388946609	PASS
TMEM177	2	119679167	119686507	-0.475215623	PASS
TMEM178	2	39664982	39717963	0.040498426	PASS
TMEM178	7	141074232	141480380	-0.388946609	PASS
TMEM179	14	104474678	104605647	-0.192197564	PASS
TMEM179	11	62787415	62790405	0.182439925	suppress_segment_too_large
TMEM18	2	667335	677439	0.040498426	PASS
TMEM181	6	158536436	158635428	-0.035448111	PASS
TMEM182	2	102736905	103019900	0.048238121	PASS
TMEM183	1	203007386	203024848	-0.256698369	PASS
TMEM184	7	1542235	1560821	-0.69513118	PASS
TMEM184	22	38219291	38273034	-0.575930959	PASS
TMEM184	4	147617383	147672044	-0.011382299	PASS
TMEM185	23	149596556	149631912	-0.012567603	PASS
TMEM185	2	120221278	120223408	-0.475215623	PASS
TMEM186	16	8780384	8797648	0.069936844	PASS
TMEM187	23	153972327	153983195	-0.012567603	PASS

TMEM189	20	50118254	50153734	-0.168668583	PASS
TMEM189	20	50081124	50153637	-0.168668583	PASS
TMEM19	12	71686087	71705046	-0.202191512	PASS
TMEM190	19	55376836	55378244	0.121336758	PASS
TMEM191	22	18527802	18530573	-0.575930959	PASS
TMEM191	22	21466423	21471269	-0.575930959	PASS
TMEM192	4	165070608	165208549	-0.011382299	PASS
TMEM196	7	19719310	19773598	-0.69513118	PASS
TMEM198	2	219543663	219550595	0.025928102	PASS
TMEM199	17	28357581	28363683	0.060555011	PASS
TMEM200	6	130365734	130443063	-0.458570262	PASS
TMEM200	1	29119428	29123935	0.033763446	PASS
TMEM200	18	5882072	5895955	-0.052305056	suppress_segment_too_large
TMEM201	1	9588922	9614873	0.373964873	suppress_segment_too_large
TMEM202	15	72398273	72408029	-0.697600509	PASS
TMEM203	9	137204082	137205638	0.365190713	suppress_segment_too_large
TMEM204	16	1528688	1555580	0.069936844	PASS
TMEM205	19	11342776	11346518	0.093487731	suppress_segment_too_large
TMEM206	1	212363931	212414901	-0.256698369	PASS
TMEM207	3	190428655	190449876	0.318278996	suppress_segment_too_large
TMEM208	16	67227103	67229278	-0.371357732	PASS
TMEM209	7	130164715	130207770	-0.388946609	PASS
TMEM210	9	137170858	137172409	0.365190713	suppress_segment_too_large
TMEM211	22	24934954	24946695	-0.575930959	PASS
TMEM212	3	171843349	171938715	0.318278996	suppress_segment_too_large
TMEM213	7	138797952	138838101	-0.388946609	PASS
TMEM214	2	27032910	27041695	0.040498426	PASS
TMEM215	9	32783499	32787399	-0.310444659	PASS
TMEM216	11	61391687	61398863	0.182439925	suppress_segment_too_large
TMEM217	6	37212180	37258155	0.048820648	PASS
TMEM218	11	125096545	125111763	-0.419612865	PASS
TMEM219	16	29940885	29973052	0.069936844	PASS
TMEM220	17	10699015	10730316	-0.139031004	PASS
TMEM221	19	17435509	17448567	0.093487731	suppress_segment_too_large
TMEM222	1	27322145	27336400	0.033763446	PASS
TMEM223	11	62771629	62792021	0.182439925	suppress_segment_too_large
TMEM225	11	123882926	123885642	-0.949752196	PASS
TMEM225	7	99598066	99610813	-0.262778904	PASS
TMEM229	7	124030916	124033023	-0.388946609	PASS
TMEM229	14	67447084	67533739	0.060212819	PASS
TMEM230	20	5068232	5113103	-0.168668583	PASS
TMEM231	16	75536744	75556286	-0.371357732	PASS
TMEM232	5	110289233	110738956	-0.293443042	PASS
TMEM233	12	119593459	119643066	0.202141228	PASS
TMEM234	1	32214472	32222359	0.033763446	PASS
TMEM235	17	78231310	78240987	0.088516311	PASS

TMEM236	10	17752252	17800868	0.039811807	PASS
TMEM237	2	201620184	201643570	0.025928102	PASS
TMEM238	19	55379245	55384598	0.121336758	PASS
TMEM239	20	2816302	2820284	-0.168668583	PASS
TMEM240	1	1535174	1540453	0.373964873	suppress_segment_too_large
TMEM241	18	23197144	23437961	-0.376397843	PASS
TMEM242	6	157289386	157323601	-0.035448111	PASS
TMEM243	7	87196160	87220587	-0.262778904	PASS
TMEM244	6	129831244	129861547	-0.458570262	PASS
TMEM245	9	109015152	109119945	0.365190713	suppress_segment_too_large
TMEM246	9	101473171	101533537	0.365190713	suppress_segment_too_large
TMEM247	2	46479565	46484425	0.040498426	PASS
TMEM248	7	66921225	66958551	-0.750061359	PASS
TMEM249	8	144352219	144354914	0.307788765	PASS
TMEM25	11	118531041	118547280	-0.356188624	PASS
TMEM250	9	136114581	136118863	0.365190713	suppress_segment_too_large
TMEM251	14	93184951	93187089	-0.192197564	PASS
TMEM252	9	68536580	68540867	-0.310444659	PASS
TMEM253	14	21098937	21103724	0.060212819	PASS
TMEM254	10	80078646	80092557	0.072405807	PASS
TMEM255	23	120258650	120311556	-0.012567603	PASS
TMEM255	13	113759240	113816995	-0.390786075	PASS
TMEM256	17	7402975	7404137	0.128406487	PASS
TMEM256	17	7389734	7404097	0.128406487	PASS
TMEM258	11	61768501	61792802	0.182439925	suppress_segment_too_large
TMEM259	19	1009648	1021179	0.079632875	PASS
TMEM26	10	61406643	61453450	0.072405807	PASS
TMEM260	14	56488354	56650606	0.060212819	PASS
TMEM262	11	65084979	65089375	0.182439925	suppress_segment_too_large
TMEM263	12	106955719	106978778	0.202141228	PASS
TMEM265	16	30740381	30744281	0.069936844	PASS
TMEM266	15	76059837	76229121	-0.697600509	PASS
TMEM267	5	43444252	43483893	-0.293443042	PASS
TMEM268	9	114611206	114646422	0.365190713	suppress_segment_too_large
TMEM269	1	42785007	42816619	0.033763446	PASS
TMEM270	7	73861159	73865893	-0.262778904	PASS
TMEM273	10	49154725	49188585	-0.404852538	PASS
TMEM30A	6	75252924	75284968	-0.368590549	PASS
TMEM30B	14	61277370	61281840	0.060212819	PASS
TMEM31	23	103710909	103714028	-0.012567603	PASS
TMEM33	4	41935120	41960572	-0.011382299	PASS
TMEM35A	23	101078720	101096364	-0.012567603	PASS
TMEM35B	1	34981535	34985353	0.033763446	PASS
TMEM37	2	119429901	119438520	-0.475215623	PASS
TMEM38A	19	16661127	16690029	0.093487731	suppress_segment_too_large
TMEM38B	9	105694544	105776612	0.365190713	suppress_segment_too_large

TMEM39A	3	119429500	119468830	0.313454204	suppress_segment_too_large
TMEM39B	1	32072031	32102866	0.033763446	PASS
TMEM40	3	12733525	12769457	0.311018244	suppress_segment_too_large
TMEM41A	3	185476496	185499057	0.318278996	suppress_segment_too_large
TMEM41B	11	9280654	9314780	-0.405444717	PASS
TMEM42	3	44861869	44865670	-0.234617007	PASS
TMEM43	3	14124940	14143679	0.311018244	suppress_segment_too_large
TMEM44	3	194587673	194633689	0.326834516	PASS
TMEM45A	3	100492619	100577444	0.313454204	suppress_segment_too_large
TMEM45B	11	129815819	129860003	-0.419612865	PASS
TMEM47	23	34627064	34657288	-0.012567603	PASS
TMEM50A	1	25337917	25362361	0.033763446	PASS
TMEM50B	21	33432485	33480011	0.12860881	suppress_segment_too_large
TMEM51	1	15152532	15220480	0.033763446	PASS
TMEM52	1	1917590	1919273	0.373964873	suppress_segment_too_large
TMEM52B	12	10170542	10191801	0.362961408	suppress_segment_too_large
TMEM53	1	44635238	44674555	0.033763446	PASS
TMEM54	1	32894594	32901438	0.033763446	PASS
TMEM56	1	95117338	95197607	0.033763446	PASS
TMEM56-I	1	95117923	95247225	0.033763446	PASS
TMEM59	1	54026681	54053504	0.033763446	PASS
TMEM59L	19	18607430	18621039	0.093487731	suppress_segment_too_large
TMEM60	7	77793728	77798580	-0.262778904	PASS
TMEM61	1	54980792	54992293	0.033763446	PASS
TMEM62	15	43123279	43185146	-0.396255771	PASS
TMEM63A	1	225845536	225882369	-0.256698369	PASS
TMEM63B	6	44126914	44155519	0.048820648	PASS
TMEM63C	14	77116568	77259495	-0.192197564	PASS
TMEM64	8	90621995	90791632	0.279445683	PASS
TMEM65	8	124306189	124372692	0.279445683	PASS
TMEM67	8	93754844	93819234	0.279445683	PASS
TMEM68	8	55696424	55773407	-0.201142258	PASS
TMEM69	1	45687214	45694443	0.033763446	PASS
TMEM70	8	73972437	73982783	-0.201142258	PASS
TMEM71	8	132685007	132760712	0.113969953	PASS
TMEM72	10	44911200	44937002	-0.404852538	PASS
TMEM74	8	108606850	108787615	0.279445683	PASS
TMEM74B	20	1180561	1185415	-0.168668583	PASS
TMEM79	1	156282935	156293185	0.103015788	PASS
TMEM80	11	695428	705028	-0.333835828	PASS
TMEM81	1	205083129	205084460	-0.256698369	PASS
TMEM82	1	15742422	15747982	0.033763446	PASS
TMEM86A	11	18693122	18704785	-0.405444717	PASS
TMEM86B	19	55226639	55229264	0.121336758	PASS
TMEM87A	15	42210452	42273663	-0.396255771	PASS
TMEM87B	2	112055223	112119318	-0.010901494	PASS

TMEM88	17	7855065	7856099		0.128406487	PASS
TMEM88B	1	1426128	1427787		0.373964873	suppress_segment_too_large
TMEM89	3	48620759	48621855		-0.234617007	PASS
TMEM8A	16	370773	387113		0.069936844	PASS
TMEM8B	9	35814451	35865518		-0.310444659	PASS
TMEM9	1	201134772	201171574		-0.256698369	PASS
TMEM91	19	41350911	41384083		0.093487731	suppress_segment_too_large
TMEM92	17	50271406	50281485		0.088516311	PASS
TMEM94	17	75441159	75500090		0.088516311	PASS
TMEM95	17	7355123	7357219		0.128406487	PASS
TMEM97	17	28319095	28328685		0.060555011	PASS
TMEM98	17	32927910	32945106		0.060555011	PASS
TMEM99	17	40819106	40836274		0.060555011	PASS
TMEM9B	11	8947294	8965011		-0.405444717	PASS
TMF1	3	69019827	69052339		-0.234617007	PASS
TMIE	3	46694528	46710886		-0.234617007	PASS
TMIGD1	17	30316333	30334059		0.060555011	PASS
TMIGD2	19	4292232	4302431		0.079632875	PASS
TMIGD3	1	111483348	111563962		0.033763446	PASS
TMLHE	23	155490115	155669944		-0.012567603	PASS
TMOD1	9	97501180	97601748		0.365190713	suppress_segment_too_large
TMOD2	15	51751561	51816368		-0.396255771	PASS
TMOD3	15	51829628	51947295		-0.396255771	PASS
TMOD4	1	151169986	151176284		0.103015788	PASS
TMPO	12	98515512	98550379		0.202141228	PASS
TMPPE	3	33090421	33096801		0.311018244	suppress_segment_too_large
TMPRSS11	4	67909385	67964140		-0.011382299	PASS
TMPRSS11	4	68226653	68245720		-0.011382299	PASS
TMPRSS11	4	67820876	67884032		-0.011382299	PASS
TMPRSS11	4	68447449	68497604		-0.011382299	PASS
TMPRSS11	4	68053198	68129880		-0.011382299	PASS
TMPRSS12	12	50842920	50887884		-0.202191512	PASS
TMPRSS13	11	117900643	117929459		-0.356188624	PASS
TMPRSS15	21	18269116	18485879		0.12860881	suppress_segment_too_large
TMPRSS2	21	41464551	41531116		-0.064425456	suppress_segment_too_large
TMPRSS3	21	42371837	42396846		-0.064425456	suppress_segment_too_large
TMPRSS4	11	118077012	118121890		-0.356188624	PASS
TMPRSS5	11	113687546	113706373		-0.356188624	PASS
TMPRSS6	22	37065436	37109713		-0.575930959	PASS
TMPRSS7	3	112034843	112081269		0.313454204	suppress_segment_too_large
TMPRSS9	19	2360238	2426239		0.079632875	PASS
TMSB10	2	84905625	84906675		0.066915641	PASS
TMSB15A	23	102513676	102516784		-0.012567603	PASS
TMSB15B	23	103918896	104076212		-0.012567603	PASS
TMSB4X	23	12975108	12977227		-0.012567603	PASS
TMTC1	12	29500840	29784759		0.362961408	suppress_segment_too_large

TMTC2	12	82686880	83134870	0.202141228	PASS
TMTC3	12	88142296	88199887	0.202141228	PASS
TMTC4	13	100603927	100675093	-0.390786075	PASS
TMUB1	7	151081080	151083546	-0.388946609	PASS
TMUB2	17	44186970	44191731	0.088516311	PASS
TMX1	14	51240162	51257546	0.060212819	PASS
TMX2	11	57712600	57740973	-0.129266491	PASS
TMX2-CTI	11	57712605	57791586	-0.129266491	PASS
TMX3	18	68673688	68715298	-0.376397843	PASS
TMX4	20	7977348	8019829	-0.168668583	PASS
TNC	9	115019578	115118257	0.365190713	suppress_segment_too_large
TNF	6	31575567	31578336	0.048820648	PASS
TNFAIP1	17	28335602	28347009	0.060555011	PASS
TNFAIP2	14	103123442	103137439	-0.192197564	PASS
TNFAIP3	6	137867188	137883312	-0.035448111	PASS
TNFAIP6	2	151357592	151380048	-0.475215623	PASS
TNFAIP8	5	119268692	119399688	-0.293443042	PASS
TNFAIP8L	19	4639518	4655568	0.079632875	PASS
TNFAIP8L	1	151156629	151159749	0.103015788	PASS
TNFAIP8L	15	51056598	51105276	-0.396255771	PASS
TNFRSF10	8	23190452	23225126	-0.345962679	PASS
TNFRSF10	8	23020133	23069179	-0.345962679	PASS
TNFRSF10	8	23102590	23117437	-0.345962679	PASS
TNFRSF10	8	23135588	23164030	-0.345962679	PASS
TNFRSF11	18	62325287	62391292	-0.376397843	PASS
TNFRSF11	8	118923557	118952200	0.279445683	PASS
TNFRSF12	16	3018445	3022383	0.069936844	PASS
TNFRSF13	17	16929816	16972118	0.024610076	PASS
TNFRSF13	22	41922023	41926818	-0.575930959	PASS
TNFRSF14	1	2555639	2565382	0.373964873	suppress_segment_too_large
TNFRSF17	16	11965107	11968068	0.069936844	PASS
TNFRSF18	1	1203508	1206691	0.373964873	suppress_segment_too_large
TNFRSF19	13	23570370	23676104	-0.390786075	PASS
TNFRSF1A	12	6328757	6342114	-0.086128028	PASS
TNFRSF1E	1	12167003	12209228	0.003932739	PASS
TNFRSF21	6	47231532	47309905	0.048820648	PASS
TNFRSF25	1	6460786	6466195	0.373964873	suppress_segment_too_large
TNFRSF4	1	1211326	1214138	0.373964873	suppress_segment_too_large
TNFRSF6E	20	63696651	63698684	-0.168668583	PASS
TNFRSF8	1	12063377	12144207	0.003932739	PASS
TNFRSF9	1	7915894	7943165	0.373964873	suppress_segment_too_large
TNFSF10	3	172505508	172523507	0.318278996	suppress_segment_too_large
TNFSF11	13	42562736	42608013	-0.390786075	PASS
TNFSF12	17	7548891	7557890	0.128406487	PASS
TNFSF12-7	17	7549099	7561601	0.128406487	PASS
TNFSF13	17	7558292	7561608	0.128406487	PASS

TNFSF13B	13	108251240	108308484	-0.390786075	PASS
TNFSF14	19	6661253	6670588	0.079632875	PASS
TNFSF15	9	114784635	114806126	0.365190713	suppress_segment_too_large
TNFSF18	1	173039960	173050963	-0.027073548	PASS
TNFSF4	1	173183734	173207313	-0.027073548	PASS
TNFSF8	9	114893343	114930595	0.365190713	suppress_segment_too_large
TNFSF9	19	6530999	6535928	0.079632875	PASS
TNIK	3	171061339	171460408	0.318278996	suppress_segment_too_large
TNIP1	5	151029945	151093577	-0.293443042	PASS
TNIP2	4	2741648	2756376	0.279089364	PASS
TNIP3	4	121131408	121227466	-0.011382299	PASS
TNK1	17	7380534	7389774	0.128406487	PASS
TNK2	3	195863364	195911945	0.326834516	PASS
TNKS	8	9555914	9782346	-0.345962679	PASS
TNKS1BP1	11	57299638	57324952	-0.129266491	PASS
TNKS2	10	91798312	91865276	-0.39678585	PASS
TNMD	23	100584802	100599885	-0.012567603	PASS
TNN	1	175067858	175148066	-0.027073548	PASS
TNNC1	3	52451102	52454070	-0.234617007	PASS
TNNC2	20	45823214	45833745	-0.168668583	PASS
TNNI1	1	201403768	201429866	-0.256698369	PASS
TNNI2	11	1838989	1841680	-0.333835828	PASS
TNNI3	19	55151767	55157773	0.121336758	PASS
TNNI3K	1	74235401	74544428	0.033763446	PASS
TNNT1	19	55132794	55149354	0.121336758	PASS
TNNT2	1	201359008	201377762	-0.256698369	PASS
TNNT3	11	1919562	1938706	-0.333835828	PASS
TNP1	2	216859458	216860064	0.025928102	PASS
TNP2	16	11267748	11269533	0.069936844	PASS
TNPO1	5	72816312	72916733	-0.293443042	PASS
TNPO2	19	12699194	12724011	0.093487731	suppress_segment_too_large
TNPO3	7	128954180	129055173	-0.388946609	PASS
TNR	1	175315194	175743770	-0.027073548	PASS
TNRC18	7	5306790	5425414	-0.69513118	PASS
TNRC6A	16	24610209	24827632	0.069936844	PASS
TNRC6B	22	40044817	40335808	-0.575930959	PASS
TNRC6C	17	77959240	78108835	0.088516311	PASS
TNS1	2	217799588	218033982	0.025928102	PASS
TNS2	12	53046969	53064372	-0.202191512	PASS
TNS3	7	47275154	47582558	-0.69513118	PASS
TNS4	17	40475828	40501597	0.060555011	PASS
TNXB	6	32041153	32115334	0.048820648	PASS
TOB1	17	50862223	50867978	0.088516311	PASS
TOB2	22	41433492	41447023	-0.575930959	PASS
TOE1	1	45339670	45343975	0.033763446	PASS
TOGARAM	14	44962208	45074431	0.060212819	PASS

TOGARAM	2	28956611	29061373	0.040498426	PASS
TOLLIP	11	1274371	1309654	-0.333835828	PASS
TOM1	22	35299275	35347994	-0.575930959	PASS
TOM1L1	17	54899387	54961956	0.088516311	PASS
TOM1L2	17	17843511	17972422	0.024610076	PASS
TOMM20	1	235109336	235128936	-0.256698369	PASS
TOMM20L	14	58395916	58408701	0.060212819	PASS
TOMM22	22	38681948	38685421	-0.575930959	PASS
TOMM34	20	44942130	44960486	-0.168668583	PASS
TOMM40	19	44890569	44903689	0.14996833	PASS
TOMM40L	1	161225939	161230744	-0.027073548	PASS
TOMM5	9	37582646	37592642	-0.310444659	PASS
TOMM6	6	41787662	41789898	0.048820648	PASS
TOMM7	7	22812628	22822852	-0.69513118	PASS
TOMM70	3	100363431	100401398	0.313454204	suppress_segment_too_large
TONSL	8	144428775	144444444	0.307788765	PASS
TOP1	20	41028818	41124487	-0.168668583	PASS
TOP1MT	8	143304384	143359979	0.307788765	PASS
TOP2A	17	40388516	40417950	0.060555011	PASS
TOP2B	3	25597905	25664907	0.311018244	suppress_segment_too_large
TOP3A	17	18271428	18315007	0.024610076	PASS
TOP3B	22	21957025	21982816	-0.575930959	PASS
TOPAZ1	3	44241886	44332098	-0.234617007	PASS
TOPBP1	3	133598175	133662380	0.313454204	suppress_segment_too_large
TOPORS	9	32540544	32552553	-0.310444659	PASS
TOR1A	9	129812944	129824134	0.365190713	suppress_segment_too_large
TOR1AIP1	1	179882042	179925000	-0.027073548	PASS
TOR1AIP2	1	179839967	179877803	-0.027073548	PASS
TOR1B	9	129803153	129811281	0.365190713	suppress_segment_too_large
TOR2A	9	127731524	127735317	0.365190713	suppress_segment_too_large
TOR3A	1	179081377	179098023	-0.027073548	PASS
TOR4A	9	137277749	137282641	0.365190713	suppress_segment_too_large
TOX	8	58805418	59119208	-0.201142258	PASS
TOX2	20	43914864	44069616	-0.168668583	PASS
TOX3	16	52438005	52547802	-0.371357732	PASS
TOX4	14	21476597	21499175	0.060212819	PASS
TP53	17	7661779	7687550	0.128406487	PASS
TP53AIP1	11	128934731	128943399	-0.419612865	PASS
TP53BP1	15	43403061	43510728	-0.396255771	PASS
TP53BP2	1	223779899	223845972	-0.256698369	PASS
TP53I11	11	44885903	44951306	0.117376172	PASS
TP53I13	17	29566052	29573157	0.060555011	PASS
TP53I3	2	24077433	24085861	0.040498426	PASS
TP53INP1	8	94925972	94949411	0.279445683	PASS
TP53INP2	20	34704290	34713439	-0.168668583	PASS
TP53RK	20	46684365	46689779	-0.168668583	PASS

TP53TG3D	16	32252719	32255922	0.069936844	PASS
TP53TG5	20	45372563	45407889	-0.168668583	PASS
TP63	3	189631416	189897279	0.318278996	suppress_segment_too_large
TP73	1	3652520	3736201	0.373964873	suppress_segment_too_large
TPBG	6	82363206	82370828	-0.368590549	PASS
TPBGL	11	75240905	75243697	0.062969153	PASS
TPCN1	12	113221050	113298585	0.202141228	PASS
TPCN2	11	69048897	69162440	0.182439925	suppress_segment_too_large
TPD52	8	80034745	80231232	0.279445683	PASS
TPD52L1	6	125119049	125264407	-0.458570262	PASS
TPD52L2	20	63865228	63891545	-0.168668583	PASS
TPD52L3	9	6328375	6331900	-0.310444659	PASS
TPGS1	19	507497	519654	0.079632875	PASS
TPGS2	18	36777647	36829216	-0.376397843	PASS
TPH1	11	18017564	18042426	-0.405444717	PASS
TPH2	12	71938846	72186618	-0.202191512	PASS
TPI1	12	6867119	6870948	-0.086128028	PASS
TPK1	7	144451941	144836395	-0.388946609	PASS
TPM1	15	63042632	63071915	-0.697600509	PASS
TPM2	9	35681992	35690121	-0.310444659	PASS
TPM3	1	154155304	154194648	0.103015788	PASS
TPM4	19	16067021	16103005	0.093487731	suppress_segment_too_large
TPMT	6	18128311	18155074	0.048820648	PASS
TPO	2	1374066	1543711	0.040498426	PASS
TPP1	11	6612763	6619485	-0.405444717	PASS
TPP2	13	102597003	102679958	-0.390786075	PASS
TPPP	5	659862	693395	0.055007813	PASS
TPPP2	14	21024109	21036276	0.060212819	PASS
TPPP3	16	67389809	67393535	-0.371357732	PASS
TPR	1	186311652	186375693	-0.027073548	PASS
TPRA1	3	127571266	127598267	0.313454204	suppress_segment_too_large
TPRG1	3	188947214	189325304	0.318278996	suppress_segment_too_large
TPRG1L	1	3625002	3630127	0.373964873	suppress_segment_too_large
TPRKB	2	73729104	73737400	-0.372959126	PASS
TPRN	9	137191617	137204193	0.365190713	suppress_segment_too_large
TPRX1	19	47801243	47819051	0.14996833	PASS
TPSAB1	16	1240696	1242554	0.069936844	PASS
TPSB2	16	1227272	1230184	0.069936844	PASS
TPSD1	16	1256059	1258998	0.069936844	PASS
TPSG1	16	1221651	1225257	0.069936844	PASS
TPST1	7	66205199	66420543	-0.750061359	PASS
TPST2	22	26521983	26596717	-0.575930959	PASS
TPT1	13	45333471	45341370	-0.390786075	PASS
TPTE	21	10521553	10606140	-0.304975024	PASS
TPTE2	13	19422877	19536762	-0.390786075	PASS
TPTEP2-C:	22	38291918	38398522	-0.575930959	PASS

TPX2	20	31739271	31801805	-0.168668583	PASS
TRA2A	7	23504780	23532041	-0.69513118	PASS
TRA2B	3	185915906	185938136	0.318278996	suppress_segment_too_large
TRABD	22	50185915	50199598	-0.808211634	PASS
TRABD2A	2	84821650	84907008	0.066915641	PASS
TRABD2B	1	47760528	47996895	0.033763446	PASS
TRADD	16	67154180	67160298	-0.371357732	PASS
TRAF1	9	120902393	120929173	0.365190713	suppress_segment_too_large
TRAF2	9	136881912	136926607	0.365190713	suppress_segment_too_large
TRAF3	14	102777476	102911500	-0.192197564	PASS
TRAF3IP1	2	238320441	238400900	0.025928102	PASS
TRAF3IP2	6	111556454	111606278	-0.368590549	PASS
TRAF3IP3	1	209756032	209782320	-0.256698369	PASS
TRAF4	17	28743984	28750958	0.060555011	PASS
TRAF5	1	211326615	211374946	-0.256698369	PASS
TRAF6	11	36487027	36510272	-0.405444717	PASS
TRAF7	16	2155698	2178129	0.069936844	PASS
TRAFD1	12	112125501	112153609	0.202141228	PASS
TRAIP	3	49828599	49856574	-0.234617007	PASS
TRAK1	3	42013802	42225889	-0.234617007	PASS
TRAK2	2	201377207	201451579	0.025928102	PASS
TRAM1	8	70573442	70608387	-0.201142258	PASS
TRAM1L1	4	117083554	117085576	-0.011382299	PASS
TRAM2	6	52497402	52576915	0.048820648	PASS
TRANK1	3	36826820	36945098	-0.234617007	PASS
TRAP1	16	3651639	3717597	0.069936844	PASS
TRAPPC1	17	7930345	7932123	0.128406487	PASS
TRAPPC1(	21	44012319	44106552	-0.064425456	suppress_segment_too_large
TRAPPC11	4	183659267	183713594	-0.462744716	PASS
TRAPPC12	2	3379675	3485094	0.040498426	PASS
TRAPPC13	5	65624716	65666233	-0.293443042	PASS
TRAPPC2	23	13712244	13734635	-0.012567603	PASS
TRAPPC2I	19	57363511	57365353	-0.180829161	PASS
TRAPPC2I	16	88856220	88862686	-0.371357732	PASS
TRAPPC3	1	36136570	36156053	0.033763446	PASS
TRAPPC3I	6	116494989	116545610	-0.368590549	PASS
TRAPPC4	11	119018432	119025454	-0.356188624	PASS
TRAPPC5	19	7680843	7687703	0.079632875	PASS
TRAPPC6/	19	45162928	45178237	0.14996833	PASS
TRAPPC6I	14	39147811	39170532	0.060212819	PASS
TRAPPC8	18	31829173	31953136	-0.376397843	PASS
TRAPPC9	8	139727725	140458579	0.113969953	PASS
TRARG1	17	1279663	1300987	0.128406487	PASS
TRAT1	3	108822698	108855005	0.313454204	suppress_segment_too_large
TRDMT1	10	17142254	17202054	0.039811807	PASS
TRDN	6	123216339	123637093	-1.97829255	PASS

TREH	11	118657316	118679690	-0.356188624	PASS
TREM1	6	41267926	41286719	0.048820648	PASS
TREM2	6	41158506	41163186	0.048820648	PASS
TREML1	6	41149342	41154337	0.048820648	PASS
TREML2	6	41190277	41201194	0.048820648	PASS
TREML4	6	41228324	41238882	0.048820648	PASS
TRERF1	6	42224931	42452051	0.048820648	PASS
TREX1	3	48465811	48467645	-0.234617007	PASS
TREX2	23	153444720	153470587	-0.012567603	PASS
TRH	3	129974305	129977938	0.313454204	suppress_segment_too_large
TRHDE	12	72087266	72670757	-0.202191512	PASS
TRHR	8	109086621	109119584	0.279445683	PASS
TRIAP1	12	120443961	120446412	0.202141228	PASS
TRIB1	8	125430321	125438405	0.279445683	PASS
TRIB2	2	12716889	12742734	0.040498426	PASS
TRIB3	20	362835	397559	-0.168668583	PASS
TRIL	7	28953358	28958292	-0.69513118	PASS
TRIM10	6	30151945	30160934	0.048820648	PASS
TRIM11	1	228393673	228406840	-0.256698369	PASS
TRIM13	13	49995888	50020481	-0.390786075	PASS
TRIM14	9	98069275	98119212	0.365190713	suppress_segment_too_large
TRIM15	6	30163206	30172696	0.048820648	PASS
TRIM16	17	15627960	15684311	-0.139031004	PASS
TRIM16L	17	18697998	18736118	0.024610076	PASS
TRIM17	1	228407940	228416861	-0.256698369	PASS
TRIM2	4	153152342	153339320	-0.011382299	PASS
TRIM21	11	4384897	4393696	-0.333835828	PASS
TRIM22	11	5689689	5737089	-0.957525224	PASS
TRIM23	5	65589680	65625975	-0.293443042	PASS
TRIM24	7	138460334	138589993	-0.388946609	PASS
TRIM25	17	56836387	56914080	0.088516311	PASS
TRIM26	6	30184455	30213427	0.048820648	PASS
TRIM27	6	28903002	28923989	0.048820648	PASS
TRIM28	19	58544091	58550722	0.063781462	PASS
TRIM29	11	120111275	120185529	-0.356188624	PASS
TRIM3	11	6448613	6474459	-0.405444717	PASS
TRIM31	6	30102897	30113106	0.048820648	PASS
TRIM32	9	116687302	116701300	0.365190713	suppress_segment_too_large
TRIM33	1	114392777	114511160	0.033763446	PASS
TRIM34	11	5619764	5644398	-0.957525224	PASS
TRIM35	8	27284887	27311319	-0.345962679	PASS
TRIM36	5	115124762	115180546	-0.293443042	PASS
TRIM37	17	58982638	59106921	0.088516311	PASS
TRIM38	6	25962802	25991226	0.048820648	PASS
TRIM39	6	30326479	30343729	0.048820648	PASS
TRIM39-R	6	30328907	30346854	0.048820648	PASS

TRIM4	7	99876958	99919600	-0.262778904	PASS
TRIM40	6	30136108	30148735	0.048820648	PASS
TRIM41	5	181222499	181235809	-0.150979337	PASS
TRIM42	3	140678039	140701150	-0.356957581	PASS
TRIM43	2	95592018	95599778	0.048238121	PASS
TRIM43B	2	95476967	95484731	0.048238121	PASS
TRIM44	11	35662805	35818007	-0.405444717	PASS
TRIM45	1	117111060	117122587	0.033763446	PASS
TRIM46	1	155173787	155184971	0.103015788	PASS
TRIM47	17	75874161	75878575	0.088516311	PASS
TRIM48	11	55262182	55271119	-0.129266491	PASS
TRIM49	11	89797655	89808575	0.062969153	PASS
TRIM49B	11	49027501	49038451	-0.621795272	PASS
TRIM49C	11	90031106	90042025	0.062969153	PASS
TRIM5	11	5663557	5938619	-0.957525224	PASS
TRIM50	7	73312539	73328082	-0.262778904	PASS
TRIM51	11	55883297	55891810	-0.129266491	PASS
TRIM51GI	11	48975324	48983826	-0.621795272	PASS
TRIM52	5	181254417	181261139	-0.150979337	PASS
TRIM54	2	27282392	27307439	0.040498426	PASS
TRIM55	8	66126896	66175487	-0.201142258	PASS
TRIM56	7	101085439	101097967	-0.388946609	PASS
TRIM58	1	247857199	247878205	-0.368111568	PASS
TRIM59	3	160432445	160485773	0.318278996	suppress_segment_too_large
TRIM6	11	5596109	5612958	-0.957525224	PASS
TRIM6-TR	11	5596725	5644398	-0.957525224	PASS
TRIM60	4	165016458	165041744	-0.011382299	PASS
TRIM61	4	164954446	164977668	-0.011382299	PASS
TRIM62	1	33145402	33182059	0.033763446	PASS
TRIM63	1	26051304	26068436	0.033763446	PASS
TRIM64	11	89968502	89975228	0.062969153	PASS
TRIM64B	11	89869282	89876017	0.062969153	PASS
TRIM64C	11	49053714	49059112	-0.621795272	PASS
TRIM65	17	75880335	75897003	0.088516311	PASS
TRIM66	11	8612037	8682694	-0.405444717	PASS
TRIM67	1	231162112	231221556	-0.256698369	PASS
TRIM68	11	4598672	4608259	-0.333835828	PASS
TRIM69	15	44728988	44767829	-0.396255771	PASS
TRIM7	5	181193924	181205293	-0.150979337	PASS
TRIM71	3	32818018	32897826	0.311018244	suppress_segment_too_large
TRIM72	16	31214021	31231537	0.069936844	PASS
TRIM73	7	75395063	75410996	-0.262778904	PASS
TRIM74	7	72959485	72969466	-0.262778904	PASS
TRIM77	11	89710299	89717872	0.062969153	PASS
TRIM8	10	102642310	102660680	-0.367947924	PASS
TRIM9	14	50975262	51096061	0.060212819	PASS

TRIML1	4	188139419	188147743	-0.462744716	PASS
TRIML2	4	188091273	188109603	-0.462744716	PASS
TRIO	5	14143702	14532128	0.055007813	PASS
TRIOBP	22	37696988	37776556	-0.575930959	PASS
TRIP10	19	6737925	6751526	0.079632875	PASS
TRIP11	14	91965991	92040896	-0.192197564	PASS
TRIP12	2	229763838	229923239	0.025928102	PASS
TRIP13	5	892643	919357	0.055007813	PASS
TRIP4	15	64387748	64455303	-0.697600509	PASS
TRIP6	7	100867138	100873454	-0.262778904	PASS
TRIQK	8	92883530	93017673	0.279445683	PASS
TRIR	19	12730640	12734775	0.093487731	suppress_segment_too_large
TRIT1	1	39838110	39883511	0.033763446	PASS
TRMO	9	97904489	97922570	0.365190713	suppress_segment_too_large
TRMT1	19	13104902	13117567	0.093487731	suppress_segment_too_large
TRMT10A	4	99546709	99564032	-0.011382299	PASS
TRMT10B	9	37753803	37778972	-0.310444659	PASS
TRMT10C	3	101561862	101566446	0.313454204	suppress_segment_too_large
TRMT11	6	125986430	126203817	-0.458570262	PASS
TRMT112	11	64316460	64318084	0.182439925	suppress_segment_too_large
TRMT12	8	124450820	124462150	0.279445683	PASS
TRMT13	1	100133150	100150497	0.033763446	PASS
TRMT1L	1	185118098	185157072	-0.027073548	PASS
TRMT2A	22	20111866	20117392	-0.575930959	PASS
TRMT2B	23	101009346	101052116	-0.012567603	PASS
TRMT44	4	8436140	8493531	0.279089364	PASS
TRMT5	14	60971451	60981358	0.060212819	PASS
TRMT6	20	5937235	5950558	-0.168668583	PASS
TRMT61A	14	103529184	103537073	-0.192197564	PASS
TRMT61B	2	28849821	28870301	0.040498426	PASS
TRMT9B	8	12945642	13031503	-0.345962679	PASS
TRMU	22	46330875	46357340	-0.808211634	PASS
TRNAU1A	1	28553085	28578545	0.033763446	PASS
TRNP1	1	26993707	27000898	0.033763446	PASS
TRNT1	3	3126916	3150879	0.311018244	suppress_segment_too_large
TRO	23	54920462	54931431	-0.012567603	PASS
TROAP	12	49323236	49331731	-0.202191512	PASS
TROVE2	1	193059422	193091777	-0.027073548	PASS
TRPA1	8	72019917	72075617	-0.201142258	PASS
TRPC1	3	142724074	142807888	0.318278996	suppress_segment_too_large
TRPC3	4	121879027	121951754	-0.011382299	PASS
TRPC4	13	37636636	37870425	-0.390786075	PASS
TRPC4AP	20	35002404	35092871	-0.168668583	PASS
TRPC5	23	111774315	112082776	-0.012567603	PASS
TRPC5OS	23	111876051	111903990	-0.012567603	PASS
TRPC6	11	101451564	101872562	0.062969153	PASS

TRPC7	5	136213320	136365545	-0.293443042	PASS
TRPM1	15	31001061	31161273	-0.396255771	PASS
TRPM2	21	44350163	44443081	-0.064425456	suppress_segment_too_large
TRPM3	9	70529063	71446904	0.365190713	suppress_segment_too_large
TRPM4	19	49157741	49211836	0.14996833	PASS
TRPM5	11	2404515	2423045	-0.333835828	PASS
TRPM6	9	74722495	74888094	0.365190713	suppress_segment_too_large
TRPM7	15	50552473	50686815	-0.396255771	PASS
TRPM8	2	233917398	234019522	0.025928102	PASS
TRPS1	8	115408496	115809673	0.279445683	PASS
TRPT1	11	64223799	64226254	0.182439925	suppress_segment_too_large
TRPV1	17	3565444	3609411	0.128406487	PASS
TRPV2	17	16415542	16437003	0.024610076	PASS
TRPV3	17	3510502	3557995	0.128406487	PASS
TRPV4	12	109783085	109833401	0.202141228	PASS
TRPV5	7	142908101	142933808	-0.388946609	PASS
TRPV6	7	142871203	142885762	-0.388946609	PASS
TRRAP	7	98877933	99050831	-0.262778904	PASS
TRUB1	10	114938193	114977676	0.063874692	PASS
TRUB2	9	128305161	128322742	0.365190713	suppress_segment_too_large
TSACC	1	156337314	156346995	0.103015788	PASS
TSC1	9	132891348	132946874	0.365190713	suppress_segment_too_large
TSC2	16	2047465	2089487	0.069936844	PASS
TSC22D1	13	44432143	44577147	-0.390786075	PASS
TSC22D2	3	150408335	150466431	0.318278996	suppress_segment_too_large
TSC22D3	23	107713221	107777342	-0.012567603	PASS
TSC22D4	7	100463359	100479279	-0.262778904	PASS
TSEN15	1	184051651	184123978	-0.027073548	PASS
TSEN2	3	12484432	12539623	0.311018244	suppress_segment_too_large
TSEN34	19	54189938	54194536	0.206912349	PASS
TSEN54	17	75516060	75524739	0.088516311	PASS
TSFM	12	57782589	57808071	-0.202191512	PASS
TSG101	11	18468336	18527232	-0.405444717	PASS
TSGA10	2	98997261	99154964	0.048238121	PASS
TSGA10IP	11	65945445	65959963	0.182439925	suppress_segment_too_large
TSGA13	7	130668648	130687432	-0.388946609	PASS
TSHB	1	115029824	115034309	0.033763446	PASS
TSHR	14	80954989	81146302	-0.192197564	PASS
TSHZ1	18	75210755	75289950	-0.376397843	PASS
TSHZ2	20	52972407	53495330	-0.168668583	PASS
TSHZ3	19	31274945	31349547	0.093487731	suppress_segment_too_large
TSKS	19	49739753	49763330	0.14996833	PASS
TSKU	11	76782251	76798154	0.062969153	PASS
TSLP	5	111070062	111078024	-0.293443042	PASS
TSN	2	121737103	121767853	-0.475215623	PASS
TSNARE1	8	142212080	142403240	0.113969953	PASS

TSNAX	1	231528653	231566524	-0.256698369	PASS
TSNAX-DI	1	231528653	231819244	-0.256698369	PASS
TSNAXIP1	16	67806765	67832148	-0.371357732	PASS
TSPAN1	1	46175073	46185958	0.033763446	PASS
TSPAN10	17	81637171	81648749	0.088516311	PASS
TSPAN11	12	30926428	30996599	0.362961408	suppress_segment_too_large
TSPAN12	7	120787320	120858402	-0.388946609	PASS
TSPAN13	7	16753535	16784536	-0.69513118	PASS
TSPAN14	10	80454166	80533123	0.072405807	PASS
TSPAN15	10	69451473	69507669	0.072405807	PASS
TSPAN16	19	11296139	11326996	0.093487731	suppress_segment_too_large
TSPAN17	5	176647387	176659054	-0.150979337	PASS
TSPAN18	11	44726465	44932421	0.117376172	PASS
TSPAN19	12	85014311	85036277	0.202141228	PASS
TSPAN2	1	115048011	115089500	0.033763446	PASS
TSPAN3	15	77041404	77083984	-0.697600509	PASS
TSPAN31	12	57738013	57750211	-0.202191512	PASS
TSPAN32	11	2301997	2318200	-0.333835828	PASS
TSPAN33	7	129144892	129169697	-0.388946609	PASS
TSPAN4	11	842808	867116	-0.333835828	PASS
TSPAN5	4	98470367	98658629	-0.011382299	PASS
TSPAN6	23	100627109	100639991	-0.012567603	PASS
TSPAN7	23	38561370	38688920	-0.012567603	PASS
TSPAN8	12	71125085	71441898	-0.202191512	PASS
TSPAN9	12	3077355	3286564	-0.086128028	PASS
TSPEAR	21	44497892	44711580	-0.064425456	suppress_segment_too_large
TSPO	22	43151514	43163242	-0.575930959	PASS
TSPO2	6	41042554	41044337	0.048820648	PASS
TSPOAP1	17	58301228	58328760	0.088516311	PASS
TSPYL1	6	116276578	116279903	-0.368590549	PASS
TSPYL2	23	53082367	53088540	-0.012567603	PASS
TSPYL4	6	116249961	116254140	-0.368590549	PASS
TSPYL5	8	97273474	97277964	0.279445683	PASS
TSPYL6	2	54253184	54256272	0.040498426	PASS
TSR1	17	2322503	2337507	0.128406487	PASS
TSR2	23	54440401	54445487	-0.012567603	PASS
TSR3	16	1349240	1351911	0.069936844	PASS
TSSC4	11	2400488	2403878	-0.333835828	PASS
TSSK1B	5	113432554	113435031	-0.293443042	PASS
TSSK2	22	19130808	19132623	-0.575930959	PASS
TSSK3	1	32351521	32364312	0.033763446	PASS
TSSK4	14	24205697	24208362	0.060212819	PASS
TSSK6	19	19512418	19515685	0.093487731	suppress_segment_too_large
TST	22	37010859	37020183	-0.575930959	PASS
TSTA3	8	143612618	143618048	0.307788765	PASS
TSTD1	1	161037631	161038990	-0.027073548	PASS

TSTD2	9	97600080	97633575	0.365190713	suppress_segment_too_large
TSTD3	6	99520693	99531918	-0.368590549	PASS
TTBK1	6	43243680	43288259	0.048820648	PASS
TTBK2	15	42738734	42920809	-0.396255771	PASS
TTC1	5	160009113	160065543	-0.150979337	PASS
TTC12	11	113314529	113383544	-0.356188624	PASS
TTC13	1	230906243	230978875	-0.256698369	PASS
TTC14	3	180602130	180617828	0.318278996	suppress_segment_too_large
TTC16	9	127716066	127731600	0.365190713	suppress_segment_too_large
TTC17	11	43358932	43494933	0.117376172	PASS
TTC19	17	15999380	16045015	0.024610076	PASS
TTC21A	3	39107704	39138903	-0.234617007	PASS
TTC21B	2	165857475	165953843	0.039726562	PASS
TTC22	1	54779712	54801267	0.033763446	PASS
TTC23	15	99136323	99251223	-0.697600509	PASS
TTC23L	5	34838833	34899456	0.055007813	PASS
TTC24	1	156579727	156586770	0.103015788	PASS
TTC25	17	41930635	41965651	0.060555011	PASS
TTC26	7	139133744	139191986	-0.388946609	PASS
TTC27	2	32628032	32821051	0.040498426	PASS
TTC28	22	27978014	28679865	-0.575930959	PASS
TTC29	4	146706638	146945882	-0.011382299	PASS
TTC3	21	37073226	37203112	-0.064425456	suppress_segment_too_large
TTC30A	2	177612992	177618966	0.039726562	PASS
TTC30B	2	177548998	177553014	0.039726562	PASS
TTC31	2	74483073	74494886	-0.372959126	PASS
TTC32	2	19896643	19901986	0.040498426	PASS
TTC33	5	40512333	40755975	-0.293443042	PASS
TTC34	1	2635976	2801717	0.373964873	suppress_segment_too_large
TTC36	11	118527472	118531197	-0.356188624	PASS
TTC37	5	95461755	95555007	-0.293443042	PASS
TTC38	22	46267961	46294008	-0.808211634	PASS
TTC39A	1	51287258	51345116	0.033763446	PASS
TTC39B	9	15163622	15307360	-0.310444659	PASS
TTC39C	18	23992773	24135610	-0.376397843	PASS
TTC4	1	54715822	54742657	0.033763446	PASS
TTC5	14	20256558	20305994	0.060212819	PASS
TTC6	14	37595847	38041442	0.060212819	PASS
TTC7A	2	46916157	47076137	0.040498426	PASS
TTC7B	14	90524564	90816479	-0.192197564	PASS
TTC8	14	88824153	88881078	-0.192197564	PASS
TTC9	14	70641787	70675360	0.060212819	PASS
TTC9B	19	40216058	40218399	0.093487731	suppress_segment_too_large
TTC9C	11	62728069	62740293	0.182439925	suppress_segment_too_large
TTF1	9	132375548	132406851	0.365190713	suppress_segment_too_large
TTF2	1	117060303	117107453	0.033763446	PASS

TTI1	20	37983007	38033468	-0.168668583	PASS
TTI2	8	33473386	33513601	-0.345962679	PASS
TTK	6	80003887	80042527	-0.368590549	PASS
TTL	2	112482154	112541739	-0.010901494	PASS
TTLL1	22	43039644	43089428	-0.575930959	PASS
TTLL10	1	1173884	1197935	0.373964873	suppress_segment_too_large
TTLL11	9	121821928	122093606	0.365190713	suppress_segment_too_large
TTLL12	22	43166622	43187133	-0.575930959	PASS
TTLL13P	15	90249530	90265482	-0.697600509	PASS
TTLL2	6	167325071	167359503	-0.035448111	PASS
TTLL3	3	9808086	9855138	0.311018244	suppress_segment_too_large
TTLL4	2	218710845	218755416	0.025928102	PASS
TTLL5	14	75633625	75955078	-0.192197564	PASS
TTLL6	17	48762235	48817214	0.088516311	PASS
TTLL7	1	83865028	83999150	0.033763446	PASS
TTLL8	22	50015123	50056935	-0.808211634	PASS
TTLL9	20	31870702	31944963	-0.168668583	PASS
TTN	2	178525989	178830802	0.039726562	PASS
TTPA	8	63048553	63086053	-0.201142258	PASS
TTPAL	20	44475886	44494603	-0.168668583	PASS
TTR	18	31557010	31599021	-0.376397843	PASS
TTYH1	19	54415219	54436900	-0.146906334	PASS
TTYH2	17	74213514	74262020	0.088516311	PASS
TTYH3	7	2631951	2664802	-0.69513118	PASS
TUB	11	8019244	8106112	-0.405444717	PASS
TUBA1A	12	49184796	49189324	-0.202191512	PASS
TUBA1B	12	49127782	49131397	-0.202191512	PASS
TUBA1C	12	49188736	49274603	-0.202191512	PASS
TUBA3C	13	19173770	19181852	-0.390786075	PASS
TUBA3D	2	131476093	131482934	-0.475215623	PASS
TUBA3E	2	130191743	130198461	-0.475215623	PASS
TUBA4A	2	219249711	219278170	0.025928102	PASS
TUBA4B	2	219253243	219272188	0.025928102	PASS
TUBA8	22	18110331	18146554	-0.575930959	PASS
TUBAL3	10	5393098	5404830	0.039811807	PASS
TUBB	6	30720201	30725426	0.048820648	PASS
TUBB1	20	59019254	59026654	-0.168668583	PASS
TUBB2A	6	3153669	3157526	0.048820648	PASS
TUBB2B	6	3224261	3231730	0.048820648	PASS
TUBB3	16	89921392	89938761	-0.371357732	PASS
TUBB4A	19	6494319	6502848	0.079632875	PASS
TUBB4B	9	137241213	137243707	0.365190713	suppress_segment_too_large
TUBB6	18	12307669	12344320	-0.052305056	suppress_segment_too_large
TUBB8	10	46892	74163	0.039811807	PASS
TUBB8P12	18	47390	49557	-0.052305056	suppress_segment_too_large
TUBD1	17	59859482	59892945	0.088516311	PASS

TUBE1	6	112070777	112087529	-0.368590549	PASS
TUBG1	17	42609676	42615234	0.088516311	PASS
TUBG2	17	42659305	42667006	0.088516311	PASS
TUBGCP2	10	133278630	133312337	0.063874692	PASS
TUBGCP3	13	112485005	112588205	-0.390786075	PASS
TUBGCP4	15	43369221	43409771	-0.396255771	PASS
TUBGCP5	15	22983192	23039673	-0.396255771	PASS
TUBGCP6	22	50217689	50244992	-0.808211634	PASS
TUFM	16	28842411	28846408	0.069936844	PASS
TUFT1	1	151540305	151583583	0.103015788	PASS
TULP1	6	35497874	35512938	0.048820648	PASS
TULP2	19	48880965	48898733	0.14996833	PASS
TULP3	12	2877223	2941140	-0.086128028	PASS
TULP4	6	158232236	158511828	-0.035448111	PASS
TUNAR	14	95876392	95925571	-0.192197564	PASS
TUSC1	9	25676389	25678440	-0.310444659	PASS
TUSC2	3	50320027	50328251	-0.234617007	PASS
TUSC3	8	15417215	15766649	-0.345962679	PASS
TUT1	11	62575045	62592177	0.182439925	suppress_segment_too_large
TUT4	1	52408282	52553487	0.033763446	PASS
TUT7	9	86287733	86354454	0.365190713	suppress_segment_too_large
TVP23A	16	10760919	10818794	0.069936844	PASS
TVP23B	17	18780995	18806714	0.024610076	PASS
TVP23C	17	15502264	15563595	-0.139031004	PASS
TVP23C-C	17	15436021	15563561	-0.139031004	PASS
TWF1	12	43793723	43806375	-0.202191512	PASS
TWF2	3	52228610	52239260	-0.234617007	PASS
TWIST1	7	19020991	19117672	-0.69513118	PASS
TWIST2	2	238848032	238910543	0.025928102	PASS
TWISTNB	7	19695462	19709087	-0.69513118	PASS
TWNK	10	100987367	100994401	-0.367947924	PASS
TWSG1	18	9334767	9402420	-0.052305056	suppress_segment_too_large
TXK	4	48066393	48134256	-0.011382299	PASS
TXLNA	1	32179686	32198285	0.033763446	PASS
TXLNB	6	139240061	139292139	-0.035448111	PASS
TXLNG	23	16786427	16844519	-0.012567603	PASS
TXN	9	110243811	110256640	0.365190713	suppress_segment_too_large
TXN2	22	36467036	36482030	-0.575930959	PASS
TXNDC11	16	11679080	11742878	0.069936844	PASS
TXNDC12	1	52020131	52056171	0.033763446	PASS
TXNDC15	5	134873803	134901525	-0.293443042	PASS
TXNDC16	14	52430590	52552522	0.060212819	PASS
TXNDC17	17	6640758	6644541	0.128406487	PASS
TXNDC2	18	9885726	9889275	-0.052305056	suppress_segment_too_large
TXNDC5	6	7881517	7910814	0.048820648	PASS
TXNDC8	9	110303521	110337884	0.365190713	suppress_segment_too_large

TXNDC9	2	99318982	99340702	0.048238121	PASS
TXNIP	1	145992435	145996600	-0.157385092	PASS
TXNL1	18	56597208	56651600	-0.376397843	PASS
TXNL4A	18	79970811	80033949	-0.376397843	PASS
TXNL4B	16	72044289	72094431	-0.371357732	PASS
TXNRD1	12	104215779	104350305	0.202141228	PASS
TXNRD2	22	19875517	19941992	-0.575930959	PASS
TXNRD3	3	126571779	126655155	0.313454204	suppress_segment_too_large
TYK2	19	10350529	10380676	0.093487731	suppress_segment_too_large
TYMP	22	50525752	50530056	-0.808211634	PASS
TYMS	18	657604	673578	-0.052305056	suppress_segment_too_large
TYR	11	89177452	89295759	0.062969153	PASS
TYRO3	15	41557675	41583586	-0.396255771	PASS
TYROBP	19	35904401	35908295	0.093487731	suppress_segment_too_large
TYRP1	9	12685439	12710290	-0.310444659	PASS
TYSND1	10	70137981	70146676	0.072405807	PASS
TYW1	7	66995173	67239519	-0.750061359	PASS
TYW1B	7	72558744	72828198	-0.262778904	PASS
TYW3	1	74733152	74766678	0.033763446	PASS
TYW5	2	199929975	199955736	0.025928102	PASS
U2AF1	21	43092956	43107587	-0.064425456	suppress_segment_too_large
U2AF1L4	19	35742464	35745445	0.093487731	suppress_segment_too_large
U2AF1L5	21	6484623	6499261	-0.304975024	PASS
U2AF2	19	55654146	55674715	0.121336758	PASS
U2SURP	3	142964497	143060546	0.318278996	suppress_segment_too_large
U52112.1	23	153872221	153909223	-0.012567603	PASS
UACA	15	70654554	70763593	-0.697600509	PASS
UAP1	1	162561506	162599842	-0.027073548	PASS
UAP1L1	9	137077501	137084539	0.365190713	suppress_segment_too_large
UBA1	23	47190861	47215128	-0.012567603	PASS
UBA2	19	34428352	34471251	0.093487731	suppress_segment_too_large
UBA3	3	69054730	69080408	-0.234617007	PASS
UBA5	3	132654446	132678097	0.313454204	suppress_segment_too_large
UBA52	19	18571730	18577550	0.093487731	suppress_segment_too_large
UBA6	4	67612652	67701179	-0.011382299	PASS
UBA7	3	49805207	49813946	-0.234617007	PASS
UBAC1	9	135932969	135961380	0.365190713	suppress_segment_too_large
UBAC2	13	99200774	99386434	-0.390786075	PASS
UBALD1	16	4608883	4615027	0.069936844	PASS
UBALD2	17	76265202	76271299	0.088516311	PASS
UBAP1	9	34179005	34252523	-0.310444659	PASS
UBAP1L	15	65092770	65115197	-0.697600509	PASS
UBAP2	9	33921693	34048949	-0.310444659	PASS
UBAP2L	1	154220179	154271510	0.103015788	PASS
UBASH3A	21	42403447	42447681	-0.064425456	suppress_segment_too_large
UBASH3B	11	122655675	122814473	-0.356188624	PASS

UBB	17	16380798	16382745		0.024610076	PASS
UBC	12	124911604	124917368		0.202141228	PASS
UBD	6	29555515	29559925		0.048820648	PASS
UBE2A	23	119574467	119591083		-0.012567603	PASS
UBE2B	5	134371179	134391992		-0.293443042	PASS
UBE2C	20	45812576	45816957		-0.168668583	PASS
UBE2D1	10	58334975	58370753		0.072405807	PASS
UBE2D2	5	139526431	139628433		-0.293443042	PASS
UBE2D3	4	102794383	102868896		-0.011382299	PASS
UBE2D4	7	43926438	43956136		-0.69513118	PASS
UBE2E1	3	23805903	23891316		0.311018244	suppress_segment_too_large
UBE2E2	3	23203020	23591793		0.311018244	suppress_segment_too_large
UBE2E3	2	180967248	181076585		0.039726562	PASS
UBE2F	2	237966827	238042782		0.025928102	PASS
UBE2F-SC	2	237967014	238099412		0.025928102	PASS
UBE2G1	17	4269259	4366628		0.128406487	PASS
UBE2G2	21	44768580	44802019		-0.064425456	suppress_segment_too_large
UBE2H	7	129830732	129952949		-0.388946609	PASS
UBE2I	16	1308880	1327018		0.069936844	PASS
UBE2J1	6	89326625	89352848		-0.368590549	PASS
UBE2J2	1	1253909	1273885		0.373964873	suppress_segment_too_large
UBE2K	4	39698044	39782792		-0.011382299	PASS
UBE2L3	22	21549447	21624034		-0.575930959	PASS
UBE2L5	13	30422488	30428616		-0.390786075	PASS
UBE2L6	11	57551656	57568284		-0.129266491	PASS
UBE2M	19	58555712	58558960		0.063781462	PASS
UBE2N	12	93405673	93442262		0.202141228	PASS
UBE2O	17	76389451	76453206		0.088516311	PASS
UBE2Q1	1	154548577	154559028		0.103015788	PASS
UBE2Q2	15	75843285	75901078		-0.697600509	PASS
UBE2Q2L	15	84172490	84182234		-0.697600509	PASS
UBE2QL1	5	6448623	6494909		0.055007813	PASS
UBE2R2	9	33817567	33920404		-0.310444659	PASS
UBE2S	19	55399745	55407777		0.121336758	PASS
UBE2T	1	202331657	202341980		-0.256698369	PASS
UBE2U	1	64203627	64267368		0.033763446	PASS
UBE2V1	20	50081124	50115959		-0.168668583	PASS
UBE2V2	8	48008400	48064708		-0.201142258	PASS
UBE2W	8	73780097	73878910		-0.201142258	PASS
UBE2Z	17	48908369	48929056		0.088516311	PASS
UBE3A	15	25333728	25439056		-0.396255771	PASS
UBE3B	12	109477402	109536705		0.202141228	PASS
UBE3C	7	157138913	157269372		-0.411415677	PASS
UBE3D	6	82892398	83065841		-0.368590549	PASS
UBE4A	11	118359585	118399211		-0.356188624	PASS
UBE4B	1	10032832	10181239		0.373964873	suppress_segment_too_large

UBFD1	16	23557362	23574389	0.069936844	PASS
UBIAD1	1	11273206	11296049	0.373964873	suppress_segment_too_large
UBL3	13	29764371	29850684	-0.390786075	PASS
UBL4A	23	154483717	154486670	-0.012567603	PASS
UBL4B	1	110112468	110113945	0.033763446	PASS
UBL5	19	9827892	9830115	0.093487731	suppress_segment_too_large
UBL7	15	74445977	74461182	-0.697600509	PASS
UBLCP1	5	159263081	159286040	-0.150979337	PASS
UBN1	16	4846665	4882360	0.069936844	PASS
UBN2	7	139230356	139308236	-0.388946609	PASS
UBOX5	20	3107573	3160196	-0.168668583	PASS
UBP1	3	33388336	33441371	0.311018244	suppress_segment_too_large
UBQLN1	9	83659963	83708203	0.365190713	suppress_segment_too_large
UBQLN2	23	56563639	56567868	-0.012567603	PASS
UBQLN3	11	5507300	5509985	-0.957525224	PASS
UBQLN4	1	156035301	156053794	0.103015788	PASS
UBQLNL	11	5514393	5516705	-0.957525224	PASS
UBR1	15	42942897	43106113	-0.396255771	PASS
UBR2	6	42564062	42693504	0.048820648	PASS
UBR3	2	169827458	170084131	0.039726562	PASS
UBR4	1	19074506	19210276	0.033763446	PASS
UBR5	8	102253012	102412841	0.279445683	PASS
UBR7	14	93207056	93229215	-0.192197564	PASS
UBTD1	10	97498868	97571209	-0.367947924	PASS
UBTD2	5	172209640	172284071	-0.150979337	PASS
UBTF	17	44205033	44221626	0.088516311	PASS
UBTFL1	11	90085950	90087131	0.062969153	PASS
UBXN1	11	62676498	62679117	0.182439925	suppress_segment_too_large
UBXN10	1	20186085	20196048	0.033763446	PASS
UBXN11	1	26281328	26318363	0.033763446	PASS
UBXN2A	2	23927285	24004909	0.040498426	PASS
UBXN2B	8	58411264	58451501	-0.201142258	PASS
UBXN4	2	135741619	135785055	-0.475215623	PASS
UBXN6	19	4444999	4457822	0.079632875	PASS
UBXN7	3	196347662	196432474	0.326834516	PASS
UBXN8	8	30732247	30767006	-0.345962679	PASS
UCHL1	4	41256413	41268455	-0.011382299	PASS
UCHL3	13	75549480	75606020	-0.390786075	PASS
UCHL5	1	193012250	193060080	-0.027073548	PASS
UCK1	9	131523801	131531268	0.365190713	suppress_segment_too_large
UCK2	1	165827531	165911618	-0.027073548	PASS
UCKL1	20	63939829	63956415	-0.168668583	PASS
UCMA	10	13221767	13234334	0.039811807	PASS
UCN	2	27307400	27308445	0.040498426	PASS
UCN2	3	48561727	48563773	-0.234617007	PASS
UCN3	10	5365009	5374692	0.039811807	PASS

UCP1	4	140559434	140568805	-0.011382299	PASS
UCP2	11	73974667	73983307	0.062969153	PASS
UCP3	11	74000281	74009435	0.062969153	PASS
UEVLD	11	18529609	18588747	-0.405444717	PASS
UFC1	1	161152776	161158856	-0.027073548	PASS
UFD1	22	19449910	19479215	-0.575930959	PASS
UFL1	6	96521595	96555276	-0.368590549	PASS
UFM1	13	38349849	38363619	-0.390786075	PASS
UFSP1	7	100888723	100889718	-0.262778904	PASS
UFSP2	4	185399540	185425985	-0.462744716	PASS
UGCG	9	111896766	111935369	0.365190713	suppress_segment_too_large
UGDH	4	39498755	39528311	-0.011382299	PASS
UGGT1	2	128091200	128195677	-0.475215623	PASS
UGGT2	13	95801580	96053482	-0.390786075	PASS
UGP2	2	63840940	63891562	-0.372959126	PASS
UGT1A1	2	233760248	233773299	0.025928102	PASS
UGT1A10	2	233636454	233773305	0.025928102	PASS
UGT1A3	2	233729108	233773299	0.025928102	PASS
UGT1A4	2	233718778	233773299	0.025928102	PASS
UGT1A5	2	233712992	233773299	0.025928102	PASS
UGT1A6	2	233691607	233773300	0.025928102	PASS
UGT1A7	2	233681938	233773299	0.025928102	PASS
UGT1A8	2	233617645	233773310	0.025928102	PASS
UGT1A9	2	233671853	233773300	0.025928102	PASS
UGT2A1	4	69588417	69653249	-0.011382299	PASS
UGT2A2	4	69589309	69639642	-0.011382299	PASS
UGT2A3	4	68928463	68951791	-0.011382299	PASS
UGT2B10	4	68815993	68831196	-0.011382299	PASS
UGT2B11	4	69199951	69214731	-0.011382299	PASS
UGT2B15	4	68646630	68670628	-0.011382299	PASS
UGT2B17	4	68537184	68568527	-0.011382299	PASS
UGT2B28	4	69280499	69295050	-0.011382299	PASS
UGT2B4	4	69480165	69526014	-0.011382299	PASS
UGT2B7	4	69051363	69112987	-0.011382299	PASS
UGT3A1	5	35951010	36001028	0.055007813	PASS
UGT3A2	5	36035017	36071358	0.055007813	PASS
UGT8	4	114598455	114678224	-0.011382299	PASS
UHMK1	1	162497251	162529629	-0.027073548	PASS
UHRF1	19	4903080	4962154	0.079632875	PASS
UHRF1BP	6	34792015	34883138	0.048820648	PASS
UHRF1BP	12	100028455	100142848	0.202141228	PASS
UHRF2	9	6413151	6507054	-0.310444659	PASS
UIMC1	5	176905005	177022633	-0.150979337	PASS
ULBP1	6	149964007	149973710	-0.035448111	PASS
ULBP2	6	149942000	149949235	-0.035448111	PASS
ULBP3	6	150063150	150069095	-0.035448111	PASS

ULK1	12	131894651	131923167	0.270848837	PASS
ULK2	17	19770829	19867936	0.024610076	PASS
ULK3	15	74836116	74843346	-0.697600509	PASS
ULK4	3	41246599	41962430	-0.234617007	PASS
UMAD1	7	7640711	7968020	-0.69513118	PASS
UMOD	16	20333052	20356301	0.069936844	PASS
UMODL1	21	42062959	42143453	-0.064425456	suppress_segment_too_large
UMPS	3	124730366	124749273	0.313454204	suppress_segment_too_large
UNC119	17	28546707	28552668	0.060555011	PASS
UNC119B	12	120710435	120723640	0.202141228	PASS
UNC13A	19	17601328	17688365	0.093487731	suppress_segment_too_large
UNC13B	9	35161992	35405338	-0.310444659	PASS
UNC13C	15	53978201	54633414	-0.697600509	PASS
UNC13D	17	75827225	75844717	0.088516311	PASS
UNC45A	15	90930180	90954093	-0.697600509	PASS
UNC45B	17	35147817	35189345	0.060555011	PASS
UNC50	2	98608579	98618515	0.048238121	PASS
UNC5A	5	176810477	176880898	-0.150979337	PASS
UNC5B	10	71212570	71302864	0.072405807	PASS
UNC5C	4	95162504	95549206	-0.011382299	PASS
UNC5CL	6	41026911	41039217	0.048820648	PASS
UNC5D	8	35235457	35796550	-0.345962679	PASS
UNC79	14	93333219	93707876	-0.192197564	PASS
UNC80	2	209771993	209999300	0.025928102	PASS
UNC93A	6	167271169	167316019	-0.035448111	PASS
UNC93B1	11	67991104	68004982	0.182439925	suppress_segment_too_large
UNCX	7	1232907	1237318	-0.69513118	PASS
UNG	12	109097574	109110992	0.202141228	PASS
UNK	17	75784771	75825799	0.088516311	PASS
UNKL	16	1363205	1414751	0.069936844	PASS
UPB1	22	24494107	24528390	-0.575930959	PASS
UPF1	19	18831938	18868236	0.093487731	suppress_segment_too_large
UPF2	10	11920022	12043170	0.039811807	PASS
UPF3A	13	114281584	114305817	-0.390786075	PASS
UPF3B	23	119805311	119852998	-0.012567603	PASS
UPK1A	19	35666516	35678483	0.093487731	suppress_segment_too_large
UPK1B	3	119173517	119205153	0.313454204	suppress_segment_too_large
UPK2	11	118925164	118958559	-0.356188624	PASS
UPK3A	22	45284982	45295874	-0.808211634	PASS
UPK3B	7	76510428	76516521	-0.262778904	PASS
UPK3BL1	7	102637025	102642791	-0.388946609	PASS
UPP1	7	48088628	48108733	-0.69513118	PASS
UPP2	2	157876702	158136154	0.039726562	PASS
UPRT	23	75274085	75304600	-0.012567603	PASS
UQCC1	20	35302566	35412141	-0.168668583	PASS
UQCC2	6	33694293	33711727	0.048820648	PASS

UQCC3	11	62670273	62673687	0.182439925	suppress_segment_too_large
UQCR10	22	29767369	29770413	-0.575930959	PASS
UQCR11	19	1597172	1605490	0.079632875	PASS
UQCRB	8	96225920	96235634	0.279445683	PASS
UQCRC1	3	48599002	48610976	-0.234617007	PASS
UQCRC2	16	21952660	21983660	0.069936844	PASS
UQCRFS1	19	29205321	29213541	0.093487731	suppress_segment_too_large
UQCRH	1	46303631	46316776	0.033763446	PASS
UQCRHL	1	15807169	15809348	0.033763446	PASS
UQCRQ	5	132866560	132868031	-0.293443042	PASS
URAD	13	27977714	27988654	-0.390786075	PASS
URB1	21	32311018	32393026	0.12860881	suppress_segment_too_large
URB2	1	229626234	229660199	-0.256698369	PASS
URGCP	7	43875894	43926411	-0.69513118	PASS
URGCP-M	7	43866558	43906589	-0.69513118	PASS
URI1	19	29923644	30016608	0.093487731	suppress_segment_too_large
URM1	9	128371319	128392016	0.365190713	suppress_segment_too_large
UROC1	3	126481281	126517773	0.313454204	suppress_segment_too_large
UROD	1	45012147	45015575	0.033763446	PASS
UROS	10	125784980	125823285	0.063874692	PASS
USB1	16	57999546	58021618	-0.371357732	PASS
USE1	19	17215346	17219829	0.093487731	suppress_segment_too_large
USF1	1	161039251	161045977	-0.027073548	PASS
USF2	19	35268978	35279821	0.093487731	suppress_segment_too_large
USF3	3	113648385	113696646	0.313454204	suppress_segment_too_large
USH1C	11	17493895	17544416	-0.405444717	PASS
USH1G	17	74916084	74923256	0.088516311	PASS
USH2A	1	215622894	216423396	-0.256698369	PASS
USHBP1	19	17249176	17282786	0.093487731	suppress_segment_too_large
USO1	4	75724593	75814286	-0.011382299	PASS
USP1	1	62436297	62451804	0.033763446	PASS
USP10	16	84699978	84779922	-0.371357732	PASS
USP11	23	47232690	47248328	-0.012567603	PASS
USP12	13	27066142	27171896	-0.390786075	PASS
USP13	3	179652755	179789401	0.318278996	suppress_segment_too_large
USP14	18	158383	214629	-0.052305056	suppress_segment_too_large
USP15	12	62260338	62417431	-0.202191512	PASS
USP16	21	29024629	29054488	0.12860881	suppress_segment_too_large
USP17L1	8	7332387	7333979	-0.345962679	PASS
USP17L10	4	9210657	9212608	0.279089364	PASS
USP17L17	4	9243879	9245830	0.279089364	PASS
USP17L18	4	9248630	9250581	0.279089364	PASS
USP17L2	8	12137168	12139077	-0.345962679	PASS
USP17L22	4	9267619	9269570	0.279089364	PASS
USP17L4	8	7337115	7338707	-0.345962679	PASS
USP17L7	8	12132417	12134438	-0.345962679	PASS

USP18	22	18149899	18177397	-0.575930959	PASS
USP19	3	49108046	49120938	-0.234617007	PASS
USP2	11	119355215	119381726	-0.356188624	PASS
USP20	9	129834698	129881838	0.365190713	suppress_segment_too_large
USP21	1	161159450	161165723	-0.027073548	PASS
USP22	17	20999593	21043760	0.024610076	PASS
USP24	1	55066359	55215113	0.033763446	PASS
USP25	21	15730025	15880069	0.12860881	suppress_segment_too_large
USP26	23	133024631	133097109	-0.012567603	PASS
USP27X	23	49879948	49882565	-0.012567603	PASS
USP28	11	113797874	113875570	-0.356188624	PASS
USP29	19	57119138	57131926	-0.180829161	PASS
USP3	15	63504511	63594640	-0.697600509	PASS
USP30	12	109023089	109088026	0.202141228	PASS
USP31	16	23061406	23149270	0.069936844	PASS
USP32	17	60179094	60422470	0.088516311	PASS
USP33	1	77695987	77759852	0.033763446	PASS
USP34	2	61187463	61470769	-0.372959126	PASS
USP35	11	78188812	78214711	0.062969153	PASS
USP36	17	78787381	78841441	0.088516311	PASS
USP37	2	218450251	218568361	0.025928102	PASS
USP38	4	143184917	143223830	-0.011382299	PASS
USP39	2	85602856	85649282	0.066915641	PASS
USP4	3	49277831	49340712	-0.234617007	PASS
USP40	2	233475520	233566782	0.025928102	PASS
USP41	22	20350578	20390758	-0.575930959	PASS
USP42	7	6104884	6161564	-0.69513118	PASS
USP43	17	9644698	9729691	-0.139031004	PASS
USP44	12	95516560	95551490	0.202141228	PASS
USP45	6	99432379	99521728	-0.368590549	PASS
USP46	4	52590972	52659335	-0.011382299	PASS
USP47	11	11841423	11959323	-0.405444717	PASS
USP48	1	21678298	21783606	0.033763446	PASS
USP49	6	41789896	41895361	0.048820648	PASS
USP5	12	6852128	6866632	-0.086128028	PASS
USP50	15	50500562	50546708	-0.396255771	PASS
USP51	23	55484616	55489202	-0.012567603	PASS
USP53	4	119212587	119295517	-0.011382299	PASS
USP54	10	73497538	73625953	0.072405807	PASS
USP6	17	5116438	5175034	0.128406487	PASS
USP6NL	10	11453946	11611754	0.039811807	PASS
USP7	16	8892094	8964514	0.069936844	PASS
USP8	15	50424380	50514419	-0.396255771	PASS
USP9X	23	41085635	41236579	-0.012567603	PASS
USPL1	13	30617693	30660770	-0.390786075	PASS
UST	6	148747328	149076990	-0.035448111	PASS

UTF1	10	133230274	133231558	0.063874692	PASS
UTP11	1	38009258	38024824	0.033763446	PASS
UTP14A	23	129906121	129929761	-0.012567603	PASS
UTP14C	13	52024691	52033600	-0.390786075	PASS
UTP15	5	73565443	73583377	-0.293443042	PASS
UTP18	17	51260528	51297936	0.088516311	PASS
UTP20	12	101280109	101386616	0.202141228	PASS
UTP23	8	116766503	116849463	0.279445683	PASS
UTP25	1	209828007	209857565	-0.256698369	PASS
UTP3	4	70688479	70690551	-0.011382299	PASS
UTP4	16	69131291	69231130	-0.371357732	PASS
UTP6	17	31860899	31901765	0.060555011	PASS
UTRN	6	144285701	144853034	-0.035448111	PASS
UTS2	1	7843083	7853512	0.373964873	suppress_segment_too_large
UTS2B	3	191267168	191330536	-0.627320105	PASS
UTS2R	17	82374230	82375586	0.088516311	PASS
UVRAG	11	75815167	76143195	0.062969153	PASS
UVSSA	4	1347266	1395992	0.279089364	PASS
UXS1	2	106093303	106194339	-0.532032081	PASS
UXT	23	47651798	47659161	-0.012567603	PASS
VAC14	16	70687439	70801161	-0.371357732	PASS
VAMP1	12	6462237	6470987	-0.086128028	PASS
VAMP2	17	8159149	8163546	0.128406487	PASS
VAMP3	1	7771269	7781432	0.373964873	suppress_segment_too_large
VAMP4	1	171700160	171742247	-0.027073548	PASS
VAMP5	2	85584408	85593412	0.066915641	PASS
VAMP7	23	155881293	155943769	-0.012567603	PASS
VAMP8	2	85561562	85582031	0.066915641	PASS
VANGL1	1	115641953	115698224	0.033763446	PASS
VANGL2	1	160400586	160428678	-0.027073548	PASS
VAPA	18	9914002	9960021	-0.052305056	suppress_segment_too_large
VAPB	20	58389122	58451101	-0.168668583	PASS
VARS	6	31777518	31795953	0.048820648	PASS
VARS2	6	30908242	30926459	0.048820648	PASS
VASH1	14	76762189	76783015	-0.192197564	PASS
VASH2	1	212950520	212992037	-0.256698369	PASS
VASN	16	4371848	4383528	0.069936844	PASS
VASP	19	45506579	45526983	0.14996833	PASS
VAT1	17	43014605	43025123	0.088516311	PASS
VAT1L	16	77788530	77980107	-0.371357732	PASS
VAV1	19	6772714	6857366	0.079632875	PASS
VAV2	9	133761894	133992604	0.365190713	suppress_segment_too_large
VAV3	1	107571160	107965144	0.033763446	PASS
VAX1	10	117128521	117138301	0.063874692	PASS
VAX2	2	70900590	70965373	-0.372959126	PASS
VBP1	23	155197007	155239817	-0.012567603	PASS

VCAM1	1	100719742	100739045	0.033763446	PASS
VCAN	5	83471465	83582303	-0.293443042	PASS
VCL	10	73995193	74121363	0.072405807	PASS
VCP	9	35056064	35073249	-0.310444659	PASS
VCPIP1	8	66628487	66667217	-0.201142258	PASS
VCPKMT	14	50108632	50116600	0.060212819	PASS
VCX	23	7842262	7844143	-0.012567603	PASS
VCX2	23	8169944	8171267	-0.012567603	PASS
VCX3A	23	6533618	6535118	-0.012567603	PASS
VCX3B	23	8464830	8466510	-0.012567603	PASS
VDAC1	5	133971915	134005133	-0.293443042	PASS
VDAC2	10	75210154	75231448	0.072405807	PASS
VDAC3	8	42391624	42405897	-0.201142258	PASS
VDR	12	47841537	47943048	-0.202191512	PASS
VEGFA	6	43770184	43786487	0.048820648	PASS
VEGFB	11	64234538	64238793	0.182439925	suppress_segment_too_large
VEGFC	4	176683538	176792727	-0.011382299	PASS
VEGFD	23	15345591	15384376	-0.012567603	PASS
VENTX	10	133237404	133241929	0.063874692	PASS
VEPH1	3	157259742	157533619	0.318278996	suppress_segment_too_large
VEZF1	17	57971547	57988259	0.088516311	PASS
VEZT	12	95217746	95302790	0.202141228	PASS
VGF	7	101162509	101165593	-0.388946609	PASS
VGLL1	23	136532152	136556807	-0.012567603	PASS
VGLL2	6	117265558	117273565	-0.368590549	PASS
VGLL3	3	86876388	86991119	0.313454204	suppress_segment_too_large
VGLL4	3	11556070	11771350	0.311018244	suppress_segment_too_large
VHL	3	10141008	10152220	0.311018244	suppress_segment_too_large
VHLL	1	156298624	156299299	0.103015788	PASS
VIL1	2	218419092	218453295	0.025928102	PASS
VILL	3	37988059	38007188	-0.234617007	PASS
VIM	10	17228259	17237593	0.039811807	PASS
VIP	6	152750798	152759765	-0.035448111	PASS
VIPAS39	14	77426675	77457952	-0.192197564	PASS
VIPR1	3	42489299	42537573	-0.234617007	PASS
VIPR2	7	159028175	159144957	-0.184834798	PASS
VIRMA	8	94487693	94553529	0.279445683	PASS
VIT	2	36696690	36814792	0.040498426	PASS
VKORC1	16	31090842	31095980	0.069936844	PASS
VKORC1L	7	65873166	65959563	-0.750061359	PASS
VLDLR	9	2621834	2660053	-0.310444659	PASS
VMA21	23	151396515	151409364	-0.012567603	PASS
VMAC	19	5904858	5910853	0.079632875	PASS
VMO1	17	4785285	4786433	0.128406487	PASS
VMP1	17	59707192	59842255	0.088516311	PASS
VN1R1	19	57454790	57457142	-0.180829161	PASS

VN1R2	19	53258292	53261837	0.206912349	PASS
VN1R4	19	53266676	53267723	0.206912349	PASS
VNN1	6	132681590	132714049	-0.458570262	PASS
VNN2	6	132743870	132763459	-0.458570262	PASS
VNN3	6	132722787	132734765	-0.458570262	PASS
VOPP1	7	55436056	55572988	-0.750061359	PASS
VPREB1	22	22244675	22245515	-0.575930959	PASS
VPREB3	22	23752743	23754468	-0.575930959	PASS
VPS11	11	119067692	119081978	-0.356188624	PASS
VPS13A	9	77177353	77421541	0.365190713	suppress_segment_too_large
VPS13B	8	99013266	99877580	0.279445683	PASS
VPS13C	15	61852389	62060473	-0.697600509	PASS
VPS13D	1	12230030	12512047	0.003932739	PASS
VPS16	20	2840703	2866732	-0.168668583	PASS
VPS18	15	40894430	40903975	-0.396255771	PASS
VPS25	17	42773436	42779599	0.088516311	PASS
VPS26A	10	69123512	69172861	0.072405807	PASS
VPS26B	11	134224645	134247792	-0.419612865	PASS
VPS26C	21	37223420	37267919	-0.064425456	suppress_segment_too_large
VPS28	8	144423601	144428563	0.307788765	PASS
VPS29	12	110491097	110502117	0.202141228	PASS
VPS33A	12	122229564	122266521	0.202141228	PASS
VPS33B	15	90998416	91022603	-0.697600509	PASS
VPS35	16	46656132	46689518	0.069936844	PASS
VPS35L	16	19555240	19706793	0.069936844	PASS
VPS36	13	52412602	52450628	-0.390786075	PASS
VPS37A	8	17246571	17302427	-0.345962679	PASS
VPS37B	12	122865328	122896444	0.202141228	PASS
VPS37C	11	61130256	61161617	0.182439925	suppress_segment_too_large
VPS37D	7	73667825	73672112	-0.262778904	PASS
VPS39	15	42158701	42208316	-0.396255771	PASS
VPS41	7	38722963	38932394	-0.69513118	PASS
VPS45	1	150067279	150145329	0.103015788	PASS
VPS4A	16	69311356	69326939	-0.371357732	PASS
VPS4B	18	63389190	63422483	-0.376397843	PASS
VPS50	7	93232340	93361121	-0.262778904	PASS
VPS51	11	65089324	65111860	0.182439925	suppress_segment_too_large
VPS52	6	33250272	33272047	0.048820648	PASS
VPS53	17	508668	721717	0.128406487	PASS
VPS54	2	63892146	64019072	-0.372959126	PASS
VPS72	1	151169987	151195321	0.103015788	PASS
VPS8	3	184812143	185052614	0.318278996	suppress_segment_too_large
VPS9D1	16	89707134	89720986	-0.371357732	PASS
VRK1	14	96797304	96931722	-0.192197564	PASS
VRK2	2	57907629	58159920	-0.372959126	PASS
VRK3	19	49976467	50025946	-0.016761391	PASS

VRTN	14	74303069	74360008	0.060212819	PASS
VSIG1	23	108044970	108079184	-0.012567603	PASS
VSIG10	12	118063593	118136026	0.202141228	PASS
VSIG10L	19	51331536	51342124	-0.016761391	PASS
VSIG2	11	124747472	124752238	-0.419612865	PASS
VSIG4	23	66021738	66040125	-0.012567603	PASS
VSIG8	1	159854316	159862657	-0.027073548	PASS
VSIR	10	71747559	71773498	0.072405807	PASS
VSNL1	2	17539126	17657018	0.040498426	PASS
VSTM1	19	54040825	54063953	0.206912349	PASS
VSTM2A	7	54542325	54571080	-0.750061359	PASS
VSTM2B	19	29526499	29564479	0.093487731	suppress_segment_too_large
VSTM2L	20	37903104	37945350	-0.168668583	PASS
VSTM4	10	49014245	49115509	-0.404852538	PASS
VSTM5	11	93818232	93850531	0.062969153	PASS
VSX1	20	25070885	25082365	-0.168668583	PASS
VSX2	14	74239472	74262738	0.060212819	PASS
VTA1	6	142147162	142224689	-0.035448111	PASS
VTCN1	1	117143587	117210960	0.033763446	PASS
VTI1A	10	112446998	112818744	0.063874692	PASS
VTI1B	14	67647075	67674831	0.060212819	PASS
VTN	17	28367276	28373091	0.060555011	PASS
VWA1	1	1434861	1442882	0.373964873	suppress_segment_too_large
VWA2	10	114239330	114291513	0.063874692	PASS
VWA3A	16	22092538	22156966	0.069936844	PASS
VWA3B	2	98087116	98313299	0.048238121	PASS
VWA5A	11	124115362	124147721	-0.949752196	PASS
VWA5B1	1	20290919	20354894	0.033763446	PASS
VWA5B2	3	184230429	184242329	0.318278996	suppress_segment_too_large
VWA7	6	31765590	31777294	0.048820648	PASS
VWA8	13	41566837	41961120	-0.390786075	PASS
VWC2	7	49773661	49921950	-2.348494016	PASS
VWC2L	2	214411065	214578959	0.025928102	PASS
VWCE	11	61258286	61295424	0.182439925	suppress_segment_too_large
VWDE	7	12330885	12403941	-0.69513118	PASS
VWF	12	5948874	6124770	-0.086128028	PASS
VXN	8	66493208	66518524	-0.201142258	PASS
WAC	10	28532493	28623112	0.039811807	PASS
WAPL	10	86435256	86521815	-0.292453993	PASS
WARS	14	100333788	100376805	-0.192197564	PASS
WARS2	1	119031216	119140671	-0.157385092	PASS
WAS	23	48676596	48691427	-0.012567603	PASS
WASF1	6	110099819	110180004	-0.368590549	PASS
WASF2	1	27404226	27490158	0.033763446	PASS
WASF3	13	26557703	26688948	-0.390786075	PASS
WASHC1	9	14521	73865	-0.310444659	PASS

WASHC2A	10	50067888	50133506	-0.404852538	PASS
WASHC2C	10	45727200	45792961	-0.404852538	PASS
WASHC3	12	102012927	102062149	0.202141228	PASS
WASHC4	12	105107324	105169134	0.202141228	PASS
WASHC5	8	125024260	125091840	0.279445683	PASS
WASL	7	123681935	123749067	-0.388946609	PASS
WBP1	2	74458329	74460891	-0.372959126	PASS
WBP11	12	14784579	14803540	0.362961408	suppress_segment_too_large
WBP1L	10	102743901	102834516	-0.367947924	PASS
WBP2	17	75845699	75856507	0.088516311	PASS
WBP2NL	22	41998725	42058456	-0.575930959	PASS
WBP4	13	41061274	41084006	-0.390786075	PASS
WDCP	2	24029340	24049575	0.040498426	PASS
WDFY1	2	223855716	223945387	0.025928102	PASS
WDFY2	13	51584455	51767707	-0.390786075	PASS
WDFY3	4	84669610	84966391	-0.011382299	PASS
WDFY4	10	48684876	48982956	-0.404852538	PASS
WDHD1	14	54938950	55027105	0.060212819	PASS
WDPCP	2	63121383	63827843	-0.372959126	PASS
WDR1	4	10074339	10116949	0.279089364	PASS
WDR11	10	120851175	120909524	0.063874692	PASS
WDR12	2	202874782	203014798	0.025928102	PASS
WDR13	23	48590042	48608867	-0.012567603	PASS
WDR17	4	176065834	176182818	-0.011382299	PASS
WDR18	19	984271	998438	0.079632875	PASS
WDR19	4	39182404	39285810	-0.011382299	PASS
WDR20	14	102139503	102224847	-0.192197564	PASS
WDR24	16	684622	690444	0.069936844	PASS
WDR25	14	100376418	100530303	-0.192197564	PASS
WDR26	1	224385143	224437033	-0.256698369	PASS
WDR27	6	169457212	169702067	-0.035448111	PASS
WDR3	1	117929720	117966542	0.033763446	PASS
WDR31	9	113313222	113340298	0.365190713	suppress_segment_too_large
WDR33	2	127701022	127811187	-0.475215623	PASS
WDR34	9	128633661	128656787	0.365190713	suppress_segment_too_large
WDR35	2	19910260	19990131	0.040498426	PASS
WDR36	5	111091716	111130502	-0.293443042	PASS
WDR37	10	1049538	1132384	0.039811807	PASS
WDR38	9	124853417	124857890	0.365190713	suppress_segment_too_large
WDR4	21	42843094	42879568	-0.064425456	suppress_segment_too_large
WDR41	5	77425970	77620611	-0.293443042	PASS
WDR43	2	28894643	28948222	0.040498426	PASS
WDR44	23	118346073	118449961	-0.012567603	PASS
WDR45	23	49071470	49101170	-0.012567603	PASS
WDR45B	17	82614562	82648553	0.088516311	PASS
WDR46	6	33279108	33289527	0.048820648	PASS

WDR47	1	108970214	109042113	0.033763446	PASS
WDR48	3	39051998	39096671	-0.234617007	PASS
WDR49	3	167478684	167653983	0.318278996	suppress_segment_too_large
WDR5	9	134135365	134159968	0.365190713	suppress_segment_too_large
WDR53	3	196554177	196568674	0.326834516	PASS
WDR54	2	74421678	74425755	-0.372959126	PASS
WDR55	5	140664676	140674124	-0.293443042	PASS
WDR59	16	74871367	75000173	-0.371357732	PASS
WDR5B	3	122412332	122416051	0.313454204	suppress_segment_too_large
WDR6	3	49007062	49015953	-0.234617007	PASS
WDR60	7	158856578	158956747	-0.184834798	PASS
WDR61	15	78277835	78299794	-0.697600509	PASS
WDR62	19	36054881	36105106	0.093487731	suppress_segment_too_large
WDR63	1	84999147	85133138	0.033763446	PASS
WDR64	1	241652278	241802133	-0.648598641	PASS
WDR66	12	121917862	122003927	0.202141228	PASS
WDR7	18	56651343	57036606	-0.376397843	PASS
WDR70	5	37379212	37753435	0.055007813	PASS
WDR72	15	53513741	53762878	-0.697600509	PASS
WDR73	15	84639281	84654343	-0.697600509	PASS
WDR74	11	62832342	62841809	0.182439925	suppress_segment_too_large
WDR75	2	189441433	189475565	0.025928102	PASS
WDR76	15	43826963	43868419	-0.396255771	PASS
WDR77	1	111439890	111449376	0.033763446	PASS
WDR78	1	66812885	66924887	0.033763446	PASS
WDR81	17	1716523	1738599	0.128406487	PASS
WDR82	3	52254421	52288020	-0.234617007	PASS
WDR83	19	12666800	12675832	0.093487731	suppress_segment_too_large
WDR83OS	19	12668071	12669489	0.093487731	suppress_segment_too_large
WDR86	7	151375909	151410727	-0.388946609	PASS
WDR87	19	37884823	37906677	0.093487731	suppress_segment_too_large
WDR88	19	33132090	33175795	0.093487731	suppress_segment_too_large
WDR89	14	63597039	63641861	0.060212819	PASS
WDR90	16	649311	667833	0.069936844	PASS
WDR91	7	135183839	135211534	-0.388946609	PASS
WDR92	2	68122936	68157560	-0.372959126	PASS
WDR93	15	89690797	89743638	-0.697600509	PASS
WDR97	8	144107726	144118315	0.307788765	PASS
WDSUB1	2	159235793	159286799	0.039726562	PASS
WDTC1	1	27234516	27308633	0.033763446	PASS
WDYHV1	8	123416725	123470028	0.279445683	PASS
WEE1	11	9573681	9593457	-0.405444717	PASS
WEE2	7	141708353	141731271	-0.388946609	PASS
WFDC1	16	84294646	84329851	-0.371357732	PASS
WFDC10A	20	45629526	45631196	-0.168668583	PASS
WFDC10B	20	45684653	45705019	-0.168668583	PASS

WFDC11	20	45648563	45670270	-0.168668583	PASS
WFDC12	20	45123425	45124465	-0.168668583	PASS
WFDC13	20	45702016	45708817	-0.168668583	PASS
WFDC2	20	45469706	45481532	-0.168668583	PASS
WFDC3	20	45747944	45791932	-0.168668583	PASS
WFDC5	20	45109452	45115172	-0.168668583	PASS
WFDC6	20	45534196	45539495	-0.168668583	PASS
WFDC8	20	45551153	45579326	-0.168668583	PASS
WFDC9	20	45607939	45631268	-0.168668583	PASS
WFIKKN1	16	629239	634116	0.069936844	PASS
WFIKKN2	17	50834650	50842348	0.088516311	PASS
WFS1	4	6269849	6303265	0.279089364	PASS
WHAMM	15	82809628	82836108	-0.697600509	PASS
WHRN	9	114402080	114505450	0.365190713	suppress_segment_too_large
WIF1	12	65050626	65121566	-0.202191512	PASS
WIPF1	2	174559572	174682916	0.039726562	PASS
WIPF2	17	40219304	40284136	0.060555011	PASS
WIPF3	7	29806486	29917066	-0.69513118	PASS
WIPI1	17	68420948	68457513	0.088516311	PASS
WIPI2	7	5190188	5233826	-0.69513118	PASS
WISP1	8	133191039	133231690	0.113969953	PASS
WISP2	20	44714844	44728509	-0.168668583	PASS
WISP3	6	112054072	112070969	-0.368590549	PASS
WIZ	19	15419980	15449951	0.093487731	suppress_segment_too_large
WLS	1	68098473	68233120	0.033763446	PASS
WNK1	12	752593	911452	-0.086128028	PASS
WNK2	9	93184916	93320572	0.365190713	suppress_segment_too_large
WNK3	23	54192823	54358642	-0.012567603	PASS
WNK4	17	42780678	42796936	0.088516311	PASS
WNT1	12	48978453	48981676	-0.202191512	PASS
WNT10A	2	218880363	218899581	0.025928102	PASS
WNT10B	12	48965340	48971763	-0.202191512	PASS
WNT11	11	76186325	76210736	0.062969153	PASS
WNT16	7	121325367	121341104	-0.388946609	PASS
WNT2	7	117275451	117323289	-0.388946609	PASS
WNT2B	1	112466541	112530165	0.033763446	PASS
WNT3	17	46762506	46833154	0.088516311	PASS
WNT3A	1	228007051	228061260	-0.256698369	PASS
WNT4	1	22117305	22143969	0.033763446	PASS
WNT5A	3	55465715	55490539	-0.234617007	PASS
WNT5B	12	1529891	1647243	-0.086128028	PASS
WNT6	2	218859821	218874233	0.025928102	PASS
WNT7A	3	13816258	13880121	0.311018244	suppress_segment_too_large
WNT7B	22	45920362	45977129	-0.808211634	PASS
WNT8A	5	138083892	138092365	-0.293443042	PASS
WNT8B	10	100463041	100483744	-0.367947924	PASS

WNT9A	1	227918656	227947898		-0.256698369 PASS
WNT9B	17	46833201	46886730		0.088516311 PASS
WRAP53	17	7686071	7703502		0.128406487 PASS
WRAP73	1	3630767	3652761		0.373964873 suppress_segment_too_large
WRB	21	39377698	39428528		-0.064425456 suppress_segment_too_large
WRB-SH3I	21	39380332	39515506		-0.064425456 suppress_segment_too_large
WRN	8	31033801	31173769		-0.345962679 PASS
WRNIP1	6	2765414	2786952		0.048820648 PASS
WSB1	17	27294076	27315926		0.060555011 PASS
WSB2	12	118032694	118062430		0.202141228 PASS
WSCD1	17	6057807	6124427		0.128406487 PASS
WSCD2	12	108129471	108250537		0.202141228 PASS
WT1	11	32387775	32435630		-0.405444717 PASS
WTAP	6	159725585	159756319		-0.035448111 PASS
WTIP	19	34481638	34512304		0.093487731 suppress_segment_too_large
WWC1	5	168291651	168472303		-0.150979337 PASS
WWC2	4	183099293	183320777		-0.462744716 PASS
WWC3	23	10015562	10144478		-0.012567603 PASS
WWOX	16	78099413	79212667		-0.371357732 PASS
WWP1	8	86342738	86478420		0.279445683 PASS
WWP2	16	69762306	69941741		-0.371357732 PASS
WWTR1	3	149517235	149736714		0.318278996 suppress_segment_too_large
XAB2	19	7619525	7629565		0.079632875 PASS
XAF1	17	6755447	6775647		0.128406487 PASS
XAGE1B	23	52512077	52520803		-0.012567603 PASS
XAGE2	23	52369030	52375680		-0.012567603 PASS
XAGE3	23	52862525	52868068		-0.012567603 PASS
XAGE5	23	52812204	52818301		-0.012567603 PASS
XBP1	22	28794555	28800597		-0.575930959 PASS
XCL1	1	168576473	168582077		-0.027073548 PASS
XCL2	1	168540765	168543997		-0.027073548 PASS
XCR1	3	46017024	46027742		-0.234617007 PASS
XDH	2	31334321	31414715		0.040498426 PASS
XG	23	2752048	2816500		-0.012567603 PASS
XIAP	23	123859724	123913979		-0.012567603 PASS
XIRP1	3	39183210	39192596		-0.234617007 PASS
XIRP2	2	166888487	167259753		0.039726562 PASS
XK	23	37685756	37732130		-0.012567603 PASS
XKR3	22	16783412	16821699		-0.575930959 PASS
XKR4	8	55102389	55542054		-0.201142258 PASS
XKR5	8	6808517	6835644		-0.345962679 PASS
XKR6	8	10896045	11201366		-0.345962679 PASS
XKR7	20	31968002	32003387		-0.168668583 PASS
XKR8	1	27959462	27968096		0.033763446 PASS
XKR9	8	70669365	70790371		-0.201142258 PASS
XKRX	23	100913445	100929433		-0.012567603 PASS

XPA	9	97674909	97697357	0.365190713	suppress_segment_too_large
XPC	3	14145147	14178783	0.311018244	suppress_segment_too_large
XPNPEP1	10	109864766	109923553	0.063874692	PASS
XPNPEP2	23	129738974	129769538	-0.012567603	PASS
XPNPEP3	22	40857077	40932815	-0.575930959	PASS
XPO1	2	61477849	61538626	-0.372959126	PASS
XPO4	13	20777329	20903048	-0.390786075	PASS
XPO5	6	43522330	43576075	0.048820648	PASS
XPO6	16	28097979	28211920	0.069936844	PASS
XPO7	8	21919671	22006585	-0.345962679	PASS
XPOT	12	64404350	64451127	-0.202191512	PASS
XPR1	1	180632004	180890251	-0.027073548	PASS
XRCC1	19	43543040	43580473	0.14996833	PASS
XRCC2	7	152644779	152676165	-0.411415677	PASS
XRCC3	14	103697609	103715504	-0.192197564	PASS
XRCC4	5	83077498	83353787	-0.293443042	PASS
XRCC5	2	216107464	216206303	0.025928102	PASS
XRCC6	22	41621119	41664048	-0.575930959	PASS
XRN1	3	142306607	142448062	-0.356957581	PASS
XRN2	20	21303304	21389827	-0.168668583	PASS
XRRA1	11	74807739	74949200	0.062969153	PASS
XXYLT1	3	195068279	195271167	0.326834516	PASS
XYLB	3	38346760	38421348	-0.234617007	PASS
XYLT1	16	17101769	17470881	0.069936844	PASS
XYLT2	17	50346092	50363138	0.088516311	PASS
YAE1	7	39566376	39610320	-0.69513118	PASS
YAF2	12	42157104	42238349	-0.202191512	PASS
YAP1	11	102110461	102233423	0.062969153	PASS
YARS	1	32775237	32818153	0.033763446	PASS
YARS2	12	32727490	32755902	0.362961408	suppress_segment_too_large
YBEY	21	46286337	46297751	-0.064425456	suppress_segment_too_large
YBX1	1	42682427	42702349	0.033763446	PASS
YBX2	17	7288252	7294615	0.128406487	PASS
YBX3	12	10699089	10723312	0.362961408	suppress_segment_too_large
YDJC	22	21628089	21630064	-0.575930959	PASS
YEATS2	3	183697818	183812625	0.318278996	suppress_segment_too_large
YEATS4	12	69359703	69390796	-0.202191512	PASS
YES1	18	721588	812546	-0.052305056	suppress_segment_too_large
YIF1A	11	66284580	66289170	0.182439925	suppress_segment_too_large
YIF1B	19	38305104	38317273	0.093487731	suppress_segment_too_large
YIPF1	1	53851719	53889834	0.033763446	PASS
YIPF2	19	10922185	10928681	0.093487731	suppress_segment_too_large
YIPF3	6	43511827	43516990	0.048820648	PASS
YIPF4	2	32277910	32316594	0.040498426	PASS
YIPF5	5	144158159	144170714	-0.293443042	PASS
YIPF6	23	68498323	68537285	-0.012567603	PASS

YIPF7	4	44622065	44678556	-0.011382299	PASS
YJEFN3	19	19528861	19537581	0.093487731	suppress_segment_too_large
YJU2	19	4247079	4269090	0.079632875	PASS
YKT6	7	44200968	44214294	-0.69513118	PASS
YLPM1	14	74763366	74859435	-0.192197564	PASS
YME1L1	10	27110112	27155266	0.039811807	PASS
YOD1	1	207043849	207052980	-0.256698369	PASS
YPEL1	22	21697544	21735834	-0.575930959	PASS
YPEL2	17	59331689	59401729	0.088516311	PASS
YPEL3	16	30092314	30096915	0.069936844	PASS
YPEL4	11	57645087	57649944	-0.129266491	PASS
YPEL5	2	30146941	30160533	0.040498426	PASS
YRDC	1	37802944	37808185	0.033763446	PASS
YTHDC1	4	68310387	68350089	-0.011382299	PASS
YTHDC2	5	113513683	113595285	-0.293443042	PASS
YTHDF1	20	63195429	63216234	-0.168668583	PASS
YTHDF2	1	28736621	28769775	0.033763446	PASS
YTHDF3	8	63168553	63212786	-0.201142258	PASS
YWHAB	20	44885676	44908532	-0.168668583	PASS
YWHAE	17	1344272	1400378	0.128406487	PASS
YWHAG	7	76326794	76359031	-0.262778904	PASS
YWHAH	22	31944461	31957603	-0.575930959	PASS
YWHAQ	2	9583972	9631014	0.040498426	PASS
YWHAZ	8	100916525	100953388	0.279445683	PASS
YY1	14	100238298	100282792	-0.192197564	PASS
YY1AP1	1	155659443	155689000	0.103015788	PASS
YY2	23	21855987	21858727	-0.012567603	PASS
Z82190.2	22	31754899	31956243	-0.575930959	PASS
Z83844.1	22	37686414	37726503	-0.575930959	PASS
Z83844.3	22	37639693	37666932	-0.575930959	PASS
Z84488.2	6	116399395	116463696	-0.368590549	PASS
Z84492.1	3	50350892	50367923	-0.234617007	PASS
Z98752.3	20	43540171	43569498	-0.168668583	PASS
ZACN	17	76071961	76083666	0.088516311	PASS
ZADH2	18	75195108	75209348	-0.376397843	PASS
ZAN	7	100733626	100797797	-0.262778904	PASS
ZAP70	2	97713560	97739862	0.048238121	PASS
ZAR1	4	48490252	48494389	-0.011382299	PASS
ZAR1L	13	32303700	32315344	-0.390786075	PASS
ZBBX	3	167240287	167381346	0.318278996	suppress_segment_too_large
ZBED1	23	2486414	2500967	-0.012567603	PASS
ZBED2	3	111592900	111595443	0.313454204	suppress_segment_too_large
ZBED3	5	77072072	77087323	-0.293443042	PASS
ZBED4	22	49853842	49890078	-0.808211634	PASS
ZBED5	11	10812074	10858796	-0.405444717	PASS
ZBED6	1	203795714	203854999	-0.256698369	PASS

ZBED6CL	7	150329789	150332721	-0.388946609	PASS
ZBED8	5	160393148	160400097	-0.150979337	PASS
ZBED9	6	28570535	28616212	0.048820648	PASS
ZBP1	20	57603846	57620576	-0.168668583	PASS
ZBTB1	14	64503712	64533690	0.060212819	PASS
ZBTB10	8	80485619	80526265	0.279445683	PASS
ZBTB11	3	101648889	101677495	0.313454204	suppress_segment_too_large
ZBTB12	6	31899607	31901992	0.048820648	PASS
ZBTB14	18	5289019	5297053	-0.052305056	suppress_segment_too_large
ZBTB16	11	114059593	114250676	-0.356188624	PASS
ZBTB17	1	15941869	15976132	0.033763446	PASS
ZBTB18	1	244048939	244057476	-0.368111568	PASS
ZBTB2	6	151364117	151391548	-0.035448111	PASS
ZBTB20	3	114314501	115147271	0.313454204	suppress_segment_too_large
ZBTB21	21	41986831	42010387	-0.064425456	suppress_segment_too_large
ZBTB22	6	33314406	33317942	0.048820648	PASS
ZBTB24	6	109462594	109483237	-0.368590549	PASS
ZBTB25	14	64449106	64505213	0.060212819	PASS
ZBTB26	9	122915566	122931500	0.365190713	suppress_segment_too_large
ZBTB3	11	62748319	62754188	0.182439925	suppress_segment_too_large
ZBTB32	19	35704527	35717038	0.093487731	suppress_segment_too_large
ZBTB33	23	120250752	120258398	-0.012567603	PASS
ZBTB34	9	126860665	126885878	0.365190713	suppress_segment_too_large
ZBTB37	1	173868082	173903549	-0.027073548	PASS
ZBTB38	3	141324213	141449792	-0.356957581	PASS
ZBTB39	12	56998834	57006446	-0.202191512	PASS
ZBTB4	17	7459366	7484263	0.128406487	PASS
ZBTB40	1	22428838	22531157	0.033763446	PASS
ZBTB41	1	197153680	197200542	-0.027073548	PASS
ZBTB42	14	104800596	104804712	-0.192197564	PASS
ZBTB43	9	126805006	126838210	0.365190713	suppress_segment_too_large
ZBTB44	11	130226677	130314908	-0.419612865	PASS
ZBTB45	19	58513530	58538911	0.063781462	PASS
ZBTB46	20	63743666	63831244	-0.168668583	PASS
ZBTB47	3	42653684	42665854	-0.234617007	PASS
ZBTB48	1	6580001	6589280	0.373964873	suppress_segment_too_large
ZBTB49	4	4290251	4321786	0.279089364	PASS
ZBTB5	9	37438114	37465399	-0.310444659	PASS
ZBTB6	9	122908056	122913330	0.365190713	suppress_segment_too_large
ZBTB7A	19	4044364	4066945	0.079632875	PASS
ZBTB7B	1	155002630	155018522	0.103015788	PASS
ZBTB7C	18	48026673	48410752	-0.376397843	PASS
ZBTB8A	1	32539427	32605939	0.033763446	PASS
ZBTB8B	1	32465069	32496686	0.033763446	PASS
ZBTB8OS	1	32600172	32650903	0.033763446	PASS
ZBTB9	6	33453970	33457548	0.048820648	PASS

ZC2HC1A	8	78666047	78719765	0.279445683	PASS
ZC2HC1B	6	143864436	143938356	-0.035448111	PASS
ZC2HC1C	14	75064170	75079987	-0.192197564	PASS
ZC3H10	12	56118159	56127514	-0.202191512	PASS
ZC3H11A	1	203795654	203854999	-0.256698369	PASS
ZC3H12A	1	37474552	37484379	0.033763446	PASS
ZC3H12B	23	65366638	65507887	-0.012567603	PASS
ZC3H12C	11	110093361	110171841	-0.356188624	PASS
ZC3H12D	6	149446795	149485061	-0.035448111	PASS
ZC3H13	13	45954465	46052759	-0.390786075	PASS
ZC3H14	14	88562909	88627596	-0.192197564	PASS
ZC3H15	2	186486156	186509363	-0.169541924	PASS
ZC3H18	16	88570381	88631966	-0.371357732	PASS
ZC3H3	8	143437655	143541453	0.307788765	PASS
ZC3H4	19	47064187	47113752	0.14996833	PASS
ZC3H6	2	112275594	112340063	-0.010901494	PASS
ZC3H7A	16	11750586	11797267	0.069936844	PASS
ZC3H7B	22	41301522	41360147	-0.575930959	PASS
ZC3H8	2	112211525	112255136	-0.010901494	PASS
ZC3HAV1	7	139043520	139109719	-0.388946609	PASS
ZC3HAV1	7	139025706	139036029	-0.388946609	PASS
ZC3HC1	7	130018286	130051451	-0.388946609	PASS
ZC4H2	23	64915802	65034713	-0.012567603	PASS
ZCCHC10	5	132996985	133026604	-0.293443042	PASS
ZCCHC12	23	118823790	118826968	-0.012567603	PASS
ZCCHC13	23	74304190	74305031	-0.012567603	PASS
ZCCHC14	16	87406246	87492045	-0.371357732	PASS
ZCCHC17	1	31296982	31364953	0.033763446	PASS
ZCCHC18	23	104112131	104115846	-0.012567603	PASS
ZCCHC2	18	62523007	62587709	-0.376397843	PASS
ZCCHC24	10	79382325	79445627	0.072405807	PASS
ZCCHC3	20	296968	300321	-0.168668583	PASS
ZCCHC4	4	25312785	25370383	0.279089364	PASS
ZCCHC7	9	37120539	37358149	-0.310444659	PASS
ZCCHC8	12	122471600	122501073	0.202141228	PASS
ZCCHC9	5	81301590	81313297	-0.293443042	PASS
ZCRB1	12	42312078	42326118	-0.202191512	PASS
ZCWPW1	7	100400826	100428992	-0.262778904	PASS
ZCWPW2	3	28349146	28538122	0.311018244	suppress_segment_too_large
ZDBF2	2	206274663	206314438	0.025928102	PASS
ZDHHC1	16	67394152	67416833	-0.371357732	PASS
ZDHHC11	5	795606	850986	0.055007813	PASS
ZDHHC11	5	710360	766952	0.055007813	PASS
ZDHHC12	9	128720869	128724127	0.365190713	suppress_segment_too_large
ZDHHC13	11	19117099	19176422	-0.405444717	PASS
ZDHHC14	6	157381133	157678146	-0.035448111	PASS

ZDHHC15	23	75368427	75523502	-0.012567603	PASS
ZDHHC16	10	97446131	97457370	-0.367947924	PASS
ZDHHC17	12	76763588	76853696	0.202141228	PASS
ZDHHC18	1	26826710	26857602	0.033763446	PASS
ZDHHC19	3	196197449	196211437	0.326834516	PASS
ZDHHC2	8	17156029	17224799	-0.345962679	PASS
ZDHHC20	13	21372573	21459370	-0.390786075	PASS
ZDHHC21	9	14611071	14693471	-0.310444659	PASS
ZDHHC22	14	77131270	77142734	-0.192197564	PASS
ZDHHC23	3	113947901	113965401	0.313454204	suppress_segment_too_large
ZDHHC24	11	66520637	66546238	0.182439925	suppress_segment_too_large
ZDHHC3	3	44915257	44976185	-0.234617007	PASS
ZDHHC4	7	6577434	6589374	-0.69513118	PASS
ZDHHC5	11	57667747	57701187	-0.129266491	PASS
ZDHHC6	10	112424428	112446917	0.063874692	PASS
ZDHHC7	16	84974181	85011535	-0.371357732	PASS
ZDHHC8	22	20129456	20148007	-0.575930959	PASS
ZDHHC9	23	129803288	129843909	-0.012567603	PASS
ZEB1	10	31318495	31529814	0.039811807	PASS
ZEB2	2	144364364	144524583	-0.475215623	PASS
ZER1	9	128729786	128772414	0.365190713	suppress_segment_too_large
ZFAND1	8	81701334	81732903	0.279445683	PASS
ZFAND2A	7	1152071	1160759	-0.69513118	PASS
ZFAND2B	2	219195237	219209651	0.025928102	PASS
ZFAND3	6	37819499	38154624	0.048820648	PASS
ZFAND4	10	45615501	45672780	-0.404852538	PASS
ZFAND5	9	72351425	72365235	0.365190713	suppress_segment_too_large
ZFAND6	15	80059568	80138393	-0.697600509	PASS
ZFAT	8	134477788	134713049	0.113969953	PASS
ZFC3H1	12	71609472	71667725	-0.202191512	PASS
ZFHX2	14	23520855	23556192	0.060212819	PASS
ZFHX3	16	72782885	73891871	-0.371357732	PASS
ZFHX4	8	76681219	76867285	-0.201142258	PASS
ZFP1	16	75148492	75172236	-0.371357732	PASS
ZFP14	19	36334453	36379199	0.093487731	suppress_segment_too_large
ZFP2	5	178895894	178933212	-0.150979337	PASS
ZFP28	19	56538948	56556810	-0.180829161	PASS
ZFP3	17	5078248	5096374	0.128406487	PASS
ZFP30	19	37613749	37692337	0.093487731	suppress_segment_too_large
ZFP36	19	39406813	39409412	0.093487731	suppress_segment_too_large
ZFP36L1	14	68787660	68796253	0.060212819	PASS
ZFP36L2	2	43222402	43226609	0.040498426	PASS
ZFP37	9	113038380	113056759	0.365190713	suppress_segment_too_large
ZFP41	8	143246821	143262705	0.307788765	PASS
ZFP42	4	187995771	188005050	-0.462744716	PASS
ZFP57	6	29672392	29681110	0.048820648	PASS

ZFP62	5	180847611	180861285	-0.150979337	PASS
ZFP64	20	52051663	52204308	-0.168668583	PASS
ZFP69	1	40477215	40496343	0.033763446	PASS
ZFP69B	1	40450102	40463718	0.033763446	PASS
ZFP82	19	36383120	36418656	0.093487731	suppress_segment_too_large
ZFP90	16	68530090	68576072	-0.371357732	PASS
ZFP91	11	58579111	58621042	-0.129266491	PASS
ZFP91-CN'	11	58579172	58624639	-0.129266491	PASS
ZFP92	23	153418322	153426481	-0.012567603	PASS
ZFPL1	11	65084210	65088400	0.182439925	suppress_segment_too_large
ZFPM1	16	88453317	88537016	-0.371357732	PASS
ZFPM2	8	104590733	105804532	0.279445683	PASS
ZFR	5	32354350	32444761	0.055007813	PASS
ZFR2	19	3804024	3869032	0.079632875	PASS
ZFX	23	24149173	24216255	-0.012567603	PASS
ZFYVE1	14	72969451	73027212	0.060212819	PASS
ZFYVE16	5	80408013	80479350	-0.293443042	PASS
ZFYVE19	15	40807086	40815084	-0.396255771	PASS
ZFYVE21	14	103715730	103733668	-0.192197564	PASS
ZFYVE26	14	67727374	67816590	0.060212819	PASS
ZFYVE27	10	97737121	97760907	-0.367947924	PASS
ZFYVE28	4	2269582	2418663	0.279089364	PASS
ZFYVE9	1	52142094	52346686	0.033763446	PASS
ZG16	16	29778240	29782973	0.069936844	PASS
ZG16B	16	2830169	2839585	0.069936844	PASS
ZGLP1	19	10304803	10309880	0.093487731	suppress_segment_too_large
ZGPAT	20	63707465	63736142	-0.168668583	PASS
ZGRF1	4	112539333	112636995	-0.011382299	PASS
ZHX1	8	123248451	123275541	0.279445683	PASS
ZHX1-C8o	8	123226189	123274284	0.279445683	PASS
ZHX2	8	122781394	122974512	0.279445683	PASS
ZHX3	20	41178448	41317672	-0.168668583	PASS
ZIC1	3	147393422	147510293	0.318278996	suppress_segment_too_large
ZIC2	13	99981772	99986773	-0.390786075	PASS
ZIC3	23	137566142	137577691	-0.012567603	PASS
ZIC4	3	147386046	147406860	0.318278996	suppress_segment_too_large
ZIC5	13	99962964	99971909	-0.390786075	PASS
ZIK1	19	57578456	57593777	-0.180829161	PASS
ZIM2	19	56774547	56840729	-0.180829161	PASS
ZIM3	19	57134096	57145202	-0.180829161	PASS
ZKSCAN1	7	100015572	100041689	-0.262778904	PASS
ZKSCAN2	16	25236001	25257931	0.069936844	PASS
ZKSCAN3	6	28349914	28369177	0.048820648	PASS
ZKSCAN4	6	28244623	28252224	0.048820648	PASS
ZKSCAN5	7	99504651	99534700	-0.262778904	PASS
ZKSCAN7	3	44555193	44594173	-0.234617007	PASS

ZKSCAN8	6	28141910	28159472	0.048820648	PASS
ZMAT1	23	101882288	101932031	-0.012567603	PASS
ZMAT2	5	140698680	140706676	-0.293443042	PASS
ZMAT3	3	179017223	179072279	0.318278996	suppress_segment_too_large
ZMAT4	8	40530590	40897833	-0.201142258	PASS
ZMAT5	22	29730956	29767011	-0.575930959	PASS
ZMIZ1	10	79069035	79316528	0.072405807	PASS
ZMIZ2	7	44748581	44769881	-0.69513118	PASS
ZMPSTE24	1	40258107	40294184	0.033763446	PASS
ZMYM1	1	35032172	35115859	0.033763446	PASS
ZMYM2	13	19958670	20091829	-0.390786075	PASS
ZMYM3	23	71239624	71255146	-0.012567603	PASS
ZMYM4	1	35268967	35422058	0.033763446	PASS
ZMYM5	13	19823482	19863636	-0.390786075	PASS
ZMYM6	1	34986165	35031968	0.033763446	PASS
ZMYND10	3	50341110	50346852	-0.234617007	PASS
ZMYND11	10	134465	254637	0.039811807	PASS
ZMYND12	1	42430329	42456267	0.033763446	PASS
ZMYND15	17	4740015	4746119	0.128406487	PASS
ZMYND19	9	137582079	137590490	0.365190713	suppress_segment_too_large
ZMYND8	20	47209214	47356889	-0.168668583	PASS
ZNF10	12	133130575	133159465	0.270848837	PASS
ZNF100	19	21722766	21767628	0.093487731	suppress_segment_too_large
ZNF101	19	19668796	19683509	0.093487731	suppress_segment_too_large
ZNF106	15	42412823	42491141	-0.396255771	PASS
ZNF107	7	64666083	64711582	-0.750061359	PASS
ZNF112	19	44326555	44367217	0.14996833	PASS
ZNF114	19	48270102	48287608	0.14996833	PASS
ZNF117	7	64971776	65006684	-0.750061359	PASS
ZNF12	7	6688433	6706923	-0.69513118	PASS
ZNF121	19	9560353	9584533	0.093487731	suppress_segment_too_large
ZNF124	1	247121975	247172016	-0.368111568	PASS
ZNF131	5	43065176	43192021	-0.293443042	PASS
ZNF132	19	58432814	58440222	0.063781462	PASS
ZNF133	20	18288283	18316996	-0.168668583	PASS
ZNF134	19	57614233	57624723	-0.180829161	PASS
ZNF135	19	58059239	58086310	0.063781462	PASS
ZNF136	19	12163064	12189881	0.093487731	suppress_segment_too_large
ZNF138	7	64794388	64833681	-0.750061359	PASS
ZNF14	19	19710471	19733097	0.093487731	suppress_segment_too_large
ZNF140	12	133079838	133107544	0.270848837	PASS
ZNF141	4	337814	384864	0.279089364	PASS
ZNF142	2	218637916	218659655	0.025928102	PASS
ZNF143	11	9460319	9528524	-0.405444717	PASS
ZNF146	19	36214602	36238774	0.093487731	suppress_segment_too_large
ZNF148	3	125225561	125375354	0.313454204	suppress_segment_too_large

ZNF154	19	57697367	57709194	-0.180829161	PASS
ZNF155	19	43967862	43998325	0.14996833	PASS
ZNF157	23	47370583	47414305	-0.012567603	PASS
ZNF16	8	144930358	144950888	0.307788765	PASS
ZNF160	19	53066606	53103436	0.206912349	PASS
ZNF165	6	28080975	28089563	0.048820648	PASS
ZNF169	9	94259311	94301454	0.365190713	suppress_segment_too_large
ZNF17	19	57411163	57421939	-0.180829161	PASS
ZNF174	16	3401235	3409370	0.069936844	PASS
ZNF175	19	51571298	51592508	-0.016761391	PASS
ZNF177	19	9363020	9382617	0.093487731	suppress_segment_too_large
ZNF18	17	11977439	11997510	-0.139031004	PASS
ZNF180	19	44474428	44500524	0.14996833	PASS
ZNF181	19	34734155	34745378	0.093487731	suppress_segment_too_large
ZNF182	23	47974851	48003978	-0.012567603	PASS
ZNF184	6	27450743	27473118	0.048820648	PASS
ZNF185	23	152914442	152973480	-0.012567603	PASS
ZNF189	9	101398873	101410660	0.365190713	suppress_segment_too_large
ZNF19	16	71464555	71565089	-0.371357732	PASS
ZNF195	11	3339261	3379222	-0.333835828	PASS
ZNF197	3	44584888	44648471	-0.234617007	PASS
ZNF2	2	95165432	95184317	0.048238121	PASS
ZNF20	19	12092843	12140407	0.093487731	suppress_segment_too_large
ZNF200	16	3222325	3236221	0.069936844	PASS
ZNF202	11	123724177	123741675	-0.949752196	PASS
ZNF205	16	3112560	3120517	0.069936844	PASS
ZNF207	17	32350117	32381886	0.060555011	PASS
ZNF208	19	21932958	22010949	0.093487731	suppress_segment_too_large
ZNF211	19	57630395	57642779	-0.180829161	PASS
ZNF212	7	149239651	149255609	-0.388946609	PASS
ZNF213	16	3129777	3142805	0.069936844	PASS
ZNF214	11	6999318	7020368	-0.405444717	PASS
ZNF215	11	6926404	7001004	-0.405444717	PASS
ZNF217	20	53567065	53609907	-0.168668583	PASS
ZNF219	14	21090046	21104722	0.060212819	PASS
ZNF22	10	45000475	45005326	-0.404852538	PASS
ZNF221	19	43951223	43967709	0.14996833	PASS
ZNF222	19	44025342	44033112	0.14996833	PASS
ZNF223	19	44051367	44067991	0.14996833	PASS
ZNF224	19	44094339	44109886	0.14996833	PASS
ZNF225	19	44112181	44134816	0.14996833	PASS
ZNF226	19	44165073	44178381	0.14996833	PASS
ZNF227	19	44207547	44237268	0.14996833	PASS
ZNF229	19	44417519	44448578	0.14996833	PASS
ZNF23	16	71447597	71463095	-0.371357732	PASS
ZNF230	19	44002948	44013926	0.14996833	PASS

ZNF232	17	5105541	5123116	0.128406487	PASS
ZNF233	19	44259880	44275317	0.14996833	PASS
ZNF234	19	44141557	44160309	0.14996833	PASS
ZNF235	19	44228729	44305046	0.14996833	PASS
ZNF236	18	76822607	76970727	-0.376397843	PASS
ZNF239	10	43556344	43574618	-0.404852538	PASS
ZNF24	18	35332212	35345482	-0.376397843	PASS
ZNF248	10	37776526	37858106	0.039811807	PASS
ZNF25	10	37949572	37976633	0.039811807	PASS
ZNF250	8	144876497	144902168	0.307788765	PASS
ZNF251	8	144720907	144756417	0.307788765	PASS
ZNF253	19	19865886	19894674	0.093487731	suppress_segment_too_large
ZNF254	19	24033405	24129961	0.093487731	suppress_segment_too_large
ZNF256	19	57940833	57947675	-0.180829161	PASS
ZNF257	19	22052452	22091480	0.093487731	suppress_segment_too_large
ZNF26	12	132986365	133032952	0.270848837	PASS
ZNF260	19	36510695	36528660	0.093487731	suppress_segment_too_large
ZNF263	16	3263800	3301401	0.069936844	PASS
ZNF264	19	57191500	57222846	-0.180829161	PASS
ZNF266	19	9412461	9435578	0.093487731	suppress_segment_too_large
ZNF267	16	31873758	31917357	0.069936844	PASS
ZNF268	12	133181409	133214831	0.270848837	PASS
ZNF273	7	64870172	64930966	-0.750061359	PASS
ZNF274	19	58183029	58213562	0.063781462	PASS
ZNF275	23	153334147	153360110	-0.012567603	PASS
ZNF276	16	89720400	89740903	-0.371357732	PASS
ZNF277	7	112206588	112343096	-0.388946609	PASS
ZNF28	19	52797409	52857600	0.206912349	PASS
ZNF280A	22	22513736	22520270	-0.575930959	PASS
ZNF280B	22	22484421	22509154	-0.575930959	PASS
ZNF280C	23	130202711	130268899	-0.012567603	PASS
ZNF280D	15	56630181	56734086	-0.697600509	PASS
ZNF281	1	200404940	200410056	-0.027073548	PASS
ZNF282	7	149195485	149226248	-0.388946609	PASS
ZNF283	19	43827292	43852017	0.14996833	PASS
ZNF284	19	44072144	44089613	0.14996833	PASS
ZNF285	19	44382298	44401608	0.14996833	PASS
ZNF286A	17	15699577	15720787	-0.139031004	PASS
ZNF286B	17	18658429	18682262	0.024610076	PASS
ZNF287	17	16551387	16569206	0.024610076	PASS
ZNF292	6	87152833	87264196	-0.368590549	PASS
ZNF296	19	45071500	45076588	0.14996833	PASS
ZNF3	7	100064033	100082548	-0.262778904	PASS
ZNF30	19	34926903	34945170	0.093487731	suppress_segment_too_large
ZNF300	5	150894392	150904983	-0.293443042	PASS
ZNF302	19	34677639	34686397	0.093487731	suppress_segment_too_large

ZNF304	19	57351307	57359898	-0.180829161	PASS
ZNF311	6	28994785	29005316	0.048820648	PASS
ZNF316	7	6637322	6656432	-0.69513118	PASS
ZNF317	19	9140380	9163424	0.093487731	suppress_segment_too_large
ZNF318	6	43307134	43369478	0.048820648	PASS
ZNF319	16	57994668	58000453	-0.371357732	PASS
ZNF32	10	43643859	43648856	-0.404852538	PASS
ZNF320	19	52863790	52897693	0.206912349	PASS
ZNF322	6	26634383	26659752	0.048820648	PASS
ZNF324	19	58467045	58475436	0.063781462	PASS
ZNF324B	19	58451604	58457833	0.063781462	PASS
ZNF326	1	89995112	90035531	0.033763446	PASS
ZNF329	19	58126252	58155110	0.063781462	PASS
ZNF330	4	141220887	141234697	-0.011382299	PASS
ZNF331	19	53519527	53580269	0.206912349	PASS
ZNF333	19	14689801	14733746	0.093487731	suppress_segment_too_large
ZNF334	20	46499630	46513559	-0.168668583	PASS
ZNF335	20	45948653	45972172	-0.168668583	PASS
ZNF337	20	25674215	25696841	-0.168668583	PASS
ZNF33A	10	38010650	38065088	0.039811807	PASS
ZNF33B	10	42574185	42638568	-0.404852538	PASS
ZNF34	8	144773114	144787345	0.307788765	PASS
ZNF341	20	33731657	33792269	-0.168668583	PASS
ZNF343	20	2481817	2524702	-0.168668583	PASS
ZNF345	19	36850361	36913029	0.093487731	suppress_segment_too_large
ZNF346	5	177022696	177081189	-0.150979337	PASS
ZNF347	19	53124072	53159075	0.206912349	PASS
ZNF35	3	44648727	44660791	-0.234617007	PASS
ZNF350	19	51964343	51986856	-0.016761391	PASS
ZNF354A	5	178711592	178730702	-0.150979337	PASS
ZNF354B	5	178859953	178888122	-0.150979337	PASS
ZNF354C	5	179060415	179083537	-0.150979337	PASS
ZNF358	19	7515292	7521026	0.079632875	PASS
ZNF362	1	33256545	33300719	0.033763446	PASS
ZNF365	10	62374192	62670301	0.072405807	PASS
ZNF366	5	72442652	72507727	-0.293443042	PASS
ZNF367	9	96385941	96418329	0.365190713	suppress_segment_too_large
ZNF37A	10	38094334	38150293	0.039811807	PASS
ZNF382	19	36604817	36634113	0.093487731	suppress_segment_too_large
ZNF383	19	37217926	37248738	0.093487731	suppress_segment_too_large
ZNF384	12	6666477	6689572	-0.086128028	PASS
ZNF385A	12	54369133	54391298	-0.202191512	PASS
ZNF385B	2	179441982	179861505	0.039726562	PASS
ZNF385C	17	42025576	42098479	0.060555011	PASS
ZNF385D	3	21412222	22373321	0.311018244	suppress_segment_too_large
ZNF391	6	27374615	27403904	0.048820648	PASS

ZNF394	7	99486519	99500324	-0.262778904	PASS
ZNF395	8	28345585	28402701	-0.345962679	PASS
ZNF396	18	35366697	35377337	-0.376397843	PASS
ZNF397	18	35241030	35267133	-0.376397843	PASS
ZNF398	7	149126416	149182802	-0.388946609	PASS
ZNF404	19	43872363	43901385	0.14996833	PASS
ZNF407	18	74597870	75065671	-0.376397843	PASS
ZNF408	11	46700818	46705912	0.117376172	PASS
ZNF41	23	47445879	47482946	-0.012567603	PASS
ZNF410	14	73886617	73932511	0.060212819	PASS
ZNF414	19	8509678	8514164	0.079632875	PASS
ZNF415	19	53107879	53133077	0.206912349	PASS
ZNF416	19	57571566	57578927	-0.180829161	PASS
ZNF417	19	57900296	57916610	-0.180829161	PASS
ZNF418	19	57921884	57935393	-0.180829161	PASS
ZNF419	19	57487711	57496097	-0.180829161	PASS
ZNF420	19	37007857	37130314	0.093487731	suppress_segment_too_large
ZNF423	16	49487524	49857919	0.069936844	PASS
ZNF425	7	149102784	149126346	-0.388946609	PASS
ZNF426	19	9523224	9538645	0.093487731	suppress_segment_too_large
ZNF428	19	43607219	43619874	0.14996833	PASS
ZNF429	19	21496682	21556270	0.093487731	suppress_segment_too_large
ZNF43	19	21804949	21852125	0.093487731	suppress_segment_too_large
ZNF430	19	21020620	21060050	0.093487731	suppress_segment_too_large
ZNF431	19	21142024	21196053	0.093487731	suppress_segment_too_large
ZNF432	19	52031379	52095738	0.206912349	PASS
ZNF433	19	12014732	12035741	0.093487731	suppress_segment_too_large
ZNF436	1	23359448	23369442	0.033763446	PASS
ZNF438	10	30820207	31031937	0.039811807	PASS
ZNF439	19	11848726	11883750	0.093487731	suppress_segment_too_large
ZNF44	19	12224686	12294887	0.093487731	suppress_segment_too_large
ZNF440	19	11814284	11835201	0.093487731	suppress_segment_too_large
ZNF441	19	11767000	11784078	0.093487731	suppress_segment_too_large
ZNF442	19	12345949	12365905	0.093487731	suppress_segment_too_large
ZNF443	19	12429707	12441112	0.093487731	suppress_segment_too_large
ZNF444	19	56132599	56160893	-0.180829161	PASS
ZNF445	3	44431720	44477670	-0.234617007	PASS
ZNF446	19	58474017	58481230	0.063781462	PASS
ZNF449	23	135344796	135363152	-0.012567603	PASS
ZNF45	19	43912629	43935278	0.14996833	PASS
ZNF451	6	57086844	57170307	0.048820648	PASS
ZNF454	5	178941191	178966433	-0.150979337	PASS
ZNF460	19	57280051	57293569	-0.180829161	PASS
ZNF461	19	36637192	36666853	0.093487731	suppress_segment_too_large
ZNF462	9	106863097	107013634	0.365190713	suppress_segment_too_large
ZNF467	7	149764182	149773479	-0.388946609	PASS

ZNF468	19	52838008	52890375	0.206912349	PASS
ZNF469	16	88427471	88440757	-0.371357732	PASS
ZNF470	19	56567511	56588911	-0.180829161	PASS
ZNF471	19	56507843	56530221	-0.180829161	PASS
ZNF473	19	50025714	50053414	-0.016761391	PASS
ZNF474	5	122129513	122153571	-0.293443042	PASS
ZNF479	7	57119614	57139864	-0.750061359	PASS
ZNF48	16	30378106	30400108	0.069936844	PASS
ZNF480	19	52297177	52325922	0.206912349	PASS
ZNF483	9	111525159	111577844	0.365190713	suppress_segment_too_large
ZNF484	9	92845031	92878038	0.365190713	suppress_segment_too_large
ZNF485	10	43606407	43617903	-0.404852538	PASS
ZNF486	19	20167228	20200490	0.093487731	suppress_segment_too_large
ZNF487	10	43436841	43483179	-0.404852538	PASS
ZNF488	10	47365496	47384273	-0.404852538	PASS
ZNF490	19	12577961	12640098	0.093487731	suppress_segment_too_large
ZNF491	19	11797667	11809622	0.093487731	suppress_segment_too_large
ZNF492	19	22634324	22667670	0.093487731	suppress_segment_too_large
ZNF493	19	21397119	21427573	0.093487731	suppress_segment_too_large
ZNF496	1	247297412	247331846	-0.368111568	PASS
ZNF497	19	58354357	58362848	0.063781462	PASS
ZNF500	16	4748239	4767624	0.069936844	PASS
ZNF501	3	44729596	44737083	-0.234617007	PASS
ZNF502	3	44712643	44723831	-0.234617007	PASS
ZNF503	10	75397830	75401906	0.072405807	PASS
ZNF506	19	19785839	19821751	0.093487731	suppress_segment_too_large
ZNF507	19	32345594	32387667	0.093487731	suppress_segment_too_large
ZNF510	9	96755865	96778129	0.365190713	suppress_segment_too_large
ZNF511	10	133308475	133313162	0.063874692	PASS
ZNF511-PI	10	133309410	133352529	0.063874692	PASS
ZNF512	2	27582969	27623215	0.040498426	PASS
ZNF512B	20	63956702	63969865	-0.168668583	PASS
ZNF513	2	27377231	27380790	0.040498426	PASS
ZNF514	2	95147330	95165413	0.048238121	PASS
ZNF516	18	76358190	76495190	-0.376397843	PASS
ZNF517	8	144798876	144811169	0.307788765	PASS
ZNF518A	10	96129715	96205288	1.054876852	PASS
ZNF518B	4	10439874	10457410	0.279089364	PASS
ZNF519	18	14057457	14132490	-0.052305056	suppress_segment_too_large
ZNF521	18	25061926	25352190	-0.376397843	PASS
ZNF524	19	55600022	55603138	0.121336758	PASS
ZNF525	19	53365693	53392217	0.206912349	PASS
ZNF526	19	42220271	42228201	0.093487731	suppress_segment_too_large
ZNF527	19	37371061	37393066	0.093487731	suppress_segment_too_large
ZNF528	19	52397849	52418412	0.206912349	PASS
ZNF529	19	36534774	36605276	0.093487731	suppress_segment_too_large

ZNF530	19	57599885	57612722	-0.180829161	PASS
ZNF532	18	58862600	58986480	-0.376397843	PASS
ZNF534	19	52429187	52452315	0.206912349	PASS
ZNF536	19	30228290	30713538	0.093487731	suppress_segment_too_large
ZNF540	19	37551406	37614097	0.093487731	suppress_segment_too_large
ZNF541	19	47520685	47555856	0.14996833	PASS
ZNF543	19	57320509	57330776	-0.180829161	PASS
ZNF544	19	58228594	58277495	0.063781462	PASS
ZNF546	19	39984134	40021041	0.093487731	suppress_segment_too_large
ZNF547	19	57363477	57379565	-0.180829161	PASS
ZNF548	19	57389850	57402992	-0.180829161	PASS
ZNF549	19	57527325	57557542	-0.180829161	PASS
ZNF550	19	57535257	57559863	-0.180829161	PASS
ZNF551	19	57681969	57717301	-0.180829161	PASS
ZNF552	19	57803841	57814913	-0.180829161	PASS
ZNF554	19	2819874	2835773	0.079632875	PASS
ZNF555	19	2841435	2860484	0.079632875	PASS
ZNF556	19	2867335	2883445	0.079632875	PASS
ZNF557	19	7069444	7087968	0.079632875	PASS
ZNF558	19	8806170	8832328	0.079632875	PASS
ZNF559	19	9323772	9351162	0.093487731	suppress_segment_too_large
ZNF559-ZI	19	9324174	9382617	0.093487731	suppress_segment_too_large
ZNF560	19	9466507	9498607	0.093487731	suppress_segment_too_large
ZNF561	19	9604680	9621399	0.093487731	suppress_segment_too_large
ZNF562	19	9641808	9675086	0.093487731	suppress_segment_too_large
ZNF563	19	12317477	12333720	0.093487731	suppress_segment_too_large
ZNF564	19	12525370	12551542	0.093487731	suppress_segment_too_large
ZNF565	19	36182060	36246257	0.093487731	suppress_segment_too_large
ZNF566	19	36445119	36489902	0.093487731	suppress_segment_too_large
ZNF567	19	36687612	36727701	0.093487731	suppress_segment_too_large
ZNF568	19	36916329	36998700	0.093487731	suppress_segment_too_large
ZNF569	19	37411155	37469275	0.093487731	suppress_segment_too_large
ZNF57	19	2900898	2918476	0.079632875	PASS
ZNF570	19	37467585	37488652	0.093487731	suppress_segment_too_large
ZNF571	19	37554782	37594790	0.093487731	suppress_segment_too_large
ZNF572	8	124973298	124979389	0.279445683	PASS
ZNF573	19	37735833	37817300	0.093487731	suppress_segment_too_large
ZNF574	19	42068477	42081549	0.093487731	suppress_segment_too_large
ZNF575	19	43525497	43536130	0.14996833	PASS
ZNF576	19	43596392	43601157	0.14996833	PASS
ZNF577	19	51804816	51890950	-0.016761391	PASS
ZNF578	19	52453576	52512154	0.206912349	PASS
ZNF579	19	55576770	55580845	0.121336758	PASS
ZNF580	19	55635016	55643469	0.121336758	PASS
ZNF581	19	55635459	55645622	0.121336758	PASS
ZNF582	19	56375846	56393545	-0.180829161	PASS

ZNF583	19	56397966	56436035	-0.180829161	PASS
ZNF584	19	58401504	58418327	0.063781462	PASS
ZNF585A	19	37106734	37172741	0.093487731	suppress_segment_too_large
ZNF585B	19	37181579	37218153	0.093487731	suppress_segment_too_large
ZNF586	19	57769655	57819939	-0.180829161	PASS
ZNF587	19	57849857	57865112	-0.180829161	PASS
ZNF587B	19	57819741	57846238	-0.180829161	PASS
ZNF589	3	48241100	48299253	-0.234617007	PASS
ZNF592	15	84748635	84806432	-0.697600509	PASS
ZNF593	1	26169871	26170873	0.033763446	PASS
ZNF594	17	5179536	5191883	0.128406487	PASS
ZNF595	4	53285	88211	0.279089364	PASS
ZNF596	8	232137	264703	-0.106598973	PASS
ZNF597	16	3432422	3443553	0.069936844	PASS
ZNF598	16	1997654	2009823	0.069936844	PASS
ZNF599	19	34758076	34773229	0.093487731	suppress_segment_too_large
ZNF600	19	52749942	52786807	0.206912349	PASS
ZNF605	12	132918308	132956306	0.270848837	PASS
ZNF606	19	57977053	58003349	0.063781462	PASS
ZNF607	19	37696363	37719790	0.093487731	suppress_segment_too_large
ZNF608	5	124636913	124748807	-0.293443042	PASS
ZNF609	15	64460742	64686068	-0.697600509	PASS
ZNF610	19	52336245	52367778	0.206912349	PASS
ZNF611	19	52702813	52735073	0.206912349	PASS
ZNF613	19	51927147	51948759	-0.016761391	PASS
ZNF614	19	52012765	52030240	0.206912349	PASS
ZNF615	19	51991332	52008230	0.206912349	PASS
ZNF616	19	52113091	52139922	0.206912349	PASS
ZNF618	9	113876282	114056591	0.365190713	suppress_segment_too_large
ZNF619	3	40477113	40490236	-0.234617007	PASS
ZNF620	3	40477131	40518736	-0.234617007	PASS
ZNF621	3	40524878	40574685	-0.234617007	PASS
ZNF622	5	16451519	16465792	0.055007813	PASS
ZNF623	8	143636013	143656418	0.307788765	PASS
ZNF624	17	16620737	16653856	0.024610076	PASS
ZNF625	19	12142090	12156729	0.093487731	suppress_segment_too_large
ZNF625-ZI	19	12132117	12156731	0.093487731	suppress_segment_too_large
ZNF626	19	20620061	20661596	0.093487731	suppress_segment_too_large
ZNF627	19	11559374	11619135	0.093487731	suppress_segment_too_large
ZNF628	19	55476332	55484487	0.121336758	PASS
ZNF629	16	30778449	30787202	0.069936844	PASS
ZNF630	23	47983356	48071658	-0.012567603	PASS
ZNF638	2	71276561	71435069	-0.372959126	PASS
ZNF639	3	179322991	179338583	0.318278996	suppress_segment_too_large
ZNF641	12	48337180	48351414	-0.202191512	PASS
ZNF644	1	90915298	91022272	0.033763446	PASS

ZNF646	16	31074422	31084196	0.069936844	PASS
ZNF648	1	182054570	182061712	-0.027073548	PASS
ZNF649	19	51889224	51905040	-0.016761391	PASS
ZNF652	17	49289206	49362473	0.088516311	PASS
ZNF653	19	11483427	11505923	0.093487731	suppress_segment_too_large
ZNF654	3	88059274	88144665	0.313454204	suppress_segment_too_large
ZNF655	7	99558406	99576453	-0.262778904	PASS
ZNF658	9	66856426	66932141	-0.310444659	PASS
ZNF66	19	20776304	20807322	0.093487731	suppress_segment_too_large
ZNF660	3	44578223	44599694	-0.234617007	PASS
ZNF662	3	42905731	42917641	-0.234617007	PASS
ZNF664	12	123971845	124015439	0.202141228	PASS
ZNF665	19	53159213	53193386	0.206912349	PASS
ZNF667	19	56439325	56478065	-0.180829161	PASS
ZNF668	16	31060843	31074320	0.069936844	PASS
ZNF669	1	247099962	247104372	-0.368111568	PASS
ZNF670	1	247036784	247078755	-0.368111568	PASS
ZNF670-ZN	1	246945547	247078811	-0.368111568	PASS
ZNF671	19	57719751	57727624	-0.180829161	PASS
ZNF672	1	248838210	248849517	-0.368111568	PASS
ZNF674	23	46497727	46545457	-0.012567603	PASS
ZNF675	19	23525631	23687220	0.093487731	suppress_segment_too_large
ZNF676	19	22179091	22215775	0.093487731	suppress_segment_too_large
ZNF677	19	53235381	53254898	0.206912349	PASS
ZNF678	1	227563543	227677443	-0.256698369	PASS
ZNF679	7	64228474	64266931	-0.750061359	PASS
ZNF680	7	64519884	64563106	-0.750061359	PASS
ZNF681	19	23739195	23758891	0.093487731	suppress_segment_too_large
ZNF682	19	19997058	20039506	0.093487731	suppress_segment_too_large
ZNF683	1	26361634	26374522	0.033763446	PASS
ZNF684	1	40531561	40548169	0.033763446	PASS
ZNF687	1	151281618	151292180	0.103015788	PASS
ZNF688	16	30569346	30572734	0.069936844	PASS
ZNF689	16	30602558	30624012	0.069936844	PASS
ZNF69	19	11887784	11914329	0.093487731	suppress_segment_too_large
ZNF691	1	42846573	42852477	0.033763446	PASS
ZNF692	1	248850006	248859144	-0.368111568	PASS
ZNF695	1	246945547	247008093	-0.368111568	PASS
ZNF696	8	143289676	143298061	0.307788765	PASS
ZNF697	1	119619422	119647773	-0.157385092	PASS
ZNF699	19	9294275	9309838	0.093487731	suppress_segment_too_large
ZNF7	8	144827464	144847509	0.307788765	PASS
ZNF70	22	23738678	23751092	-0.575930959	PASS
ZNF700	19	11925068	11950773	0.093487731	suppress_segment_too_large
ZNF701	19	52555457	52587174	0.206912349	PASS
ZNF703	8	37695751	37700021	-0.201142258	PASS

ZNF704	8	80628451	80874781	0.279445683	PASS
ZNF705A	12	8157034	8188537	-0.086128028	PASS
ZNF705D	8	12104389	12115516	-0.345962679	PASS
ZNF705E	11	71814045	71821548	0.062969153	PASS
ZNF705G	8	7355517	7385558	-0.345962679	PASS
ZNF706	8	101177878	101206193	0.279445683	PASS
ZNF707	8	143684452	143713898	0.307788765	PASS
ZNF708	19	21291160	21329425	0.093487731	suppress_segment_too_large
ZNF709	19	12461184	12513854	0.093487731	suppress_segment_too_large
ZNF71	19	56595264	56626481	-0.180829161	PASS
ZNF710	15	90001392	90082206	-0.697600509	PASS
ZNF711	23	85244032	85273362	-0.012567603	PASS
ZNF713	7	55887475	55942530	-0.750061359	PASS
ZNF714	19	21082159	21125270	0.093487731	suppress_segment_too_large
ZNF716	7	57450177	57473546	-0.750061359	PASS
ZNF717	3	75678660	75785583	-0.234617007	PASS
ZNF718	4	124480	202303	0.279089364	PASS
ZNF720	16	31713229	31794869	0.069936844	PASS
ZNF721	4	425815	499156	0.279089364	PASS
ZNF723	19	22832321	22858667	0.093487731	suppress_segment_too_large
ZNF724	19	23221599	23250390	0.093487731	suppress_segment_too_large
ZNF726	19	23914876	23945159	0.093487731	suppress_segment_too_large
ZNF727	7	64045443	64078549	-0.750061359	PASS
ZNF728	19	22975468	23003176	0.093487731	suppress_segment_too_large
ZNF729	19	22286408	22317176	0.093487731	suppress_segment_too_large
ZNF730	19	23075210	23147219	0.093487731	suppress_segment_too_large
ZNF732	4	270675	305321	0.279089364	PASS
ZNF735	7	64207090	64220508	-0.750061359	PASS
ZNF736	7	64307459	64354860	-0.750061359	PASS
ZNF737	19	20535825	20565809	0.093487731	suppress_segment_too_large
ZNF738	19	21358930	21379302	0.093487731	suppress_segment_too_large
ZNF74	22	20394115	20408461	-0.575930959	PASS
ZNF740	12	53180700	53195141	-0.202191512	PASS
ZNF746	7	149472696	149497817	-0.388946609	PASS
ZNF747	16	30530367	30535347	0.069936844	PASS
ZNF749	19	57435329	57445485	-0.180829161	PASS
ZNF750	17	82829435	82840578	0.088516311	PASS
ZNF75A	16	3305406	3318852	0.069936844	PASS
ZNF75D	23	135248920	135344087	-0.012567603	PASS
ZNF76	6	35258909	35295985	0.048820648	PASS
ZNF761	19	53431993	53458261	0.206912349	PASS
ZNF763	19	11965037	11980381	0.093487731	suppress_segment_too_large
ZNF764	16	30553764	30558498	0.069936844	PASS
ZNF765	19	53389793	53430413	0.206912349	PASS
ZNF766	19	52269571	52296046	0.206912349	PASS
ZNF768	16	30524001	30526821	0.069936844	PASS

ZNF77	19	2933218	2944971	0.079632875	PASS
ZNF770	15	34978341	34988287	-0.396255771	PASS
ZNF771	16	30407297	30431108	0.069936844	PASS
ZNF772	19	57466663	57477570	-0.180829161	PASS
ZNF773	19	57499915	57518404	-0.180829161	PASS
ZNF774	15	90352245	90369146	-0.697600509	PASS
ZNF775	7	150379335	150398631	-0.388946609	PASS
ZNF776	19	57746796	57758159	-0.180829161	PASS
ZNF777	7	149431363	149461123	-0.388946609	PASS
ZNF778	16	89217703	89237071	-0.371357732	PASS
ZNF780A	19	40069152	40090938	0.093487731	suppress_segment_too_large
ZNF780B	19	40028260	40056209	0.093487731	suppress_segment_too_large
ZNF781	19	37667751	37692322	0.093487731	suppress_segment_too_large
ZNF782	9	96816472	96875623	0.365190713	suppress_segment_too_large
ZNF783	7	149262171	149297302	-0.388946609	PASS
ZNF784	19	55620742	55624601	0.121336758	PASS
ZNF785	16	30573740	30585771	0.069936844	PASS
ZNF786	7	149069643	149090782	-0.388946609	PASS
ZNF787	19	56087366	56121280	-0.180829161	PASS
ZNF789	7	99472841	99503650	-0.262778904	PASS
ZNF79	9	127424374	127445372	0.365190713	suppress_segment_too_large
ZNF790	19	36817428	36850787	0.093487731	suppress_segment_too_large
ZNF791	19	12610918	12633840	0.093487731	suppress_segment_too_large
ZNF792	19	34956354	34964049	0.093487731	suppress_segment_too_large
ZNF793	19	37506939	37548762	0.093487731	suppress_segment_too_large
ZNF799	19	12390016	12401271	0.093487731	suppress_segment_too_large
ZNF8	19	58278951	58302805	0.063781462	PASS
ZNF80	3	114234631	114237578	0.313454204	suppress_segment_too_large
ZNF800	7	127346790	127431924	-0.388946609	PASS
ZNF804A	2	184598366	184939492	-0.169541924	PASS
ZNF804B	7	88759368	89337057	-0.262778904	PASS
ZNF805	19	57240685	57255135	-0.180829161	PASS
ZNF808	19	52527652	52564464	0.206912349	PASS
ZNF81	23	47836902	48002561	-0.012567603	PASS
ZNF813	19	53467735	53496255	0.206912349	PASS
ZNF814	19	57848731	57889074	-0.180829161	PASS
ZNF816	19	52949379	52962911	0.206912349	PASS
ZNF816-ZI	19	52928475	52962823	0.206912349	PASS
ZNF821	16	71859680	71895336	-0.371357732	PASS
ZNF823	19	11721265	11739009	0.093487731	suppress_segment_too_large
ZNF827	4	145757627	145938635	-0.011382299	PASS
ZNF829	19	36888124	36916291	0.093487731	suppress_segment_too_large
ZNF83	19	52594060	52690496	0.206912349	PASS
ZNF830	17	34961530	34963775	0.060555011	PASS
ZNF831	20	59123381	59259113	-0.168668583	PASS
ZNF835	19	56661981	56671783	-0.180829161	PASS

ZNF836	19	52153864	52171643	0.206912349	PASS
ZNF837	19	58367618	58381060	0.063781462	PASS
ZNF839	14	102317377	102342702	-0.192197564	PASS
ZNF84	12	133037292	133063304	0.270848837	PASS
ZNF841	19	52064466	52095765	0.206912349	PASS
ZNF843	16	31432593	31443160	0.069936844	PASS
ZNF844	19	12064699	12081565	0.093487731	suppress_segment_too_large
ZNF845	19	53333746	53356906	0.206912349	PASS
ZNF846	19	9751993	9793180	0.093487731	suppress_segment_too_large
ZNF85	19	20923222	20950697	0.093487731	suppress_segment_too_large
ZNF850	19	36714383	36772825	0.093487731	suppress_segment_too_large
ZNF852	3	44494847	44510636	-0.234617007	PASS
ZNF853	7	6615617	6624290	-0.69513118	PASS
ZNF860	3	31981771	31991628	0.311018244	suppress_segment_too_large
ZNF862	7	149838367	149867479	-0.388946609	PASS
ZNF865	19	55605405	55617269	0.121336758	PASS
ZNF878	19	12043805	12052939	0.093487731	suppress_segment_too_large
ZNF879	5	179023752	179035064	-0.150979337	PASS
ZNF880	19	52369917	52385795	0.206912349	PASS
ZNF883	9	112997120	113050043	0.365190713	suppress_segment_too_large
ZNF888	19	52904417	52923481	0.206912349	PASS
ZNF891	12	133106817	133130473	0.270848837	PASS
ZNF90	19	20077994	20127076	0.093487731	suppress_segment_too_large
ZNF91	19	23304991	23395560	0.093487731	suppress_segment_too_large
ZNF92	7	65373799	65401135	-0.750061359	PASS
ZNF93	19	19900913	19963464	0.093487731	suppress_segment_too_large
ZNF98	19	22391019	22532485	0.093487731	suppress_segment_too_large
ZNF99	19	22752183	22784107	0.093487731	suppress_segment_too_large
ZNFX1	20	49237946	49278426	-0.168668583	PASS
ZNHIT1	7	101217668	101224190	-0.388946609	PASS
ZNHIT2	11	65116403	65117708	0.182439925	suppress_segment_too_large
ZNHIT3	17	36486629	36499310	0.060555011	PASS
ZNHIT6	1	85649423	85708433	0.033763446	PASS
ZNRD1	6	30058899	30064909	0.048820648	PASS
ZNRF1	16	74999030	75110994	-0.371357732	PASS
ZNRF2	7	30284307	30367692	-0.69513118	PASS
ZNRF3	22	28883592	29057487	-0.575930959	PASS
ZNRF4	19	5455431	5456856	0.079632875	PASS
ZP1	11	60867562	60875693	0.182439925	suppress_segment_too_large
ZP2	16	21197450	21214510	0.069936844	PASS
ZP3	7	76397518	76442071	-0.262778904	PASS
ZP4	1	237877864	237890922	-0.648598641	PASS
ZPBP	7	49850421	50121329	-0.750061359	PASS
ZPBP2	17	39868164	39877896	0.060555011	PASS
ZPLD1	3	102099244	102479841	0.313454204	suppress_segment_too_large
ZPR1	11	116773799	116788050	-0.356188624	PASS

ZRANB1	10	124942123	124988189	0.063874692	PASS
ZRANB2	1	71063291	71081297	0.033763446	PASS
ZRANB3	2	135136916	135531236	-0.475215623	PASS
ZRSR2	23	15790472	15823260	-0.012567603	PASS
ZSCAN1	19	58034032	58054631	0.063781462	PASS
ZSCAN10	16	3088890	3099317	0.069936844	PASS
ZSCAN12	6	28378955	28399734	0.048820648	PASS
ZSCAN16	6	28124560	28130082	0.048820648	PASS
ZSCAN18	19	58083838	58118427	0.063781462	PASS
ZSCAN2	15	84600986	84627796	-0.697600509	PASS
ZSCAN20	1	33472645	33496507	0.033763446	PASS
ZSCAN21	7	100049774	100065038	-0.262778904	PASS
ZSCAN22	19	58327019	58342332	0.063781462	PASS
ZSCAN23	6	28431930	28443502	0.048820648	PASS
ZSCAN25	7	99616946	99632407	-0.262778904	PASS
ZSCAN26	6	28267010	28278224	0.048820648	PASS
ZSCAN29	15	43358172	43371025	-0.396255771	PASS
ZSCAN30	18	35251058	35290245	-0.376397843	PASS
ZSCAN31	6	28324693	28356271	0.048820648	PASS
ZSCAN32	16	3382081	3401065	0.069936844	PASS
ZSCAN4	19	57668935	57679152	-0.180829161	PASS
ZSCAN5A	19	56219670	56368383	-0.180829161	PASS
ZSCAN5B	19	56189570	56197920	-0.180829161	PASS
ZSCAN5C	19	56202301	56209452	-0.180829161	PASS
ZSCAN9	6	28224886	28233482	0.048820648	PASS
ZSWIM1	20	45881227	45885266	-0.168668583	PASS
ZSWIM2	2	186827835	186849208	-0.169541924	PASS
ZSWIM3	20	45857617	45879122	-0.168668583	PASS
ZSWIM4	19	13795460	13832230	0.093487731	suppress_segment_too_large
ZSWIM5	1	45016399	45306209	0.033763446	PASS
ZSWIM6	5	61332273	61546170	-0.293443042	PASS
ZSWIM7	17	15976560	15999717	0.024610076	PASS
ZSWIM8	10	73785582	73801797	0.072405807	PASS
ZSWIM9	19	48170692	48197620	0.14996833	PASS
ZUP1	6	116635618	116668794	-0.368590549	PASS
ZW10	11	113733187	113773811	-0.356188624	PASS
ZWILCH	15	66504959	66550128	-0.697600509	PASS
ZWINT	10	56357228	56361275	0.072405807	PASS
ZXDA	23	57906708	57910820	-0.012567603	PASS
ZXDB	23	57591652	57597545	-0.012567603	PASS
ZXDC	3	126437601	126475919	0.313454204	suppress_segment_too_large
ZYG11A	1	52842511	52894998	0.033763446	PASS
ZYG11B	1	52726467	52827342	0.033763446	PASS
ZYX	7	143381080	143391111	-0.388946609	PASS
ZZEF1	17	4004445	4143020	0.128406487	PASS

Supplementary Table 2b. Copy Number Variant calls when compared to primary for NEPC.

Gene	Chromosome	Gene_start	Gene_end	Log2Ratio(NEPCtoPrimary)	Filter
A1BG	19	58345178	58353499	0.142581352	PASS
A1CF	10	50799409	50885675	-0.389033546	PASS
A2M	12	9067664	9116229	0.505438545	PASS
A2ML1	12	8822472	8887001	0.505438545	PASS
A3GALT2	1	33306766	33321098	0.048749694	PASS
A4GALT	22	42692112	42721298	-0.740245994	PASS
A4GNT	3	138123718	138132387	0.06434429	PASS
AAAS	12	53307456	53324864	-0.743220499	PASS
AACS	12	125065379	125143333	0.028722739	PASS
AADAC	3	151814037	151828488	0.02267528	PASS
AADACL2	3	151733916	151761339	0.02267528	PASS
AADACL3	1	12716115	12728759	0.048749694	PASS
AADACL4	1	12644547	12667086	0.048749694	PASS
AADAT	4	170060222	170091699	0.142431244	suppress_segment_too_large
AAGAB	15	67201033	67255195	-0.759380635	PASS
AAK1	2	69457997	69674349	-0.46122572	PASS
AAMDC	11	77821109	77918432	0.064535884	PASS
AAMP	2	218264123	218270257	0.011001208	PASS
AANAT	17	76453351	76470117	0.051798665	PASS
AAR2	20	36236459	36270918	-0.172056771	suppress_segment_too_large
AARD	8	116938199	116944487	0.493344802	suppress_segment_too_large
AARS	16	70252295	70289543	-0.423061015	PASS
AARS2	6	44299654	44313326	0.028532667	PASS
AARSD1	17	42950526	42964498	0.041294512	PASS
AASDH	4	56338287	56387508	0.115781418	suppress_segment_too_large
AASDHPP	11	106075501	106098710	0.029107301	PASS
AASS	7	122075647	122144280	-0.712149877	PASS
AATF	17	36948875	37056871	0.041294512	PASS
AATK	17	81117295	81166077	0.051798665	PASS
ABAT	16	8674565	8784575	0.053595371	PASS
ABCA1	9	104781002	104928237	0.073960965	PASS
ABCA10	17	69147214	69244846	0.051798665	PASS
ABCA12	2	214931542	215138428	0.011001208	PASS
ABCA13	7	48171458	48647496	-2.672778419	PASS
ABCA2	9	137007227	137028922	0.073960965	PASS
ABCA3	16	2275881	2340746	0.053595371	PASS
ABCA4	1	93992835	94121132	0.079255572	PASS
ABCA5	17	69244311	69327244	0.051798665	PASS
ABCA6	17	69078702	69141888	0.051798665	PASS
ABCA7	19	1040101	1065572	-0.071973357	PASS
ABCA8	17	68867292	68955392	0.051798665	PASS
ABCA9	17	68974488	69061064	0.051798665	PASS
ABCB1	7	87503633	87713323	-0.221864321	PASS
ABCB10	1	229516582	229558695	-0.204996646	suppress_segment_too_large
ABCB11	2	168915498	169031324	0.033541467	PASS
ABCB4	7	87401697	87480435	-0.221864321	PASS
ABCB5	7	20615207	20777038	-0.72421718	PASS
ABCB6	2	219209766	219219017	0.011001208	PASS
ABCB7	23	75051554	75156732	0.158844052	PASS
ABCB8	7	151028422	151047782	-0.712149877	PASS
ABCB9	12	122920951	122981649	0.028722739	PASS
ABCC1	16	15949577	16143074	0.053595371	PASS

ABCC10	6	43427366	43450430	0.028532667	PASS
ABCC11	16	48166910	48247568	0.053595371	PASS
ABCC12	16	48081006	48156018	0.053595371	PASS
ABCC2	10	99782640	99852594	-0.414937136	PASS
ABCC3	17	50634777	50692252	0.051798665	PASS
ABCC4	13	95019829	95301475	-0.384313056	PASS
ABCC5	3	183919934	184018015	0.02267528	PASS
ABCC6	16	16148928	16223522	0.053595371	PASS
ABCC8	11	17392498	17476879	-0.39835217	PASS
ABCC9	12	21797401	21942529	0.49032213	suppress_segment_too_large
ABCD1	23	153724868	153744762	0.158844052	PASS
ABCD2	12	39550033	39619751	-0.46813528	PASS
ABCD3	1	94418455	94518666	0.079255572	PASS
ABCD4	14	74285423	74303056	0.053252507	PASS
ABCE1	4	145097932	145129179	0.142431244	suppress_segment_too_large
ABCF1	6	30571376	30597179	0.069043418	PASS
ABCF2	7	151212490	151227230	-0.712149877	PASS
ABCF3	3	184186023	184194012	0.02267528	PASS
ABCG1	21	42199689	42297244	0.209983559	PASS
ABCG2	4	88090264	88231322	-0.677982248	PASS
ABCG4	11	119149012	119162653	-0.431650509	PASS
ABCG5	2	43812472	43838865	0.034263343	PASS
ABCG8	2	43831942	43882987	0.034263343	PASS
ABHD1	2	27123789	27130812	0.034263343	PASS
ABHD10	3	111979010	111993363	0.06434429	PASS
ABHD11	7	73736094	73738867	-0.701511252	PASS
ABHD12	20	25294743	25390983	-0.172056771	suppress_segment_too_large
ABHD12B	14	50872160	50904970	0.053252507	PASS
ABHD13	13	108218379	108234255	-0.426822582	PASS
ABHD14A	3	51971426	51981199	-0.727808231	PASS
ABHD14A	3	51974706	51989183	-0.727808231	PASS
ABHD14B	3	51968510	51983409	-0.727808231	PASS
ABHD15	17	29560547	29567137	0.041294512	PASS
ABHD16A	6	31686949	31703444	0.028532667	PASS
ABHD16B	20	63861498	63862988	-0.172056771	suppress_segment_too_large
ABHD17A	19	1876810	1885547	-0.071973357	PASS
ABHD17B	9	71862452	71910931	0.073960965	PASS
ABHD17C	15	80679684	80755621	-0.759380635	PASS
ABHD18	4	127965306	128039927	0.142431244	suppress_segment_too_large
ABHD2	15	89087459	89202360	-0.759380635	PASS
ABHD3	18	21650897	21704805	-0.330515409	PASS
ABHD4	14	22598237	22613215	0.053252507	PASS
ABHD5	3	43690108	43734371	-0.727808231	PASS
ABHD6	3	58237506	58295693	-0.727808231	PASS
ABHD8	19	17292131	17310236	-0.069222905	PASS
ABI1	10	26746593	26861087	0.075711544	PASS
ABI2	2	203328219	203447723	0.011001208	PASS
ABI3	17	49210227	49223225	0.051798665	PASS
ABI3BP	3	100749156	100993515	0.22470242	PASS
ABL1	9	130713946	130887675	0.073960965	PASS
ABL2	1	179099327	179229684	0.088103245	PASS
ABLIM1	10	114431113	114685003	0.073090211	PASS
ABLIM2	4	7965310	8158832	-0.358623662	PASS
ABLIM3	5	149141483	149260542	-0.393084436	PASS

ABO	9	133250401	133275219	0.073960965	PASS
ABR	17	1003518	1229738	0.06854245	PASS
ABRA	8	106759483	106770245	0.70944438	suppress_segment_too_large
ABRACL	6	139028682	139043302	0.033894706	PASS
ABRAXAS	4	83459517	83523348	-0.677982248	PASS
ABRAXAS	10	124801785	124836670	0.073090211	PASS
ABT1	6	26596952	26600744	0.069043418	PASS
ABTB1	3	127672935	127680920	0.06434429	PASS
ABTB2	11	34150988	34358008	-0.359426047	PASS
AC000120	7	92112159	92179511	-0.710463272	PASS
AC000120	7	92131774	92245924	-0.710463272	PASS
AC001226	13	76992078	77129589	-0.384313056	PASS
AC002094	17	28364288	28368012	0.041294512	PASS
AC002310	16	30534227	30558279	0.053595371	PASS
AC002310	16	30525923	30534852	0.053595371	PASS
AC002985	19	18907013	18934397	-0.069222905	PASS
AC002996	12	111753890	111791418	0.028722739	PASS
AC003002	19	57363566	57398584	0.142581352	PASS
AC003002	19	57394183	57438457	0.142581352	PASS
AC003002	19	57389884	57419994	0.142581352	PASS
AC003005	19	57487718	57505381	0.142581352	PASS
AC003006	19	57682045	57753575	0.142581352	PASS
AC003688	17	7246829	7262089	0.06854245	PASS
AC004076	19	57437970	57477536	0.142581352	PASS
AC004080	7	27163535	27180013	-0.72421718	PASS
AC004233	16	2988256	3002016	0.053595371	PASS
AC004593	7	29122340	29514667	-0.72421718	PASS
AC004687	17	58345206	58417595	0.051798665	PASS
AC004691	7	30852273	30923812	-0.72421718	PASS
AC004754	16	249547	269943	0.053595371	PASS
AC004805	17	65538102	65641033	0.051798665	PASS
AC004832	22	30409255	30428990	-0.740245994	PASS
AC004922	7	99325879	99394653	-0.710463272	PASS
AC004997	22	30285238	30299482	-0.740245994	PASS
AC005020	7	99558695	99607810	-0.710463272	PASS
AC005041	2	74393836	74421662	-0.46122572	PASS
AC005154	7	30497077	30550761	-0.72421718	PASS
AC005258	19	2269525	2341172	-0.071973357	PASS
AC005324	17	15571491	15651653	0.06854245	PASS
AC005324	17	15699692	15744778	0.06854245	PASS
AC005520	14	73851971	73932278	0.053252507	PASS
AC005670	17	46923133	47049932	0.051798665	PASS
AC005697	17	27798806	27893365	0.041294512	PASS
AC005702	17	59940908	59973101	0.051798665	PASS
AC005726	17	28455752	28614197	0.041294512	PASS
AC005779	19	45179822	45202444	0.002743503	PASS
AC005832	12	4604944	4662643	-0.015029783	PASS
AC005833	12	4649101	4774184	-0.015029783	PASS
AC005837	17	76732978	76738522	0.051798665	PASS
AC005943	19	1578339	1605445	-0.071973357	PASS
AC006030	2	74211604	74363377	-0.46122572	PASS
AC006059	3	42705756	42804518	-0.727808231	PASS
AC006064	12	6556886	6607367	-0.015029783	PASS
AC006254	3	52813282	52835729	-0.727808231	PASS

AC006486	19	42234583	42255132	0.048917043	PASS
AC006518	12	10881011	10884255	0.49032213	suppress_segment_too_large
AC006538	19	2717769	2740048	-0.071973357	PASS
AC006978	7	30362400	30412502	-0.72421718	PASS
AC007040	2	70939318	70995336	-0.46122572	PASS
AC007192	19	18153158	18178117	-0.069222905	PASS
AC007240	2	10122739	10199288	0.034263343	PASS
AC007326	22	18906238	18947732	-0.740245994	PASS
AC007375	14	77098311	77225462	-0.155273506	suppress_segment_too_large
AC007731	22	20429241	20446620	-0.740245994	PASS
AC007846	17	5772926	6080792	0.06854245	PASS
AC007998	18	35443871	35497940	-0.330515409	PASS
AC008012	12	4273762	4405490	-0.015029783	PASS
AC008073	2	24124366	24190436	0.034263343	PASS
AC008397	19	18207961	18255419	-0.069222905	PASS
AC008403	19	48465837	48472431	0.002743503	PASS
AC008481	19	11466240	11505698	-0.107594294	PASS
AC008537	19	40801297	40898282	0.048917043	PASS
AC008575	5	112827213	112867582	-0.393084436	PASS
AC008581	5	77030902	77152155	-0.393084436	PASS
AC008676	5	157341596	157460100	0.165341871	suppress_segment_too_large
AC008687	19	49022953	49036895	0.002743503	PASS
AC008687	19	49017496	49020523	0.002743503	PASS
AC008687	19	49055793	49065076	0.002743503	PASS
AC008695	5	131425891	131796983	-0.393084436	PASS
AC008750	19	51350373	51368099	0.002743503	PASS
AC008758	19	12461559	12551474	0.391988834	PASS
AC008758	19	12379189	12401274	0.391988834	PASS
AC008758	19	12391949	12441082	0.391988834	PASS
AC008758	19	12433103	12484816	0.391988834	PASS
AC008763	19	7629796	7643048	-0.071973357	PASS
AC008763	19	7678501	7682854	-0.071973357	PASS
AC008764	19	16479067	16628204	-0.069222905	PASS
AC008764	19	16577642	16660115	-0.069222905	PASS
AC008770	19	12017888	12052967	0.391988834	PASS
AC008770	19	11925075	11979290	0.391988834	PASS
AC008878	19	7507052	7519622	-0.071973357	PASS
AC008878	19	7533595	7540059	-0.071973357	PASS
AC008878	19	7382834	7472477	-0.071973357	PASS
AC008982	19	38817471	38840178	0.048917043	PASS
AC009086	16	29663279	29695144	0.053595371	PASS
AC009119	16	83908132	83951445	-0.423061015	PASS
AC009133	16	29812261	29820092	0.053595371	PASS
AC009163	16	75528532	75545428	-0.423061015	PASS
AC009163	16	75412684	75464706	-0.423061015	PASS
AC009336	2	176151085	176173097	0.033541467	PASS
AC009690	15	72284727	72375981	-0.759380635	PASS
AC009690	15	72266746	72319946	-0.759380635	PASS
AC009879	8	66430185	66445324	0.182873287	suppress_segment_too_large
AC009879	8	66432486	66518524	0.182873287	suppress_segment_too_large
AC010132	7	42909273	42932174	-0.72421718	PASS
AC010197	12	21536203	21657798	0.49032213	suppress_segment_too_large
AC010255	5	122129622	122183949	-0.393084436	PASS
AC010319	19	17436611	17460804	-0.069222905	PASS

AC010323	19	8308283	8321379	-0.071973357	PASS
AC010325	19	50776141	50793142	0.002743503	PASS
AC010326	19	57876765	57916591	0.142581352	PASS
AC010327	19	55154757	55160671	0.142581352	PASS
AC010422	19	12643831	12648397	-0.069222905	PASS
AC010422	19	12664828	12669397	-0.069222905	PASS
AC010422	19	12525720	12580975	0.391988834	PASS
AC010422	19	12668071	12681834	-0.069222905	PASS
AC010463	19	17267418	17282966	-0.069222905	PASS
AC010487	19	52880663	52962768	0.416834572	PASS
AC010522	19	57819719	57858941	0.142581352	PASS
AC010531	16	87302876	87334273	-0.423061015	PASS
AC010542	16	66469812	66517312	-0.423061015	PASS
AC010547	16	71447600	71489311	-0.423061015	PASS
AC010615	19	21405159	21491266	-0.069222905	PASS
AC010616	19	41907705	41928516	0.048917043	PASS
AC010616	19	41956879	41994232	0.048917043	PASS
AC010619	19	49462752	49486231	0.002743503	PASS
AC010646	19	17235940	17255448	-0.069222905	PASS
AC011005	7	129126518	129130793	-0.712149877	PASS
AC011195	17	58660424	58692018	0.051798665	PASS
AC011330	15	43614208	43632283	-0.423772929	PASS
AC011448	19	19516227	19536076	-0.069222905	PASS
AC011452	19	49909501	49958391	0.002743503	PASS
AC011455	19	38915404	38949855	0.048917043	PASS
AC011462	19	41350853	41425001	0.048917043	PASS
AC011473	19	50996018	51009581	0.002743503	PASS
AC011479	19	38289151	38304910	0.048917043	PASS
AC011498	19	4528427	4540067	-0.071973357	PASS
AC011499	19	5784005	5785619	-0.071973357	PASS
AC011511	19	10305427	10316009	-0.107594294	PASS
AC011511	19	10315471	10320678	-0.107594294	PASS
AC011530	19	45779437	45785973	0.002743503	PASS
AC011604	12	20815674	21090245	0.49032213	suppress_segment_too_large
AC012184	16	70299194	70372582	-0.423061015	PASS
AC012213	8	103398658	103501895	0.70944438	suppress_segment_too_large
AC012254	18	47108188	47176345	-0.330515409	PASS
AC012309	19	37093019	37210512	0.396324882	PASS
AC012476	15	40695129	40732158	-0.423772929	PASS
AC012531	12	53985845	54034888	-0.743220499	PASS
AC012651	15	42348103	42412317	-0.423772929	PASS
AC013394	15	92883413	92949230	-0.759380635	PASS
AC013470	7	12469621	12542222	-0.72421718	PASS
AC013489	15	88459501	88546585	-0.759380635	PASS
AC013717	2	44168851	44320310	0.034263343	PASS
AC015688	17	27623364	27640777	0.041294512	PASS
AC015802	17	76563710	76570544	0.051798665	PASS
AC015813	17	57989038	58007246	0.051798665	PASS
AC017083	2	68131238	68261230	-0.46122572	PASS
AC018362	15	42497395	42548786	-0.423772929	PASS
AC018512	15	43772617	43799133	-0.423772929	PASS
AC018523	11	14493783	14520344	-0.39835217	PASS
AC018554	16	60358455	60359480	-0.423061015	PASS
AC018709	4	186426546	186555328	0.275137421	PASS

AC018755	19	51612091	51646889	0.002743503	PASS
AC019117	7	17299295	17467234	-0.72421718	PASS
AC020636	3	150882997	150941743	0.02267528	PASS
AC020907	19	35106510	35124297	0.044838622	PASS
AC020909	19	50415799	50428409	0.002743503	PASS
AC020915	19	58278966	58315197	0.142581352	PASS
AC020915	19	58228914	58315183	0.142581352	PASS
AC020922	19	55352301	55359516	0.142581352	PASS
AC021097	7	151207837	151227166	-0.712149877	PASS
AC021097	7	151207837	151210378	-0.712149877	PASS
AC021660	3	98521133	98585498	0.22470242	PASS
AC022335	12	21047179	21176895	0.49032213	suppress_segment_too_large
AC022384	3	10249372	10285796	0.06149513	PASS
AC022400	10	73796514	73811651	0.032351597	PASS
AC022414	5	77086732	77427124	-0.393084436	PASS
AC022506	12	57517712	57520480	-0.743220499	PASS
AC022826	8	73688691	73878854	0.182873287	suppress_segment_too_large
AC023055	12	55757275	55827546	-0.743220499	PASS
AC023509	12	53506688	53625979	-0.743220499	PASS
AC024592	19	5866171	5903787	-0.071973357	PASS
AC025165	12	57773028	57787046	-0.743220499	PASS
AC025263	12	69801669	69855363	-0.578548701	PASS
AC025283	16	3382113	3397745	0.053595371	PASS
AC025283	16	3365099	3479550	0.053595371	PASS
AC026316	3	170418868	170860380	0.02267528	PASS
AC026398	5	131807143	131994278	-0.393084436	PASS
AC026464	16	69335276	69351730	-0.423061015	PASS
AC026464	16	69335091	69356306	-0.423061015	PASS
AC026464	16	69299682	69322700	-0.423061015	PASS
AC026464	16	69328644	69334871	-0.423061015	PASS
AC026786	12	133130984	133202369	0.232349073	PASS
AC026954	17	7312661	7319174	0.06854245	PASS
AC027644	7	66628958	66811189	-0.701511252	PASS
AC027796	17	3585149	3636249	0.06854245	PASS
AC034102	12	56101331	56109289	-0.743220499	PASS
AC036214	8	79918860	80080775	0.617295349	suppress_segment_too_large
AC037459	8	22589274	22602084	-0.311940419	PASS
AC040162	16	67929614	67936017	-0.423061015	PASS
AC046185	17	63702845	63752097	0.051798665	PASS
AC048338	12	122207779	122266423	0.028722739	PASS
AC055811	17	17202649	17237185	0.06854245	PASS
AC058822	4	53377839	54295272	0.115781418	suppress_segment_too_large
AC067752	10	62520448	62672011	0.032351597	PASS
AC067968	19	44025354	44087318	0.415645458	PASS
AC068234	17	47253897	47344292	0.051798665	PASS
AC068533	7	66087761	66152277	-0.701511252	PASS
AC068547	2	151802651	151973949	-0.30549516	PASS
AC068580	11	1734821	1763954	-0.39835217	PASS
AC068631	3	186581995	186597103	0.02267528	PASS
AC068775	12	10412312	10436001	0.505438545	PASS
AC068790	12	123973944	124088594	0.028722739	PASS
AC068831	15	90966190	91022566	-0.759380635	PASS
AC068896	10	124617080	124791727	0.073090211	PASS
AC068946	2	219075329	219170827	0.011001208	PASS

AC068946	2	219209772	219222738	0.011001208	PASS
AC069257	3	196248230	196318222	0.02267528	PASS
AC069368	15	64841883	64930920	-0.759380635	PASS
AC069444	3	119703076	119807570	0.06434429	PASS
AC069503	12	121888809	121921470	0.028722739	PASS
AC073082	2	48632291	48755724	0.034263343	PASS
AC073111	7	150368790	150396915	-0.712149877	PASS
AC073111	7	150400702	150412470	-0.712149877	PASS
AC073283	2	47065941	47176511	0.034263343	PASS
AC073508	17	40627356	40665141	0.041294512	PASS
AC073610	12	48921963	48939663	-0.743220499	PASS
AC073610	12	48903503	48957365	-0.743220499	PASS
AC073612	12	71674204	71698968	-0.578548701	PASS
AC073896	12	56285916	56316059	-0.743220499	PASS
AC074143	16	67163385	67165815	-0.423061015	PASS
AC078927	12	66123917	66169985	-0.743220499	PASS
AC079447	2	99141485	99322741	0.064122364	PASS
AC079594	3	160227454	160449829	0.02267528	PASS
AC083800	3	119147375	119187807	0.06434429	PASS
AC084337	11	6481485	6508978	-0.39835217	PASS
AC087289	17	75898645	75930129	0.051798665	PASS
AC087632	15	64181180	64381440	-0.759380635	PASS
AC087721	15	41284013	41380743	-0.423772929	PASS
AC090004	3	14135217	14148252	0.06149513	PASS
AC090227	18	49795793	49837623	-0.330515409	PASS
AC090360	18	80034346	80097088	-0.330515409	PASS
AC090517	15	56652488	56918571	-0.759380635	PASS
AC090527	15	45587351	45676314	-0.423772929	PASS
AC091057	15	30624548	30685606	-0.423772929	PASS
AC091167	15	90249556	90272208	-0.759380635	PASS
AC091167	15	90266317	90314499	-0.759380635	PASS
AC091167	15	90234200	90265462	-0.759380635	PASS
AC091551	18	50968019	51058144	-0.330515409	PASS
AC091959	5	146203550	146339251	-0.393084436	PASS
AC092017	1	211082872	211188533	-0.204996646	suppress_segment_too_large
AC092042	3	42809446	43055871	-0.727808231	PASS
AC092073	19	34396315	34409364	0.044838622	PASS
AC092111	12	8138140	8175320	-0.015029783	PASS
AC092143	16	89919165	89936092	-0.423061015	PASS
AC092329	19	23222755	23274221	0.044838622	PASS
AC092442	4	6064977	6070162	-0.358623662	PASS
AC092587	2	99154998	99195298	0.064122364	PASS
AC092647	7	55887277	55955239	-0.701511252	PASS
AC092718	16	81053587	81096296	-0.423061015	PASS
AC092718	16	81022316	81096284	-0.423061015	PASS
AC092835	2	95207535	95259774	0.064122364	PASS
AC093155	1	86993009	87169204	0.079255572	PASS
AC093227	19	37689865	37719188	0.396324882	PASS
AC093423	1	89633140	89933250	0.079255572	PASS
AC093512	16	30053090	30070420	0.053595371	PASS
AC093525	16	2513965	2527955	0.053595371	PASS
AC093525	16	2496032	2520218	0.053595371	PASS
AC093668	7	102541501	102592444	-0.712149877	PASS
AC093668	7	102483344	102543764	-0.712149877	PASS

AC093827	4	86886472	86936178	-0.677982248	PASS
AC093899	2	169479480	169525922	0.033541467	PASS
AC096887	3	52969119	53099453	-0.727808231	PASS
AC097104	12	56449700	56488170	-0.743220499	PASS
AC097625	23	15561047	15675608	0.158844052	PASS
AC097634	3	70959226	71754229	-0.727808231	PASS
AC097636	3	51798803	51830856	-0.727808231	PASS
AC097637	3	52221081	52231190	-0.727808231	PASS
AC098484	1	42767292	42794493	-0.736943052	PASS
AC098582	4	88523810	88649847	-0.677982248	PASS
AC098588	4	144210701	145098174	0.142431244	suppress_segment_too_large
AC098650	3	28349178	29767537	0.06149513	PASS
AC098850	17	16690261	16804453	0.06854245	PASS
AC099489	16	11359845	11527245	0.053595371	PASS
AC099811	17	42119674	42154916	0.041294512	PASS
AC099850	17	59197566	59215226	0.051798665	PASS
AC100868	8	132024238	132085655	0.493344802	suppress_segment_too_large
AC104109	5	134167170	134226071	-0.393084436	PASS
AC104151	16	76553417	76819624	-0.423061015	PASS
AC104304	3	46581150	46617226	-0.727808231	PASS
AC104389	11	5254392	5678434	-0.39835217	PASS
AC104389	11	5248270	5254783	-0.39835217	PASS
AC104452	3	49416777	49429314	-0.727808231	PASS
AC104472	3	155763043	155854442	0.02267528	PASS
AC104532	19	5904841	5914707	-0.071973357	PASS
AC104581	17	7925931	7930622	0.06854245	PASS
AC104662	4	25160663	25277306	-0.358623662	PASS
AC105052	7	102582523	102642869	-0.712149877	PASS
AC105052	7	102637049	102671641	-0.712149877	PASS
AC106774	5	17491325	17491769	0.073527871	PASS
AC106886	16	30704067	30745169	0.053595371	PASS
AC107871	15	68184032	68229718	-0.759380635	PASS
AC107959	8	23084403	23115536	-0.311940419	PASS
AC108488	2	3531984	3547957	0.034263343	PASS
AC108941	4	69810780	69847786	0.115781418	suppress_segment_too_large
AC109583	3	46742092	46812558	-0.727808231	PASS
AC110275	8	43018424	43077334	0.182873287	suppress_segment_too_large
AC112128	3	113361901	113515151	0.06434429	PASS
AC112229	2	110402934	110473075	0.040971718	PASS
AC112504	3	141738474	141807919	-0.889410434	PASS
AC113348	5	178694605	178729335	0.165341871	suppress_segment_too_large
AC113554	17	63484823	63521848	0.051798665	PASS
AC114490	1	34981533	35031741	0.048749694	PASS
AC114490	1	34974356	34985313	0.048749694	PASS
AC115220	7	63998874	64016130	-0.701511252	PASS
AC116366	5	132410832	132646079	-0.393084436	PASS
AC117378	12	56714612	56741535	-0.743220499	PASS
AC117457	3	178419123	178843300	0.02267528	PASS
AC118470	1	247711813	247723764	-0.145670469	suppress_likely_unfocal_large_gain
AC118549	1	77562416	77683419	0.052232535	PASS
AC118553	1	99970011	100083321	0.079255572	PASS
AC119396	19	7348943	7383385	-0.071973357	PASS
AC119674	1	56173433	56524495	-0.736943052	PASS
AC120057	17	7240427	7244635	0.06854245	PASS

AC120114	16	29817239	29830627	0.053595371	PASS
AC124312	15	24955034	25000276	-0.423772929	PASS
AC126283	4	109713916	109801999	-0.677982248	PASS
AC126755	16	18358086	18379331	0.053595371	PASS
AC127029	17	63917203	63931385	0.051798665	PASS
AC129492	17	8150816	8162975	0.06854245	PASS
AC131160	3	183815568	183884889	0.02267528	PASS
AC134669	17	31305213	31318831	0.041294512	PASS
AC134684	8	7579644	7582653	-0.311940419	PASS
AC134980	15	22015233	22095857	-0.423772929	PASS
AC135050	16	31083439	31094956	0.053595371	PASS
AC135068	15	21637909	21638992	-0.423772929	PASS
AC135178	17	8368638	8383187	0.06854245	PASS
AC136428	16	33844784	33845229	0.053595371	PASS
AC137834	12	57249609	57296484	-0.743220499	PASS
AC138696	8	143247110	143276403	0.699132336	PASS
AC138811	16	18788063	18801519	0.053595371	PASS
AC138894	16	28456371	28492098	0.053595371	PASS
AC139491	5	176063748	176126959	0.165341871	suppress_segment_too_large
AC139530	17	81703371	81720539	0.051798665	PASS
AC140504	16	15395754	15515348	0.053595371	PASS
AC142391	5	139442316	139462707	-0.393084436	PASS
AC144573	8	37934340	37965953	0.182873287	suppress_segment_too_large
AC233992	8	144353228	144355609	0.699132336	PASS
AC235565	23	102360395	102471812	0.158844052	PASS
AC239799	1	146113652	146229000	0.079255572	PASS
AC243547	1	145927258	145977811	0.079255572	PASS
AC243756	1	146344131	146344790	0.079255572	PASS
AC243967	19	41708612	41761282	0.048917043	PASS
AC244197	23	149482543	149533935	0.158844052	PASS
AC244517	5	141223372	141245861	-0.393084436	PASS
AC245033	15	82536788	82573194	-0.759380635	PASS
AC245748	19	44329695	44401608	0.415645458	PASS
AC253536	22	23971378	23980469	-0.740245994	PASS
ACAA1	3	38103129	38137242	-0.727808231	PASS
ACAA2	18	49782167	49813960	-0.330515409	PASS
ACACA	17	37084988	37406818	0.041294512	PASS
ACACB	12	109116595	109268226	0.028722739	PASS
ACAD10	12	111686056	111757107	0.028722739	PASS
ACAD11	3	132558138	132660723	0.06434429	PASS
ACAD8	11	134253495	134265855	0.105032849	PASS
ACAD9	3	128879596	128916067	0.06434429	PASS
ACADL	2	210187939	210225491	0.011001208	PASS
ACADM	1	75724347	75787575	0.052232535	PASS
ACADS	12	120725735	120740008	0.028722739	PASS
ACADSB	10	123008979	123058311	0.073090211	PASS
ACADVL	17	7217125	7225273	0.06854245	PASS
ACAN	15	88803442	88875354	-0.759380635	PASS
ACAP1	17	7336529	7351478	0.06854245	PASS
ACAP2	3	195274736	195443078	0.02267528	PASS
ACAP3	1	1292376	1309609	-0.358281697	PASS
ACAT1	11	108121516	108147776	-0.431650509	PASS
ACAT2	6	159760328	159779055	0.033894706	PASS
ACBD3	1	226144679	226186730	-0.204996646	suppress_segment_too_large

ACBD4	17	45132600	45144181	0.051798665	PASS
ACBD5	10	27195214	27242130	0.075711544	PASS
ACBD6	1	180269653	180502954	0.088103245	PASS
ACBD7	10	15077523	15088776	0.075711544	PASS
ACCS	11	44065925	44084222	0.059795455	PASS
ACCSL	11	44047981	44059977	0.059795455	PASS
ACD	16	67657512	67660815	-0.423061015	PASS
ACE	17	63477061	63498380	0.051798665	PASS
ACE2	23	15561033	15602148	0.158844052	PASS
ACER1	19	6306142	6333629	-0.071973357	PASS
ACER2	9	19409059	19452020	0.073960965	PASS
ACER3	11	76860867	77026797	0.064535884	PASS
ACHE	7	100889994	100896974	-0.710463272	PASS
ACIN1	14	23058564	23095614	0.053252507	PASS
ACKR1	1	159203307	159206500	0.088103245	PASS
ACKR2	3	42804752	42887974	-0.727808231	PASS
ACKR3	2	236567787	236582358	0.011001208	PASS
ACKR4	3	132597237	132618967	0.06434429	PASS
ACLY	17	41866908	41930542	0.041294512	PASS
ACMSD	2	134838547	134902034	-0.359440172	PASS
ACO1	9	32384603	32454769	0.073960965	PASS
ACO2	22	41469125	41528989	-0.740245994	PASS
ACOD1	13	76948497	76958642	-0.384313056	PASS
ACOT1	14	73537114	73543794	0.053252507	PASS
ACOT11	1	54542257	54639192	-0.736943052	PASS
ACOT12	5	81330005	81394179	-0.393084436	PASS
ACOT13	6	24667035	24705065	0.069043418	PASS
ACOT2	14	73567620	73575658	0.053252507	PASS
ACOT4	14	73591706	73596496	0.053252507	PASS
ACOT6	14	73610945	73619888	0.053252507	PASS
ACOT7	1	6264269	6394391	0.364796772	PASS
ACOT8	20	45841721	45857406	-0.172056771	suppress_segment_too_large
ACOT9	23	23702253	23766475	0.158844052	PASS
ACOX1	17	75941507	75979363	0.051798665	PASS
ACOX2	3	58505136	58537319	-0.727808231	PASS
ACOX3	4	8366282	8440723	-0.358623662	PASS
ACOXL	2	110732573	111118222	0.040971718	PASS
ACP1	2	264140	278283	0.034263343	PASS
ACP2	11	47239302	47248906	0.059795455	PASS
ACP4	19	50790415	50795224	0.002743503	PASS
ACP5	19	11574660	11579008	-0.107594294	PASS
ACP6	1	147629652	147670496	0.079255572	PASS
ACP7	19	39083913	39111493	0.048917043	PASS
ACPP	3	132317367	132368298	0.06434429	PASS
ACR	22	50738196	50745334	-0.953096644	PASS
ACRBP	12	6638075	6647460	-0.015029783	PASS
ACRV1	11	125671522	125681123	-0.431650509	PASS
ACSBG1	15	78167468	78245688	-0.759380635	PASS
ACSBG2	19	6135247	6193094	-0.071973357	PASS
ACSF2	17	50426158	50474845	0.051798665	PASS
ACSF3	16	89088375	89164121	-0.423061015	PASS
ACSL1	4	184755595	184826818	-0.453876349	PASS
ACSL3	2	222860934	222944639	0.011001208	PASS
ACSL4	23	109624244	109733403	0.158844052	PASS

ACSL5	10	112374018	112428380	0.073090211	PASS
ACSL6	5	131949973	132012243	-0.393084436	PASS
ACSM1	16	20623237	20698890	0.053595371	PASS
ACSM2A	16	20451461	20487667	0.053595371	PASS
ACSM2B	16	20536226	20576427	0.053595371	PASS
ACSM3	16	20610243	20797581	0.053595371	PASS
ACSM4	12	7304284	7328724	-0.015029783	PASS
ACSM5	16	20409534	20441336	0.053595371	PASS
ACSM6	10	95194200	95228928	-0.414937136	PASS
ACSS1	20	25006230	25058980	-0.172056771	suppress_segment_too_large
ACSS2	20	34872146	34927962	-0.172056771	suppress_segment_too_large
ACSS3	12	80936414	81261205	0.271127429	suppress_segment_too_large
ACTA1	1	229431245	229434098	-0.204996646	suppress_segment_too_large
ACTA2	10	88935074	88991339	-2.417109027	suppress_large_homdel
ACTB	7	5527147	5563784	-0.72421718	PASS
ACTBL2	5	57480016	57482809	-0.284712224	PASS
ACTC1	15	34788095	34795726	-0.423772929	PASS
ACTG1	17	81509971	81523847	0.051798665	PASS
ACTG2	2	73892314	73919865	-0.46122572	PASS
ACTL10	20	33666498	33668525	-0.172056771	suppress_segment_too_large
ACTL6A	3	179562880	179588408	0.02267528	PASS
ACTL6B	7	100643097	100656461	-0.710463272	PASS
ACTL7A	9	108862266	108863759	0.073960965	PASS
ACTL7B	9	108854589	108856967	0.073960965	PASS
ACTL8	1	17755313	17827063	0.048749694	PASS
ACTL9	19	8697397	8698822	-0.071973357	PASS
ACTN1	14	68874143	68979440	0.053252507	PASS
ACTN2	1	236686454	236764631	-0.204996646	suppress_segment_too_large
ACTN3	11	66546395	66563329	0.064535884	PASS
ACTN4	19	38647649	38731583	0.048917043	PASS
ACTR10	14	58200080	58235636	0.053252507	PASS
ACTR1A	10	102461881	102502711	-0.414937136	PASS
ACTR1B	2	97655963	97664107	0.064122364	PASS
ACTR2	2	65227753	65271253	-0.46122572	PASS
ACTR3	2	113889960	113962596	0.040971718	PASS
ACTR3B	7	152759749	152855378	-0.77630473	PASS
ACTR3C	7	150243916	150323725	-0.712149877	PASS
ACTR5	20	38748442	38772520	-0.172056771	suppress_segment_too_large
ACTR6	12	100199122	100241865	0.028722739	PASS
ACTR8	3	53867066	53882202	-0.727808231	PASS
ACTRT1	23	128050962	128052398	0.158844052	PASS
ACTRT2	1	3021483	3022903	-0.358281697	PASS
ACTRT3	3	169766921	169769895	0.02267528	PASS
ACVR1	2	157736444	157875862	0.054749241	PASS
ACVR1B	12	51951667	51997078	-0.743220499	PASS
ACVR1C	2	157526767	157629005	0.054749241	PASS
ACVR2A	2	147844517	147930826	-0.30549516	PASS
ACVR2B	3	38453851	38493142	-0.727808231	PASS
ACVRL1	12	51906908	51923361	-0.743220499	PASS
ACY1	3	51983278	51989202	-0.727808231	PASS
ACY3	11	67642555	67650659	0.064535884	PASS
ACYP1	14	75053221	75069483	-0.155273506	suppress_segment_too_large
ACYP2	2	53970838	54305300	0.034263343	PASS
AD000671	19	35745678	35754519	0.044838622	PASS

AD000671	19	35739252	35745432	0.044838622	PASS
ADA	20	44619522	44652233	-0.172056771	suppress_segment_too_large
ADA2	22	17178790	17258235	-0.740245994	PASS
ADAD1	4	122378966	122429802	0.142431244	suppress_segment_too_large
ADAD2	16	84191138	84197168	-0.423061015	PASS
ADAL	15	43330657	43354555	-0.423772929	PASS
ADAM10	15	58588807	58749978	-0.759380635	PASS
ADAM11	17	44759031	44781846	0.051798665	PASS
ADAM12	10	126012381	126388455	0.073090211	PASS
ADAM15	1	155050566	155062775	0.088103245	PASS
ADAM17	2	9488486	9556732	0.034263343	PASS
ADAM18	8	39584489	39730064	0.182873287	suppress_segment_too_large
ADAM19	5	157395534	157575775	0.165341871	suppress_segment_too_large
ADAM2	8	39743735	39838289	0.182873287	suppress_segment_too_large
ADAM20	14	70522358	70535015	0.053252507	PASS
ADAM21	14	70452157	70459899	0.053252507	PASS
ADAM22	7	87934143	88202889	-0.221864321	PASS
ADAM23	2	206443539	206621130	0.011001208	PASS
ADAM28	8	24294040	24359018	-0.311940419	PASS
ADAM29	4	174829668	174978180	0.142431244	suppress_segment_too_large
ADAM30	1	119893533	119896495	0.079255572	PASS
ADAM32	8	39106990	39284911	0.182873287	suppress_segment_too_large
ADAM33	20	3667965	3682246	-0.172056771	suppress_segment_too_large
ADAM7	8	24440930	24526970	-0.311940419	PASS
ADAM8	10	133262403	133276868	0.073090211	PASS
ADAM9	8	38996869	39105144	0.182873287	suppress_segment_too_large
ADAMDE	8	24384285	24406013	-0.311940419	PASS
ADAMTS1	21	26835747	26845409	0.209983559	PASS
ADAMTS1	19	8580242	8610735	-0.071973357	PASS
ADAMTS1	5	33523535	33892192	0.073527871	PASS
ADAMTS1	9	133414358	133459402	0.073960965	PASS
ADAMTS1	10	70672803	70762441	0.032351597	PASS
ADAMTS1	11	130448974	130476641	-0.431650509	PASS
ADAMTS1	5	5140330	5320304	0.073527871	PASS
ADAMTS1	15	99971589	100342005	-0.759380635	PASS
ADAMTS1	16	77247813	77435114	-0.423061015	PASS
ADAMTS1	5	129460265	129738683	-0.393084436	PASS
ADAMTS2	5	179110851	179345430	0.165341871	suppress_segment_too_large
ADAMTS2	12	43353866	43551921	-0.46813528	PASS
ADAMTS3	4	72280969	72569386	0.115781418	suppress_segment_too_large
ADAMTS4	1	161184308	161199056	0.088103245	PASS
ADAMTS5	21	26917912	26966513	0.209983559	PASS
ADAMTS6	5	65148736	65481920	-0.393084436	PASS
ADAMTS7	15	78759203	78811431	-0.759380635	PASS
ADAMTS8	11	130404925	130428993	-0.431650509	PASS
ADAMTS9	3	64515654	64688000	-0.727808231	PASS
ADAMTSI	9	17906563	18910950	0.073960965	PASS
ADAMTSI	9	133532164	133575519	0.073960965	PASS
ADAMTSI	15	83654086	84039842	-0.759380635	PASS
ADAMTSI	1	150549369	150560937	0.079255572	PASS
ADAMTSI	19	1505018	1513604	-0.071973357	PASS
ADAP1	7	897901	955407	-0.72421718	PASS
ADAP2	17	30906344	30959322	0.041294512	PASS
ADAR	1	154582057	154628013	0.088103245	PASS

ADARB1	21	45073853	45226560	0.209983559	PASS
ADARB2	10	1177318	1737476	0.075711544	PASS
ADAT1	16	75596981	75623300	-0.423061015	PASS
ADAT2	6	143422832	143450673	0.033894706	PASS
ADAT3	19	1905378	1913447	-0.071973357	PASS
ADCK1	14	77800083	77935012	-0.155273506	suppress_segment_too_large
ADCK2	7	140673153	140696261	-0.712149877	PASS
ADCK5	8	144373101	144393242	0.699132336	PASS
ADCY1	7	45574140	45723116	-0.72421718	PASS
ADCY10	1	167809388	167914215	0.088103245	PASS
ADCY2	5	7396208	7830081	0.073527871	PASS
ADCY3	2	24819169	24919839	0.034263343	PASS
ADCY4	14	24318349	24335093	0.053252507	PASS
ADCY5	3	123282296	123449758	0.06434429	PASS
ADCY6	12	48766194	48789037	-0.743220499	PASS
ADCY7	16	50246137	50318135	0.411512417	PASS
ADCY8	8	130780301	131042426	0.493344802	suppress_segment_too_large
ADCY9	16	3953387	4116185	0.053595371	PASS
ADCYAP1	18	904943	912172	0.044197172	PASS
ADCYAP1	7	31052461	31111479	-0.72421718	PASS
ADD1	4	2843857	2930076	-0.358623662	PASS
ADD2	2	70607618	70768225	-0.46122572	PASS
ADD3	10	109996368	110135565	0.073090211	PASS
ADGB	6	146598965	146815462	0.033894706	PASS
ADGRA1	10	133070929	133131675	0.073090211	PASS
ADGRA2	8	37784191	37844896	0.182873287	suppress_segment_too_large
ADGRA3	4	22345071	22516054	-0.358623662	PASS
ADGRB1	8	142449430	142545009	0.699132336	PASS
ADGRB2	1	31727117	31764893	0.048749694	PASS
ADGRB3	6	68635367	69389511	-0.250122709	PASS
ADGRD1	12	130953907	131141469	0.028722739	PASS
ADGRD2	9	124451425	124478589	0.073960965	PASS
ADGRE1	19	6887566	6940459	-0.071973357	PASS
ADGRE2	19	14732393	14778541	-0.069222905	PASS
ADGRE3	19	14619117	14690027	-0.069222905	PASS
ADGRE5	19	14380501	14408725	-0.069222905	PASS
ADGRF1	6	46997703	47042363	0.239805577	suppress_segment_too_large
ADGRF2	6	47656436	47697797	0.239805577	suppress_segment_too_large
ADGRF3	2	26308173	26346817	0.034263343	PASS
ADGRF4	6	47685864	47722021	0.239805577	suppress_segment_too_large
ADGRF5	6	46852512	46954943	0.239805577	suppress_segment_too_large
ADGRG1	16	57610652	57665580	-0.423061015	PASS
ADGRG2	23	18989309	19122637	0.158844052	PASS
ADGRG3	16	57668187	57689378	-0.423061015	PASS
ADGRG4	23	136300963	136416888	0.158844052	PASS
ADGRG5	16	57542421	57591681	-0.423061015	PASS
ADGRG6	6	142301854	142446266	0.033894706	PASS
ADGRG7	3	100609589	100695479	0.22470242	PASS
ADGRL1	19	14147743	14206187	-0.069222905	PASS
ADGRL2	1	81306160	81992436	0.587598296	PASS
ADGRL3	4	61201258	62078335	0.115781418	suppress_segment_too_large
ADGRL4	1	78889764	79006718	0.587598296	PASS
ADGRV1	5	90529344	91164437	-0.393084436	PASS
ADH1A	4	99276367	99291028	-0.677982248	PASS

ADH1B	4	99304964	99352760	-0.677982248	PASS
ADH1C	4	99336492	99353027	-0.677982248	PASS
ADH4	4	99123657	99157792	-0.677982248	PASS
ADH5	4	99070978	99088801	-0.677982248	PASS
ADH6	4	99202638	99219537	-0.677982248	PASS
ADH7	4	99412261	99435737	-0.677982248	PASS
ADHFE1	8	66432486	66468907	0.182873287	suppress_segment_too_large
ADI1	2	3497361	3519736	0.034263343	PASS
ADIG	20	38581195	38588463	-0.172056771	suppress_segment_too_large
ADIPOQ	3	186842690	186858463	0.02267528	PASS
ADIPOR1	1	202940823	202958572	-0.204996646	suppress_segment_too_large
ADIPOR2	12	1688574	1788678	-0.015029783	PASS
ADIRF	10	86968192	86983934	-0.461254852	PASS
ADK	10	74151185	74709303	0.032351597	PASS
ADM	11	10304680	10307397	-0.39835217	PASS
ADM2	22	50481556	50486440	-0.953096644	PASS
ADM5	19	49688664	49690575	0.002743503	PASS
ADNP	20	50888916	50931421	-0.172056771	suppress_segment_too_large
ADNP2	18	80109031	80147523	-0.330515409	PASS
ADO	10	62804857	62808483	0.032351597	PASS
ADORA1	1	203090654	203167405	-0.204996646	suppress_segment_too_large
ADORA2A	22	24417879	24442360	-0.740245994	PASS
ADORA2E	17	15944917	15975746	0.06854245	PASS
ADORA3	1	111499429	111503760	0.079255572	PASS
ADPGK	15	72751369	72785846	-0.759380635	PASS
ADPRH	3	119579268	119589945	0.06434429	PASS
ADPRHL1	13	113399610	113453524	-0.426822582	PASS
ADPRHL2	1	36088875	36093932	0.048749694	PASS
ADPRM	17	10697594	10711233	0.06854245	PASS
ADRA1A	8	26748150	26867273	-0.311940419	PASS
ADRA1B	5	159865080	159972544	0.165341871	suppress_segment_too_large
ADRA1D	20	4220631	4249074	-0.172056771	suppress_segment_too_large
ADRA2A	10	111077163	111080907	0.073090211	PASS
ADRA2B	2	96112875	96116245	0.064122364	PASS
ADRA2C	4	3766348	3768526	-0.358623662	PASS
ADRB1	10	114044056	114046908	0.073090211	PASS
ADRB2	5	148825245	148828687	-0.393084436	PASS
ADRB3	8	37962991	37966965	0.182873287	suppress_segment_too_large
ADRM1	20	62302093	62308862	-0.172056771	suppress_segment_too_large
ADSL	22	40346500	40390463	-0.740245994	PASS
ADSS	1	244408494	244452134	-0.145670469	suppress_likely_unfocal_large_gain
ADSSL1	14	104724186	104747325	-0.155273506	suppress_segment_too_large
ADTRP	6	11712054	11807046	0.069043418	PASS
AEBP1	7	44104361	44114562	-0.72421718	PASS
AEBP2	12	19404045	19720801	0.49032213	suppress_segment_too_large
AEN	15	88621296	88632282	-0.759380635	PASS
AES	19	3052910	3063107	-0.071973357	PASS
AF241726.	23	37349330	38687674	0.158844052	PASS
AFAP1	4	7758714	7939926	-0.358623662	PASS
AFAP1L1	5	149271871	149341802	-0.393084436	PASS
AFAP1L2	10	114294824	114404756	0.073090211	PASS
AFDN	6	167826922	167972023	0.033894706	PASS
AFF1	4	86935002	87141054	-0.677982248	PASS
AFF2	23	148500619	149000663	0.158844052	PASS

AFF3	2	99545419	100142739	0.064122364	PASS
AFF4	5	132875379	132963634	-0.393084436	PASS
AFG1L	6	108294894	108526796	-0.355603029	PASS
AFG3L2	18	12328944	12377314	0.044197172	PASS
AFM	4	73481683	73504001	0.115781418	suppress_segment_too_large
AFMID	17	78187317	78207701	0.051798665	PASS
AFP	4	73431138	73456174	0.115781418	suppress_segment_too_large
AFTPH	2	64524305	64593005	-0.46122572	PASS
AGA	4	177430770	177442503	0.142431244	suppress_segment_too_large
AGAP1	2	235494089	236131800	0.011001208	PASS
AGAP2	12	57723761	57742157	-0.743220499	PASS
AGAP3	7	151085831	151144436	-0.712149877	PASS
AGAP4	10	45825594	45853875	-0.389033546	PASS
AGAP5	10	73674285	73698159	0.032351597	PASS
AGAP6	10	49982190	50010499	-0.389033546	PASS
AGAP9	10	47501854	47523638	-0.389033546	PASS
AGBL1	15	86079973	87029052	-0.759380635	PASS
AGBL2	11	47659591	47715389	0.059795455	PASS
AGBL3	7	134986508	135147963	-0.712149877	PASS
AGBL4	1	48532855	50023913	-0.736943052	PASS
AGBL5	2	27042364	27070622	0.034263343	PASS
AGER	6	32180968	32184324	0.028532667	PASS
AGFG1	2	227472152	227561214	0.011001208	PASS
AGFG2	7	100539211	100568219	-0.710463272	PASS
AGGF1	5	77029251	77065234	-0.393084436	PASS
AGK	7	141551189	141655244	-0.712149877	PASS
AGL	1	99850084	99924023	0.079255572	PASS
AGMAT	1	15572353	15585110	0.048749694	PASS
AGMO	7	15200318	15562015	-0.72421718	PASS
AGO1	1	35869808	35930528	0.048749694	PASS
AGO2	8	140520156	140635619	0.493344802	suppress_segment_too_large
AGO3	1	35930718	36072500	0.048749694	PASS
AGO4	1	35808172	35857890	0.048749694	PASS
AGPAT1	6	32168212	32178096	0.028532667	PASS
AGPAT2	9	136673143	136687423	0.073960965	PASS
AGPAT3	21	43865186	43986536	0.209983559	PASS
AGPAT4	6	161129979	161274061	0.033894706	PASS
AGPAT5	8	6708357	6759666	-0.311940419	PASS
AGPS	2	177392644	177559299	0.033541467	PASS
AGR2	7	16791811	16833433	-0.72421718	PASS
AGR3	7	16859405	16881987	-0.72421718	PASS
AGRN	1	1020123	1056118	-0.358281697	PASS
AGRP	16	67482571	67483813	-0.423061015	PASS
AGT	1	230702523	230714297	-0.204996646	suppress_segment_too_large
AGTPBP1	9	85546539	85742029	0.073960965	PASS
AGTR1	3	148697784	148743008	0.02267528	PASS
AGTR2	23	116170722	116174972	0.158844052	PASS
AGTRAP	1	11736084	11754802	0.364796772	PASS
AGXT	2	240868479	240880502	0.011001208	PASS
AGXT2	5	34998101	35048135	0.073527871	PASS
AHCTF1	1	246839098	246931967	-0.145670469	suppress_likely_unfocal_large_gain
AHCY	20	34280268	34311802	-0.172056771	suppress_segment_too_large
AHCYL1	1	109984686	110023741	0.079255572	PASS
AHCYL2	7	129225023	129430211	-0.712149877	PASS

AHDC1	1	27534035	27604431	0.048749694	PASS
AHI1	6	135283532	135497776	0.033894706	PASS
AHNAK	11	62433542	62556235	0.064535884	PASS
AHNAK2	14	104937244	104978357	-0.155273506	suppress_segment_too_large
AHR	7	16916359	17346152	-0.72421718	PASS
AHRR	5	304176	438291	0.073527871	PASS
AHSA1	14	77457870	77469474	-0.155273506	suppress_segment_too_large
AHSG	3	186612923	186621318	0.02267528	PASS
AHSP	16	31527864	31528803	0.053595371	PASS
AICDA	12	8602166	8612871	0.505438545	PASS
AIDA	1	222668013	222713210	-0.204996646	suppress_segment_too_large
AIF1	6	31615184	31617021	0.028532667	PASS
AIF1L	9	131096476	131123152	0.073960965	PASS
AIFM1	23	130129362	130165887	0.158844052	PASS
AIFM2	10	70098223	70132934	0.032351597	PASS
AIFM3	22	20965108	20981360	-0.740245994	PASS
AIG1	6	143060496	143340304	0.033894706	PASS
AIM2	1	159062484	159147096	0.088103245	PASS
AIMP1	4	106315544	106349226	-0.677982248	PASS
AIMP2	7	6009245	6023834	-0.72421718	PASS
AIP	11	67483041	67491103	0.064535884	PASS
AIPL1	17	6393693	6435199	0.06854245	PASS
AIRE	21	44285838	44298648	0.209983559	PASS
AJAP1	1	4654732	4792534	-0.358281697	PASS
AJM1	9	136844415	136848801	0.073960965	PASS
AJUBA	14	22971174	22982642	0.053252507	PASS
AK1	9	127866480	127877743	0.073960965	PASS
AK2	1	33007940	33080996	0.048749694	PASS
AK3	9	4709559	4742043	0.073960965	PASS
AK4	1	65147549	65232145	0.052232535	PASS
AK5	1	77282051	77559969	0.052232535	PASS
AK6	5	69350984	69370013	-0.393084436	PASS
AK7	14	96392111	96489427	-0.155273506	suppress_segment_too_large
AK8	9	132725578	132878777	0.073960965	PASS
AK9	6	109492856	109691217	-0.355603029	PASS
AKAIN1	18	5145285	5197503	0.044197172	PASS
AKAP1	17	57085092	57121349	0.051798665	PASS
AKAP10	17	19904302	19978343	0.06854245	PASS
AKAP11	13	42272153	42323267	-0.398748891	PASS
AKAP12	6	151239999	151358557	0.033894706	PASS
AKAP13	15	85380571	85749358	-0.759380635	PASS
AKAP14	23	119895837	119920716	0.158844052	PASS
AKAP17A	23	1591593	1602514	-0.350651546	PASS
AKAP2	9	110048598	110172512	0.073960965	PASS
AKAP3	12	4615508	4649047	-0.015029783	PASS
AKAP4	23	50190768	50201013	0.158844052	PASS
AKAP5	14	64465499	64474503	0.053252507	PASS
AKAP6	14	32329273	32837681	0.053252507	PASS
AKAP7	6	131135666	131283535	-0.304168115	PASS
AKAP8	19	15353385	15379798	-0.069222905	PASS
AKAP8L	19	15380048	15419141	-0.069222905	PASS
AKAP9	7	91940867	92110673	-0.710463272	PASS
AKIP1	11	8911139	8920084	-0.39835217	PASS
AKIRIN1	1	38991223	39006059	0.048749694	PASS

AKIRIN2	6	87675072	87702209	-0.355603029	PASS
AKNA	9	114334156	114394405	0.073960965	PASS
AKNAD1	1	108815898	108858228	0.079255572	PASS
AKR1A1	1	45550543	45570049	-0.736943052	PASS
AKR1B1	7	134442350	134459284	-0.712149877	PASS
AKR1B10	7	134527592	134541408	-0.712149877	PASS
AKR1B15	7	134549136	134579875	-0.712149877	PASS
AKR1C1	10	4963253	4983283	0.075711544	PASS
AKR1C2	10	4987400	5018031	0.075711544	PASS
AKR1C3	10	5035354	5107686	0.075711544	PASS
AKR1C4	10	5195462	5218949	0.075711544	PASS
AKR1D1	7	138002324	138117986	-0.712149877	PASS
AKR1E2	10	4786629	4848062	0.075711544	PASS
AKR7A2	1	19303965	19312146	0.048749694	PASS
AKR7A3	1	19282558	19289250	0.048749694	PASS
AKT1	14	104769349	104795751	-0.155273506	suppress_segment_too_large
AKT1S1	19	49869033	49878459	0.002743503	PASS
AKT2	19	40230317	40285536	0.048917043	PASS
AKT3	1	243488233	243851079	-0.287484925	PASS
AKTIP	16	53491040	53504411	-0.423061015	PASS
AL020996.	1	25811470	25823744	0.048749694	PASS
AL021546.	12	120438198	120460006	0.028722739	PASS
AL021997.	6	28267121	28281580	0.069043418	PASS
AL022238.	22	40346529	40410054	-0.740245994	PASS
AL022318.	22	39014363	39033276	-0.740245994	PASS
AL024498.	6	10747794	10930423	0.069043418	PASS
AL031281.	1	22025511	22101360	0.048749694	PASS
AL031315.	6	169419969	169702047	0.033894706	PASS
AL031708.	16	1632259	1686715	0.053595371	PASS
AL031777.	6	26195595	26199293	0.069043418	PASS
AL031847.	1	6159430	6197757	0.364796772	PASS
AL033529.	1	32465057	32600792	0.048749694	PASS
AL034430.	20	10413520	10431922	-0.172056771	suppress_segment_too_large
AL034430.	20	10420546	10420737	-0.172056771	suppress_segment_too_large
AL035078.	11	31812391	32104665	-0.359426047	PASS
AL035425.	23	108722070	108732687	0.158844052	PASS
AL035460.	20	2814968	2818066	-0.172056771	suppress_segment_too_large
AL049557.	1	111722064	111755824	0.079255572	PASS
AL049629.	11	33698261	33722467	-0.359426047	PASS
AL049634.	20	1540144	1620009	-0.172056771	suppress_segment_too_large
AL049650.	20	2467212	2508907	-0.172056771	suppress_segment_too_large
AL049697.	6	87408012	87511634	-0.355603029	PASS
AL049779.	14	67659820	67690367	0.053252507	PASS
AL049839.	14	94592058	94624646	-0.155273506	suppress_segment_too_large
AL049844.	6	143807092	143844617	0.033894706	PASS
AL049844.	6	143857318	143938471	0.033894706	PASS
AL080251.	1	18849273	18921121	0.048749694	PASS
AL096711.	6	127438406	127519001	-0.304168115	PASS
AL096814.	6	42155426	42163439	0.028532667	PASS
AL096870.	14	24332310	24337382	0.053252507	PASS
AL109811.	1	11012662	11030528	0.364796772	PASS
AL109827.	20	35632340	35674544	-0.172056771	suppress_segment_too_large
AL110118.	14	93184973	93218586	-0.155273506	suppress_segment_too_large
AL117339.	10	38094368	38360098	0.075711544	PASS

AL117348.	1	225886696	225924278	-0.204996646	suppress_segment_too_large
AL121594.	14	35122549	35317474	0.053252507	PASS
AL121722.	20	22560553	22584261	-0.172056771	suppress_segment_too_large
AL121753.	20	35267885	35280043	-0.172056771	suppress_segment_too_large
AL121758.	20	646626	675800	-0.172056771	suppress_segment_too_large
AL121845.	20	63738270	63740398	-0.172056771	suppress_segment_too_large
AL121845.	20	63708864	63739103	-0.172056771	suppress_segment_too_large
AL121900.	20	18567453	18744216	-0.172056771	suppress_segment_too_large
AL132671.	6	117318211	117573571	-0.355603029	PASS
AL132780.	14	22946270	22982258	0.053252507	PASS
AL133352.	10	100505628	100529881	-0.414937136	PASS
AL133353.	10	99651595	99732112	-0.414937136	PASS
AL133410.	9	35812960	35828732	0.073960965	PASS
AL133500.	23	72301691	72572843	0.158844052	PASS
AL135905.	6	63572012	63583587	0.239805577	suppress_segment_too_large
AL136295.	14	24189157	24213473	0.053252507	PASS
AL136295.	14	24120956	24131829	0.053252507	PASS
AL136295.	14	24180395	24190416	0.053252507	PASS
AL136295.	14	24151218	24167402	0.053252507	PASS
AL136373.	1	46532166	46543969	-0.736943052	PASS
AL136454.	1	192716132	192716653	0.088103245	PASS
AL136531.	20	1317571	1393096	-0.172056771	suppress_segment_too_large
AL138752.	9	37588413	38068687	0.073960965	PASS
AL139011.	1	160216800	160285130	0.088103245	PASS
AL139142.	1	173596060	173669837	0.088103245	PASS
AL139300.	14	103562962	103685924	-0.155273506	suppress_segment_too_large
AL139353.	14	31334312	31457441	0.053252507	PASS
AL157392.	10	13610047	13655929	0.075711544	PASS
AL157935.	9	127867850	127906022	0.073960965	PASS
AL159163.	6	166858094	166956124	0.033894706	PASS
AL160269.	9	96235331	96352058	0.073960965	PASS
AL160272.	9	117704157	117877478	0.073960965	PASS
AL162231.	9	34664163	34666112	0.073960965	PASS
AL162231.	9	34646645	34657113	0.073960965	PASS
AL162377.	13	51763951	51845159	-0.384313056	PASS
AL162417.	9	133098121	133163914	0.073960965	PASS
AL163195.	14	20582892	20609795	0.053252507	PASS
AL163636.	14	20684587	20700576	0.053252507	PASS
AL353588.	6	44273194	44378957	0.028532667	PASS
AL355102.	14	96204844	96263929	-0.155273506	suppress_segment_too_large
AL355312.	6	149817937	149896011	0.033894706	PASS
AL355315.	10	97584374	97673910	-0.414937136	PASS
AL355916.	14	61529128	61657964	0.053252507	PASS
AL355987.	9	136738167	136748453	0.073960965	PASS
AL355987.	9	136791379	136800595	0.073960965	PASS
AL357673.	1	54132686	54200073	-0.736943052	PASS
AL358075.	1	46043661	46176488	-0.736943052	PASS
AL358113.	9	69035750	69255187	0.073960965	PASS
AL358472.	1	153975850	153986246	0.088103245	PASS
AL358472.	1	153959151	153975887	0.088103245	PASS
AL359736.	13	23979700	24321031	-0.398748891	PASS
AL359922.	9	21802636	22032986	0.073960965	PASS
AL360181.	10	133390834	133420495	0.073090211	PASS
AL365205.	6	41780349	41790141	0.028532667	PASS

AL365214.	6	112236806	112350094	-0.355603029	PASS
AL365232.	6	73209746	73263196	-0.250122709	PASS
AL365273.	10	95860548	95924844	-0.414937136	PASS
AL391650.	1	26169516	26171831	0.048749694	PASS
AL445238.	13	63832361	63833531	-0.384313056	PASS
AL445685.	1	52020153	52033350	-0.736943052	PASS
AL445989.	13	63746741	63751080	-0.384313056	PASS
AL449266.	1	108837660	108963484	0.079255572	PASS
AL451062.	1	43650149	43931014	-0.736943052	PASS
AL512428.	6	24797373	24809798	0.069043418	PASS
AL512506.	13	43883800	44028526	-0.398748891	PASS
AL512785.	1	162365407	162383531	0.088103245	PASS
AL513165.	9	37512547	37592469	0.073960965	PASS
AL583836.	10	94688154	94853073	-0.414937136	PASS
AL589666.	6	85557978	85615234	-0.355603029	PASS
AL590132.	1	210858125	211134146	-0.204996646	suppress_segment_too_large
AL590764.	23	71103987	71111575	0.158844052	PASS
AL591806.	1	160997957	161038962	0.088103245	PASS
AL592490.	13	61409858	61427849	-0.384313056	PASS
AL603832.	1	112702614	112711433	0.079255572	PASS
AL645922.	6	31927698	31952048	0.028532667	PASS
AL645941.	6	32937364	32953122	0.028532667	PASS
AL662820.	6	33321386	33329286	0.028532667	PASS
AL662884.	6	32150495	32154373	0.028532667	PASS
AL662899.	6	31686962	31714072	0.028532667	PASS
AL662899.	6	31666102	31673546	0.028532667	PASS
AL669918.	6	32813767	32838822	0.028532667	PASS
AL672043.	1	46175486	46176478	-0.736943052	PASS
AL672142.	9	128941478	128957021	0.073960965	PASS
AL691442.	1	155063748	155086807	0.088103245	PASS
AL713999.	1	155169409	155187272	0.088103245	PASS
AL772284.	23	119073226	119076373	0.158844052	PASS
AL807752.	9	137031241	137040436	0.073960965	PASS
AL807752.	9	136977518	136985435	0.073960965	PASS
AL845331.	2	94588522	94604573	0.064122364	PASS
AL928654.	14	105487199	105492267	-0.155273506	suppress_segment_too_large
AL929554.	9	137168854	137171984	0.073960965	PASS
ALAD	9	113386317	113401333	0.073960965	PASS
ALAS1	3	52198086	52214327	-0.727808231	PASS
ALAS2	23	55009055	55031064	0.158844052	PASS
ALB	4	73397114	73421412	0.115781418	suppress_segment_too_large
ALCAM	3	105366909	105576900	0.22470242	PASS
ALDH16A	19	49453169	49471048	0.002743503	PASS
ALDH18A	10	95605929	95656706	-0.414937136	PASS
ALDH1A1	9	72900662	73080442	0.073960965	PASS
ALDH1A2	15	57953424	58497866	-0.759380635	PASS
ALDH1A3	15	100877714	100916626	-0.759380635	PASS
ALDH1B1	9	38392664	38398661	0.073960965	PASS
ALDH1L1	3	126103562	126197994	0.06434429	PASS
ALDH1L2	12	105019784	105084577	0.028722739	PASS
ALDH2	12	111766887	111817529	0.028722739	PASS
ALDH3A1	17	19737984	19748943	0.06854245	PASS
ALDH3A2	17	19648136	19677598	0.06854245	PASS
ALDH3B1	11	68008578	68029282	0.064535884	PASS

ALDH3B2	11	67662162	67681200	0.064535884	PASS
ALDH4A1	1	18871430	18902781	0.048749694	PASS
ALDH5A1	6	24494852	24537207	0.069043418	PASS
ALDH6A1	14	74056850	74084493	0.053252507	PASS
ALDH7A1	5	126531200	126595418	-0.393084436	PASS
ALDH8A1	6	134917390	134950122	0.033894706	PASS
ALDH9A1	1	165662216	165698863	0.088103245	PASS
ALDOA	16	30064164	30070457	0.053595371	PASS
ALDOB	9	101420578	101449664	0.073960965	PASS
ALDOC	17	28573115	28577264	0.041294512	PASS
ALG1	16	5033923	5087379	0.053595371	PASS
ALG10	12	34022281	34029694	0.49032213	suppress_segment_too_large
ALG10B	12	38316578	38329728	0.49032213	suppress_segment_too_large
ALG11	13	52012398	52032171	-0.384313056	PASS
ALG12	22	49900229	49918458	-0.953096644	PASS
ALG13	23	111665811	111760649	0.158844052	PASS
ALG14	1	94974407	95072945	0.079255572	PASS
ALG1L	3	125929275	125937039	0.06434429	PASS
ALG1L2	3	130081831	130113227	0.06434429	PASS
ALG2	9	99216426	99221956	0.073960965	PASS
ALG3	3	184242301	184249548	0.02267528	PASS
ALG5	13	36949775	37000261	-0.398748891	PASS
ALG6	1	63367575	63438562	0.052232535	PASS
ALG8	11	78100936	78139660	0.064535884	PASS
ALG9	11	111782195	111871581	-0.431650509	PASS
ALK	2	29192774	29921566	0.034263343	PASS
ALKAL1	8	52534037	52565507	0.182873287	suppress_segment_too_large
ALKAL2	2	279558	288851	0.034263343	PASS
ALKBH1	14	77672404	77708020	-0.155273506	suppress_segment_too_large
ALKBH2	12	109088188	109093631	0.028722739	PASS
ALKBH3	11	43880811	43920266	0.059795455	PASS
ALKBH4	7	102456238	102464876	-0.712149877	PASS
ALKBH5	17	18183078	18209954	0.06854245	PASS
ALKBH6	19	36009120	36014239	0.044838622	PASS
ALKBH7	19	6372433	6375251	-0.071973357	PASS
ALKBH8	11	107502726	107565746	-0.431650509	PASS
ALLC	2	3658195	3702671	0.034263343	PASS
ALMS1	2	73385758	73610793	-0.46122572	PASS
ALOX12	17	6996065	7010736	0.06854245	PASS
ALOX12B	17	8072636	8087716	0.06854245	PASS
ALOX15	17	4630902	4642294	0.06854245	PASS
ALOX15B	17	8039017	8049134	0.06854245	PASS
ALOX5	10	45374176	45446119	-0.389033546	PASS
ALOX5AP	13	30713478	30764426	-0.398748891	PASS
ALOXE3	17	8095900	8119047	0.06854245	PASS
ALPG	2	232406843	232410714	0.011001208	PASS
ALPI	2	232456123	232460032	0.011001208	PASS
ALPK1	4	112285509	112442620	-0.677982248	PASS
ALPK2	18	58481247	58628957	-0.330515409	PASS
ALPK3	15	84816680	84873482	-0.759380635	PASS
ALPL	1	21509372	21578412	0.048749694	PASS
ALPP	2	232378534	232382889	0.011001208	PASS
ALS2	2	201700554	201781189	0.011001208	PASS
ALS2CL	3	46668997	46693704	-0.727808231	PASS

ALS2CR12	2	201288271	201357398		0.011001208	PASS
ALX1	12	85280107	85301784		0.271127429	suppress_segment_too_large
ALX3	1	110059994	110070700		0.079255572	PASS
ALX4	11	44260440	44310166		0.059795455	PASS
ALYREF	17	81887844	81891586		0.051798665	PASS
AMACR	5	33986178	34008108		0.073527871	PASS
AMBN	4	70592256	70607288		0.115781418	suppress_segment_too_large
AMBP	9	114060127	114078472		0.073960965	PASS
AMBRA1	11	46396414	46594125		0.059795455	PASS
AMD1	6	110874770	110895713		-0.355603029	PASS
AMDHD1	12	95943293	95968716		0.028722739	PASS
AMDHD2	16	2520357	2531422		0.053595371	PASS
AMELX	23	11293413	11300761		0.158844052	PASS
AMER1	23	64185117	64205744		0.158844052	PASS
AMER2	13	25161684	25172288		-0.398748891	PASS
AMER3	2	130755435	130768134		-0.359440172	PASS
AMFR	16	56361452	56425538		-0.423061015	PASS
AMH	19	2249309	2252073		-0.071973357	PASS
AMHR2	12	53423855	53431534		-0.743220499	PASS
AMIGO1	1	109504175	109509738		0.079255572	PASS
AMIGO2	12	47075707	47079951		-0.46813528	PASS
AMIGO3	3	49716834	49719695		-0.727808231	PASS
AMMECR	23	110194186	110440233		0.158844052	PASS
AMMECR	2	127861630	127885922		-0.359440172	PASS
AMN	14	102922656	102933596		-0.155273506	suppress_segment_too_large
AMN1	12	31671137	31729174		0.49032213	suppress_segment_too_large
AMOT	23	112774503	112840815		0.158844052	PASS
AMOTL1	11	94706431	94876753		0.064535884	PASS
AMOTL2	3	134355874	134375479		0.06434429	PASS
AMPD1	1	114673090	114695618		0.079255572	PASS
AMPD2	1	109616104	109632051		0.079255572	PASS
AMPD3	11	10308313	10507579		-0.39835217	PASS
AMPH	7	38383704	38631567		-0.72421718	PASS
AMT	3	49416775	49422753		-0.727808231	PASS
AMTN	4	70518540	70532742		0.115781418	suppress_segment_too_large
AMY1A	1	103655290	103664554		0.079255572	PASS
AMY1C	1	103750406	103758690		0.079255572	PASS
AMY2A	1	103616811	103625780		0.079255572	PASS
AMY2B	1	103553815	103579534		0.079255572	PASS
AMZ1	7	2679522	2775500		-0.72421718	PASS
AMZ2	17	68247574	68257164		0.051798665	PASS
ANAPC1	2	111611639	111884690		0.040971718	PASS
ANAPC10	4	144831908	145098541		0.142431244	suppress_segment_too_large
ANAPC11	17	81890790	81900991		0.051798665	PASS
ANAPC13	3	134477706	134486716		0.06434429	PASS
ANAPC15	11	72106378	72112780		0.064535884	PASS
ANAPC16	10	72216000	72235860		0.032351597	PASS
ANAPC2	9	137174784	137188549		0.073960965	PASS
ANAPC4	4	25377213	25418498		-0.358623662	PASS
ANAPC5	12	121308245	121399896		0.028722739	PASS
ANAPC7	12	110372900	110403730		0.028722739	PASS
ANG	14	20684177	20698971		0.053252507	PASS
ANGEL1	14	76786178	76826246		-0.155273506	suppress_segment_too_large
ANGEL2	1	212992182	213015826		-0.204996646	suppress_segment_too_large

ANGPT1	8	107249482	107498055	0.70944438	suppress_segment_too_large
ANGPT2	8	6499651	6563409	-0.311940419	PASS
ANGPT4	20	869899	916317	-0.172056771	suppress_segment_too_large
ANGPTL1	1	178849705	178871052	0.088103245	PASS
ANGPTL2	9	127087332	127122883	0.073960965	PASS
ANGPTL3	1	62597487	62606159	0.052232535	PASS
ANGPTL4	19	8363289	8374373	-0.071973357	PASS
ANGPTL5	11	101890674	101916522	0.32805343	suppress_segment_too_large
ANGPTL6	19	10092338	10102796	-0.107594294	PASS
ANGPTL7	1	11189341	11195981	0.364796772	PASS
ANGPTL8	19	11237450	11241943	-0.107594294	PASS
ANHX	12	133218312	133236095	0.232349073	PASS
ANK1	8	41653220	41896762	0.182873287	suppress_segment_too_large
ANK2	4	112818109	113383740	-0.677982248	PASS
ANK3	10	60026298	60733490	0.032351597	PASS
ANKAR	2	189674290	189761193	0.011001208	PASS
ANKDD1A	15	64911902	64958700	-0.759380635	PASS
ANKDD1E	5	75611459	75671846	-0.393084436	PASS
ANKEF1	20	9986126	10058303	-0.172056771	suppress_segment_too_large
ANKFN1	17	55882301	56511659	0.051798665	PASS
ANKFY1	17	4163907	4263995	0.06854245	PASS
ANKH	5	14704800	14871785	0.073527871	PASS
ANKHD1	5	140401814	140539856	-0.393084436	PASS
ANKHD1-	5	140401908	140549569	-0.393084436	PASS
ANKIB1	7	92246234	92401384	-0.710463272	PASS
ANKK1	11	113387791	113400418	-0.431650509	PASS
ANKLE1	19	17281645	17287646	-0.069222905	PASS
ANKLE2	12	132725503	132761888	0.232349073	PASS
ANKMY1	2	240479422	240569209	0.011001208	PASS
ANKMY2	7	16599776	16645817	-0.72421718	PASS
ANKRA2	5	73552335	73565686	-0.393084436	PASS
ANKRD1	10	90912096	90921276	-0.414937136	PASS
ANKRD10	13	110878540	110915069	-0.426822582	PASS
ANKRD11	16	89267619	89490561	-0.423061015	PASS
ANKRD12	18	9136228	9285985	0.044197172	PASS
ANKRD13	12	109999186	110039763	0.028722739	PASS
ANKRD13	17	29589769	29614761	0.041294512	PASS
ANKRD13	1	70260588	70354734	0.052232535	PASS
ANKRD13	11	67288547	67302485	0.064535884	PASS
ANKRD16	10	5861617	5889906	0.075711544	PASS
ANKRD17	4	73073376	73258798	0.115781418	suppress_segment_too_large
ANKRD18	9	38571358	38620660	0.073960965	PASS
ANKRD18	9	33524394	33573009	0.073960965	PASS
ANKRD2	10	97572499	97583884	-0.414937136	PASS
ANKRD20	9	67859147	67902094	0.073960965	PASS
ANKRD20	9	40223285	40266392	0.073960965	PASS
ANKRD20	9	64369394	64413142	0.073960965	PASS
ANKRD22	10	88822132	88851818	-2.417109027	suppress_large_homdel
ANKRD23	2	96824526	96857934	0.064122364	PASS
ANKRD24	19	4183354	4224814	-0.071973357	PASS
ANKRD26	10	26991914	27100498	0.075711544	PASS
ANKRD27	19	32597007	32676597	0.044838622	PASS
ANKRD28	3	15667236	15859771	0.06149513	PASS
ANKRD29	18	23598926	23662885	-0.330515409	PASS

ANKRD30	10	37125788	37384111	0.075711544	PASS
ANKRD30	18	14728272	14852738	0.044197172	PASS
ANKRD30	2	132147591	132257969	-0.359440172	PASS
ANKRD31	5	75068275	75236878	-0.393084436	PASS
ANKRD33	12	51887960	51891664	-0.743220499	PASS
ANKRD33	5	10564330	10657816	0.073527871	PASS
ANKRD34	1	145959442	145964582	0.079255572	PASS
ANKRD34	5	80556755	80570488	-0.393084436	PASS
ANKRD34	15	79293285	79298235	-0.759380635	PASS
ANKRD35	1	145866560	145885866	0.079255572	PASS
ANKRD36	2	97113496	97250199	0.064122364	PASS
ANKRD36	2	97492663	97589965	0.064122364	PASS
ANKRD36	2	95836919	95991831	0.064122364	PASS
ANKRD37	4	185396021	185400628	0.275137421	PASS
ANKRD39	2	96836611	96858095	0.064122364	PASS
ANKRD40	17	50693190	50707924	0.051798665	PASS
ANKRD42	11	83193739	83260694	0.064535884	PASS
ANKRD44	2	196967017	197311173	0.011001208	PASS
ANKRD45	1	173609561	173669862	0.088103245	PASS
ANKRD46	8	100509752	100559784	0.355469066	suppress_segment_too_large
ANKRD49	11	94493629	94499583	0.064535884	PASS
ANKRD50	4	124664052	124712732	0.142431244	suppress_segment_too_large
ANKRD52	12	56237807	56258391	-0.743220499	PASS
ANKRD53	2	70978380	70985499	-0.46122572	PASS
ANKRD54	22	37830855	37849327	-0.740245994	PASS
ANKRD55	5	56099678	56233359	-0.284712224	PASS
ANKRD6	6	89433170	89633834	-0.355603029	PASS
ANKRD60	20	58218495	58228653	-0.172056771	suppress_segment_too_large
ANKRD61	7	6031376	6036386	-0.72421718	PASS
ANKRD62	18	12093853	12129749	0.044197172	PASS
ANKRD63	15	40281444	40282586	-0.423772929	PASS
ANKRD65	1	1418420	1421769	-0.358281697	PASS
ANKRD66	6	46746917	46759506	0.239805577	suppress_segment_too_large
ANKRD7	7	118214669	118496171	-0.712149877	PASS
ANKRD9	14	102501760	102509799	-0.155273506	suppress_segment_too_large
ANKS1A	6	34889265	35091413	0.028532667	PASS
ANKS1B	12	98726457	99984654	0.028722739	PASS
ANKS3	16	4696510	4734378	0.053595371	PASS
ANKS4B	16	21233665	21253845	0.053595371	PASS
ANKS6	9	98731329	98796965	0.073960965	PASS
ANKUB1	3	149761100	149968385	0.02267528	PASS
ANKZF1	2	219229757	219236669	0.011001208	PASS
ANLN	7	36389806	36453791	-0.72421718	PASS
ANO1	11	70078302	70189528	0.064535884	PASS
ANO10	3	43354859	43691594	-0.727808231	PASS
ANO2	12	5531869	5946232	-0.015029783	PASS
ANO3	11	26309599	26663288	-0.359426047	PASS
ANO4	12	100717526	101128641	0.028722739	PASS
ANO5	11	21799934	22283357	-2.208422516	PASS
ANO6	12	45215987	45440404	-0.46813528	PASS
ANO7	2	241188509	241225377	0.011001208	PASS
ANO8	19	17323223	17334829	-0.069222905	PASS
ANO9	11	417933	442011	-0.39835217	PASS
ANOS1	23	8528874	8732186	0.158844052	PASS

ANP32A	15	68778535	68820897	-0.759380635	PASS
ANP32B	9	97983361	98015943	0.073960965	PASS
ANP32D	12	48472665	48473624	-0.743220499	PASS
ANP32E	1	150218417	150236156	0.079255572	PASS
ANPEP	15	89784889	89815401	-0.759380635	PASS
ANTXR1	2	69013178	69249327	-0.46122572	PASS
ANTXR2	4	79901149	80125454	-0.677982248	PASS
ANTXRL	10	46286345	46330207	-0.389033546	PASS
ANXA1	9	73151757	73170393	0.073960965	PASS
ANXA10	4	168092515	168187690	0.142431244	suppress_segment_too_large
ANXA11	10	80150889	80205572	0.032351597	PASS
ANXA13	8	123680794	123737407	0.493344802	suppress_segment_too_large
ANXA2	15	60347134	60402883	-0.759380635	PASS
ANXA2R	5	43039081	43043170	-0.284712224	PASS
ANXA3	4	78551519	78610451	-0.677982248	PASS
ANXA4	2	69644425	69827100	-0.46122572	PASS
ANXA5	4	121667955	121697113	0.142431244	suppress_segment_too_large
ANXA6	5	151100712	151157882	-0.393084436	PASS
ANXA7	10	73375101	73414076	0.032351597	PASS
ANXA8	10	47460162	47484158	-0.389033546	PASS
ANXA8L1	10	46375627	46391784	-0.389033546	PASS
ANXA9	1	150982017	150995634	0.079255572	PASS
AOAH	7	36512949	36724549	-0.72421718	PASS
AOC1	7	150824627	150861504	-0.712149877	PASS
AOC2	17	42844600	42850707	0.041294512	PASS
AOC3	17	42851184	42858130	0.041294512	PASS
AOX1	2	200585868	200677064	0.011001208	PASS
AP000275.	21	32578822	32612281	0.209983559	PASS
AP000295.	21	33229901	33297160	0.209983559	PASS
AP000311.	21	33584687	33931607	0.209983559	PASS
AP000350.	22	23862188	23895223	-0.740245994	PASS
AP000646.	11	121024125	121113108	-0.431650509	PASS
AP000721.	11	63974620	63988346	0.064535884	PASS
AP000781.	11	57387365	57424059	0.27006553	suppress_segment_too_large
AP000812.	11	72105924	72109329	0.064535884	PASS
AP000812.	11	72080777	72110782	0.064535884	PASS
AP000944.	11	65261928	65326543	0.064535884	PASS
AP001267.	11	118401602	118482737	-0.431650509	PASS
AP001273.	11	93741664	93812378	0.064535884	PASS
AP001458.	11	62649694	62665178	0.064535884	PASS
AP001781.	11	111786286	111879425	-0.431650509	PASS
AP001931.	11	57742163	57793243	0.27006553	suppress_segment_too_large
AP002373.	11	114400682	114443932	-0.431650509	PASS
AP002495.	11	71865509	71928654	0.064535884	PASS
AP002512.	11	56459221	56473352	0.27006553	suppress_segment_too_large
AP002748.	11	66509079	66533613	0.064535884	PASS
AP002884.	11	112086903	112193805	-0.431650509	PASS
AP002990.	11	62559603	62591531	0.064535884	PASS
AP003108.	11	61429220	61485822	0.064535884	PASS
AP003419.	11	67351572	67373584	0.064535884	PASS
AP1AR	4	112231737	112270047	-0.677982248	PASS
AP1B1	22	29327680	29423179	-0.740245994	PASS
AP1G1	16	71729000	71809201	-0.423061015	PASS
AP1G2	14	23559565	23568070	0.053252507	PASS

AP1M1	19	16197578	16245907	-0.069222905	PASS
AP1M2	19	10572671	10587315	-0.107594294	PASS
AP1S1	7	101154400	101161596	-0.712149877	PASS
AP1S2	23	15825806	15854931	0.158844052	PASS
AP1S3	2	223751686	223838027	0.011001208	PASS
AP2A1	19	49766968	49807113	0.002743503	PASS
AP2A2	11	924894	1012245	-0.39835217	PASS
AP2B1	17	35578046	35726409	0.041294512	PASS
AP2M1	3	184174689	184184091	0.02267528	PASS
AP2S1	19	46838136	46850992	0.002743503	PASS
AP3B1	5	78000525	78294755	-0.393084436	PASS
AP3B2	15	82659281	82709914	-0.759380635	PASS
AP3D1	19	2100988	2164468	-0.071973357	PASS
AP3M1	10	74120255	74151063	0.032351597	PASS
AP3M2	8	42152946	42171673	0.182873287	suppress_segment_too_large
AP3S1	5	115841481	115914081	-0.393084436	PASS
AP3S2	15	89830599	89894638	-0.759380635	PASS
AP4B1	1	113894748	113905201	0.079255572	PASS
AP4E1	15	50908672	51005900	-0.423772929	PASS
AP4M1	7	100101549	100110345	-0.710463272	PASS
AP4S1	14	31025106	31096450	0.053252507	PASS
AP5B1	11	65775893	65780802	0.064535884	PASS
AP5M1	14	57268909	57298742	0.053252507	PASS
AP5S1	20	3820524	3828837	-0.172056771	suppress_segment_too_large
AP5Z1	7	4775615	4794395	-0.72421718	PASS
APAF1	12	98645141	98735433	0.028722739	PASS
APBA1	9	69427530	69672306	0.073960965	PASS
APBA2	15	28884483	29118315	-0.423772929	PASS
APBA3	19	3750819	3761699	-0.071973357	PASS
APBB1	11	6395124	6419414	-0.39835217	PASS
APBB1IP	10	26438203	26567803	0.075711544	PASS
APBB2	4	40810027	41216714	-0.753159364	PASS
APBB3	5	140558268	140564781	-0.393084436	PASS
APC	5	112707498	112846239	-0.393084436	PASS
APC2	19	1446302	1473244	-0.071973357	PASS
APCDD1	18	10454628	10489948	0.044197172	PASS
APCDD1L	20	58459101	58515131	-0.172056771	suppress_segment_too_large
APCS	1	159587825	159588865	0.088103245	PASS
APEH	3	49674002	49683963	-0.727808231	PASS
APELA	4	164877004	164898965	0.142431244	suppress_segment_too_large
APEX1	14	20455191	20457772	0.053252507	PASS
APEX2	23	55000357	55009057	0.158844052	PASS
APH1A	1	150265399	150269580	0.079255572	PASS
APH1B	15	63276018	63309126	-0.759380635	PASS
API5	11	43311963	43344529	0.059795455	PASS
APIP	11	34853094	34916499	-0.359426047	PASS
APLF	2	68467561	68655862	-0.46122572	PASS
APLN	23	129645259	129654937	0.158844052	PASS
APLNR	11	57233577	57237314	0.27006553	suppress_segment_too_large
APLP1	19	35867899	35879791	0.044838622	PASS
APLP2	11	130068147	130144811	-0.431650509	PASS
APMAP	20	24962925	24992979	-0.172056771	suppress_segment_too_large
APOA1	11	116835751	116837950	-0.431650509	PASS
APOA2	1	161222292	161223631	0.088103245	PASS

APOA4	11	116820700	116823306	-0.431650509	PASS
APOA5	11	116789367	116792420	-0.431650509	PASS
APOB	2	21001429	21044073	0.034263343	PASS
APOBEC1	12	7649400	7665903	-0.015029783	PASS
APOBEC2	6	41053304	41064511	0.028532667	PASS
APOBEC3	22	38952741	38992778	-0.740245994	PASS
APOBEC3	22	38982347	38992804	-0.740245994	PASS
APOBEC3	22	39014083	39020352	-0.740245994	PASS
APOBEC3	22	39021113	39033276	-0.740245994	PASS
APOBEC3	22	39040604	39053910	-0.740245994	PASS
APOBEC3	22	39077005	39087743	-0.740245994	PASS
APOBEC3	22	39097224	39104067	-0.740245994	PASS
APOBEC4	1	183646404	183653316	0.088103245	PASS
APOBR	16	28494649	28498970	0.053595371	PASS
APOC1	19	44914247	44919349	0.002743503	PASS
APOC2	19	44945982	44949565	0.002743503	PASS
APOC3	11	116829706	116833072	-0.431650509	PASS
APOC4	19	44942238	44945496	0.002743503	PASS
APOC4-AI	19	44942238	44949565	0.002743503	PASS
APOD	3	195568702	195584205	0.02267528	PASS
APOE	19	44905754	44909393	0.002743503	PASS
APOF	12	56360569	56362823	-0.743220499	PASS
APOH	17	66212033	66256525	0.051798665	PASS
APOL1	22	36253010	36267530	-0.740245994	PASS
APOL2	22	36226203	36239954	-0.740245994	PASS
APOL3	22	36140330	36166177	-0.740245994	PASS
APOL4	22	36189124	36204840	-0.740245994	PASS
APOL5	22	35717872	35729483	-0.740245994	PASS
APOL6	22	35648395	35668409	-0.740245994	PASS
APOLD1	12	12725917	12829975	0.49032213	suppress_segment_too_large
APOM	6	31652416	31658210	0.028532667	PASS
APOO	23	23833353	23907934	0.158844052	PASS
APOOL	23	85003826	85093316	0.158844052	PASS
APOPT1	14	103562962	103607523	-0.155273506	suppress_segment_too_large
APP	21	25880550	26171128	0.209983559	PASS
APPBP2	17	60443149	60526219	0.051798665	PASS
APPL1	3	57227726	57278105	-0.727808231	PASS
APPL2	12	105173296	105236238	0.028722739	PASS
APRT	16	88809339	88811944	-0.423061015	PASS
APTX	9	32972606	33025168	0.073960965	PASS
AQP1	7	30911694	30925516	-0.72421718	PASS
AQP10	1	154321090	154325325	0.088103245	PASS
AQP11	11	77589391	77610355	0.064535884	PASS
AQP12A	2	240691845	240698483	0.011001208	PASS
AQP12B	2	240676418	240682906	0.011001208	PASS
AQP2	12	49950741	49958881	-0.743220499	PASS
AQP3	9	33441154	33447633	0.073960965	PASS
AQP4	18	26852038	26865818	-0.330515409	PASS
AQP5	12	49961870	49965681	-0.743220499	PASS
AQP6	12	49967194	49977139	-0.743220499	PASS
AQP7	9	33383179	33402682	0.073960965	PASS
AQP8	16	25215731	25228940	0.053595371	PASS
AQP9	15	58138169	58185911	-0.759380635	PASS
AQR	15	34851782	34969839	-0.423772929	PASS

AR	23	67544032	67730619	0.158844052	PASS
ARAF	23	47561100	47571920	0.158844052	PASS
ARAP1	11	72685069	72793599	0.064535884	PASS
ARAP2	4	35948221	36244509	-0.753159364	PASS
ARAP3	5	141653401	141682221	-0.393084436	PASS
ARC	8	142611044	142614472	0.699132336	PASS
ARCN1	11	118572390	118603033	-0.431650509	PASS
AREG	4	74445134	74455009	0.115781418	suppress_segment_too_large
AREL1	14	74653437	74713115	-0.155273506	suppress_segment_too_large
ARF1	1	228082660	228099212	-0.204996646	suppress_segment_too_large
ARF3	12	48935723	48957551	-0.743220499	PASS
ARF4	3	57571363	57598220	-0.727808231	PASS
ARF5	7	127588345	127591705	-0.712149877	PASS
ARF6	14	49893092	49897054	0.053252507	PASS
ARFGAP1	20	63272785	63289793	-0.172056771	suppress_segment_too_large
ARFGAP2	11	47164299	47177125	0.059795455	PASS
ARFGAP3	22	42796502	42858106	-0.740245994	PASS
ARFGEF1	8	67173511	67343677	0.182873287	suppress_segment_too_large
ARFGEF2	20	48921890	49036693	-0.172056771	suppress_segment_too_large
ARFGEF3	6	138161921	138344663	0.033894706	PASS
ARFIP1	4	152779937	152918463	0.142431244	suppress_segment_too_large
ARFIP2	11	6474683	6481479	-0.39835217	PASS
ARFRP1	20	63698642	63708025	-0.172056771	suppress_segment_too_large
ARG1	6	131573144	131584332	-0.304168115	PASS
ARG2	14	67619798	67651720	0.053252507	PASS
ARGFX	3	121570704	121586634	0.06434429	PASS
ARGLU1	13	106541673	106568164	-0.426822582	PASS
ARHGAP1	11	46677080	46700615	0.059795455	PASS
ARHGAP1	4	147732063	148072780	0.142431244	suppress_segment_too_large
ARHGAP1	15	32615144	32639949	-0.423772929	PASS
ARHGAP1	15	30624494	30638805	-0.423772929	PASS
ARHGAP1	10	31805404	31928876	0.075711544	PASS
ARHGAP1	2	143091362	143768352	-0.30549516	PASS
ARHGAP1	16	24919385	25015666	0.053595371	PASS
ARHGAP1	6	129576132	129710225	-0.304168115	PASS
ARHGAP1	10	97222173	97292673	-0.414937136	PASS
ARHGAP1	10	97153045	97292637	-0.414937136	PASS
ARHGAP2	11	110577042	110713189	-0.431650509	PASS
ARHGAP2	10	24583609	24723668	0.075711544	PASS
ARHGAP2	10	48446034	48656265	-0.389033546	PASS
ARHGAP2	17	38419280	38512392	0.041294512	PASS
ARHGAP2	4	85475114	86002670	-0.677982248	PASS
ARHGAP2	2	68679601	68826833	-0.46122572	PASS
ARHGAP2	5	142770377	143229011	-0.393084436	PASS
ARHGAP2	17	45393902	45434421	0.051798665	PASS
ARHGAP2	18	6729718	6915716	0.044197172	PASS
ARHGAP2	1	94148988	94275068	0.079255572	PASS
ARHGAP3	1	161046946	161069970	0.088103245	PASS
ARHGAP3	3	119294373	119420714	0.06434429	PASS
ARHGAP3	11	128965060	129279324	-0.431650509	PASS
ARHGAP3	19	35774532	35788822	0.044838622	PASS
ARHGAP3	19	46918676	47005077	0.002743503	PASS
ARHGAP3	23	131058242	131089883	0.158844052	PASS
ARHGAP3	8	144529179	144605816	0.699132336	PASS

ARHGAP4	23	153907367	153934999	0.158844052	PASS
ARHGAP4	20	38601934	38651035	-0.172056771	suppress_segment_too_large
ARHGAP4	11	100687653	100993941	0.32805343	suppress_segment_too_large
ARHGAP4	17	12789539	12991643	0.06854245	PASS
ARHGAP4	19	1065923	1086628	-0.071973357	PASS
ARHGAP5	14	32076114	32159728	0.053252507	PASS
ARHGAP6	23	11137543	11665701	0.158844052	PASS
ARHGAP8	22	44752558	44862788	-0.953096644	PASS
ARHGAP9	12	57472255	57488814	-0.743220499	PASS
ARHGDIA	17	81867721	81871406	0.051798665	PASS
ARHGDIB	12	14942017	14961728	0.49032213	suppress_segment_too_large
ARHGDIG	16	268727	283010	0.053595371	PASS
ARHGEF1	19	41883161	41930150	0.048917043	PASS
ARHGEF1	8	1823976	1958641	-0.311940419	PASS
ARHGEF1	1	17539835	17697874	0.048749694	PASS
ARHGEF1	1	156934840	157045370	0.088103245	PASS
ARHGEF1	11	120336914	120489936	-0.431650509	PASS
ARHGEF1	17	8310241	8322516	0.06854245	PASS
ARHGEF1	1	3454426	3481113	-0.358281697	PASS
ARHGEF1	11	73308289	73369388	0.064535884	PASS
ARHGEF1	19	7395113	7472477	-0.071973357	PASS
ARHGEF1	1	16197854	16212609	0.048749694	PASS
ARHGEF2	1	155946851	156007070	0.088103245	PASS
ARHGEF2	12	57610180	57619379	-0.743220499	PASS
ARHGEF2	3	154121003	154257827	0.02267528	PASS
ARHGEF2	5	73626158	73941993	-0.393084436	PASS
ARHGEF3	3	56727418	57079329	-0.727808231	PASS
ARHGEF3	2	38889841	38975449	0.034263343	PASS
ARHGEF3	5	149551947	149634968	-0.393084436	PASS
ARHGEF3	4	105552620	105708093	-0.677982248	PASS
ARHGEF3	9	35658875	35675866	0.073960965	PASS
ARHGEF4	2	130836916	131047263	-0.359440172	PASS
ARHGEF4	14	21070270	21090240	0.053252507	PASS
ARHGEF5	7	144355288	144380632	-0.712149877	PASS
ARHGEF6	23	136665547	136782088	0.158844052	PASS
ARHGEF7	13	111114559	111305737	-0.426822582	PASS
ARHGEF9	23	63634967	63809274	0.158844052	PASS
ARID1A	1	26693236	26782110	0.048749694	PASS
ARID1B	6	156776020	157210779	0.033894706	PASS
ARID2	12	45729665	45908040	-0.46813528	PASS
ARID3A	19	925781	975934	-0.071973357	PASS
ARID3B	15	74541177	74598131	-0.759380635	PASS
ARID3C	9	34621379	34628107	0.073960965	PASS
ARID4A	14	58298385	58373887	0.053252507	PASS
ARID4B	1	235131634	235328219	-0.204996646	suppress_segment_too_large
ARID5A	2	96536743	96552638	0.064122364	PASS
ARID5B	10	61901300	62096944	0.032351597	PASS
ARIH1	15	72474326	72602985	-0.759380635	PASS
ARIH2	3	48918821	48986382	-0.727808231	PASS
ARIH2OS	3	48917782	48919385	-0.727808231	PASS
ARL1	12	101393120	101407820	0.028722739	PASS
ARL10	5	176365468	176401865	0.165341871	suppress_segment_too_large
ARL11	13	49628299	49633872	-0.384313056	PASS
ARL13A	23	100969708	100990829	0.158844052	PASS

ARL13B	3	93980139	94055668	0.22470242	PASS
ARL14	3	160677159	160678452	0.02267528	PASS
ARL14EP	11	30323051	30338227	-0.359426047	PASS
ARL14EPI	5	116032324	116060118	-0.393084436	PASS
ARL15	5	53883945	54310582	-0.284712224	PASS
ARL16	17	81681174	81683924	0.051798665	PASS
ARL17A	17	46516702	46579682	0.051798665	PASS
ARL17B	17	46274784	46361797	0.051798665	PASS
ARL2	11	65014113	65022184	0.064535884	PASS
ARL2-SN)	11	65014182	65040570	0.064535884	PASS
ARL2BP	16	57245098	57253635	-0.423061015	PASS
ARL3	10	102673731	102714407	-0.414937136	PASS
ARL4A	7	12686856	12690934	-0.72421718	PASS
ARL4C	2	234493041	234497053	0.011001208	PASS
ARL4D	17	43398959	43401137	0.051798665	PASS
ARL5A	2	151788984	151828492	-0.30549516	PASS
ARL5B	10	18659405	18681639	0.075711544	PASS
ARL5C	17	39156894	39167484	0.041294512	PASS
ARL6	3	97764521	97801242	0.22470242	PASS
ARL6IP1	16	18791667	18801678	0.053595371	PASS
ARL6IP4	12	122980060	122982913	0.028722739	PASS
ARL6IP5	3	69084944	69106066	-0.727808231	PASS
ARL6IP6	2	152717893	152761253	-0.30549516	PASS
ARL8A	1	202133404	202144743	-0.204996646	suppress_segment_too_large
ARL8B	3	5122220	5180912	0.06149513	PASS
ARL9	4	56505209	56525481	0.115781418	suppress_segment_too_large
ARMC1	8	65602456	65634217	0.182873287	suppress_segment_too_large
ARMC10	7	103074881	103099764	-0.712149877	PASS
ARMC12	6	35737032	35749079	0.028532667	PASS
ARMC2	6	108848416	108974472	-0.355603029	PASS
ARMC3	10	22928024	23038523	0.075711544	PASS
ARMC4	10	27812164	27999048	0.075711544	PASS
ARMC5	16	31458080	31467166	0.053595371	PASS
ARMC6	19	19033575	19060311	-0.069222905	PASS
ARMC7	17	75109952	75130265	0.051798665	PASS
ARMC8	3	138187248	138298389	0.06434429	PASS
ARMC9	2	231198546	231374837	0.011001208	PASS
ARMCX1	23	101550531	101554700	0.158844052	PASS
ARMCX2	23	101655281	101659891	0.158844052	PASS
ARMCX3	23	101622797	101627843	0.158844052	PASS
ARMCX4	23	101418287	101533459	0.158844052	PASS
ARMCX5	23	102599168	102604159	0.158844052	PASS
ARMCX6	23	101615118	101618001	0.158844052	PASS
ARMH1	1	44674692	44725591	-0.736943052	PASS
ARMH2	6	24797321	24798889	0.069043418	PASS
ARMH3	10	101845599	102056193	-0.414937136	PASS
ARMH4	14	57999735	58298139	0.053252507	PASS
ARMS2	10	122454653	122457352	0.073090211	PASS
ARMT1	6	151452258	151470101	0.033894706	PASS
ARNT	1	150809705	150876768	0.079255572	PASS
ARNT2	15	80404350	80597937	-0.759380635	PASS
ARNTL	11	13276652	13387266	-0.39835217	PASS
ARNTL2	12	27332854	27425289	0.49032213	suppress_segment_too_large
ARPC1A	7	99325910	99366262	-0.710463272	PASS

ARPC1B	7	99374249	99394816	-0.710463272	PASS
ARPC2	2	218217094	218254356	0.011001208	PASS
ARPC3	12	110434825	110450422	0.028722739	PASS
ARPC4	3	9792495	9807726	0.06149513	PASS
ARPC4-TI	3	9793082	9835401	0.06149513	PASS
ARPC5	1	183620846	183635757	0.088103245	PASS
ARPC5L	9	124862130	124877733	0.073960965	PASS
ARPIN	15	89895006	89912956	-0.759380635	PASS
ARPP19	15	52547045	52569883	-0.423772929	PASS
ARPP21	3	35638945	35794496	-0.727808231	PASS
ARR3	23	70268305	70281840	0.158844052	PASS
ARRB1	11	75264182	75351705	0.064535884	PASS
ARRB2	17	4710489	4721499	0.06854245	PASS
ARRDC1	9	137605654	137615360	0.073960965	PASS
ARRDC2	19	18001132	18014102	-0.069222905	PASS
ARRDC3	5	91368724	91383359	-0.393084436	PASS
ARRDC4	15	97960698	97973838	-0.759380635	PASS
ARRDC5	19	4890437	4902867	-0.071973357	PASS
ARSA	22	50622754	50628173	-0.953096644	PASS
ARSB	5	78777209	78986087	-0.393084436	PASS
ARSD	23	2903970	2929351	0.158844052	PASS
ARSE	23	2934632	2968310	0.158844052	PASS
ARSF	23	3041471	3112726	0.158844052	PASS
ARSG	17	68259182	68422731	0.051798665	PASS
ARSH	23	3006613	3033571	0.158844052	PASS
ARSI	5	150296343	150339307	-0.393084436	PASS
ARSJ	4	113900284	113979727	-0.677982248	PASS
ARSK	5	95555074	95605064	-0.393084436	PASS
ART1	11	3645128	3664416	-0.39835217	PASS
ART3	4	76011184	76112802	-0.677982248	PASS
ART4	12	14825569	14843533	0.49032213	suppress_segment_too_large
ART5	11	3638503	3642316	-0.39835217	PASS
ARTN	1	43933320	43937241	-0.736943052	PASS
ARV1	1	230978981	231000595	-0.204996646	suppress_segment_too_large
ARVCF	22	19969896	20016808	-0.740245994	PASS
ARX	23	25003694	25016420	0.158844052	PASS
AS3MT	10	102869516	102901899	-0.414937136	PASS
ASAH1	8	18055992	18084998	-0.311940419	PASS
ASAH2	10	50182778	50248610	-0.389033546	PASS
ASAH2B	10	50739318	50816495	-0.389033546	PASS
ASAP1	8	130052104	130443660	0.493344802	suppress_segment_too_large
ASAP2	2	9206765	9405683	0.034263343	PASS
ASAP3	1	23428563	23484568	0.048749694	PASS
ASB1	2	238426742	238452250	0.011001208	PASS
ASB10	7	151175698	151187832	-0.712149877	PASS
ASB11	23	15281697	15315656	0.158844052	PASS
ASB12	23	64224196	64230631	0.158844052	PASS
ASB13	10	5638867	5666595	0.075711544	PASS
ASB14	3	57268347	57292682	-0.727808231	PASS
ASB15	7	123567010	123639481	-0.712149877	PASS
ASB16	17	44170447	44179083	0.051798665	PASS
ASB17	1	75918873	75932431	0.052232535	PASS
ASB18	2	236194872	236264409	0.011001208	PASS
ASB2	14	93934153	93976791	-0.155273506	suppress_segment_too_large

ASB3	2	53532672	53787610	0.034263343	PASS
ASB4	7	95478444	95540232	-0.710463272	PASS
ASB5	4	176213673	176277571	0.142431244	suppress_segment_too_large
ASB6	9	129634604	129642169	0.073960965	PASS
ASB7	15	100602534	100651705	-0.759380635	PASS
ASB8	12	48147788	48181213	-0.743220499	PASS
ASB9	23	15235288	15270467	0.158844052	PASS
ASCC1	10	72096032	72217134	0.032351597	PASS
ASCC2	22	29788608	29838304	-0.740245994	PASS
ASCC3	6	100508194	100881372	-0.355603029	PASS
ASCL1	12	102957686	102960516	0.028722739	PASS
ASCL2	11	2268495	2270952	-0.39835217	PASS
ASCL3	11	8937579	8938211	-0.39835217	PASS
ASCL4	12	107774385	107776644	0.028722739	PASS
ASCL5	1	201113953	201127184	-0.204996646	suppress_segment_too_large
ASF1A	6	118894220	118909167	-0.355603029	PASS
ASF1B	19	14119509	14136956	-0.069222905	PASS
ASGR1	17	7173431	7179564	0.06854245	PASS
ASGR2	17	7101322	7115700	0.06854245	PASS
ASH1L	1	155335268	155562807	0.088103245	PASS
ASH2L	8	38105242	38144076	0.182873287	suppress_segment_too_large
ASIC1	12	50057548	50083611	-0.743220499	PASS
ASIC2	17	33013087	34174964	0.041294512	PASS
ASIC3	7	151048292	151052756	-0.712149877	PASS
ASIC4	2	219514170	219538772	0.011001208	PASS
ASIC5	4	155829729	155866273	0.142431244	suppress_segment_too_large
ASIP	20	34194569	34269344	-0.172056771	suppress_segment_too_large
ASL	7	66075798	66093558	-0.701511252	PASS
ASMT	23	1615001	1643081	-0.350651546	PASS
ASMTL	23	1403139	1453762	-0.350651546	PASS
ASNA1	19	12737139	12748323	-0.069222905	PASS
ASNS	7	97852118	97872542	-0.710463272	PASS
ASNSD1	2	189661385	189670831	0.011001208	PASS
ASPA	17	3472374	3503419	0.06854245	PASS
ASPDH	19	50511600	50514690	0.002743503	PASS
ASPG	14	104085679	104115581	-0.155273506	suppress_segment_too_large
ASPH	8	61500556	61714640	0.182873287	suppress_segment_too_large
ASPHD1	16	29900375	29919864	0.053595371	PASS
ASPHD2	22	26429273	26445015	-0.740245994	PASS
ASPM	1	197084128	197146694	0.088103245	PASS
ASPN	9	92456205	92482506	0.073960965	PASS
ASPRV1	2	69960089	69962265	-0.46122572	PASS
ASPSCR1	17	81976807	82017406	0.051798665	PASS
ASRGL1	11	62337448	62393412	0.064535884	PASS
ASS1	9	130444929	130501274	0.073960965	PASS
ASTE1	3	131013875	131027649	0.06434429	PASS
ASTL	2	96123850	96138436	0.064122364	PASS
ASTN1	1	176857302	177164973	0.088103245	PASS
ASTN2	9	116425225	117415070	0.073960965	PASS
ASXL1	20	32358330	32439319	-0.172056771	suppress_segment_too_large
ASXL2	2	25733753	25878516	0.034263343	PASS
ASXL3	18	33578233	33751192	-0.330515409	PASS
ASZ1	7	117363222	117428123	-0.712149877	PASS
ATAD2	8	123319850	123416350	0.493344802	suppress_segment_too_large

ATAD2B	2	23748664	23927114	0.034263343	PASS
ATAD3A	1	1512151	1534687	-0.358281697	PASS
ATAD3B	1	1471769	1497848	-0.358281697	PASS
ATAD3C	1	1449689	1470158	-0.358281697	PASS
ATAD5	17	30831970	30895869	0.041294512	PASS
ATAT1	6	30626842	30646823	0.069043418	PASS
ATCAY	19	3879864	3928079	-0.071973357	PASS
ATE1	10	121740421	121928801	0.073090211	PASS
ATF1	12	50763710	50821122	-0.743220499	PASS
ATF2	2	175072250	175168382	0.033541467	PASS
ATF3	1	212565334	212620777	-0.204996646	suppress_segment_too_large
ATF4	22	39519695	39522685	-0.740245994	PASS
ATF5	19	49928702	49933935	0.002743503	PASS
ATF6	1	161766294	161964070	0.088103245	PASS
ATF6B	6	32115264	32128253	0.028532667	PASS
ATF7	12	53507856	53626410	-0.743220499	PASS
ATF7IP	12	14365632	14502935	0.49032213	suppress_segment_too_large
ATF7IP2	16	10326434	10483638	0.053595371	PASS
ATG10	5	81972025	82276857	-0.393084436	PASS
ATG101	12	52069246	52077494	-0.743220499	PASS
ATG12	5	115828200	115841858	-0.393084436	PASS
ATG13	11	46617527	46674818	0.059795455	PASS
ATG14	14	55366392	55411858	0.053252507	PASS
ATG16L1	2	233210051	233295674	0.011001208	PASS
ATG16L2	11	72814308	72843674	0.064535884	PASS
ATG2A	11	64894546	64917248	0.064535884	PASS
ATG2B	14	96279202	96363870	-0.155273506	suppress_segment_too_large
ATG3	3	112532509	112562046	0.06434429	PASS
ATG4A	23	108091668	108154671	0.158844052	PASS
ATG4C	1	62784135	62865513	0.052232535	PASS
ATG4D	19	10543895	10553418	-0.107594294	PASS
ATG5	6	106045423	106325791	-0.355603029	PASS
ATG7	3	11272309	11557665	0.06149513	PASS
ATG9A	2	219219380	219229717	0.011001208	PASS
ATG9B	7	151012209	151024499	-0.712149877	PASS
ATIC	2	215311817	215349773	0.011001208	PASS
ATL1	14	50532509	50633068	0.053252507	PASS
ATL2	2	38294880	38377285	0.034263343	PASS
ATL3	11	63624087	63671921	0.064535884	PASS
ATM	11	108222484	108369102	-0.431650509	PASS
ATMIN	16	81035847	81047358	-0.423061015	PASS
ATN1	12	6924463	6942321	-0.015029783	PASS
ATOH1	4	93828753	93830964	-0.677982248	PASS
ATOH7	10	68230624	68232103	0.032351597	PASS
ATOH8	2	85751344	85788066	0.064122364	PASS
ATOX1	5	151742316	151772532	-0.393084436	PASS
ATP10A	15	25677273	25865172	-0.423772929	PASS
ATP10B	5	160563120	160852214	0.165341871	suppress_segment_too_large
ATP10D	4	47485288	47593486	0.115781418	suppress_segment_too_large
ATP11A	13	112690329	112887168	-0.426822582	PASS
ATP11B	3	182793500	182921635	0.02267528	PASS
ATP11C	23	139726346	139945276	0.158844052	PASS
ATP12A	13	24680411	24712493	-0.398748891	PASS
ATP13A1	19	19645198	19663693	-0.069222905	PASS

ATP13A2	1	16985958	17011928	0.048749694	PASS
ATP13A3	3	194402672	194498364	0.02267528	PASS
ATP13A4	3	193402077	193593111	0.02267528	PASS
ATP13A5	3	193274790	193378843	0.02267528	PASS
ATP1A1	1	116372668	116410261	0.079255572	PASS
ATP1A2	1	160115759	160143591	0.088103245	PASS
ATP1A3	19	41966582	41997497	0.048917043	PASS
ATP1A4	1	160151570	160186977	0.088103245	PASS
ATP1B1	1	169105697	169132722	0.088103245	PASS
ATP1B2	17	7646627	7657768	0.06854245	PASS
ATP1B3	3	141876124	141926514	-0.889410434	PASS
ATP1B4	23	120362085	120383249	0.158844052	PASS
ATP23	12	57906039	57959148	-0.743220499	PASS
ATP2A1	16	28878405	28904509	0.053595371	PASS
ATP2A2	12	110280756	110351093	0.028722739	PASS
ATP2A3	17	3923870	3964464	0.06854245	PASS
ATP2B1	12	89588049	89709300	0.271127429	suppress_segment_too_large
ATP2B2	3	10324023	10708007	0.06149513	PASS
ATP2B3	23	153517676	153582939	0.158844052	PASS
ATP2B4	1	203626561	203744081	-0.204996646	suppress_segment_too_large
ATP2C1	3	130850595	131016712	0.06434429	PASS
ATP2C2	16	84368527	84464187	-0.423061015	PASS
ATP4A	19	35550043	35563658	0.044838622	PASS
ATP4B	13	113648804	113658186	-0.426822582	PASS
ATP5F1A	18	46080248	46104334	-0.330515409	PASS
ATP5F1B	12	56638175	56646068	-0.743220499	PASS
ATP5F1C	10	7788129	7807815	0.075711544	PASS
ATP5F1D	19	1241746	1244826	-0.071973357	PASS
ATP5F1E	20	59025467	59032382	-0.172056771	suppress_segment_too_large
ATP5IF1	1	28236109	28246906	0.048749694	PASS
ATP5MC1	17	48892765	48895871	0.051798665	PASS
ATP5MC2	12	53632726	53677408	-0.743220499	PASS
ATP5MC3	2	175176258	175184607	0.033541467	PASS
ATP5MD	10	103389041	103396492	-0.414937136	PASS
ATP5ME	4	672436	674338	-0.358623662	PASS
ATP5MF	7	99448475	99466331	-0.710463272	PASS
ATP5MF-I	7	99419749	99466197	-0.710463272	PASS
ATP5MG	11	118401154	118431496	-0.431650509	PASS
ATP5MGL	22	42639803	42640601	-0.740245994	PASS
ATP5MPL	14	103912288	103928269	-0.155273506	suppress_segment_too_large
ATP5PB	1	111448864	111462773	0.079255572	PASS
ATP5PD	17	75038863	75046985	0.051798665	PASS
ATP5PF	21	25716503	25735673	0.209983559	PASS
ATP5PO	21	33903453	33915980	0.209983559	PASS
ATP5S	14	50312326	50335558	0.053252507	PASS
ATP6AP1	23	154428632	154436516	0.158844052	PASS
ATP6AP1I	5	82279462	82386977	-0.393084436	PASS
ATP6AP2	23	40579372	40606848	0.158844052	PASS
ATP6V0A	17	42458844	42522611	0.041294512	PASS
ATP6V0A:	12	123712318	123761755	0.028722739	PASS
ATP6V0A	7	138706294	138799560	-0.712149877	PASS
ATP6V0B	1	43974487	43978295	-0.736943052	PASS
ATP6V0C	16	2513870	2520218	0.053595371	PASS
ATP6V0D	16	67438014	67481237	-0.423061015	PASS

ATP6V0D2	8	85987323	86154228	0.617295349	suppress_segment_too_large
ATP6V0E1	5	172983757	173035445	0.165341871	suppress_segment_too_large
ATP6V0E2	7	149872968	149891204	-0.712149877	PASS
ATP6V1A	3	113747019	113812056	0.06434429	PASS
ATP6V1B1	2	70935868	70965431	-0.46122572	PASS
ATP6V1B2	8	20197367	20226819	-0.311940419	PASS
ATP6V1C1	8	103021063	103073051	0.355469066	suppress_segment_too_large
ATP6V1C2	2	10721100	10785110	0.034263343	PASS
ATP6V1D	14	67294371	67360265	0.053252507	PASS
ATP6V1E1	22	17592136	17628818	-0.740245994	PASS
ATP6V1E2	2	46490750	46542557	0.034263343	PASS
ATP6V1F	7	128862826	128865844	-0.712149877	PASS
ATP6V1FN	7	128866308	128872044	-0.712149877	PASS
ATP6V1G	9	114587746	114598373	0.073960965	PASS
ATP6V1G2	6	31544462	31548427	0.028532667	PASS
ATP6V1G2	6	31530219	31546608	0.028532667	PASS
ATP6V1G3	1	198523222	198540945	0.088103245	PASS
ATP6V1H	8	53715557	53843558	0.182873287	suppress_segment_too_large
ATP7A	23	77910656	78050395	0.158844052	PASS
ATP7B	13	51930436	52012125	-0.384313056	PASS
ATP8A1	4	42408373	42657105	-0.753159364	PASS
ATP8A2	13	25372071	26025851	-0.398748891	PASS
ATP8B1	18	57646426	57803315	-0.330515409	PASS
ATP8B2	1	154325553	154351307	0.088103245	PASS
ATP8B3	19	1782075	1812276	-0.071973357	PASS
ATP8B4	15	49858238	50182817	-0.423772929	PASS
ATP9A	20	51596514	51768634	-0.172056771	suppress_segment_too_large
ATP9B	18	79069285	79378287	-0.330515409	PASS
ATPAF1	1	46632737	46673867	-0.736943052	PASS
ATPAF2	17	17977409	18039209	0.06854245	PASS
ATR	3	142449235	142578826	0.02267528	PASS
ATRAID	2	27212027	27217178	0.034263343	PASS
ATRIP	3	48446710	48467645	-0.727808231	PASS
ATRN	20	3471040	3651122	-0.172056771	suppress_segment_too_large
ATRNL1	10	115093365	115948992	0.073090211	PASS
ATRX	23	77504878	77786233	0.158844052	PASS
ATXN1	6	16299112	16761491	0.069043418	PASS
ATXN10	22	45671798	45845307	-0.953096644	PASS
ATXN1L	16	71845991	71885268	-0.423061015	PASS
ATXN2	12	111443485	111599676	0.028722739	PASS
ATXN2L	16	28823035	28837237	0.053595371	PASS
ATXN3	14	92044496	92106625	-0.155273506	suppress_segment_too_large
ATXN3L	23	13318236	13320399	0.158844052	PASS
ATXN7	3	63864557	64003462	-0.727808231	PASS
ATXN7L1	7	105605067	105876604	-0.712149877	PASS
ATXN7L2	1	109483479	109492804	0.079255572	PASS
ATXN7L3	17	44191805	44200113	0.051798665	PASS
ATXN7L3	12	74537827	74545430	0.271127429	suppress_segment_too_large
AUH	9	91213815	91361913	0.073960965	PASS
AUNIP	1	25831913	25859458	0.048749694	PASS
AUP1	2	74526645	74529939	-0.46122572	PASS
AURKA	20	56369389	56392337	-0.172056771	suppress_segment_too_large
AURKAIP	1	1373730	1375495	-0.358281697	PASS
AURKB	17	8204733	8210600	0.06854245	PASS

AURKC	19	57230802	57235548	0.142581352	PASS
AUTS2	7	69598296	70793495	-0.701511252	PASS
AVEN	15	33866227	34039176	-0.423772929	PASS
AVIL	12	57797376	57818704	-0.743220499	PASS
AVL9	7	32495426	32588721	-0.72421718	PASS
AVP	20	3082556	3084724	-0.172056771	suppress_segment_too_large
AVPI1	10	97677424	97687323	-0.414937136	PASS
AVPR1A	12	63142759	63150942	-0.743220499	PASS
AVPR1B	1	206109692	206117699	-0.204996646	suppress_segment_too_large
AVPR2	23	153902531	153907166	0.158844052	PASS
AWAT1	23	70234655	70240627	0.158844052	PASS
AWAT2	23	70040542	70049938	0.158844052	PASS
AXDND1	1	179365720	179554735	0.088103245	PASS
AXIN1	16	287440	352673	0.053595371	PASS
AXIN2	17	65528563	65561647	0.051798665	PASS
AXL	19	41219203	41261766	0.048917043	PASS
AZGP1	7	99966720	99976157	-0.710463272	PASS
AZI2	3	28315003	28349127	0.06149513	PASS
AZIN1	8	102826357	102893864	0.355469066	suppress_segment_too_large
AZIN2	1	33081104	33120530	0.048749694	PASS
AZU1	19	825097	832018	-0.071973357	PASS
B2M	15	44711487	44718877	-0.423772929	PASS
B3GALNT	3	161083883	161105384	0.02267528	PASS
B3GALNT	1	235449923	235504481	-0.204996646	suppress_segment_too_large
B3GALT1	2	167868948	167874041	0.319987345	PASS
B3GALT2	1	193179045	193186654	0.088103245	PASS
B3GALT4	6	33277132	33284832	0.028532667	PASS
B3GALT5	21	39556442	39673137	0.209983559	PASS
B3GALT6	1	1232226	1235041	-0.358281697	PASS
B3GAT1	11	134378504	134411918	0.105032849	PASS
B3GAT2	6	70856679	70957038	-0.250122709	PASS
B3GAT3	11	62615296	62622175	0.064535884	PASS
B3GLCT	13	31199936	31332276	-0.398748891	PASS
B3GNT2	2	62196113	62224731	-0.46122572	PASS
B3GNT3	19	17794828	17813082	-0.069222905	PASS
B3GNT4	12	122203543	122208952	0.028722739	PASS
B3GNT5	3	183253244	183298504	0.02267528	PASS
B3GNT6	11	77034398	77041973	0.064535884	PASS
B3GNT7	2	231395543	231401164	0.011001208	PASS
B3GNT8	19	41425359	41428730	0.048917043	PASS
B3GNT9	16	67148105	67151214	-0.423061015	PASS
B3GNTL1	17	82942155	83051810	0.051798665	PASS
B4GALNT	12	57623410	57633355	-0.743220499	PASS
B4GALNT	17	49132460	49176840	0.051798665	PASS
B4GALNT	12	460364	563509	-0.015029783	PASS
B4GALNT	11	369796	382116	-0.39835217	PASS
B4GALT1	9	33104082	33167356	0.073960965	PASS
B4GALT2	1	43978943	43991170	-0.736943052	PASS
B4GALT3	1	161171310	161177968	0.088103245	PASS
B4GALT4	3	119211732	119241103	0.06434429	PASS
B4GALT5	20	49632945	49713878	-0.172056771	suppress_segment_too_large
B4GALT6	18	31622247	31685836	-0.330515409	PASS
B4GALT7	5	177600100	177610347	0.165341871	suppress_segment_too_large
B4GAT1	11	66345372	66347692	0.064535884	PASS

B9D1	17	19337554	19378193	0.06854245	PASS
B9D2	19	41354421	41364173	0.048917043	PASS
BAALC	8	103140710	103230305	0.355469066	suppress_segment_too_large
BAAT	9	101360417	101383519	0.073960965	PASS
BABAM1	19	17267350	17281249	-0.069222905	PASS
BABAM2	2	27889941	28338901	0.034263343	PASS
BACE1	11	117285207	117316259	-0.431650509	PASS
BACE2	21	41167801	41282518	0.209983559	PASS
BACH1	21	29194071	29630751	0.209983559	PASS
BACH2	6	89926529	90296908	-0.355603029	PASS
BAD	11	64269830	64284704	0.064535884	PASS
BAG1	9	33247820	33264720	0.073960965	PASS
BAG2	6	57172326	57189833	0.239805577	suppress_segment_too_large
BAG3	10	119651370	119677819	0.073090211	PASS
BAG4	8	38176533	38213301	0.182873287	suppress_segment_too_large
BAG5	14	103556544	103562831	-0.155273506	suppress_segment_too_large
BAG6	6	31639028	31652705	0.028532667	PASS
BAHCC1	17	81395475	81466332	0.051798665	PASS
BAHD1	15	40439721	40468242	-0.423772929	PASS
BAIAP2	17	81035122	81117432	0.051798665	PASS
BAIAP2L1	7	98291651	98401068	-0.710463272	PASS
BAIAP2L2	22	38084889	38110670	-0.740245994	PASS
BAIAP3	16	1333601	1349441	0.053595371	PASS
BAK1	6	33572547	33580293	0.028532667	PASS
BAMBI	10	28677342	28682939	0.075711544	PASS
BANF1	11	66002079	66004149	0.064535884	PASS
BANF2	20	17693672	17735871	-0.172056771	suppress_segment_too_large
BANK1	4	101411286	102074812	-0.677982248	PASS
BANP	16	87949244	88077318	-0.423061015	PASS
BAP1	3	52401013	52410350	-0.727808231	PASS
BARD1	2	214725646	214809711	0.011001208	PASS
BARHL1	9	132582185	132590266	0.073960965	PASS
BARHL2	1	90711539	90717237	0.079255572	PASS
BARX1	9	93951622	93955372	0.073960965	PASS
BARX2	11	129375940	129452279	-0.431650509	PASS
BASP1	5	17065598	17276843	0.073527871	PASS
BATF	14	75522425	75547015	-0.155273506	suppress_segment_too_large
BATF2	11	64987943	64997045	0.064535884	PASS
BATF3	1	212686418	212699985	-0.204996646	suppress_segment_too_large
BAX	19	48954815	48961798	0.002743503	PASS
BAZ1A	14	34752731	34875647	0.053252507	PASS
BAZ1B	7	73440398	73522278	-0.701511252	PASS
BAZ2A	12	56595596	56636816	-0.743220499	PASS
BAZ2B	2	159318979	159616692	0.054749241	PASS
BBC3	19	47220822	47232766	0.002743503	PASS
BBIP1	10	110898730	110919274	0.073090211	PASS
BBOF1	14	74019353	74082863	0.053252507	PASS
BBOX1	11	27040725	27127809	-0.359426047	PASS
BBS1	11	66510606	66533627	0.064535884	PASS
BBS10	12	76344474	76348442	0.271127429	suppress_segment_too_large
BBS12	4	122732702	122744943	0.142431244	suppress_segment_too_large
BBS2	16	56466836	56520283	-0.423061015	PASS
BBS4	15	72686179	72738476	-0.759380635	PASS
BBS5	2	169479178	169506655	0.033541467	PASS

BBS7	4	121824440	121870497	0.142431244	suppress_segment_too_large
BBS9	7	33129244	33606068	-0.72421718	PASS
BBX	3	107522936	107811324	0.22470242	PASS
BCAM	19	44809059	44821421	0.002743503	PASS
BCAN	1	156641390	156659532	0.088103245	PASS
BCAP29	7	107579977	107629170	-0.712149877	PASS
BCAP31	23	153700492	153724746	0.158844052	PASS
BCAR1	16	75228187	75268053	-0.423061015	PASS
BCAR3	1	93561786	93847150	0.079255572	PASS
BCAS1	20	53936777	54070594	-0.172056771	suppress_segment_too_large
BCAS2	1	114567557	114581639	0.079255572	PASS
BCAS3	17	60677453	61392838	0.051798665	PASS
BCAS4	20	50794894	50882676	-0.172056771	suppress_segment_too_large
BCAT1	12	24810022	24949459	0.49032213	suppress_segment_too_large
BCAT2	19	48795062	48811029	0.002743503	PASS
BCCIP	10	125823546	125853695	0.073090211	PASS
BCDIN3D	12	49836039	49843129	-0.743220499	PASS
BCHE	3	165772904	165837472	0.02267528	PASS
BCKDHA	19	41397460	41425005	0.048917043	PASS
BCKDHB	6	80106647	80346270	-0.250122709	PASS
BCKDK	16	31106107	31112791	0.053595371	PASS
BCL10	1	85265776	85276904	0.587598296	PASS
BCL11A	2	60450520	60555154	-0.46122572	PASS
BCL11B	14	99169287	99271524	-0.155273506	suppress_segment_too_large
BCL2	18	63123346	63320128	-0.330515409	PASS
BCL2A1	15	79960889	79971446	-0.759380635	PASS
BCL2L1	20	31664452	31723989	-0.172056771	suppress_segment_too_large
BCL2L10	15	52109263	52112775	-0.423772929	PASS
BCL2L11	2	111119378	111168447	0.040971718	PASS
BCL2L12	19	49665142	49673916	0.002743503	PASS
BCL2L13	22	17628855	17730855	-0.740245994	PASS
BCL2L14	12	12049844	12211084	0.49032213	suppress_segment_too_large
BCL2L15	1	113878168	113887547	0.079255572	PASS
BCL2L2	14	23298790	23311759	0.053252507	PASS
BCL2L2-P	14	23306835	23325369	0.053252507	PASS
BCL3	19	44747705	44760044	0.002743503	PASS
BCL6	3	187721377	187745727	0.02267528	PASS
BCL6B	17	7023020	7030290	0.06854245	PASS
BCL7A	12	122019422	122062044	0.028722739	PASS
BCL7B	7	73536356	73558002	-0.701511252	PASS
BCL7C	16	30833626	30894960	0.053595371	PASS
BCL9	1	147541412	147626216	0.079255572	PASS
BCL9L	11	118893875	118925608	-0.431650509	PASS
BCLAF1	6	136256627	136289851	0.033894706	PASS
BCLAF3	23	19912860	19970298	0.158844052	PASS
BCO1	16	81238448	81291142	-0.423061015	PASS
BCO2	11	112175467	112224699	-0.431650509	PASS
BCOR	23	40049815	40177329	0.158844052	PASS
BCORL1	23	129981107	130058083	0.158844052	PASS
BCR	22	23179704	23318037	-0.740245994	PASS
BCS1L	2	218658764	218663443	0.011001208	PASS
BDH1	3	197509783	197573323	0.02267528	PASS
BDH2	4	103079435	103099883	-0.677982248	PASS
BDKRB1	14	96255824	96268967	-0.155273506	suppress_segment_too_large

BDKRB2	14	96204679	96244166		-0.155273506	suppress_segment_too_large
BDNF	11	27654893	27722058		-0.359426047	PASS
BDP1	5	71455615	71567820		-0.393084436	PASS
BEAN1	16	66427297	66493529		-0.423061015	PASS
BECN1	17	42810134	42833350		0.041294512	PASS
BECN2	1	241957767	241959062		-0.287484925	PASS
BEGAIN	14	100537147	100587413		-0.155273506	suppress_segment_too_large
BEND2	23	18162931	18220883		0.158844052	PASS
BEND3	6	107065182	107115269		-0.355603029	PASS
BEND4	4	42110938	42152878		-0.753159364	PASS
BEND5	1	48727523	48776969		-0.736943052	PASS
BEND6	6	56955126	57027342		0.239805577	suppress_segment_too_large
BEND7	10	13438484	13528974		0.075711544	PASS
BEST1	11	61949821	61965515		0.064535884	PASS
BEST2	19	12751702	12758458		-0.069222905	PASS
BEST3	12	69643360	69699476		-0.743220499	PASS
BEST4	1	44783585	44787705		-0.736943052	PASS
BET1	7	93962762	94004382		-0.710463272	PASS
BET1L	11	167784	207428		-0.39835217	PASS
BEX1	23	103062651	103064240		0.158844052	PASS
BEX2	23	103309346	103311046		0.158844052	PASS
BEX3	23	103376340	103378077		0.158844052	PASS
BEX4	23	103215092	103217246		0.158844052	PASS
BEX5	23	102153708	102156057		0.158844052	PASS
BFAR	16	14632815	14669236		0.053595371	PASS
BFSP1	20	17493905	17569220		-0.172056771	suppress_segment_too_large
BFSP2	3	133399995	133475222		0.06434429	PASS
BGLAP	1	156241962	156243321		0.088103245	PASS
BGN	23	153494939	153509554		0.158844052	PASS
BHLHA15	7	98211427	98212979		-0.710463272	PASS
BHLHA9	17	1270559	1271460		0.06854245	PASS
BHLHB9	23	102720688	102753540		0.158844052	PASS
BHLHE22	8	64580367	64583628		0.182873287	suppress_segment_too_large
BHLHE23	20	63005927	63007035		-0.172056771	suppress_segment_too_large
BHLHE40	3	4979116	4985323		0.06149513	PASS
BHLHE41	12	26120026	26125127		0.49032213	suppress_segment_too_large
BHMG1	19	45733251	45764534		0.002743503	PASS
BHMT	5	79111779	79132290		-0.393084436	PASS
BHMT2	5	79069717	79089466		-0.393084436	PASS
BICC1	10	58513140	58831437		0.032351597	PASS
BICD1	12	32106835	32383633		0.49032213	suppress_segment_too_large
BICD2	9	92711363	92764812		0.073960965	PASS
BICDL1	12	119989869	120094494		0.028722739	PASS
BICDL2	16	3027682	3036926		0.053595371	PASS
BICRA	19	47608196	47703277		0.002743503	PASS
BICRAL	6	42746958	42868560		0.028532667	PASS
BID	22	17734138	17774770		-0.740245994	PASS
BIK	22	43110748	43129712		-0.740245994	PASS
BIN1	2	127048027	127107355		-0.359440172	PASS
BIN2	12	51281038	51324668		-0.743220499	PASS
BIN3	8	22620418	22669148		-0.311940419	PASS
BIRC2	11	102347211	102378670		0.32805343	suppress_segment_too_large
BIRC3	11	102317450	102339403		0.32805343	suppress_segment_too_large
BIRC5	17	78214186	78225636		0.051798665	PASS

BIRC6	2	32357028	32618899	0.034263343	PASS
BIRC7	20	63235883	63240507	-0.172056771	suppress_segment_too_large
BIRC8	19	53289601	53291626	0.416834572	PASS
BIVM	13	102799049	102841535	-0.426822582	PASS
BIVM-ER(	13	102799110	102875994	-0.426822582	PASS
BLCAP	20	37492472	37527931	-0.172056771	suppress_segment_too_large
BLID	11	122115354	122116215	-0.431650509	PASS
BLK	8	11486894	11564604	-0.311940419	PASS
BLM	15	90717327	90816165	-0.759380635	PASS
BLMH	17	30248195	30292056	0.041294512	PASS
BLNK	10	96191702	96271587	-0.414937136	PASS
BLOC1S1	12	55716037	55720087	-0.743220499	PASS
BLOC1S1-	12	55716036	55724703	-0.743220499	PASS
BLOC1S2	10	100273280	100286712	-0.414937136	PASS
BLOC1S3	19	45178745	45181801	0.002743503	PASS
BLOC1S4	4	6716055	6717671	-0.358623662	PASS
BLOC1S5	6	8013567	8064414	0.069043418	PASS
BLOC1S5-	6	7881522	8064364	0.069043418	PASS
BLOC1S6	15	45587123	45615999	-0.423772929	PASS
BLVRA	7	43758680	43807342	-0.72421718	PASS
BLVRB	19	40447765	40465840	0.048917043	PASS
BLZF1	1	169367970	169396540	0.088103245	PASS
BMF	15	40087890	40108892	-0.423772929	PASS
BMI1	10	22321211	22331484	0.075711544	PASS
BMP1	8	22164736	22212326	-0.311940419	PASS
BMP10	2	68865481	68871517	-0.46122572	PASS
BMP15	23	50910784	50916607	0.158844052	PASS
BMP2	20	6767664	6780280	-0.172056771	suppress_segment_too_large
BMP2K	4	78776342	78916372	-0.677982248	PASS
BMP3	4	81030965	81057531	-0.677982248	PASS
BMP4	14	53949736	53958761	0.053252507	PASS
BMP5	6	55753645	55875564	0.239805577	suppress_segment_too_large
BMP6	6	7726797	7881422	0.069043418	PASS
BMP7	20	57168748	57266629	-0.172056771	suppress_segment_too_large
BMP8A	1	39491646	39525935	0.048749694	PASS
BMP8B	1	39757182	39788861	0.048749694	PASS
BMPER	7	33904308	34156356	-0.72421718	PASS
BMPR1A	10	86756601	86932838	-0.461254852	PASS
BMPR1B	4	94757968	95158448	-0.677982248	PASS
BMPR2	2	202376936	202567751	0.011001208	PASS
BMS1	10	42782801	42834937	-0.389033546	PASS
BMT2	7	112819147	112939916	-0.712149877	PASS
BMX	23	15464246	15556529	0.158844052	PASS
BNC1	15	83255903	83284714	-0.759380635	PASS
BNC2	9	16409503	16870843	0.073960965	PASS
BNIP1	5	173144442	173164387	0.165341871	suppress_segment_too_large
BNIP2	15	59659146	59689534	-0.759380635	PASS
BNIP3	10	131966455	131982013	0.073090211	PASS
BNIP3L	8	26382898	26505636	-0.311940419	PASS
BNIPL	1	151036321	151047720	0.079255572	PASS
BOC	3	113211003	113287459	0.06434429	PASS
BOD1	5	173607514	173616660	0.165341871	suppress_segment_too_large
BOD1L1	4	13568738	13627723	-0.358623662	PASS
BOD1L2	18	57147062	57150300	-0.330515409	PASS

BOLA1	1	149887890	149900798	0.079255572	PASS
BOLA3	2	74135398	74147994	-0.46122572	PASS
BOLL	2	197726879	197786762	0.011001208	PASS
BOP1	8	144262102	144291370	0.699132336	PASS
BORA	13	72727749	72756198	-0.384313056	PASS
BORCS5	12	12357079	12469694	0.49032213	suppress_segment_too_large
BORCS6	17	8188333	8190907	0.06854245	PASS
BORCS7	10	102854223	102864961	-0.414937136	PASS
BORCS7-[illegible]	10	102854272	102901899	-0.414937136	PASS
BORCS8	19	19176903	19192591	-0.069222905	PASS
BORCS8-[illegible]	19	19145567	19192158	-0.069222905	PASS
BPGM	7	134646808	134679813	-0.712149877	PASS
BPHL	6	3118374	3153578	0.069043418	PASS
BPI	20	38304123	38337505	-0.172056771	suppress_segment_too_large
BPIFA1	20	33235995	33243311	-0.172056771	suppress_segment_too_large
BPIFA2	20	33161768	33181412	-0.172056771	suppress_segment_too_large
BPIFA3	20	33217310	33227758	-0.172056771	suppress_segment_too_large
BPIFB1	20	33273480	33309878	-0.172056771	suppress_segment_too_large
BPIFB2	20	33007600	33023709	-0.172056771	suppress_segment_too_large
BPIFB3	20	33055424	33073628	-0.172056771	suppress_segment_too_large
BPIFB4	20	33079644	33111751	-0.172056771	suppress_segment_too_large
BPIFB6	20	33031648	33044047	-0.172056771	suppress_segment_too_large
BPIFC	22	32413847	32464484	-0.740245994	PASS
BPNT1	1	220057482	220090462	-0.204996646	suppress_segment_too_large
BPTF	17	67825524	67984378	0.051798665	PASS
BRAF	7	140719327	140924928	-0.712149877	PASS
BRAP	12	111642146	111685986	0.028722739	PASS
BRAT1	7	2537877	2555727	-0.72421718	PASS
BRCA1	17	43044295	43170245	0.041294512	PASS
BRCA2	13	32315474	32400266	-0.398748891	PASS
BRCC3	23	155071420	155123074	0.158844052	PASS
BRD1	22	49773283	49827512	-0.953096644	PASS
BRD2	6	32968660	32981505	0.028532667	PASS
BRD3	9	134030305	134068535	0.073960965	PASS
BRD3OS	9	134025439	134034666	0.073960965	PASS
BRD4	19	15235519	15332545	-0.069222905	PASS
BRD7	16	50313487	50368934	0.411512417	PASS
BRD8	5	138139766	138178986	-0.393084436	PASS
BRD9	5	850291	892824	0.073527871	PASS
BRDT	1	91949371	92014426	0.079255572	PASS
BRF1	14	105209286	105315589	-0.155273506	suppress_segment_too_large
BRF2	8	37843268	37849904	0.182873287	suppress_segment_too_large
BRI3	7	98252379	98310441	-0.710463272	PASS
BRI3BP	12	124993700	125031231	0.028722739	PASS
BRICD5	16	2209253	2211950	0.053595371	PASS
BRINP1	9	119153458	119369467	0.073960965	PASS
BRINP2	1	177171497	177282422	0.088103245	PASS
BRINP3	1	190097662	190478404	0.088103245	PASS
BRIP1	17	61681266	61863521	0.051798665	PASS
BRIX1	5	34915376	34925996	0.073527871	PASS
BRK1	3	10115592	10127190	0.06149513	PASS
BRMS1	11	66337333	66345125	0.064535884	PASS
BRMS1L	14	35826318	35932325	0.053252507	PASS
BROX	1	222712553	222735196	-0.204996646	suppress_segment_too_large

BRPF1	3	9731729	9748018	0.06149513	PASS
BRPF3	6	36196744	36232790	0.028532667	PASS
BRS3	23	136487887	136493780	0.158844052	PASS
BRSK1	19	55282072	55312533	0.142581352	PASS
BRSK2	11	1389899	1462689	-0.39835217	PASS
BRWD1	21	39184176	39321559	0.209983559	PASS
BRWD3	23	80670854	80809688	0.158844052	PASS
BSCL2	11	62690275	62709845	0.064535884	PASS
BSDC1	1	32365103	32394731	0.048749694	PASS
BSG	19	571277	583493	-0.071973357	PASS
BSN	3	49554489	49671545	-0.727808231	PASS
BSND	1	54998193	55011784	-0.736943052	PASS
BSPH1	19	47968046	47992170	0.002743503	PASS
BSPRY	9	113349541	113371233	0.073960965	PASS
BST1	4	15702950	15738313	-0.358623662	PASS
BST2	19	17402939	17405648	-0.069222905	PASS
BSX	11	122977570	122981720	-0.431650509	PASS
BTAF1	10	91923769	92030325	-0.414937136	PASS
BTBD1	15	83016422	83067354	-0.759380635	PASS
BTBD10	11	13388001	13463297	-0.39835217	PASS
BTBD11	12	107318413	107659642	0.028722739	PASS
BTBD16	10	122271306	122338162	0.073090211	PASS
BTBD17	17	74356416	74361946	0.051798665	PASS
BTBD18	11	57743514	57751781	0.27006553	suppress_segment_too_large
BTBD19	1	44808482	44815585	-0.736943052	PASS
BTBD2	19	1985438	2034881	-0.071973357	PASS
BTBD3	20	11890723	11926609	-0.172056771	suppress_segment_too_large
BTBD6	14	105248490	105251093	-0.155273506	suppress_segment_too_large
BTBD7	14	93237550	93333092	-0.155273506	suppress_segment_too_large
BTBD8	1	92080305	92184725	0.079255572	PASS
BTBD9	6	38168451	38640148	0.028532667	PASS
BTC	4	74744759	74794686	0.115781418	suppress_segment_too_large
BTD	3	15601341	15647640	0.06149513	PASS
BTF3	5	73498408	73505635	-0.393084436	PASS
BTF3L4	1	52056125	52090716	-0.736943052	PASS
BTG1	12	92140278	92145897	0.271127429	suppress_segment_too_large
BTG2	1	203305491	203309602	-0.204996646	suppress_segment_too_large
BTG3	21	17593653	17612947	0.758930684	suppress_segment_too_large
BTG4	11	111467526	111512354	-0.431650509	PASS
BTK	23	101349447	101390796	0.158844052	PASS
BTLA	3	112463968	112499561	0.06434429	PASS
BTN1A1	6	26501221	26510422	0.069043418	PASS
BTN2A1	6	26457904	26476621	0.069043418	PASS
BTN2A2	6	26383096	26394874	0.069043418	PASS
BTN3A1	6	26402237	26415216	0.069043418	PASS
BTN3A2	6	26365159	26378320	0.069043418	PASS
BTN3A3	6	26440472	26453415	0.069043418	PASS
BTNL2	6	32393963	32407128	0.028532667	PASS
BTNL3	5	180988845	181006727	0.165341871	suppress_segment_too_large
BTNL8	5	180899077	180950906	0.165341871	suppress_segment_too_large
BTNL9	5	181040225	181061523	0.165341871	suppress_segment_too_large
BTRC	10	101354033	101557321	-0.414937136	PASS
BUB1	2	110637698	110678114	0.040971718	PASS
BUB1B	15	40161023	40221136	-0.423772929	PASS

BUB1B-PA	15	40217428	40276396	-0.423772929	PASS
BUB3	10	123154277	123170467	0.073090211	PASS
BUD13	11	116748170	116772988	-0.431650509	PASS
BUD23	7	73683025	73705161	-0.701511252	PASS
BUD31	7	99408641	99419616	-0.710463272	PASS
BVES	6	105096822	105137174	-0.355603029	PASS
BX276092	23	71667542	71671524	0.158844052	PASS
BYSL	6	41921188	41933046	0.028532667	PASS
BZW1	2	200810594	200827338	0.011001208	PASS
BZW2	7	16646131	16706523	-0.72421718	PASS
C10orf105	10	71711702	71737824	0.032351597	PASS
C10orf113	10	21125763	21146559	0.075711544	PASS
C10orf120	10	122697709	122699822	0.073090211	PASS
C10orf142	10	44292569	44294649	-0.389033546	PASS
C10orf143	10	130020025	130110817	0.073090211	PASS
C10orf53	10	49679651	49710261	-0.389033546	PASS
C10orf55	10	73909969	73922777	0.032351597	PASS
C10orf62	10	97589727	97590934	-0.414937136	PASS
C10orf67	10	23201916	23344845	0.075711544	PASS
C10orf71	10	49299193	49327487	-0.389033546	PASS
C10orf82	10	116663696	116670264	0.073090211	PASS
C10orf88	10	122930903	122954403	0.073090211	PASS
C10orf90	10	126424997	126670446	0.073090211	PASS
C10orf95	10	102449837	102451543	-0.414937136	PASS
C10orf99	10	84173738	84185294	0.032351597	PASS
C11orf1	11	111878935	111885975	-0.431650509	PASS
C11orf16	11	8920076	8933006	-0.39835217	PASS
C11orf21	11	2295645	2303049	-0.39835217	PASS
C11orf24	11	68261335	68272001	0.064535884	PASS
C11orf40	11	4571423	4577820	-0.39835217	PASS
C11orf42	11	6205566	6211132	-0.39835217	PASS
C11orf45	11	128899565	128906035	-0.431650509	PASS
C11orf49	11	46936689	47164385	0.059795455	PASS
C11orf52	11	111918032	111926872	-0.431650509	PASS
C11orf53	11	111245805	111286401	-0.431650509	PASS
C11orf54	11	93741591	93764749	0.064535884	PASS
C11orf58	11	16613132	16756881	-0.39835217	PASS
C11orf65	11	108308519	108467531	-0.431650509	PASS
C11orf68	11	65916808	65919117	0.064535884	PASS
C11orf71	11	114391443	114400511	-0.431650509	PASS
C11orf74	11	36594493	36659290	-0.359426047	PASS
C11orf80	11	66744451	66843328	0.064535884	PASS
C11orf86	11	66975277	66977004	0.064535884	PASS
C11orf87	11	109422120	109429114	-0.431650509	PASS
C11orf88	11	111514785	111537031	-0.431650509	PASS
C11orf91	11	33698261	33700801	-0.359426047	PASS
C11orf94	11	45906513	45907282	0.059795455	PASS
C11orf95	11	63759892	63768775	0.064535884	PASS
C11orf96	11	43942505	43944338	0.059795455	PASS
C11orf97	11	94512432	94532123	0.064535884	PASS
C11orf98	11	62662816	62665217	0.064535884	PASS
C12orf10	12	53299686	53307177	-0.743220499	PASS
C12orf29	12	88033846	88050160	0.271127429	suppress_segment_too_large
C12orf4	12	4487728	4538508	-0.015029783	PASS

C12orf40	12	39626167	39908300	-0.46813528	PASS
C12orf42	12	103237591	103496010	0.028722739	PASS
C12orf43	12	121000486	121016502	0.028722739	PASS
C12orf45	12	104986310	105074197	0.028722739	PASS
C12orf49	12	116710185	116738070	0.028722739	PASS
C12orf50	12	87980035	88034037	0.271127429	suppress_segment_too_large
C12orf54	12	48482498	48496386	-0.743220499	PASS
C12orf56	12	64264762	64391192	-0.743220499	PASS
C12orf57	12	6942978	6946003	-0.015029783	PASS
C12orf60	12	14803572	14906586	0.49032213	suppress_segment_too_large
C12orf65	12	123232916	123257959	0.028722739	PASS
C12orf66	12	64186316	64222296	-0.743220499	PASS
C12orf71	12	27081058	27082514	0.49032213	suppress_segment_too_large
C12orf73	12	103950202	103965708	0.028722739	PASS
C12orf75	12	105235290	105396097	0.028722739	PASS
C12orf76	12	110027028	110073686	0.028722739	PASS
C13orf42	13	51082119	51200252	-0.384313056	PASS
C14orf119	14	23094765	23100462	0.053252507	PASS
C14orf132	14	96039324	96093889	-0.155273506	suppress_segment_too_large
C14orf177	14	98711613	98717766	-0.155273506	suppress_segment_too_large
C14orf178	14	77760830	77769742	-0.155273506	suppress_segment_too_large
C14orf180	14	104579684	104590515	-0.155273506	suppress_segment_too_large
C14orf28	14	44897295	44907257	0.053252507	PASS
C14orf39	14	60396469	60515543	0.053252507	PASS
C14orf93	14	22985908	23010166	0.053252507	PASS
C15orf38-A	15	89834308	89912882	-0.759380635	PASS
C15orf39	15	75195643	75212169	-0.759380635	PASS
C15orf40	15	82988441	83011641	-0.759380635	PASS
C15orf41	15	36579611	36810248	-0.423772929	PASS
C15orf48	15	45430529	45448761	-0.423772929	PASS
C15orf53	15	38628005	39180499	-0.423772929	PASS
C15orf61	15	67521068	67530143	-0.759380635	PASS
C15orf62	15	40770080	40772449	-0.423772929	PASS
C15orf65	15	55408548	55418764	-0.759380635	PASS
C16orf45	16	15434295	15625028	0.053595371	PASS
C16orf46	16	81053497	81077267	-0.423061015	PASS
C16orf54	16	29742463	29746006	0.053595371	PASS
C16orf58	16	31489471	31509309	0.053595371	PASS
C16orf70	16	67109958	67148539	-0.423061015	PASS
C16orf71	16	4734272	4749396	0.053595371	PASS
C16orf72	16	9091648	9121640	0.053595371	PASS
C16orf74	16	85690084	85751129	-0.423061015	PASS
C16orf78	16	49373823	49399431	0.411512417	PASS
C16orf86	16	67666816	67668758	-0.423061015	PASS
C16orf87	16	46796607	46831411	0.053595371	PASS
C16orf89	16	5044122	5066110	0.053595371	PASS
C16orf90	16	3493484	3495480	0.053595371	PASS
C16orf91	16	1419744	1420800	0.053595371	PASS
C16orf92	16	30023334	30027736	0.053595371	PASS
C16orf95	16	87083562	87317420	-0.423061015	PASS
C16orf96	16	4556490	4600714	0.053595371	PASS
C17orf100	17	6651718	6693202	0.06854245	PASS
C17orf107	17	4899418	4902932	0.06854245	PASS
C17orf113	17	42038232	42050614	0.041294512	PASS

C17orf47	17	58541587	58544368	0.051798665	PASS
C17orf49	17	7014495	7017525	0.06854245	PASS
C17orf50	17	35760897	35765079	0.041294512	PASS
C17orf51	17	21428381	21574517	0.041294512	PASS
C17orf53	17	44141906	44162476	0.051798665	PASS
C17orf58	17	67991101	67996431	0.051798665	PASS
C17orf64	17	60392429	60431421	0.051798665	PASS
C17orf67	17	56791913	56838773	0.051798665	PASS
C17orf75	17	32324565	32350023	0.041294512	PASS
C17orf78	17	37375986	37392708	0.041294512	PASS
C17orf80	17	73232233	73248947	0.051798665	PASS
C17orf97	17	410327	431062	0.06854245	PASS
C17orf98	17	38835088	38841455	0.041294512	PASS
C17orf99	17	78146353	78166177	0.051798665	PASS
C18orf21	18	35972083	35979286	-0.330515409	PASS
C18orf25	18	46173553	46266991	-0.330515409	PASS
C18orf32	18	49477250	49487252	-0.330515409	PASS
C18orf54	18	54357917	54385218	-0.330515409	PASS
C18orf63	18	74315813	74359187	-0.330515409	PASS
C19orf12	19	29698886	29715789	0.044838622	PASS
C19orf18	19	57958437	57974534	0.142581352	PASS
C19orf24	19	1275438	1279249	-0.071973357	PASS
C19orf25	19	1461143	1479556	-0.071973357	PASS
C19orf33	19	38304161	38305009	0.048917043	PASS
C19orf38	19	10836575	10869790	-0.107594294	PASS
C19orf44	19	16496311	16521352	-0.069222905	PASS
C19orf47	19	40319536	40348527	0.048917043	PASS
C19orf48	19	50797704	50804929	0.002743503	PASS
C19orf53	19	13774168	13778462	-0.069222905	PASS
C19orf54	19	40740856	40751553	0.048917043	PASS
C19orf57	19	13882348	13906452	-0.069222905	PASS
C19orf66	19	10086122	10093252	-0.107594294	PASS
C19orf67	19	14081619	14085875	-0.069222905	PASS
C19orf70	19	5678421	5680896	-0.071973357	PASS
C19orf71	19	3539154	3544030	-0.071973357	PASS
C19orf73	19	49118397	49119140	0.002743503	PASS
C19orf81	19	50649445	50659310	0.002743503	PASS
C19orf84	19	51388289	51390574	0.002743503	PASS
C1D	2	68041130	68110948	-0.46122572	PASS
C1GALT1	7	7156934	7248651	-0.72421718	PASS
C1GALT1(	23	120625793	120630150	0.158844052	PASS
C1GALT1(	2	43675151	43676322	0.034263343	PASS
C1QA	1	22636506	22639608	0.048749694	PASS
C1QB	1	22652762	22661538	0.048749694	PASS
C1QBP	17	5432777	5448830	0.06854245	PASS
C1QC	1	22643630	22648110	0.048749694	PASS
C1QL1	17	44959693	44968071	0.051798665	PASS
C1QL2	2	119156243	119158889	-0.359440172	PASS
C1QL3	10	16513743	16522005	0.075711544	PASS
C1QL4	12	49332411	49337188	-0.743220499	PASS
C1QTNF1	17	79022814	79049788	0.051798665	PASS
C1QTNF1:	1	1242446	1246722	-0.358281697	PASS
C1QTNF2	5	160347751	160370641	0.165341871	suppress_segment_too_large
C1QTNF3	5	34019448	34043832	0.073527871	PASS

C1QTNF3-	5	33987174	34124528	0.073527871	PASS
C1QTNF4	11	47589664	47594659	0.059795455	PASS
C1QTNF5	11	119338939	119340940	-0.431650509	PASS
C1QTNF6	22	37180167	37199385	-0.740245994	PASS
C1QTNF7	4	15339818	15446166	-0.358623662	PASS
C1QTNF8	16	1090005	1096244	0.053595371	PASS
C1QTNF9	13	24307166	24322535	-0.398748891	PASS
C1QTNF9l	13	23890525	23902655	-0.398748891	PASS
C1R	12	7080199	7092607	-0.015029783	PASS
C1RL	12	7089587	7109273	-0.015029783	PASS
C1S	12	6988259	7071032	-0.015029783	PASS
C1orf100	1	244352635	244389663	-0.145670469	suppress_likely_unfocal_large_gain
C1orf105	1	172420688	172468831	0.088103245	PASS
C1orf109	1	37681570	37692249	0.048749694	PASS
C1orf112	1	169662007	169854080	0.088103245	PASS
C1orf115	1	220689845	220699157	-0.204996646	suppress_segment_too_large
C1orf116	1	207018521	207032756	-0.204996646	suppress_segment_too_large
C1orf122	1	37806979	37809454	0.048749694	PASS
C1orf123	1	53214099	53220617	-0.736943052	PASS
C1orf127	1	10946471	10982037	0.364796772	PASS
C1orf131	1	231223763	231241187	-0.204996646	suppress_segment_too_large
C1orf141	1	67092165	67231853	0.052232535	PASS
C1orf146	1	92217940	92245813	0.079255572	PASS
C1orf158	1	12746215	12763699	0.048749694	PASS
C1orf159	1	1081818	1116361	-0.358281697	PASS
C1orf162	1	111473792	111478512	0.079255572	PASS
C1orf167	1	11761787	11789585	0.364796772	PASS
C1orf174	1	3889125	3900293	-0.358281697	PASS
C1orf185	1	51102221	51148086	-0.736943052	PASS
C1orf189	1	154199372	154206333	0.088103245	PASS
C1orf194	1	109105951	109113857	0.079255572	PASS
C1orf198	1	230837119	230869589	-0.204996646	suppress_segment_too_large
C1orf21	1	184387058	184629020	0.088103245	PASS
C1orf210	1	43281883	43285617	-0.736943052	PASS
C1orf216	1	35713875	35719472	0.048749694	PASS
C1orf226	1	162366908	162386818	0.088103245	PASS
C1orf232	1	26164161	26168581	0.048749694	PASS
C1orf35	1	228100726	228105411	-0.204996646	suppress_segment_too_large
C1orf43	1	154206696	154220637	0.088103245	PASS
C1orf50	1	42767269	42779488	-0.736943052	PASS
C1orf52	1	85249953	85259672	0.587598296	PASS
C1orf53	1	197902647	197907367	0.088103245	PASS
C1orf54	1	150268200	150280916	0.079255572	PASS
C1orf56	1	151047740	151051986	0.079255572	PASS
C1orf61	1	156404250	156430701	0.088103245	PASS
C1orf68	1	152719522	152720470	0.088103245	PASS
C1orf74	1	209779208	209784559	-0.204996646	suppress_segment_too_large
C1orf87	1	59987269	60073770	-0.736943052	PASS
C1orf94	1	34166883	34219131	0.048749694	PASS
C2	6	31897785	31945672	0.028532667	PASS
C20orf141	20	2814987	2815833	-0.172056771	suppress_segment_too_large
C20orf144	20	33662284	33665619	-0.172056771	suppress_segment_too_large
C20orf173	20	35523186	35529652	-0.172056771	suppress_segment_too_large
C20orf194	20	3249305	3407625	-0.172056771	suppress_segment_too_large

C20orf202	20	1203454	1208274	-0.172056771	suppress_segment_too_large
C20orf203	20	32631652	32673941	-0.172056771	suppress_segment_too_large
C20orf204	20	64034344	64039962	-0.172056771	suppress_segment_too_large
C20orf27	20	3753508	3768387	-0.172056771	suppress_segment_too_large
C20orf85	20	58150904	58161150	-0.172056771	suppress_segment_too_large
C20orf96	20	270863	290778	-0.172056771	suppress_segment_too_large
C21orf58	21	46300181	46323875	0.209983559	PASS
C21orf62	21	32793564	32813743	0.209983559	PASS
C21orf91	21	17788967	17819386	0.758930684	suppress_segment_too_large
C22orf15	22	23763021	23765861	-0.740245994	PASS
C22orf23	22	37943050	37953669	-0.740245994	PASS
C22orf31	22	29058672	29061844	-0.740245994	PASS
C22orf39	22	19351368	19448232	-0.740245994	PASS
C22orf42	22	32149006	32159322	-0.740245994	PASS
C22orf46	22	41688939	41698136	-0.740245994	PASS
C2CD2	21	41885112	41953890	0.209983559	PASS
C2CD2L	11	119102198	119118544	-0.431650509	PASS
C2CD3	11	74012714	74171210	0.064535884	PASS
C2CD4A	15	62066977	62070917	-0.759380635	PASS
C2CD4B	15	62163535	62165283	-0.759380635	PASS
C2CD4C	19	405438	409170	-0.071973357	PASS
C2CD4D	1	151837819	151840557	0.079255572	PASS
C2CD5	12	22448583	22544546	0.49032213	suppress_segment_too_large
C2CD6	2	201487425	201619178	0.011001208	PASS
C2orf15	2	99141485	99151487	0.064122364	PASS
C2orf16	2	27537386	27582721	0.034263343	PASS
C2orf40	2	106063246	106078159	-0.439572519	PASS
C2orf42	2	70149880	70248615	-0.46122572	PASS
C2orf49	2	105337359	105349211	-0.439572519	PASS
C2orf50	2	11133053	11146790	0.034263343	PASS
C2orf66	2	196805002	196810276	0.011001208	PASS
C2orf68	2	85606654	85612066	0.064122364	PASS
C2orf69	2	199911256	199955935	0.011001208	PASS
C2orf70	2	26562582	26579532	0.034263343	PASS
C2orf72	2	231037490	231049719	0.011001208	PASS
C2orf73	2	54330034	54383742	0.034263343	PASS
C2orf74	2	61145068	61164829	-0.46122572	PASS
C2orf76	2	119302225	119366828	-0.359440172	PASS
C2orf78	2	73784189	73817147	-0.46122572	PASS
C2orf80	2	208165343	208190073	0.011001208	PASS
C2orf81	2	74414176	74421591	-0.46122572	PASS
C2orf83	2	227610090	227648606	0.011001208	PASS
C2orf88	2	189879609	190203484	0.011001208	PASS
C2orf91	2	41935368	41956806	0.034263343	PASS
C3	19	6677704	6730562	-0.071973357	PASS
C3AR1	12	8058302	8066471	-0.015029783	PASS
C3orf14	3	62318973	62336213	-0.727808231	PASS
C3orf18	3	50558025	50571027	-0.727808231	PASS
C3orf20	3	14675099	14773034	0.06149513	PASS
C3orf22	3	126526999	126558965	0.06434429	PASS
C3orf30	3	119146150	119160042	0.06434429	PASS
C3orf33	3	155762617	155806351	0.02267528	PASS
C3orf38	3	88149743	88168729	0.22470242	PASS
C3orf49	3	63819362	63848636	-0.727808231	PASS

C3orf52	3	112086335	112131004		0.06434429	PASS
C3orf56	3	127193131	127198185		0.06434429	PASS
C3orf58	3	143971798	144048719		0.02267528	PASS
C3orf62	3	49268602	49277909		-0.727808231	PASS
C3orf67	3	58717365	59050084		-0.727808231	PASS
C3orf70	3	185078050	185153014		0.02267528	PASS
C3orf80	3	160225496	160228213		0.02267528	PASS
C3orf84	3	49177632	49191858		-0.727808231	PASS
C3orf85	3	109118252	109150514		0.22470242	PASS
C4A	6	31982024	32002681		0.028532667	PASS
C4B	6	32014795	32035418		0.028532667	PASS
C4BPA	1	207104262	207144972		-0.204996646	suppress_segment_too_large
C4BPB	1	207088842	207099993		-0.204996646	suppress_segment_too_large
C4orf17	4	99511004	99542303		-0.677982248	PASS
C4orf19	4	37453941	37623495		-0.753159364	PASS
C4orf3	4	119296419	119304445		-0.754119944	PASS
C4orf33	4	129093317	129116640		0.142431244	suppress_segment_too_large
C4orf36	4	86876205	86892422		-0.677982248	PASS
C4orf45	4	158893134	159038760		0.142431244	suppress_segment_too_large
C4orf46	4	158666679	158672255		0.142431244	suppress_segment_too_large
C4orf47	4	185426249	185449826		0.275137421	PASS
C4orf48	4	2041993	2043970		-0.358623662	PASS
C4orf50	4	5897373	6200555		-0.358623662	PASS
C4orf51	4	145680115	145771032		0.142431244	suppress_segment_too_large
C4orf54	4	99636529	99654648		-0.677982248	PASS
C5	9	120952335	121050276		0.073960965	PASS
C5AR1	19	47290023	47322066		0.002743503	PASS
C5AR2	19	47332147	47347327		0.002743503	PASS
C5orf15	5	133955502	133968787		-0.393084436	PASS
C5orf22	5	31532266	31555058		0.073527871	PASS
C5orf24	5	134845680	134859737		-0.393084436	PASS
C5orf30	5	103258702	103278660		-0.393084436	PASS
C5orf34	5	43486701	43515145		-0.284712224	PASS
C5orf38	5	2752131	2755397		0.073527871	PASS
C5orf46	5	147880726	147906538		-0.393084436	PASS
C5orf47	5	173973779	174006140		0.165341871	suppress_segment_too_large
C5orf49	5	7830378	7851490		0.073527871	PASS
C5orf51	5	41904188	41921636		-0.284712224	PASS
C5orf52	5	157671553	157680143		0.165341871	suppress_segment_too_large
C5orf58	5	170232447	170252575		0.165341871	suppress_segment_too_large
C5orf60	5	179641544	179645046		0.165341871	suppress_segment_too_large
C5orf63	5	127042558	127073492		-0.393084436	PASS
C5orf67	5	56511567	56606232		-0.284712224	PASS
C6	5	41142234	41261438		-0.284712224	PASS
C6orf10	6	32288526	32371912		0.028532667	PASS
C6orf106	6	34587288	34696859		0.028532667	PASS
C6orf118	6	165279664	165309607		0.033894706	PASS
C6orf120	6	169702190	169704856		0.033894706	PASS
C6orf132	6	42101118	42142619		0.028532667	PASS
C6orf136	6	30647039	30653210		0.069043418	PASS
C6orf141	6	49550646	49561907		0.239805577	suppress_segment_too_large
C6orf15	6	31111223	31112559		0.069043418	PASS
C6orf163	6	87344849	87365463		-0.355603029	PASS
C6orf201	6	4079206	4130951		0.069043418	PASS

C6orf203	6	107028213	107051342	-0.355603029	PASS
C6orf222	6	36315757	36336885	0.028532667	PASS
C6orf223	6	44000580	44005958	0.028532667	PASS
C6orf226	6	42890265	42890816	0.028532667	PASS
C6orf47	6	31658298	31660772	0.028532667	PASS
C6orf48	6	31834608	31839766	0.028532667	PASS
C6orf52	6	10671418	10694797	0.069043418	PASS
C6orf58	6	127519455	127591817	-0.304168115	PASS
C6orf62	6	24704861	24720836	0.069043418	PASS
C6orf89	6	36871870	36928964	0.028532667	PASS
C7	5	40909252	40982939	-0.284712224	PASS
C7orf25	7	42908726	42912305	-0.72421718	PASS
C7orf26	7	6590017	6608726	-0.72421718	PASS
C7orf31	7	25134697	25180356	-0.72421718	PASS
C7orf33	7	148590565	148615860	-0.712149877	PASS
C7orf43	7	100154420	100158715	-0.710463272	PASS
C7orf50	7	996986	1138260	-0.72421718	PASS
C7orf55-LU	7	139341360	139422599	-0.712149877	PASS
C7orf57	7	48035511	48061304	-0.72421718	PASS
C7orf61	7	100456615	100464271	-0.710463272	PASS
C7orf77	7	124777292	124790810	-0.712149877	PASS
C8A	1	56854806	56918221	-0.736943052	PASS
C8B	1	56929210	56966140	-0.736943052	PASS
C8G	9	136945185	136946975	0.073960965	PASS
C8orf33	8	145052378	145066685	0.699132336	PASS
C8orf34	8	68330722	68819022	0.182873287	suppress_segment_too_large
C8orf37	8	95244919	95269201	0.355469066	suppress_segment_too_large
C8orf44	8	66667615	66685564	0.182873287	suppress_segment_too_large
C8orf48	8	13566843	13568287	-0.311940419	PASS
C8orf58	8	22599601	22604150	-0.311940419	PASS
C8orf59	8	85214076	85220421	0.617295349	suppress_segment_too_large
C8orf74	8	10672637	10700593	-0.311940419	PASS
C8orf76	8	123219957	123241398	0.493344802	suppress_segment_too_large
C8orf82	8	144525733	144529132	0.699132336	PASS
C8orf86	8	38510834	38560939	0.182873287	suppress_segment_too_large
C8orf88	8	90958637	90985257	0.617295349	suppress_segment_too_large
C8orf89	8	73241329	73259502	0.182873287	suppress_segment_too_large
C9	5	39284262	39424868	0.073527871	PASS
C9orf116	9	135495181	135501734	0.073960965	PASS
C9orf129	9	93318199	93346414	0.073960965	PASS
C9orf131	9	35041095	35045991	0.073960965	PASS
C9orf135	9	69820793	69906232	0.073960965	PASS
C9orf152	9	110190048	110208189	0.073960965	PASS
C9orf153	9	86220265	86259657	0.073960965	PASS
C9orf16	9	128160260	128163928	0.073960965	PASS
C9orf24	9	34379019	34397832	0.073960965	PASS
C9orf3	9	94726701	95087218	0.073960965	PASS
C9orf40	9	74946581	74952886	0.073960965	PASS
C9orf43	9	113410054	113429684	0.073960965	PASS
C9orf50	9	129612225	129620776	0.073960965	PASS
C9orf57	9	72051376	72072721	0.073960965	PASS
C9orf64	9	83938311	83956986	0.073960965	PASS
C9orf66	9	212824	215893	0.073960965	PASS
C9orf72	9	27546545	27573866	0.073960965	PASS

C9orf78	9	129827290	129835863	0.073960965	PASS
C9orf84	9	111686173	111795008	0.073960965	PASS
C9orf85	9	71911510	71986054	0.073960965	PASS
C9orf92	9	16203935	16410990	0.073960965	PASS
CA1	8	85327608	85379014	0.617295349	suppress_segment_too_large
CA10	17	51630313	52160017	0.051798665	PASS
CA11	19	48637942	48646312	0.002743503	PASS
CA12	15	63321378	63382161	-0.759380635	PASS
CA13	8	85220587	85284073	0.617295349	suppress_segment_too_large
CA14	1	150257159	150265078	0.079255572	PASS
CA2	8	85463852	85481493	0.617295349	suppress_segment_too_large
CA3	8	85373436	85449040	0.617295349	suppress_segment_too_large
CA4	17	60149936	60170899	0.051798665	PASS
CA5A	16	87881546	87936580	-0.423061015	PASS
CA5B	23	15688830	15788409	0.158844052	PASS
CA6	1	8945867	8975092	0.364796772	PASS
CA7	16	66844379	66854153	-0.423061015	PASS
CA8	8	60187347	60281412	0.182873287	suppress_segment_too_large
CA9	9	35673856	35681159	0.073960965	PASS
CAAP1	9	26840685	26892804	0.073960965	PASS
CAB39	2	230712845	230821075	0.011001208	PASS
CAB39L	13	49308650	49444126	-0.384313056	PASS
CABCOC(	10	61662961	61766766	0.032351597	PASS
CABIN1	22	24011192	24178628	-0.740245994	PASS
CABLES1	18	23134564	23260467	-0.330515409	PASS
CABLES2	20	62388632	62407285	-0.172056771	suppress_segment_too_large
CABP1	12	120640552	120667324	0.028722739	PASS
CABP2	11	67518912	67524517	0.064535884	PASS
CABP4	11	67452406	67460313	0.064535884	PASS
CABP5	19	48029953	48044053	0.002743503	PASS
CABP7	22	29720084	29731839	-0.740245994	PASS
CABS1	4	70334966	70337116	0.115781418	suppress_segment_too_large
CABYR	18	24138956	24161603	-0.330515409	PASS
CACFD1	9	133459965	133470848	0.073960965	PASS
CACHD1	1	64470792	64693058	0.052232535	PASS
CACNA1A	19	13206442	13633025	-0.069222905	PASS
CACNA1E	9	137877789	138124624	0.073960965	PASS
CACNA1C	12	1970786	2697950	-0.015029783	PASS
CACNA1D	3	53328963	53813733	-0.727808231	PASS
CACNA1E	1	181317690	181808084	0.088103245	PASS
CACNA1F	23	49205063	49233371	0.158844052	PASS
CACNA1C	17	50561068	50627474	0.051798665	PASS
CACNA1H	16	1153121	1221772	0.053595371	PASS
CACNA1I	22	39570753	39689737	-0.740245994	PASS
CACNA1S	1	201039512	201112566	-0.204996646	suppress_segment_too_large
CACNA2D	7	81946444	82443798	-0.701511252	PASS
CACNA2D	3	50362799	50504244	-0.727808231	PASS
CACNA2D	3	54122547	55074557	-0.727808231	PASS
CACNA2D	12	1791957	1918836	-0.015029783	PASS
CACNB1	17	39173456	39197703	0.041294512	PASS
CACNB2	10	18140677	18543557	0.075711544	PASS
CACNB3	12	48813794	48828941	-0.743220499	PASS
CACNB4	2	151832768	152099475	-0.30549516	PASS
CACNG1	17	67044590	67056797	0.051798665	PASS

CACNG2	22	36563921	36703558	-0.740245994	PASS
CACNG3	16	24255553	24362801	0.053595371	PASS
CACNG4	17	66964910	67033398	0.051798665	PASS
CACNG5	17	66835117	66885486	0.051798665	PASS
CACNG6	19	53992288	54012669	0.142581352	PASS
CACNG7	19	53909335	53943941	0.142581352	PASS
CACNG8	19	53963040	53990215	0.142581352	PASS
CACTIN	19	3610641	3626815	-0.071973357	PASS
CACUL1	10	118674167	118755249	0.073090211	PASS
CACYBP	1	174999163	175011715	0.088103245	PASS
CAD	2	27217390	27243943	0.034263343	PASS
CADM1	11	115169218	115504957	-0.431650509	PASS
CADM2	3	84958981	86074429	0.22470242	PASS
CADM3	1	159171609	159203313	0.088103245	PASS
CADM4	19	43622368	43639839	0.048917043	PASS
CADPS	3	62398346	62875389	-0.727808231	PASS
CADPS2	7	122318425	122886759	-0.712149877	PASS
CAGE1	6	7326656	7389743	0.069043418	PASS
CALB1	8	90058608	90095475	0.617295349	suppress_segment_too_large
CALB2	16	71358713	71390438	-0.423061015	PASS
CALCA	11	14966668	14972354	-0.39835217	PASS
CALCB	11	14904997	15082342	-0.39835217	PASS
CALCOC(	12	53708517	53727745	-0.743220499	PASS
CALCOC(	17	48830988	48866522	0.051798665	PASS
CALCR	7	93424487	93574730	-0.710463272	PASS
CALCRL	2	187343129	187448460	-0.030005025	PASS
CALD1	7	134744252	134970728	-0.712149877	PASS
CALHM1	10	103453387	103458888	-0.414937136	PASS
CALHM2	10	103446786	103452402	-0.414937136	PASS
CALHM3	10	103472804	103479240	-0.414937136	PASS
CALHM4	6	116529013	116558868	-0.355603029	PASS
CALHM5	6	116511646	116524792	-0.355603029	PASS
CALHM6	6	116461370	116463779	-0.355603029	PASS
CALM1	14	90396502	90408261	-0.155273506	suppress_segment_too_large
CALM2	2	47160082	47176601	0.034263343	PASS
CALM3	19	46601074	46610793	0.002743503	PASS
CALML3	10	5524009	5526771	0.075711544	PASS
CALML4	15	68190705	68206110	-0.759380635	PASS
CALML5	10	5498697	5499555	0.075711544	PASS
CALML6	1	1914827	1917296	-0.358281697	PASS
CALN1	7	71779491	72447151	-0.701511252	PASS
CALR	19	12938578	12944489	-0.069222905	PASS
CALR3	19	16479057	16496192	-0.069222905	PASS
CALU	7	128739292	128771807	-0.712149877	PASS
CALY	10	133324072	133336935	0.073090211	PASS
CAMK1	3	9757342	9769992	0.06149513	PASS
CAMK1D	10	12349482	12835545	0.075711544	PASS
CAMK1G	1	209583717	209613938	-0.204996646	suppress_segment_too_large
CAMK2A	5	150219491	150290291	-0.393084436	PASS
CAMK2B	7	44217150	44334577	-0.72421718	PASS
CAMK2D	4	113451032	113761927	-0.677982248	PASS
CAMK2G	10	73812501	73874591	0.032351597	PASS
CAMK2N1	1	20482391	20486220	0.048749694	PASS
CAMK2N2	3	184259213	184261463	0.02267528	PASS

CAMK4	5	111223653	111494886	-0.393084436	PASS
CAMKK1	17	3860315	3894891	0.06854245	PASS
CAMKK2	12	121237691	121298308	0.028722739	PASS
CAMKMT	2	44361950	44772592	0.034263343	PASS
CAMKV	3	49857988	49870222	-0.727808231	PASS
CAMLG	5	134738501	134752160	-0.393084436	PASS
CAMP	3	48223347	48225491	-0.727808231	PASS
CAMSAP1	9	135808487	135907228	0.073960965	PASS
CAMSAP2	1	200739558	200860704	-0.204996646	suppress_segment_too_large
CAMSAP3	19	7595902	7618304	-0.071973357	PASS
CAMTA1	1	6785324	7769706	0.364796772	PASS
CAMTA2	17	4967992	4987652	0.06854245	PASS
CAND1	12	67269281	67319951	-0.743220499	PASS
CAND2	3	12796472	12871916	0.06149513	PASS
CANT1	17	78991717	79009867	0.051798665	PASS
CANX	5	179678628	179730925	0.165341871	suppress_segment_too_large
CAP1	1	40040233	40072649	0.048749694	PASS
CAP2	6	17393216	17557792	0.069043418	PASS
CAPG	2	85394748	85418432	0.064122364	PASS
CAPN1	11	65180566	65212006	0.064535884	PASS
CAPN10	2	240586716	240617705	0.011001208	PASS
CAPN11	6	44158811	44184402	0.028532667	PASS
CAPN12	19	38730187	38769904	0.048917043	PASS
CAPN13	2	30722771	30820542	0.034263343	PASS
CAPN14	2	31173056	31233858	0.034263343	PASS
CAPN15	16	527717	554636	0.053595371	PASS
CAPN2	1	223701593	223776018	-0.204996646	suppress_segment_too_large
CAPN3	15	42359500	42412318	-0.423772929	PASS
CAPN5	11	77066941	77126155	0.064535884	PASS
CAPN6	23	111245103	111270523	0.158844052	PASS
CAPN7	3	15206152	15252918	0.06149513	PASS
CAPN8	1	223538007	223665734	-0.204996646	suppress_segment_too_large
CAPN9	1	230747384	230802003	-0.204996646	suppress_segment_too_large
CAPNS1	19	36139575	36150353	0.044838622	PASS
CAPNS2	16	55566672	55567687	-0.423061015	PASS
CAPRIN1	11	34051683	34101156	-0.359426047	PASS
CAPRIN2	12	30709552	30754951	0.49032213	suppress_segment_too_large
CAPS	19	5911707	5915877	-0.071973357	PASS
CAPS2	12	75275979	75390928	0.271127429	suppress_segment_too_large
CAPSL	5	35904295	35938779	0.073527871	PASS
CAPZA1	1	112619173	112671619	0.079255572	PASS
CAPZA2	7	116811070	116922049	-0.712149877	PASS
CAPZA3	12	18738101	18739187	0.49032213	suppress_segment_too_large
CAPZB	1	19338776	19485539	0.048749694	PASS
CARD10	22	37490362	37519542	-0.740245994	PASS
CARD11	7	2906141	3043945	-0.72421718	PASS
CARD14	17	80169992	80209331	0.051798665	PASS
CARD16	11	105041326	105101431	0.32805343	suppress_segment_too_large
CARD17	11	105092469	105101431	0.32805343	suppress_segment_too_large
CARD18	11	105137721	105531697	0.32805343	suppress_segment_too_large
CARD19	9	93096218	93113283	0.073960965	PASS
CARD6	5	40841184	40860175	-0.284712224	PASS
CARD8	19	48180770	48255946	0.002743503	PASS
CARD9	9	136361903	136373681	0.073960965	PASS

CARF	2	202912214	202987063	0.011001208	PASS
CARHSP1	16	8852942	8869012	0.053595371	PASS
CARM1	19	10871513	10923070	-0.107594294	PASS
CARMIL1	6	25279078	25620530	0.069043418	PASS
CARMIL2	16	67644919	67657569	-0.423061015	PASS
CARMIL3	14	24052000	24069728	0.053252507	PASS
CARNMT	9	74981020	75028423	0.073960965	PASS
CARNS1	11	67414968	67425607	0.064535884	PASS
CARS	11	3000922	3057613	-0.39835217	PASS
CARS2	13	110641412	110713603	-0.426822582	PASS
CARTPT	5	71719163	71721048	-0.393084436	PASS
CASC1	12	25108420	25195162	0.49032213	suppress_segment_too_large
CASC10	10	21492658	21497262	0.075711544	PASS
CASC3	17	40140318	40172183	0.041294512	PASS
CASC4	15	44288729	44415758	-0.423772929	PASS
CASD1	7	94509219	94557019	-0.710463272	PASS
CASK	23	41514934	41923645	0.158844052	PASS
CASKIN1	16	2177180	2196525	0.053595371	PASS
CASKIN2	17	75500261	75515583	0.051798665	PASS
CASP1	11	105025443	105035250	0.32805343	suppress_segment_too_large
CASP10	2	201182881	201229406	0.011001208	PASS
CASP14	19	15049384	15058293	-0.069222905	PASS
CASP2	7	143288215	143307696	-0.712149877	PASS
CASP3	4	184627696	184649509	-0.453876349	PASS
CASP4	11	104942866	104969436	0.32805343	suppress_segment_too_large
CASP5	11	104994235	105023168	0.32805343	suppress_segment_too_large
CASP6	4	109688622	109703583	-0.677982248	PASS
CASP7	10	113679162	113730907	0.073090211	PASS
CASP8	2	201233443	201287711	0.011001208	PASS
CASP8AP	6	89829894	89874436	-0.355603029	PASS
CASP9	1	15490832	15526534	0.048749694	PASS
CASQ1	1	160190556	160201886	0.088103245	PASS
CASQ2	1	115700007	115768781	0.079255572	PASS
CASR	3	122183683	122291629	0.06434429	PASS
CASS4	20	56412112	56460387	-0.172056771	suppress_segment_too_large
CAST	5	96525267	96779595	-0.393084436	PASS
CASTOR1	22	30285117	30289627	-0.740245994	PASS
CASTOR2	7	74964818	75024798	-0.701511252	PASS
CASTOR3	7	100200653	100272232	-0.710463272	PASS
CASZ1	1	10636604	10796650	0.364796772	PASS
CAT	11	34438925	34472062	-0.359426047	PASS
CATIP	2	218356856	218368099	0.011001208	PASS
CATSPER	11	66016752	66026517	0.064535884	PASS
CATSPER	15	43628503	43668118	-0.423772929	PASS
CATSPER	5	134967906	135011707	-0.393084436	PASS
CATSPER	1	26190561	26202968	0.048749694	PASS
CATSPER	14	91580696	91780707	-0.155273506	suppress_segment_too_large
CATSPER	19	5720677	5778734	-0.071973357	PASS
CATSPER	1	244454377	244641177	-0.145670469	suppress_likely_unfocal_large_gain
CATSPER	19	38335775	38370943	0.048917043	PASS
CATSPER	11	64300391	64304770	0.064535884	PASS
CAV1	7	116524785	116561184	-0.712149877	PASS
CAV2	7	116287380	116508541	-0.712149877	PASS
CAV3	3	8733800	8841808	0.06149513	PASS

CAVIN1	17	42402452	42423517	0.041294512	PASS
CAVIN2	2	191834302	191847255	0.011001208	PASS
CAVIN3	11	6318946	6320647	-0.39835217	PASS
CAVIN4	9	100578079	100587906	0.073960965	PASS
CBARP	19	1228287	1238027	-0.071973357	PASS
CBFA2T2	20	33490075	33650036	-0.172056771	suppress_segment_too_large
CBFA2T3	16	88874858	88977204	-0.423061015	PASS
CBFB	16	67029116	67101058	-0.423061015	PASS
CBL	11	119206276	119313926	-0.431650509	PASS
CBLB	3	105655461	105869552	0.22470242	PASS
CBLC	19	44777869	44800652	0.002743503	PASS
CBLL1	7	107743697	107761667	-0.712149877	PASS
CBLL2	23	22272943	22274461	0.158844052	PASS
CBLN1	16	49277917	49281831	0.411512417	PASS
CBLN2	18	72536680	72638521	-0.330515409	PASS
CBLN3	14	24426532	24430954	0.053252507	PASS
CBLN4	20	55997440	56005472	-0.172056771	suppress_segment_too_large
CBR1	21	36069941	36073166	0.209983559	PASS
CBR3	21	36134912	36146566	0.209983559	PASS
CBR4	4	168863770	169010275	0.142431244	suppress_segment_too_large
CBS	21	43053191	43076943	0.209983559	PASS
CBSL	21	6444869	6468040	0.220071762	PASS
CBWD1	9	121038	179147	0.073960965	PASS
CBWD2	2	113437691	113496189	0.040971718	PASS
CBWD3	9	68232003	68300015	0.073960965	PASS
CBWD5	9	65668805	65734041	0.073960965	PASS
CBWD6	9	41131306	41199261	0.073960965	PASS
CBX1	17	48070052	48101521	0.051798665	PASS
CBX2	17	79778132	79787983	0.051798665	PASS
CBX3	7	26201162	26213356	-0.72421718	PASS
CBX4	17	79833156	79839429	0.051798665	PASS
CBX5	12	54230940	54280133	-0.743220499	PASS
CBX6	22	38861450	38872314	-0.740245994	PASS
CBX7	22	39120167	39152674	-0.740245994	PASS
CBX8	17	79792132	79801683	0.051798665	PASS
CBY1	22	38656636	38673854	-0.740245994	PASS
CBY3	5	179678583	179680974	0.165341871	suppress_segment_too_large
CC2D1A	19	13906201	13930879	-0.069222905	PASS
CC2D1B	1	52345723	52366193	-0.736943052	PASS
CC2D2A	4	15469865	15601557	-0.358623662	PASS
CC2D2B	10	95907603	96032684	-0.414937136	PASS
CCAR1	10	68721012	68792377	0.032351597	PASS
CCAR2	8	22604632	22621514	-0.311940419	PASS
CCBE1	18	59430939	59697662	-0.330515409	PASS
CCDC102A	16	57512178	57536599	-0.423061015	PASS
CCDC102B	18	68715209	69055189	-0.330515409	PASS
CCDC103	17	44899142	44905390	0.051798665	PASS
CCDC105	19	15010744	15023269	-0.069222905	PASS
CCDC106	19	55641062	55653161	0.142581352	PASS
CCDC107	9	35658290	35661511	0.073960965	PASS
CCDC110	4	185445182	185471759	0.275137421	PASS
CCDC112	5	115267188	115296831	-0.393084436	PASS
CCDC113	16	58231157	58283836	-0.423061015	PASS
CCDC114	19	48296457	48321894	0.002743503	PASS

CCDC115	2	130338241	130342349	-0.359440172	PASS
CCDC116	22	21632716	21637327	-0.740245994	PASS
CCDC117	22	28772674	28789301	-0.740245994	PASS
CCDC12	3	46921726	46982010	-0.727808231	PASS
CCDC120	23	49053572	49069857	0.158844052	PASS
CCDC121	2	27625639	27629012	0.034263343	PASS
CCDC122	13	43823909	43879727	-0.398748891	PASS
CCDC124	19	17933016	17943991	-0.069222905	PASS
CCDC125	5	69280175	69332809	-0.393084436	PASS
CCDC126	7	23597379	23644708	-0.72421718	PASS
CCDC127	5	196871	218215	0.073527871	PASS
CCDC13	3	42708269	42773253	-0.727808231	PASS
CCDC130	19	13731760	13763296	-0.069222905	PASS
CCDC134	22	41800679	41826299	-0.740245994	PASS
CCDC136	7	128790757	128822132	-0.712149877	PASS
CCDC137	17	81666364	81673904	0.051798665	PASS
CCDC138	2	108786757	108885477	0.040971718	PASS
CCDC14	3	123897305	123961408	0.06434429	PASS
CCDC140	2	222298147	222305217	0.011001208	PASS
CCDC141	2	178829757	179050086	0.162734012	PASS
CCDC142	2	74471986	74483408	-0.46122572	PASS
CCDC144A	17	16689537	16777881	0.06854245	PASS
CCDC144N	17	20836447	20896140	0.06854245	PASS
CCDC146	7	77122434	77329533	-0.701511252	PASS
CCDC148	2	158171081	158456753	0.054749241	PASS
CCDC149	4	24806117	24980204	-0.358623662	PASS
CCDC15	11	124954121	125041489	-0.431650509	PASS
CCDC150	2	196639554	196763490	0.011001208	PASS
CCDC151	19	11420604	11435782	-0.107594294	PASS
CCDC152	5	42756801	42802360	-0.284712224	PASS
CCDC153	11	119189638	119196769	-0.431650509	PASS
CCDC154	16	1434383	1444556	0.053595371	PASS
CCDC155	19	49388218	49417994	0.002743503	PASS
CCDC157	22	30356635	30378658	-0.740245994	PASS
CCDC158	4	76312997	76421868	-0.677982248	PASS
CCDC159	19	11344684	11354944	-0.107594294	PASS
CCDC160	23	134237047	134246207	0.158844052	PASS
CCDC163	1	45493866	45500079	-0.736943052	PASS
CCDC166	8	143706694	143708109	0.699132336	PASS
CCDC167	6	37482920	37499922	0.028532667	PASS
CCDC168	13	102729369	102759070	1.049215493	PASS
CCDC169	13	36222008	36297840	-0.398748891	PASS
CCDC169-	13	36168794	36297842	-0.398748891	PASS
CCDC17	1	45620044	45624057	-0.736943052	PASS
CCDC170	6	151494030	151621193	0.033894706	PASS
CCDC171	9	15553086	16061663	0.073960965	PASS
CCDC172	10	116324428	116380029	0.073090211	PASS
CCDC173	2	169645425	169694433	0.033541467	PASS
CCDC174	3	14651746	14672659	0.06149513	PASS
CCDC175	14	59504539	59576831	0.053252507	PASS
CCDC177	14	69569816	69574837	0.053252507	PASS
CCDC178	18	32937402	33441101	-0.330515409	PASS
CCDC179	11	22846931	22860426	-2.208422516	PASS
CCDC18	1	93179919	93278730	0.079255572	PASS

CCDC180	9	97307304	97378524	0.073960965	PASS
CCDC181	1	169394870	169460669	0.088103245	PASS
CCDC182	17	57744481	57745312	0.051798665	PASS
CCDC183	9	136796350	136807741	0.073960965	PASS
CCDC184	12	48183584	48185926	-0.743220499	PASS
CCDC185	1	223393417	223395470	-0.204996646	suppress_segment_too_large
CCDC186	10	114120862	114174232	0.073090211	PASS
CCDC187	9	136249971	136306901	0.073960965	PASS
CCDC188	22	20148427	20151065	-0.740245994	PASS
CCDC189	16	30757423	30762710	0.053595371	PASS
CCDC190	1	162824458	162868815	0.088103245	PASS
CCDC191	3	113964137	114056613	0.06434429	PASS
CCDC192	5	127703389	127941634	-0.393084436	PASS
CCDC196	14	66411704	66498677	0.053252507	PASS
CCDC198	14	57469301	57493867	0.053252507	PASS
CCDC200	17	43216941	43305397	0.051798665	PASS
CCDC22	23	49235467	49250526	0.158844052	PASS
CCDC24	1	43991359	43996528	-0.736943052	PASS
CCDC25	8	27733311	27772653	-0.311940419	PASS
CCDC27	1	3746460	3771645	-0.358281697	PASS
CCDC28A	6	138773509	138793319	0.033894706	PASS
CCDC28B	1	32200386	32205387	0.048749694	PASS
CCDC3	10	12896625	13099652	0.075711544	PASS
CCDC30	1	42463330	42654664	-0.736943052	PASS
CCDC32	15	40528683	40565057	-0.423772929	PASS
CCDC33	15	74202705	74336472	-0.759380635	PASS
CCDC34	11	27330827	27363868	-0.359426047	PASS
CCDC36	3	49198428	49258104	-0.727808231	PASS
CCDC38	12	95867048	95942974	0.028722739	PASS
CCDC39	3	180602858	180870933	0.02267528	PASS
CCDC40	17	80036632	80100613	0.051798665	PASS
CCDC42	17	8729934	8745219	0.06854245	PASS
CCDC43	17	44673069	44689779	0.051798665	PASS
CCDC47	17	63745250	63776351	0.051798665	PASS
CCDC50	3	191329077	191398670	0.02267528	PASS
CCDC51	3	48432164	48440456	-0.727808231	PASS
CCDC54	3	107377194	107378637	0.22470242	PASS
CCDC57	17	82101460	82212830	0.051798665	PASS
CCDC58	3	122359591	122383231	0.06434429	PASS
CCDC59	12	82223681	82358805	0.271127429	suppress_segment_too_large
CCDC6	10	59788763	59906656	0.032351597	PASS
CCDC60	12	119334712	119541047	0.028722739	PASS
CCDC61	19	45995461	46021318	0.002743503	PASS
CCDC62	12	122774327	122827528	0.028722739	PASS
CCDC63	12	110846769	110907535	0.028722739	PASS
CCDC65	12	48904110	48931840	-0.743220499	PASS
CCDC66	3	56557161	56621818	-0.727808231	PASS
CCDC68	18	54901509	54959508	-0.330515409	PASS
CCDC69	5	151181052	151224145	-0.393084436	PASS
CCDC7	10	32446140	32882874	0.075711544	PASS
CCDC70	13	51861981	51866232	-0.384313056	PASS
CCDC71	3	49162535	49166321	-0.727808231	PASS
CCDC71L	7	106656765	106660996	-0.712149877	PASS
CCDC73	11	32602246	32794658	-0.359426047	PASS

CCDC74A	2	131527675	131533666	-0.359440172	PASS
CCDC74B	2	130139287	130145134	-0.359440172	PASS
CCDC77	12	389273	442645	-0.015029783	PASS
CCDC78	16	722582	726954	0.053595371	PASS
CCDC8	19	46410329	46413662	0.002743503	PASS
CCDC80	3	112596794	112649530	0.06434429	PASS
CCDC81	11	86374736	86423109	0.064535884	PASS
CCDC82	11	96352769	96389956	0.32805343	suppress_segment_too_large
CCDC83	11	85855101	85920021	0.064535884	PASS
CCDC84	11	118998142	119015791	-0.431650509	PASS
CCDC85A	2	56184123	56386173	-0.46122572	PASS
CCDC85B	11	65890112	65891635	0.064535884	PASS
CCDC85C	14	99500180	99604026	-0.155273506	suppress_segment_too_large
CCDC86	11	60842071	60851081	0.064535884	PASS
CCDC87	11	66590176	66593083	0.064535884	PASS
CCDC88A	2	55287842	55419895	-0.052193497	PASS
CCDC88B	11	64340223	64357534	0.064535884	PASS
CCDC88C	14	91271323	91417844	-0.155273506	suppress_segment_too_large
CCDC89	11	85684866	85686277	0.064535884	PASS
CCDC9	19	47255980	47273701	0.002743503	PASS
CCDC90B	11	83259097	83286407	0.064535884	PASS
CCDC91	12	28133249	28581511	0.49032213	suppress_segment_too_large
CCDC92	12	123918660	123972831	0.028722739	PASS
CCDC93	2	117915478	118014133	-0.359440172	PASS
CCDC96	4	7040849	7042939	-0.358623662	PASS
CCDC97	19	41310189	41324883	0.048917043	PASS
CCDC9B	15	40331452	40340967	-0.423772929	PASS
CCER1	12	90905622	90955176	0.271127429	suppress_segment_too_large
CCER2	19	38908980	38912158	0.048917043	PASS
CCHCR1	6	31142439	31158238	0.069043418	PASS
CCIN	9	36169394	36171334	0.073960965	PASS
CCK	3	42257825	42266207	-0.727808231	PASS
CCKAR	4	26481400	26490462	-2.804710927	PASS
CCKBR	11	6259736	6272127	-0.39835217	PASS
CCL1	17	34360328	34363231	0.041294512	PASS
CCL11	17	34285668	34288334	0.041294512	PASS
CCL13	17	34356452	34358610	0.041294512	PASS
CCL14	17	35983291	35987004	0.041294512	PASS
CCL15	17	35996440	36002038	0.041294512	PASS
CCL15-CC	17	35983656	36001621	0.041294512	PASS
CCL16	17	35976493	35981496	0.041294512	PASS
CCL17	16	57404767	57416062	-0.423061015	PASS
CCL18	17	36064280	36072032	0.041294512	PASS
CCL19	9	34689567	34691277	0.073960965	PASS
CCL2	17	34255218	34257203	0.041294512	PASS
CCL20	2	227805739	227817564	0.011001208	PASS
CCL21	9	34709005	34710124	0.073960965	PASS
CCL22	16	57358772	57366190	-0.423061015	PASS
CCL23	17	36013056	36017968	0.041294512	PASS
CCL24	7	75811665	75823356	-0.701511252	PASS
CCL25	19	8052767	8062650	-0.071973357	PASS
CCL26	7	75769533	75789896	-0.701511252	PASS
CCL27	9	34661880	34664048	0.073960965	PASS
CCL28	5	43376645	43412391	-0.284712224	PASS

CCL3	17	36088256	36090169	0.041294512	PASS
CCL3L1	17	36194869	36196758	0.041294512	PASS
CCL4	17	36103590	36105621	0.041294512	PASS
CCL4L2	17	36210924	36212878	0.041294512	PASS
CCL5	17	35871491	35880793	0.041294512	PASS
CCL7	17	34270221	34272242	0.041294512	PASS
CCL8	17	34319036	34321402	0.041294512	PASS
CCM2	7	44999475	45076469	-0.72421718	PASS
CCM2L	20	32010450	32032180	-0.172056771	suppress_segment_too_large
CCNA1	13	36431520	36442882	-0.398748891	PASS
CCNA2	4	121816444	121823933	0.142431244	suppress_segment_too_large
CCNB1	5	69167010	69178245	-0.393084436	PASS
CCNB1IP1	14	20311368	20333312	0.053252507	PASS
CCNB2	15	59105078	59125045	-0.759380635	PASS
CCNB3	23	50202713	50351910	0.158844052	PASS
CCNC	6	99542380	99568973	-0.355603029	PASS
CCND1	11	69641087	69654474	0.064535884	PASS
CCND2	12	4273772	4305350	-0.015029783	PASS
CCND3	6	41934933	42050357	0.028532667	PASS
CCNDBP1	15	43185118	43197176	-0.423772929	PASS
CCNE1	19	29811898	29824308	0.044838622	PASS
CCNE2	8	94879770	94896678	0.355469066	suppress_segment_too_large
CCNF	16	2429394	2458854	0.053595371	PASS
CCNG1	5	163437569	163446151	0.165341871	suppress_segment_too_large
CCNG2	4	77157151	77433388	-0.677982248	PASS
CCNH	5	87318416	87413019	-0.393084436	PASS
CCNI	4	77047158	77076005	-0.677982248	PASS
CCNI2	5	132747445	132754403	-0.393084436	PASS
CCNJ	10	96043394	96060870	-0.414937136	PASS
CCNJL	5	160249106	160345396	0.165341871	suppress_segment_too_large
CCNK	14	99481169	99535044	-0.155273506	suppress_segment_too_large
CCNL1	3	157146508	157160760	0.02267528	PASS
CCNL2	1	1385711	1399328	-0.358281697	PASS
CCNO	5	55231152	55233680	-0.284712224	PASS
CCNQ	23	153587919	153600045	0.158844052	PASS
CCNT1	12	48688458	48716998	-0.743220499	PASS
CCNT2	2	134918235	134959342	-0.359440172	PASS
CCNY	10	35247025	35572669	0.075711544	PASS
CCNYL1	2	207711540	207761839	0.011001208	PASS
CCP110	16	19523811	19553408	0.053595371	PASS
CCPG1	15	55340032	55408510	-0.759380635	PASS
CCR1	3	46201709	46208396	-0.727808231	PASS
CCR10	17	42678889	42683917	0.041294512	PASS
CCR2	3	46353734	46360928	-0.727808231	PASS
CCR3	3	46163604	46266706	-0.727808231	PASS
CCR4	3	32951574	32956349	0.06149513	PASS
CCR5	3	46370854	46376206	-0.727808231	PASS
CCR6	6	167111807	167139696	0.033894706	PASS
CCR7	17	40553769	40565472	0.041294512	PASS
CCR8	3	39329706	39333511	-0.727808231	PASS
CCR9	3	45886504	45903177	-0.727808231	PASS
CCRL2	3	46407163	46412997	-0.727808231	PASS
CCS	11	66592821	66606019	0.064535884	PASS
CCSAP	1	229321005	229343294	-0.204996646	suppress_segment_too_large

CCSER1	4	90127535	91601913	-0.677982248	PASS
CCSER2	10	84328558	84518521	0.032351597	PASS
CCT2	12	69585334	69601570	-0.743220499	PASS
CCT3	1	156308968	156367873	0.088103245	PASS
CCT4	2	61868089	61888804	-0.46122572	PASS
CCT5	5	10249921	10266412	0.073527871	PASS
CCT6A	7	56051630	56063989	-0.701511252	PASS
CCT6B	17	34927859	34981078	0.041294512	PASS
CCT7	2	73233420	73253021	-0.46122572	PASS
CCT8	21	29055805	29073797	0.209983559	PASS
CCT8L2	22	16590751	16592810	-0.740245994	PASS
CCZ1	7	5898725	5926550	-0.72421718	PASS
CCZ1B	7	6794134	6826770	-0.72421718	PASS
CD101	1	117001750	117036476	0.079255572	PASS
CD109	6	73695785	73828316	-0.250122709	PASS
CD14	5	140631728	140633701	-0.393084436	PASS
CD151	11	832843	839831	-0.39835217	PASS
CD160	1	145719471	145739288	0.079255572	PASS
CD163	12	7470813	7503893	-0.015029783	PASS
CD163L1	12	7346685	7479897	-0.015029783	PASS
CD164	6	109366514	109382812	-0.355603029	PASS
CD164L2	1	27379176	27383380	0.048749694	PASS
CD177	19	43353659	43363172	0.048917043	PASS
CD180	5	67179613	67196799	-0.393084436	PASS
CD19	16	28931939	28939346	0.053595371	PASS
CD1A	1	158254137	158258269	0.088103245	PASS
CD1B	1	158327951	158331531	0.088103245	PASS
CD1C	1	158289786	158293630	0.088103245	PASS
CD1D	1	158179947	158184896	0.088103245	PASS
CD1E	1	158353696	158357553	0.088103245	PASS
CD2	1	116754385	116769228	0.079255572	PASS
CD200	3	112332347	112362812	0.06434429	PASS
CD200R1	3	112921209	112975122	0.06434429	PASS
CD200R1L	3	112815709	112846856	0.06434429	PASS
CD207	2	70830214	70835822	-0.46122572	PASS
CD209	19	7739994	7747564	-0.071973357	PASS
CD22	19	35319261	35347355	0.044838622	PASS
CD226	18	69831158	69961803	-0.330515409	PASS
CD24	6	106969831	106975627	-0.355603029	PASS
CD244	1	160830160	160862855	0.088103245	PASS
CD247	1	167430640	167518610	0.088103245	PASS
CD248	11	66314487	66317044	0.064535884	PASS
CD27	12	6444867	6451718	-0.015029783	PASS
CD274	9	5450503	5470566	0.073960965	PASS
CD276	15	73683966	73714518	-0.759380635	PASS
CD28	2	203706475	203738912	0.011001208	PASS
CD2AP	6	47477789	47627263	0.239805577	suppress_segment_too_large
CD2BP2	16	30350766	30355361	0.053595371	PASS
CD300A	17	74466399	74484796	0.051798665	PASS
CD300C	17	74541108	74546143	0.051798665	PASS
CD300E	17	74609887	74623738	0.051798665	PASS
CD300LB	17	74521174	74531474	0.051798665	PASS
CD300LD	17	74579365	74592283	0.051798665	PASS
CD300LF	17	74694311	74712978	0.051798665	PASS

CD300LG	17	43847148	43863629	0.051798665	PASS
CD302	2	159768630	159798255	0.054749241	PASS
CD320	19	8302127	8308356	-0.071973357	PASS
CD33	19	51225064	51243860	0.002743503	PASS
CD34	1	207880972	207911402	-0.204996646	suppress_segment_too_large
CD36	7	80369575	80679277	-0.701511252	PASS
CD37	19	49335171	49343335	0.002743503	PASS
CD38	4	15778275	15853230	-0.358623662	PASS
CD3D	11	118338954	118342744	-0.431650509	PASS
CD3E	11	118304545	118316175	-0.431650509	PASS
CD3EAP	19	45406209	45410766	0.002743503	PASS
CD3G	11	118344344	118355161	-0.431650509	PASS
CD4	12	6786858	6820808	-0.015029783	PASS
CD40	20	46118272	46129863	-0.172056771	suppress_segment_too_large
CD40LG	23	136648193	136660390	0.158844052	PASS
CD44	11	35138870	35232402	-0.359426047	PASS
CD46	1	207752057	207795513	-0.204996646	suppress_segment_too_large
CD47	3	108043094	108091862	0.22470242	PASS
CD48	1	160678746	160711851	0.088103245	PASS
CD5	11	61102395	61127852	0.064535884	PASS
CD52	1	26317957	26320523	0.048749694	PASS
CD53	1	110871188	110899928	0.079255572	PASS
CD55	1	207321376	207386804	-0.204996646	suppress_segment_too_large
CD58	1	116514535	116571039	0.079255572	PASS
CD59	11	33703010	33736491	-0.359426047	PASS
CD5L	1	157830914	157898256	0.088103245	PASS
CD6	11	60971680	61020377	0.064535884	PASS
CD63	12	55725323	55729707	-0.743220499	PASS
CD68	17	7579467	7582113	0.06854245	PASS
CD69	12	9752486	9760901	0.505438545	PASS
CD7	17	82314868	82317602	0.051798665	PASS
CD70	19	6583183	6604103	-0.071973357	PASS
CD72	9	35609533	35646810	0.073960965	PASS
CD74	5	150401637	150412929	-0.393084436	PASS
CD79A	19	41877120	41881372	0.048917043	PASS
CD79B	17	63928740	63932354	0.051798665	PASS
CD80	3	119524293	119559602	0.06434429	PASS
CD81	11	2376177	2397419	-0.39835217	PASS
CD82	11	44564427	44620363	0.059795455	PASS
CD83	6	14117256	14136918	0.069043418	PASS
CD84	1	160541095	160579516	0.088103245	PASS
CD86	3	122055366	122121139	0.06434429	PASS
CD8A	2	86784610	86808396	0.064122364	PASS
CD8B	2	86815339	86861924	0.064122364	PASS
CD8B2	2	106487364	106544297	-0.439572519	PASS
CD9	12	6199715	6238271	-0.015029783	PASS
CD93	20	23079349	23086340	-0.172056771	suppress_segment_too_large
CD96	3	111292719	111665750	0.22470242	PASS
CD99	23	2691133	2741309	-0.350651546	PASS
CD99L2	23	150766337	150898816	0.158844052	PASS
CDA	1	20588948	20618908	0.048749694	PASS
CDADC1	13	49247925	49293485	-0.384313056	PASS
CDAN1	15	42723544	42737128	-0.423772929	PASS
CDC123	10	12195965	12250589	0.075711544	PASS

CDC14A	1	100345001	100520278	0.079255572	PASS
CDC14B	9	96490241	96619830	0.073960965	PASS
CDC16	13	114234887	114272723	-0.426822582	PASS
CDC20	1	43358955	43363203	-0.736943052	PASS
CDC20B	5	55112995	55173175	-0.284712224	PASS
CDC23	5	138187648	138213343	-0.393084436	PASS
CDC25A	3	48157146	48188402	-0.727808231	PASS
CDC25B	20	3786772	3806121	-0.172056771	suppress_segment_too_large
CDC25C	5	138285265	138338355	-0.393084436	PASS
CDC26	9	113255835	113275589	0.073960965	PASS
CDC27	17	47117703	47189422	0.051798665	PASS
CDC34	19	531712	542092	-0.071973357	PASS
CDC37	19	10391134	10420121	-0.107594294	PASS
CDC37L1	9	4679559	4708398	0.073960965	PASS
CDC40	6	110180141	110254275	-0.355603029	PASS
CDC42	1	22052627	22092946	0.048749694	PASS
CDC42BPA	1	226989865	227318474	-0.204996646	suppress_segment_too_large
CDC42BPB	14	102932379	103057462	-0.155273506	suppress_segment_too_large
CDC42BPG	11	64823387	64844569	0.064535884	PASS
CDC42EP1	22	37560447	37569405	-0.740245994	PASS
CDC42EP2	11	65314818	65322429	0.064535884	PASS
CDC42EP3	2	37641882	37738468	0.034263343	PASS
CDC42EP4	17	73283624	73312175	0.051798665	PASS
CDC42EP5	19	54465026	54473264	0.142581352	PASS
CDC42SE1	1	151050971	151070325	0.079255572	PASS
CDC42SE2	5	131245493	131398447	-0.393084436	PASS
CDC45	22	19479459	19520612	-0.740245994	PASS
CDC5L	6	44387525	44450426	0.028532667	PASS
CDC6	17	40287633	40304657	0.041294512	PASS
CDC7	1	91500893	91525764	0.079255572	PASS
CDC73	1	193121800	193254815	0.088103245	PASS
CDCA2	8	25458997	25507920	-0.311940419	PASS
CDCA3	12	6844793	6852066	-0.015029783	PASS
CDCA4	14	105009573	105021148	-0.155273506	suppress_segment_too_large
CDCA5	11	65066300	65084164	0.064535884	PASS
CDCA7	2	173354820	173368997	0.033541467	PASS
CDCA7L	7	21900900	21946084	-0.72421718	PASS
CDCA8	1	37692418	37709719	0.048749694	PASS
CDCP1	3	45082278	45146422	-0.727808231	PASS
CDCP2	1	54132968	54153770	-0.736943052	PASS
CDH1	16	68737225	68835548	-0.423061015	PASS
CDH10	5	24487100	24644978	0.073527871	PASS
CDH11	16	64943753	65126112	-0.423061015	PASS
CDH12	5	21750673	22853622	0.073527871	PASS
CDH13	16	82626803	83800640	-0.423061015	PASS
CDH15	16	89171767	89195492	-0.423061015	PASS
CDH16	16	66908122	66918984	-0.423061015	PASS
CDH17	8	94127171	94217303	0.355469066	suppress_segment_too_large
CDH18	5	19472951	20575873	0.073527871	PASS
CDH19	18	66501083	66604138	-0.330515409	PASS
CDH2	18	27950966	28177446	-0.330515409	PASS
CDH20	18	61333582	61555773	-0.330515409	PASS
CDH22	20	46173733	46308498	-0.172056771	suppress_segment_too_large
CDH23	10	71396934	71815947	0.032351597	PASS

CDH24	14	23047062	23057538	0.053252507	PASS
CDH26	20	59958427	60034011	-0.172056771	suppress_segment_too_large
CDH3	16	68636189	68722616	-0.423061015	PASS
CDH4	20	61252426	61940617	-0.172056771	suppress_segment_too_large
CDH5	16	66366622	66404786	-0.423061015	PASS
CDH6	5	31193750	31329146	0.073527871	PASS
CDH7	18	65750252	65890341	-0.330515409	PASS
CDH8	16	61647242	62037035	-0.423061015	PASS
CDH9	5	26880600	27121150	0.073527871	PASS
CDHR1	10	84194635	84219621	0.032351597	PASS
CDHR2	5	176542511	176595974	0.165341871	suppress_segment_too_large
CDHR3	7	105876796	106033773	-0.712149877	PASS
CDHR4	3	49790732	49799835	-0.727808231	PASS
CDHR5	11	616565	626078	-0.39835217	PASS
CDIP1	16	4510675	4538828	0.053595371	PASS
CDIPT	16	29858357	29863736	0.053595371	PASS
CDK1	10	60778331	60794852	0.032351597	PASS
CDK10	16	89680737	89696364	-0.423061015	PASS
CDK11A	1	1702730	1724324	-0.358281697	PASS
CDK11B	1	1635227	1659012	-0.358281697	PASS
CDK12	17	39461511	39564907	0.041294512	PASS
CDK13	7	39949646	40099580	-0.72421718	PASS
CDK14	7	90466424	91210590	-0.710463272	PASS
CDK15	2	201790461	201895550	0.011001208	PASS
CDK16	23	47217860	47229997	0.158844052	PASS
CDK17	12	96278261	96400560	0.028722739	PASS
CDK18	1	205504595	205532793	-0.204996646	suppress_segment_too_large
CDK19	6	110609978	110815958	-0.355603029	PASS
CDK2	12	55966769	55972784	-0.743220499	PASS
CDK20	9	87966441	87974753	0.073960965	PASS
CDK2AP1	12	123260971	123272334	0.028722739	PASS
CDK2AP2	11	67506497	67508649	0.064535884	PASS
CDK3	17	76000906	76005999	0.051798665	PASS
CDK4	12	57747727	57756013	-0.743220499	PASS
CDK5	7	151053812	151058530	-0.712149877	PASS
CDK5R1	17	32486619	32491256	0.041294512	PASS
CDK5R2	2	218959655	218962162	0.011001208	PASS
CDK5RAP	20	33358839	33401561	-0.172056771	suppress_segment_too_large
CDK5RAP	9	120388869	120580170	0.073960965	PASS
CDK5RAP	17	47967810	47981774	0.051798665	PASS
CDK6	7	92604921	92836594	-0.710463272	PASS
CDK7	5	69234795	69277430	-0.393084436	PASS
CDK8	13	26254104	26405238	-0.398748891	PASS
CDK9	9	127785679	127790787	0.073960965	PASS
CDKAL1	6	20534457	21232404	0.069043418	PASS
CDKL1	14	50329404	50416461	0.053252507	PASS
CDKL2	4	75578005	75630716	-0.677982248	PASS
CDKL3	5	134286350	134371047	-0.393084436	PASS
CDKL4	2	39175646	39229588	0.034263343	PASS
CDKL5	23	18425583	18653629	0.158844052	PASS
CDKN1A	6	36676460	36687339	0.028532667	PASS
CDKN1B	12	12715058	12722371	0.49032213	suppress_segment_too_large
CDKN1C	11	2883213	2885773	-0.39835217	PASS
CDKN2A	9	21967753	21995301	0.073960965	PASS

CDKN2AI	4	183444591	183448198	-0.453876349	PASS
CDKN2AI	5	134402087	134411898	-0.393084436	PASS
CDKN2B	9	22002903	22009363	0.073960965	PASS
CDKN2C	1	50960745	50974633	-0.736943052	PASS
CDKN2D	19	10566462	10569059	-0.107594294	PASS
CDKN3	14	54396849	54420218	0.053252507	PASS
CDNF	10	14819250	14838575	0.075711544	PASS
CDO1	5	115804733	115816954	-0.393084436	PASS
CDON	11	125955796	126063335	-0.431650509	PASS
CDPF1	22	46244011	46250679	-0.953096644	PASS
CDR1	23	140783578	140784366	0.158844052	PASS
CDR2	16	22345936	22437165	0.053595371	PASS
CDR2L	17	74987632	75005800	0.051798665	PASS
CDRT1	17	15565483	15619512	0.06854245	PASS
CDRT15	17	14235673	14236862	0.06854245	PASS
CDRT15L2	17	20579724	20580911	0.06854245	PASS
CDRT4	17	15436015	15503608	0.06854245	PASS
CDS1	4	84582979	84651338	-0.677982248	PASS
CDS2	20	5126786	5197887	-0.172056771	suppress_segment_too_large
CDSN	6	31115090	31120446	0.069043418	PASS
CDT1	16	88803213	88809258	-0.423061015	PASS
CDV3	3	133573730	133590261	0.06434429	PASS
CDX1	5	150166795	150184558	-0.393084436	PASS
CDX2	13	27962137	27971139	-0.398748891	PASS
CDX4	23	73447254	73455245	0.158844052	PASS
CDYL	6	4706159	4955551	0.069043418	PASS
CDYL2	16	80597906	80804329	-0.423061015	PASS
CEACAM1	19	42507304	42561234	0.048917043	PASS
CEACAM1	19	44699151	44710714	0.002743503	PASS
CEACAM1	19	51478622	51490952	0.002743503	PASS
CEACAM1	19	44662278	44684359	0.002743503	PASS
CEACAM2	19	44501677	44529788	0.002743503	PASS
CEACAM2	19	41549518	41586844	0.048917043	PASS
CEACAM3	19	41796437	41811553	0.048917043	PASS
CEACAM4	19	41618971	41627074	0.048917043	PASS
CEACAM5	19	41708585	41729798	0.048917043	PASS
CEACAM6	19	41750977	41772208	0.048917043	PASS
CEACAM7	19	41673307	41706976	0.048917043	PASS
CEACAM8	19	42580241	42595055	0.048917043	PASS
CEBPA	19	33299934	33302564	0.044838622	PASS
CEBPB	20	50190734	50192689	-0.172056771	suppress_segment_too_large
CEBPD	8	47736909	47739086	0.182873287	suppress_segment_too_large
CEBPE	14	23117304	23119616	0.053252507	PASS
CEBPG	19	33373330	33382686	0.044838622	PASS
CEBPZ	2	37201612	37231713	0.034263343	PASS
CEBPZOS	2	37196488	37216193	0.034263343	PASS
CECR2	22	17359949	17558149	-0.740245994	PASS
CEL	9	133061978	133071861	0.073960965	PASS
CELA1	12	51328443	51346679	-0.743220499	PASS
CELA2A	1	15456728	15472091	0.048749694	PASS
CELA2B	1	15465909	15491400	0.048749694	PASS
CELA3A	1	22001656	22012539	0.048749694	PASS
CELA3B	1	21977021	21998642	0.048749694	PASS
CELF1	11	47465933	47565569	0.059795455	PASS

CELF2	10	10798397	11336675	0.075711544	PASS
CELF3	1	151702404	151716814	0.079255572	PASS
CELF4	18	37243047	37566037	-0.330515409	PASS
CELF5	19	3224703	3297076	-0.071973357	PASS
CELF6	15	72284727	72320129	-0.759380635	PASS
CELSR1	22	46360834	46537170	-0.953096644	PASS
CELSR2	1	109250019	109275750	0.079255572	PASS
CELSR3	3	48636469	48662915	-0.727808231	PASS
CEMIP	15	80779343	80951776	-0.759380635	PASS
CEMIP2	9	71683366	71816690	0.073960965	PASS
CEMP1	16	2530035	2531417	0.053595371	PASS
CEND1	11	787104	790123	-0.39835217	PASS
CENPA	2	26764289	26801067	0.034263343	PASS
CENPB	20	3783851	3786690	-0.172056771	suppress_segment_too_large
CENPBD1	16	89969773	89972832	-0.423061015	PASS
CENPC	4	67468748	67545606	0.115781418	suppress_segment_too_large
CENPE	4	103105806	103198409	-0.677982248	PASS
CENPF	1	214603195	214664588	-0.204996646	suppress_segment_too_large
CENPH	5	69189548	69210357	-0.393084436	PASS
CENPI	23	101098218	101163681	0.158844052	PASS
CENPJ	13	24882284	24922889	-0.398748891	PASS
CENPK	5	65517766	65563171	-0.393084436	PASS
CENPL	1	173799550	173824720	0.088103245	PASS
CENPM	22	41938721	41947164	-0.740245994	PASS
CENPN	16	81006498	81033114	-0.423061015	PASS
CENPO	2	24793136	24822376	0.034263343	PASS
CENPP	9	92325484	92620533	0.073960965	PASS
CENPQ	6	49463378	49493107	0.239805577	suppress_segment_too_large
CENPS	1	10430443	10442809	0.364796772	PASS
CENPS-CC	1	10430102	10452153	0.364796772	PASS
CENPT	16	67828157	67847811	-0.423061015	PASS
CENPU	4	184694618	184734133	-0.453876349	PASS
CENPV	17	16342534	16353656	0.06854245	PASS
CENPVL3	23	51617024	51618968	0.158844052	PASS
CENPW	6	126340174	126348875	-0.304168115	PASS
CENPX	17	82018702	82024107	0.051798665	PASS
CEP104	1	3812081	3857214	-0.358281697	PASS
CEP112	17	65635538	66192084	0.051798665	PASS
CEP120	5	123344885	123423592	-0.393084436	PASS
CEP126	11	101915015	102001058	0.32805343	suppress_segment_too_large
CEP128	14	80476983	80959517	-0.155273506	suppress_segment_too_large
CEP131	17	81189593	81222999	0.051798665	PASS
CEP135	4	55948871	56033363	0.115781418	suppress_segment_too_large
CEP152	15	48712928	48811146	-0.423772929	PASS
CEP162	6	84124241	84227635	-0.355603029	PASS
CEP164	11	117314557	117413268	-0.431650509	PASS
CEP170	1	243124428	243255348	-0.287484925	PASS
CEP170B	14	104865280	104896770	-0.155273506	suppress_segment_too_large
CEP19	3	196706277	196712293	0.02267528	PASS
CEP192	18	12991362	13125052	0.044197172	PASS
CEP250	20	35455164	35519280	-0.172056771	suppress_segment_too_large
CEP290	12	88049014	88142216	0.271127429	suppress_segment_too_large
CEP295	11	93661639	93730358	0.064535884	PASS
CEP295NL	17	78870910	78903217	0.051798665	PASS

CEP350	1	179954738	180114880	0.088103245	PASS
CEP41	7	130393771	130442433	-0.712149877	PASS
CEP44	4	174283677	174333380	0.142431244	suppress_segment_too_large
CEP55	10	93496632	93529092	-0.414937136	PASS
CEP57	11	95789965	95832693	0.32805343	suppress_segment_too_large
CEP57L1	6	109095110	109163932	-0.355603029	PASS
CEP63	3	134485721	134575017	0.06434429	PASS
CEP68	2	65056366	65087004	-0.46122572	PASS
CEP70	3	138494344	138594538	0.06434429	PASS
CEP72	5	612272	667168	0.073527871	PASS
CEP76	18	12661833	12702777	0.044197172	PASS
CEP78	9	78236062	78279690	0.073960965	PASS
CEP83	12	94306449	94459988	0.028722739	PASS
CEP85	1	26234200	26278808	0.048749694	PASS
CEP85L	6	118460772	118710075	-0.355603029	PASS
CEP89	19	32875925	32971991	0.044838622	PASS
CEP95	17	64506588	64542461	0.051798665	PASS
CEP97	3	101723925	101770562	0.22470242	PASS
CEPT1	1	111139627	111185102	0.079255572	PASS
CER1	9	14719724	14722717	0.073960965	PASS
CERCAM	9	128411751	128437351	0.073960965	PASS
CERK	22	46684411	46738261	-0.953096644	PASS
CERKL	2	181536676	181680665	0.162734012	PASS
CERS1	19	18868545	18896727	-0.069222905	PASS
CERS2	1	150960583	150975004	0.079255572	PASS
CERS3	15	100400395	100544995	-0.759380635	PASS
CERS4	19	8206736	8262421	-0.071973357	PASS
CERS5	12	50129306	50167533	-0.743220499	PASS
CERS6	2	168455862	168775137	0.033541467	PASS
CES1	16	55802851	55833337	-0.423061015	PASS
CES2	16	66934444	66945096	-0.423061015	PASS
CES3	16	66961237	66975148	-0.423061015	PASS
CES4A	16	66988589	67009758	-0.423061015	PASS
CES5A	16	55846154	55956031	-0.423061015	PASS
CETN1	18	580357	582114	0.044197172	PASS
CETN2	23	152826973	152830777	0.158844052	PASS
CETN3	5	90392261	90409786	-0.393084436	PASS
CETP	16	56961850	56983845	-0.423061015	PASS
CFAP100	3	126394939	126436556	0.06434429	PASS
CFAP126	1	161364731	161367874	0.088103245	PASS
CFAP157	9	127706989	127716002	0.073960965	PASS
CFAP161	15	81007033	81149175	-0.759380635	PASS
CFAP20	16	58113588	58129450	-0.423061015	PASS
CFAP206	6	87407983	87464465	-0.355603029	PASS
CFAP221	2	119544432	119662251	-0.359440172	PASS
CFAP298	21	32592079	32612866	0.209983559	PASS
CFAP299	4	80335720	80963756	-0.677982248	PASS
CFAP300	11	102047443	102084560	0.32805343	suppress_segment_too_large
CFAP36	2	55519604	55545879	-0.052193497	PASS
CFAP410	21	44328944	44339402	0.209983559	PASS
CFAP43	10	104129888	104232362	-0.414937136	PASS
CFAP44	3	113286947	113441610	0.06434429	PASS
CFAP45	1	159872364	159900163	0.088103245	PASS
CFAP46	10	132808392	132942823	0.073090211	PASS

CFAP47	23	35919734	36385319	0.158844052	PASS
CFAP52	17	9576627	9643459	0.06854245	PASS
CFAP53	18	50227193	50266522	-0.330515409	PASS
CFAP54	12	96489571	96875555	0.028722739	PASS
CFAP57	1	43172149	43254358	-0.736943052	PASS
CFAP58	10	104353764	104455090	-0.414937136	PASS
CFAP61	20	20052514	20360702	-0.172056771	suppress_segment_too_large
CFAP65	2	219002846	219041527	0.011001208	PASS
CFAP69	7	90245174	90311063	-0.710463272	PASS
CFAP70	10	73253759	73358859	0.032351597	PASS
CFAP73	12	113149858	113159276	0.028722739	PASS
CFAP74	1	1921951	2003837	-0.358281697	PASS
CFAP77	9	132410043	132573317	0.073960965	PASS
CFAP97	4	185159665	185209504	0.275137421	PASS
CFAP97D1	17	43780435	43787612	0.051798665	PASS
CFAP99	4	2418974	2462942	-0.358623662	PASS
CFB	6	31945650	31952084	0.028532667	PASS
CFC1	2	130592168	130599575	-0.359440172	PASS
CFD	19	859643	863630	-0.071973357	PASS
CFDP1	16	75293698	75433485	-0.423061015	PASS
CFH	1	196651878	196747504	0.088103245	PASS
CFHR1	1	196819757	196832189	0.088103245	PASS
CFHR2	1	196943756	196959226	0.088103245	PASS
CFHR3	1	196774795	196795406	0.088103245	PASS
CFHR4	1	196888014	196918713	0.088103245	PASS
CFHR5	1	196977556	197009674	0.088103245	PASS
CFI	4	109740694	109802179	-0.677982248	PASS
CFL1	11	65823022	65862026	0.064535884	PASS
CFL2	14	34706769	34714823	0.053252507	PASS
CFLAR	2	201116104	201176687	0.011001208	PASS
CFP	23	47623172	47630305	0.158844052	PASS
CFTR	7	117465784	117715971	-0.712149877	PASS
CGA	6	87085498	87095406	-0.355603029	PASS
CGAS	6	73413515	73452276	-0.250122709	PASS
CGB1	19	49035610	49036895	0.002743503	PASS
CGB2	19	49031912	49033238	0.002743503	PASS
CGB3	19	49022869	49024333	0.002743503	PASS
CGB5	19	49043884	49045311	0.002743503	PASS
CGB7	19	49054275	49058860	0.002743503	PASS
CGB8	19	49047638	49049106	0.002743503	PASS
CGGBP1	3	88051944	88149885	0.22470242	PASS
CGN	1	151510510	151538692	0.079255572	PASS
CGNL1	15	57375967	57550727	-0.759380635	PASS
CGREF1	2	27098889	27119115	0.034263343	PASS
CGRRF1	14	54509812	54539309	0.053252507	PASS
CH25H	10	89205629	89207314	-2.417109027	suppress_large_homdel
CHAC1	15	40952962	40956519	-0.423772929	PASS
CHAC2	2	53767792	53775196	0.034263343	PASS
CHAD	17	50464496	50468966	0.051798665	PASS
CHADL	22	41229510	41240934	-0.740245994	PASS
CHAF1A	19	4402662	4445018	-0.071973357	PASS
CHAF1B	21	36385378	36419015	0.209983559	PASS
CHAMP1	13	114314335	114337626	-0.426822582	PASS
CHAT	10	49609095	49665104	-0.389033546	PASS

CHCHD1	10	73782047	73783652	0.032351597	PASS
CHCHD10	22	23765834	23768443	-0.740245994	PASS
CHCHD2	7	56101569	56106576	-0.701511252	PASS
CHCHD3	7	132784868	133082088	-0.712149877	PASS
CHCHD4	3	14112077	14124870	0.06149513	PASS
CHCHD5	2	112584240	112589275	0.040971718	PASS
CHCHD6	3	126704220	126960406	0.06434429	PASS
CHCHD7	8	56211686	56218798	0.182873287	suppress_segment_too_large
CHD1	5	98853985	98928957	-0.393084436	PASS
CHD1L	1	147242641	147295766	0.079255572	PASS
CHD2	15	92900189	93028005	-0.759380635	PASS
CHD3	17	7884806	7912760	0.06854245	PASS
CHD4	12	6570082	6614524	-0.015029783	PASS
CHD5	1	6101793	6180123	0.364796772	PASS
CHD6	20	41402101	41618494	-0.172056771	suppress_segment_too_large
CHD7	8	60678778	60868028	0.182873287	suppress_segment_too_large
CHD8	14	21385194	21456126	0.053252507	PASS
CHD9	16	53055033	53329150	-0.423061015	PASS
CHDH	3	53812335	53846390	-0.727808231	PASS
CHEK1	11	125625136	125676255	-0.431650509	PASS
CHEK2	22	28687743	28742422	-0.740245994	PASS
CHERP	19	16517889	16542530	-0.069222905	PASS
CHFR	12	132822187	132956304	0.232349073	PASS
CHGA	14	92923080	92935293	-0.155273506	suppress_segment_too_large
CHGB	20	5911430	5925361	-0.172056771	suppress_segment_too_large
CHI3L1	1	203178931	203186749	-0.204996646	suppress_segment_too_large
CHI3L2	1	111200771	111243440	0.079255572	PASS
CHIA	1	111290862	111320566	0.079255572	PASS
CHIC1	23	73563200	73687102	0.158844052	PASS
CHIC2	4	54009789	54064690	0.115781418	suppress_segment_too_large
CHID1	11	867860	915058	-0.39835217	PASS
CHIT1	1	203212827	203273641	-0.204996646	suppress_segment_too_large
CHKA	11	68052859	68121444	0.064535884	PASS
CHKB	22	50578949	50601455	-0.953096644	PASS
CHKB-CP′	22	50568869	50582965	-0.953096644	PASS
CHL1	3	196596	409417	0.06149513	PASS
CHM	23	85861180	86047562	0.158844052	PASS
CHML	1	241628853	241640254	-0.287484925	PASS
CHMP1A	16	89644431	89657845	-0.423061015	PASS
CHMP1B	18	11851396	11854449	0.044197172	PASS
CHMP2A	19	58551566	58555124	0.142581352	PASS
CHMP2B	3	87227271	87255548	0.22470242	PASS
CHMP3	2	86503431	86563479	0.064122364	PASS
CHMP4A	14	24209583	24213869	0.053252507	PASS
CHMP4B	20	33811304	33854366	-0.172056771	suppress_segment_too_large
CHMP4C	8	81732434	81759515	0.617295349	suppress_segment_too_large
CHMP5	9	33264879	33282069	0.073960965	PASS
CHMP6	17	80991598	81009517	0.051798665	PASS
CHMP7	8	23243637	23262000	-0.311940419	PASS
CHN1	2	174799363	175005369	0.033541467	PASS
CHN2	7	29146569	29514328	-0.72421718	PASS
CHODL	21	17901263	18267373	0.758930684	suppress_segment_too_large
CHORDC1	11	90201160	90223364	0.064535884	PASS
CHP1	15	41230839	41281890	-0.423772929	PASS

CHP2	16	23754627	23758951		0.053595371	PASS
CHPF	2	219538947	219543787		0.011001208	PASS
CHPF2	7	151232489	151238827		-0.712149877	PASS
CHPT1	12	101696947	101744140		0.028722739	PASS
CHRAC1	8	140511298	140517137		0.493344802	suppress_segment_too_large
CHRD	3	184380073	184390736		0.02267528	PASS
CHRDL1	23	110673856	110795819		0.158844052	PASS
CHRDL2	11	74696429	74731385		0.064535884	PASS
CHRFAM7	15	30357766	30393849		-0.423772929	PASS
CHRM1	11	62908679	62921807		0.064535884	PASS
CHRM2	7	136868669	137020255		-0.712149877	PASS
CHRM3	1	239386565	239915452		-0.287484925	PASS
CHRM4	11	46385098	46386608		0.059795455	PASS
CHRM5	15	33968720	34067457		-0.423772929	PASS
CHRNA1	2	174747592	174787935		0.033541467	PASS
CHRNA10	11	3665587	3671384		-0.39835217	PASS
CHRNA2	8	27459761	27479883		-0.311940419	PASS
CHRNA3	15	78593052	78621295		-0.759380635	PASS
CHRNA4	20	63343223	63378401		-0.172056771	suppress_segment_too_large
CHRNA5	15	78565520	78595269		-0.759380635	PASS
CHRNA6	8	42752620	42796392		0.182873287	suppress_segment_too_large
CHRNA7	15	31923438	32173018		-0.423772929	PASS
CHRNA9	4	40335329	40355217		-0.753159364	PASS
CHRNB1	17	7445061	7457707		0.06854245	PASS
CHRNB2	1	154567781	154580026		0.088103245	PASS
CHRNB3	8	42697376	42737407		0.182873287	suppress_segment_too_large
CHRNB4	15	78624119	78727754		-0.759380635	PASS
CHRND	2	232525993	232536667		0.011001208	PASS
CHRNE	17	4897774	4934438		0.06854245	PASS
CHRNG	2	232539727	232546403		0.011001208	PASS
CHST1	11	45648877	45665622		0.059795455	PASS
CHST10	2	100391860	100417656		0.064122364	PASS
CHST11	12	104455295	104762014		0.028722739	PASS
CHST12	7	2403560	2448483		-0.72421718	PASS
CHST13	3	126524283	126543291		0.06434429	PASS
CHST14	15	40470998	40474571		-0.423772929	PASS
CHST15	10	124007666	124093607		0.073090211	PASS
CHST2	3	143119331	143124014		0.02267528	PASS
CHST3	10	71964365	72013564		0.032351597	PASS
CHST4	16	71525233	71538746		-0.423061015	PASS
CHST5	16	75528535	75535247		-0.423061015	PASS
CHST6	16	75472052	75495445		-0.423061015	PASS
CHST7	23	46573784	46598408		0.158844052	PASS
CHST8	19	33621955	33773509		0.044838622	PASS
CHST9	18	26906481	27185317		-0.330515409	PASS
CHSY1	15	101175723	101251932		-0.759380635	PASS
CHSY3	5	129904472	130186634		-0.393084436	PASS
CHTF18	16	788046	800737		0.053595371	PASS
CHTF8	16	69118010	69132584		-0.423061015	PASS
CHTOP	1	153633982	153646306		0.088103245	PASS
CHUK	10	100188298	100229619		-0.414937136	PASS
CHURC1	14	64914361	64944591		0.053252507	PASS
CHURC1-l	14	64914485	65061803		0.053252507	PASS
CIAO1	2	96266132	96273349		0.064122364	PASS

CIAO2A	15	64072559	64094018	-0.759380635	PASS
CIAO2B	16	66932055	66934423	-0.423061015	PASS
CIAO3	16	729753	741329	0.053595371	PASS
CIAPIN1	16	57428169	57447528	-0.423061015	PASS
CIART	1	150282543	150287093	0.079255572	PASS
CIB1	15	90229975	90234047	-0.759380635	PASS
CIB2	15	78104606	78131544	-0.759380635	PASS
CIB3	19	16161368	16173525	-0.069222905	PASS
CIB4	2	26581202	26641368	0.034263343	PASS
CIC	19	42268537	42295797	0.048917043	PASS
CIDEA	18	12254319	12277595	0.044197172	PASS
CIDEB	14	24305096	24311430	0.053252507	PASS
CIDEC	3	9866711	9880254	0.06149513	PASS
CIITA	16	10866222	10943021	0.053595371	PASS
CILP	15	65194758	65211488	-0.759380635	PASS
CILP2	19	19538248	19546659	-0.069222905	PASS
CINP	14	102341102	102362916	-0.155273506	suppress_segment_too_large
CIP2A	3	108549869	108589644	0.22470242	PASS
CIPC	14	77098097	77117287	-0.155273506	suppress_segment_too_large
CIR1	2	174348022	174395715	0.033541467	PASS
CIRBP	19	1259384	1274880	-0.071973357	PASS
CISD1	10	58269058	58289586	0.032351597	PASS
CISD2	4	102868974	102892807	-0.677982248	PASS
CISD3	17	38730235	38735044	0.041294512	PASS
CISH	3	50606490	50611831	-0.727808231	PASS
CIT	12	119685790	119877291	0.028722739	PASS
CITED1	23	72301638	72307187	0.158844052	PASS
CITED2	6	139371807	139374620	0.033894706	PASS
CITED4	1	40861051	40862366	0.048749694	PASS
CIZ1	9	128166064	128204383	0.073960965	PASS
CKAP2	13	52455429	52476628	-0.384313056	PASS
CKAP2L	2	112736607	112764677	0.040971718	PASS
CKAP4	12	106237877	106304279	0.028722739	PASS
CKAP5	11	46743048	46846308	0.059795455	PASS
CKB	14	103519659	103523111	-0.155273506	suppress_segment_too_large
CKLF	16	66552563	66566251	-0.423061015	PASS
CKLF-CM	16	66552587	66579135	-0.423061015	PASS
CKM	19	45306414	45322977	0.002743503	PASS
CKMT1A	15	43692886	43699222	-0.423772929	PASS
CKMT1B	15	43593054	43604901	-0.423772929	PASS
CKMT2	5	81233285	81266397	-0.393084436	PASS
CKS1B	1	154974653	154979249	0.088103245	PASS
CKS2	9	89311198	89316703	0.073960965	PASS
CLASP1	2	121337776	121649587	-0.359440172	PASS
CLASP2	3	33496245	33718356	0.06149513	PASS
CLASRP	19	45039040	45070956	0.002743503	PASS
CLBA1	14	104985775	105010482	-0.155273506	suppress_segment_too_large
CLC	19	39731250	39738028	0.048917043	PASS
CLCA1	1	86468368	86500289	0.079255572	PASS
CLCA2	1	86424086	86456558	0.079255572	PASS
CLCA4	1	86547078	86580754	0.079255572	PASS
CLCC1	1	108929508	108963457	0.079255572	PASS
CLCF1	11	67364168	67374177	0.064535884	PASS
CLCN1	7	143316126	143352083	-0.712149877	PASS

CLCN2	3	184346185	184361651	0.02267528	PASS
CLCN3	4	169612633	169723673	0.142431244	suppress_segment_too_large
CLCN4	23	10156945	10237660	0.158844052	PASS
CLCN5	23	49922615	50099235	0.158844052	PASS
CLCN6	1	11806096	11848079	0.364796772	PASS
CLCN7	16	1444934	1475580	0.053595371	PASS
CLCNKA	1	16018875	16034050	0.048749694	PASS
CLCNKB	1	16043736	16057308	0.048749694	PASS
CLDN1	3	190305701	190322475	0.02267528	PASS
CLDN10	13	95433604	95579759	-0.384313056	PASS
CLDN11	3	170418865	170454733	0.02267528	PASS
CLDN12	7	90383721	90513402	-0.710463272	PASS
CLDN14	21	36460621	36576569	0.209983559	PASS
CLDN15	7	101232092	101238820	-0.712149877	PASS
CLDN16	3	190322541	190412143	0.02267528	PASS
CLDN17	21	30165564	30166756	0.209983559	PASS
CLDN18	3	137998735	138033655	0.06434429	PASS
CLDN19	1	42733093	42740254	-0.736943052	PASS
CLDN2	23	106900164	106930861	0.158844052	PASS
CLDN20	6	155264013	155276548	0.033894706	PASS
CLDN22	4	183318194	183320774	-0.453876349	PASS
CLDN23	8	8701938	8704106	-0.311940419	PASS
CLDN24	4	183321764	183322426	-0.453876349	PASS
CLDN25	11	113779747	113780500	-0.431650509	PASS
CLDN3	7	73768997	73770270	-0.701511252	PASS
CLDN34	23	9967358	9968352	0.158844052	PASS
CLDN4	7	73799542	73832693	-0.701511252	PASS
CLDN5	22	19523024	19527545	-0.740245994	PASS
CLDN6	16	3014712	3020071	0.053595371	PASS
CLDN7	17	7259903	7263983	0.06854245	PASS
CLDN8	21	30214006	30216073	0.209983559	PASS
CLDN9	16	3012456	3014505	0.053595371	PASS
CLDND1	3	98497912	98523066	0.22470242	PASS
CLDND2	19	51367098	51369003	0.002743503	PASS
CLEC10A	17	7074537	7080307	0.06854245	PASS
CLEC11A	19	50723329	50725718	0.002743503	PASS
CLEC12A	12	9951316	9995694	0.505438545	PASS
CLEC12B	12	10010627	10018619	0.505438545	PASS
CLEC14A	14	38254103	38256369	0.053252507	PASS
CLEC16A	16	10944488	11182189	0.053595371	PASS
CLEC17A	19	14583084	14611157	-0.069222905	PASS
CLEC18A	16	69950705	69964452	-0.423061015	PASS
CLEC18B	16	74408270	74421953	-0.423061015	PASS
CLEC18C	16	70173322	70187361	-0.423061015	PASS
CLEC19A	16	19285739	19322145	0.053595371	PASS
CLEC1A	12	10069554	10111627	0.505438545	PASS
CLEC1B	12	9985642	10013424	0.505438545	PASS
CLEC2A	12	9898673	9932381	0.505438545	PASS
CLEC2B	12	9852984	9870136	0.505438545	PASS
CLEC2D	12	9664969	9699555	0.505438545	PASS
CLEC2L	7	139523856	139544984	-0.712149877	PASS
CLEC3A	16	78022515	78066761	-0.423061015	PASS
CLEC3B	3	45001548	45036071	-0.727808231	PASS
CLEC4A	12	8123632	8138607	-0.015029783	PASS

CLEC4C	12	7729415	7751605	-0.015029783	PASS
CLEC4D	12	8509475	8522366	0.505438545	PASS
CLEC4E	12	8533305	8540963	0.505438545	PASS
CLEC4F	2	70808643	70820600	-0.46122572	PASS
CLEC4G	19	7728957	7733906	-0.071973357	PASS
CLEC4M	19	7763149	7769605	-0.071973357	PASS
CLEC5A	7	141927357	141947007	-0.712149877	PASS
CLEC6A	12	8455926	8478330	0.505438545	PASS
CLEC7A	12	10116777	10130258	0.505438545	PASS
CLEC9A	12	10030677	10066027	0.505438545	PASS
CLECL1	12	9715860	9733299	0.505438545	PASS
CLGN	4	140388455	140427968	0.142431244	suppress_segment_too_large
CLHC1	2	55174791	55232563	-0.052193497	PASS
CLIC1	6	31730581	31739763	0.028532667	PASS
CLIC2	23	155276211	155334657	0.158844052	PASS
CLIC3	9	136994635	136996803	0.073960965	PASS
CLIC4	1	24745357	24844324	0.048749694	PASS
CLIC5	6	45880827	46080348	0.028532667	PASS
CLIC6	21	34669389	34718227	0.209983559	PASS
CLINT1	5	157785743	157859175	0.165341871	suppress_segment_too_large
CLIP1	12	122271432	122422632	0.028722739	PASS
CLIP2	7	74289475	74405943	-0.701511252	PASS
CLIP3	19	36014660	36033343	0.044838622	PASS
CLIP4	2	29097705	29189643	0.034263343	PASS
CLK1	2	200853009	200864744	0.011001208	PASS
CLK2	1	155262868	155278491	0.088103245	PASS
CLK3	15	74598500	74645414	-0.759380635	PASS
CLK4	5	178602664	178630615	0.165341871	suppress_segment_too_large
CLLU1OS	12	92420094	92428148	0.271127429	suppress_segment_too_large
CLMN	14	95181940	95319906	-0.155273506	suppress_segment_too_large
CLMP	11	123069865	123195281	-0.431650509	PASS
CLN3	16	28474111	28495575	0.053595371	PASS
CLN5	13	76990660	77019143	-0.384313056	PASS
CLN6	15	68206992	68257211	-0.759380635	PASS
CLN8	8	1755778	1801711	-0.311940419	PASS
CLNK	4	10486395	10684865	-0.358623662	PASS
CLNS1A	11	77514936	77637805	0.064535884	PASS
CLOCK	4	55427903	55547138	0.115781418	suppress_segment_too_large
CLP1	11	57648992	57661868	0.27006553	suppress_segment_too_large
CLPB	11	72285495	72434680	0.064535884	PASS
CLPP	19	6361452	6368910	-0.071973357	PASS
CLPS	6	35794982	35797344	0.028532667	PASS
CLPSL1	6	35781017	35793675	0.028532667	PASS
CLPSL2	6	35776594	35779552	0.028532667	PASS
CLPTM1	19	44954585	44993341	0.002743503	PASS
CLPTM1L	5	1317744	1345099	0.073527871	PASS
CLPX	15	65148219	65185342	-0.759380635	PASS
CLRN1	3	150926163	150972999	0.02267528	PASS
CLRN2	4	17515165	17527104	-0.358623662	PASS
CLRN3	10	127877841	127892947	0.073090211	PASS
CLSPN	1	35720218	35769967	0.048749694	PASS
CLSTN1	1	9729026	9824526	0.364796772	PASS
CLSTN2	3	139935185	140577397	-0.889410434	PASS
CLSTN3	12	7129698	7158945	-0.015029783	PASS

CLTA	9	36190856	36304781	0.073960965	PASS
CLTB	5	176392455	176416569	0.165341871	suppress_segment_too_large
CLTC	17	59619689	59696956	0.051798665	PASS
CLTCL1	22	19179473	19291716	-0.740245994	PASS
CLTRN	23	15627318	15675012	0.158844052	PASS
CLU	8	27596917	27615031	-0.311940419	PASS
CLUAP1	16	3500924	3539048	0.053595371	PASS
CLUH	17	2689386	2712663	0.06854245	PASS
CLUL1	18	596988	650334	0.044197172	PASS
CLVS1	8	61057158	61501645	0.182873287	suppress_segment_too_large
CLVS2	6	122995971	123072927	-1.845658544	PASS
CLYBL	13	99606669	99897134	-0.384313056	PASS
CMA1	14	24505353	24508265	0.053252507	PASS
CMAS	12	22046174	22065674	0.49032213	suppress_segment_too_large
CMBL	5	10275875	10308026	0.073527871	PASS
CMC1	3	28241584	28325142	0.06149513	PASS
CMC2	16	80966448	81020270	-0.423061015	PASS
CMC4	23	155061622	155071362	0.158844052	PASS
CMIP	16	81445170	81711762	-0.423061015	PASS
CMKLR1	12	108288044	108339341	0.028722739	PASS
CMPK1	1	47333797	47378839	-0.736943052	PASS
CMPK2	2	6840570	6866635	0.034263343	PASS
CMSS1	3	99817834	100181732	0.22470242	PASS
CMTM1	16	66566393	66579137	-0.423061015	PASS
CMTM2	16	66579448	66588275	-0.423061015	PASS
CMTM3	16	66603874	66613892	-0.423061015	PASS
CMTM4	16	66614750	66696707	-0.423061015	PASS
CMTM5	14	23376773	23379772	0.053252507	PASS
CMTM6	3	32481312	32503408	0.06149513	PASS
CMTM7	3	32391671	32483067	0.06149513	PASS
CMTM8	3	32238679	32370325	0.06149513	PASS
CMTR1	6	37433219	37482827	0.028532667	PASS
CMTR2	16	71281389	71289715	-0.423061015	PASS
CMYA5	5	79689877	79800240	-0.393084436	PASS
CNBD1	8	86866442	87615219	0.617295349	suppress_segment_too_large
CNBD2	20	35954564	36030700	-0.172056771	suppress_segment_too_large
CNBP	3	129169484	129183922	0.06434429	PASS
CNDP1	18	74534440	74587212	-0.330515409	PASS
CNDP2	18	74495816	74523454	-0.330515409	PASS
CNEP1R1	16	50024410	50037088	0.411512417	PASS
CNFN	19	42387019	42390287	0.048917043	PASS
CNGA1	4	47935977	48016672	0.115781418	suppress_segment_too_large
CNGA2	23	151738451	151745304	0.158844052	PASS
CNGA3	2	98346155	98398601	0.064122364	PASS
CNGA4	11	6234765	6244429	-0.39835217	PASS
CNGB1	16	57882340	57971116	-0.423061015	PASS
CNGB3	8	86553977	86743675	0.617295349	suppress_segment_too_large
CNIH1	14	54423560	54441431	0.053252507	PASS
CNIH2	11	66278190	66285301	0.064535884	PASS
CNIH3	1	224434660	224740549	-0.204996646	suppress_segment_too_large
CNIH4	1	224356850	224379459	-0.204996646	suppress_segment_too_large
CNKSR1	1	26177403	26189886	0.048749694	PASS
CNKSR2	23	21372801	21654695	0.158844052	PASS
CNKSR3	6	154387504	154510659	0.033894706	PASS

CNMD	13	52703264	52739812	-0.384313056	PASS
CNN1	19	11538717	11550323	-0.107594294	PASS
CNN2	19	1026581	1039068	-0.071973357	PASS
CNN3	1	94896949	94927278	0.079255572	PASS
CNNM1	10	99329099	99394330	-0.414937136	PASS
CNNM2	10	102918293	103090221	-0.414937136	PASS
CNNM3	2	96816245	96833911	0.064122364	PASS
CNNM4	2	96760902	96811891	0.064122364	PASS
CNOT1	16	58519951	58629886	-0.423061015	PASS
CNOT10	3	32685145	32773875	0.06149513	PASS
CNOT11	2	101252802	101270316	0.064122364	PASS
CNOT2	12	70242994	70354993	-0.578548701	PASS
CNOT3	19	54137717	54155708	0.142581352	PASS
CNOT4	7	135361795	135510127	-0.712149877	PASS
CNOT6	5	180494412	180578405	0.165341871	suppress_segment_too_large
CNOT6L	4	77713387	77819615	-0.677982248	PASS
CNOT7	8	17224964	17246878	-0.311940419	PASS
CNOT8	5	154857553	154876793	-0.393084436	PASS
CNOT9	2	218568580	218597080	0.011001208	PASS
CNP	17	41966741	41977731	0.041294512	PASS
CNPPD1	2	219171897	219178106	0.011001208	PASS
CNPY1	7	155474206	155555450	-0.77630473	PASS
CNPY2	12	56309842	56316336	-0.743220499	PASS
CNPY3	6	42929192	42939287	0.028532667	PASS
CNPY4	7	100119613	100125511	-0.710463272	PASS
CNR1	6	88139864	88166359	-0.355603029	PASS
CNR2	1	23870526	23913362	0.048749694	PASS
CNRIP1	2	68284171	68320051	-0.46122572	PASS
CNST	1	246566444	246668584	-0.145670469	suppress_likely_unfocal_large_gain
CNTD1	17	42798792	42811587	0.041294512	PASS
CNTD2	19	40222208	40226697	0.048917043	PASS
CNTF	11	58622673	58625733	0.27006553	suppress_segment_too_large
CNTFR	9	34551432	34590140	0.073960965	PASS
CNTLN	9	17134982	17503923	0.073960965	PASS
CNTN1	12	40692442	41072418	-0.46813528	PASS
CNTN2	1	205042937	205078284	-0.204996646	suppress_segment_too_large
CNTN3	3	74262568	74521140	-0.727808231	PASS
CNTN4	3	2098813	3057956	0.06149513	PASS
CNTN5	11	99020953	100358885	0.32805343	suppress_segment_too_large
CNTN6	3	1092576	1404217	0.06149513	PASS
CNTNAP1	17	42682613	42699814	0.041294512	PASS
CNTNAP2	7	146116002	148420998	-0.712149877	PASS
CNTNAP3	9	39072767	39288315	0.073960965	PASS
CNTNAP3	9	41890314	42129510	0.073960965	PASS
CNTNAP3	9	61330717	61453030	0.073960965	PASS
CNTNAP4	16	76277278	76559238	-0.423061015	PASS
CNTNAP5	2	124025287	124915287	-0.359440172	PASS
CNTRL	9	121074863	121177610	0.073960965	PASS
CNTROB	17	7932101	7949918	0.06854245	PASS
COA1	7	43608456	43729717	-0.72421718	PASS
COA3	17	42795147	42798704	0.041294512	PASS
COA4	11	73872667	73876988	0.064535884	PASS
COA5	2	98599310	98608515	0.064122364	PASS
COA6	1	234373456	234384049	-0.204996646	suppress_segment_too_large

COA7	1	52684451	52698366	-0.736943052	PASS
COASY	17	42561467	42566277	0.041294512	PASS
COBL	7	51016212	51316818	-0.701511252	PASS
COBLL1	2	164653624	164843679	0.319987345	PASS
COCH	14	30874514	30895065	0.053252507	PASS
COG1	17	73192632	73208507	0.051798665	PASS
COG2	1	230642489	230693982	-0.204996646	suppress_segment_too_large
COG3	13	45464898	45536630	-0.398748891	PASS
COG4	16	70480568	70523565	-0.423061015	PASS
COG5	7	107201555	107564514	-0.712149877	PASS
COG6	13	39655627	39791665	-0.398748891	PASS
COG7	16	23388493	23453180	0.053595371	PASS
COG8	16	69320140	69339667	-0.423061015	PASS
COIL	17	56938187	56961054	0.051798665	PASS
COL10A1	6	116118923	116158747	-0.355603029	PASS
COL11A1	1	102876467	103108872	0.079255572	PASS
COL11A2	6	33162681	33192499	0.028532667	PASS
COL12A1	6	75084326	75206051	-0.250122709	PASS
COL13A1	10	69801867	69964275	0.032351597	PASS
COL14A1	8	120059780	120372036	0.493344802	suppress_segment_too_large
COL15A1	9	98943179	99070792	0.073960965	PASS
COL16A1	1	31652247	31704319	0.048749694	PASS
COL17A1	10	104031286	104085957	-0.414937136	PASS
COL18A1	21	45405137	45513720	0.209983559	PASS
COL19A1	6	69866571	70209976	-0.250122709	PASS
COL1A1	17	50183289	50201632	0.051798665	PASS
COL1A2	7	94394561	94431232	-0.710463272	PASS
COL20A1	20	63293186	63334851	-0.172056771	suppress_segment_too_large
COL21A1	6	56056590	56394094	0.239805577	suppress_segment_too_large
COL22A1	8	138588235	138914006	0.493344802	suppress_segment_too_large
COL23A1	5	178237618	178590555	0.165341871	suppress_segment_too_large
COL24A1	1	85729233	86156943	0.587598296	PASS
COL25A1	4	108808725	109302752	-0.677982248	PASS
COL26A1	7	101362820	101559024	-0.712149877	PASS
COL27A1	9	114155560	114312511	0.073960965	PASS
COL28A1	7	7356203	7535853	-0.72421718	PASS
COL2A1	12	47972965	48004554	-0.743220499	PASS
COL3A1	2	188974320	189012746	-0.030005025	PASS
COL4A1	13	110148963	110307233	-0.426822582	PASS
COL4A2	13	110305812	110513209	-0.426822582	PASS
COL4A3	2	227164565	227314792	0.011001208	PASS
COL4A3B	5	75356345	75512138	-0.393084436	PASS
COL4A4	2	227002711	227164453	0.011001208	PASS
COL4A5	23	108439844	108697545	0.158844052	PASS
COL4A6	23	108155607	108439497	0.158844052	PASS
COL5A1	9	134641774	134844843	0.073960965	PASS
COL5A2	2	189031896	189225312	-0.030005025	PASS
COL5A3	19	9959561	10010471	-0.107594294	PASS
COL6A1	21	45981737	46005050	0.209983559	PASS
COL6A2	21	46098097	46132849	0.209983559	PASS
COL6A3	2	237324003	237414375	0.011001208	PASS
COL6A5	3	130345516	130484844	0.06434429	PASS
COL6A6	3	130560334	130678155	0.06434429	PASS
COL7A1	3	48564073	48595267	-0.727808231	PASS

COL8A1	3	99638475	99799226	0.22470242	PASS
COL8A2	1	36095236	36125220	0.048749694	PASS
COL9A1	6	70215061	70303083	-0.250122709	PASS
COL9A2	1	40300487	40317813	0.048749694	PASS
COL9A3	20	62816244	62841159	-0.172056771	suppress_segment_too_large
COLCA2	11	111298555	111308735	-0.431650509	PASS
COLEC10	8	118995452	119106582	0.493344802	suppress_segment_too_large
COLEC11	2	3594832	3644644	0.034263343	PASS
COLEC12	18	316740	500722	0.044197172	PASS
COLGALT	19	17555594	17583162	-0.069222905	PASS
COLGALT	1	183929854	184037729	0.088103245	PASS
COLQ	3	15450133	15521751	0.06149513	PASS
COMMD1	2	61888724	62147247	-0.46122572	PASS
COMMD1	5	116084991	116412762	-0.393084436	PASS
COMMD2	3	149738470	149752499	0.02267528	PASS
COMMD3	10	22315974	22320308	0.075711544	PASS
COMMD3	10	22316388	22329542	0.075711544	PASS
COMMD4	15	75335891	75343224	-0.759380635	PASS
COMMD5	8	144841042	144853736	0.699132336	PASS
COMMD6	13	75525219	75549439	-0.384313056	PASS
COMMD7	20	32702691	32743997	-0.172056771	suppress_segment_too_large
COMMD8	4	47450796	47463719	0.115781418	suppress_segment_too_large
COMMD9	11	36269284	36289449	-0.359426047	PASS
COMP	19	18782773	18791314	-0.069222905	PASS
COMT	22	19941607	19969975	-0.740245994	PASS
COMTD1	10	75233969	75236030	0.032351597	PASS
COP1	1	175944831	176207493	0.088103245	PASS
COPA	1	160288686	160343400	0.088103245	PASS
COPB1	11	14443440	14500027	-0.39835217	PASS
COPB2	3	139355600	139389732	0.06434429	PASS
COPE	19	18899514	18919397	-0.069222905	PASS
COPG1	3	129249606	129277773	0.06434429	PASS
COPG2	7	130506238	130668748	-0.712149877	PASS
COPRS	17	31851864	31859337	0.041294512	PASS
COPS2	15	49106068	49155661	-0.423772929	PASS
COPS3	17	17246820	17281293	0.06854245	PASS
COPS4	4	83034447	83075818	-0.677982248	PASS
COPS5	8	67043079	67083783	0.182873287	suppress_segment_too_large
COPS6	7	100088954	100092200	-0.710463272	PASS
COPS7A	12	6723741	6731875	-0.015029783	PASS
COPS7B	2	231781671	231809254	0.011001208	PASS
COPS8	2	237085312	237100466	0.011001208	PASS
COPS9	2	240126563	240136807	0.011001208	PASS
COPZ1	12	54301202	54351849	-0.743220499	PASS
COPZ2	17	48026167	48038030	0.051798665	PASS
COQ10A	12	56266858	56270966	-0.743220499	PASS
COQ10B	2	197453423	197475308	0.011001208	PASS
COQ2	4	83261536	83284914	-0.677982248	PASS
COQ3	6	99369400	99394204	-0.355603029	PASS
COQ4	9	128322536	128334072	0.073960965	PASS
COQ5	12	120503274	120534434	0.028722739	PASS
COQ6	14	73949926	73963670	0.053252507	PASS
COQ7	16	19067599	19080095	0.053595371	PASS
COQ8A	1	226897536	226987545	-0.204996646	suppress_segment_too_large

COQ8B	19	40691529	40718207	0.048917043	PASS
COQ9	16	57447425	57461275	-0.423061015	PASS
CORIN	4	47593998	47838106	0.115781418	suppress_segment_too_large
CORO1A	16	30182827	30189076	0.053595371	PASS
CORO1B	11	67435510	67443821	0.064535884	PASS
CORO1C	12	108645109	108731596	0.028722739	PASS
CORO2A	9	98120975	98192640	0.073960965	PASS
CORO2B	15	68578969	68727806	-0.759380635	PASS
CORO6	17	29614756	29622907	0.041294512	PASS
CORO7	16	4354542	4425705	0.053595371	PASS
CORO7-PA	16	4340251	4420494	0.053595371	PASS
CORT	1	10449719	10451902	0.364796772	PASS
COTL1	16	84565594	84618077	-0.423061015	PASS
COX10	17	14069496	14208677	0.06854245	PASS
COX11	17	54951902	54968785	0.051798665	PASS
COX14	12	50111979	50120457	-0.743220499	PASS
COX15	10	99711844	99732100	-0.414937136	PASS
COX16	14	70325081	70359731	0.053252507	PASS
COX17	3	119654513	119677454	0.06434429	PASS
COX18	4	73052362	73069755	0.115781418	suppress_segment_too_large
COX19	7	898778	975599	-0.72421718	PASS
COX20	1	244835322	244845057	-0.145670469	suppress_likely_unfocal_large_gain
COX4I1	16	85798633	85807044	-0.423061015	PASS
COX4I2	20	31637888	31645006	-0.172056771	suppress_segment_too_large
COX5A	15	74919791	74938168	-0.759380635	PASS
COX5B	2	97646040	97648383	0.064122364	PASS
COX6A1	12	120438090	120440742	0.028722739	PASS
COX6A2	16	31427731	31428428	0.053595371	PASS
COX6B1	19	35648223	35658861	0.044838622	PASS
COX6B2	19	55349306	55354814	0.142581352	PASS
COX6C	8	99873200	99894062	0.355469066	suppress_segment_too_large
COX7A1	19	36150922	36152869	0.396324882	PASS
COX7A2	6	75237675	75250323	-0.250122709	PASS
COX7A2L	2	42333546	42425088	0.034263343	PASS
COX7B	23	77899438	77907373	0.158844052	PASS
COX7B2	4	46734827	46909235	0.115781418	suppress_segment_too_large
COX7C	5	86617904	86620962	-0.393084436	PASS
COX8A	11	63974607	63976543	0.064535884	PASS
COX8C	14	93347191	93348356	-0.155273506	suppress_segment_too_large
CP	3	149162410	149222055	0.02267528	PASS
CPA1	7	130380339	130388114	-0.712149877	PASS
CPA2	7	130266827	130289798	-0.712149877	PASS
CPA3	3	148865256	148897196	0.02267528	PASS
CPA4	7	130293134	130324180	-0.712149877	PASS
CPA5	7	130344816	130368730	-0.712149877	PASS
CPA6	8	67422038	67746385	0.182873287	suppress_segment_too_large
CPAMD8	19	16892947	17026815	-0.069222905	PASS
CPB1	3	148791102	148860187	0.02267528	PASS
CPB2	13	46053195	46105033	-0.398748891	PASS
CPD	17	30378905	30469989	0.041294512	PASS
CPE	4	165361194	165498320	0.142431244	suppress_segment_too_large
CPEB1	15	82543201	82648861	-0.759380635	PASS
CPEB2	4	15002674	15070153	-0.358623662	PASS
CPEB3	10	92046692	92291087	-0.414937136	PASS

CPEB4	5	173888280	173961976	0.165341871	suppress_segment_too_large
CPED1	7	120988677	121297444	-0.712149877	PASS
CPLANE1	5	37106228	37249428	0.073527871	PASS
CPLANE2	1	16231700	16237162	0.048749694	PASS
CPLX1	4	784957	826198	-0.358623662	PASS
CPLX2	5	175796310	175884020	0.165341871	suppress_segment_too_large
CPLX3	15	74826547	74831802	-0.759380635	PASS
CPLX4	18	59275156	59318649	-0.330515409	PASS
CPM	12	68842197	68971570	-0.743220499	PASS
CPN1	10	100042193	100081877	-0.414937136	PASS
CPN2	3	194339765	194351328	0.02267528	PASS
CPNE1	20	35626031	35664956	-0.172056771	suppress_segment_too_large
CPNE2	16	57092537	57148367	-0.423061015	PASS
CPNE3	8	86514427	86561498	0.617295349	suppress_segment_too_large
CPNE4	3	131533555	132285410	0.06434429	PASS
CPNE5	6	36740775	36840002	0.028532667	PASS
CPNE6	14	24070837	24078100	0.053252507	PASS
CPNE7	16	89575768	89597246	-0.423061015	PASS
CPNE8	12	38646822	38907430	0.49032213	suppress_segment_too_large
CPNE9	3	9703807	9729908	0.06149513	PASS
CPO	2	206939554	206969474	0.011001208	PASS
CPOX	3	98579446	98593723	0.22470242	PASS
CPPED1	16	12659799	12804017	0.053595371	PASS
CPQ	8	96645227	97149654	0.355469066	suppress_segment_too_large
CPS1	2	210477682	210679107	0.011001208	PASS
CPSF1	8	144393229	144409349	0.699132336	PASS
CPSF2	14	92121937	92172145	-0.155273506	suppress_segment_too_large
CPSF3	2	9423568	9473101	0.034263343	PASS
CPSF4	7	99438922	99457371	-0.710463272	PASS
CPSF4L	17	73248449	73262352	0.051798665	PASS
CPSF6	12	69239537	69274358	-0.743220499	PASS
CPSF7	11	61402641	61430031	0.064535884	PASS
CPT1A	11	68754620	68844410	0.064535884	PASS
CPT1B	22	50568861	50578465	-0.953096644	PASS
CPT1C	19	49690898	49713731	0.002743503	PASS
CPT2	1	53196429	53214197	-0.736943052	PASS
CPTP	1	1324756	1328897	-0.358281697	PASS
CPVL	7	28995231	29195451	-0.72421718	PASS
CPXCR1	23	88747225	88754785	0.158844052	PASS
CPXM1	20	2794069	2800637	-0.172056771	suppress_segment_too_large
CPXM2	10	123706207	123940267	0.073090211	PASS
CPZ	4	8592660	8619759	-0.358623662	PASS
CR1	1	207496147	207641765	-0.204996646	suppress_segment_too_large
CR1L	1	207645113	207738416	-0.204996646	suppress_segment_too_large
CR2	1	207454230	207489895	-0.204996646	suppress_segment_too_large
CRABP1	15	78340324	78348230	-0.759380635	PASS
CRABP2	1	156699606	156705816	0.088103245	PASS
CRACR2A	12	3606633	3764819	-0.015029783	PASS
CRACR2B	11	826144	831991	-0.39835217	PASS
CRADD	12	93677375	93894840	0.028722739	PASS
CRAMP1	16	1612325	1677908	0.053595371	PASS
CRAT	9	129094810	129111189	0.073960965	PASS
CRB1	1	197268204	197478455	0.088103245	PASS
CRB2	9	123356170	123380324	0.073960965	PASS

CRB3	19	6463777	6467221	-0.071973357	PASS
CRBN	3	3144628	3179727	0.06149513	PASS
CRCP	7	66114604	66154568	-0.701511252	PASS
CRCT1	1	152514502	152516010	0.088103245	PASS
CREB1	2	207529737	207603431	0.011001208	PASS
CREB3	9	35732335	35737004	0.073960965	PASS
CREB3L1	11	46277661	46321422	0.059795455	PASS
CREB3L2	7	137874979	138002067	-0.712149877	PASS
CREB3L3	19	4153601	4173054	-0.071973357	PASS
CREB3L4	1	153967534	153974363	0.088103245	PASS
CREB5	7	28299321	28825894	-0.72421718	PASS
CREBBP	16	3725054	3880726	0.053595371	PASS
CREBL2	12	12611827	12645108	0.49032213	suppress_segment_too_large
CREBRF	5	173056352	173139284	0.165341871	suppress_segment_too_large
CREBZF	11	85659708	85682908	0.064535884	PASS
CREG1	1	167529117	167553805	0.088103245	PASS
CREG2	2	101345551	101387595	0.064122364	PASS
CRELD1	3	9933822	9945413	0.06149513	PASS
CRELD2	22	49918167	49927540	-0.953096644	PASS
CREM	10	35126791	35212958	0.075711544	PASS
CRH	8	66176382	66178725	0.182873287	suppress_segment_too_large
CRHBP	5	76952713	76981158	-0.393084436	PASS
CRHR1	17	45784280	45835828	0.051798665	PASS
CRHR2	7	30651943	30700129	-0.72421718	PASS
CRIM1	2	36355926	36551135	0.034263343	PASS
CRIP1	14	105486317	105488947	-0.155273506	suppress_segment_too_large
CRIP2	14	105472962	105480170	-0.155273506	suppress_segment_too_large
CRIP3	6	43299710	43308797	0.028532667	PASS
CRIPT	2	46616416	46625742	0.034263343	PASS
CRISP1	6	49834257	49877096	0.239805577	suppress_segment_too_large
CRISP2	6	49692358	49713590	0.239805577	suppress_segment_too_large
CRISP3	6	49727384	49744437	0.239805577	suppress_segment_too_large
CRISPLD1	8	74984515	75034558	0.182873287	suppress_segment_too_large
CRISPLD2	16	84819984	84920768	-0.423061015	PASS
CRK	17	1420689	1463162	0.06854245	PASS
CRKL	22	20917426	20953749	-0.740245994	PASS
CRLF1	19	18572220	18607741	-0.069222905	PASS
CRLF2	23	1187549	1212750	-0.350651546	PASS
CRLF3	17	30769388	30824776	0.041294512	PASS
CRLS1	20	6006090	6040053	-0.172056771	suppress_segment_too_large
CRMP1	4	5748084	5893058	-0.358623662	PASS
CRNKL1	20	20034368	20056046	-0.172056771	suppress_segment_too_large
CRNN	1	152409243	152414263	0.546958914	PASS
CROCC	1	16740273	16972979	0.048749694	PASS
CROCC2	2	240906330	240993311	0.011001208	PASS
CROT	7	87345681	87399795	-0.221864321	PASS
CRP	1	159712289	159714589	0.088103245	PASS
CRTAC1	10	97865000	98030828	-0.414937136	PASS
CRTAM	11	122838500	122872639	-0.431650509	PASS
CRTAP	3	33113979	33147773	0.06149513	PASS
CRTC1	19	18683677	18782333	-0.069222905	PASS
CRTC2	1	153947669	153958625	0.088103245	PASS
CRTC3	15	90529925	90645345	-0.759380635	PASS
CRX	19	47819779	47843330	0.002743503	PASS

CRY1	12	106991364	107093829	0.028722739	PASS
CRY2	11	45847118	45883248	0.059795455	PASS
CRYAA	21	43169008	43172805	0.209983559	PASS
CRYAA2	21	6560714	6564489	0.220071762	PASS
CRYAB	11	111908565	111923722	-0.431650509	PASS
CRYBA1	17	29246863	29254494	0.041294512	PASS
CRYBA2	2	218990189	218993421	0.011001208	PASS
CRYBA4	22	26621964	26630672	-0.740245994	PASS
CRYBB1	22	26599278	26618088	-0.740245994	PASS
CRYBB2	22	25219522	25231869	-0.740245994	PASS
CRYBB3	22	25199850	25207363	-0.740245994	PASS
CRYBG1	6	106360808	106571978	-0.355603029	PASS
CRYBG2	1	26321698	26360080	0.048749694	PASS
CRYBG3	3	97822040	97944963	0.22470242	PASS
CRYGA	2	208160740	208163576	0.011001208	PASS
CRYGB	2	208142573	208146168	0.011001208	PASS
CRYGC	2	208128137	208129830	0.011001208	PASS
CRYGD	2	208121607	208124501	0.011001208	PASS
CRYGN	7	151428835	151440813	-0.712149877	PASS
CRYGS	3	186538441	186546702	0.02267528	PASS
CRYL1	13	20403666	20525873	-0.398748891	PASS
CRYM	16	21238874	21303083	0.053595371	PASS
CRYZ	1	74705482	74733408	0.052232535	PASS
CRYZL1	21	33589341	33643926	0.209983559	PASS
CS	12	56271699	56300392	-0.743220499	PASS
CSAD	12	53157663	53180909	-0.743220499	PASS
CSAG1	23	152727484	152733735	0.158844052	PASS
CSAG3	23	152753921	152760222	0.158844052	PASS
CSDC2	22	41560763	41577741	-0.740245994	PASS
CSDE1	1	114716913	114758676	0.079255572	PASS
CSE1L	20	49046246	49096960	-0.172056771	suppress_segment_too_large
CSF1	1	109910242	109930992	0.079255572	PASS
CSF1R	5	150053291	150113372	-0.393084436	PASS
CSF2	5	132073790	132076170	-0.393084436	PASS
CSF2RA	23	1268800	1310381	-0.350651546	PASS
CSF2RB	22	36913628	36940449	-0.740245994	PASS
CSF3	17	40015361	40017813	0.041294512	PASS
CSF3R	1	36466043	36483278	0.048749694	PASS
CSGALNA	8	19404161	19758029	-0.311940419	PASS
CSGALNA	10	43138486	43185308	-0.389033546	PASS
CSH1	17	63894909	63896661	0.051798665	PASS
CSH2	17	63872012	63873766	0.051798665	PASS
CSHL1	17	63909597	63918838	0.051798665	PASS
CSK	15	74782057	74803198	-0.759380635	PASS
CSKMT	11	62665309	62668496	0.064535884	PASS
CSMD1	8	2935353	4994972	-0.311940419	PASS
CSMD2	1	33513999	34165842	0.048749694	PASS
CSMD3	8	112222928	113437099	0.70944438	suppress_segment_too_large
CSN1S1	4	69931081	69946574	0.115781418	suppress_segment_too_large
CSN2	4	69955256	69961007	0.115781418	suppress_segment_too_large
CSN3	4	70242588	70251428	0.115781418	suppress_segment_too_large
CSNK1A1	5	149492197	149551552	-0.393084436	PASS
CSNK1A1	13	37103259	37105664	-0.398748891	PASS
CSNK1D	17	82239023	82273731	0.051798665	PASS

CSNK1E	22	38290691	38318084	-0.740245994	PASS
CSNK1G1	15	64165517	64356259	-0.759380635	PASS
CSNK1G2	19	1941149	1981338	-0.071973357	PASS
CSNK1G3	5	123512099	123617045	-0.393084436	PASS
CSNK2A1	20	472498	543835	-0.172056771	suppress_segment_too_large
CSNK2A2	16	58157907	58197920	-0.423061015	PASS
CSNK2A3	11	11351942	11353357	-0.39835217	PASS
CSNK2B	6	31665236	31670343	0.028532667	PASS
CSPG4	15	75674322	75712848	-0.759380635	PASS
CSPG5	3	47562239	47580792	-0.727808231	PASS
CSPP1	8	67062426	67196263	0.182873287	suppress_segment_too_large
CSRNP1	3	39141855	39154562	-0.727808231	PASS
CSRNP2	12	51061205	51083664	-0.743220499	PASS
CSRNP3	2	165469647	165689407	0.319987345	PASS
CSRP1	1	201483530	201509456	-0.204996646	suppress_segment_too_large
CSRP2	12	76858715	76879060	0.271127429	suppress_segment_too_large
CSRP3	11	19182037	19210571	-0.39835217	PASS
CST1	20	23747553	23751268	-0.172056771	suppress_segment_too_large
CST11	20	23450403	23452857	-0.172056771	suppress_segment_too_large
CST2	20	23823769	23826731	-0.172056771	suppress_segment_too_large
CST3	20	23626706	23638473	-0.172056771	suppress_segment_too_large
CST4	20	23685640	23689040	-0.172056771	suppress_segment_too_large
CST5	20	23875935	23879750	-0.172056771	suppress_segment_too_large
CST6	11	66011841	66013505	0.064535884	PASS
CST7	20	24949230	24959928	-0.172056771	suppress_segment_too_large
CST8	20	23491101	23496018	-0.172056771	suppress_segment_too_large
CST9	20	23602410	23605876	-0.172056771	suppress_segment_too_large
CST9L	20	23564732	23568749	-0.172056771	suppress_segment_too_large
CSTA	3	122325244	122341972	0.06434429	PASS
CSTB	21	43772511	43776445	0.209983559	PASS
CSTF1	20	56392371	56406369	-0.172056771	suppress_segment_too_large
CSTF2	23	100820359	100840932	0.158844052	PASS
CSTF2T	10	51695487	51699591	-0.389033546	PASS
CSTF3	11	33077188	33162371	-0.359426047	PASS
CSTL1	20	23439685	23444930	-0.172056771	suppress_segment_too_large
CT45A1	23	135713453	135723539	0.158844052	PASS
CT45A10	23	135881063	135889086	0.158844052	PASS
CT45A3	23	135759846	135768191	0.158844052	PASS
CT47B1	23	120872603	120875929	0.158844052	PASS
CT55	23	135156536	135171398	0.158844052	PASS
CT62	15	71110244	71115500	-0.759380635	PASS
CT83	23	116461682	116463003	0.158844052	PASS
CTAG2	23	154651972	154653579	0.158844052	PASS
CTAGE1	18	22413601	22417915	-0.330515409	PASS
CTAGE15	7	143571801	143574387	-0.712149877	PASS
CTAGE4	7	144183466	144186053	-0.712149877	PASS
CTAGE6	7	143755089	143757696	-0.712149877	PASS
CTAGE8	7	144266701	144269288	-0.712149877	PASS
CTAGE9	6	131708684	131711017	-0.304168115	PASS
CTBP1	4	1211448	1249953	-0.358623662	PASS
CTBP2	10	124984317	125161170	0.073090211	PASS
CTBS	1	84549606	84574480	0.587598296	PASS
CTC1	17	8224821	8248095	0.06854245	PASS
CTCF	16	67562407	67639185	-0.423061015	PASS

CTCFL	20	57495966	57525652		-0.172056771	suppress_segment_too_large
CTDNEP1	17	7243591	7252491		0.06854245	PASS
CTDP1	18	79679801	79756623		-0.330515409	PASS
CTDSP1	2	218398256	218405941		0.011001208	PASS
CTDSP2	12	57819927	57846739		-0.743220499	PASS
CTDSPL	3	37861960	37984469		-0.727808231	PASS
CTDSPL2	15	44427234	44529038		-0.423772929	PASS
CTF1	16	30896607	30903560		0.053595371	PASS
CTGF	6	131948176	131951373		-0.304168115	PASS
CTH	1	70411218	70439851		0.052232535	PASS
CTHRC1	8	103371515	103382997		0.355469066	suppress_segment_too_large
CTIF	18	48539046	48863217		-0.330515409	PASS
CTLA4	2	203867771	203873965		0.011001208	PASS
CTNNA1	5	138610967	138935034		-0.393084436	PASS
CTNNA2	2	79185231	80648861		-0.46122572	PASS
CTNNA3	10	65912518	67696169		0.032351597	PASS
CTNNAL1	9	108942569	109013529		0.073960965	PASS
CTNNB1	3	41194741	41260096		-0.727808231	PASS
CTNNBIP1	1	9848276	9910336		0.364796772	PASS
CTNNBL1	20	37693955	37872129		-0.172056771	suppress_segment_too_large
CTNND1	11	57753243	57819546		0.27006553	suppress_segment_too_large
CTNND2	5	10971840	11904043		0.073527871	PASS
CTNS	17	3636468	3661542		0.06854245	PASS
CTPS1	1	40979335	41012565		0.048749694	PASS
CTPS2	23	16588003	16712936		0.158844052	PASS
CTR9	11	10750987	10801625		-0.39835217	PASS
CTRB1	16	75219000	75226338		-0.423061015	PASS
CTRB2	16	75204096	75207185		-0.423061015	PASS
CTRC	1	15438439	15449242		0.048749694	PASS
CTRL	16	67927640	67932414		-0.423061015	PASS
CTSA	20	45890144	45898820		-0.172056771	suppress_segment_too_large
CTSB	8	11842524	11869448		-0.311940419	PASS
CTSC	11	88293592	88337787		0.064535884	PASS
CTSD	11	1752752	1764573		-0.39835217	PASS
CTSE	1	206009264	206023909		-0.204996646	suppress_segment_too_large
CTSF	11	66563463	66568841		0.064535884	PASS
CTSG	14	24573522	24576260		0.053252507	PASS
CTSH	15	78921058	78949574		-0.759380635	PASS
CTSK	1	150796208	150808323		0.079255572	PASS
CTSL	9	87725519	87731393		0.073960965	PASS
CTSO	4	155924118	155953917		0.142431244	suppress_segment_too_large
CTSS	1	150730196	150765957		0.079255572	PASS
CTSV	9	97029679	97039643		0.073960965	PASS
CTSW	11	65879809	65883741		0.064535884	PASS
CTSZ	20	58995185	59007247		-0.172056771	suppress_segment_too_large
CTTN	11	70398404	70436584		0.064535884	PASS
CTTNBP2	7	117710651	117874139		-0.712149877	PASS
CTTNBP2[illegible]	1	112396181	112463456		0.079255572	PASS
CTU1	19	51097606	51108370		0.002743503	PASS
CTU2	16	88706463	88715386		-0.423061015	PASS
CTXN1	19	7924485	7926166		-0.071973357	PASS
CTXN2	15	48178122	48203756		-0.423772929	PASS
CTXN3	5	127649044	127658630		-0.393084436	PASS
CTXND1	15	80195484	80252213		-0.759380635	PASS

CTXND2	1	150887136	150913292	0.079255572	PASS
CUBN	10	16823966	17129817	0.075711544	PASS
CUEDC1	17	57861243	57955323	0.051798665	PASS
CUEDC2	10	102423245	102432661	-0.414937136	PASS
CUL1	7	148697914	148801036	-0.712149877	PASS
CUL2	10	35008551	35090642	0.075711544	PASS
CUL3	2	224470150	224585397	0.011001208	PASS
CUL4A	13	113208193	113267108	-0.426822582	PASS
CUL4B	23	120524609	120575794	0.158844052	PASS
CUL5	11	108008733	108107776	-0.431650509	PASS
CUL7	6	43037617	43053945	0.028532667	PASS
CUL9	6	43182175	43224587	0.028532667	PASS
CUTA	6	33416442	33418317	0.028532667	PASS
CUTC	10	99702558	99756134	-0.414937136	PASS
CUX1	7	101815904	102283958	-0.712149877	PASS
CUX2	12	111034024	111350554	0.028722739	PASS
CUZD1	10	122832149	122850793	0.073090211	PASS
CWC15	11	94962622	94973612	0.064535884	PASS
CWC22	2	179944876	180007113	0.162734012	PASS
CWC25	17	38800434	38825481	0.041294512	PASS
CWC27	5	64768930	65018763	-0.393084436	PASS
CWF19L1	10	100232298	100267680	-0.414937136	PASS
CWF19L2	11	107326345	107457844	-0.431650509	PASS
CWH43	4	48986247	49062081	0.115781418	suppress_segment_too_large
CX3CL1	16	57372458	57385048	-0.423061015	PASS
CX3CR1	3	39263494	39281735	-0.727808231	PASS
CXADR	21	17512382	17593579	0.758930684	suppress_segment_too_large
CXCL1	4	73869393	73871242	0.115781418	suppress_segment_too_large
CXCL10	4	76021117	76023497	-0.677982248	PASS
CXCL11	4	76033682	76041415	-0.677982248	PASS
CXCL12	10	44370165	44386493	-0.389033546	PASS
CXCL13	4	77511753	77611834	-0.677982248	PASS
CXCL14	5	135570679	135579279	-0.393084436	PASS
CXCL16	17	4733526	4739922	0.06854245	PASS
CXCL17	19	42428288	42443048	0.048917043	PASS
CXCL2	4	74097035	74099293	0.115781418	suppress_segment_too_large
CXCL3	4	74036589	74038807	0.115781418	suppress_segment_too_large
CXCL5	4	73995642	73998779	0.115781418	suppress_segment_too_large
CXCL6	4	73836497	73849064	0.115781418	suppress_segment_too_large
CXCL8	4	73740506	73743716	0.115781418	suppress_segment_too_large
CXCL9	4	76001275	76007488	-0.677982248	PASS
CXCR1	2	218162845	218166995	0.011001208	PASS
CXCR2	2	218125289	218137253	0.011001208	PASS
CXCR3	23	71615916	71618517	0.158844052	PASS
CXCR4	2	136114349	136118165	-0.359440172	PASS
CXCR5	11	118883766	118897799	-0.431650509	PASS
CXCR6	3	45940933	45948353	-0.727808231	PASS
CXXC1	18	50282343	50288304	-0.330515409	PASS
CXXC4	4	104468312	104494901	-0.677982248	PASS
CXXC5	5	139647299	139683882	-0.393084436	PASS
CXorf21	23	30558824	30577844	0.158844052	PASS
CXorf36	23	45148374	45200901	0.158844052	PASS
CXorf38	23	40626921	40647554	0.158844052	PASS
CXorf40A	23	149540355	149550510	0.158844052	PASS

CXorf40B	23	149929527	149938811	0.158844052	PASS
CXorf56	23	119538149	119565408	0.158844052	PASS
CXorf57	23	106611930	106679442	0.158844052	PASS
CXorf58	23	23907801	23939509	0.158844052	PASS
CXorf65	23	71103889	71106788	0.158844052	PASS
CXorf66	23	139955725	139965520	0.158844052	PASS
CXorf67	23	51406915	51408843	0.158844052	PASS
CYB561	17	63432304	63446378	0.051798665	PASS
CYB561A3	11	61348745	61362299	0.064535884	PASS
CYB561D1	1	109494052	109502932	0.079255572	PASS
CYB561D2	3	50350695	50358460	-0.727808231	PASS
CYB5A	18	74250847	74292016	-0.330515409	PASS
CYB5B	16	69424525	69466266	-0.423061015	PASS
CYB5D1	17	7857746	7862282	0.06854245	PASS
CYB5D2	17	4143168	4187310	0.06854245	PASS
CYB5R1	1	202961869	202967280	-0.204996646	suppress_segment_too_large
CYB5R2	11	7665100	7677222	-0.39835217	PASS
CYB5R3	22	42617840	42649568	-0.740245994	PASS
CYB5R4	6	83859643	83967424	-0.355603029	PASS
CYB5RL	1	54169651	54200036	-0.736943052	PASS
CYBA	16	88643283	88651152	-0.423061015	PASS
CYBB	23	37780011	37813461	0.158844052	PASS
CYBC1	17	82442589	82450829	0.051798665	PASS
CYBRD1	2	171522247	171558133	0.033541467	PASS
CYC1	8	144095027	144097525	0.699132336	PASS
CYCS	7	25120091	25125361	-0.72421718	PASS
CYFIP1	15	22867052	22981063	-0.423772929	PASS
CYFIP2	5	157266079	157395595	0.165341871	suppress_segment_too_large
CYGB	17	76527356	76551175	0.051798665	PASS
CYHR1	8	144449582	144465677	0.699132336	PASS
CYLC1	23	83861126	83886699	0.158844052	PASS
CYLC2	9	102995311	103018488	0.073960965	PASS
CYLD	16	50742050	50801935	0.411512417	PASS
CYP11A1	15	74337759	74367740	-0.759380635	PASS
CYP11B1	8	142872356	142879846	0.699132336	PASS
CYP11B2	8	142910559	142917843	0.699132336	PASS
CYP17A1	10	102830531	102837533	-0.414937136	PASS
CYP19A1	15	51208057	51338610	-0.423772929	PASS
CYP1A1	15	74719542	74725610	-0.759380635	PASS
CYP1A2	15	74748844	74756202	-0.759380635	PASS
CYP1B1	2	38066973	38109902	0.034263343	PASS
CYP20A1	2	203238940	203305611	0.011001208	PASS
CYP21A2	6	32038265	32041670	0.028532667	PASS
CYP24A1	20	54153449	54173973	-0.172056771	suppress_segment_too_large
CYP26A1	10	93073475	93077890	-0.414937136	PASS
CYP26B1	2	72129238	72148038	-0.46122572	PASS
CYP26C1	10	93060808	93069536	-0.414937136	PASS
CYP27A1	2	218781749	218815293	0.011001208	PASS
CYP27B1	12	57762334	57768986	-0.743220499	PASS
CYP27C1	2	127184120	127220078	-0.359440172	PASS
CYP2A13	19	41088472	41096195	0.048917043	PASS
CYP2A6	19	40843538	40850447	0.048917043	PASS
CYP2A7	19	40875439	40882752	0.048917043	PASS
CYP2B6	19	40991250	41018398	0.048917043	PASS

CYP2C18	10	94683621	94736190	-0.414937136	PASS
CYP2C19	10	94762681	94853205	-0.414937136	PASS
CYP2C8	10	95036772	95069497	-0.414937136	PASS
CYP2C9	10	94938658	94989391	-0.414937136	PASS
CYP2D6	22	42126499	42130906	-0.740245994	PASS
CYP2E1	10	133520406	133561220	0.073090211	PASS
CYP2F1	19	41114432	41128366	0.048917043	PASS
CYP2J2	1	59893308	59926790	-0.736943052	PASS
CYP2R1	11	14877440	14892252	-0.39835217	PASS
CYP2S1	19	41193049	41207539	0.048917043	PASS
CYP2U1	4	107931369	107953457	-0.677982248	PASS
CYP2W1	7	983199	989640	-0.72421718	PASS
CYP39A1	6	46549580	46652830	0.239805577	suppress_segment_too_large
CYP3A4	7	99756960	99784265	-0.710463272	PASS
CYP3A43	7	99828013	99866102	-0.710463272	PASS
CYP3A5	7	99648194	99680026	-0.710463272	PASS
CYP3A7	7	99684957	99735196	-0.710463272	PASS
CYP3A7-C	7	99685226	99735093	-0.710463272	PASS
CYP46A1	14	99684304	99727301	-0.155273506	suppress_segment_too_large
CYP4A11	1	46929177	46941484	-0.736943052	PASS
CYP4A22	1	47137435	47149741	-0.736943052	PASS
CYP4B1	1	46757838	46819413	-0.736943052	PASS
CYP4F11	19	15912367	15934867	-0.069222905	PASS
CYP4F12	19	15672757	15697174	-0.069222905	PASS
CYP4F2	19	15878023	15898120	-0.069222905	PASS
CYP4F22	19	15508493	15552317	-0.069222905	PASS
CYP4F3	19	15640897	15662825	-0.069222905	PASS
CYP4F8	19	15615218	15630638	-0.069222905	PASS
CYP4V2	4	186191520	186213456	0.275137421	PASS
CYP4X1	1	47023568	47050751	-0.736943052	PASS
CYP4Z1	1	47067488	47118319	-0.736943052	PASS
CYP51A1	7	92112151	92134803	-0.710463272	PASS
CYP7A1	8	58490178	58500236	0.182873287	suppress_segment_too_large
CYP7B1	8	64587763	64798761	0.182873287	suppress_segment_too_large
CYP8B1	3	42856005	42876165	-0.727808231	PASS
CYR61	1	85580761	85583962	0.587598296	PASS
CYREN	7	135092363	135170795	-0.712149877	PASS
CYS1	2	10056780	10080944	0.034263343	PASS
CYSLTR1	23	78271464	78327691	0.158844052	PASS
CYSLTR2	13	48653711	48711226	-0.384313056	PASS
CYSRT1	9	137224635	137226311	0.073960965	PASS
CYSTM1	5	140174642	140282052	-0.393084436	PASS
CYTH1	17	78674048	78782297	0.051798665	PASS
CYTH2	19	48469032	48482314	0.002743503	PASS
CYTH3	7	6161776	6272644	-0.72421718	PASS
CYTH4	22	37282027	37315345	-0.740245994	PASS
CYTIP	2	157414619	157488961	-0.30549516	PASS
CYTL1	4	5014586	5019472	-0.358623662	PASS
CYYR1	21	26466209	26573284	0.209983559	PASS
DAAM1	14	59188646	59371405	0.053252507	PASS
DAAM2	6	39792298	39904877	0.028532667	PASS
DAB1	1	56994778	58546734	-0.736943052	PASS
DAB2	5	39371675	39462300	-0.284712224	PASS
DAB2IP	9	121567057	121785530	0.073960965	PASS

DACH1	13	71437966	71867192	-0.384313056	PASS
DACH2	23	86148458	86832604	0.158844052	PASS
DACT1	14	58633967	58648321	0.053252507	PASS
DACT2	6	168292830	168319754	0.033894706	PASS
DACT3	19	46647612	46661138	0.002743503	PASS
DAD1	14	22564905	22589269	0.053252507	PASS
DAG1	3	49468703	49535618	-0.727808231	PASS
DAGLA	11	61680433	61747001	0.064535884	PASS
DAGLB	7	6409126	6484190	-0.72421718	PASS
DALRD3	3	49015488	49022293	-0.727808231	PASS
DAND5	19	12965159	12974762	-0.069222905	PASS
DAO	12	108858932	108901043	0.028722739	PASS
DAOA	13	105465867	105491034	-0.426822582	PASS
DAP	5	10679230	10761272	0.073527871	PASS
DAP3	1	155687960	155739010	0.088103245	PASS
DAPK1	9	87497228	87708633	0.073960965	PASS
DAPK2	15	63907036	64072033	-0.759380635	PASS
DAPK3	19	3958453	3971123	-0.071973357	PASS
DAPL1	2	158795317	158862781	0.054749241	PASS
DAPP1	4	99816833	99870154	-0.677982248	PASS
DARS	2	135906677	135986100	-0.359440172	PASS
DARS2	1	173824568	173858808	0.088103245	PASS
DAW1	2	227871054	227924344	0.011001208	PASS
DAXX	6	33318558	33323016	0.028532667	PASS
DAZAP1	19	1407569	1435687	-0.071973357	PASS
DAZAP2	12	51238292	51271362	-0.743220499	PASS
DAZL	3	16586792	16670306	0.06149513	PASS
DBF4	7	87876216	87909541	-0.221864321	PASS
DBF4B	17	44708608	44752264	0.051798665	PASS
DBH	9	133635348	133659344	0.073960965	PASS
DBI	2	119366921	119372560	-0.359440172	PASS
DBN1	5	177456608	177474401	0.165341871	suppress_segment_too_large
DBNDD1	16	90004865	90020128	-0.423061015	PASS
DBNDD2	20	45406057	45410610	-0.172056771	suppress_segment_too_large
DBNL	7	44044640	44069456	-0.72421718	PASS
DBP	19	48630030	48637438	0.002743503	PASS
DBR1	3	138161012	138174949	0.06434429	PASS
DBT	1	100186919	100249834	0.079255572	PASS
DBX1	11	20156155	20160613	-0.39835217	PASS
DBX2	12	45014672	45051099	-0.46813528	PASS
DCAF1	3	51395867	51500002	-0.727808231	PASS
DCAF10	9	37800502	37867666	0.073960965	PASS
DCAF11	14	24114195	24125242	0.053252507	PASS
DCAF12	9	34086387	34127399	0.073960965	PASS
DCAF12L1	23	126549383	126552851	0.158844052	PASS
DCAF12L2	23	126163499	126166097	0.158844052	PASS
DCAF13	8	103414714	103443453	0.70944438	suppress_segment_too_large
DCAF15	19	13952492	13961449	-0.069222905	PASS
DCAF16	4	17800655	17810758	-0.358623662	PASS
DCAF17	2	171434217	171485052	0.033541467	PASS
DCAF4	14	72926332	72959703	0.053252507	PASS
DCAF4L1	4	41981696	41986467	-0.753159364	PASS
DCAF4L2	8	87870743	87874068	0.617295349	suppress_segment_too_large
DCAF5	14	69050881	69153150	0.053252507	PASS

DCAF6	1	167935783	168075843	0.088103245	PASS
DCAF7	17	63550461	63594266	0.051798665	PASS
DCAF8	1	160215715	160262531	0.088103245	PASS
DCAF8L1	23	27977993	27981449	0.158844052	PASS
DCAF8L2	23	27590382	27748821	0.158844052	PASS
DCAKD	17	45023340	45061109	0.051798665	PASS
DCANP1	5	135444214	135447348	-0.393084436	PASS
DCBLD1	6	117453817	117569858	-0.355603029	PASS
DCBLD2	3	98795941	98901689	0.22470242	PASS
DCC	18	52340172	53535903	-0.330515409	PASS
DCD	12	54644591	54648493	-0.743220499	PASS
DCDC1	11	30830369	31369810	-0.359426047	PASS
DCDC2	6	24171756	24358052	0.069043418	PASS
DCDC2B	1	32209094	32216196	0.048749694	PASS
DCDC2C	2	3703592	3847411	0.034263343	PASS
DCHS1	11	6621323	6655854	-0.39835217	PASS
DCHS2	4	154231742	154495570	0.142431244	suppress_segment_too_large
DCK	4	70992538	71030914	0.115781418	suppress_segment_too_large
DCLK1	13	35768652	36131306	-0.398748891	PASS
DCLK2	4	150078274	150257457	0.142431244	suppress_segment_too_large
DCLK3	3	36712422	36764349	-0.727808231	PASS
DCLRE1A	10	113834725	113854383	0.073090211	PASS
DCLRE1B	1	113905141	113914086	0.079255572	PASS
DCLRE1C	10	14897359	14954432	0.075711544	PASS
DCN	12	91140484	91183123	0.271127429	suppress_segment_too_large
DCP1A	3	53283428	53347610	-0.727808231	PASS
DCP1B	12	1946054	2004535	-0.015029783	PASS
DCP2	5	112976702	113020970	-0.393084436	PASS
DCPS	11	126303752	126345754	-0.431650509	PASS
DCST1	1	155033824	155050930	0.088103245	PASS
DCST2	1	155018520	155033781	0.088103245	PASS
DCSTAMI	8	104339087	104356689	0.70944438	suppress_segment_too_large
DCT	13	94436808	94479682	-0.384313056	PASS
DCTD	4	182890060	182917936	-0.453876349	PASS
DCTN1	2	74361154	74392087	-0.46122572	PASS
DCTN2	12	57530102	57547331	-0.743220499	PASS
DCTN3	9	34613545	34620523	0.073960965	PASS
DCTN4	5	150708440	150759109	-0.393084436	PASS
DCTN5	16	23641392	23677455	0.053595371	PASS
DCTN6	8	30156297	30183640	-0.311940419	PASS
DCTPP1	16	30423619	30430075	0.053595371	PASS
DCUN1D1	3	182938074	182985953	0.02267528	PASS
DCUN1D2	13	113455819	113490952	-0.426822582	PASS
DCUN1D3	16	20854925	20900384	0.053595371	PASS
DCUN1D4	4	51843000	51916837	0.115781418	suppress_segment_too_large
DCUN1D5	11	103062076	103092215	0.32805343	suppress_segment_too_large
DCX	23	111293779	111412429	0.158844052	PASS
DCXR	17	82035136	82037732	0.051798665	PASS
DDA1	19	17309518	17323301	-0.069222905	PASS
DDAH1	1	85318481	85578363	0.587598296	PASS
DDAH2	6	31727038	31730617	0.028532667	PASS
DDB1	11	61299451	61342596	0.064535884	PASS
DDB2	11	47214465	47239240	0.059795455	PASS
DDC	7	50458436	50565457	-0.701511252	PASS

DDHD1	14	53036745	53153282	0.053252507	PASS
DDHD2	8	38225218	38275558	0.182873287	suppress_segment_too_large
DDI1	11	104036580	104039188	0.32805343	suppress_segment_too_large
DDI2	1	15617500	15669044	0.048749694	PASS
DDIAS	11	82899975	82958277	0.064535884	PASS
DDIT3	12	57516588	57521737	-0.743220499	PASS
DDIT4	10	72273920	72276036	0.032351597	PASS
DDIT4L	4	100185870	100190782	-0.677982248	PASS
DDN	12	48995149	48999309	-0.743220499	PASS
DDO	6	110391771	110415562	-0.355603029	PASS
DDOST	1	20651767	20661544	0.048749694	PASS
DDR1	6	30876421	30900156	0.069043418	PASS
DDR2	1	162631373	162787400	0.088103245	PASS
DDRGK1	20	3190350	3204685	-0.172056771	suppress_segment_too_large
DDT	22	23971365	23979828	-0.740245994	PASS
DDTL	22	23966901	23972532	-0.740245994	PASS
DDX1	2	15591178	15631111	0.034263343	PASS
DDX10	11	108665025	108940930	-0.431650509	PASS
DDX11	12	31073845	31104791	0.49032213	suppress_segment_too_large
DDX17	22	38483440	38507660	-0.740245994	PASS
DDX18	2	117814650	117832379	-0.359440172	PASS
DDX19A	16	70346829	70373383	-0.423061015	PASS
DDX19B	16	70289663	70335283	-0.423061015	PASS
DDX20	1	111755245	111768016	0.079255572	PASS
DDX21	10	68956128	68985073	0.032351597	PASS
DDX23	12	48829764	48852842	-0.743220499	PASS
DDX24	14	94048291	94081245	-0.155273506	suppress_segment_too_large
DDX25	11	125903348	125943702	-0.431650509	PASS
DDX27	20	49219295	49244077	-0.172056771	suppress_segment_too_large
DDX28	16	68021274	68023442	-0.423061015	PASS
DDX31	9	132592997	132670401	0.073960965	PASS
DDX39A	19	14408819	14419383	-0.069222905	PASS
DDX39B	6	31530219	31542448	0.028532667	PASS
DDX3X	23	41333284	41364472	0.158844052	PASS
DDX4	5	55738017	55817157	-0.284712224	PASS
DDX41	5	177511577	177517469	0.165341871	suppress_segment_too_large
DDX42	17	63773603	63819317	0.051798665	PASS
DDX43	6	73394748	73417569	-0.250122709	PASS
DDX46	5	134758771	134855133	-0.393084436	PASS
DDX47	12	12813316	12829981	0.49032213	suppress_segment_too_large
DDX49	19	18919675	18928633	-0.069222905	PASS
DDX5	17	64499616	64508199	0.051798665	PASS
DDX50	10	68901278	68946847	0.032351597	PASS
DDX51	12	132136594	132144335	0.028722739	PASS
DDX52	17	37609739	37643464	0.041294512	PASS
DDX53	23	22999961	23003589	0.158844052	PASS
DDX54	12	113157174	113185479	0.028722739	PASS
DDX55	12	123602077	123620941	0.028722739	PASS
DDX56	7	44565417	44575051	-0.72421718	PASS
DDX58	9	32455705	32526324	0.073960965	PASS
DDX59	1	200623896	200669969	-0.204996646	suppress_segment_too_large
DDX6	11	118747766	118791149	-0.431650509	PASS
DDX60	4	168216293	168318807	0.142431244	suppress_segment_too_large
DDX60L	4	168356735	168537786	0.142431244	suppress_segment_too_large

DEAF1	11	644233	706715	-0.39835217	PASS
Dec1	9	114850968	115402644	0.073960965	PASS
DECR1	8	90001405	90052092	0.617295349	suppress_segment_too_large
DECR2	16	401826	412487	0.053595371	PASS
DEDD	1	161120974	161132688	0.088103245	PASS
DEDD2	19	42198598	42220140	0.048917043	PASS
DEF6	6	35297852	35321771	0.028532667	PASS
DEF8	16	89947925	89968060	-0.423061015	PASS
DEFA3	8	7015869	7018301	-0.311940419	PASS
DEFA4	8	6935822	6938338	-0.311940419	PASS
DEFA5	8	7055304	7056734	-0.311940419	PASS
DEFA6	8	6924693	6926076	-0.311940419	PASS
DEFB1	8	6870575	6878022	-0.311940419	PASS
DEFB104A	8	7836471	7841242	-0.311940419	PASS
DEFB105A	8	7821966	7823889	-0.311940419	PASS
DEFB108E	11	71833200	71837710	0.064535884	PASS
DEFB110	6	50009138	50021981	0.239805577	suppress_segment_too_large
DEFB112	6	50043575	50048651	0.239805577	suppress_segment_too_large
DEFB113	6	49968677	49969625	0.239805577	suppress_segment_too_large
DEFB114	6	49960249	49964105	0.239805577	suppress_segment_too_large
DEFB115	20	31257664	31259632	-0.172056771	suppress_segment_too_large
DEFB116	20	31303212	31308585	-0.172056771	suppress_segment_too_large
DEFB118	20	31368618	31373923	-0.172056771	suppress_segment_too_large
DEFB119	20	31377164	31390603	-0.172056771	suppress_segment_too_large
DEFB121	20	31404845	31412838	-0.172056771	suppress_segment_too_large
DEFB123	20	31440519	31450257	-0.172056771	suppress_segment_too_large
DEFB124	20	31465506	31476757	-0.172056771	suppress_segment_too_large
DEFB125	20	87250	97094	-0.172056771	suppress_segment_too_large
DEFB126	20	142369	145751	-0.172056771	suppress_segment_too_large
DEFB127	20	157470	159163	-0.172056771	suppress_segment_too_large
DEFB128	20	187853	189681	-0.172056771	suppress_segment_too_large
DEFB129	20	227258	229886	-0.172056771	suppress_segment_too_large
DEFB131A	4	9444534	9450514	-0.358623662	PASS
DEFB131E	11	71878453	71884561	0.064535884	PASS
DEFB132	20	257736	261096	-0.172056771	suppress_segment_too_large
DEFB133	6	49946021	49950265	0.239805577	suppress_segment_too_large
DEFB134	8	11993174	11996312	-0.311940419	PASS
DEFB135	8	11982321	11984590	-0.311940419	PASS
DEFB136	8	11973937	11974599	-0.311940419	PASS
DEFB4A	8	7894629	7896711	-0.311940419	PASS
DEFB4B	8	7414860	7416863	-0.311940419	PASS
DEGS1	1	224175756	224193441	-0.204996646	suppress_segment_too_large
DEGS2	14	100143957	100160163	-0.155273506	suppress_segment_too_large
DEK	6	18223868	18264823	0.069043418	PASS
DELE1	5	141923808	141942047	-0.393084436	PASS
DENND1A	9	123379654	123930152	0.073960965	PASS
DENND1E	1	197504748	197775696	0.088103245	PASS
DENND1C	19	6467207	6482557	-0.071973357	PASS
DENND2A	7	140518420	140673993	-0.712149877	PASS
DENND2C	1	114582848	114670422	0.079255572	PASS
DENND2D	1	111187174	111204535	0.079255572	PASS
DENND3	8	141117278	141195808	0.493344802	suppress_segment_too_large
DENND4A	15	65658046	65792293	-0.759380635	PASS
DENND4E	1	153929501	153946696	0.088103245	PASS

DENND4C	9	19230435	19373545	0.073960965	PASS
DENND5A	11	9138825	9265390	-0.39835217	PASS
DENND5E	12	31382223	31591097	0.49032213	suppress_segment_too_large
DENND6A	3	57625457	57693089	-0.727808231	PASS
DENND6E	22	50309030	50327060	-0.953096644	PASS
DENR	12	122752774	122771064	0.028722739	PASS
DEPDC1	1	68474152	68497221	0.052232535	PASS
DEPDC1B	5	60596912	60700190	0.07724032	PASS
DEPDC4	12	100203669	100267079	0.028722739	PASS
DEPDC5	22	31753867	31908033	-0.740245994	PASS
DEPDC7	11	33015864	33033582	-0.359426047	PASS
DEPP1	10	44970981	44978810	-0.389033546	PASS
DEPTOR	8	119873717	120050913	0.493344802	suppress_segment_too_large
DERA	12	15911172	16037282	0.49032213	suppress_segment_too_large
DERL1	8	123013164	123042423	0.493344802	suppress_segment_too_large
DERL2	17	5471251	5486811	0.06854245	PASS
DERL3	22	23834503	23839128	-0.740245994	PASS
DES	2	219418377	219426739	0.011001208	PASS
DESI1	22	41598028	41621096	-0.740245994	PASS
DESI2	1	244652935	244709033	-0.145670469	suppress_likely_unfocal_large_gain
DET1	15	88494440	88546675	-0.759380635	PASS
DEUP1	11	93329971	93438487	0.064535884	PASS
DEXI	16	10928891	10942460	0.053595371	PASS
DFFA	1	10456522	10472526	0.364796772	PASS
DFFB	1	3857267	3885429	-0.358281697	PASS
DGAT1	8	144314584	144326910	0.699132336	PASS
DGAT2	11	75759512	75801535	0.064535884	PASS
DGAT2L6	23	70177483	70205545	0.158844052	PASS
DGCR2	22	19036282	19122454	-0.740245994	PASS
DGCR6	22	18906028	18914238	-0.740245994	PASS
DGCR6L	22	20314276	20320080	-0.740245994	PASS
DGCR8	22	20080232	20111877	-0.740245994	PASS
DGKA	12	55927319	55954027	-0.743220499	PASS
DGKB	7	14145049	14974777	-0.72421718	PASS
DGKD	2	233354507	233472104	0.011001208	PASS
DGKE	17	56834099	56869567	0.051798665	PASS
DGKG	3	186105668	186362237	0.02267528	PASS
DGKH	13	42040036	42256578	-0.398748891	PASS
DGKI	7	137381037	137847092	-0.712149877	PASS
DGKK	23	50365409	50470738	0.158844052	PASS
DGKQ	4	958887	986895	-0.358623662	PASS
DGKZ	11	46332905	46380554	0.059795455	PASS
DGLUCY	14	91060333	91225632	-0.155273506	suppress_segment_too_large
DGUOK	2	73926826	73958961	-0.46122572	PASS
DHCR24	1	54849627	54887271	-0.736943052	PASS
DHCR7	11	71428193	71452868	0.064535884	PASS
DHDDS	1	26432282	26471294	0.048749694	PASS
DHDH	19	48933682	48944969	0.002743503	PASS
DHFR	5	80626228	80654983	-0.393084436	PASS
DHFR2	3	94047836	94063389	0.22470242	PASS
DHH	12	49086656	49094819	-0.743220499	PASS
DHODH	16	72008588	72027664	-0.423061015	PASS
DHPS	19	12675717	12681902	-0.069222905	PASS
DHRS1	14	24290598	24299833	0.053252507	PASS

DHRS11	17	36591798	36600806	0.041294512	PASS
DHRS12	13	51767993	51804157	-0.384313056	PASS
DHRS13	17	28897781	28903071	0.041294512	PASS
DHRS2	14	23630115	23645639	0.053252507	PASS
DHRS3	1	12567910	12617731	0.048749694	PASS
DHRS4	14	23953586	23969279	0.053252507	PASS
DHRS4L2	14	23969874	24006408	0.053252507	PASS
DHRS7	14	60144120	60169856	0.053252507	PASS
DHRS7B	17	21123364	21193265	0.041294512	PASS
DHRS7C	17	9771434	9791297	0.06854245	PASS
DHRS9	2	169064789	169096167	0.033541467	PASS
DHRSX	23	2219516	2502805	-0.350651546	PASS
DHTKD1	10	12068972	12123225	0.075711544	PASS
DHX15	4	24517441	24584550	-0.358623662	PASS
DHX16	6	30653119	30673037	0.069043418	PASS
DHX29	5	55256245	55307722	-0.284712224	PASS
DHX30	3	47802909	47850195	-0.727808231	PASS
DHX32	10	125836337	125896436	0.073090211	PASS
DHX33	17	5440912	5469060	0.06854245	PASS
DHX34	19	47349281	47382704	0.002743503	PASS
DHX35	20	38962299	39039723	-0.172056771	suppress_segment_too_large
DHX36	3	154272546	154324497	0.02267528	PASS
DHX37	12	124946825	124989122	0.028722739	PASS
DHX38	16	72093562	72112912	-0.423061015	PASS
DHX40	17	59565525	59608345	0.051798665	PASS
DHX57	2	38797729	38875934	0.034263343	PASS
DHX58	17	42101404	42112733	0.041294512	PASS
DHX8	17	43483865	43610338	0.051798665	PASS
DHX9	1	182839369	182887751	0.088103245	PASS
DIABLO	12	122207662	122227534	0.028722739	PASS
DIAPH1	5	141515016	141619055	-0.393084436	PASS
DIAPH2	23	96684663	97604997	0.158844052	PASS
DIAPH3	13	59665583	60163987	-0.384313056	PASS
DICER1	14	95086228	95158010	-0.155273506	suppress_segment_too_large
DIDO1	20	62877738	62937952	-0.172056771	suppress_segment_too_large
DIMT1	5	62387254	62403939	0.07724032	PASS
DIO1	1	53891239	53911086	-0.736943052	PASS
DIO2	14	80197527	80387757	-0.155273506	suppress_segment_too_large
DIO3	14	101561351	101563452	-0.155273506	suppress_segment_too_large
DIP2A	21	46458899	46569852	0.209983559	PASS
DIP2B	12	50504985	50748667	-0.743220499	PASS
DIP2C	10	274190	689668	0.075711544	PASS
DIRAS1	19	2714567	2721418	-0.071973357	PASS
DIRAS2	9	90609832	90643105	0.073960965	PASS
DIRAS3	1	68045896	68051631	0.052232535	PASS
DIRC1	2	188733738	188790104	-0.030005025	PASS
DIRC2	3	122794795	122881139	0.06434429	PASS
DIS3	13	72752169	72782096	-0.384313056	PASS
DIS3L	15	66293217	66333898	-0.759380635	PASS
DIS3L2	2	231961245	232344350	0.011001208	PASS
DISC1	1	231626815	232041272	-0.204996646	suppress_segment_too_large
DISP1	1	222872271	223005995	-0.204996646	suppress_segment_too_large
DISP2	15	40358235	40378639	-0.423772929	PASS
DISP3	1	11479166	11537584	0.364796772	PASS

DIXDC1	11	111927144	112022584	-0.431650509	PASS
DKC1	23	154762742	154777689	0.158844052	PASS
DKK1	10	52314296	52318042	-0.389033546	PASS
DKK2	4	106921802	107283806	-0.677982248	PASS
DKK3	11	11963106	12009769	-0.39835217	PASS
DKK4	8	42374068	42377232	0.182873287	suppress_segment_too_large
DKKL1	19	49361783	49375116	0.002743503	PASS
DLAT	11	112024814	112064390	-0.431650509	PASS
DLC1	8	13083361	13604610	-0.311940419	PASS
DLD	7	107890970	107931730	-0.712149877	PASS
DLEC1	3	38039205	38124025	-0.727808231	PASS
DLEU7	13	50711008	50843939	-0.384313056	PASS
DLG1	3	197042560	197299300	0.02267528	PASS
DLG2	11	83455012	85627922	0.064535884	PASS
DLG3	23	70444861	70505490	0.158844052	PASS
DLG4	17	7187169	7219841	0.06854245	PASS
DLG5	10	77790791	77926526	0.032351597	PASS
DLGAP1	18	3496032	4455335	0.044197172	PASS
DLGAP2	8	737596	1708474	-0.311940419	PASS
DLGAP3	1	34865436	34929585	0.048749694	PASS
DLGAP4	20	36306336	36528637	-0.172056771	suppress_segment_too_large
DLGAP5	14	55148112	55191678	0.053252507	PASS
DLK1	14	100725705	100738224	-0.155273506	suppress_segment_too_large
DLK2	6	43450352	43456632	0.028532667	PASS
DLL1	6	170282206	170306565	0.033894706	PASS
DLL3	19	39498895	39508481	0.048917043	PASS
DLL4	15	40929333	40939073	-0.423772929	PASS
DLST	14	74881891	74903745	-0.155273506	suppress_segment_too_large
DLX1	2	172084740	172089677	0.033541467	PASS
DLX2	2	172099439	172102900	0.033541467	PASS
DLX3	17	49990005	49995224	0.051798665	PASS
DLX4	17	49968970	49974959	0.051798665	PASS
DLX5	7	97020392	97024950	-0.710463272	PASS
DLX6	7	97005548	97011039	-0.710463272	PASS
DMAC1	9	7796490	7888380	0.073960965	PASS
DMAC2	19	41431318	41440717	0.048917043	PASS
DMAP1	1	44213455	44220681	-0.736943052	PASS
DMBT1	10	122560665	122643736	0.073090211	PASS
DMBX1	1	46506996	46514226	-0.736943052	PASS
DMC1	22	38518949	38570286	-0.740245994	PASS
DMD	23	31097677	33339441	0.158844052	PASS
DMGDH	5	78997606	79236038	-0.393084436	PASS
DMKN	19	35497220	35513658	0.044838622	PASS
DMP1	4	87650307	87664361	-0.677982248	PASS
DMPK	19	45769717	45782552	0.002743503	PASS
DMRT1	9	841690	969090	0.073960965	PASS
DMRT2	9	1049858	1057552	0.073960965	PASS
DMRT3	9	976964	991731	0.073960965	PASS
DMRTA1	9	22446841	22455740	0.073960965	PASS
DMRTA2	1	50417550	50423500	-0.736943052	PASS
DMRTB1	1	53459399	53467488	-0.736943052	PASS
DMRTC1	23	72872025	72943814	0.158844052	PASS
DMRTC1E	23	72777009	72848802	0.158844052	PASS
DMRTC2	19	41844743	41852333	0.048917043	PASS

DMTF1	7	87152361	87196337		-0.221864321	PASS
DMTN	8	22048995	22082527		-0.311940419	PASS
DMWD	19	45782947	45792802		0.002743503	PASS
DMXL1	5	119037772	119249138		-0.393084436	PASS
DMXL2	15	51447711	51622833		-0.423772929	PASS
DNA2	10	68414064	68472121		0.032351597	PASS
DNAAF1	16	84145287	84178767		-0.423061015	PASS
DNAAF2	14	49625174	49635230		0.053252507	PASS
DNAAF3	19	55158661	55166722		0.142581352	PASS
DNAAF4	15	55410525	55508234		-0.759380635	PASS
DNAAF5	7	726701	786475		-0.72421718	PASS
DNAH1	3	52316319	52400491		-0.727808231	PASS
DNAH10	12	123762188	123936206		0.028722739	PASS
DNAH11	7	21543215	21901839		-0.72421718	PASS
DNAH12	3	57293699	57544344		-0.727808231	PASS
DNAH14	1	224896262	225399292		-0.204996646	suppress_segment_too_large
DNAH17	17	78423697	78577394		0.051798665	PASS
DNAH2	17	7717354	7833744		0.06854245	PASS
DNAH3	16	20933111	21159441		0.053595371	PASS
DNAH5	5	13690331	13944543		0.073527871	PASS
DNAH6	2	84516455	84819589		0.064122364	PASS
DNAH7	2	195737703	196068812		0.011001208	PASS
DNAH8	6	38715341	39030529		0.028532667	PASS
DNAH9	17	11598431	11969748		0.06854245	PASS
DNAI1	9	34457414	34520989		0.073960965	PASS
DNAI2	17	74274247	74314884		0.051798665	PASS
DNAJA1	9	33025211	33039907		0.073960965	PASS
DNAJA2	16	46955362	46973788		0.053595371	PASS
DNAJA3	16	4425805	4456775		0.053595371	PASS
DNAJA4	15	78264086	78282196		-0.759380635	PASS
DNAJB1	19	14514770	14529770		-0.069222905	PASS
DNAJB11	3	186567403	186585800		0.02267528	PASS
DNAJB12	10	72332830	72355230		0.032351597	PASS
DNAJB13	11	73950319	73970366		0.064535884	PASS
DNAJB14	4	99896248	99946726		-0.677982248	PASS
DNAJB2	2	219279267	219286900		0.011001208	PASS
DNAJB4	1	77979175	78017964		0.052232535	PASS
DNAJB5	9	34989641	34998900		0.073960965	PASS
DNAJB6	7	157335381	157417439		-0.77630473	PASS
DNAJB7	22	40859549	40862126		-0.740245994	PASS
DNAJB8	3	128462439	128467248		0.06434429	PASS
DNAJB9	7	108569568	108574850		-0.712149877	PASS
DNAJC1	10	21756537	22003769		0.075711544	PASS
DNAJC10	2	182716041	182794464		-0.120408457	PASS
DNAJC11	1	6634168	6701924		0.364796772	PASS
DNAJC12	10	67796665	67838166		0.032351597	PASS
DNAJC13	3	132417526	132539032		0.06434429	PASS
DNAJC14	12	55820960	55830824		-0.743220499	PASS
DNAJC15	13	43023203	43114224		-0.398748891	PASS
DNAJC16	1	15526813	15592379		0.048749694	PASS
DNAJC17	15	40765155	40807478		-0.423772929	PASS
DNAJC18	5	139408588	139444491		-0.393084436	PASS
DNAJC19	3	180983709	180989774		0.02267528	PASS
DNAJC2	7	103312474	103344873		-0.712149877	PASS

DNAJC21	5	34929559	34958964	0.073527871	PASS
DNAJC22	12	49346917	49357546	-0.743220499	PASS
DNAJC24	11	31369840	31431849	-0.359426047	PASS
DNAJC25	9	111631352	111654351	0.073960965	PASS
DNAJC25-	9	111631386	111670229	0.073960965	PASS
DNAJC27	2	24943636	24972094	0.034263343	PASS
DNAJC28	21	33485530	33491720	0.209983559	PASS
DNAJC3	13	95677139	95794989	-0.384313056	PASS
DNAJC30	7	73680969	73683453	-0.701511252	PASS
DNAJC4	11	64230278	64234286	0.064535884	PASS
DNAJC5	20	63895182	63936031	-0.172056771	suppress_segment_too_large
DNAJC5B	8	66021560	66100526	0.182873287	suppress_segment_too_large
DNAJC5G	2	27275421	27281499	0.034263343	PASS
DNAJC6	1	65248219	65415869	0.052232535	PASS
DNAJC7	17	41976433	42021376	0.041294512	PASS
DNAJC8	1	28199456	28233025	0.048749694	PASS
DNAJC9	10	73183362	73248862	0.032351597	PASS
DNAL1	14	73644875	73703732	0.053252507	PASS
DNAL4	22	38778508	38794198	-0.740245994	PASS
DNALI1	1	37556919	37566857	0.048749694	PASS
DNASE1	16	3611728	3680143	0.053595371	PASS
DNASE1L	23	154401238	154412112	0.158844052	PASS
DNASE1L	16	2235816	2238711	0.053595371	PASS
DNASE1L	3	58192257	58214697	-0.727808231	PASS
DNASE2	19	12875211	12881468	-0.069222905	PASS
DNASE2B	1	84398532	84415018	0.587598296	PASS
DND1	5	140670794	140673586	-0.393084436	PASS
DNER	2	229357629	229714558	0.011001208	PASS
DNHD1	11	6497260	6593758	-0.39835217	PASS
DNLZ	9	136359480	136363789	0.073960965	PASS
DNM1	9	128191655	128255248	0.073960965	PASS
DNM1L	12	32679200	32745650	0.49032213	suppress_segment_too_large
DNM2	19	10718079	10833488	-0.107594294	PASS
DNM3	1	171841498	172418466	0.088103245	PASS
DNMBP	10	99875577	100009919	-0.414937136	PASS
DNMT1	19	10133345	10231286	-0.107594294	PASS
DNMT3A	2	25227855	25342590	0.034263343	PASS
DNMT3B	20	32762385	32809356	-0.172056771	suppress_segment_too_large
DNMT3L	21	44246339	44262216	0.209983559	PASS
DNPEP	2	219373527	219400022	0.011001208	PASS
DNPH1	6	43225629	43229484	0.028532667	PASS
DNTT	10	96304396	96338564	-0.414937136	PASS
DNTTIP1	20	45791937	45811427	-0.172056771	suppress_segment_too_large
DNTTIP2	1	93866283	93879918	0.079255572	PASS
DOC2A	16	30005509	30023270	0.053595371	PASS
DOC2B	17	142789	181636	0.06854245	PASS
DOCK1	10	126905409	127452517	0.073090211	PASS
DOCK10	2	224765090	225042445	0.011001208	PASS
DOCK11	23	118495898	118686163	0.158844052	PASS
DOCK2	5	169637247	170083382	0.165341871	suppress_segment_too_large
DOCK3	3	50675241	51384198	-0.727808231	PASS
DOCK4	7	111726110	112206411	-0.712149877	PASS
DOCK5	8	25184723	25418082	-0.311940419	PASS
DOCK6	19	11199295	11262481	-0.107594294	PASS

DOCK7	1	62454298	62688368	0.052232535	PASS
DOCK8	9	214854	465259	0.073960965	PASS
DOCK9	13	98793429	99086625	-0.384313056	PASS
DOHH	19	3490822	3500940	-0.071973357	PASS
DOK1	2	74549026	74557554	-0.46122572	PASS
DOK2	8	21908873	21913860	-0.311940419	PASS
DOK3	5	177501907	177511274	0.165341871	suppress_segment_too_large
DOK4	16	57471922	57487327	-0.423061015	PASS
DOK5	20	54475597	54651171	-0.172056771	suppress_segment_too_large
DOK6	18	69401055	69849087	-0.330515409	PASS
DOK7	4	3463311	3501473	-0.358623662	PASS
DOLK	9	128945530	128947619	0.073960965	PASS
DOLPP1	9	129081100	129090438	0.073960965	PASS
DONSON	21	33559542	33588708	0.209983559	PASS
DOP1A	6	83067666	83171350	-0.250122709	PASS
DOP1B	21	36156782	36294274	0.209983559	PASS
DOT1L	19	2164149	2232578	-0.071973357	PASS
DPAGT1	11	119096503	119108331	-0.431650509	PASS
DPCD	10	101570560	101609668	-0.414937136	PASS
DPEP1	16	89613308	89638456	-0.423061015	PASS
DPEP2	16	67987394	68000586	-0.423061015	PASS
DPEP2NB	16	68013436	68015911	-0.423061015	PASS
DPEP3	16	67975663	67980829	-0.423061015	PASS
DPF1	19	38211006	38229714	0.048917043	PASS
DPF2	11	65333754	65353249	0.064535884	PASS
DPF3	14	72619296	72894116	0.053252507	PASS
DPH1	17	2030110	2043430	0.06854245	PASS
DPH2	1	43970000	43973369	-0.736943052	PASS
DPH3	3	16257978	16264972	0.06149513	PASS
DPH5	1	100989623	101026088	0.079255572	PASS
DPH6	15	35217345	35546193	-0.423772929	PASS
DPH7	9	137554904	137578935	0.073960965	PASS
DPM1	20	50934867	50958555	-0.172056771	suppress_segment_too_large
DPM2	9	127935099	127938484	0.073960965	PASS
DPM3	1	155139891	155140595	0.088103245	PASS
DPP10	2	114442299	115845752	0.040971718	PASS
DPP3	11	66480013	66509657	0.064535884	PASS
DPP4	2	161992241	162074542	0.319987345	PASS
DPP6	7	153887097	154894285	-0.77630473	PASS
DPP7	9	137110542	137115177	0.073960965	PASS
DPP8	15	65442463	65517704	-0.759380635	PASS
DPP9	19	4675224	4724673	-0.071973357	PASS
DPPA2	3	109293788	109316517	0.22470242	PASS
DPPA3	12	7711454	7717559	-0.015029783	PASS
DPPA4	3	109326141	109337572	0.22470242	PASS
DPPA5	6	73353062	73354295	-0.250122709	PASS
DPRX	19	53632056	53637009	0.142581352	PASS
DPT	1	168695459	168729264	0.088103245	PASS
DPY19L1	7	34928876	35038271	-0.72421718	PASS
DPY19L2	12	63558913	63668939	-0.743220499	PASS
DPY19L3	19	32405543	32485895	0.044838622	PASS
DPY19L4	8	94719703	94793836	0.355469066	suppress_segment_too_large
DPY30	2	31867809	32039812	0.034263343	PASS
DPYD	1	97077743	97995000	0.079255572	PASS

DPYS	8	104330324	104467053	0.70944438	suppress_segment_too_large
DPYSL2	8	26514022	26658178	-0.311940419	PASS
DPYSL3	5	147390811	147510056	-0.393084436	PASS
DPYSL4	10	132186900	132205776	0.073090211	PASS
DPYSL5	2	26847747	26950351	0.034263343	PASS
DQX1	2	74518131	74526336	-0.46122572	PASS
DR1	1	93345888	93369498	0.079255572	PASS
DRAM1	12	101877351	102012130	0.028722739	PASS
DRAM2	1	111117333	111140216	0.079255572	PASS
DRAP1	11	65919257	65921561	0.064535884	PASS
DRAXIN	1	11691729	11725857	0.364796772	PASS
DRC1	2	26401916	26456711	0.034263343	PASS
DRC3	17	17972813	18016889	0.06854245	PASS
DRC7	16	57694793	57731805	-0.423061015	PASS
DRD1	5	175440039	175444208	0.165341871	suppress_segment_too_large
DRD2	11	113409615	113475691	-0.431650509	PASS
DRD3	3	114128652	114199407	0.06434429	PASS
DRD4	11	637293	640706	-0.39835217	PASS
DRD5	4	9781680	9784009	-0.358623662	PASS
DRG1	22	31399523	31530634	-0.740245994	PASS
DRG2	17	18087886	18107971	0.06854245	PASS
DRGX	10	49364181	49396016	-0.389033546	PASS
DRICH1	22	23608452	23632321	-0.740245994	PASS
DROSHA	5	31400497	31532196	0.073527871	PASS
DRP2	23	101219769	101264497	0.158844052	PASS
DSC1	18	31129236	31162856	-0.330515409	PASS
DSC2	18	31058840	31102522	-0.330515409	PASS
DSC3	18	30990008	31042815	-0.330515409	PASS
DSCAM	21	40010999	40847139	0.209983559	PASS
DSCAML1	11	117427773	117817525	-0.431650509	PASS
DSCC1	8	119833976	119856010	0.493344802	suppress_segment_too_large
DSE	6	116254173	116444860	-0.355603029	PASS
DSEL	18	67506582	67516980	-0.330515409	PASS
DSG1	18	31318089	31357029	-0.330515409	PASS
DSG2	18	31498043	31549008	-0.330515409	PASS
DSG3	18	31447795	31478702	-0.330515409	PASS
DSG4	18	31376777	31414912	-0.330515409	PASS
DSN1	20	36751791	36773818	-0.172056771	suppress_segment_too_large
DSP	6	7541575	7586717	0.069043418	PASS
DSPP	4	87608553	87616910	-0.677982248	PASS
DST	6	56457987	56954628	0.239805577	suppress_segment_too_large
DSTN	20	17569863	17609919	-0.172056771	suppress_segment_too_large
DSTYK	1	205142505	205211566	-0.204996646	suppress_segment_too_large
DTD1	20	18587893	18766644	-0.172056771	suppress_segment_too_large
DTD2	14	31446036	31457510	0.053252507	PASS
DTHD1	4	36281622	36345756	-0.753159364	PASS
DTL	1	212035553	212107400	-0.204996646	suppress_segment_too_large
DTNA	18	34493290	34891844	-0.330515409	PASS
DTNB	2	25377198	25673647	0.034263343	PASS
DTNBP1	6	15522801	15663058	0.069043418	PASS
DTWD1	15	49620980	49656232	-0.423772929	PASS
DTWD2	5	118837322	118988545	-0.393084436	PASS
DTX1	12	113056709	113098028	0.028722739	PASS
DTX2	7	76461676	76505995	-0.701511252	PASS

DTX3	12	57604622	57609804	-0.743220499	PASS
DTX3L	3	122564238	122575203	0.06434429	PASS
DTX4	11	59171430	59208587	0.27006553	suppress_segment_too_large
DUOX1	15	45129933	45165576	-0.423772929	PASS
DUOX2	15	45092650	45114344	-0.423772929	PASS
DUOXA1	15	45117367	45129938	-0.423772929	PASS
DUOXA2	15	45114321	45118421	-0.423772929	PASS
DUPD1	10	75037836	75058514	0.032351597	PASS
DUS1L	17	82057506	82065887	0.051798665	PASS
DUS2	16	67987746	68079320	-0.423061015	PASS
DUS3L	19	5784832	5791238	-0.071973357	PASS
DUS4L	7	107563484	107578464	-0.712149877	PASS
DUSP1	5	172768090	172771195	0.165341871	suppress_segment_too_large
DUSP10	1	221701424	221742176	-0.204996646	suppress_segment_too_large
DUSP11	2	73762184	73780157	-0.46122572	PASS
DUSP12	1	161749758	161757238	0.088103245	PASS
DUSP13	10	75094432	75109221	0.032351597	PASS
DUSP14	17	37489831	37513501	0.041294512	PASS
DUSP15	20	31847637	31870747	-0.172056771	suppress_segment_too_large
DUSP16	12	12474210	12562383	0.49032213	suppress_segment_too_large
DUSP18	22	30652051	30667890	-0.740245994	PASS
DUSP19	2	183078559	183100005	-0.120408457	PASS
DUSP2	2	96143166	96145440	0.064122364	PASS
DUSP21	23	44844004	44844888	0.158844052	PASS
DUSP22	6	291630	351355	0.069043418	PASS
DUSP23	1	159780932	159782543	0.088103245	PASS
DUSP26	8	33591332	33600106	-0.311940419	PASS
DUSP27	1	167094045	167129165	0.088103245	PASS
DUSP28	2	240560054	240564014	0.011001208	PASS
DUSP3	17	43766121	43778988	0.051798665	PASS
DUSP4	8	29333064	29350668	-0.311940419	PASS
DUSP5	10	110497838	110511544	0.073090211	PASS
DUSP6	12	89347232	89353271	0.271127429	suppress_segment_too_large
DUSP7	3	52048919	52056550	-0.727808231	PASS
DUSP8	11	1554044	1571920	-0.39835217	PASS
DUSP9	23	153642492	153651326	0.158844052	PASS
DUT	15	48331011	48343373	-0.423772929	PASS
DUXA	19	57154021	57167443	0.142581352	PASS
DVL1	1	1335276	1349350	-0.358281697	PASS
DVL2	17	7225341	7234545	0.06854245	PASS
DVL3	3	184155311	184173614	0.02267528	PASS
DXO	6	31969810	31972292	0.028532667	PASS
DYDC1	10	80336105	80356755	0.032351597	PASS
DYDC2	10	80344745	80368073	0.032351597	PASS
DYM	18	49041474	49461347	-0.330515409	PASS
DYNAP	18	54587757	54599493	-0.330515409	PASS
DYNC1H1	14	101964510	102056443	-0.155273506	suppress_segment_too_large
DYNC1I1	7	95772506	96110322	-0.710463272	PASS
DYNC1I2	2	171687409	171748420	0.033541467	PASS
DYNC1LI1	3	32525971	32570874	0.06149513	PASS
DYNC1LI2	16	66720893	66751798	-0.423061015	PASS
DYNC2H1	11	103109410	103479863	0.32805343	suppress_segment_too_large
DYNC2LI1	2	43774039	43810010	0.034263343	PASS
DYNLL1	12	120469850	120498493	0.028722739	PASS

DYNLL2	17	58083415	58095536	0.051798665	PASS
DYNLRB1	20	34516409	34540958	-0.172056771	suppress_segment_too_large
DYNLRB2	16	80540734	80550760	-0.423061015	PASS
DYNLT1	6	158636474	158644739	0.033894706	PASS
DYNLT3	23	37836757	37847637	0.158844052	PASS
DYRK1A	21	37365573	37526358	0.209983559	PASS
DYRK1B	19	39825350	39834201	0.048917043	PASS
DYRK2	12	67648338	67665406	-0.743220499	PASS
DYRK3	1	206635536	206684419	-0.204996646	suppress_segment_too_large
DYRK4	12	4562204	4615302	-0.015029783	PASS
DYSF	2	71453722	71686768	-0.46122572	PASS
DYTN	2	206651621	206718396	0.011001208	PASS
DZANK1	20	18383367	18467281	-0.172056771	suppress_segment_too_large
DZIP1	13	95578202	95644703	-0.384313056	PASS
DZIP1L	3	138061990	138115818	0.06434429	PASS
DZIP3	3	108589682	108694846	0.22470242	PASS
E2F1	20	33675683	33686404	-0.172056771	suppress_segment_too_large
E2F2	1	23506430	23531220	0.048749694	PASS
E2F3	6	20401906	20493715	0.069043418	PASS
E2F4	16	67192169	67198918	-0.423061015	PASS
E2F5	8	85177225	85217158	0.617295349	suppress_segment_too_large
E2F6	2	11444375	11466177	0.034263343	PASS
E2F7	12	77021247	77065580	0.271127429	suppress_segment_too_large
E2F8	11	19224063	19241620	-0.39835217	PASS
E4F1	16	2223566	2235742	0.053595371	PASS
EAF1	3	15427355	15450635	0.06149513	PASS
EAF2	3	121835183	121886526	0.06434429	PASS
EAPP	14	34515929	34539711	0.053252507	PASS
EARS2	16	23522014	23557731	0.053595371	PASS
EBAG9	8	109539711	109565996	0.70944438	suppress_segment_too_large
EBF1	5	158695916	159099761	0.165341871	suppress_segment_too_large
EBF2	8	25841730	26045397	-0.311940419	PASS
EBF3	10	129835283	129963841	0.073090211	PASS
EBF4	20	2692878	2760108	-0.172056771	suppress_segment_too_large
EBI3	19	4229498	4237531	-0.071973357	PASS
EBLN1	10	22208814	22210021	0.075711544	PASS
EBLN2	3	73061659	73063337	-0.727808231	PASS
EBNA1BP	1	43164175	43270936	-0.736943052	PASS
EBP	23	48521158	48528716	0.158844052	PASS
EBPL	13	49660723	49691475	-0.384313056	PASS
ECD	10	73130155	73169055	0.032351597	PASS
ECE1	1	21217247	21345572	0.048749694	PASS
ECE2	3	184276011	184293031	0.02267528	PASS
ECEL1	2	232479827	232487828	0.011001208	PASS
ECH1	19	38815422	38832005	0.048917043	PASS
ECHDC1	6	127288710	127343609	-0.304168115	PASS
ECHDC2	1	52895910	52927212	-0.736943052	PASS
ECHDC3	10	11742366	11764070	0.075711544	PASS
ECHS1	10	133362480	133373689	0.073090211	PASS
ECI1	16	2239395	2252300	0.053595371	PASS
ECI2	6	4115689	4135597	0.069043418	PASS
ECM1	1	150508062	150513789	0.079255572	PASS
ECM2	9	92493554	92536655	0.073960965	PASS
ECPAS	9	111360692	111484745	0.073960965	PASS

ECSCR	5	139448556	139462743	-0.393084436	PASS
ECSIT	19	11505916	11529174	-0.107594294	PASS
ECT2	3	172750682	172821474	0.02267528	PASS
ECT2L	6	138795926	138904070	0.033894706	PASS
EDA	23	69616067	70039469	0.158844052	PASS
EDA2R	23	66595637	66639298	0.158844052	PASS
EDAR	2	108894471	108989372	0.040971718	PASS
EDARADI	1	236348257	236502915	-0.204996646	suppress_segment_too_large
EDC3	15	74630558	74696292	-0.759380635	PASS
EDC4	16	67873023	67884503	-0.423061015	PASS
EDDM3A	14	20745892	20748380	0.053252507	PASS
EDDM3B	14	20768427	20770948	0.053252507	PASS
EDEM1	3	5187646	5219957	0.06149513	PASS
EDEM2	20	35115357	35147364	-0.172056771	suppress_segment_too_large
EDEM3	1	184690231	184754913	0.088103245	PASS
EDF1	9	136862119	136866286	0.073960965	PASS
EDIL3	5	83940554	84384793	-0.393084436	PASS
EDN1	6	12290363	12297194	0.069043418	PASS
EDN2	1	41478775	41484673	-0.736943052	PASS
EDN3	20	59300427	59325992	-0.172056771	suppress_segment_too_large
EDNRA	4	147480917	147544954	0.142431244	suppress_segment_too_large
EDNRB	13	77895481	77975529	-0.384313056	PASS
EDRF1	10	125719515	125764143	0.073090211	PASS
EEA1	12	92770637	92929331	0.271127429	suppress_segment_too_large
EED	11	86244544	86278813	0.064535884	PASS
EEF1A1	6	73515750	73523797	-0.250122709	PASS
EEF1A2	20	63488012	63499322	-0.172056771	suppress_segment_too_large
EEF1AKM	13	20728731	20773958	-0.398748891	PASS
EEF1AKM	10	124748149	124791870	0.073090211	PASS
EEF1AKM	12	57771492	57782541	-0.743220499	PASS
EEF1AKM	3	184249650	184259585	0.02267528	PASS
EEF1AKM	3	184249695	184293031	0.02267528	PASS
EEF1B2	2	206159585	206162928	0.011001208	PASS
EEF1D	8	143579697	143599541	0.699132336	PASS
EEF1E1	6	8073360	8102578	0.069043418	PASS
EEF1E1-B	6	8015726	8102530	0.069043418	PASS
EEF1G	11	62559601	62574086	0.064535884	PASS
EEF2	19	3976056	3985469	-0.071973357	PASS
EEF2K	16	22206282	22288732	0.053595371	PASS
EEF2KMT	16	5084304	5097808	0.053595371	PASS
EEFSEC	3	128153454	128408646	0.06434429	PASS
EEPD1	7	36153149	36301543	-0.72421718	PASS
EFCAB1	8	48710789	48735311	0.182873287	suppress_segment_too_large
EFCAB10	7	105565120	105600875	-0.712149877	PASS
EFCAB11	14	89794669	89954777	-0.155273506	suppress_segment_too_large
EFCAB12	3	129401321	129428651	0.06434429	PASS
EFCAB13	17	47323290	47441312	0.051798665	PASS
EFCAB14	1	46675159	46719064	-0.736943052	PASS
EFCAB2	1	244969705	245127164	-0.145670469	suppress_likely_unfocal_large_gain
EFCAB3	17	62343941	62416479	0.051798665	PASS
EFCAB5	17	29929200	30108452	0.041294512	PASS
EFCAB6	22	43528744	43812337	-0.740245994	PASS
EFCAB7	1	63523372	63572693	0.052232535	PASS
EFCAB8	20	32858923	32961609	-0.172056771	suppress_segment_too_large

EFCAB9	5	172194172	172203452	0.165341871	suppress_segment_too_large
EFCC1	3	129001629	129040742	0.06434429	PASS
EFEMP1	2	55865967	55924139	-0.052193497	PASS
EFEMP2	11	65866441	65873592	0.064535884	PASS
EFHB	3	19879472	19947025	0.06149513	PASS
EFHC1	6	52362123	52529886	0.239805577	suppress_segment_too_large
EFHC2	23	44147882	44343672	0.158844052	PASS
EFHD1	2	232606057	232682781	0.011001208	PASS
EFHD2	1	15409895	15430343	0.048749694	PASS
EFL1	15	82130230	82262763	-0.759380635	PASS
EFNA1	1	155127460	155134857	0.088103245	PASS
EFNA2	19	1286154	1300237	-0.071973357	PASS
EFNA3	1	155078872	155087538	0.088103245	PASS
EFNA4	1	155063731	155069553	0.088103245	PASS
EFNA5	5	107376889	107670895	-0.393084436	PASS
EFNB1	23	68828997	68842147	0.158844052	PASS
EFNB2	13	106489731	106535662	-0.426822582	PASS
EFNB3	17	7705202	7711378	0.06854245	PASS
EFR3A	8	131904088	132013642	0.493344802	suppress_segment_too_large
EFR3B	2	25042130	25159137	0.034263343	PASS
EFS	14	23356402	23365752	0.053252507	PASS
EFTUD2	17	44849943	44899662	0.051798665	PASS
EGF	4	109912884	110012266	-0.677982248	PASS
EGFL6	23	13569605	13633575	0.158844052	PASS
EGFL7	9	136658856	136672678	0.073960965	PASS
EGFL8	6	32164583	32168281	0.028532667	PASS
EGFLAM	5	38258409	38465021	0.073527871	PASS
EGFR	7	55019021	55211628	-0.701511252	PASS
EGLN1	1	231363751	231425044	-0.204996646	suppress_segment_too_large
EGLN2	19	40798996	40808433	0.048917043	PASS
EGLN3	14	33924231	34462774	0.053252507	PASS
EGR1	5	138465490	138469315	-0.393084436	PASS
EGR2	10	62811996	62919900	0.032351597	PASS
EGR3	8	22687659	22693302	-0.311940419	PASS
EGR4	2	73290929	73293705	-0.46122572	PASS
EHBP1	2	62673851	63046487	-0.46122572	PASS
EHBP1L1	11	65576038	65592650	0.064535884	PASS
EHD1	11	64851642	64888296	0.064535884	PASS
EHD2	19	47713343	47743134	0.002743503	PASS
EHD3	2	31234337	31269447	0.034263343	PASS
EHD4	15	41895939	41972578	-0.423772929	PASS
EHF	11	34621093	34661057	-0.359426047	PASS
EHHADH	3	185190624	185281990	0.02267528	PASS
EHMT1	9	137618963	137870016	0.073960965	PASS
EHMT2	6	31879759	31897687	0.028532667	PASS
EI24	11	125569216	125584687	-0.431650509	PASS
EID1	15	48877886	48880183	-0.423772929	PASS
EID2	19	39538705	39540333	0.048917043	PASS
EID2B	19	39530990	39532854	0.048917043	PASS
EID3	12	104303739	104305205	0.028722739	PASS
EIF1	17	41688885	41692668	0.041294512	PASS
EIF1AD	11	65996545	66002176	0.064535884	PASS
EIF1AX	23	20124518	20141844	0.158844052	PASS
EIF1B	3	40309684	40312424	-0.727808231	PASS

EIF2A	3	150546678	150584242	0.02267528	PASS
EIF2AK1	7	6022244	6059230	-0.72421718	PASS
EIF2AK2	2	37099210	37157065	0.034263343	PASS
EIF2AK3	2	88556741	88627576	0.064122364	PASS
EIF2AK4	15	39934146	40035591	-0.423772929	PASS
EIF2B1	12	123620406	123633766	0.028722739	PASS
EIF2B2	14	75002911	75012366	-0.155273506	suppress_segment_too_large
EIF2B3	1	44850522	44986722	-0.736943052	PASS
EIF2B4	2	27364352	27370486	0.034263343	PASS
EIF2B5	3	184135038	184146112	0.02267528	PASS
EIF2D	1	206571292	206612463	-0.204996646	suppress_segment_too_large
EIF2S1	14	67359997	67386516	0.053252507	PASS
EIF2S2	20	34088298	34112332	-0.172056771	suppress_segment_too_large
EIF2S3	23	24054716	24077971	0.158844052	PASS
EIF2S3B	12	10505602	10523135	0.505438545	PASS
EIF3A	10	119033670	119080823	0.073090211	PASS
EIF3B	7	2354086	2380745	-0.72421718	PASS
EIF3C	16	28688558	28735730	0.053595371	PASS
EIF3D	22	36510850	36529436	-0.740245994	PASS
EIF3E	8	108201216	108435333	0.70944438	suppress_segment_too_large
EIF3F	11	7970251	8001862	-0.39835217	PASS
EIF3G	19	10115017	10119918	-0.107594294	PASS
EIF3H	8	116642130	116766925	0.493344802	suppress_segment_too_large
EIF3I	1	32221928	32231604	0.048749694	PASS
EIF3J	15	44537057	44563029	-0.423772929	PASS
EIF3K	19	38619082	38636955	0.048917043	PASS
EIF3L	22	37848868	37889407	-0.740245994	PASS
EIF3M	11	32583798	32606262	-0.359426047	PASS
EIF4A1	17	7572706	7579005	0.06854245	PASS
EIF4A2	3	186783205	186789900	0.02267528	PASS
EIF4A3	17	80135214	80147183	0.051798665	PASS
EIF4B	12	53006158	53042209	-0.743220499	PASS
EIF4E	4	98871684	98930637	-0.677982248	PASS
EIF4E1B	5	176630618	176646641	0.165341871	suppress_segment_too_large
EIF4E2	2	232550052	232583644	0.011001208	PASS
EIF4E3	3	71675416	71754773	-0.727808231	PASS
EIF4EBP1	8	38030341	38060365	0.182873287	suppress_segment_too_large
EIF4EBP2	10	70404379	70428618	0.032351597	PASS
EIF4EBP3	5	140547666	140549578	-0.393084436	PASS
EIF4ENIF1	22	31436977	31496108	-0.740245994	PASS
EIF4G1	3	184314495	184335358	0.02267528	PASS
EIF4G2	11	10797050	10809110	-0.39835217	PASS
EIF4G3	1	20806292	21176888	0.048749694	PASS
EIF4H	7	74174245	74197101	-0.701511252	PASS
EIF5	14	103333544	103345025	-0.155273506	suppress_segment_too_large
EIF5A	17	7306999	7312463	0.06854245	PASS
EIF5A2	3	170888415	170908693	0.02267528	PASS
EIF5AL1	10	79512601	79516440	0.032351597	PASS
EIF5B	2	99337353	99401326	0.064122364	PASS
EIF6	20	35278911	35284985	-0.172056771	suppress_segment_too_large
EIPR1	2	3188925	3377882	0.034263343	PASS
ELAC1	18	50967991	50988121	-0.330515409	PASS
ELAC2	17	12992391	13018187	0.06854245	PASS
ELANE	19	851014	856247	-0.071973357	PASS

ELAVL1	19	7958579	8005659		-0.071973357	PASS
ELAVL2	9	23690104	23826337		0.073960965	PASS
ELAVL3	19	11451326	11481046		-0.107594294	PASS
ELAVL4	1	50048014	50203786		-0.736943052	PASS
ELF1	13	40932028	41061440		-0.398748891	PASS
ELF2	4	139028112	139177218		0.142431244	suppress_segment_too_large
ELF3	1	202007945	202017188		-0.204996646	suppress_segment_too_large
ELF4	23	130064874	130110716		0.158844052	PASS
ELF5	11	34478793	34513805		-0.359426047	PASS
ELFN1	7	1688119	1747954		-0.72421718	PASS
ELFN2	22	37367960	37427470		-0.740245994	PASS
ELK1	23	47635521	47650604		0.158844052	PASS
ELK3	12	96194382	96269835		0.028722739	PASS
ELK4	1	205597556	205631962		-0.204996646	suppress_segment_too_large
ELL	19	18442663	18522127		-0.069222905	PASS
ELL2	5	95885098	95962071		-0.393084436	PASS
ELL3	15	43772600	43777543		-0.423772929	PASS
ELMO1	7	36854361	37449249		-0.72421718	PASS
ELMO2	20	46366049	46432985		-0.172056771	suppress_segment_too_large
ELMO3	16	67199111	67204029		-0.423061015	PASS
ELMOD1	11	107591091	107666779		-0.431650509	PASS
ELMOD2	4	140524158	140553770		0.142431244	suppress_segment_too_large
ELMOD3	2	85354394	85391752		0.064122364	PASS
ELMSAN1	14	73715122	73790285		0.053252507	PASS
ELN	7	74027789	74069907		-0.701511252	PASS
ELOA	1	23743155	23762059		0.048749694	PASS
ELOA2	18	47032572	47035621		-0.330515409	PASS
ELOA3C	18	46968695	46969912		-0.330515409	PASS
ELOA3D	18	46962768	46964408		-0.330515409	PASS
ELOB	16	2771414	2777297		0.053595371	PASS
ELOC	8	73939169	73972287		0.182873287	suppress_segment_too_large
ELOF1	19	11551147	11559236		-0.107594294	PASS
ELOVL1	1	43363397	43368074		-0.736943052	PASS
ELOVL2	6	10980759	11044314		0.069043418	PASS
ELOVL3	10	102226328	102229589		-0.414937136	PASS
ELOVL4	6	79914812	79947580		-0.250122709	PASS
ELOVL5	6	53267398	53349179		0.239805577	suppress_segment_too_large
ELOVL6	4	110045846	110199199		-0.677982248	PASS
ELOVL7	5	60751791	60844389		0.07724032	PASS
ELP1	9	108867517	108934116		0.073960965	PASS
ELP2	18	36129444	36180556		-0.330515409	PASS
ELP3	8	28089673	28191156		-0.311940419	PASS
ELP4	11	31509700	31790328		-0.359426047	PASS
ELP5	17	7251416	7259940		0.06854245	PASS
ELP6	3	47495640	47513761		-0.727808231	PASS
ELSPBP1	19	47994651	48025153		0.002743503	PASS
EMB	5	50396192	50443248		-0.284712224	PASS
EMC1	1	19215664	19251552		0.048749694	PASS
EMC10	19	50476400	50490870		0.002743503	PASS
EMC2	8	108443601	108486918		0.70944438	suppress_segment_too_large
EMC3	3	9962537	10011116		0.06149513	PASS
EMC4	15	34224999	34230156		-0.423772929	PASS
EMC6	17	3668815	3669668		0.06854245	PASS
EMC7	15	34084017	34101948		-0.423772929	PASS

EMC8	16	85771758	85799608	-0.423061015	PASS
EMC9	14	24138959	24141588	0.053252507	PASS
EMCN	4	100395341	100880126	-0.677982248	PASS
EMD	23	154379197	154381523	0.158844052	PASS
EME1	17	50373220	50381483	0.051798665	PASS
EME2	16	1773207	1781708	0.053595371	PASS
EMG1	12	6970893	6997428	-0.015029783	PASS
EMID1	22	29205851	29259597	-0.740245994	PASS
EMILIN1	2	27078567	27086408	0.034263343	PASS
EMILIN2	18	2847030	2915993	0.044197172	PASS
EMILIN3	20	41359966	41366827	-0.172056771	suppress_segment_too_large
EML1	14	99737693	99942060	-0.155273506	suppress_segment_too_large
EML2	19	45606994	45645629	0.002743503	PASS
EML3	11	62602218	62612765	0.064535884	PASS
EML4	2	42169350	42332548	0.034263343	PASS
EML5	14	88612431	88792752	-0.155273506	suppress_segment_too_large
EML6	2	54723499	54972025	0.034263343	PASS
EMP1	12	13196716	13219939	0.49032213	suppress_segment_too_large
EMP2	16	10528422	10580698	0.053595371	PASS
EMP3	19	48321509	48330553	0.002743503	PASS
EMSY	11	76444923	76553025	0.064535884	PASS
EMX1	2	72916260	72936071	-0.46122572	PASS
EMX2	10	117542444	117549546	0.073090211	PASS
EN1	2	118842171	118847678	-0.359440172	PASS
EN2	7	155458129	155464831	-0.77630473	PASS
ENAH	1	225486835	225653142	-0.204996646	suppress_segment_too_large
ENAM	4	70628744	70686816	0.115781418	suppress_segment_too_large
ENC1	5	74627406	74641424	-0.393084436	PASS
ENDOD1	11	95089810	95132645	0.32805343	suppress_segment_too_large
ENDOG	9	128818474	128822677	0.073960965	PASS
ENDOU	12	47709734	47725567	-0.743220499	PASS
ENDOV	17	80415165	80438086	0.051798665	PASS
ENG	9	127815012	127854756	0.073960965	PASS
ENGASE	17	79074939	79088599	0.051798665	PASS
ENHO	9	34521040	34523041	0.073960965	PASS
ENKD1	16	67662945	67667265	-0.423061015	PASS
ENKUR	10	24981979	25062279	0.075711544	PASS
ENO1	1	8861000	8879250	0.364796772	PASS
ENO2	12	6913745	6923698	-0.015029783	PASS
ENO3	17	4948092	4957131	0.06854245	PASS
ENO4	10	116849512	116911788	0.073090211	PASS
ENOPH1	4	82430562	82461091	-0.677982248	PASS
ENOSF1	18	670324	712676	0.044197172	PASS
ENOX1	13	43213518	43786908	-0.398748891	PASS
ENOX2	23	130623369	130903317	0.158844052	PASS
ENPEP	4	110365733	110565285	-0.677982248	PASS
ENPP1	6	131808016	131895155	-0.304168115	PASS
ENPP2	8	119557086	119673453	0.493344802	suppress_segment_too_large
ENPP3	6	131628442	131747418	-0.304168115	PASS
ENPP4	6	46129993	46146699	0.239805577	suppress_segment_too_large
ENPP5	6	46159187	46170971	0.239805577	suppress_segment_too_large
ENPP6	4	184088706	184221230	-0.453876349	PASS
ENPP7	17	79730919	79742222	0.051798665	PASS
ENSA	1	150600851	150629612	0.079255572	PASS

ENTHD1	22	39743044	39893864	-0.740245994	PASS
ENTPD1	10	95711779	95869695	-0.414937136	PASS
ENTPD2	9	137048098	137054045	0.073960965	PASS
ENTPD3	3	40387156	40428744	-0.727808231	PASS
ENTPD4	8	23385783	23457695	-0.311940419	PASS
ENTPD5	14	73958010	74019399	0.053252507	PASS
ENTPD6	20	25195693	25226729	-0.172056771	suppress_segment_too_large
ENTPD7	10	99659506	99706240	-0.414937136	PASS
ENTPD8	9	137434364	137441816	0.073960965	PASS
ENTR1	9	136401922	136410609	0.073960965	PASS
ENY2	8	109334324	109345953	0.70944438	suppress_segment_too_large
EOGT	3	68975214	69013961	-0.727808231	PASS
EOMES	3	27715949	27722711	0.06149513	PASS
EP300	22	41091786	41180079	-0.740245994	PASS
EP400	12	131949920	132081102	0.028722739	PASS
EPAS1	2	46293667	46386703	0.034263343	PASS
EPB41	1	28887091	29120046	0.048749694	PASS
EPB41L1	20	36091504	36232799	-0.172056771	suppress_segment_too_large
EPB41L2	6	130839347	131063322	-0.304168115	PASS
EPB41L3	18	5392381	5630700	0.044197172	PASS
EPB41L4A	5	112142441	112419316	-0.393084436	PASS
EPB41L4B	9	109171975	109320964	0.073960965	PASS
EPB41L5	2	120013005	120179119	-0.359440172	PASS
EPB42	15	43197227	43221125	-0.423772929	PASS
EPC1	10	32267751	32378798	0.075711544	PASS
EPC2	2	148644440	148787568	-0.30549516	PASS
EPCAM	2	47345158	47387601	0.034263343	PASS
EPDR1	7	37683843	37951941	-0.72421718	PASS
EPG5	18	45847609	45967339	-0.330515409	PASS
EPGN	4	74308473	74315307	0.115781418	suppress_segment_too_large
EPHA1	7	143390289	143408892	-0.712149877	PASS
EPHA10	1	37713880	37765133	0.048749694	PASS
EPHA2	1	16124337	16156087	0.048749694	PASS
EPHA3	3	89107524	89482134	0.22470242	PASS
EPHA4	2	221418027	221574202	0.011001208	PASS
EPHA5	4	65319563	65670495	0.115781418	suppress_segment_too_large
EPHA6	3	96814581	97752460	0.22470242	PASS
EPHA7	6	93240020	93419547	-0.355603029	PASS
EPHA8	1	22563564	22603594	0.048749694	PASS
EPHB1	3	134597801	135260467	0.06434429	PASS
EPHB2	1	22710839	22921500	0.048749694	PASS
EPHB3	3	184561784	184582409	0.02267528	PASS
EPHB4	7	100802565	100827521	-0.710463272	PASS
EPHB6	7	142855061	142871094	-0.712149877	PASS
EPHX1	1	225810092	225845563	-0.204996646	suppress_segment_too_large
EPHX2	8	27490779	27545564	-0.311940419	PASS
EPHX3	19	15226919	15233435	-0.069222905	PASS
EPHX4	1	92029982	92063536	0.079255572	PASS
EPM2A	6	145382535	145736023	0.033894706	PASS
EPM2AIP1	3	36985043	36993168	-0.727808231	PASS
EPN1	19	55675226	55709858	0.142581352	PASS
EPN2	17	19215615	19336715	0.06854245	PASS
EPN3	17	50532543	50543750	0.051798665	PASS
EPO	7	100720800	100723700	-0.710463272	PASS

EPOP	17	38671703	38675421	0.041294512	PASS
EPOR	19	11377205	11384342	-0.107594294	PASS
EPPIN	20	45540626	45547752	-0.172056771	suppress_segment_too_large
EPPIN-WF	20	45536986	45547426	-0.172056771	suppress_segment_too_large
EPPK1	8	143857324	143878464	0.699132336	PASS
EPRS	1	219968601	220046658	-0.204996646	suppress_segment_too_large
EPS15	1	51354263	51519328	-0.736943052	PASS
EPS15L1	19	16355239	16472085	-0.069222905	PASS
EPS8	12	15620158	15882329	0.49032213	suppress_segment_too_large
EPS8L1	19	55072020	55087923	0.142581352	PASS
EPS8L2	11	694438	727727	-0.39835217	PASS
EPS8L3	1	109750080	109764027	0.079255572	PASS
EPSTI1	13	42886388	42992271	-0.398748891	PASS
EPX	17	58192737	58205174	0.051798665	PASS
EPYC	12	90963679	91005026	0.271127429	suppress_segment_too_large
EQTN	9	27284659	27297139	0.073960965	PASS
ERAL1	17	28854938	28861067	0.041294512	PASS
ERAP1	5	96760810	96808100	-0.393084436	PASS
ERAP2	5	96875939	96919716	-0.393084436	PASS
ERAS	23	48826513	48830138	0.158844052	PASS
ERBB2	17	39687914	39730426	0.041294512	PASS
ERBB3	12	56076799	56103505	-0.743220499	PASS
ERBB4	2	211375717	212538841	0.011001208	PASS
ERBIN	5	65926475	66082549	-0.393084436	PASS
ERC1	12	990509	1495933	-0.015029783	PASS
ERC2	3	55508308	56468363	-0.727808231	PASS
ERCC1	19	45407333	45478828	0.002743503	PASS
ERCC2	19	45349837	45370918	0.002743503	PASS
ERCC3	2	127257290	127294176	-0.359440172	PASS
ERCC4	16	13920157	13952345	0.053595371	PASS
ERCC5	13	102807146	102876001	-0.426822582	PASS
ERCC6	10	49455368	49539538	-0.389033546	PASS
ERCC6L	23	72204657	72239047	0.158844052	PASS
ERCC6L2	9	95875701	96014571	0.073960965	PASS
ERCC8	5	60873831	60945073	0.07724032	PASS
EREG	4	74365143	74388751	0.115781418	suppress_segment_too_large
ERF	19	42247572	42255157	0.048917043	PASS
ERFE	2	238158982	238168900	0.011001208	PASS
ERG	21	38380027	38661780	0.209983559	PASS
ERG28	14	75649791	75661189	-0.155273506	suppress_segment_too_large
ERGIC1	5	172834275	172952685	0.165341871	suppress_segment_too_large
ERGIC2	12	29337352	29381189	0.49032213	suppress_segment_too_large
ERGIC3	20	35542021	35557634	-0.172056771	suppress_segment_too_large
ERH	14	69380123	69398627	0.053252507	PASS
ERI1	8	9002147	9116746	-0.311940419	PASS
ERI2	16	20780193	20900349	0.053595371	PASS
ERI3	1	44221070	44355260	-0.736943052	PASS
ERICH1	8	614746	738106	-0.311940419	PASS
ERICH2	2	170783786	170798971	0.033541467	PASS
ERICH3	1	74568111	74673738	0.052232535	PASS
ERICH4	19	41443158	41444765	0.048917043	PASS
ERICH5	8	98064311	98093610	0.355469066	suppress_segment_too_large
ERICH6	3	150659885	150703971	0.02267528	PASS
ERICH6B	13	45534522	45615739	-0.398748891	PASS

ERLEC1	2	53787044	53818819	0.034263343	PASS
ERLIN1	10	100150094	100188334	-0.414937136	PASS
ERLIN2	8	37736601	37759101	0.182873287	suppress_segment_too_large
ERMAP	1	42817088	42844993	-0.736943052	PASS
ERMARD	6	169751622	169781584	0.033894706	PASS
ERMN	2	157318625	157327713	-0.30549516	PASS
ERMP1	9	5765076	5833117	0.073960965	PASS
ERN1	17	64039142	64130819	0.051798665	PASS
ERN2	16	23690326	23713500	0.053595371	PASS
ERO1A	14	52639916	52695900	0.053252507	PASS
ERO1B	1	236215555	236281985	-0.204996646	suppress_segment_too_large
ERP27	12	14914035	14939082	0.49032213	suppress_segment_too_large
ERP29	12	112013316	112023451	0.028722739	PASS
ERP44	9	99979179	100099040	0.073960965	PASS
ERRFI1	1	8004404	8026308	0.364796772	PASS
ERV3-1	7	64990809	65006743	-0.701511252	PASS
ERVFRD-1	6	11102489	11111732	0.069043418	PASS
ERVK3-1	19	58305319	58315663	0.142581352	PASS
ERVMER3	4	52722618	52751640	0.115781418	suppress_segment_too_large
ERVV-1	19	53013921	53016122	0.416834572	PASS
ERVV-2	19	53044738	53051076	0.416834572	PASS
ERVW-1	7	92468380	92477986	-0.710463272	PASS
ESAM	11	124752583	124762290	-0.431650509	PASS
ESCO1	18	21529281	21600884	-0.330515409	PASS
ESCO2	8	27771949	27812640	-0.311940419	PASS
ESD	13	46771256	46797232	-0.398748891	PASS
ESF1	20	13714322	13784886	-0.172056771	suppress_segment_too_large
ESM1	5	54977864	55022671	-0.284712224	PASS
ESPL1	12	53268299	53293643	-0.743220499	PASS
ESPN	1	6424776	6461367	0.364796772	PASS
ESPNL	2	238100157	238133287	0.011001208	PASS
ESR1	6	151656691	152129619	0.033894706	PASS
ESR2	14	64084232	64338112	0.053252507	PASS
ESRP1	8	94641074	94707466	0.355469066	suppress_segment_too_large
ESRP2	16	68229111	68238102	-0.423061015	PASS
ESRRA	11	64305572	64316743	0.064535884	PASS
ESRRB	14	76310712	76501837	-0.155273506	suppress_segment_too_large
ESRRG	1	216503246	217137755	-0.204996646	suppress_segment_too_large
ESS2	22	19130279	19144684	-0.740245994	PASS
ESX1	23	104250038	104254933	0.158844052	PASS
ESYT1	12	56118250	56144671	-0.743220499	PASS
ESYT2	7	158730995	158830253	-0.77630473	PASS
ESYT3	3	138434586	138481686	0.06434429	PASS
ETAA1	2	67397319	67412089	-0.46122572	PASS
ETF1	5	138506095	138543300	-0.393084436	PASS
ETFA	15	76215355	76311472	-0.759380635	PASS
ETFB	19	51345169	51366418	0.002743503	PASS
ETFBKMT	12	31647160	31673114	0.49032213	suppress_segment_too_large
ETFDH	4	158672125	158709623	0.142431244	suppress_segment_too_large
ETFRF1	12	25195216	25209645	0.49032213	suppress_segment_too_large
ETHE1	19	43506719	43527244	0.048917043	PASS
ETNK1	12	22625075	22690665	0.49032213	suppress_segment_too_large
ETNK2	1	204131062	204152003	-0.204996646	suppress_segment_too_large
ETNPPL	4	108742040	108763054	-0.677982248	PASS

ETS1	11	128458761	128587558	-0.431650509	PASS
ETS2	21	38805307	38824955	0.209983559	PASS
ETV1	7	13891228	13991425	-0.72421718	PASS
ETV2	19	35641745	35644871	0.044838622	PASS
ETV3	1	157121191	157138474	0.088103245	PASS
ETV3L	1	157092044	157099808	0.088103245	PASS
ETV4	17	43527844	43579620	0.051798665	PASS
ETV5	3	186046308	186110318	0.02267528	PASS
ETV6	12	11649854	11895402	0.49032213	suppress_segment_too_large
ETV7	6	36354091	36387800	0.028532667	PASS
EVA1A	2	75469302	75569722	-0.46122572	PASS
EVA1B	1	36322031	36324154	0.048749694	PASS
EVA1C	21	32412006	32515397	0.209983559	PASS
EVC	4	5711197	5814305	-0.358623662	PASS
EVC2	4	5542772	5709548	-0.358623662	PASS
EVI2A	17	31317560	31321884	0.041294512	PASS
EVI2B	17	31303766	31314112	0.041294512	PASS
EVI5	1	92508696	92792404	0.079255572	PASS
EVI5L	19	7830233	7864976	-0.071973357	PASS
EVL	14	99971449	100144236	-0.155273506	suppress_segment_too_large
EVPL	17	76004502	76027452	0.051798665	PASS
EVPLL	17	18377662	18389647	0.06854245	PASS
EVX1	7	27242700	27250493	-0.72421718	PASS
EVX2	2	176077472	176083913	0.033541467	PASS
EWSR1	22	29268009	29300525	-0.740245994	PASS
EXD1	15	41182725	41230743	-0.423772929	PASS
EXD2	14	69191511	69244020	0.053252507	PASS
EXD3	9	137306896	137423262	0.073960965	PASS
EXO1	1	241847967	241895148	-0.287484925	PASS
EXO5	1	40508741	40516556	0.048749694	PASS
EXOC1	4	55853616	55905034	0.115781418	suppress_segment_too_large
EXOC2	6	485133	693111	0.069043418	PASS
EXOC3	5	443158	471937	0.073527871	PASS
EXOC3L1	16	67184366	67190204	-0.423061015	PASS
EXOC3L2	19	45212621	45245431	0.002743503	PASS
EXOC3L4	14	103100144	103110559	-0.155273506	suppress_segment_too_large
EXOC4	7	133253073	134066589	-0.712149877	PASS
EXOC5	14	57200507	57269008	0.053252507	PASS
EXOC6	10	92834713	93059493	-0.414937136	PASS
EXOC6B	2	72175984	72826041	-0.46122572	PASS
EXOC7	17	76081017	76121576	0.051798665	PASS
EXOC8	1	231332753	231337852	-0.204996646	suppress_segment_too_large
EXOG	3	38496127	38542161	-0.727808231	PASS
EXOSC1	10	97436142	97446017	-0.414937136	PASS
EXOSC10	1	11066618	11099881	0.364796772	PASS
EXOSC2	9	130693721	130704894	0.073960965	PASS
EXOSC3	9	37766978	37801437	0.073960965	PASS
EXOSC4	8	144078626	144080647	0.699132336	PASS
EXOSC5	19	41386374	41397479	0.048917043	PASS
EXOSC7	3	44975241	45036066	-0.727808231	PASS
EXOSC8	13	36998816	37009613	-0.398748891	PASS
EXOSC9	4	121801317	121817021	0.142431244	suppress_segment_too_large
EXPH5	11	108505431	108593738	-0.431650509	PASS
EXT1	8	117794490	118111853	0.493344802	suppress_segment_too_large

EXT2	11	44095549	44245429	0.059795455	PASS
EXTL1	1	26019884	26036464	0.048749694	PASS
EXTL2	1	100872372	100895998	0.079255572	PASS
EXTL3	8	28600469	28755599	-0.311940419	PASS
EYA1	8	71197433	71592025	0.182873287	suppress_segment_too_large
EYA2	20	46894624	47188844	-0.172056771	suppress_segment_too_large
EYA3	1	27970344	28088696	0.048749694	PASS
EYA4	6	133240598	133532120	0.033894706	PASS
EYS	6	63719980	65707225	-0.021435747	PASS
EZH1	17	42700275	42745049	0.041294512	PASS
EZH2	7	148807383	148884321	-0.712149877	PASS
EZR	6	158765741	158819412	0.033894706	PASS
F10	13	113122814	113149529	-0.426822582	PASS
F11	4	186265945	186288806	0.275137421	PASS
F11R	1	160995211	161021348	0.088103245	PASS
F12	5	177402140	177409576	0.165341871	suppress_segment_too_large
F13A1	6	6144085	6321013	0.069043418	PASS
F13B	1	197038741	197067267	0.088103245	PASS
F2	11	46719180	46739506	0.059795455	PASS
F2R	5	76716043	76735781	-0.393084436	PASS
F2RL1	5	76818933	76835315	-0.393084436	PASS
F2RL2	5	76615482	76623434	-0.393084436	PASS
F2RL3	19	16888860	16892606	-0.069222905	PASS
F3	1	94529225	94541800	0.079255572	PASS
F5	1	169514166	169586588	0.088103245	PASS
F7	13	113105788	113120681	-0.426822582	PASS
F8	23	154835788	155026940	0.158844052	PASS
F9	23	139530758	139563458	0.158844052	PASS
FA2H	16	74712955	74774831	-0.423061015	PASS
FAAH	1	46394265	46413848	-0.736943052	PASS
FAAH2	23	57286706	57489196	0.158844052	PASS
FAAP100	17	81539885	81553961	0.051798665	PASS
FAAP20	1	2184461	2212720	-0.358281697	PASS
FAAP24	19	32972209	32978222	0.044838622	PASS
FABP1	2	88122982	88128116	0.064122364	PASS
FABP12	8	81524981	81531378	0.617295349	suppress_segment_too_large
FABP2	4	119317250	119322390	-0.754119944	PASS
FABP3	1	31365625	31376850	0.048749694	PASS
FABP4	8	81478419	81483263	0.617295349	suppress_segment_too_large
FABP5	8	81280363	81284777	0.617295349	suppress_segment_too_large
FABP6	5	160187367	160238735	0.165341871	suppress_segment_too_large
FABP7	6	122779475	122784074	-1.845658544	PASS
FABP9	8	81458341	81461579	0.617295349	suppress_segment_too_large
FADD	11	70203163	70207390	0.064535884	PASS
FADS1	11	61799625	61829318	0.064535884	PASS
FADS2	11	61792980	61867354	0.064535884	PASS
FADS3	11	61873519	61892051	0.064535884	PASS
FADS6	17	74877299	74893781	0.051798665	PASS
FAF1	1	50437028	50960263	-0.736943052	PASS
FAF2	5	176447628	176510074	0.165341871	suppress_segment_too_large
FAH	15	80152490	80186946	-0.759380635	PASS
FAHD1	16	1826941	1840207	0.053595371	PASS
FAHD2A	2	95402721	95416616	0.064122364	PASS
FAHD2B	2	97083583	97094882	0.064122364	PASS

FAIM	3	138608606	138633376	0.06434429	PASS
FAIM2	12	49866896	49904217	-0.743220499	PASS
FAM102A	9	127940579	127980513	0.073960965	PASS
FAM102B	1	108560089	108644900	0.079255572	PASS
FAM104A	17	73207353	73236753	0.051798665	PASS
FAM104B	23	55143102	55161310	0.158844052	PASS
FAM107A	3	58564117	58627610	-0.727808231	PASS
FAM107B	10	14518557	14774897	0.075711544	PASS
FAM110A	20	833715	857463	-0.172056771	suppress_segment_too_large
FAM110B	8	57994509	58204279	0.182873287	suppress_segment_too_large
FAM110C	2	38814	46870	0.034263343	PASS
FAM110D	1	26159020	26163962	0.048749694	PASS
FAM111A	11	59142748	59155039	0.27006553	suppress_segment_too_large
FAM111B	11	59107185	59127410	0.27006553	suppress_segment_too_large
FAM114A	4	38867677	38945739	-0.753159364	PASS
FAM114A2	5	153990128	154038936	-0.393084436	PASS
FAM117A	17	49710332	49789180	0.051798665	PASS
FAM117B	2	202635188	202769757	0.011001208	PASS
FAM118A	22	45308968	45341955	-0.953096644	PASS
FAM118B	11	126211414	126262986	-0.431650509	PASS
FAM120A	9	93451722	93566107	0.073960965	PASS
FAM120A(	9	93431441	93453592	0.073960965	PASS
FAM120B	6	170290703	170407065	0.033894706	PASS
FAM120C	23	54068324	54183281	0.158844052	PASS
FAM122A	9	68780034	68784608	0.073960965	PASS
FAM122B	23	134769566	134797232	0.158844052	PASS
FAM122C	23	134796395	134854835	0.158844052	PASS
FAM124A	13	51222334	51284241	-0.384313056	PASS
FAM124B	2	224378698	224402085	0.011001208	PASS
FAM126A	7	22889371	23014130	-0.72421718	PASS
FAM126B	2	200973718	201071671	0.011001208	PASS
FAM129A	1	184790724	184974550	0.088103245	PASS
FAM129B	9	127505339	127578989	0.073960965	PASS
FAM129C	19	17523301	17553839	-0.069222905	PASS
FAM131A	3	184335926	184348421	0.02267528	PASS
FAM131B	7	143353400	143362770	-0.712149877	PASS
FAM131C	1	16057769	16073632	0.048749694	PASS
FAM133A	23	93674013	93712274	0.158844052	PASS
FAM133B	7	92560793	92590394	-0.710463272	PASS
FAM135A	6	70412941	70561174	-0.250122709	PASS
FAM135B	8	138130023	138496822	0.493344802	suppress_segment_too_large
FAM136A	2	70295975	70302090	-0.46122572	PASS
FAM13A	4	88725955	89111398	-0.677982248	PASS
FAM13B	5	137937960	138051961	-0.393084436	PASS
FAM13C	10	59246129	59363181	0.032351597	PASS
FAM149A	4	186104419	186172667	0.275137421	PASS
FAM149B	10	73168166	73244504	0.032351597	PASS
FAM151A	1	54609182	54623556	-0.736943052	PASS
FAM151B	5	80487969	80542563	-0.393084436	PASS
FAM153A	5	177707981	177783398	0.165341871	suppress_segment_too_large
FAM153B	5	176060689	176116015	0.165341871	suppress_segment_too_large
FAM153C	5	178006405	178055559	0.165341871	suppress_segment_too_large
FAM155A	13	107163510	107866735	-0.426822582	PASS
FAM155B	23	69505241	69532508	0.158844052	PASS

FAM156A	23	52926402	52995472	0.158844052	PASS
FAM160A	4	151409216	151663632	0.142431244	suppress_segment_too_large
FAM160A:	11	6211335	6234711	-0.39835217	PASS
FAM160B	10	114821744	114899832	0.073090211	PASS
FAM160B:	8	22089159	22104898	-0.311940419	PASS
FAM161A	2	61824854	61854143	-0.46122572	PASS
FAM161B	14	73931501	73950414	0.053252507	PASS
FAM162A	3	122384176	122412334	0.06434429	PASS
FAM162B	6	116752197	116765723	-0.355603029	PASS
FAM163A	1	179743163	179816198	0.088103245	PASS
FAM163B	9	133578415	133586197	0.073960965	PASS
FAM166A	9	137243584	137247770	0.073960965	PASS
FAM166B	9	35561831	35563899	0.073960965	PASS
FAM167A	8	11421463	11475908	-0.311940419	PASS
FAM167B	1	32247233	32248856	0.048749694	PASS
FAM168A	11	73400487	73598189	0.064535884	PASS
FAM168B	2	131047876	131093460	-0.359440172	PASS
FAM169A	5	74777574	74866951	-0.393084436	PASS
FAM169B	15	98437162	98547728	-0.759380635	PASS
FAM170A	5	119629559	119635822	-0.393084436	PASS
FAM170B	10	49131154	49134008	-0.389033546	PASS
FAM171A	10	15211643	15371062	0.075711544	PASS
FAM171A:	17	44353215	44363875	0.051798665	PASS
FAM171B	2	186693971	186765965	-0.120408457	PASS
FAM172A	5	93618069	94111699	-0.393084436	PASS
FAM173A	16	720581	722601	0.053595371	PASS
FAM173B	5	10226330	10249897	0.073527871	PASS
FAM174A	5	100535305	100586741	-0.393084436	PASS
FAM174B	15	92617443	92809884	-0.759380635	PASS
FAM177A	14	35044907	35113130	0.053252507	PASS
FAM177B	1	222737207	222750805	-0.204996646	suppress_segment_too_large
FAM178B	2	96875882	96986592	0.064122364	PASS
FAM180A	7	135728348	135748846	-0.712149877	PASS
FAM180B	11	47586693	47589194	0.059795455	PASS
FAM181A	14	93918894	93929608	-0.155273506	suppress_segment_too_large
FAM181B	11	82729941	82733864	0.064535884	PASS
FAM183A	1	43145153	43156396	-0.736943052	PASS
FAM184A	6	118959763	119149387	-0.355603029	PASS
FAM184B	4	17629306	17781512	-0.358623662	PASS
FAM185A	7	102748971	102809225	-0.712149877	PASS
FAM186A	12	50326230	50396622	-0.743220499	PASS
FAM186B	12	49582885	49605639	-0.743220499	PASS
FAM187A	17	44899712	44905390	0.051798665	PASS
FAM187B	19	35224800	35228729	0.044838622	PASS
FAM189A	15	29120254	29570723	-0.423772929	PASS
FAM189A:	9	69324567	69392558	0.073960965	PASS
FAM189B	1	155247205	155255483	0.088103245	PASS
FAM192A	16	57152466	57186116	-0.423061015	PASS
FAM193A	4	2536631	2732565	-0.358623662	PASS
FAM193B	5	177519788	177554541	0.165341871	suppress_segment_too_large
FAM198A	3	42979267	43060211	-0.727808231	PASS
FAM198B	4	158124474	158173318	0.142431244	suppress_segment_too_large
FAM199X	23	104166620	104195902	0.158844052	PASS
FAM19A1	3	68004216	68545625	-0.727808231	PASS

FAM19A2	12	61708259	62279150	-0.743220499	PASS
FAM19A3	1	112720419	112727235	0.079255572	PASS
FAM19A4	3	68731766	68953297	-0.727808231	PASS
FAM19A5	22	48489460	48850912	-0.953096644	PASS
FAM200A	7	99546308	99558536	-0.710463272	PASS
FAM200B	4	15681662	15705565	-0.358623662	PASS
FAM204A	10	118297930	118342328	0.073090211	PASS
FAM205A	9	34723055	34729467	0.073960965	PASS
FAM205C	9	34889064	34895778	0.073960965	PASS
FAM206A	9	108934181	108950744	0.073960965	PASS
FAM207A	21	44940010	44976989	0.209983559	PASS
FAM208A	3	56620133	56683237	-0.727808231	PASS
FAM208B	10	5684838	5763740	0.075711544	PASS
FAM209A	20	56517187	56526142	-0.172056771	suppress_segment_too_large
FAM209B	20	56533246	56536520	-0.172056771	suppress_segment_too_large
FAM20A	17	68535113	68601389	0.051798665	PASS
FAM20B	1	179025804	179076562	0.088103245	PASS
FAM20C	7	192969	260745	-0.72421718	PASS
FAM210A	18	13663347	13726663	0.044197172	PASS
FAM210B	20	56358915	56368663	-0.172056771	suppress_segment_too_large
FAM214A	15	52581317	52709817	-0.423772929	PASS
FAM214B	9	35104112	35116341	0.073960965	PASS
FAM216A	12	110468364	110490385	0.028722739	PASS
FAM216B	13	42781550	42791549	-0.398748891	PASS
FAM217A	6	4049434	4087344	0.069043418	PASS
FAM217B	20	59933764	59948680	-0.172056771	suppress_segment_too_large
FAM218A	4	164956948	164959122	0.142431244	suppress_segment_too_large
FAM219A	9	34398184	34458570	0.073960965	PASS
FAM219B	15	74899987	74907121	-0.759380635	PASS
FAM220A	7	6329409	6348981	-0.72421718	PASS
FAM221A	7	23680130	23703249	-0.72421718	PASS
FAM221B	9	35816391	35828747	0.073960965	PASS
FAM222A	12	109714228	109770507	0.028722739	PASS
FAM222B	17	28755978	28855232	0.041294512	PASS
FAM227A	22	38578120	38656629	-0.740245994	PASS
FAM227B	15	49326962	49620931	-0.423772929	PASS
FAM228A	2	24175069	24200849	0.034263343	PASS
FAM228B	2	24076526	24169640	0.034263343	PASS
FAM229A	1	32361270	32364278	0.048749694	PASS
FAM229B	6	112087599	112102790	-0.355603029	PASS
FAM234A	16	234546	268971	0.053595371	PASS
FAM234B	12	13044284	13142521	0.49032213	suppress_segment_too_large
FAM236C	23	72912615	72913401	0.158844052	PASS
FAM236D	23	72807425	72808210	0.158844052	PASS
FAM237A	2	206642412	206649011	0.011001208	PASS
FAM240A	3	46612435	46626543	-0.727808231	PASS
FAM241A	4	112145397	112195256	-0.677982248	PASS
FAM241B	10	69630251	69633599	0.032351597	PASS
FAM24A	10	122910701	122913111	0.073090211	PASS
FAM24B	10	122849078	122879641	0.073090211	PASS
FAM25A	10	87020306	87024730	-0.461254852	PASS
FAM25C	10	47995355	47999791	-0.389033546	PASS
FAM32A	19	16185380	16192046	-0.069222905	PASS
FAM3A	23	154506159	154516242	0.158844052	PASS

FAM3B	21	41304212	41357431	0.209983559	PASS
FAM3C	7	121348851	121396364	-0.712149877	PASS
FAM3D	3	58633946	58666848	-0.727808231	PASS
FAM43A	3	194686544	194689037	0.02267528	PASS
FAM43B	1	20552439	20555010	0.048749694	PASS
FAM45A	10	119104086	119137984	0.073090211	PASS
FAM47A	23	34129756	34132314	0.158844052	PASS
FAM47B	23	34942796	34944915	0.158844052	PASS
FAM47C	23	37008397	37011666	0.158844052	PASS
FAM47E	4	76214040	76283780	-0.677982248	PASS
FAM47E-S	4	76251721	76311129	-0.677982248	PASS
FAM49A	2	16549459	16666331	0.034263343	PASS
FAM49B	8	129839593	130017129	0.493344802	suppress_segment_too_large
FAM50A	23	154444126	154450654	0.158844052	PASS
FAM50B	6	3849373	3851320	0.069043418	PASS
FAM53A	4	1617915	1684302	-0.358623662	PASS
FAM53B	10	124619292	124744269	0.073090211	PASS
FAM53C	5	138331935	138349729	-0.393084436	PASS
FAM57A	17	732412	742972	0.06854245	PASS
FAM57B	16	30024427	30052978	0.053595371	PASS
FAM69A	1	92832737	92961522	0.079255572	PASS
FAM69B	9	136712570	136724050	0.073960965	PASS
FAM69C	18	74434099	74457944	-0.330515409	PASS
FAM71A	1	212624474	212626778	-0.204996646	suppress_segment_too_large
FAM71B	5	157161846	157166264	0.165341871	suppress_segment_too_large
FAM71C	12	99647753	99650046	0.028722739	PASS
FAM71D	14	67189393	67228550	0.053252507	PASS
FAM71E1	19	50466785	50476753	0.002743503	PASS
FAM71E2	19	55354908	55363260	0.142581352	PASS
FAM71F1	7	128709061	128731743	-0.712149877	PASS
FAM71F2	7	128672288	128687872	-0.712149877	PASS
FAM72A	1	206186179	206204414	-0.204996646	suppress_segment_too_large
FAM72B	1	121167646	121185539	0.079255572	PASS
FAM72C	1	143955364	143971965	0.079255572	PASS
FAM72D	1	145096000	145112696	0.079255572	PASS
FAM76A	1	27725979	27763122	0.048749694	PASS
FAM76B	11	95768942	95790409	0.32805343	suppress_segment_too_large
FAM78A	9	131258076	131276547	0.073960965	PASS
FAM78B	1	166057426	166166969	0.088103245	PASS
FAM81A	15	59372693	59523549	-0.759380635	PASS
FAM81B	5	95391344	95450454	-0.393084436	PASS
FAM83A	8	123178960	123210079	0.493344802	suppress_segment_too_large
FAM83B	6	54846771	54942022	0.239805577	suppress_segment_too_large
FAM83C	20	35285731	35292401	-0.172056771	suppress_segment_too_large
FAM83D	20	38926312	38953106	-0.172056771	suppress_segment_too_large
FAM83E	19	48600810	48614854	0.002743503	PASS
FAM83F	22	39994949	40043529	-0.740245994	PASS
FAM83G	17	18968789	19004804	0.06854245	PASS
FAM83H	8	143723933	143733801	0.699132336	PASS
FAM84A	2	14632686	14650814	0.034263343	PASS
FAM84B	8	126552442	126558393	0.493344802	suppress_segment_too_large
FAM86B1	8	12182096	12194133	-0.311940419	PASS
FAM86B2	8	12425614	12436343	-0.311940419	PASS
FAM86C1	11	71787510	71801236	0.064535884	PASS

FAM89A	1	231018958	231040246	-0.204996646	suppress_segment_too_large
FAM89B	11	65572349	65574198	0.064535884	PASS
FAM8A1	6	17600355	17611719	0.069043418	PASS
FAM90A1	12	8221260	8227618	-0.015029783	PASS
FAM91A1	8	123768456	123815452	0.493344802	suppress_segment_too_large
FAM92A	8	93698561	93731527	0.355469066	suppress_segment_too_large
FAM92B	16	85098358	85112508	-0.423061015	PASS
FAM95C	9	38540569	38577207	0.073960965	PASS
FAM98A	2	33583658	33599382	0.034263343	PASS
FAM98B	15	38454127	38487710	-0.423772929	PASS
FAM98C	19	38403135	38409088	0.048917043	PASS
FAM9A	23	8790795	8801383	0.158844052	PASS
FAM9B	23	9024232	9164639	0.158844052	PASS
FAM9C	23	13035618	13044682	0.158844052	PASS
FAN1	15	30903852	30943108	-0.423772929	PASS
FANCA	16	89737549	89816657	-0.423061015	PASS
FANCB	23	14835961	14873069	0.158844052	PASS
FANCC	9	95099054	95426796	0.073960965	PASS
FANCD2	3	10026414	10101930	0.06149513	PASS
FANCD2O	3	10081317	10108231	0.06149513	PASS
FANCE	6	35452356	35467103	0.028532667	PASS
FANCF	11	22622890	22625841	-2.208422516	PASS
FANCG	9	35073835	35080016	0.073960965	PASS
FANCI	15	89243949	89317261	-0.759380635	PASS
FANCL	2	58159243	58241372	-0.46122572	PASS
FANCM	14	45135940	45200890	0.053252507	PASS
FANK1	10	125896539	126009592	0.073090211	PASS
FAP	2	162170684	162245151	0.319987345	PASS
FAR1	11	13668670	13732346	-0.39835217	PASS
FAR2	12	29149103	29340980	0.49032213	suppress_segment_too_large
FARP1	13	98142562	98455176	-0.384313056	PASS
FARS2	6	5261044	5829192	0.069043418	PASS
FARSA	19	12922479	12934037	-0.069222905	PASS
FARSB	2	222570536	222656337	0.011001208	PASS
FAS	10	88990582	89015785	-2.417109027	RESCUE
FASLG	1	172659018	172666874	0.088103245	PASS
FASN	17	82078333	82098332	0.051798665	PASS
FASTK	7	151076593	151080866	-0.712149877	PASS
FASTKD1	2	169529749	169573875	0.033541467	PASS
FASTKD2	2	206765357	206792509	0.011001208	PASS
FASTKD3	5	7859159	7869037	0.073527871	PASS
FASTKD5	20	3146519	3159897	-0.172056771	suppress_segment_too_large
FAT1	4	186587783	186726722	0.275137421	PASS
FAT2	5	151504093	151568944	-0.393084436	PASS
FAT3	11	92352096	92896470	0.064535884	PASS
FAT4	4	125316399	125492932	0.142431244	suppress_segment_too_large
FATE1	23	151716035	151723194	0.158844052	PASS
FAU	11	65120628	65122473	0.064535884	PASS
FAXC	6	99271169	99350062	-0.355603029	PASS
FAXDC2	5	154818491	154859252	-0.393084436	PASS
FBF1	17	75909574	75941140	0.051798665	PASS
FBH1	10	5890203	5937594	0.075711544	PASS
FBL	19	39834458	39846414	0.048917043	PASS
FBLIM1	1	15756607	15786594	0.048749694	PASS

FBLL1	5	168529116	168530634	0.165341871	suppress_segment_too_large
FBLN1	22	45502238	45601135	-0.953096644	PASS
FBLN2	3	13549131	13638422	0.06149513	PASS
FBLN5	14	91869412	91947987	-0.155273506	suppress_segment_too_large
FBLN7	2	112138385	112188216	0.040971718	PASS
FBN1	15	48408306	48645849	-0.423772929	PASS
FBN2	5	128257909	128659185	-0.393084436	PASS
FBN3	19	8065402	8149846	-0.071973357	PASS
FBP1	9	94603133	94640249	0.073960965	PASS
FBP2	9	94558720	94593793	0.073960965	PASS
FBRS	16	30658431	30670814	0.053595371	PASS
FBRSL1	12	132489551	132585188	0.028722739	PASS
FBXL12	19	9810267	9827816	-0.107594294	PASS
FBXL13	7	102813230	103074843	-0.712149877	PASS
FBXL14	12	1565993	1594165	-0.015029783	PASS
FBXL15	10	102419189	102423136	-0.414937136	PASS
FBXL16	16	692498	705829	0.053595371	PASS
FBXL17	5	107859035	108382098	-0.393084436	PASS
FBXL18	7	5431335	5513798	-0.72421718	PASS
FBXL19	16	30923055	30948783	0.053595371	PASS
FBXL2	3	33277025	33403662	0.06149513	PASS
FBXL20	17	39252644	39402523	0.041294512	PASS
FBXL22	15	63597353	63602428	-0.759380635	PASS
FBXL3	13	76992598	77027195	-0.384313056	PASS
FBXL4	6	98868538	98948006	-0.355603029	PASS
FBXL5	4	15604539	15681679	-0.358623662	PASS
FBXL6	8	144355431	144359376	0.699132336	PASS
FBXL7	5	15500196	15939795	0.073527871	PASS
FBXL8	16	67159931	67164570	-0.423061015	PASS
FBXO10	9	37510892	37576349	0.073960965	PASS
FBXO11	2	47789316	47905793	0.034263343	PASS
FBXO15	18	74073353	74147865	-0.330515409	PASS
FBXO16	8	28348287	28490318	-0.311940419	PASS
FBXO17	19	38941401	38975910	0.048917043	PASS
FBXO2	1	11637018	11655785	0.364796772	PASS
FBXO21	12	117141988	117190531	0.028722739	PASS
FBXO22	15	75903859	75942510	-0.759380635	PASS
FBXO24	7	100583982	100601117	-0.710463272	PASS
FBXO25	8	406428	477967	-0.311940419	PASS
FBXO27	19	38990714	39032785	0.048917043	PASS
FBXO28	1	224114087	224162047	-0.204996646	suppress_segment_too_large
FBXO3	11	33740939	33774543	-0.359426047	PASS
FBXO30	6	145793502	145814753	0.033894706	PASS
FBXO31	16	87326987	87392142	-0.423061015	PASS
FBXO32	8	123497889	123541206	0.493344802	suppress_segment_too_large
FBXO33	14	39397669	39432500	0.053252507	PASS
FBXO34	14	55271303	55361918	0.053252507	PASS
FBXO36	2	229922302	230013109	0.011001208	PASS
FBXO38	5	148383935	148442836	-0.393084436	PASS
FBXO39	17	6776223	6797101	0.06854245	PASS
FBXO4	5	41925254	41941743	-0.284712224	PASS
FBXO40	3	121593119	121630295	0.06434429	PASS
FBXO41	2	73254682	73284431	-0.46122572	PASS
FBXO42	1	16246839	16352454	0.048749694	PASS

FBXO43	8	100133360	100145800	0.355469066	suppress_segment_too_large
FBXO44	1	11654375	11663327	0.364796772	PASS
FBXO45	3	196568611	196589059	0.02267528	PASS
FBXO46	19	45710629	45730904	0.002743503	PASS
FBXO47	17	38936432	38967402	0.041294512	PASS
FBXO48	2	68459419	68467258	-0.46122572	PASS
FBXO5	6	152970519	152983579	0.033894706	PASS
FBXO6	1	11664124	11674354	0.364796772	PASS
FBXO7	22	32474676	32498829	-0.740245994	PASS
FBXO8	4	174236658	174284264	0.142431244	suppress_segment_too_large
FBXO9	6	53051991	53100873	0.239805577	suppress_segment_too_large
FBXW10	17	18744024	18779349	0.06854245	PASS
FBXW11	5	171861549	172006873	0.165341871	suppress_segment_too_large
FBXW12	3	48372219	48401259	-0.727808231	PASS
FBXW2	9	120751978	120793412	0.073960965	PASS
FBXW4	10	101610664	101695295	-0.414937136	PASS
FBXW5	9	136940435	136944696	0.073960965	PASS
FBXW7	4	152320544	152536063	0.142431244	suppress_segment_too_large
FBXW8	12	116910956	117031148	0.028722739	PASS
FBXW9	19	12688053	12696643	-0.069222905	PASS
FCAMR	1	206957965	206970625	-0.204996646	suppress_segment_too_large
FCAR	19	54874248	54890472	0.142581352	PASS
FCER1A	1	159289714	159308224	0.088103245	PASS
FCER1G	1	161215234	161220699	0.088103245	PASS
FCER2	19	7688758	7702146	-0.071973357	PASS
FCF1	14	74713144	74738620	-0.155273506	suppress_segment_too_large
FCGBP	19	39863323	39934626	0.048917043	PASS
FCGR1A	1	149782671	149792518	0.079255572	PASS
FCGR1B	1	121087345	121096310	0.079255572	PASS
FCGR2A	1	161505430	161524013	0.088103245	PASS
FCGR2B	1	161663147	161678654	0.088103245	PASS
FCGR3A	1	161541759	161550737	0.088103245	PASS
FCGR3B	1	161623196	161631963	0.088103245	PASS
FCGRT	19	49506816	49526333	0.002743503	PASS
FCHO1	19	17747718	17788568	-0.069222905	PASS
FCHO2	5	72955981	73090522	-0.393084436	PASS
FCHSD1	5	141639302	141651419	-0.393084436	PASS
FCHSD2	11	72836745	73142261	0.064535884	PASS
FCMR	1	206904386	206923247	-0.204996646	suppress_segment_too_large
FCN1	9	134905890	134917963	0.073960965	PASS
FCN2	9	134880812	134887520	0.073960965	PASS
FCN3	1	27369112	27374824	0.048749694	PASS
FCRL1	1	157794403	157820105	0.088103245	PASS
FCRL2	1	157745733	157777132	0.088103245	PASS
FCRL3	1	157674321	157700857	0.088103245	PASS
FCRL4	1	157573749	157598080	0.088103245	PASS
FCRL5	1	157513377	157552520	0.088103245	PASS
FCRL6	1	159800511	159816251	0.088103245	PASS
FCRLA	1	161706972	161714352	0.088103245	PASS
FCRLB	1	161721563	161728143	0.088103245	PASS
FDCSP	4	70226071	70235252	0.115781418	suppress_segment_too_large
FDFT1	8	11795573	11839309	-0.311940419	PASS
FDPS	1	155308748	155320666	0.088103245	PASS
FDX1	11	110429883	110464881	-0.431650509	PASS

FDX2	19	10310045	10316015		-0.107594294	PASS
FDXACB1	11	111874056	111881243		-0.431650509	PASS
FDXR	17	74862497	74873031		0.051798665	PASS
FECH	18	57548283	57586772		-0.330515409	PASS
FEM1A	19	4791681	4801273		-0.071973357	PASS
FEM1B	15	68277803	68295865		-0.759380635	PASS
FEM1C	5	115520908	115544894		-0.393084436	PASS
FEN1	11	61792637	61797244		0.064535884	PASS
FER	5	108747822	109196841		-0.393084436	PASS
FER1L5	2	96642737	96704887		0.064122364	PASS
FER1L6	8	123851987	124120061		0.493344802	suppress_segment_too_large
FERD3L	7	19144782	19145421		-0.72421718	PASS
FERMT1	20	6074845	6123544		-0.172056771	suppress_segment_too_large
FERMT2	14	52857268	52952435		0.053252507	PASS
FERMT3	11	64206678	64223886		0.064535884	PASS
FES	15	90883695	90895776		-0.759380635	PASS
FETUB	3	186635969	186653141		0.02267528	PASS
FEV	2	218981087	218985657		0.011001208	PASS
FEZ1	11	125442881	125592568		-0.431650509	PASS
FEZ2	2	36531805	36646087		0.034263343	PASS
FEZF1	7	122301394	122310691		-0.712149877	PASS
FEZF2	3	62369681	62374324		-0.727808231	PASS
FFAR1	19	35351552	35353862		0.044838622	PASS
FFAR2	19	35443907	35451767		0.044838622	PASS
FFAR3	19	35358460	35360485		0.044838622	PASS
FFAR4	10	93566665	93604480		-0.414937136	PASS
FGA	4	154583126	154590766		0.142431244	suppress_segment_too_large
FGB	4	154562956	154571086		0.142431244	suppress_segment_too_large
FGD1	23	54445454	54496166		0.158844052	PASS
FGD2	6	37005646	37029070		0.028532667	PASS
FGD3	9	92947451	93036236		0.073960965	PASS
FGD4	12	32399529	32646050		0.49032213	suppress_segment_too_large
FGD5	3	14810853	14934565		0.06149513	PASS
FGD6	12	95076749	95217482		0.028722739	PASS
FGF1	5	142592178	142698070		-0.393084436	PASS
FGF10	5	44303544	44389706		-0.284712224	PASS
FGF11	17	7438273	7444937		0.06854245	PASS
FGF12	3	192139395	192767764		0.02267528	PASS
FGF13	23	138614731	139222777		0.158844052	PASS
FGF14	13	101710804	102402457		-0.384313056	PASS
FGF16	23	77447405	77457278		0.158844052	PASS
FGF17	8	22042398	22048809		-0.311940419	PASS
FGF18	5	171419656	171457623		0.165341871	suppress_segment_too_large
FGF19	11	69698232	69704642		0.064535884	PASS
FGF2	4	122826708	122898236		0.142431244	suppress_segment_too_large
FGF20	8	16992169	17002181		-0.311940419	PASS
FGF21	19	48755559	48758333		0.002743503	PASS
FGF22	19	639879	644371		-0.071973357	PASS
FGF23	12	4368227	4379728		-0.015029783	PASS
FGF3	11	69809969	69819024		0.064535884	PASS
FGF4	11	69771016	69775403		0.064535884	PASS
FGF5	4	80266599	80336680		-0.677982248	PASS
FGF6	12	4428155	4445614		-0.015029783	PASS
FGF7	15	49423096	49488775		-0.423772929	PASS

FGF8	10	101770130	101780369	-0.414937136	PASS
FGF9	13	21671383	21704498	-0.398748891	PASS
FGFBP1	4	15935569	15938740	-0.358623662	PASS
FGFBP2	4	15960243	15969309	-0.358623662	PASS
FGFBP3	10	91906589	91909483	-0.414937136	PASS
FGFR1	8	38400215	38468834	0.182873287	suppress_segment_too_large
FGFR1OP	6	166999182	167094789	0.033894706	PASS
FGFR1OP	12	26938383	26966650	0.49032213	suppress_segment_too_large
FGFR2	10	121478334	121598458	0.073090211	PASS
FGFR3	4	1793293	1808872	-0.358623662	PASS
FGFR4	5	177086886	177098144	0.165341871	suppress_segment_too_large
FGFRL1	4	1009936	1026897	-0.358623662	PASS
FGG	4	154604134	154612967	0.142431244	suppress_segment_too_large
FGGY	1	59296638	59810647	-0.736943052	PASS
FGL1	8	17864380	17910365	-0.311940419	PASS
FGL2	7	77193371	77199848	-0.701511252	PASS
FGR	1	27612064	27635277	0.048749694	PASS
FH	1	241497603	241519761	-0.287484925	PASS
FHAD1	1	15247272	15400283	0.048749694	PASS
FHDC1	4	152936352	152979696	0.142431244	suppress_segment_too_large
FHIT	3	59749310	61251459	-0.727808231	PASS
FHL1	23	136146702	136211359	0.158844052	PASS
FHL2	2	105357712	105438513	-0.439572519	PASS
FHL3	1	37996770	38005606	0.048749694	PASS
FHL5	6	96562548	96616636	-0.355603029	PASS
FHOD1	16	67229387	67247658	-0.423061015	PASS
FHOD3	18	36297714	36780055	-0.330515409	PASS
FIBCD1	9	130902438	130939286	0.073960965	PASS
FIBIN	11	26994184	26996121	-0.359426047	PASS
FIBP	11	65883741	65888539	0.064535884	PASS
FICD	12	108515185	108525837	0.028722739	PASS
FIG4	6	109691312	109825428	-0.355603029	PASS
FIGLA	2	70777310	70790643	-0.46122572	PASS
FIGN	2	163593396	163736012	0.319987345	PASS
FIGNL1	7	50444128	50542535	-0.701511252	PASS
FIGNL2	12	51817840	51848766	-0.743220499	PASS
FILIP1	6	75291859	75493738	-0.250122709	PASS
FILIP1L	3	99830141	100114513	0.22470242	PASS
FIP1L1	4	53377643	53460861	0.115781418	suppress_segment_too_large
FIS1	7	101239458	101252316	-0.712149877	PASS
FITM1	14	24131275	24132849	0.053252507	PASS
FITM2	20	44302838	44311169	-0.172056771	suppress_segment_too_large
FIZ1	19	55591371	55601970	0.142581352	PASS
FJX1	11	35618419	35620868	-0.359426047	PASS
FKBP10	17	41812680	41823217	0.041294512	PASS
FKBP11	12	48921518	48926474	-0.743220499	PASS
FKBP14	7	30010587	30026684	-0.72421718	PASS
FKBP15	9	113161006	113221361	0.073960965	PASS
FKBP1A	20	1368978	1393172	-0.172056771	suppress_segment_too_large
FKBP1B	2	24049701	24063681	0.034263343	PASS
FKBP1C	6	63211446	63213024	0.239805577	suppress_segment_too_large
FKBP2	11	64241003	64244132	0.064535884	PASS
FKBP3	14	45115600	45135319	0.053252507	PASS
FKBP4	12	2794953	2805423	-0.015029783	PASS

FKBP5	6	35573585	35728583	0.028532667	PASS
FKBP6	7	73328164	73358637	-0.701511252	PASS
FKBP7	2	178463664	178478600	0.033541467	PASS
FKBP8	19	18531751	18544077	-0.069222905	PASS
FKBP9	7	32957404	33006931	-0.72421718	PASS
FKBPL	6	32128707	32130291	0.028532667	PASS
FKRP	19	46746046	46776988	0.002743503	PASS
FKTN	9	105558130	105641118	0.073960965	PASS
FLAD1	1	154983338	154993111	0.088103245	PASS
FLCN	17	17212212	17237188	0.06854245	PASS
FLG	1	152302175	152325203	0.546958914	PASS
FLG2	1	152348735	152360006	0.546958914	PASS
FLI1	11	128686535	128813267	-0.431650509	PASS
FLII	17	18244836	18258916	0.06854245	PASS
FLNA	23	154348524	154374638	0.158844052	PASS
FLNB	3	58008400	58172251	-0.727808231	PASS
FLNC	7	128830377	128859274	-0.712149877	PASS
FLOT1	6	30727709	30742733	0.069043418	PASS
FLOT2	17	28879335	28897679	0.041294512	PASS
FLRT1	11	64103188	64119173	0.064535884	PASS
FLRT2	14	85530144	85654426	-0.155273506	suppress_segment_too_large
FLRT3	20	14322988	14337616	-0.172056771	suppress_segment_too_large
FLT1	13	28300344	28495145	-0.398748891	PASS
FLT3	13	28003274	28100592	-0.398748891	PASS
FLT3LG	19	49474207	49486231	0.002743503	PASS
FLT4	5	180601506	180649624	0.165341871	suppress_segment_too_large
FLVCR1	1	212858255	212899363	-0.204996646	suppress_segment_too_large
FLVCR2	14	75578617	75663214	-0.155273506	suppress_segment_too_large
FLYWCH1	16	2911937	2951208	0.053595371	PASS
FLYWCH2	16	2883186	2899382	0.053595371	PASS
FMC1	7	139339457	139346319	-0.712149877	PASS
FMN1	15	32765545	33194733	-0.423772929	PASS
FMN2	1	240014348	240475189	-0.287484925	PASS
FMNL1	17	45221444	45247320	0.051798665	PASS
FMNL2	2	152335237	152649834	-0.30549516	PASS
FMNL3	12	49636499	49708165	-0.743220499	PASS
FMO1	1	171248471	171285978	0.088103245	PASS
FMO2	1	171185208	171211230	0.088103245	PASS
FMO3	1	171090877	171117819	0.088103245	PASS
FMO4	1	171314208	171342084	0.088103245	PASS
FMO5	1	147175351	147243050	0.079255572	PASS
FMOD	1	203340628	203351758	-0.204996646	suppress_segment_too_large
FMR1	23	147911951	147951125	0.158844052	PASS
FMR1NB	23	147981329	148026667	0.158844052	PASS
FN1	2	215360440	215436172	0.011001208	PASS
FN3K	17	82735575	82751197	0.051798665	PASS
FN3KRP	17	82716683	82730328	0.051798665	PASS
FNBP1	9	129887187	130043194	0.073960965	PASS
FNBP1L	1	93448131	93554661	0.079255572	PASS
FNBP4	11	47716517	47767443	0.059795455	PASS
FNDC1	6	159169397	159272109	0.033894706	PASS
FNDC10	1	1598012	1600096	-0.358281697	PASS
FNDC11	20	63547891	63556708	-0.172056771	suppress_segment_too_large
FNDC3A	13	48975912	49209779	-0.384313056	PASS

FNDC3B	3	172039628	172401665	0.02267528	PASS
FNDC4	2	27491883	27495245	0.034263343	PASS
FNDC5	1	32862268	32872482	0.048749694	PASS
FNDC7	1	108712657	108742740	0.079255572	PASS
FNDC8	17	35121579	35130732	0.041294512	PASS
FNDC9	5	157341600	157345721	0.165341871	suppress_segment_too_large
FNIP1	5	131641714	131797063	-0.393084436	PASS
FNIP2	4	158769138	158908049	0.142431244	suppress_segment_too_large
FNTA	8	43034194	43085788	0.182873287	suppress_segment_too_large
FNTB	14	64986720	65062652	0.053252507	PASS
FO393400.	20	32632183	32743567	-0.172056771	suppress_segment_too_large
FO681492.	10	47750864	47763592	-0.389033546	PASS
FOCAD	9	20658309	20995955	0.073960965	PASS
FOLH1	11	49146635	49208670	-0.210195174	PASS
FOLR1	11	72189558	72196323	0.064535884	PASS
FOLR2	11	72216601	72221950	0.064535884	PASS
FOLR3	11	72114869	72139892	0.064535884	PASS
FOPNL	16	15865720	15888625	0.053595371	PASS
FOS	14	75278774	75282230	-0.155273506	suppress_segment_too_large
FOSB	19	45467995	45475179	0.002743503	PASS
FOSL1	11	65892049	65900573	0.064535884	PASS
FOSL2	2	28392448	28417312	0.034263343	PASS
FOXA1	14	37589984	37596059	0.053252507	PASS
FOXA2	20	22581005	22585455	-0.172056771	suppress_segment_too_large
FOXA3	19	45863989	45873797	0.002743503	PASS
FOXB1	15	60004222	60061730	-0.759380635	PASS
FOXB2	9	77019655	77020953	0.073960965	PASS
FOXC1	6	1609972	1613897	0.069043418	PASS
FOXC2	16	86566829	86569728	-0.423061015	PASS
FOXD1	5	73444827	73448527	-0.393084436	PASS
FOXD2	1	47436017	47440691	-0.736943052	PASS
FOXD3	1	63323041	63325126	0.052232535	PASS
FOXD4	9	116231	118204	0.073960965	PASS
FOXD4L1	2	113498665	113501155	0.040971718	PASS
FOXD4L3	9	68302867	68304905	0.073960965	PASS
FOXD4L4	9	65736555	65738784	0.073960965	PASS
FOXD4L5	9	65282101	65285209	0.073960965	PASS
FOXD4L6	9	41126430	41128463	0.073960965	PASS
FOXE1	9	97853254	97856715	0.073960965	PASS
FOXE3	1	47416072	47418052	-0.736943052	PASS
FOXF1	16	86510527	86515418	-0.423061015	PASS
FOXF2	6	1389576	1395603	0.069043418	PASS
FOXG1	14	28760330	28770277	0.053252507	PASS
FOXH1	8	144473412	144476335	0.699132336	PASS
FOXI1	5	170105897	170109725	0.165341871	suppress_segment_too_large
FOXI2	10	127737235	127741186	0.073090211	PASS
FOXI3	2	88446787	88452656	0.064122364	PASS
FOXJ1	17	76136333	76141299	0.051798665	PASS
FOXJ2	12	8032703	8055503	-0.015029783	PASS
FOXJ3	1	42176539	42335877	-0.736943052	PASS
FOXK1	7	4682309	4771443	-0.72421718	PASS
FOXK2	17	82519713	82644662	0.051798665	PASS
FOXL1	16	86576368	86582160	-0.423061015	PASS
FOXL2	3	138944224	138947140	0.06434429	PASS

FOXL2NB	3	138947234	138953451	0.06434429	PASS
FOXM1	12	2857681	2877155	-0.015029783	PASS
FOXN1	17	28506243	28538896	0.041294512	PASS
FOXN2	2	48314637	48379294	0.034263343	PASS
FOXN3	14	89124871	89619149	-0.155273506	suppress_segment_too_large
FOXN4	12	109277979	109309220	0.028722739	PASS
FOXO1	13	40469953	40666597	-0.398748891	PASS
FOXO3	6	108559835	108684774	-0.355603029	PASS
FOXO4	23	71096197	71103535	0.158844052	PASS
FOXO6	1	41361922	41383590	-0.736943052	PASS
FOXP1	3	70952817	71583993	-0.727808231	PASS
FOXP2	7	114086327	114693772	-0.712149877	PASS
FOXP3	23	49250436	49264826	0.158844052	PASS
FOXP4	6	41546426	41602384	0.028532667	PASS
FOXQ1	6	1312440	1314748	0.069043418	PASS
FOXR1	11	118971707	118981291	-0.431650509	PASS
FOXR2	23	55623400	55625325	0.158844052	PASS
FOXRED1	11	126269055	126278131	-0.431650509	PASS
FOXRED2	22	36487190	36507101	-0.740245994	PASS
FOXS1	20	31844301	31845619	-0.172056771	suppress_segment_too_large
FP565260.	21	5130871	5154734	0.220071762	PASS
FP565260.:	21	5116343	5133805	0.220071762	PASS
FP565260.:	21	5022493	5040666	0.220071762	PASS
FPGS	9	127794597	127814327	0.073960965	PASS
FPGT	1	74198212	74234086	0.052232535	PASS
FPGT-TNN	1	74198235	74544393	0.052232535	PASS
FPR1	19	51745172	51804110	0.002743503	PASS
FPR2	19	51752026	51770526	0.416834572	PASS
FPR3	19	51795163	51826189	0.416834572	PASS
FRA10AC	10	93667883	93702572	-0.414937136	PASS
FRAS1	4	78057570	78544269	-0.677982248	PASS
FRAT1	10	97319267	97321915	-0.414937136	PASS
FRAT2	10	97332497	97334709	-0.414937136	PASS
FREM1	9	14734666	14910995	0.073960965	PASS
FREM2	13	38687129	38887131	-0.398748891	PASS
FREM3	4	143577302	143700675	0.142431244	suppress_segment_too_large
FRG1	4	189940788	189963204	0.275137421	PASS
FRG2	4	190024351	190027257	0.275137421	PASS
FRG2B	10	133623895	133626795	0.073090211	PASS
FRG2C	3	75664330	75667220	-0.727808231	PASS
FRK	6	115931149	116060758	-0.355603029	PASS
FRMD1	6	168053166	168101511	0.033894706	PASS
FRMD3	9	83242990	83538546	0.073960965	PASS
FRMD4A	10	13643706	14462142	0.075711544	PASS
FRMD4B	3	69169990	69542583	-0.727808231	PASS
FRMD5	15	43870761	44195252	-0.423772929	PASS
FRMD6	14	51489100	51730727	0.053252507	PASS
FRMD7	23	132076993	132128020	0.158844052	PASS
FRMD8	11	65386599	65413525	0.064535884	PASS
FRMPD1	9	37651000	37746904	0.073960965	PASS
FRMPD2	10	48153088	48274870	-0.389033546	PASS
FRMPD3	23	107522450	107605251	0.158844052	PASS
FRMPD4	23	12138466	12724523	0.158844052	PASS
FRRS1	1	99708632	99766631	0.079255572	PASS

FRRS1L	9	109130293	109167295	0.073960965	PASS
FRS2	12	69470349	69579789	-0.743220499	PASS
FRS3	6	41770176	41786542	0.028532667	PASS
FRY	13	31846713	32299122	-0.398748891	PASS
FRYL	4	48497361	48780322	0.115781418	suppress_segment_too_large
FRZB	2	182833275	182867162	-0.120408457	PASS
FSBP	8	94372170	94436952	0.355469066	suppress_segment_too_large
FSCB	14	44504342	44507279	0.053252507	PASS
FSCN1	7	5592823	5606655	-0.72421718	PASS
FSCN2	17	81528396	81537130	0.051798665	PASS
FSCN3	7	127591409	127602144	-0.712149877	PASS
FSD1	19	4304600	4323843	-0.071973357	PASS
FSD1L	9	105447796	105552433	0.073960965	PASS
FSD2	15	82755362	82806070	-0.759380635	PASS
FSHB	11	30231016	30235261	-0.359426047	PASS
FSHR	2	48962157	49154537	0.034263343	PASS
FSIP1	15	39588357	39782830	-0.423772929	PASS
FSIP2	2	185738895	185833290	-0.120408457	PASS
FST	5	53480409	53487134	-0.284712224	PASS
FSTL1	3	120392293	120451253	0.06434429	PASS
FSTL3	19	676365	683399	-0.071973357	PASS
FSTL4	5	133196455	133612564	-0.393084436	PASS
FSTL5	4	161383897	162164035	0.142431244	suppress_segment_too_large
FTCD	21	46136262	46155567	0.209983559	PASS
FTCDNL1	2	199760544	199851173	0.011001208	PASS
FTH1	11	61959718	61967660	0.064535884	PASS
FTHL17	23	31071241	31072053	0.158844052	PASS
FTL	19	48965301	48966878	0.002743503	PASS
FTMT	5	121851955	121852833	-0.393084436	PASS
FTO	16	53701692	54158512	-0.423061015	PASS
FTSJ1	23	48476021	48486364	0.158844052	PASS
FTSJ3	17	63819433	63830012	0.051798665	PASS
FUBP1	1	77944055	77979110	0.052232535	PASS
FUBP3	9	130578965	130638352	0.073960965	PASS
FUCA1	1	23845077	23868294	0.048749694	PASS
FUCA2	6	143494811	143511690	0.033894706	PASS
FUK	16	70454421	70480274	-0.423061015	PASS
FUNDC1	23	44523639	44543001	0.158844052	PASS
FUNDC2	23	155025980	155060303	0.158844052	PASS
FUOM	10	133355154	133358035	0.073090211	PASS
FURIN	15	90868592	90883458	-0.759380635	PASS
FUS	16	31180110	31194871	0.053595371	PASS
FUT1	19	48748011	48755390	0.002743503	PASS
FUT10	8	33370824	33473422	-0.311940419	PASS
FUT11	10	73772291	73780251	0.032351597	PASS
FUT2	19	48695971	48705950	0.002743503	PASS
FUT3	19	5842888	5851474	-0.071973357	PASS
FUT4	11	94543840	94549898	0.064535884	PASS
FUT5	19	5865826	5870540	-0.071973357	PASS
FUT6	19	5830610	5839731	-0.071973357	PASS
FUT7	9	137030174	137033010	0.073960965	PASS
FUT8	14	65410592	65744121	0.053252507	PASS
FUT9	6	96015984	96215612	-0.355603029	PASS
FUZ	19	49806869	49817376	0.002743503	PASS

FXN	9	69035259	69120427	0.073960965	PASS
FXR1	3	180868141	180982753	0.02267528	PASS
FXR2	17	7591230	7614871	0.06854245	PASS
FXYD1	19	35138808	35143109	0.044838622	PASS
FXYD2	11	117800844	117828698	-0.431650509	PASS
FXYD3	19	35115879	35124324	0.044838622	PASS
FXYD4	10	43371642	43376335	-0.389033546	PASS
FXYD5	19	35154730	35169883	0.044838622	PASS
FXYD6	11	117836976	117877486	-0.431650509	PASS
FXYD6-F)	11	117820163	117876667	-0.431650509	PASS
FXYD7	19	35143250	35154301	0.044838622	PASS
FYB1	5	39105255	39274528	0.073527871	PASS
FYB2	1	56718804	56819696	-0.736943052	PASS
FYCO1	3	45917899	45995824	-0.727808231	PASS
FYN	6	111660332	111873452	-0.355603029	PASS
FYTTD1	3	197737179	197787596	0.02267528	PASS
FZD1	7	91264364	91271326	-0.710463272	PASS
FZD10	12	130162459	130165740	0.028722739	PASS
FZD2	17	44557459	44559570	0.051798665	PASS
FZD3	8	28494205	28574268	-0.311940419	PASS
FZD4	11	86945679	86955391	0.064535884	PASS
FZD5	2	207762586	207769563	0.011001208	PASS
FZD6	8	103298433	103332866	0.355469066	suppress_segment_too_large
FZD7	2	202034587	202038445	0.011001208	PASS
FZD8	10	35638249	35642278	0.075711544	PASS
FZD9	7	73433783	73436120	-0.701511252	PASS
FZR1	19	3506273	3538330	-0.071973357	PASS
G0S2	1	209675420	209676388	-0.204996646	suppress_segment_too_large
G2E3	14	30559123	30620063	0.053252507	PASS
G3BP1	5	151771045	151812785	-0.393084436	PASS
G3BP2	4	75642782	75724525	-0.677982248	PASS
G6PC	17	42900797	42913369	0.041294512	PASS
G6PC2	2	168901240	168910000	0.033541467	PASS
G6PC3	17	44070735	44076344	0.051798665	PASS
G6PD	23	154531390	154547572	0.158844052	PASS
GAA	17	80101556	80119879	0.051798665	PASS
GAB1	4	143336762	143474568	0.142431244	suppress_segment_too_large
GAB2	11	78215297	78418348	0.064535884	PASS
GAB3	23	154675249	154751583	0.158844052	PASS
GAB4	22	16961936	17008222	-0.740245994	PASS
GABARAI	17	7240014	7242770	0.06854245	PASS
GABARAI	12	10212458	10223130	0.505438545	PASS
GABARAI	16	75566351	75577881	-0.423061015	PASS
GABBR1	6	29555629	29633976	0.069043418	PASS
GABBR2	9	98288082	98709197	0.073960965	PASS
GABPA	21	25734570	25772460	0.209983559	PASS
GABPB1	15	50275392	50355408	-0.423772929	PASS
GABPB2	1	151070578	151125542	0.079255572	PASS
GABRA1	5	161847063	161899981	0.165341871	suppress_segment_too_large
GABRA2	4	46248427	46475230	0.115781418	suppress_segment_too_large
GABRA3	23	152166234	152451358	0.158844052	PASS
GABRA4	4	46918900	46994407	0.115781418	suppress_segment_too_large
GABRA5	15	26866363	26949207	-0.423772929	PASS
GABRA6	5	161547063	161702593	0.165341871	suppress_segment_too_large

GABRB1	4	46993723	47426444	0.115781418	suppress_segment_too_large
GABRB2	5	161288429	161549044	0.165341871	suppress_segment_too_large
GABRB3	15	26543546	26939539	-0.423772929	PASS
GABRD	1	2019298	2030758	-0.358281697	PASS
GABRE	23	151953124	151974680	0.158844052	PASS
GABRG1	4	46035769	46124081	-0.317525751	PASS
GABRG2	5	162000057	162162977	0.165341871	suppress_segment_too_large
GABRG3	15	26971282	27541991	-0.423772929	PASS
GABRP	5	170763350	170814047	0.165341871	suppress_segment_too_large
GABRQ	23	152638183	152657534	0.158844052	PASS
GABRR1	6	89177501	89231278	-0.355603029	PASS
GABRR2	6	89257208	89315299	-0.355603029	PASS
GABRR3	3	97986673	98035304	0.22470242	PASS
GAD1	2	170813213	170861151	0.033541467	PASS
GAD2	10	26216307	26304558	0.075711544	PASS
GADD45A	1	67685061	67688338	0.052232535	PASS
GADD45B	19	2476122	2478259	-0.071973357	PASS
GADD45G	9	89605013	89606555	0.073960965	PASS
GADD45G	19	12953119	12957236	-0.069222905	PASS
GADL1	3	30726200	30894765	0.06149513	PASS
GAGE1	23	49589496	49608536	0.158844052	PASS
GAGE10	23	49303669	49319844	0.158844052	PASS
GAGE12J	23	49322057	49329384	0.158844052	PASS
GAGE2A	23	49589529	49596827	0.158844052	PASS
GAGE2E	23	49341192	49345922	0.158844052	PASS
GAK	4	849276	932373	-0.358623662	PASS
GAL	11	68683779	68691175	0.064535884	PASS
GAL3ST1	22	30554635	30574587	-0.740245994	PASS
GAL3ST3	11	66041952	66049180	0.064535884	PASS
GAL3ST4	7	100159244	100168750	-0.710463272	PASS
GALC	14	87837820	87993665	-0.155273506	suppress_segment_too_large
GALE	1	23795599	23800804	0.048749694	PASS
GALK1	17	75751594	75765711	0.051798665	PASS
GALK2	15	49155656	49367869	-0.423772929	PASS
GALM	2	38665910	38741237	0.034263343	PASS
GALNS	16	88813734	88856970	-0.423061015	PASS
GALNT1	18	35581117	35711834	-0.330515409	PASS
GALNT10	5	154190730	154420984	-0.393084436	PASS
GALNT11	7	152025674	152122347	-0.712149877	PASS
GALNT12	9	98807699	98850081	0.073960965	PASS
GALNT13	2	153871913	154453849	-0.30549516	PASS
GALNT14	2	30910467	31155202	0.034263343	PASS
GALNT15	3	16174649	16231992	0.06149513	PASS
GALNT16	14	69259277	69357033	0.053252507	PASS
GALNT17	7	71132169	71713600	-0.701511252	PASS
GALNT18	11	11270876	11622005	-0.39835217	PASS
GALNT2	1	230057990	230282124	-0.204996646	suppress_segment_too_large
GALNT3	2	165747591	165794682	0.319987345	PASS
GALNT4	12	89519408	89524806	0.271127429	suppress_segment_too_large
GALNT5	2	157257598	157314211	-0.30549516	PASS
GALNT6	12	51351247	51392867	-0.743220499	PASS
GALNT7	4	173168753	173323967	0.142431244	suppress_segment_too_large
GALNT8	12	4720341	4851927	-0.015029783	PASS
GALNT9	12	132196372	132329349	0.028722739	PASS

GALNTL5	7	151956379	152019934	-0.712149877	PASS
GALNTL6	4	171812254	173041559	0.142431244	suppress_segment_too_large
GALP	19	56176020	56185775	0.142581352	PASS
GALR1	18	77250549	77277896	-0.330515409	PASS
GALR2	17	76074794	76077541	0.051798665	PASS
GALR3	22	37823382	37825495	-0.740245994	PASS
GALT	9	34638133	34651035	0.073960965	PASS
GAMT	19	1397026	1401570	-0.071973357	PASS
GAN	16	81314947	81390884	-0.423061015	PASS
GANAB	11	62624826	62646726	0.064535884	PASS
GANC	15	42273233	42356935	-0.423772929	PASS
GAP43	3	115623324	115721490	0.06434429	PASS
GAPDH	12	6533927	6538374	-0.015029783	PASS
GAPDHS	19	35533412	35545316	0.044838622	PASS
GAPT	5	58491435	58497090	-0.284712224	PASS
GAPVD1	9	125261794	125367207	0.073960965	PASS
GAR1	4	109815510	109824740	-0.677982248	PASS
GAREM1	18	32124877	32470484	-0.330515409	PASS
GAREM2	2	26173091	26189663	0.034263343	PASS
GARNL3	9	127224265	127393660	0.073960965	PASS
GARS	7	30594681	30634033	-0.72421718	PASS
GART	21	33503931	33543491	0.209983559	PASS
GAS1	9	86944363	86947189	0.073960965	PASS
GAS2	11	22625509	22813055	-2.208422516	PASS
GAS2L1	22	29306582	29312785	-0.740245994	PASS
GAS2L2	17	35744511	35752878	0.041294512	PASS
GAS2L3	12	100573683	100628286	0.028722739	PASS
GAS6	13	113820549	113864067	-0.426822582	PASS
GAS7	17	9910609	10198551	0.06854245	PASS
GAS8	16	90019629	90044975	-0.423061015	PASS
GAST	17	41712326	41715969	0.041294512	PASS
GATA1	23	48786554	48794311	0.158844052	PASS
GATA2	3	128479427	128493185	0.06434429	PASS
GATA3	10	8045378	8075203	0.075711544	PASS
GATA4	8	11676959	11760002	-0.311940419	PASS
GATA5	20	62463497	62475970	-0.172056771	suppress_segment_too_large
GATA6	18	22169443	22202528	-0.330515409	PASS
GATAD1	7	92447448	92459432	-0.710463272	PASS
GATAD2A	19	19385826	19508931	-0.069222905	PASS
GATAD2E	1	153789030	153923360	0.088103245	PASS
GATB	4	151670504	151761023	0.142431244	suppress_segment_too_large
GATC	12	120446438	120463749	0.028722739	PASS
GATD1	11	767220	777488	-0.39835217	PASS
GATD3B	21	5079294	5128425	0.220071762	PASS
GATM	15	45361124	45402327	-0.423772929	PASS
GBA	1	155234452	155244699	0.088103245	PASS
GBA2	9	35736866	35749228	0.073960965	PASS
GBE1	3	81489699	81762161	0.22470242	PASS
GBF1	10	102245532	102382899	-0.414937136	PASS
GBGT1	9	133152948	133163945	0.073960965	PASS
GBP1	1	89052319	89065360	0.079255572	PASS
GBP2	1	89106132	89150456	0.079255572	PASS
GBP3	1	89006666	89022894	0.079255572	PASS
GBP4	1	89181148	89198932	0.079255572	PASS

GBP5	1	89258950	89272804	0.079255572	PASS
GBP6	1	89364058	89386461	0.079255572	PASS
GBP7	1	89131751	89176040	0.079255572	PASS
GBX1	7	151148589	151174745	-0.712149877	PASS
GBX2	2	236165236	236168369	0.011001208	PASS
GC	4	71741693	71804041	0.115781418	suppress_segment_too_large
GCA	2	162318840	162371595	0.319987345	PASS
GCAT	22	37807905	37817176	-0.740245994	PASS
GCC1	7	127580618	127593611	-0.712149877	PASS
GCC2	2	108448561	108509415	0.040971718	PASS
GCDH	19	12891026	12914207	-0.069222905	PASS
GCFC2	2	75652000	75710989	-0.46122572	PASS
GCG	2	162142873	162152404	0.319987345	PASS
GCGR	17	81804132	81814013	0.051798665	PASS
GCH1	14	54842008	54902852	0.053252507	PASS
GCHFR	15	40764020	40767710	-0.423772929	PASS
GCK	7	44144271	44198170	-0.72421718	PASS
GCKR	2	27496842	27523684	0.034263343	PASS
GCLC	6	53497341	53616970	0.239805577	suppress_segment_too_large
GCLM	1	93885205	93909456	0.079255572	PASS
GCM1	6	53126964	53148829	0.239805577	suppress_segment_too_large
GCM2	6	10873223	10881941	0.069043418	PASS
GCN1	12	120127203	120194710	0.028722739	PASS
GCNA	23	71578411	71613583	0.158844052	PASS
GCNT1	9	76419850	76651203	0.073960965	PASS
GCNT2	6	10492223	10629368	0.069043418	PASS
GCNT3	15	59594875	59640239	-0.759380635	PASS
GCNT4	5	75025346	75030899	-0.393084436	PASS
GCNT7	20	56491492	56525925	-0.172056771	suppress_segment_too_large
GCOM1	15	57591908	57714745	-0.759380635	PASS
GCSAM	3	112120841	112133305	0.06434429	PASS
GCSAML	1	247507058	247577690	-0.145670469	suppress_likely_unfocal_large_gain
GCSAML-	1	247524679	247532613	-0.145670469	suppress_likely_unfocal_large_gain
GCSH	16	81081938	81096425	-0.423061015	PASS
GDA	9	72114595	72257193	0.073960965	PASS
GDAP1	8	74321130	74488872	0.182873287	suppress_segment_too_large
GDAP1L1	20	44247099	44280917	-0.172056771	suppress_segment_too_large
GDAP2	1	117863485	117929630	0.079255572	PASS
GDE1	16	19501689	19522145	0.053595371	PASS
GDF1	19	18868545	18896096	-0.069222905	PASS
GDF10	10	47300386	47313547	-0.389033546	PASS
GDF11	12	55743280	55757278	-0.743220499	PASS
GDF15	19	18374731	18389176	-0.069222905	PASS
GDF2	10	47322490	47326270	-0.389033546	PASS
GDF3	12	7689782	7695776	-0.015029783	PASS
GDF5	20	35433347	35454746	-0.172056771	suppress_segment_too_large
GDF6	8	96142330	96160792	0.355469066	suppress_segment_too_large
GDF7	2	20666664	20679245	0.034263343	PASS
GDF9	5	132861181	132866884	-0.393084436	PASS
GDI1	23	154436913	154443467	0.158844052	PASS
GDI2	10	5765223	5842132	0.075711544	PASS
GDNF	5	37812677	37839686	0.073527871	PASS
GDPD1	17	59220467	59275967	0.051798665	PASS
GDPD2	23	70423031	70433390	0.158844052	PASS

GDPD3	16	30104810	30113856	0.053595371	PASS
GDPD4	11	77216558	77301687	0.064535884	PASS
GDPD5	11	75434640	75525903	0.064535884	PASS
GDPGP1	15	90233808	90245811	-0.759380635	PASS
GEM	8	94249253	94262350	0.355469066	suppress_segment_too_large
GEMIN2	14	39114223	39136973	0.053252507	PASS
GEMIN4	17	744414	753999	0.06854245	PASS
GEMIN5	5	154887416	154938209	-0.393084436	PASS
GEMIN6	2	38751534	38785000	0.034263343	PASS
GEMIN7	19	45079195	45091524	0.002743503	PASS
GEMIN8	23	14008279	14029893	0.158844052	PASS
GEN1	2	17753858	17788941	0.034263343	PASS
GET4	7	876552	896436	-0.72421718	PASS
GFAP	17	44903161	44916937	0.051798665	PASS
GFER	16	1984207	1987749	0.053595371	PASS
GFI1	1	92474762	92486876	0.079255572	PASS
GFI1B	9	132944000	132991687	0.073960965	PASS
GFM1	3	158644278	158692575	0.02267528	PASS
GFM2	5	74721204	74767371	-0.393084436	PASS
GFOD1	6	13357830	13487662	0.069043418	PASS
GFOD2	16	67674531	67719421	-0.423061015	PASS
GFPT1	2	69319769	69387254	-0.46122572	PASS
GFPT2	5	180300690	180353387	0.165341871	suppress_segment_too_large
GFRA1	10	116056925	116273467	0.073090211	PASS
GFRA2	8	21690403	21812357	-0.311940419	PASS
GFRA3	5	138252379	138274671	-0.393084436	PASS
GFRA4	20	3659292	3663399	-0.172056771	suppress_segment_too_large
GFRAL	6	55327469	55402493	0.239805577	suppress_segment_too_large
GFY	19	49423749	49428818	0.002743503	PASS
GGA1	22	37608475	37633564	-0.740245994	PASS
GGA2	16	23463542	23521995	0.053595371	PASS
GGA3	17	75236599	75262363	0.051798665	PASS
GGACT	13	100530164	100589528	-0.384313056	PASS
GGCT	7	30496621	30504844	-0.72421718	PASS
GGCX	2	85544723	85561547	0.064122364	PASS
GGH	8	63015079	63039171	0.182873287	suppress_segment_too_large
GGN	19	38384265	38388082	0.048917043	PASS
GGNBP2	17	36544888	36589848	0.041294512	PASS
GGPS1	1	235327350	235344532	-0.204996646	suppress_segment_too_large
GGT1	22	24583750	24629005	-0.740245994	PASS
GGT5	22	24219654	24245142	-0.740245994	PASS
GGT6	17	4556927	4560818	0.06854245	PASS
GGT7	20	34844720	34872860	-0.172056771	suppress_segment_too_large
GGTLC1	20	23985050	23988779	-0.172056771	suppress_segment_too_large
GGTLC2	22	22644475	22647903	-0.740245994	PASS
GH1	17	63917200	63918838	0.051798665	PASS
GH2	17	63880215	63881935	0.051798665	PASS
GHDC	17	42188799	42194532	0.041294512	PASS
GHITM	10	84139440	84153245	0.032351597	PASS
GHR	5	42423777	42721878	-0.284712224	PASS
GHRH	20	37251082	37261835	-0.172056771	suppress_segment_too_large
GHRHR	7	30938669	30993254	-0.72421718	PASS
GHRL	3	10285675	10292947	0.06149513	PASS
GHSR	3	172445133	172448456	0.02267528	PASS

GID4	17	18039292	18068404	0.06854245	PASS
GID8	20	62938119	62948475	-0.172056771	suppress_segment_too_large
GIF	11	59829268	59845501	0.27006553	suppress_segment_too_large
GIGYF1	7	100679507	100694037	-0.710463272	PASS
GIGYF2	2	232697299	232860575	0.011001208	PASS
GIMAP1	7	150716557	150724284	-0.712149877	PASS
GIMAP1-(	7	150716668	150743646	-0.712149877	PASS
GIMAP2	7	150685697	150693641	-0.712149877	PASS
GIMAP4	7	150567277	150573955	-0.712149877	PASS
GIMAP5	7	150722253	150750033	-0.712149877	PASS
GIMAP6	7	150625375	150632648	-0.712149877	PASS
GIMAP7	7	150514830	150521073	-0.712149877	PASS
GIMAP8	7	150450630	150479392	-0.712149877	PASS
GIMD1	4	106357485	106368825	-0.677982248	PASS
GIN1	5	103086000	103120151	-0.393084436	PASS
GINM1	6	149566294	149591748	0.033894706	PASS
GINS1	20	25407727	25452628	-0.172056771	suppress_segment_too_large
GINS2	16	85676198	85690073	-0.423061015	PASS
GINS3	16	58295080	58406144	-0.423061015	PASS
GINS4	8	41529206	41545046	0.182873287	suppress_segment_too_large
GIP	17	48958554	48968596	0.051798665	PASS
GIPC1	19	14477760	14496149	-0.069222905	PASS
GIPC2	1	77979542	78138449	0.052232535	PASS
GIPC3	19	3585553	3593541	-0.071973357	PASS
GIPR	19	45668244	45683724	0.002743503	PASS
GIT1	17	29573469	29594054	0.041294512	PASS
GIT2	12	109929792	109996389	0.028722739	PASS
GJA1	6	121435577	121449727	-0.355603029	PASS
GJA10	6	89894469	89921760	-0.355603029	PASS
GJA3	13	20138255	20161049	-0.398748891	PASS
GJA4	1	34792998	34795747	0.048749694	PASS
GJA5	1	147756199	147773362	0.079255572	PASS
GJA8	1	147907956	147909257	0.079255572	PASS
GJA9	1	38874451	38881587	0.048749694	PASS
GJA9-MY(	1	38864501	38881617	0.048749694	PASS
GJB1	23	71215194	71225516	0.158844052	PASS
GJB2	13	20187463	20193010	-0.398748891	PASS
GJB3	1	34781189	34786369	0.048749694	PASS
GJB4	1	34759741	34763724	0.048749694	PASS
GJB5	1	34755047	34758512	0.048749694	PASS
GJB6	13	20221962	20232395	-0.398748891	PASS
GJB7	6	87282978	87329278	-0.355603029	PASS
GJC1	17	44798448	44830816	0.051798665	PASS
GJC2	1	228149852	228159826	-0.204996646	suppress_segment_too_large
GJC3	7	99923269	99929620	-0.710463272	PASS
GJD2	15	34751032	34754965	-0.423772929	PASS
GJD3	17	40362931	40363815	0.041294512	PASS
GJD4	10	35605410	35608935	0.075711544	PASS
GJE1	6	142133090	142135151	0.033894706	PASS
GK	23	30653359	30731456	0.158844052	PASS
GK2	4	79406352	79408293	-0.677982248	PASS
GK3P	4	165277812	165279679	0.142431244	suppress_segment_too_large
GK5	3	142157527	142225607	-0.889410434	PASS
GKAP1	9	83739421	83829516	0.073960965	PASS

GKN1	2	68974573	68980974	-0.46122572	PASS
GKN2	2	68945232	68952970	-0.46122572	PASS
GLA	23	101397803	101408012	0.158844052	PASS
GLB1	3	32996608	33097230	0.06149513	PASS
GLB1L	2	219236606	219245478	0.011001208	PASS
GLB1L2	11	134331874	134378341	0.105032849	PASS
GLB1L3	11	134274245	134319564	0.105032849	PASS
GLCCI1	7	7968794	8094272	-0.72421718	PASS
GLCE	15	69160584	69272217	-0.759380635	PASS
GLDC	9	6532464	6645783	0.073960965	PASS
GLDN	15	51341629	51408013	-0.423772929	PASS
GLE1	9	128504700	128542288	0.073960965	PASS
GLG1	16	74447427	74607144	-0.423061015	PASS
GLI1	12	57460135	57472262	-0.743220499	PASS
GLI2	2	120735623	120992653	-0.359440172	PASS
GLI3	7	41960950	42237870	-0.72421718	PASS
GLI4	8	143267433	143276931	0.699132336	PASS
GLIPR1	12	75480680	75503853	0.271127429	suppress_segment_too_large
GLIPR1L1	12	75334639	75370560	0.271127429	suppress_segment_too_large
GLIPR1L2	12	75391070	75432688	0.271127429	suppress_segment_too_large
GLIPR2	9	36136536	36163913	0.073960965	PASS
GLIS1	1	53506237	53738106	-0.736943052	PASS
GLIS2	16	4314761	4339597	0.053595371	PASS
GLIS3	9	3824127	4348392	0.073960965	PASS
GLMN	1	92246402	92298987	0.079255572	PASS
GLMP	1	156290089	156295689	0.088103245	PASS
GLO1	6	38675925	38703141	0.028532667	PASS
GLOD4	17	757097	783390	0.06854245	PASS
GLOD5	23	48761750	48773648	0.158844052	PASS
GLP1R	6	39048798	39087743	0.028532667	PASS
GLP2R	17	9822206	9892102	0.06854245	PASS
GLRA1	5	151822513	151924842	-0.393084436	PASS
GLRA2	23	14529298	14731812	0.158844052	PASS
GLRA3	4	174636914	174829314	0.142431244	suppress_segment_too_large
GLRA4	23	103707224	103728655	0.158844052	PASS
GLRB	4	157076057	157172090	0.142431244	suppress_segment_too_large
GLRX	5	95751319	95823005	-0.393084436	PASS
GLRX2	1	193090866	193106114	0.088103245	PASS
GLRX3	10	130136399	130184521	0.073090211	PASS
GLRX5	14	95533503	95544724	-0.155273506	suppress_segment_too_large
GLS	2	190880827	190965552	0.011001208	PASS
GLS2	12	56470944	56488414	-0.743220499	PASS
GLT1D1	12	128853427	128984968	0.028722739	PASS
GLT6D1	9	135623656	135639540	0.073960965	PASS
GLT8D1	3	52694488	52706032	-0.727808231	PASS
GLT8D2	12	103988984	104064183	0.028722739	PASS
GLTP	12	109850943	109880488	0.028722739	PASS
GLTPD2	17	4788959	4790390	0.06854245	PASS
GLUD1	10	87050486	87094866	-0.461254852	PASS
GLUD2	23	121047588	121050080	0.158844052	PASS
GLUL	1	182381704	182392206	0.088103245	PASS
GLYAT	11	58640426	58731974	0.27006553	suppress_segment_too_large
GLYATL1	11	58905398	59043527	0.27006553	suppress_segment_too_large
GLYATL1	11	59086307	59094786	0.27006553	suppress_segment_too_large

GLYATL2	11	58834065	58904215	0.27006553	suppress_segment_too_large
GLYATL3	6	49499958	49527047	0.239805577	suppress_segment_too_large
GLYCTK	3	52287089	52293476	-0.727808231	PASS
GLYR1	16	4803203	4847342	0.053595371	PASS
GM2A	5	151212150	151270440	-0.393084436	PASS
GMCL1	2	69829642	69881396	-0.46122572	PASS
GMCL2	5	178184503	178187432	0.165341871	suppress_segment_too_large
GMDS	6	1623806	2245692	0.069043418	PASS
GMEB1	1	28668732	28719353	0.048749694	PASS
GMEB2	20	63587602	63627041	-0.172056771	suppress_segment_too_large
GMFB	14	54474484	54489196	0.053252507	PASS
GMFG	19	39328353	39342372	0.048917043	PASS
GMIP	19	19629476	19643667	-0.069222905	PASS
GML	8	142834247	142916506	0.699132336	PASS
GMNC	3	190852877	190892429	0.02267528	PASS
GMNN	6	24774931	24786099	0.069043418	PASS
GMPPA	2	219498867	219506989	0.011001208	PASS
GMPPB	3	49716844	49723951	-0.727808231	PASS
GMPR	6	16238580	16295549	0.069043418	PASS
GMPR2	14	24232422	24239242	0.053252507	PASS
GMPS	3	155870536	155944026	0.02267528	PASS
GNA11	19	3094410	3124004	-0.071973357	PASS
GNA12	7	2728112	2844324	-0.72421718	PASS
GNA13	17	65010715	65056839	0.051798665	PASS
GNA14	9	77423079	77648307	0.073960965	PASS
GNA15	19	3136193	3163769	-0.071973357	PASS
GNAI1	7	79768028	80226181	-0.701511252	PASS
GNAI2	3	50226292	50259355	-0.727808231	PASS
GNAI3	1	109548611	109618321	0.079255572	PASS
GNAL	18	11688956	11885685	0.044197172	PASS
GNAO1	16	56191347	56357457	-0.423061015	PASS
GNAQ	9	77716087	78031458	0.073960965	PASS
GNAS	20	58839718	58911192	-0.172056771	suppress_segment_too_large
GNAT1	3	50191612	50196516	-0.727808231	PASS
GNAT2	1	109603267	109619929	0.079255572	PASS
GNAT3	7	80458671	80512020	-0.701511252	PASS
GNAZ	22	23070361	23125037	-0.740245994	PASS
GNB1	1	1785285	1891117	-0.358281697	PASS
GNB1L	22	19783224	19854939	-0.740245994	PASS
GNB2	7	100673531	100679174	-0.710463272	PASS
GNB3	12	6839954	6847393	-0.015029783	PASS
GNB4	3	179396089	179451590	0.02267528	PASS
GNB5	15	52115105	52191369	-0.423772929	PASS
GNE	9	36214441	36277056	0.073960965	PASS
GNG10	9	111661335	111670246	0.073960965	PASS
GNG11	7	93921699	93928610	-0.710463272	PASS
GNG12	1	67701466	67833467	0.052232535	PASS
GNG13	16	798041	800733	0.053595371	PASS
GNG14	19	12687998	12688422	-0.069222905	PASS
GNG2	14	51826195	51979342	0.053252507	PASS
GNG3	11	62707658	62709201	0.064535884	PASS
GNG4	1	235547687	235650754	-0.204996646	suppress_segment_too_large
GNG5	1	84498325	84506565	0.587598296	PASS
GNG7	19	2511219	2702709	-0.071973357	PASS

GNG8	19	46634076	46634685	0.002743503	PASS
GNGT1	7	93591573	93911265	-0.710463272	PASS
GNGT2	17	49202791	49210574	0.051798665	PASS
GNL1	6	30541377	30557174	0.069043418	PASS
GNL2	1	37566816	37595935	0.048749694	PASS
GNL3	3	52681156	52694492	-0.727808231	PASS
GNL3L	23	54530211	54561071	0.158844052	PASS
GNLY	2	85685175	85698854	0.064122364	PASS
GNMT	6	42960758	42963880	0.028532667	PASS
GNPAT	1	231241207	231277973	-0.204996646	suppress_segment_too_large
GNPDA1	5	141991749	142013041	-0.393084436	PASS
GNPDA2	4	44682200	44726595	-0.317525751	PASS
GNPNAT1	14	52775194	52791668	0.053252507	PASS
GNPTAB	12	101745497	101830938	0.028722739	PASS
GNPTG	16	1351923	1364113	0.053595371	PASS
GNRH1	8	25419260	25424654	-0.311940419	PASS
GNRH2	20	3043622	3045747	-0.172056771	suppress_segment_too_large
GNRHR	4	67739328	67754360	0.115781418	suppress_segment_too_large
GNS	12	64713445	64759447	-0.743220499	PASS
GOLGA1	9	124878357	124948492	0.073960965	PASS
GOLGA2	9	128255829	128275995	0.073960965	PASS
GOLGA3	12	132768909	132828858	0.232349073	PASS
GOLGA4	3	37243177	37366751	-0.727808231	PASS
GOLGA5	14	92794231	92839963	-0.155273506	suppress_segment_too_large
GOLGA6A	15	74069857	74082550	-0.759380635	PASS
GOLGA6B	15	72654738	72666394	-0.759380635	PASS
GOLGA6C	15	75258599	75270199	-0.759380635	PASS
GOLGA6D	15	75282835	75295807	-0.759380635	PASS
GOLGA6L	15	23128268	23136822	-0.423772929	PASS
GOLGA6L	15	82339998	82349475	-0.759380635	PASS
GOLGA6L	15	23439498	23447234	-0.423772929	PASS
GOLGA6L	15	22458903	22469230	-0.423772929	PASS
GOLGA6L	15	84235773	84245368	-0.759380635	PASS
GOLGA6L	15	28841833	28848675	-0.423772929	PASS
GOLGA6L	15	82430018	82439153	-0.759380635	PASS
GOLGA7	8	41490396	41510980	0.182873287	suppress_segment_too_large
GOLGA7B	10	97850239	97871580	-0.414937136	PASS
GOLGA8A	15	34379068	34437466	-0.423772929	PASS
GOLGA8B	15	34525207	34588503	-0.423772929	PASS
GOLGA8F	15	28378621	28392018	-0.423772929	PASS
GOLGA8H	15	30604126	30614561	-0.423772929	PASS
GOLGA8J	15	30083053	30093499	-0.423772929	PASS
GOLGA8K	15	32392782	32403292	-0.423772929	PASS
GOLGA8M	15	28701954	28738384	-0.423772929	PASS
GOLGA8N	15	32593456	32607310	-0.423772929	PASS
GOLGA8O	15	32445106	32455634	-0.423772929	PASS
GOLGA8Q	15	30552078	30562501	-0.423772929	PASS
GOLGA8R	15	30403740	30414162	-0.423772929	PASS
GOLGA8S	15	23354748	23367231	-0.423772929	PASS
GOLGA8T	15	30135149	30145567	-0.423772929	PASS
GOLGB1	3	121663199	121749767	0.06434429	PASS
GOLIM4	3	168008677	168095975	0.02267528	PASS
GOLM1	9	86026146	86100173	0.073960965	PASS
GOLPH3	5	32124704	32174350	0.073527871	PASS

GOLPH3L	1	150646225	150697144	0.079255572	PASS
GOLT1A	1	204198160	204214092	-0.204996646	suppress_segment_too_large
GOLT1B	12	21501781	21518408	0.49032213	suppress_segment_too_large
GON4L	1	155749662	155859400	0.088103245	PASS
GON7	14	93202894	93207094	-0.155273506	suppress_segment_too_large
GOPC	6	117560269	117602542	-0.355603029	PASS
GORAB	1	170532129	170553446	0.088103245	PASS
GORASP1	3	39096659	39108363	-0.727808231	PASS
GORASP2	2	170928464	170967129	0.033541467	PASS
GOSR1	17	30477362	30527592	0.041294512	PASS
GOSR2	17	46923075	46975524	0.051798665	PASS
GOT1	10	99396870	99431569	-0.414937136	PASS
GOT1L1	8	37934281	37940129	0.182873287	suppress_segment_too_large
GOT2	16	58707131	58734357	-0.423061015	PASS
GP1BA	17	4932297	4935030	0.06854245	PASS
GP1BB	22	19722945	19724771	-0.740245994	PASS
GP2	16	20309572	20327808	0.053595371	PASS
GP5	3	194394821	194398354	0.02267528	PASS
GP6	19	55013705	55038264	0.142581352	PASS
GP9	3	129060767	129062406	0.06434429	PASS
GPA33	1	167052836	167166479	0.088103245	PASS
GPAA1	8	144082590	144086216	0.699132336	PASS
GPALPP1	13	44989529	45037669	-0.398748891	PASS
GPAM	10	112149864	112215377	0.073090211	PASS
GPANK1	6	31661229	31666283	0.028532667	PASS
GPAT2	2	96021946	96039451	0.064122364	PASS
GPAT3	4	83535914	83605875	-0.677982248	PASS
GPAT4	8	41577187	41625001	0.182873287	suppress_segment_too_large
GPATCH1	19	33080880	33130542	0.044838622	PASS
GPATCH1	2	37084451	37099244	0.034263343	PASS
GPATCH2	1	217426992	217631082	-0.204996646	suppress_segment_too_large
GPATCH2	14	76151916	76254342	-0.155273506	suppress_segment_too_large
GPATCH3	1	26890488	26900466	0.048749694	PASS
GPATCH4	1	156594487	156601496	0.088103245	PASS
GPATCH8	17	44395284	44503430	0.051798665	PASS
GPBAR1	2	218259496	218263859	0.011001208	PASS
GPBP1	5	57173948	57264679	-0.284712224	PASS
GPBP1L1	1	45627304	45688113	-0.736943052	PASS
GPC1	2	240435671	240468078	0.011001208	PASS
GPC2	7	100169606	100177372	-0.710463272	PASS
GPC3	23	133535745	133985895	0.158844052	PASS
GPC4	23	133300103	133415490	0.158844052	PASS
GPC5	13	91398607	92873682	-0.384313056	PASS
GPC6	13	93226842	94407401	-0.384313056	PASS
GPCPD1	20	5544404	5611026	-0.172056771	suppress_segment_too_large
GPD1	12	50103819	50111319	-0.743220499	PASS
GPD1L	3	32105689	32168713	0.06149513	PASS
GPD2	2	156435290	156613735	-0.30549516	PASS
GPER1	7	1082208	1093815	-0.72421718	PASS
GPHA2	11	64934471	64935888	0.064535884	PASS
GPHB5	14	63312835	63318879	0.053252507	PASS
GPHN	14	66507407	67181803	0.053252507	PASS
GPI	19	34359480	34402413	0.044838622	PASS
GPIHBP1	8	143213193	143217170	0.699132336	PASS

GPKOW	23	49113389	49123801	0.158844052	PASS
GPLD1	6	24424565	24495205	0.069043418	PASS
GPM6A	4	175632934	176002664	0.142431244	suppress_segment_too_large
GPM6B	23	13771031	13938638	0.158844052	PASS
GPN1	2	27628247	27651508	0.034263343	PASS
GPN2	1	26876133	26890297	0.048749694	PASS
GPN3	12	110452484	110469268	0.028722739	PASS
GPNMB	7	23235967	23275108	-0.72421718	PASS
GPR1	2	206175316	206218047	0.011001208	PASS
GPR101	23	137030148	137031674	0.158844052	PASS
GPR107	9	130053426	130140169	0.073960965	PASS
GPR108	19	6729914	6737603	-0.071973357	PASS
GPR119	23	130384440	130385447	0.158844052	PASS
GPR12	13	26755200	26760785	-0.398748891	PASS
GPR132	14	105049389	105065445	-0.155273506	suppress_segment_too_large
GPR135	14	59429022	59465342	0.053252507	PASS
GPR137	11	64270062	64289500	0.064535884	PASS
GPR137B	1	236142505	236221865	-0.204996646	suppress_segment_too_large
GPR137C	14	52553148	52637713	0.053252507	PASS
GPR139	16	20031485	20073917	0.053595371	PASS
GPR141	7	37683796	37833788	-0.72421718	PASS
GPR142	17	74367407	74372622	0.051798665	PASS
GPR143	23	9725346	9786297	0.158844052	PASS
GPR146	7	1044576	1059261	-0.72421718	PASS
GPR148	2	130729070	130730336	-0.359440172	PASS
GPR149	3	154337672	154429715	0.02267528	PASS
GPR15	3	98531899	98533150	0.22470242	PASS
GPR150	5	95620078	95622142	-0.393084436	PASS
GPR151	5	146513103	146516190	-0.393084436	PASS
GPR152	11	67451301	67452729	0.064535884	PASS
GPR153	1	6247346	6260975	0.364796772	PASS
GPR155	2	174431571	174487094	0.033541467	PASS
GPR156	3	120164645	120285094	0.06434429	PASS
GPR157	1	9100305	9129170	0.364796772	PASS
GPR158	10	25174802	25602226	0.075711544	PASS
GPR160	3	170037929	170085403	0.02267528	PASS
GPR161	1	168079543	168137667	0.088103245	PASS
GPR162	12	6821545	6829972	-0.015029783	PASS
GPR17	2	127645864	127652639	-0.359440172	PASS
GPR171	3	151197832	151203201	0.02267528	PASS
GPR173	23	53049091	53080615	0.158844052	PASS
GPR174	23	79144663	79175315	0.158844052	PASS
GPR176	15	39799032	39920892	-0.423772929	PASS
GPR179	17	38325530	38343847	0.041294512	PASS
GPR18	13	99254714	99261744	-0.384313056	PASS
GPR180	13	94601903	94634645	-0.384313056	PASS
GPR182	12	56994446	56998441	-0.743220499	PASS
GPR183	13	99294530	99307405	-0.384313056	PASS
GPR19	12	12660891	12696207	0.49032213	suppress_segment_too_large
GPR20	8	141356500	141367267	0.699132336	PASS
GPR21	9	123034527	123035696	0.073960965	PASS
GPR22	7	107470018	107475659	-0.712149877	PASS
GPR25	1	200872955	200874178	-0.204996646	suppress_segment_too_large
GPR26	10	123666355	123694607	0.073090211	PASS

GPR27	3	71754050	71756496	-0.727808231	PASS
GPR3	1	27392644	27395814	0.048749694	PASS
GPR31	6	167156271	167158329	0.033894706	PASS
GPR32	19	50770464	50771732	0.002743503	PASS
GPR33	14	31482875	31488142	0.053252507	PASS
GPR34	23	41688973	41697277	0.158844052	PASS
GPR35	2	240605431	240631259	0.011001208	PASS
GPR37	7	124745997	124765627	-0.712149877	PASS
GPR37L1	1	202122858	202133592	-0.204996646	suppress_segment_too_large
GPR39	2	132416574	132646559	-0.359440172	PASS
GPR4	19	45589764	45602208	0.002743503	PASS
GPR42	19	35370929	35372962	0.044838622	PASS
GPR45	2	105241743	105243049	-0.439572519	PASS
GPR50	23	151176653	151181465	0.158844052	PASS
GPR52	1	174448111	174449198	0.088103245	PASS
GPR55	2	230907318	230961066	0.011001208	PASS
GPR6	6	109978256	109980718	-0.355603029	PASS
GPR61	1	109539872	109548406	0.079255572	PASS
GPR62	3	51955314	51957504	-0.727808231	PASS
GPR63	6	96794126	96837463	-0.355603029	PASS
GPR65	14	88005124	88014811	-0.155273506	suppress_segment_too_large
GPR68	14	91232532	91253925	-0.155273506	suppress_segment_too_large
GPR75	2	53852913	53859989	0.034263343	PASS
GPR75-AS	2	53670293	53860160	0.034263343	PASS
GPR78	4	8558725	8619761	-0.358623662	PASS
GPR82	23	41724155	41730135	0.158844052	PASS
GPR83	11	94377311	94401419	0.064535884	PASS
GPR84	12	54362445	54364487	-0.743220499	PASS
GPR85	7	113078331	113087778	-0.712149877	PASS
GPR87	3	151294086	151316952	0.02267528	PASS
GPR88	1	100538137	100542018	0.079255572	PASS
GPR89A	1	145607990	145670648	0.079255572	PASS
GPR89B	1	147928393	147993521	0.079255572	PASS
GPRASP1	23	102651366	102659083	0.158844052	PASS
GPRASP2	23	102712176	102717733	0.158844052	PASS
GPRC5A	12	12890782	12917937	0.49032213	suppress_segment_too_large
GPRC5B	16	19856691	19886167	0.053595371	PASS
GPRC5C	17	74424851	74451653	0.051798665	PASS
GPRC5D	12	12940775	12952147	0.49032213	suppress_segment_too_large
GPRC6A	6	116792085	116829037	-0.355603029	PASS
GPRIN1	5	176595802	176610133	0.165341871	suppress_segment_too_large
GPRIN2	10	46549044	46555530	-0.389033546	PASS
GPRIN3	4	89236386	89308010	-0.677982248	PASS
GPS1	17	82050691	82057470	0.051798665	PASS
GPS2	17	7311324	7315564	0.06854245	PASS
GPSM1	9	136327476	136359605	0.073960965	PASS
GPSM2	1	108875350	108934545	0.079255572	PASS
GPSM3	6	32190766	32195523	0.028532667	PASS
GPT	8	144502973	144507174	0.699132336	PASS
GPT2	16	46884378	46931297	0.053595371	PASS
GPX2	14	64939152	64942905	0.053252507	PASS
GPX3	5	151020438	151028993	-0.393084436	PASS
GPX4	19	1103926	1106791	-0.071973357	PASS
GPX5	6	28525925	28534952	0.069043418	PASS

GPX6	6	28503296	28528215	0.069043418	PASS
GPX7	1	52602372	52609051	-0.736943052	PASS
GPX8	5	55160118	55167071	-0.284712224	PASS
GRAMD1A	19	34994784	35026471	0.044838622	PASS
GRAMD1B	11	123358428	123627774	-0.431650509	PASS
GRAMD1C	3	113828182	113947174	0.06434429	PASS
GRAMD2A	15	72159807	72197785	-0.759380635	PASS
GRAMD2B	5	126360132	126496494	-0.393084436	PASS
GRAMD4	22	46576012	46679790	-0.953096644	PASS
GRAP	17	19020673	19047637	0.06854245	PASS
GRAP2	22	39901082	39973721	-0.740245994	PASS
GRAPL	17	19127469	19159176	0.06854245	PASS
GRASP	12	52006940	52015889	-0.743220499	PASS
GRB10	7	50590063	50793462	-0.701511252	PASS
GRB14	2	164492812	164621848	0.319987345	PASS
GRB2	17	75318076	75405709	0.051798665	PASS
GRB7	17	39737927	39747291	0.041294512	PASS
GREB1	2	11482341	11642788	0.034263343	PASS
GREB1L	18	21242242	21525417	-0.330515409	PASS
GREM1	15	32717974	32745107	-0.423772929	PASS
GREM2	1	240489573	240612149	-0.287484925	PASS
GRHL1	2	9951693	10002277	0.034263343	PASS
GRHL2	8	101492432	101669726	0.355469066	suppress_segment_too_large
GRHL3	1	24319322	24364482	0.048749694	PASS
GRHPR	9	37422666	37436990	0.073960965	PASS
GRIA1	5	153489615	153813869	-0.393084436	PASS
GRIA2	4	157204182	157366075	0.142431244	suppress_segment_too_large
GRIA3	23	123184153	123490915	0.158844052	PASS
GRIA4	11	105609994	105982092	0.32805343	suppress_segment_too_large
GRID1	10	85599555	86366493	0.032351597	PASS
GRID2	4	92303622	93810157	-0.677982248	PASS
GRID2IP	7	6497462	6551436	-0.72421718	PASS
GRIFIN	7	2474844	2476894	-0.72421718	PASS
GRIK1	21	29536933	29940033	0.209983559	PASS
GRIK2	6	101181257	102070083	-0.355603029	PASS
GRIK3	1	36795527	37034129	0.048749694	PASS
GRIK4	11	120511700	120988904	-0.431650509	PASS
GRIK5	19	41998321	42069498	0.048917043	PASS
GRIN1	9	137138390	137168762	0.073960965	PASS
GRIN2A	16	9753404	10182754	0.053595371	PASS
GRIN2B	12	13437942	13981957	0.49032213	suppress_segment_too_large
GRIN2C	17	74842023	74861504	0.051798665	PASS
GRIN2D	19	48394875	48444931	0.002743503	PASS
GRIN3A	9	101569353	101738580	0.073960965	PASS
GRIN3B	19	1000419	1009732	-0.071973357	PASS
GRINA	8	143990058	143993415	0.699132336	PASS
GRIP1	12	66347431	67069162	-0.743220499	PASS
GRIP2	3	14489111	14556075	0.06149513	PASS
GRIPAP1	23	48973720	49002264	0.158844052	PASS
GRK1	13	113667155	113737735	-0.426822582	PASS
GRK2	11	67266410	67286556	0.064535884	PASS
GRK3	22	25564849	25729294	-0.740245994	PASS
GRK4	4	2963608	3040747	-0.358623662	PASS
GRK5	10	119207589	119459742	0.073090211	PASS

GRK6	5	177403204	177442901	0.165341871	suppress_segment_too_large
GRK7	3	141778148	141818490	-0.889410434	PASS
GRM1	6	146027646	146437598	0.033894706	PASS
GRM2	3	51707070	51718613	-0.727808231	PASS
GRM3	7	86643914	86864884	-0.221864321	PASS
GRM4	6	34018645	34155622	0.028532667	PASS
GRM5	11	88504576	89065945	0.064535884	PASS
GRM6	5	178977587	178996206	0.165341871	suppress_segment_too_large
GRM7	3	6770001	7741533	0.06149513	PASS
GRM8	7	126438598	127253294	-0.712149877	PASS
GRN	17	44345086	44353102	0.051798665	PASS
GRP	18	59220168	59230774	-0.330515409	PASS
GRPEL1	4	7058906	7068197	-0.358623662	PASS
GRPEL2	5	149345430	149354583	-0.393084436	PASS
GRPR	23	16123556	16153021	0.158844052	PASS
GRSF1	4	70815782	70839945	0.115781418	suppress_segment_too_large
GRTP1	13	113324164	113364148	-0.426822582	PASS
GRWD1	19	48445773	48457022	0.002743503	PASS
GRXCR1	4	42893267	43030658	-0.317525751	PASS
GRXCR2	5	145858521	145937126	-0.393084436	PASS
GSAP	7	77310751	77416400	-0.701511252	PASS
GSC	14	94768216	94770230	-0.155273506	suppress_segment_too_large
GSC2	22	19148576	19150283	-0.740245994	PASS
GSDMA	17	39953263	39977766	0.041294512	PASS
GSDMB	17	39904595	39919854	0.041294512	PASS
GSDMC	8	129748196	129786888	0.493344802	suppress_segment_too_large
GSDMD	8	143553207	143563062	0.699132336	PASS
GSDME	7	24698351	24758113	-0.72421718	PASS
GSE1	16	85169525	85676204	-0.423061015	PASS
GSG1	12	13083560	13103683	0.49032213	suppress_segment_too_large
GSG1L	16	27787535	28063509	0.053595371	PASS
GSG1L2	17	9802386	9822071	0.06854245	PASS
GSK3A	19	42230186	42242625	0.048917043	PASS
GSK3B	3	119821323	120094417	0.06434429	PASS
GSKIP	14	96363452	96387288	-0.155273506	suppress_segment_too_large
GSN	9	121207794	121332843	0.073960965	PASS
GSPT1	16	11868128	11916082	0.053595371	PASS
GSPT2	23	51743431	51746232	0.158844052	PASS
GSR	8	30678061	30727999	-0.311940419	PASS
GSS	20	34928433	34956027	-0.172056771	suppress_segment_too_large
GSTA1	6	52791664	52803910	0.239805577	suppress_segment_too_large
GSTA2	6	52750089	52763569	0.239805577	suppress_segment_too_large
GSTA3	6	52896639	52909685	0.239805577	suppress_segment_too_large
GSTA4	6	52977948	52995378	0.239805577	suppress_segment_too_large
GSTA5	6	52831655	52846095	0.239805577	suppress_segment_too_large
GSTCD	4	105708778	105847728	-0.677982248	PASS
GSTK1	7	143244093	143270854	-0.712149877	PASS
GSTM1	1	109687814	109709039	0.079255572	PASS
GSTM2	1	109668022	109709551	0.079255572	PASS
GSTM3	1	109733932	109741038	0.079255572	PASS
GSTM4	1	109656081	109674836	0.079255572	PASS
GSTM5	1	109711780	109775428	0.079255572	PASS
GSTO1	10	104235356	104267459	-0.414937136	PASS
GSTO2	10	104268873	104304945	-0.414937136	PASS

GSTP1	11	67583595	67586656	0.064535884	PASS
GSTT2B	22	23957414	23961186	-0.740245994	PASS
GSTZ1	14	77320884	77331597	-0.155273506	suppress_segment_too_large
GSX1	13	27792643	27794768	-0.398748891	PASS
GSX2	4	54099523	54102505	0.115781418	suppress_segment_too_large
GTDC1	2	143938068	144332568	-0.30549516	PASS
GTF2A1	14	81175452	81221377	-0.155273506	suppress_segment_too_large
GTF2A1L	2	48617798	48733148	0.034263343	PASS
GTF2A2	15	59638062	59657541	-0.759380635	PASS
GTF2B	1	88852932	88891944	0.079255572	PASS
GTF2E1	3	120742637	120783069	0.06434429	PASS
GTF2E2	8	30578318	30658251	-0.311940419	PASS
GTF2F1	19	6379569	6393981	-0.071973357	PASS
GTF2F2	13	45120515	45284909	-0.398748891	PASS
GTF2H1	11	18322295	18367044	-0.39835217	PASS
GTF2H2	5	71032670	71067689	-0.393084436	PASS
GTF2H2C	5	69560208	69594723	-0.393084436	PASS
GTF2H3	12	123633739	123662606	0.028722739	PASS
GTF2H4	6	30908184	30914106	0.069043418	PASS
GTF2H5	6	158168350	158199344	0.033894706	PASS
GTF2I	7	74657667	74760692	-0.701511252	PASS
GTF2IRD1	7	74453790	74602604	-0.701511252	PASS
GTF2IRD2	7	74796144	74851551	-0.701511252	PASS
GTF2IRD2	7	75092573	75149817	-0.701511252	PASS
GTF3A	13	27424544	27435823	-0.398748891	PASS
GTF3C1	16	27459555	27549913	0.053595371	PASS
GTF3C2	2	27325849	27357034	0.034263343	PASS
GTF3C3	2	196763032	196799725	0.011001208	PASS
GTF3C4	9	132670035	132694955	0.073960965	PASS
GTF3C5	9	133030675	133058503	0.073960965	PASS
GTF3C6	6	110958560	110967890	-0.355603029	PASS
GTPBP1	22	38705723	38738299	-0.740245994	PASS
GTPBP10	7	90335223	90391455	-0.710463272	PASS
GTPBP2	6	43605316	43629162	0.028532667	PASS
GTPBP3	19	17334920	17342735	-0.069222905	PASS
GTPBP4	10	988019	1019936	0.075711544	PASS
GTPBP6	23	304529	318819	-0.350651546	PASS
GTPBP8	3	112990918	113015060	0.06434429	PASS
GTSE1	22	46296741	46330810	-0.953096644	PASS
GTSF1	12	54455950	54473602	-0.743220499	PASS
GTSF1L	20	43726164	43726998	-0.172056771	suppress_segment_too_large
GUCA1A	6	42155406	42180056	0.028532667	PASS
GUCA1B	6	42184401	42194916	0.028532667	PASS
GUCA1C	3	108907792	108953895	0.22470242	PASS
GUCA2A	1	42162691	42164718	-0.736943052	PASS
GUCA2B	1	42153421	42155824	-0.736943052	PASS
GUCD1	22	24540423	24555935	-0.740245994	PASS
GUCY1A1	4	155666711	155732349	0.142431244	suppress_segment_too_large
GUCY1A2	11	106674012	107018524	0.029107301	PASS
GUCY1B1	4	155758992	155807591	0.142431244	suppress_segment_too_large
GUCY2C	12	14612632	14696585	0.49032213	suppress_segment_too_large
GUCY2D	17	8002594	8020339	0.06854245	PASS
GUCY2F	23	109372906	109482072	0.158844052	PASS
GUF1	4	44678427	44700926	-0.317525751	PASS

GUK1	1	228139962	228148984	-0.204996646	suppress_segment_too_large
GULP1	2	188291669	188595931	-0.030005025	PASS
GUSB	7	65960684	65982314	-0.701511252	PASS
GVQW2	6	138725211	138773652	0.033894706	PASS
GVQW3	11	76381313	76414619	0.064535884	PASS
GXYLT1	12	42081845	42144879	-0.46813528	PASS
GXYLT2	3	72888073	72998138	-0.727808231	PASS
GYG1	3	148991341	149027668	0.02267528	PASS
GYG2	23	2828788	2882820	0.158844052	PASS
GYPA	4	144109303	144140786	0.142431244	suppress_segment_too_large
GYPB	4	143996104	144019345	0.142431244	suppress_segment_too_large
GYPC	2	126655933	126696670	-0.359440172	PASS
GYPE	4	143870867	143905563	0.142431244	suppress_segment_too_large
GYS1	19	48968125	48993310	0.002743503	PASS
GYS2	12	21536189	21604847	0.49032213	suppress_segment_too_large
GZF1	20	23362182	23373063	-0.172056771	suppress_segment_too_large
GZMA	5	55102648	55110252	-0.284712224	PASS
GZMB	14	24630954	24634267	0.053252507	PASS
GZMH	14	24606480	24609699	0.053252507	PASS
GZMK	5	55024253	55034570	-0.284712224	PASS
GZMM	19	544034	549924	-0.071973357	PASS
H1F0	22	37805093	37807436	-0.740245994	PASS
H1FNT	12	48328980	48330279	-0.743220499	PASS
H1FOO	3	129543214	129551467	0.06434429	PASS
H1FX	3	129314771	129316277	0.06434429	PASS
H2AFB1	23	154884972	154885558	0.158844052	PASS
H2AFJ	12	14774383	14778002	0.49032213	suppress_segment_too_large
H2AFV	7	44826791	44848083	-0.72421718	PASS
H2AFX	11	119093854	119095467	-0.431650509	PASS
H2AFY	5	135333900	135399914	-0.393084436	PASS
H2AFY2	10	70052796	70112280	0.032351597	PASS
H2AFZ	4	99948086	99950388	-0.677982248	PASS
H2BFM	23	104039949	104042454	0.158844052	PASS
H2BFS	21	5972924	43565648	0.220071762	PASS
H2BFWT	23	104011147	104013687	0.158844052	PASS
H3F3A	1	226061851	226072001	-0.204996646	suppress_segment_too_large
H3F3B	17	75776434	75785893	0.051798665	PASS
H3F3C	12	31791185	31792241	0.49032213	suppress_segment_too_large
H6PD	1	9234775	9271337	0.364796772	PASS
HAAO	2	42767089	42792593	0.034263343	PASS
HABP2	10	113550837	113589602	0.073090211	PASS
HABP4	9	96450201	96491336	0.073960965	PASS
HACD1	10	17589032	17617377	0.075711544	PASS
HACD2	3	123490820	123585185	0.06434429	PASS
HACD3	15	65530418	65578352	-0.759380635	PASS
HACD4	9	20999515	21031636	0.073960965	PASS
HACE1	6	104728093	104859919	-0.355603029	PASS
HACL1	3	15560704	15601852	0.06149513	PASS
HADH	4	107989714	108035175	-0.677982248	PASS
HADHA	2	26190635	26244726	0.034263343	PASS
HADHB	2	26243170	26290468	0.034263343	PASS
HAGH	16	1795620	1827194	0.053595371	PASS
HAGHL	16	726936	735525	0.053595371	PASS
HAL	12	95972662	95996365	0.028722739	PASS

HAMP	19	35280716	35285143	0.044838622	PASS
HAND1	5	154474972	154478264	-0.393084436	PASS
HAND2	4	173524969	173530229	0.142431244	suppress_segment_too_large
HAO1	20	7882981	7940474	-0.172056771	suppress_segment_too_large
HAO2	1	119368779	119394130	0.079255572	PASS
HAP1	17	41717742	41734644	0.041294512	PASS
HAPLN1	5	83637805	83721613	-0.393084436	PASS
HAPLN2	1	156619294	156625725	0.088103245	PASS
HAPLN3	15	88877288	88895626	-0.759380635	PASS
HAPLN4	19	19255641	19262796	-0.069222905	PASS
HARBI1	11	46602861	46617909	0.059795455	PASS
HARS	5	140673904	140691862	-0.393084436	PASS
HARS2	5	140691426	140699318	-0.393084436	PASS
HAS1	19	51713112	51723994	0.002743503	PASS
HAS2	8	121612116	121641390	0.493344802	suppress_segment_too_large
HAS3	16	69105564	69118719	-0.423061015	PASS
HASPIN	17	3723903	3726699	0.06854245	PASS
HAT1	2	171922448	171983682	0.033541467	PASS
HAUS1	18	46104332	46128333	-0.330515409	PASS
HAUS2	15	42548810	42569994	-0.423772929	PASS
HAUS3	4	2227464	2242164	-0.358623662	PASS
HAUS4	14	22946228	22957161	0.053252507	PASS
HAUS5	19	35612744	35625349	0.044838622	PASS
HAUS6	9	19053143	19103119	0.073960965	PASS
HAUS7	23	153447666	153495516	0.158844052	PASS
HAUS8	19	17049729	17075625	-0.069222905	PASS
HAVCR1	5	157029413	157059119	-0.393084436	PASS
HAVCR2	5	157085832	157142869	-0.393084436	PASS
HAX1	1	154272511	154275875	0.088103245	PASS
HBA1	16	176680	177522	0.053595371	PASS
HBA2	16	172876	173710	0.053595371	PASS
HBB	11	5225464	5229395	-0.39835217	PASS
HBD	11	5232678	5243657	-0.39835217	PASS
HBE1	11	5268345	5505652	-0.39835217	PASS
HBEGF	5	140332843	140346631	-0.393084436	PASS
HBG1	11	5248079	5249859	-0.39835217	PASS
HBG2	11	5253190	5505605	-0.39835217	PASS
HBM	16	153892	166768	0.053595371	PASS
HBP1	7	107168961	107202529	-0.712149877	PASS
HBQ1	16	180453	181181	0.053595371	PASS
HBS1L	6	134960378	135103056	0.033894706	PASS
HBZ	16	152687	154503	0.053595371	PASS
HCAR1	12	122726076	122730843	0.028722739	PASS
HCAR2	12	122701293	122703343	0.028722739	PASS
HCAR3	12	122714756	122716892	0.028722739	PASS
HCCS	23	11111301	11123078	0.158844052	PASS
HCFC1	23	153947553	153971807	0.158844052	PASS
HCFC1R1	16	3022620	3024286	0.053595371	PASS
HCFC2	12	104064457	104106524	0.028722739	PASS
HCK	20	32052188	32101856	-0.172056771	suppress_segment_too_large
HCLS1	3	121631399	121660927	0.06434429	PASS
HCN1	5	45254950	45696498	-0.284712224	PASS
HCN2	19	589893	617159	-0.071973357	PASS
HCN3	1	155277583	155289848	0.088103245	PASS

HCN4	15	73319859	73369264	-0.759380635	PASS
HCRT	17	42184060	42185452	0.041294512	PASS
HCRTR1	1	31617686	31632518	0.048749694	PASS
HCRTR2	6	55106460	55282620	0.239805577	suppress_segment_too_large
HCST	19	35902480	35904377	0.044838622	PASS
HDAC1	1	32292086	32333635	0.048749694	PASS
HDAC10	22	50245183	50251405	-0.953096644	PASS
HDAC11	3	13479724	13506424	0.06149513	PASS
HDAC2	6	113933028	114011308	-0.355603029	PASS
HDAC3	5	141620876	141636870	-0.393084436	PASS
HDAC4	2	239048168	239401654	0.011001208	PASS
HDAC5	17	44076746	44123702	0.051798665	PASS
HDAC6	23	48801377	48824982	0.158844052	PASS
HDAC7	12	47782722	47833132	-0.743220499	PASS
HDAC8	23	72329516	72573199	0.158844052	PASS
HDAC9	7	18086949	19002416	-0.72421718	PASS
HDC	15	50241947	50266026	-0.423772929	PASS
HDDC2	6	125219962	125302078	-0.304168115	PASS
HDDC3	15	90929964	90935196	-0.759380635	PASS
HDGF	1	156742107	156766925	0.088103245	PASS
HDGFL1	6	22569493	22571666	0.069043418	PASS
HDGFL2	19	4472287	4502211	-0.071973357	PASS
HDGFL3	15	83112738	83208018	-0.759380635	PASS
HDHD2	18	47107403	47150520	-0.330515409	PASS
HDHD3	9	113373419	113376999	0.073960965	PASS
HDHD5	22	17137511	17165287	-0.740245994	PASS
HDLBP	2	241227264	241317061	0.011001208	PASS
HDX	23	84317874	84502479	0.158844052	PASS
HEATR1	1	236549005	236604504	-0.204996646	suppress_segment_too_large
HEATR3	16	50065941	50106387	0.411512417	PASS
HEATR4	14	73478484	73558947	0.053252507	PASS
HEATR5A	14	31291791	31420582	0.053252507	PASS
HEATR5B	2	36968383	37084342	0.034263343	PASS
HEATR6	17	60043194	60078931	0.051798665	PASS
HEATR9	17	35854946	35868891	0.041294512	PASS
HEBP1	12	12974864	13000273	0.49032213	suppress_segment_too_large
HEBP2	6	138403531	138422197	0.033894706	PASS
HECA	6	139135112	139180802	0.033894706	PASS
HECTD1	14	31100112	31207804	0.053252507	PASS
HECTD2	10	91409280	91514829	-0.414937136	PASS
HECTD3	1	45002540	45011329	-0.736943052	PASS
HECTD4	12	112160188	112382439	0.028722739	PASS
HECW1	7	43112599	43566001	-0.72421718	PASS
HECW2	2	196189099	196593692	0.011001208	PASS
HEG1	3	124965710	125055958	0.06434429	PASS
HELB	12	66302545	66347645	-0.743220499	PASS
HELLS	10	94501434	94613905	-0.414937136	PASS
HELQ	4	83407343	83455856	-0.677982248	PASS
HELT	4	185018841	185020804	0.275137421	PASS
HELZ	17	67070438	67245989	0.051798665	PASS
HELZ2	20	63558086	63574239	-0.172056771	suppress_segment_too_large
HEMGN	9	97926791	97944856	0.073960965	PASS
HEMK1	3	50569152	50596168	-0.727808231	PASS
HENMT1	1	108648290	108661526	0.079255572	PASS

HEPACAM	11	124919193	124936412	-0.431650509	PASS
HEPACAM	7	93188586	93226524	-0.710463272	PASS
HEPH	23	66162549	66268867	0.158844052	PASS
HEPHL1	11	94021361	94113751	0.064535884	PASS
HEPN1	11	124919244	124920677	-0.431650509	PASS
HERC1	15	63608618	63833942	-0.759380635	PASS
HERC2	15	28111040	28322152	-0.423772929	PASS
HERC3	4	88592423	88708542	-0.677982248	PASS
HERC4	10	67921899	68075348	0.032351597	PASS
HERC5	4	88457117	88506163	-0.677982248	PASS
HERC6	4	88378739	88443111	-0.677982248	PASS
HERPUD1	16	56932048	56944863	-0.423061015	PASS
HERPUD2	7	35632659	35695571	-0.72421718	PASS
HES1	3	194136145	194138732	0.02267528	PASS
HES2	1	6412418	6424670	0.364796772	PASS
HES3	1	6244192	6245578	0.364796772	PASS
HES4	1	998962	1000172	-0.358281697	PASS
HES5	1	2528745	2530245	-0.358281697	PASS
HES6	2	238238267	238240662	0.011001208	PASS
HES7	17	8120590	8124092	0.06854245	PASS
HESX1	3	57197838	57227606	-0.727808231	PASS
HEXA	15	72340919	72376476	-0.759380635	PASS
HEXB	5	74640023	74722647	-0.393084436	PASS
HEXDC	17	82418318	82442645	0.051798665	PASS
HEXIM1	17	45148502	45152101	0.051798665	PASS
HEXIM2	17	45160700	45170040	0.051798665	PASS
HEY1	8	79764010	79767863	0.617295349	suppress_segment_too_large
HEY2	6	125747664	125761269	-0.304168115	PASS
HEYL	1	39624153	39639945	0.048749694	PASS
HFE	6	26087281	26098343	0.069043418	PASS
HFM1	1	91260766	91404869	0.079255572	PASS
HGD	3	120628173	120682571	0.06434429	PASS
HGF	7	81699008	81770438	-0.701511252	PASS
HGFAC	4	3441887	3449495	-0.358623662	PASS
HGH1	8	144137769	144140843	0.699132336	PASS
HGS	17	81683326	81703138	0.051798665	PASS
HGSNAT	8	43140455	43202855	0.182873287	suppress_segment_too_large
HHAT	1	210328252	210676296	-0.204996646	suppress_segment_too_large
HHATL	3	42692663	42702827	-0.727808231	PASS
HHEX	10	92689951	92695646	-0.414937136	PASS
HHIP	4	144646021	144745271	0.142431244	suppress_segment_too_large
HHIPL1	14	99645110	99680569	-0.155273506	suppress_segment_too_large
HHIPL2	1	222522258	222548103	-0.204996646	suppress_segment_too_large
HHLA1	8	132061486	132111159	0.493344802	suppress_segment_too_large
HHLA2	3	108296490	108378285	0.22470242	PASS
HHLA3	1	70354805	70385339	0.052232535	PASS
HIBADH	7	27525442	27662995	-0.72421718	PASS
HIBCH	2	190189735	190344193	0.011001208	PASS
HIC1	17	2054154	2063241	0.06854245	PASS
HIC2	22	21417404	21451463	-0.740245994	PASS
HID1	17	74950743	74973166	0.051798665	PASS
HIF1A	14	61695513	61748259	0.053252507	PASS
HIF1AN	10	100529072	100559998	-0.414937136	PASS
HIF3A	19	46297046	46343433	0.002743503	PASS

HIGD1A	3	42784298	42804531	-0.727808231	PASS
HIGD1B	17	44846353	44850480	0.051798665	PASS
HIGD1C	12	50953922	50970506	-0.743220499	PASS
HIGD2A	5	176388747	176389771	0.165341871	suppress_segment_too_large
HIGD2B	15	72675782	72686149	-0.759380635	PASS
HIKESHI	11	86302211	86345931	0.064535884	PASS
HILPDA	7	128455849	128458418	-0.712149877	PASS
HINFP	11	119121587	119136044	-0.431650509	PASS
HINT1	5	131159027	131171735	-0.393084436	PASS
HINT2	9	35812960	35815354	0.073960965	PASS
HINT3	6	125956781	125980244	-0.304168115	PASS
HIP1	7	75533300	75738962	-0.701511252	PASS
HIP1R	12	122834453	122862960	0.028722739	PASS
HIPK1	1	113929192	113977869	0.079255572	PASS
HIPK2	7	139561570	139777778	-0.712149877	PASS
HIPK3	11	33256672	33357023	-0.359426047	PASS
HIPK4	19	40379271	40390187	0.048917043	PASS
HIRA	22	19330698	19447450	-0.740245994	PASS
HIRIP3	16	29992321	29996436	0.053595371	PASS
HIST1H1A	6	26017085	26017732	0.069043418	PASS
HIST1H1B	6	27866849	27867529	0.069043418	PASS
HIST1H1C	6	26055787	26056428	0.069043418	PASS
HIST1H1D	6	26234268	26234933	0.069043418	PASS
HIST1H1E	6	26156354	26157107	0.069043418	PASS
HIST1H1T	6	26107419	26108136	0.069043418	PASS
HIST1H2A	6	26033176	26033568	0.069043418	PASS
HIST1H2A	6	26124145	26139116	0.069043418	PASS
HIST1H2A	6	26198851	26199243	0.069043418	PASS
HIST1H2A	6	26216975	26217483	0.069043418	PASS
HIST1H2A	6	27133042	27135291	0.069043418	PASS
HIST1H2A	6	27147129	27147515	0.069043418	PASS
HIST1H2A	6	27808199	27808701	0.069043418	PASS
HIST1H2A	6	27814354	27814740	0.069043418	PASS
HIST1H2A	6	27837947	27838339	0.069043418	PASS
HIST1H2A	6	27865355	27865747	0.069043418	PASS
HIST1H2A	6	27892757	27893149	0.069043418	PASS
HIST1H2B	6	25726777	25727292	0.069043418	PASS
HIST1H2B	6	26043277	26043657	0.069043418	PASS
HIST1H2B	6	26114873	26123926	0.069043418	PASS
HIST1H2B	6	26158146	26171349	0.069043418	PASS
HIST1H2B	6	26172059	26184655	0.069043418	PASS
HIST1H2B	6	26199520	26200715	0.069043418	PASS
HIST1H2B	6	26215159	26216692	0.069043418	PASS
HIST1H2B	6	26251651	26253710	0.069043418	PASS
HIST1H2B	6	26272976	26273356	0.069043418	PASS
HIST1H2B	6	27125897	27132750	0.069043418	PASS
HIST1H2B	6	27146418	27146798	0.069043418	PASS
HIST1H2B	6	27807444	27807931	0.069043418	PASS
HIST1H2B	6	27815044	27815424	0.069043418	PASS
HIST1H2B	6	27837760	27865798	0.069043418	PASS
HIST1H2B	6	27893463	27893843	0.069043418	PASS
HIST1H3A	6	26020490	26020900	0.069043418	PASS
HIST1H3B	6	26031650	26032060	0.069043418	PASS
HIST1H3C	6	26045411	26045821	0.069043418	PASS

HIST1H3D	6	26196840	26197250	0.069043418	PASS
HIST1H3E	6	26224199	26227473	0.069043418	PASS
HIST1H3F	6	26250195	26250605	0.069043418	PASS
HIST1H3G	6	26269405	26271815	0.069043418	PASS
HIST1H3H	6	27810064	27811300	0.069043418	PASS
HIST1H3I	6	27871905	27872315	0.069043418	PASS
HIST1H3J	6	27890382	27893106	0.069043418	PASS
HIST1H4A	6	26021679	26021990	0.069043418	PASS
HIST1H4B	6	26026815	26027252	0.069043418	PASS
HIST1H4C	6	26103876	26104310	0.069043418	PASS
HIST1H4D	6	26188765	26189076	0.069043418	PASS
HIST1H4E	6	26204552	26206038	0.069043418	PASS
HIST1H4F	6	26240426	26240737	0.069043418	PASS
HIST1H4G	6	26246681	26246977	0.069043418	PASS
HIST1H4H	6	26277609	26285638	0.069043418	PASS
HIST1H4I	6	27138588	27139881	0.069043418	PASS
HIST1H4J	6	27824108	27824480	0.069043418	PASS
HIST1H4K	6	27831216	27831527	0.069043418	PASS
HIST1H4L	6	27873199	27873510	0.069043418	PASS
HIST2H2A	1	149887524	149887916	0.079255572	PASS
HIST2H2A	1	149886975	149887364	0.079255572	PASS
HIST2H2B	1	149884459	149886652	0.079255572	PASS
HIST2H2B	1	149782689	149812373	0.079255572	PASS
HIST2H3D	1	149813271	149813681	0.079255572	PASS
HIST2H3P	1	143905556	143905966	0.079255572	PASS
HIST3H2A	1	228456979	228457873	-0.204996646	suppress_segment_too_large
HIST3H2B	1	228458107	228460470	-0.204996646	suppress_segment_too_large
HIST3H3	1	228424845	228425325	-0.204996646	suppress_segment_too_large
HIST4H4	12	14767999	14771131	0.49032213	suppress_segment_too_large
HIVEP1	6	12008762	12164999	0.069043418	PASS
HIVEP2	6	142751467	142956698	0.033894706	PASS
HIVEP3	1	41506365	42035925	-0.736943052	PASS
HJURP	2	233833416	233854566	0.011001208	PASS
HJV	1	146017468	146036746	0.079255572	PASS
HK1	10	69269984	69401884	0.032351597	PASS
HK2	2	74833981	74893359	-0.46122572	PASS
HK3	5	176880869	176899332	0.165341871	suppress_segment_too_large
HKDC1	10	69220303	69267559	0.032351597	PASS
HKR1	19	37312837	37369365	0.396324882	PASS
HLA-A	6	29941260	29945884	0.069043418	PASS
HLA-B	6	31269491	31357188	0.069043418	PASS
HLA-C	6	31268749	31272130	0.069043418	PASS
HLA-DMA	6	32948613	32969094	0.028532667	PASS
HLA-DME	6	32934629	32941070	0.028532667	PASS
HLA-DOA	6	33004178	33009612	0.028532667	PASS
HLA-DOB	6	32812763	32820466	0.028532667	PASS
HLA-DPA	6	33064569	33080775	0.028532667	PASS
HLA-DPB	6	33075926	33087201	0.028532667	PASS
HLA-DQA	6	32628179	32647062	0.028532667	PASS
HLA-DQA	6	32741342	32747215	0.028532667	PASS
HLA-DQB	6	32659467	32668383	0.028532667	PASS
HLA-DQB	6	32756098	32763534	0.028532667	PASS
HLA-DRA	6	32439842	32445046	0.028532667	PASS
HLA-DRB	6	32578769	32589848	0.028532667	PASS

HLA-DRB	6	32517343	32530287	0.028532667	PASS
HLA-E	6	30489467	30494205	0.069043418	PASS
HLA-F	6	29722775	29738528	0.069043418	PASS
HLA-G	6	29826967	29831125	0.069043418	PASS
HLCS	21	36750888	36990236	0.209983559	PASS
HLF	17	55265012	55325065	0.051798665	PASS
HLTF	3	149030127	149086554	0.02267528	PASS
HLX	1	220879400	220885059	-0.204996646	suppress_segment_too_large
HM13	20	31514410	31577923	-0.172056771	suppress_segment_too_large
HMBOX1	8	28890394	29064764	-0.311940419	PASS
HMBS	11	119084866	119093549	-0.431650509	PASS
HMCES	3	129278828	129306186	0.06434429	PASS
HMCN1	1	185734551	186190949	0.088103245	PASS
HMCN2	9	130265882	130434123	0.073960965	PASS
HMG20A	15	77420412	77485607	-0.759380635	PASS
HMG20B	19	3572777	3579088	-0.071973357	PASS
HMGA1	6	34236873	34246231	0.028532667	PASS
HMGA2	12	65824131	65966295	-0.743220499	PASS
HMGB1	13	30456704	30617597	-0.398748891	PASS
HMGB2	4	173331695	173335125	0.142431244	suppress_segment_too_large
HMGB3	23	150980509	150990775	0.158844052	PASS
HMGB4	1	33860475	33864791	0.048749694	PASS
HMGCL	1	23801885	23838620	0.048749694	PASS
HMGCLL1	6	55434369	55579214	0.239805577	suppress_segment_too_large
HMGCR	5	75336329	75362104	-0.393084436	PASS
HMGCS1	5	43289395	43313512	-0.284712224	PASS
HMGCS2	1	119747996	119768905	0.079255572	PASS
HMGN1	21	39342315	39349647	0.209983559	PASS
HMGN2	1	26472450	26475972	0.048749694	PASS
HMGN3	6	79201245	79234738	-0.250122709	PASS
HMGN4	6	26538405	26546254	0.069043418	PASS
HMGN5	23	81113701	81201942	0.158844052	PASS
HMGXB3	5	150000046	150053142	-0.393084436	PASS
HMGXB4	22	35257452	35295807	-0.740245994	PASS
HMHB1	5	143812161	143820719	-0.393084436	PASS
HMMR	5	163460203	163491945	0.165341871	suppress_segment_too_large
HMOX1	22	35380361	35394214	-0.740245994	PASS
HMOX2	16	4474690	4510347	0.053595371	PASS
HMSD	18	63949301	63981774	-0.330515409	PASS
HMX1	4	8846076	8871817	-0.358623662	PASS
HMX2	10	123148122	123150672	0.073090211	PASS
HMX3	10	123135962	123137741	0.073090211	PASS
HNF1A	12	120978543	121002512	0.028722739	PASS
HNF1B	17	37686432	37745247	0.041294512	PASS
HNF4A	20	44355700	44434596	-0.172056771	suppress_segment_too_large
HNF4G	8	75407914	75566843	0.182873287	suppress_segment_too_large
HNMT	2	137964020	138016364	-0.359440172	PASS
HNRNPA0	5	137745651	137754376	-0.393084436	PASS
HNRNPA1	12	54280193	54287088	-0.743220499	PASS
HNRNPA1	13	52642425	52643796	-0.384313056	PASS
HNRNPA1	16	51553436	51647132	-0.423061015	PASS
HNRNPA2	7	26173057	26201529	-0.72421718	PASS
HNRNPA3	2	177212563	177223958	0.033541467	PASS
HNRNPAB	5	178204507	178211163	0.165341871	suppress_segment_too_large

HNRNPC	14	21209136	21269494	0.053252507	PASS
HNRNPCI	1	12847408	12848725	0.048749694	PASS
HNRNPCI	1	13115496	13116854	0.048749694	PASS
HNRNPCI	1	13060869	13062229	0.048749694	PASS
HNRNPD	4	82352498	82374503	-0.677982248	PASS
HNRNPDI	4	82422564	82430408	-0.677982248	PASS
HNRNPF	10	43385617	43409166	-0.389033546	PASS
HNRNPH1	5	179614178	179634784	0.165341871	suppress_segment_too_large
HNRNPH2	23	101408295	101414133	0.158844052	PASS
HNRNPH3	10	68331174	68343191	0.032351597	PASS
HNRNPK	9	83968083	83980616	0.073960965	PASS
HNRNPL	19	38836388	38852347	0.048917043	PASS
HNRNPLI	2	38561978	38603586	0.034263343	PASS
HNRNPM	19	8444767	8489114	-0.071973357	PASS
HNRNPR	1	23303771	23344336	0.048749694	PASS
HNRNPU	1	244840638	244864560	-0.145670469	suppress_likely_unfocal_large_gain
HNRNPUI	19	41262496	41307598	0.048917043	PASS
HNRNPUI	11	62712630	62727472	0.064535884	PASS
HNRNPUI	11	62690275	62727384	0.064535884	PASS
HOGA1	10	97584323	97612802	-0.414937136	PASS
HOMER1	5	79372636	79514217	-0.393084436	PASS
HOMER2	15	82836946	82986153	-0.759380635	PASS
HOMER3	19	18929201	18941261	-0.069222905	PASS
HOMEZ	14	23272422	23299447	0.053252507	PASS
HOOK1	1	59814786	59876378	-0.736943052	PASS
HOOK2	19	12763003	12872740	-0.069222905	PASS
HOOK3	8	42896932	43030539	0.182873287	suppress_segment_too_large
HOPX	4	56647988	56681899	0.115781418	suppress_segment_too_large
HORMAD	1	150698060	150720888	0.079255572	PASS
HORMAD	22	30080174	30177075	-0.740245994	PASS
HOXA1	7	27092993	27095996	-0.72421718	PASS
HOXA10	7	27170591	27180261	-0.72421718	PASS
HOXA11	7	27181510	27185223	-0.72421718	PASS
HOXA13	7	27193503	27200106	-0.72421718	PASS
HOXA2	7	27100354	27102811	-0.72421718	PASS
HOXA3	7	27106184	27152581	-0.72421718	PASS
HOXA4	7	27128507	27130799	-0.72421718	PASS
HOXA5	7	27141052	27143668	-0.72421718	PASS
HOXA6	7	27145396	27150603	-0.72421718	PASS
HOXA7	7	27153716	27157936	-0.72421718	PASS
HOXA9	7	27162435	27175180	-0.72421718	PASS
HOXB1	17	48528526	48531001	0.051798665	PASS
HOXB13	17	48724763	48729178	0.051798665	PASS
HOXB2	17	48540894	48544989	0.051798665	PASS
HOXB3	17	48548870	48604912	0.051798665	PASS
HOXB4	17	48575513	48580111	0.051798665	PASS
HOXB5	17	48591257	48593961	0.051798665	PASS
HOXB6	17	48595751	48604992	0.051798665	PASS
HOXB7	17	48607227	48633572	0.051798665	PASS
HOXB8	17	48611377	48614939	0.051798665	PASS
HOXB9	17	48621159	48626356	0.051798665	PASS
HOXC10	12	53985065	53990279	-0.743220499	PASS
HOXC11	12	53973126	53977643	-0.743220499	PASS
HOXC12	12	53954834	53958956	-0.743220499	PASS

HOXC13	12	53938765	53946544	-0.743220499	PASS
HOXC4	12	54016931	54056030	-0.743220499	PASS
HOXC5	12	54032853	54035358	-0.743220499	PASS
HOXC6	12	53990624	54030823	-0.743220499	PASS
HOXC8	12	54009106	54012362	-0.743220499	PASS
HOXC9	12	53994895	54003337	-0.743220499	PASS
HOXD1	2	176188579	176190907	0.033541467	PASS
HOXD10	2	176108790	176119942	0.033541467	PASS
HOXD11	2	176104216	176109754	0.033541467	PASS
HOXD12	2	176099730	176101193	0.033541467	PASS
HOXD13	2	176092891	176095938	0.033541467	PASS
HOXD3	2	176136612	176173102	0.033541467	PASS
HOXD4	2	176151222	176153226	0.033541467	PASS
HOXD8	2	176129694	176132695	0.033541467	PASS
HOXD9	2	176122720	176124937	0.033541467	PASS
HP	16	72054592	72061055	-0.423061015	PASS
HP1BP3	1	20742661	20787323	0.048749694	PASS
HPCA	1	32885994	32898441	0.048749694	PASS
HPCAL1	2	10302889	10427617	0.034263343	PASS
HPCAL4	1	39678648	39691689	0.048749694	PASS
HPD	12	121839527	121863596	0.028722739	PASS
HPDL	1	45326905	45328533	-0.736943052	PASS
HPF1	4	169729465	169757953	0.142431244	suppress_segment_too_large
HPGD	4	174490177	174523154	0.142431244	suppress_segment_too_large
HPGDS	4	94298535	94342876	-0.677982248	PASS
HPN	19	35040506	35066571	0.044838622	PASS
HPR	16	72063148	72077246	-0.423061015	PASS
HPRT1	23	134460153	134520513	0.158844052	PASS
HPS1	10	98416198	98446947	-0.414937136	PASS
HPS3	3	149129584	149173732	0.02267528	PASS
HPS4	22	26443423	26483837	-0.740245994	PASS
HPS5	11	18278668	18322198	-0.39835217	PASS
HPS6	10	102065390	102068038	-0.414937136	PASS
HPSE	4	83292461	83335153	-0.677982248	PASS
HPSE2	10	98457077	99235862	-0.414937136	PASS
HPX	11	6431049	6442617	-0.39835217	PASS
HR	8	22114415	22133384	-0.311940419	PASS
HRAS	11	532242	537287	-0.39835217	PASS
HRASLS	3	193241125	193277738	0.02267528	PASS
HRASLS2	11	63552770	63563383	0.064535884	PASS
HRASLS5	11	63461404	63491194	0.064535884	PASS
HRC	19	49151198	49155424	0.002743503	PASS
HRCT1	9	35906192	35907141	0.073960965	PASS
HRG	3	186660216	186678240	0.02267528	PASS
HRH1	3	11137093	11263557	0.06149513	PASS
HRH2	5	175658030	175710756	0.165341871	suppress_segment_too_large
HRH3	20	62214970	62220267	-0.172056771	suppress_segment_too_large
HRH4	18	24460629	24479957	-0.330515409	PASS
HRNR	1	152212082	152224193	0.546958914	PASS
HS1BP3	2	20560448	20651089	0.034263343	PASS
HS2ST1	1	86914648	87109998	0.079255572	PASS
HS3ST1	4	11393150	11429765	-0.358623662	PASS
HS3ST2	16	22814177	22916338	0.053595371	PASS
HS3ST3A1	17	13495689	13601927	0.06854245	PASS

HS3ST3B1	17	14301083	14349404	0.06854245	PASS
HS3ST4	16	25692026	26137688	0.053595371	PASS
HS3ST5	6	114055586	114343045	-0.355603029	PASS
HS3ST6	16	1911463	1918440	0.053595371	PASS
HS6ST1	2	128236716	128318577	-0.359440172	PASS
HS6ST2	23	132626016	132961395	0.158844052	PASS
HS6ST3	13	96090839	96839562	-0.384313056	PASS
HSBP1	16	83807843	83819737	-0.423061015	PASS
HSBP1L1	18	79964561	79970822	-0.330515409	PASS
HSCB	22	28742031	28757515	-0.740245994	PASS
HSD11B1	1	209686178	209734950	-0.204996646	suppress_segment_too_large
HSD11B1I	19	5680604	5688523	-0.071973357	PASS
HSD11B2	16	67430652	67437553	-0.423061015	PASS
HSD17B1	17	42549214	42555213	0.041294512	PASS
HSD17B1(	23	53431258	53434373	0.158844052	PASS
HSD17B11	4	87336610	87391386	-0.677982248	PASS
HSD17B12	11	43680558	43856617	0.059795455	PASS
HSD17B13	4	87303789	87322906	-0.677982248	PASS
HSD17B14	19	48813017	48836678	0.002743503	PASS
HSD17B2	16	82035004	82098534	-0.423061015	PASS
HSD17B3	9	96235306	96302176	0.073960965	PASS
HSD17B4	5	119452443	119637199	-0.393084436	PASS
HSD17B6	12	56752161	56787790	-0.743220499	PASS
HSD17B7	1	162790702	162812817	0.088103245	PASS
HSD17B8	6	33204642	33206831	0.028532667	PASS
HSD3B1	1	119507198	119515054	0.079255572	PASS
HSD3B2	1	119414931	119423035	0.079255572	PASS
HSD3B7	16	30985207	30989152	0.053595371	PASS
HSDL1	16	84122146	84145192	-0.423061015	PASS
HSDL2	9	112379937	112472410	0.073960965	PASS
HSF1	8	144291591	144314722	0.699132336	PASS
HSF2	6	122399546	122433119	-0.355603029	PASS
HSF2BP	21	43529192	43659493	0.209983559	PASS
HSF4	16	67164681	67169945	-0.423061015	PASS
HSF5	17	58420167	58488384	0.051798665	PASS
HSFX1	23	149774068	149776867	0.158844052	PASS
HSFX3	23	149548210	149549932	0.158844052	PASS
HSFX4	23	149929645	149931287	0.158844052	PASS
HSH2D	19	16134028	16158575	-0.069222905	PASS
HSP90AA1	14	102080738	102139699	-0.155273506	suppress_segment_too_large
HSP90AB1	6	44246166	44253888	0.028532667	PASS
HSP90B1	12	103930107	103953645	0.028722739	PASS
HSPA12A	10	116671192	116850236	0.073090211	PASS
HSPA12B	20	3732667	3753111	-0.172056771	suppress_segment_too_large
HSPA13	21	14371115	14383484	0.758930684	suppress_segment_too_large
HSPA14	10	14838160	14871741	0.075711544	PASS
HSPA1A	6	31815464	31817946	0.028532667	PASS
HSPA1B	6	31827735	31830255	0.028532667	PASS
HSPA1L	6	31809619	31815065	0.028532667	PASS
HSPA2	14	64535905	64546173	0.053252507	PASS
HSPA4	5	133051962	133106449	-0.393084436	PASS
HSPA4L	4	127781821	127840733	0.142431244	suppress_segment_too_large
HSPA5	9	125234853	125241330	0.073960965	PASS
HSPA6	1	161524540	161526910	0.088103245	PASS

HSPA8	11	123057489	123063230	-0.431650509	PASS
HSPA9	5	138553756	138575724	-0.393084436	PASS
HSPB1	7	76302544	76304295	-0.701511252	PASS
HSPB11	1	53916574	53945929	-0.736943052	PASS
HSPB2	11	111912242	111914093	-0.431650509	PASS
HSPB2-C1	11	111912736	111926871	-0.431650509	PASS
HSPB3	5	54455601	54456384	-0.284712224	PASS
HSPB6	19	35754569	35758079	0.044838622	PASS
HSPB7	1	16014028	16019594	0.048749694	PASS
HSPB8	12	119178642	119221131	0.028722739	PASS
HSPB9	17	42121431	42123352	0.041294512	PASS
HSPBAP1	3	122740003	122793824	0.06434429	PASS
HSPBP1	19	55262231	55280381	0.142581352	PASS
HSPD1	2	197486581	197516737	0.011001208	PASS
HSPE1	2	197499994	197503457	0.011001208	PASS
HSPE1-M(	2	197500413	197550726	0.011001208	PASS
HSPG2	1	21822245	21937297	0.048749694	PASS
HSPH1	13	31134974	31162388	-0.398748891	PASS
HTATIP2	11	20363685	20383783	-0.39835217	PASS
HTATSF1	23	136497079	136512346	0.158844052	PASS
HTD2	3	58306262	58318574	-0.727808231	PASS
HTN1	4	70050402	70058845	0.115781418	suppress_segment_too_large
HTN3	4	70028413	70036538	0.115781418	suppress_segment_too_large
HTR1A	5	63960356	63962507	-0.393084436	PASS
HTR1B	6	77461753	77463773	-0.250122709	PASS
HTR1D	1	23191895	23194729	0.048749694	PASS
HTR1E	6	86937306	87016683	-0.355603029	PASS
HTR1F	3	87990696	87993835	0.22470242	PASS
HTR2A	13	46831550	46897076	-0.398748891	PASS
HTR2B	2	231108230	231125118	0.011001208	PASS
HTR2C	23	114584078	114910061	0.158844052	PASS
HTR3A	11	113974881	113990313	-0.431650509	PASS
HTR3B	11	113904677	113946565	-0.431650509	PASS
HTR3C	3	184053047	184060671	0.02267528	PASS
HTR3D	3	184031544	184039369	0.02267528	PASS
HTR3E	3	184097064	184106995	0.02267528	PASS
HTR4	5	148451032	148677235	-0.393084436	PASS
HTR5A	7	155070324	155087392	-0.77630473	PASS
HTR6	1	19665287	19679562	0.048749694	PASS
HTR7	10	90740823	90857698	-0.414937136	PASS
HTRA1	10	122458551	122514908	0.073090211	PASS
HTRA2	2	74529377	74533348	-0.46122572	PASS
HTRA3	4	8269765	8307111	-0.358623662	PASS
HTRA4	8	38974164	38988662	0.182873287	suppress_segment_too_large
HTT	4	3041422	3243960	-0.358623662	PASS
HUNK	21	31873315	32044633	0.209983559	PASS
HUS1	7	47963288	47979553	-0.72421718	PASS
HUS1B	6	655939	656963	0.069043418	PASS
HUWE1	23	53532096	53686729	0.158844052	PASS
HVCN1	12	110627841	110704950	0.028722739	PASS
HYAL1	3	50299889	50312381	-0.727808231	PASS
HYAL2	3	50317790	50322906	-0.727808231	PASS
HYAL3	3	50292831	50299468	-0.727808231	PASS
HYAL4	7	123828983	123877478	-0.712149877	PASS

HYDIN	16	70807378	71230722	-0.423061015	PASS
HYI	1	43451003	43453989	-0.736943052	PASS
HYKK	15	78507564	78537372	-0.759380635	PASS
HYLS1	11	125883614	125900648	-0.431650509	PASS
HYOU1	11	119044189	119057202	-0.431650509	PASS
HYPK	15	43796142	43803043	-0.423772929	PASS
HYPM	23	37990817	37991317	0.158844052	PASS
IAH1	2	9473658	9496543	0.034263343	PASS
IAPP	12	21354959	21379978	0.49032213	suppress_segment_too_large
IARS	9	92210207	92293756	0.073960965	PASS
IARS2	1	220094102	220148041	-0.204996646	suppress_segment_too_large
IBA57	1	228165815	228182257	-0.204996646	suppress_segment_too_large
IBSP	4	87799581	87812435	-0.677982248	PASS
IBTK	6	82169983	82247754	-0.250122709	PASS
ICA1	7	8113184	8262687	-0.72421718	PASS
ICA1L	2	202773150	202871985	0.011001208	PASS
ICAM1	19	10270835	10286615	-0.107594294	PASS
ICAM2	17	64002594	64020634	0.051798665	PASS
ICAM3	19	10333776	10339823	-0.107594294	PASS
ICAM4	19	10286967	10288522	-0.107594294	PASS
ICAM5	19	10289981	10296778	-0.107594294	PASS
ICE1	5	5420664	5490234	0.073527871	PASS
ICE2	15	60419609	60479160	-0.759380635	PASS
ICK	6	53001279	53061802	0.239805577	suppress_segment_too_large
ICMT	1	6221193	6235972	0.364796772	PASS
ICOS	2	203936748	203961577	0.011001208	PASS
ID1	20	31605283	31606515	-0.172056771	suppress_segment_too_large
ID2	2	8678845	8684453	0.034263343	PASS
ID3	1	23557918	23559794	0.048749694	PASS
ID4	6	19837386	19840684	0.069043418	PASS
IDE	10	92451684	92574076	-0.414937136	PASS
IDH1	2	208236227	208266074	0.011001208	PASS
IDH2	15	90083045	90102504	-0.759380635	PASS
IDH3A	15	78131498	78171949	-0.759380635	PASS
IDH3B	20	2658395	2664219	-0.172056771	suppress_segment_too_large
IDH3G	23	153785766	153794523	0.158844052	PASS
IDI1	10	1039152	1049170	0.075711544	PASS
IDI2	10	1018907	1025859	0.075711544	PASS
IDNK	9	83623049	83644130	0.073960965	PASS
IDO1	8	39902275	39928444	0.182873287	suppress_segment_too_large
IDO2	8	39934614	40016391	0.182873287	suppress_segment_too_large
IDS	23	149476990	149521096	0.158844052	PASS
IDUA	4	986997	1004506	-0.358623662	PASS
IER2	19	13150415	13154908	-0.069222905	PASS
IER3	6	30743199	30744554	0.069043418	PASS
IER3IP1	18	47152834	47176374	-0.330515409	PASS
IER5	1	181088712	181092899	0.088103245	PASS
IER5L	9	129175552	129178262	0.073960965	PASS
IFFO1	12	6538375	6556083	-0.015029783	PASS
IFFO2	1	18904281	18956686	0.048749694	PASS
IFI16	1	158999968	159055155	0.088103245	PASS
IFI27	14	94104836	94116698	-0.155273506	suppress_segment_too_large
IFI27L1	14	94081282	94103846	-0.155273506	suppress_segment_too_large
IFI27L2	14	94127779	94130253	-0.155273506	suppress_segment_too_large

IFI30	19	18173162	18178117	-0.069222905	PASS
IFI35	17	43006725	43014456	0.041294512	PASS
IFI44	1	78649796	78664078	0.587598296	PASS
IFI44L	1	78619922	78646145	0.587598296	PASS
IFI6	1	27666061	27672218	0.048749694	PASS
IFIH1	2	162267079	162318703	0.319987345	PASS
IFIT1	10	89392546	89406486	-0.414937136	PASS
IFIT1B	10	89378056	89385205	-0.414937136	PASS
IFIT2	10	89283694	89309276	-0.414937136	PASS
IFIT3	10	89327894	89340971	-0.414937136	PASS
IFIT5	10	89414586	89421001	-0.414937136	PASS
IFITM1	11	313506	315272	-0.39835217	PASS
IFITM10	11	1732410	1750591	-0.39835217	PASS
IFITM2	11	307631	315272	-0.39835217	PASS
IFITM3	11	319669	327537	-0.39835217	PASS
IFITM5	11	298200	299526	-0.39835217	PASS
IFNA1	9	21440454	21441316	0.073960965	PASS
IFNA10	9	21206181	21207143	0.073960965	PASS
IFNA13	9	21367424	21368962	0.073960965	PASS
IFNA14	9	21239002	21239991	0.073960965	PASS
IFNA16	9	21216373	21217311	0.073960965	PASS
IFNA17	9	21227243	21228222	0.073960965	PASS
IFNA2	9	21384254	21385388	0.073960965	PASS
IFNA21	9	21165637	21166660	0.073960965	PASS
IFNA4	9	21186694	21187671	0.073960965	PASS
IFNA5	9	21304326	21305312	0.073960965	PASS
IFNA6	9	21349835	21351378	0.073960965	PASS
IFNA7	9	21201469	21202205	0.073960965	PASS
IFNA8	9	21409147	21410185	0.073960965	PASS
IFNAR1	21	33324477	33359862	0.209983559	PASS
IFNAR2	21	33229901	33265675	0.209983559	PASS
IFNB1	9	21077105	21077963	0.073960965	PASS
IFNE	9	21480839	21482313	0.073960965	PASS
IFNG	12	68154768	68159747	-0.743220499	PASS
IFNGR1	6	137197484	137219449	0.033894706	PASS
IFNGR2	21	33402896	33479348	0.209983559	PASS
IFNK	9	27524314	27526498	0.073960965	PASS
IFNL1	19	39296325	39298673	0.048917043	PASS
IFNL2	19	39268514	39270092	0.048917043	PASS
IFNL3	19	39243553	39245129	0.048917043	PASS
IFNLR1	1	24154157	24187959	0.048749694	PASS
IFNW1	9	21140214	21142145	0.073960965	PASS
IFRD1	7	112422968	112481017	-0.712149877	PASS
IFRD2	3	50287732	50292918	-0.727808231	PASS
IFT122	3	129440036	129520507	0.06434429	PASS
IFT140	16	1510427	1612110	0.053595371	PASS
IFT172	2	27444371	27489789	0.034263343	PASS
IFT20	17	28328325	28335489	0.041294512	PASS
IFT22	7	101313367	101321823	-0.712149877	PASS
IFT27	22	36758202	36776256	-0.740245994	PASS
IFT43	14	75902136	76084585	-0.155273506	suppress_segment_too_large
IFT46	11	118544528	118572970	-0.431650509	PASS
IFT52	20	43590931	43647296	-0.172056771	suppress_segment_too_large
IFT57	3	108160812	108222570	0.22470242	PASS

IFT74	9	26947039	27062930	0.073960965	PASS
IFT80	3	160256986	160399880	0.02267528	PASS
IFT81	12	110124335	110218797	0.028722739	PASS
IFT88	13	20567069	20691437	-0.398748891	PASS
IGBP1	23	70133449	70166324	0.158844052	PASS
IGDCC3	15	65327127	65378040	-0.759380635	PASS
IGDCC4	15	65381464	65423072	-0.759380635	PASS
IGF1	12	102395874	102481744	0.028722739	PASS
IGF1R	15	98648539	98964530	-0.759380635	PASS
IGF2	11	2129112	2158658	-0.39835217	PASS
IGF2BP1	17	48997412	49055650	0.051798665	PASS
IGF2BP2	3	185643739	185825056	0.02267528	PASS
IGF2BP3	7	23310209	23470467	-0.72421718	PASS
IGF2R	6	159969099	160113507	0.033894706	PASS
IGFALS	16	1790413	1794971	0.053595371	PASS
IGFBP1	7	45888357	45893668	-0.72421718	PASS
IGFBP2	2	216632828	216664436	0.011001208	PASS
IGFBP3	7	45912245	45921874	-0.72421718	PASS
IGFBP4	17	40443461	40457731	0.041294512	PASS
IGFBP5	2	216672105	216695525	0.011001208	PASS
IGFBP6	12	53097436	53102345	-0.743220499	PASS
IGFBP7	4	57030773	57110385	0.115781418	suppress_segment_too_large
IGFBPL1	9	38408994	38424447	0.073960965	PASS
IGFL1	19	46229752	46231243	0.002743503	PASS
IGFL2	19	46143106	46161299	0.002743503	PASS
IGFL3	19	46120071	46124674	0.002743503	PASS
IGFL4	19	46039748	46077118	0.002743503	PASS
IGFLR1	19	35738801	35742453	0.044838622	PASS
IGFN1	1	201190825	201228952	-0.204996646	suppress_segment_too_large
IGHMBP2	11	68903842	68940602	0.064535884	PASS
IGIP	5	140125935	140129392	-0.393084436	PASS
IGLL1	22	23573125	23580308	-0.740245994	PASS
IGLL5	22	22887780	22896107	-0.740245994	PASS
IGLON5	19	51311848	51330354	0.002743503	PASS
IGSF1	23	131273506	131578899	0.158844052	PASS
IGSF10	3	151425384	151458709	0.02267528	PASS
IGSF11	3	118900557	119146068	0.06434429	PASS
IGSF21	1	18107746	18378483	0.048749694	PASS
IGSF22	11	18704305	18726230	-0.39835217	PASS
IGSF23	19	44613630	44636781	0.002743503	PASS
IGSF3	1	116574399	116667755	0.079255572	PASS
IGSF5	21	39745407	39802096	0.209983559	PASS
IGSF6	16	21639537	21652660	0.053595371	PASS
IGSF8	1	160091340	160098943	0.088103245	PASS
IGSF9	1	159927039	159945604	0.088103245	PASS
IGSF9B	11	133908564	133956985	0.105032849	PASS
IHH	2	219054420	219060467	0.011001208	PASS
IK	5	140647058	140662479	-0.393084436	PASS
IKBIP	12	98613405	98645113	0.028722739	PASS
IKBKB	8	42271302	42332653	0.182873287	suppress_segment_too_large
IKBKE	1	206470476	206496889	-0.204996646	suppress_segment_too_large
IKBKG	23	154541199	154565046	0.158844052	PASS
IKZF1	7	50304068	50405101	-0.701511252	PASS
IKZF2	2	212999691	213152427	0.011001208	PASS

IKZF3	17	39757715	39864188	0.041294512	PASS
IKZF4	12	56007659	56038435	-0.743220499	PASS
IKZF5	10	122990806	123008817	0.073090211	PASS
IL10	1	206767602	206772494	-0.204996646	suppress_segment_too_large
IL10RA	11	117986348	118003037	-0.431650509	PASS
IL10RB	21	33266358	33310187	0.209983559	PASS
IL11	19	55364389	55370463	0.142581352	PASS
IL11RA	9	34650702	34661892	0.073960965	PASS
IL12A	3	159988750	159996019	0.02267528	PASS
IL12B	5	159314783	159330887	0.165341871	suppress_segment_too_large
IL12RB1	19	18058995	18098944	-0.069222905	PASS
IL12RB2	1	67307364	67397090	0.052232535	PASS
IL13	5	132656263	132661110	-0.393084436	PASS
IL13RA1	23	118727572	118794539	0.158844052	PASS
IL13RA2	23	115003975	115019977	0.158844052	PASS
IL15	4	141636599	141733987	0.142431244	suppress_segment_too_large
IL15RA	10	5943639	5978187	0.075711544	PASS
IL16	15	81159575	81314058	-0.759380635	PASS
IL17A	6	52186375	52190638	0.239805577	suppress_segment_too_large
IL17B	5	149371324	149404202	-0.393084436	PASS
IL17C	16	88638591	88640473	-0.423061015	PASS
IL17D	13	20702127	20723098	-0.398748891	PASS
IL17F	6	52236681	52244537	0.239805577	suppress_segment_too_large
IL17RA	22	17084954	17115694	-0.740245994	PASS
IL17RB	3	53846580	53865800	-0.727808231	PASS
IL17RC	3	9917074	9933630	0.06149513	PASS
IL17RD	3	57089982	57170306	-0.727808231	PASS
IL17RE	3	9902612	9916402	0.06149513	PASS
IL17REL	22	49994513	50012659	-0.953096644	PASS
IL18	11	112143251	112164117	-0.431650509	PASS
IL18BP	11	71998541	72005715	0.064535884	PASS
IL18R1	2	102311529	102398775	0.064122364	PASS
IL18RAP	2	102418689	102452565	0.064122364	PASS
IL19	1	206798870	206842981	-0.204996646	suppress_segment_too_large
IL1A	2	112773915	112784590	0.040971718	PASS
IL1B	2	112829751	112836903	0.040971718	PASS
IL1F10	2	113067970	113075850	0.040971718	PASS
IL1R1	2	102064544	102179874	0.064122364	PASS
IL1R2	2	101991844	102028544	0.064122364	PASS
IL1RAP	3	190514051	190659750	0.02267528	PASS
IL1RAPL1	23	28587399	29956723	0.158844052	PASS
IL1RAPL2	23	104566315	105767829	0.158844052	PASS
IL1RL1	2	102311502	102352037	0.064122364	PASS
IL1RL2	2	102186973	102240002	0.064122364	PASS
IL1RN	2	113107214	113134016	0.040971718	PASS
IL2	4	122451470	122456725	0.142431244	suppress_segment_too_large
IL20	1	206865354	206869223	-0.204996646	suppress_segment_too_large
IL20RA	6	136999971	137045180	0.033894706	PASS
IL20RB	3	136946230	137011085	0.06434429	PASS
IL21	4	122610108	122621069	0.142431244	suppress_segment_too_large
IL21R	16	27402162	27452042	0.053595371	PASS
IL22	12	68248242	68253604	-0.743220499	PASS
IL22RA1	1	24119771	24143121	0.048749694	PASS
IL22RA2	6	137143820	137173648	0.033894706	PASS

IL23A	12	56334174	56340410	-0.743220499	PASS
IL23R	1	67138907	67259979	0.052232535	PASS
IL24	1	206897443	206904139	-0.204996646	suppress_segment_too_large
IL25	14	23372809	23376403	0.053252507	PASS
IL26	12	68201351	68225821	-0.743220499	PASS
IL27	16	28499362	28512051	0.053595371	PASS
IL27RA	19	14031748	14053216	-0.069222905	PASS
IL2RA	10	6010689	6062370	0.075711544	PASS
IL2RB	22	37125838	37175054	-0.740245994	PASS
IL2RG	23	71107404	71112108	0.158844052	PASS
IL3	5	132060529	132063204	-0.393084436	PASS
IL31	12	122172030	122174199	0.028722739	PASS
IL31RA	5	55851379	55922853	-0.284712224	PASS
IL32	16	3065297	3082192	0.053595371	PASS
IL33	9	6215786	6257983	0.073960965	PASS
IL34	16	70579895	70660682	-0.423061015	PASS
IL36A	2	113005461	113008044	0.040971718	PASS
IL36B	2	113022091	113052867	0.040971718	PASS
IL36G	2	112973203	112985665	0.040971718	PASS
IL36RN	2	113058638	113065382	0.040971718	PASS
IL37	2	112912971	112918882	0.040971718	PASS
IL3RA	23	1336616	1382689	-0.350651546	PASS
IL4	5	132673986	132682676	-0.393084436	PASS
IL4I1	19	49889654	49929539	0.002743503	PASS
IL4R	16	27313668	27364778	0.053595371	PASS
IL5	5	132541444	132556838	-0.393084436	PASS
IL5RA	3	3066326	3126613	0.06149513	PASS
IL6	7	22725884	22732002	-0.72421718	PASS
IL6R	1	154405193	154469450	0.088103245	PASS
IL6ST	5	55935095	55994993	-0.284712224	PASS
IL7	8	78675743	78805523	0.617295349	suppress_segment_too_large
IL7R	5	35852695	35879603	0.073527871	PASS
IL9	5	135892246	135895827	-0.393084436	PASS
IL9R	23	155997581	156010817	-0.537824465	PASS
ILDR1	3	121987323	122022258	0.06434429	PASS
ILDR2	1	166895711	166975482	0.088103245	PASS
ILF2	1	153661788	153671048	0.088103245	PASS
ILF3	19	10654261	10692417	-0.107594294	PASS
ILK	11	6603708	6610874	-0.39835217	PASS
ILKAP	2	238170401	238203729	0.011001208	PASS
ILVBL	19	15114984	15125785	-0.069222905	PASS
IMMP1L	11	31432401	31509645	-0.359426047	PASS
IMMP2L	7	110663051	111562517	-0.712149877	PASS
IMMT	2	86143932	86195770	0.064122364	PASS
IMP3	15	75639085	75648706	-0.759380635	PASS
IMP4	2	130342225	130347810	-0.359440172	PASS
IMPA1	8	81657961	81686693	0.617295349	suppress_segment_too_large
IMPA2	18	11981025	12030883	0.044197172	PASS
IMPACT	18	24426616	24453535	-0.330515409	PASS
IMPAD1	8	56957933	56993844	0.182873287	suppress_segment_too_large
IMPDH1	7	128392277	128410252	-0.712149877	PASS
IMPDH2	3	49024325	49029408	-0.727808231	PASS
IMPG1	6	75921115	76072678	-0.250122709	PASS
IMPG2	3	101222546	101320560	0.22470242	PASS

INA	10	103277163	103290351	-0.414937136	PASS
INAFM1	19	47274453	47275707	0.002743503	PASS
INAFM2	15	40323664	40326715	-0.423772929	PASS
INAVA	1	200891048	200915735	-0.204996646	suppress_segment_too_large
INCA1	17	4988130	4997610	0.06854245	PASS
INCENP	11	62123973	62153163	0.064535884	PASS
INF2	14	104689606	104722535	-0.155273506	suppress_segment_too_large
ING1	13	110712736	110723339	-0.426822582	PASS
ING2	4	183504994	183511096	-0.453876349	PASS
ING3	7	120950749	120977216	-0.712149877	PASS
ING4	12	6650280	6663148	-0.015029783	PASS
INHA	2	219569162	219575713	0.011001208	PASS
INHBA	7	41667168	41705834	-0.72421718	PASS
INHBB	2	120346143	120351808	-0.359440172	PASS
INHBC	12	57434760	57450828	-0.743220499	PASS
INHBE	12	57452323	57459280	-0.743220499	PASS
INIP	9	112683926	112718236	0.073960965	PASS
INKA1	3	49803254	49805030	-0.727808231	PASS
INKA2	1	111680630	111739424	0.079255572	PASS
INMT	7	30697985	30757602	-0.72421718	PASS
INMT-MII	7	30752137	30892081	-0.72421718	PASS
INO80	15	40978880	41116354	-0.423772929	PASS
INO80B	2	74455023	74457960	-0.46122572	PASS
INO80B-W	2	74455088	74460884	-0.46122572	PASS
INO80C	18	35452230	35497991	-0.330515409	PASS
INO80D	2	205993721	206086303	0.011001208	PASS
INO80E	16	29995294	30005793	0.053595371	PASS
INPP1	2	190343470	190371665	0.011001208	PASS
INPP4A	2	98444854	98594390	0.064122364	PASS
INPP4B	4	142023160	142847432	0.142431244	suppress_segment_too_large
INPP5A	10	132537820	132783480	0.073090211	PASS
INPP5B	1	37860697	37947057	0.048749694	PASS
INPP5D	2	233059967	233207903	0.011001208	PASS
INPP5E	9	136428619	136439823	0.073960965	PASS
INPP5F	10	119726042	119829278	0.073090211	PASS
INPP5J	22	31122731	31134696	-0.740245994	PASS
INPP5K	17	1494571	1516888	0.06854245	PASS
INPPL1	11	72223701	72239105	0.064535884	PASS
INS	11	2159779	2161341	-0.39835217	PASS
INS-IGF2	11	2132538	2161209	-0.39835217	PASS
INSC	11	15112424	15247208	-0.39835217	PASS
INSIG1	7	155297776	155310235	-0.77630473	PASS
INSIG2	2	118088452	118110997	-0.359440172	PASS
INSL3	19	17816512	17821574	-0.069222905	PASS
INSL4	9	5231419	5235304	0.073960965	PASS
INSL5	1	66797741	66801256	0.052232535	PASS
INSL6	9	5123880	5185668	0.073960965	PASS
INSM1	20	20368121	20370949	-0.172056771	suppress_segment_too_large
INSM2	14	35534042	35537054	0.053252507	PASS
INSR	19	7112255	7294034	-0.071973357	PASS
INSRR	1	156840063	156859018	0.088103245	PASS
INSYN1	15	73735431	73752747	-0.759380635	PASS
INSYN2	10	127135426	127196158	0.073090211	PASS
INSYN2B	5	169864264	169980740	0.165341871	suppress_segment_too_large

INTS1	7	1470277	1504367	-0.72421718	PASS
INTS10	8	19817140	19852083	-0.311940419	PASS
INTS11	1	1311585	1324691	-0.358281697	PASS
INTS12	4	105682627	105895986	-0.677982248	PASS
INTS13	12	26905181	26938326	0.49032213	suppress_segment_too_large
INTS14	15	65578753	65611289	-0.759380635	PASS
INTS2	17	61865367	61928016	0.051798665	PASS
INTS3	1	153728067	153774808	0.088103245	PASS
INTS4	11	77878720	77994678	0.064535884	PASS
INTS5	11	62646848	62653302	0.064535884	PASS
INTS6	13	51354077	51454264	-0.384313056	PASS
INTS6L	23	135520643	135582510	0.158844052	PASS
INTS7	1	211940399	212035542	-0.204996646	suppress_segment_too_large
INTS8	8	94813311	94881746	0.355469066	suppress_segment_too_large
INTS9	8	28767661	28890242	-0.311940419	PASS
INTU	4	127623271	127726737	0.142431244	suppress_segment_too_large
INVS	9	100099256	100301000	0.073960965	PASS
IP6K1	3	49724294	49786542	-0.727808231	PASS
IP6K2	3	48688003	48740353	-0.727808231	PASS
IP6K3	6	33721670	33746905	0.028532667	PASS
IPCEF1	6	154154496	154356792	0.033894706	PASS
IPMK	10	58191517	58267934	0.032351597	PASS
IPO11	5	62403972	62628582	0.07724032	PASS
IPO13	1	43946939	43968022	-0.736943052	PASS
IPO4	14	24180219	24188964	0.053252507	PASS
IPO5	13	97953658	98024297	-0.384313056	PASS
IPO7	11	9384622	9448126	-0.39835217	PASS
IPO8	12	30628988	30695986	0.49032213	suppress_segment_too_large
IPO9	1	201829141	201884294	-0.204996646	suppress_segment_too_large
IPP	1	45694324	45750650	-0.736943052	PASS
IPPK	9	92613184	92670265	0.073960965	PASS
IQANK1	8	143734140	143790644	0.699132336	PASS
IQCA1	2	236324147	236507542	0.011001208	PASS
IQCA1L	7	151190873	151205496	-0.712149877	PASS
IQCB1	3	121769763	121835079	0.06434429	PASS
IQCC	1	32205661	32208687	0.048749694	PASS
IQCD	12	113195441	113221094	0.028722739	PASS
IQCE	7	2558972	2614734	-0.72421718	PASS
IQCF1	3	51894876	51903335	-0.727808231	PASS
IQCF2	3	51861629	51863424	-0.727808231	PASS
IQCF3	3	51826883	51830856	-0.727808231	PASS
IQCF5	3	51873721	51875584	-0.727808231	PASS
IQCF6	3	51778561	51779241	-0.727808231	PASS
IQCG	3	197889075	197960142	0.02267528	PASS
IQCH	15	67254800	67502260	-0.759380635	PASS
IQCJ	3	158962928	159266307	0.02267528	PASS
IQCJ-SCH	3	158962235	159897366	0.02267528	PASS
IQCK	16	19716456	19858467	0.053595371	PASS
IQCM	4	149351903	149896233	0.142431244	suppress_segment_too_large
IQCN	19	18257097	18274509	-0.069222905	PASS
IQGAP1	15	90388218	90502243	-0.759380635	PASS
IQGAP2	5	76403249	76708132	-0.393084436	PASS
IQGAP3	1	156525405	156572604	0.088103245	PASS
IQSEC1	3	12897220	13283281	0.06149513	PASS

IQSEC2	23	53225828	53321350	0.158844052	PASS
IQSEC3	12	66765	178460	-0.015029783	PASS
IQUB	7	123452400	123535077	-0.712149877	PASS
IRAK1	23	154010500	154019980	0.158844052	PASS
IRAK1BP1	6	78867472	78946440	-0.250122709	PASS
IRAK2	3	10164865	10243743	0.06149513	PASS
IRAK3	12	66188879	66254622	-0.743220499	PASS
IRAK4	12	43758944	43789543	-0.46813528	PASS
IREB2	15	78437431	78501456	-0.759380635	PASS
IRF1	5	132481609	132490798	-0.393084436	PASS
IRF2	4	184387713	184474580	-0.453876349	PASS
IRF2BP1	19	45883607	45886170	0.002743503	PASS
IRF2BP2	1	234604269	234609525	-0.204996646	suppress_segment_too_large
IRF2BPL	14	77024543	77028699	-0.155273506	suppress_segment_too_large
IRF3	19	49659569	49665875	0.002743503	PASS
IRF4	6	391739	411447	0.069043418	PASS
IRF5	7	128937612	128950035	-0.712149877	PASS
IRF6	1	209785623	209806175	-0.204996646	suppress_segment_too_large
IRF7	11	612553	615999	-0.39835217	PASS
IRF8	16	85898803	85922609	-0.423061015	PASS
IRF9	14	24161053	24166565	0.053252507	PASS
IRGC	19	43716010	43720021	0.048917043	PASS
IRGM	5	150846523	150900736	-0.393084436	PASS
IRGQ	19	43584369	43596135	0.048917043	PASS
IRS1	2	226731317	226799759	0.011001208	PASS
IRS2	13	109752698	109786568	-0.426822582	PASS
IRS4	23	108732482	108736409	0.158844052	PASS
IRX1	5	3596054	3601403	0.073527871	PASS
IRX2	5	2745845	2751662	0.073527871	PASS
IRX3	16	54283304	54286763	-0.423061015	PASS
IRX4	5	1877413	1887236	0.073527871	PASS
IRX5	16	54930862	54934485	-0.423061015	PASS
IRX6	16	55323760	55330760	-0.423061015	PASS
ISCA1	9	86264546	86283102	0.073960965	PASS
ISCA2	14	74493720	74497106	-0.155273506	suppress_segment_too_large
ISCU	12	108562582	108569384	0.028722739	PASS
ISG15	1	1001138	1014540	-0.358281697	PASS
ISG20	15	88636153	88656483	-0.759380635	PASS
ISG20L2	1	156721891	156728799	0.088103245	PASS
ISL1	5	51383391	51394738	-0.284712224	PASS
ISL2	15	76336724	76342476	-0.759380635	PASS
ISLR	15	74173671	74176872	-0.759380635	PASS
ISLR2	15	74100311	74138540	-0.759380635	PASS
ISM1	20	13221771	13300651	-0.172056771	suppress_segment_too_large
ISM2	14	77474394	77498850	-0.155273506	suppress_segment_too_large
ISOC1	5	129094751	129114028	-0.393084436	PASS
ISOC2	19	55452985	55462343	0.142581352	PASS
ISPD	7	16087527	16421322	-0.72421718	PASS
IST1	16	71845996	71931199	-0.423061015	PASS
ISX	22	35066136	35087387	-0.740245994	PASS
ISY1	3	129127415	129161293	0.06434429	PASS
ISY1-RAB	3	129087575	129161036	0.06434429	PASS
ISYNA1	19	18434388	18438301	-0.069222905	PASS
ITCH	20	34363235	34511393	-0.172056771	suppress_segment_too_large

ITFG1	16	47154387	47464149	0.053595371	PASS
ITFG2	12	2812622	2859791	-0.015029783	PASS
ITGA1	5	52787896	52959210	-0.284712224	PASS
ITGA10	1	145891208	145910189	0.079255572	PASS
ITGA11	15	68296533	68432162	-0.759380635	PASS
ITGA2	5	52989326	53094779	-0.284712224	PASS
ITGA2B	17	44372180	44389505	0.051798665	PASS
ITGA3	17	50055968	50090481	0.051798665	PASS
ITGA4	2	181457202	181536187	0.162734012	PASS
ITGA5	12	54395261	54419460	-0.743220499	PASS
ITGA6	2	172427354	172506282	0.033541467	PASS
ITGA7	12	55684568	55716043	-0.743220499	PASS
ITGA8	10	15513949	15720125	0.075711544	PASS
ITGA9	3	37452115	37823514	-0.727808231	PASS
ITGAD	16	31393312	31426505	0.053595371	PASS
ITGAE	17	3714628	3801243	0.06854245	PASS
ITGAL	16	30472658	30523185	0.053595371	PASS
ITGAM	16	31259967	31332892	0.053595371	PASS
ITGAV	2	186590065	186680901	-0.120408457	PASS
ITGAX	16	31355134	31382997	0.053595371	PASS
ITGB1	10	32900319	33005792	0.075711544	PASS
ITGB1BP1	2	9403475	9423547	0.034263343	PASS
ITGB1BP2	23	71301734	71305371	0.158844052	PASS
ITGB2	21	44885953	44931989	0.209983559	PASS
ITGB3	17	47253846	47311816	0.051798665	PASS
ITGB3BP	1	63440770	63593721	0.052232535	PASS
ITGB4	17	75721328	75757818	0.051798665	PASS
ITGB5	3	124761948	124901418	0.06434429	PASS
ITGB6	2	160099666	160200313	0.054749241	PASS
ITGB7	12	53191318	53207307	-0.743220499	PASS
ITGB8	7	20330702	20415754	-0.72421718	PASS
ITGBL1	13	101452593	101720856	-0.384313056	PASS
ITIH1	3	52777592	52792068	-0.727808231	PASS
ITIH2	10	7703269	7749520	0.075711544	PASS
ITIH3	3	52794768	52809009	-0.727808231	PASS
ITIH4	3	52812975	52831479	-0.727808231	PASS
ITIH5	10	7559270	7666998	0.075711544	PASS
ITIH6	23	54748899	54798240	0.158844052	PASS
ITK	5	157142933	157255191	0.165341871	suppress_segment_too_large
ITLN1	1	160876539	160885170	0.088103245	PASS
ITLN2	1	160945025	160954799	0.088103245	PASS
ITM2A	23	79360384	79367667	0.158844052	PASS
ITM2B	13	48232612	48270357	-3.165473388	PASS
ITM2C	2	230864639	230879248	0.011001208	PASS
ITPA	20	3208868	3223870	-0.172056771	suppress_segment_too_large
ITPK1	14	92936914	93116320	-0.155273506	suppress_segment_too_large
ITPKA	15	41493393	41503551	-0.423772929	PASS
ITPKB	1	226631690	226739323	-0.204996646	suppress_segment_too_large
ITPKC	19	40717103	40740860	0.048917043	PASS
ITPR1	3	4493345	4847840	0.06149513	PASS
ITPR2	12	26336515	26833198	0.49032213	suppress_segment_too_large
ITPR3	6	33620365	33696574	0.028532667	PASS
ITPRID1	7	31514071	31658720	-0.72421718	PASS
ITPRID2	2	181891833	181930738	0.162734012	PASS

ITPRIP	10	104309698	104338448	-0.414937136	PASS
ITPRIPL1	2	96325331	96330517	0.064122364	PASS
ITPRIPL2	16	19113932	19121629	0.053595371	PASS
ITSN1	21	33642400	33899861	0.209983559	PASS
ITSN2	2	24202864	24360714	0.034263343	PASS
IVD	15	40405795	40435947	-0.423772929	PASS
IVL	1	152908545	152911886	0.088103245	PASS
IVNS1ABI	1	185296388	185317329	0.088103245	PASS
IWS1	2	127436207	127526886	-0.359440172	PASS
IYD	6	150368892	150405969	0.033894706	PASS
IZUMO1	19	48740852	48746909	0.002743503	PASS
IZUMO1R	11	94305592	94307721	0.064535884	PASS
IZUMO2	19	50152548	50163195	0.002743503	PASS
IZUMO3	9	24542952	24545946	0.073960965	PASS
IZUMO4	19	2096429	2099593	-0.071973357	PASS
JADE1	4	128809623	128875224	0.142431244	suppress_segment_too_large
JADE2	5	134524312	134583230	-0.393084436	PASS
JADE3	23	46912276	47061242	0.158844052	PASS
JAG1	20	10637684	10673999	-0.172056771	suppress_segment_too_large
JAG2	14	105140981	105168824	-0.155273506	suppress_segment_too_large
JAGN1	3	9890572	9894349	0.06149513	PASS
JAK1	1	64833229	64966504	0.052232535	PASS
JAK2	9	4984390	5128183	0.073960965	PASS
JAK3	19	17824780	17848071	-0.069222905	PASS
JAKMIP1	4	6026203	6200591	-0.358623662	PASS
JAKMIP2	5	147585439	147782848	-0.393084436	PASS
JAKMIP3	10	132104671	132184809	0.073090211	PASS
JAM2	21	25639272	25717562	0.209983559	PASS
JAM3	11	134068925	134152001	0.105032849	PASS
JAML	11	118193740	118225094	-0.431650509	PASS
JARID2	6	15246296	15522040	0.069043418	PASS
JAZF1	7	27830573	28180743	-0.72421718	PASS
JCAD	10	30012800	30115494	0.075711544	PASS
JCHAIN	4	70655541	70681817	0.115781418	suppress_segment_too_large
JDP2	14	75427716	75474111	-0.155273506	suppress_segment_too_large
JHY	11	122882528	122959798	-0.431650509	PASS
JKAMP	14	59484443	59505410	0.053252507	PASS
JMJD1C	10	63167221	63521850	0.032351597	PASS
JMJD4	1	227730425	227735411	-0.204996646	suppress_segment_too_large
JMJD6	17	76712832	76726799	0.051798665	PASS
JMJD7	15	41828085	41837581	-0.423772929	PASS
JMJD7-PL	15	41828095	41848155	-0.423772929	PASS
JMJD8	16	681671	684528	0.053595371	PASS
JMY	5	79236189	79327215	-0.393084436	PASS
JOSD1	22	38685543	38701556	-0.740245994	PASS
JOSD2	19	50505998	50511353	0.002743503	PASS
JPH1	8	74234700	74321328	0.182873287	suppress_segment_too_large
JPH2	20	44111695	44187578	-0.172056771	suppress_segment_too_large
JPH3	16	87601835	87698156	-0.423061015	PASS
JPH4	14	23568035	23578800	0.053252507	PASS
JPT1	17	75135248	75168281	0.051798665	PASS
JPT2	16	1678256	1702280	0.053595371	PASS
JRK	8	142657460	142681968	0.699132336	PASS
JRKL	11	96389989	96507574	0.32805343	suppress_segment_too_large

JSRP1	19	2252252	2269759	-0.071973357	PASS
JTB	1	153974269	153977688	0.088103245	PASS
JUN	1	58780788	58784327	-0.736943052	PASS
JUNB	19	12791496	12793315	-0.069222905	PASS
JUND	19	18279760	18281622	-0.069222905	PASS
JUP	17	41754604	41786931	0.041294512	PASS
KAAG1	6	24356903	24358284	0.069043418	PASS
KALRN	3	124080023	124726325	0.06434429	PASS
KANK1	9	470291	746106	0.073960965	PASS
KANK2	19	11164267	11197791	-0.107594294	PASS
KANK3	19	8322584	8343262	-0.071973357	PASS
KANK4	1	62236979	62319414	0.052232535	PASS
KANSL1	17	46029916	46225389	0.051798665	PASS
KANSL1L	2	210021423	210171383	0.011001208	PASS
KANSL2	12	48653401	48682238	-0.743220499	PASS
KANSL3	2	96593170	96642787	0.064122364	PASS
KANTR	23	53094145	53170914	0.158844052	PASS
KARS	16	75627474	75648643	-0.423061015	PASS
KAT14	20	18138118	18188387	-0.172056771	suppress_segment_too_large
KAT2A	17	42113108	42121358	0.041294512	PASS
KAT2B	3	20040023	20154404	0.06149513	PASS
KAT5	11	65711996	65719604	0.064535884	PASS
KAT6A	8	41929479	42052026	0.182873287	suppress_segment_too_large
KAT6B	10	74824927	75032624	0.032351597	PASS
KAT7	17	49788555	49835030	0.051798665	PASS
KAT8	16	31115754	31131393	0.053595371	PASS
KATNA1	6	149594873	149648972	0.033894706	PASS
KATNAL1	13	30202630	30307484	-0.398748891	PASS
KATNAL2	18	46917492	47102243	-0.330515409	PASS
KATNB1	16	57735730	57757250	-0.423061015	PASS
KATNBL1	15	34140674	34210096	-0.423772929	PASS
KAZALD1	10	101061841	101068131	-0.414937136	PASS
KAZN	1	13892792	15118043	0.048749694	PASS
KBTBD11	8	1973878	2006936	-0.311940419	PASS
KBTBD11·	8	1763888	1958627	-0.311940419	PASS
KBTBD12	3	127915232	127987671	0.06434429	PASS
KBTBD13	15	65076816	65079939	-0.759380635	PASS
KBTBD2	7	32868172	32894131	-0.72421718	PASS
KBTBD3	11	106051098	106077765	0.32805343	suppress_segment_too_large
KBTBD4	11	47572197	47579015	0.059795455	PASS
KBTBD6	13	41127569	41132746	-0.398748891	PASS
KBTBD7	13	41189833	41194566	-0.398748891	PASS
KBTBD8	3	66998307	67011210	-0.727808231	PASS
KCMF1	2	84971093	85059472	0.064122364	PASS
KCNA1	12	4909905	4918256	-0.015029783	PASS
KCNA10	1	110517217	110519175	0.079255572	PASS
KCNA2	1	110519837	110631474	0.079255572	PASS
KCNA3	1	110672465	110675033	0.079255572	PASS
KCNA4	11	30009741	30017023	-0.359426047	PASS
KCNA5	12	5043989	5046788	-0.015029783	PASS
KCNA6	12	4809176	4813412	-0.015029783	PASS
KCNA7	19	49067418	49072941	0.002743503	PASS
KCNAB1	3	156037701	156539138	0.02267528	PASS
KCNAB2	1	5991466	6101193	0.364796772	PASS

KCNAB3	17	7921859	7929803	0.06854245	PASS
KCNB1	20	49293394	49484297	-0.172056771	suppress_segment_too_large
KCNB2	8	72537391	72938349	0.182873287	suppress_segment_too_large
KCNC1	11	17734774	17856804	-0.39835217	PASS
KCNC2	12	75040077	75209868	0.271127429	suppress_segment_too_large
KCNC3	19	50311937	50333515	0.002743503	PASS
KCNC4	1	110211343	110283100	0.079255572	PASS
KCND1	23	48961378	48971569	0.158844052	PASS
KCND2	7	120273668	120750331	-0.712149877	PASS
KCND3	1	111770662	111989155	0.079255572	PASS
KCNE1	21	34446688	34512275	0.209983559	PASS
KCNE1B	21	7816675	7829926	0.220071762	PASS
KCNE2	21	34364024	34371389	0.209983559	PASS
KCNE3	11	74454841	74467729	0.064535884	PASS
KCNE4	2	223051814	223198399	0.011001208	PASS
KCNE5	23	109623700	109625172	0.158844052	PASS
KCNF1	2	10911937	10914225	0.034263343	PASS
KCNG1	20	51003656	51023129	-0.172056771	suppress_segment_too_large
KCNG2	18	79863668	79900184	-0.330515409	PASS
KCNG3	2	42442017	42494097	0.034263343	PASS
KCNG4	16	84218667	84239750	-0.423061015	PASS
KCNH1	1	210676823	211134180	-0.204996646	suppress_segment_too_large
KCNH2	7	150944961	150978315	-0.712149877	PASS
KCNH3	12	49539157	49558308	-0.743220499	PASS
KCNH4	17	42156891	42181278	0.041294512	PASS
KCNH5	14	62699454	63102037	0.053252507	PASS
KCNH6	17	63523334	63548977	0.051798665	PASS
KCNH7	2	162371407	162838730	0.319987345	PASS
KCNH8	3	19148454	19535646	0.06149513	PASS
KCNIP1	5	170353487	170736632	0.165341871	suppress_segment_too_large
KCNIP2	10	101825974	101843920	-0.414937136	PASS
KCNIP3	2	95297304	95386083	0.064122364	PASS
KCNIP4	4	20728616	21948799	-0.358623662	PASS
KCNJ1	11	128836315	128867373	-0.431650509	PASS
KCNJ10	1	159998651	160070483	0.088103245	PASS
KCNJ11	11	17385859	17389331	-0.39835217	PASS
KCNJ12	17	21376197	21419872	0.041294512	PASS
KCNJ13	2	232766464	232776568	0.011001208	PASS
KCNJ14	19	48455509	48466980	0.002743503	PASS
KCNJ15	21	38155549	38307357	0.209983559	PASS
KCNJ16	17	70053429	70135608	0.051798665	PASS
KCNJ18	17	21692523	21704612	0.041294512	PASS
KCNJ2	17	70168673	70180048	0.051798665	PASS
KCNJ3	2	154698299	154858352	-0.30549516	PASS
KCNJ4	22	38426327	38455199	-0.740245994	PASS
KCNJ5	11	128891356	128921035	-0.431650509	PASS
KCNJ6	21	37607376	38121345	0.209983559	PASS
KCNJ8	12	21764955	21775581	0.49032213	suppress_segment_too_large
KCNJ9	1	160081570	160090563	0.088103245	PASS
KCNK1	1	233614004	233672512	-0.204996646	suppress_segment_too_large
KCNK10	14	88180103	88326907	-0.155273506	suppress_segment_too_large
KCNK12	2	47516581	47570939	0.034263343	PASS
KCNK13	14	90061765	90185857	-0.155273506	suppress_segment_too_large
KCNK15	20	44745780	44752313	-0.172056771	suppress_segment_too_large

KCNK16	6	39314698	39322968	0.028532667	PASS
KCNK17	6	39299001	39314553	0.028532667	PASS
KCNK18	10	117197489	117210299	0.073090211	PASS
KCNK2	1	215005775	215237093	-0.204996646	suppress_segment_too_large
KCNK3	2	26692690	26733420	0.034263343	PASS
KCNK4	11	64291302	64300031	0.064535884	PASS
KCNK5	6	39188973	39229450	0.028532667	PASS
KCNK6	19	38319844	38332076	0.048917043	PASS
KCNK7	11	65592855	65595996	0.064535884	PASS
KCNK9	8	139600838	139704109	0.493344802	suppress_segment_too_large
KCNMA1	10	76869601	77638369	0.032351597	PASS
KCNMB1	5	170374671	170389677	0.165341871	suppress_segment_too_large
KCNMB2	3	178272932	178844429	0.02267528	PASS
KCNMB3	3	179236691	179267002	0.02267528	PASS
KCNMB4	12	70366276	70434292	-0.578548701	PASS
KCNN1	19	17951293	18000080	-0.069222905	PASS
KCNN2	5	114055545	114496500	-0.393084436	PASS
KCNN3	1	154697455	154870280	0.088103245	PASS
KCNN4	19	43766533	43781261	0.048917043	PASS
KCNQ1	11	2444684	2849110	-0.39835217	PASS
KCNQ2	20	63400210	63472677	-0.172056771	suppress_segment_too_large
KCNQ3	8	132120858	132481019	0.493344802	suppress_segment_too_large
KCNQ4	1	40783814	40840452	0.048749694	PASS
KCNQ5	6	72621792	73198851	-0.250122709	PASS
KCNRG	13	50015254	50020922	-0.384313056	PASS
KCNS1	20	45092310	45101112	-0.172056771	suppress_segment_too_large
KCNS2	8	98427022	98432848	0.355469066	suppress_segment_too_large
KCNS3	2	17877847	18361616	0.034263343	PASS
KCNT1	9	135702185	135795508	0.073960965	PASS
KCNT2	1	196225779	196609225	0.088103245	PASS
KCNU1	8	36784324	36936128	0.182873287	suppress_segment_too_large
KCNV1	8	109963645	109975847	0.70944438	suppress_segment_too_large
KCNV2	9	2717502	2730037	0.073960965	PASS
KCP	7	128862451	128910719	-0.712149877	PASS
KCTD1	18	26454910	26657401	-0.330515409	PASS
KCTD10	12	109448656	109477544	0.028722739	PASS
KCTD11	17	7351889	7354944	0.06854245	PASS
KCTD12	13	76880166	76886390	-0.384313056	PASS
KCTD13	16	29905012	29927035	0.053595371	PASS
KCTD14	11	78015715	78046191	0.064535884	PASS
KCTD15	19	33795933	33815763	0.044838622	PASS
KCTD16	5	144170832	144485686	-0.393084436	PASS
KCTD17	22	37051736	37063390	-0.740245994	PASS
KCTD18	2	200488952	200519784	0.011001208	PASS
KCTD19	16	67289428	67326763	-0.423061015	PASS
KCTD2	17	75032575	75065889	0.051798665	PASS
KCTD20	6	36442767	36491143	0.028532667	PASS
KCTD21	11	78171249	78188822	0.064535884	PASS
KCTD3	1	215567392	215621807	-0.204996646	suppress_segment_too_large
KCTD4	13	45192853	45194717	-0.398748891	PASS
KCTD5	16	2682475	2709030	0.053595371	PASS
KCTD6	3	58492114	58502360	-0.727808231	PASS
KCTD7	7	66628767	66649067	-0.701511252	PASS
KCTD8	4	44173909	44448807	-0.317525751	PASS

KCTD9	8	25427847	25458476		-0.311940419	PASS
KDELC1	13	102784281	102799007		-0.426822582	PASS
KDELC2	11	108472105	108498432		-0.431650509	PASS
KDELR1	19	48382570	48391553		0.002743503	PASS
KDELR2	7	6445953	6484242		-0.72421718	PASS
KDELR3	22	38468062	38483447		-0.740245994	PASS
KDF1	1	26949562	26960406		0.048749694	PASS
KDM1A	1	23019443	23083689		0.048749694	PASS
KDM1B	6	18155329	18223853		0.069043418	PASS
KDM2A	11	67119269	67258087		0.064535884	PASS
KDM2B	12	121429097	121581015		0.028722739	PASS
KDM3A	2	86440647	86492716		0.064122364	PASS
KDM3B	5	138352596	138437028		-0.393084436	PASS
KDM4A	1	43650158	43705515		-0.736943052	PASS
KDM4B	19	4969113	5153595		-0.071973357	PASS
KDM4C	9	6720863	7175648		0.073960965	PASS
KDM4D	11	94973681	94999518		0.064535884	PASS
KDM4E	11	95025258	95027596		0.064535884	PASS
KDM4F	11	95049422	95051338		0.32805343	suppress_segment_too_large
KDM5A	12	280129	389454		-0.015029783	PASS
KDM5B	1	202724495	202809470		-0.204996646	suppress_segment_too_large
KDM5C	23	53191321	53225422		0.158844052	PASS
KDM6A	23	44873177	45112602		0.158844052	PASS
KDM6B	17	7839904	7854796		0.06854245	PASS
KDM7A	7	140084746	140177035		-0.712149877	PASS
KDM8	16	27203495	27221768		0.053595371	PASS
KDR	4	55078259	55125595		0.115781418	suppress_segment_too_large
KDSR	18	63327726	63367510		-0.330515409	PASS
KEAP1	19	10486120	10503741		-0.107594294	PASS
KEL	7	142941114	142962681		-0.712149877	PASS
KERA	12	91050491	91057983		0.271127429	suppress_segment_too_large
KHDC1	6	73241314	73310365		-0.250122709	PASS
KHDC1L	6	73223544	73225770		-0.250122709	PASS
KHDC3L	6	73362677	73364171		-0.250122709	PASS
KHDC4	1	155913043	155934400		0.088103245	PASS
KHDRBS1	1	32013829	32060850		0.048749694	PASS
KHDRBS2	6	61679960	62286227		0.239805577	suppress_segment_too_large
KHDRBS3	8	135457457	135656722		0.493344802	suppress_segment_too_large
KHK	2	27086747	27100772		0.034263343	PASS
KHNYN	14	24429286	24441834		0.053252507	PASS
KHSRP	19	6413348	6424794		-0.071973357	PASS
KIAA0040	1	175156987	175192999		0.088103245	PASS
KIAA0100	17	28614440	28645454		0.041294512	PASS
KIAA0232	4	6781375	6884170		-0.358623662	PASS
KIAA0319	6	24544104	24646155		0.069043418	PASS
KIAA0319	1	35433490	35557950		0.048749694	PASS
KIAA0355	19	34254537	34355586		0.044838622	PASS
KIAA0391	14	35121846	35277614		0.053252507	PASS
KIAA0408	6	127440343	127459391		-0.304168115	PASS
KIAA0513	16	85027751	85094230		-0.423061015	PASS
KIAA0556	16	27550133	27780369		0.053595371	PASS
KIAA0586	14	58427385	58551289		0.053252507	PASS
KIAA0753	17	6578148	6640927		0.06854245	PASS
KIAA0825	5	94152966	94618597		-0.393084436	PASS

KIAA0895	7	36324221	36390125	-0.72421718	PASS
KIAA0895	16	67175602	67184040	-0.423061015	PASS
KIAA0930	22	45190338	45240769	-0.953096644	PASS
KIAA1024	15	79432516	79472290	-0.759380635	PASS
KIAA1024	5	129748079	129766732	-0.393084436	PASS
KIAA1109	4	122152333	122362758	0.142431244	suppress_segment_too_large
KIAA1143	3	44737661	44761662	-0.727808231	PASS
KIAA1147	7	141656728	141702153	-0.712149877	PASS
KIAA1191	5	176346061	176361968	0.165341871	suppress_segment_too_large
KIAA1210	23	119078635	119150579	0.158844052	PASS
KIAA1211	4	56049073	56328625	0.115781418	suppress_segment_too_large
KIAA1211	2	98793846	98936259	0.064122364	PASS
KIAA1217	10	23694746	24547848	0.075711544	PASS
KIAA1257	3	128909866	129002690	0.06434429	PASS
KIAA1324	1	109113679	109206781	0.079255572	PASS
KIAA1324	7	86876906	87059699	-0.221864321	PASS
KIAA1328	18	36829106	37232172	-0.330515409	PASS
KIAA1522	1	32741885	32774970	0.048749694	PASS
KIAA1549	7	138831381	138981318	-0.712149877	PASS
KIAA1549	11	33376466	33674102	-0.359426047	PASS
KIAA1551	12	31959370	31993107	0.49032213	suppress_segment_too_large
KIAA1586	6	57046532	57055239	0.239805577	suppress_segment_too_large
KIAA1614	1	180913154	180951614	0.088103245	PASS
KIAA1671	22	24952730	25197448	-0.740245994	PASS
KIAA1755	20	38210488	38260772	-0.172056771	suppress_segment_too_large
KIAA1841	2	61065871	61138034	-0.46122572	PASS
KIAA1958	9	112486847	112669397	0.073960965	PASS
KIAA2012	2	202073289	202205203	0.011001208	PASS
KIAA2013	1	11919591	11926428	0.364796772	PASS
KIAA2026	9	5881596	6007901	0.073960965	PASS
KIDINS220	2	8721081	8837630	0.034263343	PASS
KIF11	10	92593286	92655395	-0.414937136	PASS
KIF12	9	114086126	114099291	0.073960965	PASS
KIF13A	6	17759183	17987623	0.069043418	PASS
KIF13B	8	29067279	29263124	-0.311940419	PASS
KIF14	1	200551497	200620734	-0.204996646	suppress_segment_too_large
KIF15	3	44761717	44873376	-0.727808231	PASS
KIF16B	20	16272104	16573434	-0.172056771	suppress_segment_too_large
KIF17	1	20664014	20718017	0.048749694	PASS
KIF18A	11	28020620	28108308	-0.359426047	PASS
KIF18B	17	44924709	44947711	0.051798665	PASS
KIF19	17	74326210	74355820	0.051798665	PASS
KIF1A	2	240713761	240821036	0.011001208	PASS
KIF1B	1	10210805	10381603	0.364796772	PASS
KIF1BP	10	68988721	69043544	0.032351597	PASS
KIF1C	17	4997948	5028401	0.06854245	PASS
KIF20A	5	138178719	138187715	-0.393084436	PASS
KIF20B	10	89701610	89774939	-0.414937136	PASS
KIF21A	12	39293228	39443390	-0.46813528	PASS
KIF21B	1	200969390	201023700	-0.204996646	suppress_segment_too_large
KIF22	16	29790719	29805385	0.053595371	PASS
KIF23	15	69414246	69448427	-0.759380635	PASS
KIF24	9	34252381	34311371	0.073960965	PASS
KIF25	6	167996241	168045089	0.033894706	PASS

KIF26A	14	104138723	104180894	-0.155273506	suppress_segment_too_large
KIF26B	1	245154985	245709431	-0.145670469	suppress_likely_unfocal_large_gain
KIF27	9	83836698	83921465	0.073960965	PASS
KIF2A	5	62306162	62537249	0.07724032	PASS
KIF2B	17	53822901	53825213	0.051798665	PASS
KIF2C	1	44739818	44767767	-0.736943052	PASS
KIF3A	5	132692628	132737638	-0.393084436	PASS
KIF3B	20	32277664	32335011	-0.172056771	suppress_segment_too_large
KIF3C	2	25926596	25982749	0.034263343	PASS
KIF4A	23	70290090	70420832	0.158844052	PASS
KIF4B	5	155013755	155018132	-0.393084436	PASS
KIF5A	12	57549998	57586632	-0.743220499	PASS
KIF5B	10	32009010	32056431	0.075711544	PASS
KIF5C	2	148875250	149026759	-0.30549516	PASS
KIF6	6	39329990	39725405	0.028532667	PASS
KIF7	15	89608789	89655451	-0.759380635	PASS
KIF9	3	47228026	47283451	-0.727808231	PASS
KIFAP3	1	169921326	170085208	0.088103245	PASS
KIFC1	6	33391536	33409924	0.028532667	PASS
KIFC2	8	144466043	144474202	0.699132336	PASS
KIFC3	16	57758217	57863053	-0.423061015	PASS
KIN	10	7750962	7787981	0.075711544	PASS
KIR2DL1	19	54769811	54784322	0.142581352	PASS
KIR2DL3	19	54738515	54753052	0.142581352	PASS
KIR2DL4	19	54803535	54814517	0.142581352	PASS
KIR3DL1	19	54816468	54830778	0.142581352	PASS
KIR3DL2	19	54850443	54867207	0.142581352	PASS
KIR3DL3	19	54724479	54736536	0.142581352	PASS
KIRREL1	1	157993273	158100262	0.088103245	PASS
KIRREL2	19	35855861	35867109	0.044838622	PASS
KIRREL3	11	126423359	127003460	-0.431650509	PASS
KISS1	1	204190341	204196486	-0.204996646	suppress_segment_too_large
KISS1R	19	917287	921015	-0.071973357	PASS
KIT	4	54657918	54740715	0.115781418	suppress_segment_too_large
KITLG	12	88492793	88580851	0.271127429	suppress_segment_too_large
KIZ	20	21125983	21246622	-0.172056771	suppress_segment_too_large
KL	13	33016433	33066145	-0.398748891	PASS
KLB	4	39406853	39451536	-0.753159364	PASS
KLC1	14	103561896	103714249	-0.155273506	suppress_segment_too_large
KLC2	11	66257294	66267860	0.064535884	PASS
KLC3	19	45333434	45351520	0.002743503	PASS
KLC4	6	43040777	43075099	0.028532667	PASS
KLF1	19	12884422	12887199	-0.069222905	PASS
KLF10	8	102648779	102655902	0.355469066	suppress_segment_too_large
KLF11	2	10042849	10054836	0.034263343	PASS
KLF12	13	73686089	73995056	-0.384313056	PASS
KLF13	15	31326855	31435665	-0.423772929	PASS
KLF14	7	130731235	130734176	-0.712149877	PASS
KLF15	3	126342635	126357442	0.06434429	PASS
KLF16	19	1852399	1863568	-0.071973357	PASS
KLF17	1	44118850	44135140	-0.736943052	PASS
KLF18	1	44137821	44141631	-0.736943052	PASS
KLF2	19	16324817	16327874	-0.069222905	PASS
KLF3	4	38664196	38701042	-0.753159364	PASS

KLF4	9	107484852	107490482	0.073960965	PASS
KLF5	13	73054976	73077542	-0.384313056	PASS
KLF6	10	3775996	3785281	0.075711544	PASS
KLF7	2	207074137	207167267	0.011001208	PASS
KLF8	23	56232421	56287889	0.158844052	PASS
KLF9	9	70384597	70414624	0.073960965	PASS
KLHDC1	14	49693105	49753152	0.053252507	PASS
KLHDC10	7	130070510	130135720	-0.712149877	PASS
KLHDC2	14	49767608	49786385	0.053252507	PASS
KLHDC3	6	43014103	43021298	0.028532667	PASS
KLHDC4	16	87696485	87765992	-0.423061015	PASS
KLHDC7A	1	18480982	18486126	0.048749694	PASS
KLHDC7B	22	50545891	50551023	-0.953096644	PASS
KLHDC8A	1	205336065	205357090	-0.204996646	suppress_segment_too_large
KLHDC8B	3	49171611	49176486	-0.727808231	PASS
KLHDC9	1	161098361	161100346	0.088103245	PASS
KLHL1	13	69700594	70108493	-0.384313056	PASS
KLHL10	17	41835685	41848384	0.041294512	PASS
KLHL11	17	41853545	41865431	0.041294512	PASS
KLHL12	1	202891100	202928636	-0.204996646	suppress_segment_too_large
KLHL13	23	117897813	118117340	0.158844052	PASS
KLHL14	18	32672671	32773062	-0.330515409	PASS
KLHL15	23	23983720	24027186	0.158844052	PASS
KLHL17	1	960587	965715	-0.358281697	PASS
KLHL18	3	47282917	47346816	-0.727808231	PASS
KLHL2	4	165207618	165323156	0.142431244	suppress_segment_too_large
KLHL20	1	173714941	173786702	0.088103245	PASS
KLHL21	1	6590724	6614607	0.364796772	PASS
KLHL22	22	20441519	20495883	-0.740245994	PASS
KLHL23	2	169694488	169776989	0.033541467	PASS
KLHL24	3	183635568	183684477	0.02267528	PASS
KLHL25	15	85759323	85795030	-0.759380635	PASS
KLHL26	19	18636965	18671714	-0.069222905	PASS
KLHL28	14	44924319	45042322	0.053252507	PASS
KLHL29	2	23385217	23708611	0.034263343	PASS
KLHL3	5	137617500	137736090	-0.393084436	PASS
KLHL30	2	238138722	238152947	0.011001208	PASS
KLHL31	6	53647901	53665708	0.239805577	suppress_segment_too_large
KLHL32	6	96924620	97140754	-0.355603029	PASS
KLHL33	14	20425852	20436158	0.053252507	PASS
KLHL34	23	21654690	21658330	0.158844052	PASS
KLHL35	11	75422394	75430629	0.064535884	PASS
KLHL36	16	84648525	84667686	-0.423061015	PASS
KLHL38	8	123645527	123652950	0.493344802	suppress_segment_too_large
KLHL4	23	87517749	87670050	0.158844052	PASS
KLHL40	3	42685519	42692544	-0.727808231	PASS
KLHL41	2	169509702	169526262	0.033541467	PASS
KLHL42	12	27780020	27803040	0.49032213	suppress_segment_too_large
KLHL5	4	39045039	39126857	-0.753159364	PASS
KLHL6	3	183487531	183555689	0.02267528	PASS
KLHL7	7	23105758	23177914	-0.72421718	PASS
KLHL8	4	87160103	87240314	-0.677982248	PASS
KLHL9	9	21329671	21335380	0.073960965	PASS
KLK1	19	50819148	50823787	0.002743503	PASS

KLK10	19	51012739	51020175		0.002743503	PASS
KLK11	19	51022216	51028039		0.002743503	PASS
KLK12	19	51029092	51035230		0.002743503	PASS
KLK13	19	51056206	51065114		0.002743503	PASS
KLK14	19	51077495	51084245		0.002743503	PASS
KLK15	19	50825289	50837213		0.002743503	PASS
KLK2	19	50861568	50880567		0.002743503	PASS
KLK3	19	50854915	50860764		0.002743503	PASS
KLK4	19	50906352	50910738		0.002743503	PASS
KLK5	19	50943303	50953093		0.002743503	PASS
KLK6	19	50958631	50969673		0.002743503	PASS
KLK7	19	50976482	50984099		0.002743503	PASS
KLK8	19	50996007	51002711		0.002743503	PASS
KLK9	19	51003111	51009634		0.002743503	PASS
KLKB1	4	186208979	186258471		0.275137421	PASS
KLLN	10	87859161	87863437		-2.417109027	suppress_large_homdel
KLRB1	12	9594551	9607886		0.505438545	PASS
KLRC1	12	10442264	10454685		0.505438545	PASS
KLRC2	12	10426854	10442300		0.505438545	PASS
KLRC3	12	10412312	10420595		0.505438545	PASS
KLRC4	12	10407382	10409757		0.505438545	PASS
KLRC4-KI	12	10372353	10410146		0.505438545	PASS
KLRD1	12	10226058	10329600		0.505438545	PASS
KLRF1	12	9827481	9845007		0.505438545	PASS
KLRF2	12	9881489	9895833		0.505438545	PASS
KLRG1	12	8950044	9010760		0.505438545	PASS
KLRG2	7	139452690	139483712		-0.712149877	PASS
KLRK1	12	10372353	10391874		0.505438545	PASS
KMO	1	241532134	241595642		-0.287484925	PASS
KMT2A	11	118436464	118526832		-0.431650509	PASS
KMT2B	19	35718019	35738878		0.044838622	PASS
KMT2C	7	152134922	152436005		-0.712149877	PASS
KMT2D	12	49018975	49059774		-0.743220499	PASS
KMT2E	7	105014179	105114361		-0.712149877	PASS
KMT5A	12	123383773	123409358		0.028722739	PASS
KMT5B	11	68154863	68213828		0.064535884	PASS
KMT5C	19	55339853	55348120		0.142581352	PASS
KNCN	1	46545644	46551527		-0.736943052	PASS
KNDC1	10	133160447	133226412		0.073090211	PASS
KNG1	3	186717276	186744410		0.02267528	PASS
KNL1	15	40594020	40664342		-0.423772929	PASS
KNOP1	16	19701934	19718235		0.053595371	PASS
KNSTRN	15	40382721	40394246		-0.423772929	PASS
KNTC1	12	122527246	122626396		0.028722739	PASS
KPNA1	3	122421949	122514945		0.06434429	PASS
KPNA2	17	68035519	68046842		0.051798665	PASS
KPNA3	13	49699307	49792921		-0.384313056	PASS
KPNA4	3	160494995	160565588		0.02267528	PASS
KPNA5	6	116681187	116741866		-0.355603029	PASS
KPNA6	1	32108038	32176568		0.048749694	PASS
KPNA7	7	99173574	99207506		-0.710463272	PASS
KPNB1	17	47649476	47685505		0.051798665	PASS
KPRP	1	152759561	152762052		0.088103245	PASS
KPTN	19	47475144	47484268		0.002743503	PASS

[truncated: 405,709 more chars]
